# Identifying breast cancer risk loci by global differential allele-specific expression (DASE) analysis in mammary epithelial transcriptome

**DOI:** 10.1186/1471-2164-13-570

**Published:** 2012-10-30

**Authors:** Chuan Gao, Karthik Devarajan, Yan Zhou, Carolyn M Slater, Mary B Daly, Xiaowei Chen

**Affiliations:** 1 Cancer Epigenetics Program, Fox Chase Cancer Center, 333 Cottman Avenue, Philadelphia, PA, 19111, USA; 2 Department of Biostatics and Bioinformatics, Fox Chase Cancer Center, Philadelphia, PA, 19111, USA; 3 Department of Biostatics and Bioinformatics, Fox Chase Cancer Center, Philadelphia, PA, 19111, USA; 4 Department of Clinical Genetics, Fox Chase Cancer Center, Philadelphia, PA, 19111, USA

**Keywords:** Differential allele-specific expression, Breast cancer susceptibility, SNP array, *DMBT1*

## Abstract

**Background:**

The significant mortality associated with breast cancer (BCa) suggests a need to improve current research strategies to identify new genes that predispose women to breast cancer. Differential allele-specific expression (DASE) has been shown to contribute to phenotypic variables in humans and recently to the pathogenesis of cancer. We previously reported that nonsense-mediated mRNA decay (NMD) could lead to DASE of *BRCA1/2,* which is associated with elevated susceptibility to breast cancer. In addition to truncation mutations, multiple genetic and epigenetic factors can contribute to DASE, and we propose that DASE is a functional index for *cis*-acting regulatory variants and pathogenic mutations, and that global analysis of DASE in breast cancer precursor tissues can be used to identify novel causative alleles for breast cancer susceptibility.

**Results:**

To test our hypothesis, we employed the Illumina® Omni1-Quad BeadChip in paired genomic DNA (gDNA) and double-stranded cDNA (ds-cDNA) samples prepared from eight BCa patient-derived normal mammary epithelial lines (HMEC). We filtered original array data according to heterozygous genotype calls and calculated DASE values using the Log ratio of cDNA allele intensity, which was normalized to the corresponding gDNA. We developed two statistical methods, SNP- and gene-based approaches, which allowed us to identify a list of 60 candidate DASE loci (DASE ≥ 2.00, *P* ≤ 0.01, FDR ≤ 0.05) by both methods. Ingenuity Pathway Analysis of DASE loci revealed one major breast cancer-relevant interaction network, which includes two known cancer causative genes, *ZNF331* (DASE = 2.31, *P* = 0.0018, FDR = 0.040) and *USP6* (DASE = 4.80, *P* = 0.0013, FDR = 0.013), and a breast cancer causative gene, *DMBT1* (DASE=2.03, *P* = 0.0017, FDR = 0.014). Sequence analysis of a 5′ RACE product of *DMBT1* demonstrated that *rs2981745,* a putative breast cancer risk locus, appears to be one of the causal variants leading to DASE in *DMBT1*.

**Conclusions:**

Our study demonstrated for the first time that global DASE analysis is a powerful new approach to identify breast cancer risk allele(s).

## Background

Breast cancer is the most common cancer and the second most common cause of cancer-related death in women. It is estimated that one out of eight American women will develop breast cancer some time in their lifespan and 3.0% will die from this disease [1]. For the year 2012, about 226,870 new invasive breast cancer diagnoses and 39,970 breast cancer related deaths are expected in the United States [2]. Due to this high prevalence and severe consequences, genetic factors contributing to breast cancer risk have been intensively studied.

Family history is known to be associated with 20% - 30% of breast cancer incidence in the United States [3]. Pedigree analysis of clustered familial cases followed by positional cloning in the 1990s led to the discovery of tumor suppressor genes, *BRCA1*[4] and *BRCA2*[5], two major breast cancer susceptibility loci. Deleterious mutations in these genes increase the risk of developing breast cancer by more than 10 fold and overall account for 15% - 30% of observed risks in familial breast cancer cases [6]. To discover breast cancer susceptibility alleles that constitute the remainder of genetic risk, genes associated with *BRCA1/2* pathways were investigated in *BRCA1/2* mutation negative familial cases. Such candidate gene approaches revealed that germline mutations of *TP53*[7], *PTEN*[8], *ATM*[9], *CHEK2*[10], *BRIP1*[11], *PALB2*[12], *NBS1*[13] and *RAD50*[14] are correlated with breast cancer risk, but to a much more moderate extent than *BRCA1* and *BRCA2*. Therefore, new unbiased genomic approaches are needed for identifying genetic factors that influence breast cancer susceptibility.

Over the last decade, advances in array technologies have resulted in the ability to evaluate the expression of thousands of genes simultaneously. These platforms offer a powerful tool to test multiple biomarkers for breast cancer tumorigenesis and prognosis, as well as targeted breast cancer therapy [15]. However, gene expression assessed by current techniques represents the total level of transcripts produced by both parental alleles. The absolute transcript level failed to resolve potential imbalances in relative allelic contribution to the total expression. This perspective is particularly important for familial breast cancers, where an individual inherits a germline mutation on one parental allele, followed by a somatic mutation of the second allele in the tumor cells. Previously, we have reported that mutant *BRCA1* transcripts containing premature stop codons were eliminated or destabilized by nonsense-mediated mRNA decay (NMD) [16] and could lead to a state of haploinsufficiency. As a result, the ratio between the expressions from the wild-type allele and the corresponding mutant allele was significantly increased, resulting in what we coined differential allele-specific expression (DASE) or allelic imbalance (AI) [17].

DASE is a common phenomenon in human tissues [18]. Although its contribution to breast cancer susceptibility has been implicated [17], it has not been studied on a transcriptome-wide scale in breast cancer precursor tissues. Since the phenomenon of DASE at a locus may help identify nearby *cis*-acting transcriptional and epigenetic regulatory sites as well as mutations resulting in non-mediated RNA decay [16,18], we propose that DASE is a sensitive functional index for genetic variants, and can be used as a novel approach to identify risk alleles for breast tumorigenesis. The main objectives of this study are to identify genes with DASE by comparing the allele-specific expression (ASE) and to demonstrate that global DASE analysis could be a powerful new approach to identify breast cancer risk alleles.

## Results

### Global DASE profiling in HMECs

**Figure 1:**
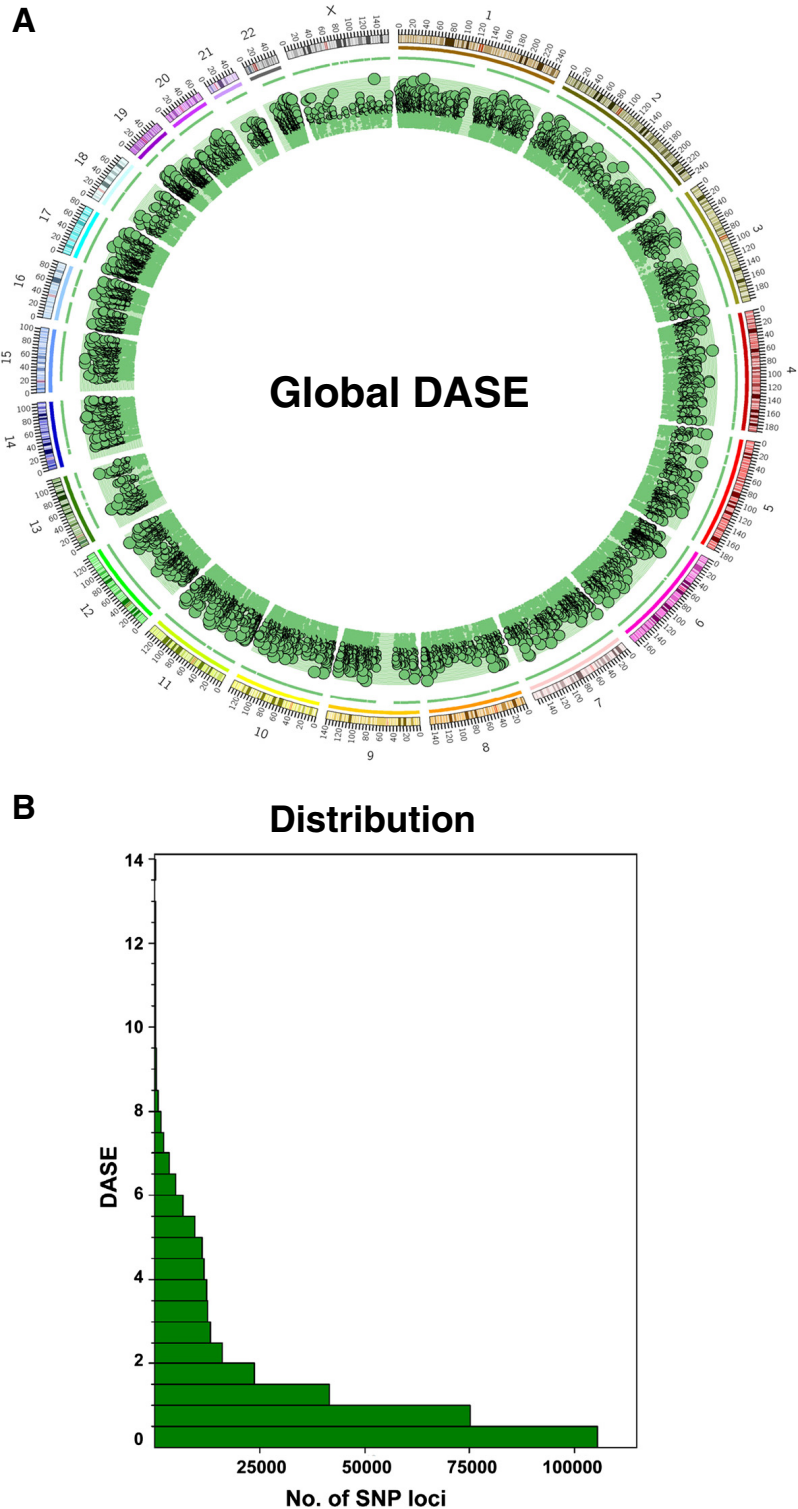
**Global DASE mapping.** (**A**). This Circos plot illustrates the global DASE profiles mapping to each chromosome (outer layer) across eight HMECs. Each green dot within the green circular ring area represents the DASE value for a SNP; (**B**). Histogram of DASE. This diagram uses the number of SNP loci against the DASE value.

There have been successful applications of Illumina’s Infinium assay on global DASE analysis [19,20] since it provides genotyping results based on quantified fluorescent signal intensity of both alleles at a specific SNP site [21]. The samples we used are paired gDNAs and ds-cDNAs derived from eight human mammary epithelial cell lines. In this study, we performed a transcriptome-wide DASE analysis using Illumina’s HumanOmni1-Quad BeadChip platform (Version 1B). Among the total 1,140,419 markers on the Omni1 BeadChip, we focused on SNP markers representing transcribed regions of the female genome for global DASE analysis. Raw data from the array were filtered as described in the Methods section for quality control purposes, and 35,690 qualifying SNPs, representing 8,779 transcribed loci crossing all eight samples, survived for the final DASE analysis. The global DASE pattern at each SNP locus is shown as a Circos plot in Figure 1A (Detailed data are included in Additional file 1: Table S1). As shown in the DASE distribution histogram (Figure 1B), about 30% of loci are with a DASE ≥ 2, which we used as the cut-off to define a locus with a positive DASE event. This result is consistent with previous array studies which suggest DASE is a relatively common event across the human genome [20,22].

### Identification of candidate loci exhibiting significant DASE

**Figure 2:**
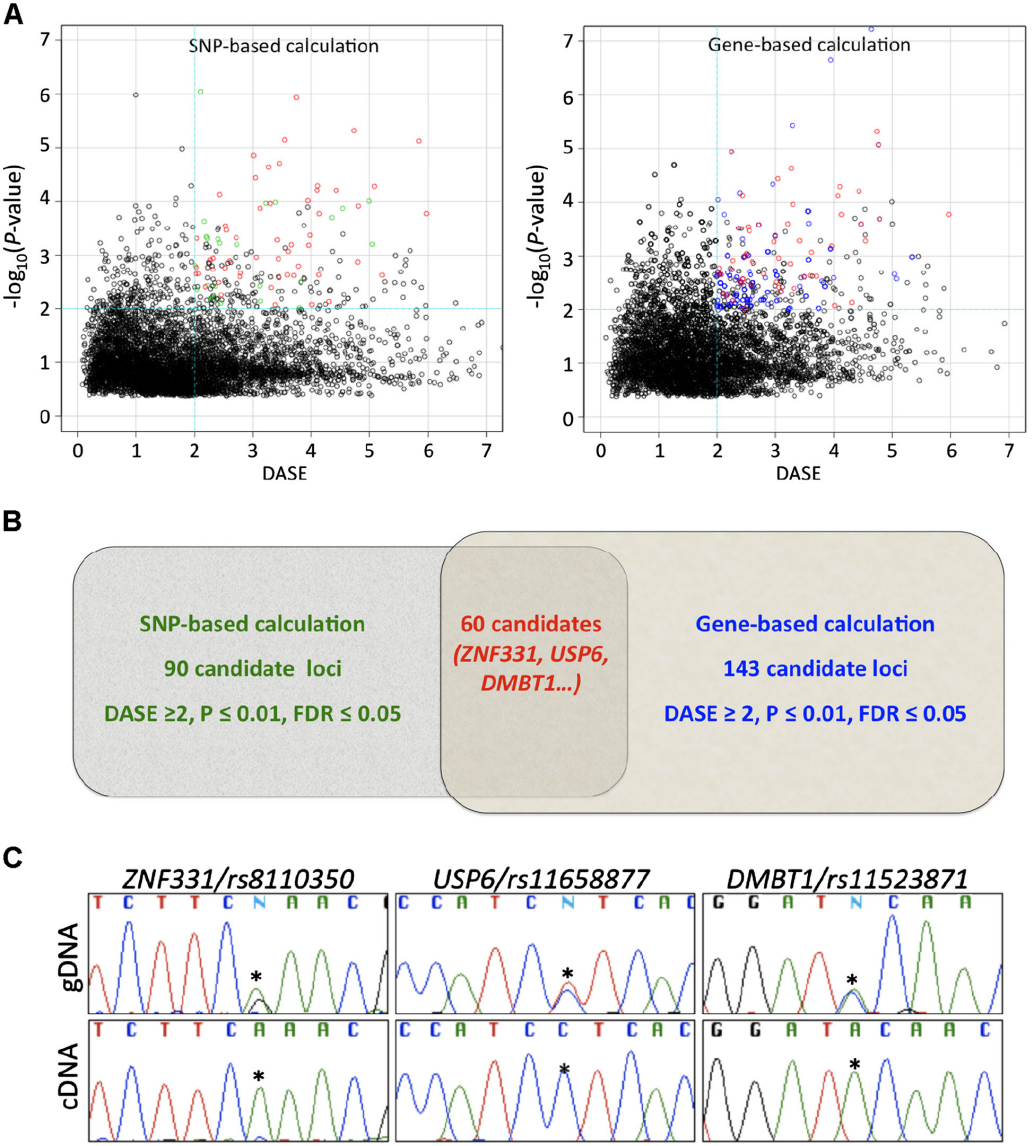
**Identification of DASE loci.** (**A**) We developed 2 statistical methods, SNP-based and gene-based calculation, to assign DASE values to transcribed loci. (**B**) Using the same selection stringency, sixty candidate loci were identified by both methods. Among them, *ZNF331* and *USP6* are classified as cancer causative by the Cancer Gene Census at Sanger Institute. The *DMBT1* locus has been reported to contain breast cancer risk associated variants. (**C**) We used these three genes as examples to show our DASE validation process. Comparing sequencing chromagraphs between cDNA and gDNA amplicons we could easily tell a typical DASE pattern from all of them.

DASE values of transcribed loci were calculated as described in the Methods section. Although a previous study successfully validated candidate loci exhibiting a fold change of 1.5 [23], we raised the stringency by arbitrarily setting the DASE value cut-off bar at 2, equal to a 4-fold variance between alleles, to ensure the significance of the findings. By using SNP-based calculation, 93 SNPs representing 90 transcribed genes showed statistical significance (*P*≤0.01 and FDR≤0.05) (Left panel, Figure 2A). Similarly, using gene-based calculation, 143 genes exhibited statistical significance (*P*≤0.01 and FDR≤0.05) (Right panel, Figure 2A). However, each method presents some degree of limitation. For example, the SNP-based DASE measurement may only represent certain transcribed isoforms of a certain gene, and it is practically true when the targeted SNPs are located in the 5′ or 3′ UTRs of this gene. For the gene-based approach, an outlier of DASE value from one SNP could have too much weight for final DASE results. To decrease the chance for false positive “hits’, it is important that only gene candidates (total 60) discovered by both methods (Figure 2B, Table 1) were carried forward for further analysis.

**Table 1:** DASE Candidate Loci Identified by both SNP- and Gene-based Approaches.*

**Gene_ID**	**SNP**	**SNP-based**			**Gene-based**		
		**DASE**	***P*-Value**	**FDR**	**DASE**	***P*-Value**	**FDR**
*ACPP*	*rs14192*	3.83	0.010497	0.0442	2.32	0.005517	0.0216
*ADAMTSL3*	*rs2277849*	3.13	0.007309	0.0359	2.25	0.014546	0.0280
*AGBL1*	*rs10520618*	3.12	0.000134	0.0279	2.37	0.000305	0.0113
*C1orf94*	*rs6698707*	3.27	0.000023	0.0016	3.27	0.000024	0.0088
*CEP170P1*	*rs28720014*	3.59	0.002355	0.0180	3.59	0.002355	0.0282
*CHRAC1*	*rs10216653*	4.31	0.007459	0.0360	4.31	0.007459	0.0425
*CTAGE11P*	*rs477274*	2.42	0.003269	0.0237	2.42	0.003269	0.0319
*DMBT1*	*rs11523871*	2.03	0.001676	0.0140	2.03	0.001676	0.0268
*FAM154B*	*rs16973457*	2.03	0.002254	0.0423	2.03	0.002254	0.0427
*FLJ39061*	*rs10221698*	4.01	0.008501	0.0379	4.01	0.008501	0.0442
*FLJ42393*	*rs344952*	2.04	0.000490	0.0449	2.25	0.000012	0.0093
*GRIN3A*	*rs10989563*	3.78	0.001589	0.0385	3.25	0.001386	0.0364
*GRK4*	*rs1024323*	2.37	0.000845	0.0532	2.71	0.000261	0.0113
	*rs2960306*	3.01	0.000014	0.0053	-	-	-
*HCG4*	*rs1611213*	2.19	0.003925	0.0257	2.16	0.005494	0.0098
*HLA-G*	*rs1130363*	2.52	0.001700	0.0399	3.49	0.002469	0.0170
*HNRNPKP1*	*rs182844*	2.53	0.000285	0.0071	2.53	0.000285	0.0113
*HS6ST1P1*	*rs6423191*	4.73	0.000005	0.0002	4.73	0.000005	0.0005
*KHSRPP1*	*rs12380505*	2.27	0.001212	0.0342	2.27	0.001212	0.0364
*LAP3P2*	*rs12199346*	3.55	0.000007	0.0010	3.21	0.000250	0.0113
*LOC283398*	*rs7139313*	3.74	0.000001	0.0003	2.58	0.003455	0.0178
*LOC341056*	*rs7933723*	3.70	0.002423	0.0423	3.70	0.002423	0.0428
*LOC387703*	*rs7070947*	3.61	0.000854	0.0305	3.61	0.000854	0.0355
*LOC440973*	*rs605795*	2.79	0.002160	0.0423	2.79	0.002160	0.0427
*LOC442238*	*rs985937*	4.13	0.000168	0.0114	4.13	0.000168	0.0152
*LOC642590*	*rs9455190*	3.66	0.000645	0.0486	2.50	0.000915	0.0355
*LOC643618*	*rs732531*	3.79	0.009696	0.0417	3.12	0.012274	0.0268
*LOC645521*	*rs1976809*	4.11	0.000052	0.0091	4.11	0.000052	0.0128
*LOC646970*	*rs614805*	3.38	0.008546	0.0379	3.09	0.004693	0.0207
*LOC727884*	*rs12371762*	5.21	0.002386	0.0423	4.76	0.000009	0.0002
*LOC729675*	*rs13137565*	2.36	0.003844	0.0498	2.36	0.003844	0.0522
*MAGEC2*	*rs3765272*	3.30	0.000109	0.0091	3.30	0.000109	0.0128
*MMP20*	*rs1784423*	2.32	0.001136	0.0133	2.25	0.001791	0.0268
*MUC16*	*rs2547068*	3.88	0.012106	0.0462	2.61	0.003190	0.0072
*MYADML*	*rs11684598*	5.97	0.000167	0.0114	5.97	0.000167	0.0152
*NLRP1*	*rs11651270*	2.39	0.013058	0.0479	2.87	0.000835	0.0105
*NSUN4*	*rs17361749*	2.43	0.000076	0.0091	2.43	0.000076	0.0128
*OR13H1*	*rs17316625*	3.12	0.003801	0.0498	3.12	0.003801	0.0522
*OR6N1*	*rs857825*	2.78	0.002543	0.0431	2.44	0.002721	0.0443
	*rs857826*	3.98	0.000430	0.0449	-	-	-
*PHF2P1*	*rs9553323*	5.08	0.000053	0.0021	4.47	0.000252	0.0048
*PRDM14*	*rs10089937*	2.07	0.002243	0.0423	2.07	0.002243	0.0427
*PTCHD3*	*rs2484173*	4.13	0.002351	0.0423	3.00	0.000427	0.0128
*PTTG2*	*rs6811863*	3.06	0.001091	0.0342	3.06	0.001091	0.0364
*RPL4P4*	*rs13099317*	4.43	0.000063	0.0011	4.43	0.000063	0.0044
*RPL9P16*	*rs7439293*	3.44	0.001159	0.0133	3.06	0.003857	0.0329
*SLAMF1*	*rs1061217*	3.42	0.000524	0.0085	3.42	0.000524	0.0137
*SMARCE1P3*	*rs11852150*	3.46	0.000020	0.0016	2.97	0.000543	0.0137
*SZT2*	*rs2027130*	2.72	0.000478	0.0085	2.39	0.003010	0.0313
*TARDBPP2*	*rs9528094*	4.81	0.000122	0.0279	2.46	0.009678	0.0454
*TEX34*	*rs11651968*	2.60	0.001264	0.0135	2.60	0.001264	0.0252
*TLK2P1*	*rs3744516*	4.00	0.004876	0.0516	4.78	0.000203	0.0046
	*rs4795846*	5.84	0.000008	0.0053	-	-	-
*TMCC3*	*rs1290005*	3.45	0.002658	0.0198	2.10	0.005092	0.0209
*UFM1*	*rs2485783*	3.96	0.000659	0.0082	3.96	0.000659	0.0100
*UQCRFS1P2*	*rs13238715*	3.95	0.000097	0.0091	3.02	0.003106	0.0177
*USP6*	*rs11658877*	4.80	0.001324	0.0135	4.54	0.000530	0.0086
*WASF4*	*rs909713*	4.10	0.000063	0.0091	4.07	0.000076	0.0128
*WNT3A*	*rs752107*	3.04	0.000037	0.0020	3.04	0.000037	0.0088
*XIAP*	*rs5958343*	2.46	0.005591	0.0327	2.27	0.006924	0.0423
*XPNPEP2*	*rs3747343*	2.55	0.000343	0.0073	2.55	0.000343	0.0113
*ZBTB8OS*	*rs3753603*	3.31	0.004053	0.0259	3.31	0.004053	0.0329
*ZNF331*	*rs8110350*	2.31	0.001826	0.0403	2.48	0.001014	0.0121

*: Only loci with DASE ≥ 2; P ≤ 0.01; and FDR ≤ 0.05 were included.

### Ingenuity pathway analysis (IPA) analysis revealed interaction networks within candidate DASE loci

**Figure 3:**
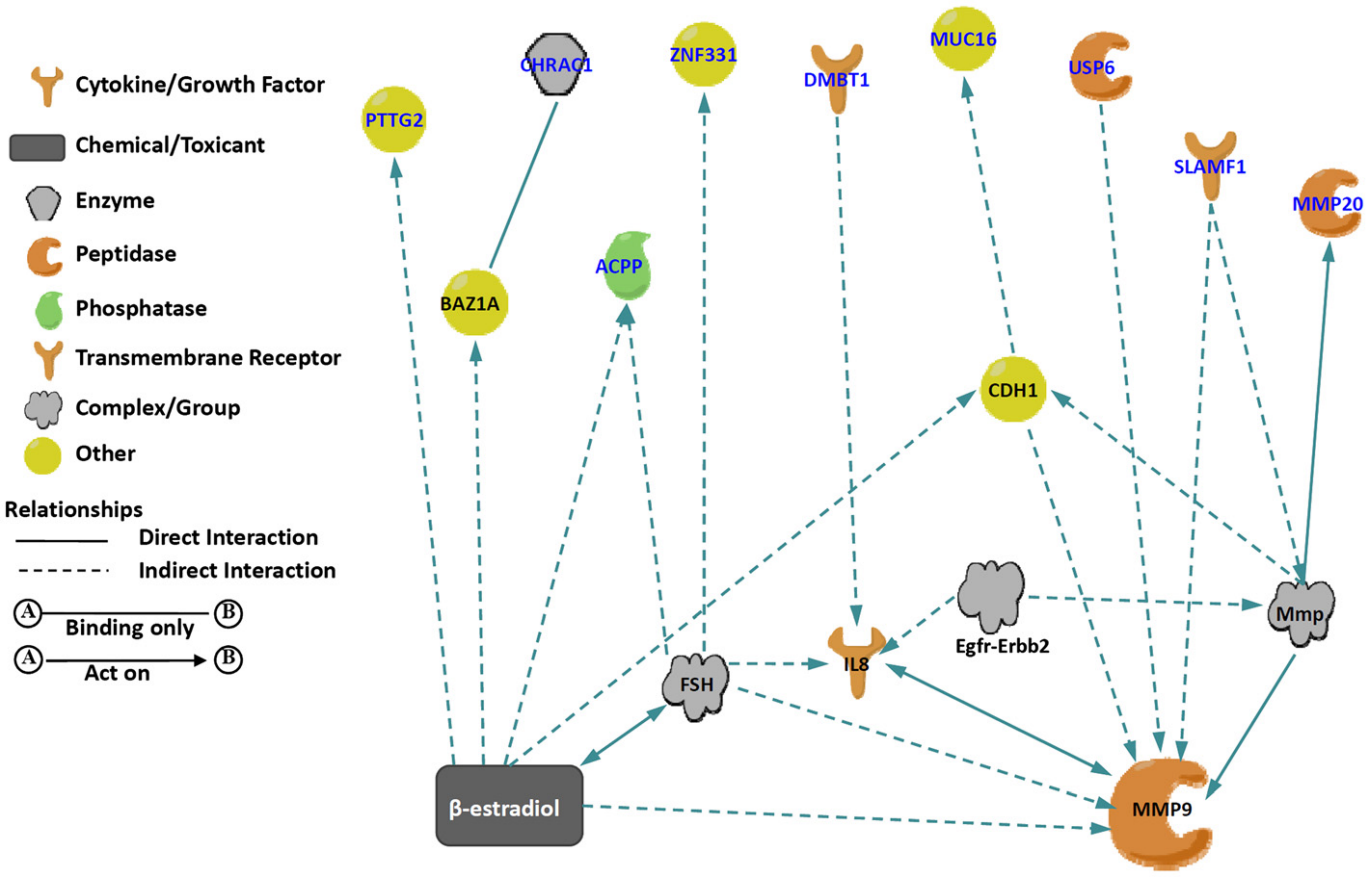
**Functional “DASE” networks.** Ingeunity® Pathway analysis revealed one major interaction network in DASE candidates, including *ZNF331*, *USP6* and *DMBT1* and six other candidates. Most of them are downstream players of sex hormones (β-estradiol) and MMP9, which suggests their potential of being breast cancer risk alleles.

To help interpret the candidate DASE loci in the context of biological processes, pathways and networks, IPA analyses were performed on our DASE candidates. The result showed that 24 out of 34 protein coding loci are involved in known molecular interactions. Among those interactions, there are two major networks. Interestingly, one of the major networks covers 9 DASE candidates, including cancer causative genes *ZNF331* and *USP6* and known breast cancer associated gene *DMBT1*, and most of them are downstream players of sex hormones (β-estradiol) and MMP pathways, suggesting their potential for being breast cancer risk alleles (Figure 3). In addition, IPA analysis also revealed a variety of biological functions that candidate DASE loci are significantly associated with (*P*<0.05, Additional file 2: Table S2). The cellular functions of these genes are wide-ranging, including cell proliferation, cell death, and inflammation.

### Validating candidate DASE loci by Sanger sequencing

We utilized Sanger sequencing to validate nine DASE candidates in one major interaction network (Figure 3). In brief, regions flanking DASE-associated SNPs were amplified by PCR followed by sequencing and comparing trace chromatograms between paired gDNA and cDNA samples. The DASE value between two alleles in a sample of a given SNP was evaluated by measuring the peak height of each allele in the chromograms originated from the cDNA sample, justified by that originated from the genomic DNA sample. Figure 2C showed the examples of sequencing trace files in the validation of DASE in *ZNF331*, *USP6*, and *DMBT1*. A positive DASE event by sequencing is defined when the height of the peak representing one allele is less than half of the peak height of the other allele. As summarized in Table 2, we clearly observed DASE in 9 candidate loci by sequencing in 31 out of 39 (79%) of samples with DASE identified by global DASE analysis. These results supported that our approach reported here to identify DASE loci by high dense SNP array is successful.

**Table 2:** Validation of DASE Candidate by Sanger Sequencing

**Gene ID**	**SNP ID**	**Heterozygous samples**	DASE events* by array	**DASE events by sequencing**
*ACPP*	*rs14192*	5	4	3
*CHRAC1*	*rs10216653*	6	5	1
*DMBT1*	*rs11523871*	6	6	6
*MMP20*	*rs1784423*	6	6	6
*MUC16*	*rs2547068*	6	4	3
*PTTG2*	*rs6811863*	5	5	4
*SLAMF1*	*rs1061217*	6	6	2^†^
*USP6*	*rs11658877*	6	6	2^†^
*ZNF331*	*rs8110350*	5	5	4
**Total:**		**51**	**47**	**31**^**§**^

*: A positive DASE event is defined when the ratio of ASE levels from one allele to another allele is either more that 2 or less that 0.5.

^†^: Only two samples were able to be validated because of unsuccessful cDNA sequencing at this locus for some samples.

^§^: DASE were confirmed by sequencing in total 31 out of 39 samples whose cDNA sequencings were successful.

### Identifying causative variants for DASE in *DMBT1*

**Figure 4:**
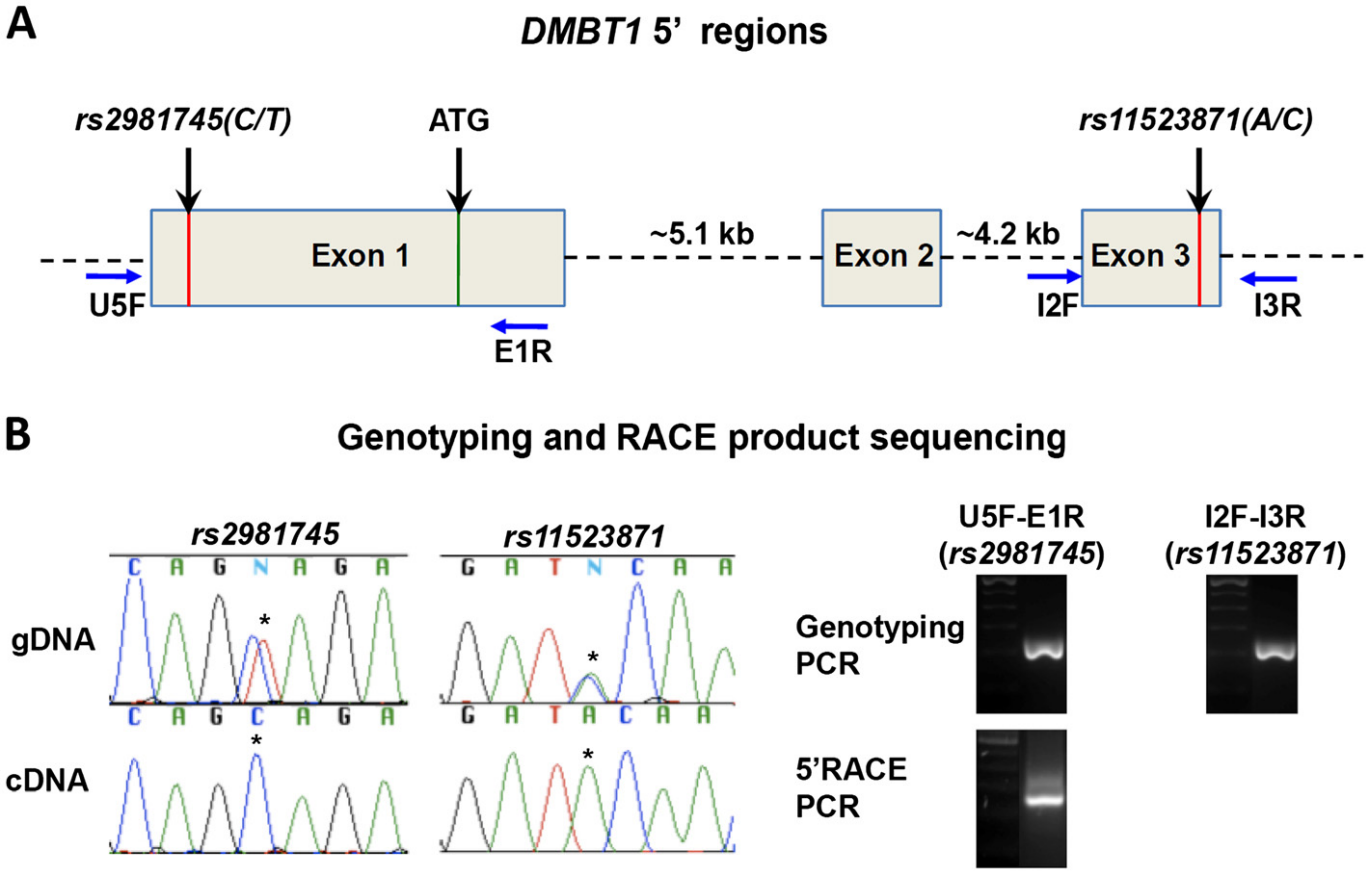
**RACE analysis for *DMBT1 *5′UTR.** (**A**) DASE candidate *DMBT1* (DASE=2.03, *P*=0.0017, FDR=0.014) was identified by analyzing *rs11523871* located in the third exon. SNP *rs2981745* in the first exon of *DMBT1* has been known to be associated with breast cancer risk [24]. These two SNPs are ~10 kb from each other. (**B**) Genotyping and sequencing of *DMBT1* 5′ RACE product confirmed that *rs2981745-C* is linked with *rs11523871-A* in our HMECs.

Candidate DASE locus *DMBT1* was identified and validated by analyzing SNP *rs11523871* (Figure 2C). A nearby SNP, *rs2981745* (C>T) in 5′UTR region of *DMBT1*, has been reported to be associated with increased breast cancer risk and *rs2981745*-T has decreased promoter activity compared with *rs2981745*-C [24]. Since *rs2981745* is not covered in the HumanOmni1 BeadChip, we carried out additional genotyping across all eight HMECs and found *rs2981745* heterozygous in 6 HMECs that are also heterozygous for *rs11523871* (Data not shown)*.* Sequence analysis of *DMBT1* 5′ RACE product revealed that *rs11523871-C* co-presents with *rs2981745-A* in all six HMECs, which suggests that DASE observed in *DMBT1* was caused by the loss of expression of *rs2981745T* allele (Figure 4). To examine if any variants in *DMBT1* 3′ UTR could also contribute to DASE in *DMBT1*, we fully sequenced 3′UTR region in the genomic DNAs of all 8 HMECs and identified two common SNPs, *rs8441* and *rs7383*, both presented in samples HMEC-1 and −5 (Additional file 3: Figure S1). We further sequenced the same regions in cDNA products isolated from these two HMEC lines. The results from HMEC-1 are consistent with DASE pattern discovered by analyzing heterozygous *rs11523871* in this sample (Table 2). Importantly, the results from HEMC-5, in which both *rs11523871* and *rs2981745* are homozygous, revealed typical bi-allelic expression (Additional file 3: Figure S1). We then used an online miRNA targeting tool, Probability of Interaction by Target Accessibility (PITA) [25], to compare the effects of these variants on miRNA binding, and only very subtle differences of miRNA targeting were found among all genotype combinations (Additional file 4: Figure S2). Taken together, we concluded that in our study, variants in *DMBT1* 3′UTR region unlikely contribute to DASE. Our data suggested that *rs2981745*, if not exclusively, appears to be one of the causative variants for DASE in *DMBT1*.

## Discussion

In our current study, we demonstrated transcriptome-wide DASE analysis as a novel approach to identify breast cancer susceptibility loci. The HumanOmni1-Quad BeadChip we utilized has state of the art coverage of common SNPs on the human transcriptome. Our study identified 60 candidate DASE loci by both SNP- and gene-based methods (Table 1 and Figure 2). Pathway analysis reveals one major DASE gene network which is likely associated with breast tumorigenesis (Figure 3). Using PCR and Sanger sequencing, we successfully validated the DASE predictions in this breast cancer-related network, which includes cancer causative genes *ZNF331* and *USP6,* and breast cancer risk associated gene *DMBT1* (Table 2). By analyzing the 5′UTR region of *DMBT1*, we successfully identified *rs2981745* as the causal variant for the DASE in *DMBT1* (Figure 4). Therefore, we presented an example supporting our original expectation that DASE analysis may lead to the discovery of functional DASE-causing variants. DASE in *ZNF331* has possibly resulted from genomic imprinting as indicated from studies in human extraembryonic tissues [26], but we are not able to verify such speculation for the lack of genetic material from parents. Nevertheless, we reported for the first time about such phenomenon of *ZNF331* in primary cultures of adult human tissue. We did not investigate further with the 26 non-coding DASE candidate loci in current studies largely because very little information could be obtained to help interpret their roles. However, there have been a few studies denoting the importance of non-coding RNAs in cellular processes during recent years [27,28]. Considering the high percentage (nearly 50%) of such loci in our candidate list, it is an important next step for us to validate non-coding candidate DASE loci and study their likely roles in breast tumorigenesis.

As the Illumina Human Omni1-Quad Array is originally designed for targeting genomic DNA sequences instead of cDNA sequences, intronic SNPs and many SNPs at the exon/intron boundary are left out for DASE profiling. Although the number of “usable” SNPs covers the majority of transcripts, the coverage could be further increased with newly developed platforms with additional SNP markers. Furthermore, the size of the probes on the Illumina SNP array is 50mer, and it is likely difficult for them to pick up small size transcripts, such as miRNAs. Therefore, it would be logical to consider designing customized BeadChips covering pre-selected probes to improve both cost-effectiveness and specificity, and to reduce the data processing load for global DASE profiling as well. In addition, we observed fluorescent interference between X and Y channels during the data analysis due to the initial probe design [29], and it was able to be alleviated by canonical data normalization. Despite these limitations, Infinium-based BeadChip is still a practical platform for whole transcriptome DASE analysis as indicated by the results of successful validation using Sanger sequencing (Table 2).

As mentioned in the Introduction, currently known high and moderate penetrance risk alleles help only to explain a fraction of familial breast cancer incidents and the existence of more susceptibility genes are likely to be very rare. Thus, it is plausible that the remaining cases could be complemented by the synergistic effects of multiple low penetrance alleles, each conferring an elevated risk of <1.5 fold [30]. The completion of the Human Genome Project and fast development of SNP array technologies have made it practical to perform genome-wide association studies (GWAS) to identify genetic factors that account for breast tumorigenesis. Since the first wave of GWAS to search for such alleles, 22 loci have been reported to significantly associate with breast cancer risk by 16 studies [31-34]. Among those candidate risk loci, nearly half (*2q35, 3p24.1, 5p12, 5q11.2, 6q25.1, 8q24.21, 10q26.13, 11p15.5, 16q21.1-21.2*) were reproducible in multiple independent studies, which denotes the reliability of GWAS prediction. Despite these successes, the mechanisms of how these GWA variants affect breast cancer pathogenesis are often unknown, because in most cases it is not clear which gene(s) are associated with GWA signals [35,36]. Therefore, understanding the function of these breast cancer associated variants and the mechanisms of how they contribute to breast cancer is a logical next step to validate GWAS findings. Until now, most of them are merely SNP tags for yet unknown breast cancer causal variants [37]. A couple of exceptions so far were *rs2981582-*A [31] and *rs1219648-*G [32] on *FGF2R* locus. The discovery of these two common low risk variants eventually pinpointed *rs2981576* and *rs2981578* as causal variants by genetic mapping and CHIP assay [38]. Evaluation of the functional impact of putative causal variants identified by GWAS could be very challenging as many GWA signals (e.g., 8q24) map some distance from the nearest coding regions, and are likely to mediate disease predisposition through remote regulatory effects on transcription [39]. In addition, the causal alleles involved in breast cancer susceptibility are likely to have moderate molecular and cellular effects, and the measurable effects of an allele in a given functional assay may not exhibit a causal role in breast cancer pathogenesis by itself. Therefore, novel strategies are required to functionally characterize the multiple GWA signals in a genome-wide manner for convincing genetic evidence. Based on previous reports [24,40], our study has pinpointed a likely causal variant for DASE in *DMBT1* (Figure 4). In addition, genetic mapping in mice has indicated that *DMBT1* is a candidate modifier of mammary tumors and breast cancer risk [41], and our results further support that allelic loss of expression in *DMBT1* could contribute to breast cancer development, which is consistent with the reports from previous studies [42,43]. These findings support the idea that global DASE profiling mapping could be a powerful approach to validate GWAS findings when the DASE loci map is overlapped with existing data from GWAS [44].

In our study, we have compared top DASE loci identified in our genome-wide ASE studies with the current cancer genome database provided by Cancer Gene Census (Sanger Institute) and have identified two DASE loci (*USP6* and *ZNF331*) listed as cancerous genes [45]. Somatic mutations in *USP6* (p.V678A and p.R475Q) and in *ZNF331* (p.G193E) have been reported in 1% of primary breast cancer and 2% of ovarian cancer, respectively [45]. These findings suggest that global DASE profiling could be a powerful tool in combination with currently available cancer genome databases to identify novel breast cancer “driver” genes. Furthermore, on-going cancer genome sequencing projects (e.g. Cancer Genome Atlas by NCI) have identified thousands of so-called variants of unknown/uncertain significance (VUS), including variations typically characterized by a single base change, or a change to several intronic bases. This large amount of VUS data produced by the next generation sequencing-based cancer genome projects has been termed “overkill” from a clinical perspective [46]. Currently, the consequence of most VUS in oncogenesis has yet to be established. As DASE is a sensitive functional index for pathogenic mutations, it can be applied for validating these VUS at a genome-wide scale as well.

## Conclusions

We have demonstrated for the first time that global DASE analysis is a novel approach to identify breast cancer risk alleles. The results from our study are very promising and we expect our strategy will help validate the functional variants identified by the GWAS and Cancer Genome Projects. Importantly, the research strategy developed here could be easily applied to investigating susceptibility for many other types of cancers.

## Methods

### Primary human mammary epithelial cultures (HMECs)

For studies reported here, eight HMEC lines, tested *BRCA1/2* mutation negative, were utilized as starting material for DASE analysis. Under an approved protocol by the Institutional Review Board (IRB) at Fox Chase Cancer Center, we routinely derived primary HMEC lines from adjacent or contralateral normal mammary tissue of breast cancer patients using an established commercial protocol of EpiCult®-B human mammary epithelial cell culture (Stemcell Technologies, BC, Canada). Established primary HMEC lines were maintained in medium containing 1:1 DMEM/F12 (Life Technologies, Carlsbad, CA), 2.438 g/L sodium bicarbonate, 5% chelated horse serum, 20 ng/ml EGF (BD Biosciences, San Jose, CA), 100 ng/ml cholera toxin (Sigma-Aldrich, St. Louis, MO), 10 mg/L insulin (Sigma-Aldrich, St. Louis, MO), 0.5 mg/L hydrocortisone (Sigma-Aldrich, St. Louis, MO), Antibiotic-Antimycotic (Life Technologies, Carsbad, CA), and 0.04 mM calcium chloride (Sigma-Aldrich, St. Louis, MO).

### Preparation of DNA, RNA and double-stranded cDNA

Genomic DNAs (gDNAs) were isolated from HMEC lines by phenol-chloroform extraction as previously described [47]. Total RNAs were isolated from HMEC lines by cell lysis in guanidinium isotiocyanate buffer supplemented with 2-mercaptoethanol (BME) followed by phenol-chloroform extraction using a protocol as previously described [48]. After re-dissolving, RNAs were treated with DNase (TURBO DNA-free kit, Ambion, Austin, TX) to remove possible genomic DNA contamination. The concentrations of genomic DNA and RNA stocks were measured using a ND-1000 spectrometer (NanoDrop, Wilmington, DE). To perform the DASE profiling, double stranded (ds)-cDNAs were synthesized from total RNAs using the SuperScript® Double-Stranded cDNA Synthesis Kit (Life Technologies, Carlsbad, CA) and random hexamers following manufacturer’s instructions.

### Genome-wide DASE profiling

DASE profiling was performed using Illumina’s HumanOmni1-Quad BeadChip SNP array platform (Illumina, San Diego, CA), which has more than one million SNP loci, including more than 120,000 SNPs in transcribed regions. For each HMEC, ds-cDNA (derived from 20–50 μg total RNA) and 200 ng gDNA were loaded to the BeadChip according to manufacturer’s instructions. Samples of gDNA and ds-cDNA to be used for the parallel genotyping and DASE profiling were denatured, neutralized and then underwent PCR-free whole-genome amplification followed by fragmentation according to the Infinium HD Assay Super Protocol. The ds-cDNA and gDNA pairs from each sample were individually hybridized to BeadChips and processed following standard Infinium procedures. Raw data from the assay was generated by scanning processed BeadChips using an iScan Reader. The scanned images were processed in the genotyping module (Ver. 3.3.7) of BeadStudio software (Ver. 3.1.3.0) to export a tab delimited file consisting of the SNP locus, the genotypes and quantified fluorescent signal intensities (*X*_*raw*_, *Y*_*raw*_). This genome-wide map illustrating the global DASE distribution was drawn using a visualization tool, Circos [49].

### Data filtering and statistical analysis

Raw data were filtered before DASE calculation. Firstly, data from CNV (copy number variation) markers, Y chromosomal markers and markers that are not located in transcribed regions were discarded. Secondly, to avoid possible false positives from background noise, a cut-off bar of combined signal intensities from the ds-cDNA sample (*Xraw* + *Yraw* ≥ 500) was imposed to filter out non-expressed SNPs. In addition, readings from SNP sites with ambiguous genotyping results were removed. For each sample, raw signal intensities corresponding to ds-cDNA and gDNA for each allele at each SNP site were background corrected. After these pre-processing steps, specific ds-cDNA allele intensities were normalized to their corresponding gDNA allele intensities to eliminate probe specific effects and potential variations occurring during BeadChip scanning. The DASE value between two alleles X and Y in a sample of a given SNP was then calculated as the absolute value of the normalized log_2_-ratio given by DASE = ABS(log_2_ [(*DX*_*raw*_/*GX*_*raw*_) / (*DY*_*raw*_/*GY*_*raw*_)]), which was also used by other groups [50]. Using the absolute DASE value (log_2_ ratio) for the computation enables us to quantify DASE based only on magnitude of change without regard to direction of change, as direction of change cannot be incorporated due to the lack of a standard reference allele. Without using the absolute DASE value, the averaged DASE would likely be neutralized in the gene-based approach described below. The distribution of DASE was determined to be gamma distributed using maximum likelihood methods and quantile-quantile plots. For each SNP, *p*-value is then calculated based on the fitted gamma distribution for DASE by testing the null hypothesis that mean DASE is zero against the two-sided alternative.

In our filtered dataset, we focused only on heterozygous individuals in assessing allelic imbalances. To assign DASE value to transcribed loci, we used two approaches in parallel. In the first approach, we extracted SNPs for which at least 3 out of the 8 HMECs were heterozygous. For each of these SNPs, their DASE values in heterozygous individuals were calculated separately and the average value was recorded. In the second approach, we extracted all the transcribed-region SNPs with heterozygous genotypes for each gene. The average DASE value was calculated for each corresponding gene, and only those genes with DASE values in at least 3 out of the 8 HMECs were included in final analysis. For each sample, DASE was determined to have a heavy right-tailed skewed distribution based on SNP-level data as well as gene-level data. The top panels of Additional file 5: Figure S3 displays the density of DASE for a typical sample determined using kernel density methods. Using maximum likelihood methods and quantile-quantile (QQ) plots, the distribution of DASE was determined to be approximately gamma distributed. The bottom panels of Additional file 5: Figure S3 display the QQ plot for a typical sample. A generalized linear model approach (based on gamma regression) was used to identify SNPs with mean DASE significantly different from zero. A *p*-value cut-off of 0.01 and a false discovery rate (FDR) cut-off of 0.05 were utilized to determine statistical significance of each SNP. FDR was calculated using the Benjamini-Hochberg step-up method to account for multiple testing [51]. Biological significance of each SNP was determined based on a mean DASE value of at least 2. A plot of *p*-value or FDR versus mean DASE enabled visualization of the relationship between statistical and biological significance. SNPs identified based on statistical significance as well as biological significance were interrogated for molecular pathways and biological function in bioinformatics analyses. This analysis was repeated on gene-level data obtained as outlined above. All computations were performed using the R statistical language and environment [52].

### Ingenuity pathway analysis (IPA)

Biological and interaction networks of candidate DASE loci were generated using IPA (Ingenuity® Systems). IPA explores the set of input genes to identify networks by using Ingenuity Pathways Knowledge Base for interactions between identified ‘Focus Genes’. For each network, IPA computes a score according to the fit of the user's set of significant genes. The score suggests the likelihood of the Focus Genes in a network from Ingenuity’s knowledge base being found together due to random chance. A score of 3 was used as the cutoff for identifying gene networks, which predicts that there is only a 1/1000 chance that the focus genes shown in a network are due to random chance. Therefore, a score of 3 or higher indicates a 99.9% confidence level to exclude random chance. In this study, the candidate gene list was uploaded into the application for biological function enrichment analysis, and networks of Network Eligible Molecules were then algorithmically generated based on their connectivity.

### DASE validation

Genomic and mRNA sequences flanking selected SNPs were retrieved from NCBI and primers were designed accordingly using the web-based Primer3 software (http://frodo.wi.mit.edu/primer3/). The sequences of primers are available upon request. PCR amplification was performed using GoTaq® Green Master Mix (Promega) and relevant gDNA and cDNA samples on a thermal cycler (Applied Biosystems, Model 2720) using the following program: 94°C 3 minutes for initial denaturing, followed by 10 cycles touchdown PCR (94°C 30 seconds, 65°C −55°C <−1°C / cycle> 30 seconds, 72°C 30 seconds) and 35 cycles regular PCR (94°C 30 seconds, 60°C 30 seconds, 72°C 30 seconds), final extension for 5 minutes at 72°C and then hold at 4°C. PCR product purification and Sanger sequencing were performed by Beckman Coulter Genomic Services (Danvers, MA). Sequencing trace files were analyzed using Sequencher software (v4.1.4., Gene Codes, MI). The DASE value between two alleles X and Y in a sample of a given SNP was then calculated using the peak height of each allele in the chromograms originated from cDNA samples, justified by that originated from genomic DNA samples. A positive DASE event by sequencing is defined when the height of the peak representing one allele is less than half of the peak height of the other allele. The fact that we chose a different threshold (DASE=1) for DASE validation by Sanger sequencing is justified by the different data dynamic ranges between these two platforms. The SNP array gives numeric results with a dynamic range of 2^16^. On the other hand, Sanger sequencing gives graphic trace files, and the usable peak-heights for quantification are usually within a few dozen pixels. Based on this dissimilarity, we chose different thresholds for DASE calling for each method.

### *DMBT1* 5′ RLM-RACE

The amplification of 5′ UTR region of *DMBT1* was performed using the FirstChoice RLM-RACE kit (Life Technologies) following the manufacture’s manual. In brief, a 5 μg RNA sample isolated from each HMEC was treated with calf intestine alkaline phosphatase (CIP) to remove 5′-phosphates from fragmented RNA ribosomal RNA and tRNA, followed by tobacco acid pyrophosphatase (TAP) treatment to remove the cap structure of intact mRNA. A 5′RACE RNA adapter was ligated to CIP/TAP treated RNA by T4 RNA ligase, and then reverse transcription was performed using random decamers. The resulting cDNAs were used as a template for PCR with a 5′ RACE Outer Primer (5′ *GCTGATGGCGATGAATGAACACTG* 3′, binds to 5′RACE adapter), and a gene specific primer *DMBT1*-5Ro (5′ *CTCAGGGCCAAACCAGAA* 3′) complementary to the region (+288, +308) of the *DMBT1* cDNA. A nested PCR was performed with a 5′ RACE Inner Primer (5′ *CGCGGATCCGAACACTGCGTTTGCTGGCTTTGATG* 3′, binds the 5′RACE adapter) and a *DMBT1*-5Ri (5′ *GGTTGACTCCAAGGAAATCG* 3′) primer, complementary to the region (+194, +213) of the *DMBT1* cDNA. The PCR products were purified and sequenced using *DMBT1*-5Ri.

## Competing interests

The authors declare that they have no competing interests.

## Authors’ contributions

CG and XC carried out the original study design, global DASE analysis and validation studies, and drafted the manuscript. KD participated in the design of the study and performed the statistical analysis and YZ performed IPA analysis. CS established the HMEC lines and isolated RNAs and DNAs for the array analysis. MD participated in the participated in study design and helped to draft the manuscript. All authors read and approved the final manuscript.

## Funding

This work was kindly supported by the Susan G. Komen for the Cure (KG100274 to X.C.), the Eileen Stein Jacoby Fund, and the Risk Assessment and Presentation Keystone Program at Fox Chase Cancer Center.

## Supplementary Material

Additional file 1 **Table S1.** Global DASE analysis. Click here for file

Additional file 2 **Table S2.** Functional analysis by IPA. Click here for file

Additional file 3 **Figure S1.** Sequencing analysis of *DMBT1* 3′UTRs. Click here for file

Additional file 4 **Figure S2.** Predictions of microRNA-targeting at *DMBT1* 3′UTR. Click here for file

Additional file 5 **Figure S3.** Distribution of DASE. Click here for file
