# Supplementary material for: Identifying breast cancer risk loci by global differential allele-specific expression (DASE) analysis in mammary epithelial transcriptome
Source: BMC Genomics. 2012 Oct 30;13:570. doi: 10.1186/1471-2164-13-570 (PMC3532379; doi:10.1186/1471-2164-13-570)
Supplement: Additional file 2 — Table S2. Functional analysis by IPA. [file 1471-2164-13-570-S2.pdf]

Table S2. Function analysis by IPA

**Biological Function**	**Molecules**	***P*-value**
Dermatological Diseases and Conditions	ZNF331,SLAMF1,NLRP1,HLA-G,ACPP,GRIN3A	7.49E-04-3.85E-02
Immunological Disease	ZNF331,HCG4,FLJ39061,ADAMTSL3,TMCC3,AGBL1,NLRP1,HLA-G,XIAP,ACPP	7.49E-04-2.26E-02
Cell Death	SLAMF1,WNT3A,NLRP1,PRDM14,HLA-G,XIAP,GRIN3A	1.51E-03-4.86E-02
Cell Morphology	SLAMF1,WNT3A,HLA-G,GRIN3A,DMBT1	1.51E-03-2.68E-02
Cellular Compromise	SLAMF1,XIAP,GRIN3A	1.51E-03-4.14E-02
Cellular Growth and Proliferation	WNT3A,SLAMF1,PRDM14,HLA-G,XIAP,ACPP	1.51E-03-4.96E-02
Cellular Movement	WNT3A,XIAP,DMBT1	1.51E-03-2.83E-02
Embryonic Development	MMP20,WNT3A,PRDM14,XIAP,DMBT1	1.51E-03-4.86E-02
Genetic Disorder	ZNF331,MMP20,ADAMTSL3,SLAMF1,NLRP1,AGBL1,FLJ42393,XIAP,DMBT1,ACPP,GRIN3A	1.51E-03-3.85E-02
Hair and Skin Development and Function	WNT3A	1.51E-03-1.5E-02
Hematological System Development and Function	WNT3A,SLAMF1,HLA-G,DMBT1	1.51E-03-4.27E-02
Immune Cell Trafficking	DMBT1	1.51E-03-1.51E-03
Nervous System Development and Function	WNT3A,GRIN3A	1.51E-03-2.68E-02
Neurological Disease	ZNF331,NLRP1,AGBL1,XIAP,FLJ42393,ACPP,DMBT1,GRIN3A	1.51E-03-2.97E-02
Organ Development	MMP20,WNT3A	1.51E-03-4.86E-02
Organismal Development	MMP20,WNT3A,PRDM14,XIAP	1.51E-03-4.86E-02
Organismal Injury and Abnormalities	XIAP,GRIN3A	1.51E-03-3.85E-02
Tissue Development	MMP20,WNT3A,PRDM14,DMBT1	1.51E-03-4.86E-02
Tissue Morphology	MMP20,WNT3A	1.51E-03-1.35E-02
Tumor Morphology	WNT3A,XIAP	1.51E-03-4.96E-02
Cancer	MUC16,NLRP1,PRDM14,XIAP,GRIN3A,DMBT1,MAGEC2 (includes others)	3.01E-03-4.66E-02
Cell Cycle	GRK4,WNT3A	3.01E-03-2.46E-02
Cellular Assembly and Organization	WNT3A,CHRAC1,HLA-G,XIAP	3.01E-03-3.7E-02
Cellular Development	SLAMF1,WNT3A,PRDM14,HLA-G,XIAP,ACPP,DMBT1	3.01E-03-4.88E-02
Cellular Function and Maintenance	WNT3A,SLAMF1,HLA-G,GRIN3A	3.01E-03-2.68E-02
Connective Tissue Development and Function	WNT3A	3.01E-03-4.52E-03
Dental Disease	MMP20	3.01E-03-3.01E-03
Developmental Disorder	MMP20,GRIN3A	3.01E-03-1.5E-02
Gastrointestinal Disease	MMP20,ADAMTSL3,AGBL1,GRIN3A,DMBT1,ACPP	3.01E-03-3.7E-02
Humoral Immune Response	WNT3A,SLAMF1	3.01E-03-4.27E-02
Infectious Disease	FLJ39061,SLAMF1,NLRP1,PRDM14,USP6,XIAP,DMBT1,GRIN3A	3.01E-03-3.85E-02
Skeletal and Muscular Disorders	ZNF331,MMP20,ADAMTSL3,WNT3A,AGBL1,HLA-G,XIAP,ACPP	3.01E-03-3.01E-02
Skeletal and Muscular System Development and Function	WNT3A	3.01E-03-4.57E-02
Cardiovascular System Development and Function	WNT3A,XIAP	4.52E-03-4.86E-02
Digestive System Development and Function	MMP20	4.52E-03-6.02E-03
Antimicrobial Response	NLRP1,HLA-G,DMBT1	6.02E-03-7.55E-03
Respiratory Disease	XIAP,GRIN3A	7.52E-03-1.65E-02
Inflammatory Response	NLRP1,HLA-G	7.55E-03-4.57E-02
Cell-To-Cell Signaling and Interaction	SLAMF1,HLA-G,GRIN3A,DMBT1	7.72E-03-3.12E-02
Psychological Disorders	ZNF331,NLRP1,AGBL1,FLJ42393,ACPP,GRIN3A	7.99E-03-3.85E-02
Hematopoiesis	WNT3A,SLAMF1,HLA-G,DMBT1	9.01E-03-2.09E-02
Auditory Disease	GRIN3A	1.05E-02-1.05E-02
Cell-mediated Immune Response	SLAMF1,HLA-G	1.05E-02-1.8E-02
Lymphoid Tissue Structure and Development	SLAMF1,HLA-G	1.05E-02-1.8E-02
Reproductive System Disease	MUC16,NLRP1,PRDM14,XIAP,GRIN3A,DMBT1,MAGEC2 (includes others)	1.05E-02-4.66E-02
Cellular Response to Therapeutics	XIAP	1.2E-02-1.2E-02
Ophthalmic Disease	GRIN3A	1.2E-02-1.2E-02
Renal and Urological Disease	GRIN3A	1.8E-02-1.8E-02
Reproductive System Development and Function	WNT3A,PRDM14	1.94E-02-4.43E-02
Gene Expression	WNT3A	2.09E-02-2.09E-02
Inflammatory Disease	ZNF331,ADAMTSL3,WNT3A,AGBL1,HLA-G,XIAP,GRIN3A,DMBT1,ACPP	2.09E-02-3.7E-02
Behavior	ACPP	2.39E-02-2.39E-02
DNA Replication, Recombination, and Repair	CHRAC1	2.53E-02-2.53E-02
Carbohydrate Metabolism	ACPP	2.83E-02-2.83E-02
Lipid Metabolism	ACPP	2.83E-02-2.83E-02
Molecular Transport	ACPP	2.83E-02-2.83E-02
Small Molecule Biochemistry	ACPP	2.83E-02-2.83E-02
Cardiovascular Disease	GRK4,TMCC3,OR6N1,ACPP,C17orf46,GRIN3A	2.99E-02-3.12E-02
Connective Tissue Disorders	ZNF331,ADAMTSL3,WNT3A,AGBL1,HLA-G,XIAP,ACPP	3.01E-02-3.01E-02
Nutritional Disease	GRIN3A	3.56E-02-3.56E-02
Post-Translational Modification	USP6	4.29E-02-4.29E-02
Endocrine System Development and Function	WNT3A	4.86E-02-4.86E-02
Organ Morphology	WNT3A	4.86E-02-4.86E-02
