# Supplementary material for: Identifying breast cancer risk loci by global differential allele-specific expression (DASE) analysis in mammary epithelial transcriptome
Source: BMC Genomics. 2012 Oct 30;13:570. doi: 10.1186/1471-2164-13-570 (PMC3532379; doi:10.1186/1471-2164-13-570)
Supplement: Additional file 3 — Figure S1. Sequencing analysis of DMBT1 3′UTRs. [file 1471-2164-13-570-S3.pdf]

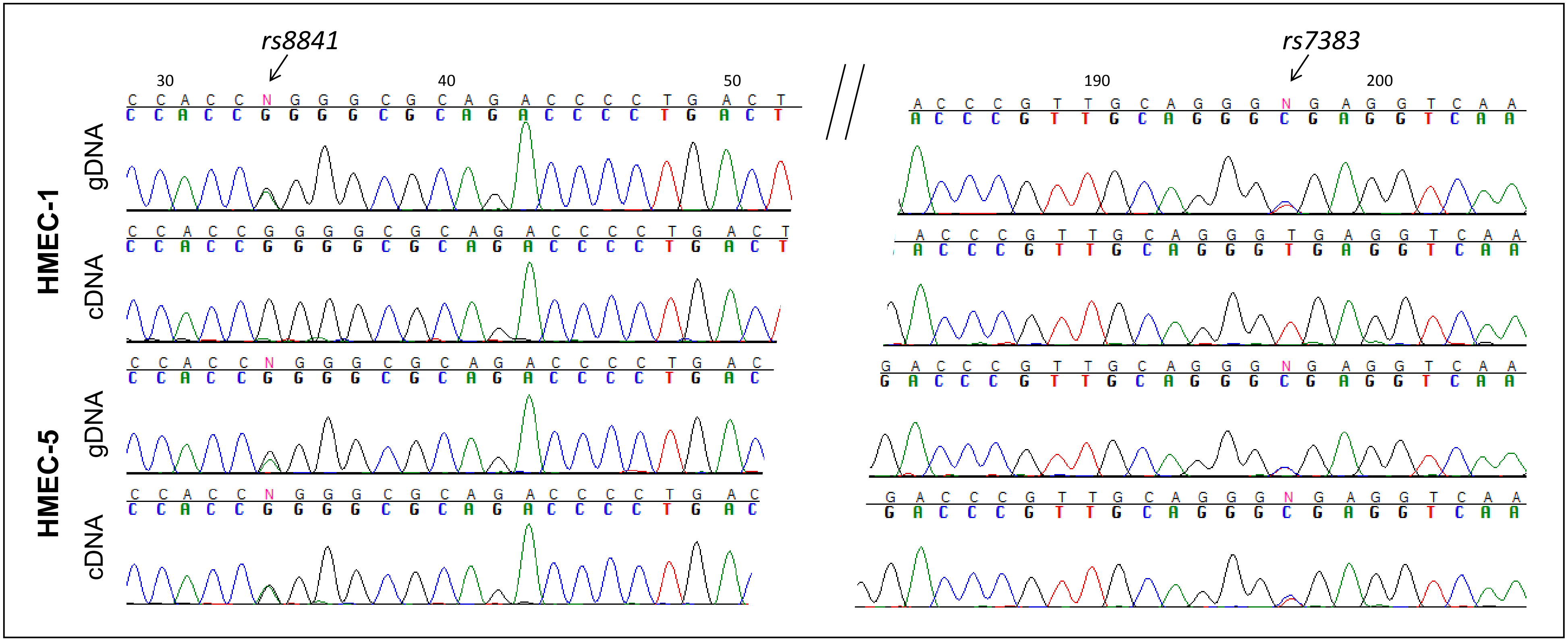

Figure S1. Sequencing analysis of *DMBT1* 3'UTRs. We sequenced 3'UTR regions in genomic DNAs of all 8 HMECs and identified two SNPs, *rs8441* and *rs7383*, both presented in samples HMEC-1 and -5. We then sequenced the same region using cDNAs isolated from these two HMEC lines. The results revealed a DASE expression pattern for *DMBT1* in HMEC-1 and a bi-allelic expression pattern in HMEC-5.
