# Supplementary material for: Identifying breast cancer risk loci by global differential allele-specific expression (DASE) analysis in mammary epithelial transcriptome
Source: BMC Genomics. 2012 Oct 30;13:570. doi: 10.1186/1471-2164-13-570 (PMC3532379; doi:10.1186/1471-2164-13-570)
Supplement: Additional file 4 — Figure S2. Predictions of microRNA-targeting at DMBT1 3′UTR. [file 1471-2164-13-570-S4.pdf]

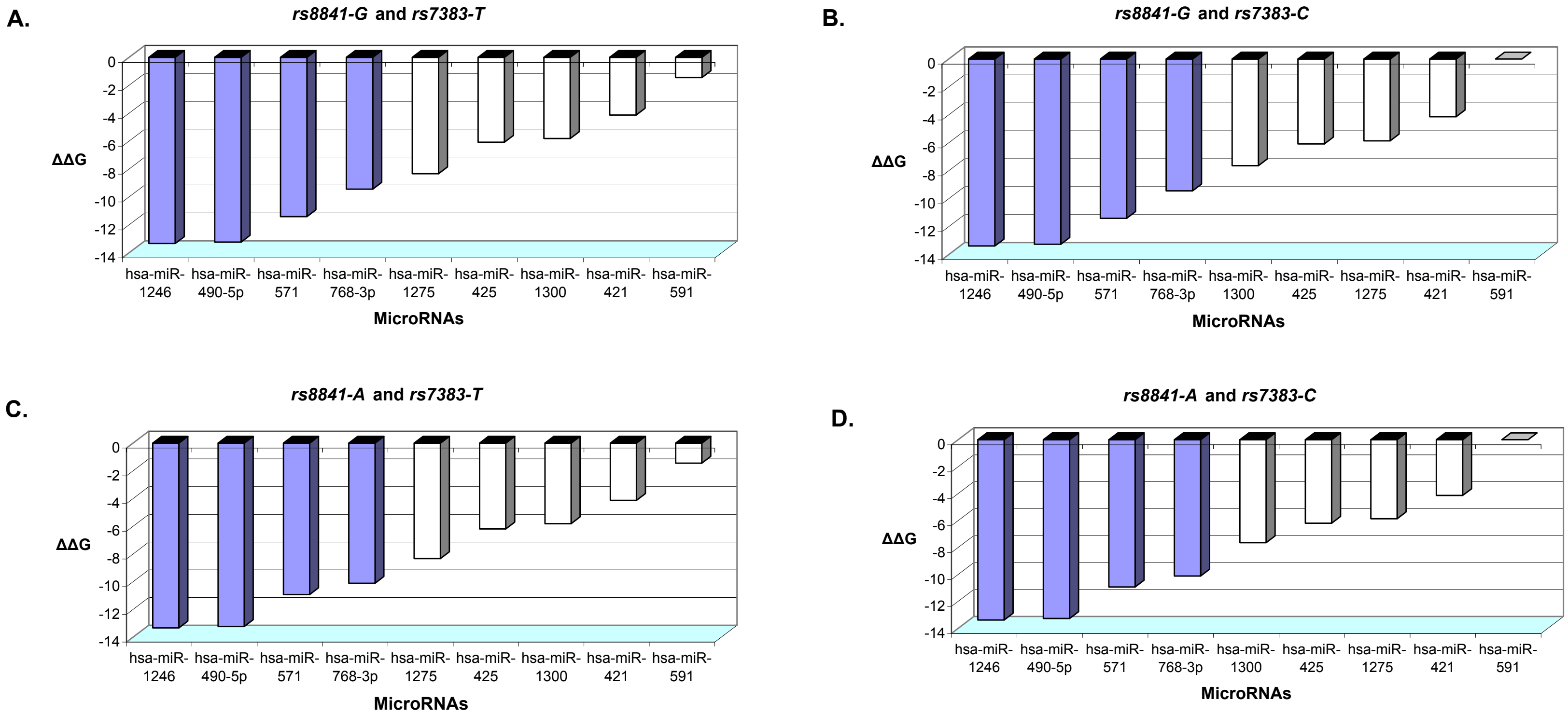

Figure S2. Predictions of microRNA-targeting at DMBT1 3'UTR. An online miRNA-targeting tool, Probability of Interaction by Target Accessibility (PITA - http://genie.weizmann.ac.il/pubs/mir07/mir07_dyn_data.html), was utilized to compare the effects of the variants at 3'UTR of *DMBT1* on miRNA binding. Moderate search stringency (e.g., minimum seed size = 7, no single mismatch or G:U wobble allowed) were applied for the analysis. Predicted miRNAs with ΔΔG values below -10 are highlighted in blue and are likely to be functional in endogenous microRNA expression levels *(**A,B,C,D:** Different genotype combinations of two SNPs, rs8841 and rs7383, which were identified in sample HMEC-1 and -5).*
