# Supplementary material for: Identifying breast cancer risk loci by global differential allele-specific expression (DASE) analysis in mammary epithelial transcriptome
Source: BMC Genomics. 2012 Oct 30;13:570. doi: 10.1186/1471-2164-13-570 (PMC3532379; doi:10.1186/1471-2164-13-570)

Figure S3. Distribution of DASE. The top panels of the figure display the probability density function of DASE plotted against DASE for a typical sample. It shows a heavy tight-tailed distribution based on SNP-level data (**A**) as well as gene-level data (**B**). The bottom panels of the figure display the quantiles of the estimated gamma distribution of DASE, determined using maximum likelihood methods, plotted against the quantiles of DASE for a typical sample based on SNP-level data (**C**) and gene-level data (**D**).

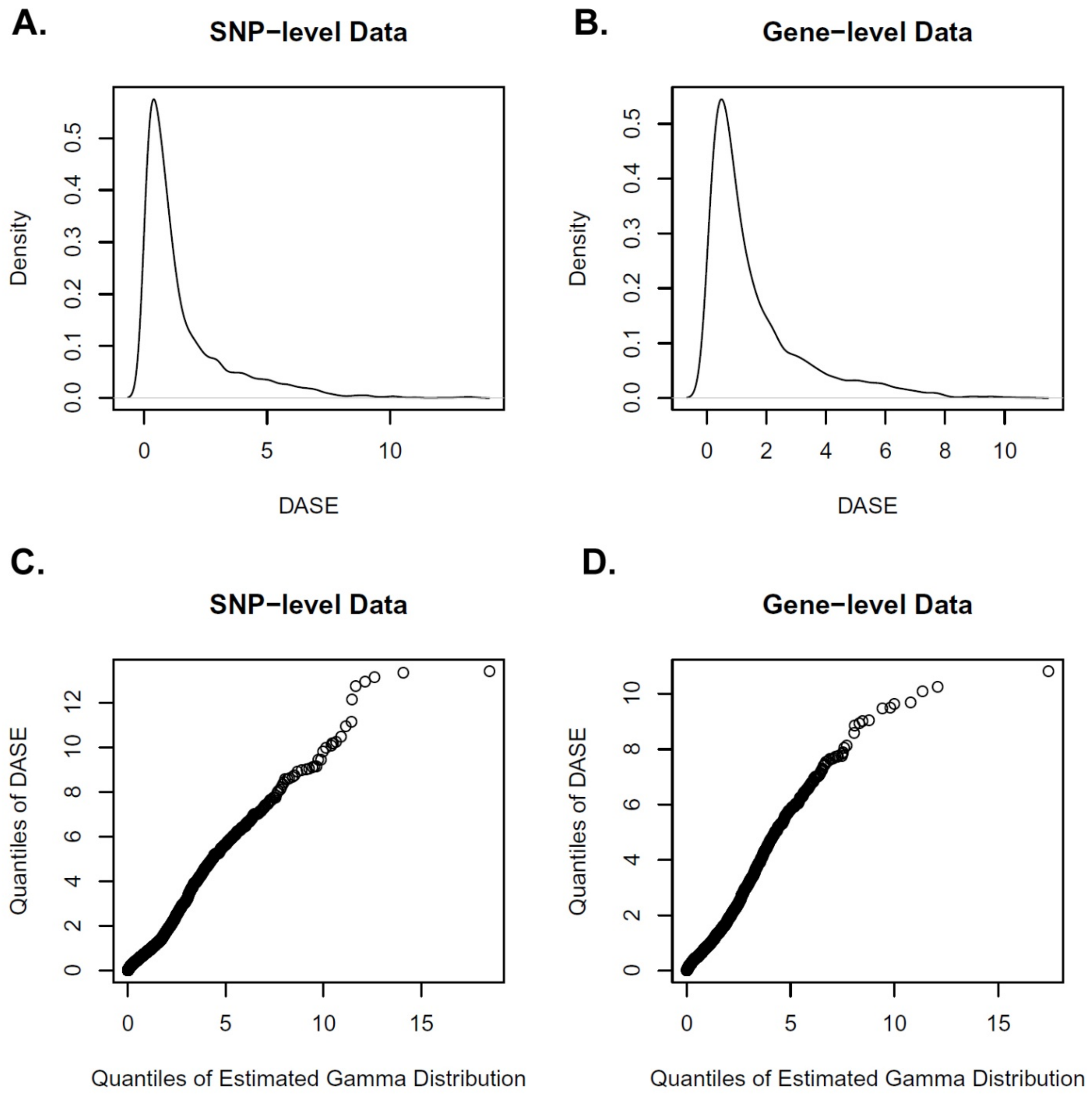

Supplement: Additional file 5 — Figure S3. Distribution of DASE. [file 1471-2164-13-570-S5.pdf]
