# Supplementary material for: Identifying breast cancer risk loci by global differential allele-specific expression (DASE) analysis in mammary epithelial transcriptome
Source: BMC Genomics. 2012 Oct 30;13:570. doi: 10.1186/1471-2164-13-570 (PMC3532379; doi:10.1186/1471-2164-13-570)
Supplement: Additional file 1 — Table S1. Global DASE analysis. [file 1471-2164-13-570-S1.pdf]

Table S1. Global DASE analysis

SNP ID	gene_symbol	chr	coordinate	Sample ID	DASE
rs3748597	NOC2L	1	878522	8	0.56472
rs4970441	KLHL17	1	887188	8	0.148737
rs41285812	PLEKHN1	1	895985	2	2.499262
rs28561399	PLEKHN1	1	900336	2	1.857875
rs2340592	C1orf170	1	900798	2	4.524085
rs2340592	C1orf170	1	900798	4	0.52439
GA000031	AGRN	1	980243	1	2.878857
GA000031	AGRN	1	980243	4	4.225445
GA000031	AGRN	1	980243	3	7.423973
rs3766191	C1orf159	1	1007450	4	0.765256
rs3766191	C1orf159	1	1007450	1	4.153483
rs4074137	C1orf159	1	1016570	1	1.269004
rs4074137	C1orf159	1	1016570	2	1.456134
rs1320571	TTLL10	1	1110294	8	2.416536
rs3813204	TTLL10	1	1110877	7	0.645796
rs12036216	SDF4	1	1142976	6	0.811158
rs12093154	FAM132A	1	1168788	8	3.697012
rs12093154	FAM132A	1	1168788	6	2.961987
GA018415	MIB2	1	1541790	7	0.479041
GA018415	MIB2	1	1541790	2	6.521957
rs12755088	MIB2	1	1548655	4	1.145726
rs12755088	MIB2	1	1548655	3	0.407419
SNP1-1628854	CDC2L2	1	1628854	4	6.716753
SNP1-1675900	NADK	1	1675900	6	0.42066
SNP1-1675900	NADK	1	1675900	1	0.437761
rs1884455	TMEM52	1	1839604	4	4.944524
rs3820011	KIAA1751	1	1878053	1	1.386055
SNP1-1890046	KIAA1751	1	1890046	6	1.375868
SNP1-1890046	KIAA1751	1	1890046	1	0.013181
SNP1-1890046	KIAA1751	1	1890046	2	0.671798
SNP1-1890046	KIAA1751	1	1890046	7	1.623036
rs16824588	KIAA1751	1	1890092	6	0.039202
rs16824588	KIAA1751	1	1890092	3	1.324634
rs16824588	KIAA1751	1	1890092	2	1.151325
rs16824588	KIAA1751	1	1890092	1	0.059151
rs12184	PRKCZ	1	1977853	5	1.084595
rs12184	PRKCZ	1	1977853	3	0.161011
rs12184	PRKCZ	1	1977853	1	0.42942
rs3123595	C1orf86	1	2107418	2	3.160142
rs34587196	MORN1	1	2280003	5	5.776355
rs7512269	PLCH2	1	2408485	1	5.530447
rs2296442	PLCH2	1	2420477	3	0.126792

rs2296442	PLCH2	1	2420477	8	2.264457
rs2296442	PLCH2	1	2420477	1	1.130557
rs2296442	PLCH2	1	2420477	6	3.462337
rs2296442	PLCH2	1	2420477	2	0.280803
rs2296442	PLCH2	1	2420477	4	0.49238
rs2494620	PANK4	1	2431218	7	0.613003
rs2494620	PANK4	1	2431218	2	0.741784
rs2494620	PANK4	1	2431218	1	0.560575
SNP1-2486265	TNFRSF14	1	2486265	8	0.282896
SNP1-2486265	TNFRSF14	1	2486265	2	0.782545
SNP1-2486265	TNFRSF14	1	2486265	5	0.994711
SNP1-2486265	TNFRSF14	1	2486265	3	1.141057
SNP1-2516606	MMEL1	1	2516606	6	1.836374
SNP1-2516606	MMEL1	1	2516606	5	5.181309
SNP1-2516606	MMEL1	1	2516606	4	0.264026
SNP1-2525473	MMEL1	1	2525473	6	2.070887
SNP1-2525473	MMEL1	1	2525473	5	2.327321
SNP1-2525473	MMEL1	1	2525473	2	3.078952
rs4576609	ACTRT2	1	2928125	6	0.285804
rs4576609	ACTRT2	1	2928125	7	0.619722
rs4576609	ACTRT2	1	2928125	3	0.509031
rs4576609	ACTRT2	1	2928125	2	0.869806
rs3795262	ACTRT2	1	2928557	3	0.649376
rs3795262	ACTRT2	1	2928557	7	1.309358
rs3795263	ACTRT2	1	2928849	1	0.626988
rs3795263	ACTRT2	1	2928849	3	0.019215
rs2236518	PRDM16	1	3342732	5	1.093587
rs2236518	PRDM16	1	3342732	4	0.050629
rs2821039	MEGF6	1	3396867	1	4.02201
SNP1-3400270	MEGF6	1	3400270	1	4.186529
rs7553399	MEGF6	1	3406309	1	2.717446
rs7553399	MEGF6	1	3406309	5	0.385063
SNP1-3407071	MEGF6	1	3407071	4	0.626121
SNP1-3407071	MEGF6	1	3407071	1	3.854645
SNP1-3414248	MEGF6	1	3414248	4	5.318592
SNP1-3414248	MEGF6	1	3414248	1	6.033573
SNP1-3418468	MEGF6	1	3418468	1	5.934405
SNP1-3418468	MEGF6	1	3418468	4	1.779521
SNP1-3501794	MEGF6	1	3501794	3	0.639749
rs2296034	WDR8	1	3541652	2	4.942978
rs1801174	TP73	1	3628534	6	0.812858
rs3903158	KIAA0495	1	3645396	2	5.035084
rs2298223	KIAA0495	1	3649602	2	3.974553
rs2275819	LOC388588	1	3679268	7	0.108005
rs2275831	KIAA0562	1	3735784	2	5.216241
rs2275822	KIAA0562	1	3751339	1	2.257394
rs2275822	KIAA0562	1	3751339	3	0.144909

rs1061968	AJAP1	1	4671913	7	0.915112
rs389959	AJAP1	1	4734466	1	0.963731
rs389959	AJAP1	1	4734466	5	0.796145
rs41280800	NPHP4	1	5855971	7	0.930259
rs1287637	NPHP4	1	5857749	7	1.484777
rs1287637	NPHP4	1	5857749	8	1.721133
rs3747992	NPHP4	1	5859755	3	0.872454
rs3747992	NPHP4	1	5859755	6	0.034682
rs3747992	NPHP4	1	5859755	4	2.362749
rs3747992	NPHP4	1	5859755	5	0.6942
rs3747992	NPHP4	1	5859755	7	3.658478
rs3747992	NPHP4	1	5859755	8	11.32615
rs3747990	NPHP4	1	5859914	3	0.591589
rs12120967	NPHP4	1	5887968	3	0.156201
rs3205229	KCNAB2	1	6081149	7	0.742856
rs3205229	KCNAB2	1	6081149	4	0.020627
rs3205229	KCNAB2	1	6081149	3	0.856258
rs3205229	KCNAB2	1	6081149	1	0.311402
rs3827728	CHD5	1	6084994	5	0.936373
rs3827728	CHD5	1	6084994	6	1.270992
rs3827728	CHD5	1	6084994	8	0.227351
rs3810993	CHD5	1	6085170	7	0.57242
rs3810993	CHD5	1	6085170	3	0.11855
rs3810990	CHD5	1	6085232	3	1.337288
rs11587	CHD5	1	6085728	7	0.108682
rs11587	CHD5	1	6085728	2	4.499495
rs2092507	CHD5	1	6086995	5	2.554026
rs1883768	CHD5	1	6088006	3	1.750109
rs1883768	CHD5	1	6088006	1	3.660076
SNP1-6106679	CHD5	1	6106679	8	0.422417
SNP1-6106679	CHD5	1	6106679	7	0.448981
SNP1-6106679	CHD5	1	6106679	1	1.184947
rs2250358	CHD5	1	6119456	7	5.172811
rs2250358	CHD5	1	6119456	8	0.030451
rs2250358	CHD5	1	6119456	1	0.794542
rs2250358	CHD5	1	6119456	6	1.508407
rs2273032	CHD5	1	6124832	1	3.42943
rs2273032	CHD5	1	6124832	3	0.908387
rs17436816	CHD5	1	6126748	2	3.523067
rs12074369	CHD5	1	6138323	6	0.4445
rs4908540	RNF207	1	6188683	5	0.33243
SNP1-6195793	RNF207	1	6195793	6	0.396878
rs846111	NULL	1	6201957	5	1.109897
rs846111	NULL	1	6201957	3	2.018877
rs846111	NULL	1	6201957	2	2.489437
rs1802353	ICMT	1	6207267	6	4.925378
SNP1-6236525	GPR153	1	6236525	5	0.456303

SNP1-6236525	GPR153	1	6236525	7	0.37633
SNP1-6236525	GPR153	1	6236525	4	1.0002
SNP1-6236525	GPR153	1	6236525	2	0.699331
rs11590458	GPR153	1	6237409	2	3.849214
rs11590458	GPR153	1	6237409	4	0.241895
rs11590458	GPR153	1	6237409	5	5.514804
rs11800462	TNFRSF25	1	6447088	6	1.954419
SNP1-6451055	PLEKHG5	1	6451055	8	0.258739
GA000154	PLEKHG5	1	6451775	8	6.042107
GA000154	PLEKHG5	1	6451775	6	8.352664
rs1556035	PLEKHG5	1	6502194	1	0.041026
rs1556035	PLEKHG5	1	6502194	7	0.014908
rs1010584	PLEKHG5	1	6502430	7	0.740775
rs1010584	PLEKHG5	1	6502430	1	0.485673
rs4908923	NOL9	1	6537122	2	0.392673
rs4908923	NOL9	1	6537122	1	0.567503
rs17029626	ZBTB48	1	6562703	6	1.058335
rs17029626	ZBTB48	1	6562703	4	0.081791
rs12727844	THAP3	1	6616794	3	0.776403
rs12727844	THAP3	1	6616794	7	0.814923
rs12727844	THAP3	1	6616794	1	3.226297
rs200458	DNAJC11	1	6627222	4	0.097287
rs12137794	DNAJC11	1	6628531	1	4.377498
rs1044245	CAMTA1	1	7751694	3	0.34843
rs1044245	CAMTA1	1	7751694	4	0.08396
rs17031614	PER3	1	7810192	6	5.064187
rs2640908	PER3	1	7812528	5	0.144008
rs2640908	PER3	1	7812528	2	5.539337
rs13306061	UTS2	1	7836032	3	0.559651
rs2289731	SLC45A1	1	8313475	1	1.189276
rs2289731	SLC45A1	1	8313475	8	1.254287
rs2289731	SLC45A1	1	8313475	3	1.054156
rs7535752	SLC45A1	1	8318147	1	6.462673
rs7535752	SLC45A1	1	8318147	3	0.832789
rs5840	SLC2A5	1	9019828	6	0.213875
rs5840	SLC2A5	1	9019828	2	2.863323
rs5840	SLC2A5	1	9019828	5	1.110569
rs5840	SLC2A5	1	9019828	3	0.261001
rs1063137	SLC2A5	1	9019838	5	1.204296
rs1063137	SLC2A5	1	9019838	6	0.52127
rs1063137	SLC2A5	1	9019838	3	0.474811
rs1063137	SLC2A5	1	9019838	8	1.787547
SNP1-9088272	GPR157	1	9088272	5	0.418322
SNP1-9088272	GPR157	1	9088272	3	0.174712
SNP1-9088272	GPR157	1	9088272	4	0.167992
SNP1-9088272	GPR157	1	9088272	8	4.373002
rs6688832	H6PD	1	9246497	3	0.349709

rs1294014	H6PD	1	9248263	8	3.573358
rs1294014	H6PD	1	9248263	4	1.900095
rs1294014	H6PD	1	9248263	5	0.643024
rs1294014	H6PD	1	9248263	2	4.805674
rs1294014	H6PD	1	9248263	3	0.613023
rs11121356	H6PD	1	9253066	2	7.299106
rs1135426	PIK3CD	1	9710095	7	1.002787
rs1135427	PIK3CD	1	9710427	7	1.051177
rs1135427	PIK3CD	1	9710427	8	0.893924
rs1135427	PIK3CD	1	9710427	1	0.773466
rs1135427	PIK3CD	1	9710427	2	1.200854
rs1135427	PIK3CD	1	9710427	4	1.467575
rs1135427	PIK3CD	1	9710427	3	0.90995
rs1220394	CTNNBIP1	1	9832093	3	0.719937
rs2297881	KIF1B	1	10320154	5	0.8805
SNP1-10395787	PGD	1	10395787	5	5.615436
rs649639	APITD1	1	10412872	5	1.407382
rs649639	APITD1	1	10412872	1	1.3426
rs649639	APITD1	1	10412872	6	1.455235
rs1307497	APITD1	1	10434643	6	0.22575
rs284322	CASZ1	1	10648085	6	8.546347
rs284322	CASZ1	1	10648085	4	0.945104
SNP1-10936705	C1orf127	1	10936705	3	1.503024
SNP1-10936705	C1orf127	1	10936705	7	1.186139
SNP1-10936705	C1orf127	1	10936705	6	1.053228
SNP1-10936705	C1orf127	1	10936705	2	1.691893
rs28991014	ANGPTL7	1	11177825	6	0.390171
SNP1-11483751	PTCHD2	1	11483751	5	1.456953
rs2817580	PTCHD2	1	11484180	5	0.93806
rs2594310	PTCHD2	1	11519042	5	0.545331
rs2745294	PTCHD2	1	11519499	5	1.444469
rs1063301	FBXO6	1	11656636	7	0.80131
rs1063301	FBXO6	1	11656636	5	0.180031
rs1063301	FBXO6	1	11656636	4	1.164733
rs1063301	FBXO6	1	11656636	3	0.09728
rs4845881	C1orf167	1	11750906	1	0.390856
rs4845881	C1orf167	1	11750906	5	0.59242
rs1537516	MTHFR	1	11770448	6	6.129332
rs198411	CLCN6	1	11822972	5	7.877903
rs5068	NPPA	1	11828561	6	1.693825
rs5067	NPPA	1	11828568	5	1.080513
rs5065	NPPA	1	11828655	5	1.262609
rs4846067	LOC390997	1	11862853	5	0.420124
rs6684539	RNU5E	1	11890860	7	1.465174
rs6684539	RNU5E	1	11890860	6	1.1198
rs6684539	RNU5E	1	11890860	1	0.975792
rs7551175	PLOD1	1	11932543	4	1.062118

rs2273285	PLOD1	1	11933056	4	6.18981
rs7514577	PLOD1	1	11946822	1	3.239286
rs7514577	PLOD1	1	11946822	5	0.128403
rs7514577	PLOD1	1	11946822	3	0.159054
rs7514577	PLOD1	1	11946822	4	0.037663
rs945439	TNFRSF1B	1	12171529	6	0.213683
rs945439	TNFRSF1B	1	12171529	2	1.491504
rs945439	TNFRSF1B	1	12171529	4	1.487447
rs945439	TNFRSF1B	1	12171529	8	1.690851
rs1061628	TNFRSF1B	1	12190586	8	2.609024
rs1061628	TNFRSF1B	1	12190586	4	1.687961
rs5746073	TNFRSF1B	1	12191224	3	1.702549
rs5746073	TNFRSF1B	1	12191224	1	5.0464
rs4845898	VPS13D	1	12262206	3	0.099478
rs4845898	VPS13D	1	12262206	1	7.540184
rs4845898	VPS13D	1	12262206	5	5.213334
rs2295338	VPS13D	1	12352198	8	7.483558
rs3000859	AADACL3	1	12698931	1	2.030772
SNP1-12702205	AADACL3	1	12702205	4	2.099899
rs11121969	AADACL3	1	12708336	2	1.507744
rs355025	PDPN	1	13783004	3	3.012375
rs2495061	PRDM2	1	13904319	1	0.212518
rs2495061	PRDM2	1	13904319	3	0.028389
rs3795753	PRDM2	1	13904343	2	4.423692
rs3795753	PRDM2	1	13904343	1	0.634532
rs3795753	PRDM2	1	13904343	3	7.342802
rs17393663	PRDM2	1	13904402	7	0.693236
rs17393663	PRDM2	1	13904402	1	4.904305
rs1046331	PRDM2	1	14015590	2	4.126556
rs3820012	PRDM2	1	14022725	8	7.457534
rs2697962	PRDM2	1	14023579	2	5.071482
rs1055412	NULL	1	15313862	1	3.14845
rs1055412	NULL	1	15313862	7	0.311785
rs910105	NULL	1	15314513	5	0.98457
rs910105	NULL	1	15314513	1	5.402727
rs910105	NULL	1	15314513	7	0.044273
rs2235789	NULL	1	15314645	8	2.693133
rs2235789	NULL	1	15314645	5	0.30497
rs2235789	NULL	1	15314645	1	3.84175
rs1476356	C1orf126	1	15315955	5	6.072625
rs2235787	C1orf126	1	15316063	8	0.18528
rs2235787	C1orf126	1	15316063	1	5.799003
rs2235787	C1orf126	1	15316063	7	0.46491
rs2235786	C1orf126	1	15316446	5	0.160726
rs2235786	C1orf126	1	15316446	7	0.443091
rs2235786	C1orf126	1	15316446	1	4.656317
rs2235783	NULL	1	15317074	1	2.889704

rs2235783	NULL	1	15317074	7	0.289691
rs3753318	TMEM51	1	15413985	8	0.497947
rs3753318	TMEM51	1	15413985	7	1.021792
rs3753318	TMEM51	1	15413985	4	0.376223
rs3753315	TMEM51	1	15413999	8	4.735782
rs3753315	TMEM51	1	15413999	4	3.103173
rs3753315	TMEM51	1	15413999	7	3.361575
rs10489962	FHAD1	1	15512168	3	0.993213
rs3765357	FHAD1	1	15526150	6	0.227377
rs12126178	FHAD1	1	15540906	1	0.592496
rs4661330	FHAD1	1	15559646	3	1.667516
rs4661330	FHAD1	1	15559646	5	0.590645
rs4661330	FHAD1	1	15559646	6	0.115749
rs2294490	FHAD1	1	15596430	5	0.556205
rs2294490	FHAD1	1	15596430	4	0.591557
rs3820071	ELA2B	1	15681354	1	5.154461
rs3766160	ELA2B	1	15681459	1	4.761135
rs4645983	CASP9	1	15723190	1	4.333454
rs9298	DNAJC16	1	15769607	1	4.438204
rs6700212	AGMAT	1	15771843	5	0.176851
rs16851855	LOC645317	1	15803456	1	4.885599
rs16851855	LOC645317	1	15803456	8	2.474328
rs16851855	LOC645317	1	15803456	5	3.385616
rs848209	SPEN	1	16128594	7	3.871632
rs848209	SPEN	1	16128594	3	0.195976
rs848209	SPEN	1	16128594	6	6.502294
rs1763617	C1orf64	1	16203403	3	0.354202
rs872222	HSPB7	1	16217251	3	0.403403
rs945419	HSPB7	1	16217445	7	0.451846
rs1805152	CLCNKA	1	16229088	7	0.030219
SNP1-16250587	CLCNKB	1	16250587	6	0.879915
SNP1-16250587	CLCNKB	1	16250587	4	0.189997
rs5252	CLCNKB	1	16250802	6	0.559994
rs3754334	EPHA2	1	16324354	1	3.247263
rs221058	ARHGEF19	1	16407233	5	0.730015
rs221058	ARHGEF19	1	16407233	8	0.813544
rs525409	SPATA21	1	16604097	5	0.079197
rs525409	SPATA21	1	16604097	6	3.558236
rs525409	SPATA21	1	16604097	8	0.132463
rs525409	SPATA21	1	16604097	1	4.528803
rs157248	NULL	1	16690264	7	0.372418
rs157248	NULL	1	16690264	1	0.51263
rs15786	ATP13A2	1	17185179	3	0.149514
rs2235926	PADI2	1	17267868	5	6.405509
rs2235926	PADI2	1	17267868	8	6.951801
rs2235926	PADI2	1	17267868	3	0.220655
rs11581590	PADI2	1	17269179	4	7.520936

rs11203297	PADI2	1	17291611	3	0.10278
rs11203297	PADI2	1	17291611	5	0.589666
rs3750300	PADI3	1	17458721	1	2.361665
rs2272629	PADI3	1	17465903	1	4.407513
rs2293916	PADI3	1	17470242	4	0.474505
rs2293916	PADI3	1	17470242	1	3.202615
rs16824888	PADI3	1	17482332	3	0.029418
rs16824888	PADI3	1	17482332	6	0.111399
rs2977301	PADI3	1	17483189	1	5.993486
rs2977301	PADI3	1	17483189	6	1.160261
rs2977301	PADI3	1	17483189	8	1.225598
rs2977301	PADI3	1	17483189	3	1.293472
rs11203367	PADI4	1	17530203	1	0.337036
rs11203367	PADI4	1	17530203	7	0.31164
rs11203367	PADI4	1	17530203	6	0.083023
rs1748020	PADI4	1	17541196	3	0.169879
rs2240335	PADI4	1	17547124	8	0.737055
rs2240335	PADI4	1	17547124	1	2.237063
rs2240335	PADI4	1	17547124	7	0.680732
rs2240335	PADI4	1	17547124	5	0.52354
rs2526830	PADI6	1	17600706	3	1.46642
rs2526830	PADI6	1	17600706	1	1.841738
rs2526830	PADI6	1	17600706	5	0.134216
rs2526830	PADI6	1	17600706	8	0.622884
rs942457	RCC2	1	17612173	3	0.703953
rs942457	RCC2	1	17612173	8	4.756512
rs942457	RCC2	1	17612173	1	1.137725
rs942457	RCC2	1	17612173	2	0.577664
rs2270976	ARHGEF10L	1	17896277	3	1.051712
rs2270976	ARHGEF10L	1	17896277	7	0.093111
rs2270976	ARHGEF10L	1	17896277	1	5.093054
rs2296035	ACTL8	1	18022153	3	0.055588
rs1043459	IGSF21	1	18577449	3	0.21709
rs7528572	KLHDC7A	1	18680656	4	1.045281
rs7528572	KLHDC7A	1	18680656	2	2.314214
rs7528572	KLHDC7A	1	18680656	1	2.583205
rs2992753	KLHDC7A	1	18680879	1	2.756491
rs2992745	KLHDC7A	1	18682483	1	4.282732
rs2992745	KLHDC7A	1	18682483	5	0.431151
rs2992745	KLHDC7A	1	18682483	8	0.35565
rs2992745	KLHDC7A	1	18682483	3	1.370865
rs12061708	KLHDC7A	1	18682503	3	0.159474
rs12061708	KLHDC7A	1	18682503	4	1.573685
rs2076020	PAX7	1	18890992	5	0.880869
rs1140477	ALDH4A1	1	19071001	4	0.571209
rs1140477	ALDH4A1	1	19071001	1	3.475784
rs1140477	ALDH4A1	1	19071001	5	0.226308

rs1140477	ALDH4A1	1	19071001	7	0.233182
rs11740	ALDH4A1	1	19071808	6	1.603303
rs11740	ALDH4A1	1	19071808	2	1.887004
rs11740	ALDH4A1	1	19071808	1	1.123495
rs11740	ALDH4A1	1	19071808	3	0.844441
rs11740	ALDH4A1	1	19071808	4	2.134539
GA018627	ALDH4A1	1	19073575	4	4.395048
rs2230709	ALDH4A1	1	19074515	2	5.71275
rs2230709	ALDH4A1	1	19074515	5	0.380276
rs2230709	ALDH4A1	1	19074515	6	6.926505
GA018629	ALDH4A1	1	19075513	2	4.457767
GA018629	ALDH4A1	1	19075513	6	0.559996
GA018629	ALDH4A1	1	19075513	4	0.176644
GA018629	ALDH4A1	1	19075513	3	0.366604
GA018629	ALDH4A1	1	19075513	5	1.212659
rs12584	UBR4	1	19285848	5	0.032704
rs709682	KIAA0090	1	19437925	6	0.155449
rs709683	KIAA0090	1	19437931	3	0.37915
rs709683	KIAA0090	1	19437931	5	0.298282
rs709683	KIAA0090	1	19437931	2	7.700951
rs1042380	MRTO4	1	19456591	5	0.411329
rs7527253	AFAR3	1	19465786	5	0.943574
rs41264079	PQLC2	1	19525461	7	0.695864
rs41264079	PQLC2	1	19525461	6	0.376372
rs1534985	C1orf151	1	19825705	3	1.399404
rs1534985	C1orf151	1	19825705	4	0.165891
rs2281237	C1orf151	1	19825752	8	0.315838
rs11804596	C1orf151	1	19825919	6	3.483369
rs11804596	C1orf151	1	19825919	1	4.802949
rs880554	C1orf151	1	19826601	4	0.771646
rs3820317	RNF186	1	20013168	5	0.32827
rs1541185	RNF186	1	20014115	2	1.613635
rs1541185	RNF186	1	20014115	1	0.547109
rs1541185	RNF186	1	20014115	6	0.175632
rs1541185	RNF186	1	20014115	5	0.204814
rs10916668	OTUD3	1	20105673	2	4.482268
rs10916668	OTUD3	1	20105673	5	0.242109
rs17401735	OTUD3	1	20109318	4	0.637468
rs17401735	OTUD3	1	20109318	3	0.794779
rs17401735	OTUD3	1	20109318	5	0.821644
rs10492999	OTUD3	1	20110347	2	0.358191
rs10492999	OTUD3	1	20110347	5	1.492299
rs3738122	OTUD3	1	20111447	5	0.172324
rs3738122	OTUD3	1	20111447	1	5.189124
rs3738122	OTUD3	1	20111447	7	0.717645
rs3738122	OTUD3	1	20111447	6	4.30606
rs3738122	OTUD3	1	20111447	3	1.239172

rs4744	PLA2G2A	1	20177513	7	1.304448
rs2020887	PLA2G5	1	20283919	3	0.140095
rs617180	PLA2G2D	1	20311900	5	0.68187
rs578459	PLA2G2D	1	20312109	3	1.783592
rs578459	PLA2G2D	1	20312109	5	0.015322
rs584367	PLA2G2D	1	20314641	3	1.062416
rs6426616	PLA2G2C	1	20363105	4	1.730022
rs1044332	C1orf166	1	20698830	1	4.921462
rs1044332	C1orf166	1	20698830	3	1.391557
rs1044332	C1orf166	1	20698830	6	6.073717
rs529974	C1orf166	1	20699497	1	4.055173
rs476622	LOC441878	1	20725782	3	0.009975
rs3795509	FAM43B	1	20753896	3	0.174209
rs3795509	FAM43B	1	20753896	6	0.264729
rs8064	PINK1	1	20850186	5	0.308284
rs8064	PINK1	1	20850186	8	5.472235
rs10799659	KIF17	1	20863492	3	0.186187
rs631357	KIF17	1	20865406	5	0.226463
rs2296225	KIF17	1	20903629	8	1.943909
rs2296225	KIF17	1	20903629	1	1.54073
rs671905	SH2D5	1	20919679	3	0.154433
rs671905	SH2D5	1	20919679	6	2.674759
rs671905	SH2D5	1	20919679	1	0.456148
rs671905	SH2D5	1	20919679	4	1.178008
rs671905	SH2D5	1	20919679	2	0.164596
rs659046	SH2D5	1	20920312	1	1.196462
rs659046	SH2D5	1	20920312	4	0.934321
rs659046	SH2D5	1	20920312	6	1.626081
rs659046	SH2D5	1	20920312	3	1.128819
rs12729575	HP1BP3	1	20948861	5	8.693596
rs6423191	LOC728969	1	21630456	2	4.800872
rs6423191	LOC728969	1	21630456	1	5.413627
rs6423191	LOC728969	1	21630456	7	5.476861
rs6423191	LOC728969	1	21630456	6	4.744007
rs6423191	LOC728969	1	21630456	4	3.896804
rs6423191	LOC728969	1	21630456	3	5.418481
rs6423191	LOC728969	1	21630456	5	3.389314
rs1827293	NBPF3	1	21667975	4	0.16101
rs1780316	ALPL	1	21762222	6	1.726337
SNP1-21776718	ALPL	1	21776718	4	0.362702
SNP1-21776718	ALPL	1	21776718	2	2.840266
SNP1-21776718	ALPL	1	21776718	5	0.391803
rs2275363	RAP1GAP	1	21813142	4	0.377325
rs2275363	RAP1GAP	1	21813142	7	1.168595
rs2275363	RAP1GAP	1	21813142	5	0.012056
rs2275363	RAP1GAP	1	21813142	3	0.259218
rs1059218	USP48	1	21878252	1	4.301936

rs1059218	USP48	1	21878252	8	0.576435
rs1059218	USP48	1	21878252	7	0.060467
rs17459097	HSPG2	1	22032607	4	0.44119
rs17459097	HSPG2	1	22032607	8	0.264971
rs2229482	HSPG2	1	22071832	3	1.370281
rs2254357	HSPG2	1	22089191	2	5.506987
rs2254357	HSPG2	1	22089191	3	0.573198
rs9187	ELA3A	1	22208892	5	0.332865
rs9187	ELA3A	1	22208892	6	1.183051
rs11580218	LOC343384	1	22522074	5	1.359592
rs11580218	LOC343384	1	22522074	4	0.675043
rs881646	ZBTB40	1	22725302	1	4.800444
rs881646	ZBTB40	1	22725302	6	7.407793
rs606002	EPHA8	1	22788340	4	4.255482
rs606002	EPHA8	1	22788340	6	2.05538
rs606002	EPHA8	1	22788340	1	0.543772
rs11584845	EPHA8	1	22788554	3	0.414885
rs999765	EPHA8	1	22796460	5	0.149388
rs172378	C1QA	1	22838025	5	2.973936
rs172378	C1QA	1	22838025	7	1.639393
rs172378	C1QA	1	22838025	2	3.268515
rs172378	C1QA	1	22838025	6	3.389995
rs9434	C1QC	1	22846884	1	1.961206
rs9434	C1QC	1	22846884	7	1.513557
rs9434	C1QC	1	22846884	3	2.015237
rs9434	C1QC	1	22846884	5	1.91704
rs16827981	LUZP1	1	23308125	5	0.171512
rs16827981	LUZP1	1	23308125	4	0.354
rs665977	C1orf213	1	23568739	7	0.415316
rs665977	C1orf213	1	23568739	3	0.093292
rs665977	C1orf213	1	23568739	2	4.015175
rs1198432	DDEFL1	1	23628742	3	1.691586
SNP1-23633615	DDEFL1	1	23633615	7	1.455064
rs3820028	E2F2	1	23707073	7	0.921507
rs3820028	E2F2	1	23707073	4	3.986947
rs3820028	E2F2	1	23707073	5	6.11606
rs2075993	E2F2	1	23708951	1	4.845842
rs2075993	E2F2	1	23708951	8	2.030758
rs2075993	E2F2	1	23708951	7	0.698709
rs2075993	E2F2	1	23708951	4	0.508867
rs2075993	E2F2	1	23708951	5	0.697192
rs2229583	CNR2	1	24073570	2	0.750733
rs2229583	CNR2	1	24073570	5	2.727078
rs2229583	CNR2	1	24073570	7	3.380532
rs12145360	MYOM3	1	24267398	3	0.353005
rs6424152	MYOM3	1	24285746	3	0.808143
rs7533060	MYOM3	1	24288657	8	1.07089

rs7533060	MYOM3	1	24288657	5	0.917153
rs7533060	MYOM3	1	24288657	3	0.81467
rs7533060	MYOM3	1	24288657	2	1.177427
rs4276859	MYOM3	1	24288720	3	0.516012
rs6700245	MYOM3	1	24290002	3	1.379936
rs7556176	MYOM3	1	24291355	3	0.098983
rs7556176	MYOM3	1	24291355	8	2.494904
rs6678540	MYOM3	1	24294061	1	5.423061
rs6678540	MYOM3	1	24294061	2	5.048156
rs6678540	MYOM3	1	24294061	5	3.863764
rs6678540	MYOM3	1	24294061	3	2.974855
rs3795300	IL22RA1	1	24319801	2	1.054741
rs3795300	IL22RA1	1	24319801	8	0.0573
rs3795300	IL22RA1	1	24319801	4	0.813423
rs3795300	IL22RA1	1	24319801	1	0.023202
rs17852648	IL22RA1	1	24320671	2	3.590665
rs17852648	IL22RA1	1	24320671	4	0.279085
rs16829204	IL22RA1	1	24327275	3	2.041815
rs10903022	IL22RA1	1	24337700	2	1.09917
rs10903022	IL22RA1	1	24337700	1	0.748943
rs10903022	IL22RA1	1	24337700	3	0.807881
rs10903022	IL22RA1	1	24337700	8	0.767043
rs2486668	GRHL3	1	24530650	1	6.201007
rs2486668	GRHL3	1	24530650	3	0.041802
rs11576645	GRHL3	1	24541254	5	3.803897
rs11576645	GRHL3	1	24541254	7	0.141399
rs10903080	GRHL3	1	24554228	7	0.280397
rs10903080	GRHL3	1	24554228	4	3.721804
rs10903080	GRHL3	1	24554228	8	4.889333
rs12090888	C1orf201	1	24582992	4	1.265592
rs9259	CLIC4	1	25040711	3	0.323978
rs9259	CLIC4	1	25040711	1	4.033238
rs9259	CLIC4	1	25040711	4	0.144786
rs3093647	TMEM50A	1	25542118	1	3.641243
rs4537542	LDLRAP1	1	25767864	8	9.122631
rs760597	SEPN1	1	26010849	6	6.398651
rs2294226	LOC646471	1	26019036	2	1.144683
rs2294226	LOC646471	1	26019036	8	1.853514
rs2294226	LOC646471	1	26019036	7	2.074406
rs2294226	LOC646471	1	26019036	1	0.88679
rs2294226	LOC646471	1	26019036	4	1.112406
rs2294224	LOC646471	1	26019101	1	0.45996
rs2294224	LOC646471	1	26019101	6	0.625411
rs2294224	LOC646471	1	26019101	4	6.961708
rs2294224	LOC646471	1	26019101	7	6.621912
rs2294224	LOC646471	1	26019101	2	0.187701
rs213637	PAQR7	1	26061212	6	0.15154

rs213637	PAQR7	1	26061212	4	0.453246
rs213637	PAQR7	1	26061212	1	0.581427
rs213637	PAQR7	1	26061212	7	2.831385
rs159530	C1orf215	1	26083378	1	0.573475
rs159530	C1orf215	1	26083378	4	3.063982
rs159530	C1orf215	1	26083378	2	0.292722
rs159530	C1orf215	1	26083378	7	0.456064
rs213641	STMN1	1	26104943	2	0.140312
rs213641	STMN1	1	26104943	5	0.468116
rs213641	STMN1	1	26104943	4	3.033931
rs213641	STMN1	1	26104943	6	0.132936
rs213641	STMN1	1	26104943	1	0.232891
rs3008245	EXTL1	1	26221061	8	0.97319
rs11247847	EXTL1	1	26230243	5	0.127402
rs11247847	EXTL1	1	26230243	7	0.350054
rs11247848	EXTL1	1	26230254	5	0.49013
rs2802315	EXTL1	1	26234781	5	0.277729
rs2802315	EXTL1	1	26234781	4	0.074118
rs4659404	SLC30A2	1	26237961	7	0.210442
rs2997452	SLC30A2	1	26238136	5	0.207254
rs2997452	SLC30A2	1	26238136	7	0.985606
rs3748854	GRRP1	1	26358104	3	0.604923
rs3748854	GRRP1	1	26358104	4	0.173374
rs12069762	GRRP1	1	26361534	5	1.382436
rs17356937	CNKSR1	1	26376623	5	0.302722
rs1071849	CD52	1	26519313	1	4.65024
rs1071849	CD52	1	26519313	3	3.214513
rs1071849	CD52	1	26519313	4	2.471542
rs17645	CD52	1	26519317	1	3.327119
rs17645	CD52	1	26519317	4	1.97971
rs17645	CD52	1	26519317	3	2.603806
rs17163868	AIM1L	1	26520968	1	4.437014
rs17163868	AIM1L	1	26520968	4	2.022873
rs17163868	AIM1L	1	26520968	3	2.821222
rs10794531	ZNF683	1	26566832	3	1.235921
rs10794531	ZNF683	1	26566832	1	0.94131
rs10794532	ZNF683	1	26566847	1	0.202123
rs10794532	ZNF683	1	26566847	5	0.714026
rs9438623	LIN28	1	26628506	7	4.665754
rs9438623	LIN28	1	26628506	4	3.658919
rs9438623	LIN28	1	26628506	1	5.118013
rs11247957	LIN28	1	26628761	2	0.387646
rs11247957	LIN28	1	26628761	1	0.303578
rs6697410	LIN28	1	26628796	7	0.026151
rs6697410	LIN28	1	26628796	4	0.143009
rs6697410	LIN28	1	26628796	3	0.083288
rs6697410	LIN28	1	26628796	1	2.64068

rs11542704	SFN	1	27062911	1	2.565048
rs3170660	ATPBD1B	1	27083308	7	10.78333
rs3170660	ATPBD1B	1	27083308	2	3.604881
rs3170660	ATPBD1B	1	27083308	5	0.207062
rs3170660	ATPBD1B	1	27083308	8	6.955216
rs7504	NR0B2	1	27110737	7	0.508645
rs7504	NR0B2	1	27110737	6	1.051098
rs7504	NR0B2	1	27110737	5	1.327581
rs11538549	NUDC	1	27120870	6	6.258045
rs11538549	NUDC	1	27120870	8	6.65019
rs7866	NUDC	1	27145431	5	0.027335
rs3010109	C1orf172	1	27151140	3	0.069211
rs3010109	C1orf172	1	27151140	4	7.108195
rs3813795	SYTL1	1	27552384	4	0.376327
rs3813795	SYTL1	1	27552384	7	0.153555
SNP1-27560053	MAP3K6	1	27560053	7	0.150709
SNP1-27560053	MAP3K6	1	27560053	2	0.064834
SNP1-27560053	MAP3K6	1	27560053	3	0.384521
SNP1-27560053	MAP3K6	1	27560053	6	6.607217
SNP1-27560053	MAP3K6	1	27560053	1	0.221775
rs2076457	AHDC1	1	27749069	7	0.090752
rs6564	C1orf38	1	28085562	7	0.45083
rs6565	C1orf38	1	28085744	7	0.320575
rs6565	C1orf38	1	28085744	5	0.350218
rs6565	C1orf38	1	28085744	1	3.463156
rs6565	C1orf38	1	28085744	6	1.634245
rs3813804	SMPDL3B	1	28154793	1	0.666363
rs3813804	SMPDL3B	1	28154793	5	0.885134
rs3813804	SMPDL3B	1	28154793	2	0.089334
rs4654327	OPRD1	1	29062725	5	0.221213
rs2249138	EPB41	1	29316310	3	0.412353
rs9578	EPB41	1	29318185	5	0.546254
rs2452788	TMEM200B	1	29319398	2	6.114068
rs2452788	TMEM200B	1	29319398	3	0.594348
rs2230677	SFRS4	1	29347981	3	0.096491
rs2640464	MECR	1	29392365	3	0.180493
rs17363069	MECR	1	29392611	8	5.885454
rs17363069	MECR	1	29392611	4	5.050363
rs17363069	MECR	1	29392611	6	6.801532
rs17363069	MECR	1	29392611	1	4.732076
rs1128400	MECR	1	29415224	3	1.031024
rs1746059	MATN1	1	30958974	2	4.755999
rs1746059	MATN1	1	30958974	5	0.190444
rs8048	LAPTM5	1	30978383	4	0.128599
rs8048	LAPTM5	1	30978383	5	0.523479
rs8048	LAPTM5	1	30978383	3	4.520064
rs8048	LAPTM5	1	30978383	2	5.708769

rs1050663	LAPTM5	1	30987951	3	1.575455
rs41269523	SDC3	1	31122570	1	6.360687
rs12760571	PUM1	1	31177991	7	5.348262
rs2275741	PUM1	1	31199402	5	0.284124
rs2275741	PUM1	1	31199402	2	1.610871
rs2275741	PUM1	1	31199402	4	0.103091
rs1056526	HCRTR1	1	31857491	6	1.883762
rs2271928	COL16A1	1	31900540	7	0.438326
rs2271928	COL16A1	1	31900540	3	1.142645
rs2271928	COL16A1	1	31900540	4	0.670548
rs10914468	COL16A1	1	31936793	8	5.868111
rs13426	SPOCD1	1	32028673	7	0.348693
rs12117510	KPNA6	1	32410006	5	0.137747
rs704885	ZBTB8	1	32839178	5	3.77706
rs704885	ZBTB8	1	32839178	1	3.665786
rs12567940	ZBTB8	1	32840089	5	0.354631
rs422215	ZBTB8	1	32841341	1	2.857209
rs422215	ZBTB8	1	32841341	7	1.213866
rs422215	ZBTB8	1	32841341	3	1.506286
rs12136921	ZBTB8	1	32843365	1	4.582568
rs3753603	ZBTB8OS	1	32860024	2	0.328817
rs3753603	ZBTB8OS	1	32860024	7	2.938335
rs3753603	ZBTB8OS	1	32860024	6	3.319299
rs3753603	ZBTB8OS	1	32860024	4	4.843534
rs3753603	ZBTB8OS	1	32860024	3	4.080917
rs3753603	ZBTB8OS	1	32860024	1	4.372987
rs699005	YARS	1	33018389	2	0.006748
rs699005	YARS	1	33018389	1	0.143598
rs699005	YARS	1	33018389	7	6.605591
rs1284365	S100PBP	1	33096675	3	0.08423
rs1284365	S100PBP	1	33096675	6	0.325552
rs1284365	S100PBP	1	33096675	5	0.048457
rs3480	FNDC5	1	33100752	4	0.450421
rs3480	FNDC5	1	33100752	2	1.418608
rs3480	FNDC5	1	33100752	7	0.460184
rs3480	FNDC5	1	33100752	3	0.515025
rs3480	FNDC5	1	33100752	5	0.230596
rs622407	TRIM62	1	33419537	7	0.610993
rs11061	PHC2	1	33562257	6	1.499969
rs11554674	PHC2	1	33563083	6	0.406215
rs12026290	PHC2	1	33592721	6	0.536588
rs16835507	ZSCAN20	1	33717779	6	3.027315
rs16835507	ZSCAN20	1	33717779	1	0.359321
rs16835507	ZSCAN20	1	33717779	4	0.734034
rs16835507	ZSCAN20	1	33717779	2	0.633807
rs705207	C1orf94	1	34457050	2	3.161326
rs6698707	C1orf94	1	34457164	5	4.239222

rs6698707	C1orf94	1	34457164	6	2.978502
rs6698707	C1orf94	1	34457164	3	2.755697
rs6698707	C1orf94	1	34457164	2	3.208771
rs6698707	C1orf94	1	34457164	7	2.978529
rs6698707	C1orf94	1	34457164	4	3.462195
rs705193	GJA4	1	35033819	1	2.25993
rs705193	GJA4	1	35033819	8	4.563261
rs705193	GJA4	1	35033819	2	2.724315
rs705193	GJA4	1	35033819	3	0.426331
rs34924462	ZMYM4	1	35619619	1	3.821994
rs3795501	C1orf216	1	35953559	4	0.397859
SNP1-35975172	CLSPN	1	35975172	6	0.008366
SNP1-35975172	CLSPN	1	35975172	8	0.244606
rs399393	TEKT2	1	36326260	4	0.619987
rs399393	TEKT2	1	36326260	8	0.810253
rs399393	TEKT2	1	36326260	6	0.250228
rs1007028	COL8A2	1	36334671	6	0.678885
rs17442970	C1orf102	1	36660400	2	6.116589
rs16822954	C1orf102	1	36661592	8	1.009115
SNP1-36670654	C1orf102	1	36670654	4	0.234606
SNP1-36670654	C1orf102	1	36670654	3	6.565741
SNP1-36670654	C1orf102	1	36670654	5	0.432014
rs3917981	CSF3R	1	36709646	3	0.168745
rs3917981	CSF3R	1	36709646	6	0.573685
rs6691840	GRIK3	1	37098064	4	0.297382
rs6691840	GRIK3	1	37098064	3	0.464187
rs2273013	SNIP1	1	37792334	3	0.067275
rs2273013	SNIP1	1	37792334	6	0.31634
rs3856137	DNALI1	1	37804559	1	5.528386
rs4652964	RSPO1	1	37850887	8	0.080415
rs4652964	RSPO1	1	37850887	4	0.539918
rs4652964	RSPO1	1	37850887	7	1.589048
SNP1-37852104	RSPO1	1	37852104	1	0.542566
rs6670599	EPHA10	1	37958310	7	1.042987
rs12726343	EPHA10	1	37958793	3	0.612586
rs12726343	EPHA10	1	37958793	7	0.718388
rs12405650	EPHA10	1	37961327	7	2.01059
rs12405650	EPHA10	1	37961327	3	2.368261
rs17464871	EPHA10	1	37961355	7	1.34097
rs17464871	EPHA10	1	37961355	3	0.520647
rs17511304	EPHA10	1	37961374	7	0.416695
rs17511304	EPHA10	1	37961374	3	1.375278
rs12751325	MTF1	1	38061970	7	3.780297
rs12751325	MTF1	1	38061970	3	0.39863
rs11210870	SF3A3	1	38222497	3	0.608744
rs11210870	SF3A3	1	38222497	6	1.825232
rs783824	KIAA0754	1	39653352	3	4.208066

rs653064	KIAA0754	1	39654588	2	4.975283
rs668556	MACF1	1	39686950	8	7.459939
rs668556	MACF1	1	39686950	2	9.40465
rs668556	MACF1	1	39686950	5	0.366718
rs668556	MACF1	1	39686950	4	0.529247
rs668556	MACF1	1	39686950	1	0.004274
rs668556	MACF1	1	39686950	3	0.257948
rs1746838	MACF1	1	39690475	3	0.545539
rs3738676	BMP8A	1	39764175	1	4.533489
rs1180341	BMP8A	1	39765395	3	0.412638
rs1180341	BMP8A	1	39765395	7	1.616338
rs1180341	BMP8A	1	39765395	4	2.133974
rs1180341	BMP8A	1	39765395	1	1.738612
rs1180343	LOC728448	1	39766019	7	0.453408
rs1180343	LOC728448	1	39766019	3	0.342377
rs1180343	LOC728448	1	39766019	1	2.241439
rs1180343	LOC728448	1	39766019	5	1.350587
rs1180343	LOC728448	1	39766019	2	1.341449
rs1180343	LOC728448	1	39766019	4	2.563976
rs10839	HPCAL4	1	39917405	1	0.71521
rs562056	BMP8B	1	39998208	7	0.648703
rs562056	BMP8B	1	39998208	5	4.851479
rs3845570	TRIT1	1	40121718	7	2.147236
rs3845570	TRIT1	1	40121718	3	1.657634
rs3845570	TRIT1	1	40121718	1	0.309977
rs3845570	TRIT1	1	40121718	2	2.371736
rs41312030	SMAP2	1	40655334	8	0.229725
SNP1-40753413	C1orf176	1	40753413	6	0.012189
rs17412556	RIMS3	1	40861164	7	0.382388
rs17412556	RIMS3	1	40861164	4	0.28942
rs7520333	RIMS3	1	40862837	6	2.111953
rs570671	RIMS3	1	40863714	1	5.291016
rs570671	RIMS3	1	40863714	7	1.118049
rs570671	RIMS3	1	40863714	6	5.894268
rs679663	RIMS3	1	40864605	4	5.642114
rs679663	RIMS3	1	40864605	7	5.138616
rs4660468	KCNQ4	1	41057674	8	0.527311
rs4660468	KCNQ4	1	41057674	7	0.154298
rs4660468	KCNQ4	1	41057674	3	0.708032
rs11577910	CTPS	1	41250031	8	4.110719
rs12121539	SLFNL1	1	41253874	8	0.890155
rs1138293	SLFNL1	1	41258832	5	0.704674
rs3738369	SLFNL1	1	41258995	3	1.3709
rs3738369	SLFNL1	1	41258995	5	1.115001
rs17357954	SLFNL1	1	41259924	4	0.493273
rs17357954	SLFNL1	1	41259924	5	2.355496
rs17357954	SLFNL1	1	41259924	8	2.006853

rs17357954	SLFNL1	1	41259924	2	1.949932
rs17357954	SLFNL1	1	41259924	7	1.556447
rs17357954	SLFNL1	1	41259924	6	1.320273
rs11809423	HIVEP3	1	41749116	5	0.173472
rs17363472	HIVEP3	1	41819795	5	0.03257
rs12049046	RP4-692D3.1	1	42815290	4	0.448284
rs12049046	RP4-692D3.1	1	42815290	5	0.431186
rs12037518	RP4-692D3.1	1	42819644	4	0.615913
rs12037518	RP4-692D3.1	1	42819644	5	0.410665
rs12036818	RP4-692D3.1	1	42880775	4	1.123324
rs7556478	PPIH	1	42904240	4	0.484474
rs912075	CLDN19	1	42973113	7	1.09677
rs912075	CLDN19	1	42973113	5	2.221485
rs12124733	ERMAP	1	43068705	4	11.53704
rs12124733	ERMAP	1	43068705	5	5.706233
rs33950227	ERMAP	1	43068760	4	3.972189
rs33950227	ERMAP	1	43068760	7	0.702188
rs33950227	ERMAP	1	43068760	3	0.831731
rs33950227	ERMAP	1	43068760	2	4.623078
rs33953680	ERMAP	1	43068782	2	1.383597
rs33953680	ERMAP	1	43068782	1	0.802484
rs33953680	ERMAP	1	43068782	3	0.346535
rs33953680	ERMAP	1	43068782	7	1.072848
rs33953680	ERMAP	1	43068782	4	0.554475
rs12406279	ERMAP	1	43083226	7	0.366278
rs12406279	ERMAP	1	43083226	3	4.239523
rs4658	SLC2A1	1	43164837	2	0.956421
rs12074551	hCG_23177	1	43391183	4	1.905677
rs12074551	hCG_23177	1	43391183	1	3.824121
rs7163	EBNA1BP2	1	43405123	1	2.923609
rs7163	EBNA1BP2	1	43405123	4	9.724656
rs1049276	EBNA1BP2	1	43409071	1	4.467573
rs1049276	EBNA1BP2	1	43409071	4	0.013284
rs1782386	WDR65	1	43410997	1	3.751784
rs1782386	WDR65	1	43410997	4	0.466329
rs663824	WDR65	1	43422095	2	1.045367
rs663824	WDR65	1	43422095	1	0.198403
rs663824	WDR65	1	43422095	4	0.698072
rs603123	WDR65	1	43436805	2	1.004712
rs603123	WDR65	1	43436805	1	1.412263
rs603123	WDR65	1	43436805	4	0.724606
rs3120276	TIE1	1	43552151	6	1.819485
rs839753	C1orf84	1	43628165	5	0.028848
rs839753	C1orf84	1	43628165	1	0.08751
rs839753	C1orf84	1	43628165	3	0.842892
rs839753	C1orf84	1	43628165	2	0.826624
rs839769	C1orf84	1	43644654	3	1.149827

rs839769	C1orf84	1	43644654	5	1.007536
rs839769	C1orf84	1	43644654	4	0.655269
rs839769	C1orf84	1	43644654	8	3.195248
rs2027130	KIAA0467	1	43679483	6	2.567478
rs2027130	KIAA0467	1	43679483	3	2.127124
rs2027130	KIAA0467	1	43679483	8	2.289183
rs2027130	KIAA0467	1	43679483	4	1.861208
rs2027130	KIAA0467	1	43679483	2	3.940434
rs2027130	KIAA0467	1	43679483	5	3.509795
rs2251804	KIAA0467	1	43689996	4	1.69544
rs2251804	KIAA0467	1	43689996	5	0.770705
rs2251804	KIAA0467	1	43689996	8	0.319269
rs2251804	KIAA0467	1	43689996	6	4.550918
rs2251804	KIAA0467	1	43689996	3	1.145759
rs3828151	PTPRF	1	43830852	5	0.054348
rs3828151	PTPRF	1	43830852	3	0.444299
rs3828151	PTPRF	1	43830852	1	4.818313
rs631248	PTPRF	1	43843808	8	0.375683
rs631248	PTPRF	1	43843808	7	0.615764
rs641365	PTPRF	1	43856094	1	5.127435
rs641365	PTPRF	1	43856094	7	7.400176
rs6801	ST3GAL3	1	44169233	7	0.060703
rs6801	ST3GAL3	1	44169233	1	7.072994
rs6801	ST3GAL3	1	44169233	5	0.048369
rs6801	ST3GAL3	1	44169233	4	0.508904
rs2242637	ARTN	1	44173971	1	4.294118
rs2242637	ARTN	1	44173971	4	0.511481
rs17402858	IPO13	1	44195209	5	5.546314
rs17402858	IPO13	1	44195209	1	5.716583
rs2286241	ATP6V0B	1	44213356	3	0.547416
rs2248253	SLC6A9	1	44235757	1	2.486041
rs2248829	SLC6A9	1	44240822	1	4.438311
rs2485652	KLF17	1	44367997	2	0.891492
rs2485652	KLF17	1	44367997	5	0.529936
rs2485652	KLF17	1	44367997	1	0.427298
rs3795713	KIF2C	1	44991482	6	0.731542
rs11584031	LOC149478	1	45052303	4	0.016998
rs11573587	PTCH2	1	45065905	3	1.505182
rs7525308	PTCH2	1	45066105	4	0.31315
rs7525308	PTCH2	1	45066105	2	0.804839
rs11556200	EIF2B3	1	45216625	6	0.150347
rs489676	EIF2B3	1	45224814	4	0.838254
rs489676	EIF2B3	1	45224814	2	3.945566
rs8681	HECTD3	1	45241193	2	3.891304
rs41312020	HECTD3	1	45242173	6	4.541919
rs7551698	ZSWIM5	1	45277307	2	4.165871
rs7551698	ZSWIM5	1	45277307	6	0.154354

rs3893383	TESK2	1	45583510	4	6.555062
rs6429567	TESK2	1	45729287	2	1.620362
rs3790583	TESK2	1	45729415	7	0.182204
rs11580609	MMACHC	1	45748443	2	2.964621
rs11580609	MMACHC	1	45748443	4	0.147815
rs11580609	MMACHC	1	45748443	6	4.35671
rs7903	MMACHC	1	45749059	6	0.254693
rs7903	MMACHC	1	45749059	7	0.098625
rs2230658	NASP	1	45851441	7	0.177164
rs2230658	NASP	1	45851441	4	0.621533
rs2275085	CCDC17	1	45858439	4	5.511727
rs3014246	CCDC17	1	45858664	2	5.412353
rs3014246	CCDC17	1	45858664	4	0.311896
rs3014242	CCDC17	1	45860164	7	0.42419
rs3014242	CCDC17	1	45860164	4	0.707455
rs3811436	GPBP1L1	1	45898467	7	0.805986
rs3811436	GPBP1L1	1	45898467	4	0.532318
rs10749856	IPP	1	45937433	2	2.135365
rs10749856	IPP	1	45937433	7	1.252677
rs10749856	IPP	1	45937433	4	1.276873
rs1707336	MAST2	1	46266047	4	5.485677
rs1707336	MAST2	1	46266047	1	0.435266
rs1707336	MAST2	1	46266047	2	2.331646
rs1707337	PIK3R3	1	46281356	7	0.613587
rs1707337	PIK3R3	1	46281356	2	3.697848
rs1707337	PIK3R3	1	46281356	4	2.76008
rs1707303	PIK3R3	1	46370860	7	0.514932
rs1707303	PIK3R3	1	46370860	4	0.488974
rs41292543	UQCRH	1	46547370	4	4.994781
rs41292543	UQCRH	1	46547370	6	6.046794
rs17361749	NSUN4	1	46579067	2	2.510209
rs17361749	NSUN4	1	46579067	7	2.085443
rs17361749	NSUN4	1	46579067	3	2.941777
rs17361749	NSUN4	1	46579067	1	2.233062
rs17361749	NSUN4	1	46579067	5	2.398992
rs324420	FAAH	1	46643348	5	0.128476
rs547581	LOC729041	1	46683479	1	0.24933
rs547581	LOC729041	1	46683479	8	1.176955
rs547581	LOC729041	1	46683479	7	4.592309
rs547581	LOC729041	1	46683479	6	0.373328
rs1258036	KNCN	1	46784274	3	0.821995
rs1258036	KNCN	1	46784274	5	0.812487
rs885127	KNCN	1	46784731	1	5.707445
rs10890404	KNCN	1	46785767	2	4.066649
rs10890404	KNCN	1	46785767	1	1.06761
rs10890404	KNCN	1	46785767	3	1.508557
rs8602	MKNK1	1	46796118	8	6.602133

rs8602	MKNK1	1	46796118	2	7.002494
rs6671527	MOBKL2C	1	46853266	4	1.02318
rs6671527	MOBKL2C	1	46853266	3	0.934012
rs6671527	MOBKL2C	1	46853266	5	0.590914
rs6671527	MOBKL2C	1	46853266	1	2.772972
SNP1-46853328	MOBKL2C	1	46853328	2	3.86408
SNP1-46853328	MOBKL2C	1	46853328	8	0.888454
rs4646487	CYP4B1	1	47051762	3	0.4979
rs2297810	CYP4B1	1	47053446	6	5.708931
rs4646491	CYP4B1	1	47053471	6	2.937243
rs2056900	CYP4A22	1	47380372	5	0.93603
rs2056900	CYP4A22	1	47380372	3	0.931759
rs2056900	CYP4A22	1	47380372	4	0.173117
rs10789501	CYP4A22	1	47382076	4	1.380006
rs10789501	CYP4A22	1	47382076	2	1.628647
rs10789501	CYP4A22	1	47382076	5	0.478102
rs10789501	CYP4A22	1	47382076	3	1.07034
rs2070929	TAL1	1	47456194	1	0.478388
rs977747	TAL1	1	47457264	3	0.278439
rs3125630	STIL	1	47540501	5	0.079899
rs3125630	STIL	1	47540501	3	0.370721
rs3814006	LOC388630	1	48232494	4	0.86555
rs3814006	LOC388630	1	48232494	6	4.800604
rs3814006	LOC388630	1	48232494	8	4.514445
rs6679038	LOC388630	1	48236131	1	1.51198
rs6679038	LOC388630	1	48236131	4	1.462962
rs10788882	LOC391037	1	48403025	3	0.705516
rs10788882	LOC391037	1	48403025	4	0.056429
rs10788882	LOC391037	1	48403025	6	0.490942
rs7539694	AGBL4	1	48822262	7	0.754958
rs2480683	ELAVL4	1	50433998	4	1.737044
rs3176451	CDKN2C	1	51207191	1	0.222488
rs7512076	TXNDC12	1	52258692	3	0.930017
rs2809918	KTI12	1	52270700	1	4.975935
SNP1-52476079	ZFYVE9	1	52476079	3	0.290807
rs17364363	CC2D1B	1	52589547	3	1.321762
rs11555349	CC2D1B	1	52600972	3	0.477621
rs3087471	ORC1L	1	52611210	8	2.04576
rs17107416	ZYG11B	1	53061872	5	0.261431
rs480299	ZYG11A	1	53092862	5	0.44385
rs480299	ZYG11A	1	53092862	1	0.880719
rs480299	ZYG11A	1	53092862	8	0.12343
rs480299	ZYG11A	1	53092862	3	0.985799
rs7552139	ECHDC2	1	53159967	2	2.087802
rs7552139	ECHDC2	1	53159967	3	0.268661
rs7552139	ECHDC2	1	53159967	7	0.291603
rs7552139	ECHDC2	1	53159967	6	0.536966

rs7552139	ECHDC2	1	53159967	4	4.086984
rs7552139	ECHDC2	1	53159967	8	5.348169
rs11581638	SCP2	1	53232278	6	2.873616
rs10493171	LOC653511	1	53233852	6	0.690531
rs1288386	PODN	1	53308066	2	0.742841
rs1288389	PODN	1	53317185	1	0.866377
rs1288389	PODN	1	53317185	5	2.020881
rs2282340	PODN	1	53323653	5	0.498085
rs1288399	SLC1A7	1	53326073	1	0.717274
rs1288401	SLC1A7	1	53326342	1	0.836261
rs2297660	LRP8	1	53504903	1	0.781618
rs2297660	LRP8	1	53504903	2	1.267026
rs2297660	LRP8	1	53504903	8	0.016781
rs2297660	LRP8	1	53504903	4	1.005305
rs2297660	LRP8	1	53504903	6	1.915528
rs2297660	LRP8	1	53504903	3	0.97644
rs3820198	LRP8	1	53565239	5	2.631756
rs3820198	LRP8	1	53565239	8	0.770476
rs3820198	LRP8	1	53565239	4	1.231192
rs3820198	LRP8	1	53565239	7	3.707539
SNP1-53566099	LRP8	1	53566099	4	0.463256
SNP1-53566099	LRP8	1	53566099	7	0.208743
SNP1-53566099	LRP8	1	53566099	6	0.074806
SNP1-53566099	LRP8	1	53566099	5	1.064668
rs2296759	GLIS1	1	53744720	5	1.186244
rs3817871	YIPF1	1	54127523	1	2.977159
rs11206244	DIO1	1	54148289	2	1.672791
rs11206244	DIO1	1	54148289	1	1.151791
rs11206244	DIO1	1	54148289	7	1.016289
rs11206244	DIO1	1	54148289	5	0.325403
rs11206244	DIO1	1	54148289	3	0.320513
rs12096978	CDCP2	1	54391694	3	0.184201
rs7519887	CDCP2	1	54391992	1	1.356248
rs13571	MRPL37	1	54454508	6	6.119067
rs13571	MRPL37	1	54454508	3	0.064824
rs1617287	ACOT11	1	54847428	4	0.686686
rs1617287	ACOT11	1	54847428	2	5.022225
SNP1-54891732	C1orf175	1	54891732	1	0.621315
SNP1-54891732	C1orf175	1	54891732	3	1.346553
rs1655519	C1orf175	1	54892103	7	0.600922
rs1655519	C1orf175	1	54892103	1	5.017135
rs1655519	C1orf175	1	54892103	5	0.163153
rs1655519	C1orf175	1	54892103	6	4.291472
rs1655519	C1orf175	1	54892103	3	0.306555
rs17399810	C1orf175	1	54892122	2	5.587837
rs17399810	C1orf175	1	54892122	6	3.523927
rs17399810	C1orf175	1	54892122	5	0.366933

rs17399810	C1orf175	1	54892122	7	0.834711
rs480963	C1orf175	1	54909117	3	0.087775
rs480963	C1orf175	1	54909117	1	3.216012
rs480963	C1orf175	1	54909117	5	0.390437
rs480963	C1orf175	1	54909117	7	3.922298
rs570218	C1orf175	1	54912329	7	0.137964
rs1065173	C1orf175	1	54939430	4	1.13863
rs1065173	C1orf175	1	54939430	7	0.924702
rs646356	C1orf175	1	54940361	1	2.888674
rs1147990	TTC4	1	54954888	2	6.528225
rs1147990	TTC4	1	54954888	5	4.9053
rs13046	TTC4	1	54980342	5	0.154388
rs13046	TTC4	1	54980342	2	5.22548
rs11577368	PARS2	1	54997339	7	0.025988
rs11577368	PARS2	1	54997339	2	3.571238
rs2286202	TTC22	1	55023272	5	1.309479
rs2286202	TTC22	1	55023272	4	1.202642
rs2286202	TTC22	1	55023272	8	3.082915
rs1147984	TTC22	1	55039433	6	0.143174
rs1147984	TTC22	1	55039433	1	2.296671
rs1147984	TTC22	1	55039433	8	2.24596
rs1147984	TTC22	1	55039433	4	0.108963
rs757581	C1orf177	1	55044362	1	0.755316
rs9782980	C1orf177	1	55046168	8	2.77663
rs9782980	C1orf177	1	55046168	1	4.19961
rs9782980	C1orf177	1	55046168	6	0.874268
rs9782980	C1orf177	1	55046168	4	1.500581
rs17111100	C1orf177	1	55080281	7	0.345868
rs17111100	C1orf177	1	55080281	8	0.71639
rs6682884	BSND	1	55246913	3	1.442941
rs6682884	BSND	1	55246913	7	0.544314
rs11583680	PCSK9	1	55278256	3	0.635224
rs11583680	PCSK9	1	55278256	1	2.088745
rs540796	PCSK9	1	55296785	4	0.976235
rs540796	PCSK9	1	55296785	5	0.604964
rs540796	PCSK9	1	55296785	3	1.733644
rs540796	PCSK9	1	55296785	7	1.462534
rs562556	PCSK9	1	55296825	7	0.978715
rs562556	PCSK9	1	55296825	5	0.213787
rs562556	PCSK9	1	55296825	3	1.227362
rs562556	PCSK9	1	55296825	4	0.263422
rs13312	USP24	1	55305330	3	0.392315
rs13312	USP24	1	55305330	7	7.928065
rs13312	USP24	1	55305330	4	0.284103
rs1165222	USP24	1	55410663	3	0.935321
rs1165222	USP24	1	55410663	5	6.313278
rs1165222	USP24	1	55410663	4	0.020219

rs1165222	USP24	1	55410663	7	0.880847
rs17131774	LOC729423	1	58286678	1	1.639902
rs1109896	OMA1	1	58784992	5	1.030438
rs1109896	OMA1	1	58784992	6	0.474408
rs1109895	OMA1	1	58785019	5	0.601158
rs17118103	MYSM1	1	58920706	1	0.183872
rs2280274	CYP2J2	1	60131665	2	4.945823
rs11572191	CYP2J2	1	60164859	3	0.176539
rs626251	C1orf87	1	60239402	6	2.897085
rs12737449	C1orf87	1	60278371	5	0.291962
rs12737449	C1orf87	1	60278371	3	0.456324
rs12737449	C1orf87	1	60278371	6	1.381042
rs12737449	C1orf87	1	60278371	4	0.6872
rs10493277	C1orf87	1	60279332	3	0.050364
rs2498982	INADL	1	62289271	1	0.166372
rs2498982	INADL	1	62289271	8	7.69647
rs2498982	INADL	1	62289271	6	0.084378
rs2498982	INADL	1	62289271	5	10.83455
rs2457829	L1TD1	1	62445134	2	2.695712
rs7542665	L1TD1	1	62445625	3	1.422716
rs7533274	L1TD1	1	62445813	3	0.570031
rs7533274	L1TD1	1	62445813	4	0.047772
rs4625314	L1TD1	1	62448207	1	4.743898
rs4625314	L1TD1	1	62448207	4	1.256432
rs4625314	L1TD1	1	62448207	7	1.165154
rs4625314	L1TD1	1	62448207	8	1.98342
rs4625314	L1TD1	1	62448207	6	1.619852
rs4625314	L1TD1	1	62448207	3	1.407467
rs11207933	L1TD1	1	62448679	7	0.163019
rs11207933	L1TD1	1	62448679	5	2.337091
rs1056517	L1TD1	1	62449636	7	0.33029
rs1056517	L1TD1	1	62449636	3	0.405096
rs1056517	L1TD1	1	62449636	1	4.291662
rs1056517	L1TD1	1	62449636	4	0.909844
rs1056517	L1TD1	1	62449636	8	0.610211
rs17123220	ANKRD38	1	62476274	1	5.886107
rs2941679	ANKRD38	1	62485812	5	0.703788
rs2666472	ANKRD38	1	62501372	3	0.154686
rs2666472	ANKRD38	1	62501372	5	1.087766
rs2666472	ANKRD38	1	62501372	7	0.267532
rs2666472	ANKRD38	1	62501372	4	0.841652
rs2260581	ANKRD38	1	62501449	3	0.872445
rs2260581	ANKRD38	1	62501449	5	0.050461
rs2260581	ANKRD38	1	62501449	6	1.763027
rs2260581	ANKRD38	1	62501449	4	2.455048
rs2262110	ANKRD38	1	62501506	4	2.91631
rs2262110	ANKRD38	1	62501506	6	1.360416

rs2262110	ANKRD38	1	62501506	5	0.78095
rs2262110	ANKRD38	1	62501506	7	0.559788
rs2262110	ANKRD38	1	62501506	3	0.733875
rs12127930	ANKRD38	1	62512653	7	2.347789
rs2666507	ANKRD38	1	62513037	5	0.845015
rs2666507	ANKRD38	1	62513037	7	0.504326
rs11208084	LOC199897	1	63239010	6	2.599176
rs11208084	LOC199897	1	63239010	8	1.89183
rs2273367	EFCAB7	1	63793684	3	0.239585
rs3828064	CACHD1	1	64919590	7	1.240036
rs2230587	JAK1	1	65083850	3	0.106236
rs2230587	JAK1	1	65083850	2	5.105156
rs4582839	DNAJC6	1	65633275	8	4.320283
rs4582839	DNAJC6	1	65633275	4	0.45413
rs4582839	DNAJC6	1	65633275	5	5.188186
rs4915691	DNAJC6	1	65640107	3	0.762272
rs8179183	LEPR	1	65848540	2	4.61974
rs17415296	LEPR	1	65871601	2	4.056121
SNP1-65872910	LEPR	1	65872910	3	0.25124
rs1805096	LEPR	1	65874845	5	0.187612
rs1805096	LEPR	1	65874845	2	6.589531
rs1805096	LEPR	1	65874845	4	3.024048
rs1805096	LEPR	1	65874845	7	1.24065
SNP1-65875343	LEPR	1	65875343	3	0.161287
SNP1-65875343	LEPR	1	65875343	2	4.260604
rs2270866	LOC645227	1	66280856	4	0.565724
rs783036	PDE4B	1	66603958	7	0.105046
rs783036	PDE4B	1	66603958	5	3.154798
rs783036	PDE4B	1	66603958	1	6.31536
rs1886686	WDR78	1	67163056	1	0.45592
rs1886686	WDR78	1	67163056	3	4.34887
rs1495963	IL12RB2	1	67567907	6	0.737615
rs2307145	IL12RB2	1	67606115	7	0.26654
rs4297265	IL12RB2	1	67624923	3	1.177535
rs4297265	IL12RB2	1	67624923	6	0.195753
rs4297265	IL12RB2	1	67624923	7	1.333853
rs2229546	IL12RB2	1	67634108	6	0.214458
rs2229546	IL12RB2	1	67634108	2	2.662135
rs1046852	GPR177	1	68363692	1	4.180219
rs1046852	GPR177	1	68363692	7	1.014269
rs17130529	GPR177	1	68386908	1	0.32079
rs17130529	GPR177	1	68386908	2	1.833297
rs2820489	LOC645291	1	68401128	6	1.996012
rs505292	LOC391048	1	70769740	7	0.766919
rs5682	PTGER3	1	71249460	2	1.884771
rs5680	PTGER3	1	71249903	3	0.201937
rs11583800	ZRANB2	1	71309162	3	0.389036

rs17591292	LRRC44	1	74418646	1	3.315745
rs17591292	LRRC44	1	74418646	3	1.068567
rs11806946	LRRC44	1	74436297	3	1.00544
rs11806946	LRRC44	1	74436297	4	0.902698
rs11806946	LRRC44	1	74436297	2	3.848141
rs1412825	LRRC44	1	74436402	4	2.07826
rs1412825	LRRC44	1	74436402	2	4.181613
rs1412825	LRRC44	1	74436402	5	0.997
rs1412825	LRRC44	1	74436402	3	1.401008
rs12723334	C1orf173	1	74810433	1	0.688614
rs11210490	C1orf173	1	74870014	1	3.238586
rs1948874	SLC44A5	1	75444964	8	0.610882
rs1948874	SLC44A5	1	75444964	4	0.282692
rs12703	RABGGTB	1	76030480	5	0.084441
rs12703	RABGGTB	1	76030480	6	0.937562
rs5745325	MSH4	1	76042048	4	0.261175
rs5745325	MSH4	1	76042048	7	0.012282
rs5745325	MSH4	1	76042048	1	0.541824
rs5745325	MSH4	1	76042048	5	0.418979
rs879268	LOC729766	1	76245527	3	0.321074
rs1184626	ST6GALNAC3	1	76865768	2	0.2811
rs1184626	ST6GALNAC3	1	76865768	1	0.478717
rs12403583	ST6GALNAC3	1	76867943	1	0.639214
rs12403583	ST6GALNAC3	1	76867943	7	0.215679
rs41291532	ST6GALNAC3	1	76869210	2	2.07096
rs41291532	ST6GALNAC3	1	76869210	1	0.683229
rs2689674	hCG_22804	1	77367162	6	5.320572
rs1619903	AK5	1	77521252	1	0.430103
rs1619903	AK5	1	77521252	2	0.786402
rs1619903	AK5	1	77521252	3	0.047043
rs2689680	AK5	1	77521374	1	0.413816
rs2689680	AK5	1	77521374	7	0.99631
rs2689680	AK5	1	77521374	5	3.590686
rs2689680	AK5	1	77521374	3	1.391624
rs10873950	AK5	1	77797190	1	3.305157
rs10873950	AK5	1	77797190	7	0.974912
rs4949811	AK5	1	77798042	1	6.517399
rs3509	AK5	1	77798139	7	0.258184
rs3509	AK5	1	77798139	1	3.911625
rs273240	IFI44L	1	78858751	5	1.941232
rs273240	IFI44L	1	78858751	6	1.21801
rs1057920	IFI44L	1	78884279	6	0.16582
rs1057920	IFI44L	1	78884279	5	0.356786
rs1057920	IFI44L	1	78884279	1	3.62646
rs11163290	LOC729817	1	81665573	3	0.308997
rs1108500	LOC646555	1	81741872	3	4.57411
rs1108500	LOC646555	1	81741872	1	5.64254

rs17131763	LOC729828	1	81960734	7	3.833809
rs17131763	LOC729828	1	81960734	5	3.201144
rs10782824	PRKACB	1	84443172	6	3.117911
rs10782824	PRKACB	1	84443172	5	0.036801
rs10782824	PRKACB	1	84443172	3	0.346015
rs17131308	PRKACB	1	84475062	5	0.455386
rs17131635	UOX	1	84608287	1	0.678654
rs17131635	UOX	1	84608287	4	0.028575
rs17131635	UOX	1	84608287	5	0.298471
rs7511984	DNASE2B	1	84650816	5	0.568166
rs3768250	DNASE2B	1	84652968	4	1.441808
rs3768250	DNASE2B	1	84652968	1	3.67247
rs3768250	DNASE2B	1	84652968	5	0.231844
rs2292191	BXDC5	1	84717577	1	3.787964
rs2292191	BXDC5	1	84717577	8	0.24567
rs2292191	BXDC5	1	84717577	3	0.166084
rs7528067	BXDC5	1	84733696	1	4.809481
rs1043493	CTBS	1	84793237	1	6.43488
rs1043493	CTBS	1	84793237	8	6.290016
rs2994949	SSX2IP	1	84885791	1	2.709448
rs2994949	SSX2IP	1	84885791	4	0.820495
rs7522239	MCOLN3	1	85256954	5	0.69426
rs2028304	MCOLN3	1	85260618	5	6.448435
rs2028304	MCOLN3	1	85260618	8	1.841291
rs709783	WDR63	1	85367054	6	2.65173
SNP1-85428504	SYDE2	1	85428504	4	0.038095
rs1060846	BCL10	1	85504653	3	0.011562
rs1060846	BCL10	1	85504653	7	0.087641
rs1060846	BCL10	1	85504653	4	1.364062
rs1060846	BCL10	1	85504653	5	6.884511
rs1060843	BCL10	1	85514745	3	0.538732
rs1060843	BCL10	1	85514745	1	3.926981
rs11555520	LOC646626	1	85516040	4	1.742967
rs11555520	LOC646626	1	85516040	7	2.316196
rs11555520	LOC646626	1	85516040	5	0.608903
rs11555520	LOC646626	1	85516040	1	1.048688
rs2735592	LOC646626	1	85516329	7	3.101561
rs641712	COL24A1	1	86260820	5	2.69437
rs641712	COL24A1	1	86260820	1	2.74087
rs641712	COL24A1	1	86260820	2	2.43143
rs11161830	CLCA2	1	86677176	5	6.669472
rs11161830	CLCA2	1	86677176	1	6.194753
rs2734705	CLCA1	1	86724912	1	0.022852
rs2734705	CLCA1	1	86724912	2	0.545465
rs2734705	CLCA1	1	86724912	5	0.564661
rs1517903	CLCA3	1	86886833	3	2.054826
rs754001	15-Sep	1	87152574	1	2.26263

rs754001	15-Sep	1	87152574	4	0.4753
rs754001	15-Sep	1	87152574	2	2.732494
rs754001	15-Sep	1	87152574	5	2.623696
rs101028	15-Sep	1	87152584	5	2.84694
rs101028	15-Sep	1	87152584	1	2.458029
rs101028	15-Sep	1	87152584	2	2.965108
rs6686259	LOC339524	1	87370705	5	2.102535
rs6686259	LOC339524	1	87370705	1	3.174292
rs6686259	LOC339524	1	87370705	3	3.720229
rs430600	PKN2	1	88998564	2	3.206359
rs430600	PKN2	1	88998564	5	0.09298
rs430600	PKN2	1	88998564	7	0.10686
rs430600	PKN2	1	88998564	8	10.11023
rs4658360	GBP6	1	89619999	5	0.398723
rs4658360	GBP6	1	89619999	4	0.170101
rs4658360	GBP6	1	89619999	3	0.757045
rs4658360	GBP6	1	89619999	1	0.422539
rs4658146	GBP6	1	89620032	2	2.099616
rs4658146	GBP6	1	89620032	1	2.521693
rs4658146	GBP6	1	89620032	5	1.876241
rs4658146	GBP6	1	89620032	3	1.854054
rs4658146	GBP6	1	89620032	4	1.501616
rs959460	GBP6	1	89622330	6	0.469901
rs959460	GBP6	1	89622330	3	0.7892
rs3795832	LRRC8B	1	89822203	3	4.2525
rs3795832	LRRC8B	1	89822203	5	2.908741
rs3795832	LRRC8B	1	89822203	1	4.281515
rs3795832	LRRC8B	1	89822203	4	3.342416
rs3795832	LRRC8B	1	89822203	7	4.432333
rs3795832	LRRC8B	1	89822203	8	3.785277
rs3795832	LRRC8B	1	89822203	6	3.997247
rs3795832	LRRC8B	1	89822203	2	4.546186
rs2703984	LOC492303	1	90232332	3	0.283094
rs11808927	ZNF326	1	90243503	7	0.541011
rs11808927	ZNF326	1	90243503	3	0.159162
rs3893101	LOC645931	1	91567082	8	4.453613
rs3893101	LOC645931	1	91567082	2	5.059033
rs13447455	CDC7	1	91739033	8	0.833104
rs1804506	TGFBR3	1	91920601	1	0.872687
rs1804506	TGFBR3	1	91920601	3	0.869503
rs284878	TGFBR3	1	91946848	5	0.071784
rs1805113	TGFBR3	1	91950526	2	3.00525
rs1805113	TGFBR3	1	91950526	4	0.95007
rs1805112	TGFBR3	1	91958245	7	0.252698
rs1805112	TGFBR3	1	91958245	3	1.320079
rs1805112	TGFBR3	1	91958245	2	1.54342
rs1805110	TGFBR3	1	92099633	1	1.662864

rs1325432	GFI1	1	92713438	5	3.602822
rs1886683	EVI5	1	92748611	7	2.951095
rs1886683	EVI5	1	92748611	1	0.394974
rs1886683	EVI5	1	92748611	2	2.92382
rs1886683	EVI5	1	92748611	5	7.178178
rs6690764	EVI5	1	92749178	8	4.207498
rs6690764	EVI5	1	92749178	5	0.254003
rs6690764	EVI5	1	92749178	1	0.857603
rs1886682	EVI5	1	92749863	5	0.49594
rs1886682	EVI5	1	92749863	1	0.99858
rs6603979	EVI5	1	92751942	1	0.411833
rs6603979	EVI5	1	92751942	5	5.994468
rs6603979	EVI5	1	92751942	7	0.166044
rs6603979	EVI5	1	92751942	2	0.144234
rs6603979	EVI5	1	92751942	8	1.876722
rs10874744	SNORA66	1	93078905	5	0.145138
rs10874744	SNORA66	1	93078905	3	1.092708
rs10874744	SNORA66	1	93078905	1	5.347147
rs10874744	SNORA66	1	93078905	7	1.146043
rs10874767	CCDC18	1	93516643	7	0.025674
rs10874767	CCDC18	1	93516643	4	2.222923
rs2235971	DNTTIP2	1	94117304	3	0.543485
rs2235971	DNTTIP2	1	94117304	4	6.103286
rs3747961	ABCA4	1	94231009	1	3.795111
rs3747961	ABCA4	1	94231009	3	0.414279
rs3747961	ABCA4	1	94231009	4	1.145112
rs6666652	ABCA4	1	94234305	3	1.475844
rs6666652	ABCA4	1	94234305	4	0.969084
rs1762114	ABCA4	1	94243663	3	1.201471
rs1762114	ABCA4	1	94243663	4	0.336091
rs2275029	ABCA4	1	94246433	7	0.896629
rs2275029	ABCA4	1	94246433	5	0.820399
rs1801574	ABCA4	1	94248976	6	0.688289
rs1801574	ABCA4	1	94248976	5	3.039806
rs1801574	ABCA4	1	94248976	7	0.515321
rs1801574	ABCA4	1	94248976	1	3.648099
rs1801466	ABCA4	1	94249055	1	1.213688
rs3354	F3	1	94768407	1	2.514614
rs3354	F3	1	94768407	2	5.648882
rs6577322	SNX7	1	98937022	4	0.200972
rs6577322	SNX7	1	98937022	5	0.661955
rs6577322	SNX7	1	98937022	3	1.071662
rs764051	PAP2D	1	99129546	7	1.498837
rs309087	PAP2D	1	99191235	5	0.016981
rs3818852	PALMD	1	99932765	5	0.333046
rs3818852	PALMD	1	99932765	3	0.463759
rs7537296	FRRS1	1	99976236	1	0.54243

rs7537296	FRRS1	1	99976236	3	1.26081
rs7514021	FRRS1	1	99976281	1	0.057273
rs7514021	FRRS1	1	99976281	3	0.406805
rs7514021	FRRS1	1	99976281	2	1.967955
rs1416236	FRRS1	1	99987075	1	1.279894
rs1416236	FRRS1	1	99987075	3	1.00268
rs1416236	FRRS1	1	99987075	2	1.030854
rs687513	CCDC76	1	100371455	4	0.904581
rs614805	LOC646970	1	100570061	8	2.292899
rs614805	LOC646970	1	100570061	3	3.104918
rs614805	LOC646970	1	100570061	6	7.286343
rs614805	LOC646970	1	100570061	5	1.865915
rs614805	LOC646970	1	100570061	4	2.57424
rs614805	LOC646970	1	100570061	7	3.162329
rs7534479	LOC646970	1	100570725	3	2.434016
rs7534479	LOC646970	1	100570725	7	1.538149
rs7534479	LOC646970	1	100570725	1	2.512765
rs529224	CDC14A	1	100590766	7	0.024944
rs529224	CDC14A	1	100590766	6	0.837888
rs529224	CDC14A	1	100590766	5	0.265287
rs529224	CDC14A	1	100590766	1	2.171384
rs529224	CDC14A	1	100590766	3	0.135685
rs3737578	EDG1	1	101477084	4	0.570566
rs3737578	EDG1	1	101477084	1	1.589609
rs3737577	EDG1	1	101477120	1	0.001373
rs3737577	EDG1	1	101477120	2	1.817737
rs3737577	EDG1	1	101477120	3	0.50044
rs3737577	EDG1	1	101477120	6	0.628
rs3737577	EDG1	1	101477120	5	1.360035
rs2229783	COL11A1	1	103125039	4	1.253948
rs2229783	COL11A1	1	103125039	3	0.314417
rs2229783	COL11A1	1	103125039	2	4.42265
rs12731843	COL11A1	1	103264049	5	0.837491
rs10881350	LOC727839	1	106233900	3	1.055855
rs10881350	LOC727839	1	106233900	1	1.929182
rs10881350	LOC727839	1	106233900	8	2.917247
rs2232016	PRMT6	1	107401441	5	0.829677
rs2232016	PRMT6	1	107401441	2	1.32326
rs2232016	PRMT6	1	107401441	1	0.526639
rs2232016	PRMT6	1	107401441	4	1.231771
rs8458	VAV3	1	107915379	8	5.135352
rs8458	VAV3	1	107915379	2	0.669931
rs8458	VAV3	1	107915379	5	0.875975
rs8458	VAV3	1	107915379	6	0.234247
rs8458	VAV3	1	107915379	1	1.080192
SNP1-10798683	VAV3	1	107986832	4	0.801141
rs7528153	VAV3	1	108109250	2	5.202136

rs7528153	VAV3	1	108109250	4	0.228109
rs7528153	VAV3	1	108109250	8	3.361494
rs2358529	FAM102B	1	108981561	7	4.177221
rs11582005	FNDC7	1	109069964	5	0.279319
rs11582005	FNDC7	1	109069964	1	2.619858
SNP1-10907009б	FNDC7	1	109070096	6	0.548899
rs4970809	FNDC7	1	109074979	8	0.525478
rs4970809	FNDC7	1	109074979	1	2.586222
rs4970809	FNDC7	1	109074979	3	1.584187
rs4970809	FNDC7	1	109074979	5	0.238654
rs1124427	NULL	1	109090876	5	4.750133
rs11102337	NULL	1	109091734	5	0.407001
rs11102337	NULL	1	109091734	3	0.049724
rs11102337	NULL	1	109091734	8	0.839089
rs11102337	NULL	1	109091734	1	6.548635
rs7522157	C1orf62	1	109167809	4	2.730659
rs7522157	C1orf62	1	109167809	6	4.79981
rs7522157	C1orf62	1	109167809	1	5.706722
rs7522157	C1orf62	1	109167809	5	0.312405
rs7522157	C1orf62	1	109167809	8	1.069785
rs12060255	C1orf62	1	109178593	4	0.002654
rs11580913	C1orf62	1	109193185	7	0.463656
rs17563390	C1orf62	1	109196501	1	3.316071
rs7519409	C1orf62	1	109201183	1	2.789064
rs7551142	C1orf62	1	109201233	8	0.715588
rs667524	KIAA1324	1	109458469	3	0.01542
rs667524	KIAA1324	1	109458469	7	0.241468
rs658779	KIAA1324	1	109536821	3	0.6076
rs659543	KIAA1324	1	109536939	3	0.262676
rs2359244	KIAA1324	1	109538655	3	0.046509
rs913179	KIAA1324	1	109544008	3	0.979056
rs1052878	KIAA1324	1	109547141	2	4.995288
rs2281894	CELSR2	1	109612067	7	5.289938
rs2281894	CELSR2	1	109612067	5	6.449667
SNP1-10961968	CELSR2	1	109619681	5	0.813704
rs3738777	MYBPHL	1	109638358	8	1.272773
rs3738777	MYBPHL	1	109638358	3	0.385044
rs3850615	MYBPHL	1	109641261	3	0.695251
rs6666119	CYB561D1	1	109841833	3	1.146403
rs6666119	CYB561D1	1	109841833	5	6.918326
rs2297797	CYB561D1	1	109842203	2	2.528307
rs2297797	CYB561D1	1	109842203	4	0.547488
rs2297797	CYB561D1	1	109842203	5	0.463076
rs2297797	CYB561D1	1	109842203	3	0.208977
rs2304355	GNAT2	1	109957047	5	0.606557
rs2304355	GNAT2	1	109957047	8	1.232043
rs568686	AMPD2	1	109975298	6	0.381792

rs11807	GSTM5	1	110062265	8	3.992089
rs11807	GSTM5	1	110062265	6	0.915764
rs11807	GSTM5	1	110062265	1	3.379385
rs3814309	GSTM3	1	110078926	7	3.529109
rs1537236	GSTM3	1	110080495	1	3.504292
rs1332018	GSTM3	1	110084495	1	2.959525
rs1332018	GSTM3	1	110084495	3	0.684204
rs1332018	GSTM3	1	110084495	8	1.327069
rs3818562	EPS8L3	1	110101964	7	0.719922
rs41281344	FAM40A	1	110385780	7	5.025682
rs2784140	SLC6A17	1	110539819	3	1.254947
rs2784140	SLC6A17	1	110539819	8	2.525435
rs2784140	SLC6A17	1	110539819	5	0.477791
SNP1-110567977	KCNC4	1	110567977	8	10.1413
rs17025562	KCNC4	1	110576718	1	4.718026
rs3738750	SLC16A4	1	110725876	3	0.400105
rs3738750	SLC16A4	1	110725876	7	0.341617
rs3738750	SLC16A4	1	110725876	2	3.247476
rs6698159	HBXIP	1	110751800	5	0.034497
rs6674775	HBXIP	1	110751962	2	0.647751
rs6674775	HBXIP	1	110751962	3	0.478449
rs3748731	KCNA10	1	110862534	3	0.539954
rs3748731	KCNA10	1	110862534	1	0.30025
rs3887820	KCNA2	1	110949603	1	1.562661
rs1494324	CD53	1	111217364	4	0.876426
rs12030938	CD53	1	111243381	3	0.278027
rs12030938	CD53	1	111243381	6	2.703803
rs12030938	CD53	1	111243381	5	0.716948
rs1936939	CD53	1	111244078	5	0.069259
rs1936939	CD53	1	111244078	8	0.109439
rs1936939	CD53	1	111244078	6	0.213905
rs1936939	CD53	1	111244078	3	0.121969
rs1043096	CEPT1	1	111483830	6	4.898082
rs17027410	CHIA	1	111663345	1	2.751402
rs17027410	CHIA	1	111663345	8	2.030055
rs2820092	CHIA	1	111663597	5	1.036451
rs2820092	CHIA	1	111663597	1	2.813656
rs3738303	C1orf88	1	111695703	6	0.906025
rs3738303	C1orf88	1	111695703	4	0.499052
rs9645351	C1orf88	1	111696393	5	0.270036
rs9645351	C1orf88	1	111696393	7	0.261654
rs9645351	C1orf88	1	111696393	1	4.502947
rs1058530	C1orf88	1	111697010	6	1.031485
rs1058530	C1orf88	1	111697010	1	3.567
rs10857881	LOC441897	1	111735211	1	3.883473
rs7825	OVGP1	1	111758620	6	0.090994
rs7825	OVGP1	1	111758620	5	0.541574

rs7825	OVGP1	1	111758620	1	0.482975
rs7825	OVGP1	1	111758620	3	0.14121
rs17027633	OVGP1	1	111763779	4	0.727241
rs2275794	OVGP1	1	111765785	4	0.343664
rs954739	OVGP1	1	111767814	2	2.348013
rs954739	OVGP1	1	111767814	1	0.704848
rs3754032	WDR77	1	111784127	5	0.426018
rs3754032	WDR77	1	111784127	1	0.247659
rs11548427	ATP5F1	1	111793291	4	0.388548
rs6703267	C1orf162	1	111820180	1	0.587313
rs6703267	C1orf162	1	111820180	5	0.632037
rs1054680	C1orf162	1	111822552	4	0.702651
rs1054680	C1orf162	1	111822552	2	1.418219
rs1054680	C1orf162	1	111822552	1	1.118502
rs1054680	C1orf162	1	111822552	5	4.181073
rs1054680	C1orf162	1	111822552	3	1.308484
rs9025	ADORA3	1	111827721	5	0.046468
rs2275797	ADORA3	1	111834808	3	0.233411
rs1415792	ADORA3	1	111843718	6	3.104461
rs1415792	ADORA3	1	111843718	3	2.126714
rs1415792	ADORA3	1	111843718	4	2.123036
rs1415793	ADORA3	1	111843759	2	2.183857
rs1415793	ADORA3	1	111843759	4	2.051753
rs1415793	ADORA3	1	111843759	3	3.144352
rs1544223	ADORA3	1	111848080	3	0.571737
rs1544223	ADORA3	1	111848080	2	4.297429
rs12033257	KCND3	1	112120007	8	0.630741
rs12033257	KCND3	1	112120007	7	0.661429
rs2273368	WNT2B	1	112865294	1	4.678391
rs2273368	WNT2B	1	112865294	7	0.248192
rs2273368	WNT2B	1	112865294	4	3.107668
SNP1-11305617	PPM1J	1	113056171	3	0.157565
rs11803863	LRIG2	1	113437384	6	0.957783
SNP1-11402682	MAGI3	1	114026829	1	7.059721
SNP1-11402682	MAGI3	1	114026829	3	1.085571
SNP1-11402682	MAGI3	1	114026829	6	3.713586
SNP1-11402839	MAGI3	1	114028398	4	1.137101
SNP1-11402980	MAGI3	1	114029805	6	1.518728
SNP1-11402980	MAGI3	1	114029805	3	0.234846
rs3811021	PTPN22	1	114158186	5	2.11748
rs1217382	BCL2L15	1	114225176	1	2.082459
rs12022378	DCLRE1B	1	114249912	6	0.640577
SNP1-11425732	DCLRE1B	1	114257324	1	5.44285
rs6674384	DCLRE1B	1	114257430	2	1.706399
SNP1-11430652	HIPK1	1	114306523	2	5.067377
SNP1-11430652	HIPK1	1	114306523	8	0.177409
rs1125002	TRIM33	1	114739698	4	2.307983

rs12083584	TRIM33	1	114774952	1	0.898883
rs12083584	TRIM33	1	114774952	6	0.255748
rs11084	SYCP1	1	115339456	1	1.539155
rs11084	SYCP1	1	115339456	5	2.311271
rs1052480	TSPAN2	1	115392581	5	1.681492
rs1052480	TSPAN2	1	115392581	6	1.972802
rs1052480	TSPAN2	1	115392581	1	1.998948
rs7551034	TSPAN2	1	115392686	6	3.900042
rs7551034	TSPAN2	1	115392686	5	1.289336
rs7551034	TSPAN2	1	115392686	2	0.524135
rs7551034	TSPAN2	1	115392686	7	0.256007
rs10776795	TSPAN2	1	115406321	1	3.802466
rs10776795	TSPAN2	1	115406321	3	0.918974
rs6330	NGFB	1	115630836	7	1.644368
rs3204955	VANGL1	1	116036800	1	6.011397
rs4839472	VANGL1	1	116039019	1	4.586452
rs4839472	VANGL1	1	116039019	7	0.507709
rs17034226	VANGL1	1	116040001	7	7.563167
rs17034226	VANGL1	1	116040001	3	0.399538
rs17034226	VANGL1	1	116040001	1	3.757728
rs17034226	VANGL1	1	116040001	4	1.40114
rs4548441	VANGL1	1	116041364	7	0.364314
rs4548441	VANGL1	1	116041364	1	5.284907
rs4548441	VANGL1	1	116041364	3	1.449269
rs4548441	VANGL1	1	116041364	4	0.483841
rs4548441	VANGL1	1	116041364	8	7.013208
rs7413162	CASQ2	1	116045400	4	0.014719
rs7413162	CASQ2	1	116045400	1	2.198802
rs10754377	NHLH2	1	116181695	7	0.69897
rs10754377	NHLH2	1	116181695	5	0.846903
rs2488429	SLC22A15	1	116371053	2	3.113719
rs3748743	SLC22A15	1	116406946	2	5.368914
rs3748742	SLC22A15	1	116410708	8	0.888321
rs3748742	SLC22A15	1	116410708	2	4.158917
rs3748741	SLC22A15	1	116411356	8	0.123832
rs3748741	SLC22A15	1	116411356	2	4.966056
rs10802159	C1orf161	1	116477558	8	0.902362
rs6670668	C1orf161	1	116478071	8	0.129624
rs1034919	RP4-781D12.3	1	116877028	7	0.695572
rs1034919	RP4-781D12.3	1	116877028	6	0.155114
rs1034919	RP4-781D12.3	1	116877028	5	1.909756
rs1034919	RP4-781D12.3	1	116877028	1	3.119499
rs1034920	RP4-781D12.3	1	116877922	7	0.345937
rs1034920	RP4-781D12.3	1	116877922	6	1.770194
rs1034920	RP4-781D12.3	1	116877922	1	2.124384
rs1034920	RP4-781D12.3	1	116877922	5	0.963288
rs1034921	RP4-781D12.3	1	116877926	5	0.000289

rs1034921	RP4-781D12.3	1	116877926	1	2.793352
rs1034921	RP4-781D12.3	1	116877926	7	0.091294
rs1034921	RP4-781D12.3	1	116877926	6	0.074253
rs9887785	GAPDHL9	1	117058220	3	1.615283
rs12408586	LOC339396	1	117084250	7	1.291588
rs12408586	LOC339396	1	117084250	5	1.421405
SNP1-11730546	PTGFRN	1	117305463	2	4.383496
rs7554376	IGSF2	1	117354083	7	1.902637
rs2249265	IGSF2	1	117361249	1	4.011861
rs3736907	IGSF2	1	117362452	4	2.802331
rs3736907	IGSF2	1	117362452	7	8.210757
rs3736907	IGSF2	1	117362452	5	1.531545
SNP1-11736974	IGSF2	1	117369740	8	3.786875
rs2244501	TTF2	1	117404483	3	0.835953
rs2244501	TTF2	1	117404483	7	1.676475
rs2244501	TTF2	1	117404483	4	0.990708
rs12118664	TRIM45	1	117456161	1	3.96127
rs12118664	TRIM45	1	117456161	7	0.619056
rs12118664	TRIM45	1	117456161	5	0.175943
rs1048635	TRIM45	1	117456381	6	1.397897
rs1289658	TRIM45	1	117457611	5	4.656324
rs1289658	TRIM45	1	117457611	1	2.057301
rs1289658	TRIM45	1	117457611	6	0.26624
rs1289658	TRIM45	1	117457611	7	0.640206
rs749902	TRIM45	1	117462277	7	0.046469
rs749902	TRIM45	1	117462277	5	11.21362
rs749902	TRIM45	1	117462277	1	3.721762
rs6657335	TRIM45	1	117465569	5	0.155899
rs6657335	TRIM45	1	117465569	1	4.159678
rs6657335	TRIM45	1	117465569	7	0.834008
rs34957002	VTCN1	1	117554978	7	0.330978
rs12120771	MAN1A2	1	117867442	7	0.182233
rs12120771	MAN1A2	1	117867442	8	7.378869
rs865443	FAM46C	1	117967100	5	0.536182
rs1627787	FAM46C	1	117967496	5	6.29035
rs1627787	FAM46C	1	117967496	4	1.12106
rs1627787	FAM46C	1	117967496	1	5.338911
rs2066411	FAM46C	1	117968273	5	0.403051
rs2066411	FAM46C	1	117968273	7	0.516827
rs2066411	FAM46C	1	117968273	1	4.112434
rs2066411	FAM46C	1	117968273	4	0.792323
rs10923360	FAM46C	1	117968400	6	2.612854
rs10923360	FAM46C	1	117968400	7	0.600544
rs10923360	FAM46C	1	117968400	8	3.86217
rs10923360	FAM46C	1	117968400	2	0.355848
rs10923360	FAM46C	1	117968400	1	1.064392
rs826403	FAM46C	1	117968722	1	0.782128

rs826403	FAM46C	1	117968722	8	0.12709
rs826403	FAM46C	1	117968722	7	1.372985
rs826403	FAM46C	1	117968722	2	2.432907
rs826403	FAM46C	1	117968722	5	0.914242
rs826403	FAM46C	1	117968722	6	0.6525
rs866111	FAM46C	1	117969176	2	5.086132
rs866111	FAM46C	1	117969176	5	0.471582
rs11580608	FAM46C	1	117969757	8	0.100244
rs11580608	FAM46C	1	117969757	1	4.262335
rs11580608	FAM46C	1	117969757	6	0.739284
rs11580608	FAM46C	1	117969757	5	0.371491
rs11580608	FAM46C	1	117969757	7	0.02639
rs11581122	FAM46C	1	117970349	4	1.002915
rs11581122	FAM46C	1	117970349	1	4.609146
rs11581122	FAM46C	1	117970349	7	1.598448
rs2282456	FAM46C	1	117970986	1	6.130249
rs2282456	FAM46C	1	117970986	8	4.728279
rs2282456	FAM46C	1	117970986	5	0.423282
rs2282456	FAM46C	1	117970986	6	0.194271
rs2282456	FAM46C	1	117970986	7	0.304881
rs10754367	SPAG17	1	118445953	1	2.336976
rs10754367	SPAG17	1	118445953	8	0.374267
rs10494217	TBX15	1	119270711	5	0.38396
rs8928	WARS2	1	119375597	1	3.85743
rs8928	WARS2	1	119375597	7	0.588232
rs3790548	WARS2	1	119376820	2	2.167348
rs346683	LOC644237	1	119940454	7	0.755857
rs346683	LOC644237	1	119940454	5	0.836279
rs6689350	LOC391081	1	119953605	1	1.517546
rs6689350	LOC391081	1	119953605	2	2.090272
rs6689350	LOC391081	1	119953605	6	1.082312
rs6689350	LOC391081	1	119953605	3	2.124212
rs6689350	LOC391081	1	119953605	4	1.001565
rs6689350	LOC391081	1	119953605	5	0.826089
rs12067843	ZNF697	1	119968204	4	1.75739
rs12067843	ZNF697	1	119968204	5	2.673321
rs1052972	REG4	1	120138308	4	2.104044
rs1052972	REG4	1	120138308	5	2.091946
rs1052972	REG4	1	120138308	3	2.082953
rs1052972	REG4	1	120138308	6	2.153118
rs699778	REG4	1	120138399	3	0.553595
rs699778	REG4	1	120138399	4	0.048204
rs699778	REG4	1	120138399	6	0.138236
rs699778	REG4	1	120138399	5	0.645407
rs835575	NOTCH2	1	120258086	6	5.742575
rs11590105	ITGA10	1	144244532	3	0.950322
rs11590105	ITGA10	1	144244532	4	0.565728

rs2282014	ITGA10	1	144253638	7	7.245645
rs6670984	ANKRD35	1	144272951	3	1.916878
rs11579366	ANKRD35	1	144273650	3	0.253901
rs11584787	PRKAB2	1	145095103	8	0.873075
rs11584787	PRKAB2	1	145095103	7	0.039506
rs11584787	PRKAB2	1	145095103	6	0.026591
rs11584787	PRKAB2	1	145095103	3	0.003711
rs11584787	PRKAB2	1	145095103	4	0.446762
rs11584787	PRKAB2	1	145095103	1	6.377102
rs4950315	CHD1L	1	145202716	5	0.759204
rs4950315	CHD1L	1	145202716	1	5.852651
rs17356233	CHD1L	1	145207126	5	0.094295
rs17356233	CHD1L	1	145207126	1	5.930043
rs10900376	BCL9	1	145479986	5	1.225995
rs2153463	ACP6	1	145590934	7	0.174556
rs2153463	ACP6	1	145590934	8	4.657569
rs2153463	ACP6	1	145590934	6	5.42001
rs2153463	ACP6	1	145590934	3	0.540828
rs563625	FLJ39739	1	146317915	5	0.786154
rs563625	FLJ39739	1	146317915	4	0.363992
rs563625	FLJ39739	1	146317915	7	0.230404
rs563625	FLJ39739	1	146317915	6	0.783635
rs563625	FLJ39739	1	146317915	3	0.901611
rs4649592	LOC729135	1	147941416	5	1.356346
rs1044808	BOLA1	1	148138529	5	0.028183
rs1044808	BOLA1	1	148138529	4	0.332166
rs7534365	SV2A	1	148142748	5	0.733528
rs12131104	SV2A	1	148155963	5	2.873734
SNP1-148172791	MTMR11	1	148172791	4	0.413444
SNP1-148172791	MTMR11	1	148172791	5	0.726677
rs11205303	MTMR11	1	148173037	2	2.354947
rs11205303	MTMR11	1	148173037	5	0.525026
rs11205303	MTMR11	1	148173037	7	0.848441
rs1868992	MTMR11	1	148174732	5	0.801227
rs2147324	C1orf51	1	148522211	7	1.011036
rs2147324	C1orf51	1	148522211	6	0.779526
SNP1-14854717	MRPS21	1	148547174	7	2.166441
rs8006	MRPS21	1	148547425	5	0.180084
rs8006	MRPS21	1	148547425	4	1.260679
rs8006	MRPS21	1	148547425	6	3.568625
rs8006	MRPS21	1	148547425	7	0.342625
SNP1-14869656	KIAA0460	1	148696568	5	1.072601
SNP1-14874997	ECM1	1	148749979	3	0.54904
SNP1-14874997	ECM1	1	148749979	6	5.070508
SNP1-14879266	ADAMTSL4	1	148792668	6	6.406791
SNP1-14879266	ADAMTSL4	1	148792668	4	5.871452
SNP1-14879266	ADAMTSL4	1	148792668	3	0.301132

SNP1-148792668	ADAMTSL4	1	148792668	7	0.361317
rs7517	ENSA	1	148863035	3	0.673877
rs41271951	CTSS	1	149003844	5	2.709787
rs7532008	ANXA9	1	149226974	4	0.6375
rs7532008	ANXA9	1	149226974	3	0.453961
SNP1-149241732	FAM63A	1	149241732	3	0.021678
SNP1-149241732	FAM63A	1	149241732	4	7.22042
rs12068365	BNIPL	1	149282492	2	4.229685
rs12068365	BNIPL	1	149282492	5	0.169641
rs6587553	BNIPL	1	149285804	3	0.576862
rs6587553	BNIPL	1	149285804	1	5.230927
rs6587553	BNIPL	1	149285804	8	0.77257
rs4970998	MGC29891	1	149357805	8	0.152943
rs4970998	MGC29891	1	149357805	3	0.44506
rs4970998	MGC29891	1	149357805	1	3.952494
rs4971005	SEMA6C	1	149371222	3	0.69551
rs4971005	SEMA6C	1	149371222	8	3.085917
rs4971005	SEMA6C	1	149371222	1	5.127352
rs10788800	SEMA6C	1	149371574	3	0.423295
rs10788800	SEMA6C	1	149371574	1	0.449931
rs4971007	SEMA6C	1	149374761	8	0.777477
rs4971007	SEMA6C	1	149374761	1	5.482027
rs4971007	SEMA6C	1	149374761	3	1.289665
rs10749661	PSMD4	1	149505675	2	3.058542
rs10749661	PSMD4	1	149505675	8	1.314469
rs10749661	PSMD4	1	149505675	3	0.185279
rs10749661	PSMD4	1	149505675	7	0.394105
rs1056847	PI4KB	1	149554796	7	5.267722
rs1056847	PI4KB	1	149554796	3	0.142866
rs2296840	PSMB4	1	149638671	4	0.364174
rs12125310	POGZ	1	149698256	3	0.427297
rs12125310	POGZ	1	149698256	5	0.395421
rs12125310	POGZ	1	149698256	2	4.194553
rs12038198	CGN	1	149763826	2	4.531277
rs3828054	TUFT1	1	149779519	7	1.200846
rs12749	TUFT1	1	149822365	1	6.154799
rs1048694	SNX27	1	149936621	2	1.35099
rs1048694	SNX27	1	149936621	1	0.169826
rs1048694	SNX27	1	149936621	3	0.20046
rs3790508	TNRC4	1	149942110	7	0.601572
rs12409493	LINGO4	1	150039791	3	0.642467
rs2932586	KRT8P28	1	150189432	4	0.114961
rs2932586	KRT8P28	1	150189432	6	1.787791
rs1496045	HRNR	1	150451328	6	0.51959
rs1496045	HRNR	1	150451328	1	3.178885
rs4845423	HRNR	1	150451574	1	3.636425
rs6587651	HRNR	1	150459745	1	4.941292

rs6587651	HRNR	1	150459745	6	0.034261
rs12405278	FLG	1	150548891	6	0.737196
rs12405278	FLG	1	150548891	1	2.041694
rs2065954	FLG2	1	150587968	1	2.648454
rs2282302	FLG2	1	150595993	1	1.947453
rs2282303	FLG2	1	150596059	1	3.376245
rs3818831	FLG2	1	150597864	1	1.711075
rs10888486	CRNN	1	150648686	1	1.073428
rs1053588	CRCT1	1	150754687	7	1.275216
SNP1-15091527	LCE2C	1	150915275	1	1.865088
SNP1-15091527	LCE2C	1	150915275	6	0.664887
SNP1-15091527	LCE2C	1	150915275	2	1.362272
SNP1-15091527	LCE2C	1	150915275	5	1.151778
SNP1-15091527	LCE2C	1	150915275	7	0.805211
SNP1-15092642	LCE2B	1	150926429	5	1.665543
rs41308367	LCE2B	1	150926472	5	1.683917
rs4845480	KPRP	1	150999060	8	2.27248
rs4845480	KPRP	1	150999060	7	1.873733
rs16834461	KPRP	1	150999191	7	1.520039
rs4329520	KPRP	1	150999925	7	1.567607
rs4329520	KPRP	1	150999925	5	0.397001
rs4329520	KPRP	1	150999925	1	0.285191
rs4329520	KPRP	1	150999925	8	1.283472
rs2297487	KPRP	1	151001014	7	2.409266
rs1048534	LCE1C	1	151044198	1	2.111752
rs1048534	LCE1C	1	151044198	7	1.439214
rs3170863	SPRR4	1	151211218	7	0.812436
rs6671524	SPRR3	1	151240871	3	0.161236
rs3181787	SPRR1B	1	151271433	2	0.11492
rs3181787	SPRR1B	1	151271433	1	1.430324
rs3181787	SPRR1B	1	151271433	3	0.107894
rs3181787	SPRR1B	1	151271433	8	0.513601
rs3181787	SPRR1B	1	151271433	4	0.189082
rs1048296	SPRR2D	1	151278864	1	1.031733
rs1048296	SPRR2D	1	151278864	8	0.890225
rs1048296	SPRR2D	1	151278864	3	1.489785
rs509194	SPRR2G	1	151388795	8	4.479249
rs843971	PGLYRP3	1	151544047	6	1.962706
rs843971	PGLYRP3	1	151544047	5	0.056266
rs843971	PGLYRP3	1	151544047	7	1.805915
rs843971	PGLYRP3	1	151544047	1	5.653313
rs2570440	PGLYRP4	1	151569451	7	2.187348
rs10888551	PGLYRP4	1	151569511	1	0.906617
rs3006448	PGLYRP4	1	151580777	7	3.21326
rs3014863	PGLYRP4	1	151582203	7	0.8296
rs3818632	S100A13	1	151871143	6	1.660625
rs3818632	S100A13	1	151871143	8	3.972158

rs3818632	S100A13	1	151871143	3	1.054189
rs3818632	S100A13	1	151871143	7	1.142844
rs3818632	S100A13	1	151871143	4	1.925241
GA019434	NPR1	1	151928964	1	4.172049
GA019434	NPR1	1	151928964	3	1.113023
rs11264680	CRTC2	1	152192702	8	3.786693
rs11264680	CRTC2	1	152192702	6	0.747867
rs11264680	CRTC2	1	152192702	1	1.076805
rs11264680	CRTC2	1	152192702	2	1.142326
rs11264680	CRTC2	1	152192702	7	3.101328
rs11264875	NUP210L	1	152261271	7	1.081822
rs11264875	NUP210L	1	152261271	6	0.316571
rs11264875	NUP210L	1	152261271	1	1.675789
rs7654	TPM3	1	152396254	8	5.368605
rs1194587	ATP8B2	1	152583777	5	1.319577
rs1194587	ATP8B2	1	152583777	1	3.379526
rs1194587	ATP8B2	1	152583777	3	1.348153
rs1194587	ATP8B2	1	152583777	6	1.251071
rs1194587	ATP8B2	1	152583777	7	0.625739
rs2297607	ATP8B2	1	152587566	8	0.09924
rs2297607	ATP8B2	1	152587566	3	0.509497
rs2297607	ATP8B2	1	152587566	5	0.771063
rs8192282	IL6R	1	152668303	7	0.803388
rs2229238	IL6R	1	152704520	7	0.393201
rs12568083	SHE	1	152722573	6	1.216002
rs4845635	SHE	1	152740755	6	2.815898
rs4845635	SHE	1	152740755	7	2.387409
rs12753680	TDRD10	1	152741524	6	1.026462
rs12750774	TDRD10	1	152783101	1	2.312573
rs12750774	TDRD10	1	152783101	5	0.894201
rs2072660	CHRNB2	1	152815345	1	0.301104
rs2072660	CHRNB2	1	152815345	2	0.746818
rs1131820	KCNN3	1	153011476	6	1.019098
GA001167	PMVK	1	153175793	2	4.309876
GA001167	PMVK	1	153175793	1	0.681524
GA001167	PMVK	1	153175793	8	7.118739
GA001167	PMVK	1	153175793	6	5.83227
rs10908453	DCST2	1	153272715	5	0.2578
rs10908453	DCST2	1	153272715	3	0.047853
rs6680600	DCST1	1	153273623	5	0.183612
rs6680600	DCST1	1	153273623	3	0.359838
rs6680600	DCST1	1	153273623	4	0.365313
SNP1-153300542	ADAM15	1	153300542	6	0.458549
SNP1-153300542	ADAM15	1	153300542	5	1.456747
SNP1-153300542	ADAM15	1	153300542	8	1.337548
rs17723260	EFNA3	1	153325287	5	0.512172
rs17723260	EFNA3	1	153325287	7	0.81395

rs17723260	EFNA3	1	153325287	1	2.67432
rs17723260	EFNA3	1	153325287	6	0.783001
rs4745	EFNA1	1	153372851	4	5.169291
rs4745	EFNA1	1	153372851	5	0.306804
rs4745	EFNA1	1	153372851	6	2.655385
rs4745	EFNA1	1	153372851	2	5.852759
rs3814316	TRIM46	1	153416342	5	4.299625
rs3814316	TRIM46	1	153416342	8	0.36268
rs3814316	TRIM46	1	153416342	4	0.327465
rs1611772	MUC1	1	153425229	3	0.572023
rs4072037	MUC1	1	153428691	4	2.965868
rs4072037	MUC1	1	153428691	2	6.098889
rs4072037	MUC1	1	153428691	8	3.814741
rs4072037	MUC1	1	153428691	7	3.696761
VG01S1161	GBA	1	153472841	3	2.853908
rs12034326	GBA	1	153481097	5	0.041387
rs12034326	GBA	1	153481097	6	0.065627
rs12034326	GBA	1	153481097	4	0.753244
rs3180018	SCAMP3	1	153496755	5	0.38194
rs3180018	SCAMP3	1	153496755	4	0.179056
rs1046188	SCAMP3	1	153498698	4	5.234737
rs1046188	SCAMP3	1	153498698	5	5.913235
rs932972	PKLR	1	153526720	5	1.176796
rs932972	PKLR	1	153526720	6	0.858445
rs932972	PKLR	1	153526720	4	1.23542
rs932972	PKLR	1	153526720	8	0.354026
rs1052177	PKLR	1	153526974	5	0.873039
rs1052177	PKLR	1	153526974	4	0.742034
rs1052176	PKLR	1	153527007	5	2.895173
rs1052176	PKLR	1	153527007	4	3.274442
rs1052176	PKLR	1	153527007	6	3.312998
rs1052176	PKLR	1	153527007	8	3.231238
rs1052176	PKLR	1	153527007	2	4.213713
rs41264231	ASH1L	1	153584306	7	0.053448
rs7539	YY1AP1	1	153896107	1	6.479892
rs3820594	SYT11	1	154096135	5	0.542932
rs928596	ARHGEF2	1	154214499	6	0.330681
rs928596	ARHGEF2	1	154214499	4	0.222291
rs928596	ARHGEF2	1	154214499	5	0.582378
rs1889533	ARHGEF2	1	154214554	6	3.771699
rs1889533	ARHGEF2	1	154214554	4	1.358244
rs1889533	ARHGEF2	1	154214554	5	1.145713
rs2297792	UBQLN4	1	154278068	4	1.715981
rs2297792	UBQLN4	1	154278068	5	0.187356
rs11264432	MEX3A	1	154309539	1	0.325066
rs11264432	MEX3A	1	154309539	4	0.473118
rs11264432	MEX3A	1	154309539	7	0.171131

rs11264432	MEX3A	1	154309539	6	4.561884
rs3814314	MEX3A	1	154311807	7	0.441464
rs3814314	MEX3A	1	154311807	5	0.313174
rs2075165	PAQR6	1	154482523	5	0.54889
rs2075165	PAQR6	1	154482523	1	1.364008
rs2273833	SMG5	1	154499813	7	0.211596
rs2273833	SMG5	1	154499813	4	5.991758
rs2273833	SMG5	1	154499813	6	0.657498
rs1570805	C1orf85	1	154531272	1	3.147554
rs2245623	RHBG	1	154613755	3	3.41898
rs1925950	MEF2D	1	154717364	3	0.699339
rs1925950	MEF2D	1	154717364	8	3.244875
rs11264498	IQGAP3	1	154793011	3	0.156754
rs11264498	IQGAP3	1	154793011	6	0.00838
rs11264498	IQGAP3	1	154793011	2	4.358101
rs1056695	BCAN	1	154888876	1	0.063252
rs11582300	NES	1	154906377	3	0.52654
rs11582300	NES	1	154906377	7	1.09419
rs3748571	NES	1	154906932	4	0.125889
rs3828043	NES	1	154907127	8	1.670361
rs3828043	NES	1	154907127	2	1.517966
rs3828043	NES	1	154907127	4	0.933125
rs951781	NES	1	154908161	7	4.277486
rs951781	NES	1	154908161	3	0.15064
rs11579761	ISG20L2	1	154959642	6	0.206476
rs926103	SH2D2A	1	155051606	8	0.871745
rs926103	SH2D2A	1	155051606	3	0.095674
rs926103	SH2D2A	1	155051606	6	0.07289
rs926103	SH2D2A	1	155051606	7	0.520919
SNP1-155080651	INSRR	1	155080651	1	1.715534
SNP1-155080651	INSRR	1	155080651	3	0.577945
rs6337	NTRK1	1	155115619	2	1.396891
rs6337	NTRK1	1	155115619	6	1.422735
rs6337	NTRK1	1	155115619	4	0.201303
rs41299597	PEAR1	1	155149381	1	1.745572
rs822441	PEAR1	1	155149620	6	1.518532
rs822441	PEAR1	1	155149620	1	0.512115
rs11264581	PEAR1	1	155150117	6	1.801813
rs12137505	PEAR1	1	155150170	6	1.162544
rs12137505	PEAR1	1	155150170	7	0.412999
rs12137505	PEAR1	1	155150170	1	1.192102
rs12137505	PEAR1	1	155150170	3	0.761964
rs4661012	PEAR1	1	155152115	3	1.645046
rs4661012	PEAR1	1	155152115	7	0.326486
SNP1-155152391	PEAR1	1	155152391	2	1.343325
SNP1-155152391	PEAR1	1	155152391	8	7.315206
rs12119908	C1orf92	1	155168846	8	0.087036

rs822430	C1orf92	1	155168883	7	1.21861
rs822430	C1orf92	1	155168883	8	0.173986
rs822430	C1orf92	1	155168883	3	1.358448
rs3187878	ARHGEF11	1	155171268	8	0.403761
SNP1-155173739	ARHGEF11	1	155173739	7	0.822204
SNP1-155173739	ARHGEF11	1	155173739	3	0.615615
rs3845586	NULL	1	155751077	1	1.694735
rs6427384	FCRL5	1	155775506	3	0.470068
rs12036228	FCRL5	1	155780715	7	1.924124
rs12036228	FCRL5	1	155780715	5	2.455807
rs12036228	FCRL5	1	155780715	1	2.766108
rs849827	FCRL4	1	155811855	7	2.245318
rs849827	FCRL4	1	155811855	1	2.902624
rs849827	FCRL4	1	155811855	2	1.364208
rs6691569	FCRL3	1	155914722	7	1.1627
rs6691569	FCRL3	1	155914722	3	1.471985
rs11805944	FCRL3	1	155914779	8	2.418705
rs2282283	FCRL3	1	155914995	1	2.812054
rs12129399	FCRL2	1	155982430	1	5.191656
rs2873413	FCRL2	1	156004128	1	0.155465
rs2873413	FCRL2	1	156004128	4	0.219548
rs2873413	FCRL2	1	156004128	3	0.072596
rs1016849	FCRL1	1	156031588	3	2.977266
rs36008419	KIRREL	1	156324257	4	0.011289
rs36008419	KIRREL	1	156324257	1	5.530208
rs36008419	KIRREL	1	156324257	6	0.005561
rs16840096	CD1B	1	156564584	4	0.15836
rs6679056	OR10R2	1	156716938	6	4.255211
rs6679056	OR10R2	1	156716938	7	4.393605
rs6679056	OR10R2	1	156716938	1	3.63137
rs1418843	OR10R2	1	156717006	7	2.774882
rs857825	OR6N1	1	157002315	4	2.114147
rs857825	OR6N1	1	157002315	7	4.041589
rs857825	OR6N1	1	157002315	5	2.213307
rs857825	OR6N1	1	157002315	3	2.043073
rs857825	OR6N1	1	157002315	1	3.503987
rs857826	OR6N1	1	157002364	7	3.77756
rs857826	OR6N1	1	157002364	4	4.531797
rs857826	OR6N1	1	157002364	3	4.150402
rs857826	OR6N1	1	157002364	1	3.460541
rs1864346	OR6N1	1	157003069	5	0.36258
rs1864346	OR6N1	1	157003069	7	1.596659
rs1864346	OR6N1	1	157003069	1	0.083544
rs1864346	OR6N1	1	157003069	3	0.082261
rs2276404	IFI16	1	157246574	2	2.143689
rs2276404	IFI16	1	157246574	7	0.203437
rs866484	IFI16	1	157253101	8	0.207573

rs866484	IFI16	1	157253101	3	0.502198
rs866484	IFI16	1	157253101	2	2.101428
rs3737523	IFI16	1	157288351	6	5.164391
rs862999	CADM3	1	157436265	7	2.226276
rs862999	CADM3	1	157436265	4	0.541431
rs862999	CADM3	1	157436265	3	0.875864
rs13962	DARC	1	157442151	5	3.110081
rs13962	DARC	1	157442151	2	1.517465
rs13962	DARC	1	157442151	4	1.929071
rs13962	DARC	1	157442151	3	1.288363
rs1205	CRP	1	157948857	5	0.251342
rs1205	CRP	1	157948857	2	1.921305
SNP1-15804553	FCRL6	1	158045534	1	1.94077
SNP1-15804553	FCRL6	1	158045534	5	0.964555
SNP1-15805199	FCRL6	1	158051994	1	2.594234
SNP1-15806653	SLAMF8	1	158066534	1	3.177973
SNP1-15806653	SLAMF8	1	158066534	7	0.524483
rs2494507	VSIG8	1	158099049	8	1.491436
rs7550537	CCDC19	1	158117025	7	5.357912
rs16842789	CCDC19	1	158120875	2	1.327544
rs12409352	PIGM	1	158267059	2	6.433836
rs1053074	KCNJ10	1	158275745	3	0.794949
rs1053074	KCNJ10	1	158275745	5	0.539986
rs1053074	KCNJ10	1	158275745	7	0.008391
SNP1-15832414	KCNJ9	1	158324146	5	0.736028
SNP1-15832414	KCNJ9	1	158324146	2	0.85998
SNP1-15832414	KCNJ9	1	158324146	1	0.568443
GA001274	KCNJ9	1	158324588	1	1.146538
GA001274	KCNJ9	1	158324588	3	0.537638
GA001274	KCNJ9	1	158324588	5	0.964041
rs2753268	KCNJ9	1	158324876	7	0.665982
rs2753268	KCNJ9	1	158324876	1	4.165942
rs2753268	KCNJ9	1	158324876	5	0.139735
rs6427504	ATP1A4	1	158391499	8	1.796467
rs11265338	ATP1A4	1	158400829	1	1.487648
rs11265338	ATP1A4	1	158400829	3	1.63181
rs10594	PEX19	1	158513809	3	0.910641
rs7521304	NHLH1	1	158608639	6	0.039703
rs7521304	NHLH1	1	158608639	3	0.776714
rs3766388	CD84	1	158784103	7	0.79775
rs1061217	SLAMF1	1	158846639	5	3.464283
rs1061217	SLAMF1	1	158846639	3	2.089279
rs1061217	SLAMF1	1	158846639	8	3.819994
rs1061217	SLAMF1	1	158846639	1	5.19638
rs1061217	SLAMF1	1	158846639	7	2.731975
rs1061217	SLAMF1	1	158846639	4	3.19056
rs1126644	CD48	1	158915173	1	2.541125

rs1126644	CD48	1	158915173	5	1.742855
rs1980606	CD48	1	158921410	1	1.16972
rs1980606	CD48	1	158921410	3	0.401803
rs11265473	SLAMF7	1	158990072	1	2.996341
rs518721	SLAMF7	1	158990141	2	1.4032
rs518721	SLAMF7	1	158990141	1	0.778081
rs509749	LY9	1	159060184	6	0.735186
SNP1-159187038	ITLN2	1	159187038	1	1.915421
SNP1-159187038	ITLN2	1	159187038	3	3.23751
SNP1-159187038	ITLN2	1	159187038	6	2.863929
SNP1-159187038	ITLN2	1	159187038	7	1.832344
SNP1-159187038	ITLN2	1	159187038	5	1.558245
SNP1-159187038	ITLN2	1	159187038	4	2.020658
SNP1-159187038	ITLN2	1	159187038	2	1.411713
rs1062826	F11R	1	159234629	3	0.051007
rs1062826	F11R	1	159234629	5	0.264366
rs1062826	F11R	1	159234629	8	0.173376
rs1062826	F11R	1	159234629	4	0.465369
GA019599	F11R	1	159257530	5	0.152491
GA019599	F11R	1	159257530	8	5.502296
GA019599	F11R	1	159257530	4	0.598624
GA019599	F11R	1	159257530	3	0.644303
rs3737787	USF1	1	159276147	2	4.545299
rs3737787	USF1	1	159276147	3	0.726507
rs3737787	USF1	1	159276147	8	0.636367
rs3737787	USF1	1	159276147	5	0.340154
rs3737787	USF1	1	159276147	4	0.17402
rs2774279	ARHGAP30	1	159284180	5	0.001795
rs2774279	ARHGAP30	1	159284180	6	0.055721
rs2301286	PPOX	1	159402848	6	1.635977
rs3813620	B4GALT3	1	159411629	1	4.76046
rs3813620	B4GALT3	1	159411629	4	0.104775
rs4233367	ADAMTS4	1	159429661	3	0.584901
rs4233367	ADAMTS4	1	159429661	5	0.118449
GA001306	ADAMTS4	1	159434628	3	0.327976
rs3813623	NDUFS2	1	159435789	1	1.466851
rs3813623	NDUFS2	1	159435789	2	0.995339
rs3813623	NDUFS2	1	159435789	5	0.313285
rs1136207	NDUFS2	1	159450140	5	3.881138
rs1136207	NDUFS2	1	159450140	1	0.695654
rs1136207	NDUFS2	1	159450140	2	0.226851
rs1136207	NDUFS2	1	159450140	6	0.428351
rs3813628	TOMM40L	1	159462790	5	0.205259
rs3813628	TOMM40L	1	159462790	6	1.417828
rs3813628	TOMM40L	1	159462790	1	1.832831
rs2307424	NR1I3	1	159469229	5	0.720735
rs2307424	NR1I3	1	159469229	6	0.115288

rs2307424	NR1I3	1	159469229	1	1.974622
rs4477295	PCP4L1	1	159520904	3	1.200048
rs8266	SDHC	1	159599232	7	1.281602
rs12239492	SDHC	1	159600899	7	0.520654
rs16832869	C1orf192	1	159601359	7	1.70487
rs1801274	FCGR2A	1	159746369	3	0.263176
rs1079109	HSPA6	1	159761664	7	0.453157
rs1079109	HSPA6	1	159761664	2	2.970204
rs753856	HSPA6	1	159762758	2	4.296457
rs753856	HSPA6	1	159762758	7	0.868497
rs844	FCGR2B	1	159914157	1	3.255604
rs844	FCGR2B	1	159914157	7	2.955834
rs844	FCGR2B	1	159914157	2	4.302784
rs4301614	FCRLA	1	159948472	4	1.098516
rs11746	FCRLA	1	159949760	8	1.223699
rs11746	FCRLA	1	159949760	2	4.2014
rs11746	FCRLA	1	159949760	7	0.648841
rs7520519	FCRLB	1	159962263	2	4.212012
rs7520519	FCRLB	1	159962263	7	3.217548
rs7520519	FCRLB	1	159962263	4	0.787641
rs7520519	FCRLB	1	159962263	8	4.108713
rs12022536	OLFML2B	1	160242858	3	0.220816
rs348624	NOS1AP	1	160601880	8	1.344403
rs348624	NOS1AP	1	160601880	7	1.661545
rs2282397	C1orf111	1	160610726	1	1.989987
rs2282397	C1orf111	1	160610726	5	0.290763
rs34001279	SH2D1B	1	160633727	5	0.000768
rs2298258	DDR2	1	161003740	2	4.604177
rs2298258	DDR2	1	161003740	3	0.192941
rs12134171	C1orf110	1	161089164	5	0.29663
SNP1-161092081	C1orf110	1	161092081	5	0.673882
SNP1-161092081	C1orf110	1	161092081	3	0.816413
rs10917682	RGS5	1	161378828	1	2.890115
rs1056515	RGS5	1	161379884	1	4.26903
rs1056515	RGS5	1	161379884	4	0.165608
rs1056515	RGS5	1	161379884	6	0.579543
rs2661280	RGS5	1	161379999	7	1.354069
rs2661280	RGS5	1	161379999	8	0.583147
rs2661280	RGS5	1	161379999	3	0.362888
rs1056514	RGS5	1	161380190	8	1.313485
rs1056514	RGS5	1	161380190	3	1.287852
rs1056514	RGS5	1	161380190	7	1.234827
rs3806368	RGS5	1	161380502	3	0.765816
rs1054943	RGS5	1	161380720	3	0.181062
rs3820487	RGS5	1	161380982	6	1.933497
rs3806366	RGS5	1	161381945	4	1.542538
rs3806366	RGS5	1	161381945	3	0.212976

rs3806365	RGS5	1	161382055	6	1.106531
rs3806365	RGS5	1	161382055	4	1.01715
rs3806365	RGS5	1	161382055	3	0.446842
rs1395960	RGS5	1	161382146	3	5.04008
rs1395960	RGS5	1	161382146	4	2.571579
rs1395960	RGS5	1	161382146	6	0.200922
rs1509022	NUF2	1	161575843	1	0.276338
rs1509022	NUF2	1	161575843	2	2.427895
rs17402900	LMX1A	1	163438301	2	3.031007
rs17402900	LMX1A	1	163438301	7	2.639434
rs16840972	LMX1A	1	163438629	1	2.454268
rs9970062	LMX1A	1	163485303	5	0.021353
rs2134095	RXRG	1	163644176	1	0.748561
rs113471	RXRG	1	163664739	3	0.2318
rs367199	LRRC52	1	163799812	5	0.96918
rs11585763	LOC401975	1	164258785	3	0.2137
rs6692476	LOC401975	1	164258836	3	0.523792
rs6692476	LOC401975	1	164258836	4	0.269443
rs12741275	FMO9P	1	164860378	3	0.983735
rs524612	LOC646688	1	164983265	3	4.862392
rs524612	LOC646688	1	164983265	4	0.069038
rs1815531	LOC646688	1	164983830	8	0.066153
rs1815531	LOC646688	1	164983830	2	0.288868
rs1815531	LOC646688	1	164983830	3	0.587243
rs1815531	LOC646688	1	164983830	1	0.032797
rs1815531	LOC646688	1	164983830	6	0.016422
rs12461	GPA33	1	165289005	5	0.842624
rs12461	GPA33	1	165289005	4	2.029991
rs3753914	GPA33	1	165289325	3	0.156821
rs3753914	GPA33	1	165289325	7	0.487923
rs3753914	GPA33	1	165289325	1	0.078937
rs12076250	GPA33	1	165289544	1	0.375077
rs12076250	GPA33	1	165289544	7	0.144802
rs12076250	GPA33	1	165289544	2	0.453159
rs12076250	GPA33	1	165289544	4	1.987738
rs2281962	GPA33	1	165326384	3	0.513074
rs2281962	GPA33	1	165326384	5	1.453744
rs267747	DUSP27	1	165364569	3	0.354653
rs1052231	CD247	1	165666698	6	0.416994
rs1052231	CD247	1	165666698	1	2.302581
rs1052230	CD247	1	165666705	6	0.077645
rs1052230	CD247	1	165666705	1	5.695127
rs3831958	CD247	1	165666815	6	1.690362
rs3831958	CD247	1	165666815	1	3.414022
rs12025624	CREG1	1	165789653	3	0.842756
rs2297777	RCSD1	1	165941277	6	0.068556
rs2297777	RCSD1	1	165941277	8	1.320106

rs2297777	RCSD1	1	165941277	4	1.815985
rs2297777	RCSD1	1	165941277	7	1.353067
rs8623	MPZL1	1	166026462	6	1.173371
rs203795	SAC	1	166084263	5	1.273895
rs2071921	SAC	1	166092109	5	0.061409
rs1050037	BRP44	1	166154040	7	0.502451
rs203861	BRP44	1	166160383	1	0.046795
rs203861	BRP44	1	166160383	5	0.34511
rs203861	BRP44	1	166160383	2	0.058801
SNP1-16637212(	GPR161	1	166372120	7	1.195625
rs3885717	TBX19	1	166548885	7	4.225162
rs3885717	TBX19	1	166548885	4	0.759348
rs3885717	TBX19	1	166548885	8	0.068087
rs3885717	TBX19	1	166548885	1	3.411772
rs3885717	TBX19	1	166548885	3	0.158507
rs1042882	XCL1	1	166817159	8	2.845695
rs1042882	XCL1	1	166817159	4	1.690091
rs16861320	DPT	1	166931970	4	0.686021
rs3795611	LOC391132	1	167174523	5	0.736251
rs2272801	C1orf114	1	167633140	6	0.205917
SNP1-16765758	C1orf114	1	167657581	5	0.284121
SNP1-16765758	C1orf114	1	167657581	1	0.226187
SNP1-16765758	C1orf114	1	167657581	4	0.393542
SNP1-16765758	C1orf114	1	167657581	6	0.566298
rs3820059	C1orf114	1	167657778	1	0.740988
rs3820059	C1orf114	1	167657778	3	0.869832
rs12091844	SLC19A2	1	167699897	4	5.641291
rs9332701	F5	1	167751391	1	1.831967
rs6030	F5	1	167765599	6	2.399204
rs6030	F5	1	167765599	3	2.31765
rs6032	F5	1	167778179	6	0.468518
rs6032	F5	1	167778179	2	1.421118
rs6032	F5	1	167778179	1	1.818593
SNP1-16777835	F5	1	167778358	6	2.793546
SNP1-16777835	F5	1	167778358	3	1.180658
rs4524	F5	1	167778379	6	0.029516
rs6016	F5	1	167778744	3	0.456429
rs6016	F5	1	167778744	6	0.498672
rs6035	F5	1	167788473	6	0.014247
rs6022	F5	1	167796450	8	1.695829
rs6029	F5	1	167796597	8	1.506464
rs6133	SELP	1	167831970	6	1.186301
rs6133	SELP	1	167831970	7	0.271226
rs909628	SELL	1	167927289	6	2.261294
rs4987310	SELL	1	167940462	4	0.321388
rs1051091	SELL	1	167944333	6	0.047482
rs1051091	SELL	1	167944333	4	0.501315

rs3766132	C1orf112	1	168031192	8	6.068785
rs3766132	C1orf112	1	168031192	7	0.573554
rs10919246	C1orf112	1	168031676	8	6.155009
rs10919246	C1orf112	1	168031676	5	7.131916
rs10919246	C1orf112	1	168031676	7	0.126662
rs6668114	C1orf112	1	168034704	7	5.368043
rs12143301	SCYL3	1	168090414	6	1.949268
rs520525	LOC730132	1	168904957	1	1.364215
rs2294740	C1orf129	1	169195295	7	2.033443
rs2266782	FMO3	1	169343590	5	0.728011
rs2266782	FMO3	1	169343590	4	1.188413
rs1736557	FMO3	1	169346704	7	0.693473
rs909530	FMO3	1	169349798	6	0.122326
rs909530	FMO3	1	169349798	4	0.050559
rs909530	FMO3	1	169349798	5	0.677147
rs2266780	FMO3	1	169349866	6	0.427047
rs2266780	FMO3	1	169349866	4	0.267669
rs2266780	FMO3	1	169349866	5	0.441377
rs11812061	FMO6P	1	169382269	6	1.071277
rs11812061	FMO6P	1	169382269	5	0.301388
rs11812061	FMO6P	1	169382269	4	0.326929
rs2272797	FMO6P	1	169385464	6	1.852505
rs2272797	FMO6P	1	169385464	5	0.557609
rs2272797	FMO6P	1	169385464	4	0.614623
rs7889839	FMO6P	1	169387797	6	0.289894
rs7889839	FMO6P	1	169387797	4	0.885837
rs7889839	FMO6P	1	169387797	5	0.112657
rs16864125	FMO6P	1	169396970	1	0.052943
rs16864125	FMO6P	1	169396970	2	0.663279
rs2020860	FMO2	1	169429207	1	3.374713
rs28369860	FMO2	1	169432427	1	5.991274
rs28369914	FMO2	1	169445114	2	5.064752
rs7512785	FMO2	1	169445911	7	3.396323
rs7512785	FMO2	1	169445911	4	2.924039
rs7877	FMO1	1	169521514	1	2.758985
rs235501	BAT2D1	1	169822789	2	5.172965
rs235501	BAT2D1	1	169822789	4	0.891637
SNP1-16988814	MYOC	1	169888148	5	2.163011
SNP1-16988814	MYOC	1	169888148	8	2.319808
rs9661596	LOC391135	1	169919287	2	2.567641
rs9661596	LOC391135	1	169919287	4	3.612651
rs9661596	LOC391135	1	169919287	6	1.272386
rs9661596	LOC391135	1	169919287	7	2.196912
rs2294719	KIAA0859	1	170017674	2	3.50914
rs2294719	KIAA0859	1	170017674	4	0.071629
rs2294719	KIAA0859	1	170017674	7	0.961984
rs2294719	KIAA0859	1	170017674	5	0.83717

rs2294719	KIAA0859	1	170017674	8	6.287059
rs2294720	KIAA0859	1	170017859	4	0.347494
rs2294720	KIAA0859	1	170017859	2	4.533059
rs2294720	KIAA0859	1	170017859	5	0.299382
rs2294720	KIAA0859	1	170017859	7	1.031061
rs2294720	KIAA0859	1	170017859	8	2.7485
rs2232825	KIAA0859	1	170030172	6	0.483916
rs2232825	KIAA0859	1	170030172	5	8.553045
rs3736790	DNM3	1	170223426	1	0.904675
rs3736790	DNM3	1	170223426	2	0.798079
rs3736790	DNM3	1	170223426	4	0.165492
rs3736790	DNM3	1	170223426	6	1.242746
rs10489282	DNM3	1	170643669	3	0.008415
rs9425601	DNM3	1	170643885	3	1.416796
rs9425601	DNM3	1	170643885	6	0.127142
rs9425601	DNM3	1	170643885	5	1.266272
rs9425601	DNM3	1	170643885	8	0.562372
rs17278379	DNM3	1	170647907	8	2.606968
rs17278379	DNM3	1	170647907	3	0.538339
rs7528296	DNM3	1	170648006	8	5.55884
rs7528296	DNM3	1	170648006	2	4.66255
rs7528296	DNM3	1	170648006	6	1.606599
rs7528296	DNM3	1	170648006	5	0.249893
rs2239816	C1orf9	1	170768266	1	4.864884
rs2239816	C1orf9	1	170768266	4	0.487203
rs2239816	C1orf9	1	170768266	5	0.815642
rs6663179	LOC391139	1	171377576	7	1.297616
rs6663179	LOC391139	1	171377576	3	1.486366
rs3900307	TNFSF4	1	171420065	3	0.421815
rs7514229	TNFSF4	1	171420927	1	0.508871
rs7514229	TNFSF4	1	171420927	3	0.51256
rs7514229	TNFSF4	1	171420927	8	0.022047
rs17372309	TNFSF4	1	171421162	1	2.237138
rs17372309	TNFSF4	1	171421162	8	0.696796
rs17372309	TNFSF4	1	171421162	6	0.048941
rs12140760	LOC646870	1	171696618	8	1.540313
rs12140760	LOC646870	1	171696618	4	1.481467
rs12140760	LOC646870	1	171696618	2	2.525564
rs12140760	LOC646870	1	171696618	3	0.719307
rs5878	SERPINC1	1	172145455	3	0.209087
rs5878	SERPINC1	1	172145455	4	2.941296
rs5877	SERPINC1	1	172145485	3	0.865274
rs5877	SERPINC1	1	172145485	4	1.55809
rs6703547	RC3H1	1	172174435	5	1.242817
rs6703547	RC3H1	1	172174435	4	1.072869
rs7516240	GPR52	1	172683854	3	0.775596
rs1034462	CACYBP	1	173246946	4	0.896182

rs1034462	CACYBP	1	173246946	6	0.76629
rs10912897	TNN	1	173380201	1	0.020835
rs859427	TNR	1	173615346	1	0.90976
rs859437	TNR	1	173622014	7	0.062708
rs859437	TNR	1	173622014	3	0.064326
rs859437	TNR	1	173622014	2	0.333243
rs12118034	PAPPA2	1	175075991	5	0.781372
rs1044299	PAPPA2	1	175078496	7	0.860453
rs1044299	PAPPA2	1	175078496	3	0.084672
rs1044299	PAPPA2	1	175078496	6	3.19263
rs1044299	PAPPA2	1	175078496	5	0.937813
rs1044299	PAPPA2	1	175078496	8	1.06814
rs2228956	ASTN1	1	175130490	3	1.163175
rs172917	ASTN1	1	175259176	8	0.252194
rs3176441	FAM5B	1	175465621	3	0.465916
rs3176443	FAM5B	1	175514477	8	4.069216
rs3829804	LOC400796	1	175629290	5	0.429146
rs3829804	LOC400796	1	175629290	3	1.043255
rs3829799	LOC400796	1	175629502	1	2.540114
SNP1-17616827	SEC16B	1	176168278	1	0.486341
SNP1-17616827	SEC16B	1	176168278	7	0.558409
SNP1-17616827	SEC16B	1	176168278	4	5.158698
SNP1-17616827	SEC16B	1	176168278	2	3.725993
rs3813649	SEC16B	1	176169348	4	1.046929
rs3813649	SEC16B	1	176169348	2	2.510652
rs3813649	SEC16B	1	176169348	7	0.653242
rs3813649	SEC16B	1	176169348	1	0.700013
rs591120	SEC16B	1	176169376	7	0.102005
rs7522194	SEC16B	1	176172092	1	0.81478
rs7522194	SEC16B	1	176172092	7	2.157454
rs7522194	SEC16B	1	176172092	4	0.36606
rs7522194	SEC16B	1	176172092	2	0.338587
rs12129817	SEC16B	1	176190063	3	1.363317
rs7413442	SEC16B	1	176196563	2	4.850297
rs7413442	SEC16B	1	176196563	3	0.662792
rs7413442	SEC16B	1	176196563	6	0.165163
rs12410468	LOC730102	1	176266390	5	0.882782
rs12410468	LOC730102	1	176266390	1	0.75852
rs12410468	LOC730102	1	176266390	2	0.377124
rs12410468	LOC730102	1	176266390	4	0.736899
rs2746325	FLJ44005	1	176730214	1	0.024865
rs10913567	C1orf220	1	176778564	1	1.098829
rs10913567	C1orf220	1	176778564	6	2.526634
rs12568310	C1orf220	1	176781245	1	6.836353
rs4650995	C1orf220	1	176783939	6	0.123943
rs4650995	C1orf220	1	176783939	3	1.106513
rs4650995	C1orf220	1	176783939	1	4.636266

rs4650996	C1orf220	1	176783988	1	0.907083
rs4233175	C1orf220	1	176784214	3	0.423495
rs10913569	C1orf220	1	176784631	3	1.506603
rs12026782	LOC646976	1	176960082	1	5.969296
rs1544124	ANGPTL1	1	177086782	7	1.863191
rs1544124	ANGPTL1	1	177086782	3	0.91671
rs4652352	FAM20B	1	177308923	6	3.725497
rs4652352	FAM20B	1	177308923	1	5.989799
rs4652352	FAM20B	1	177308923	8	0.513851
rs4652352	FAM20B	1	177308923	5	0.458644
rs4652352	FAM20B	1	177308923	7	1.878452
rs4652352	FAM20B	1	177308923	4	1.224034
rs4652352	FAM20B	1	177308923	3	0.62017
rs3766625	FAM20B	1	177310176	4	1.24544
rs3766625	FAM20B	1	177310176	7	5.469637
rs3766625	FAM20B	1	177310176	6	1.40193
rs2296377	TOR3A	1	177317923	8	0.460594
rs2296377	TOR3A	1	177317923	3	0.60269
rs2296377	TOR3A	1	177317923	1	2.956606
rs2296377	TOR3A	1	177317923	5	0.088422
rs1128952	TOR3A	1	177331748	3	0.884775
rs1128952	TOR3A	1	177331748	7	0.236163
rs1128952	TOR3A	1	177331748	8	7.782713
rs1128952	TOR3A	1	177331748	1	5.926745
rs1318056	ABL2	1	177378768	4	0.243206
rs11577579	C1orf125	1	177680728	8	3.904948
SNP1-17777066	C1orf125	1	177770666	8	1.292384
SNP1-17777066	C1orf125	1	177770666	1	2.118615
SNP1-17777066	C1orf125	1	177770666	5	1.333379
SNP1-17777066	C1orf125	1	177770666	2	0.136533
rs2274623	NPHS2	1	177786731	1	2.198743
rs744446	LOC126860	1	177824674	7	1.359518
rs744446	LOC126860	1	177824674	5	1.853521
rs12070589	TDRD5	1	177829330	5	3.011172
rs35448215	TDRD5	1	177897865	6	3.555233
rs35448215	TDRD5	1	177897865	2	0.992453
rs35448215	TDRD5	1	177897865	4	0.026457
rs35448215	TDRD5	1	177897865	1	2.367914
rs35448215	TDRD5	1	177897865	8	4.155967
rs1052447	LOC730113	1	177978272	5	0.077326
rs2477120	CEP350	1	178256365	1	0.114228
rs2477120	CEP350	1	178256365	2	2.588135
rs2477120	CEP350	1	178256365	8	0.715205
rs2477120	CEP350	1	178256365	4	0.156127
rs10913939	QSOX1	1	178411108	1	4.676075
rs2298206	QSOX1	1	178411711	4	5.061474
rs2298206	QSOX1	1	178411711	6	0.656624

rs17855475	QSOX1	1	178414635	1	5.543297
rs7521513	QSOX1	1	178426277	1	4.098741
rs17302207	KIAA1614	1	179171886	3	0.327674
rs3795505	KIAA1614	1	179171975	3	0.603455
rs3795504	KIAA1614	1	179172071	7	0.302294
rs3795504	KIAA1614	1	179172071	3	0.498055
rs12023606	KIAA1614	1	179181589	7	0.122802
rs870046	LOC391142	1	179189430	3	0.795666
rs870046	LOC391142	1	179189430	8	6.693924
rs870046	LOC391142	1	179189430	4	0.45683
rs6425663	IER5	1	179324497	4	1.834853
rs6425663	IER5	1	179324497	2	4.738962
rs3747955	IER5	1	179324936	2	1.315838
rs3747955	IER5	1	179324936	1	0.915663
rs16858474	ZNF648	1	180291476	1	3.551116
rs912902	GLUL	1	180627553	7	1.901845
rs912902	GLUL	1	180627553	2	5.254392
rs2038891	TEDDM1	1	180634171	2	2.192246
rs2038891	TEDDM1	1	180634171	4	0.794671
rs2038891	TEDDM1	1	180634171	3	0.780481
rs6424853	TEDDM1	1	180634516	4	0.042193
rs6424853	TEDDM1	1	180634516	3	0.701906
rs6424853	TEDDM1	1	180634516	6	0.171924
rs6698722	TEDDM1	1	180635701	2	1.565858
rs6674281	TEDDM1	1	180635856	6	0.531791
rs6674281	TEDDM1	1	180635856	3	0.675994
rs6674281	TEDDM1	1	180635856	4	0.463302
rs7551829	TEDDM1	1	180636361	2	3.014391
rs7551829	TEDDM1	1	180636361	7	0.345996
rs1048260	RNASEL	1	180809474	1	0.319444
rs1048260	RNASEL	1	180809474	2	4.794315
rs1048260	RNASEL	1	180809474	5	0.222972
rs680431	RGS16	1	180835352	5	11.91951
rs680431	RGS16	1	180835352	2	3.712713
rs680431	RGS16	1	180835352	7	4.74945
rs20562	LAMC1	1	181351309	2	4.835955
SNP1-18135237	LAMC1	1	181352378	5	0.234557
SNP1-18135237	LAMC1	1	181352378	6	7.725432
SNP1-18135237	LAMC1	1	181352378	8	9.10105
rs4651146	LAMC1	1	181372157	8	1.73932
rs4651146	LAMC1	1	181372157	6	2.955839
rs4651146	LAMC1	1	181372157	5	3.716442
rs3818419	LAMC1	1	181372328	5	0.298177
rs3818419	LAMC1	1	181372328	6	4.854343
rs3818419	LAMC1	1	181372328	8	1.077
rs3359	LAMC1	1	181378664	5	0.784013
rs3359	LAMC1	1	181378664	6	0.297917

rs3359	LAMC1	1	181378664	8	6.429233
rs1547714	LAMC1	1	181380742	8	3.793196
rs1547714	LAMC1	1	181380742	5	0.458771
rs1547714	LAMC1	1	181380742	6	6.183402
rs2274980	LAMC2	1	181451239	3	0.260862
rs2274980	LAMC2	1	181451239	4	0.299368
rs11586699	LAMC2	1	181451313	2	0.118088
rs11586699	LAMC2	1	181451313	1	0.15724
rs1129723	LAMC2	1	181454226	3	0.93218
rs1129723	LAMC2	1	181454226	4	5.255448
rs1129723	LAMC2	1	181454226	6	0.159344
rs10797863	LAMC2	1	181480033	6	1.403238
rs2021320	NMNAT2	1	181485971	6	7.309307
rs3738818	NMNAT2	1	181488256	4	0.225553
rs2298083	SMG7	1	181782051	6	9.506978
rs1044879	SMG7	1	181789375	8	8.631143
rs1044879	SMG7	1	181789375	3	0.325825
rs1044879	SMG7	1	181789375	2	2.079019
rs2274065	NCF2	1	181826327	8	0.950899
rs2231241	ARPC5	1	181863326	2	0.580788
rs1174656	APOBEC4	1	181883310	1	0.227646
rs10911388	APOBEC4	1	181883404	2	1.260676
rs10911391	APOBEC4	1	181883717	2	2.843606
rs1174658	APOBEC4	1	181883728	5	1.150218
rs1174658	APOBEC4	1	181883728	1	4.957939
rs6699011	APOBEC4	1	181889002	2	3.525192
rs1053092	GLT25D2	1	182171811	1	3.045991
rs1053092	GLT25D2	1	182171811	5	0.043783
rs1053092	GLT25D2	1	182171811	8	0.052538
rs2986573	GLT25D2	1	182172632	7	0.095287
rs2986573	GLT25D2	1	182172632	4	3.842867
rs2986573	GLT25D2	1	182172632	2	2.14672
rs2986573	GLT25D2	1	182172632	3	0.158912
rs11583641	GLT25D2	1	182172868	1	4.227698
rs11583641	GLT25D2	1	182172868	3	0.549109
rs11583641	GLT25D2	1	182172868	4	1.334757
rs11583641	GLT25D2	1	182172868	7	1.014262
rs3828027	GLT25D2	1	182173875	4	0.999606
rs3828027	GLT25D2	1	182173875	3	0.561864
rs2296713	GLT25D2	1	182176340	5	2.416471
rs2296713	GLT25D2	1	182176340	8	4.87115
rs7535067	C1orf21	1	182863207	2	5.455744
rs1046239	EDEM3	1	182926828	7	1.02391
rs1046239	EDEM3	1	182926828	1	8.702028
SNP1-182938721	EDEM3	1	182938721	4	7.730188
rs7360	FAM129A	1	183027056	1	2.893542
rs7360	FAM129A	1	183027056	7	0.635519

rs682331	FAM129A	1	183027988	3	0.027525
rs682331	FAM129A	1	183027988	8	0.855137
rs682331	FAM129A	1	183027988	7	0.199712
rs581100	FAM129A	1	183030690	8	5.443783
rs581100	FAM129A	1	183030690	3	0.255692
rs581100	FAM129A	1	183030690	1	4.95793
rs12030554	C1orf25	1	183354257	7	3.951737
rs12030554	C1orf25	1	183354257	8	0.180971
rs12030554	C1orf25	1	183354257	4	0.239792
rs12030554	C1orf25	1	183354257	5	0.136359
rs12030554	C1orf25	1	183354257	1	0.03165
rs950327	C1orf26	1	183402368	1	0.595633
rs950327	C1orf26	1	183402368	5	0.537988
rs950327	C1orf26	1	183402368	7	0.160209
rs950327	C1orf26	1	183402368	4	0.8846
rs10489579	C1orf26	1	183410344	4	2.969924
rs10489579	C1orf26	1	183410344	7	0.914518
rs10489579	C1orf26	1	183410344	8	0.295586
rs10489579	C1orf26	1	183410344	5	4.662103
rs10489579	C1orf26	1	183410344	1	0.256359
rs10489579	C1orf26	1	183410344	2	1.501136
rs12041704	C1orf26	1	183507097	1	1.132332
rs12041704	C1orf26	1	183507097	3	0.502231
SNP1-184218124	HMCN1	1	184218124	2	0.565396
SNP1-184218124	HMCN1	1	184218124	7	0.267034
SNP1-184218124	HMCN1	1	184218124	6	0.042785
SNP1-184218124	HMCN1	1	184218124	4	1.672797
SNP1-184218124	HMCN1	1	184218124	1	0.270926
SNP1-184218124	HMCN1	1	184218124	5	0.180704
SNP1-184218124	HMCN1	1	184218124	3	1.019963
SNP1-184218124	HMCN1	1	184218124	8	0.963516
rs6665753	HMCN1	1	184251140	8	3.304591
rs6665753	HMCN1	1	184251140	3	0.842176
rs6665753	HMCN1	1	184251140	7	1.01147
rs12129650	HMCN1	1	184293097	7	0.732261
rs12129650	HMCN1	1	184293097	8	0.576502
rs12129650	HMCN1	1	184293097	3	0.681591
rs12129650	HMCN1	1	184293097	5	2.029432
rs10911825	HMCN1	1	184368162	8	1.990168
rs10911825	HMCN1	1	184368162	7	1.069222
rs10911825	HMCN1	1	184368162	6	0.176638
rs2273779	PRG4	1	184540617	4	2.856689
rs3737940	PRG4	1	184544612	2	3.575294
rs3737940	PRG4	1	184544612	7	1.657882
rs3737940	PRG4	1	184544612	4	1.599115
rs3737940	PRG4	1	184544612	8	1.40183
rs3737940	PRG4	1	184544612	6	5.395527

SNP1-18458786	TPR	1	184587865	4	7.450393
SNP1-18458786	TPR	1	184587865	6	7.0783
rs8824	C1orf27	1	184656926	8	4.468291
rs8824	C1orf27	1	184656926	2	4.555503
rs8824	C1orf27	1	184656926	4	0.7265
rs8824	C1orf27	1	184656926	6	6.027863
rs35066734	PDC	1	184679536	7	1.589918
rs5277	PTGS2	1	184914820	4	1.09396
rs10753015	C1orf99	1	185877855	3	1.416573
rs16829304	LOC647132	1	188225406	2	4.991334
rs16829304	LOC647132	1	188225406	1	4.810873
rs4606	RGS2	1	191047795	4	5.011286
rs4606	RGS2	1	191047795	7	0.721335
rs4606	RGS2	1	191047795	3	0.358638
rs4606	RGS2	1	191047795	6	0.577914
rs10921310	GLRX2	1	191341274	5	0.119941
rs912071	GLRX2	1	191341731	5	0.324144
rs1065489	CFH	1	194976397	6	0.940693
rs1065489	CFH	1	194976397	2	4.941976
rs17514281	F13B	1	195292912	1	2.555566
SNP1-19533732	ASPM	1	195337320	4	0.174542
SNP1-19533732	ASPM	1	195337320	1	2.917385
SNP1-19533981	ASPM	1	195339819	3	0.204194
SNP1-19533981	ASPM	1	195339819	8	1.362376
rs4639796	ZBTB41	1	195393272	8	5.305836
rs4639796	ZBTB41	1	195393272	7	0.527495
rs10922251	DENND1B	1	195788433	1	4.343955
rs6677361	DENND1B	1	195788500	1	4.829303
rs6677361	DENND1B	1	195788500	4	1.381462
rs2270763	C1orf53	1	196141572	3	0.810939
rs2270763	C1orf53	1	196141572	4	1.94579
rs2270763	C1orf53	1	196141572	5	0.036768
rs2690034	NR5A2	1	198410467	5	0.332697
rs12744809	DDX59	1	198859948	1	0.210546
rs2808257	DDX59	1	198860628	1	2.058344
rs6665604	DDX59	1	198884861	6	3.55171
rs2271018	C1orf106	1	199134115	2	0.235655
rs2271018	C1orf106	1	199134115	8	6.227645
rs2271018	C1orf106	1	199134115	1	0.67965
rs2271018	C1orf106	1	199134115	4	0.517523
rs2271017	C1orf106	1	199134175	8	0.097947
rs2271017	C1orf106	1	199134175	1	4.554812
rs425079	C1orf81	1	199168039	1	1.994771
rs3208703	KIF21B	1	199206265	2	2.534878
rs3198583	KIF21B	1	199206803	2	1.982667
rs3198583	KIF21B	1	199206803	1	1.662516
rs3198583	KIF21B	1	199206803	4	2.576164

rs11586032	KIF21B	1	199209008	7	0.585363
rs13374149	CACNA1S	1	199279107	3	1.812706
rs2282415	DKFZp434B1231	1	199449986	5	2.246116
rs2275673	DKFZp434B1231	1	199450898	1	2.304916
rs2275673	DKFZp434B1231	1	199450898	5	2.595467
rs2275673	DKFZp434B1231	1	199450898	3	1.951803
rs3738270	DKFZp434B1231	1	199461742	2	1.676143
rs3738270	DKFZp434B1231	1	199461742	7	2.561794
rs3738270	DKFZp434B1231	1	199461742	4	2.393598
rs3738270	DKFZp434B1231	1	199461742	3	2.373413
rs14061	DKFZp434B1231	1	199464404	8	0.708986
rs14061	DKFZp434B1231	1	199464404	5	1.823185
rs14061	DKFZp434B1231	1	199464404	7	2.033058
rs14061	DKFZp434B1231	1	199464404	1	1.470493
rs2268147	PKP1	1	199519489	7	0.032474
rs2268147	PKP1	1	199519489	2	4.735168
rs11805972	LAD1	1	199622276	6	6.058415
rs3738281	LAD1	1	199624927	4	0.659556
rs2799680	TNNI1	1	199641300	8	1.64929
rs2799680	TNNI1	1	199641300	7	1.378996
rs1256945	TNNI1	1	199642281	8	0.377268
rs1256945	TNNI1	1	199642281	7	0.351239
rs1256946	TNNI1	1	199642374	7	0.460126
rs1256948	TNNI1	1	199642884	8	3.442449
rs1256948	TNNI1	1	199642884	6	0.724732
rs1262295	TNNI1	1	199644061	1	0.133672
rs4915521	TNNI1	1	199644508	1	1.47037
rs3738286	TNNI1	1	199653539	2	4.913628
rs12409710	PHLDA3	1	199701926	5	0.872991
rs1256930	PHLDA3	1	199704100	7	0.326106
rs2279681	TMEM58	1	200127639	4	0.335682
rs2279681	TMEM58	1	200127639	8	0.710593
rs2279681	TMEM58	1	200127639	5	0.083052
rs2279681	TMEM58	1	200127639	6	0.902636
rs2279681	TMEM58	1	200127639	7	0.399457
rs2279681	TMEM58	1	200127639	1	6.267824
rs8028	LMOD1	1	200132386	5	0.967452
rs2820312	LMOD1	1	200135880	5	0.188755
rs2819360	ELF3	1	200243877	7	0.217655
rs2819360	ELF3	1	200243877	4	0.372808
rs2819360	ELF3	1	200243877	3	0.315466
rs2819360	ELF3	1	200243877	1	5.019062
rs9427715	ELF3	1	200244450	4	0.753599
rs9427715	ELF3	1	200244450	7	0.811121
rs9427715	ELF3	1	200244450	5	0.732327
rs9427715	ELF3	1	200244450	1	3.027276
rs9427715	ELF3	1	200244450	3	0.029717

rs10920304	ELF3	1	200244946	1	3.898376
rs10920304	ELF3	1	200244946	4	0.31868
rs10920304	ELF3	1	200244946	3	0.65642
rs10920304	ELF3	1	200244946	7	0.723808
rs3806357	ELF3	1	200246078	7	0.103772
rs2819361	ELF3	1	200246742	4	7.291317
rs2819361	ELF3	1	200246742	1	4.465481
rs2819361	ELF3	1	200246742	3	0.264467
rs2819361	ELF3	1	200246742	5	0.316998
rs3795596	GPR37L1	1	200359038	1	0.289779
rs7516762	GPR37L1	1	200359203	1	1.574315
rs2070257	PTPN7	1	200383625	1	0.952626
rs10920362	LGR6	1	200449931	3	0.861938
SNP1-20055437	LGR6	1	200554377	2	0.448192
rs788792	LGR6	1	200555085	4	1.385593
rs4950858	PPP1R12B	1	200818723	5	0.880211
rs4950858	PPP1R12B	1	200818723	4	3.266855
rs4950858	PPP1R12B	1	200818723	3	0.3127
rs504261	SYT2	1	200832571	6	0.140905
rs504261	SYT2	1	200832571	3	0.560277
rs12139944	LOC641515	1	201061410	1	0.63017
rs12139944	LOC641515	1	201061410	3	0.584957
rs12139944	LOC641515	1	201061410	6	0.483377
rs12094889	LOC148709	1	201108956	1	1.253944
rs12094889	LOC148709	1	201108956	6	0.335713
rs12094889	LOC148709	1	201108956	7	1.470892
rs11590469	LOC148709	1	201109750	1	0.536299
rs11590469	LOC148709	1	201109750	7	0.773036
rs11590469	LOC148709	1	201109750	6	0.64237
rs6692075	LOC730246	1	201147925	7	0.981971
rs6692075	LOC730246	1	201147925	4	4.557161
rs6692075	LOC730246	1	201147925	8	5.50009
rs6692075	LOC730246	1	201147925	6	1.754055
rs6692075	LOC730246	1	201147925	1	1.34107
rs6692075	LOC730246	1	201147925	5	0.738551
rs3736314	PPFIA4	1	201291390	6	3.209509
rs6682652	PPFIA4	1	201312206	5	1.477861
rs6682652	PPFIA4	1	201312206	7	1.830916
GA001571	PPFIA4	1	201313089	4	1.454403
GA001571	PPFIA4	1	201313089	7	0.673288
rs16851198	LOC730226	1	201509317	5	2.107138
rs2297952	LOC730226	1	201509403	5	1.903656
rs7208	FMOD	1	201576503	7	2.514427
rs7208	FMOD	1	201576503	8	1.264456
rs7208	FMOD	1	201576503	5	2.849387
rs4605	FMOD	1	201576589	1	1.386064
rs4605	FMOD	1	201576589	5	4.205073

rs4605	FMOD	1	201576589	2	0.173139
rs4605	FMOD	1	201576589	3	0.282822
rs3738022	FMOD	1	201577839	5	2.603594
rs3738022	FMOD	1	201577839	7	2.972874
rs3738022	FMOD	1	201577839	8	2.69133
rs1166	PRELP	1	201722844	5	4.168189
rs1166	PRELP	1	201722844	3	0.47224
rs1166	PRELP	1	201722844	6	5.36838
rs1166	PRELP	1	201722844	4	0.659929
rs28484961	LOC647250	1	201764901	5	1.056415
rs2298870	SNRPE	1	202105896	5	4.314959
rs2298870	SNRPE	1	202105896	3	0.026856
rs2298870	SNRPE	1	202105896	6	1.259447
rs2298870	SNRPE	1	202105896	1	4.515594
rs4598553	SNRPE	1	202106660	3	1.603899
rs4598553	SNRPE	1	202106660	6	0.497293
rs4598553	SNRPE	1	202106660	1	6.695474
rs4598553	SNRPE	1	202106660	5	0.102583
rs1138617	SOX13	1	202363253	5	0.907545
SNP1-20238757	ETNK2	1	202387575	5	4.023628
rs6689607	GOLT1A	1	202438719	5	0.154817
rs6689607	GOLT1A	1	202438719	2	3.222607
rs11793	PLEKHA6	1	202457035	4	0.275621
rs4489623	PLEKHA6	1	202457199	2	3.269319
rs4489623	PLEKHA6	1	202457199	4	6.200835
rs17334387	PIK3C2B	1	202663453	4	0.520596
rs3765156	PIK3C2B	1	202691651	4	0.621942
rs4252745	MDM4	1	202785810	3	0.058164
rs4252745	MDM4	1	202785810	4	0.858717
rs4252745	MDM4	1	202785810	5	0.371729
rs4252745	MDM4	1	202785810	6	2.688641
rs4252745	MDM4	1	202785810	8	0.061685
rs4252745	MDM4	1	202785810	2	2.21878
rs3789046	LRRN2	1	202853428	5	0.09362
rs3789046	LRRN2	1	202853428	2	2.177913
rs3789046	LRRN2	1	202853428	1	0.874908
rs3789046	LRRN2	1	202853428	8	3.527289
rs3789046	LRRN2	1	202853428	4	0.781529
rs3789046	LRRN2	1	202853428	3	0.007412
rs11588857	LRRN2	1	202853670	2	3.863241
rs11588857	LRRN2	1	202853670	4	1.704398
rs3747632	LRRN2	1	202853863	1	0.757886
rs3747632	LRRN2	1	202853863	5	0.099356
rs3747632	LRRN2	1	202853863	3	0.107575
rs3789044	LRRN2	1	202855724	7	0.466457
rs17415240	NFASC	1	203212494	2	4.953708
rs2794866	NFASC	1	203255158	1	1.343381

rs2841633	NFASC	1	203255786	1	2.931038
rs2841633	NFASC	1	203255786	6	0.575437
rs2841633	NFASC	1	203255786	3	0.672736
rs1042831	CNTN2	1	203309873	6	1.05036
rs1042831	CNTN2	1	203309873	3	0.383328
rs1042831	CNTN2	1	203309873	5	0.393558
rs11240351	CNTN2	1	203310962	3	0.758454
rs11240351	CNTN2	1	203310962	6	0.117634
rs11240351	CNTN2	1	203310962	5	1.638807
rs7518906	CNTN2	1	203311489	4	0.594417
rs16855045	CNTN2	1	203313517	2	0.90669
rs16855045	CNTN2	1	203313517	5	0.383348
rs12048743	RIPK5	1	203381496	6	9.121918
rs12048743	RIPK5	1	203381496	3	0.376419
rs12048743	RIPK5	1	203381496	5	0.246729
rs10900452	RIPK5	1	203382051	3	0.063241
rs3851294	RIPK5	1	203397036	4	0.164898
rs1779411	TMCC2	1	203505153	1	0.837558
rs1668867	TMCC2	1	203506874	2	3.574349
rs1668867	TMCC2	1	203506874	1	1.302535
rs17728	TMCC2	1	203508282	4	0.559918
rs7550061	NUAK2	1	203538915	4	1.502983
rs7550061	NUAK2	1	203538915	7	4.807429
rs7550061	NUAK2	1	203538915	3	1.272123
rs1106202	NUAK2	1	203539606	4	1.707512
rs1106202	NUAK2	1	203539606	6	1.451121
rs2275868	NUAK2	1	203544404	5	0.450203
rs10900482	KLHDC8A	1	203572725	3	0.584052
rs10900482	KLHDC8A	1	203572725	7	3.036307
rs10900482	KLHDC8A	1	203572725	8	0.631092
rs10900482	KLHDC8A	1	203572725	4	1.129993
rs10900482	KLHDC8A	1	203572725	2	4.375536
rs1105386	KLHDC8A	1	203574970	5	0.754218
rs1105387	KLHDC8A	1	203574991	5	0.408128
rs3795547	PCTK3	1	203767452	6	0.382351
rs3795547	PCTK3	1	203767452	2	5.151369
rs7526132	MFSD4	1	203820708	7	0.913158
rs7526132	MFSD4	1	203820708	2	1.023229
rs7526132	MFSD4	1	203820708	3	2.480749
rs7526132	MFSD4	1	203820708	4	1.161947
rs7526132	MFSD4	1	203820708	1	0.924703
rs7515820	MFSD4	1	203820718	7	0.103866
rs7515820	MFSD4	1	203820718	2	1.529468
rs7515820	MFSD4	1	203820718	1	1.517966
rs7515820	MFSD4	1	203820718	4	0.125179
rs7515820	MFSD4	1	203820718	3	3.410272
rs3088136	ELK4	1	203868087	1	5.009749

rs17347787	SLC45A3	1	203894931	4	3.475085
rs17347787	SLC45A3	1	203894931	1	3.701131
rs16856287	RAB7L1	1	204005823	6	0.501624
rs1361754	PM20D1	1	204068495	3	2.414551
rs4951025	PM20D1	1	204078451	5	2.699523
rs4951025	PM20D1	1	204078451	4	3.414171
SNP1-204081108	PM20D1	1	204081108	3	4.271163
SNP1-204081108	PM20D1	1	204081108	5	0.498894
SNP1-204081108	PM20D1	1	204081108	2	4.189858
rs1891460	PM20D1	1	204081120	3	3.428379
rs6669481	SLC26A9	1	204150897	1	0.514357
rs28391411	C1orf186	1	204406197	1	1.41243
rs28391411	C1orf186	1	204406197	3	0.990823
rs2297545	IKBKE	1	204717730	1	0.520466
rs2297545	IKBKE	1	204717730	5	0.315617
rs2297545	IKBKE	1	204717730	2	1.003275
rs2297545	IKBKE	1	204717730	3	0.998964
rs3748022	IKBKE	1	204736088	3	8.737995
rs3748022	IKBKE	1	204736088	5	0.157351
rs10836	IKBKE	1	204736792	1	0.385724
rs10836	IKBKE	1	204736792	5	0.226066
rs10836	IKBKE	1	204736792	7	4.683222
rs10836	IKBKE	1	204736792	3	0.232795
rs7541244	RASSF5	1	204827097	3	0.024008
rs7541445	RASSF5	1	204827309	6	0.402795
rs7541445	RASSF5	1	204827309	7	0.059745
rs7541445	RASSF5	1	204827309	1	0.643442
rs7541445	RASSF5	1	204827309	2	2.230795
rs7541445	RASSF5	1	204827309	4	5.755839
rs34526867	MAPKAPK2	1	204971663	3	0.178931
rs3024496	IL10	1	205008487	4	4.14478
rs2243158	IL19	1	205074264	3	0.041006
rs2243191	IL19	1	205082580	4	0.801278
rs2243191	IL19	1	205082580	3	0.997594
rs2243191	IL19	1	205082580	7	1.347808
rs2243193	IL19	1	205082848	4	0.394568
rs1150258	IL24	1	205141528	3	0.982087
rs1150258	IL24	1	205141528	2	2.723635
rs13190	PIGR	1	205168916	3	0.247293
rs13190	PIGR	1	205168916	2	3.255713
rs13190	PIGR	1	205168916	7	0.109302
rs6667268	PFKFB2	1	205312627	2	1.802475
rs6667268	PFKFB2	1	205312627	8	1.049991
rs6667268	PFKFB2	1	205312627	7	0.448594
rs6667268	PFKFB2	1	205312627	3	0.558954
rs6667268	PFKFB2	1	205312627	1	0.686472
rs8942	C4BPB	1	205336542	8	1.672068

rs8942	C4BPB	1	205336542	5	0.99133
rs8942	C4BPB	1	205336542	4	0.222821
rs1126618	C4BPA	1	205364303	5	3.492415
SNP1-20536669	C4BPA	1	205366693	2	4.422845
SNP1-20570857	CR2	1	205708573	1	4.73944
rs1048971	CR2	1	205712945	7	0.916055
rs1048971	CR2	1	205712945	3	0.020317
rs1048971	CR2	1	205712945	1	0.368571
rs1048971	CR2	1	205712945	4	0.596136
rs1048971	CR2	1	205712945	6	1.708451
rs1048971	CR2	1	205712945	2	2.581182
rs17615	CR2	1	205713085	4	3.485538
rs17615	CR2	1	205713085	1	2.102092
rs17616	CR2	1	205713546	4	1.666244
rs17616	CR2	1	205713546	1	2.971358
rs17258982	CR2	1	205719987	7	0.799919
rs17258982	CR2	1	205719987	2	3.946852
rs17258982	CR2	1	205719987	1	0.967604
rs761277	LOC148696	1	206059634	5	0.33082
rs761277	LOC148696	1	206059634	3	0.160245
rs761277	LOC148696	1	206059634	7	2.153431
rs7572	CD34	1	206126632	2	0.146684
rs7572	CD34	1	206126632	8	1.148615
rs1664227	PLXNA2	1	206456709	6	9.543665
rs1664227	PLXNA2	1	206456709	7	5.596009
rs1664227	PLXNA2	1	206456709	1	0.47567
rs1664227	PLXNA2	1	206456709	2	1.098374
rs4844658	PLXNA2	1	206456785	5	0.730158
rs4844658	PLXNA2	1	206456785	6	6.787664
SNP1-20645772	PLXNA2	1	206457721	4	0.479717
rs2800919	LOC391158	1	207472376	8	3.790069
rs2800919	LOC391158	1	207472376	5	4.286253
rs2800919	LOC391158	1	207472376	2	5.268346
rs11119315	CAMK1G	1	207851829	1	3.554973
rs11119315	CAMK1G	1	207851829	5	1.164214
rs2206107	CAMK1G	1	207853506	5	0.981412
rs2206107	CAMK1G	1	207853506	1	5.404893
rs2206107	CAMK1G	1	207853506	3	1.478353
rs2206107	CAMK1G	1	207853506	6	0.500295
rs2206107	CAMK1G	1	207853506	7	1.116304
rs2206107	CAMK1G	1	207853506	4	1.559695
GA019902	G0S2	1	207915625	1	4.782899
GA019902	G0S2	1	207915625	5	1.601459
GA019902	G0S2	1	207915625	8	5.145811
rs2272867	TRAF3IP3	1	207996202	6	0.912766
rs2272867	TRAF3IP3	1	207996202	5	0.133844
rs2272868	TRAF3IP3	1	207996226	4	0.419613

rs17015169	TRAF3IP3	1	208003537	8	0.712974
rs669694	TRAF3IP3	1	208017383	1	0.271918
rs669694	TRAF3IP3	1	208017383	6	0.331541
rs861019	IRF6	1	208042009	2	3.061123
rs861019	IRF6	1	208042009	7	0.641752
rs7539638	C1orf107	1	208092374	6	0.738736
rs7539638	C1orf107	1	208092374	8	3.818365
rs6689839	C1orf107	1	208093649	2	0.704508
rs6689839	C1orf107	1	208093649	1	0.691597
rs6689839	C1orf107	1	208093649	8	0.506534
rs1319072	C1orf107	1	208094277	8	0.061816
rs17188183	SYT14	1	208400704	5	0.211853
rs17188183	SYT14	1	208400704	1	0.544787
rs4609425	SYT14	1	208401689	3	0.534326
rs4609425	SYT14	1	208401689	1	0.887842
rs2066721	HHAT	1	208644496	7	0.72092
rs2066721	HHAT	1	208644496	4	1.258911
rs2066721	HHAT	1	208644496	5	0.020291
rs7522025	HHAT	1	208915642	2	0.130168
rs3135473	KCNH1	1	208923208	5	0.298225
rs3135473	KCNH1	1	208923208	7	0.15656
rs3135473	KCNH1	1	208923208	8	0.510282
rs1135317	KCNH1	1	208924080	8	0.673514
rs1135317	KCNH1	1	208924080	5	0.466696
rs1135317	KCNH1	1	208924080	2	4.33579
rs1533219	RCOR3	1	209554699	6	0.421611
rs1046690	TRAF5	1	209613124	3	0.395163
rs1046690	TRAF5	1	209613124	8	1.889179
rs11119746	RD3	1	209717658	7	1.02625
rs11119748	RD3	1	209718808	1	0.437245
rs41275374	INTS7	1	210186668	6	8.869601
rs3767861	C1orf75	1	210604176	4	0.026425
rs3767861	C1orf75	1	210604176	1	5.295175
rs3767861	C1orf75	1	210604176	5	0.088683
rs3767862	C1orf75	1	210604325	1	6.031349
rs3767862	C1orf75	1	210604325	5	1.970386
rs3767862	C1orf75	1	210604325	4	1.643442
rs518793	C1orf75	1	210654829	5	1.083071
rs1126526	ATF3	1	210860128	4	6.045181
rs1126526	ATF3	1	210860128	3	0.058845
rs3122712	FAM71A	1	210865600	3	0.930758
rs3122713	FAM71A	1	210866493	6	0.519408
rs3795842	FAM71A	1	210866572	3	0.186331
GA019916	NSL1	1	211031718	7	1.273091
rs1133462	TATDN3	1	211056514	8	4.485757
rs1133462	TATDN3	1	211056514	5	0.088622
rs1133462	TATDN3	1	211056514	1	0.40004

SNP1-211253154	ANGEL2	1	211253154	6	0.618555
rs2070065	CENPF	1	212877867	4	0.664605
rs3795522	CENPF	1	212880748	4	5.539229
rs3795520	CENPF	1	212881205	4	6.856934
SNP1-212884838	CENPF	1	212884838	4	6.971462
SNP1-212886603	CENPF	1	212886603	4	0.896816
rs869378	LOC391162	1	213111577	1	0.73312
rs6657250	USH2A	1	214286404	7	0.622983
rs10779261	USH2A	1	214661929	1	2.111712
rs1048126	GPATCH2	1	215670470	6	0.136412
rs1048126	GPATCH2	1	215670470	7	0.304421
rs265140	GPATCH2	1	215670507	1	5.552384
rs6604561	SPATA17	1	216014361	4	5.561898
rs6604561	SPATA17	1	216014361	1	7.060128
rs11466412	TGFB2	1	216681366	6	0.032157
rs900	TGFB2	1	216681528	7	5.088206
rs17517067	LOC643717	1	217155201	7	5.957699
rs3762307	LOC728510	1	217675199	3	0.805299
rs3762307	LOC728510	1	217675199	4	0.323543
SNP1-218223327	EPRS	1	218223327	5	9.147069
rs12136801	BPNT1	1	218329682	5	0.874349
rs12045447	RAB3GAP2	1	218411076	8	1.28912
rs12045447	RAB3GAP2	1	218411076	7	0.465154
rs7540014	LOC643779	1	218557120	6	2.309227
rs7540014	LOC643779	1	218557120	7	4.594058
rs7540014	LOC643779	1	218557120	1	3.623683
rs35322308	MARK1	1	218871074	4	0.535661
rs2738755	HLX	1	219124269	4	0.462875
rs2738755	HLX	1	219124269	3	1.350846
SNP1-220780244	HHIPL2	1	220780244	7	1.848531
SNP1-220780244	HHIPL2	1	220780244	6	3.815356
rs3008651	TAF1A	1	220829858	2	2.429048
rs3008651	TAF1A	1	220829858	6	0.986536
rs3008651	TAF1A	1	220829858	7	0.141884
SNP1-220869685	MIA3	1	220869685	7	0.125583
rs3811465	C1orf58	1	220952742	7	3.495538
SNP1-220986518	FLJ43505	1	220986518	7	0.179458
rs6683071	FLJ43505	1	220989974	7	0.696146
rs2609383	DISP1	1	221183095	6	5.691516
rs2609383	DISP1	1	221183095	2	5.322134
SNP1-221245101	DISP1	1	221245101	3	0.165784
SNP1-221245101	DISP1	1	221245101	8	1.049905
SNP1-221245101	DISP1	1	221245101	2	3.169456
rs6679661	C1orf65	1	221633890	4	0.493792
rs6682552	C1orf65	1	221634431	4	0.729181
rs17739	TP53BP2	1	222034576	4	3.976136
rs6604731	LOC644220	1	222118062	5	1.768382

rs6604731	LOC644220	1	222118062	7	1.073073
rs6604731	LOC644220	1	222118062	3	1.31844
rs11585297	CNIH3	1	222994591	1	4.256275
rs11585297	CNIH3	1	222994591	7	1.823873
rs2501107	DNAH14	1	223297231	6	0.904321
rs2501107	DNAH14	1	223297231	7	0.521857
rs3128651	DNAH14	1	223334966	7	0.001906
rs3128651	DNAH14	1	223334966	6	0.454649
rs10495237	DNAH14	1	223572917	7	0.364401
rs10495237	DNAH14	1	223572917	5	0.548361
rs7527925	DNAH14	1	223600842	4	0.391337
rs7527925	DNAH14	1	223600842	7	0.694144
rs7527925	DNAH14	1	223600842	1	1.240398
rs7527925	DNAH14	1	223600842	8	0.680949
rs3856154	DNAH14	1	223631638	5	0.40819
rs3856154	DNAH14	1	223631638	1	4.729195
rs3856154	DNAH14	1	223631638	7	0.410324
rs950210	DNAH14	1	223635864	7	0.265161
rs950210	DNAH14	1	223635864	1	3.761874
rs950210	DNAH14	1	223635864	8	0.420661
rs3738040	EPHX1	1	224079664	4	0.041375
rs3738040	EPHX1	1	224079664	8	1.481249
rs3738040	EPHX1	1	224079664	6	4.286782
SNP1-22410153	TMEM63A	1	224101536	6	1.889194
SNP1-22410153	TMEM63A	1	224101536	4	5.493926
SNP1-22410153	TMEM63A	1	224101536	5	1.653436
SNP1-22410153	TMEM63A	1	224101536	3	0.367295
SNP1-22410153	TMEM63A	1	224101536	1	0.681734
GA019956	PYCR2	1	224178568	8	1.3579
GA019956	PYCR2	1	224178568	2	1.160582
rs6664468	C1orf55	1	224238098	8	7.979565
rs6664468	C1orf55	1	224238098	7	0.402305
rs6664468	C1orf55	1	224238098	1	0.115044
rs6664468	C1orf55	1	224238098	4	0.430315
rs2306120	ACBD3	1	224419121	1	4.61289
rs1136410	PARP1	1	224621925	3	0.721336
rs1136410	PARP1	1	224621925	8	0.372028
rs1136410	PARP1	1	224621925	6	0.51988
rs1805414	PARP1	1	224639987	6	1.038264
rs1805414	PARP1	1	224639987	1	0.21764
rs1805414	PARP1	1	224639987	2	0.842062
rs1805414	PARP1	1	224639987	7	0.648872
rs1805414	PARP1	1	224639987	3	0.971189
rs1144839	ITPKB	1	224886875	6	0.358299
rs1144839	ITPKB	1	224886875	5	0.143388
rs6667260	ITPKB	1	224990561	5	1.254297
rs6667260	ITPKB	1	224990561	7	4.775584

rs6667260	ITPKB	1	224990561	8	0.025774
rs6667260	ITPKB	1	224990561	1	2.55809
rs1295645	PSEN2	1	225125660	4	2.297801
rs1295645	PSEN2	1	225125660	5	1.503021
rs1295645	PSEN2	1	225125660	1	5.10452
rs3177404	PSEN2	1	225129852	7	0.337353
rs6759	PSEN2	1	225136360	2	3.287735
rs6759	PSEN2	1	225136360	8	0.249999
rs6759	PSEN2	1	225136360	5	0.149304
rs1045247	CDC42BPA	1	225249198	2	3.550367
rs1045247	CDC42BPA	1	225249198	1	0.48659
rs3088389	JMJD4	1	225985813	5	0.454855
rs3088389	JMJD4	1	225985813	1	0.181587
rs3088389	JMJD4	1	225985813	2	8.567371
rs4837	JMJD4	1	225986980	7	0.385056
SNP1-22602130(	C1orf142	1	226021300	7	0.634652
rs8192633	WNT9A	1	226176094	5	0.151972
rs752107	WNT3A	1	226313974	7	2.895308
rs752107	WNT3A	1	226313974	1	2.973849
rs752107	WNT3A	1	226313974	8	2.312636
rs752107	WNT3A	1	226313974	5	3.388105
rs752107	WNT3A	1	226313974	4	3.895936
rs752107	WNT3A	1	226313974	3	2.784352
SNP1-22642930!	C1orf69	1	226429305	3	0.402673
SNP1-22642930!	C1orf69	1	226429305	8	0.590566
SNP1-22642930!	C1orf69	1	226429305	5	0.26627
SNP1-22642930!	C1orf69	1	226429305	7	0.637244
SNP1-22642930!	C1orf69	1	226429305	4	0.556504
GA001737	C1orf69	1	226430373	5	0.623731
rs4653915	C1orf69	1	226431228	8	1.137303
rs4653915	C1orf69	1	226431228	7	1.568452
rs4653915	C1orf69	1	226431228	6	0.732318
rs4653915	C1orf69	1	226431228	5	1.43139
rs4653915	C1orf69	1	226431228	3	1.514943
rs7532924	C1orf145	1	226460552	3	0.552227
rs7532924	C1orf145	1	226460552	4	1.259611
rs7532924	C1orf145	1	226460552	7	0.121974
rs7532924	C1orf145	1	226460552	5	0.095508
rs1620111	OBSCN	1	226468670	6	0.762041
rs1620111	OBSCN	1	226468670	5	0.714268
rs1620111	OBSCN	1	226468670	8	0.11165
rs1620111	OBSCN	1	226468670	7	1.213945
rs1620111	OBSCN	1	226468670	3	1.106381
rs1771487	OBSCN	1	226468744	3	0.663116
rs1771487	OBSCN	1	226468744	8	0.839128
rs1771487	OBSCN	1	226468744	7	0.481393
rs1771487	OBSCN	1	226468744	5	0.640524

rs1771487	OBSCN	1	226468744	4	1.877529
rs1771487	OBSCN	1	226468744	6	1.002584
rs1757152	OBSCN	1	226478931	7	0.160373
rs1757152	OBSCN	1	226478931	4	0.161396
rs1757152	OBSCN	1	226478931	6	0.089542
rs1757152	OBSCN	1	226478931	3	0.334797
rs1757152	OBSCN	1	226478931	5	0.51193
rs1771455	OBSCN	1	226497718	7	0.22687
rs1771455	OBSCN	1	226497718	5	0.082762
rs7517088	OBSCN	1	226501018	5	2.146099
rs1188721	OBSCN	1	226530871	5	0.15193
rs1188722	OBSCN	1	226530899	5	0.512882
rs1188722	OBSCN	1	226530899	7	0.273194
SNP1-22653092[illegible]	OBSCN	1	226530926	1	3.773105
rs4653939	OBSCN	1	226561319	5	0.112151
rs4653939	OBSCN	1	226561319	6	0.37899
rs4653939	OBSCN	1	226561319	3	0.192866
rs4653939	OBSCN	1	226561319	4	0.454156
rs435776	OBSCN	1	226561413	6	1.018084
rs435776	OBSCN	1	226561413	7	1.25305
rs435776	OBSCN	1	226561413	3	0.99926
rs435776	OBSCN	1	226561413	4	1.106213
rs1150912	OBSCN	1	226570300	5	0.697968
rs1150912	OBSCN	1	226570300	7	0.365916
rs4653942	OBSCN	1	226571827	3	0.513207
rs1188729	OBSCN	1	226572291	5	0.015179
rs1188729	OBSCN	1	226572291	7	0.117357
rs373610	OBSCN	1	226576050	5	0.766388
rs373610	OBSCN	1	226576050	7	0.189403
rs1188710	OBSCN	1	226595186	7	0.802389
rs1188710	OBSCN	1	226595186	6	0.385093
rs1188710	OBSCN	1	226595186	3	0.329572
rs1188710	OBSCN	1	226595186	4	0.128844
rs12035900	OBSCN	1	226614524	5	0.451903
rs12035900	OBSCN	1	226614524	7	0.597021
SNP1-22661482[illegible]	OBSCN	1	226614820	4	2.703115
SNP1-22661482[illegible]	OBSCN	1	226614820	3	0.284763
rs35155240	NULL	1	226617049	3	0.407169
SNP1-22662551[illegible]	OBSCN	1	226625515	3	0.61636
rs500049	NULL	1	226626617	5	0.837883
rs500049	NULL	1	226626617	1	0.508878
rs500049	NULL	1	226626617	4	0.712977
SNP1-22663150[illegible]	OBSCN	1	226631507	6	0.147685
SNP1-22663150[illegible]	OBSCN	1	226631507	4	0.091704
SNP1-22663150[illegible]	OBSCN	1	226631507	3	0.272056
rs503203	TRIM17	1	226669283	3	0.224877
rs503203	TRIM17	1	226669283	6	2.330163

rs503203	TRIM17	1	226669283	4	1.250457
rs12542	RNF187	1	226748953	3	0.025032
rs12542	RNF187	1	226748953	4	1.028256
rs12542	RNF187	1	226748953	6	4.952017
rs11547452	RNF187	1	226749158	6	0.522559
rs11547452	RNF187	1	226749158	3	0.655478
rs13349	RHOU	1	226947752	8	4.437115
rs13349	RHOU	1	226947752	4	3.867425
rs15452	RAB4A	1	227506262	4	0.217254
rs15452	RAB4A	1	227506262	5	0.106182
rs15452	RAB4A	1	227506262	6	1.17083
rs15452	RAB4A	1	227506262	3	0.111289
rs2282081	C1orf96	1	227526869	3	0.140095
rs2282081	C1orf96	1	227526869	6	3.832037
rs3738187	ABCB10	1	227749887	2	3.21949
rs3738187	ABCB10	1	227749887	4	2.513035
rs3738187	ABCB10	1	227749887	7	0.896272
rs3738187	ABCB10	1	227749887	6	0.456629
rs3213494	GALNT2	1	228482547	8	5.130314
rs16851328	GALNT2	1	228482753	7	0.024898
rs16851328	GALNT2	1	228482753	2	3.556279
rs7022	GALNT2	1	228484287	4	0.474228
rs7022	GALNT2	1	228484287	2	0.483583
rs7022	GALNT2	1	228484287	1	0.441549
rs7022	GALNT2	1	228484287	7	6.791845
rs12089782	PGBD5	1	228524947	1	3.116804
rs7079	AGT	1	228904954	5	0.40371
rs7079	AGT	1	228904954	2	2.445037
rs7079	AGT	1	228904954	7	0.811758
rs7079	AGT	1	228904954	4	0.426553
rs4762	AGT	1	228912600	5	0.272038
rs4762	AGT	1	228912600	1	4.201308
rs4762	AGT	1	228912600	4	0.022656
rs7517389	CAPN9	1	228976962	6	2.342012
rs7517389	CAPN9	1	228976962	7	2.404179
rs1933631	CAPN9	1	228981352	6	2.13845
rs1933631	CAPN9	1	228981352	1	5.784144
rs3811502	TTC13	1	229109355	2	7.549852
rs3811502	TTC13	1	229109355	1	0.167779
rs3811502	TTC13	1	229109355	3	0.089529
rs3811502	TTC13	1	229109355	7	0.890422
rs3811502	TTC13	1	229109355	4	0.468157
rs4658912	LOC644006	1	229225706	4	4.757022
rs4658912	LOC644006	1	229225706	6	0.395873
rs4658912	LOC644006	1	229225706	5	4.951311
rs11586657	TRIM67	1	229422535	1	3.514373
rs11586657	TRIM67	1	229422535	6	1.314443

rs11586657	TRIM67	1	229422535	5	1.439102
rs2274067	C1orf131	1	229443429	5	0.110485
rs2274067	C1orf131	1	229443429	3	0.53549
rs2274067	C1orf131	1	229443429	7	0.276479
rs574553	GNPAT	1	229468525	6	0.168085
rs574553	GNPAT	1	229468525	4	2.028329
rs7544596	EGLN1	1	229625963	7	0.348868
rs1630250	LOC440731	1	229730826	3	0.304838
rs1630250	LOC440731	1	229730826	8	5.549655
rs1630250	LOC440731	1	229730826	5	0.177804
rs3738401	DISC1	1	229896918	7	0.285468
rs3738401	DISC1	1	229896918	3	1.642959
rs11122324	DISC1	1	229925804	3	0.513212
rs11122324	DISC1	1	229925804	1	0.452301
rs12133766	DISC1	1	230020768	8	0.269412
rs3082	DISC1	1	230069015	3	0.252855
rs9729179	DISC1	1	230240796	3	1.92421
rs980989	DISC1	1	230242818	3	0.327016
rs980989	DISC1	1	230242818	4	0.13637
rs9308481	DISC1	1	230242929	4	3.445688
rs9308481	DISC1	1	230242929	7	0.948454
rs9308481	DISC1	1	230242929	3	0.067959
rs11803088	DISC1	1	230243392	8	0.552111
rs11803088	DISC1	1	230243392	1	0.005015
rs11803088	DISC1	1	230243392	4	0.283315
rs16856322	DISC1	1	230243610	3	0.616573
rs16856322	DISC1	1	230243610	4	1.645055
rs12757779	SIPA1L2	1	230648059	2	0.527293
rs12757779	SIPA1L2	1	230648059	4	0.738347
rs6424243	LOC729336	1	230834771	1	3.386835
rs2814526	KIAA1383	1	231008013	8	4.060109
rs12725558	C1orf57	1	231158086	6	0.586604
rs12725558	C1orf57	1	231158086	7	0.96912
rs12725558	C1orf57	1	231158086	5	0.559772
rs12725558	C1orf57	1	231158086	3	0.608017
SNP1-23120070	PCNXL2	1	231200709	4	5.191113
SNP1-23120070	PCNXL2	1	231200709	3	0.027444
rs3795375	KIAA1804	1	231581557	8	5.044077
rs3795375	KIAA1804	1	231581557	6	5.112105
rs963982	KIAA1804	1	231581694	6	0.628134
SNP1-23158172	KIAA1804	1	231581725	8	4.184236
rs17227790	LOC728987	1	231832150	7	0.745709
rs2991957	LOC440733	1	232559638	4	0.748612
rs2991957	LOC440733	1	232559638	1	0.824734
rs2991957	LOC440733	1	232559638	2	1.342534
rs270502	TARBP1	1	232670009	1	3.748134
rs2789367	GGPS1	1	233573541	7	0.044529

rs2789367	GGPS1	1	233573541	1	1.133163
rs2789367	GGPS1	1	233573541	2	0.193044
rs2789367	GGPS1	1	233573541	3	1.129317
rs12756717	GNG4	1	233780785	3	1.600832
rs12756717	GNG4	1	233780785	1	3.967175
rs12756717	GNG4	1	233780785	7	0.488274
rs14326	GNG4	1	233782146	6	0.214894
rs14326	GNG4	1	233782146	7	2.659214
rs14326	GNG4	1	233782146	3	0.493949
rs14326	GNG4	1	233782146	1	4.740964
rs3768066	LYST	1	234038386	3	0.040745
rs3768066	LYST	1	234038386	8	2.046403
rs873	NID1	1	234207362	3	1.65204
rs873	NID1	1	234207362	2	5.327828
rs35714220	NID1	1	234243468	5	2.40608
rs7519217	LOC343508	1	234341428	4	3.401622
rs7519217	LOC343508	1	234341428	6	2.567258
rs7519217	LOC343508	1	234341428	1	4.591986
rs1726630	ERO1LB	1	234447972	2	2.987978
rs1726630	ERO1LB	1	234447972	8	1.382298
rs1726630	ERO1LB	1	234447972	7	0.64569
rs1726630	ERO1LB	1	234447972	1	0.465161
rs1726630	ERO1LB	1	234447972	5	0.714204
rs1726630	ERO1LB	1	234447972	3	0.384499
SNP1-23447985	ERO1LB	1	234479853	3	0.382456
SNP1-23447985	ERO1LB	1	234479853	7	0.600759
SNP1-23447985	ERO1LB	1	234479853	5	3.092649
SNP1-23447985	ERO1LB	1	234479853	1	1.16238
SNP1-23447985	ERO1LB	1	234479853	6	3.857603
SNP1-23447985	ERO1LB	1	234479853	2	1.390116
rs966365	EDARADD	1	234624394	4	0.10001
rs604070	EDARADD	1	234712293	1	0.533459
rs604070	EDARADD	1	234712293	4	0.368409
rs604070	EDARADD	1	234712293	2	0.466775
rs604070	EDARADD	1	234712293	7	6.259381
rs6428955	EDARADD	1	234714359	5	1.408752
rs6428955	EDARADD	1	234714359	2	2.356461
rs6428955	EDARADD	1	234714359	8	4.157726
rs6428955	EDARADD	1	234714359	4	4.236743
rs6428955	EDARADD	1	234714359	1	2.814318
rs2737713	LGALS8	1	234767430	6	0.394159
rs2737713	LGALS8	1	234767430	7	1.024894
rs1041942	LGALS8	1	234778574	6	1.898798
rs1041942	LGALS8	1	234778574	7	7.801859
rs1041942	LGALS8	1	234778574	8	4.401249
rs2275687	HEATR1	1	234784549	8	5.076581
rs2275687	HEATR1	1	234784549	6	0.002643

rs2275687	HEATR1	1	234784549	7	4.356494
rs1126627	HEATR1	1	234785243	8	1.17909
rs653737	HEATR1	1	234796579	8	2.922439
rs653737	HEATR1	1	234796579	7	1.129189
rs653737	HEATR1	1	234796579	1	3.927038
rs653737	HEATR1	1	234796579	6	4.627459
rs2564739	HEATR1	1	234822460	6	3.886223
rs10737812	MTR	1	235125451	6	0.139001
rs10737812	MTR	1	235125451	2	3.239643
rs10737812	MTR	1	235125451	4	0.052104
rs1804742	MTR	1	235129251	2	3.404044
rs1804742	MTR	1	235129251	6	2.406698
rs1804742	MTR	1	235129251	1	0.169613
SNP1-23513103	MTR	1	235131031	8	0.172504
rs34967813	RYR2	1	235908013	1	0.323
rs1054888	LOC339535	1	236710723	7	1.341147
rs1054888	LOC339535	1	236710723	5	1.250856
rs1054888	LOC339535	1	236710723	8	4.827388
rs882869	FMN2	1	238704194	1	1.976035
rs2185289	LOC645916	1	238866705	7	4.772001
rs2185288	LOC645916	1	238866739	1	2.461507
rs2185288	LOC645916	1	238866739	7	1.947292
rs1053230	KMO	1	239821971	3	1.055933
rs3193688	KMO	1	239825189	6	0.233436
rs3193688	KMO	1	239825189	1	6.157715
rs1053183	KMO	1	239825254	1	5.102983
rs1053183	KMO	1	239825254	6	7.127227
rs676750	CHML	1	239862907	3	0.189419
rs676750	CHML	1	239862907	5	0.398599
rs676750	CHML	1	239862907	2	4.704665
rs10802990	WDR64	1	239974440	3	0.499631
rs10802990	WDR64	1	239974440	5	0.075177
rs1635517	EXO1	1	240078656	3	1.626907
rs1635517	EXO1	1	240078656	7	1.900539
rs735943	EXO1	1	240096774	3	0.248554
rs4149963	EXO1	1	240102005	1	5.169954
rs3851304	LOC441925	1	240188419	5	0.494371
rs34091549	LOC391183	1	241002620	3	2.266265
rs976529	SDCCAG8	1	241538085	8	0.104937
rs2994330	LOC729199	1	241713831	2	1.472763
rs2994330	LOC729199	1	241713831	8	0.089413
rs2994330	LOC729199	1	241713831	1	1.115233
rs3006922	LOC729199	1	241713977	2	1.721571
rs2994329	LOC729199	1	241714544	8	0.834705
rs11586356	C1orf101	1	242707839	2	5.208728
rs7544794	C1orf101	1	242790715	4	0.855024
rs10927267	C1orf101	1	242802481	4	0.000846

rs11579106	FAM36A	1	243074738	3	0.099224
rs10927387	EFCAB2	1	243312025	1	4.990622
rs10927387	EFCAB2	1	243312025	3	0.543484
rs10927387	EFCAB2	1	243312025	5	5.202821
rs1047707	EFCAB2	1	243317643	1	4.128197
rs1047707	EFCAB2	1	243317643	5	0.535621
rs1047707	EFCAB2	1	243317643	3	0.92507
rs4379666	LOC729761	1	243384058	7	0.107033
rs871840	KIF26B	1	243916718	3	0.041871
rs3737918	KIF26B	1	243933134	6	0.970588
rs8025	SMYD3	1	243979463	1	2.97634
rs8025	SMYD3	1	243979463	8	0.325468
rs7527781	SMYD3	1	244647306	8	0.499446
rs7527781	SMYD3	1	244647306	6	0.659348
rs7527781	SMYD3	1	244647306	1	4.36076
rs7527781	SMYD3	1	244647306	5	0.184838
rs7527781	SMYD3	1	244647306	7	6.148957
rs6693807	LOC343165	1	245460278	1	0.766129
rs6693807	LOC343165	1	245460278	7	0.072532
rs10754558	NLRP3	1	245678659	8	0.587319
rs10754558	NLRP3	1	245678659	1	1.913273
rs4925663	OR2B11	1	245681240	1	2.398256
rs1151687	OR2G2	1	245818642	4	0.195961
rs1151687	OR2G2	1	245818642	3	2.096185
rs1151687	OR2G2	1	245818642	7	1.485549
rs1151687	OR2G2	1	245818642	5	1.867031
rs28446289	OR13G1	1	245902297	3	0.088886
rs28446289	OR13G1	1	245902297	4	2.214521
rs28446289	OR13G1	1	245902297	5	0.588102
rs28446289	OR13G1	1	245902297	1	0.40309
rs1151640	OR13G1	1	245902573	1	2.583707
rs1151640	OR13G1	1	245902573	3	1.05218
rs1151640	OR13G1	1	245902573	7	1.245429
rs2282316	OR6F1	1	245942038	7	0.25039
rs1552812	OR1C1	1	245987723	7	0.518774
rs1552812	OR1C1	1	245987723	4	2.515903
rs4925724	LOC646510	1	246058232	3	1.42017
rs4925724	LOC646510	1	246058232	5	0.102617
rs3737277	TRIM58	1	246090589	7	2.788467
rs946696	TRIM58	1	246094842	5	0.600807
rs946696	TRIM58	1	246094842	7	0.954206
SNP1-24610591	TRIM58	1	246105917	7	0.9982
rs4925753	TRIM58	1	246106727	7	1.74898
rs4925753	TRIM58	1	246106727	5	1.381574
rs10888267	OR2W3	1	246126046	8	0.596508
rs10888267	OR2W3	1	246126046	5	0.326807
rs12135078	OR2W3	1	246126079	3	1.597369

rs12139390	OR2W3	1	246126099	3	0.162066
rs12139390	OR2W3	1	246126099	7	0.088959
rs11204546	OR2W3	1	246126335	3	0.287296
rs11204546	OR2W3	1	246126335	5	0.853814
rs11204546	OR2W3	1	246126335	4	2.248841
rs6664332	OR2AK2	1	246195552	4	1.210346
rs6664332	OR2AK2	1	246195552	8	1.167462
rs6664332	OR2AK2	1	246195552	5	2.665945
rs954474	OR2T6	1	246618183	5	1.344354
rs954475	OR2T6	1	246618259	5	0.817971
rs954475	OR2T6	1	246618259	3	0.933668
rs4509608	OR5BU1	1	246912081	2	1.662527
rs4509608	OR5BU1	1	246912081	1	1.779412
rs11555324	ZNF672	1	247108077	4	3.40162
SNP2-267003	ACP1	2	267003	4	0.306762
SNP2-267003	ACP1	2	267003	3	0.251579
SNP2-267003	ACP1	2	267003	1	3.780682
SNP2-267003	ACP1	2	267003	7	0.452863
rs4927635	NULL	2	1299150	1	0.935075
rs17415659	TPO	2	1476810	7	2.135822
rs17415659	TPO	2	1476810	8	2.210631
rs17415659	TPO	2	1476810	5	1.441145
rs732608	TPO	2	1478906	4	0.04658
rs732608	TPO	2	1478906	2	0.436492
rs732608	TPO	2	1478906	7	2.749564
rs732608	TPO	2	1478906	5	0.425607
rs732608	TPO	2	1478906	6	0.613888
rs1042589	TPO	2	1525334	6	3.066066
rs1042589	TPO	2	1525334	3	0.643379
rs6739414	LOC648230	2	2633087	1	0.10974
rs6836	TSSC1	2	3171805	1	3.781731
rs6836	TSSC1	2	3171805	8	7.153139
rs10865541	TTC15	2	3371082	6	2.835092
rs10865541	TTC15	2	3371082	8	6.722916
SNP2-3371302	TTC15	2	3371302	4	5.503266
SNP2-3371302	TTC15	2	3371302	3	0.159369
SNP2-3371302	TTC15	2	3371302	2	0.208104
SNP2-3371302	TTC15	2	3371302	1	0.206723
SNP2-3371302	TTC15	2	3371302	6	0.683756
rs4971514	TTC15	2	3448470	1	4.671219
rs4971514	TTC15	2	3448470	4	0.691444
rs4971514	TTC15	2	3448470	6	0.814538
rs4971514	TTC15	2	3448470	3	0.657531
rs11127430	LOC388923	2	3464249	1	4.528462
rs11127430	LOC388923	2	3464249	3	0.860161
rs11127430	LOC388923	2	3464249	6	0.188904
rs11127430	LOC388923	2	3464249	4	8.918442

rs1129241	LOC643517	2	3557460	1	1.517602
rs1129241	LOC643517	2	3557460	4	1.31621
rs10186193	RNASEH1	2	3575849	1	1.337928
rs10186193	RNASEH1	2	3575849	3	0.653476
rs7567833	COLEC11	2	3669423	8	0.548035
rs28381982	ALLC	2	3727031	8	1.681455
rs28381982	ALLC	2	3727031	4	1.562101
rs4371338	SOX11	2	5755481	1	0.964255
rs4371338	SOX11	2	5755481	7	1.289557
rs4371338	SOX11	2	5755481	8	2.083229
rs4371338	SOX11	2	5755481	4	2.384256
rs13419910	SOX11	2	5757137	4	1.247365
rs13419910	SOX11	2	5757137	7	0.630297
rs13419910	SOX11	2	5757137	8	0.399845
rs6729137	SOX11	2	5757912	4	0.203083
rs2709437	FLJ41046	2	6040943	6	1.177686
rs1870744	FLJ42418	2	6789300	5	1.7192
rs4669114	RSAD2	2	6954072	5	0.782308
rs4669114	RSAD2	2	6954072	1	1.011137
rs7570384	RSAD2	2	6954129	7	0.968686
rs10495546	RSAD2	2	6954207	1	1.425314
rs6431837	RSAD2	2	6954262	7	0.853011
rs1045239	RSAD2	2	6954625	7	0.200146
rs1045239	RSAD2	2	6954625	4	1.900168
rs1045239	RSAD2	2	6954625	5	0.479771
rs1045239	RSAD2	2	6954625	1	3.225234
rs364891	RNF144A	2	7054518	2	1.855913
rs309304	RNF144A	2	7098937	2	2.114615
rs309304	RNF144A	2	7098937	1	0.326736
rs309304	RNF144A	2	7098937	6	0.156801
rs309304	RNF144A	2	7098937	5	0.559839
rs309303	RNF144A	2	7099011	7	2.29079
rs309303	RNF144A	2	7099011	5	2.528977
rs309303	RNF144A	2	7099011	2	5.189714
rs309303	RNF144A	2	7099011	6	1.740577
rs11888409	DDEF2	2	9438325	7	0.416089
rs2715860	DDEF2	2	9445987	5	0.253981
rs2715860	DDEF2	2	9445987	2	3.721238
rs2715860	DDEF2	2	9445987	7	0.788494
rs2715860	DDEF2	2	9445987	1	0.060885
rs6728908	DDEF2	2	9451225	7	1.157306
rs1048610	ADAM17	2	9552307	3	0.195274
rs1048610	ADAM17	2	9552307	8	6.460888
rs1048610	ADAM17	2	9552307	6	6.423098
rs12692386	ADAM17	2	9613357	2	1.106532
rs12692386	ADAM17	2	9613357	6	0.5347
rs12692386	ADAM17	2	9613357	3	1.839275

SNP2-9968411	TAF1B	2	9968411	3	0.749605
SNP2-9968411	TAF1B	2	9968411	7	0.108012
SNP2-9968411	TAF1B	2	9968411	8	5.433659
SNP2-9968411	TAF1B	2	9968411	6	0.189127
rs1469541	UNQ5830	2	10062799	1	3.135826
rs1469541	UNQ5830	2	10062799	5	0.457505
rs4444493	KLF11	2	10110629	1	5.357219
rs4444493	KLF11	2	10110629	4	0.009273
SNP2-10268050	C2orf48	2	10268050	6	0.30645
rs7577583	C2orf48	2	10269303	4	2.067317
rs7577583	C2orf48	2	10269303	7	0.497029
rs7577583	C2orf48	2	10269303	5	1.048586
rs7577583	C2orf48	2	10269303	2	1.142022
rs7577583	C2orf48	2	10269303	8	0.999317
rs13023336	HPCAL1	2	10361482	4	1.426609
rs13023336	HPCAL1	2	10361482	8	3.025214
rs3732114	NOL10	2	10629729	1	5.220159
rs3732114	NOL10	2	10629729	7	0.088341
rs1060011	PDIA6	2	10841117	6	7.589574
rs3732105	KCNF1	2	10971191	3	0.535852
rs3732105	KCNF1	2	10971191	8	0.983833
rs7560331	FLJ33534	2	11182281	6	1.764275
rs7560331	FLJ33534	2	11182281	5	0.37429
rs7560331	FLJ33534	2	11182281	7	1.689304
rs9808232	ROCK2	2	11276571	2	4.134025
rs9808232	ROCK2	2	11276571	3	1.439378
rs9808232	ROCK2	2	11276571	4	0.556553
SNP2-11624144	GREB1	2	11624144	7	4.554371
rs2304399	GREB1	2	11670086	7	0.309815
rs2304402	GREB1	2	11691775	3	1.207842
rs2304402	GREB1	2	11691775	1	3.292189
rs2304402	GREB1	2	11691775	5	0.940527
rs6710817	NAG	2	15290013	7	0.862293
rs7590340	NAG	2	15518892	1	2.360861
rs7590340	NAG	2	15518892	7	0.147808
rs10153729	DDX1	2	15649310	8	3.895324
rs10153729	DDX1	2	15649310	2	0.822756
rs10153729	DDX1	2	15649310	1	0.301962
rs10153729	DDX1	2	15649310	5	0.265082
rs10153729	DDX1	2	15649310	4	0.712102
rs10153729	DDX1	2	15649310	3	0.681792
rs1976809	LOC645521	2	17329493	5	4.111619
rs1976809	LOC645521	2	17329493	6	4.041765
rs1976809	LOC645521	2	17329493	7	3.971656
rs1976809	LOC645521	2	17329493	3	4.894069
rs1976809	LOC645521	2	17329493	8	3.511016
rs834514	RAD51AP2	2	17560054	3	0.419166

rs834514	RAD51AP2	2	17560054	1	4.355048
rs834514	RAD51AP2	2	17560054	4	1.757338
rs834514	RAD51AP2	2	17560054	7	0.159448
rs834514	RAD51AP2	2	17560054	6	2.881008
rs834514	RAD51AP2	2	17560054	8	1.814783
rs17314548	RAD51AP2	2	17560237	6	2.360679
rs2710673	SMC6	2	17708777	3	0.652582
rs2710673	SMC6	2	17708777	5	1.546393
rs6531053	LOC284952	2	17799261	6	0.199663
rs6531053	LOC284952	2	17799261	3	0.450357
rs6531053	LOC284952	2	17799261	4	0.07786
rs6531053	LOC284952	2	17799261	1	3.803036
rs1812152	GEN1	2	17806256	7	0.349631
rs1812152	GEN1	2	17806256	3	0.217115
SNP2-17826604	GEN1	2	17826604	3	0.309677
rs3747660	NT5C1B	2	18608239	7	0.002064
SNP2-18629637	NT5C1B	2	18629637	7	0.423733
rs2304589	TTC32	2	19964968	7	0.853512
rs2304588	TTC32	2	19965198	7	0.472747
rs1056233	WDR35	2	19976569	7	1.008373
rs1056233	WDR35	2	19976569	3	0.61195
rs1056233	WDR35	2	19976569	1	5.04082
rs1056233	WDR35	2	19976569	4	1.238949
rs6748924	WDR35	2	19976702	8	3.859946
rs1060742	WDR35	2	20052496	7	1.038908
rs28452699	MATN3	2	20066410	1	3.44097
rs28452699	MATN3	2	20066410	4	1.357833
rs28452699	MATN3	2	20066410	7	0.324678
rs1513830	LOC727885	2	20115495	6	1.136061
rs1513830	LOC727885	2	20115495	1	2.394495
rs1131351	SDC1	2	20265861	5	6.329029
rs1131351	SDC1	2	20265861	2	5.026159
rs1131351	SDC1	2	20265861	6	0.802852
rs1131351	SDC1	2	20265861	7	4.404397
rs2289084	hCG_1795065	2	20593078	1	5.363466
rs3821217	HS1BP3	2	20681767	6	1.170378
rs3821217	HS1BP3	2	20681767	7	5.522013
rs3821217	HS1BP3	2	20681767	1	3.960884
rs3732150	HS1BP3	2	20681998	5	0.192932
rs3732150	HS1BP3	2	20681998	1	2.500485
rs3732150	HS1BP3	2	20681998	8	0.107818
rs3732150	HS1BP3	2	20681998	3	0.966917
rs3732149	HS1BP3	2	20682245	7	0.072971
rs3732149	HS1BP3	2	20682245	1	3.820089
rs3796064	HS1BP3	2	20701799	4	0.214391
rs1367117	APOB	2	21117405	2	1.475562
rs2712098	KLHL29	2	23784218	3	0.095709

rs3731620	C2orf44	2	24115564	4	6.781458
rs1134516	PFN4	2	24196036	4	4.857953
rs2303291	ITSN2	2	24284688	2	3.374287
rs2303291	ITSN2	2	24284688	6	1.541342
rs2543662	ITSN2	2	24296775	6	0.619171
rs2543662	ITSN2	2	24296775	2	6.207271
rs1203	ITSN2	2	24296834	6	3.950721
rs1203	ITSN2	2	24296834	2	4.297788
rs7603997	ITSN2	2	24378462	2	5.436508
rs7603997	ITSN2	2	24378462	6	1.609758
rs11125744	NCOA1	2	24759431	4	9.631383
rs17737058	NCOA1	2	24845073	8	6.636018
rs17737058	NCOA1	2	24845073	3	0.212962
rs17737058	NCOA1	2	24845073	1	6.403925
rs1044040	CENPO	2	24896129	4	0.100851
rs1172293	RBJ	2	25021424	8	3.934101
rs6749438	RBJ	2	25043631	5	1.149137
rs3731631	EFR3B	2	25212926	5	0.782291
rs3731631	EFR3B	2	25212926	6	0.594985
rs3731631	EFR3B	2	25212926	4	1.505883
rs3731631	EFR3B	2	25212926	2	0.832313
rs2164808	EFR3B	2	25230680	1	1.029076
rs2164808	EFR3B	2	25230680	3	0.336721
rs2164808	EFR3B	2	25230680	2	0.058547
rs2164808	EFR3B	2	25230680	7	0.008451
rs2164808	EFR3B	2	25230680	6	1.464977
rs2118404	EFR3B	2	25230833	3	0.59345
rs2276598	DNMT3A	2	25323006	1	3.633314
rs2276598	DNMT3A	2	25323006	3	0.139737
rs1061485	KIF3C	2	26003006	1	5.294591
rs1061485	KIF3C	2	26003006	5	0.386568
SNP2-26388305	GPR113	2	26388305	2	2.635493
SNP2-26388305	GPR113	2	26388305	5	1.406674
SNP2-26388305	GPR113	2	26388305	7	0.715176
SNP2-26388305	GPR113	2	26388305	6	0.734198
SNP2-26388305	GPR113	2	26388305	3	1.564074
SNP2-26388305	GPR113	2	26388305	4	2.509922
rs2052937	GPR113	2	26390198	3	2.625071
rs2052937	GPR113	2	26390198	4	1.915245
rs2052937	GPR113	2	26390198	7	0.932403
rs2052937	GPR113	2	26390198	5	1.139921
rs934280	SELI	2	26461496	2	3.376235
rs1367231	SELI	2	26469905	2	3.44313
rs3795958	C2orf39	2	26520634	3	0.020719
rs3795958	C2orf39	2	26520634	5	0.039427
rs3795958	C2orf39	2	26520634	8	1.650377
rs3795958	C2orf39	2	26520634	1	1.913629

rs6749978	OTOF	2	26554412	7	0.563747
SNP2-26653926	LOC339778	2	26653926	1	0.386935
SNP2-26653926	LOC339778	2	26653926	7	0.361538
SNP2-26653926	LOC339778	2	26653926	6	4.085817
SNP2-26653926	LOC339778	2	26653926	3	0.873604
SNP2-26653926	LOC339778	2	26653926	2	0.01934
SNP2-26653926	LOC339778	2	26653926	8	0.696966
rs744539	CIB4	2	26657626	1	1.697996
rs744539	CIB4	2	26657626	2	1.240348
rs744539	CIB4	2	26657626	7	1.242473
rs744540	CIB4	2	26657647	1	0.31415
rs744540	CIB4	2	26657647	7	0.163583
rs935172	CIB4	2	26657751	1	1.235113
rs41288797	KHK	2	27174019	3	1.509995
rs2384571	CGREF1	2	27178228	3	1.332782
rs2384571	CGREF1	2	27178228	6	4.390078
rs2384571	CGREF1	2	27178228	4	1.383218
rs3739092	CAD	2	27315580	8	9.26759
rs3739092	CAD	2	27315580	3	0.692815
rs2304684	CAD	2	27319872	4	7.871372
GA001964	IFT172	2	27529791	5	0.682162
GA001964	IFT172	2	27529791	7	5.088813
GA001964	IFT172	2	27529791	8	7.160039
GA001964	IFT172	2	27529791	3	0.26908
rs1260326	GCKR	2	27584444	7	0.20202
rs1260326	GCKR	2	27584444	3	0.597676
rs1260326	GCKR	2	27584444	4	1.334272
rs1260326	GCKR	2	27584444	1	4.605959
rs1260326	GCKR	2	27584444	8	3.737501
rs1919125	C2orf16	2	27654907	3	0.335006
rs1919125	C2orf16	2	27654907	4	0.24776
rs1919125	C2orf16	2	27654907	7	0.144979
rs1919125	C2orf16	2	27654907	6	4.294144
rs1919125	C2orf16	2	27654907	5	0.347222
rs1919125	C2orf16	2	27654907	1	6.654261
rs3749147	XAB1	2	27705422	6	0.344585
rs3749147	XAB1	2	27705422	7	0.705579
rs3749147	XAB1	2	27705422	3	0.353353
rs3749147	XAB1	2	27705422	8	1.852629
rs13013484	LOC729833	2	27842325	1	0.357217
rs13013484	LOC729833	2	27842325	4	0.355633
rs13013484	LOC729833	2	27842325	5	0.073353
rs35746006	PLB1	2	28675117	5	0.273136
rs35746006	PLB1	2	28675117	7	0.486742
rs7601771	PLB1	2	28678297	4	0.148548
rs7601771	PLB1	2	28678297	8	0.346073
rs7601771	PLB1	2	28678297	5	0.353904

rs34289907	PLB1	2	28681129	5	0.207705
rs2199619	PLB1	2	28708462	4	0.692617
rs2272387	PLB1	2	28719264	4	2.075743
rs2272387	PLB1	2	28719264	7	2.303356
rs2272386	PLB1	2	28719973	7	2.905598
rs2272386	PLB1	2	28719973	4	2.971541
SNP2-29079008	LOC165186	2	29079008	1	0.162269
SNP2-29093564	LOC165186	2	29093564	5	0.477889
SNP2-29094177	LOC165186	2	29094177	4	2.924792
SNP2-29094177	LOC165186	2	29094177	5	2.992788
SNP2-29109878	LOC165186	2	29109878	3	0.056323
rs10187620	FLJ34931	2	29138485	7	0.882275
rs10187620	FLJ34931	2	29138485	5	0.417287
rs7607027	FLJ34931	2	29138714	5	0.096009
rs7607027	FLJ34931	2	29138714	3	0.131921
rs17692899	FLJ34931	2	29138758	3	0.445078
rs17692899	FLJ34931	2	29138758	4	0.027899
rs1562393	FLJ34931	2	29138960	5	0.268187
rs1562392	FLJ34931	2	29139079	3	0.164019
rs4464231	FLJ34931	2	29139217	3	1.226225
rs958584	FLJ34931	2	29139576	3	0.433445
rs719691	FLJ34931	2	29139697	5	0.268827
rs719691	FLJ34931	2	29139697	4	2.376168
rs719691	FLJ34931	2	29139697	3	2.392696
rs13016696	FLJ34931	2	29140409	4	0.946611
rs13016696	FLJ34931	2	29140409	3	0.21827
rs13016696	FLJ34931	2	29140409	2	4.920859
rs10495767	FLJ34931	2	29140696	2	4.685268
rs10495767	FLJ34931	2	29140696	4	1.988565
rs10495767	FLJ34931	2	29140696	3	0.324668
rs10196859	FLJ34931	2	29140731	2	2.348646
rs10196859	FLJ34931	2	29140731	3	1.43534
rs10196859	FLJ34931	2	29140731	4	0.830587
rs10182566	FLJ34931	2	29141212	3	0.295117
rs10182566	FLJ34931	2	29141212	2	3.880907
rs10182566	FLJ34931	2	29141212	4	0.683727
rs13385188	FLJ34931	2	29149180	2	4.129825
rs13385188	FLJ34931	2	29149180	4	0.797581
rs13385188	FLJ34931	2	29149180	3	0.513517
SNP2-29236760	CLIP4	2	29236760	6	3.476783
SNP2-29236760	CLIP4	2	29236760	4	1.024349
rs3795850	ALK	2	29298962	5	0.184963
rs2293563	ALK	2	29303323	7	1.146494
rs2246745	ALK	2	29794033	2	0.820913
rs2246745	ALK	2	29794033	1	0.022882
rs2246745	ALK	2	29794033	7	0.242692
rs2246745	ALK	2	29794033	3	0.380785

rs13386455	LBH	2	30334369	5	1.384199
rs13386455	LBH	2	30334369	8	0.981496
rs10171296	LBH	2	30334813	8	7.407332
rs10171296	LBH	2	30334813	5	0.886368
rs10171305	LBH	2	30334847	5	0.302115
rs10171305	LBH	2	30334847	8	9.049255
rs9558	LBH	2	30335421	5	0.809001
rs2609945	LYCAT	2	30717830	8	0.033541
rs2609945	LYCAT	2	30717830	4	1.810189
rs2280404	CAPN13	2	30799400	7	1.169802
SNP2-30829465	CAPN13	2	30829465	8	2.348764
SNP2-30829465	CAPN13	2	30829465	1	5.367603
rs508405	CAPN13	2	30834444	7	0.649293
rs508405	CAPN13	2	30834444	8	0.180978
rs13021213	CAPN13	2	30863716	5	0.591957
rs2288100	GALNT14	2	31032322	5	0.326022
rs2288100	GALNT14	2	31032322	8	0.776052
rs4952051	CAPN14	2	31249690	7	1.114593
rs4952051	CAPN14	2	31249690	4	1.20652
rs4952051	CAPN14	2	31249690	3	1.312561
rs4952051	CAPN14	2	31249690	1	4.642566
rs12992405	CAPN14	2	31250836	3	0.034305
rs7422120	CAPN14	2	31264157	3	1.929215
rs7422120	CAPN14	2	31264157	4	2.895924
rs7422120	CAPN14	2	31264157	1	2.152838
rs7587515	CAPN14	2	31268355	1	2.033887
rs7587515	CAPN14	2	31268355	3	0.010599
rs7587515	CAPN14	2	31268355	7	1.019443
rs644926	EHD3	2	31343231	6	5.051698
rs630359	EHD3	2	31344220	7	1.10362
rs630359	EHD3	2	31344220	6	7.066232
rs630359	EHD3	2	31344220	2	3.900691
rs207440	XDH	2	31415916	8	5.360904
rs1884725	XDH	2	31425290	1	1.657722
rs2295475	XDH	2	31443351	5	0.207578
rs4407290	XDH	2	31460174	5	0.071603
rs9332975	SRD5A2	2	31603921	3	1.136973
rs455060	NLRC4	2	32328613	5	0.235534
rs455060	NLRC4	2	32328613	8	0.783504
rs455060	NLRC4	2	32328613	1	3.752821
rs2366894	BIRC6	2	32567210	4	0.957239
rs2366894	BIRC6	2	32567210	5	0.278048
rs2366894	BIRC6	2	32567210	1	5.333364
rs1043463	BIRC6	2	32696799	5	0.0109
rs1043463	BIRC6	2	32696799	6	0.869098
SNP2-32718938	TTC27	2	32718938	5	0.348312
rs2290448	LTBP1	2	33300706	3	1.904879

rs2290448	LTBP1	2	33300706	5	0.076923
rs2290448	LTBP1	2	33300706	8	1.225933
rs2290448	LTBP1	2	33300706	6	0.030631
rs2290448	LTBP1	2	33300706	7	0.766211
rs6706620	LTBP1	2	33426059	2	4.278419
rs6706620	LTBP1	2	33426059	1	0.235501
rs6706620	LTBP1	2	33426059	3	0.483356
rs6543720	RASGRP3	2	33590361	3	0.567296
rs6543720	RASGRP3	2	33590361	2	2.74879
rs35933402	RASGRP3	2	33612899	1	4.904894
SNP2-33617680	RASGRP3	2	33617680	7	1.562397
rs10514775	RASGRP3	2	33642536	6	0.535981
rs10514775	RASGRP3	2	33642536	2	6.302676
rs10514775	RASGRP3	2	33642536	8	4.109948
rs6709378	RASGRP3	2	33642572	6	9.308905
rs6709378	RASGRP3	2	33642572	3	0.288518
rs6709378	RASGRP3	2	33642572	4	4.04785
rs6709378	RASGRP3	2	33642572	7	0.728766
rs6708	RASGRP3	2	33643038	1	0.59652
rs6708	RASGRP3	2	33643038	2	0.474573
rs6708	RASGRP3	2	33643038	7	0.806881
rs6708	RASGRP3	2	33643038	4	0.150949
rs6708	RASGRP3	2	33643038	3	0.173948
rs6708	RASGRP3	2	33643038	6	0.2035
rs11684598	MYADML	2	33806125	7	5.533273
rs11684598	MYADML	2	33806125	2	7.571303
rs11684598	MYADML	2	33806125	5	5.585907
rs11684598	MYADML	2	33806125	4	6.142082
rs11684598	MYADML	2	33806125	6	5.023058
rs1045543	CRIM1	2	36629775	4	0.056951
rs1045543	CRIM1	2	36629775	8	5.114505
rs1045543	CRIM1	2	36629775	5	0.184955
rs1045543	CRIM1	2	36629775	3	0.257688
rs848642	FEZ2	2	36636390	6	0.116612
rs848642	FEZ2	2	36636390	3	0.082316
rs848642	FEZ2	2	36636390	8	0.736494
rs2072526	VIT	2	36889438	5	0.57056
rs12474977	CCDC75	2	37176964	2	4.374189
rs45439591	SULT6B1	2	37255860	1	1.069527
rs3213745	CEBPZ	2	37312215	4	0.282881
rs3213745	CEBPZ	2	37312215	1	0.97763
rs3213745	CEBPZ	2	37312215	7	1.585878
rs3213745	CEBPZ	2	37312215	2	4.070561
rs10177176	PRKD3	2	37371549	2	0.733101
rs10177176	PRKD3	2	37371549	7	0.244094
rs4670697	QPCT	2	37453492	2	5.726591
rs3731852	CDC42EP3	2	37724441	3	0.203787

rs3731852	CDC42EP3	2	37724441	2	3.998043
rs3731852	CDC42EP3	2	37724441	4	8.111798
rs10694	CDC42EP3	2	37725643	3	0.153388
rs4670779	LOC344382	2	37897848	5	7.146269
rs4670779	LOC344382	2	37897848	4	0.985523
rs4670799	FAM82A	2	38031910	1	2.651239
rs4670799	FAM82A	2	38031910	5	1.281789
rs4670799	FAM82A	2	38031910	6	0.447555
rs4670799	FAM82A	2	38031910	8	0.162657
rs4670799	FAM82A	2	38031910	4	0.064795
rs4670799	FAM82A	2	38031910	3	0.366533
rs4670799	FAM82A	2	38031910	7	0.219978
rs9341266	CYP1B1	2	38149498	7	6.075586
rs232620	C2orf58	2	38212168	3	0.417574
rs232620	C2orf58	2	38212168	4	2.011558
rs232620	C2orf58	2	38212168	6	0.083579
rs17420264	LOC391367	2	38366297	4	1.027684
rs2121897	LOC391367	2	38366589	8	0.727325
rs2121897	LOC391367	2	38366589	4	0.209445
rs34873284	ARL6IP2	2	38391083	2	2.15184
rs34873284	ARL6IP2	2	38391083	5	3.155916
rs34873284	ARL6IP2	2	38391083	7	0.723452
rs6741892	GALM	2	38770474	4	0.317235
rs6741892	GALM	2	38770474	5	3.608438
rs3112178	GALM	2	38814471	7	0.058554
rs3770681	DHX57	2	38903645	7	0.411721
rs3770681	DHX57	2	38903645	3	1.23445
rs3770681	DHX57	2	38903645	6	0.073924
rs13386861	LDHAL3	2	41901360	4	5.454267
rs13386861	LDHAL3	2	41901360	1	0.375383
rs4952530	FLJ44450	2	41971005	5	0.631283
rs4952530	FLJ44450	2	41971005	3	0.481117
rs4952530	FLJ44450	2	41971005	2	0.985062
rs4633960	FLJ44450	2	41973654	1	0.09993
rs4633960	FLJ44450	2	41973654	3	0.772751
rs4633960	FLJ44450	2	41973654	6	0.789412
rs4633960	FLJ44450	2	41973654	5	1.565904
rs4633961	FLJ44450	2	41973702	6	0.43531
rs4633961	FLJ44450	2	41973702	1	2.71999
rs4633961	FLJ44450	2	41973702	3	0.732029
rs4633961	FLJ44450	2	41973702	5	2.57825
rs2374377	FLJ44450	2	41973810	3	1.828288
rs2374377	FLJ44450	2	41973810	6	2.599187
rs2374377	FLJ44450	2	41973810	1	2.151684
rs2374377	FLJ44450	2	41973810	5	1.428773
rs2374378	FLJ44450	2	41973864	1	1.143053
rs2374378	FLJ44450	2	41973864	3	1.700727

rs2374378	FLJ44450	2	41973864	5	0.888766
rs2374378	FLJ44450	2	41973864	6	2.871982
rs2374378	FLJ44450	2	41973864	2	2.95762
rs2374379	FLJ44450	2	41973947	3	1.114615
rs2374379	FLJ44450	2	41973947	1	0.285942
rs2374379	FLJ44450	2	41973947	6	1.043932
rs2374379	FLJ44450	2	41973947	5	0.516019
rs2374379	FLJ44450	2	41973947	2	0.767873
rs1044305	LOC91461	2	42138388	1	1.761521
rs2424	LOC91461	2	42139079	6	0.370896
rs2424	LOC91461	2	42139079	8	0.24468
rs2424	LOC91461	2	42139079	2	0.666734
rs1997	COX7A2L	2	42431310	2	0.386264
rs1997	COX7A2L	2	42431310	5	0.884871
rs1997	COX7A2L	2	42431310	1	0.424726
rs11684852	KCNG3	2	42523124	5	3.840014
rs11691584	KCNG3	2	42523902	5	3.101748
rs3796075	NULL	2	42575914	4	0.805118
rs3796075	NULL	2	42575914	5	0.920293
rs3796075	NULL	2	42575914	3	0.755243
rs2278584	OXER1	2	42843248	4	0.121679
rs2278584	OXER1	2	42843248	5	1.227208
rs2278585	OXER1	2	42843498	5	0.515714
rs2304661	HAAO	2	42847988	5	0.538927
rs2304661	HAAO	2	42847988	8	0.837976
rs2304661	HAAO	2	42847988	3	0.801377
rs3816182	HAAO	2	42869208	1	4.423907
rs3816182	HAAO	2	42869208	5	6.038279
rs3816183	HAAO	2	42869223	3	1.398758
rs745763	MGC40574	2	43309158	1	4.476358
rs745763	MGC40574	2	43309158	4	0.288151
rs755862	hCG_1645220	2	43756015	3	0.009588
rs755862	hCG_1645220	2	43756015	2	2.372744
rs755862	hCG_1645220	2	43756015	5	1.150309
rs755862	hCG_1645220	2	43756015	1	0.004302
rs755862	hCG_1645220	2	43756015	4	0.095254
rs12623703	PLEKHH2	2	43780963	5	7.073549
rs12623703	PLEKHH2	2	43780963	4	0.411887
rs12623703	PLEKHH2	2	43780963	2	4.347815
rs10165660	PLEKHH2	2	43846952	5	0.376317
rs10165660	PLEKHH2	2	43846952	7	1.726084
rs13522	PLEKHH2	2	43848201	7	1.472641
rs13522	PLEKHH2	2	43848201	5	0.289611
rs13522	PLEKHH2	2	43848201	6	0.080432
rs13522	PLEKHH2	2	43848201	8	0.355383
rs9309107	DYNC2LI1	2	43875330	7	0.762563
rs9309107	DYNC2LI1	2	43875330	6	3.691742

rs9309107	DYNC2LI1	2	43875330	5	0.080333
rs9309107	DYNC2LI1	2	43875330	3	0.392479
rs9309107	DYNC2LI1	2	43875330	8	0.072253
rs2278357	ABCG5	2	43893343	4	0.047234
rs2278356	ABCG5	2	43893379	3	1.009912
rs6720173	ABCG5	2	43893905	2	3.14794
rs6720173	ABCG5	2	43893905	4	3.783362
rs6720173	ABCG5	2	43893905	6	4.747862
rs6544718	ABCG8	2	43958429	7	1.223908
rs6544718	ABCG8	2	43958429	6	1.111041
rs6544718	ABCG8	2	43958429	5	3.067353
rs3738985	SLC3A1	2	44356292	1	2.198367
rs3738985	SLC3A1	2	44356292	4	0.029306
rs3738985	SLC3A1	2	44356292	6	0.144203
rs3738985	SLC3A1	2	44356292	5	0.139335
rs3738985	SLC3A1	2	44356292	3	0.078043
rs343954	C2orf34	2	44853213	5	0.329846
rs343954	C2orf34	2	44853213	3	0.7117
rs2276655	SRBD1	2	45499016	3	0.113661
rs2276655	SRBD1	2	45499016	7	0.20352
rs880671	ATP6V1E2	2	46599760	6	4.600344
rs880671	ATP6V1E2	2	46599760	1	6.129206
rs880671	ATP6V1E2	2	46599760	3	0.784609
rs896211	ATP6V1E2	2	46600186	2	4.361669
rs6544910	LOC730078	2	46735050	3	0.013713
rs6544910	LOC730078	2	46735050	1	0.157412
rs6544910	LOC730078	2	46735050	5	0.867805
rs17773492	LOC388948	2	46898825	1	5.051856
rs17773492	LOC388948	2	46898825	3	0.243483
rs17773492	LOC388948	2	46898825	6	4.037814
rs17773492	LOC388948	2	46898825	7	1.980349
rs17773492	LOC388948	2	46898825	5	1.376304
rs2278715	LOC388948	2	46902913	4	1.289383
rs2278715	LOC388948	2	46902913	1	0.027083
rs2278715	LOC388948	2	46902913	5	0.59634
rs2278715	LOC388948	2	46902913	6	7.278542
rs2278715	LOC388948	2	46902913	2	0.049402
rs1053952	LOC388948	2	46902957	4	0.211174
rs1053952	LOC388948	2	46902957	8	0.838429
rs1053952	LOC388948	2	46902957	6	0.425928
rs1053952	LOC388948	2	46902957	1	0.236064
rs1053952	LOC388948	2	46902957	2	0.520085
rs17035887	MCFD2	2	46983448	8	7.840705
rs17480869	TTC7A	2	47074126	6	3.130838
rs3739100	TTC7A	2	47132405	2	5.307766
rs815804	C2orf61	2	47235846	7	1.496404
rs815804	C2orf61	2	47235846	6	0.852489

rs11675417	hCG_2040054	2	47607949	1	1.167304
rs35742253	FOXN2	2	48427379	7	1.751073
rs13015195	FLJ46838	2	48611087	5	0.352501
rs13410391	FLJ46838	2	48611327	6	0.605698
rs940389	STON1	2	48661656	6	3.212384
rs940389	STON1	2	48661656	5	0.124421
rs3749145	STON1	2	48662402	5	0.618189
rs3749145	STON1	2	48662402	8	3.677485
rs3749145	STON1	2	48662402	6	1.677509
rs3792234	STON1	2	48663097	3	0.33818
rs3792234	STON1	2	48663097	4	0.246043
rs3792234	STON1	2	48663097	8	2.586419
rs3792234	STON1	2	48663097	5	1.058827
rs3792234	STON1	2	48663097	7	0.330834
rs2140551	STON1	2	48675885	5	0.123398
rs2140551	STON1	2	48675885	3	0.90124
SNP2-48679154	STON1	2	48679154	3	0.429693
rs13396590	GTF2A1L	2	48727166	3	2.447988
SNP2-48727429	GTF2A1L	2	48727429	3	2.250553
rs11125179	LHCGR	2	48769375	7	2.63399
rs6165	FSHR	2	49044545	3	0.540847
rs1394205	FSHR	2	49235089	8	0.882347
rs1394205	FSHR	2	49235089	4	1.365278
rs3930	ASB3	2	53751029	3	0.088612
rs3930	ASB3	2	53751029	2	6.149338
rs3930	ASB3	2	53751029	5	0.242815
rs1049894	PSME4	2	53947460	1	3.846778
rs805408	PSME4	2	53973529	6	6.108166
rs805408	PSME4	2	53973529	4	0.319089
rs805408	PSME4	2	53973529	2	4.413
rs805423	PSME4	2	53980545	1	0.130834
rs805423	PSME4	2	53980545	6	0.115004
rs805423	PSME4	2	53980545	5	0.287878
rs805423	PSME4	2	53980545	2	1.223279
rs805316	PSME4	2	53987248	6	0.265163
rs805316	PSME4	2	53987248	2	3.227284
rs805316	PSME4	2	53987248	4	0.326656
SNP2-54051333	PSME4	2	54051333	5	0.424115
rs1974771	LOC729060	2	54132047	4	0.250954
rs7590819	LOC729060	2	54132108	4	0.647679
rs7590819	LOC729060	2	54132108	2	1.326934
rs1833497	LOC729060	2	54132827	8	3.749757
rs1833497	LOC729060	2	54132827	2	0.74145
rs1833497	LOC729060	2	54132827	1	1.105449
rs843706	TSPYL6	2	54333873	5	0.035347
rs843706	TSPYL6	2	54333873	3	0.923829
rs843705	TSPYL6	2	54335096	4	0.391683

rs843705	TSPYL6	2	54335096	5	0.469208
rs843705	TSPYL6	2	54335096	7	0.268315
rs843705	TSPYL6	2	54335096	2	1.628863
rs843705	TSPYL6	2	54335096	3	0.494857
rs10165485	TSPYL6	2	54335140	3	1.062643
rs843704	TSPYL6	2	54336468	5	0.907312
rs843704	TSPYL6	2	54336468	4	1.107298
rs843704	TSPYL6	2	54336468	2	3.31549
rs843704	TSPYL6	2	54336468	7	1.023611
SNP2-54415516	FLJ40298	2	54415516	1	0.795144
rs2280718	FLJ40298	2	54441100	3	0.492164
rs2280718	FLJ40298	2	54441100	5	0.724779
rs2280718	FLJ40298	2	54441100	4	0.66077
rs3796022	RPL23AP13	2	54608733	5	1.00107
rs3796022	RPL23AP13	2	54608733	4	3.901909
rs17346629	FLJ42562	2	55039898	5	0.012721
rs9677948	C2orf63	2	55290438	7	0.672392
SNP2-55312877	C2orf63	2	55312877	1	3.065985
rs2576693	CCDC88A	2	55370092	7	0.015035
rs17347807	CCDC88A	2	55370791	2	5.245164
rs3748943	NULL	2	55500265	2	1.057528
rs3748943	NULL	2	55500265	7	1.155269
rs3748943	NULL	2	55500265	1	0.781598
rs782652	PNPT1	2	55716279	8	3.273444
rs782652	PNPT1	2	55716279	2	0.031849
rs782652	PNPT1	2	55716279	1	0.21464
SNP2-56273800	CCDC85A	2	56273800	3	0.805617
rs11902680	CCDC85A	2	56465229	1	1.180084
rs11902680	CCDC85A	2	56465229	4	0.322885
rs10198826	LOC442017	2	60763537	6	0.44871
rs10198826	LOC442017	2	60763537	4	0.779301
rs12479056	PUS10	2	61028816	8	0.489763
rs12479056	PUS10	2	61028816	6	6.841011
rs1979045	KIAA1841	2	61218259	4	0.264203
rs3213944	LOC339804	2	61225802	7	0.262524
rs3213944	LOC339804	2	61225802	3	0.646875
rs746032	AHSA2	2	61258512	3	0.506294
rs746032	AHSA2	2	61258512	5	0.871482
rs746032	AHSA2	2	61258512	7	0.518424
rs3856522	AHSA2	2	61259369	5	5.650663
rs3856522	AHSA2	2	61259369	3	0.215953
GA020328	XPO1	2	61618578	3	0.709533
GA020328	XPO1	2	61618578	4	0.30388
GA020328	XPO1	2	61618578	7	4.082625
GA020328	XPO1	2	61618578	5	5.064558
rs4672457	FLJ13305	2	61920431	3	1.321414
rs11125895	FLJ13305	2	61922862	7	0.45575

rs11125895	FLJ13305	2	61922862	1	5.15337
rs4270331	FLJ13305	2	61934516	3	1.362713
rs4233979	TMEM17	2	62581406	3	0.815466
rs4233979	TMEM17	2	62581406	5	0.38737
rs4672527	TMEM17	2	62583178	5	5.499751
rs4672527	TMEM17	2	62583178	2	2.674305
rs11125981	LOC647112	2	63765265	1	3.897096
rs4293553	FLJ36848	2	63921212	7	4.316217
rs4293553	FLJ36848	2	63921212	3	0.153536
rs4293553	FLJ36848	2	63921212	6	0.444085
rs4293553	FLJ36848	2	63921212	2	3.427144
rs17753351	PELI1	2	64188898	3	0.025016
rs1837484	PELI1	2	64191245	2	0.392959
rs1837484	PELI1	2	64191245	3	0.972785
rs1837484	PELI1	2	64191245	1	1.732754
rs2576837	PELI1	2	64192781	1	0.367256
rs2576837	PELI1	2	64192781	3	0.008563
rs7850	SLC1A4	2	65103426	8	0.038665
rs7850	SLC1A4	2	65103426	2	0.000263
rs7850	SLC1A4	2	65103426	4	2.760436
rs1058922	SLC1A4	2	65104045	4	0.995671
rs7572857	CEP68	2	65150302	1	0.839151
rs7572857	CEP68	2	65150302	7	0.040283
rs7572857	CEP68	2	65150302	2	6.235027
rs7572857	CEP68	2	65150302	3	0.493479
SNP2-65152802	CEP68	2	65152802	8	8.145479
SNP2-65152802	CEP68	2	65152802	3	0.486509
SNP2-65152802	CEP68	2	65152802	2	4.789604
rs2008312	hCG_1986447	2	65308148	4	1.662721
rs2008312	hCG_1986447	2	65308148	5	0.613685
rs2008312	hCG_1986447	2	65308148	3	1.461909
rs12622537	LOC730198	2	66507478	3	0.649327
SNP2-67485335	ETAA1	2	67485335	4	1.254326
SNP2-67485335	ETAA1	2	67485335	6	8.136292
SNP2-67485335	ETAA1	2	67485335	7	5.564279
SNP2-67485335	ETAA1	2	67485335	8	6.049103
SNP2-67485335	ETAA1	2	67485335	5	0.515327
rs2044693	PNO1	2	68238601	8	0.658605
rs2044693	PNO1	2	68238601	5	1.188333
rs2044693	PNO1	2	68238601	4	0.933524
rs10197082	PNO1	2	68256334	4	1.141163
rs10197082	PNO1	2	68256334	5	0.997239
rs10197082	PNO1	2	68256334	8	3.52085
rs3816281	PLEK	2	68461451	5	1.222078
rs3816281	PLEK	2	68461451	6	0.325257
rs3816281	PLEK	2	68461451	3	0.006532
rs1063479	PLEK	2	68476418	7	0.991533

rs1063479	PLEK	2	68476418	6	1.704099
rs1063479	PLEK	2	68476418	1	2.512229
rs1050181	PLEK	2	68476456	7	0.563785
rs1050181	PLEK	2	68476456	5	1.308367
rs1050181	PLEK	2	68476456	1	1.642568
rs1050181	PLEK	2	68476456	3	1.269138
rs6725743	PLEK	2	68476494	3	0.96719
rs6742200	PLEK	2	68476806	3	0.783129
rs3732044	PLEK	2	68477419	3	0.107874
rs3732044	PLEK	2	68477419	7	0.246536
rs3732044	PLEK	2	68477419	5	1.137052
rs10185052	C2orf13	2	68659421	3	0.379046
rs11685672	ARHGAP25	2	68855785	4	2.114183
rs11685672	ARHGAP25	2	68855785	8	2.803697
rs11685672	ARHGAP25	2	68855785	6	2.907997
rs2280310	ARHGAP25	2	68894004	5	0.880826
rs2280310	ARHGAP25	2	68894004	3	0.753593
rs17604346	ARHGAP25	2	68899910	5	1.595882
rs4241344	ARHGAP25	2	68903443	5	0.705055
rs4241344	ARHGAP25	2	68903443	3	1.284205
rs3749132	ARHGAP25	2	68907001	5	0.517292
rs3749132	ARHGAP25	2	68907001	3	0.281462
rs4453725	NFU1	2	69512630	2	4.777486
rs4453725	NFU1	2	69512630	8	8.169078
rs4453725	NFU1	2	69512630	4	0.057898
rs6721259	AAK1	2	69563423	5	0.049295
rs6721259	AAK1	2	69563423	1	3.850791
rs2276689	AAK1	2	69595113	1	0.776043
rs2276689	AAK1	2	69595113	4	0.37972
rs2276689	AAK1	2	69595113	5	0.502026
rs2276689	AAK1	2	69595113	2	1.031883
rs7603932	AAK1	2	69599800	5	6.347324
rs3087933	SASP	2	70041042	5	0.087012
rs3796097	SASP	2	70042180	3	1.493986
rs3796097	SASP	2	70042180	4	1.016811
rs11556184	PCYOX1	2	70338824	4	1.472136
rs7595934	PCYOX1	2	70358476	6	0.609769
rs7595934	PCYOX1	2	70358476	5	6.802502
rs7595934	PCYOX1	2	70358476	3	0.31522
rs7595934	PCYOX1	2	70358476	2	4.444446
rs473698	TGFA	2	70529089	4	0.602755
rs473698	TGFA	2	70529089	5	0.315828
rs473698	TGFA	2	70529089	1	6.180029
rs473698	TGFA	2	70529089	7	0.503817
rs473698	TGFA	2	70529089	6	5.279914
rs2166975	TGFA	2	70531502	4	0.35163
rs2166975	TGFA	2	70531502	5	0.643952

rs17698193	ADD2	2	70743006	5	0.572292
rs7566541	FIGLA	2	70866112	4	2.366866
rs7566476	FIGLA	2	70866242	3	0.619491
rs7566476	FIGLA	2	70866242	4	2.166949
rs2287100	CLEC4F	2	70889590	4	3.968898
rs2287101	CLEC4F	2	70889990	4	3.650198
rs722896	CLEC4F	2	70896969	4	4.241319
rs722896	CLEC4F	2	70896969	3	0.866356
rs2075221	CLEC4F	2	70897719	3	1.231845
rs2075221	CLEC4F	2	70897719	4	2.274441
rs2075221	CLEC4F	2	70897719	5	0.601069
rs2080390	CD207	2	70911734	7	2.252899
rs13383830	CD207	2	70911814	7	1.583594
SNP2-70912343	CD207	2	70912343	4	2.998173
SNP2-70912343	CD207	2	70912343	2	4.333221
SNP2-70912343	CD207	2	70912343	7	1.589644
SNP2-70912343	CD207	2	70912343	3	0.72008
SNP2-70912343	CD207	2	70912343	5	0.460091
rs3213749	CD207	2	70912414	7	2.990023
rs17006436	CD207	2	70913619	1	6.188066
rs17718987	CD207	2	70914444	8	0.469173
rs17718987	CD207	2	70914444	5	0.491336
rs17718987	CD207	2	70914444	3	1.439914
rs17718987	CD207	2	70914444	6	1.73084
rs17662453	CD207	2	70914616	7	1.527147
rs17662453	CD207	2	70914616	2	0.8656
rs10489990	CD207	2	70916156	5	3.392089
rs10489990	CD207	2	70916156	1	0.734165
rs10489990	CD207	2	70916156	3	1.28048
rs10489990	CD207	2	70916156	4	2.083002
rs10489990	CD207	2	70916156	2	0.28437
rs2234494	VAX2	2	70981232	7	1.314419
rs2234500	VAX2	2	71013730	7	0.929892
rs3796098	ANKRD53	2	71065323	6	0.485237
rs3796098	ANKRD53	2	71065323	8	4.230576
rs3796098	ANKRD53	2	71065323	5	0.844436
SNP2-71065913	ANKRD53	2	71065913	6	0.008839
SNP2-71065913	ANKRD53	2	71065913	5	0.645239
SNP2-71204995	MCEE	2	71204995	3	0.163028
SNP2-71204995	MCEE	2	71204995	6	0.273813
rs3821248	PAIP2B	2	71267515	2	5.946348
rs2257136	ZNF638	2	71412432	5	0.330869
rs2257136	ZNF638	2	71412432	1	4.27033
rs6741336	DYSF	2	71534458	8	9.065283
rs6741336	DYSF	2	71534458	3	0.045371
rs36122356	DYSF	2	71601431	4	0.256312
rs36122356	DYSF	2	71601431	6	1.179999

rs35984374	DYSF	2	71632337	2	5.110435
rs2303596	DYSF	2	71633723	3	0.528635
rs2303596	DYSF	2	71633723	2	4.497133
rs2288355	DYSF	2	71648660	8	0.04167
rs2288355	DYSF	2	71648660	7	2.279782
rs2288355	DYSF	2	71648660	6	6.638238
rs2288355	DYSF	2	71648660	1	3.981976
rs707718	CYP26B1	2	72209957	1	6.073873
rs707718	CYP26B1	2	72209957	3	0.008387
rs3768647	CYP26B1	2	72210963	1	5.398297
rs653220	EXOC6B	2	72561382	1	5.3056
rs653220	EXOC6B	2	72561382	7	0.353736
rs653220	EXOC6B	2	72561382	6	0.449523
rs12233055	SFXN5	2	73079598	1	1.081873
rs12233055	SFXN5	2	73079598	8	4.590704
rs1046186	RAB11FIP5	2	73155195	1	0.086666
rs1046186	RAB11FIP5	2	73155195	8	4.517784
rs1864488	RAB11FIP5	2	73193216	1	4.457116
rs1864488	RAB11FIP5	2	73193216	8	1.093091
rs1053329	CCT7	2	73314991	1	3.749627
rs1053329	CCT7	2	73314991	8	0.606874
rs1053329	CCT7	2	73314991	3	0.384124
rs1045902	FBXO41	2	73336565	3	0.607386
rs1045902	FBXO41	2	73336565	8	4.778333
rs1045902	FBXO41	2	73336565	1	4.015242
rs1218	FBXO41	2	73336694	4	0.782069
rs34363861	FBXO41	2	73343920	4	2.300632
rs34363861	FBXO41	2	73343920	8	0.16279
rs34363861	FBXO41	2	73343920	3	0.366172
rs34363861	FBXO41	2	73343920	1	4.162538
rs2037814	ALMS1	2	73529177	2	5.77053
rs2037814	ALMS1	2	73529177	8	7.368239
SNP2-73532521	ALMS1	2	73532521	5	0.139825
rs6724782	ALMS1	2	73533498	2	2.385628
rs2017116	ALMS1	2	73570611	2	4.65774
rs1052161	ALMS1	2	73682046	8	5.297065
rs4852974	NAT8B	2	73781439	6	0.068489
rs4852974	NAT8B	2	73781439	3	0.835818
rs4852974	NAT8B	2	73781439	2	3.703101
rs17009399	STAMBP	2	73909821	7	0.809126
rs4853018	SLC4A5	2	74320102	6	0.475929
rs4853018	SLC4A5	2	74320102	8	1.1149
rs4853018	SLC4A5	2	74320102	5	0.599888
rs17009792	SLC4A5	2	74342831	8	0.007882
rs17009792	SLC4A5	2	74342831	7	0.932025
rs9789404	KRT18P26	2	74388199	5	1.505631
rs11555696	DCTN1	2	74442113	5	5.740667

rs2240444	LOC388963	2	74495132	5	6.154985
rs2268416	GCS1	2	74543547	5	0.331442
rs17009998	LBX2	2	74578686	5	0.967507
rs13023123	LBX2	2	74583570	4	0.308547
rs6546909	DQX1	2	74599830	5	0.050498
rs1183739	HTRA2	2	74610056	1	0.192422
rs1183739	HTRA2	2	74610056	4	0.496889
rs2306808	HK2	2	74967297	7	0.446458
rs2306808	HK2	2	74967297	4	0.154131
rs2306808	HK2	2	74967297	6	0.633903
rs10194657	HK2	2	74968616	1	0.314005
rs10194657	HK2	2	74968616	4	0.308142
rs10194657	HK2	2	74968616	2	6.084173
rs10194657	HK2	2	74968616	7	7.14376
rs881	TACR1	2	75129937	8	0.808716
rs881	TACR1	2	75129937	7	0.18411
rs41382847	MRPL19	2	75732867	7	4.867234
rs17741121	C2orf3	2	75743280	5	5.42635
rs6721752	REGL	2	79216759	5	0.823187
rs1982543	LOC442021	2	81275213	5	0.709292
rs1968722	LOC388965	2	84371949	5	5.565535
rs1968722	LOC388965	2	84371949	1	6.008115
rs1968722	LOC388965	2	84371949	2	5.253104
rs11891970	FLJ37357	2	84654116	6	1.299338
rs11891970	FLJ37357	2	84654116	5	1.842545
rs11547160	TCF7L1	2	85389926	5	5.152011
rs7607284	TGOLN2	2	85400887	1	5.086196
rs7607284	TGOLN2	2	85400887	5	0.169947
rs7607284	TGOLN2	2	85400887	7	5.88607
rs1112848	TGOLN2	2	85403255	1	4.614552
rs10460585	TGOLN2	2	85408773	7	0.456891
rs10460585	TGOLN2	2	85408773	3	0.58701
rs7564372	RBED1	2	85443797	6	6.150508
rs955592	RBED1	2	85449329	8	0.929
SNP2-85471424	RBED1	2	85471424	5	0.623268
rs11544042	USP39	2	85696859	1	5.729866
rs2304564	USP39	2	85706178	5	0.719279
rs7316	SFTPB	2	85739524	1	2.005895
rs11127	GNLY	2	85778240	8	2.139896
rs11127	GNLY	2	85778240	1	2.739452
rs11127	GNLY	2	85778240	7	3.664027
rs11127	GNLY	2	85778240	5	2.106521
rs12845	GNLY	2	85779359	7	0.156189
rs12845	GNLY	2	85779359	4	1.093571
rs12845	GNLY	2	85779359	8	1.559427
rs12845	GNLY	2	85779359	1	0.800123
rs12845	GNLY	2	85779359	5	2.238516

rs736991	ATOH8	2	85867856	2	0.119565
rs11395	PTCD3	2	86219118	1	3.878258
rs11395	PTCD3	2	86219118	6	5.018474
rs11395	PTCD3	2	86219118	3	0.621022
rs2278084	PTCD3	2	86221252	6	0.268128
SNP2-86286751	MRPL35	2	86286751	1	0.608974
SNP2-86286751	MRPL35	2	86286751	3	0.808001
rs2241432	MRPL35	2	86291657	3	0.548958
rs2241432	MRPL35	2	86291657	1	0.021314
rs2241432	MRPL35	2	86291657	8	4.979485
rs2241432	MRPL35	2	86291657	2	1.591818
rs2276625	REEP1	2	86335346	2	1.378652
rs2276625	REEP1	2	86335346	7	0.376533
rs2276625	REEP1	2	86335346	8	0.364592
rs2276625	REEP1	2	86335346	1	0.345841
rs2276625	REEP1	2	86335346	3	1.34953
rs3020729	CD8A	2	86865804	2	1.402151
rs3020729	CD8A	2	86865804	6	6.372061
rs3020729	CD8A	2	86865804	3	1.251025
rs3020729	CD8A	2	86865804	4	0.291789
rs35555413	CD8B	2	86896336	5	0.955391
rs3970063	LOC730259	2	87457994	3	0.381669
rs3970063	LOC730259	2	87457994	1	0.113995
rs3970063	LOC730259	2	87457994	4	0.133713
rs3970063	LOC730259	2	87457994	2	1.400399
rs3970063	LOC730259	2	87457994	6	0.111675
rs3970063	LOC730259	2	87457994	7	0.004656
rs3970063	LOC730259	2	87457994	5	5.839397
rs1542088	SMYD1	2	88168518	7	1.022567
rs1542088	SMYD1	2	88168518	5	3.74528
rs1542088	SMYD1	2	88168518	2	0.110501
rs1542088	SMYD1	2	88168518	1	0.470505
rs1542087	SMYD1	2	88168672	5	3.094604
rs1542087	SMYD1	2	88168672	1	3.981089
rs2919882	SMYD1	2	88177235	4	0.303829
rs2919882	SMYD1	2	88177235	1	0.851151
rs1441640	SMYD1	2	88191846	8	0.447757
rs1441640	SMYD1	2	88191846	3	0.434733
rs1441640	SMYD1	2	88191846	2	2.394875
rs1441640	SMYD1	2	88191846	5	3.735073
rs1441640	SMYD1	2	88191846	7	0.720969
rs1441641	SMYD1	2	88192109	7	0.523005
rs1441641	SMYD1	2	88192109	1	3.936745
rs1441641	SMYD1	2	88192109	5	2.46776
rs1441641	SMYD1	2	88192109	4	0.623378
rs2919865	SMYD1	2	88192720	4	2.199472
rs2919865	SMYD1	2	88192720	1	4.030006

rs2919865	SMYD1	2	88192720	7	0.01674
rs2919865	SMYD1	2	88192720	5	3.24558
rs2970889	SMYD1	2	88193019	5	3.852026
rs2970889	SMYD1	2	88193019	4	0.101583
rs2970889	SMYD1	2	88193019	7	0.408929
rs2970889	SMYD1	2	88193019	1	6.780211
rs1978939	SMYD1	2	88193154	1	6.67297
rs1978939	SMYD1	2	88193154	7	1.190005
rs1978939	SMYD1	2	88193154	4	0.286944
rs1978939	SMYD1	2	88193154	5	4.408022
rs1561730	SMYD1	2	88193454	7	4.739677
rs1561730	SMYD1	2	88193454	4	0.165854
rs1561730	SMYD1	2	88193454	1	4.567234
rs1561730	SMYD1	2	88193454	5	3.403321
rs2229169	ADRA2B	2	96144443	5	0.078882
rs9333567	ADRA2B	2	96145580	4	1.584982
rs1657502	ASTL	2	96159335	5	0.063976
rs2276650	STARD7	2	96224886	1	1.924494
rs2279105	KIAA1754L	2	96357484	1	4.528678
rs2279105	KIAA1754L	2	96357484	6	0.190951
rs2279105	KIAA1754L	2	96357484	3	0.519369
rs2279105	KIAA1754L	2	96357484	5	7.76006
rs2305935	NCAPH	2	96393974	5	0.545362
rs2305935	NCAPH	2	96393974	3	0.136499
rs2305935	NCAPH	2	96393974	6	0.220608
rs2305935	NCAPH	2	96393974	1	3.915961
rs3731938	LINCR	2	96529931	1	0.544328
rs3731938	LINCR	2	96529931	8	0.488322
rs3731938	LINCR	2	96529931	6	5.358146
rs3731938	LINCR	2	96529931	2	0.909419
rs11677797	LOC51252	2	96923486	4	1.313219
rs4243786	LOC51252	2	96927472	8	4.36432
SNP2-97643522	ACTR1B	2	97643522	8	1.896681
SNP2-97643522	ACTR1B	2	97643522	2	2.51823
SNP2-98145871	VWA3B	2	98145871	5	0.523743
SNP2-98175864	VWA3B	2	98175864	1	0.233546
rs11896729	UNC50	2	98592077	4	5.087898
rs11896729	UNC50	2	98592077	7	1.696003
rs11896729	UNC50	2	98592077	5	0.438927
rs3203856	UNC50	2	98592453	4	0.520472
rs3203856	UNC50	2	98592453	5	5.224468
rs3203856	UNC50	2	98592453	8	0.986907
rs3203856	UNC50	2	98592453	2	0.182136
rs3203856	UNC50	2	98592453	7	0.013592
rs12618769	MGAT4A	2	98606363	4	0.83408
rs12618769	MGAT4A	2	98606363	7	0.776253
rs2277876	MRPL30	2	99164030	7	0.341023

SNP2-99180046	MRPL30	2	99180046	6	1.001222
SNP2-99180046	MRPL30	2	99180046	4	1.274427
SNP2-99180046	MRPL30	2	99180046	5	0.766681
SNP2-99180046	MRPL30	2	99180046	8	6.375942
rs3087403	REV1	2	99425302	7	0.231709
rs2309821	LONRF2	2	100266736	1	0.613256
rs2309821	LONRF2	2	100266736	5	0.436204
rs2309821	LONRF2	2	100266736	7	0.742504
rs3748928	LONRF2	2	100266811	7	0.865381
rs2309822	LONRF2	2	100266838	7	0.578738
rs2309822	LONRF2	2	100266838	1	0.920598
rs4851287	LONRF2	2	100282204	7	0.657954
rs1530031	CHST10	2	100375758	4	0.023933
rs1530031	CHST10	2	100375758	3	0.111952
rs1530031	CHST10	2	100375758	1	2.486645
rs1530031	CHST10	2	100375758	5	0.083668
rs1530030	CHST10	2	100376032	3	0.007039
rs3748930	CHST10	2	100376514	5	0.74117
rs3748930	CHST10	2	100376514	3	0.783115
rs3748930	CHST10	2	100376514	1	3.09126
rs3828193	CHST10	2	100397993	3	0.115184
rs1562313	NPAS2	2	100953887	8	0.535858
rs9223	NPAS2	2	100976259	6	0.399241
rs1062062	TBC1D8	2	100994357	7	0.150671
rs1007752	C2orf29	2	101245455	1	4.164624
rs1007752	C2orf29	2	101245455	3	0.417956
rs4539813	CREG2	2	101331799	7	2.331841
rs6726192	CREG2	2	101331880	1	0.456302
rs6726192	CREG2	2	101331880	3	0.975674
rs6726192	CREG2	2	101331880	7	1.137328
rs6726192	CREG2	2	101331880	2	0.110348
rs6543032	CREG2	2	101333778	7	1.02674
rs6543032	CREG2	2	101333778	3	1.204965
rs6543032	CREG2	2	101333778	2	1.904474
rs6543032	CREG2	2	101333778	1	1.550195
rs2230400	IL1R2	2	101992580	5	2.487412
rs2302620	IL1RL2	2	102208899	2	0.650322
rs2302620	IL1RL2	2	102208899	6	1.43736
rs2302620	IL1RL2	2	102208899	1	1.073676
rs2302612	IL1RL2	2	102218140	2	0.888052
rs2302612	IL1RL2	2	102218140	5	0.883915
rs2302612	IL1RL2	2	102218140	1	0.616649
rs12905	IL1RL1	2	102326439	7	1.907863
rs12905	IL1RL1	2	102326439	5	0.917071
rs12905	IL1RL1	2	102326439	6	1.822118
rs3771175	IL1RL1	2	102326642	1	0.997201
rs3821204	IL1RL1	2	102326713	7	4.106587

rs3821204	IL1RL1	2	102326713	2	2.660442
rs2160203	IL1RL1	2	102327256	4	0.664136
rs2160203	IL1RL1	2	102327256	1	0.940216
rs2160203	IL1RL1	2	102327256	8	0.630125
rs4988956	IL1RL1	2	102334439	1	3.029697
rs1035130	IL18R1	2	102367834	5	0.253885
rs1035130	IL18R1	2	102367834	7	0.069673
rs1035130	IL18R1	2	102367834	2	4.148605
rs1035130	IL18R1	2	102367834	6	0.691028
rs11465656	IL18R1	2	102379821	7	0.774587
SNP2-10237984(	IL18R1	2	102379840	7	1.200138
rs3771157	IL18R1	2	102379864	6	1.25481
SNP2-10238073	IL18R1	2	102380734	2	1.752767
SNP2-10238073	IL18R1	2	102380734	6	0.336842
SNP2-10238073	IL18R1	2	102380734	7	1.274418
SNP2-10238112	IL18R1	2	102381128	6	0.166613
SNP2-10238112	IL18R1	2	102381128	1	3.300982
rs7603250	IL18RAP	2	102435266	4	6.364878
rs2732839	TMEM182	2	102744992	1	2.905296
rs2540321	TMEM182	2	102800056	8	6.355657
rs2540321	TMEM182	2	102800056	1	4.939033
rs2540321	TMEM182	2	102800056	4	0.118419
rs3811547	LOC284998	2	104729699	7	4.264845
rs2241797	TGFBRAP1	2	105252393	4	0.937235
rs2241797	TGFBRAP1	2	105252393	3	0.017195
rs2241797	TGFBRAP1	2	105252393	2	2.958598
rs2241797	TGFBRAP1	2	105252393	7	9.218619
rs2241797	TGFBRAP1	2	105252393	6	0.831507
rs10469862	LOC729030	2	105876339	3	0.375055
rs1034813	ST6GAL2	2	106869964	7	0.749111
rs2219078	SULT1C3	2	108241630	1	0.248292
rs2219078	SULT1C3	2	108241630	7	0.393065
rs17036058	SULT1C2	2	108277183	5	1.210569
rs11689841	SULT1C2	2	108292004	1	2.113474
rs11689841	SULT1C2	2	108292004	3	0.819956
rs12477830	LOC151234	2	108305167	4	0.924621
rs12477830	LOC151234	2	108305167	3	0.548617
rs1402467	SULT1C4	2	108361240	3	1.633901
rs1478517	EDAR	2	108877369	3	0.140116
rs13001699	EDAR	2	108878666	6	1.090278
rs12623957	EDAR	2	108880086	6	3.883587
rs3829700	10-Sep	2	109728782	4	0.135615
rs3829700	10-Sep	2	109728782	2	0.010906
rs3829700	10-Sep	2	109728782	8	0.055302
rs3829700	10-Sep	2	109728782	1	1.236225
rs3829700	10-Sep	2	109728782	5	0.242651
rs3829700	10-Sep	2	109728782	7	1.048997

rs33958626	NPHP1	2	110316315	7	0.091576
SNP2-111279346	ACOXL	2	111279346	1	2.541714
rs4533473	FLJ44006	2	111572528	1	1.59575
rs13027171	MERTK	2	112403459	1	0.076043
rs7604639	MERTK	2	112468399	6	1.992682
rs7604639	MERTK	2	112468399	7	0.236993
rs7604639	MERTK	2	112468399	3	0.393061
rs7604639	MERTK	2	112468399	4	0.450011
rs7604639	MERTK	2	112468399	1	2.791932
rs3811634	MERTK	2	112471414	2	2.15711
rs3811634	MERTK	2	112471414	7	0.744066
rs3811634	MERTK	2	112471414	6	2.454629
rs3811634	MERTK	2	112471414	1	1.51264
rs3811635	MERTK	2	112471472	3	1.248487
rs3811635	MERTK	2	112471472	4	1.555218
rs3811635	MERTK	2	112471472	6	0.819648
rs3811635	MERTK	2	112471472	1	3.886913
rs4848281	FBLN7	2	112661659	7	4.738233
rs4848281	FBLN7	2	112661659	1	3.929841
rs4848281	FBLN7	2	112661659	8	5.221489
rs11123130	FBLN7	2	112661691	1	5.031661
rs7599551	ZC3H8	2	112690395	5	0.516332
rs7599551	ZC3H8	2	112690395	1	3.076689
SNP2-112784093	ZC3H6	2	112784093	4	6.823223
rs3811040	CKAP2L	2	113215037	3	0.320545
rs3811040	CKAP2L	2	113215037	1	3.967395
rs3811040	CKAP2L	2	113215037	5	0.352215
rs17042341	CKAP2L	2	113230630	4	2.32057
rs17561	IL1A	2	113253694	1	0.522994
rs1071676	IL1B	2	113303904	3	0.217066
rs1071676	IL1B	2	113303904	8	4.684904
rs1071676	IL1B	2	113303904	5	0.774514
rs3811047	IL1F7	2	113387881	3	2.419694
rs2708943	IL1F7	2	113391180	4	1.946925
SNP2-113391192	IL1F7	2	113391192	2	2.812225
SNP2-113391192	IL1F7	2	113391192	4	0.299543
rs2708947	IL1F7	2	113392690	2	2.64121
rs2708947	IL1F7	2	113392690	4	0.133789
rs7584409	IL1F9	2	113459205	3	0.324537
rs11673918	IL1F8	2	113501673	3	1.511075
rs2305150	IL1F8	2	113505787	3	1.66153
rs2305150	IL1F8	2	113505787	6	0.985307
rs1992762	IL1F8	2	113526884	5	1.610228
rs2515401	IL1F5	2	113536947	6	1.08957
rs2515401	IL1F5	2	113536947	5	1.953672
rs3180235	IL1F5	2	113537143	6	2.328512
rs3180235	IL1F5	2	113537143	5	1.759478

rs2515403	IL1F5	2	113537203	5	1.704457
rs2515403	IL1F5	2	113537203	6	0.205063
rs2515404	IL1F5	2	113537588	3	3.100609
rs2515404	IL1F5	2	113537588	6	1.122233
rs768627	IL1F5	2	113538124	5	1.565206
rs768627	IL1F5	2	113538124	6	2.239942
rs13005572	IL1F10	2	113549452	5	0.914412
rs13005572	IL1F10	2	113549452	1	2.031168
rs4849167	PSD4	2	113657310	3	0.781073
rs4849167	PSD4	2	113657310	6	0.405979
rs4849167	PSD4	2	113657310	2	5.006676
rs2276561	PSD4	2	113672842	7	0.836533
rs2276561	PSD4	2	113672842	4	3.028812
rs2276561	PSD4	2	113672842	1	4.203086
rs2019137	PAX8	2	113690103	4	0.895901
rs2019137	PAX8	2	113690103	1	4.351133
rs2019137	PAX8	2	113690103	7	0.252175
rs895412	PAX8	2	113690435	4	2.338717
rs895412	PAX8	2	113690435	1	4.623109
rs895412	PAX8	2	113690435	7	2.900515
rs874898	PAX8	2	113690667	2	3.725816
rs874898	PAX8	2	113690667	1	0.142077
rs7605071	LOC728055	2	114363317	3	0.637678
rs7605071	LOC728055	2	114363317	7	1.572235
rs7605071	LOC728055	2	114363317	6	1.22016
rs7564594	LOC728055	2	114363902	7	0.145214
rs272008	DPP10	2	116318101	3	1.061382
rs2303348	DDX18	2	118300410	4	2.643239
rs2303348	DDX18	2	118300410	5	0.709032
rs2303348	DDX18	2	118300410	6	0.78405
rs2303348	DDX18	2	118300410	3	0.818298
rs2303348	DDX18	2	118300410	8	5.928803
rs11556267	CCDC93	2	118392899	2	0.705129
rs11556267	CCDC93	2	118392899	1	0.246619
rs17569222	CCDC93	2	118394283	1	4.07029
rs6761637	MARCO	2	119455533	3	0.2548
rs6758051	C1QL2	2	119630571	3	0.785665
rs17013371	STEAP3	2	119721782	1	5.694355
rs17805141	STEAP3	2	119722039	6	8.321878
rs8600	LOC130355	2	119776391	6	3.306804
rs6730220	LOC130355	2	119814321	5	2.580045
rs6730220	LOC130355	2	119814321	1	1.563106
rs6730220	LOC130355	2	119814321	8	0.869908
rs6730220	LOC130355	2	119814321	4	1.800782
rs6730220	LOC130355	2	119814321	2	6.003837
rs6730220	LOC130355	2	119814321	7	1.112368
rs6761357	MGC33657	2	120130553	3	0.22164

SNP2-120154993	TMEM177	2	120154993	1	4.603726
SNP2-120154993	TMEM177	2	120154993	8	5.930429
SNP2-120154993	TMEM177	2	120154993	6	1.347503
SNP2-120154993	TMEM177	2	120154993	5	6.710488
rs1983406	TMEM177	2	120155700	1	6.113044
rs1983406	TMEM177	2	120155700	5	1.192636
rs1983406	TMEM177	2	120155700	8	0.349791
rs2278741	GLI2	2	121465537	3	0.399336
rs2278741	GLI2	2	121465537	1	0.369491
rs3795853	TFCP2L1	2	121698078	3	0.183096
rs11890430	TFCP2L1	2	121698420	4	4.36394
rs11890430	TFCP2L1	2	121698420	3	0.896841
rs11890430	TFCP2L1	2	121698420	8	0.268132
rs6568	GYPC	2	127170700	5	0.940672
rs7568070	CYP27C1	2	127669524	6	0.096025
rs7568070	CYP27C1	2	127669524	1	2.336214
rs7568070	CYP27C1	2	127669524	5	0.829417
rs1803541	ERCC3	2	127731383	8	0.418903
rs9282675	ERCC3	2	127768196	3	0.501704
rs3732209	MAP3K2	2	127796276	1	3.320103
rs3732209	MAP3K2	2	127796276	5	0.16797
rs3732209	MAP3K2	2	127796276	3	0.301856
rs2404991	MYO7B	2	128038240	3	0.238876
rs891515	LIMS2	2	128113277	8	0.857532
rs891515	LIMS2	2	128113277	5	0.209002
rs1048211	SFT2D3	2	128175509	1	0.006849
rs1048211	SFT2D3	2	128175509	7	0.168594
rs1048211	SFT2D3	2	128175509	4	0.708721
rs34550770	WDR33	2	128180586	4	0.115365
rs17534123	WDR33	2	128238677	8	0.136921
rs10183605	AMMECR1L	2	128358837	4	0.11708
rs10183605	AMMECR1L	2	128358837	5	0.463677
rs10183605	AMMECR1L	2	128358837	2	5.655593
rs3177472	CCDC74B	2	130613704	8	4.583005
rs3929204	LOC284988	2	130685166	5	6.379923
rs3929204	LOC284988	2	130685166	7	5.839168
rs11542411	IMP4	2	130817204	1	4.539647
rs6752575	LOC730032	2	131388571	3	0.578797
SNP2-131405253	ARHGEF4	2	131405253	3	6.178154
rs7573518	LOC389043	2	131882857	7	3.995
rs7573518	LOC389043	2	131882857	6	3.436499
rs35837041	LOC389049	2	132197350	3	3.847392
rs2241764	GPR39	2	132891234	3	0.814948
rs2241764	GPR39	2	132891234	5	0.561311
rs2241764	GPR39	2	132891234	1	0.524591
rs2241764	GPR39	2	132891234	7	0.82251
rs2241764	GPR39	2	132891234	4	0.148155

rs2241764	GPR39	2	132891234	2	3.867997
rs1131671	LYPD1	2	133119077	5	4.912721
SNP2-133259044	NAP5	2	133259044	5	4.713353
SNP2-133259044	NAP5	2	133259044	4	0.197749
SNP2-133259044	NAP5	2	133259044	8	1.058252
rs1112542	YSK4	2	135460886	3	0.920998
rs10928526	YSK4	2	135462192	3	0.646335
SNP2-135704597	ZRANB3	2	135704597	5	6.119285
rs1042712	LCT	2	136262314	1	0.552862
rs1042712	LCT	2	136262314	4	0.269792
rs1042712	LCT	2	136262314	3	0.197085
rs1042712	LCT	2	136262314	2	5.43762
rs1042712	LCT	2	136262314	7	4.035404
rs1042712	LCT	2	136262314	5	0.813375
rs2322659	LCT	2	136272129	5	0.100879
rs2322659	LCT	2	136272129	7	0.819916
rs2322659	LCT	2	136272129	1	1.018259
rs2322659	LCT	2	136272129	3	1.340725
rs2304371	LCT	2	136278027	3	1.124464
rs3754689	LCT	2	136307216	3	1.443035
rs3754689	LCT	2	136307216	5	0.38201
rs6738266	DARS	2	136459642	5	0.313958
rs4954474	THSD7B	2	137634392	5	0.120574
rs4245861	HNMT	2	138489164	3	0.116991
rs4245861	HNMT	2	138489164	1	5.653292
rs4646333	HNMT	2	138489699	1	4.759117
rs4646333	HNMT	2	138489699	3	0.835377
rs1455158	HNMT	2	138490108	3	0.348905
rs1455158	HNMT	2	138490108	1	4.621247
rs1455156	HNMT	2	138490281	1	5.195306
rs1455156	HNMT	2	138490281	3	0.203817
rs6726278	LOC440917	2	138761322	3	4.984139
rs6726278	LOC440917	2	138761322	1	4.516462
rs13033503	LOC440917	2	138762457	7	0.162848
rs13033503	LOC440917	2	138762457	1	0.059833
rs13382964	LOC440917	2	138762855	1	2.963834
rs4954919	LOC647002	2	138781881	8	4.36268
rs13404361	SPOPL	2	139045361	5	5.824294
rs12470151	LOC129560	2	139375744	3	2.324576
rs12470151	LOC129560	2	139375744	6	2.266295
rs1486961	LRP1B	2	140705983	1	5.068112
rs16843826	LRP1B	2	140825001	7	0.870716
rs16843864	LRP1B	2	140847165	7	0.850534
rs16843864	LRP1B	2	140847165	8	0.472411
rs4954672	LRP1B	2	140991046	2	2.497311
rs4954672	LRP1B	2	140991046	5	0.449836
rs10164457	LRP1B	2	142605607	7	0.898922

rs10211565	LRP1B	2	142605624	2	3.06907
rs897172	ORC4L	2	148494742	3	0.025003
rs897172	ORC4L	2	148494742	8	0.158623
rs897172	ORC4L	2	148494742	7	0.039586
rs897172	ORC4L	2	148494742	6	1.161668
rs897172	ORC4L	2	148494742	5	8.279937
rs1196665	LYPD6	2	150037158	4	1.155126
rs1196665	LYPD6	2	150037158	2	2.912489
rs1196665	LYPD6	2	150037158	5	0.166637
rs1196665	LYPD6	2	150037158	8	0.157285
rs871	LYPD6	2	150038219	2	3.126794
rs4667419	LOC642340	2	150152059	4	0.989973
rs4667419	LOC642340	2	150152059	5	2.642797
rs4667419	LOC642340	2	150152059	8	0.739454
rs4667393	FLJ32955	2	150423086	3	0.872257
rs11551174	NMI	2	151843661	5	5.31268
SNP2-15192875	TNFAIP6	2	151928756	1	1.303127
rs1046668	TNFAIP6	2	151934816	1	2.136594
rs1046668	TNFAIP6	2	151934816	7	1.678954
rs2290368	RIF1	2	151974719	1	2.789278
rs2290368	RIF1	2	151974719	7	1.653052
rs2290368	RIF1	2	151974719	6	0.292447
rs2290368	RIF1	2	151974719	3	0.842947
rs2290368	RIF1	2	151974719	8	3.438769
rs1061305	NEB	2	152055225	7	4.672218
rs1061305	NEB	2	152055225	3	2.270755
rs1061305	NEB	2	152055225	2	6.162866
rs1061305	NEB	2	152055225	4	1.425835
rs1061305	NEB	2	152055225	6	3.289043
rs4664475	NEB	2	152095799	5	0.390768
rs4664475	NEB	2	152095799	1	4.818651
rs4664475	NEB	2	152095799	4	0.734346
rs2288210	NEB	2	152130322	5	0.585367
rs2288210	NEB	2	152130322	8	2.136316
rs2288210	NEB	2	152130322	1	4.876832
rs2288210	NEB	2	152130322	4	1.226201
rs2288211	NEB	2	152130333	7	0.758881
rs2288211	NEB	2	152130333	2	0.151833
rs2288211	NEB	2	152130333	5	0.089731
rs2288211	NEB	2	152130333	6	0.13033
rs2288211	NEB	2	152130333	1	2.530226
SNP2-15218427	NEB	2	152184274	4	0.072476
SNP2-15218427	NEB	2	152184274	6	0.298358
SNP2-15218427	NEB	2	152184274	2	5.070101
SNP2-15218427	NEB	2	152184274	7	0.844799
rs6713162	NEB	2	152204772	8	1.084694
rs6713162	NEB	2	152204772	3	2.847112

rs10170273	NEB	2	152229342	3	2.462735
rs7426114	NEB	2	152235818	8	2.068098
rs7426114	NEB	2	152235818	3	3.441461
SNP2-152235854	NEB	2	152235854	3	2.122523
rs6735208	NEB	2	152244744	3	1.898244
rs6735208	NEB	2	152244744	8	1.384226
rs4611637	NEB	2	152282227	3	1.420793
rs7597215	CACNB4	2	152403437	3	0.423
rs6744595	LOC391453	2	153939388	3	1.702944
rs3863911	LOC642635	2	153986486	1	0.258631
rs3863911	LOC642635	2	153986486	2	2.154988
rs707081	GALNT13	2	155015863	3	2.694944
rs707081	GALNT13	2	155015863	2	4.47233
rs707081	GALNT13	2	155015863	6	0.042569
rs707081	GALNT13	2	155015863	4	0.091072
rs707081	GALNT13	2	155015863	7	0.141106
rs707081	GALNT13	2	155015863	5	0.781836
rs707081	GALNT13	2	155015863	1	0.269625
rs707084	GALNT13	2	155016307	3	2.989065
rs707084	GALNT13	2	155016307	4	1.13879
rs707084	GALNT13	2	155016307	2	1.403717
rs707084	GALNT13	2	155016307	5	1.079589
rs7582614	GALNT13	2	155016918	3	3.324853
rs7582614	GALNT13	2	155016918	7	0.486639
rs7582614	GALNT13	2	155016918	6	1.130416
rs7582614	GALNT13	2	155016918	2	5.887557
rs17266469	GPD2	2	157134218	4	3.175939
rs4556933	ACVR1C	2	158152135	6	4.373245
rs4556933	ACVR1C	2	158152135	8	0.562638
rs4556933	ACVR1C	2	158152135	7	0.580328
rs4556933	ACVR1C	2	158152135	4	0.041604
rs4556933	ACVR1C	2	158152135	3	0.228102
rs17805784	CCDC148	2	158736910	5	0.396506
rs17805784	CCDC148	2	158736910	7	2.032404
rs17805784	CCDC148	2	158736910	4	0.777155
rs7559772	CCDC148	2	158874315	7	0.334326
rs16842890	CCDC148	2	158903528	3	0.30962
rs17810398	DAPL1	2	159369116	7	1.205178
rs9869	DAPL1	2	159371845	5	1.172155
rs9869	DAPL1	2	159371845	1	0.345696
rs9869	DAPL1	2	159371845	4	0.883571
rs10016	DAPL1	2	159380688	3	2.28002
rs10016	DAPL1	2	159380688	1	0.232603
rs10016	DAPL1	2	159380688	2	1.739752
SNP2-15979490(	TANC1	2	159794900	7	0.152621
SNP2-15979490(	TANC1	2	159794900	4	2.642063
SNP2-15979490(	TANC1	2	159794900	3	0.024935

SNP2-15979490(	TANC1	2	159794900	6	9.682967
rs6707733	LOC728059	2	160027836	7	4.631496
rs1126842	CD302	2	160335563	1	0.083224
rs1126842	CD302	2	160335563	8	4.146053
rs1126842	CD302	2	160335563	6	3.567266
rs1126842	CD302	2	160335563	5	0.2308
rs1126842	CD302	2	160335563	2	2.842601
rs35941588	LY75	2	160381765	3	0.353982
rs3951216	LY75	2	160419291	7	1.036998
rs3951216	LY75	2	160419291	6	5.109095
rs3951216	LY75	2	160419291	5	0.103853
SNP2-16058750!	PLA2R1	2	160587505	3	0.732739
SNP2-16058750!	PLA2R1	2	160587505	6	0.664142
SNP2-16059368	PLA2R1	2	160593688	2	2.995954
SNP2-16059368	PLA2R1	2	160593688	1	0.736366
SNP2-16059368	PLA2R1	2	160593688	6	3.366377
SNP2-16059368	PLA2R1	2	160593688	5	0.170606
SNP2-16059368	PLA2R1	2	160593688	8	0.696845
rs1430	RBMS1	2	160882939	1	0.219748
rs1430	RBMS1	2	160882939	2	0.574901
rs1430	RBMS1	2	160882939	3	0.288052
rs1430	RBMS1	2	160882939	4	0.883323
rs17574	DPP4	2	162638225	5	0.566515
rs2298771	SCN1A	2	166601034	6	1.808689
rs2298771	SCN1A	2	166601034	1	0.319555
SNP2-16684935!	SCN9A	2	166849355	3	0.418404
rs13402180	SCN9A	2	166853241	3	1.177488
rs13414203	SCN9A	2	166853388	5	0.179188
rs11888208	SCN7A	2	167021697	5	0.905887
rs7565062	SCN7A	2	167042331	2	2.500375
SNP2-16781394	XIRP2	2	167813941	7	0.730606
SNP2-16782404	XIRP2	2	167824043	4	0.540148
rs836722	B3GALT1	2	168383434	2	1.889569
rs492594	G6PC2	2	169472422	1	0.180742
rs2232328	G6PC2	2	169472792	5	0.521077
rs496550	ABCB11	2	169487958	7	0.434712
rs496550	ABCB11	2	169487958	4	0.450743
rs496550	ABCB11	2	169487958	2	3.028309
rs496550	ABCB11	2	169487958	5	0.614208
rs495714	ABCB11	2	169488010	4	0.040763
rs495714	ABCB11	2	169488010	5	0.405351
rs495714	ABCB11	2	169488010	7	1.75788
rs473351	ABCB11	2	169488142	7	0.816112
rs473351	ABCB11	2	169488142	2	4.056575
rs497692	ABCB11	2	169497262	8	4.071955
SNP2-16953322	ABCB11	2	169533229	7	0.244113
rs2287622	ABCB11	2	169538574	3	1.076916

rs2287622	ABCB11	2	169538574	7	1.394536
rs7563233	ABCB11	2	169550992	7	0.300443
rs3732040	DHRS9	2	169660776	7	0.866632
rs831043	LRP2	2	169811582	5	0.808413
rs830994	LRP2	2	169837774	3	2.505153
rs1879466	BBS5	2	170044270	1	3.504225
rs28763868	KBTBD10	2	170075345	4	0.543336
rs16857030	FASTKD1	2	170111352	4	0.104118
rs16857030	FASTKD1	2	170111352	2	4.520776
rs3820952	LOC129881	2	170259085	8	2.100422
rs3820952	LOC129881	2	170259085	5	0.031912
rs3820952	LOC129881	2	170259085	2	0.848117
rs3820952	LOC129881	2	170259085	1	2.012238
rs3820952	LOC129881	2	170259085	7	1.227033
rs3820952	LOC129881	2	170259085	3	1.213351
rs3820952	LOC129881	2	170259085	4	2.92291
rs10190853	NULL	2	170381089	1	3.443672
rs10190853	NULL	2	170381089	3	0.054843
rs2161916	MYO3B	2	170782133	3	0.549498
rs2161916	MYO3B	2	170782133	1	0.761237
rs2161916	MYO3B	2	170782133	4	0.002742
rs2161916	MYO3B	2	170782133	6	0.289732
rs2161916	MYO3B	2	170782133	2	2.083221
rs17694708	LOC643684	2	171343152	1	4.020464
rs17694708	LOC643684	2	171343152	4	0.820798
rs17694708	LOC643684	2	171343152	5	0.283013
rs3764916	LOC285141	2	171357648	7	0.748224
rs769395	GAD1	2	171425049	1	1.702344
rs13023973	METTL8	2	171896614	1	0.251011
SNP2-17201789	C2orf37	2	172017897	4	0.995256
rs3795996	C2orf37	2	172046220	4	0.823389
rs1047255	CYBRD1	2	172120254	3	1.505349
rs2542938	CYBRD1	2	172121513	2	5.123632
rs11757	SLC25A12	2	172349291	1	0.025385
rs11757	SLC25A12	2	172349291	2	4.117798
rs11757	SLC25A12	2	172349291	7	0.31343
rs788172	DLX1	2	172661684	3	0.393922
rs788172	DLX1	2	172661684	7	0.723565
rs788173	DLX1	2	172661706	7	1.252899
rs743605	DLX2	2	172675548	1	1.33299
rs743605	DLX2	2	172675548	3	0.091848
rs743605	DLX2	2	172675548	7	0.610737
rs17286988	ITGA6	2	173077246	8	0.523229
rs17286988	ITGA6	2	173077246	3	0.087836
rs11887008	ZAK	2	173799334	1	2.536469
SNP2-17383963	ZAK	2	173839638	3	0.056042
rs3739153	OLA1	2	174645524	4	2.661818

rs3739153	OLA1	2	174645524	5	0.286979
rs3739153	OLA1	2	174645524	8	3.949429
rs3739153	OLA1	2	174645524	3	0.636957
rs3739153	OLA1	2	174645524	1	3.511314
rs6737629	OLA1	2	174647414	3	0.205568
rs6737629	OLA1	2	174647414	5	0.005058
rs6737629	OLA1	2	174647414	4	0.1551
rs6737629	OLA1	2	174647414	7	0.369368
rs6737629	OLA1	2	174647414	1	5.235405
rs17316500	OLA1	2	174647433	7	0.976892
rs10930663	GPR155	2	175005606	2	2.95394
rs10930663	GPR155	2	175005606	3	0.23287
rs10930663	GPR155	2	175005606	1	0.208376
GA020792	ATP5G3	2	175754413	5	0.785606
rs12621733	KIAA1715	2	176499888	2	5.319823
rs12622749	KIAA1715	2	176501175	2	3.613944
rs12622783	KIAA1715	2	176501262	2	3.766518
rs863678	HOXD11	2	176682350	8	1.085506
rs6745764	HOXD11	2	176682368	3	0.158427
rs11694628	LOC129841	2	176815802	3	1.889487
rs2305161	MTX2	2	176842418	5	0.857024
rs2305161	MTX2	2	176842418	3	2.116681
rs3821004	TTC30A	2	178188309	7	0.620025
rs16865448	TTC30A	2	178188778	7	0.606308
rs16865449	TTC30A	2	178189018	7	0.032313
rs13034886	PDE11A	2	178201547	6	1.124391
rs13034886	PDE11A	2	178201547	8	0.205126
rs13034886	PDE11A	2	178201547	7	0.386569
rs6734528	PDE11A	2	178201977	8	6.853177
rs6734528	PDE11A	2	178201977	6	0.853354
rs6734528	PDE11A	2	178201977	7	3.260281
rs6734683	PDE11A	2	178202258	8	3.749741
rs6734683	PDE11A	2	178202258	6	1.833811
rs6734683	PDE11A	2	178202258	7	0.57899
rs6720891	PDE11A	2	178390849	1	0.351756
rs6720891	PDE11A	2	178390849	2	1.667354
rs6720891	PDE11A	2	178390849	5	0.083313
rs1435573	PDE11A	2	178644619	8	0.502873
rs1435573	PDE11A	2	178644619	6	4.254507
rs1435573	PDE11A	2	178644619	3	0.20703
rs1435573	PDE11A	2	178644619	1	5.514016
rs12478760	OSBPL6	2	178893297	3	0.887103
rs12478760	OSBPL6	2	178893297	1	2.810145
rs3997878	PRKRA	2	179004872	7	4.454435
rs3997878	PRKRA	2	179004872	4	4.670849
rs3997878	PRKRA	2	179004872	1	4.082478
rs3997878	PRKRA	2	179004872	3	4.927345

SNP2-179034062	DFNB59	2	179034062	5	3.752167
rs3813250	TTN	2	179104204	5	4.719172
SNP2-179109986	TTN	2	179109986	5	4.157229
SNP2-179118528	TTN	2	179118528	2	5.179916
rs11694623	TTN	2	179121698	6	5.094126
rs3813247	TTN	2	179138306	5	0.82267
SNP2-179141467	TTN	2	179141467	5	4.20216
rs3813245	TTN	2	179142385	5	0.046536
rs12464787	TTN	2	179148275	7	1.355361
rs12464787	TTN	2	179148275	2	4.189184
rs4894029	TTN	2	179156094	5	4.461214
rs2042996	TTN	2	179159666	5	2.881787
rs2163009	TTN	2	179163453	5	3.351889
rs2303831	TTN	2	179180938	5	0.082058
rs2288566	TTN	2	179182711	5	0.10689
rs6723526	TTN	2	179206287	1	5.931221
rs6723526	TTN	2	179206287	3	0.542266
rs2288563	TTN	2	179207775	5	0.17576
rs2742344	TTN	2	179277645	5	0.396564
rs2562839	TTN	2	179286975	5	0.045637
rs2562836	TTN	2	179288067	5	0.598259
SNP2-179291098	TTN	2	179291098	2	1.880692
rs746578	TTN	2	179313425	5	3.176438
rs922984	TTN	2	179324132	5	3.514907
rs2291311	TTN	2	179337706	5	0.584859
rs2291312	TTN	2	179339459	5	0.977322
rs2306636	TTN	2	179343181	5	0.079342
SNP2-179346106	TTN	2	179346106	5	0.90705
SNP2-179358653	TTN	2	179358653	7	0.231616
SNP2-179358653	TTN	2	179358653	3	0.420262
rs3749123	ZNF385B	2	180016028	1	0.541833
rs2367796	ZNF385B	2	180016273	5	0.853907
rs10490691	ITGA4	2	182109361	4	0.961739
rs10490691	ITGA4	2	182109361	5	4.606908
rs12614187	CERKL	2	182110736	5	3.289874
rs12614187	CERKL	2	182110736	4	1.60143
SNP2-182489119	SSFA2	2	182489119	7	0.263216
rs6729801	DNAJC10	2	183293000	1	2.440199
rs6729801	DNAJC10	2	183293000	3	0.529591
rs192618	DNAJC10	2	183335749	7	0.588282
rs9288088	NCKAP1	2	183507803	5	0.125622
rs9288088	NCKAP1	2	183507803	8	0.033982
rs9288088	NCKAP1	2	183507803	3	0.713275
rs9288088	NCKAP1	2	183507803	6	3.517192
rs9288088	NCKAP1	2	183507803	1	7.908812
rs2675117	KRT8P10	2	183645160	1	0.238928
rs2675117	KRT8P10	2	183645160	2	0.348878

rs2675117	KRT8P10	2	183645160	6	0.148553
rs2368335	DUSP19	2	183669913	1	0.633379
rs2705730	DUSP19	2	183671935	8	0.028561
rs2705730	DUSP19	2	183671935	6	1.790619
rs2705730	DUSP19	2	183671935	3	0.221162
rs2944346	DUSP19	2	183672414	1	0.967357
rs2944346	DUSP19	2	183672414	6	0.121806
rs898946	LOC129402	2	183833818	2	2.298425
rs898946	LOC129402	2	183833818	7	0.441845
rs898946	LOC129402	2	183833818	5	1.197585
rs12477430	ZNF804A	2	185510608	7	2.020941
rs17826534	FLJ44048	2	186379602	6	0.379997
rs17826534	FLJ44048	2	186379602	5	0.001441
rs17826534	FLJ44048	2	186379602	7	0.958088
rs17826534	FLJ44048	2	186379602	1	5.456369
rs17826534	FLJ44048	2	186379602	3	0.653644
rs1862066	FLJ44048	2	186380157	7	0.03782
rs1862066	FLJ44048	2	186380157	3	0.345137
rs1862066	FLJ44048	2	186380157	6	0.792712
rs1862066	FLJ44048	2	186380157	1	3.906223
rs1862066	FLJ44048	2	186380157	5	0.180137
rs4666690	FLJ44048	2	186381142	5	0.406367
rs4666690	FLJ44048	2	186381142	3	0.680814
rs4666690	FLJ44048	2	186381142	1	0.368351
rs13026081	KIAA1946	2	187334583	7	0.321521
rs13026081	KIAA1946	2	187334583	4	1.201574
rs13026081	KIAA1946	2	187334583	6	0.059284
rs13026081	KIAA1946	2	187334583	3	0.961051
rs2595381	KIAA1946	2	187335812	4	2.899248
rs2682851	KIAA1946	2	187336567	4	0.462303
rs10195758	ZSWIM2	2	187401400	4	0.838286
rs696092	CALCRL	2	187918459	3	0.446958
rs696092	CALCRL	2	187918459	5	1.152862
rs696092	CALCRL	2	187918459	2	2.85221
rs696092	CALCRL	2	187918459	6	1.006551
rs3771095	CALCRL	2	187919357	7	0.340826
rs3771095	CALCRL	2	187919357	5	0.945727
rs3771095	CALCRL	2	187919357	2	2.656327
rs8176629	TFPI	2	188039766	6	1.33389
rs8273	GULP1	2	189168798	4	1.300572
rs13914	COL5A2	2	189605876	1	4.9489
rs10197596	COL5A2	2	189612478	3	1.002546
rs1225099	ANKAR	2	190310758	3	0.55729
SNP2-190328461	OSGEPL1	2	190328461	3	0.234212
SNP2-190328461	OSGEPL1	2	190328461	2	3.578589
rs2289405	ORMDL1	2	190344284	1	4.029761
rs2289405	ORMDL1	2	190344284	6	6.135648

rs6942	ORMDL1	2	190344656	6	0.742391
rs6942	ORMDL1	2	190344656	2	7.099494
rs6942	ORMDL1	2	190344656	1	0.244986
rs5742933	PMS1	2	190357561	6	3.75738
rs5742933	PMS1	2	190357561	1	3.548699
rs5742933	PMS1	2	190357561	4	1.086524
SNP2-190772998	MGC13057	2	190772998	3	0.138197
SNP2-190772998	MGC13057	2	190772998	8	0.007279
SNP2-190772998	MGC13057	2	190772998	4	5.178123
rs11542	HIBCH	2	190777934	3	0.434031
rs11542	HIBCH	2	190777934	8	0.309357
rs11542	HIBCH	2	190777934	4	0.867473
rs11542	HIBCH	2	190777934	7	0.164442
rs2293765	NAB1	2	191229090	7	1.438456
rs2293765	NAB1	2	191229090	6	5.182104
rs2293765	NAB1	2	191229090	4	2.535206
rs2293765	NAB1	2	191229090	1	3.90613
rs2293765	NAB1	2	191229090	3	0.566354
rs1546648	GLS	2	191536448	4	0.798268
rs13035504	GLS	2	191537598	2	5.023121
rs13035504	GLS	2	191537598	1	0.325984
rs3088307	GLS	2	191537657	3	1.041273
rs3088307	GLS	2	191537657	2	3.720221
rs3088307	GLS	2	191537657	7	13.87306
rs13035710	LOC729245	2	192248196	2	5.200498
rs1489802	DNAH7	2	196499521	7	0.761362
rs1489802	DNAH7	2	196499521	4	1.609864
rs1489802	DNAH7	2	196499521	5	1.867865
SNP2-196530249	DNAH7	2	196530249	1	4.639478
SNP2-196530249	DNAH7	2	196530249	5	0.489153
rs6719500	DNAH7	2	196533647	3	1.862775
rs6434811	DNAH7	2	196574665	3	0.157894
SNP2-196600940	DNAH7	2	196600940	8	2.320288
rs7577213	HECW2	2	197165486	1	4.667533
rs7577213	HECW2	2	197165486	3	0.158077
SNP2-197229995	FLJ39660	2	197229995	6	0.673354
SNP2-197229995	FLJ39660	2	197229995	8	2.798808
SNP2-197229995	FLJ39660	2	197229995	4	4.280695
rs17271281	FLJ39660	2	197249541	1	5.798978
rs3795920	PGAP1	2	197411321	8	0.732041
rs3795920	PGAP1	2	197411321	6	3.699309
rs3795920	PGAP1	2	197411321	4	0.759717
rs3754822	COQ10B	2	198035559	1	5.52529
rs3792157	MOBKL3	2	198125104	1	4.699039
rs7592344	MARS2	2	198280706	4	0.942238
rs1064213	PLCL1	2	198658485	8	0.692847
rs16832051	C2orf60	2	200502029	6	0.021342

rs16832051	C2orf60	2	200502029	3	0.208074
rs6435036	C2orf60	2	200503816	3	0.828807
rs6435036	C2orf60	2	200503816	7	1.937897
rs6435036	C2orf60	2	200503816	4	0.658925
rs6435036	C2orf60	2	200503816	6	2.985106
rs6435036	C2orf60	2	200503816	5	0.184883
rs2292309	C2orf47	2	200528600	5	0.253904
rs281766	C2orf47	2	200528750	2	4.638083
rs281766	C2orf47	2	200528750	4	0.83616
SNP2-20123457	AOX1	2	201234575	3	2.608826
SNP2-20147648	NIF3L1	2	201476483	7	0.334046
SNP2-20147648	NIF3L1	2	201476483	5	0.788304
SNP2-20147648	NIF3L1	2	201476483	1	4.654081
rs3900115	CASP10	2	201758922	7	1.024371
rs3900115	CASP10	2	201758922	4	0.563623
rs3900115	CASP10	2	201758922	1	3.757095
rs3900115	CASP10	2	201758922	8	7.00218
rs13010627	CASP10	2	201782343	5	4.396362
rs13010627	CASP10	2	201782343	2	4.596637
rs13006529	CASP10	2	201790704	8	1.568922
rs13006529	CASP10	2	201790704	4	0.170233
rs13006529	CASP10	2	201790704	1	5.722058
rs13006529	CASP10	2	201790704	7	0.604976
rs3769823	CASP8	2	201831240	8	0.704284
rs3769823	CASP8	2	201831240	7	0.157978
rs3769823	CASP8	2	201831240	5	2.931723
rs3769823	CASP8	2	201831240	1	4.136405
rs3769823	CASP8	2	201831240	6	0.035648
rs1045485	CASP8	2	201857834	3	0.105023
rs1045487	CASP8	2	201857941	6	0.3985
rs1045487	CASP8	2	201857941	4	0.244089
rs1045494	CASP8	2	201860026	6	0.991823
rs1045494	CASP8	2	201860026	4	0.040832
rs1035142	ALS2CR12	2	201861323	5	0.226702
rs1035142	ALS2CR12	2	201861323	6	1.646558
rs1035142	ALS2CR12	2	201861323	1	2.918061
rs700635	ALS2CR12	2	201861470	7	0.825545
rs700635	ALS2CR12	2	201861470	5	0.471065
rs700635	ALS2CR12	2	201861470	1	5.744094
rs700635	ALS2CR12	2	201861470	8	0.622243
rs700635	ALS2CR12	2	201861470	6	1.69322
rs700636	ALS2CR12	2	201861497	4	3.524489
rs17468277	ALS2CR12	2	201862445	3	0.029095
rs13014235	ALS2CR12	2	201923737	1	7.710589
rs13014235	ALS2CR12	2	201923737	5	0.052927
rs13014235	ALS2CR12	2	201923737	8	2.720005
rs13014235	ALS2CR12	2	201923737	6	0.129111

rs13014235	ALS2CR12	2	201923737	7	0.12307
rs13014235	ALS2CR12	2	201923737	3	0.550304
rs10804117	ALS2CR11	2	202118545	1	3.97189
rs3754932	MPP4	2	202218059	1	0.245023
rs3754932	MPP4	2	202218059	4	0.053888
rs10931969	MPP4	2	202266932	3	1.132915
rs10931969	MPP4	2	202266932	4	0.00071
rs10931969	MPP4	2	202266932	5	1.319494
rs11300	ALS2	2	202273815	7	0.407971
rs3219168	ALS2	2	202284066	4	0.838707
rs3219168	ALS2	2	202284066	5	0.626965
rs3219168	ALS2	2	202284066	3	2.010183
rs4673222	FZD7	2	202610155	6	6.283401
rs10221698	FLJ39061	2	202647899	6	3.848917
rs10221698	FLJ39061	2	202647899	7	8.4919
rs10221698	FLJ39061	2	202647899	1	4.187363
rs10221698	FLJ39061	2	202647899	3	2.5223
rs10221698	FLJ39061	2	202647899	4	2.988384
rs10221698	FLJ39061	2	202647899	5	2.019799
rs13015146	LOC339809	2	202770348	7	0.219294
rs13015146	LOC339809	2	202770348	4	0.61914
rs13015146	LOC339809	2	202770348	3	0.876112
rs6435156	BMPR2	2	203133720	4	1.803341
rs16839311	ALS2CR13	2	203339801	5	0.131426
rs7597959	LOC442064	2	203346118	3	2.121886
rs7597959	LOC442064	2	203346118	5	2.409591
rs7597959	LOC442064	2	203346118	6	2.150215
rs3181113	CD28	2	204310155	4	1.078658
rs3181113	CD28	2	204310155	3	1.297213
rs4675379	ICOS	2	204534340	4	0.746087
rs4675379	ICOS	2	204534340	1	1.496917
rs236843	PARD3B	2	205538236	4	0.78196
rs236843	PARD3B	2	205538236	8	4.212823
rs236843	PARD3B	2	205538236	7	0.59604
rs236843	PARD3B	2	205538236	1	5.7244
rs3732083	GPR1	2	206749298	3	1.27525
rs3732083	GPR1	2	206749298	4	1.87876
rs3732083	GPR1	2	206749298	8	4.340097
rs3732083	GPR1	2	206749298	1	1.898603
rs3732082	GPR1	2	206749686	3	1.236983
rs3755227	GPR1	2	206750178	3	0.485504
rs3755227	GPR1	2	206750178	7	1.41952
rs2287633	MDH1B	2	207311135	4	4.050897
rs2287632	MDH1B	2	207311466	4	0.301158
rs2287631	MDH1B	2	207311479	4	0.637075
rs13420911	CPO	2	207528521	5	2.673723
rs13420911	CPO	2	207528521	1	2.138303

rs13420911	CPO	2	207528521	7	2.116385
rs11903403	CPO	2	207532629	5	0.5429
rs11903403	CPO	2	207532629	6	0.405189
rs11903403	CPO	2	207532629	1	0.045414
SNP2-207535465	CPO	2	207535465	6	0.134616
rs7582305	CPO	2	207542228	2	3.785123
rs7582305	CPO	2	207542228	4	0.603199
rs7582305	CPO	2	207542228	3	0.802152
rs7582305	CPO	2	207542228	7	0.844757
rs971493	LOC646091	2	208710281	5	2.521079
rs971493	LOC646091	2	208710281	2	2.642201
rs3820900	PTH2R	2	209066272	3	0.014328
rs3820900	PTH2R	2	209066272	1	1.284103
rs1057390	PTH2R	2	209067196	7	0.750957
rs1057392	PTH2R	2	209067307	1	3.644591
rs10198749	LOC402116	2	209752729	7	0.98381
rs10198749	LOC402116	2	209752729	5	0.929523
rs6435517	LOC402116	2	209753538	2	4.944908
rs6435517	LOC402116	2	209753538	6	4.246749
rs6435517	LOC402116	2	209753538	8	1.087263
rs6720659	MAP2	2	210265625	1	5.920699
rs6720659	MAP2	2	210265625	5	0.717905
rs6720659	MAP2	2	210265625	4	0.245101
rs741007	MAP2	2	210265787	1	4.508288
rs741007	MAP2	2	210265787	4	0.293048
rs741007	MAP2	2	210265787	5	0.2832
rs741006	MAP2	2	210266407	4	0.935171
rs741006	MAP2	2	210266407	1	5.466052
rs741006	MAP2	2	210266407	5	0.153496
rs2239672	MAP2	2	210268205	1	4.970853
rs2239672	MAP2	2	210268205	5	0.681175
rs2239672	MAP2	2	210268205	4	0.728329
rs16843618	MAP2	2	210304065	4	0.776778
rs16843618	MAP2	2	210304065	5	0.56812
rs16843618	MAP2	2	210304065	1	8.441502
SNP2-210613439	C2orf67	2	210613439	1	6.650236
rs2286963	ACADL	2	210768295	5	0.183383
rs2286963	ACADL	2	210768295	6	0.076078
rs10932334	LANCL1	2	211049594	5	0.499082
rs10932334	LANCL1	2	211049594	1	5.511351
rs10932334	LANCL1	2	211049594	7	0.26347
rs10932334	LANCL1	2	211049594	3	7.040526
rs2287599	CPS1	2	211189502	5	0.684235
rs2287599	CPS1	2	211189502	3	0.844059
SNP2-211248752	CPS1	2	211248752	2	4.631793
rs10932349	CPS1	2	211251334	5	0.692757
rs6709554	IKZF2	2	213720753	1	4.273325

rs6709554	IKZF2	2	213720753	5	2.288134
rs6709554	IKZF2	2	213720753	8	1.614303
rs2042791	SPAG16	2	214435466	1	3.193182
rs2042791	SPAG16	2	214435466	3	1.906554
rs12623569	SPAG16	2	214502988	1	3.60935
rs726070	ABCA12	2	215521576	2	3.352015
rs17501532	ABCA12	2	215604942	2	1.137906
rs17501837	ABCA12	2	215610019	8	0.511363
rs17501837	ABCA12	2	215610019	3	1.11984
rs1263	FN1	2	215933588	3	1.34712
rs1263	FN1	2	215933588	2	3.852171
rs1263	FN1	2	215933588	4	0.860284
rs1264	FN1	2	215933597	6	0.196604
rs7596677	FN1	2	215981152	1	3.580598
rs7596677	FN1	2	215981152	5	0.999081
rs7596677	FN1	2	215981152	3	0.18084
rs2289202	FN1	2	216006329	6	0.829089
rs876771	4-Mar	2	216856662	7	0.085428
rs11575194	IGFBP5	2	217251973	3	0.398849
rs918949	TNS1	2	218382942	5	0.028221
rs918949	TNS1	2	218382942	2	0.587769
rs918949	TNS1	2	218382942	1	0.381401
rs918949	TNS1	2	218382942	6	1.443653
rs12694422	TNS1	2	218390719	6	0.820089
rs12694422	TNS1	2	218390719	7	0.370146
rs12694422	TNS1	2	218390719	1	3.240598
rs12694422	TNS1	2	218390719	5	0.74818
rs3796026	TNS1	2	218391016	1	3.576997
rs3796026	TNS1	2	218391016	6	2.899226
rs3796026	TNS1	2	218391016	7	0.912937
rs3796026	TNS1	2	218391016	5	0.990172
rs2571445	TNS1	2	218391399	7	0.672094
rs2571442	TNS1	2	218391502	1	1.574877
rs2571442	TNS1	2	218391502	7	0.080777
rs2571442	TNS1	2	218391502	5	0.344819
rs3796028	TNS1	2	218403347	1	0.654756
rs3796028	TNS1	2	218403347	7	0.663014
rs1004814	TNS1	2	218420551	7	3.112992
rs1004814	TNS1	2	218420551	2	2.734918
rs1004814	TNS1	2	218420551	6	0.881849
rs1364641	TNS1	2	218420851	7	0.987286
rs1364641	TNS1	2	218420851	6	0.142298
rs1364641	TNS1	2	218420851	2	4.371891
rs1364642	TNS1	2	218420971	6	0.917022
rs3796031	TNS1	2	218421163	7	0.562651
rs3796031	TNS1	2	218421163	6	0.40702
rs3796033	TNS1	2	218421527	6	0.238689

rs3796033	TNS1	2	218421527	7	0.595484
rs4674259	IL8RB	2	218699250	6	1.455295
rs4674259	IL8RB	2	218699250	1	1.69074
rs1126580	IL8RB	2	218709211	1	0.655029
rs2292555	TMBIM1	2	218855126	7	0.770182
rs2292555	TMBIM1	2	218855126	8	0.27969
rs2292555	TMBIM1	2	218855126	4	1.182204
rs2292555	TMBIM1	2	218855126	1	6.099457
rs4324314	C2orf62	2	218930090	1	1.003525
rs2276631	SLC11A1	2	218957257	6	0.304014
rs1059823	SLC11A1	2	218968088	3	0.428384
rs1059823	SLC11A1	2	218968088	5	0.476428
rs500317	USP37	2	219141197	1	4.207187
rs500317	USP37	2	219141197	6	3.595553
rs500422	USP37	2	219141239	1	6.68189
rs500422	USP37	2	219141239	8	2.585044
rs500422	USP37	2	219141239	5	3.664233
rs500422	USP37	2	219141239	6	3.649732
rs3731867	PLCD4	2	219180978	1	2.183977
rs3731867	PLCD4	2	219180978	8	1.377104
rs3731867	PLCD4	2	219180978	3	0.53222
rs3731867	PLCD4	2	219180978	2	0.67968
rs612874	PLCD4	2	219201168	6	0.969564
rs612874	PLCD4	2	219201168	5	1.793904
rs612874	PLCD4	2	219201168	3	1.092121
rs612874	PLCD4	2	219201168	8	4.023422
rs612874	PLCD4	2	219201168	1	2.688453
rs3770212	ZNF142	2	219216507	5	0.420027
rs3770212	ZNF142	2	219216507	2	4.340278
rs3770213	ZNF142	2	219216616	2	4.514354
rs3770213	ZNF142	2	219216616	3	0.02898
rs3770213	ZNF142	2	219216616	1	0.369646
rs3770213	ZNF142	2	219216616	8	0.069849
rs3770214	ZNF142	2	219217232	1	0.364497
rs3770214	ZNF142	2	219217232	3	1.012963
rs3770214	ZNF142	2	219217232	2	0.788599
rs4674324	ZNF142	2	219231677	5	0.501195
rs4674324	ZNF142	2	219231677	1	3.1767
rs4674324	ZNF142	2	219231677	6	0.693297
rs4674324	ZNF142	2	219231677	3	0.675576
rs2303562	RNF25	2	219237491	6	2.491787
rs2303562	RNF25	2	219237491	5	0.845197
rs2303564	RNF25	2	219238125	2	4.700501
rs2303564	RNF25	2	219238125	5	3.360309
rs16859170	RNF25	2	219241622	6	0.499343
rs16859170	RNF25	2	219241622	2	4.668933
rs16859170	RNF25	2	219241622	5	0.300428

rs45586733	STK36	2	219261667	2	2.461591
rs1344642	STK36	2	219263506	5	0.280977
rs1344642	STK36	2	219263506	1	3.581332
rs1344642	STK36	2	219263506	3	0.328012
rs1863704	STK36	2	219270919	3	0.284639
rs1863704	STK36	2	219270919	2	0.629054
rs1863704	STK36	2	219270919	1	0.517697
rs12993599	STK36	2	219271846	2	5.08772
rs12993599	STK36	2	219271846	6	6.057298
rs12993599	STK36	2	219271846	5	5.154853
rs3731877	TTLL4	2	219310743	6	0.917646
rs3731877	TTLL4	2	219310743	3	0.135438
rs3731877	TTLL4	2	219310743	1	7.110281
SNP2-219603792	CCDC108	2	219603792	5	0.776854
SNP2-219603792	CCDC108	2	219603792	1	1.767383
SNP2-219603792	CCDC108	2	219603792	3	0.399813
SNP2-219603792	CCDC108	2	219603792	7	0.3076
rs4674354	CCDC108	2	219611967	3	0.195495
rs3731878	IHH	2	219628809	7	0.085099
rs3731878	IHH	2	219628809	5	0.342669
rs1127101	C2orf24	2	219748574	7	1.287364
rs3210652	FAM134A	2	219755084	5	5.961014
rs3210652	FAM134A	2	219755084	3	0.536791
rs3210652	FAM134A	2	219755084	1	4.180231
SNP2-219780675	ZFAND2B	2	219780675	4	0.2942
SNP2-219780675	ZFAND2B	2	219780675	1	3.659607
rs1109867	ABCB6	2	219791697	6	0.612283
rs1109867	ABCB6	2	219791697	2	0.266871
rs11552796	GLB1L	2	219816544	6	4.320121
rs11552796	GLB1L	2	219816544	2	2.833425
rs3731892	TUBA4B	2	219844595	6	2.112186
rs41272699	DES	2	219993120	3	0.000845
rs1058261	DES	2	219993553	4	0.237658
rs1058261	DES	2	219993553	5	2.439924
rs1058261	DES	2	219993553	1	4.160549
rs12920	DES	2	219993910	1	4.517058
rs12920	DES	2	219993910	5	0.458363
rs12920	DES	2	219993910	4	0.09862
rs1058284	DES	2	219994386	1	4.203141
rs1058284	DES	2	219994386	5	0.049428
rs1058284	DES	2	219994386	4	0.71559
rs45519033	SPEG	2	220050248	8	2.711883
SNP2-220056995	SPEG	2	220056995	5	1.116028
SNP2-220056995	SPEG	2	220056995	6	4.654736
SNP2-220056995	SPEG	2	220056995	4	0.962199
GA002821	SPEG	2	220062352	8	5.948638
GA002821	SPEG	2	220062352	4	1.259574

GA002821	SPEG	2	220062352	1	3.427411
rs12473286	NULL	2	220064764	6	4.818395
rs12473286	NULL	2	220064764	1	5.994639
rs12473286	NULL	2	220064764	4	2.694985
rs12473286	NULL	2	220064764	5	0.463116
rs11689281	ACCN4	2	220110915	1	1.709253
rs11689281	ACCN4	2	220110915	5	2.35211
SNP2-220129029	OBSL1	2	220129029	7	0.446188
SNP2-220129029	OBSL1	2	220129029	5	0.401568
SNP2-220129029	OBSL1	2	220129029	2	8.520723
SNP2-220129029	OBSL1	2	220129029	6	6.041673
SNP2-220129029	OBSL1	2	220129029	1	0.283983
rs1983210	OBSL1	2	220129661	5	5.195677
rs1983210	OBSL1	2	220129661	7	0.034578
rs1983210	OBSL1	2	220129661	2	0.761666
rs1983210	OBSL1	2	220129661	1	0.568587
rs1983210	OBSL1	2	220129661	6	1.855637
SNP2-220141201	OBSL1	2	220141201	5	0.95068
rs673951	STK11IP	2	220181599	3	0.9366
rs673951	STK11IP	2	220181599	7	5.205506
rs673951	STK11IP	2	220181599	5	0.030558
rs635311	SLC4A3	2	220210611	7	0.940603
rs635311	SLC4A3	2	220210611	1	4.468671
rs635311	SLC4A3	2	220210611	4	0.947747
rs684428	SLC4A3	2	220213509	4	0.483101
rs684428	SLC4A3	2	220213509	1	4.913536
rs9758	EPHA4	2	221991244	5	0.154414
rs9758	EPHA4	2	221991244	4	0.654115
rs4674664	SGPP2	2	223131606	4	5.546846
rs12467164	FARSB	2	223229041	1	3.03983
rs12720447	KCNE4	2	223625861	6	0.990454
rs12621643	KCNE4	2	223626227	5	0.060237
rs3795884	KCNE4	2	223626607	2	1.413926
rs3795884	KCNE4	2	223626607	3	0.414184
rs721233	SCG2	2	224170958	7	9.690332
rs16864975	SCG2	2	224171408	8	0.65136
rs11678024	LOC646696	2	224277912	6	0.650225
rs3738961	AP1S3	2	224410549	3	0.27172
rs3738961	AP1S3	2	224410549	6	5.671864
rs3738961	AP1S3	2	224410549	1	4.18429
rs13022532	WDFY1	2	224449454	2	7.689741
rs3738954	FAM124B	2	224953132	3	0.524581
rs3738953	FAM124B	2	224953167	2	5.181521
rs3845837	FAM124B	2	224974911	1	3.962411
rs2396092	CUL3	2	225043534	5	3.39996
rs2396092	CUL3	2	225043534	6	0.687521
rs4674908	CUL3	2	225043839	7	0.218117

rs4674908	CUL3	2	225043839	1	4.709115
rs4674908	CUL3	2	225043839	3	0.006626
rs4674908	CUL3	2	225043839	5	0.518619
rs3768899	CUL3	2	225044553	6	4.811381
rs3768899	CUL3	2	225044553	5	4.766037
rs10498160	CUL3	2	225044775	6	0.259931
rs10498160	CUL3	2	225044775	5	0.884166
rs3768898	CUL3	2	225045014	6	1.169855
rs3768898	CUL3	2	225045014	5	0.145299
rs10498161	CUL3	2	225045133	6	0.980283
rs10498161	CUL3	2	225045133	5	0.293066
rs1045654	DOCK10	2	225338477	5	0.132985
rs1045654	DOCK10	2	225338477	4	0.287743
rs1045654	DOCK10	2	225338477	1	1.863673
rs4674941	DOCK10	2	225379145	7	1.436921
rs4674941	DOCK10	2	225379145	1	0.407561
rs7572725	DOCK10	2	225392410	7	1.085627
rs7572725	DOCK10	2	225392410	1	2.199672
rs11884382	KIAA1486	2	226224590	3	4.356984
rs3791734	KIAA1486	2	226224673	3	0.025058
rs10180401	KIAA1486	2	226225157	4	0.94482
rs4627553	KIAA1486	2	226225426	4	0.255091
rs1801278	IRS1	2	227368788	2	5.717844
rs2228557	COL4A4	2	227580426	1	5.722314
rs2228557	COL4A4	2	227580426	4	2.153384
rs2228557	COL4A4	2	227580426	7	1.57643
rs2228557	COL4A4	2	227580426	5	1.403282
rs2228557	COL4A4	2	227580426	8	0.477027
rs2228557	COL4A4	2	227580426	3	1.38734
rs10187726	COL4A4	2	227581239	4	1.100165
rs10187726	COL4A4	2	227581239	5	3.745289
rs10187726	COL4A4	2	227581239	8	2.137426
rs10187726	COL4A4	2	227581239	1	3.884465
rs10187726	COL4A4	2	227581239	3	1.453336
rs10187726	COL4A4	2	227581239	7	1.319754
rs3817490	COL4A4	2	227600863	1	1.546439
rs2272199	COL4A4	2	227600964	1	0.985851
rs4675141	COL4A4	2	227624076	1	3.207131
rs13027659	COL4A4	2	227624091	1	1.553223
rs13424243	COL4A3	2	227810967	7	3.937037
rs13424243	COL4A3	2	227810967	8	4.761685
rs10178458	COL4A3	2	227819679	5	0.356737
SNP2-22782934[illegible]	COL4A3	2	227829345	3	1.938557
SNP2-22782934[illegible]	COL4A3	2	227829345	7	2.149958
SNP2-22782934[illegible]	COL4A3	2	227829345	2	1.256699
rs11677877	COL4A3	2	227839413	1	4.143719
rs2070735	COL4A3	2	227885145	1	4.390887

rs7587228	COL4A3	2	227885723	1	3.152517
rs4470338	COL4A3	2	227887282	4	0.541956
rs4470338	COL4A3	2	227887282	7	1.132015
rs4428010	TM4SF20	2	227935338	4	0.72916
rs4428010	TM4SF20	2	227935338	7	1.088867
rs13426457	HRB	2	228124956	3	0.816805
rs2138405	DKFZp547H025	2	228183371	6	0.531047
rs2138405	DKFZp547H025	2	228183371	1	0.079698
rs2138404	DKFZp547H025	2	228183411	5	1.379974
rs2138402	DKFZp547H025	2	228184497	6	0.323399
rs2138402	DKFZp547H025	2	228184497	2	0.941046
rs7557339	LOC729968	2	228303801	8	4.575752
rs1715828	WDR69	2	228466798	7	0.079525
rs1715828	WDR69	2	228466798	8	6.128235
rs1715828	WDR69	2	228466798	1	5.937899
rs1715828	WDR69	2	228466798	3	1.243332
rs3811515	SPHKAP	2	228591273	7	2.890443
rs7355333	SLC16A14	2	230608009	4	0.578296
SNP2-23061920	SLC16A14	2	230619209	5	0.036197
SNP2-23061920	SLC16A14	2	230619209	8	3.599993
rs3948463	SP110	2	230745104	2	5.159629
rs3948463	SP110	2	230745104	6	6.173878
SNP2-23078535	SP110	2	230785356	4	6.819038
SNP2-23078535	SP110	2	230785356	5	0.569183
SNP2-23078535	SP110	2	230785356	6	0.323158
SNP2-23078535	SP110	2	230785356	1	5.288487
SNP2-23078592	SP110	2	230785920	5	9.472663
SNP2-23078592	SP110	2	230785920	6	5.753552
SNP2-23078592	SP110	2	230785920	8	8.167863
SNP2-23078592	SP110	2	230785920	1	0.281964
rs10498247	SP140	2	230886066	1	4.48059
rs10498247	SP140	2	230886066	4	2.302024
rs7559665	LOC93349	2	230965056	8	1.119831
rs7590429	LOC93349	2	230966394	5	2.962907
rs7590429	LOC93349	2	230966394	4	0.78664
rs7590429	LOC93349	2	230966394	1	4.153109
rs11692506	LOC93349	2	230976169	5	0.735791
rs11692506	LOC93349	2	230976169	1	1.144303
rs11692506	LOC93349	2	230976169	8	1.04284
rs11692506	LOC93349	2	230976169	2	2.057797
rs17273079	LOC93349	2	230976568	2	2.837305
rs17273079	LOC93349	2	230976568	1	0.004495
rs17273079	LOC93349	2	230976568	8	3.590678
rs17273079	LOC93349	2	230976568	5	0.09983
rs2396763	LOC646839	2	231145323	2	0.687611
rs2396763	LOC646839	2	231145323	3	0.658031
rs2396763	LOC646839	2	231145323	7	0.522833

rs6716468	CAB39	2	231391865	4	0.195036
rs1992188	GPR55	2	231480680	5	1.241667
rs1992188	GPR55	2	231480680	4	0.346744
rs1992188	GPR55	2	231480680	3	3.781672
rs1992186	GPR55	2	231483541	3	4.03503
rs4972979	SPATA3	2	231587078	5	1.33812
rs3731783	LOC257407	2	231621300	5	0.579715
rs13430407	PSMD1	2	231743673	3	0.479182
rs13430407	PSMD1	2	231743673	5	0.586648
rs13430407	PSMD1	2	231743673	2	2.345251
rs2290130	B3GNT7	2	231971371	3	0.184731
rs2290130	B3GNT7	2	231971371	1	4.860533
rs7580705	C2orf52	2	232081540	1	3.36999
rs2245601	CHRND	2	233099181	1	2.099741
rs2245601	CHRND	2	233099181	7	1.017538
rs2767	CHRND	2	233108318	5	0.615397
rs2099489	CHRNG	2	233118538	3	0.482216
rs2276557	EFHD1	2	233206913	6	2.367056
rs1801251	KCNJ13	2	233341704	8	0.95896
rs1801251	KCNJ13	2	233341704	5	3.99636
rs1801251	KCNJ13	2	233341704	4	4.583632
rs1801251	KCNJ13	2	233341704	6	0.363061
rs3738904	TNRC15	2	233430337	7	1.722729
rs1141328	NULL	2	233633410	8	0.38496
rs1141328	NULL	2	233633410	2	1.926956
rs1141328	NULL	2	233633410	3	0.263618
rs6861	ATG16L1	2	233868852	6	0.262747
rs6861	ATG16L1	2	233868852	8	1.735645
SNP2-23388947	SAG	2	233889477	4	2.499386
rs9752946	DGKD	2	234036090	8	4.224836
rs1053895	DGKD	2	234043641	8	0.149217
rs1053895	DGKD	2	234043641	6	0.927662
rs882428	DGKD	2	234044862	4	0.641263
rs2971863	USP40	2	234050873	2	0.72331
rs2971863	USP40	2	234050873	1	1.126614
rs2971863	USP40	2	234050873	4	0.582798
rs2167884	USP40	2	234114055	4	0.135788
rs2167884	USP40	2	234114055	1	0.273886
rs10929303	UGT1A8	2	234346155	7	1.611008
rs10929303	UGT1A8	2	234346155	6	1.654846
rs10929303	UGT1A8	2	234346155	8	0.085356
rs10929303	UGT1A8	2	234346155	1	4.566771
rs10929303	UGT1A8	2	234346155	4	0.00812
rs8330	UGT1A8	2	234346384	6	4.031815
rs8330	UGT1A8	2	234346384	1	5.356571
rs8330	UGT1A8	2	234346384	4	4.293075
rs2361503	LOC339766	2	234369057	4	2.830494

rs2361503	LOC339766	2	234369057	7	2.380388
rs2361503	LOC339766	2	234369057	6	2.406582
rs759173	LOC339766	2	234369429	5	2.179295
rs759173	LOC339766	2	234369429	6	1.440954
rs759173	LOC339766	2	234369429	1	1.900404
rs6754859	LOC339766	2	234392725	1	1.639906
rs6754859	LOC339766	2	234392725	5	0.550747
rs1500480	LOC339766	2	234392817	7	4.63208
rs2270856	LOC339766	2	234406547	3	0.057483
rs3732215	HJURP	2	234415281	4	7.12346
rs3732215	HJURP	2	234415281	7	0.336123
rs3732215	HJURP	2	234415281	1	0.855925
rs529963	HJURP	2	234423239	7	4.812641
rs529963	HJURP	2	234423239	1	5.90475
rs188492	MSL3L2	2	234438815	1	4.431994
rs188492	MSL3L2	2	234438815	7	0.182817
rs188492	MSL3L2	2	234438815	6	1.362533
rs188492	MSL3L2	2	234438815	3	0.119061
rs13384181	MSL3L2	2	234440085	3	0.430502
rs13384181	MSL3L2	2	234440085	6	0.818732
rs213545	MSL3L2	2	234440680	4	0.904252
rs213545	MSL3L2	2	234440680	8	3.947305
rs213545	MSL3L2	2	234440680	2	5.724394
rs213545	MSL3L2	2	234440680	3	1.067545
rs213545	MSL3L2	2	234440680	6	0.955007
rs11563208	TRPM8	2	234569817	6	2.398061
rs11563208	TRPM8	2	234569817	8	5.979804
rs11563208	TRPM8	2	234569817	5	0.219722
rs11901004	TRPM8	2	234591999	8	0.31668
rs2052029	TRPM8	2	234592427	3	0.36809
rs28902238	TRPM8	2	234592432	1	4.668421
rs28902238	TRPM8	2	234592432	5	6.945603
rs763379	TRPM8	2	234592520	1	5.358725
rs763379	TRPM8	2	234592520	5	2.237734
rs763380	TRPM8	2	234592529	1	4.369557
rs763380	TRPM8	2	234592529	5	0.130478
rs8245	ARL4C	2	235068726	7	0.341147
rs8245	ARL4C	2	235068726	1	2.429809
rs3795962	SH3BP4	2	235616558	6	5.063579
rs3795962	SH3BP4	2	235616558	7	7.108419
rs3795962	SH3BP4	2	235616558	3	0.122318
rs3795962	SH3BP4	2	235616558	4	7.722373
rs4589703	SH3BP4	2	235627545	1	3.611596
rs2292708	CENTG2	2	236372905	4	0.546613
rs2292708	CENTG2	2	236372905	3	1.523602
rs2292708	CENTG2	2	236372905	2	2.048618
rs2292708	CENTG2	2	236372905	6	1.777359

rs2292708	CENTG2	2	236372905	5	1.737416
rs1050785	COL6A3	2	237897491	8	1.167882
rs1050785	COL6A3	2	237897491	5	0.128058
rs1050785	COL6A3	2	237897491	1	2.352227
rs7436	COL6A3	2	237897550	7	0.88118
rs7436	COL6A3	2	237897550	1	5.45039
rs4663722	COL6A3	2	237898149	7	1.615137
rs4663722	COL6A3	2	237898149	1	2.725172
rs6728818	COL6A3	2	237909702	8	0.691275
rs6728818	COL6A3	2	237909702	6	0.002762
rs6728818	COL6A3	2	237909702	4	1.474488
rs36104025	COL6A3	2	237912473	4	0.240542
rs4433949	COL6A3	2	237914369	3	0.583799
rs4433949	COL6A3	2	237914369	8	3.010073
rs4433949	COL6A3	2	237914369	1	1.989723
rs2291795	COL6A3	2	237917804	4	2.146498
rs3790993	COL6A3	2	237923553	1	0.926351
rs3790993	COL6A3	2	237923553	3	0.710665
rs3790993	COL6A3	2	237923553	8	1.389845
rs12622093	COL6A3	2	237948344	3	0.874956
rs3751109	MLPH	2	238091933	3	2.235406
rs3751109	MLPH	2	238091933	8	0.206074
rs3751109	MLPH	2	238091933	4	0.39545
rs2292881	MLPH	2	238098988	3	0.276147
rs11883500	MLPH	2	238099173	3	1.342527
rs2292884	MLPH	2	238107965	8	0.50608
rs2292884	MLPH	2	238107965	4	0.846126
rs3817362	MLPH	2	238113746	6	0.127121
SNP2-238159481	RAB17	2	238159481	6	0.208726
SNP2-238159481	RAB17	2	238159481	3	0.06666
SNP2-238159481	RAB17	2	238159481	1	3.631841
rs2292873	RAB17	2	238164183	1	5.899542
rs2292872	RAB17	2	238164273	3	1.151602
rs7609062	LRRFIP1	2	238336628	2	3.039537
rs3739038	LRRFIP1	2	238337442	4	4.926684
rs3739038	LRRFIP1	2	238337442	6	6.658996
rs3739038	LRRFIP1	2	238337442	1	0.822428
rs3739038	LRRFIP1	2	238337442	2	5.77447
rs4663795	RBM44	2	238397755	1	5.073593
rs4663795	RBM44	2	238397755	4	0.022893
rs1128552	SCLY	2	238664622	5	0.688927
rs1128552	SCLY	2	238664622	3	0.837566
rs12993309	SCLY	2	238671618	6	0.9786
rs12993309	SCLY	2	238671618	8	6.680352
rs12993309	SCLY	2	238671618	3	0.136522
rs12993309	SCLY	2	238671618	4	4.603719
rs12993309	SCLY	2	238671618	5	0.778162

rs1054641	SCLY	2	238672589	3	0.0551
rs935415	ESPNL	2	238681278	3	1.003066
SNP2-238703814	ESPNL	2	238703814	5	1.86481
SNP2-238703814	ESPNL	2	238703814	3	3.061144
SNP2-238703814	ESPNL	2	238703814	6	1.892627
SNP2-238703814	ESPNL	2	238703814	4	2.422656
rs935412	ESPNL	2	238706565	1	1.608226
rs935412	ESPNL	2	238706565	3	0.698106
rs935411	KLHL30	2	238716289	7	0.53879
rs3795905	FAM132B	2	238740551	5	0.894137
rs3820817	FAM132B	2	238740674	6	1.400593
rs3820817	FAM132B	2	238740674	1	1.076904
rs7572285	LOC151174	2	238798795	8	0.222861
rs7572285	LOC151174	2	238798795	4	0.337528
rs7572285	LOC151174	2	238798795	1	1.774727
rs7572285	LOC151174	2	238798795	2	2.236959
SNP2-238826696	PER2	2	238826696	2	4.928748
rs2304670	PER2	2	238830375	5	0.039221
rs477041	ASB1	2	239020734	5	5.489948
rs477041	ASB1	2	239020734	4	0.029565
rs514795	ASB1	2	239023591	3	0.05204
rs514795	ASB1	2	239023591	1	6.316457
rs514795	ASB1	2	239023591	5	0.181965
rs514795	ASB1	2	239023591	4	4.281373
rs514795	ASB1	2	239023591	6	0.270879
rs13383477	FLJ43879	2	239512115	8	3.072124
rs13383477	FLJ43879	2	239512115	6	3.882996
rs13393217	HDAC4	2	239637498	3	0.634982
rs13393217	HDAC4	2	239637498	1	3.255154
rs11683242	FLJ45964	2	240165231	5	0.794642
rs11683242	FLJ45964	2	240165231	8	2.536574
rs11683242	FLJ45964	2	240165231	1	2.421278
rs2008714	FLJ45964	2	240172268	7	0.909372
rs2008714	FLJ45964	2	240172268	4	0.720173
rs1574030	LOC150935	2	240386665	3	1.077158
rs6728493	LOC643905	2	240629935	3	1.254269
rs6732185	LOC643905	2	240630048	1	0.940926
rs6732185	LOC643905	2	240630048	3	0.181733
rs6732185	LOC643905	2	240630048	2	0.009383
rs4292120	MYEOV2	2	240714953	6	0.12659
rs4292120	MYEOV2	2	240714953	4	0.708267
rs4292120	MYEOV2	2	240714953	1	0.846511
rs4292120	MYEOV2	2	240714953	7	0.938933
rs1126920	GPC1	2	241054152	1	4.3634
SNP2-241054156	GPC1	2	241054156	7	0.723945
rs2228331	GPC1	2	241054201	5	0.437016
rs2228331	GPC1	2	241054201	4	0.159958

rs2228331	GPC1	2	241054201	2	12.32275
rs13013933	GPC1	2	241055203	2	3.743162
rs3792215	GPC1	2	241055322	2	2.07678
rs3792215	GPC1	2	241055322	5	0.143618
rs3792215	GPC1	2	241055322	4	3.503866
rs3792215	GPC1	2	241055322	8	0.030521
rs3792214	GPC1	2	241055374	4	0.604363
rs3792214	GPC1	2	241055374	5	0.274041
rs3792214	GPC1	2	241055374	8	0.357875
SNP2-241112268	ANKMY1	2	241112268	3	0.41096
rs3792269	CAPN10	2	241180152	7	0.421917
rs3792269	CAPN10	2	241180152	6	0.371261
rs3792269	CAPN10	2	241180152	1	4.096824
rs3749166	CAPN10	2	241186094	6	7.126569
rs3749172	GPR35	2	241218922	6	1.674213
rs3749172	GPR35	2	241218922	1	0.779892
rs3749172	GPR35	2	241218922	3	1.444349
rs4613	KIF1A	2	241301960	3	0.354812
rs4613	KIF1A	2	241301960	1	0.417828
rs34116584	AGXT	2	241456987	7	0.094032
rs4426527	AGXT	2	241466189	7	1.118155
rs13397515	C2orf54	2	241474723	6	5.729426
rs13397515	C2orf54	2	241474723	5	0.333815
rs13397515	C2orf54	2	241474723	3	0.744161
rs13397515	C2orf54	2	241474723	8	1.277213
rs13397515	C2orf54	2	241474723	1	4.180277
rs13422330	C2orf54	2	241474812	6	0.461016
rs13422330	C2orf54	2	241474812	8	3.666677
rs13422330	C2orf54	2	241474812	3	0.00917
rs13422330	C2orf54	2	241474812	4	1.319148
rs13422330	C2orf54	2	241474812	1	2.522851
rs13422330	C2orf54	2	241474812	5	0.235829
SNP2-241476471	C2orf54	2	241476471	8	0.586168
SNP2-241476471	C2orf54	2	241476471	4	2.319548
rs10193144	LOC728763	2	241521395	7	1.120574
rs17440466	SNED1	2	241659757	1	1.687383
rs17440466	SNED1	2	241659757	3	0.41513
rs2108485	SNED1	2	241670415	1	6.406624
rs4035	MTERFD2	2	241675281	1	3.622922
rs2067987	MTERFD2	2	241676599	3	0.281533
rs10933529	MTERFD2	2	241676955	1	5.140904
SNP2-241678099	MTERFD2	2	241678099	1	6.920903
rs16843241	SNED1	2	241679763	1	5.212872
rs16843242	SNED1	2	241679954	1	4.605184
rs16843242	SNED1	2	241679954	3	0.457575
rs3815291	SNED1	2	241681213	1	0.53913
rs3815291	SNED1	2	241681213	5	0.295459

rs2302047	MTERFD2	2	241682443	5	4.948431
rs2302047	MTERFD2	2	241682443	6	4.997862
rs2074824	PASK	2	241700450	1	3.428537
rs2074824	PASK	2	241700450	3	0.30543
SNP2-241714617	PASK	2	241714617	3	0.623607
rs2240542	PASK	2	241714987	5	0.34625
rs2240542	PASK	2	241714987	3	0.548653
rs2240544	PASK	2	241715503	3	0.301515
rs1470414	PASK	2	241726169	1	0.991883
rs6709462	PASK	2	241730935	8	1.674848
rs6709462	PASK	2	241730935	2	1.065128
rs6709462	PASK	2	241730935	1	0.382803
rs6709462	PASK	2	241730935	4	0.383887
rs3806599	LOC644302	2	241737594	1	3.314953
rs7593101	TMEM16G	2	241800258	5	1.129917
rs7593101	TMEM16G	2	241800258	1	0.066584
rs7590653	TMEM16G	2	241812032	5	0.297599
rs7590653	TMEM16G	2	241812032	1	4.260827
SNP2-242056261	FARP2	2	242056261	4	0.54682
SNP2-242056261	FARP2	2	242056261	7	0.386424
SNP2-242056261	FARP2	2	242056261	5	0.533381
rs17453555	LOC728249	2	242066481	2	0.376463
rs17453555	LOC728249	2	242066481	5	0.302571
rs17453555	LOC728249	2	242066481	1	0.523537
SNP2-242261762	ATG4B	2	242261762	6	6.761596
SNP2-242261762	ATG4B	2	242261762	7	0.33275
SNP2-242261762	ATG4B	2	242261762	8	0.57607
rs7370843	D2HGDH	2	242322777	3	1.078681
rs7370843	D2HGDH	2	242322777	1	0.204092
rs7370843	D2HGDH	2	242322777	8	0.382506
SNP2-242339348	D2HGDH	2	242339348	7	1.523749
SNP2-242339348	D2HGDH	2	242339348	3	2.050093
SNP2-242406493	NEU4	2	242406493	6	1.826193
SNP2-242406493	NEU4	2	242406493	2	1.841578
SNP2-242462682	FLJ33590	2	242462682	5	0.086118
SNP2-242463033	FLJ33590	2	242463033	4	2.570156
SNP2-242463033	FLJ33590	2	242463033	7	2.261749
SNP2-242463033	FLJ33590	2	242463033	6	0.465672
rs2272522	CHL1	3	336508	7	0.300688
rs2272522	CHL1	3	336508	5	0.231497
rs7431184	LOC727810	3	1922477	5	0.979757
rs7629924	CNTN4	3	3042850	1	1.951142
rs7629924	CNTN4	3	3042850	8	0.913452
rs6790908	CNTN4	3	3051401	1	0.497221
rs6790908	CNTN4	3	3051401	5	0.988188
rs6790908	CNTN4	3	3051401	8	0.611375
rs6803232	CNTN4	3	3051443	5	1.681005

rs6803232	CNTN4	3	3051443	8	1.269548
rs6802588	CNTN4	3	3056750	5	0.423725
rs6802588	CNTN4	3	3056750	3	0.512915
rs340833	IL5RA	3	3086458	3	1.219311
rs14275	SUMF1	3	4378153	1	0.194278
rs14275	SUMF1	3	4378153	2	4.662865
rs14275	SUMF1	3	4378153	3	0.072221
rs14275	SUMF1	3	4378153	7	0.028509
rs901854	ITPR1	3	4831234	3	0.350125
rs901854	ITPR1	3	4831234	4	0.939777
rs3749380	GRM7	3	6878297	4	1.40861
rs3749380	GRM7	3	6878297	3	0.724986
rs7614915	GRM7	3	7595382	4	0.488741
rs7614915	GRM7	3	7595382	3	0.654381
rs7614915	GRM7	3	7595382	5	1.056386
rs1485175	GRM7	3	7595789	6	0.541172
rs1485174	GRM7	3	7595828	5	1.678254
rs1485174	GRM7	3	7595828	6	1.718697
rs1485174	GRM7	3	7595828	3	1.666349
rs2276800	C3orf32	3	8650539	5	1.585255
rs11476	CAV3	3	8763198	5	0.62208
rs2324728	OXTR	3	8767728	1	0.329389
rs2324728	OXTR	3	8767728	3	0.587617
rs2324728	OXTR	3	8767728	6	0.419991
rs9872310	OXTR	3	8768381	3	1.09122
rs9872310	OXTR	3	8768381	6	0.001323
rs6770632	OXTR	3	8768724	2	2.02348
rs6770632	OXTR	3	8768724	1	0.906571
rs6770632	OXTR	3	8768724	6	0.741615
rs6770632	OXTR	3	8768724	3	0.122288
rs1042778	OXTR	3	8769545	6	2.439414
rs1042778	OXTR	3	8769545	7	0.250476
rs1042778	OXTR	3	8769545	1	1.182264
rs1042778	OXTR	3	8769545	5	0.350952
rs237902	OXTR	3	8784184	8	1.442956
rs237902	OXTR	3	8784184	3	1.783844
rs237902	OXTR	3	8784184	7	1.687751
rs237902	OXTR	3	8784184	2	1.107689
rs237902	OXTR	3	8784184	1	2.099548
SNP3-8784222	OXTR	3	8784222	4	2.380825
SNP3-8784222	OXTR	3	8784222	2	3.250893
SNP3-8784222	OXTR	3	8784222	1	2.909202
rs2258767	THUMPD3	3	9379887	8	6.287098
rs1129174	THUMPD3	3	9401224	6	1.303491
rs1129174	THUMPD3	3	9401224	5	0.330409
rs2596912	LOC440944	3	9413746	5	1.064739
rs2596912	LOC440944	3	9413746	8	0.022475

rs2596912	LOC440944	3	9413746	6	0.329577
rs2596912	LOC440944	3	9413746	7	0.130485
rs2596912	LOC440944	3	9413746	3	0.980896
SNP3-9492369	SETD5	3	9492369	3	0.158855
rs2648593	LHFPL4	3	9570374	5	3.158932
rs17222536	CIDEC	3	9883935	1	4.213477
SNP3-9909753	JAGN1	3	9909753	5	0.617578
SNP3-9909753	JAGN1	3	9909753	3	0.008997
SNP3-9923059	IL17RE	3	9923059	7	0.789706
rs3774207	CRELD1	3	9960656	4	8.610418
rs3774207	CRELD1	3	9960656	1	4.036877
SNP3-9965800	PRRT3	3	9965800	4	0.149258
SNP3-9965800	PRRT3	3	9965800	1	3.065895
SNP3-9966101	PRRT3	3	9966101	4	1.665229
SNP3-9966101	PRRT3	3	9966101	1	3.382405
rs7626117	FANCD2	3	10115671	1	5.26483
rs7626117	FANCD2	3	10115671	4	3.868279
rs7647987	FANCD2	3	10115696	1	5.73949
rs7647987	FANCD2	3	10115696	4	3.545545
rs3172417	FANCD2	3	10117949	8	3.317715
rs3172417	FANCD2	3	10117949	2	1.376735
rs3172417	FANCD2	3	10117949	1	0.756116
rs7645667	C3orf10	3	10132337	4	0.251364
rs2075352	TATDN2	3	10286939	4	8.838734
rs1063429	TATDN2	3	10295968	7	0.866666
rs1063429	TATDN2	3	10295968	5	3.784286
rs514658	TATDN2	3	10296734	5	0.212453
rs41147	ATP2B2	3	10343102	8	2.379339
rs41147	ATP2B2	3	10343102	4	0.44601
rs41147	ATP2B2	3	10343102	5	0.575361
rs41147	ATP2B2	3	10343102	1	2.96032
rs14154	ATP2B2	3	10343113	5	2.592545
rs14154	ATP2B2	3	10343113	8	1.363291
rs14154	ATP2B2	3	10343113	1	4.058523
rs14154	ATP2B2	3	10343113	4	2.094176
rs35674	ATP2B2	3	10343577	1	0.7566
rs35674	ATP2B2	3	10343577	4	0.635261
rs35674	ATP2B2	3	10343577	5	1.184632
rs35674	ATP2B2	3	10343577	2	1.104599
rs1719571	ATP2B2	3	10344180	8	1.280713
rs2289274	ATP2B2	3	10388715	1	1.955271
rs2289274	ATP2B2	3	10388715	4	1.291039
rs2289274	ATP2B2	3	10388715	8	0.188247
rs901865	HRH1	3	11275707	4	0.645925
rs901865	HRH1	3	11275707	3	0.861655
rs2067466	HRH1	3	11275780	3	0.120612
rs1809049	HRH1	3	11277884	5	0.856134

rs346070	HRH1	3	11278874	3	0.56989
rs346070	HRH1	3	11278874	4	1.260834
SNP3-11375019	ATG7	3	11375019	7	0.323853
SNP3-11375019	ATG7	3	11375019	1	3.574547
rs1046593	ATG7	3	11571402	4	5.91928
rs408600	C3orf31	3	11862991	7	0.499828
rs408600	C3orf31	3	11862991	5	2.086829
rs408600	C3orf31	3	11862991	4	0.208925
rs2289708	SYN2	3	12200353	3	0.199484
rs709161	TSEN2	3	12501188	4	1.660402
rs709161	TSEN2	3	12501188	2	3.986156
rs709161	TSEN2	3	12501188	8	3.632113
rs709161	TSEN2	3	12501188	5	5.010625
SNP3-12519829	TSEN2	3	12519829	1	4.697334
rs2648307	MKRN2	3	12588866	4	0.342191
rs2648307	MKRN2	3	12588866	1	0.045764
rs2648307	MKRN2	3	12588866	5	0.210348
rs3773332	TMEM40	3	12775797	3	0.171563
rs2305398	CAND2	3	12831856	1	1.157453
rs2305398	CAND2	3	12831856	3	0.494043
rs4684883	CAND2	3	12836586	1	1.683086
rs3817121	CAND2	3	12836608	1	3.030381
rs1079361	CAND2	3	12850880	3	0.394885
rs1079361	CAND2	3	12850880	4	4.37164
rs2290311	RPL32	3	12858071	3	0.133767
rs2290311	RPL32	3	12858071	4	3.99359
rs2290311	RPL32	3	12858071	8	1.355064
rs665064	NUP210	3	13342406	3	0.035996
rs665064	NUP210	3	13342406	5	1.320736
rs665064	NUP210	3	13342406	4	1.476017
rs665064	NUP210	3	13342406	8	3.782567
rs3773302	NUP210	3	13354400	3	0.640629
rs3773302	NUP210	3	13354400	4	0.35937
rs3773302	NUP210	3	13354400	5	0.093491
rs2280085	NUP210	3	13370475	4	0.203621
SNP3-13374786	NUP210	3	13374786	7	3.460049
SNP3-13374786	NUP210	3	13374786	5	8.659607
SNP3-13374786	NUP210	3	13374786	3	0.021336
rs3732666	FBLN2	3	13587937	7	0.222418
rs3732666	FBLN2	3	13587937	1	1.522704
rs9843344	FBLN2	3	13645537	4	0.134919
rs9843344	FBLN2	3	13645537	3	0.489279
rs9843344	FBLN2	3	13645537	5	0.237017
rs4684968	FBLN2	3	13645777	5	4.701262
rs4684968	FBLN2	3	13645777	3	0.227934
rs4684968	FBLN2	3	13645777	2	4.401241
rs4684968	FBLN2	3	13645777	4	0.606397

rs1061376	FBLN2	3	13654336	7	0.392024
rs1061376	FBLN2	3	13654336	1	0.657678
rs1061376	FBLN2	3	13654336	5	1.16192
rs4685076	TMEM43	3	14149428	3	0.061907
rs4685076	TMEM43	3	14149428	7	0.040095
rs4685076	TMEM43	3	14149428	2	1.208154
rs2341983	SLC6A6	3	14502943	1	0.559226
rs2341983	SLC6A6	3	14502943	2	7.431448
rs2341983	SLC6A6	3	14502943	7	0.365941
rs11713359	SLC6A6	3	14503464	7	0.004633
rs11713359	SLC6A6	3	14503464	1	0.456819
rs4685171	GRIP2	3	14530220	3	0.616854
rs11717411	C3orf19	3	14688125	7	0.710499
rs11717411	C3orf19	3	14688125	6	0.178537
rs11717411	C3orf19	3	14688125	4	0.213992
rs9821143	C3orf20	3	14699417	7	2.406513
rs34230332	C3orf20	3	14700882	4	2.363243
rs6765537	C3orf20	3	14730576	8	1.384221
rs6765537	C3orf20	3	14730576	2	0.614621
rs6765537	C3orf20	3	14730576	1	1.104981
rs6765537	C3orf20	3	14730576	7	1.261006
rs6765537	C3orf20	3	14730576	5	1.446296
rs6790129	C3orf20	3	14730621	5	0.014392
rs6790129	C3orf20	3	14730621	7	0.084732
rs6790129	C3orf20	3	14730621	1	0.129884
rs6790129	C3orf20	3	14730621	2	0.539837
rs6790129	C3orf20	3	14730621	8	0.891515
rs2164356	FGD5	3	14914483	3	1.268216
rs17536979	NR2C2	3	15055700	7	0.346723
rs4685240	LOC344875	3	15178176	7	1.108846
rs4685240	LOC344875	3	15178176	5	0.587069
rs11705926	LOC344875	3	15181721	3	0.068744
rs7639618	LOC344875	3	15191433	4	0.738431
rs1563415	EAF1	3	15451015	3	0.577494
rs1563415	EAF1	3	15451015	4	1.687584
rs1563415	EAF1	3	15451015	1	3.328384
rs1563415	EAF1	3	15451015	5	5.716446
rs17041129	EAF1	3	15455730	7	0.188132
rs6778562	EAF1	3	15457779	1	5.561567
rs6778562	EAF1	3	15457779	6	6.874013
rs3274	COLQ	3	15466760	6	0.62461
rs3274	COLQ	3	15466760	4	1.003944
rs10154896	COLQ	3	15467640	1	5.607007
rs983600	ANKRD28	3	15684144	7	5.516518
rs2271077	GALNTL2	3	16236424	3	0.456221
rs15767	GALNTL2	3	16243978	5	0.236153
rs15767	GALNTL2	3	16243978	3	0.706483

rs15767	GALNTL2	3	16243978	1	0.191511
rs14210	GALNTL2	3	16244070	1	0.901257
rs14210	GALNTL2	3	16244070	6	0.186881
rs14210	GALNTL2	3	16244070	2	2.624763
rs14210	GALNTL2	3	16244070	5	1.152211
rs6777976	OXNAD1	3	16302913	1	6.460334
rs6777976	OXNAD1	3	16302913	6	0.455765
rs842424	RFTN1	3	16332982	2	3.654417
rs842424	RFTN1	3	16332982	1	0.376454
rs842424	RFTN1	3	16332982	6	0.251018
SNP3-16339958	RFTN1	3	16339958	1	1.309311
SNP3-16339958	RFTN1	3	16339958	2	3.442817
SNP3-16339958	RFTN1	3	16339958	6	3.304168
rs7653834	PLCL2	3	17026332	7	1.287412
rs7653834	PLCL2	3	17026332	3	0.239317
rs11549706	PLCL2	3	17106505	1	4.045006
rs11549706	PLCL2	3	17106505	3	1.144135
rs11549706	PLCL2	3	17106505	8	0.905469
rs1138454	TBC1D5	3	17183271	4	0.233073
rs1138454	TBC1D5	3	17183271	6	3.492804
rs1138454	TBC1D5	3	17183271	5	2.779921
rs1138454	TBC1D5	3	17183271	8	0.115994
SNP3-19934760	EFHB	3	19934760	3	2.139653
SNP3-19934760	EFHB	3	19934760	2	2.79087
SNP3-19934760	EFHB	3	19934760	5	0.451088
rs2929362	EFHB	3	19936335	1	3.337523
rs17180771	EFHB	3	19950143	3	0.75221
rs2929349	EFHB	3	19950586	2	5.744276
rs2929349	EFHB	3	19950586	5	0.173884
rs8682	RAB5A	3	20001413	6	0.694836
rs3021408	PCAF	3	20088834	6	3.433481
rs3021408	PCAF	3	20088834	7	2.148387
rs1052930	SGOL1	3	20187193	6	2.711068
rs1052930	SGOL1	3	20187193	7	0.601099
rs826371	THRB	3	24138649	4	0.149325
rs826371	THRB	3	24138649	5	0.209626
rs3752874	THRB	3	24159999	4	0.639704
rs3752874	THRB	3	24159999	6	0.534543
rs3752874	THRB	3	24159999	7	2.745291
rs11129270	NEK10	3	27232947	1	3.387799
rs3213930	NEK10	3	27272820	7	1.835605
rs3213930	NEK10	3	27272820	5	0.077506
rs11129280	NEK10	3	27301101	5	0.275432
rs11129280	NEK10	3	27301101	7	0.850548
rs11129280	NEK10	3	27301101	1	3.773762
rs11129280	NEK10	3	27301101	3	1.496449
rs10510594	NEK10	3	27301455	5	0.327307

rs10510594	NEK10	3	27301455	3	0.841063
rs10510594	NEK10	3	27301455	1	4.373832
rs10510594	NEK10	3	27301455	7	0.859869
rs10510592	NEK10	3	27307824	5	0.686554
rs10510592	NEK10	3	27307824	7	0.891465
rs10510592	NEK10	3	27307824	1	4.939835
rs10510592	NEK10	3	27307824	3	0.410863
rs3736312	SLC4A7	3	27417340	5	6.902005
rs3755652	SLC4A7	3	27447940	5	0.295613
rs1563656	ZCWPW2	3	28495421	8	0.271918
rs1563656	ZCWPW2	3	28495421	3	0.523821
rs13100556	ZCWPW2	3	28508662	1	4.758272
rs3816573	ZCWPW2	3	28541480	3	0.289088
rs3816573	ZCWPW2	3	28541480	8	1.130836
rs6791484	LOC339897	3	31198053	5	1.425675
rs17027217	LOC339897	3	31198317	1	2.064355
rs17027217	LOC339897	3	31198317	2	2.399617
rs17027217	LOC339897	3	31198317	6	1.0771
rs17027957	OSBPL10	3	31700482	5	0.630785
rs11129475	LOC728665	3	32005548	3	0.164507
rs12497202	KRT18P15	3	32275640	7	1.825153
rs12497202	KRT18P15	3	32275640	2	0.412597
rs12497202	KRT18P15	3	32275640	4	0.699994
rs12497202	KRT18P15	3	32275640	1	0.03257
rs6550143	LOC391526	3	32637535	7	5.214564
rs6550143	LOC391526	3	32637535	6	1.621899
rs11711396	FLJ45032	3	33107760	2	6.240029
rs17192791	FBXL2	3	33381129	5	7.684918
SNP3-33854774	PDCD6IP	3	33854774	1	5.669405
SNP3-33854774	PDCD6IP	3	33854774	4	0.151991
SNP3-33854774	PDCD6IP	3	33854774	5	5.246933
SNP3-33854774	PDCD6IP	3	33854774	2	0.093819
SNP3-33854774	PDCD6IP	3	33854774	7	0.283897
rs9813017	PDCD6IP	3	33868991	1	3.950037
rs9813017	PDCD6IP	3	33868991	4	0.933046
rs1127732	PDCD6IP	3	33880570	5	0.84443
rs1127732	PDCD6IP	3	33880570	2	4.49291
rs9860326	NULL	3	35658108	3	0.295144
rs3821447	ARPP-21	3	35701752	3	0.134739
rs45549732	STAC	3	36502724	4	0.585643
rs2176995	STAC	3	36563891	3	0.498545
rs2176995	STAC	3	36563891	5	0.612279
rs2176995	STAC	3	36563891	1	6.117273
rs9311135	LOC645548	3	36784123	3	0.560204
rs1799977	MLH1	3	37028572	3	1.16178
rs1799977	MLH1	3	37028572	1	4.323227
rs1799977	MLH1	3	37028572	6	1.542113

rs1799977	MLH1	3	37028572	5	0.686655
rs1799977	MLH1	3	37028572	4	2.489652
rs12630114	DLEC1	3	38126735	3	0.328607
rs12630114	DLEC1	3	38126735	1	1.961638
rs12630114	DLEC1	3	38126735	8	1.653639
rs12630114	DLEC1	3	38126735	4	1.230824
SNP3-38142099	ACAA1	3	38142099	4	0.782912
rs156265	ACAA1	3	38145814	4	3.996853
rs156265	ACAA1	3	38145814	3	0.692317
rs156265	ACAA1	3	38145814	1	0.307789
rs2073714	SLC22A14	3	38322855	8	0.308864
rs818819	SLC22A14	3	38325483	8	1.614816
rs818818	SLC22A14	3	38325523	8	0.448198
rs818817	SLC22A14	3	38325547	8	0.972975
rs240033	SLC22A14	3	38332965	5	0.370577
rs1805124	SCN5A	3	38620424	1	0.948835
SNP3-38743304	SCN10A	3	38743304	1	1.541934
rs17852414	WDR48	3	39110555	8	0.580835
rs17852414	WDR48	3	39110555	6	2.525643
rs1112438	TTC21A	3	39127349	5	2.326121
rs1112438	TTC21A	3	39127349	3	1.407271
rs1112438	TTC21A	3	39127349	6	0.985552
rs1112438	TTC21A	3	39127349	8	2.155894
rs1274972	TTC21A	3	39136460	3	0.635742
rs1274972	TTC21A	3	39136460	6	2.474889
rs1274972	TTC21A	3	39136460	8	6.292764
rs1274972	TTC21A	3	39136460	5	4.146414
rs1274971	TTC21A	3	39136468	5	0.114773
rs1274971	TTC21A	3	39136468	3	1.027529
rs1274971	TTC21A	3	39136468	6	3.592647
rs1274971	TTC21A	3	39136468	8	5.295496
rs1274957	AXUD1	3	39160081	3	0.757294
rs1274957	AXUD1	3	39160081	6	0.691793
rs1274957	AXUD1	3	39160081	5	5.824992
rs1274957	AXUD1	3	39160081	8	6.209322
rs784517	AXUD1	3	39161732	6	1.839398
rs784517	AXUD1	3	39161732	5	3.216544
rs784517	AXUD1	3	39161732	3	0.707935
rs11710546	CX3CR1	3	39281223	6	0.541053
rs11710546	CX3CR1	3	39281223	1	0.33528
rs3732378	CX3CR1	3	39282166	6	0.325343
rs3732379	CX3CR1	3	39282260	6	0.693123
rs3732379	CX3CR1	3	39282260	1	0.358144
rs3772146	RPSA	3	39423208	3	1.139259
rs3772146	RPSA	3	39423208	8	0.242184
rs3772146	RPSA	3	39423208	1	0.055733
rs3772146	RPSA	3	39423208	2	0.364636

rs1317317	MYRIP	3	40206887	7	0.620595
rs4618168	MYRIP	3	40261021	2	0.63959
rs4618168	MYRIP	3	40261021	7	0.805542
rs4618168	MYRIP	3	40261021	1	0.432006
rs13081294	MYRIP	3	40276277	1	0.686478
rs1047855	ENTPD3	3	40443900	1	0.548049
rs1047855	ENTPD3	3	40443900	2	3.334601
rs4602359	ZNF621	3	40554361	1	0.290677
rs4602359	ZNF621	3	40554361	6	2.130739
SNP3-41731990	ULK4	3	41731990	7	5.580559
SNP3-41731990	ULK4	3	41731990	3	0.530356
rs1716975	ULK4	3	41935010	7	1.065487
rs1716975	ULK4	3	41935010	3	0.860583
rs2272007	ULK4	3	41971140	3	0.281926
rs7372751	TRAK1	3	42227928	6	0.312319
rs7372751	TRAK1	3	42227928	8	7.568364
rs7372751	TRAK1	3	42227928	1	5.272885
rs7372751	TRAK1	3	42227928	3	0.036346
rs7372751	TRAK1	3	42227928	4	0.057134
SNP3-42654781	NKTR	3	42654781	8	3.295303
rs2290976	ZBTB47	3	42683636	8	2.515534
rs123509	KBTBD5	3	42708472	5	0.751022
rs123509	KBTBD5	3	42708472	1	1.168205
rs123509	KBTBD5	3	42708472	7	0.113584
rs123509	KBTBD5	3	42708472	3	0.458841
rs3846062	KBTBD5	3	42708671	3	0.104571
rs3846062	KBTBD5	3	42708671	1	0.390653
rs3846062	KBTBD5	3	42708671	5	1.353915
SNP3-42710154	HHATL	3	42710154	1	1.177426
SNP3-42710154	HHATL	3	42710154	5	1.485252
SNP3-42710154	HHATL	3	42710154	3	0.617826
SNP3-42710154	HHATL	3	42710154	6	0.109331
rs339697	HHATL	3	42710278	5	4.000796
rs17238798	CCDC13	3	42774769	7	0.905137
rs17238798	CCDC13	3	42774769	2	0.881605
rs17238798	CCDC13	3	42774769	6	4.747493
rs17238798	CCDC13	3	42774769	1	0.776267
rs2228468	CCBP2	3	42882116	3	0.892375
rs2228468	CCBP2	3	42882116	5	2.305637
rs2228468	CCBP2	3	42882116	8	3.508767
rs2228468	CCBP2	3	42882116	7	1.520006
rs1366046	CCBP2	3	42883779	5	2.808191
rs1366046	CCBP2	3	42883779	3	0.959637
rs1366046	CCBP2	3	42883779	7	1.397572
rs1366046	CCBP2	3	42883779	2	3.592435
rs1366046	CCBP2	3	42883779	6	0.648293
rs1366046	CCBP2	3	42883779	8	0.079085

rs12494055	CYP8B1	3	42888849	3	0.138312
rs3732860	CYP8B1	3	42889016	3	1.876538
rs3732860	CYP8B1	3	42889016	6	1.906486
rs3732860	CYP8B1	3	42889016	8	1.10116
rs1427801	ZNF662	3	42931277	4	3.572733
rs834579	ZNF662	3	42931988	5	0.635765
rs834579	ZNF662	3	42931988	2	4.183078
rs2948619	LOC729085	3	43049434	6	0.885241
rs2948619	LOC729085	3	43049434	5	3.555872
rs2948619	LOC729085	3	43049434	8	2.410478
rs2948619	LOC729085	3	43049434	1	0.114893
rs2936817	LOC729085	3	43049438	5	1.494641
rs2936817	LOC729085	3	43049438	8	0.01212
rs2936817	LOC729085	3	43049438	6	1.30083
rs536119	LOC729085	3	43070105	4	0.449925
rs3732858	LOC729085	3	43072769	1	5.966705
rs17409162	TMEM16K	3	43566331	4	0.766736
SNP3-43593284	TMEM16K	3	43593284	4	3.88387
rs9833423	FLJ36157	3	44258676	3	2.410601
rs9833423	FLJ36157	3	44258676	1	2.942045
rs7645375	FLJ36157	3	44258812	1	1.297042
rs11130025	C3orf23	3	44355212	8	4.624432
rs11130025	C3orf23	3	44355212	4	4.20726
rs11130025	C3orf23	3	44355212	1	1.930563
GA003188	C3orf23	3	44376259	3	0.758601
GA003188	C3orf23	3	44376259	4	0.290064
GA003188	C3orf23	3	44376259	1	5.457149
rs4610267	ZNF445	3	44460918	3	0.548822
rs4610267	ZNF445	3	44460918	2	0.23895
rs4610267	ZNF445	3	44460918	8	0.767207
rs4610267	ZNF445	3	44460918	5	3.447105
rs4610267	ZNF445	3	44460918	1	0.526607
rs4610267	ZNF445	3	44460918	4	0.307328
rs6441857	ZNF167	3	44571900	7	0.867578
rs6441857	ZNF167	3	44571900	3	0.033686
rs6441857	ZNF167	3	44571900	4	0.023456
rs6441857	ZNF167	3	44571900	1	0.956943
rs6441857	ZNF167	3	44571900	8	0.381298
rs2034476	ZNF167	3	44587899	4	2.668892
rs2034476	ZNF167	3	44587899	1	0.029829
rs2034476	ZNF167	3	44587899	3	0.682267
rs2034476	ZNF167	3	44587899	2	5.580068
rs2034476	ZNF167	3	44587899	7	0.390089
rs13100959	ZNF197	3	44641610	2	2.397785
rs13100959	ZNF197	3	44641610	7	1.035894
rs13100959	ZNF197	3	44641610	3	0.706876
rs13100959	ZNF197	3	44641610	8	4.909032

rs13100959	ZNF197	3	44641610	1	0.932862
rs13100959	ZNF197	3	44641610	4	0.424747
rs17376517	ZNF502	3	44740225	8	0.202663
rs4682752	ZNF501	3	44750966	4	6.369764
rs4682752	ZNF501	3	44750966	1	0.474695
rs4682752	ZNF501	3	44750966	7	0.619904
rs2271087	TGM4	3	44904291	1	3.487788
rs2271087	TGM4	3	44904291	5	3.454024
rs2271087	TGM4	3	44904291	7	3.963967
SNP3-44923594	TGM4	3	44923594	2	1.288632
SNP3-44923594	TGM4	3	44923594	4	0.647214
SNP3-44923594	TGM4	3	44923594	1	0.630967
SNP3-44923594	TGM4	3	44923594	5	0.189595
rs1395388	TGM4	3	44923678	4	0.062952
rs13963	CLEC3B	3	45052127	2	1.881569
rs13963	CLEC3B	3	45052127	6	0.763822
rs13963	CLEC3B	3	45052127	1	1.307658
rs13963	CLEC3B	3	45052127	4	0.857478
rs13963	CLEC3B	3	45052127	5	0.997052
rs11063	CDCP1	3	45099173	8	5.229958
SNP3-45109826	CDCP1	3	45109826	1	0.104535
rs33749	TMEM158	3	45241559	8	5.76793
rs28413188	LARS2	3	45405155	6	1.024002
rs267237	LIMD1	3	45612257	2	1.039569
rs267237	LIMD1	3	45612257	4	1.117585
rs267237	LIMD1	3	45612257	1	0.067694
rs267237	LIMD1	3	45612257	7	0.411908
rs267237	LIMD1	3	45612257	6	0.457935
rs267237	LIMD1	3	45612257	5	6.745499
rs2251109	SLC6A20	3	45771955	5	0.456105
rs2251109	SLC6A20	3	45771955	1	2.181145
rs17213127	SLC6A20	3	45773230	1	4.820834
rs1994491	FYCO1	3	45935424	6	0.557272
rs3796373	FYCO1	3	45935855	8	7.104936
rs3796373	FYCO1	3	45935855	5	6.025072
rs7652331	FYCO1	3	45937756	4	0.72354
rs7652331	FYCO1	3	45937756	6	6.777534
rs6441934	FYCO1	3	45937806	4	6.446305
rs13079869	FYCO1	3	45983091	6	0.359992
rs3796375	FYCO1	3	45983794	5	0.20411
rs3796375	FYCO1	3	45983794	8	2.592859
rs3796375	FYCO1	3	45983794	6	0.036458
rs3796375	FYCO1	3	45983794	4	0.649119
rs3796375	FYCO1	3	45983794	2	2.840836
SNP3-45984491	FYCO1	3	45984491	6	2.464841
rs4682801	FYCO1	3	45996222	4	0.337686
rs4682801	FYCO1	3	45996222	7	0.737668

rs3136667	CCR1	3	46219288	1	1.188906
rs2037391	RTP3	3	46514527	3	0.756549
SNP3-46561626	LRRC2	3	46561626	1	1.877183
SNP3-46595764	TDGF1	3	46595764	8	6.318432
SNP3-46595764	TDGF1	3	46595764	3	2.714701
SNP3-46595764	TDGF1	3	46595764	5	2.304659
SNP3-46595764	TDGF1	3	46595764	1	1.702727
SNP3-46595764	TDGF1	3	46595764	6	2.486319
SNP3-46595764	TDGF1	3	46595764	7	0.635849
SNP3-46595764	TDGF1	3	46595764	2	1.063908
rs7625303	ALS2CL	3	46688461	3	0.149694
SNP3-46759471	TESSP5	3	46759471	7	0.863806
rs2305637	NBEAL2	3	47020850	7	0.164182
rs2305637	NBEAL2	3	47020850	4	0.398066
SNP3-47257307	KIF9	3	47257307	1	4.813742
SNP3-47257307	KIF9	3	47257307	3	0.955755
SNP3-47257307	KIF9	3	47257307	4	0.618274
SNP3-47257307	KIF9	3	47257307	5	6.359224
SNP3-47257307	KIF9	3	47257307	6	0.815934
SNP3-47257307	KIF9	3	47257307	8	5.071781
rs295458	KLHL18	3	47360589	6	0.194232
rs295458	KLHL18	3	47360589	1	5.359957
rs295458	KLHL18	3	47360589	3	1.040636
rs295458	KLHL18	3	47360589	4	0.449146
rs295458	KLHL18	3	47360589	8	5.349038
rs295458	KLHL18	3	47360589	5	3.505273
rs1531875	PTPN23	3	47427790	5	0.078818
rs1531875	PTPN23	3	47427790	1	4.413108
rs1531875	PTPN23	3	47427790	8	7.090398
rs1531875	PTPN23	3	47427790	3	0.122648
rs1531875	PTPN23	3	47427790	4	1.350295
rs1531875	PTPN23	3	47427790	6	0.582971
rs1875103	MAP4	3	47869105	4	0.023422
SNP3-47933041	MAP4	3	47933041	8	3.327444
rs9862913	MAP4	3	47991176	4	0.3292
rs11711953	MAP4	3	48015287	6	0.598888
rs11718329	ZNF589	3	48284832	6	1.685448
rs11718329	ZNF589	3	48284832	4	5.066583
rs11718350	SPINK8	3	48335996	4	1.78135
rs11718350	SPINK8	3	48335996	6	2.232299
rs12486046	TREX1	3	48482186	5	0.729121
rs2228561	COL7A1	3	48603018	3	1.089085
rs9834639	TMEM89	3	48634013	3	0.041358
rs13324142	SLC26A6	3	48644451	3	0.933038
rs6773261	CELSR3	3	48650068	3	4.786842
rs6773261	CELSR3	3	48650068	7	0.15014
rs3821875	CELSR3	3	48672658	3	0.011762

rs3087866	DALRD3	3	49029696	7	0.013972
rs3087866	DALRD3	3	49029696	5	0.051577
rs11552724	USP19	3	49131477	3	0.118443
rs4279134	USP19	3	49131680	6	0.699873
rs4279134	USP19	3	49131680	7	0.507077
rs4279134	USP19	3	49131680	3	0.60622
rs4279134	USP19	3	49131680	5	4.671595
rs34759087	LAMB2	3	49137288	7	0.349207
rs34759087	LAMB2	3	49137288	2	0.163154
rs1134043	CCDC71	3	49175141	3	0.043265
rs9837625	LOC646498	3	49190970	3	0.855689
rs12715434	CCDC36	3	49270029	3	0.619929
rs7626076	STGC3	3	49273143	5	0.301201
rs13077498	C3orf62	3	49288982	3	0.006426
rs3448	RHOA	3	49371755	5	0.373296
rs3448	RHOA	3	49371755	7	0.171372
rs3448	RHOA	3	49371755	6	0.201607
rs1464569	NICN1	3	49435354	7	1.15368
rs1464569	NICN1	3	49435354	6	1.624363
rs1464569	NICN1	3	49435354	5	0.865241
rs4855839	DAG1	3	49482851	3	0.699781
rs4855839	DAG1	3	49482851	5	0.176901
SNP3-49664214	BSN	3	49664214	6	1.031811
SNP3-49664214	BSN	3	49664214	1	0.821062
SNP3-49664214	BSN	3	49664214	7	0.937833
SNP3-49664214	BSN	3	49664214	3	0.723549
rs11709525	BSN	3	49665500	7	0.046471
rs11709525	BSN	3	49665500	3	0.706057
rs9858542	BSN	3	49676987	6	0.029886
rs9823546	BSN	3	49680516	4	0.279406
rs9823546	BSN	3	49680516	7	0.125759
rs9823546	BSN	3	49680516	3	0.039368
rs9823546	BSN	3	49680516	6	0.9949
rs9823546	BSN	3	49680516	1	1.941916
GA003289	BSN	3	49681742	3	0.903362
GA003289	BSN	3	49681742	1	2.967221
GA003289	BSN	3	49681742	5	0.530381
GA003289	BSN	3	49681742	7	0.823949
GA003289	BSN	3	49681742	4	0.487043
rs1060962	BSN	3	49683506	7	0.676182
rs1060962	BSN	3	49683506	1	3.119074
rs1060962	BSN	3	49683506	3	0.220629
rs1060962	BSN	3	49683506	5	0.104074
rs1060962	BSN	3	49683506	4	1.251421
rs1060970	BSN	3	49683773	1	0.082224
rs1060970	BSN	3	49683773	4	0.446979
rs1060970	BSN	3	49683773	7	0.471784

rs1131095	APEH	3	49689229	3	0.754605
rs1131095	APEH	3	49689229	6	1.087105
rs1131095	APEH	3	49689229	7	0.7282
rs1131095	APEH	3	49689229	1	1.599442
rs1131095	APEH	3	49689229	4	0.762273
SNP3-49724980	RNF123	3	49724980	3	0.271785
rs7628207	AMIGO3	3	49729974	5	0.216575
rs7628207	AMIGO3	3	49729974	6	0.636399
rs7629936	LOC389118	3	49811711	5	1.212298
rs6797500	CAMKV	3	49871622	3	0.042188
rs6797500	CAMKV	3	49871622	1	0.196285
rs2681781	CAMKV	3	49873277	3	0.866662
rs2681781	CAMKV	3	49873277	6	1.935131
rs1317215	LOC389120	3	49887295	3	0.734617
rs1317215	LOC389120	3	49887295	1	1.022402
rs2230590	MST1R	3	49911106	6	0.408194
rs2230590	MST1R	3	49911106	3	0.800135
rs868891	MON1A	3	49924075	1	4.444382
rs868891	MON1A	3	49924075	6	5.972142
rs2023953	RBM6	3	50078921	3	0.271226
rs1138536	RBM5	3	50128360	1	3.911243
rs12629572	C3orf45	3	50299676	7	0.437072
rs12629572	C3orf45	3	50299676	8	0.917398
rs1076872	IFRD2	3	50304830	6	0.431664
rs1076872	IFRD2	3	50304830	7	7.00606
rs1076872	IFRD2	3	50304830	3	0.815516
rs1076872	IFRD2	3	50304830	1	4.845392
rs1076872	IFRD2	3	50304830	4	1.104702
rs13100173	HYAL3	3	50307701	3	0.547651
rs13100173	HYAL3	3	50307701	1	0.932595
rs13100173	HYAL3	3	50307701	2	0.130676
rs13100173	HYAL3	3	50307701	4	0.369058
rs13100173	HYAL3	3	50307701	6	7.802753
rs13100173	HYAL3	3	50307701	8	9.61539
rs28365992	HYAL1	3	50316000	7	1.934665
rs4687569	RBM15B	3	51407935	6	5.729859
rs4687569	RBM15B	3	51407935	8	0.789416
rs28547534	PARP3	3	51953260	2	5.42173
rs11924827	PCBP4	3	51966655	5	5.261898
rs11924827	PCBP4	3	51966655	2	0.455395
rs11924827	PCBP4	3	51966655	1	0.038122
rs893056	WDR51A	3	52084604	6	0.06823
rs893056	WDR51A	3	52084604	7	0.038053
rs352140	TLR9	3	52231737	5	0.523694
rs352140	TLR9	3	52231737	1	1.105432
rs352143	TWF2	3	52239947	6	0.002515
rs352143	TWF2	3	52239947	3	0.679043

rs164637	TWF2	3	52240255	6	0.976941
SNP3-52365829	DNAH1	3	52365829	6	0.455309
rs11714402	DNAH1	3	52366775	3	1.03811
rs11714402	DNAH1	3	52366775	6	1.438003
rs419050	DNAH1	3	52401924	6	1.102475
rs11708581	DNAH1	3	52404028	3	1.306033
rs419752	DNAH1	3	52404705	6	0.958409
rs12163565	DNAH1	3	52405566	2	3.495168
rs12163565	DNAH1	3	52405566	3	0.621407
rs12163565	DNAH1	3	52405566	5	0.936758
rs13091025	SEMA3G	3	52442364	1	0.014062
rs1541495	NISCH	3	52467747	6	0.104772
rs1010553	STAB1	3	52515813	3	3.726261
rs9853056	STAB1	3	52530997	3	0.885537
rs9853056	STAB1	3	52530997	1	0.212558
rs7639267	NT5DC2	3	52543845	1	3.577575
rs7639267	NT5DC2	3	52543845	4	1.505673
rs7639267	NT5DC2	3	52543845	5	0.929178
rs7639267	NT5DC2	3	52543845	3	1.382197
rs2251219	PBRM1	3	52559827	4	5.025733
rs2251219	PBRM1	3	52559827	1	0.864538
rs2251219	PBRM1	3	52559827	5	1.054167
rs2251219	PBRM1	3	52559827	6	1.139796
rs2251219	PBRM1	3	52559827	2	4.797469
rs17264436	PBRM1	3	52585691	3	0.341168
rs1108842	GNL3	3	52695120	2	0.763327
rs1108842	GNL3	3	52695120	4	0.466987
rs1108842	GNL3	3	52695120	1	0.708093
rs1108842	GNL3	3	52695120	5	0.400453
rs6617	SPCS1	3	52715222	1	0.202517
rs6617	SPCS1	3	52715222	5	7.24275
rs6617	SPCS1	3	52715222	2	0.385017
rs6617	SPCS1	3	52715222	4	4.703485
rs6617	SPCS1	3	52715222	6	0.649538
rs1029871	NEK4	3	52772674	5	0.060412
rs1029871	NEK4	3	52772674	1	0.310961
rs1029871	NEK4	3	52772674	6	0.450023
rs1029871	NEK4	3	52772674	4	0.905726
rs1029871	NEK4	3	52772674	2	7.251347
SNP3-52796051	ITIH1	3	52796051	1	0.605298
rs4687657	ITIH4	3	52827578	4	0.593899
rs4687657	ITIH4	3	52827578	8	4.848384
rs4687657	ITIH4	3	52827578	1	0.622448
rs4687657	ITIH4	3	52827578	2	1.713511
rs4687657	ITIH4	3	52827578	5	0.082947
rs2276816	ITIH4	3	52835856	1	0.329256
rs2276816	ITIH4	3	52835856	8	0.12606

rs2276816	ITIH4	3	52835856	2	0.272168
rs2276817	ITIH4	3	52835976	4	0.675829
rs2276817	ITIH4	3	52835976	1	0.288475
rs2276817	ITIH4	3	52835976	5	0.423873
rs2276817	ITIH4	3	52835976	2	1.184637
rs2071042	ITIH4	3	52839624	2	2.505161
rs2071042	ITIH4	3	52839624	5	1.244429
rs2071042	ITIH4	3	52839624	4	1.310237
rs2071042	ITIH4	3	52839624	1	1.364699
rs2071041	ITIH4	3	52839733	1	0.264973
rs11626	TMEM110	3	52849024	6	0.08332
rs7645611	RFT1	3	53100337	6	5.665905
rs2230494	PRKCD	3	53195255	5	0.217768
rs312480	CACNA1D	3	53504180	6	0.834459
rs2250736	CACNA1D	3	53675590	3	0.032357
rs17053501	CACNA1D	3	53779529	2	1.604125
rs2241808	CHDH	3	53831693	6	1.062796
rs2241808	CHDH	3	53831693	1	0.505418
SNP3-53832957	CHDH	3	53832957	1	0.524538
rs709324	IL17RB	3	53874605	8	1.68528
rs709324	IL17RB	3	53874605	6	0.319648
rs709324	IL17RB	3	53874605	5	0.075278
rs709324	IL17RB	3	53874605	7	0.362776
rs709324	IL17RB	3	53874605	3	0.510043
rs3773603	CACNA2D3	3	54855501	2	1.038514
rs3773603	CACNA2D3	3	54855501	1	0.858264
rs3773603	CACNA2D3	3	54855501	8	0.151764
rs17054524	CACNA2D3	3	54905834	3	2.352934
rs17054524	CACNA2D3	3	54905834	4	1.07215
rs17054785	CACNA2D3	3	55083189	2	1.530513
rs1047898	WNT5A	3	55474821	6	0.187329
rs1047898	WNT5A	3	55474821	1	0.821265
rs1047898	WNT5A	3	55474821	5	0.621812
rs1047898	WNT5A	3	55474821	4	4.183815
rs1047898	WNT5A	3	55474821	7	0.631649
rs1047898	WNT5A	3	55474821	2	1.835723
SNP3-56305441	ERC2	3	56305441	7	0.261337
rs3732514	C3orf63	3	56642520	2	2.81578
rs9835332	C3orf63	3	56642722	3	0.72938
rs9835332	C3orf63	3	56642722	2	3.210669
rs9835332	C3orf63	3	56642722	1	0.249919
rs9835332	C3orf63	3	56642722	8	0.46984
rs6767903	C3orf63	3	56672907	6	0.234809
rs6794450	C3orf63	3	56673306	6	0.62338
rs3732511	ARHGEF3	3	56741475	6	0.354427
rs3732511	ARHGEF3	3	56741475	2	0.64877
rs1545981	IL17RD	3	57115012	8	9.332832

rs1545981	IL17RD	3	57115012	3	0.501342
rs4299455	IL17RD	3	57178438	7	4.387544
rs4299455	IL17RD	3	57178438	3	1.063511
rs4299455	IL17RD	3	57178438	1	1.462082
rs2241988	2'-PDE	3	57517213	4	0.552349
rs2241988	2'-PDE	3	57517213	3	0.426102
rs2241988	2'-PDE	3	57517213	5	0.086948
rs2241988	2'-PDE	3	57517213	2	3.183205
rs7618684	2'-PDE	3	57521033	4	0.445839
rs7618684	2'-PDE	3	57521033	2	0.393349
rs7618684	2'-PDE	3	57521033	5	0.091469
rs7618684	2'-PDE	3	57521033	8	2.559079
rs6762914	FAM116A	3	57587663	8	0.909441
rs6762914	FAM116A	3	57587663	3	1.301307
rs17058639	SLMAP	3	57857641	3	0.419498
rs17058639	SLMAP	3	57857641	1	3.102363
rs2362903	FLNB	3	58087480	5	0.599663
rs2362903	FLNB	3	58087480	2	2.16664
rs2362903	FLNB	3	58087480	4	1.459248
rs2362903	FLNB	3	58087480	6	0.661747
rs13079906	FLNB	3	58129217	8	0.280875
rs13079906	FLNB	3	58129217	4	4.947257
rs1131264	FLNB	3	58132559	7	6.989631
rs1131264	FLNB	3	58132559	5	0.277586
rs1131264	FLNB	3	58132559	6	0.816759
rs1131264	FLNB	3	58132559	8	0.566435
rs11706920	ABHD6	3	58254712	7	0.514742
rs11706920	ABHD6	3	58254712	2	3.533196
rs9986	ABHD6	3	58255446	1	5.023186
rs9986	ABHD6	3	58255446	8	4.710086
rs9986	ABHD6	3	58255446	3	0.397732
SNP3-58370903	PXK	3	58370903	1	4.058492
SNP3-58370903	PXK	3	58370903	7	0.558219
SNP3-58370903	PXK	3	58370903	4	0.802658
SNP3-58370903	PXK	3	58370903	6	6.341396
SNP3-58385594	PXK	3	58385594	1	6.141477
SNP3-58385594	PXK	3	58385594	7	0.492298
SNP3-58385594	PXK	3	58385594	6	4.754912
SNP3-58385594	PXK	3	58385594	4	0.886246
rs7231	PDHB	3	58388505	2	2.187605
rs7231	PDHB	3	58388505	3	0.041174
rs7231	PDHB	3	58388505	6	0.017247
rs7614260	FAM107A	3	58526604	7	3.196118
rs2306671	FAM107A	3	58538191	4	0.451687
rs2306671	FAM107A	3	58538191	3	0.350388
rs2306671	FAM107A	3	58538191	7	0.272018
rs2306671	FAM107A	3	58538191	5	0.203622

rs17255497	FHIT	3	59972152	1	4.825147
rs17255497	FHIT	3	59972152	6	0.070879
rs2292245	PTPRG	3	62164229	1	4.187956
rs1046025	PSMD6	3	63971302	8	6.391796
rs1046025	PSMD6	3	63971302	7	8.81691
rs1046025	PSMD6	3	63971302	1	3.474777
rs697287	PRICKLE2	3	64186142	7	0.367683
rs697287	PRICKLE2	3	64186142	2	1.821635
rs6771712	ADAMTS9	3	64562858	1	5.011162
rs6771712	ADAMTS9	3	64562858	7	1.42378
rs11708083	MAGI1	3	65320068	6	4.44397
rs13874	SLC25A26	3	66502646	4	2.266997
rs13874	SLC25A26	3	66502646	7	0.43472
rs13874	SLC25A26	3	66502646	6	0.720899
rs332373	LRIG1	3	66513877	4	0.336027
rs6549143	FAM19A4	3	68864903	1	0.677237
rs4855535	FAM19A4	3	69017124	1	1.317953
rs1532918	TMF1	3	69165398	4	0.757447
rs1532918	TMF1	3	69165398	3	0.128944
rs1532918	TMF1	3	69165398	5	1.089545
rs1532918	TMF1	3	69165398	6	0.249279
GA003445	TMF1	3	69184059	7	1.312276
rs10489	ARL6IP5	3	69233842	2	5.222224
rs4361282	FRMD4B	3	69381923	3	0.709626
rs1498845	LOC642487	3	69667543	4	4.402659
rs1498845	LOC642487	3	69667543	6	6.461906
rs6549270	LOC654340	3	70257951	6	4.124765
rs6549270	LOC654340	3	70257951	1	4.640845
rs6549270	LOC654340	3	70257951	7	4.523854
rs4594588	NULL	3	70746879	3	1.892958
rs17008544	FOXP1	3	71491009	8	1.961163
SNP3-72974237	SHQ1	3	72974237	7	1.071245
rs2291463	PDZRN3	3	73536015	7	0.893006
rs2291463	PDZRN3	3	73536015	5	0.61712
rs2291463	PDZRN3	3	73536015	3	1.11327
rs2291463	PDZRN3	3	73536015	6	1.156867
rs2291463	PDZRN3	3	73536015	8	0.084147
rs7623635	FAM86D	3	75559476	3	1.324148
SNP3-78767714	ROBO1	3	78767714	4	5.286971
rs2290081	GBE1	3	81893415	5	0.702752
rs35215849	LOC728144	3	86145880	3	2.165138
rs13074432	VGLL3	3	87072024	8	0.201297
rs13074432	VGLL3	3	87072024	4	1.123478
rs13074432	VGLL3	3	87072024	3	0.32903
rs13074432	VGLL3	3	87072024	1	1.188177
rs2279720	CHMP2B	3	87359389	4	0.515072
rs1051436	CGGBP1	3	88184220	2	3.670441

rs1398197	EPHA3	3	89581118	8	4.579704
SNP3-89604383	EPHA3	3	89604383	4	0.003424
SNP3-89604383	EPHA3	3	89604383	8	0.125806
rs1054750	EPHA3	3	89604415	4	0.93986
rs17855824	DHFRL1	3	95262550	1	5.335878
rs13316505	LOC644063	3	97552603	7	0.560951
rs13316505	LOC644063	3	97552603	4	0.630724
rs13316505	LOC644063	3	97552603	3	0.472731
rs4857294	ARL6	3	98999808	5	0.009726
rs9853906	OR5H6	3	99466632	3	1.849855
rs9822460	OR5K4	3	99556003	3	1.004157
rs13068323	OR5K3	3	99592330	7	0.884101
rs13068323	OR5K3	3	99592330	3	0.389089
SNP3-99790320	CPOX	3	99790320	3	0.224117
SNP3-99790320	CPOX	3	99790320	5	6.107597
SNP3-99790320	CPOX	3	99790320	4	7.428599
SNP3-99790320	CPOX	3	99790320	1	2.562432
rs16840059	LOC339843	3	99860047	7	3.329557
rs13080329	COL8A1	3	100946500	4	1.744588
rs13080329	COL8A1	3	100946500	6	1.210989
rs793440	FILIP1L	3	101125866	6	1.127402
rs793440	FILIP1L	3	101125866	7	1.701909
rs3796131	FILIP1L	3	101315599	6	0.577474
rs3796131	FILIP1L	3	101315599	4	0.212457
rs11537816	C3orf26	3	101369352	6	2.18915
rs11537816	C3orf26	3	101369352	7	1.228157
rs17213695	LOC391559	3	101427071	8	4.085075
rs17213695	LOC391559	3	101427071	5	4.089727
rs17213695	LOC391559	3	101427071	4	4.493928
rs7641299	LNP1	3	101653243	3	0.374322
rs7641299	LNP1	3	101653243	5	1.651097
rs7641299	LNP1	3	101653243	2	0.686253
rs1144122	GPR128	3	101837214	3	0.873233
rs1144122	GPR128	3	101837214	5	1.157826
rs1144122	GPR128	3	101837214	4	0.182715
rs1048364	ABI3BP	3	101951190	4	0.076648
rs1048364	ABI3BP	3	101951190	7	0.53165
rs10936352	ABI3BP	3	101956195	7	0.400267
rs10936352	ABI3BP	3	101956195	3	1.085717
rs10936352	ABI3BP	3	101956195	5	2.495293
rs2245370	ABI3BP	3	102100370	7	0.172237
rs2245370	ABI3BP	3	102100370	3	0.319392
rs2245370	ABI3BP	3	102100370	1	2.891508
rs3732895	ABI3BP	3	102194939	4	1.714965
rs348867	IMPG2	3	102432532	1	0.404683
rs348867	IMPG2	3	102432532	2	0.450312
rs348867	IMPG2	3	102432532	5	0.39192

rs348867	IMPG2	3	102432532	7	1.125138
rs348867	IMPG2	3	102432532	3	1.926516
rs571391	IMPG2	3	102445844	1	1.051096
rs571391	IMPG2	3	102445844	6	0.362151
rs571391	IMPG2	3	102445844	5	0.389872
rs571391	IMPG2	3	102445844	3	1.259148
rs571391	IMPG2	3	102445844	7	1.33419
SNP3-103023077	FAM55C	3	103023077	2	1.425046
SNP3-103023077	FAM55C	3	103023077	7	4.091045
rs6784389	ZPLD1	3	103640107	1	3.053873
rs17822848	ZPLD1	3	103680306	8	0.132765
rs17822848	ZPLD1	3	103680306	1	0.538385
rs17822848	ZPLD1	3	103680306	2	1.130239
rs17822848	ZPLD1	3	103680306	6	2.270466
rs709564	CCDC54	3	108579237	2	1.761487
rs709564	CCDC54	3	108579237	6	1.237381
rs709564	CCDC54	3	108579237	5	0.793397
rs17828926	IFT57	3	109363163	1	5.928838
rs16854512	HHLA2	3	109563937	1	0.640459
rs7635040	MYH15	3	109582550	5	2.224912
rs7635040	MYH15	3	109582550	3	0.711886
rs7635040	MYH15	3	109582550	7	0.513127
rs9873504	MYH15	3	109618370	7	4.331721
rs12638212	MYH15	3	109642667	7	0.461344
rs9868484	MYH15	3	109671683	5	1.576431
rs4299484	MYH15	3	109672317	3	0.084351
rs2278911	KIAA1524	3	109780950	5	1.219538
rs6804162	GUCA1C	3	110122074	5	0.993361
rs2715687	GUCA1C	3	110122113	1	0.42879
rs2290057	MORC1	3	110186277	5	1.851688
rs3762697	MORC1	3	110236904	1	2.701373
rs605795	LOC440973	3	111118349	1	3.873773
rs605795	LOC440973	3	111118349	3	3.137778
rs605795	LOC440973	3	111118349	8	3.079546
rs605795	LOC440973	3	111118349	2	1.536346
rs605795	LOC440973	3	111118349	7	2.335177
rs938213	ZBED2	3	112796544	1	2.310346
rs17423699	PLCXD2	3	112915495	3	0.58286
rs937557	PLCXD2	3	113047529	5	0.019114
rs3177104	PHLDB2	3	113177814	1	5.447
rs3177104	PHLDB2	3	113177814	8	5.86808
rs3177104	PHLDB2	3	113177814	3	0.989666
rs35978996	TAGLN3	3	113201136	6	0.735198
rs2292583	TAGLN3	3	113202476	6	0.434494
rs1492489	GCET2	3	113323993	7	0.677663
rs1492489	GCET2	3	113323993	2	2.819802
rs9288938	SLC9A10	3	113403806	3	0.765168

rs9288938	SLC9A10	3	113403806	7	0.333761
rs9288938	SLC9A10	3	113403806	1	1.155917
SNP3-113464568	SLC9A10	3	113464568	4	0.142034
SNP3-113464568	SLC9A10	3	113464568	7	0.329243
SNP3-113464568	SLC9A10	3	113464568	2	1.967238
rs9809404	SLC9A10	3	113464614	2	3.212211
rs9809404	SLC9A10	3	113464614	4	0.77616
rs9809404	SLC9A10	3	113464614	7	1.075827
rs9872691	SLC9A10	3	113467797	3	0.364784
rs9872691	SLC9A10	3	113467797	7	0.917685
rs9872691	SLC9A10	3	113467797	2	0.441626
rs9872691	SLC9A10	3	113467797	1	0.307169
rs9828502	SLC9A10	3	113479244	6	0.54548
rs9828502	SLC9A10	3	113479244	1	0.073373
rs9828502	SLC9A10	3	113479244	2	0.72232
rs9828502	SLC9A10	3	113479244	3	0.102974
rs9828502	SLC9A10	3	113479244	7	0.350172
rs2272022	CD200	3	113546540	8	5.108945
rs1050572	CD200	3	113549252	5	0.390896
rs1050572	CD200	3	113549252	4	0.290701
rs6807798	CCDC80	3	113842616	8	4.41727
rs7644174	C3orf17	3	114205016	8	2.696321
rs3814398	BOC	3	114474002	2	0.002279
rs11710894	BOC	3	114474649	1	0.479993
rs11710894	BOC	3	114474649	6	0.299048
rs11710894	BOC	3	114474649	2	0.250906
rs13094203	BOC	3	114476057	1	3.956281
rs13094203	BOC	3	114476057	7	2.348114
rs13094203	BOC	3	114476057	3	0.109742
rs2306792	WDR52	3	114564525	3	1.03719
rs13059888	WDR52	3	114610741	8	1.714759
rs13059888	WDR52	3	114610741	7	0.559357
rs13059888	WDR52	3	114610741	4	0.068395
rs13059888	WDR52	3	114610741	3	0.125627
rs13059888	WDR52	3	114610741	2	3.775429
rs33990195	SIDT1	3	114803167	1	2.681806
rs12493635	KIAA2018	3	114855709	2	4.393335
rs12493635	KIAA2018	3	114855709	8	0.018374
rs12493635	KIAA2018	3	114855709	7	0.560417
rs12493635	KIAA2018	3	114855709	3	0.353889
rs9866806	KIAA2018	3	114862555	3	0.952356
rs9866806	KIAA2018	3	114862555	7	0.410239
rs9866806	KIAA2018	3	114862555	8	11.70369
rs13079383	GRAMD1C	3	115077067	1	3.663393
rs13079383	GRAMD1C	3	115077067	4	1.748134
rs13079383	GRAMD1C	3	115077067	5	1.361789
rs3732787	GRAMD1C	3	115148710	3	0.092747

rs3732787	GRAMD1C	3	115148710	1	0.963911
rs3732787	GRAMD1C	3	115148710	4	0.139345
rs12629415	ZDHHC23	3	115162279	2	7.485956
rs17603649	KIAA1407	3	115220258	8	5.117859
rs6438191	ZNF80	3	115438010	4	1.107367
rs3821560	LSAMP	3	117054054	5	0.041956
rs3821560	LSAMP	3	117054054	3	0.847643
rs2903250	IGSF11	3	120131750	6	0.829758
SNP3-12023623	IGSF11	3	120236232	5	1.791936
SNP3-12023623	IGSF11	3	120236232	6	1.707061
rs9289122	C3orf30	3	120349737	5	0.486077
rs17281535	UPK1B	3	120375161	5	1.883119
rs3764779	B4GALT4	3	120428486	8	1.327739
rs3764779	B4GALT4	3	120428486	5	0.143963
rs3764779	B4GALT4	3	120428486	6	6.918481
rs1132202	TMEM39A	3	120633181	1	5.205394
rs1132202	TMEM39A	3	120633181	3	0.697701
rs1132202	TMEM39A	3	120633181	4	0.438915
rs2282173	TMEM39A	3	120665132	3	0.80621
rs2282173	TMEM39A	3	120665132	1	3.915234
rs2282173	TMEM39A	3	120665132	4	9.426763
rs3732420	KTELC1	3	120678856	1	2.576683
rs1938	KTELC1	3	120694557	7	0.366697
rs17281703	CD80	3	120726239	4	0.440515
rs17281703	CD80	3	120726239	1	3.611456
rs17281703	CD80	3	120726239	3	0.537819
rs1599795	CD80	3	120726545	5	0.576612
rs20568	ADPRH	3	120783829	2	6.522624
rs20568	ADPRH	3	120783829	3	1.193668
rs25676	ADPRH	3	120788069	2	3.247984
rs25676	ADPRH	3	120788069	5	4.605134
rs25676	ADPRH	3	120788069	3	0.660558
rs8422	POPDC2	3	120843634	4	1.412239
rs8422	POPDC2	3	120843634	3	0.416525
rs8422	POPDC2	3	120843634	2	3.02974
rs8007	POPDC2	3	120843940	2	2.689024
rs8007	POPDC2	3	120843940	4	0.26267
rs1880040	POPDC2	3	120861562	4	0.16025
rs1880040	POPDC2	3	120861562	2	3.294311
rs2873788	POPDC2	3	120861667	3	0.714574
rs4688023	POPDC2	3	120861876	4	0.545542
rs4688024	POPDC2	3	120862042	3	0.96575
rs7634701	C3orf15	3	120904624	3	0.227539
rs7612861	C3orf15	3	120904653	3	0.125566
rs7612861	C3orf15	3	120904653	5	0.563382
rs10511394	C3orf15	3	120967291	4	0.939199
rs10511394	C3orf15	3	120967291	3	3.422613

rs10511394	C3orf15	3	120967291	5	0.148764
rs1129708	C3orf15	3	120968276	5	0.650673
rs1129708	C3orf15	3	120968276	3	1.659308
rs12721607	NR1I2	3	121008893	3	1.322141
rs3732359	NR1I2	3	121019119	3	0.410027
rs10511395	NR1I2	3	121019249	4	0.792307
rs10511395	NR1I2	3	121019249	7	0.809667
rs10511395	NR1I2	3	121019249	5	0.526978
rs3732360	NR1I2	3	121019271	3	0.379553
rs1054190	NR1I2	3	121019408	4	1.083456
rs1054190	NR1I2	3	121019408	5	1.007211
rs1054191	NR1I2	3	121019587	4	2.00361
rs1054191	NR1I2	3	121019587	7	2.509533
rs1054191	NR1I2	3	121019587	5	4.21577
rs3814057	NR1I2	3	121019944	3	0.435888
rs3814058	NR1I2	3	121019981	3	0.307491
rs16831036	LRRC58	3	121531293	2	5.06572
rs16831036	LRRC58	3	121531293	4	0.003778
SNP3-121872006	HGD	3	121872006	2	5.582856
SNP3-121872006	HGD	3	121872006	4	0.227084
SNP3-121872006	HGD	3	121872006	8	0.045523
SNP3-121872006	HGD	3	121872006	3	0.53124
SNP3-121872006	HGD	3	121872006	6	0.432132
rs6782025	STXBP5L	3	122620666	1	1.796961
rs6782033	STXBP5L	3	122620705	1	1.441088
rs1381057	POLQ	3	122637664	1	2.684773
rs41540016	POLQ	3	122637812	4	5.260977
SNP3-122823226	FBXO40	3	122823226	5	0.690536
rs1128163	HCLS1	3	122833263	7	0.080032
rs1128163	HCLS1	3	122833263	3	1.391895
rs1128163	HCLS1	3	122833263	4	1.036512
rs1128158	HCLS1	3	122833283	6	0.704035
rs1128158	HCLS1	3	122833283	4	0.745116
rs1128158	HCLS1	3	122833283	3	1.510743
rs1128154	HCLS1	3	122833306	3	1.967654
rs1128154	HCLS1	3	122833306	4	0.548628
rs1128154	HCLS1	3	122833306	6	1.103525
rs2070180	HCLS1	3	122834028	5	0.671911
rs2070180	HCLS1	3	122834028	7	1.454512
rs3772126	HCLS1	3	122837273	3	0.089443
rs3772126	HCLS1	3	122837273	4	0.020952
rs3732407	GOLGB1	3	122899313	4	0.071834
rs3732407	GOLGB1	3	122899313	3	0.26028
rs3732407	GOLGB1	3	122899313	8	1.595286
rs2293616	SLC15A2	3	123124383	3	0.7528
rs2293616	SLC15A2	3	123124383	2	6.724396
rs2293616	SLC15A2	3	123124383	5	0.245618

rs2293616	SLC15A2	3	123124383	4	6.377117
rs2257212	SLC15A2	3	123126494	3	0.259245
rs2257212	SLC15A2	3	123126494	4	0.505315
rs2257212	SLC15A2	3	123126494	5	0.741658
rs1143670	SLC15A2	3	123129331	2	3.883616
rs1143670	SLC15A2	3	123129331	5	0.56886
rs1143670	SLC15A2	3	123129331	3	1.125585
rs1143670	SLC15A2	3	123129331	4	0.176623
rs1143671	SLC15A2	3	123129976	3	0.34647
rs2877561	ILDR1	3	123194741	3	1.30964
rs2877561	ILDR1	3	123194741	4	0.172758
rs2877561	ILDR1	3	123194741	8	3.613346
SNP3-12319498ɛ	ILDR1	3	123194988	8	4.947232
SNP3-12319498ɛ	ILDR1	3	123194988	3	3.959115
SNP3-12319498ɛ	ILDR1	3	123194988	2	1.13116
SNP3-12319498ɛ	ILDR1	3	123194988	1	6.245053
SNP3-12319498ɛ	ILDR1	3	123194988	5	4.534065
rs2681417	CD86	3	123307887	8	1.311079
rs1129055	CD86	3	123321009	6	1.702587
rs1129055	CD86	3	123321009	8	1.551793
rs1915087	CD86	3	123321481	6	0.462032
rs12636058	WDR5B	3	123613441	8	2.751367
rs12636058	WDR5B	3	123613441	6	0.014824
rs12636058	WDR5B	3	123613441	3	1.29556
rs12636058	WDR5B	3	123613441	2	1.830796
rs12636058	WDR5B	3	123613441	1	1.028403
rs9843165	WDR5B	3	123613801	3	6.008901
rs3828362	WDR5B	3	123614467	1	6.014348
rs3828362	WDR5B	3	123614467	6	3.520338
rs3828362	WDR5B	3	123614467	8	3.944636
rs16833133	KPNA1	3	123627563	4	0.622784
rs16833133	KPNA1	3	123627563	5	5.583872
SNP3-12374233(	PARP9	3	123742330	8	0.409193
SNP3-12374233(	PARP9	3	123742330	4	0.84884
SNP3-12374233(	PARP9	3	123742330	5	5.218139
rs7616403	DTX3L	3	123774043	1	4.220122
rs1106346	PARP15	3	123836727	5	1.97194
rs1106346	PARP15	3	123836727	1	3.637934
rs1106346	PARP15	3	123836727	3	1.494973
rs10934615	PARP14	3	123930893	7	0.037433
rs10934615	PARP14	3	123930893	8	2.587833
rs3806643	HSPBAP1	3	123995321	1	2.860077
rs3806643	HSPBAP1	3	123995321	7	0.137108
rs891406	DIRC2	3	123996654	1	4.856188
rs2276774	SEMA5B	3	124129518	3	1.14659
rs1976716	PDIA5	3	124347126	5	2.716078
rs1976716	PDIA5	3	124347126	2	2.772823

rs1976716	PDIA5	3	124347126	4	0.405825
rs1137138	SEC22A	3	124473711	5	0.341467
rs1137138	SEC22A	3	124473711	8	0.511471
rs1137138	SEC22A	3	124473711	6	6.079685
rs6438805	MYLK	3	124815236	5	0.086098
rs6438805	MYLK	3	124815236	8	7.474191
rs6438805	MYLK	3	124815236	6	1.219194
rs17298941	MYLK	3	124858757	6	7.661545
rs3732487	MYLK	3	124902263	1	5.07295
rs35156360	MYLK	3	124934622	5	0.02065
rs2700408	MYLK	3	125078122	8	2.999426
rs2272486	KALRN	3	125470729	4	0.103551
rs2272486	KALRN	3	125470729	3	0.224917
rs2289778	KALRN	3	125527639	8	2.176018
rs2289778	KALRN	3	125527639	2	1.917139
rs2289778	KALRN	3	125527639	5	0.204728
rs2276740	KALRN	3	125531454	8	2.256913
rs2289843	KALRN	3	125678784	7	1.094464
rs6765634	KALRN	3	125720281	1	0.424729
rs6765634	KALRN	3	125720281	2	0.920089
rs6765634	KALRN	3	125720281	3	0.115752
rs7613442	KALRN	3	125720820	1	1.284014
rs7613442	KALRN	3	125720820	3	0.48379
rs1708303	KALRN	3	125834006	5	0.150807
rs1708303	KALRN	3	125834006	3	0.285139
rs1708303	KALRN	3	125834006	6	0.115809
rs1660038	KALRN	3	125834114	5	1.696278
rs1660038	KALRN	3	125834114	6	1.110584
rs1660038	KALRN	3	125834114	3	2.587642
rs1660038	KALRN	3	125834114	2	5.430222
rs2289427	KALRN	3	125901536	4	1.205272
rs2289427	KALRN	3	125901536	1	2.031853
rs2289427	KALRN	3	125901536	8	1.118667
rs2289427	KALRN	3	125901536	3	1.823074
SNP3-12590156	KALRN	3	125901569	4	2.553861
SNP3-12590156	KALRN	3	125901569	1	3.299285
SNP3-12590156	KALRN	3	125901569	5	1.421102
rs4234221	KALRN	3	125921019	5	0.549231
rs4234221	KALRN	3	125921019	3	0.497119
rs332512	KALRN	3	125921129	1	1.435048
rs332512	KALRN	3	125921129	3	0.486698
rs332514	KALRN	3	125922257	3	0.070226
rs1139538	UMPS	3	125931981	7	1.164719
rs1139538	UMPS	3	125931981	4	0.948134
rs1139538	UMPS	3	125931981	3	1.496279
rs2291085	ITGB5	3	125998004	3	0.893503
rs4679392	MUC13	3	126129284	4	1.368291

rs4679392	MUC13	3	126129284	7	1.027845
rs4679392	MUC13	3	126129284	8	1.62355
rs1909586	HEG1	3	126170457	3	0.875079
rs1909586	HEG1	3	126170457	7	2.284194
rs1909586	HEG1	3	126170457	5	0.018218
rs1472516	HEG1	3	126171481	2	6.28314
rs1472516	HEG1	3	126171481	3	0.148676
rs1472516	HEG1	3	126171481	5	0.115835
rs10804567	HEG1	3	126214175	5	0.093428
rs10804567	HEG1	3	126214175	3	0.604971
rs10804567	HEG1	3	126214175	7	0.64007
SNP3-12622079	HEG1	3	126220797	7	0.24692
SNP3-12622079	HEG1	3	126220797	2	2.116613
SNP3-12622079	HEG1	3	126220797	5	1.166598
SNP3-12622079	HEG1	3	126220797	3	0.781817
rs2981482	SLC12A8	3	126285578	1	3.485695
rs2981482	SLC12A8	3	126285578	7	0.603627
rs2333223	SNX4	3	126648725	1	5.333752
rs6438964	SLC41A3	3	127258118	1	2.318719
rs6438964	SLC41A3	3	127258118	3	2.495367
rs6438964	SLC41A3	3	127258118	2	1.955475
rs1055451	CCDC37	3	127638015	1	3.420204
rs15058	ZXDC	3	127639266	1	4.868037
rs7131	ZXDC	3	127639350	5	12.36772
rs7131	ZXDC	3	127639350	4	6.099351
rs7131	ZXDC	3	127639350	6	8.017673
rs7131	ZXDC	3	127639350	7	0.462375
rs7131	ZXDC	3	127639350	2	1.583489
rs1799398	UROC1	3	127683093	6	1.376993
rs1799398	UROC1	3	127683093	3	0.030378
rs1687477	UROC1	3	127684947	4	1.619234
rs1687477	UROC1	3	127684947	6	2.008757
rs1056524	CHST13	3	127743892	6	2.248678
rs1056524	CHST13	3	127743892	3	1.180774
rs1056522	CHST13	3	127744035	7	0.090056
rs869463	C3orf22	3	127751608	7	0.048557
rs2272487	CHCHD6	3	127934627	1	0.567134
rs41266467	PLXNA1	3	128204994	7	0.219415
rs9856665	PLXNA1	3	128237334	7	2.190949
rs9856665	PLXNA1	3	128237334	3	0.517695
rs9856665	PLXNA1	3	128237334	5	0.739528
rs9856665	PLXNA1	3	128237334	6	0.486973
rs9856665	PLXNA1	3	128237334	1	5.086613
rs920232	PODXL2	3	128861960	1	4.698565
rs920232	PODXL2	3	128861960	7	0.075033
rs782444	MGLL	3	128892631	1	3.054316
rs782444	MGLL	3	128892631	3	1.258193

rs782444	MGLL	3	128892631	6	0.539691
rs782444	MGLL	3	128892631	8	0.051
rs814131	MGLL	3	128893605	3	0.613613
rs814131	MGLL	3	128893605	1	5.212994
rs814131	MGLL	3	128893605	6	2.528509
rs814131	MGLL	3	128893605	8	0.612736
rs2981026	DNAJB8	3	129664764	5	1.156691
rs13071640	DNAJB8	3	129665068	3	0.479267
rs3803	GATA2	3	129682070	7	0.079952
rs2335052	GATA2	3	129687641	5	0.2525
rs1573858	GATA2	3	129688550	8	7.613438
rs6762732	C3orf27	3	129775548	3	0.384236
rs2465354	C3orf27	3	129777587	5	2.198688
rs2465354	C3orf27	3	129777587	1	3.178011
rs4494951	ACAD9	3	130110577	3	0.991845
rs876755	ACAD9	3	130110623	2	0.103252
rs789212	KIAA1257	3	130169602	1	0.340274
rs789212	KIAA1257	3	130169602	3	0.477138
rs16859327	C3orf25	3	130616932	5	1.487455
rs3796392	C3orf25	3	130619817	5	1.535258
SNP3-13061987‹	C3orf25	3	130619878	3	0.477676
rs3774788	C3orf25	3	130623179	6	1.578536
rs3774788	C3orf25	3	130623179	7	0.816142
rs2307293	MBD4	3	130633075	1	6.858188
SNP3-13069699‹	IFT122	3	130696992	6	7.077339
SNP3-13069699‹	IFT122	3	130696992	7	0.628637
rs2269736	RHO	3	130730216	5	1.16704
rs2410	RHO	3	130735483	5	0.400921
SNP3-13075060‹	H1FOO	3	130750600	7	2.745831
rs2625973	PLXND1	3	130767508	7	0.410479
rs2625973	PLXND1	3	130767508	2	4.031255
rs2625973	PLXND1	3	130767508	3	1.917574
rs2625973	PLXND1	3	130767508	8	4.525329
rs2245285	PLXND1	3	130769103	8	0.515475
rs2245285	PLXND1	3	130769103	7	7.463748
rs2245285	PLXND1	3	130769103	3	1.220674
rs7428344	LOC729375	3	131308790	5	1.715127
rs7428344	LOC729375	3	131308790	1	0.197118
rs7428344	LOC729375	3	131308790	6	0.778373
rs9849611	LOC646300	3	131467888	3	1.412637
rs7614116	COL6A6	3	131850759	3	1.170743
rs7636656	COL6A6	3	131850991	5	0.007212
rs17748751	COL6A6	3	131877418	3	0.480167
rs17748751	COL6A6	3	131877418	5	2.672227
rs7639697	NEK11	3	132376534	6	0.591494
rs12639101	NEK11	3	132376608	8	1.355726
rs3738000	NEK11	3	132430125	8	9.260855

rs3738000	NEK11	3	132430125	4	6.092106
rs3738000	NEK11	3	132430125	6	0.117512
rs3738000	NEK11	3	132430125	5	8.292993
rs7648888	CPNE4	3	133236213	3	1.437512
rs7648888	CPNE4	3	133236213	8	1.429787
rs7648888	CPNE4	3	133236213	4	1.296425
rs7648888	CPNE4	3	133236213	7	6.607289
SNP3-133558397	ACPP	3	133558397	2	0.177758
SNP3-133558397	ACPP	3	133558397	7	0.303325
SNP3-133558397	ACPP	3	133558397	3	0.053478
SNP3-133558397	ACPP	3	133558397	6	0.425736
SNP3-133558397	ACPP	3	133558397	4	5.83959
SNP3-133558397	ACPP	3	133558397	5	4.157699
SNP3-133558397	ACPP	3	133558397	1	0.941468
rs1042330	ACPP	3	133558436	3	1.045386
rs1042330	ACPP	3	133558436	7	1.353048
rs1042330	ACPP	3	133558436	4	4.17809
rs1042330	ACPP	3	133558436	5	0.081856
rs1042330	ACPP	3	133558436	6	2.919282
rs1042330	ACPP	3	133558436	1	0.254738
rs1042330	ACPP	3	133558436	2	1.81626
rs2239698	ACPP	3	133558886	1	4.130586
rs2239698	ACPP	3	133558886	5	0.939522
rs14192	ACPP	3	133559241	4	5.741893
rs14192	ACPP	3	133559241	5	3.831548
rs14192	ACPP	3	133559241	3	6.575396
rs14192	ACPP	3	133559241	1	0.130649
rs14192	ACPP	3	133559241	2	2.257046
rs14192	ACPP	3	133559241	6	4.436622
rs9877018	ACAD11	3	133844286	1	4.482996
rs11708200	NPHP3	3	133883178	2	3.890001
rs1197314	TMEM108	3	134581935	7	2.523595
rs6792846	CDV3	3	134790181	7	0.772674
rs17301766	TOPBP1	3	134839480	2	3.823311
rs17301766	TOPBP1	3	134839480	4	0.057575
rs3192149	TOPBP1	3	134851052	4	1.272634
rs8649	TF	3	134969648	1	0.332504
rs1107413	SRPRB	3	135007407	1	0.741568
rs1107413	SRPRB	3	135007407	2	0.352863
rs1107413	SRPRB	3	135007407	6	1.023207
rs1107413	SRPRB	3	135007407	3	0.071019
rs3811659	RAB6B	3	135026698	4	0.096891
rs1043147	RAB6B	3	135027688	4	0.09811
rs12637730	RAB6B	3	135028987	4	2.95298
rs2293374	RAB6B	3	135029935	4	3.718595
rs2293373	RAB6B	3	135030382	4	1.341312
SNP3-135148899	SLCO2A1	3	135148899	3	0.821018

SNP3-135148899	SLCO2A1	3	135148899	5	6.163393
SNP3-135148899	SLCO2A1	3	135148899	2	4.550717
rs1874883	CEP63	3	135687761	4	1.013794
rs1874883	CEP63	3	135687761	3	0.368575
rs1874883	CEP63	3	135687761	7	0.387745
rs1874883	CEP63	3	135687761	5	1.183126
rs1874883	CEP63	3	135687761	8	0.568893
rs9868985	CEP63	3	135760960	6	2.886519
rs9868985	CEP63	3	135760960	4	0.470767
rs13071253	LOC729534	3	135799343	2	2.418727
rs13071253	LOC729534	3	135799343	3	0.277113
rs13071253	LOC729534	3	135799343	4	0.853772
rs13071253	LOC729534	3	135799343	5	0.702047
rs9857995	LOC729534	3	135799396	8	1.2831
rs9857995	LOC729534	3	135799396	4	1.064971
rs9857995	LOC729534	3	135799396	3	1.056858
rs9857995	LOC729534	3	135799396	5	0.49707
rs9857995	LOC729534	3	135799396	7	0.163565
rs13078595	LOC645218	3	136055947	1	0.72498
rs7644369	EPHB1	3	136153214	4	0.273012
rs7644369	EPHB1	3	136153214	7	0.232253
rs7644369	EPHB1	3	136153214	1	2.665487
rs3732566	EPHB1	3	136381432	6	0.458016
rs3732566	EPHB1	3	136381432	3	1.164839
rs3732566	EPHB1	3	136381432	7	0.593972
rs3732566	EPHB1	3	136381432	4	4.255322
rs3732566	EPHB1	3	136381432	8	5.684199
rs10608	EPHB1	3	136461859	1	4.31304
rs10608	EPHB1	3	136461859	8	0.851911
SNP3-137203230	PPP2R3A	3	137203230	3	0.281315
SNP3-137203230	PPP2R3A	3	137203230	8	6.029248
SNP3-137203230	PPP2R3A	3	137203230	1	3.354301
rs9845457	MSL2L1	3	137397166	1	2.15971
rs9845457	MSL2L1	3	137397166	3	0.130188
rs9845457	MSL2L1	3	137397166	8	3.555196
rs9845457	MSL2L1	3	137397166	7	1.133144
rs9845457	MSL2L1	3	137397166	5	1.178273
rs1052620	TMEM22	3	138057211	1	4.180259
rs1052620	TMEM22	3	138057211	5	0.127316
rs2589258	CLDN18	3	139234694	5	2.278781
rs2589258	CLDN18	3	139234694	7	0.109778
rs2589258	CLDN18	3	139234694	6	0.262714
rs2589258	CLDN18	3	139234694	1	5.515016
rs442800	DZIP1L	3	139269132	5	2.528079
rs442800	DZIP1L	3	139269132	2	6.653021
rs442800	DZIP1L	3	139269132	1	0.455258
rs2246945	A4GNT	3	139326166	1	0.775119

rs2622737	DBR1	3	139362683	5	0.067209
rs2622737	DBR1	3	139362683	7	0.365021
rs2622737	DBR1	3	139362683	1	5.472747
rs2622737	DBR1	3	139362683	6	0.895366
rs2622737	DBR1	3	139362683	4	0.019874
rs2622737	DBR1	3	139362683	8	0.115403
SNP3-139603888	MRAS	3	139603888	4	8.595117
rs9818870	MRAS	3	139604812	4	0.539591
SNP3-139605167	MRAS	3	139605167	4	5.576783
SNP3-139669706	FAM62C	3	139669706	8	0.654385
rs2347469	FAM62C	3	139671721	8	0.413745
rs3813264	FAM62C	3	139672348	8	0.665349
rs1673608	CEP70	3	139771853	1	4.395253
rs1673608	CEP70	3	139771853	4	2.621768
rs811322	FAIM	3	139812552	4	1.015067
rs811322	FAIM	3	139812552	8	1.384706
rs811322	FAIM	3	139812552	1	4.101301
rs641320	FAIM	3	139830647	5	0.689241
rs9816111	NMNAT3	3	140879516	1	0.77194
rs9816111	NMNAT3	3	140879516	3	1.432566
rs10804675	CLSTN2	3	141765551	3	0.732457
rs13078522	CLSTN2	3	141768194	8	5.506344
rs9876490	TRIM42	3	141889950	1	2.869667
rs3210458	ACPL2	3	142494320	1	5.334893
rs2312500	LOC646730	3	142868380	3	1.990452
rs2312500	LOC646730	3	142868380	2	3.162678
rs6767392	LOC646730	3	142887521	1	0.543162
rs3184648	RNF7	3	142946836	3	0.81644
rs3184648	RNF7	3	142946836	1	1.944544
rs1533501	GRK7	3	142979840	7	0.43656
rs36009541	GRK7	3	143018248	7	1.638726
SNP3-143018298	GRK7	3	143018298	7	1.14436
SNP3-143018298	GRK7	3	143018298	4	1.082567
rs2227930	ATR	3	143760265	6	4.121823
rs2227930	ATR	3	143760265	7	0.007434
rs2227930	ATR	3	143760265	1	0.156402
rs2227930	ATR	3	143760265	2	4.200504
rs2227930	ATR	3	143760265	4	7.201476
rs6763202	SLC9A9	3	144775702	1	3.178042
rs6763202	SLC9A9	3	144775702	4	0.342067
rs1242069	SLC9A9	3	144853891	7	0.366695
rs13092702	LOC440981	3	147601985	3	0.966389
rs13092702	LOC440981	3	147601985	1	0.581424
rs13092702	LOC440981	3	147601985	6	0.255459
rs13092702	LOC440981	3	147601985	2	0.404144
rs13092702	LOC440981	3	147601985	5	0.103726
rs1054736	PLSCR2	3	147633865	3	1.337171

rs1054736	PLSCR2	3	147633865	5	0.487345
rs1054736	PLSCR2	3	147633865	1	0.290374
rs9289748	FLJ30375	3	148620666	8	5.443912
rs1059502	CPB1	3	150045000	7	2.095426
rs1059502	CPB1	3	150045000	1	1.002651
rs1059502	CPB1	3	150045000	2	0.884869
rs2229361	HLTF	3	150240554	5	0.273909
rs1802837	TM4SF4	3	150676374	5	0.267495
rs1802837	TM4SF4	3	150676374	6	2.491001
rs12636725	TM4SF4	3	150703207	1	1.118672
rs11557682	RNF13	3	151013315	7	3.280043
rs11557682	RNF13	3	151013315	5	0.441899
rs11557682	RNF13	3	151013315	8	0.214059
rs11557682	RNF13	3	151013315	3	0.551673
rs11557682	RNF13	3	151013315	6	4.456578
rs879634	TSC22D2	3	151611082	5	0.488556
rs879634	TSC22D2	3	151611082	8	3.867076
rs2626	EIF2A	3	151786357	4	0.421297
rs2626	EIF2A	3	151786357	2	6.612481
rs2626	EIF2A	3	151786357	5	2.450546
SNP3-15187450	C3orf44	3	151874500	3	0.699961
rs17281287	C3orf44	3	151904109	8	0.956095
rs17281287	C3orf44	3	151904109	1	1.319686
rs17281287	C3orf44	3	151904109	5	0.216377
rs2168445	LOC646951	3	152105550	3	0.353619
rs11914988	CLRN1	3	152144532	3	0.285144
rs3796242	CLRN1	3	152173129	1	0.485758
rs17290219	MED12L	3	152364464	6	0.865828
rs1220995	P2RY14	3	152413762	3	0.438044
rs1220995	P2RY14	3	152413762	5	1.123023
rs1472122	GPR87	3	152517292	8	4.355036
rs1472122	GPR87	3	152517292	3	1.180226
rs1472122	GPR87	3	152517292	1	4.319412
rs1472122	GPR87	3	152517292	7	1.220186
rs6440735	P2RY13	3	152527422	1	4.955774
rs12497019	P2RY13	3	152527593	5	2.296044
rs12497019	P2RY13	3	152527593	3	0.299552
rs12497019	P2RY13	3	152527593	4	1.578628
rs1388628	P2RY13	3	152527936	3	0.434384
rs1388627	P2RY13	3	152527985	3	0.162054
rs1491980	P2RY13	3	152528351	1	1.90286
rs1466684	P2RY13	3	152528998	1	1.302334
rs3732765	MED12L	3	152573114	4	0.230884
rs3732765	MED12L	3	152573114	3	0.778757
rs3732768	MED12L	3	152595258	3	1.39222
rs3732768	MED12L	3	152595258	8	0.18274
rs3732768	MED12L	3	152595258	5	3.154376

rs3732768	MED12L	3	152595258	2	1.875245
rs3732768	MED12L	3	152595258	7	0.884727
rs7611956	MED12L	3	152633910	4	0.721949
rs7611956	MED12L	3	152633910	2	3.781103
rs2172250	IGSF10	3	152637356	4	0.031211
rs2172250	IGSF10	3	152637356	7	0.635651
rs6781302	IGSF10	3	152638310	7	4.38898
rs6781302	IGSF10	3	152638310	1	0.833606
rs6781302	IGSF10	3	152638310	4	1.216611
rs6798252	IGSF10	3	152649112	7	0.914376
rs7619322	IGSF10	3	152654129	7	1.328842
rs7621591	IGSF10	3	152654232	7	0.930654
rs842049	hCG_1806964	3	153466638	1	3.994732
rs842049	hCG_1806964	3	153466638	7	0.84698
rs842049	hCG_1806964	3	153466638	4	0.80292
rs6785014	RAP2B	3	154368289	2	3.432118
rs6785014	RAP2B	3	154368289	8	0.157253
rs6785014	RAP2B	3	154368289	7	0.301179
SNP3-155322649	SGEF	3	155322649	7	0.401297
SNP3-155322649	SGEF	3	155322649	2	3.174754
SNP3-155323079	SGEF	3	155323079	7	4.022223
SNP3-155426460	SGEF	3	155426460	1	3.409623
rs191651	SGEF	3	155456581	7	1.178876
rs191651	SGEF	3	155456581	2	6.620175
rs9438	DHX36	3	155501581	8	0.718078
rs9438	DHX36	3	155501581	3	0.568567
rs9438	DHX36	3	155501581	2	5.019779
rs9438	DHX36	3	155501581	4	0.110007
rs9438	DHX36	3	155501581	7	0.306782
rs7610758	MME	3	156382538	7	0.972957
rs1816522	PLCH1	3	156683257	2	3.868193
rs359569	PLCH1	3	156796728	5	0.045391
rs359569	PLCH1	3	156796728	3	0.571379
rs6806798	C3orf33	3	156963791	1	0.208852
rs7653384	C3orf33	3	156964303	1	0.49309
rs7653384	C3orf33	3	156964303	8	0.083741
rs7653384	C3orf33	3	156964303	6	0.065074
rs358733	C3orf33	3	156967996	1	1.046695
rs6781558	VN2R1P	3	157234178	8	1.268832
rs907060	KCNAB1	3	157737781	2	0.816615
rs907060	KCNAB1	3	157737781	1	0.000521
rs3773714	SSR3	3	157742660	1	5.10094
rs3773714	SSR3	3	157742660	5	0.021561
rs3773714	SSR3	3	157742660	7	0.182886
rs6441095	LOC653801	3	157874885	2	1.552553
rs344053	TIPARP	3	157906505	2	4.015859
rs10936063	KRT18P34	3	158363577	3	0.259448

rs10936063	KRT18P34	3	158363577	7	1.069758
rs10936063	KRT18P34	3	158363577	5	0.405022
rs10936065	KRT18P34	3	158363639	5	1.240568
rs10936065	KRT18P34	3	158363639	3	1.320794
rs10936065	KRT18P34	3	158363639	7	0.880813
rs11918974	VEPH1	3	158564018	5	0.303796
rs11918974	VEPH1	3	158564018	3	0.30286
rs11918974	VEPH1	3	158564018	6	0.176088
rs1048243	SHOX2	3	159298155	4	0.470969
rs1048243	SHOX2	3	159298155	1	0.514625
rs1048243	SHOX2	3	159298155	7	1.558078
rs8455	LXN	3	159871474	4	0.992362
rs8455	LXN	3	159871474	1	0.584009
rs8455	LXN	3	159871474	3	0.46045
rs568408	IL12A	3	161196161	7	4.429117
rs11921898	BRD7P2	3	161302261	5	1.192278
rs11921898	BRD7P2	3	161302261	7	2.523215
rs34182424	IFT80	3	161566612	4	0.641924
rs34182424	IFT80	3	161566612	6	7.792002
rs7629	TRIM59	3	161635999	1	0.110805
rs7629	TRIM59	3	161635999	3	0.912636
rs4679885	TRIM59	3	161637441	2	0.244607
rs4679885	TRIM59	3	161637441	5	2.824259
rs4679885	TRIM59	3	161637441	4	0.671213
rs4679885	TRIM59	3	161637441	1	1.48655
rs4679885	TRIM59	3	161637441	3	1.597838
rs2279457	LOC401098	3	161650986	3	0.250396
rs2279457	LOC401098	3	161650986	4	0.594707
rs2279457	LOC401098	3	161650986	2	0.544617
rs2279457	LOC401098	3	161650986	1	0.528265
rs1993891	LOC646085	3	162530161	6	0.713217
rs4856703	LOC646085	3	162530414	5	1.239626
rs4856703	LOC646085	3	162530414	3	1.68123
rs960212	LOC646085	3	162530668	2	2.745541
rs960212	LOC646085	3	162530668	6	0.106782
rs3921595	LOC131149	3	162704399	1	1.722064
rs11922760	SLITRK3	3	166388264	5	0.04738
rs3792293	PDCD10	3	168935198	7	0.642641
rs3792293	PDCD10	3	168935198	6	0.080801
rs3792293	PDCD10	3	168935198	4	1.321783
rs2229697	SERPINI1	3	168993166	7	0.431847
SNP3-16922971	GOLIM4	3	169229716	5	1.385735
SNP3-16922971	GOLIM4	3	169229716	4	1.082387
SNP3-16922971	GOLIM4	3	169229716	6	0.621826
rs17466625	EVI1	3	170293568	2	2.362263
rs17466625	EVI1	3	170293568	5	0.196936
rs10936600	LRRC34	3	170997279	7	3.254148

rs10936600	LRRC34	3	170997279	2	5.69462
rs10936600	LRRC34	3	170997279	5	0.98419
rs3772172	SKIL	3	171560407	7	1.002925
rs3772172	SKIL	3	171560407	4	0.576194
rs4955729	FLJ37228	3	171662276	3	0.794069
rs4955730	FLJ37228	3	171662315	3	0.566756
rs6792607	EIF5A2	3	172093010	6	1.525743
rs6792607	EIF5A2	3	172093010	5	0.092807
rs7652177	FNDC3B	3	173451771	1	0.113245
rs7652177	FNDC3B	3	173451771	7	0.020587
rs7652177	FNDC3B	3	173451771	8	6.789853
rs7652177	FNDC3B	3	173451771	6	0.221726
rs7652177	FNDC3B	3	173451771	2	6.547205
rs2270568	FNDC3B	3	173529555	3	0.408557
rs2270568	FNDC3B	3	173529555	5	0.424342
rs2270568	FNDC3B	3	173529555	1	3.725794
rs2270568	FNDC3B	3	173529555	6	0.104645
rs2270568	FNDC3B	3	173529555	4	1.033113
rs2232169	GHSR	3	173648451	3	0.661911
rs2232169	GHSR	3	173648451	6	1.155788
rs2232165	GHSR	3	173648838	3	0.008838
rs1131532	TNFSF10	3	173706997	3	0.532567
rs3733023	AADACL1	3	173833700	1	6.143589
rs3733023	AADACL1	3	173833700	5	0.059857
rs3733023	AADACL1	3	173833700	3	0.039175
rs3733023	AADACL1	3	173833700	4	0.824861
rs16846616	SPATA16	3	174317776	7	0.271105
rs16846616	SPATA16	3	174317776	3	0.091914
rs9290502	LOC647212	3	175751228	5	0.583338
rs9290502	LOC647212	3	175751228	3	1.385386
rs10222515	TBL1XR1	3	178224424	2	2.045921
rs10222515	TBL1XR1	3	178224424	3	5.501732
rs10222515	TBL1XR1	3	178224424	1	1.135711
rs6443583	KCNMB2	3	180043275	2	1.683601
rs6443583	KCNMB2	3	180043275	5	0.234977
rs6443583	KCNMB2	3	180043275	6	1.226029
rs1183319	KCNMB3	3	180451801	4	0.813364
rs1183319	KCNMB3	3	180451801	5	0.108346
rs1183319	KCNMB3	3	180451801	7	0.283164
rs1183319	KCNMB3	3	180451801	8	0.150319
rs11720871	KCNMB3	3	180459471	1	3.478771
rs11720871	KCNMB3	3	180459471	8	1.076271
rs11720871	KCNMB3	3	180459471	7	1.763478
rs11720871	KCNMB3	3	180459471	4	1.328644
rs10936996	KCNMB3	3	180459768	6	0.387529
rs10936996	KCNMB3	3	180459768	7	0.531027
rs13324543	ACTL6A	3	180781693	1	3.528549

rs13324543	ACTL6A	3	180781693	6	0.336204
rs13324543	ACTL6A	3	180781693	8	0.180125
rs2276803	USP13	3	180853848	8	4.819655
rs2276803	USP13	3	180853848	2	4.634767
rs9867478	LOC131054	3	181524772	6	3.767992
rs1532485	TTC14	3	181802675	2	0.528604
rs1532485	TTC14	3	181802675	4	0.541204
rs1532485	TTC14	3	181802675	1	1.129263
rs2270968	MCCC1	3	184237903	2	3.47133
rs2270968	MCCC1	3	184237903	6	6.071858
rs35070271	MCF2L2	3	184380614	8	3.140899
rs35070271	MCF2L2	3	184380614	4	3.072695
rs3755648	KLHL24	3	184850910	4	0.137558
rs9856997	KLHL24	3	184880201	4	3.653937
rs2606225	MAP6D1	3	185017181	2	2.461446
rs2606225	MAP6D1	3	185017181	6	0.446911
rs2606225	MAP6D1	3	185017181	3	0.226215
rs2606225	MAP6D1	3	185017181	1	0.137455
rs2255019	MAP6D1	3	185017607	1	1.083355
rs2255019	MAP6D1	3	185017607	2	0.785156
rs2255019	MAP6D1	3	185017607	3	0.358286
rs3732581	PARL	3	185041096	3	0.422302
rs3732581	PARL	3	185041096	1	0.359191
rs3732581	PARL	3	185041096	2	3.277295
rs3732581	PARL	3	185041096	8	8.406983
rs3732581	PARL	3	185041096	5	3.106396
rs3805114	ABCC5	3	185120416	4	4.519441
rs562	ABCC5	3	185120539	8	5.265552
rs562	ABCC5	3	185120539	4	0.154019
rs562	ABCC5	3	185120539	1	6.165818
rs562	ABCC5	3	185120539	5	0.283539
rs7636910	ABCC5	3	185182210	1	5.516853
rs7636910	ABCC5	3	185182210	5	0.725295
rs7636910	ABCC5	3	185182210	4	1.171901
rs6443930	HTR3D	3	185236988	5	0.538006
rs6807670	HTR3C	3	185260992	1	4.155696
rs6807670	HTR3C	3	185260992	2	5.341366
rs6807670	HTR3C	3	185260992	5	4.326336
rs34611203	HTR3E	3	185300666	2	1.25312
rs11545172	PSMD2	3	185502375	3	0.118943
rs11545172	PSMD2	3	185502375	1	3.51653
rs2230571	EIF4G1	3	185529164	7	0.222455
rs2230571	EIF4G1	3	185529164	2	4.8112
rs2230571	EIF4G1	3	185529164	4	2.647092
rs13322510	FAM131A	3	185541275	7	1.241462
rs13322510	FAM131A	3	185541275	1	0.809348
rs13322510	FAM131A	3	185541275	3	0.827968

rs2228291	CLCN2	3	185557741	1	0.041218
rs2228291	CLCN2	3	185557741	7	0.105596
rs11916995	LOC391600	3	185700371	3	0.583211
rs12011	MAGEF1	3	185912412	3	0.534377
rs12011	MAGEF1	3	185912412	2	0.402615
rs12011	MAGEF1	3	185912412	1	0.301183
rs11555405	VPS8	3	186183120	5	0.968442
rs11555405	VPS8	3	186183120	3	0.423931
rs11555405	VPS8	3	186183120	1	1.6954
rs1043435	LOC285382	3	186280459	1	1.407575
rs1043435	LOC285382	3	186280459	5	0.509528
rs1043435	LOC285382	3	186280459	4	0.128832
rs6444028	LOC285382	3	186280816	4	1.295969
rs16859583	LOC285382	3	186280982	5	0.085962
rs11919970	EHHADH	3	186392827	1	4.998409
rs11919970	EHHADH	3	186392827	5	0.049324
rs13099317	LOC647276	3	186618142	3	4.59492
rs13099317	LOC647276	3	186618142	8	5.226738
rs13099317	LOC647276	3	186618142	5	3.824565
rs13099317	LOC647276	3	186618142	2	6.562023
rs13099317	LOC647276	3	186618142	7	2.983825
rs13099317	LOC647276	3	186618142	6	3.538055
rs13099317	LOC647276	3	186618142	4	4.264822
SNP3-18662919	MAP3K13	3	186629193	4	6.368631
rs2575	DGKG	3	187350085	6	3.869754
rs2575	DGKG	3	187350085	5	3.702217
rs2575	DGKG	3	187350085	2	3.791066
rs2574	DGKG	3	187350244	5	0.376573
rs2574	DGKG	3	187350244	6	4.851003
rs2574	DGKG	3	187350244	2	2.870521
rs1047115	FETUB	3	187841060	1	1.375841
rs1047115	FETUB	3	187841060	3	0.74843
SNP3-18787780	HRG	3	187877807	1	0.263236
SNP3-18787780	HRG	3	187877807	5	0.407267
SNP3-18787780	HRG	3	187877807	4	0.568679
SNP3-18787780	HRG	3	187877807	2	0.813086
rs1042445	HRG	3	187878130	4	1.494271
rs1042445	HRG	3	187878130	5	1.483679
rs1042445	HRG	3	187878130	2	2.42375
rs1042445	HRG	3	187878130	3	0.118665
rs1050274	KNG1	3	187918064	1	0.784035
rs4686837	ST6GAL1	3	188222371	3	0.178836
rs888390	RTP1	3	188401130	8	0.094662
rs1109452	MASP1	3	188435282	1	0.629607
rs850312	MASP1	3	188436502	1	2.248605
SNP3-18857135	RTP4	3	188571350	2	6.66839
SNP3-18857135	RTP4	3	188571350	1	0.173916

SNP3-188571350	RTP4	3	188571350	6	1.312533
SNP3-188571350	RTP4	3	188571350	7	0.291503
rs1533595	RTP4	3	188571597	4	7.630363
rs1533595	RTP4	3	188571597	3	0.693959
rs1533595	RTP4	3	188571597	1	4.542516
rs1533595	RTP4	3	188571597	6	5.129022
rs1533595	RTP4	3	188571597	5	0.645345
SNP3-188928905	BCL6	3	188928905	2	0.303711
SNP3-188928905	BCL6	3	188928905	8	0.534087
rs13096262	FLJ42393	3	189379606	5	3.320053
rs13096262	FLJ42393	3	189379606	1	2.074966
rs13096262	FLJ42393	3	189379606	6	2.425188
rs344952	FLJ42393	3	189381287	4	2.075259
rs344952	FLJ42393	3	189381287	3	2.326017
rs344952	FLJ42393	3	189381287	6	2.043333
rs344952	FLJ42393	3	189381287	5	1.720632
rs1136644	LPP	3	190073140	6	3.245493
rs1136644	LPP	3	190073140	4	0.845997
rs1136644	LPP	3	190073140	1	2.216343
rs1136644	LPP	3	190073140	7	0.707633
rs1136644	LPP	3	190073140	5	4.946322
rs1136644	LPP	3	190073140	8	1.447743
rs9847957	FAM79B	3	190372530	3	0.162259
rs1055152	FAM79B	3	190521342	8	0.196327
rs1055152	FAM79B	3	190521342	3	0.451173
rs9851974	FAM79B	3	190522305	4	0.362274
rs17489176	FAM79B	3	190523207	3	0.265814
rs9861864	FAM79B	3	190523798	8	0.491013
rs9861864	FAM79B	3	190523798	3	0.812747
rs9861864	FAM79B	3	190523798	2	3.08129
rs536435	LOC391603	3	191367563	2	3.908895
rs6788319	UTS2D	3	192482611	1	0.178292
rs6444535	UTS2D	3	192482684	1	0.548938
rs6444535	UTS2D	3	192482684	5	1.236154
rs35340712	CCDC50	3	192558596	1	2.765629
rs35340712	CCDC50	3	192558596	5	0.56959
rs1460924	FGF12	3	193343229	5	1.083187
rs1460924	FGF12	3	193343229	3	0.242917
rs1460923	FGF12	3	193343425	3	0.105455
rs1460923	FGF12	3	193343425	5	0.822159
rs11720096	FGF12	3	193343750	4	0.017109
rs11720096	FGF12	3	193343750	5	1.036328
rs11720096	FGF12	3	193343750	3	0.536155
rs10049459	FGF12	3	193343809	3	0.634783
rs10049459	FGF12	3	193343809	5	1.190302
rs10049459	FGF12	3	193343809	4	0.109196
rs1460922	FGF12	3	193344215	5	1.416884

rs1460922	FGF12	3	193344215	3	0.693188
rs2271791	ATP13A5	3	194477237	8	1.190625
rs2271791	ATP13A5	3	194477237	5	0.157671
rs6797429	ATP13A5	3	194563108	6	2.202814
rs6797429	ATP13A5	3	194563108	5	1.143304
rs6797429	ATP13A5	3	194563108	2	4.663644
rs6797429	ATP13A5	3	194563108	8	0.331371
rs6797429	ATP13A5	3	194563108	3	0.423596
rs6797429	ATP13A5	3	194563108	4	2.877912
rs35424709	ATP13A4	3	194654673	6	0.234549
rs3796181	ATP13A4	3	194666511	1	3.818838
rs3796181	ATP13A4	3	194666511	5	0.89406
rs3796181	ATP13A4	3	194666511	7	0.423953
SNP3-19481937(	OPA1	3	194819370	5	1.667061
rs11713634	CPN2	3	195543273	4	4.850522
rs3732477	CPN2	3	195544214	5	2.464583
rs15304	LSG1	3	195842965	2	0.8307
rs15304	LSG1	3	195842965	7	6.963552
rs15304	LSG1	3	195842965	6	0.285104
rs35480846	LSG1	3	195854753	5	9.209285
rs1773218	FAM43A	3	195888309	7	1.046332
rs1773218	FAM43A	3	195888309	2	5.526954
rs3752798	FAM43A	3	195888694	5	0.138423
rs9870815	MUC20	3	196938876	7	2.174263
rs9870815	MUC20	3	196938876	2	1.006527
SNP3-196947657	MUC20	3	196947657	4	3.439183
rs2291652	MUC4	3	196963462	3	1.177499
rs2291652	MUC4	3	196963462	5	0.952577
rs2291652	MUC4	3	196963462	4	0.923398
rs2291652	MUC4	3	196963462	1	1.040636
rs2246901	MUC4	3	196974680	3	0.304393
rs2550240	MUC4	3	196981550	7	0.549885
rs2550240	MUC4	3	196981550	1	0.854612
rs2550240	MUC4	3	196981550	5	2.801824
rs2550240	MUC4	3	196981550	3	1.74439
rs2259102	MUC4	3	196982808	3	0.313853
rs2259292	MUC4	3	196986783	7	0.816403
rs2259292	MUC4	3	196986783	5	1.46054
rs2688513	MUC4	3	196990443	3	3.643648
SNP3-19700012	MUC4	3	197000121	7	0.436212
rs3749331	MUC4	3	197000700	5	0.421605
rs2177336	MUC4	3	197001273	7	5.425425
rs2177336	MUC4	3	197001273	3	0.40786
rs1806440	MUC4	3	197001519	5	0.885806
rs1106502	MUC4	3	197001653	3	0.947543
rs1106502	MUC4	3	197001653	7	0.787384
rs1104760	MUC4	3	197001716	3	1.126149

rs1104760	MUC4	3	197001716	7	0.692476
rs882605	MUC4	3	197001948	3	0.55896
rs13433937	TNK2	3	197075453	4	1.536763
SNP3-19707886[illegible]	TNK2	3	197078865	8	1.172413
SNP3-19707886[illegible]	TNK2	3	197078865	3	0.997922
SNP3-19707886[illegible]	TNK2	3	197078865	6	0.168529
SNP3-19707886[illegible]	TNK2	3	197078865	7	1.021136
SNP3-19707886[illegible]	TNK2	3	197078865	5	0.332135
SNP3-19707886[illegible]	TNK2	3	197078865	4	1.947248
SNP3-19707886[illegible]	TNK2	3	197078865	2	8.142344
SNP3-19707886[illegible]	TNK2	3	197078865	1	0.393772
rs1522394	ZDHHC19	3	197422574	3	0.811373
rs1522394	ZDHHC19	3	197422574	1	0.301707
rs36016914	TM4SF19	3	197535259	3	0.750289
rs6785339	TM4SF19	3	197538836	3	2.082017
rs6785339	TM4SF19	3	197538836	1	3.096716
rs6785339	TM4SF19	3	197538836	5	0.363873
rs6785339	TM4SF19	3	197538836	8	1.006755
rs6785339	TM4SF19	3	197538836	4	0.920182
rs11185525	UBXD7	3	197564561	2	5.336094
SNP3-19809749[illegible]	SENP5	3	198097498	3	0.15414
rs335824	LOC152217	3	198154309	3	0.276576
rs335824	LOC152217	3	198154309	2	5.904752
rs335824	LOC152217	3	198154309	1	0.398964
rs45625439	MFI2	3	198226640	7	0.081187
SNP3-19891643[illegible]	KIAA0226	3	198916433	3	0.616399
SNP3-19891643[illegible]	KIAA0226	3	198916433	1	4.912644
rs3205525	FYTTD1	3	198979731	7	0.057209
rs1273557	FYTTD1	3	198993495	2	6.018017
rs1273557	FYTTD1	3	198993495	7	1.638809
rs36078463	LRCH3	3	199063863	5	0.387955
rs17720399	ABCA11	4	409955	3	0.250435
rs13150531	PIGG	4	507622	8	5.359529
SNP4-510853	PIGG	4	510853	8	6.767512
SNP4-517677	PIGG	4	517677	8	7.015766
GA022032	ATP5I	4	658009	4	3.156196
rs2242237	CPLX1	4	769394	4	0.278509
rs2242237	CPLX1	4	769394	8	3.878696
rs2242237	CPLX1	4	769394	2	2.818616
rs1026738	GAK	4	848941	6	2.156795
rs35117553	DGKQ	4	950542	2	1.615055
rs4583705	SLC26A1	4	963036	1	4.212418
rs3796622	SLC26A1	4	973060	6	0.164379
rs3796622	SLC26A1	4	973060	1	1.616294
rs6836258	IDUA	4	986248	6	5.023711
rs6836258	IDUA	4	986248	7	0.39451
SNP4-986690	IDUA	4	986690	7	0.463979

rs17728127	RNF212	4	1056769	5	5.882386
rs2279279	SPON2	4	1154277	5	0.988884
rs2279279	SPON2	4	1154277	7	1.949385
rs2279279	SPON2	4	1154277	6	0.648777
rs2279279	SPON2	4	1154277	2	0.342572
SNP4-1155748	SPON2	4	1155748	6	0.638337
SNP4-1155748	SPON2	4	1155748	5	0.898917
SNP4-1155748	SPON2	4	1155748	7	0.254974
GA003867	SPON2	4	1155764	2	4.100789
rs1680030	C4orf42	4	1235500	6	5.777371
rs1680030	C4orf42	4	1235500	3	0.169085
rs1680030	C4orf42	4	1235500	7	5.306355
rs28522910	KIAA1530	4	1364774	3	0.921335
rs28522910	KIAA1530	4	1364774	5	1.210299
rs6599309	CRIPAK	4	1378115	5	0.121373
rs6599309	CRIPAK	4	1378115	3	0.661339
rs2247341	SLBP	4	1671115	5	4.657694
rs2247341	SLBP	4	1671115	1	4.392063
rs2247341	SLBP	4	1671115	4	1.114228
rs17802877	TMEM129	4	1690144	7	3.284176
rs17802877	TMEM129	4	1690144	1	4.838395
rs17802877	TMEM129	4	1690144	3	1.193651
rs17802877	TMEM129	4	1690144	4	0.278665
SNP4-1699354	TACC3	4	1699354	3	0.242631
SNP4-1699354	TACC3	4	1699354	5	0.579072
rs1063742	TACC3	4	1699458	7	0.184089
rs1063742	TACC3	4	1699458	3	0.894444
rs1063742	TACC3	4	1699458	4	4.577109
rs1063742	TACC3	4	1699458	1	4.338339
SNP4-1699751	TACC3	4	1699751	4	0.559394
SNP4-1699751	TACC3	4	1699751	7	0.167542
SNP4-1699751	TACC3	4	1699751	3	0.126877
SNP4-1699751	TACC3	4	1699751	1	4.912986
rs1063743	TACC3	4	1699786	7	5.668199
rs1063743	TACC3	4	1699786	3	0.546686
rs798757	TACC3	4	1700013	2	4.492861
rs11248073	TACC3	4	1707300	1	5.361089
rs11248073	TACC3	4	1707300	4	2.086488
rs11248073	TACC3	4	1707300	8	2.06728
rs11248073	TACC3	4	1707300	5	0.815529
rs11248073	TACC3	4	1707300	7	1.829722
rs28681468	LETM1	4	1793818	2	1.535316
rs10488835	WHSC1	4	1917122	5	1.426658
rs10488836	POLN	4	2130692	1	5.162923
rs9328764	POLN	4	2146252	1	4.420361
rs10011549	POLN	4	2164744	1	4.592297
rs10018786	POLN	4	2164822	1	2.397313

rs2353552	POLN	4	2179863	7	0.155638
rs2353552	POLN	4	2179863	3	0.053095
rs2353552	POLN	4	2179863	5	0.127504
rs1052682	MXD4	4	2221999	1	3.410751
rs2051561	ZFYVE28	4	2245633	5	0.786511
rs2051561	ZFYVE28	4	2245633	7	0.327515
rs661301	ZFYVE28	4	2275851	1	0.726575
rs661301	ZFYVE28	4	2275851	3	0.04619
rs661301	ZFYVE28	4	2275851	7	0.146301
rs17768776	ZFYVE28	4	2276057	1	0.985035
rs16843413	SH3BP2	4	2796710	7	0.747696
rs16843413	SH3BP2	4	2796710	4	0.212556
rs1263416	SH3BP2	4	2807561	8	0.439697
rs1263416	SH3BP2	4	2807561	7	0.030736
rs1263416	SH3BP2	4	2807561	3	0.597327
rs10021191	GRK4	4	2935493	2	2.014772
rs10021191	GRK4	4	2935493	7	1.716791
rs10021191	GRK4	4	2935493	3	8.775448
rs10021191	GRK4	4	2935493	4	0.050369
rs10021191	GRK4	4	2935493	5	0.973554
rs2960306	GRK4	4	2960297	3	2.791297
rs2960306	GRK4	4	2960297	2	3.387229
rs2960306	GRK4	4	2960297	7	3.18423
rs2960306	GRK4	4	2960297	5	2.787011
rs2960306	GRK4	4	2960297	4	2.906946
rs1024323	GRK4	4	2975841	7	2.330321
rs1024323	GRK4	4	2975841	4	2.856694
rs1024323	GRK4	4	2975841	5	2.038662
rs1024323	GRK4	4	2975841	3	2.259416
rs35605687	GRK4	4	2985351	3	1.277228
rs35605687	GRK4	4	2985351	5	1.647286
rs35605687	GRK4	4	2985351	1	2.92222
rs1801058	GRK4	4	3008948	7	5.093779
SNP4-3118368	HTT	4	3118368	7	3.312436
rs363125	HTT	4	3159345	6	1.00978
rs362304	HTT	4	3212070	2	3.640199
rs362304	HTT	4	3212070	6	6.849522
rs3213507	RGS12	4	3387643	3	0.02524
rs3213507	RGS12	4	3387643	2	0.848588
rs3213507	RGS12	4	3387643	7	0.855353
rs3748034	HGFAC	4	3415889	1	0.498162
rs6811423	DOK7	4	3464398	1	4.958227
rs6811423	DOK7	4	3464398	5	2.993357
rs6811423	DOK7	4	3464398	4	0.123844
rs6811856	DOK7	4	3464624	5	0.202723
rs6811856	DOK7	4	3464624	4	4.143254
rs6811856	DOK7	4	3464624	1	0.720468

rs6831659	DOK7	4	3464645	5	1.233222
rs6831659	DOK7	4	3464645	1	1.794866
rs6850908	DOK7	4	3464696	4	0.663482
rs6850908	DOK7	4	3464696	5	0.503049
rs6850908	DOK7	4	3464696	1	1.471832
rs11733064	DOK7	4	3465081	7	5.06458
rs1054662	DOK7	4	3465948	8	0.862414
rs1054662	DOK7	4	3465948	2	0.997092
rs1054662	DOK7	4	3465948	1	0.730598
SNP4-3489560	LRPAP1	4	3489560	6	0.089898
SNP4-3489560	LRPAP1	4	3489560	4	0.179527
SNP4-3489560	LRPAP1	4	3489560	7	0.749475
rs45554432	LRPAP1	4	3496440	4	0.257921
rs45554432	LRPAP1	4	3496440	7	0.589875
rs2916464	TMEM128	4	4300521	6	3.716464
rs2916464	TMEM128	4	4300521	2	0.240454
rs2916464	TMEM128	4	4300521	1	0.982582
rs2916464	TMEM128	4	4300521	5	1.133898
SNP4-4300785	TMEM128	4	4300785	6	0.318562
rs2272746	TMEM128	4	4300810	6	3.379863
rs2272746	TMEM128	4	4300810	5	1.840102
rs2272746	TMEM128	4	4300810	4	1.875536
rs2272746	TMEM128	4	4300810	2	3.59207
rs2272739	LYAR	4	4327376	7	0.450781
rs1048898	LYAR	4	4342733	2	2.101102
rs1048898	LYAR	4	4342733	4	3.216382
rs1048898	LYAR	4	4342733	3	0.792756
rs1048898	LYAR	4	4342733	1	0.045398
rs10002237	D4S234E	4	4470512	2	4.746781
rs16837029	STK32B	4	5383992	7	0.594938
rs2287576	EVC2	4	5615092	5	0.338064
rs2287576	EVC2	4	5615092	2	1.228319
rs4689278	EVC2	4	5741803	3	0.830099
rs4689278	EVC2	4	5741803	8	0.977761
rs4689278	EVC2	4	5741803	1	3.660913
rs4688962	EVC	4	5800862	5	0.001982
rs4688962	EVC	4	5800862	1	0.32999
rs4688962	EVC	4	5800862	6	4.780247
rs4688962	EVC	4	5800862	4	0.438413
rs4688962	EVC	4	5800862	2	4.943091
rs2279252	EVC	4	5851395	2	2.476187
rs1057052	CRMP1	4	5873639	7	0.709386
rs1057052	CRMP1	4	5873639	6	1.826649
rs1057052	CRMP1	4	5873639	5	0.032416
rs12331	CRMP1	4	5889414	4	1.04538
rs12331	CRMP1	4	5889414	2	2.059579
rs12331	CRMP1	4	5889414	5	4.434945

rs12331	CRMP1	4	5889414	7	7.152381
rs12331	CRMP1	4	5889414	1	0.437419
rs12331	CRMP1	4	5889414	3	1.931678
rs9457	WFS1	4	6355700	5	0.224947
rs9457	WFS1	4	6355700	8	0.226889
rs9457	WFS1	4	6355700	4	0.156467
rs9457	WFS1	4	6355700	3	0.107322
rs9457	WFS1	4	6355700	7	6.434613
rs9457	WFS1	4	6355700	2	0.121621
SNP4-6666957	MAN2B2	4	6666957	5	5.217422
rs4689490	MAN2B2	4	6670105	8	1.464913
rs11737783	S100P	4	6749565	2	6.223823
rs3822268	KIAA0232	4	6934415	2	1.092602
rs3822268	KIAA0232	4	6934415	3	0.155345
rs6841334	KIAA0232	4	6936197	1	0.042645
rs6841334	KIAA0232	4	6936197	6	0.950314
rs6841334	KIAA0232	4	6936197	3	0.155801
rs6841334	KIAA0232	4	6936197	7	0.791003
rs6841334	KIAA0232	4	6936197	2	1.58874
rs6828784	KIAA0232	4	6936716	4	0.566007
rs6828784	KIAA0232	4	6936716	5	0.028056
rs3756255	TBC1D14	4	7083825	6	1.136687
rs3756255	TBC1D14	4	7083825	2	4.362419
rs3756255	TBC1D14	4	7083825	8	0.400853
rs3756255	TBC1D14	4	7083825	7	0.056755
rs3756255	TBC1D14	4	7083825	3	0.070831
rs868489	MGC21874	4	7106154	3	0.482382
rs868489	MGC21874	4	7106154	2	1.650932
rs868489	MGC21874	4	7106154	7	1.025178
rs11937667	MGC21874	4	7107938	2	6.607241
rs11937667	MGC21874	4	7107938	7	0.206829
rs10937782	MGC21874	4	7108007	7	0.258876
rs4689833	SORCS2	4	7765390	3	0.936233
rs4689833	SORCS2	4	7765390	1	4.298505
rs4689833	SORCS2	4	7765390	5	1.996182
rs28531835	SORCS2	4	7767810	6	0.091338
rs2285781	SORCS2	4	7767912	6	2.386822
rs2285781	SORCS2	4	7767912	4	1.042456
rs2285781	SORCS2	4	7767912	7	2.67973
rs13442	SORCS2	4	7794183	7	1.594583
rs4689158	SORCS2	4	7794874	4	1.830629
rs4689158	SORCS2	4	7794874	3	0.760465
rs4689158	SORCS2	4	7794874	6	4.024841
rs4689158	SORCS2	4	7794874	5	0.839489
rs12642285	SORCS2	4	7794946	7	2.126173
rs2285763	AFAP1	4	7814204	7	2.27906
rs2285762	AFAP1	4	7814305	1	4.892335

rs2285762	AFAP1	4	7814305	4	0.556495
rs10155361	AFAP1	4	7815031	2	6.609073
rs10155361	AFAP1	4	7815031	6	0.21824
rs10155361	AFAP1	4	7815031	4	3.977628
rs10050114	AFAP1	4	7815401	4	0.779432
rs10050114	AFAP1	4	7815401	2	7.833623
rs10050114	AFAP1	4	7815401	5	5.41067
rs10050114	AFAP1	4	7815401	6	0.820807
rs2158274	AFAP1	4	7815416	7	4.770772
rs2158274	AFAP1	4	7815416	4	0.650366
rs2158274	AFAP1	4	7815416	1	5.135209
rs2285761	AFAP1	4	7821623	7	1.492867
rs4696886	ABLIM2	4	8018583	2	4.872311
rs2292735	ABLIM2	4	8018775	2	6.0087
rs2677605	SH3TC1	4	8257983	2	2.418804
rs2677605	SH3TC1	4	8257983	8	1.193643
rs2677605	SH3TC1	4	8257983	3	1.760219
rs2677605	SH3TC1	4	8257983	1	2.4471
rs2677605	SH3TC1	4	8257983	5	1.320939
rs2677605	SH3TC1	4	8257983	7	1.638453
rs1281138	SH3TC1	4	8270929	3	1.490519
rs1281148	SH3TC1	4	8280613	5	0.011904
rs1281148	SH3TC1	4	8280613	8	4.648317
rs1281148	SH3TC1	4	8280613	7	1.121771
rs1281148	SH3TC1	4	8280613	4	0.722737
rs1281148	SH3TC1	4	8280613	1	1.196076
rs1281148	SH3TC1	4	8280613	2	0.464433
rs1281151	SH3TC1	4	8281135	7	0.834282
rs1281151	SH3TC1	4	8281135	1	1.138216
rs1281151	SH3TC1	4	8281135	5	0.917452
rs1281151	SH3TC1	4	8281135	4	0.440114
rs8153	SH3TC1	4	8293640	8	4.800613
rs8153	SH3TC1	4	8293640	3	0.052936
rs708244	ACOX3	4	8419504	7	4.344384
rs3103097	NULL	4	8563612	6	0.509675
rs9685931	GPR78	4	8639923	7	0.503852
rs9799717	GPR78	4	8640163	7	0.530328
rs1131140	CPZ	4	8666996	7	4.140897
rs1131140	CPZ	4	8666996	3	1.692795
rs1050464	CPZ	4	8672286	3	1.159802
rs3733591	SLC2A9	4	9531228	2	0.791572
SNP4-9531268	SLC2A9	4	9531268	6	0.369514
rs3733589	SLC2A9	4	9596422	2	7.514545
rs6820230	SLC2A9	4	9636640	6	0.558223
rs6820230	SLC2A9	4	9636640	3	1.771473
rs6820230	SLC2A9	4	9636640	5	0.182662
rs13441	WDR1	4	9708438	3	0.052955

rs2109603	LOC643446	4	10982054	4	2.536364
rs2109603	LOC643446	4	10982054	6	1.969449
rs2109603	LOC643446	4	10982054	3	2.452158
rs13120194	LOC643446	4	10982126	5	5.212269
rs3822226	NKX3-2	4	13151772	1	0.258182
rs3822226	NKX3-2	4	13151772	2	1.295923
SNP4-13210435	FAM44A	4	13210435	4	5.287276
SNP4-13210435	FAM44A	4	13210435	6	5.191435
SNP4-13210435	FAM44A	4	13210435	1	5.36976
SNP4-13210435	FAM44A	4	13210435	7	6.926945
rs17745712	FAM44A	4	13213516	8	0.148017
rs17745712	FAM44A	4	13213516	6	6.809587
rs17745712	FAM44A	4	13213516	7	5.674094
rs12644869	LOC152742	4	13722865	5	1.688026
rs12644869	LOC152742	4	13722865	4	1.46659
rs12644869	LOC152742	4	13722865	7	0.627735
rs3846413	LOC152742	4	13749309	2	4.74524
rs4698714	LOC152742	4	13750963	4	1.439239
rs4698714	LOC152742	4	13750963	1	0.892419
rs4698714	LOC152742	4	13750963	5	0.229314
rs2314590	C1QTNF7	4	15054631	4	0.110567
rs2314590	C1QTNF7	4	15054631	7	1.614633
rs2314590	C1QTNF7	4	15054631	2	4.809739
rs13104095	C1QTNF7	4	15055109	1	3.11083
rs6815254	LOC285550	4	15301156	1	3.499098
rs6815254	LOC285550	4	15301156	5	2.103891
rs6815254	LOC285550	4	15301156	3	0.070731
rs2072313	FGFBP1	4	15546829	3	1.121211
rs758329	FGFBP2	4	15573583	5	0.385229
rs758329	FGFBP2	4	15573583	3	0.2588
rs758329	FGFBP2	4	15573583	6	0.708821
rs758329	FGFBP2	4	15573583	8	0.835136
rs758329	FGFBP2	4	15573583	4	0.62281
rs4698432	FGFBP2	4	15573861	7	1.213154
rs4698432	FGFBP2	4	15573861	3	1.977438
rs4698432	FGFBP2	4	15573861	5	1.270233
rs4698432	FGFBP2	4	15573861	1	5.661673
rs7686732	PROM1	4	15579299	1	3.284713
rs1046234	TAPT1	4	15773551	2	2.596857
rs872478	LDB2	4	16113282	5	1.127604
rs872478	LDB2	4	16113282	1	2.004325
rs699459	QDPR	4	17097170	3	0.137102
rs699459	QDPR	4	17097170	5	0.417395
rs2286771	KIAA1276	4	17252946	7	1.160117
rs2286771	KIAA1276	4	17252946	5	0.030139
rs7690457	C4orf30	4	17414477	5	0.43103
rs11941723	NCAPG	4	17421626	5	0.212738

rs2074974	NCAPG	4	17421713	8	0.214633
rs2074974	NCAPG	4	17421713	2	2.438637
rs2074974	NCAPG	4	17421713	6	0.45336
rs2074974	NCAPG	4	17421713	7	0.454261
rs2302212	NCAPG	4	17427983	6	1.896232
rs2302212	NCAPG	4	17427983	2	2.234311
rs2302212	NCAPG	4	17427983	7	1.23194
rs2302212	NCAPG	4	17427983	5	1.444789
rs2302212	NCAPG	4	17427983	1	1.594948
SNP4-17428793	NCAPG	4	17428793	2	3.7055
SNP4-17428793	NCAPG	4	17428793	1	3.820353
SNP4-17428793	NCAPG	4	17428793	3	4.455455
SNP4-17428793	NCAPG	4	17428793	6	3.248225
SNP4-17428793	NCAPG	4	17428793	5	1.687466
SNP4-17428793	NCAPG	4	17428793	4	2.422777
rs7688403	NCAPG	4	17454336	2	8.047567
rs7688403	NCAPG	4	17454336	6	0.556052
rs7688403	NCAPG	4	17454336	1	0.025208
rs7688403	NCAPG	4	17454336	7	0.033357
rs7688403	NCAPG	4	17454336	5	0.212185
rs7655084	SLIT2	4	19864404	5	0.680041
rs7655084	SLIT2	4	19864404	7	2.417361
rs7655084	SLIT2	4	19864404	3	0.609299
rs7690492	SLIT2	4	20153282	5	0.468189
rs1379659	SLIT2	4	20229781	2	2.595476
rs1379659	SLIT2	4	20229781	7	0.650423
rs17552929	C4orf28	4	20338064	8	5.86135
rs12640448	KCNIP4	4	20340430	1	3.465062
rs12640448	KCNIP4	4	20340430	5	1.616448
rs12640448	KCNIP4	4	20340430	6	0.179654
rs3765122	KCNIP4	4	20461342	8	1.634331
rs3765122	KCNIP4	4	20461342	2	5.363426
rs3765122	KCNIP4	4	20461342	7	0.803896
rs870368	KCNIP4	4	21155315	3	1.042093
rs870368	KCNIP4	4	21155315	5	1.149339
rs966045	UM9(5)	4	21457180	3	0.023839
rs1513572	UM9(5)	4	21459403	3	0.264481
rs6821591	PPARGC1A	4	23406098	4	3.25514
rs6821591	PPARGC1A	4	23406098	5	4.094298
rs6821591	PPARGC1A	4	23406098	6	1.251171
rs3755863	PPARGC1A	4	23424620	4	0.062994
rs3755863	PPARGC1A	4	23424620	2	3.127691
rs3755863	PPARGC1A	4	23424620	6	4.441472
rs3755863	PPARGC1A	4	23424620	5	0.789435
rs8192678	PPARGC1A	4	23424760	1	0.472355
rs8192678	PPARGC1A	4	23424760	4	0.67181
rs8192678	PPARGC1A	4	23424760	6	0.499859

rs17574213	PPARGC1A	4	23424779	4	1.379206
rs2970847	PPARGC1A	4	23425022	2	3.893989
rs2970847	PPARGC1A	4	23425022	7	0.267255
SNP4-24410413	SOD3	4	24410413	6	1.041095
SNP4-24410413	SOD3	4	24410413	2	1.779144
GA022147	SOD3	4	24411074	6	2.632321
GA022147	SOD3	4	24411074	2	0.48666
rs12510565	DKFZp761B107	4	24440301	6	0.349309
rs16876184	DKFZp761B107	4	24440548	5	0.837849
rs12511680	DKFZp761B107	4	24441407	6	0.283854
rs16876189	DKFZp761B107	4	24441822	5	0.437146
rs8235	LGI2	4	24612601	3	0.174829
rs2232030	LGI2	4	24614069	3	1.521658
rs2232030	LGI2	4	24614069	7	1.851108
rs2232030	LGI2	4	24614069	1	0.853398
rs2232030	LGI2	4	24614069	5	1.357968
rs2232029	LGI2	4	24614121	4	1.634611
rs2232029	LGI2	4	24614121	2	0.699112
rs2232025	LGI2	4	24614552	4	0.284407
SNP4-24641233	LGI2	4	24641233	5	2.036415
rs6834255	PI4K2B	4	24888418	6	0.009203
rs2305670	LOC389203	4	25524956	5	4.989396
rs2305670	LOC389203	4	25524956	1	4.471934
rs2305670	LOC389203	4	25524956	8	0.334035
rs1488268	FLJ45721	4	26818657	3	0.020879
rs13130950	FLJ45721	4	26818694	6	1.555473
rs1047012	PCDH7	4	30334794	4	0.480332
rs1047012	PCDH7	4	30334794	6	2.401456
rs1047012	PCDH7	4	30334794	1	4.111419
rs977931	PCDH7	4	30335085	6	0.689131
rs977931	PCDH7	4	30335085	1	4.861011
rs977931	PCDH7	4	30335085	4	1.764381
rs2133514	LOC645716	4	30822350	3	1.664786
rs4833069	CENTD1	4	35758273	4	0.341007
rs4634233	KIAA1239	4	37121710	4	0.167067
rs4634233	KIAA1239	4	37121710	3	0.511949
rs11724784	KIAA1239	4	37122280	3	0.468001
rs936587	KIAA1239	4	37125448	3	0.376701
rs936587	KIAA1239	4	37125448	7	0.679492
rs936587	KIAA1239	4	37125448	4	1.011245
rs10569	PGM2	4	37539780	7	3.948369
rs10569	PGM2	4	37539780	2	1.80973
rs6811863	PTTG2	4	37638581	5	3.745586
rs6811863	PTTG2	4	37638581	1	3.049134
rs6811863	PTTG2	4	37638581	3	1.69292
rs6811863	PTTG2	4	37638581	6	3.585967
rs6811863	PTTG2	4	37638581	2	3.213475

SNP4-37699689	TBC1D1	4	37699689	7	5.972377
rs2637713	FLJ13197	4	38303732	4	0.349162
rs2637713	FLJ13197	4	38303732	7	0.114673
rs2637713	FLJ13197	4	38303732	3	1.602698
rs11096955	TLR10	4	38452502	3	0.360723
rs11096957	TLR10	4	38452886	3	1.293061
rs10856838	TLR10	4	38453568	6	0.21683
rs10856838	TLR10	4	38453568	3	0.051644
rs10856838	TLR10	4	38453568	5	1.490963
rs10856839	TLR10	4	38453631	7	0.693704
rs10856839	TLR10	4	38453631	3	1.346524
rs10856839	TLR10	4	38453631	5	0.07463
rs10856839	TLR10	4	38453631	6	1.307481
rs4274855	TLR10	4	38453866	7	0.114296
rs5743565	TLR1	4	38482378	7	1.572896
rs3821985	TLR6	4	38506407	7	0.636628
rs3821985	TLR6	4	38506407	6	5.57988
rs3821985	TLR6	4	38506407	5	0.803029
rs3821985	TLR6	4	38506407	3	0.416514
rs3821985	TLR6	4	38506407	4	6.446559
rs3821985	TLR6	4	38506407	1	0.017836
rs3821985	TLR6	4	38506407	2	3.460969
SNP4-38664360	TMEM156	4	38664360	3	0.956755
SNP4-38676700	TMEM156	4	38676700	1	2.303035
rs2711941	KLHL5	4	38740557	4	1.051824
rs3733276	KLHL5	4	38793306	1	3.492274
rs3733276	KLHL5	4	38793306	3	0.471369
rs3733276	KLHL5	4	38793306	4	0.593156
rs4975017	KLB	4	39126624	1	3.941562
rs4975017	KLB	4	39126624	8	0.624028
rs4975017	KLB	4	39126624	3	1.138417
rs2260645	LOC401127	4	39159079	2	4.787752
rs2260645	LOC401127	4	39159079	4	3.284513
rs6838749	KRT18P25	4	39698436	2	0.766537
rs6838749	KRT18P25	4	39698436	6	1.086006
rs6838749	KRT18P25	4	39698436	7	3.651287
rs6838749	KRT18P25	4	39698436	5	1.271123
rs6838749	KRT18P25	4	39698436	1	0.804005
rs6838749	KRT18P25	4	39698436	4	0.768507
rs7686844	KRT18P25	4	39698906	5	1.240286
rs7686844	KRT18P25	4	39698906	4	0.478447
rs7686844	KRT18P25	4	39698906	7	1.461608
rs7686844	KRT18P25	4	39698906	1	1.499923
rs794001	N4BP2	4	39797957	4	0.566894
rs794001	N4BP2	4	39797957	3	0.618864
SNP4-39800185	N4BP2	4	39800185	7	1.648602
rs2245466	RHOH	4	39875241	2	4.811661

rs10022491	CHRNA9	4	40032665	4	0.184792
rs10009228	CHRNA9	4	40051179	6	0.137456
rs2261167	NSUN7	4	40505504	4	3.533249
rs9993759	LOC728136	4	41578240	2	0.070332
rs2660320	WDR21B	4	41678875	6	1.950894
rs2660320	WDR21B	4	41678875	4	0.533667
rs2660332	WDR21B	4	41679858	8	1.584689
rs2660332	WDR21B	4	41679858	4	2.479515
rs2581439	WDR21B	4	41681614	4	0.033235
rs13143747	KCTD8	4	43871717	7	0.946546
rs13143747	KCTD8	4	43871717	5	5.476316
rs12374299	GABRG1	4	45733502	3	0.141612
rs12374299	GABRG1	4	45733502	7	0.59726
rs976156	GABRG1	4	45780817	3	0.483007
rs6447517	GABRA4	4	46623503	3	0.200245
rs7660336	GABRA4	4	46624425	4	0.070118
SNP4-47209442	ATP10D	4	47209442	3	0.28685
SNP4-47209442	ATP10D	4	47209442	6	0.598358
SNP4-47209442	ATP10D	4	47209442	4	0.871968
SNP4-47209442	ATP10D	4	47209442	1	4.987812
SNP4-47209442	ATP10D	4	47209442	7	0.457697
SNP4-47254759	ATP10D	4	47254759	2	0.13934
SNP4-47254759	ATP10D	4	47254759	1	0.200088
SNP4-47254759	ATP10D	4	47254759	4	0.393125
SNP4-47254759	ATP10D	4	47254759	6	0.350528
rs16851681	ATP10D	4	47273728	4	0.524152
rs16851681	ATP10D	4	47273728	7	1.245806
rs16851681	ATP10D	4	47273728	3	0.853623
rs1316874	ATP10D	4	47283960	5	0.107149
rs1316874	ATP10D	4	47283960	3	0.463139
rs1316874	ATP10D	4	47283960	6	1.446227
rs4695253	CORIN	4	47291389	6	0.091103
rs4695253	CORIN	4	47291389	4	0.16565
rs4695253	CORIN	4	47291389	3	0.085272
rs12641823	CORIN	4	47291465	3	0.428381
rs12641823	CORIN	4	47291465	6	0.462522
rs12641823	CORIN	4	47291465	4	0.177898
rs12641823	CORIN	4	47291465	5	0.341265
rs3749585	CORIN	4	47292347	4	0.497217
rs3749585	CORIN	4	47292347	3	1.356089
rs3749585	CORIN	4	47292347	5	0.737668
rs3749585	CORIN	4	47292347	6	0.736349
rs3749584	CORIN	4	47292411	8	0.228444
rs3749584	CORIN	4	47292411	7	0.381137
rs2289433	CORIN	4	47534686	2	1.049062
rs2289433	CORIN	4	47534686	8	2.170402
rs2289433	CORIN	4	47534686	1	1.483871

rs28642966	CNGA1	4	47640052	7	0.69631
rs28642966	CNGA1	4	47640052	3	1.065111
rs28642966	CNGA1	4	47640052	4	0.081784
rs10938519	NPAL1	4	47733341	1	4.739385
rs1507924	TXK	4	47764044	4	3.438313
rs9996527	TXK	4	47764453	4	0.692004
rs9996527	TXK	4	47764453	3	0.926821
SNP4-47776852	TXK	4	47776852	2	3.453725
rs7658300	TXK	4	47810021	7	0.452858
rs7658300	TXK	4	47810021	3	0.802325
rs7658300	TXK	4	47810021	4	1.065002
rs7658300	TXK	4	47810021	2	4.292982
rs3805184	TEC	4	47832992	4	1.915156
rs3805184	TEC	4	47832992	8	0.416513
rs3805184	TEC	4	47832992	1	5.392638
rs17574371	TEC	4	47842066	7	0.102846
rs17574371	TEC	4	47842066	3	0.932284
rs3811759	SLC10A4	4	48185767	6	0.026551
rs3811759	SLC10A4	4	48185767	5	0.183788
rs3811759	SLC10A4	4	48185767	3	1.694327
rs17472127	ZAR1	4	48191125	3	0.03302
rs17472127	ZAR1	4	48191125	5	0.073181
rs3747690	FLJ21511	4	48683207	2	2.623663
rs3747690	FLJ21511	4	48683207	5	0.036782
rs13531	DCUN1D4	4	52474805	3	0.154681
rs13531	DCUN1D4	4	52474805	7	3.97095
rs13531	DCUN1D4	4	52474805	6	0.490245
rs13531	DCUN1D4	4	52474805	4	0.184091
rs13531	DCUN1D4	4	52474805	2	1.008232
rs1519588	LOC339977	4	52554913	3	0.723941
rs1519588	LOC339977	4	52554913	7	1.296437
rs1519588	LOC339977	4	52554913	6	1.066525
rs170424	SGCB	4	52582783	7	0.655335
rs170424	SGCB	4	52582783	3	0.823094
rs6835977	SCFD2	4	53434429	6	3.243251
rs1351514	SCFD2	4	53926867	1	5.455132
rs1351514	SCFD2	4	53926867	3	0.496431
rs1014056	LNX1	4	54020747	3	0.448384
rs1014056	LNX1	4	54020747	1	3.822842
rs17084051	LOC442108	4	54782338	3	0.727918
SNP4-54834528	PDGFRA	4	54834528	8	7.238384
SNP4-54834528	PDGFRA	4	54834528	3	0.181636
rs2228230	PDGFRA	4	54846797	8	6.93805
rs2228230	PDGFRA	4	54846797	3	0.745756
rs2228230	PDGFRA	4	54846797	5	6.248804
rs1870377	KDR	4	55667731	7	7.020934
rs1870377	KDR	4	55667731	1	5.442348

rs576732	SRD5A3	4	55931894	4	0.042859
rs576732	SRD5A3	4	55931894	2	5.485568
rs576732	SRD5A3	4	55931894	8	0.257855
rs11658	EXOC1	4	56465846	5	0.457965
SNP4-56515169	CEP135	4	56515169	2	3.315588
SNP4-56906109	AASDH	4	56906109	5	9.507191
SNP4-56906109	AASDH	4	56906109	2	7.084802
SNP4-56906109	AASDH	4	56906109	8	5.603352
SNP4-56943473	AASDH	4	56943473	8	1.413675
rs2030364	LOC644196	4	56980666	5	0.428251
rs2030364	LOC644196	4	56980666	2	2.701994
rs3822001	PPAT	4	56996507	5	0.04921
rs3822001	PPAT	4	56996507	2	2.162093
rs3822001	PPAT	4	56996507	8	1.517087
rs17086879	SRP72	4	57051345	8	2.206086
rs34419325	SRP72	4	57056310	4	0.586781
rs4429779	HOPX	4	57217403	8	1.241224
rs4429779	HOPX	4	57217403	6	0.286017
rs3796529	REST	4	57492171	7	0.143909
rs3796529	REST	4	57492171	3	0.262567
rs6821028	NULL	4	61976044	8	4.414548
rs6821028	NULL	4	61976044	6	0.17471
rs6821028	NULL	4	61976044	5	0.212836
rs6821028	NULL	4	61976044	4	0.911158
rs7692496	CENPC1	4	68020604	5	0.195332
rs7692496	CENPC1	4	68020604	8	1.416817
rs7692496	CENPC1	4	68020604	7	0.336293
rs1056787	CENPC1	4	68062665	1	6.17804
rs13149974	STAP1	4	68125563	4	0.090211
rs1054593	STAP1	4	68155023	5	0.497997
rs1054593	STAP1	4	68155023	6	1.165368
rs1038426	GNRHR	4	68286130	4	1.248196
rs1038426	GNRHR	4	68286130	3	0.831931
rs1038426	GNRHR	4	68286130	1	4.155088
rs1038427	GNRHR	4	68286297	3	1.220734
rs1038427	GNRHR	4	68286297	1	3.894779
rs1038427	GNRHR	4	68286297	4	0.407335
rs1038427	GNRHR	4	68286297	7	1.508104
rs13150734	GNRHR	4	68287533	3	1.068774
rs13150734	GNRHR	4	68287533	4	0.28531
rs13150734	GNRHR	4	68287533	1	4.387529
rs17635850	GNRHR	4	68287964	3	0.149626
rs17635850	GNRHR	4	68287964	1	3.786172
rs4986942	GNRHR	4	68302196	1	5.716357
rs2062302	GNRHR	4	68303903	3	0.358936
rs2062302	GNRHR	4	68303903	4	0.689054
rs2062301	GNRHR	4	68303927	3	0.342799

rs2062301	GNRHR	4	68303927	4	7.262434
rs2062301	GNRHR	4	68303927	7	1.861568
rs13138607	GNRHR	4	68304145	3	0.044069
rs13138607	GNRHR	4	68304145	4	0.377518
rs353163	TMPRSS11A	4	68467369	3	1.312803
rs353163	TMPRSS11A	4	68467369	1	1.607219
rs1370840	TMPRSS11A	4	68492841	4	0.078681
rs1438444	TMPRSS11F	4	68601691	4	4.670597
rs10018943	TMPRSS11F	4	68602212	4	0.351493
rs3819257	SYT14L	4	68611132	4	0.511543
rs2319797	TMPRSS11B	4	68777102	4	0.767138
rs1715081	YTHDC1	4	68859393	7	2.03442
rs1715081	YTHDC1	4	68859393	4	5.171979
rs10033058	YTHDC1	4	68860003	7	1.076686
rs10033058	YTHDC1	4	68860003	4	1.281169
rs10033058	YTHDC1	4	68860003	6	1.955369
rs3100	UGT2B15	4	69547273	1	2.051717
rs2018024	LOC642381	4	69897507	7	2.750087
rs2018024	LOC642381	4	69897507	8	3.27375
rs2292092	SULT1B1	4	70627379	2	0.552354
rs2292092	SULT1B1	4	70627379	3	0.949244
rs2292092	SULT1B1	4	70627379	1	0.394143
SNP4-71058688	C4orf40	4	71058688	4	3.076363
SNP4-71058688	C4orf40	4	71058688	5	3.137709
rs3196714	ODAM	4	71103078	5	1.023451
rs777793	C4orf7	4	71135522	3	0.368562
rs777793	C4orf7	4	71135522	4	0.095175
rs777793	C4orf7	4	71135522	6	1.418613
SNP4-71266977	SMR3A	4	71266977	5	1.896058
SNP4-71266977	SMR3A	4	71266977	4	3.681294
SNP4-71266977	SMR3A	4	71266977	6	3.54478
SNP4-71266977	SMR3A	4	71266977	3	3.035755
rs3733493	SMR3B	4	71290356	3	1.539795
rs3733493	SMR3B	4	71290356	6	4.447145
rs3733493	SMR3B	4	71290356	4	3.746119
rs2306948	MUC7	4	71374317	6	1.83292
rs2306948	MUC7	4	71374317	4	1.346097
rs6826961	MUC7	4	71381290	6	1.875812
rs6826961	MUC7	4	71381290	4	2.591261
rs3733491	MUC7	4	71382443	6	2.694254
rs7660807	AMTN	4	71423140	5	0.030805
rs7660807	AMTN	4	71423140	7	0.091024
rs17676820	AMTN	4	71431578	3	0.37441
rs17676820	AMTN	4	71431578	4	0.413074
rs35266919	AMBN	4	71503574	1	0.533921
rs35266919	AMBN	4	71503574	3	0.24299
rs3733488	UTP3	4	71773139	2	4.339859

rs6836323	MOBKL1A	4	72070540	5	6.433622
rs1043218	SLC4A4	4	72655099	2	0.921448
rs788908	ADAMTS3	4	73633150	6	0.014955
rs788908	ADAMTS3	4	73633150	5	0.274688
rs788908	ADAMTS3	4	73633150	4	1.42854
rs1052080	COX18	4	74139486	6	1.732683
rs1052080	COX18	4	74139486	7	4.261914
rs1052080	COX18	4	74139486	8	4.627883
rs1052080	COX18	4	74139486	3	0.900694
rs2367288	LOC728045	4	74932962	3	1.12165
rs2367288	LOC728045	4	74932962	1	0.957861
rs2367288	LOC728045	4	74932962	7	0.88875
SNP4-74939062	PF4V1	4	74939062	5	0.938338
rs352047	CXCL5	4	75080954	2	0.57421
rs425535	CXCL5	4	75082861	2	4.890036
rs16850883	MTHFD2L	4	75387649	5	0.131331
rs16850883	MTHFD2L	4	75387649	4	4.886672
rs16850883	MTHFD2L	4	75387649	3	1.028456
rs16850883	MTHFD2L	4	75387649	6	5.032118
rs1017733	EREG	4	75471214	4	0.319983
rs1017733	EREG	4	75471214	8	0.696809
rs1017733	EREG	4	75471214	1	4.733044
SNP4-75938541	BTC	4	75938541	6	0.420731
rs9090	DKFZP564O0823	4	76191437	2	5.868749
rs890289	DKFZP564O0823	4	76192056	2	4.085356
rs1043633	DKFZP564O0823	4	76192854	2	4.677611
rs2306175	C4orf26	4	76708369	1	4.642688
rs2306175	C4orf26	4	76708369	3	0.782471
rs2306174	C4orf26	4	76708606	1	2.208897
rs41282391	USO1	4	76949225	3	1.211123
rs2242470	SDAD1	4	77097712	3	1.631035
rs2242470	SDAD1	4	77097712	6	0.985167
rs9994667	CXCL11	4	77174272	3	1.085586
rs1128864	ART3	4	77252614	3	0.03525
rs17001890	FLJ25770	4	77524370	4	0.121838
rs17001890	FLJ25770	4	77524370	3	1.227872
SNP4-77850449	SHROOM3	4	77850449	3	0.099568
SNP4-77850449	SHROOM3	4	77850449	4	3.448998
rs344140	SHROOM3	4	77879186	7	0.462928
rs344140	SHROOM3	4	77879186	2	0.330676
rs344140	SHROOM3	4	77879186	4	0.412816
rs344140	SHROOM3	4	77879186	1	0.41302
rs344140	SHROOM3	4	77879186	6	0.081739
rs344141	SHROOM3	4	77879755	1	0.684508
rs344141	SHROOM3	4	77879755	3	0.203802
rs2703130	ANKRD56	4	78036898	4	0.215714
rs873090	11-Sep	4	78090040	2	5.480876

rs873090	11-Sep	4	78090040	5	0.286923
rs873090	11-Sep	4	78090040	8	2.646248
rs873090	11-Sep	4	78090040	4	2.808742
rs4150050	CCNG2	4	78298719	1	6.395894
rs4150052	CCNG2	4	78298796	7	0.295034
rs1052563	CXCL13	4	78751256	5	0.405411
rs28695846	MRPL1	4	79003037	1	3.121064
rs12504081	FRAS1	4	79424723	5	0.015644
rs12504081	FRAS1	4	79424723	7	5.219971
rs12504081	FRAS1	4	79424723	2	4.877759
rs12504081	FRAS1	4	79424723	3	0.912422
rs12504081	FRAS1	4	79424723	8	0.218169
rs12504081	FRAS1	4	79424723	1	0.489602
rs345514	FRAS1	4	79457673	2	2.399716
rs345514	FRAS1	4	79457673	6	0.598163
rs6851427	FRAS1	4	79604743	1	3.831971
rs6851427	FRAS1	4	79604743	8	0.456092
rs6851427	FRAS1	4	79604743	7	0.646423
rs11098194	FRAS1	4	79619892	7	0.510486
rs11098194	FRAS1	4	79619892	8	0.803885
rs11098194	FRAS1	4	79619892	1	4.553622
rs3749487	FRAS1	4	79656179	6	4.813376
rs3749487	FRAS1	4	79656179	2	5.68065
rs931605	FRAS1	4	79662861	4	0.159172
rs931605	FRAS1	4	79662861	1	6.306343
rs931605	FRAS1	4	79662861	7	0.554144
rs3749485	FRAS1	4	79681584	1	4.839793
rs3749485	FRAS1	4	79681584	7	0.01947
rs3749485	FRAS1	4	79681584	6	0.098685
rs1564458	PAQR3	4	80059980	2	3.816837
rs13140055	ANTXR2	4	81212820	6	0.053874
rs13140055	ANTXR2	4	81212820	7	0.611138
rs13140055	ANTXR2	4	81212820	1	1.726744
rs11557908	ANTXR2	4	81213057	4	1.345744
rs11557908	ANTXR2	4	81213057	1	3.395713
rs11557908	ANTXR2	4	81213057	7	0.193693
rs2292842	PRDM8	4	81344005	2	1.029968
rs2292842	PRDM8	4	81344005	7	0.529555
rs2288338	HNRPD	4	83493658	8	0.263751
rs2288338	HNRPD	4	83493658	4	0.568205
GA004127	HNRPDL	4	83569978	8	0.891939
rs3733227	SCD5	4	83801235	1	6.356442
rs3733227	SCD5	4	83801235	6	3.695817
rs3733227	SCD5	4	83801235	5	0.706101
rs3733227	SCD5	4	83801235	7	0.482539
rs6830422	LIN54	4	84067989	6	1.311453
rs6830422	LIN54	4	84067989	1	0.15602

rs4396979	LOC391674	4	84387677	1	2.925052
rs4396979	LOC391674	4	84387677	6	0.230469
rs4396979	LOC391674	4	84387677	3	0.503599
rs348700	LOC152845	4	85385044	4	0.829675
rs348700	LOC152845	4	85385044	7	4.815823
rs348700	LOC152845	4	85385044	8	2.314893
rs348700	LOC152845	4	85385044	2	0.755191
rs7681742	WDFY3	4	85949776	7	4.752056
rs346498	ARHGAP24	4	87070584	2	3.088851
rs346498	ARHGAP24	4	87070584	3	0.843359
rs346498	ARHGAP24	4	87070584	7	1.104857
rs934648	ARHGAP24	4	87142476	4	1.485087
rs934648	ARHGAP24	4	87142476	3	0.558649
rs958	MAPK10	4	87156898	8	3.736844
rs958	MAPK10	4	87156898	7	0.914028
rs958	MAPK10	4	87156898	1	2.531806
rs958	MAPK10	4	87156898	2	0.786017
rs13106574	SLC10A6	4	87988953	1	1.383316
rs13106574	SLC10A6	4	87988953	4	1.024505
rs13106574	SLC10A6	4	87988953	7	0.246714
SNP4-88028044	C4orf36	4	88028044	3	0.336996
rs340638	AFF1	4	88147228	6	0.84537
rs340638	AFF1	4	88147228	8	3.806259
rs6531985	HSD17B11	4	88512911	2	1.188885
rs6531985	HSD17B11	4	88512911	1	0.16185
rs6531985	HSD17B11	4	88512911	5	1.428801
rs6531985	HSD17B11	4	88512911	4	0.819672
rs9933	SPARCL1	4	88620640	2	3.100423
rs9933	SPARCL1	4	88620640	5	0.181813
rs9933	SPARCL1	4	88620640	3	0.29799
rs8342	SPARCL1	4	88635247	2	0.767959
rs8342	SPARCL1	4	88635247	5	0.273513
rs8342	SPARCL1	4	88635247	3	0.396309
rs1049539	SPARCL1	4	88669372	1	3.088839
rs1049539	SPARCL1	4	88669372	4	0.764997
rs10019009	DMP1	4	88802159	8	4.431502
rs13144371	IBSP	4	88951770	1	0.589814
rs13144371	IBSP	4	88951770	2	0.135318
rs2231135	ABCG2	4	89299018	3	1.615724
rs3177413	PIGY	4	89662185	6	1.020857
rs3737488	HERC3	4	89826928	6	0.041191
rs7698491	HERC3	4	89826955	1	3.122085
rs2602122	NAP1L5	4	89836464	4	3.097175
rs28435799	NAP1L5	4	89836954	1	4.351567
rs1059122	FAM13A1	4	89866447	4	6.429021
rs1059122	FAM13A1	4	89866447	1	3.761986
rs1059122	FAM13A1	4	89866447	7	5.803758

rs1059122	FAM13A1	4	89866447	6	0.106932
rs1801915	FAM13A1	4	89872280	6	3.608048
rs7657817	FAM13A1	4	89887882	4	4.541724
rs7657817	FAM13A1	4	89887882	6	0.402505
rs1431550	GPRIN3	4	90387438	5	0.879492
rs1431550	GPRIN3	4	90387438	6	5.149186
rs1431550	GPRIN3	4	90387438	4	0.086861
rs1431550	GPRIN3	4	90387438	3	0.229379
rs1431550	GPRIN3	4	90387438	8	4.933198
rs1442138	MMRN1	4	91035317	1	2.887059
rs1442138	MMRN1	4	91035317	8	3.045407
rs11730314	MGC48628	4	91921845	4	0.10346
rs11730314	MGC48628	4	91921845	1	0.39881
rs7653977	LOC728394	4	92459301	3	0.496761
rs7653977	LOC728394	4	92459301	5	0.343108
rs7653977	LOC728394	4	92459301	1	0.182765
rs7653977	LOC728394	4	92459301	6	0.694894
rs6532289	LOC728394	4	92459825	5	4.276252
rs6532289	LOC728394	4	92459825	1	0.502759
rs6532289	LOC728394	4	92459825	6	4.142383
rs6532289	LOC728394	4	92459825	3	0.564429
rs6532289	LOC728394	4	92459825	2	0.21862
rs12642449	PDLIM5	4	95727778	3	0.554978
rs12642449	PDLIM5	4	95727778	8	3.789259
rs1043853	PDLIM5	4	95807297	3	0.604258
rs35120448	UNC5C	4	96385293	1	2.918957
rs783960	C4orf37	4	99112460	7	0.589737
rs783960	C4orf37	4	99112460	3	0.264738
rs783960	C4orf37	4	99112460	6	2.598178
rs783960	C4orf37	4	99112460	4	1.085287
rs783959	C4orf37	4	99112499	6	2.611521
rs783959	C4orf37	4	99112499	3	0.298108
rs783959	C4orf37	4	99112499	4	0.429867
rs783959	C4orf37	4	99112499	7	0.49575
rs17558193	C4orf37	4	99268616	6	0.395908
rs17558193	C4orf37	4	99268616	7	1.032399
rs17558193	C4orf37	4	99268616	3	0.995985
rs17558193	C4orf37	4	99268616	4	1.663835
rs4699605	C4orf37	4	99283402	4	1.439526
rs4699605	C4orf37	4	99283402	7	1.742566
rs4699605	C4orf37	4	99283402	6	0.6553
rs4699605	C4orf37	4	99283402	2	4.402594
rs4699605	C4orf37	4	99283402	3	0.493027
rs6810489	LOC728710	4	100096468	2	0.044224
rs6810489	LOC728710	4	100096468	5	1.320829
rs6810489	LOC728710	4	100096468	7	0.845669
rs6810489	LOC728710	4	100096468	4	0.451778

rs6810489	LOC728710	4	100096468	6	0.185051
rs1042365	ADH4	4	100264523	5	0.121884
rs1042365	ADH4	4	100264523	1	0.296246
rs1042364	ADH4	4	100264597	6	1.704714
rs1042364	ADH4	4	100264597	5	0.594509
rs1042364	ADH4	4	100264597	1	0.900922
rs1126673	ADH4	4	100264639	1	1.747851
rs1126673	ADH4	4	100264639	6	0.329776
rs1126673	ADH4	4	100264639	4	1.375817
rs1126673	ADH4	4	100264639	2	0.789073
rs1126672	ADH4	4	100266835	4	2.541975
rs1126672	ADH4	4	100266835	1	0.11302
rs1126672	ADH4	4	100266835	2	0.414581
rs1126672	ADH4	4	100266835	5	1.271755
rs1126672	ADH4	4	100266835	6	0.281386
rs1126671	ADH4	4	100267437	1	0.629355
rs1126671	ADH4	4	100267437	4	0.286602
rs1126671	ADH4	4	100267437	6	1.144378
rs1126670	ADH4	4	100271756	6	2.685507
rs1126670	ADH4	4	100271756	4	1.534675
rs1126670	ADH4	4	100271756	1	1.61522
rs1126670	ADH4	4	100271756	2	0.08004
rs2032349	ADH4	4	100281842	5	0.751278
rs698	ADH1C	4	100479812	3	0.21057
rs698	ADH1C	4	100479812	4	1.854268
rs698	ADH1C	4	100479812	2	4.507829
rs284787	ADH7	4	100552579	5	3.000805
rs894369	ADH7	4	100552869	4	1.518048
rs894369	ADH7	4	100552869	1	2.65665
rs3805331	ADH7	4	100552955	7	2.360265
rs284786	ADH7	4	100553000	5	0.472
rs4147554	ADH7	4	100553089	7	0.390053
rs971074	ADH7	4	100560884	1	6.246482
rs971074	ADH7	4	100560884	5	1.633431
rs13143848	C4orf17	4	100662743	8	0.862686
rs10026790	RG9MTD2	4	100703566	3	0.867843
rs10026790	RG9MTD2	4	100703566	1	5.104751
rs3816873	MTTP	4	100723687	1	0.083115
rs3816873	MTTP	4	100723687	3	0.13315
rs17599091	MTTP	4	100731942	1	1.296164
rs12641768	LOC285556	4	100777617	8	0.289658
rs4699384	LOC285556	4	100789704	1	0.579889
rs4699384	LOC285556	4	100789704	8	0.108886
rs1491242	LOC285556	4	100790024	8	0.252908
rs1491242	LOC285556	4	100790024	1	0.410286
rs715239	DAPP1	4	100957058	1	5.857885
rs715239	DAPP1	4	100957058	3	0.293866

rs15924	DAPP1	4	101009330	6	6.172142
rs17029673	LOC256880	4	101091388	3	0.901615
rs17029673	LOC256880	4	101091388	7	1.265184
rs17029673	LOC256880	4	101091388	8	0.162027
rs17029673	LOC256880	4	101091388	2	3.352596
rs1063821	DDIT4L	4	101326299	1	5.826174
rs1063821	DDIT4L	4	101326299	7	0.811535
rs1063821	DDIT4L	4	101326299	3	0.189814
rs2903209	DDIT4L	4	101326315	3	0.182523
rs2903209	DDIT4L	4	101326315	7	1.552687
rs2903209	DDIT4L	4	101326315	2	2.854749
rs3819185	DDIT4L	4	101326398	3	1.154136
rs3819185	DDIT4L	4	101326398	7	2.250667
rs3819184	DDIT4L	4	101326440	7	0.021274
rs3819184	DDIT4L	4	101326440	2	3.398755
rs3819184	DDIT4L	4	101326440	3	0.271156
rs3819183	DDIT4L	4	101326499	2	4.080672
rs3819183	DDIT4L	4	101326499	3	1.105825
rs3819182	DDIT4L	4	101326687	3	0.357182
rs3819182	DDIT4L	4	101326687	1	4.055454
rs17029766	DDIT4L	4	101326825	6	0.097784
rs17029766	DDIT4L	4	101326825	1	4.205403
rs17029766	DDIT4L	4	101326825	5	3.838133
rs11734827	DDIT4L	4	101327031	7	0.355202
rs11734827	DDIT4L	4	101327031	3	0.940063
rs11726613	DDIT4L	4	101327080	5	0.392157
rs11726613	DDIT4L	4	101327080	2	2.508714
rs11726613	DDIT4L	4	101327080	3	0.550377
rs11726613	DDIT4L	4	101327080	8	0.249157
rs11734833	DDIT4L	4	101327092	5	0.020484
rs11726694	DDIT4L	4	101327155	2	5.390819
rs11726694	DDIT4L	4	101327155	3	0.987354
rs11726694	DDIT4L	4	101327155	5	0.084
rs11726650	DDIT4L	4	101327214	2	4.648564
rs11726650	DDIT4L	4	101327214	5	0.165249
rs11726650	DDIT4L	4	101327214	3	0.962562
rs11726650	DDIT4L	4	101327214	8	1.876559
rs10007201	DDIT4L	4	101327355	3	0.247386
rs10007201	DDIT4L	4	101327355	8	0.113506
rs10007201	DDIT4L	4	101327355	5	0.518526
rs10007201	DDIT4L	4	101327355	1	4.50674
rs3749604	DDIT4L	4	101328184	3	0.381061
rs3749604	DDIT4L	4	101328184	2	5.179658
rs3749604	DDIT4L	4	101328184	7	1.419386
rs9990699	LOC728771	4	101952470	3	0.786509
rs9990699	LOC728771	4	101952470	4	3.542265
rs9990699	LOC728771	4	101952470	1	0.245171

rs10516486	BANK1	4	102970299	5	0.819199
rs10516486	BANK1	4	102970299	2	5.038591
rs10516486	BANK1	4	102970299	3	1.793288
rs3113676	BANK1	4	103184066	4	1.084766
rs6842661	BANK1	4	103214286	4	1.168751
rs6842661	BANK1	4	103214286	3	0.529103
rs223391	UBE2D3	4	103967359	6	0.941163
rs223391	UBE2D3	4	103967359	8	2.898582
rs223391	UBE2D3	4	103967359	5	4.739416
rs223391	UBE2D3	4	103967359	2	4.704457
rs3733632	TACR3	4	104860384	6	1.232918
rs4413407	CXXC4	4	105612810	2	4.801323
rs4413407	CXXC4	4	105612810	4	0.347218
rs17253672	KIAA1546	4	106375636	7	0.265614
rs7670522	KIAA1546	4	106379814	3	0.589655
rs7670522	KIAA1546	4	106379814	8	1.200127
rs13787	PPA2	4	106536878	3	0.104444
rs13787	PPA2	4	106536878	1	6.68771
rs13787	PPA2	4	106536878	7	0.399646
rs13787	PPA2	4	106536878	5	0.277805
rs3864227	LOC402182	4	106667201	5	1.636609
rs3864227	LOC402182	4	106667201	7	1.225378
rs3864227	LOC402182	4	106667201	6	1.890276
rs3864227	LOC402182	4	106667201	4	2.12472
rs4340795	NPNT	4	107081179	4	0.377441
rs4340795	NPNT	4	107081179	2	4.762398
rs4340795	NPNT	4	107081179	7	4
rs4340795	NPNT	4	107081179	5	1.370023
rs11733075	NPNT	4	107109803	7	0.923987
rs11733103	NPNT	4	107109819	7	0.499452
rs11735326	NPNT	4	107111343	7	1.069912
rs3733635	DKK2	4	108176354	8	1.257752
rs973126	PAPSS1	4	108860749	1	3.970911
rs3756271	CYP2U1	4	109093488	4	0.099287
rs3756271	CYP2U1	4	109093488	5	3.76411
rs3756271	CYP2U1	4	109093488	7	0.632763
rs3756271	CYP2U1	4	109093488	6	2.858918
rs3756271	CYP2U1	4	109093488	1	4.65679
rs3756271	CYP2U1	4	109093488	3	0.184535
rs3756271	CYP2U1	4	109093488	8	5.529991
rs4245927	LEF1	4	109189039	1	3.096919
rs4245927	LEF1	4	109189039	5	1.038217
rs7686569	FLJ37673	4	109697228	5	1.189846
rs7686569	FLJ37673	4	109697228	1	1.752722
rs7686569	FLJ37673	4	109697228	4	0.043214
rs7686569	FLJ37673	4	109697228	6	1.200074
rs3796934	FLJ37673	4	109761110	1	0.36317

rs7657133	RRH	4	110984821	3	1.703836
rs6839680	RRH	4	110984945	6	4.482305
rs6839680	RRH	4	110984945	5	0.418903
rs17040904	LRIT3	4	110992160	7	0.369773
rs4698797	LRIT3	4	110992516	3	0.302734
rs4698797	LRIT3	4	110992516	5	2.595188
rs7679048	LRIT3	4	111005739	3	0.239167
rs7679048	LRIT3	4	111005739	5	0.288071
rs7679048	LRIT3	4	111005739	2	2.667688
rs2347131	LRIT3	4	111010810	3	2.12101
rs4444903	EGF	4	111053559	4	0.880861
rs11568943	EGF	4	111102570	2	0.113311
rs11568943	EGF	4	111102570	3	8.550978
rs11568943	EGF	4	111102570	7	0.388979
rs11568993	EGF	4	111116764	2	3.662401
rs11568993	EGF	4	111116764	3	0.431802
rs11569017	EGF	4	111121560	7	5.97214
rs11569017	EGF	4	111121560	2	4.093527
rs11569017	EGF	4	111121560	3	0.145106
rs4698803	EGF	4	111133876	7	0.489766
rs4698803	EGF	4	111133876	4	0.373323
rs10004479	LOC391686	4	112085786	2	1.080264
rs3203648	TIFA	4	113417579	1	0.768347
rs4834267	LOC645237	4	113497960	6	0.032831
rs4834267	LOC645237	4	113497960	5	0.018599
rs4834267	LOC645237	4	113497960	4	0.548068
rs4834267	LOC645237	4	113497960	7	0.275668
rs1009475	LOC645237	4	113500196	2	1.282675
rs1009475	LOC645237	4	113500196	5	0.207325
rs1009475	LOC645237	4	113500196	1	1.080938
rs231253	ALPK1	4	113582071	8	0.460883
rs231253	ALPK1	4	113582071	6	1.965952
rs231253	ALPK1	4	113582071	7	8.585226
rs231253	ALPK1	4	113582071	1	6.273511
rs231253	ALPK1	4	113582071	5	0.106469
rs17605622	LOC91431	4	113701595	3	0.829033
rs17669218	C4orf21	4	113744172	8	0.830173
rs17669218	C4orf21	4	113744172	3	1.107575
SNP4-11375884(	C4orf21	4	113758840	8	3.670764
rs13104310	C4orf21	4	113773834	5	1.123027
rs13104310	C4orf21	4	113773834	8	2.222024
rs13104310	C4orf21	4	113773834	2	4.122494
rs13104310	C4orf21	4	113773834	1	0.183609
rs3733617	ANK2	4	114497726	8	0.524068
rs10013743	ANK2	4	114498871	2	3.41615
rs11731775	CAMK2D	4	114902163	7	0.868168
rs11731775	CAMK2D	4	114902163	1	2.157677

rs10028980	NDST3	4	119278737	3	0.056525
rs617430	NDST3	4	119381243	1	1.152218
rs617430	NDST3	4	119381243	5	0.043062
rs617430	NDST3	4	119381243	2	0.742247
SNP4-119439357	PRSS12	4	119439357	2	3.070212
SNP4-119439357	PRSS12	4	119439357	4	0.399162
rs28720014	CEP170L	4	119663904	1	2.812444
rs28720014	CEP170L	4	119663904	2	4.280234
rs28720014	CEP170L	4	119663904	7	6.028299
rs28720014	CEP170L	4	119663904	5	1.579058
rs28720014	CEP170L	4	119663904	4	2.765695
rs28720014	CEP170L	4	119663904	3	4.092793
rs17263971	SYNPO2	4	120167494	8	0.593098
rs17263971	SYNPO2	4	120167494	3	0.456439
rs17263971	SYNPO2	4	120167494	6	2.564177
rs17263971	SYNPO2	4	120167494	7	1.042002
rs7698598	SYNPO2	4	120171095	4	1.464003
SNP4-120172403	SYNPO2	4	120172403	1	4.864717
SNP4-120172403	SYNPO2	4	120172403	6	0.833278
SNP4-120172403	SYNPO2	4	120172403	5	0.558274
rs17050125	SYNPO2	4	120199949	3	0.276389
rs17050125	SYNPO2	4	120199949	1	1.151255
rs1058450	SYNPO2	4	120200088	1	0.270226
rs1058450	SYNPO2	4	120200088	7	1.39881
rs1058450	SYNPO2	4	120200088	6	0.72186
rs17050116	SYNPO2	4	120200744	3	0.870278
rs10021107	SYNPO2	4	120200812	3	0.961456
rs1047894	SYNPO2	4	120201276	3	0.855282
rs7687613	MYOZ2	4	120304896	1	3.349688
rs878372	FABP2	4	120458228	8	0.886309
rs878372	FABP2	4	120458228	2	1.578079
rs10212775	FABP2	4	120458692	6	0.172812
rs10212775	FABP2	4	120458692	2	1.31381
rs11724758	FABP2	4	120459326	1	0.656542
rs13124532	PDE5A	4	120635893	1	3.140434
rs17006190	PDE5A	4	120638286	2	3.438362
rs17006190	PDE5A	4	120638286	8	1.652522
rs1160185	SAR1P3	4	121482947	1	1.490989
rs1160185	SAR1P3	4	121482947	7	0.998451
rs12499000	PRDM5	4	121925651	5	1.559426
rs12499000	PRDM5	4	121925651	3	0.378411
rs12499000	PRDM5	4	121925651	7	0.652036
rs12499000	PRDM5	4	121925651	1	4.519256
rs343192	PRDM5	4	121957499	5	0.239823
rs343192	PRDM5	4	121957499	1	5.038532
rs343192	PRDM5	4	121957499	3	0.171333
rs343192	PRDM5	4	121957499	6	0.444993

rs343192	PRDM5	4	121957499	7	0.011703
rs2276959	C4orf31	4	122186257	1	0.524513
rs11098602	TNIP3	4	122272091	7	0.56453
rs11098602	TNIP3	4	122272091	1	1.626918
rs11098602	TNIP3	4	122272091	3	0.372185
rs6846553	TNIP3	4	122272673	1	2.928813
rs6846553	TNIP3	4	122272673	7	0.008144
SNP4-12228776	TNIP3	4	122287766	3	0.38771
rs17051298	TNIP3	4	122295189	3	0.103496
rs10000692	TNIP3	4	122297767	3	0.962966
rs2013534	LOC729109	4	122510581	1	0.917003
rs2013534	LOC729109	4	122510581	7	1.127615
rs2013534	LOC729109	4	122510581	8	1.471207
rs2013534	LOC729109	4	122510581	5	1.445631
rs692613	LOC729112	4	122590616	4	0.295936
rs692616	LOC729112	4	122590698	5	0.821686
rs1507996	BBS7	4	122965179	5	4.189241
rs1507996	BBS7	4	122965179	3	0.393521
rs1507996	BBS7	4	122965179	6	4.598449
rs1507996	BBS7	4	122965179	4	0.458751
rs11732666	TRPC3	4	123043502	2	3.712917
rs11732666	TRPC3	4	123043502	3	2.140456
rs7658836	KIAA1109	4	123365201	4	5.360046
rs7658836	KIAA1109	4	123365201	8	6.747584
SNP4-12341183	KIAA1109	4	123411833	8	6.974
SNP4-12341183	KIAA1109	4	123411833	4	1.178223
SNP4-12349641	KIAA1109	4	123496411	2	6.135632
SNP4-12349641	KIAA1109	4	123496411	1	1.169936
rs13134412	NUDT6	4	124063166	4	5.153496
rs13134412	NUDT6	4	124063166	8	1.27445
rs300572	SPRY1	4	124540374	4	0.698757
rs300572	SPRY1	4	124540374	3	0.55237
rs300572	SPRY1	4	124540374	5	0.488652
rs300572	SPRY1	4	124540374	6	3.805057
rs300572	SPRY1	4	124540374	2	5.888156
rs17009618	FAT4	4	126556153	6	0.9305
rs1567047	FAT4	4	126592192	4	0.384777
rs1567047	FAT4	4	126592192	5	0.124443
rs1567047	FAT4	4	126592192	8	1.892775
SNP4-12659242	FAT4	4	126592425	8	4.146928
rs1014866	FAT4	4	126632025	7	0.567915
rs1014866	FAT4	4	126632025	8	0.604653
rs1014867	FAT4	4	126632341	2	2.796785
rs313098	LOC132817	4	127705080	4	2.596035
rs313098	LOC132817	4	127705080	3	2.530014
rs313098	LOC132817	4	127705080	5	2.227076
rs313098	LOC132817	4	127705080	8	1.545001

rs3749507	INTU	4	128784450	3	0.624242
rs3749507	INTU	4	128784450	6	2.76704
rs3749507	INTU	4	128784450	5	0.448977
rs3749507	INTU	4	128784450	4	1.19135
rs3749507	INTU	4	128784450	8	5.472501
rs2305958	SLC25A31	4	128909386	5	3.110305
rs3811740	PLK4	4	129026669	4	0.037701
rs3811740	PLK4	4	129026669	5	0.039447
rs3811740	PLK4	4	129026669	8	0.610054
rs3811740	PLK4	4	129026669	6	3.5945
rs12645577	LARP2	4	129320093	5	0.128591
rs12645577	LARP2	4	129320093	6	0.317779
rs12645577	LARP2	4	129320093	8	0.423151
rs12645577	LARP2	4	129320093	4	4.015923
rs10028124	SCLT1	4	130086730	8	6.484413
rs10028124	SCLT1	4	130086730	5	1.365997
rs10028124	SCLT1	4	130086730	6	1.43201
rs1757935	C4orf33	4	130243209	4	0.480919
rs1757935	C4orf33	4	130243209	2	6.859951
rs2271570	C4orf33	4	130250094	8	0.751022
rs2271570	C4orf33	4	130250094	4	4.972689
rs3749589	PCDH10	4	134293508	5	0.712259
rs11099273	LOC132430	4	135340516	4	1.45511
rs11099273	LOC132430	4	135340516	3	0.847321
rs11099273	LOC132430	4	135340516	8	0.856694
rs2044116	LOC132430	4	135340818	4	0.087993
rs2044116	LOC132430	4	135340818	8	0.159834
rs2044116	LOC132430	4	135340818	3	0.795336
rs10009368	LOC132430	4	135341051	8	0.357573
rs10009368	LOC132430	4	135341051	3	0.977564
rs28568985	LOC389223	4	136186045	7	2.43264
rs28568985	LOC389223	4	136186045	1	3.826377
rs28568985	LOC389223	4	136186045	2	3.907734
rs17239836	LOC729578	4	137938839	3	4.109202
rs10018837	PCDH18	4	138669133	8	1.160766
rs10018837	PCDH18	4	138669133	3	0.511458
rs10018837	PCDH18	4	138669133	1	3.285215
rs10018837	PCDH18	4	138669133	7	1.349614
rs6838248	SLC7A11	4	139359944	5	6.713306
rs6838248	SLC7A11	4	139359944	7	6.689642
rs13137997	RAB33B	4	140614325	1	2.576804
rs7435309	SETD7	4	140649300	3	0.953923
rs1026049	SETD7	4	140649658	1	4.927458
rs1026049	SETD7	4	140649658	7	0.248284
rs1026049	SETD7	4	140649658	5	0.097918
rs2132845	MGST2	4	140806575	2	5.56735
rs11944843	MAML3	4	140857153	8	0.629204

rs3822043	MAML3	4	140859729	2	5.056079
rs3822043	MAML3	4	140859729	4	0.999495
rs3805317	CLGN	4	141529676	4	0.261503
rs2321270	LOC646484	4	141641767	7	3.364037
rs2270565	UCP1	4	141702921	4	0.365465
rs17006560	RNF150	4	142008646	1	5.932959
rs17006560	RNF150	4	142008646	3	0.345975
rs1057972	IL15	4	142873882	5	1.383308
rs1057972	IL15	4	142873882	2	1.658095
rs1057972	IL15	4	142873882	8	1.308642
rs1057972	IL15	4	142873882	7	0.065953
rs1057972	IL15	4	142873882	3	0.542109
rs13137565	LOC729675	4	144325148	8	1.102512
rs13137565	LOC729675	4	144325148	1	3.405669
rs13137565	LOC729675	4	144325148	3	2.354692
rs13137565	LOC729675	4	144325148	6	2.90021
rs13137565	LOC729675	4	144325148	7	2.031278
rs4240334	GYPE	4	145011955	6	0.863227
rs4240334	GYPE	4	145011955	8	1.339871
rs4240334	GYPE	4	145011955	1	0.890079
rs1808991	GYPE	4	145012409	3	6.046077
rs1808991	GYPE	4	145012409	6	4.635419
rs1808991	GYPE	4	145012409	1	4.783333
rs1808991	GYPE	4	145012409	4	4.725481
SNP4-14513816	GYPB	4	145138162	6	0.472945
SNP4-14513816	GYPB	4	145138162	4	0.171165
SNP4-14513816	GYPB	4	145138162	3	0.360168
SNP4-14513816	GYPB	4	145138162	1	0.149696
SNP4-14513816	GYPB	4	145138162	7	0.104647
rs1132787	GYPA	4	145249996	4	1.550794
rs11727676	HHIP	4	145878514	7	0.688402
rs13144151	NULL	4	146622615	3	0.296605
rs1390981	LOC729497	4	146766151	2	0.773796
rs1390981	LOC729497	4	146766151	1	3.714533
rs1390981	LOC729497	4	146766151	7	5.919093
rs1390981	LOC729497	4	146766151	3	4.25664
rs1390981	LOC729497	4	146766151	5	4.517205
rs4835011	MMAA	4	146779686	3	0.411262
rs10013280	TTC29	4	148008159	1	0.293735
rs10013280	TTC29	4	148008159	2	0.618965
rs10013280	TTC29	4	148008159	7	0.085419
rs10013280	TTC29	4	148008159	5	1.401951
rs35231662	TTC29	4	148008178	5	0.345056
rs35231662	TTC29	4	148008178	3	0.120036
rs1801708	EDNRA	4	148621819	8	1.054046
rs1801708	EDNRA	4	148621819	1	3.809746
rs5342	EDNRA	4	148684221	6	2.289621

rs5343	EDNRA	4	148684442	2	1.456233
rs5343	EDNRA	4	148684442	8	7.405183
rs10305937	EDNRA	4	148684876	2	4.107716
rs11557361	LOC90826	4	148779643	4	0.899859
rs2871	NR3C2	4	149219474	1	6.597664
rs2871	NR3C2	4	149219474	6	3.897256
rs2871	NR3C2	4	149219474	4	3.905225
rs5536	NR3C2	4	149220143	1	5.666671
rs17024360	NR3C2	4	149221533	1	4.99446
rs5525	NR3C2	4	149575966	6	5.282752
rs5525	NR3C2	4	149575966	8	0.78389
rs3749574	LRBA	4	151426577	6	7.365039
rs13105060	MAB21L2	4	151724772	8	4.691075
rs11735845	LRBA	4	151984786	6	0.873372
SNP4-15243193(	ESSPL	4	152431936	7	0.510799
SNP4-15243193(	ESSPL	4	152431936	6	1.921005
SNP4-15243193(	ESSPL	4	152431936	5	0.016239
SNP4-15243193(	ESSPL	4	152431936	8	4.730732
SNP4-15243193(	ESSPL	4	152431936	2	3.94682
rs17275536	LOC729830	4	152718075	8	1.221187
rs17275536	LOC729830	4	152718075	7	0.495931
rs17275536	LOC729830	4	152718075	3	0.190445
rs4696354	TIGD4	4	153910292	8	0.770932
rs4696354	TIGD4	4	153910292	6	1.31292
rs4696354	TIGD4	4	153910292	3	0.093975
rs4696354	TIGD4	4	153910292	7	0.191287
rs3811833	FHDC1	4	154115808	6	0.262454
rs3811833	FHDC1	4	154115808	5	0.321423
rs4696370	FHDC1	4	154117628	5	0.234167
rs4696370	FHDC1	4	154117628	6	0.01604
rs1059354	FHDC1	4	154119023	6	3.486737
rs1059354	FHDC1	4	154119023	5	0.516512
rs3749538	TRIM2	4	154476345	2	3.048398
rs3749538	TRIM2	4	154476345	5	1.419049
rs10001845	KIAA0922	4	154697672	3	0.071527
rs7669418	KIAA0922	4	154733077	8	2.70242
rs7669418	KIAA0922	4	154733077	5	0.298003
rs7669418	KIAA0922	4	154733077	7	0.594313
rs7669418	KIAA0922	4	154733077	4	4.168286
rs7669741	KIAA0922	4	154733172	4	1.062486
rs7669741	KIAA0922	4	154733172	5	0.89975
rs7669741	KIAA0922	4	154733172	8	0.996338
rs35627765	KIAA0922	4	154773327	6	3.343112
rs1339	RNF175	4	154851013	8	5.932899
rs1339	RNF175	4	154851013	3	0.181185
rs1339	RNF175	4	154851013	5	0.760492
rs1339	RNF175	4	154851013	4	3.027534

rs1337	RNF175	4	154851037	4	3.890745
rs1337	RNF175	4	154851037	6	1.47585
rs17031387	DCHS2	4	155438811	4	0.219049
rs11935573	DCHS2	4	155461022	1	4.010971
rs11935573	DCHS2	4	155461022	3	0.438893
rs11935573	DCHS2	4	155461022	4	0.341195
SNP4-15546118!	DCHS2	4	155461185	8	0.702593
rs6858157	DCHS2	4	155475627	3	2.839345
rs6858157	DCHS2	4	155475627	4	1.175272
rs6858157	DCHS2	4	155475627	8	1.494701
rs6858157	DCHS2	4	155475627	1	3.087652
rs7656522	DCHS2	4	155630515	2	2.718291
rs6056	FGB	4	155708271	2	2.463697
SNP4-15593863!	RBM46	4	155938639	3	0.174404
rs156502	RBM46	4	155939724	2	4.345212
rs156502	RBM46	4	155939724	3	0.431524
rs156502	RBM46	4	155939724	5	1.127915
rs1047214	NPY2R	4	156355126	5	2.923375
rs1047214	NPY2R	4	156355126	1	2.453928
rs1047214	NPY2R	4	156355126	2	3.421979
rs3088086	MAP9	4	156486311	1	1.689297
rs3088086	MAP9	4	156486311	3	0.633198
rs3088086	MAP9	4	156486311	5	0.24252
rs11099999	MAP9	4	156487303	3	0.120281
rs11099999	MAP9	4	156487303	5	0.085163
rs11099999	MAP9	4	156487303	1	5.900527
rs2305050	MAP9	4	156493218	5	0.384107
rs2305050	MAP9	4	156493218	1	5.253519
rs2305050	MAP9	4	156493218	3	1.167871
rs17377679	MAP9	4	156516510	1	5.080337
rs17377679	MAP9	4	156516510	6	1.574973
rs17377679	MAP9	4	156516510	4	0.276774
rs2305981	GUCY1B3	4	156940648	8	2.973211
rs2305981	GUCY1B3	4	156940648	2	2.857811
rs2305981	GUCY1B3	4	156940648	7	0.488903
rs11943855	hCG_1814936	4	157777176	3	0.377172
rs11943855	hCG_1814936	4	157777176	1	1.021411
rs10028490	C4orf18	4	159267052	2	1.239196
rs2009748	LOC646890	4	159418832	1	2.569219
rs12331717	LOC92345	4	164307509	5	0.962854
rs12331717	LOC92345	4	164307509	3	0.625986
rs4552421	NPY1R	4	164465113	1	5.26326
rs13306006	NPY1R	4	164473165	2	5.399456
rs12507653	LOC729743	4	164473783	3	1.315483
rs12507653	LOC729743	4	164473783	5	0.78881
rs12507653	LOC729743	4	164473783	1	0.153718
rs11735477	TKTL2	4	164612567	1	1.011451

rs2276977	FLJ11184	4	164635380	1	5.865448
rs1133505	1-Mar	4	164668558	1	0.332639
rs1133505	1-Mar	4	164668558	8	0.199059
rs1133505	1-Mar	4	164668558	3	0.702632
rs1133505	1-Mar	4	164668558	6	0.327201
rs1133505	1-Mar	4	164668558	5	0.07009
rs1133505	1-Mar	4	164668558	7	1.28455
rs1133505	1-Mar	4	164668558	2	1.235944
rs1133505	1-Mar	4	164668558	4	4.089223
rs6816526	1-Mar	4	164668723	3	0.612126
rs6816526	1-Mar	4	164668723	7	1.299493
rs6816526	1-Mar	4	164668723	5	4.435023
rs6816526	1-Mar	4	164668723	4	0.698932
rs6816526	1-Mar	4	164668723	6	3.27224
rs13130399	1-Mar	4	164686274	5	0.165532
rs13130399	1-Mar	4	164686274	3	0.758931
rs13130399	1-Mar	4	164686274	2	0.190333
rs13130399	1-Mar	4	164686274	4	0.918116
rs13130399	1-Mar	4	164686274	7	0.275303
rs13130399	1-Mar	4	164686274	8	0.498288
rs13130399	1-Mar	4	164686274	1	0.380984
rs7698990	LOC646954	4	165073820	6	3.405598
rs17584342	LOC653794	4	166057081	3	1.401145
rs17584342	LOC653794	4	166057081	1	2.164759
rs4359860	LOC391710	4	166079401	7	0.51985
rs4359860	LOC391710	4	166079401	3	0.437468
rs4359860	LOC391710	4	166079401	1	0.627192
rs10020204	LOC391711	4	166089273	1	0.498725
rs10034083	C4orf39	4	166098457	1	5.432783
rs10034083	C4orf39	4	166098457	7	0.993705
rs6536886	TRIM60	4	166172705	3	0.017303
rs6536886	TRIM60	4	166172705	6	0.527716
rs6536886	TRIM60	4	166172705	4	0.042865
rs6536886	TRIM60	4	166172705	7	0.464458
rs6819917	TRIM75	4	166200592	3	0.137321
rs17045936	TRIM75	4	166201120	3	1.201936
rs9999037	TMEM192	4	166219645	5	0.034589
rs2279723	TLL1	4	167241809	5	0.085172
rs2279723	TLL1	4	167241809	1	0.418972
rs2279723	TLL1	4	167241809	7	0.092074
rs1497949	TLL1	4	167244165	1	0.553756
rs1497949	TLL1	4	167244165	5	0.224512
rs1497948	TLL1	4	167244355	7	1.920339
rs1497948	TLL1	4	167244355	5	0.589603
rs1497948	TLL1	4	167244355	1	0.859927
rs1057377	SPOCK3	4	167892648	6	4.205304
rs1057377	SPOCK3	4	167892648	2	5.38296

rs1057377	SPOCK3	4	167892648	1	4.317852
rs12880	DDX60L	4	169514736	8	3.863916
rs12880	DDX60L	4	169514736	4	4.908875
rs12880	DDX60L	4	169514736	5	0.345949
SNP4-169537513	DDX60L	4	169537513	3	1.741752
SNP4-169537513	DDX60L	4	169537513	6	4.468953
SNP4-169579619	DDX60L	4	169579619	1	3.764702
SNP4-169579619	DDX60L	4	169579619	4	0.594814
SNP4-169579619	DDX60L	4	169579619	6	0.565928
SNP4-169579619	DDX60L	4	169579619	5	0.93437
rs2319850	DDX60L	4	169584979	1	6.125912
rs13151700	DDX60L	4	169599132	2	6.415919
rs13151700	DDX60L	4	169599132	6	3.008737
rs13151700	DDX60L	4	169599132	5	6.353896
rs1806729	PALLD	4	169669416	3	0.61066
SNP4-169843224	PALLD	4	169843224	4	0.382532
SNP4-169843224	PALLD	4	169843224	6	5.367729
rs12510359	LOC727835	4	169913967	2	0.953588
rs12510359	LOC727835	4	169913967	1	0.061132
rs7439293	LOC727835	4	169914061	2	4.064072
rs7439293	LOC727835	4	169914061	1	1.482968
rs7439293	LOC727835	4	169914061	5	2.945763
rs7439293	LOC727835	4	169914061	3	2.765743
rs7439293	LOC727835	4	169914061	6	4.641168
rs7439293	LOC727835	4	169914061	8	4.726297
rs17054604	CBR4	4	170146168	3	0.071605
SNP4-170635029	NEK1	4	170635029	1	0.71583
SNP4-170635029	NEK1	4	170635029	4	2.724374
SNP4-170713700	NEK1	4	170713700	2	6.318353
rs17055101	CLCN3	4	170870965	2	4.175297
rs1047642	C4orf27	4	170899810	4	0.634574
rs3733432	LOC441052	4	172970363	7	0.660121
rs2019683	GALNT17	4	174199000	5	0.342201
rs744724	NBLA00301	4	174696398	1	1.883323
rs10565	KIAA1712	4	175477703	8	0.103991
rs8752	HPGD	4	175649052	1	3.015201
rs8752	HPGD	4	175649052	4	3.360421
rs1050145	HPGD	4	175679731	8	0.484425
rs1050145	HPGD	4	175679731	2	2.665929
rs12648678	GLRA3	4	175834855	2	1.274689
rs12648678	GLRA3	4	175834855	3	0.94848
rs12651268	GLRA3	4	175924716	3	3.05959
rs12651268	GLRA3	4	175924716	7	0.880988
rs3733398	GPM6A	4	176792579	2	2.240333
rs11736872	WDR17	4	177337638	3	0.497526
rs11736872	WDR17	4	177337638	4	3.07127
rs11736872	WDR17	4	177337638	2	1.652462

rs11736872	WDR17	4	177337638	8	0.422813
rs11736872	WDR17	4	177337638	5	1.219745
rs11736872	WDR17	4	177337638	7	1.391937
rs11736872	WDR17	4	177337638	6	0.582231
rs7699964	WDR17	4	177338054	3	0.812491
rs12502935	SPATA4	4	177350773	4	0.347004
rs6832177	SPATA4	4	177350830	2	4.016171
rs6832177	SPATA4	4	177350830	4	0.778557
rs6832177	SPATA4	4	177350830	3	0.20901
rs6832177	SPATA4	4	177350830	7	1.037031
rs2291244	SPATA4	4	177350950	5	0.663315
rs13109244	ASB5	4	177373143	5	2.852894
rs6827525	ASB5	4	177374982	5	2.201981
rs6827525	ASB5	4	177374982	3	1.528696
rs34193982	NEIL3	4	178499778	5	0.086116
rs34193982	NEIL3	4	178499778	3	1.348709
rs2309626	MGC45800	4	183300005	8	0.267707
rs11737546	MGC45800	4	183300377	6	1.413924
rs11737546	MGC45800	4	183300377	5	0.461608
rs17263582	ODZ3	4	183911691	7	1.095269
rs17263582	ODZ3	4	183911691	6	1.508033
rs11223	ODZ3	4	183957844	3	0.363454
rs11223	ODZ3	4	183957844	6	0.110941
rs2309777	ODZ3	4	183959737	8	6.069977
rs2309777	ODZ3	4	183959737	6	0.791675
rs2309777	ODZ3	4	183959737	1	4.984788
rs2309777	ODZ3	4	183959737	3	0.708638
rs2309777	ODZ3	4	183959737	4	2.483955
rs7277	DCTD	4	184048507	8	1.754205
rs7277	DCTD	4	184048507	2	7.726239
rs2016910	FAM92A3	4	184196220	5	1.404679
rs2016910	FAM92A3	4	184196220	3	1.671508
rs2016910	FAM92A3	4	184196220	7	1.462402
SNP4-18442722	WWC2	4	184427227	3	0.151267
SNP4-18444088	WWC2	4	184440880	4	0.155306
SNP4-18444088	WWC2	4	184440880	5	0.083225
SNP4-18444088	WWC2	4	184440880	7	0.195049
SNP4-18444088	WWC2	4	184440880	6	0.755357
rs10015804	RWDD4A	4	184807709	4	0.244068
rs10015804	RWDD4A	4	184807709	3	1.078424
rs4479748	ENPP6	4	185249392	3	0.635707
rs4479748	ENPP6	4	185249392	1	2.312598
rs6819481	LOC391722	4	185475394	2	5.24973
rs793896	LOC728175	4	185487646	3	0.12679
rs4647603	CASP3	4	185806724	1	5.827221
rs6825561	MLF1IP	4	185852526	1	4.758134
rs14969	CCDC111	4	185852758	1	4.613687

rs6818447	MLF1IP	4	185887148	1	4.941488
rs11132270	LOC728203	4	186162916	1	3.251014
rs11132270	LOC728203	4	186162916	7	0.440718
rs11132270	LOC728203	4	186162916	6	0.285095
rs6839413	KIAA1430	4	186327812	6	0.308272
rs6839413	KIAA1430	4	186327812	7	1.350961
rs6839413	KIAA1430	4	186327812	1	0.05517
rs6814673	KIAA1430	4	186328185	1	0.429648
rs3797032	LRP2BP	4	186536650	4	6.863393
rs3797032	LRP2BP	4	186536650	7	0.165511
rs1048329	LRP2BP	4	186536752	4	0.538652
rs3733646	C4orf20	4	186558441	1	0.138661
rs3733646	C4orf20	4	186558441	4	0.437576
rs2289720	C4orf20	4	186584049	2	3.289835
rs2289720	C4orf20	4	186584049	1	1.600745
rs2289720	C4orf20	4	186584049	7	2.281184
rs2289720	C4orf20	4	186584049	5	1.986803
rs2120413	CCDC110	4	186603376	2	2.835703
SNP4-18661689	CCDC110	4	186616893	3	0.056652
SNP4-18661689	CCDC110	4	186616893	1	5.709617
SNP4-18661689	CCDC110	4	186616893	7	0.389448
SNP4-18661689	CCDC110	4	186616893	6	7.121497
SNP4-18661689	CCDC110	4	186616893	4	2.79292
SNP4-18661723	CCDC110	4	186617237	4	0.081069
SNP4-18661723	CCDC110	4	186617237	7	0.546033
SNP4-18661723	CCDC110	4	186617237	1	3.675924
SNP4-18661723	CCDC110	4	186617237	6	2.210701
SNP4-18661723	CCDC110	4	186617237	3	0.765683
rs11132306	CCDC110	4	186617509	8	0.287059
rs11132306	CCDC110	4	186617509	3	0.581078
rs11132306	CCDC110	4	186617509	7	0.977468
rs11132306	CCDC110	4	186617509	6	5.680298
rs11132306	CCDC110	4	186617509	4	0.092919
rs11132306	CCDC110	4	186617509	1	5.302566
rs7699687	CCDC110	4	186618109	4	4.414362
rs7699687	CCDC110	4	186618109	6	1.562582
rs7699687	CCDC110	4	186618109	7	0.593957
rs7699687	CCDC110	4	186618109	1	4.148882
rs7699687	CCDC110	4	186618109	3	0.749098
rs11132309	CCDC110	4	186619200	7	0.960412
rs11132309	CCDC110	4	186619200	6	1.649382
rs11132309	CCDC110	4	186619200	3	0.137605
rs11132309	CCDC110	4	186619200	8	0.077144
rs11132309	CCDC110	4	186619200	1	5.098178
rs11132309	CCDC110	4	186619200	4	0.084
rs10031753	SORBS2	4	186744403	6	0.643646
rs3775291	TLR3	4	187241068	7	5.336321

rs4862650	FAM149A	4	187311827	6	3.292557
rs4862650	FAM149A	4	187311827	1	5.044902
SNP4-187323556	FAM149A	4	187323556	3	3.114511
SNP4-187323556	FAM149A	4	187323556	1	6.020986
rs1877319	FLJ38576	4	187347776	3	0.212998
rs7696703	FLJ38576	4	187347850	1	4.309838
rs13146272	CYP4V2	4	187357205	3	0.0799
rs13146272	CYP4V2	4	187357205	1	3.134346
rs3736455	CYP4V2	4	187359313	3	1.64391
rs3736455	CYP4V2	4	187359313	1	4.596332
rs3736455	CYP4V2	4	187359313	6	0.553465
rs6842047	CYP4V2	4	187370570	1	3.547979
rs6842047	CYP4V2	4	187370570	6	1.920326
rs3733402	KLKB1	4	187395028	5	0.371223
rs3733402	KLKB1	4	187395028	3	0.705085
rs4253301	KLKB1	4	187410006	6	0.648165
rs4253301	KLKB1	4	187410006	1	2.975014
rs4253301	KLKB1	4	187410006	4	1.73423
SNP4-187821581	FAT	4	187821581	8	0.377613
rs1877731	FAT	4	187865392	3	0.045455
rs1877731	FAT	4	187865392	7	0.231027
rs367863	FAT	4	187866134	3	0.19682
rs6815391	ZFP42	4	189161973	3	1.008375
rs2279550	TRIML2	4	189249722	7	5.28744
rs2279550	TRIML2	4	189249722	1	3.78934
rs2279550	TRIML2	4	189249722	4	2.279786
rs11934889	TRIML1	4	189297604	5	0.016538
rs2077107	TUBB4Q	4	191140682	8	1.807279
rs2077107	TUBB4Q	4	191140682	7	3.575698
rs12519352	PLEKHG4B	5	234730	8	3.128543
rs12519352	PLEKHG4B	5	234730	3	0.112417
rs11133847	PLEKHG4B	5	234762	4	2.669375
rs11133847	PLEKHG4B	5	234762	5	1.659475
rs11133847	PLEKHG4B	5	234762	8	1.320258
rs11133847	PLEKHG4B	5	234762	2	2.669417
rs11133847	PLEKHG4B	5	234762	3	0.616148
rs13176160	PLEKHG4B	5	236401	8	0.829996
rs4956991	PLEKHG4B	5	236516	2	4.357552
rs4956991	PLEKHG4B	5	236516	4	1.893539
rs4956991	PLEKHG4B	5	236516	3	2.463899
rs4956991	PLEKHG4B	5	236516	8	2.416478
rs4956991	PLEKHG4B	5	236516	5	1.704094
rs4956925	PLEKHG4B	5	237336	3	0.69074
rs4956925	PLEKHG4B	5	237336	4	1.066997
rs4956925	PLEKHG4B	5	237336	8	0.658739
rs4956925	PLEKHG4B	5	237336	5	0.447416
rs1053477	PLEKHG4B	5	237393	3	0.20496

rs1053477	PLEKHG4B	5	237393	5	0.27579
rs1053477	PLEKHG4B	5	237393	4	0.151395
rs1053477	PLEKHG4B	5	237393	8	0.303536
rs4956993	PLEKHG4B	5	237702	5	0.383443
rs4956993	PLEKHG4B	5	237702	8	1.066735
rs4956993	PLEKHG4B	5	237702	3	0.545021
rs4956993	PLEKHG4B	5	237702	4	0.35794
rs1053478	PLEKHG4B	5	237750	8	2.253642
rs1053478	PLEKHG4B	5	237750	3	0.737279
rs1053478	PLEKHG4B	5	237750	5	0.689258
rs1053479	PLEKHG4B	5	237760	3	0.902106
rs1053479	PLEKHG4B	5	237760	5	0.154846
rs1053479	PLEKHG4B	5	237760	4	0.766883
rs6554947	PLEKHG4B	5	238160	3	0.109316
rs7727015	PLEKHG4B	5	238498	3	0.144142
rs7734578	PLEKHG4B	5	238519	2	2.744374
rs7734578	PLEKHG4B	5	238519	5	1.029565
rs7734578	PLEKHG4B	5	238519	4	0.353263
rs7734578	PLEKHG4B	5	238519	3	1.132964
rs1993519	PLEKHG4B	5	241321	5	0.575331
rs17559144	PLEKHG4B	5	241719	4	1.141507
rs17559144	PLEKHG4B	5	241719	5	0.847248
rs17559144	PLEKHG4B	5	241719	8	1.674821
rs17559144	PLEKHG4B	5	241719	7	1.176063
rs17559144	PLEKHG4B	5	241719	3	0.54383
rs6874933	PLEKHG4B	5	241747	5	0.949224
rs29680	PLEKHG4B	5	241896	1	1.672188
rs29680	PLEKHG4B	5	241896	3	0.954005
rs6883247	PLEKHG4B	5	241918	3	0.372039
rs13474	CCDC127	5	258077	2	2.624871
rs2672779	AHRR	5	488055	4	0.456617
rs2672779	AHRR	5	488055	3	0.574966
rs2672779	AHRR	5	488055	2	2.163856
rs2672779	AHRR	5	488055	5	0.113221
rs2672779	AHRR	5	488055	7	0.042087
rs2247114	SLC9A3	5	528104	7	0.006472
rs2247114	SLC9A3	5	528104	6	1.682444
rs2247114	SLC9A3	5	528104	4	0.051996
rs2247114	SLC9A3	5	528104	3	1.028761
rs868649	CEP72	5	693705	2	2.423844
rs868649	CEP72	5	693705	4	0.519456
rs383167	TPPP	5	715030	2	0.717509
rs383167	TPPP	5	715030	4	1.199273
rs383167	TPPP	5	715030	1	0.308082
rs1709542	TPPP	5	715543	4	1.462385
rs1709542	TPPP	5	715543	1	0.739937
rs1709542	TPPP	5	715543	2	0.785933

rs1709542	TPPP	5	715543	6	0.030418
rs1709542	TPPP	5	715543	8	6.062565
rs16900955	TPPP	5	717952	4	0.824042
rs1697963	TPPP	5	718280	5	4.296082
rs1697963	TPPP	5	718280	8	7.919229
rs1697963	TPPP	5	718280	2	0.226443
rs1697963	TPPP	5	718280	1	0.324406
rs3747733	ZDHHC11	5	904046	7	1.318461
GA022510	TRIP13	5	967664	3	1.061987
rs6865765	SLC12A7	5	1134702	2	1.796814
rs2853690	TERT	5	1306744	3	0.302428
rs2736098	TERT	5	1347086	3	0.499358
rs2736098	TERT	5	1347086	4	1.148497
rs2736098	TERT	5	1347086	7	0.395523
rs1809939	SLC6A3	5	1446063	5	1.967484
rs1809939	SLC6A3	5	1446063	1	1.285065
rs1809939	SLC6A3	5	1446063	3	1.443262
rs8352	LPCAT1	5	1516051	1	2.253466
rs8352	LPCAT1	5	1516051	7	0.422745
rs8352	LPCAT1	5	1516051	4	6.644011
rs8352	LPCAT1	5	1516051	3	0.322416
rs8352	LPCAT1	5	1516051	8	9.109422
rs8352	LPCAT1	5	1516051	5	0.66585
rs3733796	LPCAT1	5	1547909	3	0.772867
rs3733796	LPCAT1	5	1547909	1	5.580073
rs17588991	LPCAT1	5	1554695	6	0.841236
rs4975753	IRX4	5	1933865	5	0.951779
rs2962564	LOC728878	5	2793131	3	0.623195
rs2962564	LOC728878	5	2793131	1	1.029879
rs1019747	ADAMTS16	5	5199377	3	0.640474
rs1019747	ADAMTS16	5	5199377	7	0.172697
rs1019747	ADAMTS16	5	5199377	6	1.618762
rs1019747	ADAMTS16	5	5199377	4	1.645751
rs6555335	ADAMTS16	5	5253281	5	0.100899
rs2964475	KIAA0947	5	5460814	1	4.323133
rs2964475	KIAA0947	5	5460814	7	0.255451
rs3822430	SRD5A1	5	6704970	1	3.673873
rs3822430	SRD5A1	5	6704970	7	1.165086
rs2966952	FASTKD3	5	7921030	8	5.822061
rs2287779	MTRR	5	7942216	1	5.520913
rs16879334	MTRR	5	7944506	1	3.482372
rs16879334	MTRR	5	7944506	7	0.263501
rs10512963	LOC729506	5	8511746	4	1.250901
rs10512963	LOC729506	5	8511746	8	2.437731
rs9313239	LOC729506	5	8512533	4	2.08551
rs9313239	LOC729506	5	8512533	5	4.921749
rs9313239	LOC729506	5	8512533	7	2.164439

rs1448229	LOC729506	5	8512661	5	0.489702
rs1448229	LOC729506	5	8512661	4	0.050575
rs17235366	SEMA5A	5	9092288	8	0.345849
rs786843	SEMA5A	5	9093141	8	4.060463
rs786840	SEMA5A	5	9094935	8	0.209874
rs1806095	SEMA5A	5	9243404	1	0.015119
rs1806095	SEMA5A	5	9243404	2	0.300658
rs2438652	LOC134145	5	10292261	6	0.079527
rs2438652	LOC134145	5	10292261	7	1.523301
rs2578618	CCT5	5	10303443	5	4.460535
rs2578618	CCT5	5	10303443	2	0.076306
rs2578639	CCT5	5	10318719	2	5.735253
rs2578639	CCT5	5	10318719	5	1.149098
rs1287736	CMBL	5	10331403	2	3.60175
rs3995688	CMBL	5	10331657	7	1.061916
rs3995688	CMBL	5	10331657	2	1.807601
rs3995688	CMBL	5	10331657	6	1.28864
rs3995688	CMBL	5	10331657	5	0.409328
rs3995688	CMBL	5	10331657	1	1.226683
rs3995688	CMBL	5	10331657	4	0.281706
rs11745612	LOC651746	5	10691180	3	1.656847
rs1566622	CTNND2	5	11026727	8	5.011913
rs1566622	CTNND2	5	11026727	2	5.443339
rs1566622	CTNND2	5	11026727	4	0.832772
rs3734111	DNAH5	5	13754525	6	1.852018
rs3734111	DNAH5	5	13754525	8	5.394551
rs3734111	DNAH5	5	13754525	5	0.214543
SNP5-13770471	DNAH5	5	13770471	4	1.624105
rs30169	DNAH5	5	13772022	6	3.068478
rs30169	DNAH5	5	13772022	5	0.267333
rs30169	DNAH5	5	13772022	8	4.828212
rs30169	DNAH5	5	13772022	1	5.547718
rs30168	DNAH5	5	13772089	1	4.555531
rs30168	DNAH5	5	13772089	5	0.698182
rs30168	DNAH5	5	13772089	8	0.310401
rs30168	DNAH5	5	13772089	6	2.55338
rs17263496	DNAH5	5	13790444	1	4.684353
rs6554812	DNAH5	5	13815972	7	0.929322
rs6554812	DNAH5	5	13815972	3	1.282213
rs10513155	DNAH5	5	13841886	7	1.518482
rs10513155	DNAH5	5	13841886	8	0.533769
rs10078391	DNAH5	5	13864775	8	0.243802
rs10078391	DNAH5	5	13864775	1	1.606633
rs1348689	DNAH5	5	13882799	1	5.0132
rs1348689	DNAH5	5	13882799	7	0.644857
rs1348689	DNAH5	5	13882799	5	0.42241
rs1348689	DNAH5	5	13882799	4	0.707784

rs10041113	DNAH5	5	13898045	7	0.245889
rs10041113	DNAH5	5	13898045	1	4.76128
rs10041113	DNAH5	5	13898045	8	0.091728
rs10041113	DNAH5	5	13898045	5	0.408636
rs6554827	DNAH5	5	13917728	3	1.027219
rs6554827	DNAH5	5	13917728	4	0.434209
rs6554827	DNAH5	5	13917728	1	3.307138
rs7703349	DNAH5	5	13918980	3	0.389053
rs7703349	DNAH5	5	13918980	1	2.708779
rs10057007	DNAH5	5	13935841	1	3.197486
rs4701997	DNAH5	5	13947894	8	0.249023
rs4701997	DNAH5	5	13947894	4	0.449166
rs4701997	DNAH5	5	13947894	1	3.247439
rs1445823	DNAH5	5	13953345	6	1.045909
rs1445823	DNAH5	5	13953345	1	4.123827
rs1530498	DNAH5	5	13955220	1	3.040184
rs1530498	DNAH5	5	13955220	5	0.519188
rs3213936	DNAH5	5	13966885	7	0.072897
rs3213936	DNAH5	5	13966885	8	0.217378
rs3213936	DNAH5	5	13966885	6	2.078044
rs3213936	DNAH5	5	13966885	1	4.662587
rs1530496	DNAH5	5	13984340	6	0.782548
rs1530496	DNAH5	5	13984340	4	0.379313
rs1530496	DNAH5	5	13984340	8	1.217735
rs26107	TRIO	5	14551727	4	1.147236
rs25959	FAM105A	5	14664106	5	1.310948
rs31990	LOC642954	5	14851587	8	1.048988
rs31990	LOC642954	5	14851587	5	1.591952
rs31990	LOC642954	5	14851587	3	1.831591
rs31990	LOC642954	5	14851587	6	1.465662
rs31990	LOC642954	5	14851587	2	4.118603
rs31990	LOC642954	5	14851587	1	1.667936
rs3810847	FBXL7	5	15991553	8	0.339653
rs162848	FAM134B	5	16531200	3	0.018293
rs33684	FAM134B	5	16561898	1	4.721058
rs33684	FAM134B	5	16561898	5	0.33687
SNP5-16625153	FAM134B	5	16625153	5	5.142818
rs35003	FAM134B	5	16670099	7	0.079813
rs35003	FAM134B	5	16670099	1	1.062436
rs35003	FAM134B	5	16670099	5	0.437467
rs25901	MYO10	5	16726975	5	0.697995
rs25901	MYO10	5	16726975	3	0.66155
rs25901	MYO10	5	16726975	7	4.294926
rs4866303	LOC391767	5	17667821	5	1.592315
rs17285716	CDH18	5	19626931	4	0.915187
rs6451993	CDH12	5	21787813	4	0.05991
rs6451993	CDH12	5	21787813	8	0.103959

rs6451993	CDH12	5	21787813	5	0.850131
rs4371716	CDH12	5	22114341	5	3.011774
rs3805547	PRDM9	5	23563764	1	1.937733
rs2302903	CDH6	5	31353286	5	2.247023
rs2302903	CDH6	5	31353286	2	1.8559
rs2291109	C5orf22	5	31568186	4	4.555293
rs10066063	PDZD2	5	32126051	2	0.760293
rs10066063	PDZD2	5	32126051	7	5.412013
rs10066063	PDZD2	5	32126051	5	4.446702
rs10066063	PDZD2	5	32126051	1	0.364185
rs16889442	PDZD2	5	32128827	5	4.163903
rs16889442	PDZD2	5	32128827	7	1.520417
rs16889442	PDZD2	5	32128827	1	1.210442
rs16889442	PDZD2	5	32128827	2	1.184114
SNP5-32436023	ZFR	5	32436023	1	4.834753
SNP5-32436023	ZFR	5	32436023	6	7.717964
SNP5-32436023	ZFR	5	32436023	8	0.201307
rs10079994	LOC340113	5	32983455	6	1.38897
rs4867491	LOC340113	5	32984837	5	1.690612
rs4867491	LOC340113	5	32984837	2	3.207702
rs10941035	LOC340113	5	32997426	5	0.061391
rs17549362	LOC728553	5	33198370	3	4.346268
rs2304621	TARS	5	33476881	7	0.680175
rs2304621	TARS	5	33476881	6	3.783039
rs26722	SLC45A2	5	33999627	5	1.487016
rs34677	AMACR	5	34034525	7	0.708226
rs34677	AMACR	5	34034525	4	1.406746
rs3195676	AMACR	5	34043857	5	1.27417
rs3195676	AMACR	5	34043857	3	0.433516
rs3195676	AMACR	5	34043857	1	4.179301
rs840391	C1QTNF3	5	34054261	3	0.120389
rs840391	C1QTNF3	5	34054261	5	3.989973
rs840391	C1QTNF3	5	34054261	7	0.398529
rs840391	C1QTNF3	5	34054261	1	5.11652
rs840391	C1QTNF3	5	34054261	4	0.711985
rs840391	C1QTNF3	5	34054261	8	0.679921
rs840390	C1QTNF3	5	34054380	4	0.123029
rs840389	C1QTNF3	5	34054441	1	4.626715
rs840389	C1QTNF3	5	34054441	5	0.58245
rs840389	C1QTNF3	5	34054441	3	0.105166
rs840389	C1QTNF3	5	34054441	4	0.130272
rs840388	C1QTNF3	5	34054647	3	0.084732
rs840388	C1QTNF3	5	34054647	5	1.17115
rs840388	C1QTNF3	5	34054647	4	0.341215
rs840388	C1QTNF3	5	34054647	1	1.904126
rs840385	C1QTNF3	5	34056115	8	0.505791
rs840385	C1QTNF3	5	34056115	1	5.405568

rs840385	C1QTNF3	5	34056115	7	0.914507
rs840385	C1QTNF3	5	34056115	4	0.213003
rs840385	C1QTNF3	5	34056115	3	0.282476
rs840385	C1QTNF3	5	34056115	5	5.815715
rs6451173	FLJ25439	5	34876598	1	1.053384
SNP5-35108469	PRLR	5	35108469	1	4.593387
rs16902381	SPEF2	5	35694996	7	2.114839
rs6891905	SPEF2	5	35706498	1	0.174577
rs7710284	SPEF2	5	35728532	7	0.478586
rs6451206	SPEF2	5	35736355	1	5.472808
SNP5-35912008	IL7R	5	35912008	1	4.686555
rs3088073	C5orf33	5	36229764	2	1.298651
rs3088073	C5orf33	5	36229764	6	3.255718
rs3088073	C5orf33	5	36229764	7	0.049773
rs3088073	C5orf33	5	36229764	8	6.29696
rs2973033	GDNF	5	37875390	8	0.089829
rs3110234	LIFR	5	38521822	3	0.492474
rs3110234	LIFR	5	38521822	6	0.174072
rs834011	OSMR	5	38969643	1	3.843984
rs1975089	RICTOR	5	38977847	1	4.611496
rs6863066	FYB	5	39255372	6	3.885844
rs1050903	DAB2	5	39430250	4	0.329972
rs1050903	DAB2	5	39430250	5	1.087038
rs1697938	CARD6	5	40890439	7	0.661207
rs1697938	CARD6	5	40890439	5	0.697838
rs1697938	CARD6	5	40890439	1	0.213912
rs1697938	CARD6	5	40890439	3	0.021296
rs13157656	C7	5	41000609	6	1.183501
rs704040	FLJ40243	5	41033953	3	0.510997
rs13174484	FLJ40243	5	41105609	3	0.168144
rs9200	C6	5	41178363	1	1.385252
rs9200	C6	5	41178363	3	1.601672
rs9200	C6	5	41178363	4	3.078792
rs7701760	PLCXD3	5	41346492	5	1.006718
rs1373639	PLCXD3	5	41346714	5	1.227356
rs11737958	PLCXD3	5	41347921	5	1.210214
rs6179	GHR	5	42735801	5	0.516222
rs6179	GHR	5	42735801	7	0.70811
rs6179	GHR	5	42735801	8	0.687337
rs6180	GHR	5	42754996	7	2.44749
rs6180	GHR	5	42754996	5	0.491261
rs6180	GHR	5	42754996	2	1.216044
rs6180	GHR	5	42754996	4	0.290796
rs6180	GHR	5	42754996	1	0.693574
rs2910875	GHR	5	42755583	2	0.630448
rs2910875	GHR	5	42755583	1	0.362572
rs2910875	GHR	5	42755583	4	0.60125

rs316408	LOC644010	5	43102531	8	4.436675
rs17243515	MGC42105	5	43228178	1	3.325077
rs17243515	MGC42105	5	43228178	7	0.177307
rs17302931	MGC42105	5	43228210	7	1.553204
rs17302931	MGC42105	5	43228210	1	1.381207
rs13659	HMGCS1	5	43326457	6	0.136727
rs13659	HMGCS1	5	43326457	1	0.130391
rs13659	HMGCS1	5	43326457	2	1.43817
rs13659	HMGCS1	5	43326457	7	0.389224
rs10055801	C5orf34	5	43550753	2	0.851036
rs10055801	C5orf34	5	43550753	1	0.379656
rs10055801	C5orf34	5	43550753	3	0.159323
rs11954894	LOC642366	5	50709888	8	0.077785
rs11954894	LOC642366	5	50709888	7	2.222927
rs2270756	PELO	5	52119768	5	0.988378
rs3088364	ITGA1	5	52284785	4	0.859922
rs3088364	ITGA1	5	52284785	1	0.22539
rs3088364	ITGA1	5	52284785	8	4.656906
rs3088364	ITGA1	5	52284785	7	0.436676
rs3088364	ITGA1	5	52284785	6	2.252181
rs3088364	ITGA1	5	52284785	3	0.948669
rs3088363	ITGA1	5	52284812	4	4.420427
rs3088363	ITGA1	5	52284812	3	1.047623
rs3088363	ITGA1	5	52284812	2	1.081869
rs3088363	ITGA1	5	52284812	8	9.391233
rs3088363	ITGA1	5	52284812	6	3.106515
rs3088363	ITGA1	5	52284812	1	0.601038
rs1047481	ITGA1	5	52285233	2	1.99195
rs1047481	ITGA1	5	52285233	6	0.199688
rs1047481	ITGA1	5	52285233	3	0.448362
rs1047481	ITGA1	5	52285233	4	0.305504
rs1047481	ITGA1	5	52285233	8	3.365555
rs1047481	ITGA1	5	52285233	7	0.307278
rs1047481	ITGA1	5	52285233	1	0.325926
SNP5-52433762	MOCS2	5	52433762	7	0.124102
SNP5-52433762	MOCS2	5	52433762	1	2.156382
rs722910	FST	5	52817354	6	1.748935
rs722910	FST	5	52817354	8	0.041679
rs35941	ARL15	5	53642052	4	0.34097
rs3822313	ESM1	5	54310631	7	0.385598
rs3822313	ESM1	5	54310631	1	1.304439
rs3822313	ESM1	5	54310631	3	0.178769
rs444527	CDC20B	5	54445856	1	0.846763
rs1021580	CDC20B	5	54475223	3	0.57157
rs4865953	RNF138P1	5	54860814	2	3.37473
rs4865615	FLJ90709	5	54996430	2	4.059678
rs4865615	FLJ90709	5	54996430	1	0.60541

rs4865615	FLJ90709	5	54996430	4	0.372479
rs4865615	FLJ90709	5	54996430	6	0.522109
rs957459	DDX4	5	55118231	2	1.694821
rs1009639	IL31RA	5	55191159	8	0.758274
rs161704	IL31RA	5	55242201	1	0.864439
rs161704	IL31RA	5	55242201	6	0.170741
rs161704	IL31RA	5	55242201	2	0.155397
rs161704	IL31RA	5	55242201	7	1.365359
rs17653152	LOC345645	5	55607777	8	4.805573
rs17653152	LOC345645	5	55607777	5	3.344249
rs17653152	LOC345645	5	55607777	3	3.534244
rs17653152	LOC345645	5	55607777	6	1.302114
rs832583	MAP3K1	5	56213974	4	3.2787
rs832546	MAP3K1	5	56226957	4	0.92341
SNP5-56269247	MIER3	5	56269247	3	0.178698
rs13159014	DKFZp686D0972	5	56812010	1	3.502229
rs11950021	LOC728103	5	57448054	4	0.432789
rs11950021	LOC728103	5	57448054	7	0.824156
rs11950021	LOC728103	5	57448054	5	1.178388
rs32613	PLK2	5	57786024	2	5.788311
rs7704785	C5orf29	5	57826094	5	2.681907
rs7704785	C5orf29	5	57826094	7	1.580922
rs7704785	C5orf29	5	57826094	3	0.81825
rs1389308	C5orf29	5	57827068	7	0.74971
rs829259	PDE4D	5	58303733	8	5.652911
rs829259	PDE4D	5	58303733	7	0.008382
rs829259	PDE4D	5	58303733	5	0.056603
rs16878354	ELOVL7	5	60083785	1	5.752415
rs13358053	ELOVL7	5	60085334	3	0.460075
rs153377	FLJ37543	5	61037303	3	1.989747
rs32170	IPO11	5	61882830	1	0.085658
rs32170	IPO11	5	61882830	2	0.631505
rs275819	SFRS12IP1	5	64059737	2	0.457511
rs275819	SFRS12IP1	5	64059737	4	1.365627
rs3756739	SFRS12IP1	5	64100167	5	0.886002
rs16893435	ADAMTS6	5	64480678	6	0.057902
GA004492	CENPK	5	64894711	3	1.642533
GA004492	CENPK	5	64894711	2	0.780775
rs28126	NULL	5	64960826	3	0.156096
rs28126	NULL	5	64960826	5	0.24416
rs28126	NULL	5	64960826	4	0.797604
rs27138	SGTB	5	65000893	4	1.123462
rs27138	SGTB	5	65000893	5	0.854045
rs27394	SGTB	5	65001513	4	0.024871
rs27394	SGTB	5	65001513	3	0.858114
rs27394	SGTB	5	65001513	2	4.174683
rs27394	SGTB	5	65001513	5	0.234848

rs3213837	ERBB2IP	5	65357067	5	0.077581
rs6893041	FLJ46010	5	65649994	7	0.547377
rs11955611	LOC202227	5	65903674	4	0.825637
rs5744463	CD180	5	66528168	8	0.614559
rs164572	SLC30A5	5	68454810	1	0.118843
rs1128761	CCNB1	5	68507003	3	0.885729
rs2242350	MRPS36	5	68549355	5	0.20448
rs2242350	MRPS36	5	68549355	4	2.816833
rs2242350	MRPS36	5	68549355	1	3.24289
rs2242350	MRPS36	5	68549355	8	0.018667
rs2242350	MRPS36	5	68549355	7	0.095917
rs2242350	MRPS36	5	68549355	6	0.166453
rs2972388	CDK7	5	68567009	6	0.35026
rs2972388	CDK7	5	68567009	7	0.637881
rs2972388	CDK7	5	68567009	4	3.151468
rs2972388	CDK7	5	68567009	8	1.919974
rs2972388	CDK7	5	68567009	1	5.195529
rs2972388	CDK7	5	68567009	5	0.237572
rs12651858	CCDC125	5	68613223	6	0.322434
rs4421064	CCDC125	5	68614184	4	0.108454
rs4421064	CCDC125	5	68614184	5	0.03745
rs4421064	CCDC125	5	68614184	1	3.685278
rs4421064	CCDC125	5	68614184	7	0.308643
rs28409706	NAIP	5	70344007	3	0.931335
rs3761966	BDP1	5	70836231	5	0.080104
rs3761966	BDP1	5	70836231	3	0.330476
rs3761966	BDP1	5	70836231	1	3.168688
SNP5-70842213	BDP1	5	70842213	6	1.176524
SNP5-70842213	BDP1	5	70842213	5	0.391139
SNP5-70842213	BDP1	5	70842213	2	3.490179
SNP5-70842213	BDP1	5	70842213	8	0.592292
SNP5-70842213	BDP1	5	70842213	7	5.659716
SNP5-70842213	BDP1	5	70842213	1	0.35657
rs10064079	MCCC2	5	70980831	3	1.095198
rs10064079	MCCC2	5	70980831	7	1.52793
rs10064079	MCCC2	5	70980831	6	0.403577
rs343109	ANKRA2	5	72897122	4	4.348676
rs2112159	ANKRA2	5	72897211	4	2.678222
rs343118	UTP15	5	72912532	4	0.183759
rs2973566	RGNEF	5	73184237	6	2.732937
rs2973566	RGNEF	5	73184237	3	0.456542
rs2973566	RGNEF	5	73184237	2	0.494541
rs2931423	RGNEF	5	73199587	3	0.21732
rs2931423	RGNEF	5	73199587	6	0.159462
rs11556477	GFM2	5	74098637	6	0.565951
rs6874609	TINP1	5	74105619	4	0.32135
SNP5-74145596	KIAA0888	5	74145596	4	5.865243

rs3811986	GCNT4	5	74360193	2	2.62448
rs3811986	GCNT4	5	74360193	4	0.367659
rs3811986	GCNT4	5	74360193	5	0.45414
rs3811986	GCNT4	5	74360193	6	0.386786
rs3811986	GCNT4	5	74360193	3	2.045526
rs3811987	GCNT4	5	74360304	3	0.620154
rs3811987	GCNT4	5	74360304	2	2.06681
rs3811987	GCNT4	5	74360304	6	3.411496
rs3811987	GCNT4	5	74360304	5	2.737229
rs3811987	GCNT4	5	74360304	4	3.509395
rs3811987	GCNT4	5	74360304	8	0.261916
rs10942729	ANKRD31	5	74400056	3	1.353203
rs10942729	ANKRD31	5	74400056	4	1.112281
rs961098	ANKRD31	5	74436142	3	0.417915
rs961098	ANKRD31	5	74436142	2	6.247796
rs1422699	ANKRD31	5	74478166	4	0.950067
rs1422699	ANKRD31	5	74478166	8	1.544014
rs1422699	ANKRD31	5	74478166	6	0.298311
rs1422699	ANKRD31	5	74478166	3	0.017224
rs6888707	ANKRD31	5	74478676	6	0.710819
rs6888707	ANKRD31	5	74478676	4	0.599171
rs6888707	ANKRD31	5	74478676	3	0.78818
rs6893216	ANKRD31	5	74478720	4	0.6184
rs1422698	ANKRD31	5	74478888	8	1.317511
rs1422698	ANKRD31	5	74478888	4	2.167477
rs1422698	ANKRD31	5	74478888	3	0.948731
rs1422698	ANKRD31	5	74478888	6	2.885092
rs9332464	LOC728780	5	74957442	4	1.732186
rs34358	LOC728780	5	75000878	4	4.82149
rs34358	LOC728780	5	75000878	2	3.091553
rs34358	LOC728780	5	75000878	8	4.971139
rs10070440	SV2C	5	75463691	5	0.404561
rs2270927	SV2C	5	75627466	3	0.244233
rs2270927	SV2C	5	75627466	2	1.723995
rs2270927	SV2C	5	75627466	6	4.744971
rs1047530	F2RL2	5	75947155	1	0.734727
rs1047530	F2RL2	5	75947155	5	0.20852
rs1047530	F2RL2	5	75947155	3	0.283736
rs1047530	F2RL2	5	75947155	2	0.087244
rs1047530	F2RL2	5	75947155	4	0.324647
rs1047529	F2RL2	5	75947220	3	0.440324
rs1047529	F2RL2	5	75947220	8	0.021378
rs1047529	F2RL2	5	75947220	4	0.299719
rs1047529	F2RL2	5	75947220	5	0.19689
rs1047529	F2RL2	5	75947220	1	0.051196
rs2069693	F2RL2	5	75947471	3	0.90023
rs2069693	F2RL2	5	75947471	1	0.309848

rs2069693	F2RL2	5	75947471	5	3.586912
rs2069693	F2RL2	5	75947471	4	0.159828
rs2069693	F2RL2	5	75947471	8	0.177489
rs1047494	F2RL2	5	75947743	1	0.100424
rs1047494	F2RL2	5	75947743	4	0.017537
rs1047494	F2RL2	5	75947743	5	0.720763
rs2069689	F2RL2	5	75948157	3	1.10506
rs2069689	F2RL2	5	75948157	1	0.598132
rs2069689	F2RL2	5	75948157	4	0.352626
rs2069689	F2RL2	5	75948157	2	0.003657
rs2069689	F2RL2	5	75948157	5	0.160574
rs961536	F2RL2	5	75948599	3	2.417281
rs2069700	F2RL2	5	75949759	4	1.371362
rs2910819	IQGAP2	5	75968625	4	4.867493
rs2910819	IQGAP2	5	75968625	2	4.962831
rs2455230	IQGAP2	5	75968721	7	0.793396
rs2455230	IQGAP2	5	75968721	2	4.74305
rs2455230	IQGAP2	5	75968721	4	0.065112
rs2431363	IQGAP2	5	75984406	6	1.572162
rs2431363	IQGAP2	5	75984406	2	3.868516
rs2431363	IQGAP2	5	75984406	7	0.13094
rs2431363	IQGAP2	5	75984406	4	3.155545
rs34592828	IQGAP2	5	76032665	2	5.633108
rs2242991	F2RL1	5	76150615	5	0.052118
rs1529505	F2RL1	5	76150719	5	0.659526
rs1529505	F2RL1	5	76150719	1	2.029018
rs1529505	F2RL1	5	76150719	3	0.254661
rs1320308	S100Z	5	76207008	7	0.572129
SNP5-76394780	AGGF1	5	76394780	1	5.180194
SNP5-76394780	AGGF1	5	76394780	7	0.094683
SNP5-76394780	AGGF1	5	76394780	4	2.051524
rs33204	WDR41	5	76769840	6	3.526415
rs33204	WDR41	5	76769840	4	3.570234
rs33204	WDR41	5	76769840	2	5.62613
rs6453373	AP3B1	5	77460784	4	0.178287
rs4530741	LOC728769	5	77691868	1	1.186806
rs4072852	LOC728769	5	77692056	1	0.459213
rs4072852	LOC728769	5	77692056	2	0.398546
rs6874309	SCAMP1	5	77810254	7	0.468374
rs6874309	SCAMP1	5	77810254	4	0.480059
rs6874309	SCAMP1	5	77810254	5	0.103466
rs6874309	SCAMP1	5	77810254	8	0.444126
rs7704939	ARSB	5	78109954	3	0.187043
rs7704939	ARSB	5	78109954	1	0.765777
rs7704939	ARSB	5	78109954	2	0.311291
rs754566	ARSB	5	78110674	1	5.072658
rs754566	ARSB	5	78110674	4	0.218895

rs2173012	ARSB	5	78111916	4	0.543268
rs25413	ARSB	5	78170957	1	3.602252
rs25413	ARSB	5	78170957	7	0.664319
rs25413	ARSB	5	78170957	8	0.628895
rs25414	ARSB	5	78170997	2	2.484892
rs17220759	ARSB	5	78217179	4	0.860628
rs163126	ARSB	5	78318005	4	0.307799
rs163126	ARSB	5	78318005	1	1.089521
rs163126	ARSB	5	78318005	7	0.053421
rs163126	ARSB	5	78318005	2	0.492824
rs163126	ARSB	5	78318005	5	0.094083
rs1805072	DMGDH	5	78364316	7	0.601671
rs1805072	DMGDH	5	78364316	1	1.411672
rs248385	DMGDH	5	78376013	6	0.173514
rs248385	DMGDH	5	78376013	3	0.333174
rs248385	DMGDH	5	78376013	2	2.375993
rs682985	BHMT2	5	78409187	3	0.07028
rs4703772	BHMT	5	78457791	3	0.458118
rs585800	BHMT	5	78462964	8	3.597753
rs13182512	JMY	5	78609546	2	3.865509
rs13182512	JMY	5	78609546	1	0.108687
rs13182512	JMY	5	78609546	6	0.324527
SNP5-79060535	CMYA5	5	79060535	4	6.39371
SNP5-79060535	CMYA5	5	79060535	5	0.956577
SNP5-79060535	CMYA5	5	79060535	1	3.747558
SNP5-79060535	CMYA5	5	79060535	6	3.843768
SNP5-79062116	CMYA5	5	79062116	4	9.125083
SNP5-79062116	CMYA5	5	79062116	1	5.905929
SNP5-79062116	CMYA5	5	79062116	5	9.17153
SNP5-79062116	CMYA5	5	79062116	6	1.252785
rs16877141	CMYA5	5	79065482	5	0.165078
rs16877141	CMYA5	5	79065482	1	5.150893
rs16877141	CMYA5	5	79065482	6	10.69113
rs6859595	CMYA5	5	79067128	5	0.142957
rs6859595	CMYA5	5	79067128	1	7.445355
rs6859595	CMYA5	5	79067128	4	0.008748
rs6859595	CMYA5	5	79067128	6	7.174294
SNP5-79067314	CMYA5	5	79067314	3	0.064232
rs10060444	MTX3	5	79310351	7	2.32488
rs10060444	MTX3	5	79310351	8	2.645425
rs10060444	MTX3	5	79310351	5	0.096337
rs6870377	MTX3	5	79311905	8	5.138241
rs6870377	MTX3	5	79311905	5	0.155641
rs6870377	MTX3	5	79311905	6	0.666786
rs6870377	MTX3	5	79311905	7	1.128463
rs10064975	MTX3	5	79312971	6	0.25441
rs10064975	MTX3	5	79312971	7	0.895938

rs17196706	MTX3	5	79313091	5	0.162134
rs11738136	LOC644936	5	79634345	1	1.002956
rs1052345	SPZ1	5	79653384	3	0.490396
rs369998	FAM151B	5	79851414	3	0.438507
rs369998	FAM151B	5	79851414	2	7.277704
rs32857	ANKRD34B	5	79891128	1	4.21991
rs1805355	MSH3	5	80001785	5	0.74839
rs1805355	MSH3	5	80001785	3	0.155476
rs184967	MSH3	5	80185737	5	6.060207
rs26279	MSH3	5	80204693	5	0.798489
rs26279	MSH3	5	80204693	1	0.121421
rs26279	MSH3	5	80204693	2	0.523294
SNP5-80445282	RASGRF2	5	80445282	5	0.161226
rs10942942	RASGRF2	5	80445485	5	0.719195
rs10942942	RASGRF2	5	80445485	4	0.843492
rs2897389	RASGRF2	5	80557673	6	1.384788
rs2897389	RASGRF2	5	80557673	3	1.443479
rs2897389	RASGRF2	5	80557673	5	1.875288
rs545	CKMT2	5	80597876	6	0.541678
rs545	CKMT2	5	80597876	5	0.379664
rs545	CKMT2	5	80597876	3	0.398425
rs10052929	ZCCHC9	5	80633761	1	2.078405
rs10052929	ZCCHC9	5	80633761	7	0.599379
rs10052929	ZCCHC9	5	80633761	4	0.761911
rs10052929	ZCCHC9	5	80633761	6	0.059693
rs10052929	ZCCHC9	5	80633761	3	0.824551
rs16878594	ZCCHC9	5	80636375	4	0.228571
rs16878594	ZCCHC9	5	80636375	1	5.102373
rs16878594	ZCCHC9	5	80636375	7	0.163313
rs1864183	ATG10	5	81584972	4	2.356726
rs1864183	ATG10	5	81584972	7	0.244522
rs1864183	ATG10	5	81584972	8	2.249103
rs2270901	FLJ41309	5	81717787	4	0.317275
rs2270901	FLJ41309	5	81717787	6	0.836155
rs2270901	FLJ41309	5	81717787	1	5.478009
rs2287926	VCAN	5	82851164	7	6.386612
rs2287926	VCAN	5	82851164	5	0.845974
rs309557	VCAN	5	82870386	5	0.519021
rs309557	VCAN	5	82870386	4	5.734586
rs309557	VCAN	5	82870386	1	0.830179
rs309557	VCAN	5	82870386	2	0.230456
rs309557	VCAN	5	82870386	3	0.711322
rs160279	VCAN	5	82871301	4	0.258649
rs160279	VCAN	5	82871301	1	0.08658
rs160279	VCAN	5	82871301	3	0.340501
rs160279	VCAN	5	82871301	7	0.481097
rs160278	VCAN	5	82871480	2	4.19892

rs160278	VCAN	5	82871480	1	1.217025
rs160278	VCAN	5	82871480	5	0.656166
rs160278	VCAN	5	82871480	3	1.230295
rs160278	VCAN	5	82871480	4	0.086266
rs160278	VCAN	5	82871480	7	0.763239
rs2269287	EDIL3	5	83396276	2	2.704458
rs2269287	EDIL3	5	83396276	7	0.084097
rs684958	LOC645181	5	84881644	7	5.456542
rs684958	LOC645181	5	84881644	3	5.044149
rs7720113	LOC729011	5	89269589	2	3.108153
rs4873	CETN3	5	89739397	1	0.272856
rs4873	CETN3	5	89739397	3	0.170821
rs4873	CETN3	5	89739397	7	0.322383
rs4873	CETN3	5	89739397	4	1.009797
rs2303801	POLR3G	5	89806524	5	4.227047
rs2303801	POLR3G	5	89806524	4	0.216591
rs2303801	POLR3G	5	89806524	8	3.258197
rs2303801	POLR3G	5	89806524	2	5.008393
rs2303801	POLR3G	5	89806524	3	0.632981
rs950692	GPR98	5	89979189	5	0.48886
rs950692	GPR98	5	89979189	1	4.77665
rs950692	GPR98	5	89979189	7	1.108975
rs950692	GPR98	5	89979189	4	0.627006
rs2366777	GPR98	5	89979327	7	1.552762
rs2366777	GPR98	5	89979327	5	0.263883
rs2366777	GPR98	5	89979327	1	1.460719
rs2366777	GPR98	5	89979327	4	1.163101
rs2366777	GPR98	5	89979327	8	1.937245
rs2366777	GPR98	5	89979327	6	1.349145
rs2366777	GPR98	5	89979327	3	1.530158
rs2366777	GPR98	5	89979327	2	1.110457
rs17543819	GPR98	5	89989605	6	0.078914
rs17543819	GPR98	5	89989605	2	4.404147
rs17544552	GPR98	5	90015274	6	0.839629
rs17544552	GPR98	5	90015274	2	3.239762
rs4916684	GPR98	5	90015345	3	0.345424
rs4916684	GPR98	5	90015345	2	2.602005
rs4916684	GPR98	5	90015345	4	0.150418
rs4916684	GPR98	5	90015345	1	0.935942
rs4916684	GPR98	5	90015345	5	0.212101
SNP5-90015447	GPR98	5	90015447	4	0.066439
SNP5-90015447	GPR98	5	90015447	5	4.30997
SNP5-90015447	GPR98	5	90015447	8	2.048055
SNP5-90015447	GPR98	5	90015447	1	4.008252
SNP5-90015506	GPR98	5	90015506	5	1.170894
SNP5-90015506	GPR98	5	90015506	4	0.610924
SNP5-90015506	GPR98	5	90015506	1	3.78073

rs2366926	GPR98	5	90024260	3	0.014008
rs16876822	GPR98	5	90025535	3	1.213005
rs1878878	GPR98	5	90026080	1	0.265079
rs1878878	GPR98	5	90026080	6	0.073819
rs1878878	GPR98	5	90026080	3	1.072998
rs1878878	GPR98	5	90026080	2	1.37917
rs1878878	GPR98	5	90026080	4	0.99575
rs1878878	GPR98	5	90026080	5	0.701091
rs16869032	GPR98	5	90052627	8	0.26912
rs16869032	GPR98	5	90052627	1	5.278711
rs16869032	GPR98	5	90052627	5	0.893107
rs16869032	GPR98	5	90052627	4	2.642548
rs17624033	GPR98	5	90077266	3	0.089299
rs17624033	GPR98	5	90077266	7	0.161425
SNP5-90088045	GPR98	5	90088045	3	1.754608
SNP5-90088045	GPR98	5	90088045	7	1.243247
rs2247870	GPR98	5	90187345	4	1.250299
rs2247870	GPR98	5	90187345	8	3.40254
rs2247870	GPR98	5	90187345	7	1.970077
rs13355819	LOC729040	5	90612035	3	0.711531
rs13355819	LOC729040	5	90612035	4	0.743618
rs13355819	LOC729040	5	90612035	8	3.649465
rs867831	LOC729040	5	90612247	1	4.180094
rs17638113	ARRDC3	5	90700781	8	4.066093
rs2636	MCTP1	5	94076243	1	0.356188
rs2636	MCTP1	5	94076243	2	3.615708
rs2636	MCTP1	5	94076243	6	6.124037
rs7726891	FAM81B	5	94775479	4	1.046113
rs7726891	FAM81B	5	94775479	1	0.968763
SNP5-94775513	FAM81B	5	94775513	4	1.269854
SNP5-94781766	FAM81B	5	94781766	1	1.881499
rs10054161	RFESD	5	95008919	5	0.565497
rs10054161	RFESD	5	95008919	3	0.897003
rs4561	GLRX	5	95178069	5	0.01704
rs4561	GLRX	5	95178069	2	3.488397
rs4561	GLRX	5	95178069	6	3.722428
rs4561	GLRX	5	95178069	8	1.736494
rs4561	GLRX	5	95178069	3	0.858969
rs3797207	LOC727938	5	95195665	5	0.037607
rs3797207	LOC727938	5	95195665	6	1.836783
rs7734301	LOC727938	5	95195755	2	2.038986
rs7734301	LOC727938	5	95195755	5	0.285732
rs17085193	C5orf27	5	95220349	2	0.487064
rs17085249	ELL2	5	95262171	8	0.654619
rs17085249	ELL2	5	95262171	7	2.947723
rs17085249	ELL2	5	95262171	4	0.850659
rs17085249	ELL2	5	95262171	3	0.851573

rs3811942	PCSK1	5	95754196	7	0.139375
rs3811942	PCSK1	5	95754196	1	4.550298
rs6234	PCSK1	5	95754730	6	0.923203
rs6233	PCSK1	5	95758868	7	1.068073
rs6233	PCSK1	5	95758868	6	0.248134
rs6233	PCSK1	5	95758868	8	0.062475
rs6233	PCSK1	5	95758868	4	0.209607
rs6230	PCSK1	5	95794603	1	1.739235
rs6230	PCSK1	5	95794603	8	0.710315
rs7724759	CAST	5	96102243	6	7.467356
rs7724759	CAST	5	96102243	3	2.435491
rs7724759	CAST	5	96102243	8	3.891422
rs7724759	CAST	5	96102243	2	7.419016
rs754615	CAST	5	96112090	5	0.064489
rs754615	CAST	5	96112090	8	0.866909
rs754615	CAST	5	96112090	2	3.920331
rs754615	CAST	5	96112090	6	7.053483
rs754615	CAST	5	96112090	3	0.2847
rs27433	CAST	5	96135691	3	0.155048
rs27433	CAST	5	96135691	4	5.814827
rs7063	CAST	5	96135967	3	0.011582
rs7063	CAST	5	96135967	8	1.065876
rs7063	CAST	5	96135967	2	1.251882
rs7063	CAST	5	96135967	5	0.234647
rs26653	ERAP1	5	96165006	3	0.07798
rs26653	ERAP1	5	96165006	7	0.962274
rs26653	ERAP1	5	96165006	4	4.734516
SNP5-96165351	ERAP1	5	96165351	1	0.38758
SNP5-96165351	ERAP1	5	96165351	5	0.565012
SNP5-96165351	ERAP1	5	96165351	2	0.384349
rs2549796	ERAP2	5	96271099	2	4.307969
rs2549796	ERAP2	5	96271099	6	3.906942
rs2549796	ERAP2	5	96271099	4	2.542874
rs1056893	ERAP2	5	96271195	4	2.344114
rs2255546	ERAP2	5	96274871	6	7.315008
rs2255546	ERAP2	5	96274871	7	10.18269
rs2255546	ERAP2	5	96274871	2	0.461359
rs2255546	ERAP2	5	96274871	4	3.438195
rs11746232	LNPEP	5	96389215	7	0.526075
rs11746232	LNPEP	5	96389215	1	5.127475
rs11746232	LNPEP	5	96389215	3	0.757239
rs316233	LIX1	5	96453380	3	0.067724
rs2544773	RIOK2	5	96539227	3	0.054163
rs2544773	RIOK2	5	96539227	7	0.607332
rs2544773	RIOK2	5	96539227	8	0.482831
rs2544773	RIOK2	5	96539227	1	6.976895
rs979348	LOC391813	5	97099891	4	2.240625

rs979348	LOC391813	5	97099891	6	2.969624
rs13541	RGMB	5	98159405	4	7.296256
rs13541	RGMB	5	98159405	3	0.078854
rs13541	RGMB	5	98159405	7	0.552516
rs13541	RGMB	5	98159405	8	3.040111
rs1428439	ST8SIA4	5	100266700	7	0.555138
rs1428439	ST8SIA4	5	100266700	2	0.918771
rs1428439	ST8SIA4	5	100266700	5	0.896777
rs1428439	ST8SIA4	5	100266700	1	0.481795
rs4703217	SLCO4C1	5	101598390	5	0.097288
rs7719298	SLCO4C1	5	101599589	5	1.147108
SNP5-10160299(	SLCO4C1	5	101602990	4	0.99166
SNP5-10160299(	SLCO4C1	5	101602990	7	1.259882
SNP5-10160299(	SLCO4C1	5	101602990	5	0.174404
rs10479190	SLCO4C1	5	101659689	2	1.590526
rs10479190	SLCO4C1	5	101659689	7	1.015144
rs10479190	SLCO4C1	5	101659689	5	0.900084
rs249496	PAM	5	102229489	5	0.5438
rs249496	PAM	5	102229489	4	0.296683
rs249496	PAM	5	102229489	7	1.979143
rs249496	PAM	5	102229489	1	0.631884
rs249496	PAM	5	102229489	8	0.085875
rs249496	PAM	5	102229489	3	0.690441
rs249496	PAM	5	102229489	2	1.796325
rs249496	PAM	5	102229489	6	0.277355
SNP5-10292257	NUDT12	5	102922572	4	8.733647
SNP5-10292257	NUDT12	5	102922572	8	0.078628
rs34499946	FER	5	108309808	7	0.922456
rs2289277	TSLP	5	110436966	5	2.643215
rs2289277	TSLP	5	110436966	3	0.60997
rs2289277	TSLP	5	110436966	8	4.850765
rs2289277	TSLP	5	110436966	6	8.031565
rs2289278	TSLP	5	110437047	6	5.653516
rs2289278	TSLP	5	110437047	4	1.275342
rs2289278	TSLP	5	110437047	8	0.578928
rs11466749	TSLP	5	110440484	4	1.048063
rs11466749	TSLP	5	110440484	6	0.157306
rs11466750	TSLP	5	110440793	4	2.132936
rs2229995	APC	5	112206694	3	0.325829
rs33556	DCP2	5	112340427	6	6.977204
rs33556	DCP2	5	112340427	4	0.590777
rs33556	DCP2	5	112340427	3	0.800174
rs33556	DCP2	5	112340427	2	0.199516
rs17372511	DCP2	5	112382797	6	0.792175
rs17372511	DCP2	5	112382797	4	0.270125
rs17372511	DCP2	5	112382797	2	5.495549
rs7670	DCP2	5	112384548	4	3.250845

rs7670	DCP2	5	112384548	6	1.430428
rs17135317	MCC	5	112434761	2	4.951457
rs17135317	MCC	5	112434761	4	4.339831
rs17135317	MCC	5	112434761	6	3.140855
rs26980	TSSK1B	5	112797926	8	0.052861
rs26980	TSSK1B	5	112797926	1	5.613511
rs2974527	TRIM36	5	114488479	6	0.614588
rs2278696	TRIM36	5	114544129	1	0.11851
rs17138632	FLJ90650	5	115366857	3	0.172051
rs10078759	FLJ90650	5	115369537	4	0.470091
rs10078759	FLJ90650	5	115369537	6	0.383992
rs1508886	FLJ90650	5	115390047	3	1.26847
rs1508886	FLJ90650	5	115390047	6	0.799204
rs1508886	FLJ90650	5	115390047	1	1.07829
rs2048220	COMMD10	5	115497672	2	0.780137
rs2048220	COMMD10	5	115497672	6	1.039059
rs17132673	DTWD2	5	118203539	1	0.041881
rs17132673	DTWD2	5	118203539	5	0.007863
rs17132673	DTWD2	5	118203539	4	0.329711
rs17132673	DTWD2	5	118203539	2	1.13709
rs17132677	DTWD2	5	118203573	6	1.870266
SNP5-11854177	DMXL1	5	118541775	5	1.237722
SNP5-11854177	DMXL1	5	118541775	3	8.095887
rs3797345	TNFAIP8	5	118757773	5	0.765077
rs25640	HSD17B4	5	118839432	5	0.190217
rs25640	HSD17B4	5	118839432	6	0.359544
rs25640	HSD17B4	5	118839432	1	3.486793
rs25640	HSD17B4	5	118839432	3	0.183346
rs2434600	LOC340069	5	118998669	1	0.52962
rs2434600	LOC340069	5	118998669	7	0.931721
SNP5-12004971	PRR16	5	120049716	3	0.219043
SNP5-12004971	PRR16	5	120049716	2	1.683803
SNP5-12004971	PRR16	5	120049716	1	0.511218
rs10069230	PRR16	5	120050658	6	1.203831
rs10069230	PRR16	5	120050658	5	1.225932
SNP5-12151586	ZNF474	5	121515868	5	0.750491
SNP5-12151640	ZNF474	5	121516405	7	0.107934
SNP5-12151640	ZNF474	5	121516405	5	0.416194
SNP5-12151640	ZNF474	5	121516405	8	0.088536
SNP5-12151640	ZNF474	5	121516405	6	0.21716
rs10070158	SNCAIP	5	121767440	6	1.970263
rs10036124	PRDM6	5	122551474	6	0.555963
rs1047437	CCDC100	5	122713626	2	4.556426
rs1047437	CCDC100	5	122713626	7	0.146238
rs1047437	CCDC100	5	122713626	1	0.210382
rs7703447	ZNF608	5	124004866	8	2.805503
rs7703447	ZNF608	5	124004866	6	0.331442

rs7703447	ZNF608	5	124004866	7	1.092092
rs7703447	ZNF608	5	124004866	4	0.922722
rs3812052	MEGF10	5	126820873	7	0.827522
rs3812052	MEGF10	5	126820873	5	0.273986
rs3812052	MEGF10	5	126820873	1	0.215068
rs3812052	MEGF10	5	126820873	2	3.69667
rs3756722	MEGF10	5	126823145	2	2.078864
rs3756722	MEGF10	5	126823145	4	0.281001
rs3756722	MEGF10	5	126823145	5	0.487582
rs3756722	MEGF10	5	126823145	1	0.629977
rs2898043	MEGF10	5	126824484	1	0.529052
rs182844	LOC389322	5	126875628	5	1.700306
rs182844	LOC389322	5	126875628	4	3.380829
rs182844	LOC389322	5	126875628	1	3.303394
rs182844	LOC389322	5	126875628	6	2.241589
rs182844	LOC389322	5	126875628	2	1.995504
rs182844	LOC389322	5	126875628	7	2.564314
rs1050687	FBN2	5	127621631	5	1.193688
rs7288	FBN2	5	127621769	5	0.042493
rs2577539	SLC27A6	5	128329110	2	1.12314
rs2577539	SLC27A6	5	128329110	1	0.759561
rs2577539	SLC27A6	5	128329110	7	1.362611
rs2577539	SLC27A6	5	128329110	5	0.547501
rs2577539	SLC27A6	5	128329110	8	3.388795
rs2577540	SLC27A6	5	128329641	7	0.435577
rs2577540	SLC27A6	5	128329641	2	5.053496
rs2577540	SLC27A6	5	128329641	3	0.382596
rs1047999	SLC27A6	5	128397054	8	1.881888
rs1047999	SLC27A6	5	128397054	3	0.751541
rs1047999	SLC27A6	5	128397054	5	0.009169
rs1127827	ISOC1	5	128458656	1	1.153727
rs1127827	ISOC1	5	128458656	5	0.467555
rs1127827	ISOC1	5	128458656	3	1.257283
rs11749126	ADAMTS19	5	129067955	7	0.241925
rs2015018	CHSY-2	5	129549025	2	0.637823
rs2015018	CHSY-2	5	129549025	1	1.836956
rs2015018	CHSY-2	5	129549025	7	1.492927
rs17692694	LOC402229	5	130358366	1	3.998162
rs4706009	LYRM7	5	130563943	3	0.556891
rs4706009	LYRM7	5	130563943	5	0.345432
rs3488	CDC42SE2	5	130757514	3	0.717709
rs3775998	FNIP1	5	131007231	2	0.467577
rs3775998	FNIP1	5	131007231	1	0.178492
rs26008	FNIP1	5	131036093	2	0.343707
rs25882	CSF2	5	131439359	3	0.396488
rs1007602	PDLIM4	5	131630065	5	0.413746
rs1007602	PDLIM4	5	131630065	6	1.391773

rs9895	PDLIM4	5	131635841	8	7.067692
rs9895	PDLIM4	5	131635841	6	0.63722
rs272867	FLJ44796	5	131708956	8	0.739061
rs272867	FLJ44796	5	131708956	2	3.762169
rs274558	SLC22A5	5	131749073	2	3.436756
rs2522050	LOC441108	5	131824508	7	0.667709
rs2706395	LOC441108	5	131824702	7	0.027492
rs960757	IRF1	5	131854209	5	3.956641
rs960757	IRF1	5	131854209	8	0.71274
rs11242115	IRF1	5	131854312	7	4.917767
rs11242115	IRF1	5	131854312	8	0.43288
rs11242115	IRF1	5	131854312	2	3.209651
rs11242115	IRF1	5	131854312	5	4.954636
rs20541	IL13	5	132023863	1	1.866334
rs1295685	IL13	5	132024344	1	0.235569
rs848	IL13	5	132024399	1	1.158532
rs11242126	KIF3A	5	132058313	5	0.253967
rs11242126	KIF3A	5	132058313	2	0.247569
rs11242126	KIF3A	5	132058313	1	0.606002
rs757537	ANKRD43	5	132178970	4	1.690764
rs708459	LEAP2	5	132236067	2	3.622979
rs708459	LEAP2	5	132236067	7	0.341632
rs3749820	FSTL4	5	132560962	8	8.791005
rs3749820	FSTL4	5	132560962	7	1.829062
rs3749819	FSTL4	5	132561034	8	6.935664
rs13183672	FSTL4	5	132561416	8	2.395023
rs13183672	FSTL4	5	132561416	7	1.954883
rs3798104	FSTL4	5	132561920	8	0.968554
rs3749817	FSTL4	5	132562945	7	0.59733
rs17683306	FSTL4	5	132680180	4	0.832712
rs17683306	FSTL4	5	132680180	5	0.038888
rs17167484	UBE2B	5	133734893	3	0.795334
rs11558540	SAR1B	5	133984567	1	5.458805
rs11558540	SAR1B	5	133984567	6	7.700633
rs1700488	LOC389328	5	134402776	5	1.824273
rs1394600	LOC389328	5	134403217	5	1.945417
rs1394600	LOC389328	5	134403217	1	3.630306
rs3733728	H2AFY	5	134698640	4	0.045857
rs12520809	C5orf20	5	134810409	7	0.075837
rs10515477	LOC729304	5	135089231	5	1.041439
rs2069885	IL9	5	135256064	5	2.48601
rs40986	FBXL21	5	135304213	8	4.945702
rs40986	FBXL21	5	135304213	7	2.879242
rs40986	FBXL21	5	135304213	3	2.607651
rs2066913	FBXL21	5	135304713	5	0.490928
rs2516561	LECT2	5	135310614	5	3.02617
rs1442	TGFBI	5	135410888	7	0.59914

rs1442	TGFBI	5	135410888	4	0.217006
rs1442	TGFBI	5	135410888	3	0.55064
rs1442	TGFBI	5	135410888	6	0.279755
rs1442	TGFBI	5	135410888	2	3.222816
rs1133170	TGFBI	5	135419273	7	0.465942
rs1133170	TGFBI	5	135419273	3	0.262924
rs1133170	TGFBI	5	135419273	2	6.800588
rs10043215	SPOCK1	5	136339938	5	0.847665
rs10043215	SPOCK1	5	136339938	7	1.74575
rs10043215	SPOCK1	5	136339938	2	4.813421
rs1051853	SPOCK1	5	136341700	8	3.245096
rs1051853	SPOCK1	5	136341700	7	1.132532
rs1051853	SPOCK1	5	136341700	3	0.42439
rs3813314	KLHL3	5	136982618	5	0.319264
rs3813314	KLHL3	5	136982618	4	3.050275
rs3813314	KLHL3	5	136982618	1	0.106319
rs3813314	KLHL3	5	136982618	3	0.90509
rs3813314	KLHL3	5	136982618	7	0.650772
rs3813314	KLHL3	5	136982618	2	0.343855
rs3813315	KLHL3	5	136982760	6	1.722481
rs3813315	KLHL3	5	136982760	3	2.251845
rs3813315	KLHL3	5	136982760	4	2.422638
rs3813315	KLHL3	5	136982760	8	6.155691
rs3813315	KLHL3	5	136982760	5	0.159409
rs3813315	KLHL3	5	136982760	7	1.549751
rs2057680	KLHL3	5	136984376	5	0.736008
rs2057680	KLHL3	5	136984376	3	0.105973
rs2057680	KLHL3	5	136984376	2	1.764248
rs2057680	KLHL3	5	136984376	7	0.813346
rs2057680	KLHL3	5	136984376	6	0.709189
rs2057680	KLHL3	5	136984376	4	0.066965
rs7444370	KLHL3	5	136984582	6	0.625186
rs17171525	KLHL3	5	136989465	1	1.944422
rs423258	BRD8	5	137503686	1	1.508036
rs423258	BRD8	5	137503686	3	1.556105
rs10952	GFRA3	5	137616239	5	7.045402
rs10952	GFRA3	5	137616239	4	0.403995
rs10952	GFRA3	5	137616239	1	4.594145
rs10952	GFRA3	5	137616239	3	7.535718
rs10952	GFRA3	5	137616239	8	3.850617
rs3734166	CDC25C	5	137693222	7	2.893744
rs6861656	CDC25C	5	137695411	1	1.036309
rs6861656	CDC25C	5	137695411	5	0.97377
rs10073922	JMJD1B	5	137745112	5	0.484012
rs10073922	JMJD1B	5	137745112	3	1.127822
rs10073922	JMJD1B	5	137745112	1	0.804869
rs10073922	JMJD1B	5	137745112	2	1.142448

rs10073922	JMJD1B	5	137745112	4	0.589924
rs4835796	REEP2	5	137810014	2	3.56779
rs4835796	REEP2	5	137810014	7	0.170687
rs4835796	REEP2	5	137810014	3	0.054163
rs3798153	REEP2	5	137810103	3	0.127811
rs3798153	REEP2	5	137810103	2	5.206563
rs3798153	REEP2	5	137810103	7	0.20703
rs1046576	REEP2	5	137810234	3	0.566967
rs288039	LRRTM2	5	138234099	8	2.47488
rs288039	LRRTM2	5	138234099	3	0.569464
rs288036	LRRTM2	5	138238794	3	0.425897
rs288036	LRRTM2	5	138238794	8	5.617987
rs1059110	CTNNA1	5	138294445	8	0.648178
rs1059110	CTNNA1	5	138294445	3	0.5964
rs17031	CTNNA1	5	138298441	3	0.28683
rs33972313	SLC23A1	5	138743401	1	1.473031
rs10063870	LOC389333	5	138756432	5	1.185088
rs10063870	LOC389333	5	138756432	4	0.893554
rs10063870	LOC389333	5	138756432	8	1.156674
rs10063870	LOC389333	5	138756432	6	0.187357
rs10063870	LOC389333	5	138756432	1	0.932137
rs10900862	LOC202051	5	138760478	2	3.266059
rs10900862	LOC202051	5	138760478	6	4.250639
rs10900862	LOC202051	5	138760478	4	5.767057
rs10900862	LOC202051	5	138760478	8	0.380361
rs10900862	LOC202051	5	138760478	5	0.537116
rs10900862	LOC202051	5	138760478	1	1.335918
rs11554776	TMEM173	5	138841262	5	0.28145
rs11554776	TMEM173	5	138841262	6	0.931147
rs187167	LOC727793	5	139009184	8	0.297692
rs187167	LOC727793	5	139009184	5	0.095976
rs356445	CXXC5	5	139040670	5	7.519178
rs17286536	PSD2	5	139203269	7	0.395096
rs11538394	C5orf32	5	139554234	6	0.400261
rs11538394	C5orf32	5	139554234	3	0.86321
rs11538394	C5orf32	5	139554234	8	3.048146
rs11538394	C5orf32	5	139554234	2	2.89748
rs4150237	HBEGF	5	139693945	2	2.888778
rs1042184	HBEGF	5	139706145	3	3.697985
rs6860077	SLC4A9	5	139725338	5	0.431328
rs6860077	SLC4A9	5	139725338	7	0.408616
rs6860077	SLC4A9	5	139725338	6	0.11241
rs6860077	SLC4A9	5	139725338	2	2.236162
rs2249213	SRA1	5	139917330	7	0.731178
rs2249213	SRA1	5	139917330	4	3.708218
rs2249213	SRA1	5	139917330	6	4.328106
rs2249213	SRA1	5	139917330	1	6.07527

rs2249213	SRA1	5	139917330	5	0.011252
rs2569190	CD14	5	139993100	1	4.336496
rs2569190	CD14	5	139993100	7	4.080829
rs2569190	CD14	5	139993100	5	0.115004
rs2569190	CD14	5	139993100	4	1.739413
rs17208187	TMCO6	5	140003422	2	1.401959
rs17208187	TMCO6	5	140003422	1	0.048099
rs17208187	TMCO6	5	140003422	4	0.240926
rs2286394	WDR55	5	140028728	1	0.023598
rs2286394	WDR55	5	140028728	4	1.492399
SNP5-14002889	WDR55	5	140028891	6	6.770099
GA022788	HARS	5	140037719	3	0.156973
rs3733708	PCDHA3	5	140161918	5	0.093684
rs3733708	PCDHA3	5	140161918	1	0.833505
rs3733708	PCDHA3	5	140161918	7	0.719515
rs3733708	PCDHA3	5	140161918	4	0.400607
rs3733707	PCDHA3	5	140162076	5	0.201699
rs3733707	PCDHA3	5	140162076	4	0.077963
rs3733707	PCDHA3	5	140162076	1	1.592411
rs3733707	PCDHA3	5	140162076	7	0.863631
rs1030163	PCDHA2	5	140165316	5	0.268613
rs1030163	PCDHA2	5	140165316	4	0.005658
rs1030163	PCDHA2	5	140165316	1	1.189263
rs3822346	PCDHA4	5	140167506	1	0.181557
rs3822346	PCDHA4	5	140167506	2	1.05224
rs3822346	PCDHA4	5	140167506	7	1.757613
rs3822346	PCDHA4	5	140167506	5	1.184829
rs3822346	PCDHA4	5	140167506	4	0.964065
rs192231	PCDHA11	5	140228723	4	4.37352
rs192231	PCDHA11	5	140228723	7	0.739599
rs192231	PCDHA11	5	140228723	2	4.16254
rs192231	PCDHA11	5	140228723	8	6.889561
rs192231	PCDHA11	5	140228723	5	0.721963
rs251377	PCDHA12	5	140235976	4	1.071657
rs251377	PCDHA12	5	140235976	2	1.660245
rs251377	PCDHA12	5	140235976	7	1.393448
rs251377	PCDHA12	5	140235976	8	3.179786
rs11744560	PCDHA13	5	140242676	5	0.401054
rs11744560	PCDHA13	5	140242676	2	3.390984
rs155820	PCDHAC1	5	140287930	7	1.330169
rs155360	PCDHAC1	5	140289200	5	1.612973
rs155360	PCDHAC1	5	140289200	4	0.029403
rs155808	PCDHAC2	5	140326896	7	0.541846
rs155807	PCDHAC2	5	140327237	7	0.76892
rs12153295	PCDHAC2	5	140327582	5	0.133757
rs12153295	PCDHAC2	5	140327582	4	0.445438
rs12153295	PCDHAC2	5	140327582	8	9.339281

rs31853	PCDHB2	5	140454940	8	3.201885
rs3733699	PCDHB3	5	140461659	2	4.542874
rs3733699	PCDHB3	5	140461659	4	5.671945
rs3733699	PCDHB3	5	140461659	8	5.561096
rs6887594	PCDHB3	5	140463561	3	1.540963
rs6887594	PCDHB3	5	140463561	7	0.614202
rs3776100	PCDHB4	5	140482361	3	0.73381
rs3733697	PCDHB4	5	140482528	4	1.192718
rs3776099	PCDHB4	5	140483025	8	3.41634
rs3776099	PCDHB4	5	140483025	6	0.021881
rs3776096	PCDHB6	5	140510713	3	1.798274
rs3776096	PCDHB6	5	140510713	7	0.540932
rs17629216	PCDHB6	5	140512593	3	0.080284
rs17286891	PCDHB7	5	140532958	4	0.657959
rs17286891	PCDHB7	5	140532958	2	4.993584
rs17286891	PCDHB7	5	140532958	5	1.124582
rs17286891	PCDHB7	5	140532958	8	0.64402
rs2910313	PCDHB7	5	140533765	4	0.648239
rs2910313	PCDHB7	5	140533765	3	0.013579
rs2910313	PCDHB7	5	140533765	2	1.271171
rs3733695	PCDHB7	5	140533848	4	2.784491
rs3733695	PCDHB7	5	140533848	5	1.647354
rs6892507	PCDHB7	5	140534992	5	0.373844
rs6892507	PCDHB7	5	140534992	8	5.844934
rs6892507	PCDHB7	5	140534992	4	2.3139
rs6892507	PCDHB7	5	140534992	3	0.799869
rs6892507	PCDHB7	5	140534992	7	0.739688
rs6892507	PCDHB7	5	140534992	6	2.202916
rs2950845	PCDHB8	5	140538396	8	6.586397
rs2950845	PCDHB8	5	140538396	7	2.545084
rs2950845	PCDHB8	5	140538396	6	1.576692
rs17096961	PCDHB8	5	140540033	8	0.08908
rs17096961	PCDHB8	5	140540033	5	0.69398
rs2740584	PCDHB16	5	140541952	3	0.960731
rs2740584	PCDHB16	5	140541952	6	0.175536
rs2204704	PCDHB16	5	140542167	4	0.019964
rs2204704	PCDHB16	5	140542167	3	0.912822
rs4151695	PCDHB16	5	140542257	2	2.707733
rs4151695	PCDHB16	5	140542257	4	1.963839
rs4151695	PCDHB16	5	140542257	8	0.788743
rs10069559	PCDHB16	5	140544845	4	0.057317
rs12654953	PCDHB9	5	140551157	5	0.026403
rs12654953	PCDHB9	5	140551157	7	0.650643
rs12657056	PCDHB9	5	140551158	7	4.111154
rs3756323	PCDHB11	5	140559542	5	1.179866
rs3756323	PCDHB11	5	140559542	8	1.720998
rs3756323	PCDHB11	5	140559542	4	1.606989

rs917535	PCDHB11	5	140559551	6	0.141029
rs638054	PCDHB11	5	140561694	3	0.580052
rs638054	PCDHB11	5	140561694	7	4.576405
rs2910006	PCDHB12	5	140570950	4	0.908006
rs2910006	PCDHB12	5	140570950	2	0.795439
rs2910006	PCDHB12	5	140570950	7	1.681987
rs2910333	PCDHB13	5	140576888	4	3.332072
rs2910333	PCDHB13	5	140576888	7	1.606737
rs2907309	PCDHB18	5	140594222	7	1.967344
rs2907309	PCDHB18	5	140594222	8	1.375964
rs2907309	PCDHB18	5	140594222	5	3.328719
rs2907307	PCDHB18	5	140595453	5	0.420718
rs2907307	PCDHB18	5	140595453	7	1.256071
rs3749780	SLC25A2	5	140662941	2	2.633518
rs3749780	SLC25A2	5	140662941	5	0.200966
rs11952797	SLC25A2	5	140663449	1	4.570318
rs11952797	SLC25A2	5	140663449	5	0.231817
rs11952797	SLC25A2	5	140663449	7	1.496959
rs17097187	PCDHGA1	5	140692037	3	2.302563
rs17097187	PCDHGA1	5	140692037	6	1.99111
rs11575947	PCDHGA3	5	140704019	3	0.938852
rs17097231	PCDHGB2	5	140719963	2	4.661026
rs2240697	PCDHGB3	5	140731312	1	5.704838
rs2240697	PCDHGB3	5	140731312	5	0.200887
rs2240697	PCDHGB3	5	140731312	7	0.391936
rs2024141	PCDHGA7	5	140745184	2	2.174466
rs4151699	PCDHGA8	5	140752378	7	4.369447
rs4151699	PCDHGA8	5	140752378	5	0.664751
rs4151699	PCDHGA8	5	140752378	4	0.317493
rs4912606	PCDHGA9	5	140765079	1	4.933019
rs3749768	PCDHGB6	5	140770003	2	3.038842
rs17208397	PCDHGB7	5	140778823	1	6.248883
rs17208397	PCDHGB7	5	140778823	5	0.488521
rs7732160	PCDHGA11	5	140780892	2	5.387524
rs3805695	PCDHGC3	5	140838429	2	4.572576
rs2074912	PCDHGC5	5	140850700	1	2.152168
rs2074912	PCDHGC5	5	140850700	5	0.149163
rs2074912	PCDHGC5	5	140850700	7	0.7616
rs918558	PCDHGC5	5	140851133	5	0.499538
rs918558	PCDHGC5	5	140851133	1	4.066014
rs32955	FCHSD1	5	140999497	2	7.981149
rs32955	FCHSD1	5	140999497	6	2.009791
rs32956	FCHSD1	5	141000065	6	4.512668
rs41098	FCHSD1	5	141000284	6	1.499401
SNP5-141004181	FCHSD1	5	141004181	4	0.23348
rs17287002	FCHSD1	5	141010824	1	4.12157
rs3822357	PCDH1	5	141224540	7	7.749855

rs153148	PCDH12	5	141304807	7	1.207862
rs153148	PCDH12	5	141304807	6	1.505951
rs153148	PCDH12	5	141304807	1	0.781909
rs153148	PCDH12	5	141304807	5	0.863202
rs153148	PCDH12	5	141304807	3	0.811633
rs153148	PCDH12	5	141304807	8	0.464345
rs153148	PCDH12	5	141304807	4	1.43522
rs164073	PCDH12	5	141315425	2	0.295557
rs164073	PCDH12	5	141315425	8	0.680821
rs164073	PCDH12	5	141315425	5	0.299522
rs164073	PCDH12	5	141315425	7	0.198048
rs164073	PCDH12	5	141315425	4	0.227153
rs164073	PCDH12	5	141315425	1	0.23868
rs164073	PCDH12	5	141315425	6	0.013266
rs164074	PCDH12	5	141315468	5	0.246564
rs164074	PCDH12	5	141315468	2	2.967345
rs164075	PCDH12	5	141316448	7	0.90157
rs164075	PCDH12	5	141316448	3	1.313781
rs164075	PCDH12	5	141316448	2	1.292331
rs164075	PCDH12	5	141316448	8	2.560707
rs164075	PCDH12	5	141316448	4	1.295231
rs164075	PCDH12	5	141316448	1	0.242621
rs164075	PCDH12	5	141316448	5	4.214201
rs164076	PCDH12	5	141317310	6	0.731838
rs164076	PCDH12	5	141317310	4	0.452781
rs164076	PCDH12	5	141317310	3	0.080841
rs164076	PCDH12	5	141317310	5	0.411347
rs164077	PCDH12	5	141317409	2	0.774253
rs164077	PCDH12	5	141317409	1	1.10642
rs187543	RNF14	5	141328818	1	0.260931
rs252133	RNF14	5	141328892	1	2.199314
rs252133	RNF14	5	141328892	7	0.761912
rs364287	RNF14	5	141347991	1	5.795442
rs364287	RNF14	5	141347991	7	0.239464
rs364287	RNF14	5	141347991	6	7.080971
rs33999	FGF1	5	141953533	3	0.251493
rs33999	FGF1	5	141953533	2	1.992974
rs34000	FGF1	5	141953685	2	1.324661
rs34000	FGF1	5	141953685	6	0.098818
rs34000	FGF1	5	141953685	3	0.708802
rs2278688	FGF1	5	141954266	8	0.605973
rs17099029	FGF1	5	141954717	8	3.512228
rs34002	FGF1	5	141954756	2	5.35501
rs34002	FGF1	5	141954756	3	1.600209
rs34002	FGF1	5	141954756	6	3.217995
rs187034	KCTD16	5	143834797	1	3.614239
rs187034	KCTD16	5	143834797	3	0.016156

rs349704	KCTD16	5	143835224	1	3.895603
rs349704	KCTD16	5	143835224	3	0.171304
rs349703	KCTD16	5	143835275	1	3.155815
rs349703	KCTD16	5	143835275	3	0.334751
rs358651	KCTD16	5	143835799	1	2.603361
rs349701	KCTD16	5	143835846	3	0.045862
rs349701	KCTD16	5	143835846	1	0.776113
rs349700	KCTD16	5	143836108	8	0.206258
rs349700	KCTD16	5	143836108	1	3.53634
rs349700	KCTD16	5	143836108	3	0.067617
rs349698	KCTD16	5	143836721	1	3.158065
rs349698	KCTD16	5	143836721	8	0.659096
rs349698	KCTD16	5	143836721	3	1.149156
rs2569006	LOC643226	5	145226278	5	0.399052
rs2569006	LOC643226	5	145226278	3	0.389023
rs2569006	LOC643226	5	145226278	1	0.515617
rs6877277	PLAC8L1	5	145458048	6	0.792285
rs6877277	PLAC8L1	5	145458048	4	1.842486
rs6877277	PLAC8L1	5	145458048	1	4.12885
rs6877277	PLAC8L1	5	145458048	7	1.719999
rs12187913	PLAC8L1	5	145464037	6	0.266393
rs12187913	PLAC8L1	5	145464037	1	4.825839
rs12187913	PLAC8L1	5	145464037	4	0.41037
rs11954297	RBM27	5	145648491	4	7.002314
rs11954297	RBM27	5	145648491	7	0.265908
rs11954297	RBM27	5	145648491	1	4.101001
rs11954297	RBM27	5	145648491	6	6.144507
rs4705103	TCERG1	5	145870221	1	2.554643
rs4705103	TCERG1	5	145870221	3	0.0131
rs7709485	GPR151	5	145875089	4	0.708377
rs7709485	GPR151	5	145875089	3	0.460774
rs7709485	GPR151	5	145875089	2	4.086778
rs4705132	STK32A	5	146599399	1	3.621735
rs4705132	STK32A	5	146599399	7	0.545315
rs4705132	STK32A	5	146599399	6	0.098111
rs4705132	STK32A	5	146599399	3	2.917453
rs2116766	JAKMIP2	5	147004669	7	1.856314
rs2116765	JAKMIP2	5	147004702	3	0.801598
rs11319	SPINK1	5	147184385	5	0.19162
rs11956228	MGC23985	5	147261501	7	0.617673
rs7722926	MGC23985	5	147266206	7	1.763754
rs6892205	SPINK5	5	147455579	5	1.689853
rs6892205	SPINK5	5	147455579	3	1.852299
rs34482796	SPINK5	5	147457744	5	0.650219
rs34482796	SPINK5	5	147457744	6	0.030744
rs34482796	SPINK5	5	147457744	3	1.046623
rs2303063	SPINK5	5	147460220	3	0.338631

rs2303063	SPINK5	5	147460220	6	0.612261
rs2303063	SPINK5	5	147460220	5	0.185258
rs33920397	SPINK5	5	147479863	4	1.373084
rs33920397	SPINK5	5	147479863	5	1.431464
rs1042711	ADRB2	5	148186541	3	0.527321
rs1042711	ADRB2	5	148186541	1	4.395228
rs1042711	ADRB2	5	148186541	8	0.873138
rs1042717	ADRB2	5	148186839	7	0.666804
rs2217638	SH3TC2	5	148344548	8	0.29645
rs4560536	SH3TC2	5	148344894	8	0.260692
rs4560536	SH3TC2	5	148344894	4	0.871535
rs4560536	SH3TC2	5	148344894	6	0.616884
rs10040798	SH3TC2	5	148347517	8	3.957342
rs4705303	SH3TC2	5	148347836	8	0.59555
rs4705303	SH3TC2	5	148347836	4	1.110963
rs4562031	SH3TC2	5	148348644	8	0.876576
rs891919	SH3TC2	5	148349701	2	3.839196
rs891919	SH3TC2	5	148349701	4	0.719365
rs891919	SH3TC2	5	148349701	8	5.164049
rs891919	SH3TC2	5	148349701	6	0.150286
rs17109198	SH3TC2	5	148349905	8	0.560929
rs2069087	SH3TC2	5	148353347	6	7.147709
rs1347130	SH3TC2	5	148357175	8	3.605756
rs1347130	SH3TC2	5	148357175	6	6.367854
rs1347130	SH3TC2	5	148357175	4	0.752638
rs17109208	SH3TC2	5	148358668	8	6.989445
rs6885467	SH3TC2	5	148359748	4	0.620648
rs6885467	SH3TC2	5	148359748	2	0.273107
rs998304	SH3TC2	5	148362558	8	0.966073
rs6875902	SH3TC2	5	148388086	8	1.728118
rs17722293	SH3TC2	5	148402467	6	4.030449
rs4705336	PCYOX1L	5	148728095	5	7.440279
rs4705336	PCYOX1L	5	148728095	8	0.187691
rs4705336	PCYOX1L	5	148728095	1	0.59621
rs4705336	PCYOX1L	5	148728095	2	0.480197
rs2400891	FLJ41603	5	148969315	5	0.373661
rs2400891	FLJ41603	5	148969315	2	1.494471
rs2400891	FLJ41603	5	148969315	8	1.014045
rs2400891	FLJ41603	5	148969315	1	1.258941
rs2400891	FLJ41603	5	148969315	6	1.114457
rs2400891	FLJ41603	5	148969315	3	1.394207
SNP5-14898382(	FLJ41603	5	148983820	7	0.188722
rs9324624	FLJ41603	5	148986833	2	9.504594
rs9324624	FLJ41603	5	148986833	4	0.75502
rs9324624	FLJ41603	5	148986833	6	0.240552
rs9324624	FLJ41603	5	148986833	5	0.887572
rs9324624	FLJ41603	5	148986833	7	1.079466

rs9324624	FLJ41603	5	148986833	3	0.406419
rs9324624	FLJ41603	5	148986833	1	0.66976
rs2279134	FLJ41603	5	148992244	1	0.404856
rs2279134	FLJ41603	5	148992244	6	2.861435
rs2279134	FLJ41603	5	148992244	8	0.65912
rs2279134	FLJ41603	5	148992244	2	0.497683
rs2279134	FLJ41603	5	148992244	5	3.743855
rs2279134	FLJ41603	5	148992244	4	0.381604
rs2279134	FLJ41603	5	148992244	3	0.035105
rs32588	PPARGC1B	5	149180236	2	3.716182
rs32588	PPARGC1B	5	149180236	6	0.574418
rs32588	PPARGC1B	5	149180236	7	0.564457
rs32588	PPARGC1B	5	149180236	1	0.006368
rs17572019	PPARGC1B	5	149192664	5	0.053171
rs17572019	PPARGC1B	5	149192664	8	3.978895
rs11959820	PPARGC1B	5	149192703	7	0.702437
rs32577	PPARGC1B	5	149192993	7	0.325696
rs32577	PPARGC1B	5	149192993	2	0.812413
rs32577	PPARGC1B	5	149192993	1	0.207872
rs2277925	PDE6A	5	149304099	5	2.420038
rs10875553	TIGD6	5	149355125	3	0.445061
rs10875553	TIGD6	5	149355125	8	7.133686
rs10875553	TIGD6	5	149355125	1	3.053366
rs3828609	CSF1R	5	149413056	6	1.133288
rs13117	CSF1R	5	149413313	7	0.730608
rs13117	CSF1R	5	149413313	2	2.556773
rs13117	CSF1R	5	149413313	6	0.131481
SNP5-14943002(	CSF1R	5	149430020	8	2.072401
rs10079250	CSF1R	5	149430325	7	0.628579
rs2228422	CSF1R	5	149437871	7	0.094014
rs2228422	CSF1R	5	149437871	8	0.113333
rs2228422	CSF1R	5	149437871	3	0.509581
rs216123	CSF1R	5	149440746	8	1.50277
rs216123	CSF1R	5	149440746	7	1.176953
rs216123	CSF1R	5	149440746	2	0.405728
rs216123	CSF1R	5	149440746	3	0.20934
rs216123	CSF1R	5	149440746	4	0.792943
rs2240793	SLC6A7	5	149563493	6	1.305101
rs2240793	SLC6A7	5	149563493	2	1.770645
rs2240794	SLC6A7	5	149563529	2	6.570107
rs2240794	SLC6A7	5	149563529	8	0.103845
rs2240794	SLC6A7	5	149563529	6	0.783373
rs2240795	SLC6A7	5	149569840	7	0.769289
rs13153325	SLC6A7	5	149570326	1	0.604131
rs13153325	SLC6A7	5	149570326	7	0.243723
rs7713638	TCOF1	5	149739289	4	0.936629
rs7713638	TCOF1	5	149739289	7	0.874975

rs2273232	NDST1	5	149892720	3	0.750941
rs6863809	NDST1	5	149914306	6	1.579569
rs11954652	DCTN4	5	150078076	3	0.707695
rs11548	GPX3	5	150387976	3	1.519254
rs2233290	TNIP1	5	150416696	3	0.150179
rs1133202	ANXA6	5	150469583	1	0.45891
rs2228458	ANXA6	5	150499181	5	2.821007
rs2228458	ANXA6	5	150499181	3	0.607601
rs2228458	ANXA6	5	150499181	1	1.38245
rs248427	CCDC69	5	150545201	6	5.016304
rs248427	CCDC69	5	150545201	4	3.35849
SNP5-150627081	GM2A	5	150627081	5	3.486495
SNP5-150627081	GM2A	5	150627081	2	3.16761
SNP5-150627081	GM2A	5	150627081	1	3.450934
SNP5-150627081	GM2A	5	150627081	6	3.924047
SNP5-150627081	GM2A	5	150627081	3	2.866333
SNP5-150627081	GM2A	5	150627081	4	10.7952
rs11167542	LOC402233	5	150740475	2	1.799529
rs11167542	LOC402233	5	150740475	5	1.889845
rs11167542	LOC402233	5	150740475	6	0.97073
rs165323	SLC36A1	5	150852105	4	0.31808
rs165342	FAT2	5	150864986	8	4.289435
rs165342	FAT2	5	150864986	4	0.47182
rs165341	FAT2	5	150865032	4	0.170623
rs165341	FAT2	5	150865032	8	5.689437
rs165341	FAT2	5	150865032	2	2.963445
rs2304029	FAT2	5	150871965	3	0.221278
rs3734047	FAT2	5	150881790	7	0.337136
rs3734047	FAT2	5	150881790	5	0.3887
rs3734049	FAT2	5	150881823	7	0.265872
rs3734049	FAT2	5	150881823	5	0.010968
rs2304024	FAT2	5	150889006	5	5.196482
rs1432643	FAT2	5	150894377	8	4.228434
rs1432643	FAT2	5	150894377	3	0.077741
rs1432643	FAT2	5	150894377	2	5.117976
rs998076	FAT2	5	150904962	7	0.697573
rs3549	SPARC	5	151021343	2	2.734582
rs3549	SPARC	5	151021343	3	0.976386
rs3549	SPARC	5	151021343	4	3.241084
rs1054204	SPARC	5	151022743	1	0.140676
rs1054204	SPARC	5	151022743	4	0.94886
rs1054204	SPARC	5	151022743	2	1.381932
rs1054204	SPARC	5	151022743	3	0.121702
rs1054204	SPARC	5	151022743	6	0.02253
rs982716	NMUR2	5	151751549	4	3.45922
rs982716	NMUR2	5	151751549	3	3.691182
rs4958303	NMUR2	5	151751866	4	4.225041

rs4958303	NMUR2	5	151751866	3	4.240173
rs4958532	NMUR2	5	151752045	3	5.080153
rs4958532	NMUR2	5	151752045	4	2.127999
rs1895245	NMUR2	5	151752248	3	2.859332
rs478020	MFAP3	5	153409362	7	1.174531
rs478020	MFAP3	5	153409362	2	3.630205
rs478020	MFAP3	5	153409362	4	0.478928
rs17535045	MFAP3	5	153413749	1	5.803039
rs17535045	MFAP3	5	153413749	7	0.648362
rs4958733	GALNT10	5	153763780	5	0.236869
rs4958733	GALNT10	5	153763780	6	0.716832
rs4958733	GALNT10	5	153763780	7	0.678077
rs4958733	GALNT10	5	153763780	2	2.236962
rs4958733	GALNT10	5	153763780	8	1.00418
rs6580076	GALNT10	5	153763946	4	4.616741
rs6580076	GALNT10	5	153763946	1	0.301183
rs6580076	GALNT10	5	153763946	8	0.417228
rs888979	GALNT10	5	153777068	5	0.969236
rs888979	GALNT10	5	153777068	8	3.779418
rs888979	GALNT10	5	153777068	1	4.930996
rs888979	GALNT10	5	153777068	4	4.631357
rs3776997	GALNT10	5	153777300	3	0.104296
rs3822711	GALNT10	5	153777488	1	5.31198
rs3822711	GALNT10	5	153777488	7	4.425791
rs3172941	GALNT10	5	153778073	7	0.169751
rs3172941	GALNT10	5	153778073	1	0.410724
rs3172941	GALNT10	5	153778073	6	5.805071
rs3172941	GALNT10	5	153778073	4	4.625449
rs3172941	GALNT10	5	153778073	5	5.054381
rs3172941	GALNT10	5	153778073	2	0.308239
rs10796	GALNT10	5	153778561	5	0.25274
rs10796	GALNT10	5	153778561	4	1.167237
rs10796	GALNT10	5	153778561	2	4.757256
rs3867430	C5orf4	5	154180805	5	0.150068
rs3867430	C5orf4	5	154180805	8	0.247604
rs3867430	C5orf4	5	154180805	3	0.254022
rs3867430	C5orf4	5	154180805	6	0.544839
rs3867430	C5orf4	5	154180805	4	0.385091
rs6865950	GEMIN5	5	154255979	2	5.206022
rs3857412	SGCD	5	156119760	5	0.871275
rs284445	SGCD	5	156120506	4	0.353874
rs284445	SGCD	5	156120506	1	2.249686
rs284445	SGCD	5	156120506	2	0.601892
rs284445	SGCD	5	156120506	8	0.153562
rs1389818	SGCD	5	156120597	5	1.074177
rs10041876	SGCD	5	156123374	5	0.315001
rs1845479	SGCD	5	156123792	5	0.116809

rs3913482	SGCD	5	156124093	5	0.673926
rs12522248	HAVCR1	5	156412004	1	2.961104
rs1553316	HAVCR1	5	156412087	6	0.438145
rs1553316	HAVCR1	5	156412087	4	0.508324
rs9313439	HAVCR2	5	156445760	4	0.601041
rs9313439	HAVCR2	5	156445760	8	0.756726
rs9313439	HAVCR2	5	156445760	6	0.732869
rs27988	ITK	5	156613263	3	2.878927
rs7715719	C5orf40	5	156702246	5	0.082627
rs7715719	C5orf40	5	156702246	1	1.088936
rs7715719	C5orf40	5	156702246	6	0.044188
rs7715719	C5orf40	5	156702246	4	0.242487
rs7715719	C5orf40	5	156702246	2	0.29015
rs7715719	C5orf40	5	156702246	7	0.233494
rs10037485	C5orf40	5	156702711	2	0.245505
rs10037485	C5orf40	5	156702711	1	0.839598
rs10037485	C5orf40	5	156702711	7	0.273898
rs10037485	C5orf40	5	156702711	4	1.487598
rs10214194	C5orf40	5	156702787	5	0.324098
rs6860507	ICHTHYIN	5	156831268	7	1.102757
rs6860507	ICHTHYIN	5	156831268	1	0.97774
SNP5-15683250[illegible]	ICHTHYIN	5	156832503	8	7.54426
rs3822692	ICHTHYIN	5	156833456	1	0.658029
rs3822692	ICHTHYIN	5	156833456	4	1.031199
rs3822692	ICHTHYIN	5	156833456	2	2.5849
rs3822692	ICHTHYIN	5	156833456	5	0.916705
rs10404	ADAM19	5	156837211	6	5.411883
rs10404	ADAM19	5	156837211	1	4.866078
rs11466822	ADAM19	5	156838095	7	0.680913
rs11466822	ADAM19	5	156838095	1	2.385123
rs11466822	ADAM19	5	156838095	5	1.910987
rs6895849	ADAM19	5	156839397	8	0.081174
rs3822585	ADAM19	5	156839564	2	0.177551
rs11466816	ADAM19	5	156839688	7	1.241238
rs11466816	ADAM19	5	156839688	6	1.464474
rs11466816	ADAM19	5	156839688	5	0.327531
rs11466816	ADAM19	5	156839688	2	3.806566
rs11466815	ADAM19	5	156839749	5	0.810356
rs11466815	ADAM19	5	156839749	7	0.581864
rs10067096	ADAM19	5	156849918	7	0.073356
rs10067096	ADAM19	5	156849918	5	0.254777
rs3749797	SOX30	5	157011053	1	0.08418
rs2270819	THG1L	5	157090992	3	0.048231
rs2270819	THG1L	5	157090992	5	0.161483
rs2270819	THG1L	5	157090992	2	2.802446
rs2270819	THG1L	5	157090992	1	0.113938
rs2270818	THG1L	5	157091017	1	0.434195

rs2270818	THG1L	5	157091017	2	0.6221
rs2270818	THG1L	5	157091017	3	0.947278
rs2270818	THG1L	5	157091017	5	0.227766
rs2270817	THG1L	5	157091131	3	0.329702
rs2270817	THG1L	5	157091131	7	4.010381
rs2279770	LSM11	5	157116317	2	1.19002
rs2279770	LSM11	5	157116317	5	0.501324
rs2279770	LSM11	5	157116317	7	0.800238
rs2279770	LSM11	5	157116317	1	0.886935
rs3212227	IL12B	5	158675528	3	0.301344
rs3213120	IL12B	5	158675686	4	0.121869
rs3213119	IL12B	5	158676366	4	0.228167
rs3729604	ADRA1B	5	159277039	5	6.535221
rs13161018	FABP6	5	159591851	4	0.296674
rs13161018	FABP6	5	159591851	3	0.929015
rs13161018	FABP6	5	159591851	2	0.899769
rs13161018	FABP6	5	159591851	1	0.480231
rs9216	C1QTNF2	5	159708625	1	0.288282
rs6892951	ATP10B	5	159922761	2	4.715007
rs6892951	ATP10B	5	159922761	6	0.009507
rs6892951	ATP10B	5	159922761	5	7.002282
rs6892951	ATP10B	5	159922761	4	5.447832
rs6892951	ATP10B	5	159922761	8	3.221508
rs6892951	ATP10B	5	159922761	1	1.046532
rs6894908	ATP10B	5	160048357	4	0.205298
rs6894908	ATP10B	5	160048357	3	0.511389
rs6894908	ATP10B	5	160048357	7	1.146914
rs13188991	GABRA6	5	161045582	1	1.622876
rs211037	GABRG2	5	161460858	5	0.841127
rs211037	GABRG2	5	161460858	4	0.415754
rs299284	HMMR	5	162829228	3	0.082634
rs17061795	MAT2B	5	162873577	3	0.086135
rs17070023	ODZ2	5	167607050	7	0.458959
rs17070023	ODZ2	5	167607050	2	4.964389
rs17632540	ODZ2	5	167621874	5	0.037862
rs17632540	ODZ2	5	167621874	1	0.890331
rs17632540	ODZ2	5	167621874	2	2.84558
rs17551608	WWC1	5	167768117	1	2.955529
SNP5-16781361(	WWC1	5	167813610	3	0.043138
SNP5-16781361(	WWC1	5	167813610	8	6.154069
SNP5-16781361(	WWC1	5	167813610	1	3.374201
SNP5-16781361(	WWC1	5	167813610	4	5.670965
rs292486	SLIT3	5	168031041	3	0.817882
rs292486	SLIT3	5	168031041	4	0.211551
rs35305517	SLIT3	5	168045505	6	5.657354
rs3797715	SLIT3	5	168166052	1	5.002887
rs4867964	SLIT3	5	168177025	3	0.693379

rs4867964	SLIT3	5	168177025	6	1.31798
rs17558310	CCDC99	5	168943373	5	0.59385
rs17558310	CCDC99	5	168943373	2	4.962607
rs3797713	CCDC99	5	168961059	4	5.976123
rs3797713	CCDC99	5	168961059	3	0.256807
rs3797713	CCDC99	5	168961059	6	5.393253
rs2306559	DOCK2	5	169062000	1	0.456746
rs13155521	DOCK2	5	169107062	2	3.215881
rs2287727	DOCK2	5	169368291	5	0.967954
rs2287727	DOCK2	5	169368291	3	0.907104
rs2287727	DOCK2	5	169368291	1	4.869434
rs9791113	DOCK2	5	169387519	4	0.87739
rs1045168	DOCK2	5	169437321	4	0.328304
rs1045168	DOCK2	5	169437321	6	0.73448
rs1045176	DOCK2	5	169442598	4	1.90814
rs1045176	DOCK2	5	169442598	2	3.928506
rs1045176	DOCK2	5	169442598	7	1.208717
rs9307	DOCK2	5	169442601	2	3.457377
rs9307	DOCK2	5	169442601	7	0.993708
rs9307	DOCK2	5	169442601	4	0.501783
rs9307	DOCK2	5	169442601	5	0.089073
rs1045192	DOCK2	5	169442768	4	0.005822
rs1045192	DOCK2	5	169442768	7	1.482005
rs6873124	FOXI1	5	169468312	2	0.741948
rs6873124	FOXI1	5	169468312	4	4.450982
rs6873124	FOXI1	5	169468312	7	0.349491
rs10039796	LCP2	5	169607743	3	0.322324
rs10039796	LCP2	5	169607743	7	1.60079
rs10039796	LCP2	5	169607743	1	0.346019
rs10039796	LCP2	5	169607743	5	1.117741
rs315717	LCP2	5	169617741	2	3.096692
rs314156	KCNMB1	5	169737989	8	0.29434
rs314156	KCNMB1	5	169737989	6	1.612372
rs2656841	KCNMB1	5	169738207	1	0.280141
rs2656841	KCNMB1	5	169738207	2	2.39515
rs11739136	KCNMB1	5	169743374	4	1.795592
rs11739136	KCNMB1	5	169743374	5	2.924434
rs17563665	KCNMB1	5	169748849	1	2.187215
rs1055381	KCNIP1	5	170095949	3	1.274703
rs1055381	KCNIP1	5	170095949	5	2.669615
rs7725726	GABRP	5	170148248	2	2.550255
rs2914331	RANBP17	5	170658852	2	5.778014
rs2914331	RANBP17	5	170658852	7	0.364054
rs2914331	RANBP17	5	170658852	1	0.0933
rs2914331	RANBP17	5	170658852	3	0.636578
rs4868141	STK10	5	171456041	5	5.434467
rs10050631	SH3PXD2B	5	171696779	5	1.05384

rs10050631	SH3PXD2B	5	171696779	2	5.590333
rs10050631	SH3PXD2B	5	171696779	7	0.688532
rs17714774	LOC401218	5	172122233	4	3.715303
rs17075508	SNORA74B	5	172380368	6	0.690874
rs17075508	SNORA74B	5	172380368	4	6.783325
rs1965668	LOC391850	5	172586007	6	2.292818
rs1965668	LOC391850	5	172586007	7	3.358421
rs1965668	LOC391850	5	172586007	3	2.761003
rs703752	NKX2-5	5	172592117	8	1.300791
rs703752	NKX2-5	5	172592117	4	0.73212
rs703752	NKX2-5	5	172592117	3	0.229763
rs703752	NKX2-5	5	172592117	2	1.447767
rs703752	NKX2-5	5	172592117	1	2.025343
rs703752	NKX2-5	5	172592117	5	1.134315
rs2277923	NKX2-5	5	172594630	1	1.066867
rs1564823	CPEB4	5	173315800	1	5.099043
rs4457100	HMP19	5	173467483	4	0.996425
rs4457100	HMP19	5	173467483	2	0.527802
rs14459	MSX2	5	174090317	3	0.280931
rs156373	SFXN1	5	174887467	3	0.383231
rs156373	SFXN1	5	174887467	7	1.308793
rs10516139	UBXD8	5	175868923	4	1.233327
rs17078320	PCLKC	5	175930852	1	0.950093
rs2358747	PCLKC	5	175938083	1	0.723293
rs4868660	PCLKC	5	175944495	1	0.562636
rs4868660	PCLKC	5	175944495	7	0.57557
rs2291442	PCLKC	5	175950061	3	0.88353
rs2291442	PCLKC	5	175950061	7	0.243918
rs3736432	UNC5A	5	176240110	1	6.432775
rs3736432	UNC5A	5	176240110	4	0.326517
rs3736432	UNC5A	5	176240110	3	2.766787
rs2278492	HK3	5	176247065	1	0.744566
rs3733876	UIMC1	5	176315601	1	3.894199
rs1966265	FGFR4	5	176449237	7	0.394601
rs376618	FGFR4	5	176450403	5	1.034928
rs376618	FGFR4	5	176450403	3	1.515191
rs9489	FGFR4	5	176457635	1	4.887013
rs1363405	NSD1	5	176569488	3	0.972965
rs1363405	NSD1	5	176569488	2	4.785522
rs1363405	NSD1	5	176569488	6	0.264333
rs3733875	NSD1	5	176569846	2	5.071806
SNP5-176571112	NSD1	5	176571112	3	0.0801
SNP5-176653757	NSD1	5	176653757	8	0.227251
SNP5-176653757	NSD1	5	176653757	3	0.098113
rs11740250	NSD1	5	176653878	1	4.97353
rs11740250	NSD1	5	176653878	3	0.068112
rs11740250	NSD1	5	176653878	8	6.581292

rs9313754	RAB24	5	176663284	5	0.215526
rs9313754	RAB24	5	176663284	7	4.409489
rs9313754	RAB24	5	176663284	3	0.131822
rs28670911	PRELID1	5	176665722	2	6.433679
rs17876047	F12	5	176764195	6	0.965948
rs1801020	F12	5	176769138	2	3.949561
rs335435	GRK6	5	176800549	6	4.631641
GA004936	PDLIM7	5	176850503	1	4.312278
rs6634	TMED9	5	176955704	7	2.877343
rs6634	TMED9	5	176955704	8	0.398545
rs6634	TMED9	5	176955704	2	2.873656
rs6634	TMED9	5	176955704	3	0.415029
rs6886539	LOC202181	5	176991736	5	1.089321
rs6886539	LOC202181	5	176991736	7	0.989854
rs6886539	LOC202181	5	176991736	8	0.007113
rs2913858	AGXT2L2	5	177592384	3	0.011871
rs13741	COL23A1	5	177597606	6	3.582482
rs1544925	COL23A1	5	177598092	7	0.452578
rs890802	COL23A1	5	177615983	3	0.74734
rs890802	COL23A1	5	177615983	7	0.292376
rs2004503	LOC643618	5	178269728	3	0.800322
rs2004503	LOC643618	5	178269728	1	0.995283
rs732531	LOC643618	5	178269821	6	3.100894
rs732531	LOC643618	5	178269821	5	3.360452
rs732531	LOC643618	5	178269821	1	4.420399
rs732531	LOC643618	5	178269821	8	7.163312
rs732531	LOC643618	5	178269821	2	0.167177
rs732531	LOC643618	5	178269821	7	4.536249
rs7724141	ZFP2	5	178272661	5	5.190791
rs7724141	ZFP2	5	178272661	4	0.693387
rs12187167	ZFP2	5	178292428	1	6.011194
rs12187167	ZFP2	5	178292428	4	0.848736
rs12187167	ZFP2	5	178292428	5	0.537923
rs12186577	ZFP2	5	178292595	4	0.281879
rs12186577	ZFP2	5	178292595	5	0.323839
rs12186577	ZFP2	5	178292595	1	3.72952
rs12186584	ZFP2	5	178292662	5	1.505817
rs12186584	ZFP2	5	178292662	1	5.567727
rs12186584	ZFP2	5	178292662	4	0.695944
rs6867221	ZNF454	5	178324466	7	0.510466
rs6867221	ZNF454	5	178324466	3	0.476747
rs12719860	ZNF454	5	178324508	3	0.789347
rs12719860	ZNF454	5	178324508	7	0.326035
rs7725272	GRM6	5	178338994	2	2.37795
rs7725272	GRM6	5	178338994	7	0.343698
rs7725272	GRM6	5	178338994	3	0.776161
rs17078875	GRM6	5	178342556	3	0.406007

rs11746675	GRM6	5	178346553	5	0.211244
rs2071246	GRM6	5	178348894	1	1.61189
rs1445846	ZNF354C	5	178439675	3	0.384933
rs1445846	ZNF354C	5	178439675	8	4.496041
rs1445846	ZNF354C	5	178439675	4	3.730467
rs1445846	ZNF354C	5	178439675	2	5.768572
rs1445844	ZNF354C	5	178439876	4	0.449354
rs1445844	ZNF354C	5	178439876	3	0.516535
rs1054480	ADAMTS2	5	178473581	8	0.446896
rs1054480	ADAMTS2	5	178473581	1	2.374201
rs2303644	ADAMTS2	5	178487651	8	1.232667
rs1972715	ADAMTS2	5	178495573	8	1.603302
rs398829	ADAMTS2	5	178567278	7	0.194834
rs4701135	RUFY1	5	178953228	5	0.130083
rs4701135	RUFY1	5	178953228	2	1.001187
rs4701135	RUFY1	5	178953228	7	0.169118
rs7700649	LOC729595	5	179053869	8	0.829385
rs7700649	LOC729595	5	179053869	7	1.78402
rs6895902	MAML1	5	179134453	5	4.911647
rs6895902	MAML1	5	179134453	3	0.324789
rs6895902	MAML1	5	179134453	6	0.077264
rs10060182	LOC51149	5	179218358	8	0.130717
rs10060182	LOC51149	5	179218358	4	1.937824
rs10060182	LOC51149	5	179218358	7	0.263088
SNP5-179235527	TBC1D9B	5	179235527	8	6.342298
SNP5-179235527	TBC1D9B	5	179235527	6	0.009124
rs2278662	LOC729618	5	179460501	1	3.853142
rs2278661	RASGEF1C	5	179460673	1	2.082265
rs2278661	RASGEF1C	5	179460673	7	1.220221
rs7725201	RASGEF1C	5	179461730	1	3.575384
rs7725201	RASGEF1C	5	179461730	4	0.894711
rs7725201	RASGEF1C	5	179461730	8	4.223822
rs11546322	RASGEF1C	5	179496041	8	0.64463
rs11546322	RASGEF1C	5	179496041	3	0.045564
rs11546322	RASGEF1C	5	179496041	2	2.902571
rs7725	GFPT2	5	179660563	3	1.264361
rs2303007	GFPT2	5	179673433	3	0.397739
rs2127379	LOC643836	5	180220173	7	0.584227
rs2127380	LOC643836	5	180220238	3	0.529564
rs2127380	LOC643836	5	180220238	5	0.251476
rs2127380	LOC643836	5	180220238	6	5.378419
rs2127380	LOC643836	5	180220238	4	4.377182
rs7703365	BTNL8	5	180309624	5	0.986181
rs7703365	BTNL8	5	180309624	3	0.017834
rs34645360	BTNL3	5	180365650	1	0.268063
rs34645360	BTNL3	5	180365650	3	0.3952
rs10434808	BTNL9	5	180419691	5	0.039802

rs10434808	BTNL9	5	180419691	3	0.424946
rs10434810	BTNL9	5	180419750	3	0.547304
rs2545098	TRIM41	5	180583350	6	1.028445
rs2545098	TRIM41	5	180583350	1	1.23849
rs2545098	TRIM41	5	180583350	2	0.825733
rs2545098	TRIM41	5	180583350	5	0.439978
rs6601178	TRIM41	5	180583837	6	0.201703
rs11242777	NULL	6	243915	3	0.110283
rs11242777	NULL	6	243915	8	0.206134
rs1050979	IRF4	6	355417	1	1.185098
rs1050979	IRF4	6	355417	6	0.456137
rs1050979	IRF4	6	355417	7	0.34012
rs1050979	IRF4	6	355417	2	0.123056
rs1877175	IRF4	6	355493	1	1.47679
rs1877176	IRF4	6	355513	1	0.421811
rs1877176	IRF4	6	355513	2	1.185077
rs1877176	IRF4	6	355513	4	1.415287
rs9392502	IRF4	6	355608	4	0.322665
rs9392502	IRF4	6	355608	3	0.366232
rs2316515	IRF4	6	355848	1	0.128737
rs2316515	IRF4	6	355848	7	1.37566
rs2316515	IRF4	6	355848	2	0.767211
rs2316515	IRF4	6	355848	3	0.695861
rs872071	IRF4	6	356064	1	0.078454
rs872071	IRF4	6	356064	2	0.699892
rs872071	IRF4	6	356064	6	0.398373
rs11242982	LOC642335	6	482157	2	1.511404
rs11242982	LOC642335	6	482157	5	0.108764
rs11242982	LOC642335	6	482157	1	0.831224
rs11242982	LOC642335	6	482157	3	0.051452
rs9328435	LOC646773	6	766327	1	1.175892
rs17816516	NULL	6	1049875	2	0.788661
rs17816516	NULL	6	1049875	6	2.25611
rs2293783	FOXF2	6	1340042	3	0.593566
rs7761242	NULL	6	1459366	1	3.061317
rs7761242	NULL	6	1459366	8	0.041035
rs1545849	C6orf195	6	2567910	3	0.52357
rs9501863	C6orf195	6	2568422	6	0.397066
rs9501863	C6orf195	6	2568422	1	4.307909
rs9501863	C6orf195	6	2568422	7	0.324107
rs6902511	C6orf195	6	2568819	1	0.656122
rs9503233	C6orf195	6	2568929	1	2.348431
rs9503233	C6orf195	6	2568929	7	1.131521
rs17135340	C6orf195	6	2569052	3	0.14098
rs17135340	C6orf195	6	2569052	8	0.158909
rs34857522	RP11-145H9.1	6	2610937	3	0.667455
rs34857522	RP11-145H9.1	6	2610937	2	2.915474

rs34857522	RP11-145H9.1	6	2610937	8	0.29338
rs17135412	RP11-145H9.1	6	2611427	3	1.398094
rs12203065	RP11-145H9.1	6	2611477	1	1.494008
rs12203132	RP11-145H9.1	6	2611548	1	2.237475
rs12203132	RP11-145H9.1	6	2611548	4	0.946438
rs12203132	RP11-145H9.1	6	2611548	8	0.691619
rs9503245	RP11-145H9.1	6	2611847	1	1.194858
rs9503245	RP11-145H9.1	6	2611847	4	0.750847
rs733985	RP11-145H9.1	6	2611931	5	1.29145
rs733985	RP11-145H9.1	6	2611931	3	0.950857
rs733985	RP11-145H9.1	6	2611931	1	0.770702
rs6904200	RP11-145H9.1	6	2612030	5	1.359179
rs6904200	RP11-145H9.1	6	2612030	4	0.580011
rs6904200	RP11-145H9.1	6	2612030	8	0.472134
rs9501868	RP11-145H9.1	6	2612348	8	2.946556
rs9501868	RP11-145H9.1	6	2612348	4	0.731335
rs9501868	RP11-145H9.1	6	2612348	1	2.915713
rs9501869	RP11-145H9.1	6	2612369	4	1.26523
rs9501869	RP11-145H9.1	6	2612369	1	3.621224
rs9501872	RP11-145H9.1	6	2612499	1	1.583613
rs9501872	RP11-145H9.1	6	2612499	8	0.440545
rs9501872	RP11-145H9.1	6	2612499	6	0.549554
rs9501873	RP11-145H9.1	6	2612688	1	2.370377
rs9501873	RP11-145H9.1	6	2612688	8	0.46034
rs9501873	RP11-145H9.1	6	2612688	6	1.148042
rs12206682	RP11-145H9.1	6	2613070	4	0.198865
rs12206682	RP11-145H9.1	6	2613070	6	0.206516
rs12206682	RP11-145H9.1	6	2613070	1	2.571712
rs2296356	RP11-145H9.1	6	2694380	3	0.127074
rs192907	SERPINB9	6	2832770	4	0.894528
rs192907	SERPINB9	6	2832770	2	3.252721
rs1052886	SERPINB9	6	2835189	6	0.341379
rs1052886	SERPINB9	6	2835189	4	0.434473
rs1052886	SERPINB9	6	2835189	8	2.209997
rs318486	SERPINB9	6	2835282	7	0.593114
rs318486	SERPINB9	6	2835282	4	0.551611
rs2326106	DKFZp686I15217	6	2934581	1	0.690566
rs4149367	NQO2	6	2960864	3	0.367162
rs6955	NQO2	6	2964926	2	6.035736
rs6955	NQO2	6	2964926	3	0.022835
rs6955	NQO2	6	2964926	1	0.347866
rs2272990	RIPK1	6	3022140	3	0.653712
rs41300839	TUBB2A	6	3100946	3	0.320807
rs1127485	C6orf85	6	3215005	7	6.012247
rs3813485	C6orf85	6	3215120	7	5.857213
rs3211066	C6orf85	6	3216787	7	5.740884
rs9501975	C6orf85	6	3217806	7	3.165792

rs742541	C6orf145	6	3668331	1	3.138644
rs226959	C6orf145	6	3672810	1	5.238558
rs6597007	FAM50B	6	3796192	4	5.835564
rs3196512	FAM50B	6	3796544	5	0.361177
rs3196512	FAM50B	6	3796544	4	0.795663
rs3196512	FAM50B	6	3796544	3	0.727153
rs9503893	PRPF4B	6	3976997	7	3.255358
rs9503893	PRPF4B	6	3976997	1	5.218751
rs9503893	PRPF4B	6	3976997	6	4.944998
rs9503893	PRPF4B	6	3976997	4	0.700556
rs10485172	C6orf146	6	4014132	4	0.557083
rs595413	C6orf146	6	4014165	6	1.234373
rs595413	C6orf146	6	4014165	4	1.154573
rs639905	C6orf146	6	4014684	4	1.805611
rs11751668	C6orf201	6	4070473	7	0.329074
rs659305	C6orf201	6	4070654	8	3.641359
rs3177253	PECI	6	4078854	7	0.038126
rs9405753	KU-MEL-3	6	4556828	1	3.151774
rs9405753	KU-MEL-3	6	4556828	6	3.147508
rs9405753	KU-MEL-3	6	4556828	3	2.537663
rs3812178	CDYL	6	4661036	7	0.760715
rs1749144	RPP40	6	4943962	3	0.367443
rs393628	LYRM4	6	5054118	5	0.655844
rs393628	LYRM4	6	5054118	1	4.852686
rs1050783	F13A1	6	6090691	1	5.504832
rs5988	F13A1	6	6097136	7	1.476471
rs5988	F13A1	6	6097136	1	3.604644
rs5986	F13A1	6	6119855	4	0.568607
rs5982	F13A1	6	6119865	2	4.623629
rs5982	F13A1	6	6119865	3	3.796714
rs1334576	RREB1	6	7156817	6	4.302727
SNP6-7175679	RREB1	6	7175679	5	0.155113
SNP6-7175679	RREB1	6	7175679	3	0.031688
SNP6-7192343	RREB1	6	7192343	3	0.92399
SNP6-7192343	RREB1	6	7192343	6	0.720703
rs6597266	RIOK1	6	7335076	8	0.892065
rs6597266	RIOK1	6	7335076	3	0.857062
rs6597266	RIOK1	6	7335076	6	6.192789
rs6597266	RIOK1	6	7335076	5	6.086851
rs6597269	LOC644051	6	7462238	4	4.571859
rs6597269	LOC644051	6	7462238	1	3.446314
rs17604693	DSP	6	7510726	1	4.971502
rs2064217	DSP	6	7522259	1	4.030289
rs2744380	DSP	6	7530966	8	0.148691
rs2744380	DSP	6	7530966	1	4.199717
rs2744380	DSP	6	7530966	4	0.925354
rs2744380	DSP	6	7530966	6	0.152727

rs3734586	C6orf151	6	7546719	8	0.117028
rs3734586	C6orf151	6	7546719	1	5.763829
rs2806218	C6orf151	6	7555485	6	1.8351
rs2806218	C6orf151	6	7555485	2	3.067796
rs2806218	C6orf151	6	7555485	3	0.83934
rs2806218	C6orf151	6	7555485	5	2.355618
rs17558	BMP6	6	7807588	2	1.664917
rs8643	TXNDC5	6	7828072	3	0.56949
rs8643	TXNDC5	6	7828072	8	8.14322
rs16870746	GCM2	6	10990056	5	0.130176
rs4713039	SYCP2L	6	11014242	6	1.646959
rs4713039	SYCP2L	6	11014242	8	1.342435
rs4713039	SYCP2L	6	11014242	4	0.146874
rs4713039	SYCP2L	6	11014242	3	0.489383
rs3798751	SYCP2L	6	11050950	3	0.346185
rs3798751	SYCP2L	6	11050950	4	0.7926
rs3734397	ELOVL2	6	11090834	1	2.49807
rs1099	HERV-FRD	6	11210857	8	1.184744
rs1099	HERV-FRD	6	11210857	6	2.121943
rs1099	HERV-FRD	6	11210857	5	0.447477
rs13214666	HERV-FRD	6	11211523	5	0.736763
rs13214666	HERV-FRD	6	11211523	3	0.7824
rs13214666	HERV-FRD	6	11211523	7	0.560508
rs1044411	NEDD9	6	11291889	1	5.936482
rs1044411	NEDD9	6	11291889	8	0.198142
rs1044411	NEDD9	6	11291889	3	0.144686
rs1044411	NEDD9	6	11291889	6	5.212692
rs1044411	NEDD9	6	11291889	5	0.418778
rs10601	NEDD9	6	11293243	4	4.855898
rs10601	NEDD9	6	11293243	7	5.682811
rs16871074	NEDD9	6	11301846	1	5.806784
rs16871074	NEDD9	6	11301846	8	7.465707
rs2034	NEDD9	6	11308842	3	0.449051
rs2034	NEDD9	6	11308842	6	4.224262
rs2034	NEDD9	6	11308842	1	4.303713
rs3798734	NEDD9	6	11309143	6	3.17129
rs3798734	NEDD9	6	11309143	3	0.549435
rs3798734	NEDD9	6	11309143	1	5.048466
SNP6-12232573	HIVEP1	6	12232573	5	0.157656
SNP6-12232573	HIVEP1	6	12232573	7	4.659471
SNP6-12232573	HIVEP1	6	12232573	2	0.101609
SNP6-12232573	HIVEP1	6	12232573	1	0.185631
SNP6-13720057	SIRT5	6	13720057	5	7.825715
rs280162	RNF182	6	14086254	6	0.600615
rs280162	RNF182	6	14086254	5	0.905554
rs280162	RNF182	6	14086254	7	1.499007
rs280162	RNF182	6	14086254	1	0.315843

rs280163	RNF182	6	14086597	6	0.316652
rs280163	RNF182	6	14086597	1	1.420698
rs2147200	RNF182	6	14086833	6	0.848444
rs2147200	RNF182	6	14086833	7	1.108769
rs2147200	RNF182	6	14086833	5	1.467576
rs2147200	RNF182	6	14086833	1	0.826073
rs2147201	RNF182	6	14087505	1	1.000019
rs2147201	RNF182	6	14087505	6	0.492249
rs2235258	JARID2	6	15621461	3	0.128791
rs17470454	DTNBP1	6	15631427	1	5.496998
rs2294264	LOC644906	6	16216410	4	0.736654
rs2294264	LOC644906	6	16216410	5	0.184364
rs2294264	LOC644906	6	16216410	3	0.55027
rs2294264	LOC644906	6	16216410	1	0.044709
rs1042391	GMPR	6	16398740	6	7.085412
rs1042391	GMPR	6	16398740	1	5.366185
rs6723	GMPR	6	16403568	1	5.630485
rs6723	GMPR	6	16403568	6	1.319053
rs111168	ATXN1	6	16414338	7	0.057957
rs111168	ATXN1	6	16414338	5	0.627167
rs111168	ATXN1	6	16414338	6	7.933522
rs10807614	CAP2	6	17651281	4	0.788141
rs10807614	CAP2	6	17651281	6	1.110714
rs10807614	CAP2	6	17651281	7	1.336741
rs10807614	CAP2	6	17651281	5	1.117032
rs10807614	CAP2	6	17651281	8	3.69502
rs6919110	FAM8A1	6	17708598	1	0.795645
rs942376	KIF13A	6	17889429	4	6.537
rs3734234	KIF13A	6	17902473	7	1.75716
rs3734234	KIF13A	6	17902473	4	1.834431
rs3734234	KIF13A	6	17902473	1	3.82467
rs3734234	KIF13A	6	17902473	3	1.015854
rs2842934	TPMT	6	18247193	7	0.726444
rs1800460	TPMT	6	18247207	1	5.66505
rs1800460	TPMT	6	18247207	5	4.779786
rs4716224	AOF1	6	18269542	2	3.58459
rs4716224	AOF1	6	18269542	3	0.549051
rs6903583	AOF1	6	18321967	1	1.226738
rs6903583	AOF1	6	18321967	2	0.058076
rs6903583	AOF1	6	18321967	3	1.014647
rs6903583	AOF1	6	18321967	5	0.768122
rs2296198	RNF144B	6	18507729	5	1.369747
rs9396868	RNF144B	6	18575501	2	3.74665
rs9396868	RNF144B	6	18575501	7	1.87911
rs1936905	KRT18P38	6	19721057	5	0.304953
rs1936906	KRT18P38	6	19721067	6	2.821835
rs1936906	KRT18P38	6	19721067	3	1.372342

rs6915322	LOC729177	6	22244139	1	4.071888
rs9466269	LOC729177	6	22244388	1	3.869571
rs9466269	LOC729177	6	22244388	8	1.412541
rs3734215	HDGFL1	6	22679302	5	2.612597
rs3734215	HDGFL1	6	22679302	3	2.134014
rs6937665	DCDC2	6	24280905	7	0.770181
rs6937665	DCDC2	6	24280905	1	5.354454
rs6456593	DCDC2	6	24282308	7	0.464587
rs6456594	DCDC2	6	24282312	1	0.244925
rs6456594	DCDC2	6	24282312	2	2.450996
rs6456594	DCDC2	6	24282312	7	1.097199
rs9358755	DCDC2	6	24282934	6	0.124392
rs2274305	DCDC2	6	24399182	1	3.241924
rs2274305	DCDC2	6	24399182	7	0.433509
rs3761788	MRS2L	6	24523359	5	0.565056
rs1042303	GPLD1	6	24545437	5	0.528168
rs1042303	GPLD1	6	24545437	4	0.269947
rs1042303	GPLD1	6	24545437	8	0.774012
rs1042303	GPLD1	6	24545437	2	1.724869
rs9358767	GPLD1	6	24553808	4	1.674556
rs6924628	GPLD1	6	24562371	7	0.633387
rs9461026	GPLD1	6	24581763	7	0.393628
rs1126617	GPLD1	6	24597631	7	3.855182
rs1126617	GPLD1	6	24597631	5	0.455865
rs1126617	GPLD1	6	24597631	8	2.128569
rs2760183	KIAA0319	6	24652880	7	0.881289
rs699463	KIAA0319	6	24652882	2	4.849787
rs699463	KIAA0319	6	24652882	8	1.43138
rs699463	KIAA0319	6	24652882	5	1.318388
rs699463	KIAA0319	6	24652882	4	0.882163
rs12196913	KIAA0319	6	24653286	1	0.357951
rs12196913	KIAA0319	6	24653286	2	2.161783
rs12196913	KIAA0319	6	24653286	4	0.466812
rs4504469	KIAA0319	6	24696863	5	1.176635
rs4504469	KIAA0319	6	24696863	1	1.374676
rs2038135	KIAA0319	6	24753946	1	1.853813
rs2294689	TTRAP	6	24761252	7	6.375756
rs1129644	TTRAP	6	24761355	5	1.159175
rs1129644	TTRAP	6	24761355	1	4.308723
rs17243157	THEM2	6	24775302	3	1.373869
rs17243157	THEM2	6	24775302	4	3.822059
rs2754775	GMNN	6	24883160	4	0.315447
rs2754775	GMNN	6	24883160	3	0.928493
rs2754775	GMNN	6	24883160	5	1.352323
rs303006	CMAH	6	25192697	3	0.302728
rs303006	CMAH	6	25192697	5	0.673027
rs11794	SCGN	6	25809697	4	1.32689

rs11794	SCGN	6	25809697	1	3.04459
rs11794	SCGN	6	25809697	8	0.733488
rs10105	SCGN	6	25809882	8	0.803075
rs17320558	HIST1H2BA	6	25835506	1	0.42184
rs1141034	SLC17A4	6	25888311	1	2.551887
rs1141034	SLC17A4	6	25888311	8	1.956486
SNP6-25958824	SLC17A3	6	25958824	4	4.281909
SNP6-25958824	SLC17A3	6	25958824	1	5.036927
SNP6-26022832	SLC17A2	6	26022832	7	2.128048
rs3734528	HIST1H4A	6	26130223	2	3.130109
rs3734528	HIST1H4A	6	26130223	7	0.10267
rs3734528	HIST1H4A	6	26130223	8	0.129894
rs3752419	HIST1H4B	6	26135412	8	0.836119
rs3752419	HIST1H4B	6	26135412	4	1.044483
rs2213284	HIST1H3B	6	26139847	5	0.62034
rs2213284	HIST1H3B	6	26139847	4	1.888013
rs198845	HIST1H1T	6	26215769	5	0.160378
rs198845	HIST1H1T	6	26215769	6	3.571773
rs198845	HIST1H1T	6	26215769	4	0.235726
rs198844	HIST1H1T	6	26216261	4	0.072123
rs198844	HIST1H1T	6	26216261	1	2.907547
rs198844	HIST1H1T	6	26216261	6	0.200848
rs198844	HIST1H1T	6	26216261	5	0.546164
rs34550794	HIST1H2AD	6	26306993	6	1.321569
rs41266803	HIST1H3D	6	26307433	6	6.762127
rs41266803	HIST1H3D	6	26307433	7	4.521378
rs16891458	HIST1H1D	6	26342455	6	0.598525
rs16891458	HIST1H1D	6	26342455	7	0.195812
rs2223591	HIST1H3G	6	26379220	3	1.222746
rs2393593	HIST1H4H	6	26393662	2	2.873013
rs2393593	HIST1H4H	6	26393662	4	3.440793
SNP6-26478727	BTN3A2	6	26478727	2	8.146895
SNP6-26478727	BTN3A2	6	26478727	4	0.499811
rs1979	BTN3A2	6	26485570	7	1.734495
SNP6-26496384	BTN2A2	6	26496384	7	0.682002
SNP6-26496384	BTN2A2	6	26496384	1	2.957774
rs3927423	BTN2A2	6	26501466	8	0.125007
rs4712990	BTN3A1	6	26521723	4	0.551347
rs4712990	BTN3A1	6	26521723	2	3.49365
rs3198487	BTN3A1	6	26522352	2	5.093658
rs3198487	BTN3A1	6	26522352	4	0.384677
rs10946829	BTN2A3	6	26534466	4	0.242162
rs10946829	BTN2A3	6	26534466	2	2.029999
SNP6-26534499	BTN2A3	6	26534499	2	2.130396
rs2893848	BTN2A3	6	26538960	4	3.7646
rs12207181	BTN2A3	6	26539264	8	1.00388
SNP6-26539319	BTN2A3	6	26539319	4	1.313714

SNP6-26539319	BTN2A3	6	26539319	2	4.487271
rs17611438	BTN3A3	6	26551832	4	2.335589
rs3846848	BTN3A3	6	26554188	4	0.305975
rs3846848	BTN3A3	6	26554188	2	3.887625
rs6907302	BTN2A1	6	26566205	4	0.441687
rs6907302	BTN2A1	6	26566205	2	3.522241
rs3736782	BTN1A1	6	26613382	1	2.181877
rs9393728	BTN1A1	6	26617309	1	0.89499
rs1056668	BTN1A1	6	26618584	1	0.98728
rs12204145	ABT1	6	26708135	8	6.081125
rs3800314	LOC442172	6	27344027	1	1.123277
rs2235233	POM121L2	6	27387831	5	1.379432
rs2235233	POM121L2	6	27387831	3	0.911248
rs2235233	POM121L2	6	27387831	1	1.062151
rs17739298	FKSG83	6	27401028	6	0.842324
rs17739298	FKSG83	6	27401028	2	1.174442
rs9468076	ZNF204	6	27446869	8	2.139641
rs9468076	ZNF204	6	27446869	4	0.12
rs10807020	NULL	6	27464881	7	0.715764
rs10807020	NULL	6	27464881	4	1.421901
rs10807020	NULL	6	27464881	5	1.101203
rs10807020	NULL	6	27464881	1	3.900223
rs10807021	NULL	6	27464904	4	2.31254
rs10807021	NULL	6	27464904	1	4.859815
rs10807021	NULL	6	27464904	5	0.95233
rs6904883	NULL	6	27465287	1	3.288916
rs6904883	NULL	6	27465287	7	0.215652
rs6904883	NULL	6	27465287	5	0.00079
rs6904883	NULL	6	27465287	4	0.222301
rs2206251	NULL	6	27466371	5	0.25854
rs2206251	NULL	6	27466371	1	5.620316
rs2206251	NULL	6	27466371	7	0.201654
rs2206251	NULL	6	27466371	4	0.460694
rs2206252	NULL	6	27466441	1	2.384357
rs2206252	NULL	6	27466441	4	0.695384
rs2206252	NULL	6	27466441	5	0.642753
rs2206252	NULL	6	27466441	7	0.415614
rs766093	ZNF391	6	27476102	2	1.905509
rs2076305	MCFD2L	6	27483590	5	5.042421
rs2076305	MCFD2L	6	27483590	1	5.846086
rs2076305	MCFD2L	6	27483590	4	4.887085
rs1883216	ZNF184	6	27533164	7	0.693051
rs1883216	ZNF184	6	27533164	1	3.706009
rs1883216	ZNF184	6	27533164	5	0.794244
rs7767176	OR2B6	6	28033346	7	0.378402
rs904142	OR2B6	6	28033435	1	3.19438
rs9380030	OR2B6	6	28033806	1	3.501381

rs203878	ZNF165	6	28156975	6	0.138798
rs203878	ZNF165	6	28156975	2	3.115583
rs203878	ZNF165	6	28156975	7	0.8284
rs9380068	LOC222699	6	28291221	1	5.163551
rs1150702	LOC222699	6	28292076	2	6.444673
rs1150703	LOC222699	6	28292239	2	1.996308
rs1736894	LOC222699	6	28294533	2	1.484142
rs1736894	LOC222699	6	28294533	7	0.399854
rs1736894	LOC222699	6	28294533	8	3.694741
rs1736894	LOC222699	6	28294533	6	0.790705
rs1233712	ZNF193	6	28301110	2	2.69376
rs1736895	ZKSCAN4	6	28327805	6	0.152083
rs1736895	ZKSCAN4	6	28327805	8	2.78986
rs1736895	ZKSCAN4	6	28327805	2	2.511155
rs1635	NKAPL	6	28335583	1	5.184335
rs9461446	NKAPL	6	28335612	1	5.636735
rs1679709	NKAPL	6	28336321	1	0.890759
rs1679709	NKAPL	6	28336321	2	1.273659
rs11965538	ZNF187	6	28347894	1	0.514961
rs11965538	ZNF187	6	28347894	2	6.172465
rs3800324	PGBD1	6	28372660	1	1.267028
rs3800325	PGBD1	6	28372671	6	2.974803
rs6456811	PGBD1	6	28378026	6	1.972417
rs9461449	PGBD1	6	28378043	7	1.404525
rs9461449	PGBD1	6	28378043	8	6.896793
rs9461449	PGBD1	6	28378043	1	5.458472
rs1062169	PGBD1	6	28378230	6	0.143173
SNP6-28439073	ZKSCAN3	6	28439073	1	1.927357
SNP6-28439106	ZKSCAN3	6	28439106	8	7.980515
SNP6-28439106	ZKSCAN3	6	28439106	6	0.188363
SNP6-28439106	ZKSCAN3	6	28439106	1	1.08049
SNP6-28439106	ZKSCAN3	6	28439106	7	0.478885
rs7775132	ZSCAN12	6	28454992	8	0.539693
rs7775132	ZSCAN12	6	28454992	6	0.474157
rs1052215	ZSCAN12	6	28456137	7	2.349249
rs1052215	ZSCAN12	6	28456137	1	0.59856
rs1052215	ZSCAN12	6	28456137	6	0.249595
rs1052215	ZSCAN12	6	28456137	2	1.230607
rs450630	ZNF452	6	28650403	8	0.007644
rs450630	ZNF452	6	28650403	6	4.25852
rs370520	ZNF452	6	28650499	2	1.652121
rs370520	ZNF452	6	28650499	6	4.88741
rs370520	ZNF452	6	28650499	7	2.493441
rs370520	ZNF452	6	28650499	8	5.246134
rs17336532	ZNF452	6	28651243	1	5.037713
rs17336532	ZNF452	6	28651243	6	4.268799
rs9368568	LOC646160	6	28709280	7	1.06803

rs1233627	NOL5BP	6	28859706	2	2.464577
rs1233627	NOL5BP	6	28859706	1	3.057874
rs2071790	C6orf100	6	29019781	1	0.504997
rs3749977	OR2J3	6	29188323	1	1.365969
rs6930033	OR5V1	6	29431884	2	2.419909
rs2074470	OR11A1	6	29502406	1	2.142663
rs2074470	OR11A1	6	29502406	2	1.963177
rs9257857	OR11A1	6	29502905	6	1.390683
rs2074465	OR10C1	6	29516455	2	0.969423
rs3128854	OR2H1	6	29539677	1	0.609143
rs2076486	UBD	6	29631851	3	1.168044
rs2076485	UBD	6	29631931	3	0.385792
rs2076485	UBD	6	29631931	1	3.2888
rs2076485	UBD	6	29631931	7	2.560736
rs2076484	UBD	6	29631982	3	0.597058
rs2235698	OR2H2	6	29663757	7	0.429597
rs2235698	OR2H2	6	29663757	1	5.470414
rs2235698	OR2H2	6	29663757	3	0.954311
rs29230	GABBR1	6	29684372	1	3.587337
rs29230	GABBR1	6	29684372	6	1.420168
rs29230	GABBR1	6	29684372	7	2.270316
rs9468571	MOG	6	29732873	4	2.932825
rs2857766	MOG	6	29741982	6	2.140673
rs2857766	MOG	6	29741982	5	0.291418
rs2535238	ZFP57	6	29753017	6	4.387414
rs2535238	ZFP57	6	29753017	4	3.937675
rs2535238	ZFP57	6	29753017	7	3.046287
rs2747421	ZFP57	6	29753097	4	2.525563
rs2747421	ZFP57	6	29753097	7	9.103782
rs2394160	RP3-377H14.5	6	29811241	6	3.763876
rs2394160	RP3-377H14.5	6	29811241	3	0.445715
rs2394160	RP3-377H14.5	6	29811241	4	0.846798
rs2517945	RP3-377H14.5	6	29811412	3	0.11414
rs2517945	RP3-377H14.5	6	29811412	6	0.810135
rs2517945	RP3-377H14.5	6	29811412	8	0.166063
rs2517945	RP3-377H14.5	6	29811412	4	0.52489
rs2523389	RP3-377H14.5	6	29814107	6	2.312408
rs2523389	RP3-377H14.5	6	29814107	3	0.193689
rs2523389	RP3-377H14.5	6	29814107	4	0.376655
rs2523389	RP3-377H14.5	6	29814107	8	0.521769
rs1610621	IFITM4P	6	29826769	7	1.955071
rs1610621	IFITM4P	6	29826769	3	1.88088
rs1610621	IFITM4P	6	29826769	6	3.129762
rs1610621	IFITM4P	6	29826769	4	0.346462
rs17185111	HCG4	6	29866989	2	5.087211
rs3734818	HCG4	6	29867201	4	0.782234
rs3734818	HCG4	6	29867201	3	1.252863

rs1610643	HCG4	6	29867289	3	0.474598
rs1610643	HCG4	6	29867289	1	3.366064
rs1610643	HCG4	6	29867289	7	0.904116
rs1610643	HCG4	6	29867289	5	0.569291
rs1610643	HCG4	6	29867289	8	1.989175
rs17185132	HCG4	6	29867490	3	0.498262
rs17185132	HCG4	6	29867490	4	9.03394
rs7764777	HCG4	6	29868128	8	2.645312
rs7764777	HCG4	6	29868128	1	3.789713
rs7764777	HCG4	6	29868128	5	0.956797
rs1611212	HCG4	6	29868502	3	0.808998
rs1611212	HCG4	6	29868502	5	0.424548
rs1611212	HCG4	6	29868502	1	3.26036
rs1611212	HCG4	6	29868502	6	0.477562
rs1611213	HCG4	6	29868569	6	3.244535
rs1611213	HCG4	6	29868569	5	1.034736
rs1611213	HCG4	6	29868569	4	1.494148
rs1611213	HCG4	6	29868569	1	3.73761
rs1611213	HCG4	6	29868569	8	1.929325
rs1611213	HCG4	6	29868569	7	1.698729
rs1610719	HCG4	6	29868656	4	0.906536
rs1610719	HCG4	6	29868656	1	2.757996
rs1610719	HCG4	6	29868656	7	0.492868
rs1610719	HCG4	6	29868656	8	0.26867
rs1610719	HCG4	6	29868656	5	1.278861
rs1610719	HCG4	6	29868656	6	0.390105
rs1130363	HLA-G	6	29905675	1	3.287982
rs1130363	HLA-G	6	29905675	2	2.71118
rs1130363	HLA-G	6	29905675	6	1.454004
rs1130363	HLA-G	6	29905675	7	3.086336
rs1130363	HLA-G	6	29905675	5	2.084404
rs915669	HLA-G	6	29906404	1	2.017041
rs915669	HLA-G	6	29906404	6	4.345979
rs915669	HLA-G	6	29906404	5	4.731154
rs915669	HLA-G	6	29906404	2	2.161993
rs915669	HLA-G	6	29906404	7	0.87665
rs1610696	HLA-G	6	29906782	7	0.218002
rs1610696	HLA-G	6	29906782	6	10.28397
rs1610696	HLA-G	6	29906782	5	0.890006
rs1610696	HLA-G	6	29906782	3	2.567882
rs1610696	HLA-G	6	29906782	8	6.626835
rs1610696	HLA-G	6	29906782	1	5.170949
rs7768484	HLA-H	6	29963957	8	5.237933
rs7768484	HLA-H	6	29963957	5	2.925223
rs3094185	HCG2P7	6	29975374	1	5.466797
rs3094185	HCG2P7	6	29975374	7	2.731052
rs2231118	HLA-A	6	30020831	3	0.568905

rs2231118	HLA-A	6	30020831	6	0.297283
rs2231118	HLA-A	6	30020831	1	3.382738
rs2523943	HCG9	6	30050906	3	0.336821
rs2523943	HCG9	6	30050906	4	0.601192
rs422640	HCG9	6	30050984	3	0.252287
rs6904029	HCG9	6	30051046	3	0.107305
rs6904029	HCG9	6	30051046	5	0.06086
rs6903753	HCG9	6	30051157	6	0.838853
rs6903753	HCG9	6	30051157	5	1.367267
rs6903753	HCG9	6	30051157	3	2.50346
rs2071568	HCG9	6	30051260	5	0.901872
rs2071568	HCG9	6	30051260	6	0.190032
rs2071568	HCG9	6	30051260	3	1.207249
rs7770557	ZNRD1	6	30137088	8	4.692625
rs7770557	ZNRD1	6	30137088	2	4.118418
rs7770557	ZNRD1	6	30137088	3	0.187667
rs1150738	PPP1R11	6	30143068	4	0.677241
rs1150738	PPP1R11	6	30143068	3	0.229981
rs2074482	PPP1R11	6	30144450	4	0.172369
rs2074482	PPP1R11	6	30144450	5	0.328863
rs1245371	RNF39	6	30146331	8	1.456927
rs2301753	RNF39	6	30147219	4	3.878175
rs2301753	RNF39	6	30147219	8	0.942215
rs2301753	RNF39	6	30147219	5	5.902641
rs2301753	RNF39	6	30147219	2	3.769447
rs1116221	TRIM31	6	30179309	7	1.890847
rs1116221	TRIM31	6	30179309	6	1.358674
rs1116221	TRIM31	6	30179309	4	0.653017
rs3132679	TRIM31	6	30183822	1	5.441594
rs3132679	TRIM31	6	30183822	3	0.799692
rs3132679	TRIM31	6	30183822	7	0.375956
rs2023472	TRIM31	6	30183843	7	0.313616
rs2023472	TRIM31	6	30183843	4	0.896011
rs2523989	TRIM31	6	30186254	4	1.013901
rs2523989	TRIM31	6	30186254	3	2.11398
rs2239529	TRIM31	6	30186309	4	0.310277
rs2239529	TRIM31	6	30186309	3	1.279643
rs3734838	TRIM31	6	30188210	5	1.028557
rs3734838	TRIM31	6	30188210	7	2.032822
rs2517598	TRIM31	6	30188253	3	2.173816
rs2517598	TRIM31	6	30188253	4	0.3796
rs2844793	TRIM31	6	30188475	3	1.579988
rs2844793	TRIM31	6	30188475	4	0.500933
rs757262	TRIM40	6	30222934	5	0.387713
rs9261530	TRIM10	6	30228143	4	0.081598
rs2857433	TRIM10	6	30228612	5	0.016503
rs2857433	TRIM10	6	30228612	4	0.219103

rs2857433	TRIM10	6	30228612	6	0.761228
rs2285795	TRIM10	6	30228891	4	1.930476
rs2285795	TRIM10	6	30228891	5	2.757487
rs9261532	TRIM10	6	30228924	5	1.075576
rs9261532	TRIM10	6	30228924	6	3.048885
rs3734840	TRIM10	6	30229584	8	1.04512
rs2523733	TRIM15	6	30239494	3	0.306485
rs2523733	TRIM15	6	30239494	7	1.365302
rs929156	TRIM15	6	30247678	4	1.399091
rs929156	TRIM15	6	30247678	6	0.45156
rs929156	TRIM15	6	30247678	5	0.56657
rs1063280	TRIM15	6	30248321	3	0.031024
rs1063280	TRIM15	6	30248321	1	1.576773
rs2523721	TRIM26	6	30274245	3	0.052247
rs2072107	TRIM26	6	30274914	5	1.231697
rs2072107	TRIM26	6	30274914	2	4.934049
rs3094615	TRIM26	6	30289096	4	0.503323
rs3094615	TRIM26	6	30289096	6	1.330875
rs3094615	TRIM26	6	30289096	8	1.964847
rs3094615	TRIM26	6	30289096	3	0.172647
rs3094615	TRIM26	6	30289096	5	0.233539
rs9391806	FLJ45422	6	30335376	1	0.622065
rs9391806	FLJ45422	6	30335376	3	0.143062
rs2023477	FLJ45422	6	30337442	1	3.510652
rs2023477	FLJ45422	6	30337442	4	0.25726
rs2023477	FLJ45422	6	30337442	6	0.770656
rs2023477	FLJ45422	6	30337442	3	0.976213
rs2523746	FLJ45422	6	30338064	6	1.114616
rs2523746	FLJ45422	6	30338064	3	0.616186
rs2523746	FLJ45422	6	30338064	1	4.438439
rs2523746	FLJ45422	6	30338064	4	0.601737
rs2523746	FLJ45422	6	30338064	8	0.07381
rs2516700	FLJ45422	6	30339050	4	1.860872
rs2516700	FLJ45422	6	30339050	8	5.63213
rs2516700	FLJ45422	6	30339050	3	0.80445
rs2516700	FLJ45422	6	30339050	6	1.803058
rs2516700	FLJ45422	6	30339050	1	5.206606
rs2516697	FLJ45422	6	30340493	4	1.060964
rs2516697	FLJ45422	6	30340493	1	5.355453
rs2516697	FLJ45422	6	30340493	6	0.500072
rs2516697	FLJ45422	6	30340493	8	1.087562
rs2516697	FLJ45422	6	30340493	3	1.268111
rs2844765	FLJ45422	6	30342473	8	0.649134
rs2844765	FLJ45422	6	30342473	6	0.299718
rs2844765	FLJ45422	6	30342473	1	4.544555
rs2844765	FLJ45422	6	30342473	4	0.760226
rs2844765	FLJ45422	6	30342473	3	0.67213

rs2844764	FLJ45422	6	30342647	4	0.742889
rs2844764	FLJ45422	6	30342647	8	0.338159
rs2844764	FLJ45422	6	30342647	6	0.05115
rs2844764	FLJ45422	6	30342647	3	0.441704
rs2844764	FLJ45422	6	30342647	1	4.900828
rs5030609	TRIM39	6	30404660	3	0.89169
rs5030609	TRIM39	6	30404660	4	0.316236
rs5030609	TRIM39	6	30404660	8	3.01705
rs5030609	TRIM39	6	30404660	1	6.040103
rs5030609	TRIM39	6	30404660	6	0.48146
rs2844750	RPP21	6	30421247	5	0.099676
rs2844750	RPP21	6	30421247	4	0.075619
rs2844750	RPP21	6	30421247	6	3.640887
rs6986	RPP21	6	30421319	2	0.498867
rs6986	RPP21	6	30421319	3	0.700412
rs6986	RPP21	6	30421319	5	0.521796
rs2074505	GNL1	6	30629116	2	1.248485
rs3132594	C6orf136	6	30723239	7	3.244956
rs9262152	MDC1	6	30788895	5	4.083941
rs8233	TUBB	6	30800944	7	6.517172
rs3094124	IER3	6	30819784	2	8.128737
rs2229933	DDR1	6	30965051	6	6.033045
rs2229933	DDR1	6	30965051	7	0.289292
rs2229933	DDR1	6	30965051	3	0.452269
rs2229933	DDR1	6	30965051	1	4.009228
rs1264319	DDR1	6	30970402	7	4.175538
rs2230121	GTF2H4	6	30988190	8	7.760701
rs1264305	VARS2	6	30990256	2	3.829815
rs1264305	VARS2	6	30990256	3	0.216624
rs1264305	VARS2	6	30990256	6	2.05926
rs1264305	VARS2	6	30990256	1	0.45855
rs1264303	VARS2	6	30990492	6	2.68724
rs1264303	VARS2	6	30990492	1	1.785733
rs1264303	VARS2	6	30990492	2	2.144776
rs1264303	VARS2	6	30990492	3	2.578167
rs6926224	VARS2	6	30990668	2	5.39205
rs2249459	VARS2	6	30995967	3	0.314858
rs2517468	VARS2	6	30996148	3	0.348159
rs2517468	VARS2	6	30996148	1	0.61731
rs2517468	VARS2	6	30996148	6	2.267545
rs2074511	VARS2	6	30997368	6	5.586611
rs2074511	VARS2	6	30997368	7	6.854531
rs2074511	VARS2	6	30997368	1	3.635663
SNP6-30997382	VARS2	6	30997382	3	0.104269
rs2532942	VARS2	6	31000301	2	1.120979
rs2532942	VARS2	6	31000301	1	0.72112
rs2532942	VARS2	6	31000301	3	0.223657

rs3131787	SFTPG	6	31007503	3	2.117014
rs2286656	SFTPG	6	31007550	1	4.467406
rs2286656	SFTPG	6	31007550	7	0.861366
rs3132580	DPCR1	6	31028103	3	4.147096
rs2240804	DPCR1	6	31028869	6	0.55565
rs2240804	DPCR1	6	31028869	3	1.402124
rs2240804	DPCR1	6	31028869	1	0.808617
rs2240804	DPCR1	6	31028869	2	4.033325
rs2240803	DPCR1	6	31028936	1	4.992154
rs2240803	DPCR1	6	31028936	7	0.118787
rs12190030	DPCR1	6	31029343	2	0.194717
rs12190030	DPCR1	6	31029343	1	0.499473
rs2844697	LOC646570	6	31040288	7	0.376376
rs2517416	C6orf205	6	31063908	1	4.763534
rs2517416	C6orf205	6	31063908	6	2.719758
rs2517416	C6orf205	6	31063908	5	1.789352
rs886403	C6orf205	6	31065597	8	2.167862
rs886403	C6orf205	6	31065597	3	1.960259
rs2517554	LOC729788	6	31101478	4	2.262329
rs2517554	LOC729788	6	31101478	1	1.412442
rs2517554	LOC729788	6	31101478	7	0.721548
rs2517554	LOC729788	6	31101478	6	1.916835
rs2523898	LOC729788	6	31101512	1	1.562685
rs2523898	LOC729788	6	31101512	5	1.484127
rs2523898	LOC729788	6	31101512	8	0.929335
rs2523898	LOC729788	6	31101512	4	1.021504
rs2523898	LOC729788	6	31101512	3	1.314464
rs4713420	LOC729788	6	31101546	5	0.799847
rs12110470	LOC729792	6	31105795	1	0.692742
rs12110470	LOC729792	6	31105795	8	1.224705
rs12110785	LOC729792	6	31105803	1	0.851759
rs12110785	LOC729792	6	31105803	3	0.416858
rs3132555	CDSN	6	31190889	1	4.842176
rs3132555	CDSN	6	31190889	8	5.560504
rs3132555	CDSN	6	31190889	6	1.038507
rs3132555	CDSN	6	31190889	5	7.279891
rs3095298	CDSN	6	31190911	5	3.55465
rs3095298	CDSN	6	31190911	4	0.522419
rs3095298	CDSN	6	31190911	2	4.016227
rs3130982	CDSN	6	31192054	4	8.346515
rs3130982	CDSN	6	31192054	1	1.659041
rs3130982	CDSN	6	31192054	2	6.159658
rs3094215	CDSN	6	31192663	6	3.491166
rs3094215	CDSN	6	31192663	2	0.081793
rs3094215	CDSN	6	31192663	1	0.525516
rs3094215	CDSN	6	31192663	4	3.508181
rs3130983	CDSN	6	31192771	4	7.376431

rs3130983	CDSN	6	31192771	6	7.631348
rs3130983	CDSN	6	31192771	1	0.355958
rs3130983	CDSN	6	31192771	2	0.65546
rs3132552	CDSN	6	31193248	5	2.136399
rs3132552	CDSN	6	31193248	6	2.881702
rs3132552	CDSN	6	31193248	8	4.437911
rs3132552	CDSN	6	31193248	1	5.779765
rs3095318	CDSN	6	31196124	8	1.358589
rs3095318	CDSN	6	31196124	5	0.974522
rs3815087	PSORS1C1	6	31201566	1	4.817866
rs3815087	PSORS1C1	6	31201566	3	0.359228
rs1265099	PSORS1C2	6	31213392	3	0.990782
rs1265099	PSORS1C2	6	31213392	4	1.204301
rs1265099	PSORS1C2	6	31213392	5	0.21738
rs1265099	PSORS1C2	6	31213392	1	3.068477
rs2074478	PSORS1C2	6	31213612	3	0.745086
rs2074478	PSORS1C2	6	31213612	7	2.039198
rs2074478	PSORS1C2	6	31213612	6	0.786633
rs2233954	PSORS1C2	6	31213651	7	0.172157
rs9263726	PSORS1C1	6	31214478	5	3.303627
rs9263726	PSORS1C1	6	31214478	8	3.52272
rs9263726	PSORS1C1	6	31214478	7	0.661936
rs9263726	PSORS1C1	6	31214478	4	3.373923
rs9263726	PSORS1C1	6	31214478	1	5.323917
SNP6-31215627	PSORS1C1	6	31215627	5	8.885405
SNP6-31215627	PSORS1C1	6	31215627	4	1.075938
SNP6-31215627	PSORS1C1	6	31215627	1	2.036981
SNP6-31215627	PSORS1C1	6	31215627	8	3.948604
SNP6-31215627	PSORS1C1	6	31215627	7	1.176372
SNP6-31215627	PSORS1C1	6	31215627	2	3.784748
rs1576	CCHCR1	6	31218370	8	0.817787
rs1576	CCHCR1	6	31218370	3	0.163653
rs1576	CCHCR1	6	31218370	7	0.523535
rs1576	CCHCR1	6	31218370	2	5.155782
rs1576	CCHCR1	6	31218370	1	0.159176
rs1576	CCHCR1	6	31218370	5	0.590596
rs1576	CCHCR1	6	31218370	6	1.282572
rs130079	CCHCR1	6	31220716	2	5.039201
rs130079	CCHCR1	6	31220716	7	5.597126
rs130079	CCHCR1	6	31220716	3	0.001434
rs2027937	CCHCR1	6	31224481	3	0.690866
rs2027937	CCHCR1	6	31224481	6	5.187368
rs130066	CCHCR1	6	31230294	7	4.9839
rs130066	CCHCR1	6	31230294	6	5.065628
rs130077	CCHCR1	6	31230309	2	0.539889
rs130077	CCHCR1	6	31230309	8	1.147658
rs130077	CCHCR1	6	31230309	1	0.524933

rs130077	CCHCR1	6	31230309	4	0.145501
rs130077	CCHCR1	6	31230309	5	1.636733
rs130076	CCHCR1	6	31230461	3	0.434242
rs3130453	CCHCR1	6	31232828	6	0.708787
rs3130453	CCHCR1	6	31232828	7	0.763893
rs3130453	CCHCR1	6	31232828	4	0.367917
rs3130453	CCHCR1	6	31232828	2	5.994319
SNP6-31233236	CCHCR1	6	31233236	1	5.647035
SNP6-31233236	CCHCR1	6	31233236	5	0.085989
SNP6-31233236	CCHCR1	6	31233236	6	0.849417
rs12529697	CCHCR1	6	31233386	3	1.55202
rs3130932	POU5F1	6	31241922	8	4.218988
rs3130932	POU5F1	6	31241922	5	5.851244
rs3130932	POU5F1	6	31241922	6	3.643964
rs3130932	POU5F1	6	31241922	1	4.154361
rs9263872	HCG27	6	31278587	7	0.002365
rs9263872	HCG27	6	31278587	5	3.027311
rs9263872	HCG27	6	31278587	4	6.985391
rs9263872	HCG27	6	31278587	1	4.543991
rs9263872	HCG27	6	31278587	3	0.80742
rs9263875	HCG27	6	31278893	5	3.543441
rs9263875	HCG27	6	31278893	8	5.196551
rs9263875	HCG27	6	31278893	3	0.396463
rs1049281	HLA-C	6	31344546	7	0.291486
rs1049281	HLA-C	6	31344546	1	4.171242
SNP6-31346238	HLA-C	6	31346238	8	1.867522
SNP6-31346238	HLA-C	6	31346238	1	2.519702
rs1140546	HLA-B	6	31430867	7	0.375563
rs1140546	HLA-B	6	31430867	2	0.39407
rs9501572	LOC729816	6	31441799	2	0.470713
rs9501572	LOC729816	6	31441799	4	1.838634
rs9501572	LOC729816	6	31441799	8	1.197834
rs9501572	LOC729816	6	31441799	7	1.197798
rs9501572	LOC729816	6	31441799	6	1.289797
rs9266379	LOC729816	6	31442160	5	0.139438
rs9266379	LOC729816	6	31442160	1	1.433602
rs9266379	LOC729816	6	31442160	3	1.630593
rs2596427	LOC729816	6	31442372	3	1.87284
rs2844519	MICA	6	31479409	6	0.744854
rs9380254	MICA	6	31486314	5	0.688702
rs1131896	MICA	6	31487094	7	0.056277
rs1131896	MICA	6	31487094	3	1.106762
rs1131896	MICA	6	31487094	5	0.541131
rs1131896	MICA	6	31487094	1	2.168164
rs1131896	MICA	6	31487094	8	0.274288
SNP6-31540061	HCP5	6	31540061	8	4.965635
rs11757571	HCP5	6	31540766	1	4.087944

rs11757602	HCP5	6	31540781	1	4.17834
rs9267143	3.8-1	6	31547816	4	0.532134
rs9267143	3.8-1	6	31547816	6	0.89937
rs9267146	3.8-1	6	31547873	6	1.466056
rs9267146	3.8-1	6	31547873	4	0.157208
rs9267147	3.8-1	6	31547903	4	3.549259
rs9267147	3.8-1	6	31547903	6	2.592518
rs14597	3.8-1	6	31547993	5	0.703346
rs14597	3.8-1	6	31547993	4	1.039606
rs14597	3.8-1	6	31547993	2	0.7471
rs14597	3.8-1	6	31547993	7	0.436797
rs14597	3.8-1	6	31547993	1	1.01309
rs3131639	MICB	6	31581525	4	2.982091
rs2516480	MCCD1	6	31604770	3	0.171658
rs1129640	BAT1	6	31614603	7	1.071649
rs1129640	BAT1	6	31614603	6	1.419713
rs2239527	BAT1	6	31617758	4	1.520441
rs2239527	BAT1	6	31617758	1	0.062381
rs2239528	BAT1	6	31618084	4	0.86932
rs2239528	BAT1	6	31618084	7	0.656064
rs2071594	ATP6V1G2	6	31620699	1	0.169832
rs2071594	ATP6V1G2	6	31620699	4	0.484667
rs2071593	ATP6V1G2	6	31620778	6	0.177644
rs2071593	ATP6V1G2	6	31620778	4	0.268689
SNP6-31633891	NFKBIL1	6	31633891	6	4.681807
rs2229094	LTA	6	31648535	3	0.650624
rs1041981	LTA	6	31648763	1	0.445051
rs1052248	LST1	6	31664560	3	0.178376
rs1052248	LST1	6	31664560	4	0.007616
rs1052248	LST1	6	31664560	7	0.056578
rs3087617	LST1	6	31664635	4	0.662654
rs3179003	NCR3	6	31664907	1	1.323882
rs34816476	NCR3	6	31665360	4	0.645521
rs2259571	AIF1	6	31691806	3	0.094449
rs11538264	BAT2	6	31711168	2	8.472908
rs11538264	BAT2	6	31711168	7	0.99696
rs35502919	BAT2	6	31712334	4	3.384364
rs2295665	BAT4	6	31740665	6	6.541286
rs2295665	BAT4	6	31740665	5	4.830755
rs2295665	BAT4	6	31740665	1	6.501414
SNP6-31740878	BAT4	6	31740878	2	1.6264
SNP6-31740878	BAT4	6	31740878	7	0.292062
rs11758242	LY6G5B	6	31747824	2	5.967189
rs11758242	LY6G5B	6	31747824	7	0.735455
rs9267532	LY6G5B	6	31747958	3	1.183388
rs9267532	LY6G5B	6	31747958	8	0.65425
rs9267535	LY6G5C	6	31757128	8	8.917852

rs9267535	LY6G5C	6	31757128	3	0.308765
rs11552114	BAT5	6	31768827	2	5.6675
rs11552114	BAT5	6	31768827	7	0.209793
rs9267547	C6orf21	6	31783480	2	1.491389
rs9267547	C6orf21	6	31783480	3	0.209157
rs9267547	C6orf21	6	31783480	8	1.355576
rs2242653	C6orf21	6	31783744	5	1.825227
rs2242653	C6orf21	6	31783744	1	0.706024
rs453098	C6orf25	6	31799636	3	0.933986
rs9267551	DDAH2	6	31805936	5	2.154352
rs28381349	MSH5	6	31817024	7	0.936136
rs28381349	MSH5	6	31817024	2	2.340286
rs1802127	MSH5	6	31837904	7	4.649059
rs1802127	MSH5	6	31837904	2	6.94295
rs2075799	HSPA1L	6	31886508	2	4.14926
rs2075799	HSPA1L	6	31886508	7	0.514144
rs2075799	HSPA1L	6	31886508	3	0.056441
rs733539	C6orf48	6	31915468	1	3.78996
rs13118	NEU1	6	31935265	3	0.238318
rs2242664	SLC44A4	6	31947310	8	0.592555
rs2242664	SLC44A4	6	31947310	1	1.250651
rs2075798	SLC44A4	6	31954720	3	0.317923
rs2075798	SLC44A4	6	31954720	6	1.655587
rs7887	EHMT2	6	31972526	5	0.466189
rs7887	EHMT2	6	31972526	7	0.26301
rs7887	EHMT2	6	31972526	8	3.616627
rs7887	EHMT2	6	31972526	4	0.044376
rs7887	EHMT2	6	31972526	1	2.898771
rs2844455	ZBTB12	6	31977652	1	0.565691
rs2844455	ZBTB12	6	31977652	4	0.432157
rs2844455	ZBTB12	6	31977652	5	0.676948
rs2844455	ZBTB12	6	31977652	8	0.815516
rs9332739	C2	6	32011783	1	3.509265
rs1042663	C2	6	32013109	5	0.032785
rs4151667	CFB	6	32022003	1	3.662046
rs4151669	CFB	6	32023123	1	3.962209
rs4151651	CFB	6	32023593	8	4.053211
rs4151672	CFB	6	32027809	1	3.72648
rs17201466	SKIV2L	6	32034579	3	1.212129
rs17201466	SKIV2L	6	32034579	6	1.751781
rs17201466	SKIV2L	6	32034579	4	1.28546
rs438999	SKIV2L	6	32036285	5	0.82932
rs2734331	SKIV2L	6	32038330	7	0.841112
rs2734331	SKIV2L	6	32038330	2	2.090608
rs410851	SKIV2L	6	32044647	6	0.229528
rs410851	SKIV2L	6	32044647	5	0.975968
rs410851	SKIV2L	6	32044647	8	4.087813

rs449643	SKIV2L	6	32044658	2	4.769499
rs449643	SKIV2L	6	32044658	5	0.011778
rs11970671	CYP21A2	6	32115844	3	0.867362
rs4713498	TNXB	6	32119295	5	0.262821
rs4713498	TNXB	6	32119295	3	1.052797
rs2894232	TNXB	6	32119623	5	0.550365
rs185819	TNXB	6	32158045	3	0.311254
rs185819	TNXB	6	32158045	5	0.258126
rs2228628	CREBL1	6	32196832	4	9.779285
rs35580488	FKBPL	6	32205399	5	4.541994
rs169494	FKBPL	6	32205854	2	2.46671
SNP6-32230450	PPT2	6	32230450	8	8.032209
SNP6-32230450	PPT2	6	32230450	3	0.454533
rs41268924	EGFL8	6	32243115	4	2.879975
rs1061807	AGPAT1	6	32244816	1	2.428515
rs3130349	RNF5	6	32255674	8	2.344494
rs3130349	RNF5	6	32255674	3	0.275192
rs2070600	AGER	6	32259421	4	0.334457
rs1800684	AGER	6	32259972	6	5.721009
rs1800684	AGER	6	32259972	5	5.254975
rs8192583	NOTCH4	6	32271252	8	5.948467
rs520803	NOTCH4	6	32296581	8	0.679716
rs520803	NOTCH4	6	32296581	1	2.270864
rs520803	NOTCH4	6	32296581	7	1.082411
rs520692	NOTCH4	6	32296618	8	1.937816
rs520692	NOTCH4	6	32296618	1	3.566247
rs520692	NOTCH4	6	32296618	7	1.88807
rs415929	NOTCH4	6	32297010	1	0.847137
rs415929	NOTCH4	6	32297010	8	0.292109
rs443198	NOTCH4	6	32298384	8	0.873459
rs367398	NOTCH4	6	32299708	1	1.820321
rs16870005	C6orf10	6	32369131	2	0.294693
rs3749966	C6orf10	6	32369485	2	2.491375
rs3129941	C6orf10	6	32445664	1	0.652578
rs3129941	C6orf10	6	32445664	5	1.270944
rs3129941	C6orf10	6	32445664	8	0.663529
rs3129941	C6orf10	6	32445664	2	1.049398
rs2076523	BTNL2	6	32478813	8	1.730033
rs2076523	BTNL2	6	32478813	2	2.212424
rs14004	HLA-DRA	6	32515687	1	0.569672
rs14004	HLA-DRA	6	32515687	5	0.207664
rs3135391	HLA-DRA	6	32518965	6	0.416032
rs3135391	HLA-DRA	6	32518965	1	2.252944
rs3135391	HLA-DRA	6	32518965	5	0.157946
rs8084	HLA-DRA	6	32519013	8	6.43066
rs8084	HLA-DRA	6	32519013	1	0.537741
rs8084	HLA-DRA	6	32519013	6	0.028224

rs8084	HLA-DRA	6	32519013	5	0.439873
rs8084	HLA-DRA	6	32519013	2	0.078775
rs3177928	HLA-DRA	6	32520413	6	0.117085
rs7196	HLA-DRA	6	32520549	6	0.140709
rs7196	HLA-DRA	6	32520549	5	5.750274
rs7196	HLA-DRA	6	32520549	1	1.569724
rs7196	HLA-DRA	6	32520549	2	4.600678
SNP6-32595220	HLA-DRB5	6	32595220	5	5.68748
SNP6-32605940	HLA-DRB5	6	32605940	6	3.886395
SNP6-32605940	HLA-DRB5	6	32605940	1	3.602057
SNP6-32605940	HLA-DRB5	6	32605940	5	1.96293
rs9269693	HLA-DRB1	6	32654844	1	1.514011
rs35445101	HLA-DRB1	6	32654857	1	0.338714
rs35445101	HLA-DRB1	6	32654857	7	1.390151
rs12722042	HLA-DQA1	6	32713287	2	0.321
SNP6-32717104	HLA-DQA1	6	32717104	1	0.905826
rs707952	HLA-DQA1	6	32717784	7	2.319698
rs1130399	HLA-DQB1	6	32737733	1	0.717207
SNP6-32740576	HLA-DQB1	6	32740576	5	0.523152
SNP6-32740576	HLA-DQB1	6	32740576	6	2.744703
SNP6-32742347	HLA-DQB1	6	32742347	6	2.447025
SNP6-32742347	HLA-DQB1	6	32742347	5	0.479741
rs9276436	HLA-DQA2	6	32822061	7	1.404443
rs9276558	HLA-DQB2	6	32832039	5	0.986654
rs7774954	HLA-DQB2	6	32832167	7	3.600707
rs762815	HLA-DQB2	6	32837620	5	3.94924
rs1573649	HLA-DQB2	6	32839236	5	4.05415
rs1573649	HLA-DQB2	6	32839236	1	2.80231
rs1573649	HLA-DQB2	6	32839236	6	3.261894
rs2070120	HLA-DOB	6	32888892	4	2.998398
rs2070121	HLA-DOB	6	32889532	6	5.601849
rs2070121	HLA-DOB	6	32889532	4	6.362816
rs45459295	HLA-DOB	6	32891064	3	2.050198
rs45459295	HLA-DOB	6	32891064	5	0.230267
rs45459295	HLA-DOB	6	32891064	1	4.09454
rs45459295	HLA-DOB	6	32891064	7	1.464444
SNP6-32892654	HLA-DOB	6	32892654	7	0.337142
SNP6-32892654	HLA-DOB	6	32892654	2	0.918517
rs2071470	HLA-DOB	6	32892731	3	2.261576
rs2071470	HLA-DOB	6	32892731	2	1.509129
rs2071469	HLA-DOB	6	32892761	4	2.482947
rs2071469	HLA-DOB	6	32892761	3	1.302238
rs2071469	HLA-DOB	6	32892761	6	1.515714
rs2071469	HLA-DOB	6	32892761	2	3.027097
rs16870907	TAP2	6	32897785	7	1.239475
rs241456	TAP2	6	32903943	2	5.944902
rs241453	TAP2	6	32904204	2	2.423223

rs241449	TAP2	6	32904631	2	4.316455
rs2228396	TAP2	6	32905787	1	5.301327
rs2228396	TAP2	6	32905787	4	1.253993
rs4576294	TAP2	6	32906526	8	4.834792
rs2228397	TAP2	6	32908202	6	0.654273
rs2228397	TAP2	6	32908202	4	0.120989
rs2228397	TAP2	6	32908202	2	1.823449
rs2228397	TAP2	6	32908202	5	1.085345
rs2228397	TAP2	6	32908202	3	0.12967
rs2228397	TAP2	6	32908202	8	0.230528
rs1800454	TAP2	6	32908390	4	0.651886
rs1800454	TAP2	6	32908390	1	4.672911
rs1800454	TAP2	6	32908390	7	0.591663
rs1800454	TAP2	6	32908390	6	6.216653
401753	TAP2	6	32913568	1	0.53772
401753	TAP2	6	32913568	7	7.100757
401753	TAP2	6	32913568	8	0.647179
401753	TAP2	6	32913568	4	1.587691
401753	TAP2	6	32913568	5	0.039368
401753	TAP2	6	32913568	2	4.536764
rs2071552	TAP2	6	32914439	6	1.034002
rs2071552	TAP2	6	32914439	1	0.115108
rs2071552	TAP2	6	32914439	5	0.689838
rs2071552	TAP2	6	32914439	4	4.506739
rs2071543	PSMB8	6	32919607	2	5.765705
rs8842	TAP1	6	32921139	7	0.618879
rs8842	TAP1	6	32921139	2	2.329335
rs3198005	TAP1	6	32921192	5	0.829888
SNP6-32924421	TAP1	6	32924421	6	2.891193
GA023748	TAP1	6	32924750	2	4.435429
GA023748	TAP1	6	32924750	7	0.331164
rs2127679	TAP1	6	32926214	6	1.175725
rs17879632	TAP1	6	32927946	6	0.683363
rs10751	HLA-DMB	6	33010561	2	4.447627
rs10751	HLA-DMB	6	33010561	4	1.006525
rs10751	HLA-DMB	6	33010561	6	3.095715
rs17617321	HLA-DMB	6	33014564	3	0.125605
rs17617321	HLA-DMB	6	33014564	6	4.814066
rs17617333	HLA-DMB	6	33014630	6	6.021499
rs17617333	HLA-DMB	6	33014630	3	0.047421
rs17583782	HLA-DMB	6	33014694	3	0.297407
rs17583782	HLA-DMB	6	33014694	6	1.342147
rs45514397	HLA-DMB	6	33014694	6	1.710221
rs45514397	HLA-DMB	6	33014694	3	0.231349
rs6926628	HLA-DMA	6	33025476	6	0.829388
rs6926628	HLA-DMA	6	33025476	3	0.198687
rs9276935	BRD2	6	33044419	6	0.328629

rs206781	BRD2	6	33054111	8	6.208661
rs206781	BRD2	6	33054111	3	0.613282
rs592625	HLA-DOA	6	33080668	7	0.024082
rs376892	HLA-DOA	6	33080865	2	1.0403
rs2581	HLA-DOA	6	33082379	3	2.443763
GA023780	HLA-DPA1	6	33144413	3	0.206283
rs12722009	HLA-DPA1	6	33145397	3	0.870644
SNP6-33156664	HLA-DPB1	6	33156664	3	2.507087
SNP6-33156664	HLA-DPB1	6	33156664	7	0.267536
rs9277533	HLA-DPB1	6	33162699	7	2.31327
rs9277533	HLA-DPB1	6	33162699	3	1.77549
rs2295119	LOC646702	6	33168848	3	0.350915
rs1799911	COL11A2	6	33246655	5	0.192914
rs1799910	COL11A2	6	33247306	4	0.037259
rs9277934	COL11A2	6	33261506	3	0.528102
rs9277934	COL11A2	6	33261506	5	0.754079
rs2072915	RXRB	6	33270060	4	0.123999
rs2072915	RXRB	6	33270060	5	5.369377
rs2072915	RXRB	6	33270060	3	0.005224
SNP6-33277160	SLC39A7	6	33277160	5	0.132003
rs1547387	SLC39A7	6	33277873	5	4.565836
rs17215210	VPS52	6	33347457	1	6.028317
rs3130257	WDR46	6	33364449	1	4.835922
rs1059288	TAPBP	6	33375650	3	0.29965
rs1059288	TAPBP	6	33375650	4	0.754871
rs3130100	ZBTB22	6	33391744	4	0.587236
rs3130100	ZBTB22	6	33391744	3	1.529865
rs3130267	MYL8P	6	33414772	4	2.878161
rs3130267	MYL8P	6	33414772	3	2.756387
rs3130267	MYL8P	6	33414772	1	3.350644
rs211449	LYPLA2P1	6	33441894	4	0.270951
rs211449	LYPLA2P1	6	33441894	7	5.032202
rs465223	KIFC1	6	33467694	7	0.6476
rs465223	KIFC1	6	33467694	8	7.113643
rs465223	KIFC1	6	33467694	3	0.111623
rs4231	PHF1	6	33491952	3	0.257953
rs4231	PHF1	6	33491952	4	3.167112
rs4231	PHF1	6	33491952	1	2.878381
rs407710	FLJ43752	6	33662235	2	1.797776
rs4559081	FLJ43752	6	33663326	3	0.762481
rs4559081	FLJ43752	6	33663326	1	1.84403
rs210162	FLJ43752	6	33668874	2	3.376478
rs2229637	ITPR3	6	33751536	4	0.751979
rs2229637	ITPR3	6	33751536	1	0.562469
rs11967839	ITPR3	6	33756122	2	1.047677
rs2966	IHPK3	6	33797498	8	0.902253
rs2967	IHPK3	6	33797512	8	0.388041

rs559290	IHPK3	6	33797934	8	0.895246
rs623813	IHPK3	6	33811067	5	0.525687
rs545787	IHPK3	6	33811208	8	0.454972
rs12189725	IHPK3	6	33811372	5	0.702818
rs12189725	IHPK3	6	33811372	4	1.759669
rs12189725	IHPK3	6	33811372	7	1.35342
rs12189725	IHPK3	6	33811372	1	3.544347
rs12662447	IHPK3	6	33822651	1	2.707571
rs12662447	IHPK3	6	33822651	7	1.107153
rs12662447	IHPK3	6	33822651	4	2.923131
rs12662447	IHPK3	6	33822651	5	2.299129
SNP6-34620173	SPDEF	6	34620173	7	0.089148
rs1201872	C6orf106	6	34663687	1	0.692524
rs1201872	C6orf106	6	34663687	2	4.965604
rs4259246	UHRF1BP1	6	34911925	3	0.234428
rs4259246	UHRF1BP1	6	34911925	2	0.678515
rs4259246	UHRF1BP1	6	34911925	4	1.832395
rs4259246	UHRF1BP1	6	34911925	7	1.759068
rs4259246	UHRF1BP1	6	34911925	1	0.572022
SNP6-34934899	UHRF1BP1	6	34934899	1	5.966302
rs9469913	UHRF1BP1	6	34935063	3	0.840184
rs11552398	ANKS1A	6	35166509	6	3.420305
SNP6-35196359	TCP11	6	35196359	8	0.172478
SNP6-35196359	TCP11	6	35196359	6	1.746307
rs28360552	ZNF76	6	35361952	7	1.218253
rs28360552	ZNF76	6	35361952	2	0.722716
rs28360552	ZNF76	6	35361952	1	1.245125
rs16874216	ZNF76	6	35371219	1	0.191969
rs16874216	ZNF76	6	35371219	7	0.595635
rs16874262	DEF6	6	35373689	7	5.306338
rs9658134	PPARD	6	35486776	3	0.458462
rs9658167	PPARD	6	35502058	7	5.217221
rs1051952	TULP1	6	35573764	8	0.111654
rs1051952	TULP1	6	35573764	7	0.117079
rs2064318	TULP1	6	35585003	1	0.332963
rs12211728	LOC340204	6	35863636	2	2.9698
rs12211728	LOC340204	6	35863636	7	0.314768
rs12211728	LOC340204	6	35863636	5	0.685793
rs12211728	LOC340204	6	35863636	4	3.784655
rs12211728	LOC340204	6	35863636	3	0.188908
rs2295852	SLC26A8	6	36031224	8	1.277719
rs2295852	SLC26A8	6	36031224	5	1.188442
rs2295852	SLC26A8	6	36031224	4	0.916428
rs17713154	SLC26A8	6	36075750	7	0.09739
rs17713154	SLC26A8	6	36075750	2	2.761777
rs743923	SLC26A8	6	36088099	3	0.943068
rs3748045	BRPF3	6	36306555	6	0.60575

rs3748045	BRPF3	6	36306555	1	0.713008
rs3748045	BRPF3	6	36306555	4	0.140553
rs3748045	BRPF3	6	36306555	8	1.134621
rs12199580	PNPLA1	6	36378108	8	0.692835
rs4713956	PNPLA1	6	36383436	8	2.038192
rs4713956	PNPLA1	6	36383436	6	0.250258
rs41270098	C6orf222	6	36402842	2	1.857248
rs2234080	ETV7	6	36444742	5	1.336095
rs2234071	ETV7	6	36463350	6	1.884803
rs2234071	ETV7	6	36463350	2	3.856483
rs2234071	ETV7	6	36463350	4	0.554428
rs2234071	ETV7	6	36463350	7	1.577199
rs2234071	ETV7	6	36463350	5	0.531114
rs584196	KCTD20	6	36564221	7	6.223117
rs584196	KCTD20	6	36564221	1	5.760775
rs584196	KCTD20	6	36564221	6	4.728112
rs2071809	STK38	6	36571605	6	0.533126
rs12199346	LOC389386	6	36749524	8	3.492188
rs12199346	LOC389386	6	36749524	6	3.411696
rs12199346	LOC389386	6	36749524	3	3.647832
rs12199346	LOC389386	6	36749524	5	3.781029
rs12199346	LOC389386	6	36749524	2	2.801819
rs12199346	LOC389386	6	36749524	4	4.173782
rs4711459	LOC389386	6	36750002	5	0.642175
rs4711460	LOC389386	6	36750106	5	2.412746
rs10947623	LOC389386	6	36750814	5	0.054719
rs87152	FLJ43093	6	36808384	1	1.544259
rs236460	FLJ43093	6	36808811	6	0.902024
rs236460	FLJ43093	6	36808811	1	0.422966
rs236459	FLJ43093	6	36808877	1	0.809544
rs9368958	CPNE5	6	36816534	3	0.617461
rs9368958	CPNE5	6	36816534	1	1.446956
rs9368958	CPNE5	6	36816534	6	1.813318
rs1065261	CPNE5	6	36816799	5	0.42256
rs1065261	CPNE5	6	36816799	3	0.355257
rs1065260	CPNE5	6	36816848	6	0.669213
rs1065260	CPNE5	6	36816848	3	0.161559
rs1065260	CPNE5	6	36816848	1	0.199212
rs1065260	CPNE5	6	36816848	5	0.022365
rs1065259	CPNE5	6	36816895	6	3.790925
rs1065259	CPNE5	6	36816895	3	1.240554
rs1065259	CPNE5	6	36816895	5	1.629227
rs1065259	CPNE5	6	36816895	1	2.275341
rs1065258	CPNE5	6	36817043	5	1.495581
rs1065258	CPNE5	6	36817043	3	0.026992
rs763048	CPNE5	6	36841110	3	0.621952
rs763048	CPNE5	6	36841110	5	0.120308

rs12194408	PPIL1	6	36947576	3	0.06208
rs1405070	PI16	6	37030363	1	3.167959
rs1405070	PI16	6	37030363	3	1.578719
rs1063021	PI16	6	37040305	7	0.711902
rs1063021	PI16	6	37040305	3	0.4686
rs1046279	PI16	6	37040549	3	0.095795
rs1046279	PI16	6	37040549	7	1.310326
rs831510	FGD2	6	37084615	8	0.835551
rs831510	FGD2	6	37084615	5	0.936201
rs831510	FGD2	6	37084615	4	0.525882
rs831510	FGD2	6	37084615	3	0.502233
rs12202642	FGD2	6	37087561	3	1.199842
rs708017	FGD2	6	37104751	1	4.004414
rs708017	FGD2	6	37104751	7	2.008137
rs9394402	LOC646875	6	37153527	1	0.861473
rs9394402	LOC646875	6	37153527	6	0.974213
rs9394402	LOC646875	6	37153527	3	0.456947
GA023892	PIM1	6	37249940	5	0.000357
GA023892	PIM1	6	37249940	7	0.179739
rs1055461	C6orf128	6	37288021	6	5.814579
rs1874736	KIAA0082	6	37555972	3	0.837967
rs1874736	KIAA0082	6	37555972	6	3.377066
rs1874736	KIAA0082	6	37555972	8	3.714027
rs12191311	MDGA1	6	37711685	3	0.492066
rs12191311	MDGA1	6	37711685	1	0.365023
rs3846874	MDGA1	6	37731544	5	3.339749
rs3846874	MDGA1	6	37731544	3	2.155865
rs2273110	MDGA1	6	37734120	5	0.186694
rs3823430	DNAH8	6	38858866	2	4.00594
rs9357283	DNAH8	6	38908142	5	1.723238
rs2061907	DNAH8	6	38933307	5	1.024579
rs6918287	GLP1R	6	39141580	3	0.140987
rs2235868	GLP1R	6	39148632	2	5.389838
rs2235868	GLP1R	6	39148632	3	1.58867
rs2235868	GLP1R	6	39148632	5	1.866441
rs2235868	GLP1R	6	39148632	4	0.97678
rs2235868	GLP1R	6	39148632	7	0.021541
rs1042044	GLP1R	6	39149480	4	1.099821
rs1042044	GLP1R	6	39149480	5	1.047848
rs1042044	GLP1R	6	39149480	8	0.900726
rs1042044	GLP1R	6	39149480	3	1.637019
rs1042044	GLP1R	6	39149480	2	4.767836
rs1042044	GLP1R	6	39149480	6	2.354044
rs1126476	GLP1R	6	39156469	3	0.04829
rs1126476	GLP1R	6	39156469	1	0.277538
rs1126476	GLP1R	6	39156469	5	0.270037
rs1126476	GLP1R	6	39156469	7	0.073402

rs10305516	GLP1R	6	39162567	3	2.765239
rs4714210	GLP1R	6	39163463	8	2.224798
rs4714210	GLP1R	6	39163463	4	2.046632
rs4714210	GLP1R	6	39163463	1	2.621135
rs4714210	GLP1R	6	39163463	7	1.163313
rs4714210	GLP1R	6	39163463	2	1.724476
rs4714210	GLP1R	6	39163463	5	1.475093
rs4714210	GLP1R	6	39163463	6	1.197237
rs4714210	GLP1R	6	39163463	3	1.977155
rs10305522	GLP1R	6	39163494	6	0.813339
rs10305522	GLP1R	6	39163494	2	5.595661
rs10305522	GLP1R	6	39163494	5	1.492137
rs10305522	GLP1R	6	39163494	7	0.119709
rs10947804	KCNK17	6	39390014	1	1.513653
rs10947804	KCNK17	6	39390014	5	5.039303
rs10947804	KCNK17	6	39390014	7	1.108115
rs10947804	KCNK17	6	39390014	8	0.684189
rs10947804	KCNK17	6	39390014	4	2.153868
rs11756091	KCNK16	6	39390784	5	1.41342
rs11756686	KIF6	6	39411944	4	2.195376
rs3008816	DAAM2	6	39980218	2	4.610173
rs9394693	LRFN2	6	40508846	4	0.122539
rs736795	UNC5CL	6	41106119	5	0.146225
rs736795	UNC5CL	6	41106119	7	0.811415
rs2234255	TREM2	6	41235521	7	0.638633
rs17328707	TREML2	6	41267131	8	3.28978
rs7754593	TREML2	6	41267200	3	0.595216
rs3800342	TREML2	6	41267510	1	0.177071
rs3800342	TREML2	6	41267510	3	0.072647
rs3747742	TREML2	6	41270496	3	3.543855
rs3747742	TREML2	6	41270496	6	3.829218
rs3747742	TREML2	6	41270496	1	2.392028
rs11962624	TREML3	6	41284737	3	0.648969
rs11962624	TREML3	6	41284737	7	0.836832
rs11962624	TREML3	6	41284737	2	0.626947
rs9349180	TREML3	6	41293452	8	1.309469
rs9349180	TREML3	6	41293452	7	0.514377
rs9369265	TREML4	6	41304583	5	2.741912
rs2101269	TREML4	6	41304705	5	0.716761
rs7769759	TREML4	6	41305835	5	1.334879
rs13204001	TREML4	6	41313135	1	0.739183
rs13204001	TREML4	6	41313135	5	0.444161
rs13204001	TREML4	6	41313135	3	0.795503
rs13204353	TREML4	6	41313213	3	0.753687
rs2234246	TREM1	6	41351718	7	2.184006
rs2234246	TREM1	6	41351718	4	0.490449
rs2234246	TREM1	6	41351718	6	2.236375

rs2236369	NCR2	6	41417530	1	0.45463
rs2273962	NCR2	6	41426416	6	3.636476
rs9471607	FOXP4	6	41675106	8	2.885019
rs9471607	FOXP4	6	41675106	7	0.53765
rs9471607	FOXP4	6	41675106	3	0.088598
rs913075	FOXP4	6	41676884	8	0.121647
rs913075	FOXP4	6	41676884	7	0.492239
rs913075	FOXP4	6	41676884	1	4.600131
rs913075	FOXP4	6	41676884	5	0.075045
rs913076	FOXP4	6	41677096	8	1.13647
rs913076	FOXP4	6	41677096	7	3.681133
rs913076	FOXP4	6	41677096	3	0.738211
rs2274578	MED20	6	41996805	6	7.145236
rs2274578	MED20	6	41996805	2	3.53893
rs2274578	MED20	6	41996805	7	7.821796
rs2274578	MED20	6	41996805	5	0.208389
rs10947995	BYSL	6	41997201	7	1.294675
rs9688934	C6orf132	6	42218149	5	0.137362
rs9688934	C6orf132	6	42218149	4	0.203201
rs9688934	C6orf132	6	42218149	8	0.76004
rs9688934	C6orf132	6	42218149	1	6.284938
rs2207730	GUCA1A	6	42231295	5	1.583467
rs9471793	GUCA1A	6	42249247	6	0.763596
rs9471793	GUCA1A	6	42249247	5	1.41441
rs3749920	GUCA1B	6	42260311	5	0.894166
rs3749920	GUCA1B	6	42260311	1	5.023663
rs1474867	GUCA1B	6	42270553	5	0.619502
rs1474867	GUCA1B	6	42270553	1	4.337869
rs1474867	GUCA1B	6	42270553	7	0.464728
rs1474867	GUCA1B	6	42270553	3	2.185555
rs4714580	MRPS10	6	42282536	1	3.489797
rs4714580	MRPS10	6	42282536	7	0.669643
rs4714580	MRPS10	6	42282536	3	0.793623
rs4714580	MRPS10	6	42282536	5	4.174782
rs2295275	TRERF1	6	42332524	1	4.481363
rs5014584	UBR2	6	42708297	5	0.485307
rs835	PRPH2	6	42773866	4	4.492983
rs835	PRPH2	6	42773866	6	3.862385
rs434102	PRPH2	6	42774039	3	1.466949
rs434102	PRPH2	6	42774039	5	3.498751
rs434102	PRPH2	6	42774039	1	4.002727
rs390659	PRPH2	6	42774142	5	0.466147
rs390659	PRPH2	6	42774142	3	0.513427
rs390659	PRPH2	6	42774142	1	3.879928
rs7764439	PRPH2	6	42797733	7	2.060831
rs7764439	PRPH2	6	42797733	3	2.980962
rs3800291	FLJ38717	6	42859435	7	0.30661

rs3800291	FLJ38717	6	42859435	2	0.436204
rs3800291	FLJ38717	6	42859435	1	0.022181
rs9471932	KIAA0240	6	42932885	4	0.16028
rs7764865	KIAA0240	6	42943465	7	4.215471
rs736158	PEX6	6	43039732	3	1.139191
rs736158	PEX6	6	43039732	7	0.964964
rs2274515	PEX6	6	43041504	3	0.521391
rs2274515	PEX6	6	43041504	7	0.568771
rs11751058	MEA1	6	43088196	1	5.574732
rs11751058	MEA1	6	43088196	8	5.035706
rs2273917	CUL7	6	43116276	3	0.250661
rs2273917	CUL7	6	43116276	7	0.497827
rs4711738	CUL7	6	43128166	3	0.183691
rs4711738	CUL7	6	43128166	7	0.12865
rs4714661	KLC4	6	43142985	3	1.033907
SNP6-43261765	PARC	6	43261765	6	0.295557
rs7832	C6orf108	6	43301855	3	0.528062
rs10948072	ZNF318	6	43415839	7	3.370997
rs700008	ABCC10	6	43507815	8	0.811649
rs35192247	DLK2	6	43526506	2	4.661319
rs34764062	DLK2	6	43530174	3	0.98321
rs2231767	YIPF3	6	43589099	2	4.633392
rs2231767	YIPF3	6	43589099	3	0.964226
SNP6-43592510	YIPF3	6	43592510	5	0.028289
rs1106841	XPO5	6	43604640	1	0.709751
rs2010963	VEGFA	6	43846328	3	0.223366
rs2010963	VEGFA	6	43846328	2	5.170048
rs2010963	VEGFA	6	43846328	7	0.604864
rs2295333	C6orf223	6	44076792	3	1.947103
rs700006	MRPL14	6	44189488	5	3.565117
rs700006	MRPL14	6	44189488	1	2.073324
rs700006	MRPL14	6	44189488	6	1.125903
rs4714761	TMEM63B	6	44225585	2	2.945067
GA005842	TMEM63B	6	44225642	2	5.693886
SNP6-44255799	CAPN11	6	44255799	6	0.565411
SNP6-44255799	CAPN11	6	44255799	5	0.753252
SNP6-44255799	CAPN11	6	44255799	7	0.813686
SNP6-44255799	CAPN11	6	44255799	3	0.364653
SNP6-44255799	CAPN11	6	44255799	8	0.936267
rs7761137	CAPN11	6	44259468	4	5.640891
rs7761137	CAPN11	6	44259468	6	0.514951
rs7761137	CAPN11	6	44259468	5	0.818046
rs28385699	NFKBIE	6	44334411	2	5.79193
rs516582	TCTE1	6	44358143	1	1.275276
rs516582	TCTE1	6	44358143	3	0.66779
rs2297336	TCTE1	6	44361743	3	0.72654
rs2297336	TCTE1	6	44361743	1	2.782166

rs324146	TCTE1	6	44363437	3	0.064809
rs2297334	CDC5L	6	44463377	5	0.482053
rs2297334	CDC5L	6	44463377	4	4.400266
rs2297333	CDC5L	6	44463401	5	4.104371
rs2297333	CDC5L	6	44463401	4	0.384058
rs2273666	CDC5L	6	44521441	5	0.31711
rs2273666	CDC5L	6	44521441	4	6.088178
rs9369514	SUPT3H	6	44905249	4	5.295085
rs9369514	SUPT3H	6	44905249	6	6.736938
rs9369514	SUPT3H	6	44905249	1	0.518262
rs9369514	SUPT3H	6	44905249	8	5.941869
rs9472463	SUPT3H	6	45398612	6	0.672514
rs11752816	ENPP4	6	46215271	1	3.829263
rs7451713	ENPP4	6	46215711	2	8.069451
rs7451713	ENPP4	6	46215711	3	0.621693
rs2295017	ENPP5	6	46235795	8	0.213263
rs2295017	ENPP5	6	46235795	1	0.009638
rs2295017	ENPP5	6	46235795	2	5.458597
rs2295017	ENPP5	6	46235795	3	0.492023
rs2295017	ENPP5	6	46235795	5	0.229977
rs3822892	ENPP5	6	46246574	1	4.604887
rs3799893	RCAN2	6	46297046	1	4.001679
rs3734655	RCAN2	6	46298476	1	4.062606
rs9381461	CYP39A1	6	46625701	7	0.204789
rs9381461	CYP39A1	6	46625701	2	0.752965
rs9381461	CYP39A1	6	46625701	4	0.519665
rs7761731	CYP39A1	6	46671776	8	0.697599
rs3757241	SLC25A27	6	46731657	1	5.207357
rs3757241	SLC25A27	6	46731657	4	1.049676
rs3799277	TDRD6	6	46765016	4	0.704608
rs3799277	TDRD6	6	46765016	1	5.41319
rs1805017	PLA2G7	6	46792181	8	5.171111
rs1805017	PLA2G7	6	46792181	5	6.505808
SNP6-46909178	MEP1A	6	46909178	4	4.700106
rs2277115	GPR110	6	47117910	4	0.107574
rs2277115	GPR110	6	47117910	7	4.650404
rs4715039	GPR111	6	47739269	1	3.837234
rs13212023	GPR111	6	47754707	5	2.45056
rs13212023	GPR111	6	47754707	7	0.767395
rs13212023	GPR111	6	47754707	8	1.46527
rs8180544	GPR111	6	47757181	7	0.792175
rs8180544	GPR111	6	47757181	8	2.203408
rs8180544	GPR111	6	47757181	4	1.545491
rs8180544	GPR111	6	47757181	1	6.342608
rs17541107	GPR111	6	47757224	7	0.327953
rs17541107	GPR111	6	47757224	4	0.922041
rs17541107	GPR111	6	47757224	5	0.970035

rs17541107	GPR111	6	47757224	1	5.853905
rs9296573	GPR111	6	47762701	1	1.906133
rs9369738	GPR115	6	47790563	7	0.213909
rs9381619	OPN5	6	47901250	3	1.116939
SNP6-49523407	MUT	6	49523407	7	6.660736
SNP6-49523407	MUT	6	49523407	6	0.483395
rs699957	CRISP2	6	49789216	1	3.643142
rs699957	CRISP2	6	49789216	3	2.009039
rs1864312	CRISP3	6	49809398	1	0.787166
rs1864312	CRISP3	6	49809398	4	1.669552
rs495335	CRISP3	6	49809482	1	0.913628
rs495335	CRISP3	6	49809482	4	2.393839
rs780556	CRISP1	6	49910656	1	0.474033
rs12205497	CRISP1	6	49922338	3	0.471845
rs2857514	TFAP2B	6	50919242	3	0.329195
rs2857514	TFAP2B	6	50919242	7	3.392227
rs2857514	TFAP2B	6	50919242	1	4.83502
rs2857514	TFAP2B	6	50919242	8	0.319354
rs2784200	PKHD1	6	51589630	5	0.863587
rs2784200	PKHD1	6	51589630	7	0.66556
rs9381994	PKHD1	6	51591920	7	0.442625
rs4715227	PKHD1	6	51599843	7	3.781919
rs765525	PKHD1	6	51721136	3	0.442673
rs9349603	PKHD1	6	51828797	7	0.040688
rs12210295	PKHD1	6	51840766	3	0.014775
rs1266923	PKHD1	6	51932639	1	1.048061
rs6901799	PKHD1	6	52055216	3	0.815933
rs3748066	PAQR8	6	52375967	1	2.838633
rs17591381	PAQR8	6	52376849	3	0.507111
rs17591381	PAQR8	6	52376849	8	3.035111
rs3799274	PAQR8	6	52377005	8	4.81483
rs3799274	PAQR8	6	52377005	1	4.364509
rs3799274	PAQR8	6	52377005	7	1.586087
rs3799274	PAQR8	6	52377005	4	1.140511
rs655113	PAQR8	6	52377110	5	1.268634
rs655113	PAQR8	6	52377110	4	2.294585
rs655113	PAQR8	6	52377110	3	1.677314
rs655113	PAQR8	6	52377110	7	1.620289
rs3180068	PAQR8	6	52377644	2	4.654132
rs10484875	PAQR8	6	52377895	3	0.586866
rs10484875	PAQR8	6	52377895	8	7.734488
rs10807445	PAQR8	6	52378951	3	0.685156
rs1297	PAQR8	6	52380272	4	0.872175
rs1297	PAQR8	6	52380272	1	4.752144
rs1297	PAQR8	6	52380272	7	2.4091
rs3804506	EFHC1	6	52411250	7	0.024782
rs3804506	EFHC1	6	52411250	4	0.056585

rs3804506	EFHC1	6	52411250	1	2.852474
rs17851770	EFHC1	6	52465030	2	5.156635
rs17851770	EFHC1	6	52465030	5	0.546776
rs9474230	TRAM2	6	52474843	4	2.932587
rs9474230	TRAM2	6	52474843	6	0.399045
rs2670125	LOC730101	6	52637863	6	0.275407
rs2670125	LOC730101	6	52637863	1	0.143318
rs2670125	LOC730101	6	52637863	2	0.259723
rs2670125	LOC730101	6	52637863	4	0.638852
rs2180314	GSTA2	6	52725690	3	0.604224
rs2180314	GSTA2	6	52725690	6	1.60528
rs6458868	LOC647169	6	52738112	1	2.300321
rs405729	GSTA4	6	52950740	7	1.291741
rs405729	GSTA4	6	52950740	6	1.000511
rs405729	GSTA4	6	52950740	3	0.414364
rs405729	GSTA4	6	52950740	2	4.331174
rs2397142	LOC647190	6	53308258	6	1.791469
rs2397142	LOC647190	6	53308258	8	4.909222
rs2397142	LOC647190	6	53308258	7	0.026717
rs2397142	LOC647190	6	53308258	3	0.015505
rs2397142	LOC647190	6	53308258	2	3.681769
rs2397142	LOC647190	6	53308258	5	3.092944
rs209510	LOC647190	6	53309994	5	5.549961
rs209510	LOC647190	6	53309994	2	5.241598
rs209510	LOC647190	6	53309994	8	1.232584
rs4562128	LOC647190	6	53310565	6	3.737632
rs4562128	LOC647190	6	53310565	7	2.141345
rs4562128	LOC647190	6	53310565	3	0.692141
rs4562128	LOC647190	6	53310565	5	0.164177
rs4562128	LOC647190	6	53310565	2	4.787646
rs3823111	KLHL31	6	53624994	5	1.840928
rs3823111	KLHL31	6	53624994	4	0.02006
rs10948758	KLHL31	6	53627021	4	0.365918
rs10948758	KLHL31	6	53627021	7	0.37808
rs10948758	KLHL31	6	53627021	5	1.020931
rs10948758	KLHL31	6	53627021	3	0.008903
rs3799260	KLHL31	6	53627564	3	1.253087
rs3799260	KLHL31	6	53627564	7	0.641324
rs12210809	KLHL31	6	53627816	3	0.373687
rs17625497	C6orf142	6	53991802	1	0.825806
rs4712056	C6orf142	6	54097485	4	3.099342
rs4712056	C6orf142	6	54097485	8	3.501753
rs4712056	C6orf142	6	54097485	1	4.427091
rs3756990	BMP5	6	55728210	1	4.022151
rs41271330	BMP5	6	55746987	5	0.962147
rs41271330	BMP5	6	55746987	4	0.703901
rs41271330	BMP5	6	55746987	1	4.661813

rs41271330	BMP5	6	55746987	8	0.67149
rs3734444	BMP5	6	55847512	6	2.586885
rs3734444	BMP5	6	55847512	3	0.593618
rs3734444	BMP5	6	55847512	1	0.808067
rs35471617	COL21A1	6	56141053	5	0.26081
rs35471617	COL21A1	6	56141053	4	0.345551
rs6911502	LOC442221	6	56405527	1	5.489134
rs6911502	LOC442221	6	56405527	5	5.719144
rs6911502	LOC442221	6	56405527	2	5.123051
SNP6-56525504	DST	6	56525504	1	0.255536
SNP6-56525504	DST	6	56525504	2	3.23773
rs16888055	DST	6	56579954	2	6.843964
rs13193465	C6orf65	6	56928207	5	1.518704
rs13193465	C6orf65	6	56928207	1	1.446682
SNP6-57025479	KIAA1586	6	57025479	8	6.111075
SNP6-57025479	KIAA1586	6	57025479	4	4.202174
SNP6-57025479	KIAA1586	6	57025479	7	0.342481
rs1411578	RAB23	6	57162802	7	0.702935
rs1411578	RAB23	6	57162802	1	4.157578
rs1879	LOC727916	6	64339528	3	0.064903
rs913543	BAI3	6	70127894	7	2.531547
rs913543	BAI3	6	70127894	1	1.347821
rs913543	BAI3	6	70127894	6	2.962746
rs913543	BAI3	6	70127894	3	1.780106
rs9354880	LMBRD1	6	70464186	4	0.232407
rs9354880	LMBRD1	6	70464186	1	2.300965
rs1736	COL19A1	6	70973719	4	4.622727
rs1201814	COL19A1	6	70974694	1	0.10605
rs1201814	COL19A1	6	70974694	4	0.175918
rs9455190	LOC642590	6	71374681	8	3.088512
rs9455190	LOC642590	6	71374681	4	3.474129
rs9455190	LOC642590	6	71374681	2	3.852307
rs9455190	LOC642590	6	71374681	6	4.22343
rs2691511	LOC642590	6	71374792	2	0.786439
rs2691511	LOC642590	6	71374792	8	1.420719
rs2691511	LOC642590	6	71374792	6	1.172175
rs2691511	LOC642590	6	71374792	3	1.769994
rs17707714	SMAP1	6	71627601	1	4.262554
rs2273888	OGFRL1	6	72063233	6	0.555487
rs12214894	LOC642681	6	73397014	4	0.401404
rs12214894	LOC642681	6	73397014	7	1.019485
SNP6-73961278	KCNQ5	6	73961278	8	0.079366
SNP6-73961278	KCNQ5	6	73961278	4	0.740242
rs6927398	KHDC1	6	73991856	4	0.951197
rs496530	OOEP	6	74135745	4	3.361258
rs496530	OOEP	6	74135745	8	1.024137
rs496530	OOEP	6	74135745	3	1.310203

rs558198	DDX43	6	74161278	3	0.206641
rs311686	DDX43	6	74182596	3	1.228851
rs311686	DDX43	6	74182596	4	2.748591
rs311686	DDX43	6	74182596	2	5.158507
rs311686	DDX43	6	74182596	8	0.939079
rs311685	DDX43	6	74182609	3	0.74673
rs311685	DDX43	6	74182609	2	6.721981
rs311685	DDX43	6	74182609	8	0.539473
rs311685	DDX43	6	74182609	4	2.187418
rs311683	DDX43	6	74183662	8	0.431857
rs311683	DDX43	6	74183662	3	0.928328
rs311683	DDX43	6	74183662	4	0.625142
rs311683	DDX43	6	74183662	2	1.121135
rs3734518	SLC17A5	6	74361136	5	0.09259
rs3734518	SLC17A5	6	74361136	6	0.45353
rs3734518	SLC17A5	6	74361136	3	0.075046
rs3734518	SLC17A5	6	74361136	8	2.196215
rs3734518	SLC17A5	6	74361136	2	2.61958
rs3734518	SLC17A5	6	74361136	1	0.85136
rs2917862	CD109	6	74578668	3	1.104269
rs970547	COL12A1	6	75854022	4	0.017435
rs970547	COL12A1	6	75854022	7	0.483951
rs970547	COL12A1	6	75854022	1	3.171682
rs2951916	LOC643227	6	76231577	5	1.477146
rs3778005	IMPG1	6	76690105	2	4.926124
rs3778005	IMPG1	6	76690105	3	1.092419
rs3778005	IMPG1	6	76690105	4	0.689289
rs3734313	IMPG1	6	76697501	4	0.800691
rs3734313	IMPG1	6	76697501	3	0.771636
rs10943299	IMPG1	6	76697523	6	2.212297
rs10943299	IMPG1	6	76697523	7	1.816443
rs3734311	IMPG1	6	76717271	4	1.837307
rs6908105	IRAK1BP1	6	79651816	3	0.605786
rs6908105	IRAK1BP1	6	79651816	5	0.672558
rs16890805	LCA5	6	80252140	1	5.61123
rs2803195	LCA5	6	80285392	5	0.836723
rs2803195	LCA5	6	80285392	7	1.28241
rs343705	ELOVL4	6	80681249	4	1.790361
rs343705	ELOVL4	6	80681249	3	2.627269
rs7759915	LOC643562	6	80828706	5	0.210345
rs3967160	LOC643562	6	80834327	2	4.189678
rs3967160	LOC643562	6	80834327	1	4.205543
rs7758761	BCKDHB	6	81112018	2	3.653369
rs7740958	BCKDHB	6	81112132	2	0.051163
rs3812145	LOC728360	6	82611735	8	0.721024
rs3812145	LOC728360	6	82611735	3	1.278524
rs3812145	LOC728360	6	82611735	4	0.406619

rs3763244	LOC728360	6	82612340	6	2.166276
rs3763244	LOC728360	6	82612340	5	1.012468
rs3763244	LOC728360	6	82612340	2	6.363866
rs770911	TPBG	6	83131084	1	2.171133
rs770911	TPBG	6	83131084	4	0.079087
rs770911	TPBG	6	83131084	3	0.74844
rs4706975	UBE2CBP	6	83659302	2	0.003715
rs4706975	UBE2CBP	6	83659302	1	0.100444
rs7739323	UBE2CBP	6	83723764	5	0.195251
rs7739323	UBE2CBP	6	83723764	8	6.120896
rs9449776	C6orf117	6	84855778	1	5.065496
rs2875382	C6orf117	6	84855904	1	0.113755
rs9449777	C6orf117	6	84855950	1	5.489818
rs1045649	C6orf117	6	84856885	1	4.357343
rs3219	C6orf117	6	84857097	1	5.595146
rs3734451	LOC442233	6	85197118	3	1.479197
rs3857479	KRT18P30	6	86054896	5	3.407974
rs3812138	NT5E	6	86255952	1	2.913925
rs3812138	NT5E	6	86255952	4	0.598429
rs3812138	NT5E	6	86255952	7	0.458438
rs6631	CGA	6	87851978	2	2.654277
rs6631	CGA	6	87851978	1	0.011393
rs6631	CGA	6	87851978	8	0.581491
rs1055409	CGA	6	87852029	1	4.359517
rs1055409	CGA	6	87852029	8	0.898349
rs1055409	CGA	6	87852029	4	0.230419
rs3812132	ZNF292	6	88027020	8	0.121134
rs3812132	ZNF292	6	88027020	7	0.179873
rs3812132	ZNF292	6	88027020	4	0.410262
rs3812132	ZNF292	6	88027020	2	4.16219
rs3812132	ZNF292	6	88027020	3	0.180953
rs3812130	GJB7	6	88050480	7	0.081862
rs3812130	GJB7	6	88050480	3	1.567757
rs3812130	GJB7	6	88050480	8	1.157033
rs3812130	GJB7	6	88050480	4	4.866857
rs3812130	GJB7	6	88050480	2	2.833556
rs6934603	GJB7	6	88050734	2	3.956148
rs6934603	GJB7	6	88050734	4	0.213742
rs6934603	GJB7	6	88050734	3	0.62828
rs6934603	GJB7	6	88050734	8	2.293462
rs6934603	GJB7	6	88050734	7	0.909029
rs4707358	GJB7	6	88051291	8	0.54998
rs4707358	GJB7	6	88051291	7	0.842725
rs4707358	GJB7	6	88051291	2	3.018102
rs4707358	GJB7	6	88051291	4	0.179346
rs397516	C6orf162	6	88107771	8	2.411658
rs397516	C6orf162	6	88107771	7	0.603865

rs397516	C6orf162	6	88107771	2	4.401288
rs397516	C6orf162	6	88107771	4	0.865036
rs9353482	C6orf163	6	88131841	3	0.893514
rs16879281	C6orf165	6	88183142	3	0.374704
rs2307393	ORC3L	6	88419662	4	0.501118
rs806368	CNR1	6	88906819	4	0.07964
rs12720071	CNR1	6	88907900	2	1.900351
rs4707436	CNR1	6	88908470	1	0.138341
rs2610767	RNGTT	6	89378958	8	0.524078
rs1796743	GABRR1	6	89945463	1	0.483777
rs1796743	GABRR1	6	89945463	3	0.120287
rs282129	GABRR2	6	90024217	5	0.159382
rs282129	GABRR2	6	90024217	7	1.054187
rs282117	GABRR2	6	90038132	4	1.946431
rs282117	GABRR2	6	90038132	1	5.417687
rs282117	GABRR2	6	90038132	6	0.276338
rs282117	GABRR2	6	90038132	3	0.791086
rs282117	GABRR2	6	90038132	7	2.361145
rs10502	UBE2J1	6	90096389	2	3.974014
rs10502	UBE2J1	6	90096389	1	0.121631
SNP6-90397167	ANKRD6	6	90397167	1	4.495124
SNP6-90429295	MDN1	6	90429295	1	3.383485
rs9353689	MDN1	6	90447164	3	0.019685
rs9353689	MDN1	6	90447164	1	3.968876
rs34766278	MDN1	6	90453853	1	4.376006
rs4053618	MDN1	6	90516175	1	3.68176
rs4053618	MDN1	6	90516175	5	2.245904
rs1036853	MDN1	6	90556223	1	4.001228
rs292245	LOC644269	6	90652767	5	0.094737
rs17513276	BACH2	6	90694146	7	1.038689
rs17513276	BACH2	6	90694146	6	2.997537
rs17513276	BACH2	6	90694146	3	1.344113
rs1065272	BACH2	6	90695274	6	0.734256
rs1065272	BACH2	6	90695274	5	0.057219
rs1055403	BACH2	6	90695684	5	1.184388
rs17765013	BACH2	6	90698572	3	1.261112
rs17765013	BACH2	6	90698572	6	4.420907
rs17765013	BACH2	6	90698572	2	2.865944
rs9451298	BACH2	6	90717040	1	1.40713
rs9451298	BACH2	6	90717040	4	0.12529
rs1504215	BACH2	6	91062948	1	2.912495
rs2172710	MAP3K7	6	91282458	5	5.766475
rs2278106	EPHA7	6	94176940	8	1.232449
rs9387522	MANEA	6	96161656	1	4.174829
rs9387522	MANEA	6	96161656	3	0.056042
rs4078339	FUT9	6	96759092	7	0.934842
rs3934926	FUT9	6	96759929	7	0.079242

rs4275065	FUT9	6	96760199	7	1.336615
rs2273622	FHL5	6	97165288	7	2.918528
rs392127	GPR63	6	97354287	5	5.779983
rs392127	GPR63	6	97354287	1	7.501132
rs392127	GPR63	6	97354287	3	0.682244
rs35143662	KLHL32	6	97521670	5	0.433721
rs221527	C6orf168	6	99878261	6	0.266191
rs221527	C6orf168	6	99878261	8	0.726679
rs221527	C6orf168	6	99878261	7	1.833909
rs9376137	COQ3	6	99948777	5	0.865024
rs9376137	COQ3	6	99948777	1	4.511959
rs6915401	USP45	6	99987560	7	0.358451
rs13208607	USP45	6	99990236	7	0.914924
rs1323717	USP45	6	100069455	8	0.512451
rs3734346	PRDM13	6	100169487	8	1.71628
rs3734346	PRDM13	6	100169487	5	1.433669
rs6941007	LOC728012	6	100548632	6	2.370199
rs6941007	LOC728012	6	100548632	3	2.041941
rs6941007	LOC728012	6	100548632	1	2.437414
rs6941007	LOC728012	6	100548632	5	3.478802
rs985937	LOC442238	6	100647908	8	4.536067
rs985937	LOC442238	6	100647908	5	3.98364
rs985937	LOC442238	6	100647908	2	3.636825
rs985937	LOC442238	6	100647908	7	3.426714
rs985937	LOC442238	6	100647908	1	5.086316
rs13219623	LOC442239	6	100691055	3	1.332252
rs592436	LOC728098	6	101444875	1	4.334011
rs592436	LOC728098	6	101444875	6	3.664332
rs592436	LOC728098	6	101444875	4	3.120011
rs592436	LOC728098	6	101444875	3	4.5724
rs1890227	BVES	6	105651496	7	2.774318
rs1190281	POPDC3	6	105712558	8	1.750241
rs1190281	POPDC3	6	105712558	2	3.617429
rs1190282	POPDC3	6	105712685	8	5.616728
rs1149311	LOC728010	6	105857103	3	0.487652
rs1149311	LOC728010	6	105857103	7	0.132699
rs9486069	PREP	6	105928142	8	4.547241
rs9486069	PREP	6	105928142	7	0.912953
rs811925	PRDM1	6	106654065	2	1.43163
rs811925	PRDM1	6	106654065	7	2.145829
rs811925	PRDM1	6	106654065	3	0.16389
rs1010273	PRDM1	6	106661718	2	1.370748
rs1340623	AIM1	6	107066692	7	8.338721
rs1340623	AIM1	6	107066692	3	0.360436
SNP6-10709805	AIM1	6	107098054	7	0.251282
rs3101493	QRSL1	6	107222087	6	1.742
rs3101493	QRSL1	6	107222087	7	0.632657

rs3101493	QRSL1	6	107222087	8	3.707438
rs3101493	QRSL1	6	107222087	5	4.876743
rs3101493	QRSL1	6	107222087	2	1.759077
rs3734748	SOBP	6	108086368	7	1.245439
rs3734748	SOBP	6	108086368	1	0.648795
rs10554	SOBP	6	108087099	7	0.258963
rs10554	SOBP	6	108087099	1	0.012695
rs11751218	SCML4	6	108148494	3	2.391231
rs11751218	SCML4	6	108148494	1	0.005161
rs17282829	LOC642741	6	108432317	6	2.588567
rs17282829	LOC642741	6	108432317	4	2.255567
rs17282829	LOC642741	6	108432317	5	1.9835
rs17282829	LOC642741	6	108432317	1	2.056775
SNP6-10878467(	LACE1	6	108784670	4	0.678548
rs9386758	ARMC2	6	109304072	1	5.360281
rs11153136	ARMC2	6	109340418	1	3.864264
rs3734649	ARMC2	6	109401445	7	0.38924
rs16822	SESN1	6	109414403	7	0.874897
rs949882	FLJ37396	6	109692755	4	1.091512
rs949882	FLJ37396	6	109692755	1	0.935273
rs949882	FLJ37396	6	109692755	7	0.847511
rs949882	FLJ37396	6	109692755	8	0.130554
rs949882	FLJ37396	6	109692755	2	0.044918
rs949882	FLJ37396	6	109692755	3	1.083175
rs9487048	C6orf184	6	109722296	7	0.797797
rs9487048	C6orf184	6	109722296	3	0.857748
rs9487048	C6orf184	6	109722296	4	0.13951
rs9400272	C6orf184	6	109723455	7	0.820389
rs9400272	C6orf184	6	109723455	3	0.173024
rs6568580	FLJ42177	6	109955796	1	0.075695
rs6568580	FLJ42177	6	109955796	2	1.72739
rs4354185	GPR6	6	110408354	7	0.552646
rs4354185	GPR6	6	110408354	4	0.646834
rs12210538	SLC22A16	6	110866701	6	3.237631
rs12210538	SLC22A16	6	110866701	3	0.344
rs12210538	SLC22A16	6	110866701	4	1.306582
rs12210538	SLC22A16	6	110866701	5	1.366557
rs723685	SLC22A16	6	110870568	2	2.971709
rs723685	SLC22A16	6	110870568	8	1.447312
rs714368	SLC22A16	6	110884821	3	0.476581
rs714368	SLC22A16	6	110884821	5	1.708301
rs1775125	LOC441166	6	111286311	2	0.881726
rs1775125	LOC441166	6	111286311	3	3.705132
rs1775125	LOC441166	6	111286311	4	4.857459
rs706956	LOC442244	6	111351509	3	2.088468
rs3204953	REV3L	6	111735319	5	0.366358
rs3218600	REV3L	6	111802592	1	4.881983

rs3218591	REV3L	6	111802822	5	0.399219
SNP6-112527208	LOC619208	6	112527208	6	6.38655
rs2213842	LOC619208	6	112530503	5	0.749835
rs2213842	LOC619208	6	112530503	4	0.998202
rs1050353	LAMA4	6	112542605	3	0.052426
rs11757455	LAMA4	6	112629545	2	5.863255
rs352091	FLJ34503	6	114348919	2	0.423063
rs352091	FLJ34503	6	114348919	1	0.530693
rs17791808	FLJ34503	6	114349112	1	2.259529
rs17791808	FLJ34503	6	114349112	5	2.374743
rs2282237	HS3ST5	6	114485663	3	0.718456
rs2282237	HS3ST5	6	114485663	1	1.20078
rs2105171	FRK	6	116369550	7	0.129703
rs2105171	FRK	6	116369550	3	0.276017
rs2105171	FRK	6	116369550	4	3.025273
rs1931895	TSPYL4	6	116679664	8	0.21126
rs1931895	TSPYL4	6	116679664	2	6.000155
rs2232470	TSPYL4	6	116681776	7	0.615779
rs2232470	TSPYL4	6	116681776	8	0.372521
rs2232470	TSPYL4	6	116681776	3	0.402466
rs2232470	TSPYL4	6	116681776	5	2.207518
rs3749895	TSPYL1	6	116707467	1	3.739313
rs3749895	TSPYL1	6	116707467	5	7.022563
rs3749895	TSPYL1	6	116707467	4	3.413765
SNP6-116827207	DSE	6	116827207	8	0.479682
SNP6-116891490	FAM26F	6	116891490	1	5.342959
SNP6-116891543	FAM26F	6	116891543	4	2.343848
SNP6-116891543	FAM26F	6	116891543	8	0.167597
SNP6-116986431	FAM26D	6	116986431	6	1.324518
rs6927567	RSHL3	6	117057427	1	0.591864
rs6927567	RSHL3	6	117057427	2	0.715912
rs6927567	RSHL3	6	117057427	8	0.246283
rs784133	RSHL3	6	117057526	3	0.044363
rs784133	RSHL3	6	117057526	6	0.062899
rs9488991	RSHL3	6	117058371	8	0.939166
rs6925922	RSHL3	6	117060762	1	0.427899
rs6925922	RSHL3	6	117060762	8	0.018
rs4946188	C6orf113	6	117079875	6	3.087348
rs4946188	C6orf113	6	117079875	1	1.007706
rs6901250	GPRC6A	6	117220718	3	0.367997
rs6924002	GPRC6A	6	117220916	1	2.990825
rs6901971	GPRC6A	6	117221096	3	0.577802
rs619203	ROS1	6	117728877	4	0.209888
rs619203	ROS1	6	117728877	8	6.248998
rs1998206	ROS1	6	117832141	3	0.114637
rs1045855	GOPC	6	117988902	4	7.271172
rs3752847	GOPC	6	118030372	7	3.343633

rs3734345	SLC35F1	6	118743735	1	0.366374
SNP6-118993654	C6orf204	6	118993654	3	0.389203
SNP6-118993654	C6orf204	6	118993654	5	0.317003
SNP6-118993654	C6orf204	6	118993654	8	5.417688
SNP6-118993996	C6orf204	6	118993996	5	4.446191
SNP6-118993996	C6orf204	6	118993996	7	1.638002
rs1885125	MCM9	6	119297778	2	2.778321
rs1885125	MCM9	6	119297778	4	2.830504
rs1046226	MAN1A1	6	119541265	1	6.440079
rs1046226	MAN1A1	6	119541265	7	0.304038
rs1046226	MAN1A1	6	119541265	3	1.101686
rs1330107	LOC728727	6	120207436	2	3.539308
rs1330107	LOC728727	6	120207436	7	1.611621
rs1330107	LOC728727	6	120207436	3	0.26686
rs7745023	C6orf170	6	121619069	6	2.154916
rs7745023	C6orf170	6	121619069	4	9.038004
rs7745023	C6orf170	6	121619069	1	4.888471
rs7745023	C6orf170	6	121619069	5	0.443813
rs7767455	C6orf170	6	121684550	2	5.77323
rs7767455	C6orf170	6	121684550	7	1.881099
rs7767455	C6orf170	6	121684550	1	0.880505
rs6926219	HSF2	6	122762505	1	1.247767
rs13371	SERINC1	6	122806725	1	4.197452
rs13371	SERINC1	6	122806725	6	8.203828
rs1132643	PKIB	6	123088715	3	0.631495
rs1132643	PKIB	6	123088715	4	5.197348
rs1132643	PKIB	6	123088715	2	1.765574
rs1132643	PKIB	6	123088715	5	0.0105
rs1132643	PKIB	6	123088715	6	2.004395
rs28385609	SMPDL3A	6	123164163	1	3.458821
rs2295005	HINT3	6	126319923	5	3.303276
rs2295005	HINT3	6	126319923	7	1.956974
rs2295005	HINT3	6	126319923	3	0.006249
rs2295005	HINT3	6	126319923	2	5.286018
rs2295005	HINT3	6	126319923	8	7.700305
rs9388464	HINT3	6	126340957	3	0.272115
rs9388464	HINT3	6	126340957	5	0.101475
rs1892172	RSPO3	6	127518209	3	1.092101
rs3734446	KIAA0408	6	127804059	1	0.900627
rs2326593	KIAA0408	6	127805778	1	1.988116
rs2326593	KIAA0408	6	127805778	3	0.634255
rs2875982	KIAA0408	6	127805834	3	1.183553
rs2875982	KIAA0408	6	127805834	5	1.834557
rs17205908	KIAA0408	6	127805998	1	0.860779
rs6902288	KIAA0408	6	127810455	1	2.307176
rs6902288	KIAA0408	6	127810455	3	0.463311
rs3734447	C6orf174	6	127813145	3	0.473025

rs3734447	C6orf174	6	127813145	1	2.381582
rs1140366	LAMA2	6	129412799	7	0.595521
rs3749877	LAMA2	6	129764082	7	0.768482
rs3749877	LAMA2	6	129764082	8	0.260177
rs3749878	LAMA2	6	129764118	7	1.141351
rs3749878	LAMA2	6	129764118	5	0.86612
rs2297738	LAMA2	6	129803805	1	5.29519
rs2229850	LAMA2	6	129849407	7	1.834474
rs2229850	LAMA2	6	129849407	3	0.517081
rs2229850	LAMA2	6	129849407	2	0.780233
rs1049476	LAMA2	6	129879303	3	0.313852
rs12197456	ARHGAP18	6	129946942	7	0.587377
rs12197456	ARHGAP18	6	129946942	5	0.028969
rs9375666	C6orf191	6	130194145	5	0.710378
rs7758540	C6orf191	6	130194172	6	1.700152
rs7758540	C6orf191	6	130194172	5	0.624847
rs7776426	C6orf191	6	130194213	5	1.342088
rs9492393	C6orf191	6	130196360	1	2.146176
rs1055374	SAMD3	6	130507242	4	0.224762
rs1055374	SAMD3	6	130507242	5	0.688606
rs3813360	TMEM200A	6	130803491	8	4.301595
rs3813359	TMEM200A	6	130803650	8	3.174929
rs3813359	TMEM200A	6	130803650	1	2.907218
rs4895896	LOC285733	6	131190024	5	0.354778
rs9402288	LOC285733	6	131190075	6	0.442837
rs9402288	LOC285733	6	131190075	1	3.333019
rs9402289	LOC285733	6	131190179	1	2.87915
rs4895897	LOC285733	6	131190234	8	0.945039
rs4895897	LOC285733	6	131190234	5	0.491192
rs942	LOC285733	6	131197919	1	5.172687
rs942	LOC285733	6	131197919	5	0.598486
rs714709	LOC285733	6	131199232	2	2.473947
SNP6-13122099	EPB41L2	6	131220995	3	0.054669
rs17059736	EPB41L2	6	131232531	1	5.573645
rs2781667	MED23	6	131936837	4	0.644141
rs2781667	MED23	6	131936837	5	4.669932
rs17601580	ENPP3	6	132103113	2	3.949115
rs1044548	ENPP1	6	132253456	6	0.32695
rs1044558	ENPP1	6	132253507	6	4.434198
rs1044558	ENPP1	6	132253507	8	2.835834
rs553	ENPP1	6	132253733	8	0.480615
rs553	ENPP1	6	132253733	6	0.341789
rs8192620	TAAR1	6	133007972	1	3.598375
rs8192620	TAAR1	6	133007972	7	4.073807
SNP6-13308759	VNN3	6	133087595	5	1.091623
SNP6-13308759	VNN3	6	133087595	7	0.050523
rs1883613	VNN2	6	133118756	5	0.043114

rs1883613	VNN2	6	133118756	6	0.140278
rs11154699	RPS12	6	133177579	5	0.20585
rs11154699	RPS12	6	133177579	4	0.648846
rs11154699	RPS12	6	133177579	7	3.260375
rs11154699	RPS12	6	133177579	8	2.846545
rs9389034	SNORD100	6	133179703	7	0.58677
rs9389034	SNORD100	6	133179703	5	0.088965
rs9493627	EYA4	6	133831421	7	0.083724
rs9493627	EYA4	6	133831421	3	0.321858
rs3734279	EYA4	6	133891659	2	2.554869
rs3734279	EYA4	6	133891659	5	2.396868
rs3734279	EYA4	6	133891659	4	2.137047
rs3734279	EYA4	6	133891659	1	0.346192
rs12190287	TCF21	6	134256218	5	0.279151
rs35776966	SLC2A12	6	134350418	8	0.728693
rs35776966	SLC2A12	6	134350418	5	1.269594
rs1385066	SLC2A12	6	134352567	5	0.756842
rs17063228	SLC2A12	6	134353032	5	0.183552
rs7739722	HBS1L	6	135323918	1	3.381698
rs13196486	HBS1L	6	135325567	1	4.610268
rs13196486	HBS1L	6	135325567	4	2.192092
rs9399129	HBS1L	6	135326841	4	0.921432
rs4594969	HBS1L	6	135327279	1	4.506667
rs4594969	HBS1L	6	135327279	4	0.436912
rs1052502	AHI1	6	135648258	2	4.486923
rs35433555	AHI1	6	135809975	5	0.08219
rs1883818	GAPDHL19	6	135982174	6	0.93325
rs1883818	GAPDHL19	6	135982174	8	4.355521
rs1681	MAP7	6	136705641	6	0.037135
rs2076260	MAP3K5	6	137019261	5	0.299988
rs2076260	MAP3K5	6	137019261	4	0.628053
rs2076260	MAP3K5	6	137019261	3	0.629101
rs9321567	MAP3K5	6	137067959	1	2.487382
rs9321567	MAP3K5	6	137067959	3	0.526417
rs202566	IL22RA2	6	137506767	1	0.658152
rs28366	IL22RA2	6	137507780	6	3.33092
rs28366	IL22RA2	6	137507780	1	0.232146
rs28366	IL22RA2	6	137507780	2	1.477772
rs276466	IL22RA2	6	137508307	3	0.898023
rs276466	IL22RA2	6	137508307	4	3.302536
rs6901613	LOC442263	6	138069355	7	0.540727
rs6901613	LOC442263	6	138069355	2	1.203893
rs6901613	LOC442263	6	138069355	1	1.812097
rs6901613	LOC442263	6	138069355	5	2.392268
rs648802	PERP	6	138455026	2	5.01894
rs648802	PERP	6	138455026	8	0.841004
rs648802	PERP	6	138455026	4	0.57427

rs9402958	PBOV1	6	138579125	6	0.531959
rs9402959	PBOV1	6	138579170	6	2.077618
rs3813338	PBOV1	6	138579552	6	3.016813
rs11758517	PBOV1	6	138581249	6	0.6799
rs203145	KIAA1244	6	138656841	4	0.382728
rs203145	KIAA1244	6	138656841	7	0.080118
rs4598095	NHSL1	6	139065005	3	0.000793
rs1044418	REPS1	6	139271565	8	7.117168
rs3173082	HECA	6	139529529	6	3.674166
rs3173082	HECA	6	139529529	5	0.30576
rs3173082	HECA	6	139529529	8	1.530191
rs3777668	HECA	6	139540868	8	0.76448
rs3777668	HECA	6	139540868	7	0.260684
rs3777668	HECA	6	139540868	5	0.319057
rs3777668	HECA	6	139540868	6	3.975225
rs17068451	TXLNB	6	139618429	6	0.342763
rs1131400	CITED2	6	139736754	8	3.966541
rs1131400	CITED2	6	139736754	4	8.565777
rs1131400	CITED2	6	139736754	7	0.553681
rs1131400	CITED2	6	139736754	3	0.408497
rs2143390	GPR126	6	142744830	6	0.456602
rs2143390	GPR126	6	142744830	4	0.465654
rs2294773	GPR126	6	142807098	7	1.068795
rs2294773	GPR126	6	142807098	4	0.014239
rs2294774	GPR126	6	142807158	4	5.866317
rs6940880	LOC729320	6	143302293	8	2.664971
rs1138410	ADAT2	6	143788776	1	3.268526
rs1138410	ADAT2	6	143788776	5	0.241099
rs1138410	ADAT2	6	143788776	8	1.021354
rs1061220	ADAT2	6	143788963	5	0.426487
rs1061220	ADAT2	6	143788963	4	2.407993
rs6918444	C6orf94	6	144227107	3	0.338744
rs6918444	C6orf94	6	144227107	5	0.743663
rs6918444	C6orf94	6	144227107	1	0.700381
rs1049846	PLAGL1	6	144303173	3	4.38029
rs1049846	PLAGL1	6	144303173	4	0.864555
rs1049846	PLAGL1	6	144303173	5	4.16614
SNP6-144853012	UTRN	6	144853012	3	0.439324
rs12204734	UTRN	6	144893894	3	0.161968
rs4305737	UTRN	6	145093287	1	0.549159
rs399516	LOC645749	6	145852542	3	0.638695
rs2235481	EPM2A	6	146098541	8	0.367199
rs2235481	EPM2A	6	146098541	7	0.361841
rs2235481	EPM2A	6	146098541	3	0.341989
rs2235481	EPM2A	6	146098541	4	0.503183
rs3811102	FBXO30	6	146167486	3	0.739022
rs3811102	FBXO30	6	146167486	5	0.361487

rs3811102	FBXO30	6	146167486	7	6.835349
rs3811102	FBXO30	6	146167486	4	0.632616
rs3811102	FBXO30	6	146167486	8	0.074621
rs3811102	FBXO30	6	146167486	6	0.138722
rs2942	GRM1	6	146796833	1	0.131062
rs2942	GRM1	6	146796833	5	1.72035
rs6923492	GRM1	6	146797017	4	3.266114
rs702346	SAMD5	6	147928911	4	1.602553
rs702347	SAMD5	6	147929262	4	1.081155
rs702347	SAMD5	6	147929262	3	2.462725
rs702347	SAMD5	6	147929262	8	0.570514
rs702347	SAMD5	6	147929262	7	0.682211
rs702349	SAMD5	6	147929715	1	2.704849
rs702349	SAMD5	6	147929715	5	2.440817
rs702349	SAMD5	6	147929715	4	0.594317
rs702349	SAMD5	6	147929715	8	4.518041
rs702349	SAMD5	6	147929715	7	0.423338
rs702350	SAMD5	6	147930187	8	2.552652
rs702351	SAMD5	6	147930620	4	0.48433
rs702351	SAMD5	6	147930620	1	1.219561
rs2294787	SASH1	6	148896677	6	0.361552
rs7896	MAP3K7IP2	6	149773169	2	5.282527
rs7896	MAP3K7IP2	6	149773169	5	0.857177
rs7896	MAP3K7IP2	6	149773169	4	3.815791
rs2744434	MAP3K7IP2	6	149773597	2	3.225476
rs7747948	ZC3H12D	6	149837215	5	1.294159
rs10872646	NUP43	6	150109368	5	0.506919
rs10872646	NUP43	6	150109368	1	0.725853
rs10457852	LOC442267	6	150243413	4	1.355159
rs10457852	LOC442267	6	150243413	6	1.504673
rs10457852	LOC442267	6	150243413	2	4.624968
rs10457852	LOC442267	6	150243413	5	0.182591
rs4870078	LOC442267	6	150243505	4	0.388999
rs4870078	LOC442267	6	150243505	2	1.483895
rs4870078	LOC442267	6	150243505	6	0.357502
rs4870078	LOC442267	6	150243505	5	1.170112
rs9397053	LOC442267	6	150243726	1	0.466046
rs9397053	LOC442267	6	150243726	4	0.383766
rs9397053	LOC442267	6	150243726	8	1.887048
rs2342767	RAET1E	6	150251410	5	0.532276
rs2342767	RAET1E	6	150251410	2	0.244962
rs2342767	RAET1E	6	150251410	1	0.526869
rs2342767	RAET1E	6	150251410	6	2.796233
rs9371533	RAET1E	6	150252374	7	3.241453
rs9371533	RAET1E	6	150252374	1	0.792346
rs9371533	RAET1E	6	150252374	6	0.908601
rs9371533	RAET1E	6	150252374	5	1.063609

rs9383921	RAET1E	6	150252378	1	1.062301
rs9383921	RAET1E	6	150252378	6	0.797978
rs9383921	RAET1E	6	150252378	2	1.314887
rs9383921	RAET1E	6	150252378	5	0.840554
rs2151910	RAET1E	6	150252816	8	6.799639
rs2151910	RAET1E	6	150252816	5	0.452817
rs9383583	RAET1E	6	150253696	1	5.067795
rs9383583	RAET1E	6	150253696	4	1.185639
rs9383583	RAET1E	6	150253696	8	5.657567
rs2076286	IYD	6	150761073	6	0.123877
rs2076285	IYD	6	150761097	6	0.543503
rs2076284	IYD	6	150761306	6	0.215621
rs675804	IYD	6	150761403	6	0.000619
rs675804	IYD	6	150761403	7	0.895561
rs675804	IYD	6	150761403	1	0.709046
rs675804	IYD	6	150761403	8	0.145886
rs672605	IYD	6	150762132	1	1.967898
rs672605	IYD	6	150762132	7	1.160262
rs9322260	IYD	6	150762187	1	0.112269
rs9322260	IYD	6	150762187	4	0.607839
rs9322260	IYD	6	150762187	6	0.961229
rs9322260	IYD	6	150762187	7	0.237374
rs601223	IYD	6	150762264	1	0.036119
rs601223	IYD	6	150762264	7	0.327968
rs660153	IYD	6	150762601	7	0.254682
rs660153	IYD	6	150762601	1	1.661694
rs587713	IYD	6	150762922	6	0.639374
rs657972	IYD	6	150763094	5	1.104255
rs657972	IYD	6	150763094	1	0.524512
rs657972	IYD	6	150763094	7	0.65193
rs657972	IYD	6	150763094	6	1.906041
rs6911086	IYD	6	150763138	6	0.927458
rs6911086	IYD	6	150763138	5	1.326882
rs6933986	IYD	6	150763808	5	0.041389
rs11758050	IYD	6	150763903	4	0.268042
rs11758050	IYD	6	150763903	7	1.112735
rs11758050	IYD	6	150763903	5	0.354141
rs7763368	IYD	6	150765309	5	0.201996
rs652042	IYD	6	150765757	8	2.911161
rs3734737	IYD	6	150766546	2	1.929545
rs3734737	IYD	6	150766546	7	0.806386
rs3734737	IYD	6	150766546	5	3.59436
rs3734737	IYD	6	150766546	4	0.230772
rs956440	IYD	6	150766971	7	0.218501
rs803413	RP1-44A20.4	6	151190770	4	0.056823
rs803413	RP1-44A20.4	6	151190770	7	0.402538
rs17080414	PLEKHG1	6	151206162	8	4.587243

rs9397027	PLEKHG1	6	151206457	3	0.316532
rs2294792	AKAP12	6	151668727	3	1.101382
rs2294792	AKAP12	6	151668727	4	0.579922
rs2294792	AKAP12	6	151668727	2	2.070489
rs2294792	AKAP12	6	151668727	8	2.231675
rs2294792	AKAP12	6	151668727	5	0.028452
rs3734799	AKAP12	6	151711865	2	5.529771
rs900654	AKAP12	6	151712590	2	2.506756
rs900654	AKAP12	6	151712590	8	3.924125
SNP6-15171397	AKAP12	6	151713978	7	5.757814
SNP6-15171397	AKAP12	6	151713978	2	1.248719
rs9478198	AKAP12	6	151715106	5	0.367344
rs12201388	AKAP12	6	151715282	2	2.056394
rs12201388	AKAP12	6	151715282	7	0.667216
SNP6-15171596	AKAP12	6	151715963	2	3.783418
SNP6-15171596	AKAP12	6	151715963	7	0.105121
rs1065310	RMND1	6	151767993	8	6.390764
rs1065310	RMND1	6	151767993	4	4.02969
rs3736175	RMND1	6	151814857	1	3.696655
rs13201167	C6orf211	6	151815197	4	0.66626
rs13201167	C6orf211	6	151815197	8	1.336213
rs13201167	C6orf211	6	151815197	1	0.530863
rs13201167	C6orf211	6	151815197	7	0.344048
rs13201167	C6orf211	6	151815197	2	0.745769
rs17081470	C6orf97	6	151955958	7	1.363786
rs17081470	C6orf97	6	151955958	5	0.720825
rs6929137	C6orf97	6	151978370	6	0.583692
rs6929137	C6orf97	6	151978370	3	0.93094
rs6929137	C6orf97	6	151978370	5	1.35922
rs6929137	C6orf97	6	151978370	7	1.190866
rs6932260	C6orf97	6	151981253	3	1.664458
rs6932260	C6orf97	6	151981253	6	4.694445
rs6932260	C6orf97	6	151981253	2	2.872073
rs6932260	C6orf97	6	151981253	1	2.714201
rs6904261	C6orf97	6	151981320	7	4.814558
rs6904261	C6orf97	6	151981320	5	0.288846
rs6904261	C6orf97	6	151981320	6	1.101802
rs6904261	C6orf97	6	151981320	3	0.671217
rs6932603	C6orf97	6	151981463	6	0.295854
rs6932603	C6orf97	6	151981463	1	0.364046
rs6932603	C6orf97	6	151981463	3	0.691218
rs6932603	C6orf97	6	151981463	2	5.604427
SNP6-15198156	C6orf97	6	151981566	3	0.126351
SNP6-15198156	C6orf97	6	151981566	1	1.145814
SNP6-15198271	C6orf97	6	151982713	7	1.183377
rs3734806	C6orf97	6	151983154	5	1.026133
rs3734806	C6orf97	6	151983154	7	0.937657

rs2077647	ESR1	6	152170770	5	3.517146
rs2077647	ESR1	6	152170770	4	2.40123
rs2228480	ESR1	6	152461788	2	3.048132
rs2228480	ESR1	6	152461788	4	0.700944
rs2228480	ESR1	6	152461788	3	0.739585
rs3798577	ESR1	6	152462823	6	1.977944
rs3798577	ESR1	6	152462823	2	4.074928
rs3798577	ESR1	6	152462823	4	0.149706
rs3798577	ESR1	6	152462823	3	0.43616
rs3798577	ESR1	6	152462823	5	1.428467
rs3798758	ESR1	6	152463547	5	3.513143
rs3798758	ESR1	6	152463547	8	1.706784
rs2295190	SYNE1	6	152485437	4	0.626396
rs2295190	SYNE1	6	152485437	5	0.140036
SNP6-15249498	SYNE1	6	152494984	3	0.164265
rs2747662	SYNE1	6	152508367	5	1.175151
rs2747662	SYNE1	6	152508367	4	0.64708
rs2747662	SYNE1	6	152508367	1	0.7005
rs2747662	SYNE1	6	152508367	3	0.292098
rs2252755	SYNE1	6	152510881	1	0.317801
rs2252755	SYNE1	6	152510881	3	0.349751
rs2252748	SYNE1	6	152511024	7	0.848612
rs2252748	SYNE1	6	152511024	5	0.634763
rs2252748	SYNE1	6	152511024	4	1.301128
rs2252748	SYNE1	6	152511024	1	0.700497
rs2252748	SYNE1	6	152511024	3	0.079268
rs12664753	SYNE1	6	152656893	2	3.167848
rs12664753	SYNE1	6	152656893	5	0.436768
rs12664753	SYNE1	6	152656893	7	0.508893
rs12664753	SYNE1	6	152656893	8	1.031567
rs2306916	SYNE1	6	152689374	4	6.360752
rs2306916	SYNE1	6	152689374	1	5.494886
rs2306916	SYNE1	6	152689374	7	0.227611
rs10499268	SYNE1	6	152694292	3	0.897606
rs28385621	SYNE1	6	152699834	3	0.417327
rs4645434	SYNE1	6	152706954	2	2.678715
rs4645434	SYNE1	6	152706954	1	0.1578
rs4645434	SYNE1	6	152706954	4	0.642522
rs4645434	SYNE1	6	152706954	6	1.704514
SNP6-15271754	SYNE1	6	152717547	1	0.539165
SNP6-15271754	SYNE1	6	152717547	6	0.006989
SNP6-15271754	SYNE1	6	152717547	4	1.107564
rs6913579	SYNE1	6	152735877	6	0.414569
rs6913579	SYNE1	6	152735877	1	0.386708
rs6913579	SYNE1	6	152735877	2	1.791189
rs6913579	SYNE1	6	152735877	4	2.240939
rs688136	VIP	6	153121754	6	4.440028

rs1713	FBXO5	6	153333616	6	4.168344
rs2073260	FBXO5	6	153338234	2	1.650827
rs2073260	FBXO5	6	153338234	4	0.027761
rs2073260	FBXO5	6	153338234	8	0.477236
rs2073260	FBXO5	6	153338234	1	0.488851
rs2073260	FBXO5	6	153338234	3	0.243215
rs2073262	LOC729616	6	153346281	2	3.514232
rs17085103	PIP3-E	6	154517641	7	0.589249
rs9479767	PIP3-E	6	154518220	3	0.075087
rs17277929	PIP3-E	6	154519605	1	1.138434
rs17277929	PIP3-E	6	154519605	5	0.112961
rs2236256	PIP3-E	6	154520132	3	0.185255
rs2236259	PIP3-E	6	154520585	3	0.558779
rs9371823	LOC646274	6	155070602	5	1.053287
rs931312	TIAM2	6	155493044	3	1.784721
rs931312	TIAM2	6	155493044	7	0.902198
rs931312	TIAM2	6	155493044	1	3.104946
rs41284214	TIAM2	6	155528211	1	4.256725
rs41284214	TIAM2	6	155528211	6	6.545592
rs1048584	TIAM2	6	155620291	5	0.021509
rs1048584	TIAM2	6	155620291	3	0.029488
rs1048584	TIAM2	6	155620291	8	7.604137
rs1048587	TIAM2	6	155620376	6	1.99312
rs9480098	CLDN20	6	155627017	4	0.288154
rs9480098	CLDN20	6	155627017	5	0.357353
rs9480098	CLDN20	6	155627017	2	5.831857
rs927718	CLDN20	6	155638593	3	0.91138
rs927718	CLDN20	6	155638593	8	0.702086
rs927718	CLDN20	6	155638593	5	0.874891
rs577	SNX9	6	158285023	7	5.70888
rs577	SNX9	6	158285023	1	3.181752
rs577	SNX9	6	158285023	6	1.001336
rs2296507	SYNJ2	6	158419290	7	0.794012
rs2296507	SYNJ2	6	158419290	5	0.224032
rs2502598	SYNJ2	6	158439177	7	9.018729
rs4478433	TULP4	6	158653875	6	0.33233
rs9456307	TULP4	6	158849430	6	0.955946
rs3749854	TULP4	6	158849477	6	0.473545
rs3749854	TULP4	6	158849477	4	0.127176
rs3749854	TULP4	6	158849477	5	0.238601
rs6880	TULP4	6	158852805	8	0.39082
rs6880	TULP4	6	158852805	3	0.066979
rs6880	TULP4	6	158852805	5	0.234853
rs6880	TULP4	6	158852805	4	0.441006
rs3123101	SYTL3	6	159105551	6	0.026585
rs3123101	SYTL3	6	159105551	4	5.648347
rs3123101	SYTL3	6	159105551	8	0.577811

rs3123101	SYTL3	6	159105551	3	0.961527
rs34582178	RSPH3	6	159327434	4	6.003238
rs34582178	RSPH3	6	159327434	3	1.383274
rs34582178	RSPH3	6	159327434	1	4.887185
rs41267753	RSPH3	6	159327435	7	1.89268
rs4709266	TAGAP	6	159376254	4	0.817781
rs4709266	TAGAP	6	159376254	3	0.400758
rs4709266	TAGAP	6	159376254	7	1.023849
rs2235822	TAGAP	6	159376830	3	0.077452
rs2235822	TAGAP	6	159376830	4	1.279548
rs1738074	TAGAP	6	159385965	4	0.287632
rs1738074	TAGAP	6	159385965	5	0.01797
rs1738074	TAGAP	6	159385965	1	0.560498
rs509648	FNDC1	6	159570966	8	1.536001
SNP6-15957507	FNDC1	6	159575074	4	1.297731
SNP6-15957537	FNDC1	6	159575373	4	2.540841
SNP6-15958086	FNDC1	6	159580869	4	0.303244
rs551392	FNDC1	6	159592409	8	6.246986
rs551392	FNDC1	6	159592409	5	4.453884
rs294899	FNDC1	6	159592445	5	3.650544
rs294899	FNDC1	6	159592445	4	0.234611
rs550443	FNDC1	6	159592532	8	1.96276
rs550443	FNDC1	6	159592532	5	0.589665
rs419009	FNDC1	6	159612586	4	5.074878
rs5746136	SOD2	6	160023074	4	4.326375
rs5746136	SOD2	6	160023074	5	0.142504
rs9347344	ACAT2	6	160118349	5	0.832745
rs3818299	SNORA29	6	160126621	8	6.099087
rs3818299	SNORA29	6	160126621	3	0.852574
rs3818299	SNORA29	6	160126621	5	5.527515
rs1128670	MRPL18	6	160131626	8	5.443382
rs1128670	MRPL18	6	160131626	3	2.900729
rs8191808	IGF2R	6	160389500	7	0.160838
rs614754	IGF2R	6	160425189	8	7.0083
rs1888727	LOC729603	6	160434531	8	0.999511
rs1888727	LOC729603	6	160434531	3	1.177737
rs1888727	LOC729603	6	160434531	4	3.701976
rs1888727	LOC729603	6	160434531	5	1.521024
rs1803989	IGF2R	6	160437471	4	0.991821
rs1803989	IGF2R	6	160437471	2	4.662958
rs1867351	SLC22A1	6	160463113	1	0.732163
rs1867351	SLC22A1	6	160463113	4	0.246118
rs1867351	SLC22A1	6	160463113	3	0.224164
rs1867351	SLC22A1	6	160463113	5	2.290537
rs12208357	SLC22A1	6	160463138	7	0.379327
rs624249	SLC22A2	6	160599390	5	1.57391
rs2292334	SLC22A3	6	160778178	1	2.672586

rs2292334	SLC22A3	6	160778178	5	0.776972
rs3088441	SLC22A3	6	160792599	1	5.003026
rs2076828	SLC22A3	6	160792776	6	0.04151
rs2076828	SLC22A3	6	160792776	4	0.225573
rs2076828	SLC22A3	6	160792776	5	1.129738
rs2076828	SLC22A3	6	160792776	8	0.633545
rs2076828	SLC22A3	6	160792776	3	1.559853
rs14224	PLG	6	161057769	3	0.338477
rs9458136	AGPAT4	6	161471208	3	0.238483
rs9458136	AGPAT4	6	161471208	2	4.612983
rs11759408	AGPAT4	6	161474798	7	1.37093
rs11759408	AGPAT4	6	161474798	5	0.678759
rs3734462	AGPAT4	6	161477652	8	4.56966
rs6934457	RIPPLY2	6	161502525	1	0.570556
rs6934457	RIPPLY2	6	161502525	2	1.213004
rs6934457	RIPPLY2	6	161502525	3	0.712736
rs6934457	RIPPLY2	6	161502525	5	0.24188
rs9306	PACRG	6	163656194	4	0.344927
rs3756879	QKI	6	163910298	4	0.304069
rs3756879	QKI	6	163910298	3	0.077971
rs1511746	PDE10A	6	165721780	6	1.202823
rs220740	PDE10A	6	165752217	1	4.126447
rs9459553	LOC346085	6	166398372	5	0.562893
rs3099270	T	6	166491515	3	2.311252
rs3099270	T	6	166491515	1	2.185036
rs16898752	T	6	166491543	3	2.504067
rs16898752	T	6	166491543	6	3.328946
rs16898752	T	6	166491543	2	2.351457
rs16898752	T	6	166491543	1	2.642891
rs3127328	T	6	166492035	6	3.99092
rs2305089	T	6	166499260	1	0.507089
rs2305089	T	6	166499260	6	0.321804
rs2305089	T	6	166499260	5	0.124196
rs3099266	T	6	166501137	5	0.392816
rs3099266	T	6	166501137	3	0.373367
rs1546854	BRP44L	6	166716423	7	0.414894
rs1546854	BRP44L	6	166716423	5	3.558116
rs2984	RPS6KA2	6	166746011	5	0.519552
rs2984	RPS6KA2	6	166746011	7	1.000589
rs2984	RPS6KA2	6	166746011	6	4.351451
rs2984	RPS6KA2	6	166746011	1	3.34669
rs6911624	LOC645468	6	167034039	5	0.359856
rs6911624	LOC645468	6	167034039	3	1.895436
rs6911624	LOC645468	6	167034039	1	2.800977
rs3817828	LOC645468	6	167036150	6	4.358297
rs3817828	LOC645468	6	167036150	4	1.264854
rs3817828	LOC645468	6	167036150	2	4.520624

rs3817828	LOC645468	6	167036150	8	0.136906
rs3817828	LOC645468	6	167036150	5	3.81434
rs3093007	CCR6	6	167469765	4	0.538638
rs3093007	CCR6	6	167469765	1	2.552467
rs2071171	CCR6	6	167470032	1	3.465876
rs2071171	CCR6	6	167470032	7	1.116174
rs2071171	CCR6	6	167470032	3	1.059698
rs2071171	CCR6	6	167470032	8	0.55613
rs3093006	CCR6	6	167471442	5	2.142008
rs2076005	UNC93A	6	167624934	3	0.586837
rs2076005	UNC93A	6	167624934	1	2.036871
rs909545	TTLL2	6	167674651	3	1.922284
rs909546	TTLL2	6	167674692	3	0.579309
rs909546	TTLL2	6	167674692	7	0.206361
rs909546	TTLL2	6	167674692	6	0.111568
rs909546	TTLL2	6	167674692	2	1.043707
SNP6-16767470(	TTLL2	6	167674700	1	1.97023
SNP6-16767470(	TTLL2	6	167674700	6	0.725444
rs2981978	TTLL2	6	167675423	3	0.726146
rs3213590	MLLT4	6	168058827	5	0.390314
rs9364373	MLLT4	6	168106838	3	7.316446
rs9364373	MLLT4	6	168106838	1	6.027478
rs9364373	MLLT4	6	168106838	7	3.097444
rs1415	MLLT4	6	168107720	3	0.15567
rs1415	MLLT4	6	168107720	1	4.312515
rs1415	MLLT4	6	168107720	7	0.787423
rs1044292	MLLT4	6	168114604	5	0.668104
rs2171982	C6orf54	6	168137269	6	0.705928
rs1132782	THBS2	6	169358348	7	0.752693
rs7382711	THBS2	6	169363461	1	4.315747
rs7382711	THBS2	6	169363461	4	0.680325
rs7382711	THBS2	6	169363461	5	1.173831
rs34987335	THBS2	6	169376821	2	2.664607
rs6941625	WDR27	6	169724852	8	0.29718
rs6941625	WDR27	6	169724852	7	0.21839
rs6941625	WDR27	6	169724852	4	0.245048
rs6941625	WDR27	6	169724852	5	0.588425
rs9478086	WDR27	6	169793974	8	1.380154
rs9478086	WDR27	6	169793974	1	5.019065
rs4716383	C6orf120	6	169844192	5	0.338791
rs13194101	TCTE3	6	169885225	4	1.480303
rs3807067	C6orf122	6	169932125	7	0.674205
rs3807067	C6orf122	6	169932125	2	1.728085
rs3807067	C6orf122	6	169932125	1	0.611696
rs3807067	C6orf122	6	169932125	5	3.384365
rs3734776	DLL1	6	170434870	7	0.339495
rs17860736	FAM120B	6	170457833	8	1.105422

rs17860736	FAM120B	6	170457833	3	0.140406
rs12717	PSMB1	6	170704225	3	0.052879
rs12717	PSMB1	6	170704225	5	0.244342
rs12717	PSMB1	6	170704225	2	4.803895
rs12717	PSMB1	6	170704225	4	0.050188
rs12717	PSMB1	6	170704225	6	0.160394
rs12189695	PDCD2	6	170732968	5	1.158089
rs12189695	PDCD2	6	170732968	6	2.077309
rs4720951	HEATR2	7	776745	8	9.429387
rs4720951	HEATR2	7	776745	1	0.042059
rs4720951	HEATR2	7	776745	2	3.359089
rs4720951	HEATR2	7	776745	4	0.65003
rs4720951	HEATR2	7	776745	3	0.62177
rs3922641	HEATR2	7	781314	3	0.17636
rs3922641	HEATR2	7	781314	1	0.831125
rs3922641	HEATR2	7	781314	8	1.517995
rs3922641	HEATR2	7	781314	2	0.088579
rs9458	HEATR2	7	792020	4	0.313925
rs13171	C7orf20	7	897215	5	4.464716
rs13171	C7orf20	7	897215	4	0.134708
SNP7-906707	CENTA1	7	906707	2	0.573081
rs8591	C7orf50	7	1003325	5	5.850382
rs11761941	GPR146	7	1063709	6	0.759067
rs3808351	GPER	7	1093185	5	0.563083
rs3808351	GPER	7	1093185	1	4.451217
rs3802141	GPER	7	1097882	1	1.734683
rs3802141	GPER	7	1097882	4	0.625381
rs3802141	GPER	7	1097882	2	1.739558
rs3802141	GPER	7	1097882	5	0.888854
SNP7-1099197	GPER	7	1099197	1	5.722961
SNP7-1099197	GPER	7	1099197	6	6.25864
rs1133043	GPER	7	1099831	1	0.39104
rs1133043	GPER	7	1099831	4	5.08356
rs1133043	GPER	7	1099831	2	6.158295
rs1133122	ZFAND2A	7	1159098	1	0.27911
rs3752715	INTS1	7	1509340	3	5.80619
rs1369011	INTS1	7	1510507	3	1.013946
rs1369011	INTS1	7	1510507	7	0.038685
rs1369011	INTS1	7	1510507	2	2.454256
rs3735656	MAFK	7	1547447	7	0.862626
rs3735656	MAFK	7	1547447	1	0.252447
rs3735656	MAFK	7	1547447	2	0.109909
rs3735656	MAFK	7	1547447	4	7.632833
rs3735656	MAFK	7	1547447	3	0.941217
rs3735656	MAFK	7	1547447	6	0.068257
rs35932242	NUDT1	7	2256162	6	7.069684
rs2895	LFNG	7	2535296	6	0.530562

SNP7-2612052	IQCE	7	2612052	5	0.535237
SNP7-2613322	IQCE	7	2613322	2	6.196668
rs3735110	IQCE	7	2616345	5	0.006338
rs3735110	IQCE	7	2616345	3	0.441466
rs3823603	IQCE	7	2616749	5	0.200536
rs3823603	IQCE	7	2616749	3	0.112098
rs13188	TTYH3	7	2669990	2	4.444453
rs798473	AMZ1	7	2706541	8	0.865016
rs7776970	AMZ1	7	2719013	2	2.707928
rs7778736	AMZ1	7	2719458	1	1.213073
rs7778736	AMZ1	7	2719458	3	1.54
rs7778736	AMZ1	7	2719458	5	0.260614
rs4719646	AMZ1	7	2721151	6	0.363327
rs4719646	AMZ1	7	2721151	1	5.247832
rs798531	GNA12	7	2736593	1	3.089275
rs798531	GNA12	7	2736593	6	6.976497
rs798531	GNA12	7	2736593	7	0.30782
rs1621509	CARD11	7	2936206	6	4.854801
rs1621509	CARD11	7	2936206	2	4.597749
rs659182	SDK1	7	4155601	8	0.398665
rs659182	SDK1	7	4155601	2	0.415921
rs659182	SDK1	7	4155601	1	0.240092
rs601424	SDK1	7	4180403	7	1.084657
rs601424	SDK1	7	4180403	8	1.235472
rs671756	SDK1	7	4180466	2	1.531822
rs671756	SDK1	7	4180466	8	0.649127
rs671756	SDK1	7	4180466	6	1.591778
rs671694	SDK1	7	4180501	8	1.576952
rs671694	SDK1	7	4180501	1	1.320548
rs3750019	FOXK1	7	4775204	7	0.320157
rs3750019	FOXK1	7	4775204	1	3.245531
rs17135096	FOXK1	7	4776186	1	4.910369
rs17135121	KIAA0415	7	4790497	7	0.401217
rs17135121	KIAA0415	7	4790497	1	0.297981
rs17135121	KIAA0415	7	4790497	2	1.167952
rs11772411	KIAA0415	7	4791833	2	0.381658
rs11772411	KIAA0415	7	4791833	7	0.594411
rs11772411	KIAA0415	7	4791833	1	0.210217
rs17135128	KIAA0415	7	4793856	2	1.474756
rs17135128	KIAA0415	7	4793856	1	0.23522
rs17135128	KIAA0415	7	4793856	7	2.278904
SNP7-4807839	RADIL	7	4807839	7	0.201275
rs3763384	RADIL	7	4842583	6	0.995186
rs3763384	RADIL	7	4842583	8	3.876706
rs3763384	RADIL	7	4842583	3	0.853848
rs3763384	RADIL	7	4842583	7	0.121781
rs17135247	PAPOLB	7	4866867	8	5.18843

rs6463205	LOC729720	7	4988749	6	2.191641
rs12674330	RBAK	7	5070801	2	3.519152
rs11771682	RBAK	7	5071359	1	5.540017
rs13244634	WIPI2	7	5239990	2	1.754117
rs13244634	WIPI2	7	5239990	8	0.328268
rs13244634	WIPI2	7	5239990	4	1.534851
rs13244634	WIPI2	7	5239990	1	1.280838
rs6950111	SLC29A4	7	5305240	7	0.167782
rs6950111	SLC29A4	7	5305240	5	0.026042
rs6950111	SLC29A4	7	5305240	4	0.940962
rs6950111	SLC29A4	7	5305240	1	2.30604
rs11975313	FBXL18	7	5497480	6	4.118827
rs2228006	PMS2	7	5993301	1	5.227475
rs2228006	PMS2	7	5993301	6	3.462262
SNP7-6159990	USP42	7	6159990	4	0.623792
SNP7-6160277	USP42	7	6160277	1	2.396115
rs17136052	PSCD3	7	6169260	1	5.824203
rs836559	DAGLB	7	6415288	8	6.647189
rs836559	DAGLB	7	6415288	3	0.066034
rs836559	DAGLB	7	6415288	5	1.26011
rs836559	DAGLB	7	6415288	1	0.673946
rs836559	DAGLB	7	6415288	7	0.337968
rs836559	DAGLB	7	6415288	2	6.575769
rs6463598	LOC729834	7	6903863	8	2.286871
rs6463598	LOC729834	7	6903863	3	1.770014
rs1047763	C1GALT1	7	7250094	1	5.226867
rs6952195	COL28A1	7	7480760	5	0.875883
rs6952195	COL28A1	7	7480760	7	0.859996
rs10486176	COL28A1	7	7512216	5	0.656095
rs17169194	RPA3	7	7647222	2	2.855662
rs12112667	RPA3	7	7692110	1	4.200613
rs12112667	RPA3	7	7692110	4	0.647487
rs1057947	ICA1	7	8119504	6	6.78541
SNP7-10988755	PHF14	7	10988755	6	0.033897
SNP7-10988755	PHF14	7	10988755	8	0.304084
SNP7-10988755	PHF14	7	10988755	2	4.580528
SNP7-10988755	PHF14	7	10988755	5	0.678032
rs1432	THSD7A	7	11453464	3	0.150036
rs1432	THSD7A	7	11453464	8	0.040798
rs2285744	THSD7A	7	11476086	7	1.487013
rs2285744	THSD7A	7	11476086	4	1.027811
rs3735502	THSD7A	7	11488067	8	0.8559
rs3735502	THSD7A	7	11488067	4	0.504815
rs3735502	THSD7A	7	11488067	7	0.697145
rs47	THSD7A	7	11547646	1	0.081655
rs47	THSD7A	7	11547646	4	0.41857
rs47	THSD7A	7	11547646	7	0.300347

rs2074603	THSD7A	7	11547659	8	0.357045
rs2074603	THSD7A	7	11547659	7	0.860357
rs2074603	THSD7A	7	11547659	1	1.283667
rs2074603	THSD7A	7	11547659	4	0.34252
rs2043539	TMEM106B	7	12220405	7	1.314932
rs2043539	TMEM106B	7	12220405	1	5.782124
rs2043539	TMEM106B	7	12220405	5	0.55961
rs2043539	TMEM106B	7	12220405	3	0.036172
rs6967385	FLJ14712	7	12357844	7	0.397239
rs6967385	FLJ14712	7	12357844	2	7.340579
rs6967241	NULL	7	12385633	1	0.150554
rs17165995	NULL	7	12399872	7	7.069258
rs11275	LOC389465	7	12477837	2	3.630294
rs2033604	SCIN	7	12659665	1	0.39177
rs2033604	SCIN	7	12659665	6	0.575799
rs2033604	SCIN	7	12659665	7	1.173242
rs2033604	SCIN	7	12659665	3	0.952353
rs2033604	SCIN	7	12659665	5	0.591528
rs2280633	ARL4A	7	12694351	7	5.66592
rs2280633	ARL4A	7	12694351	3	0.234978
rs2280633	ARL4A	7	12694351	6	0.036304
rs722661	LOC646161	7	13878170	1	0.241934
rs4721286	ETV1	7	13898507	7	0.977382
SNP7-13995233	ETV1	7	13995233	4	0.224452
SNP7-13995233	ETV1	7	13995233	7	0.1865
SNP7-13995233	ETV1	7	13995233	5	0.561777
SNP7-13995233	ETV1	7	13995233	3	0.626431
rs17167943	DGKB	7	14182629	8	4.727565
rs17167943	DGKB	7	14182629	1	2.84442
rs17167943	DGKB	7	14182629	6	3.697968
rs17167943	DGKB	7	14182629	3	2.736407
rs6966154	DGKB	7	14184198	1	0.512308
rs17199	MEOX2	7	15618295	5	1.135873
rs1050290	MEOX2	7	15692591	4	1.036685
rs1050290	MEOX2	7	15692591	5	0.941621
rs1050290	MEOX2	7	15692591	3	2.411778
rs1050290	MEOX2	7	15692591	6	1.653172
rs1044984	ANKMY2	7	16606726	2	6.69747
rs1044984	ANKMY2	7	16606726	3	0.150328
rs1044984	ANKMY2	7	16606726	7	6.614339
rs543387	ANKMY2	7	16606883	2	3.013567
rs543387	ANKMY2	7	16606883	8	5.677731
rs15504	AGR2	7	16798062	7	0.78298
rs15504	AGR2	7	16798062	6	2.043193
rs3800961	PRPS1L1	7	18033607	3	3.287036
rs3800961	PRPS1L1	7	18033607	2	2.952551
rs2074633	HDAC9	7	19002445	2	2.679544

rs2526630	HDAC9	7	19003103	2	4.085713
rs2526630	HDAC9	7	19003103	7	0.603563
rs2526630	HDAC9	7	19003103	3	0.172277
rs7812296	HDAC9	7	19003263	2	1.846396
rs7812296	HDAC9	7	19003263	3	0.895777
rs7812296	HDAC9	7	19003263	7	0.293675
rs7776735	HDAC9	7	19003434	3	0.447643
rs2285925	TMEM196	7	19726715	3	0.31129
rs2285927	TMEM196	7	19726958	8	2.737453
SNP7-20166057	7A5	7	20166057	3	0.598336
rs34603556	ABCB5	7	20657572	3	0.479909
rs10254317	ABCB5	7	20734538	4	2.43225
rs6461515	ABCB5	7	20745171	5	0.042351
rs3735440	SP4	7	21517595	4	0.10297
rs3735440	SP4	7	21517595	3	0.750125
rs3735440	SP4	7	21517595	8	0.028881
rs2285942	DNAH11	7	21549442	5	0.973879
rs2285942	DNAH11	7	21549442	2	3.260281
rs2285943	DNAH11	7	21549488	8	1.551086
rs2285943	DNAH11	7	21549488	2	4.82201
rs2285943	DNAH11	7	21549488	1	4.625422
rs10950854	DNAH11	7	21565758	2	6.026049
rs10950854	DNAH11	7	21565758	4	4.040516
rs10950854	DNAH11	7	21565758	8	2.218499
rs10269582	DNAH11	7	21606886	6	2.402913
rs10269582	DNAH11	7	21606886	2	2.88382
rs10269582	DNAH11	7	21606886	1	3.65873
rs10269582	DNAH11	7	21606886	4	2.738168
rs10224537	DNAH11	7	21606930	5	1.511054
rs10224537	DNAH11	7	21606930	7	1.651378
rs10224537	DNAH11	7	21606930	6	1.476397
rs10224537	DNAH11	7	21606930	1	4.482511
rs11768670	DNAH11	7	21732022	3	1.525473
rs11768670	DNAH11	7	21732022	1	5.48777
SNP7-21741894	DNAH11	7	21741894	7	2.306621
rs2072221	DNAH11	7	21741968	1	3.831483
rs2072221	DNAH11	7	21741968	2	4.112882
rs2072221	DNAH11	7	21741968	8	2.334097
rs2072221	DNAH11	7	21741968	4	1.640594
rs9639393	DNAH11	7	21745803	2	1.805629
rs9639393	DNAH11	7	21745803	1	3.774258
rs6965750	DNAH11	7	21790647	7	0.175228
rs6965750	DNAH11	7	21790647	6	4.619467
rs4722067	DNAH11	7	21868091	7	0.950243
rs4722067	DNAH11	7	21868091	8	2.082317
rs4722067	DNAH11	7	21868091	2	2.971998
rs4722067	DNAH11	7	21868091	5	1.772607

rs12537531	DNAH11	7	21898569	2	3.738325
rs12537531	DNAH11	7	21898569	3	0.207365
rs12537531	DNAH11	7	21898569	8	0.3421
rs12537531	DNAH11	7	21898569	1	0.516599
rs12537531	DNAH11	7	21898569	7	1.792616
SNP7-21907348	DNAH11	7	21907348	4	7.975065
SNP7-21907348	DNAH11	7	21907348	7	0.68034
rs2177142	MGC87042	7	22425849	7	0.652934
rs2177142	MGC87042	7	22425849	6	1.716202
rs2177142	MGC87042	7	22425849	4	1.101313
rs4607514	FAM126A	7	22950227	8	7.838402
rs4607514	FAM126A	7	22950227	4	5.927505
rs4607514	FAM126A	7	22950227	3	0.372567
rs4607514	FAM126A	7	22950227	1	0.048645
rs4607514	FAM126A	7	22950227	2	6.18911
rs858246	NUPL2	7	23188017	5	1.669419
rs858246	NUPL2	7	23188017	1	2.114804
rs858238	NUPL2	7	23205658	5	6.672427
rs858238	NUPL2	7	23205658	1	3.52342
rs858238	NUPL2	7	23205658	4	6.241
rs10634	NUPL2	7	23207121	1	3.874741
rs10634	NUPL2	7	23207121	4	0.449451
rs10634	NUPL2	7	23207121	5	0.276296
rs858291	LOC730020	7	23213886	4	0.45656
rs858291	LOC730020	7	23213886	1	1.405784
rs858291	LOC730020	7	23213886	5	0.829095
rs199355	GPNMB	7	23263058	1	1.64558
rs199355	GPNMB	7	23263058	7	0.690959
rs199355	GPNMB	7	23263058	3	1.943075
rs199354	GPNMB	7	23263097	1	4.198612
rs199354	GPNMB	7	23263097	4	2.741906
rs199354	GPNMB	7	23263097	5	0.404906
rs156404	C7orf30	7	23307143	5	1.419256
rs156404	C7orf30	7	23307143	1	4.839563
rs156404	C7orf30	7	23307143	4	0.434907
rs3087888	IGF2BP3	7	23317605	7	0.243939
rs156414	IGF2BP3	7	23323846	7	0.605008
rs156414	IGF2BP3	7	23323846	3	0.521184
rs156414	IGF2BP3	7	23323846	1	0.571612
rs156414	IGF2BP3	7	23323846	2	1.469944
rs4722266	STK31	7	23760554	4	0.988215
rs9785023	NPY	7	24291534	6	1.278832
rs9785023	NPY	7	24291534	1	0.59451
rs9785023	NPY	7	24291534	5	1.37659
rs9785023	NPY	7	24291534	4	4.999098
rs9785023	NPY	7	24291534	3	1.880525
rs12979	DFNA5	7	24704689	5	0.305383

rs12979	DFNA5	7	24704689	8	5.652117
rs17274530	DFNA5	7	24705140	2	5.231887
rs1861525	CYCS	7	25128127	8	1.343643
rs1861525	CYCS	7	25128127	7	1.325455
rs2717885	C7orf31	7	25140857	4	0.840681
rs2717885	C7orf31	7	25140857	1	1.865473
rs2717886	C7orf31	7	25141222	6	0.30072
rs2717886	C7orf31	7	25141222	7	0.591275
rs2717886	C7orf31	7	25141222	4	0.262987
rs2717886	C7orf31	7	25141222	8	0.04073
rs2717886	C7orf31	7	25141222	1	3.086639
rs12671270	LOC285941	7	26652459	8	2.226546
rs12671270	LOC285941	7	26652459	7	0.455516
rs12671270	LOC285941	7	26652459	6	1.442775
rs17323934	SKAP2	7	26870855	2	6.145727
rs17323934	SKAP2	7	26870855	7	0.332814
rs6969780	HOXA3	7	27125661	1	5.155313
rs6969780	HOXA3	7	27125661	7	0.216245
rs6969780	HOXA3	7	27125661	6	4.320147
rs17471888	HOXA4	7	27134538	8	0.319621
rs17500863	HOXA6	7	27152045	8	0.734568
rs17500987	HOXA9	7	27169710	8	5.927074
rs17501090	HOXA11	7	27187979	4	3.162914
rs17501090	HOXA11	7	27187979	1	3.844915
rs17427861	HOXA11	7	27188934	7	0.894789
rs757181	HOXA13	7	27203483	1	1.385598
rs757181	HOXA13	7	27203483	4	1.902868
rs2074399	EVX1	7	27249008	7	1.609869
rs2190305	CREB5	7	28825975	2	0.228988
rs2190305	CREB5	7	28825975	1	0.186163
rs2190305	CREB5	7	28825975	5	0.599501
rs2190305	CREB5	7	28825975	3	0.050624
rs4722856	CREB5	7	28826384	2	2.878683
rs740252	KIAA0644	7	28964290	4	1.344758
rs7313	CPVL	7	29036734	7	1.580473
rs7313	CPVL	7	29036734	6	4.13838
rs7313	CPVL	7	29036734	1	0.826585
rs1052200	CPVL	7	29036845	2	4.765587
rs1052200	CPVL	7	29036845	8	0.518623
rs323182	CPVL	7	29118896	7	2.491321
rs323182	CPVL	7	29118896	6	6.076805
rs323182	CPVL	7	29118896	8	0.644539
rs2011337	CPVL	7	29118941	6	3.64918
rs2011337	CPVL	7	29118941	8	1.410288
rs1059182	CHN2	7	29519196	6	0.597383
rs1059182	CHN2	7	29519196	3	0.758776
rs1059182	CHN2	7	29519196	4	2.888075

rs12611	CHN2	7	29519302	3	0.330802
rs1059185	CHN2	7	29519508	7	1.661983
rs1059185	CHN2	7	29519508	5	3.133101
rs1059185	CHN2	7	29519508	3	0.186079
rs1059185	CHN2	7	29519508	1	4.156144
rs1059185	CHN2	7	29519508	4	3.54949
rs1558634	CHN2	7	29519864	6	0.725562
rs1558634	CHN2	7	29519864	2	5.218897
rs1558634	CHN2	7	29519864	4	0.657538
rs1558634	CHN2	7	29519864	7	1.090331
rs1558634	CHN2	7	29519864	3	0.313249
rs11536448	CHN2	7	29520158	7	1.900293
SNP7-29572813	PRR15	7	29572813	2	5.829196
SNP7-29572813	PRR15	7	29572813	4	0.568462
SNP7-29572813	PRR15	7	29572813	6	5.788099
SNP7-29572813	PRR15	7	29572813	7	0.057701
rs1728643	LOC646762	7	29655440	3	3.325178
rs1728643	LOC646762	7	29655440	4	3.662609
rs4722959	WIPF3	7	29910587	3	0.024326
rs4722959	WIPF3	7	29910587	1	0.788008
rs4722959	WIPF3	7	29910587	2	0.138171
rs10231810	WIPF3	7	29911451	1	0.131566
rs10231810	WIPF3	7	29911451	2	0.552948
rs10231810	WIPF3	7	29911451	3	0.117299
rs12532093	WIPF3	7	29911807	3	0.13348
rs12532093	WIPF3	7	29911807	1	4.031376
rs10264876	WIPF3	7	29912122	1	2.672469
rs10264876	WIPF3	7	29912122	2	2.228565
rs10264876	WIPF3	7	29912122	3	3.020909
rs1049394	SCRN1	7	29927329	7	0.536624
rs1049394	SCRN1	7	29927329	4	0.209831
rs1049394	SCRN1	7	29927329	3	0.329388
rs1049394	SCRN1	7	29927329	1	1.56743
rs2269977	SCRN1	7	29928409	2	3.004192
rs2269977	SCRN1	7	29928409	1	0.246408
rs2269977	SCRN1	7	29928409	3	0.610344
rs2269977	SCRN1	7	29928409	4	0.166645
rs6976789	SCRN1	7	29929459	1	0.018746
rs6976789	SCRN1	7	29929459	2	4.331549
rs6976789	SCRN1	7	29929459	7	0.476454
rs6976789	SCRN1	7	29929459	4	6.487296
rs6976789	SCRN1	7	29929459	3	0.161006
rs17158486	SCRN1	7	29929691	7	0.021265
rs35960711	SCRN1	7	29946996	7	0.016644
rs17150692	FKBP14	7	30032485	7	1.197697
rs3750089	C7orf41	7	30167124	4	1.205765
rs3750089	C7orf41	7	30167124	2	0.678138

rs3750089	C7orf41	7	30167124	1	1.230704
rs3750089	C7orf41	7	30167124	3	1.049256
rs2110394	C7orf41	7	30167938	1	1.003997
rs11156	C7orf41	7	30168188	5	0.718715
rs5743372	NOD1	7	30431560	1	4.765449
rs2075821	NOD1	7	30457836	6	3.19734
rs2075821	NOD1	7	30457836	7	0.581473
rs2075820	NOD1	7	30458762	7	0.885122
rs2075820	NOD1	7	30458762	8	3.922522
rs2075818	NOD1	7	30462907	6	6.298355
rs2075818	NOD1	7	30462907	8	8.767023
rs2075818	NOD1	7	30462907	7	5.528558
rs5743335	NOD1	7	30465354	5	0.388403
rs2906766	NOD1	7	30466100	8	0.2973
rs2906766	NOD1	7	30466100	7	4.997815
rs2906766	NOD1	7	30466100	6	4.954868
rs2906766	NOD1	7	30466100	5	0.44897
rs2302340	INMT	7	30761856	7	1.512421
SNP7-30846938	FLJ22374	7	30846938	3	0.421132
rs28362739	AQP1	7	30929834	1	1.298018
rs11537660	AQP1	7	30930294	1	0.503375
rs11537660	AQP1	7	30930294	2	0.300252
rs1049305	AQP1	7	30930347	5	0.65018
rs11537661	AQP1	7	30930670	1	5.114705
rs7811042	CCDC129	7	31559258	6	0.658951
rs7811042	CCDC129	7	31559258	2	1.833308
rs2286711	CCDC129	7	31561033	6	4.52036
rs2286711	CCDC129	7	31561033	5	3.769848
rs2286711	CCDC129	7	31561033	2	3.100758
rs2159513	CCDC129	7	31659495	4	1.288498
rs2159513	CCDC129	7	31659495	8	2.14866
rs12669290	C7orf16	7	31713774	3	0.145681
rs34185	C7orf16	7	31714559	3	0.939744
rs3213709	PDE1C	7	31815231	8	1.555089
rs3213709	PDE1C	7	31815231	4	1.024243
rs3213709	PDE1C	7	31815231	1	4.157212
rs3213709	PDE1C	7	31815231	7	0.203122
rs17362028	LOC442525	7	32478326	4	0.662049
rs17362028	LOC442525	7	32478326	5	2.250062
rs17362028	LOC442525	7	32478326	1	0.537847
rs17362028	LOC442525	7	32478326	3	0.631072
rs1993050	KIAA0241	7	32579545	5	0.123617
rs11769075	LOC401321	7	32768292	4	0.881832
rs11769075	LOC401321	7	32768292	5	0.245532
rs11769075	LOC401321	7	32768292	1	5.299108
rs1045530	KBTBD2	7	32874660	2	0.300696
rs1045530	KBTBD2	7	32874660	1	0.595826

rs1127607	FKBP9	7	33012569	6	1.208845
rs3750117	NT5C3	7	33027471	7	1.78613
rs3750117	NT5C3	7	33027471	4	0.00056
rs3750117	NT5C3	7	33027471	5	4.949297
rs3750117	NT5C3	7	33027471	1	6.289138
rs6968741	BMPER	7	34159287	7	0.817997
rs11972404	BMPER	7	34160114	8	2.916774
rs887020	NPSR1	7	34664507	5	1.325665
rs17170011	NPSR1	7	34833707	1	0.877815
rs6972158	NPSR1	7	34855707	1	2.298786
rs6972158	NPSR1	7	34855707	7	0.272326
rs6972158	NPSR1	7	34855707	8	2.428493
rs10275028	NPSR1	7	34855747	8	1.829244
rs1127886	DPY19L1	7	34935023	4	0.494296
rs1127886	DPY19L1	7	34935023	6	1.083307
rs1127886	DPY19L1	7	34935023	8	1.097078
rs1127886	DPY19L1	7	34935023	5	0.163629
rs1127886	DPY19L1	7	34935023	7	6.577879
rs1127886	DPY19L1	7	34935023	2	2.070484
rs3779234	HERPUD2	7	35676367	6	1.045517
rs3779234	HERPUD2	7	35676367	3	1.00438
rs3779234	HERPUD2	7	35676367	1	3.898648
rs3779234	HERPUD2	7	35676367	4	0.76014
rs3801339	LOC646913	7	35914335	4	0.747618
rs3801339	LOC646913	7	35914335	1	4.949293
rs3801339	LOC646913	7	35914335	8	0.707715
rs3801339	LOC646913	7	35914335	5	0.642069
rs3801339	LOC646913	7	35914335	7	1.404837
rs2241142	EEPD1	7	36305492	5	0.207183
rs13224904	EEPD1	7	36306155	8	4.788596
rs13224904	EEPD1	7	36306155	4	0.58996
rs13224904	EEPD1	7	36306155	6	0.438891
rs13224824	EEPD1	7	36306249	4	0.485451
rs13224824	EEPD1	7	36306249	8	0.409203
rs13224824	EEPD1	7	36306249	6	0.262907
rs13224824	EEPD1	7	36306249	7	1.012411
rs196618	EEPD1	7	36306659	6	3.425458
rs196619	EEPD1	7	36306833	6	0.693468
rs196620	EEPD1	7	36306878	6	4.180441
rs196621	EEPD1	7	36306949	2	4.401413
SNP7-36413880	ANLN	7	36413880	1	4.33164
SNP7-36413880	ANLN	7	36413880	4	0.130258
rs2052197	ANLN	7	36458737	5	7.301497
rs3735386	AOAH	7	36622560	8	4.106454
rs3735386	AOAH	7	36622560	3	0.5228
rs3735386	AOAH	7	36622560	6	3.852365
rs11976480	AOAH	7	36730197	4	0.593661

rs11976480	AOAH	7	36730197	1	0.815652
rs2228411	AOAH	7	36730213	8	0.19983
rs2717968	ELMO1	7	37265325	3	0.25096
rs2598116	SFRP4	7	37912873	5	0.976746
rs17236975	SFRP4	7	37913483	7	1.728027
rs1802074	SFRP4	7	37913628	5	0.176097
rs2072505	AMPH	7	38390564	8	0.186585
rs2072505	AMPH	7	38390564	4	1.166235
rs2072505	AMPH	7	38390564	1	0.987866
rs1058656	AMPH	7	38397961	1	0.1239
rs2392591	KRT8P20	7	38661735	3	1.934578
rs10226586	KRT8P20	7	38662109	2	0.469819
rs17416951	LOC340286	7	38691682	1	1.755045
rs17416951	LOC340286	7	38691682	3	0.051835
rs11980087	LOC340286	7	38692329	5	0.890661
rs11980087	LOC340286	7	38692329	7	0.558544
rs11980087	LOC340286	7	38692329	1	1.283822
rs17508140	LOC340286	7	38692998	2	1.944678
rs17508140	LOC340286	7	38692998	3	3.284617
rs17508140	LOC340286	7	38692998	1	2.928019
rs2302123	POU6F2	7	39439233	2	1.104422
rs2302123	POU6F2	7	39439233	1	1.346033
rs2302122	POU6F2	7	39439371	2	3.652437
rs2010706	C7orf10	7	40866492	6	4.477498
rs2010706	C7orf10	7	40866492	2	7.249023
rs2010706	C7orf10	7	40866492	5	4.566656
rs2010706	C7orf10	7	40866492	4	6.018072
rs929387	GLI3	7	41972203	7	1.461043
rs929387	GLI3	7	41972203	1	2.015623
rs2304327	HECW1	7	43485862	1	0.734757
rs6977217	LOC442673	7	43925034	6	2.211055
rs6977217	LOC442673	7	43925034	1	2.699249
rs6977217	LOC442673	7	43925034	5	5.412856
GA024458	DBNL	7	44066189	3	0.331563
GA024458	DBNL	7	44066189	8	0.206677
rs7779139	POLM	7	44079219	1	4.971167
rs7779139	POLM	7	44079219	8	4.064433
rs7779139	POLM	7	44079219	4	0.359218
rs3218660	POLM	7	44079398	3	0.037916
rs3218660	POLM	7	44079398	8	4.208399
rs3218660	POLM	7	44079398	4	0.781089
SNP7-44114010	AEBP1	7	44114010	5	4.217839
SNP7-44114010	AEBP1	7	44114010	8	2.283648
SNP7-44114010	AEBP1	7	44114010	7	2.832775
rs2595701	AEBP1	7	44115078	5	1.953173
rs2595701	AEBP1	7	44115078	8	1.827941
rs3217972	AEBP1	7	44120393	5	2.524892

SNP7-44218718	YKT6	7	44218718	2	3.468978
SNP7-44218718	YKT6	7	44218718	5	5.427623
rs1127065	CAMK2B	7	44226396	5	1.958207
rs1043084	NUDCD3	7	44390778	3	0.726457
rs11550029	NUDCD3	7	44410647	3	0.328486
rs11550029	NUDCD3	7	44410647	8	0.362822
SNP7-44496718	NUDCD3	7	44496718	3	0.051767
SNP7-44496718	NUDCD3	7	44496718	8	4.491569
rs3187907	NPC1L1	7	44518734	3	0.086462
SNP7-44577760	DDX56	7	44577760	6	0.911035
rs8580	TMED4	7	44587361	5	0.777369
rs8580	TMED4	7	44587361	2	5.925126
rs17551563	OGDH	7	44612710	8	0.397876
rs2070607	OGDH	7	44714103	8	0.207765
rs1297837	H2AFV	7	44836062	1	4.195498
rs1297837	H2AFV	7	44836062	8	1.244023
rs13221185	PURB	7	44887232	7	0.323167
rs13221185	PURB	7	44887232	1	5.286296
rs13221185	PURB	7	44887232	3	0.019066
rs13221185	PURB	7	44887232	4	0.308658
rs11555636	MYO1G	7	44976248	3	0.177878
SNP7-44983146	MYO1G	7	44983146	1	1.366909
SNP7-44983146	MYO1G	7	44983146	3	0.356901
rs1294962	CCM2	7	45033542	1	3.155327
rs2289365	CCM2	7	45033792	4	1.027555
rs2289365	CCM2	7	45033792	6	1.164157
rs3735493	NACAD	7	45088849	1	0.608554
rs3735494	NACAD	7	45090811	1	0.123064
rs3735494	NACAD	7	45090811	4	0.649007
rs3735495	NACAD	7	45090990	4	0.009656
rs3735495	NACAD	7	45090990	1	0.464794
rs4553	TBRG4	7	45106417	6	1.076538
rs4553	TBRG4	7	45106417	1	3.650377
rs12702121	RAMP3	7	45189784	4	1.404406
rs3735669	ADCY1	7	45721107	3	0.766831
rs3735669	ADCY1	7	45721107	7	0.29174
rs2280496	ADCY1	7	45723522	5	2.435908
rs2280496	ADCY1	7	45723522	1	2.612817
rs2280496	ADCY1	7	45723522	6	1.137743
rs2280496	ADCY1	7	45723522	7	0.616329
rs2280495	ADCY1	7	45723868	7	0.055841
rs2461127	ADCY1	7	45724331	3	0.184141
rs2461127	ADCY1	7	45724331	1	1.161705
rs2471267	ADCY1	7	45725787	5	0.683868
rs2471267	ADCY1	7	45725787	8	0.584609
rs12754	ADCY1	7	45727719	4	0.416776
rs3735666	ADCY1	7	45728477	4	0.601138

rs2960287	13-Sep	7	45731759	8	2.031336
rs2960287	13-Sep	7	45731759	1	5.249123
rs2960287	13-Sep	7	45731759	4	3.953172
rs2960287	13-Sep	7	45731759	3	1.383509
rs2960287	13-Sep	7	45731759	5	0.389076
rs2960287	13-Sep	7	45731759	6	0.592029
rs4988515	IGFBP1	7	45899125	3	0.181248
rs4619	IGFBP1	7	45899194	3	0.187813
rs7454	IGFBP1	7	45899531	3	0.354004
rs3210894	TNS3	7	47283849	6	5.399072
rs3210894	TNS3	7	47283849	1	5.218282
rs3750164	TNS3	7	47289981	5	0.240049
rs3750164	TNS3	7	47289981	6	0.42213
rs3750164	TNS3	7	47289981	3	0.684265
rs3750164	TNS3	7	47289981	4	3.910168
rs11763044	TNS3	7	47448127	5	0.531222
rs11763044	TNS3	7	47448127	2	1.592542
rs11763044	TNS3	7	47448127	1	0.222254
rs10261525	TNS3	7	47535243	5	0.586843
rs17545279	PKD1L1	7	47781054	8	1.908734
rs13231277	PKD1L1	7	47806912	5	0.149028
rs13231277	PKD1L1	7	47806912	4	0.232301
rs13231277	PKD1L1	7	47806912	7	0.937031
rs13231277	PKD1L1	7	47806912	8	3.119232
rs2290386	PKD1L1	7	47819362	8	6.014062
SNP7-47880085	PKD1L1	7	47880085	4	1.228881
rs1470859	PKD1L1	7	47880104	7	1.902738
SNP7-47883612	PKD1L1	7	47883612	3	2.201937
rs2686817	PKD1L1	7	47935452	6	5.04014
rs2686817	PKD1L1	7	47935452	4	0.894264
rs2686817	PKD1L1	7	47935452	5	0.149266
rs2686817	PKD1L1	7	47935452	3	1.142153
rs885337	PKD1L1	7	47938100	4	4.081726
rs885337	PKD1L1	7	47938100	3	0.468356
rs885337	PKD1L1	7	47938100	5	0.333427
rs885337	PKD1L1	7	47938100	1	0.463446
rs885337	PKD1L1	7	47938100	6	1.019044
rs885338	PKD1L1	7	47938151	2	3.456464
rs885338	PKD1L1	7	47938151	7	1.190529
rs10951942	C7orf57	7	48047620	7	1.472944
rs10951942	C7orf57	7	48047620	6	5.998804
rs10951942	C7orf57	7	48047620	8	0.468671
rs10951942	C7orf57	7	48047620	3	1.060616
rs10951942	C7orf57	7	48047620	1	3.499505
rs2708912	C7orf57	7	48052602	7	1.09128
rs7790252	C7orf57	7	48066541	3	1.566464
rs7790252	C7orf57	7	48066541	7	0.392729

rs7790252	C7orf57	7	48066541	6	1.922934
rs7790252	C7orf57	7	48066541	8	1.1032
rs7790252	C7orf57	7	48066541	1	2.775683
SNP7-48256031	ABCA13	7	48256031	1	4.488971
SNP7-48256031	ABCA13	7	48256031	5	0.976077
SNP7-48256031	ABCA13	7	48256031	8	1.403258
rs17547816	ABCA13	7	48282204	1	5.176631
rs17547816	ABCA13	7	48282204	7	1.551925
SNP7-48282630	ABCA13	7	48282630	5	1.967702
rs1358066	ABCA13	7	48283220	5	1.449426
rs1358066	ABCA13	7	48283220	6	1.117712
rs1358066	ABCA13	7	48283220	1	4.319739
rs17547830	ABCA13	7	48284056	6	1.69082
rs17547830	ABCA13	7	48284056	5	0.526795
rs6583483	ABCA13	7	48284495	1	4.673111
rs6955212	ABCA13	7	48284825	1	4.449279
rs1880736	ABCA13	7	48286342	5	1.790793
rs1880736	ABCA13	7	48286342	6	1.144147
rs6583546	ABCA13	7	48307390	6	0.690176
rs6583546	ABCA13	7	48307390	5	4.040816
rs3931814	ABCA13	7	48320193	5	2.48502
rs17548783	ABCA13	7	48420703	4	0.814966
rs17548783	ABCA13	7	48420703	3	1.743146
rs17548783	ABCA13	7	48420703	7	0.658787
rs6955686	ABCA13	7	48657146	5	0.459749
rs6955686	ABCA13	7	48657146	3	1.320654
SNP7-49812923	VWC2	7	49812923	1	0.818117
rs11980379	IKZF1	7	50437475	1	1.401444
rs801142	COBL	7	51254964	6	6.329767
rs801142	COBL	7	51254964	1	5.741871
rs11238247	DKFZp564N2472	7	53071048	3	0.991001
rs11238247	DKFZp564N2472	7	53071048	7	0.686213
rs11238247	DKFZp564N2472	7	53071048	5	1.064605
rs11238248	DKFZp564N2472	7	53071065	3	0.25651
rs1184320	DKFZp564N2472	7	53071167	5	2.606963
rs1184320	DKFZp564N2472	7	53071167	8	1.823024
rs1184320	DKFZp564N2472	7	53071167	7	1.269143
rs3862849	VSTM2A	7	54586113	7	0.784641
rs3862849	VSTM2A	7	54586113	4	0.020344
rs3862849	VSTM2A	7	54586113	6	0.043315
rs2709289	SEC61G	7	54794422	2	5.461121
rs2709289	SEC61G	7	54794422	3	0.517717
rs10228436	EGFR	7	55205762	1	0.179562
rs10228436	EGFR	7	55205762	4	0.527115
rs10228436	EGFR	7	55205762	6	5.096331
rs10228436	EGFR	7	55205762	7	0.783917
rs2293347	EGFR	7	55236410	1	4.712089

rs1267	LANCL2	7	55468742	1	0.432526
rs1267	LANCL2	7	55468742	8	3.404896
rs1267	LANCL2	7	55468742	2	0.391785
rs1267	LANCL2	7	55468742	3	0.11985
rs10239854	LOC442308	7	55681776	1	1.91727
rs10239854	LOC442308	7	55681776	8	0.979474
rs10239854	LOC442308	7	55681776	6	1.545064
rs10239854	LOC442308	7	55681776	5	2.854319
rs10239854	LOC442308	7	55681776	4	2.042529
rs11238393	PHKG1	7	56116856	3	0.983018
rs11238393	PHKG1	7	56116856	5	0.354022
rs11238393	PHKG1	7	56116856	8	0.599834
rs11238393	PHKG1	7	56116856	7	0.559022
rs13238521	PHKG1	7	56117433	4	0.329521
rs9642239	LOC441233	7	57474574	3	0.336954
rs13229184	LOC441233	7	57475757	3	1.376704
SNP7-63804237	ZNF107	7	63804237	6	0.305959
rs7804955	LOC168474	7	63950142	5	1.187933
rs2017252	ZNF273	7	64026524	2	5.466488
rs2017252	ZNF273	7	64026524	5	0.417174
rs2017252	ZNF273	7	64026524	7	0.948944
rs3807068	NULL	7	64077048	7	1.613942
rs3807068	NULL	7	64077048	8	1.398038
rs3815684	LOC285908	7	65480430	7	0.968429
rs6950137	LOC641765	7	65614045	2	2.493987
rs6950137	LOC641765	7	65614045	1	1.781034
rs6950137	LOC641765	7	65614045	5	1.45965
rs4718382	KCTD7	7	65742344	7	0.984374
rs9791713	KCTD7	7	65742633	5	3.554128
rs41303396	TYW1	7	66101373	4	0.532417
rs7776775	TYW1	7	66285634	6	0.092833
rs7776775	TYW1	7	66285634	4	6.390532
SNP7-66340763	TYW1	7	66340763	4	2.040279
SNP7-66340763	TYW1	7	66340763	2	4.849367
SNP7-66340763	TYW1	7	66340763	6	3.625442
SNP7-66411448	STAG3L4	7	66411448	4	3.524785
SNP7-66411448	STAG3L4	7	66411448	1	5.535307
SNP7-66411448	STAG3L4	7	66411448	8	4.377141
SNP7-66411448	STAG3L4	7	66411448	7	4.161634
SNP7-66411448	STAG3L4	7	66411448	6	6.550709
SNP7-66411448	STAG3L4	7	66411448	2	3.166975
SNP7-66411448	STAG3L4	7	66411448	3	4.141852
SNP7-66411448	STAG3L4	7	66411448	5	3.610626
rs887715	STAG3L4	7	66423819	1	1.446903
rs887715	STAG3L4	7	66423819	6	1.960266
rs887715	STAG3L4	7	66423819	5	0.702013
rs887715	STAG3L4	7	66423819	4	3.451502

rs887715	STAG3L4	7	66423819	8	1.464319
rs887715	STAG3L4	7	66423819	3	1.205482
rs3735260	AUTS2	7	68702573	6	0.390903
rs13232120	TBL2	7	72621246	8	4.401425
rs13232120	TBL2	7	72621246	7	6.767043
rs13232120	TBL2	7	72621246	3	0.64492
rs1051921	MLXIPL	7	72645879	7	0.572619
rs1051921	MLXIPL	7	72645879	3	2.473821
rs1051921	MLXIPL	7	72645879	5	0.203569
rs35332062	MLXIPL	7	72649978	3	1.950986
rs35332062	MLXIPL	7	72649978	7	1.413883
rs35332062	MLXIPL	7	72649978	8	1.270429
rs1376	WBSCR18	7	72733375	3	0.320499
rs1376	WBSCR18	7	72733375	4	0.764556
rs4717803	WBSCR18	7	72733580	4	0.226816
rs4717803	WBSCR18	7	72733580	3	0.4769
rs867500	STX1A	7	72752376	3	0.089555
rs867500	STX1A	7	72752376	6	6.656085
rs867500	STX1A	7	72752376	4	0.412872
rs1569061	STX1A	7	72752417	8	6.704192
SNP7-72917595	WBSCR28	7	72917595	7	0.897044
SNP7-72917595	WBSCR28	7	72917595	1	4.593003
rs2071307	ELN	7	73108650	3	0.776519
rs2071307	ELN	7	73108650	1	1.304436
rs539518	CLIP2	7	73391186	4	3.553226
rs539518	CLIP2	7	73391186	5	6.063241
rs5874	CLIP2	7	73457960	4	0.66783
rs5874	CLIP2	7	73457960	1	6.167865
rs5874	CLIP2	7	73457960	3	0.052574
rs5874	CLIP2	7	73457960	6	0.538304
rs1167829	HIP1	7	75002954	8	5.200008
rs1167829	HIP1	7	75002954	6	4.275013
rs1167830	HIP1	7	75004337	6	0.124319
rs1167794	HIP1	7	75010206	7	0.146908
rs1167794	HIP1	7	75010206	3	0.552714
rs2302009	CCL26	7	75236934	7	0.961212
rs2302009	CCL26	7	75236934	6	1.022881
rs11465293	CCL24	7	75280659	1	3.892218
rs7223	RHBDD2	7	75355956	6	5.462401
rs8509	TMEM120A	7	75454513	5	8.739007
rs8509	TMEM120A	7	75454513	8	0.032
rs8565	STYXL1	7	75468210	5	1.420968
rs8565	STYXL1	7	75468210	2	3.825587
rs1044484	STYXL1	7	75472623	8	5.868408
rs1044484	STYXL1	7	75472623	4	0.408439
rs1044484	STYXL1	7	75472623	3	0.641266
rs2302435	STYXL1	7	75514923	5	0.573368

rs2302435	STYXL1	7	75514923	6	0.57926
rs2302435	STYXL1	7	75514923	7	1.475158
rs2302435	STYXL1	7	75514923	2	0.96006
rs2302438	STYXL1	7	75515001	6	0.542615
rs2302438	STYXL1	7	75515001	5	0.565207
rs2302438	STYXL1	7	75515001	8	4.651957
rs2302438	STYXL1	7	75515001	7	0.174677
rs3178297	MDH2	7	75533549	7	1.278187
rs3178297	MDH2	7	75533549	1	3.924181
rs1046304	YWHAG	7	75795580	8	4.270863
rs12540545	SRCRB4D	7	75876784	5	0.475197
rs12540545	SRCRB4D	7	75876784	6	1.873365
rs3095481	LOC641808	7	76589293	6	3.253959
rs3095481	LOC641808	7	76589293	1	2.448715
rs3095481	LOC641808	7	76589293	3	2.784298
rs3095481	LOC641808	7	76589293	2	3.405811
rs3763402	CCDC146	7	76754755	4	0.709649
rs8124	CCDC146	7	76762354	1	4.850927
rs4727366	tcag7.1314	7	76828114	8	5.771519
rs4727366	tcag7.1314	7	76828114	4	0.116841
rs4727366	tcag7.1314	7	76828114	3	0.254376
rs1527263	tcag7.1314	7	76829871	2	0.668026
rs1527263	tcag7.1314	7	76829871	3	0.773899
rs1527263	tcag7.1314	7	76829871	8	0.308856
rs6949654	tcag7.1314	7	76873328	1	4.209376
SNP7-77103076	PTPN12	7	77103076	6	6.451651
rs1009524	MAGI2	7	77627339	7	0.938695
rs3735442	MAGI2	7	77957045	7	1.492709
rs3735442	MAGI2	7	77957045	6	1.086005
rs12721456	GNAI1	7	79670962	6	0.497866
rs10241877	GNAI1	7	79680093	8	0.25702
rs10241877	GNAI1	7	79680093	1	3.499586
rs1049654	CD36	7	80113391	4	0.110431
rs1049654	CD36	7	80113391	6	1.010131
rs5745678	HGF	7	81209983	6	0.660895
rs5745678	HGF	7	81209983	4	3.936018
rs5745678	HGF	7	81209983	5	0.55344
rs5745676	HGF	7	81210092	6	1.428881
rs5745676	HGF	7	81210092	4	0.22561
rs5745676	HGF	7	81210092	5	0.388905
rs5745646	HGF	7	81219262	6	0.270563
rs5745646	HGF	7	81219262	4	0.231611
rs5745646	HGF	7	81219262	5	0.572431
rs2040957	SEMA3D	7	84464324	6	0.667835
rs2040957	SEMA3D	7	84464324	8	2.396622
rs2040957	SEMA3D	7	84464324	4	0.15124
rs10261725	SEMA3D	7	84465167	5	1.207529

rs10261725	SEMA3D	7	84465167	4	4.865515
rs10261725	SEMA3D	7	84465167	8	0.419741
rs7800072	SEMA3D	7	84466925	5	0.49619
rs7800072	SEMA3D	7	84466925	6	0.936073
rs7800072	SEMA3D	7	84466925	4	1.462091
rs17559084	SEMA3D	7	84482436	5	0.928324
rs17559084	SEMA3D	7	84482436	8	2.521306
rs17559084	SEMA3D	7	84482436	6	0.406076
rs17559084	SEMA3D	7	84482436	4	4.143591
SNP7-86385770	KIAA1324L	7	86385770	8	0.639258
rs2109505	ABCB4	7	86917342	4	0.027002
rs1045642	ABCB1	7	86976581	4	0.438327
rs1045642	ABCB1	7	86976581	7	0.097533
rs9655955	SLC25A40	7	87343366	6	12.04444
rs6977687	DBF4	7	87375963	5	0.435712
rs6977687	DBF4	7	87375963	6	0.137469
rs6977687	DBF4	7	87375963	8	0.719377
rs9655959	STEAP4	7	87746324	1	5.632417
SNP7-87751157	STEAP4	7	87751157	1	5.873351
SNP7-87751297	STEAP4	7	87751297	6	0.243115
SNP7-87751297	STEAP4	7	87751297	2	4.917137
SNP7-87751297	STEAP4	7	87751297	5	1.326251
SNP7-87751297	STEAP4	7	87751297	8	3.652552
rs6465147	LOC442709	7	88106915	7	3.545416
rs6465147	LOC442709	7	88106915	1	4.240691
rs6465147	LOC442709	7	88106915	2	3.839674
rs2293583	MGC26647	7	88261817	3	0.715747
rs2293583	MGC26647	7	88261817	1	0.750603
rs2293583	MGC26647	7	88261817	5	0.015211
SNP7-88801997	ZNF804B	7	88801997	5	1.971505
SNP7-88803077	ZNF804B	7	88803077	4	2.535965
rs194503	STEAP2	7	89679020	1	0.968169
rs194503	STEAP2	7	89679020	6	1.377845
rs194503	STEAP2	7	89679020	5	0.03877
rs17621350	STEAP2	7	89699893	1	4.158265
rs17621350	STEAP2	7	89699893	4	0.803399
rs17621350	STEAP2	7	89699893	7	0.2165
rs17621350	STEAP2	7	89699893	8	1.823562
rs194527	STEAP2	7	89700620	6	1.86203
rs194527	STEAP2	7	89700620	5	2.052231
rs194528	STEAP2	7	89700934	7	7.399461
rs194528	STEAP2	7	89700934	6	1.455373
rs194528	STEAP2	7	89700934	5	1.567265
rs194529	STEAP2	7	89702249	1	5.238046
rs194529	STEAP2	7	89702249	6	2.277934
rs194529	STEAP2	7	89702249	7	4.52664
rs194529	STEAP2	7	89702249	5	1.575911

rs11563905	GTPBP10	7	89813987	6	0.584326
rs3810874	CLDN12	7	89880793	3	0.230754
rs3810874	CLDN12	7	89880793	2	5.707782
rs3810874	CLDN12	7	89880793	4	5.679026
rs11767691	PFTK1	7	90176786	7	0.428316
rs11767691	PFTK1	7	90176786	4	0.221329
rs11771034	PFTK1	7	90675152	3	0.407702
rs9008	MTERF	7	91340343	5	0.180018
rs9008	MTERF	7	91340343	1	0.33606
rs9008	MTERF	7	91340343	2	1.190274
rs10225885	AKAP9	7	91508943	5	0.431598
rs10225885	AKAP9	7	91508943	8	0.144005
SNP7-91546834	AKAP9	7	91546834	4	1.131164
SNP7-91550634	AKAP9	7	91550634	2	3.728645
SNP7-91550634	AKAP9	7	91550634	5	0.948705
rs8039	CYP51A1	7	91580284	4	0.645351
rs8039	CYP51A1	7	91580284	1	0.13762
rs8039	CYP51A1	7	91580284	6	4.781357
rs8039	CYP51A1	7	91580284	7	4.923592
rs1861635	ANKIB1	7	91865473	7	0.177918
rs1861635	ANKIB1	7	91865473	1	4.850791
SNP7-92079387	CDK6	7	92079387	6	0.522683
SNP7-92079387	CDK6	7	92079387	8	1.148148
SNP7-92079387	CDK6	7	92079387	4	0.034666
SNP7-92079387	CDK6	7	92079387	3	0.354212
rs3731389	CDK6	7	92082129	3	0.393106
rs10279499	SAMD9	7	92571702	8	5.077597
rs4267	SAMD9L	7	92597398	7	4.182288
rs1029357	SAMD9L	7	92600617	7	0.269733
rs1801197	CALCR	7	92893689	3	0.844629
rs4264	TFPI2	7	93354013	7	0.293767
rs4262	GNG11	7	93389364	1	0.093282
rs4262	GNG11	7	93389364	5	0.111862
rs4262	GNG11	7	93389364	2	0.032253
rs4262	GNG11	7	93389364	4	0.162356
rs6945362	BET1	7	93459329	5	0.348266
rs6945362	BET1	7	93459329	3	0.607638
rs6945362	BET1	7	93459329	4	0.678939
rs10953105	BET1	7	93460183	3	0.497969
rs1800248	COL1A2	7	93894285	8	0.153394
rs1800248	COL1A2	7	93894285	2	5.910988
SNP7-93895628	COL1A2	7	93895628	7	0.332037
rs1060399	COL1A2	7	93897835	2	0.52968
rs1060399	COL1A2	7	93897835	4	0.873293
rs1060399	COL1A2	7	93897835	1	0.60322
rs3750105	PEG10	7	94132618	6	4.510785
rs13073	PEG10	7	94134705	3	4.330155

rs13073	PEG10	7	94134705	1	4.628872
rs13073	PEG10	7	94134705	4	0.893562
rs11764692	PPP1R9A	7	94758928	6	0.593458
rs11764692	PPP1R9A	7	94758928	5	9.258883
rs3917579	PON1	7	94765279	5	0.020928
rs3735590	PON1	7	94765431	5	0.312596
rs854552	PON1	7	94765860	5	0.706097
rs854552	PON1	7	94765860	2	2.323832
rs662	PON1	7	94775382	6	2.108514
rs662	PON1	7	94775382	5	0.292088
rs662	PON1	7	94775382	7	2.67372
rs854560	PON1	7	94784020	2	2.104072
rs13226149	PON3	7	94863536	7	1.099773
rs6931	PDK4	7	95050838	5	0.137655
rs7802814	PDK4	7	95051547	7	0.799225
rs11765552	LMTK2	7	97660051	4	0.070429
SNP7-97684976	DKFZP434B0335	7	97684976	6	0.186013
SNP7-97684976	DKFZP434B0335	7	97684976	5	0.869437
SNP7-97690335	DKFZP434B0335	7	97690335	6	0.880985
SNP7-97690335	DKFZP434B0335	7	97690335	5	0.79186
rs2291745	DKFZP434B0335	7	97692329	4	0.055764
rs2291745	DKFZP434B0335	7	97692329	8	0.539147
rs2291745	DKFZP434B0335	7	97692329	6	4.165748
rs2291750	DKFZP434B0335	7	97701081	4	2.658176
rs2291750	DKFZP434B0335	7	97701081	8	4.111794
rs1874344	DKFZP434B0335	7	97711930	4	0.285431
rs1874344	DKFZP434B0335	7	97711930	6	5.410513
rs1874344	DKFZP434B0335	7	97711930	5	2.99541
rs7015	BRI3	7	97758559	8	7.217212
SNP7-97868313	BAIAP2L1	7	97868313	4	2.938746
SNP7-97868313	BAIAP2L1	7	97868313	6	6.856754
SNP7-97868313	BAIAP2L1	7	97868313	8	0.330992
SNP7-97868313	BAIAP2L1	7	97868313	2	4.836893
rs219815	TRRAP	7	98448620	4	0.261734
rs1007008	NULL	7	98464472	4	4.142021
rs219797	SMURF1	7	98487987	5	0.025334
rs219797	SMURF1	7	98487987	7	0.825521
rs219797	SMURF1	7	98487987	3	0.564607
rs219797	SMURF1	7	98487987	4	0.267784
rs1045012	ARPC1B	7	98822290	4	0.210132
rs3177186	ARPC1B	7	98822335	2	4.274952
rs17854665	CPSF4	7	98887975	4	3.517466
rs17854665	CPSF4	7	98887975	6	3.098537
rs6947974	ZNF655	7	98994442	2	5.247143
rs952319	ZNF655	7	99009385	1	0.605319
rs952319	ZNF655	7	99009385	4	5.4576
rs952319	ZNF655	7	99009385	2	0.702064

rs952319	ZNF655	7	99009385	6	1.2635
rs10282706	ZNF498	7	99055360	1	4.651949
rs10282706	ZNF498	7	99055360	4	0.897585
rs10282706	ZNF498	7	99055360	6	0.412681
rs1859690	ZNF498	7	99065108	4	0.064513
rs1859690	ZNF498	7	99065108	6	0.845285
rs15524	CYP3A5	7	99083850	6	1.047212
rs15524	CYP3A5	7	99083850	1	5.756835
rs15524	CYP3A5	7	99083850	4	0.600768
rs10211	CYP3A7	7	99140930	6	0.302632
rs10211	CYP3A7	7	99140930	4	0.912605
rs12360	CYP3A7	7	99141030	3	0.680881
rs12360	CYP3A7	7	99141030	5	0.949265
rs12360	CYP3A7	7	99141030	8	0.587739
rs12360	CYP3A7	7	99141030	2	1.182363
rs12360	CYP3A7	7	99141030	1	0.853617
rs2257401	CYP3A7	7	99144621	4	1.677558
rs2257401	CYP3A7	7	99144621	6	0.013523
rs17161997	OR2AE1	7	99311794	7	0.360316
rs17277546	TRIM4	7	99327507	7	1.113781
rs11558476	ZSCAN21	7	99492625	4	1.75479
rs11558476	ZSCAN21	7	99492625	1	4.565513
rs1534310	MCM7	7	99537105	3	0.673568
rs1534310	MCM7	7	99537105	5	0.242483
rs4134932	AP4M1	7	99542384	1	5.540499
rs3807479	TAF6	7	99554837	3	0.518814
rs3807479	TAF6	7	99554837	1	2.537642
rs3807479	TAF6	7	99554837	5	1.238352
rs3735241	STAG3	7	99634082	4	0.184591
rs3735241	STAG3	7	99634082	3	1.222782
rs3735241	STAG3	7	99634082	5	1.239556
rs3735241	STAG3	7	99634082	1	3.637403
rs1043915	STAG3	7	99637781	5	0.662243
rs941287	GATS	7	99645409	1	6.148053
rs941287	GATS	7	99645409	5	0.054287
rs941287	GATS	7	99645409	4	4.824346
rs941287	GATS	7	99645409	3	0.664057
rs705867	PVRIG	7	99656863	4	0.154583
rs705867	PVRIG	7	99656863	3	0.8599
rs705867	PVRIG	7	99656863	5	0.128705
rs705867	PVRIG	7	99656863	1	0.170922
rs2405442	PILRA	7	99809249	5	1.381348
rs2405442	PILRA	7	99809249	3	2.583473
rs7783159	ZCWPW1	7	99855390	1	3.348976
rs7783159	ZCWPW1	7	99855390	3	0.873679
rs7783159	ZCWPW1	7	99855390	6	1.017855
rs7783159	ZCWPW1	7	99855390	4	4.794516

rs7783159	ZCWPW1	7	99855390	7	0.050816
rs12539172	C7orf51	7	99929731	3	0.922667
rs12539172	C7orf51	7	99929731	5	0.248949
SNP7-99998200	HRBL	7	99998200	6	0.508743
SNP7-99998200	HRBL	7	99998200	1	3.371069
rs34440977	LRCH4	7	100017996	6	1.98016
rs2075674	TFR2	7	100062967	1	0.67088
rs2075674	TFR2	7	100062967	5	0.760071
rs2075674	TFR2	7	100062967	4	0.805928
rs2075674	TFR2	7	100062967	8	0.52506
rs2075674	TFR2	7	100062967	2	0.961356
rs221789	GNB2	7	100109457	2	1.804899
rs221789	GNB2	7	100109457	4	2.447735
rs2272572	PERQ1	7	100123824	4	0.512435
rs2272572	PERQ1	7	100123824	8	0.454863
rs2272572	PERQ1	7	100123824	2	0.227433
rs2272572	PERQ1	7	100123824	5	0.004075
rs2272572	PERQ1	7	100123824	1	0.695229
GA024740	GIGYF1	7	100124371	1	0.471695
GA024740	GIGYF1	7	100124371	5	0.002774
GA024740	GIGYF1	7	100124371	8	2.259188
GA024740	GIGYF1	7	100124371	2	1.260455
GA024740	GIGYF1	7	100124371	4	0.138242
GA024742	POP7	7	100142800	4	0.857294
GA024742	POP7	7	100142800	2	4.420822
SNP7-10020903(	ZAN	7	100209036	8	2.002714
SNP7-10020903(	ZAN	7	100209036	1	2.773215
SNP7-10020903(	ZAN	7	100209036	6	2.907798
SNP7-10020903(	ZAN	7	100209036	2	3.381912
SNP7-10020903(	ZAN	7	100209036	5	1.96238
SNP7-10020903(	ZAN	7	100209036	7	1.387979
rs314299	ZAN	7	100209409	1	0.154672
rs542137	ZAN	7	100211013	7	1.908907
rs542137	ZAN	7	100211013	5	0.356107
rs539445	ZAN	7	100211303	4	0.641629
rs539445	ZAN	7	100211303	7	0.18353
rs539445	ZAN	7	100211303	2	1.155804
rs539445	ZAN	7	100211303	1	0.384899
SNP7-10021501{	ZAN	7	100215018	5	1.136046
SNP7-10021501{	ZAN	7	100215018	1	2.051623
rs314344	ZAN	7	100232939	2	0.134545
rs314344	ZAN	7	100232939	1	0.314987
rs314344	ZAN	7	100232939	7	0.33436
rs2230585	EPHB4	7	100248533	7	0.85132
rs2230585	EPHB4	7	100248533	1	0.108183
rs2230585	EPHB4	7	100248533	8	4.686508
rs2230585	EPHB4	7	100248533	5	0.336757

rs2230585	EPHB4	7	100248533	4	0.90735
rs2230585	EPHB4	7	100248533	2	1.00674
rs314359	EPHB4	7	100249214	1	0.133886
rs314359	EPHB4	7	100249214	7	0.923745
rs314359	EPHB4	7	100249214	2	0.604028
rs144173	EPHB4	7	100254186	2	1.268767
rs144173	EPHB4	7	100254186	5	0.781469
rs144173	EPHB4	7	100254186	8	1.421829
rs144173	EPHB4	7	100254186	7	1.274833
rs144173	EPHB4	7	100254186	1	0.741561
rs144173	EPHB4	7	100254186	4	1.122354
rs6706	TRIP6	7	100308980	4	1.071071
rs6706	TRIP6	7	100308980	3	3.91451
rs11171	ARS2	7	100324046	2	2.276187
rs13241786	tcag7.1226	7	100324592	2	3.171572
rs13241786	tcag7.1226	7	100324592	8	7.091869
rs13241786	tcag7.1226	7	100324592	4	1.085433
rs13241786	tcag7.1226	7	100324592	1	0.340959
rs13241786	tcag7.1226	7	100324592	5	0.561009
rs10953305	LOC729931	7	100330504	7	0.744883
rs10953305	LOC729931	7	100330504	5	1.889113
rs10953305	LOC729931	7	100330504	8	1.046007
rs10953305	LOC729931	7	100330504	1	0.974768
rs10953305	LOC729931	7	100330504	6	1.951454
rs10953305	LOC729931	7	100330504	4	1.2063
SNP7-10046223	MUC17	7	100462232	2	0.107601
SNP7-10046269	MUC17	7	100462696	3	0.190137
rs1006507	C7orf52	7	100600754	5	0.40425
rs1006507	C7orf52	7	100600754	4	0.03586
rs1006507	C7orf52	7	100600754	8	0.220434
rs1006507	C7orf52	7	100600754	1	1.199753
rs1859528	C7orf52	7	100600798	4	0.596906
rs1859528	C7orf52	7	100600798	8	0.338598
rs1859528	C7orf52	7	100600798	1	4.923384
rs1859528	C7orf52	7	100600798	5	0.746533
GA024770	PLOD3	7	100645954	6	5.522035
rs1056678	PLOD3	7	100647356	2	0.922068
rs1056678	PLOD3	7	100647356	7	0.640841
rs1056678	PLOD3	7	100647356	6	0.065257
rs17393069	EMID2	7	100987447	4	0.900306
rs17393069	EMID2	7	100987447	5	0.190182
rs2240389	MYLC2PL	7	101043553	7	0.272847
rs17498164	CUX1	7	101623869	7	1.041718
rs17498164	CUX1	7	101623869	6	0.490565
rs17498164	CUX1	7	101623869	4	1.211219
rs10279449	FBXL13	7	102249872	4	2.5123
rs10279449	FBXL13	7	102249872	1	4.034189

rs10279449	FBXL13	7	102249872	3	1.046075
rs17135923	FBXL13	7	102340839	5	0.690939
rs3800939	LRRC17	7	102361951	1	2.969135
rs3800939	LRRC17	7	102361951	4	5.877508
rs3800939	LRRC17	7	102361951	3	1.044269
rs3808039	RELN	7	102938672	1	2.172452
rs2229864	RELN	7	102942941	5	2.275077
SNP7-103079437	RELN	7	103079437	1	0.196791
rs766981	MLL5	7	104529851	7	1.219286
SNP7-105402642	FLJ23834	7	105402642	8	1.839424
rs10258293	FLJ23834	7	105449926	7	0.865885
rs2230460	PIK3CG	7	106311925	1	1.678774
rs2230460	PIK3CG	7	106311925	4	1.522132
rs2230460	PIK3CG	7	106311925	5	2.195169
rs2712218	SLC26A4	7	107143995	3	1.400007
SNP7-107214558	SLC26A3	7	107214558	5	0.891147
rs8721	DLD	7	107346958	5	1.125404
rs8721	DLD	7	107346958	3	0.202666
rs8721	DLD	7	107346958	7	0.456233
rs3177964	LAMB1	7	107356753	2	1.451549
rs3177964	LAMB1	7	107356753	7	4.532067
rs3177964	LAMB1	7	107356753	8	4.252488
rs3177964	LAMB1	7	107356753	3	0.707435
rs3177964	LAMB1	7	107356753	5	0.351695
rs4730283	LAMB1	7	107381225	1	1.729891
rs4730283	LAMB1	7	107381225	2	0.538891
rs25659	LAMB1	7	107414006	8	2.324391
rs25659	LAMB1	7	107414006	3	0.074511
rs9690688	LAMB4	7	107507398	6	0.903268
rs9690688	LAMB4	7	107507398	1	1.280012
rs3815246	LAMB4	7	107526118	6	0.350697
rs451252	LAMB4	7	107526184	6	0.41831
rs451252	LAMB4	7	107526184	3	0.438407
rs449077	NRCAM	7	107577163	7	0.403388
rs401433	NRCAM	7	107612125	3	0.811607
rs2227272	THAP5	7	107996859	4	1.056593
rs2227272	THAP5	7	107996859	8	2.406236
rs2227272	THAP5	7	107996859	1	4.754637
rs2074796	IFRD1	7	111899515	7	0.577183
rs2074796	IFRD1	7	111899515	5	5.557998
rs2074796	IFRD1	7	111899515	6	8.59669
rs2074796	IFRD1	7	111899515	3	0.420859
rs2256044	GPR85	7	112511653	4	1.323725
rs2256044	GPR85	7	112511653	1	2.843032
rs1575012	GPR85	7	112512031	8	2.118161
rs1575012	GPR85	7	112512031	1	1.323086
rs10274994	PPP1R3A	7	113302589	7	0.081859

rs10274994	PPP1R3A	7	113302589	3	0.03797
rs10274994	PPP1R3A	7	113302589	1	0.132869
rs7789198	PPP1R3A	7	113303463	3	0.986622
rs4730700	TFEC	7	115364898	4	1.497523
rs10229962	TFEC	7	115365360	3	1.171815
rs8940	CAV2	7	115933310	3	0.352151
rs8940	CAV2	7	115933310	1	3.039101
rs8940	CAV2	7	115933310	6	1.270403
rs1049334	CAV1	7	115987616	2	7.399076
rs1049334	CAV1	7	115987616	6	1.82211
rs2228946	WNT2	7	116705321	3	0.124301
rs1800076	CFTR	7	116936383	5	1.387856
rs1800136	CFTR	7	117094344	6	0.131448
rs798937	C7orf58	7	120527339	4	0.495206
rs35391640	WNT16	7	120756648	1	0.007228
rs2707466	WNT16	7	120766325	6	1.778975
rs2707466	WNT16	7	120766325	7	1.331474
rs2707466	WNT16	7	120766325	5	0.304524
rs2707466	WNT16	7	120766325	8	1.704599
rs6961822	LOC136143	7	120868165	7	3.008647
rs6961822	LOC136143	7	120868165	3	3.969936
rs10234745	IQUB	7	122884704	6	0.90599
rs10234745	IQUB	7	122884704	4	0.260335
rs10500091	IQUB	7	122907226	4	0.472676
rs10500091	IQUB	7	122907226	6	0.183326
rs10255061	IQUB	7	122939255	4	0.277622
rs4731119	WASL	7	123176329	6	0.957129
rs4731119	WASL	7	123176329	5	0.382507
rs1034955	HYAL4	7	123295545	1	0.277953
rs1034955	HYAL4	7	123295545	7	0.072082
rs1034955	HYAL4	7	123295545	4	5.287323
rs724356	GPR37	7	124174610	6	0.926452
rs1871773	LOC646837	7	124462175	4	2.6299
rs1871773	LOC646837	7	124462175	1	2.061903
rs1871773	LOC646837	7	124462175	6	2.294453
rs1871773	LOC646837	7	124462175	7	2.759986
rs712723	GRM8	7	125866380	5	2.893929
rs712723	GRM8	7	125866380	6	2.052039
rs712723	GRM8	7	125866380	3	1.683799
SNP7-12701210	GCC1	7	127012108	2	3.459527
rs712700	PAX4	7	127038143	3	1.388098
rs3823994	LRRC4	7	127457093	7	1.153852
rs3823994	LRRC4	7	127457093	6	0.461196
rs3097362	hCG_1651889	7	127788639	5	0.809441
rs3097362	hCG_1651889	7	127788639	7	0.441044
rs3097362	hCG_1651889	7	127788639	3	0.096919
rs2228075	IMPDH1	7	127821865	6	0.408174

rs11769769	FAM71F2	7	128099686	3	0.80682
rs11769769	FAM71F2	7	128099686	5	1.078942
rs11769769	FAM71F2	7	128099686	7	0.662756
SNP7-12810311{	FAM71F2	7	128103118	5	0.626074
SNP7-12810311{	FAM71F2	7	128103118	2	1.52523
SNP7-12810311{	FAM71F2	7	128103118	3	0.38025
SNP7-12810311{	FAM71F2	7	128103118	7	0.004731
rs6467210	FAM71F2	7	128104894	3	1.137208
rs6467210	FAM71F2	7	128104894	7	0.839018
rs6467210	FAM71F2	7	128104894	5	0.295592
rs6467210	FAM71F2	7	128104894	6	2.306783
rs1109552	FAM71F2	7	128110270	3	0.465688
rs1109552	FAM71F2	7	128110270	7	0.468237
rs1109552	FAM71F2	7	128110270	6	1.249965
rs1109552	FAM71F2	7	128110270	5	0.352131
rs1109552	FAM71F2	7	128110270	2	4.146359
rs745229	FAM71F1	7	128146127	3	0.343034
rs339065	FAM71F1	7	128158534	8	1.000994
rs101721	FAM71F1	7	128158651	7	0.334013
rs101721	FAM71F1	7	128158651	3	0.774798
rs2290228	CALU	7	128175884	2	0.00784
rs2290228	CALU	7	128175884	1	0.107292
rs2290228	CALU	7	128175884	8	3.412355
rs1799922	OPN1SW	7	128202431	7	0.426094
rs1799922	OPN1SW	7	128202431	4	0.483502
rs1799922	OPN1SW	7	128202431	5	0.429952
rs2270590	CCDC136	7	128243152	4	0.819221
rs2270590	CCDC136	7	128243152	5	1.11321
rs2291561	FLNC	7	128264783	5	0.336781
rs2291561	FLNC	7	128264783	6	1.102343
rs2291562	FLNC	7	128265339	6	0.744964
rs2291568	FLNC	7	128275129	6	1.732185
rs2291569	FLNC	7	128275970	6	1.104585
rs2291569	FLNC	7	128275970	5	2.155092
GA024891	FLNC	7	128286315	2	3.540835
SNP7-12829274(	ATP6V1F	7	128292746	2	1.281097
SNP7-12829274(	ATP6V1F	7	128292746	6	5.268331
SNP7-12829274(	ATP6V1F	7	128292746	4	2.33986
SNP7-12829274(	ATP6V1F	7	128292746	1	2.315698
SNP7-12829274(	ATP6V1F	7	128292746	7	1.615527
SNP7-12829274(	ATP6V1F	7	128292746	8	4.454928
SNP7-12829274(	ATP6V1F	7	128292746	3	1.903871
SNP7-12829274(	ATP6V1F	7	128292746	5	1.228688
SNP7-12837672	IRF5	7	128376724	7	1.370829
SNP7-12837672	IRF5	7	128376724	3	2.407343
SNP7-12837672	IRF5	7	128376724	4	1.24128
SNP7-12837686	IRF5	7	128376869	3	3.220087

SNP7-128376869	IRF5	7	128376869	7	1.622247
SNP7-128376869	IRF5	7	128376869	4	1.971503
rs3824	SMO	7	128640089	2	7.001693
rs9607	SMO	7	128640293	4	1.170407
rs9607	SMO	7	128640293	2	5.304063
rs7639	UBE2H	7	129260667	5	5.635811
rs7639	UBE2H	7	129260667	7	2.315641
rs7639	UBE2H	7	129260667	6	5.812341
rs7639	UBE2H	7	129260667	1	6.1199
rs7639	UBE2H	7	129260667	4	6.567744
rs7639	UBE2H	7	129260667	3	6.256615
rs7639	UBE2H	7	129260667	8	0.385236
rs1046691	UBE2H	7	129260975	1	3.252365
rs1046691	UBE2H	7	129260975	3	0.44717
rs1046691	UBE2H	7	129260975	5	4.006599
rs9641870	CPA2	7	129703751	5	0.379356
rs9641870	CPA2	7	129703751	1	3.239275
rs1544705	CPA5	7	129789553	1	0.513511
rs1544705	CPA5	7	129789553	7	1.27555
rs11765961	CPA5	7	129795495	7	1.320056
SNP7-129795521	CPA5	7	129795521	4	1.266539
SNP7-129795521	CPA5	7	129795521	3	1.400115
SNP7-129795521	CPA5	7	129795521	1	1.289102
SNP7-129795521	CPA5	7	129795521	2	1.560211
rs968404	CPA1	7	129809277	3	0.337009
rs12706927	CPA1	7	129810775	4	1.189976
rs12706927	CPA1	7	129810775	1	0.929311
rs12706927	CPA1	7	129810775	3	1.321095
rs12706927	CPA1	7	129810775	2	1.371774
rs2287371	TSGA14	7	129868048	7	0.487389
rs2287371	TSGA14	7	129868048	3	0.394469
rs2287371	TSGA14	7	129868048	8	0.617361
rs3807139	TSGA13	7	130021865	4	0.778097
rs1965692	LOC442723	7	130577997	6	0.324325
rs1965692	LOC442723	7	130577997	1	1.26803
rs1965692	LOC442723	7	130577997	2	1.96924
rs1965692	LOC442723	7	130577997	4	0.938823
rs1045046	PODXL	7	130836876	4	0.234289
rs1045046	PODXL	7	130836876	5	0.164456
rs1045046	PODXL	7	130836876	6	0.150283
rs3735035	PODXL	7	130846499	4	7.200714
rs1862047	SLC35B4	7	133628618	3	0.032715
rs1862047	SLC35B4	7	133628618	4	0.608915
rs1862047	SLC35B4	7	133628618	8	0.382192
rs1862047	SLC35B4	7	133628618	6	0.173255
rs1862047	SLC35B4	7	133628618	5	0.484855
rs1862048	SLC35B4	7	133628707	3	0.061376

rs1862048	SLC35B4	7	133628707	6	0.855876
rs1862048	SLC35B4	7	133628707	4	0.002919
rs1862048	SLC35B4	7	133628707	8	0.555264
rs1862048	SLC35B4	7	133628707	5	0.375621
rs1035328	SLC35B4	7	133628959	4	1.211428
rs1035328	SLC35B4	7	133628959	8	3.760871
rs1035328	SLC35B4	7	133628959	3	1.517089
rs1035328	SLC35B4	7	133628959	6	1.48982
rs1035328	SLC35B4	7	133628959	5	2.872084
rs2241334	SLC35B4	7	133629765	4	0.166404
rs2241334	SLC35B4	7	133629765	6	0.196308
rs2241334	SLC35B4	7	133629765	5	0.235796
rs2241334	SLC35B4	7	133629765	3	0.185951
rs2241334	SLC35B4	7	133629765	8	0.030878
rs2037004	AKR1B10	7	133863151	1	5.770794
rs2037004	AKR1B10	7	133863151	3	0.508271
rs1722883	AKR1B10	7	133865943	1	0.009253
rs4728329	AKR1B10	7	133876367	3	0.777807
rs3800691	BPGM	7	133993867	5	1.434847
rs2244697	CALD1	7	134305405	2	1.442844
rs2288237	TMEM140	7	134500117	6	0.148403
rs2288237	TMEM140	7	134500117	7	0.522931
rs2288237	TMEM140	7	134500117	2	0.206979
rs2288237	TMEM140	7	134500117	8	2.499893
rs2288237	TMEM140	7	134500117	4	2.762449
rs2288237	TMEM140	7	134500117	5	0.661218
rs2288237	TMEM140	7	134500117	1	1.365496
rs3110800	SLC13A4	7	135043416	2	2.670059
rs3110800	SLC13A4	7	135043416	6	1.894672
rs3110800	SLC13A4	7	135043416	7	2.964861
rs3110800	SLC13A4	7	135043416	5	0.645053
rs3110800	SLC13A4	7	135043416	4	1.23316
rs3110800	SLC13A4	7	135043416	1	0.659254
rs10227032	UNQ1940	7	135065286	8	0.446145
rs10227032	UNQ1940	7	135065286	4	0.35722
rs10227032	UNQ1940	7	135065286	2	1.232965
rs10227032	UNQ1940	7	135065286	5	0.882086
rs17168527	MTPN	7	135265280	6	0.460992
rs17168527	MTPN	7	135265280	4	0.971148
rs10275735	LOC392100	7	136049252	4	4.655251
rs10275735	LOC392100	7	136049252	5	3.624872
rs10275735	LOC392100	7	136049252	1	4.115178
rs7779548	DGKI	7	136725080	8	1.284488
rs7779548	DGKI	7	136725080	1	4.819338
rs7779548	DGKI	7	136725080	3	0.85206
rs1376608	DGKI	7	136725301	8	0.079455
rs1376608	DGKI	7	136725301	1	4.289972

rs1376608	DGKI	7	136725301	3	1.905619
rs3735026	DGKI	7	136725379	1	3.471831
rs3735026	DGKI	7	136725379	8	1.135841
rs3735026	DGKI	7	136725379	3	1.716158
rs3735025	DGKI	7	136725384	3	2.318214
rs3735025	DGKI	7	136725384	1	3.849255
rs7783145	CREB3L2	7	137215041	2	0.002047
rs7783145	CREB3L2	7	137215041	8	0.717601
rs7783145	CREB3L2	7	137215041	1	0.251559
rs7783145	CREB3L2	7	137215041	3	0.283874
rs1872929	AKR1D1	7	137451953	5	0.457295
rs1872930	AKR1D1	7	137451984	5	1.118889
rs1439793	SVOPL	7	137929768	3	1.773664
rs1439793	SVOPL	7	137929768	6	0.151437
rs1439793	SVOPL	7	137929768	1	4.492906
rs13238715	tcag7.441	7	138037087	6	4.682082
rs13238715	tcag7.441	7	138037087	7	3.344088
rs13238715	tcag7.441	7	138037087	2	4.275706
rs13238715	tcag7.441	7	138037087	5	3.984053
rs13238715	tcag7.441	7	138037087	1	3.443719
rs6957669	tcag7.441	7	138038319	4	1.147757
rs6957669	tcag7.441	7	138038319	3	0.234762
rs10258719	ATP6V0A4	7	138106528	1	0.185823
rs10258719	ATP6V0A4	7	138106528	7	9.663849
rs2034507	LOC155006	7	138140423	1	1.634009
rs2034507	LOC155006	7	138140423	8	2.885294
rs2718131	KIAA1549	7	138253456	2	0.495955
rs2718131	KIAA1549	7	138253456	3	0.673224
rs2718131	KIAA1549	7	138253456	1	0.082246
rs10265929	ZC3HAV1L	7	138361443	1	5.59801
rs3087999	ZC3HAV1	7	138382814	1	3.540831
rs3087999	ZC3HAV1	7	138382814	5	0.127499
rs3087999	ZC3HAV1	7	138382814	4	0.357869
rs2297236	ZC3HAV1	7	138390577	8	0.600058
rs2297236	ZC3HAV1	7	138390577	5	0.235624
rs2297236	ZC3HAV1	7	138390577	6	0.615617
rs9683	LUC7L2	7	138758279	7	0.643149
rs9683	LUC7L2	7	138758279	6	0.665452
SNP7-13881847	KLRG2	7	138818474	8	1.198681
SNP7-13881847	KLRG2	7	138818474	5	0.958911
rs7456421	LOC653052	7	139062261	6	0.946449
rs7456421	LOC653052	7	139062261	2	5.512157
rs4074826	LOC653052	7	139062429	7	9.409945
rs4074826	LOC653052	7	139062429	2	2.26495
rs1044703	PARP12	7	139370211	3	0.073292
rs1128589	SLC37A3	7	139680763	2	1.708214
rs1128589	SLC37A3	7	139680763	1	0.606776

rs1128589	SLC37A3	7	139680763	3	0.714871
SNP7-139726556	SLC37A3	7	139726556	6	1.042797
SNP7-139726556	SLC37A3	7	139726556	1	0.033668
SNP7-139726556	SLC37A3	7	139726556	4	0.646283
SNP7-139726556	SLC37A3	7	139726556	2	6.918698
SNP7-139726556	SLC37A3	7	139726556	8	8.019981
rs4726807	RAB19	7	139772308	3	0.693812
rs6852	DENND2A	7	139864933	4	0.462481
rs6852	DENND2A	7	139864933	7	3.027375
rs6464833	DENND2A	7	139873762	7	0.048304
rs6464833	DENND2A	7	139873762	3	0.17408
rs2293177	DENND2A	7	139891029	3	0.152476
rs2234002	TAS2R4	7	141125269	6	3.431206
rs2227264	TAS2R5	7	141136707	6	0.469381
rs1726866	TAS2R38	7	141319174	1	2.21545
rs1726866	TAS2R38	7	141319174	7	0.301651
rs1726866	TAS2R38	7	141319174	5	0.866464
rs1726866	TAS2R38	7	141319174	3	0.240313
rs713598	TAS2R38	7	141319814	1	2.192194
rs713598	TAS2R38	7	141319814	3	0.184493
rs1894317	TRY1	7	141601605	1	1.511289
rs7786497	TRY1	7	141603981	5	0.145771
rs361359	TRBV7-3	7	141753068	4	0.352368
rs361359	TRBV7-3	7	141753068	3	0.532319
rs361359	TRBV7-3	7	141753068	5	0.498125
rs9885986	OR9A2	7	142434184	2	1.860529
rs9885986	OR9A2	7	142434184	7	0.428159
SNP7-142546768	PIP	7	142546768	3	0.937527
rs2272251	CLCN1	7	142752959	2	3.660098
rs2272251	CLCN1	7	142752959	3	2.013985
rs1804527	EPHA1	7	142798945	6	0.00956
rs34372369	EPHA1	7	142802391	1	5.526223
rs4595035	TAS2R60	7	142851597	5	0.32647
rs4595035	TAS2R60	7	142851597	2	0.190887
rs10278721	TAS2R41	7	142885467	2	4.562036
rs13229174	OR2F2	7	143263766	5	0.496552
rs2961144	OR2A5	7	143378803	1	1.134108
rs2961144	OR2A5	7	143378803	5	1.915708
rs2961144	OR2A5	7	143378803	4	1.311886
rs6464573	OR2A5	7	143379031	1	0.503688
rs6464573	OR2A5	7	143379031	5	0.75455
rs4407791	OR2A5	7	143379297	6	2.463531
rs4407791	OR2A5	7	143379297	1	2.314851
rs4407791	OR2A5	7	143379297	3	2.421006
rs2961135	OR2A25	7	143402870	3	0.397325
rs10230228	OR2A2	7	143437621	5	1.113824
rs10230228	OR2A2	7	143437621	1	3.475316

rs10230228	OR2A2	7	143437621	2	2.845424
rs10252253	OR2A2	7	143438237	5	3.251972
rs10252253	OR2A2	7	143438237	1	4.154204
rs2961160	OR2A14	7	143457536	3	0.317803
rs2961160	OR2A14	7	143457536	1	2.69519
rs2961160	OR2A14	7	143457536	4	0.197308
rs987456	CNTNAP2	7	147743920	3	3.384512
rs987456	CNTNAP2	7	147743920	5	0.933881
rs1062072	CNTNAP2	7	147745798	6	3.809618
rs1062072	CNTNAP2	7	147745798	7	0.841994
rs2530311	CNTNAP2	7	147747383	6	1.963434
rs10251347	CNTNAP2	7	147748379	1	4.697567
rs2530310	CNTNAP2	7	147748575	4	0.583534
rs17171006	CNTNAP2	7	147748663	1	3.07085
rs822532	RNY5	7	148269156	6	0.712696
rs1137432	ZNF425	7	148432591	6	5.910312
rs1137432	ZNF425	7	148432591	1	4.981713
rs1137432	ZNF425	7	148432591	3	0.272094
rs1058059	ZNF398	7	148510067	1	4.87606
rs1058059	ZNF398	7	148510067	3	0.094019
rs1058059	ZNF398	7	148510067	6	0.289502
rs6977581	ZNF783	7	148621866	6	2.864126
rs1044678	ZNF783	7	148625174	4	4.272526
rs1044678	ZNF783	7	148625174	2	4.711958
rs10224963	LOC729986	7	148752905	2	1.412489
rs10224963	LOC729986	7	148752905	7	0.359814
rs10224963	LOC729986	7	148752905	6	0.091015
rs10224963	LOC729986	7	148752905	1	0.660618
rs4725821	ZNF767	7	148876530	6	0.583473
rs709065	KRBA1	7	149058342	8	0.293541
SNP7-14906130(	KRBA1	7	149061300	4	0.573349
SNP7-14911026ε	SSPO	7	149110268	3	0.450749
SNP7-14911026ε	SSPO	7	149110268	2	3.427392
SNP7-14911417(	SSPO	7	149114170	1	0.406748
SNP7-14911417(	SSPO	7	149114170	2	4.843867
SNP7-14911417(	SSPO	7	149114170	7	0.52654
SNP7-14911417(	SSPO	7	149114170	3	0.60468
SNP7-14911417(	SSPO	7	149114170	8	0.222918
rs855686	NULL	7	149114306	3	0.669646
rs855686	NULL	7	149114306	7	0.596625
rs855686	NULL	7	149114306	8	0.134582
rs855686	NULL	7	149114306	1	0.000205
rs855686	NULL	7	149114306	2	0.189159
rs709060	NULL	7	149115992	1	0.886817
rs709060	NULL	7	149115992	3	0.54973
rs709060	NULL	7	149115992	7	0.246381
rs1076277	NULL	7	149120424	3	0.430117

rs2240362	SSPO	7	149144010	6	2.901879
rs2240362	SSPO	7	149144010	8	2.242618
rs1005603	SSPO	7	149147814	1	0.007726
rs1005603	SSPO	7	149147814	3	0.251342
rs10233245	SSPO	7	149149081	3	1.472874
SNP7-149154128	SSPO	7	149154128	2	3.513316
SNP7-149154128	SSPO	7	149154128	7	0.429096
rs10261977	SSPO	7	149159195	1	0.989229
rs10261977	SSPO	7	149159195	5	1.735668
rs4725833	LOC643641	7	149187451	2	2.215202
rs4725833	LOC643641	7	149187451	7	1.086717
rs2240361	LOC643641	7	149189342	8	4.884886
rs2240361	LOC643641	7	149189342	2	1.804619
rs2074700	LOC643641	7	149190206	8	2.540797
rs2074700	LOC643641	7	149190206	7	1.726722
rs2074700	LOC643641	7	149190206	2	3.473339
rs2074701	LOC643641	7	149190398	8	1.634661
SNP7-149192256	LOC643641	7	149192256	4	0.613368
rs2373465	LOC643641	7	149194067	7	0.756953
SNP7-149207751	ATP6V0E2	7	149207751	7	0.225304
rs3735171	LRRC61	7	149664815	5	0.207843
rs3735171	LRRC61	7	149664815	1	2.242323
rs3735171	LRRC61	7	149664815	3	0.965234
rs3735171	LRRC61	7	149664815	4	0.638186
rs1047575	RARRES2	7	149666433	7	0.87151
rs1047575	RARRES2	7	149666433	5	1.616702
rs1047575	RARRES2	7	149666433	1	4.695273
rs1047575	RARRES2	7	149666433	3	0.607746
rs1047575	RARRES2	7	149666433	4	1.126997
rs4721	RARRES2	7	149666459	3	0.113961
SNP7-149699538	REPIN1	7	149699538	4	0.134851
SNP7-149699538	REPIN1	7	149699538	7	0.551888
SNP7-149699538	REPIN1	7	149699538	2	0.655631
SNP7-149699538	REPIN1	7	149699538	5	0.394491
SNP7-149699538	REPIN1	7	149699538	1	0.959726
rs940453	ZNF775	7	149724634	7	0.495085
rs759011	GIMAP5	7	150070433	4	0.01012
rs759011	GIMAP5	7	150070433	5	2.382439
rs759011	GIMAP5	7	150070433	7	0.953309
rs1046355	GIMAP5	7	150070976	5	0.896228
rs1046355	GIMAP5	7	150070976	7	0.781622
rs2302481	TMEM176B	7	150119401	6	2.111788
rs2302480	TMEM176B	7	150119428	6	5.282907
rs2302479	TMEM176B	7	150119549	2	1.954142
rs2302479	TMEM176B	7	150119549	4	0.841673
rs2072443	TMEM176B	7	150121309	4	0.726445
rs2072443	TMEM176B	7	150121309	8	4.009641

rs2072443	TMEM176B	7	150121309	6	3.783436
SNP7-15013238[illegible]	TMEM176A	7	150132388	7	0.075599
rs9088	TMEM176A	7	150132449	2	3.38964
rs9088	TMEM176A	7	150132449	7	0.283807
rs9088	TMEM176A	7	150132449	5	2.663313
rs1049742	ABP1	7	150185486	3	1.192131
rs10893	ABP1	7	150186848	5	0.652292
rs4725373	ABP1	7	150188555	1	1.303761
rs4725373	ABP1	7	150188555	5	0.127414
SNP7-15018859[illegible]	ABP1	7	150188598	5	3.227226
SNP7-15018859[illegible]	ABP1	7	150188598	1	4.859919
SNP7-15018859[illegible]	ABP1	7	150188598	6	2.104286
rs1805123	KCNH2	7	150276467	8	1.504257
rs1805123	KCNH2	7	150276467	1	0.097389
rs740952	KCNH2	7	150280536	5	0.889537
rs740952	KCNH2	7	150280536	2	4.391972
rs7830	ATG9B	7	150340504	4	0.565732
rs7830	ATG9B	7	150340504	5	0.120094
rs7830	ATG9B	7	150340504	3	1.93332
rs7830	ATG9B	7	150340504	2	1.435511
rs7830	ATG9B	7	150340504	8	2.614566
rs7830	ATG9B	7	150340504	6	5.915855
rs7830	ATG9B	7	150340504	1	0.250595
rs11977275	ACCN3	7	150378870	6	0.097122
rs310597	ASB10	7	150503979	3	0.259089
rs6949587	ABCF2	7	150547161	1	1.438301
rs6949587	ABCF2	7	150547161	3	0.21949
rs7795959	LOC401433	7	150707507	2	2.956052
rs7795959	LOC401433	7	150707507	7	1.420482
rs7795959	LOC401433	7	150707507	1	0.058682
rs7795959	LOC401433	7	150707507	3	0.121697
rs11767693	WDR86	7	150728256	6	1.546592
rs11767693	WDR86	7	150728256	3	0.93352
rs11767693	WDR86	7	150728256	2	3.455872
rs11767693	WDR86	7	150728256	8	3.429373
rs11767693	WDR86	7	150728256	5	0.139377
rs2075001	CRYGN	7	150764204	1	2.821387
rs13224758	LOC644090	7	151135392	6	0.127484
rs13224758	LOC644090	7	151135392	5	0.137315
rs10257529	LOC644090	7	151135432	5	0.705181
rs10257529	LOC644090	7	151135432	3	0.499106
rs4530949	LOC644090	7	151137918	1	3.48466
SNP7-15144140[illegible]	GALNT11	7	151441409	1	4.671039
rs967093	GALNT11	7	151449710	7	0.400174
SNP7-15149061[illegible]	MLL3	7	151490616	6	2.068765
rs7793141	LOC644333	7	152076988	6	2.289121
rs7793141	LOC644333	7	152076988	8	1.506932

rs7793141	LOC644333	7	152076988	1	2.374583
rs7793141	LOC644333	7	152076988	4	2.969773
rs7793141	LOC644333	7	152076988	5	4.081081
rs7793141	LOC644333	7	152076988	3	1.920761
rs7793141	LOC644333	7	152076988	7	2.153509
rs7793141	LOC644333	7	152076988	2	1.823233
rs6962199	FLJ16734	7	153092539	4	0.868455
rs6962199	FLJ16734	7	153092539	7	0.360644
rs6962199	FLJ16734	7	153092539	2	2.414064
rs6962199	FLJ16734	7	153092539	6	0.671696
rs3807218	DPP6	7	154092045	6	1.400513
rs3807218	DPP6	7	154092045	5	2.921303
rs2293353	DPP6	7	154298561	1	2.277073
rs2293353	DPP6	7	154298561	4	0.048681
rs2293353	DPP6	7	154298561	6	1.990156
rs17515800	DPP6	7	154298576	1	2.231742
rs3734961	DPP6	7	154316495	8	0.3503
rs3734961	DPP6	7	154316495	1	2.754552
rs3734961	DPP6	7	154316495	5	1.298091
rs3734961	DPP6	7	154316495	4	1.716847
rs1525752	DPP6	7	154316545	3	0.120777
rs1047064	DPP6	7	154316738	8	1.236684
rs1047064	DPP6	7	154316738	5	1.894722
rs1047064	DPP6	7	154316738	1	4.721087
rs1047064	DPP6	7	154316738	4	3.859449
rs1631327	HTR5A	7	154507622	5	0.13336
rs3735576	NULL	7	155223841	6	0.368871
rs2108192	RBM33	7	155260717	6	0.086483
rs9333594	SHH	7	155297702	7	4.229839
rs9333594	SHH	7	155297702	6	3.043023
rs9333594	SHH	7	155297702	2	3.073548
rs7805578	C7orf13	7	156124492	8	5.492363
SNP7-15616189	RNF32	7	156161894	4	5.879033
rs12007	RNF32	7	156162381	4	3.248411
rs2302443	NOM1	7	156454985	2	1.48834
rs2302443	NOM1	7	156454985	1	0.201612
rs2302443	NOM1	7	156454985	5	0.173133
rs7803794	NOM1	7	156457804	1	1.445946
rs7803794	NOM1	7	156457804	5	3.571501
rs6974109	LOC393078	7	156927990	6	0.624595
rs6974109	LOC393078	7	156927990	3	0.22125
rs6974109	LOC393078	7	156927990	1	0.115215
rs4716746	LOC393078	7	156954053	2	3.544263
rs1049329	PTPRN2	7	157024589	3	1.822828
rs1049325	PTPRN2	7	157024663	3	1.339334
rs221252	PTPRN2	7	157024911	8	0.110341
rs221253	PTPRN2	7	157024933	8	0.359615

rs3189441	PTPRN2	7	157025155	3	1.63303
rs221254	PTPRN2	7	157025680	8	0.024831
rs1130499	PTPRN2	7	157623905	2	1.043096
rs1130499	PTPRN2	7	157623905	1	0.594527
rs1130497	PTPRN2	7	157652640	1	1.687986
rs1130496	PTPRN2	7	157652656	1	0.347357
rs1130496	PTPRN2	7	157652656	6	0.498162
rs1130496	PTPRN2	7	157652656	5	1.009035
rs1130495	PTPRN2	7	157652672	3	1.027697
rs3800855	PTPRN2	7	157677910	4	0.34101
rs3800855	PTPRN2	7	157677910	1	4.714878
rs3800855	PTPRN2	7	157677910	3	0.870557
rs3800855	PTPRN2	7	157677910	7	0.292847
rs4909245	NCAPG2	7	158126493	6	0.132127
rs4909245	NCAPG2	7	158126493	4	3.430826
rs4909245	NCAPG2	7	158126493	3	1.861173
rs4909245	NCAPG2	7	158126493	7	0.534215
rs35914882	FAM62B	7	158221030	6	0.322301
rs35914882	FAM62B	7	158221030	7	0.993328
rs2527202	WDR60	7	158431610	7	1.346894
rs2527202	WDR60	7	158431610	3	1.922926
rs2527202	WDR60	7	158431610	6	0.729442
rs885863	VIPR2	7	158513730	8	1.544731
rs885863	VIPR2	7	158513730	4	4.732852
rs1062610	VIPR2	7	158513936	4	4.490772
rs885861	VIPR2	7	158514308	5	0.640216
rs885861	VIPR2	7	158514308	8	0.378758
rs885861	VIPR2	7	158514308	4	2.291143
rs2730220	VIPR2	7	158514819	3	0.227895
rs763786	ZNF596	8	186884	8	2.993925
rs763786	ZNF596	8	186884	6	3.452249
rs763786	ZNF596	8	186884	7	3.018129
rs3750097	ZNF596	8	187042	6	1.740426
rs3750097	ZNF596	8	187042	8	4.924627
rs3750097	ZNF596	8	187042	7	0.091874
rs10105657	LOC389607	8	597251	8	1.99606
rs10105657	LOC389607	8	597251	3	1.802758
rs10105657	LOC389607	8	597251	5	1.238179
rs10105657	LOC389607	8	597251	2	0.847822
rs10105657	LOC389607	8	597251	1	1.500579
rs10105657	LOC389607	8	597251	4	3.913864
rs11781283	ERICH1	8	671206	7	0.541789
rs11781283	ERICH1	8	671206	3	0.317252
rs2977176	DLGAP2	8	1638203	7	0.049459
rs2977176	DLGAP2	8	1638203	5	0.35649
rs7830545	DLGAP2	8	1643725	1	0.513927
rs7830545	DLGAP2	8	1643725	7	0.163843

rs3812477	NULL	8	1721859	7	0.091926
rs3812477	NULL	8	1721859	1	6.787576
rs3812477	NULL	8	1721859	3	0.406206
rs3812477	NULL	8	1721859	4	0.828914
rs3812477	NULL	8	1721859	6	6.671986
rs1043228	KBTBD11	8	1942219	4	0.027864
rs1043228	KBTBD11	8	1942219	5	2.359946
rs2235122	MYOM2	8	2027705	7	1.186366
rs968381	MYOM2	8	2036238	5	0.533027
rs583087	CSMD1	8	2783242	3	0.587923
rs667859	CSMD1	8	2808152	7	0.14714
rs667859	CSMD1	8	2808152	5	0.074592
rs667595	CSMD1	8	2819546	5	2.414661
rs2161752	CSMD1	8	3188284	4	4.34392
rs1512803	LOC392180	8	5694401	4	2.83992
rs1512803	LOC392180	8	5694401	1	2.875648
rs1512803	LOC392180	8	5694401	5	4.110228
rs3020221	ANGPT2	8	6366171	1	1.875113
rs3020221	ANGPT2	8	6366171	5	0.218162
rs3020221	ANGPT2	8	6366171	4	0.378107
rs3739390	ANGPT2	8	6407898	7	0.12853
rs3739391	ANGPT2	8	6407942	3	0.225867
rs3739391	ANGPT2	8	6407942	7	0.05337
rs2912010	MCPH1	8	6466394	1	0.092111
rs2912010	MCPH1	8	6466394	6	2.641692
rs2912010	MCPH1	8	6466394	3	1.010729
rs2912010	MCPH1	8	6466394	2	1.375712
rs2912010	MCPH1	8	6466394	8	3.102255
rs2433149	MCPH1	8	6488159	2	1.565996
rs2433149	MCPH1	8	6488159	6	4.000735
rs2433149	MCPH1	8	6488159	1	0.194026
rs2433149	MCPH1	8	6488159	4	0.02318
rs17078203	XKR5	8	6653898	1	1.873154
rs2741098	XKR5	8	6677686	3	0.585057
rs2741098	XKR5	8	6677686	1	1.008234
rs2741098	XKR5	8	6677686	6	0.045184
rs2741098	XKR5	8	6677686	7	5.248542
rs1800971	DEFB1	8	6715526	3	0.572223
rs1800971	DEFB1	8	6715526	8	1.901454
rs1800972	DEFB1	8	6722833	5	0.508729
rs4840952	PRAGMIN	8	8271487	3	0.199195
rs4840952	PRAGMIN	8	8271487	6	0.265473
rs4840952	PRAGMIN	8	8271487	1	5.186792
rs2921005	PRAGMIN	8	8271523	4	5.969667
rs4840953	PRAGMIN	8	8271602	3	0.177306
rs4840953	PRAGMIN	8	8271602	1	5.266657
rs4840953	PRAGMIN	8	8271602	6	1.321812

SNP8-8272124	PRAGMIN	8	8272124	3	0.47566
SNP8-8272124	PRAGMIN	8	8272124	1	4.861032
rs9644774	CLDN23	8	8597242	6	0.605248
rs9644774	CLDN23	8	8597242	1	0.121836
rs12153	CLDN23	8	8598319	5	6.619199
rs12153	CLDN23	8	8598319	4	5.788966
rs12153	CLDN23	8	8598319	3	0.756286
rs2288672	THEX1	8	8898030	4	0.008624
rs2288672	THEX1	8	8898030	1	4.161806
rs11777327	PPP1R3B	8	9033739	8	3.878113
rs330922	PPP1R3B	8	9035208	1	4.169129
rs330922	PPP1R3B	8	9035208	3	0.718059
rs35052906	TNKS	8	9605110	5	0.286344
rs13265931	TNKS	8	9646741	3	0.876518
rs4841215	TNKS	8	9675718	5	0.72229
rs7816990	RP1L1	8	10501354	1	3.470366
rs7816990	RP1L1	8	10501354	3	0.175083
rs7816990	RP1L1	8	10501354	5	0.14287
rs12542071	RP1L1	8	10501571	1	2.614762
rs12542075	RP1L1	8	10501616	1	1.270442
rs12542104	RP1L1	8	10501717	3	0.191399
rs12542104	RP1L1	8	10501717	1	0.864698
rs4840499	RP1L1	8	10504986	7	0.325537
rs1139843	SOX7	8	10619507	1	6.514416
rs3021494	C8orf15	8	11020944	1	5.254822
rs3021494	C8orf15	8	11020944	6	0.969352
rs2164272	MTMR9	8	11199906	1	9.114264
rs2164272	MTMR9	8	11199906	6	1.064917
rs6995404	MTMR9	8	11219558	1	4.936868
rs6995404	MTMR9	8	11219558	8	0.967213
rs6995404	MTMR9	8	11219558	5	4.587377
rs3021511	C8orf13	8	11318295	4	0.652221
rs3021511	C8orf13	8	11318295	1	2.38675
rs2409764	C8orf13	8	11318683	1	1.084142
rs2409764	C8orf13	8	11318683	6	2.777294
rs2409764	C8orf13	8	11318683	2	0.566333
rs2409764	C8orf13	8	11318683	7	1.436047
rs922483	BLK	8	11389321	3	0.235049
rs1042689	BLK	8	11459202	1	0.040735
rs1042689	BLK	8	11459202	5	0.23488
rs1042689	BLK	8	11459202	3	0.62606
rs1062219	GATA4	8	11653819	6	2.3834
rs1062219	GATA4	8	11653819	7	1.314667
rs12458	GATA4	8	11654649	7	0.966684
rs12458	GATA4	8	11654649	1	2.077813
rs1534862	NEIL2	8	11681212	6	3.040343
rs1534862	NEIL2	8	11681212	4	1.167279

rs1534862	NEIL2	8	11681212	7	1.89204
rs1534862	NEIL2	8	11681212	3	3.17412
rs1534862	NEIL2	8	11681212	1	3.857031
rs8191667	NEIL2	8	11681225	4	0.943478
rs8191667	NEIL2	8	11681225	1	4.989157
rs8191667	NEIL2	8	11681225	7	0.291235
rs709821	CTSB	8	11740003	7	0.292017
rs709821	CTSB	8	11740003	1	3.076564
rs1139354	LONRF1	8	12645091	3	0.020504
rs622106	C8orf79	8	12923048	5	1.262872
rs622106	C8orf79	8	12923048	2	0.368819
SNP8-12923178	C8orf79	8	12923178	8	0.879342
SNP8-12923178	C8orf79	8	12923178	7	0.058378
SNP8-12923178	C8orf79	8	12923178	2	0.175336
SNP8-12923178	C8orf79	8	12923178	1	0.555759
SNP8-12923178	C8orf79	8	12923178	5	0.672555
rs3739308	C8orf79	8	12923353	4	3.927506
SNP8-12923470	C8orf79	8	12923470	7	0.250261
rs608909	C8orf79	8	12923705	7	0.147429
rs608909	C8orf79	8	12923705	5	0.475091
rs608909	C8orf79	8	12923705	2	4.203588
rs608052	C8orf79	8	12923910	7	0.205828
rs475181	C8orf79	8	12924148	5	1.907913
rs475181	C8orf79	8	12924148	7	0.168482
rs475181	C8orf79	8	12924148	2	2.463426
rs472347	C8orf79	8	12924476	2	2.696785
rs579024	C8orf79	8	12924892	2	0.975281
rs578076	C8orf79	8	12924999	1	1.446407
rs1044011	DLC1	8	12986080	4	0.425828
rs1044011	DLC1	8	12986080	1	3.622943
rs2027	DLC1	8	12986713	1	3.403709
rs486596	DLC1	8	12986881	3	0.229249
rs658856	DLC1	8	12996664	1	4.152335
rs658856	DLC1	8	12996664	6	1.071767
rs13273355	C8orf48	8	13468954	6	0.430567
rs9650391	MSR1	8	16010913	3	0.575564
rs17624989	EFHA2	8	17024400	3	0.010413
rs17624989	EFHA2	8	17024400	8	0.436072
rs4921756	MTMR7	8	17243324	2	1.520055
rs4921756	MTMR7	8	17243324	6	0.410344
rs6586610	SLC7A2	8	17467787	5	0.993739
rs6586610	SLC7A2	8	17467787	2	3.700063
rs10481	MTUS1	8	17545845	8	0.213092
rs1043883	MTUS1	8	17547711	6	0.80238
rs1043883	MTUS1	8	17547711	7	9.300392
rs1043883	MTUS1	8	17547711	4	1.894869
rs1043883	MTUS1	8	17547711	2	4.109464

rs1043883	MTUS1	8	17547711	1	0.03988
rs1043883	MTUS1	8	17547711	3	0.249459
rs437548	MTUS1	8	17599309	3	0.316163
rs437548	MTUS1	8	17599309	6	0.469813
rs10283	PCM1	8	17929686	8	0.260259
rs10283	PCM1	8	17929686	7	1.231635
rs10283	PCM1	8	17929686	1	4.975633
rs13263632	ASAH1	8	17986484	7	0.296047
rs1041983	NAT2	8	18302075	3	0.392723
rs1041983	NAT2	8	18302075	5	0.008328
rs1799929	NAT2	8	18302274	2	3.08426
rs1799930	NAT2	8	18302383	5	0.546994
rs8695	PSD3	8	18432534	3	3.039732
rs901934	PSD3	8	18435454	7	0.118283
rs2306825	PSD3	8	18701085	6	0.504076
rs2306825	PSD3	8	18701085	7	0.313334
rs2306825	PSD3	8	18701085	3	1.124304
rs13263453	PSD3	8	18773776	3	12.08592
rs13263453	PSD3	8	18773776	4	5.925835
SNP8-18774097	PSD3	8	18774097	5	0.690658
SNP8-18774097	PSD3	8	18774097	1	2.664828
SNP8-18774097	PSD3	8	18774097	3	0.454231
rs1574288	SH2D4A	8	19234771	4	0.199158
rs1574288	SH2D4A	8	19234771	3	0.196497
rs1574288	SH2D4A	8	19234771	5	3.625438
SNP8-19265980	SH2D4A	8	19265980	5	5.056275
SNP8-19265980	SH2D4A	8	19265980	2	9.271598
SNP8-19265980	SH2D4A	8	19265980	4	0.30582
SNP8-19849988	LPL	8	19849988	8	2.514211
rs1121923	LPL	8	19853715	6	1.218205
rs4922115	LPL	8	19867110	4	2.231426
rs4922115	LPL	8	19867110	1	0.101546
rs4922115	LPL	8	19867110	5	1.961666
rs4922115	LPL	8	19867110	8	2.142736
rs3208305	LPL	8	19867928	1	0.418774
rs3208305	LPL	8	19867928	8	4.109933
rs3208305	LPL	8	19867928	5	0.558568
rs3208305	LPL	8	19867928	2	0.891428
rs3208305	LPL	8	19867928	4	0.046821
rs15285	LPL	8	19868947	5	0.396172
rs15285	LPL	8	19868947	4	0.222792
rs15285	LPL	8	19868947	1	0.622603
rs2270637	SLC18A1	8	20081107	5	0.603869
rs2270637	SLC18A1	8	20081107	3	1.133658
rs2270637	SLC18A1	8	20081107	1	0.434316
rs2270637	SLC18A1	8	20081107	6	0.473323
rs1075	LZTS1	8	20149265	3	0.074829

rs1075	LZTS1	8	20149265	4	0.657388
rs732338	LZTS1	8	20150660	4	1.231077
rs732338	LZTS1	8	20150660	7	0.539596
rs732338	LZTS1	8	20150660	2	1.488075
rs904002	LZTS1	8	20151154	7	1.375592
rs732337	LZTS1	8	20151170	7	0.264427
rs2306646	XPO7	8	21902532	4	0.326256
rs2306646	XPO7	8	21902532	7	0.008739
rs2306646	XPO7	8	21902532	8	0.370141
SNP8-21995513	EPB49	8	21995513	4	5.469898
SNP8-22034523	HR	8	22034523	4	1.47731
SNP8-22040710	HR	8	22040710	6	1.338177
SNP8-22040710	HR	8	22040710	4	0.012808
rs35574275	REEP4	8	22053683	8	7.473813
rs35574275	REEP4	8	22053683	6	0.5514
rs11784926	BMP1	8	22114849	4	0.039486
rs11996036	BMP1	8	22115308	6	3.698238
rs1126677	POLR3D	8	22161447	8	1.445598
rs1126677	POLR3D	8	22161447	3	0.945313
rs1126677	POLR3D	8	22161447	1	3.811945
rs1126677	POLR3D	8	22161447	4	0.475843
rs1126677	POLR3D	8	22161447	5	0.848243
rs1049437	PPP3CC	8	22354602	8	1.633864
rs1049437	PPP3CC	8	22354602	4	0.325077
rs1049437	PPP3CC	8	22354602	5	0.647722
rs7430	PPP3CC	8	22454359	1	4.467474
rs7430	PPP3CC	8	22454359	6	6.922943
rs7430	PPP3CC	8	22454359	4	10.47275
rs2449331	SORBS3	8	22484603	6	4.062036
rs3735893	PDLIM2	8	22508649	1	1.634502
rs3735893	PDLIM2	8	22508649	5	0.822734
rs11785755	PDLIM2	8	22509168	1	1.783516
rs11136091	PDLIM2	8	22509873	3	0.275429
rs3735894	PDLIM2	8	22510771	1	2.501457
rs3735894	PDLIM2	8	22510771	5	0.546874
rs7843828	KIAA1967	8	22519568	1	2.146762
rs7843828	KIAA1967	8	22519568	5	9.226782
rs3736147	KIAA1967	8	22527769	1	5.304331
rs3736147	KIAA1967	8	22527769	5	0.004686
SNP8-22582504	BIN3	8	22582504	3	8.032344
SNP8-22582504	BIN3	8	22582504	6	4.892838
rs3853	PEBP4	8	22626789	1	2.603645
rs1047406	PEBP4	8	22626880	7	0.147889
rs1047406	PEBP4	8	22626880	3	0.251264
rs1047406	PEBP4	8	22626880	5	0.295067
rs1047406	PEBP4	8	22626880	1	0.812015
rs1047406	PEBP4	8	22626880	2	0.013413

rs1047406	PEBP4	8	22626880	4	0.045669
rs1047398	PEBP4	8	22626963	4	0.020172
rs1047398	PEBP4	8	22626963	1	0.170962
rs1047398	PEBP4	8	22626963	7	2.936326
rs1047398	PEBP4	8	22626963	5	0.352581
rs1047398	PEBP4	8	22626963	3	1.474395
rs1129474	PEBP4	8	22640663	5	0.532559
rs1129474	PEBP4	8	22640663	6	0.133066
rs1129474	PEBP4	8	22640663	4	1.034763
rs1129474	PEBP4	8	22640663	2	3.177847
rs1047266	TNFRSF10B	8	22956646	8	7.203596
rs34941234	TNFRSF10D	8	23049053	7	1.826732
SNP8-23058035	TNFRSF10D	8	23058035	5	0.043725
SNP8-23058035	TNFRSF10D	8	23058035	3	0.625277
SNP8-23077291	TNFRSF10D	8	23077291	1	3.72176
SNP8-23077291	TNFRSF10D	8	23077291	8	7.458174
rs20576	TNFRSF10A	8	23114165	4	2.495361
rs20576	TNFRSF10A	8	23114165	2	1.66751
rs4871857	TNFRSF10A	8	23115269	4	0.719122
rs6557634	TNFRSF10A	8	23116201	4	0.465734
rs4563894	CHMP7	8	23159818	3	0.660496
rs4563894	CHMP7	8	23159818	1	1.889869
rs4581062	CHMP7	8	23159856	3	0.256004
rs2294123	CHMP7	8	23160140	3	0.831172
rs2294123	CHMP7	8	23160140	8	0.918685
rs12155720	CHMP7	8	23174388	1	3.765719
rs7813349	LOXL2	8	23210545	5	0.698719
rs7813349	LOXL2	8	23210545	8	4.953985
rs7813349	LOXL2	8	23210545	6	5.774639
rs7813349	LOXL2	8	23210545	7	1.50089
rs2280936	LOXL2	8	23211282	5	0.398518
rs2280936	LOXL2	8	23211282	8	0.222681
rs1051146	LOXL2	8	23241952	1	4.563949
rs1051146	LOXL2	8	23241952	3	0.20098
rs1051146	LOXL2	8	23241952	8	0.199978
rs2272641	ENTPD4	8	23350706	3	0.541986
rs3736032	SLC25A37	8	23479642	2	4.25024
rs6996616	ADAM28	8	24226996	1	5.504597
rs2291577	ADAMDEC1	8	24312415	1	0.366908
rs3765124	ADAMDEC1	8	24317471	1	0.523965
rs2709618	DOCK5	8	25323539	5	7.861769
rs2709618	DOCK5	8	25323539	3	0.257417
rs3812422	CDCA2	8	25372578	8	1.471078
rs3812422	CDCA2	8	25372578	5	0.592467
rs3812422	CDCA2	8	25372578	6	0.293922
rs3812421	CDCA2	8	25372706	6	4.854229
rs3812421	CDCA2	8	25372706	5	0.972156

rs3812421	CDCA2	8	25372706	8	0.57232
rs10866845	EBF2	8	25764184	5	0.396803
rs2197181	PNMA2	8	26418944	1	3.253132
rs2197181	PNMA2	8	26418944	3	0.011271
rs2197181	PNMA2	8	26418944	6	0.756686
rs2197181	PNMA2	8	26418944	5	0.530049
rs2233699	PNMA2	8	26422670	5	0.342016
rs2233699	PNMA2	8	26422670	3	0.33923
rs3739214	STMN4	8	27157196	6	0.782825
rs3739214	STMN4	8	27157196	3	0.262285
rs4279551	TRIM35	8	27200649	4	0.192056
rs4279551	TRIM35	8	27200649	7	0.176196
rs4279551	TRIM35	8	27200649	5	0.159123
rs6988218	PTK2B	8	27225047	1	0.141313
rs6988218	PTK2B	8	27225047	2	2.736598
rs6988218	PTK2B	8	27225047	4	3.15166
rs6988218	PTK2B	8	27225047	3	1.920684
rs6988218	PTK2B	8	27225047	7	0.270689
rs6988218	PTK2B	8	27225047	6	0.057605
rs6988218	PTK2B	8	27225047	8	0.333889
rs6988218	PTK2B	8	27225047	5	0.258526
rs2241649	PTK2B	8	27310997	3	0.766239
rs2241649	PTK2B	8	27310997	7	1.161209
rs2241649	PTK2B	8	27310997	6	1.814946
rs2241649	PTK2B	8	27310997	2	1.031008
rs2241649	PTK2B	8	27310997	1	1.069037
rs1045511	PTK2B	8	27311063	7	5.835948
rs1045511	PTK2B	8	27311063	2	4.721864
rs1045511	PTK2B	8	27311063	6	1.08468
rs1045511	PTK2B	8	27311063	1	0.61151
rs1045511	PTK2B	8	27311063	3	9.363717
rs45578935	PTK2B	8	27357642	7	1.206471
rs751019	PTK2B	8	27364502	8	1.334172
rs751019	PTK2B	8	27364502	7	0.515068
rs751019	PTK2B	8	27364502	6	1.820634
rs751019	PTK2B	8	27364502	1	3.797734
rs751019	PTK2B	8	27364502	3	0.371135
rs2292974	CHRNA2	8	27374308	3	1.844716
rs2292974	CHRNA2	8	27374308	6	0.908488
rs2292974	CHRNA2	8	27374308	7	2.554671
rs2292974	CHRNA2	8	27374308	8	1.936549
rs2292974	CHRNA2	8	27374308	1	2.981935
rs2292975	CHRNA2	8	27374342	7	3.158539
rs2292975	CHRNA2	8	27374342	3	3.503073
rs2292975	CHRNA2	8	27374342	6	1.641496
rs2292975	CHRNA2	8	27374342	1	3.087733
rs2292977	CHRNA2	8	27374552	2	0.550091

rs891398	CHRNA2	8	27380739	4	1.3384
rs751141	EPHX2	8	27429782	8	6.503865
rs9331947	CLU	8	27510794	1	2.412086
rs17057523	SCARA3	8	27584365	3	0.862662
rs17057523	SCARA3	8	27584365	5	0.66028
rs485609	SCARA3	8	27586295	3	0.133403
rs485609	SCARA3	8	27586295	2	4.737588
rs485609	SCARA3	8	27586295	8	0.059159
rs485609	SCARA3	8	27586295	7	1.265585
rs7842666	CCDC25	8	27686071	7	0.163665
rs7842666	CCDC25	8	27686071	4	1.134901
rs7842666	CCDC25	8	27686071	5	0.149491
rs1052874	PBK	8	27723638	3	0.947605
rs1052874	PBK	8	27723638	2	6.916257
rs1052874	PBK	8	27723638	8	6.534574
rs1052874	PBK	8	27723638	7	0.708625
rs2685390	SCARA5	8	27906080	3	0.153434
rs1046511	ZNF395	8	28262194	5	7.616172
rs1046511	ZNF395	8	28262194	1	3.162458
rs1046511	ZNF395	8	28262194	7	0.004176
rs6986672	KIF13B	8	29061012	1	3.792399
rs6986672	KIF13B	8	29061012	6	1.014274
rs13271770	DUSP4	8	29259110	6	0.425966
rs1455915	DUSP4	8	29262045	8	0.188855
rs2637819	DUSP4	8	29263791	4	0.257278
rs2637819	DUSP4	8	29263791	2	3.918747
rs7844812	MBOAT4	8	30109106	6	0.480578
rs7844812	MBOAT4	8	30109106	5	0.906886
SNP8-30822280	TEX15	8	30822280	4	0.267812
rs1800391	WRN	8	31058246	7	0.085532
rs10954782	LOC642513	8	31196182	1	1.187192
rs10954782	LOC642513	8	31196182	4	1.018096
rs11785668	NRG1	8	32705534	5	0.266023
rs16880994	FUT10	8	33430477	3	0.064755
rs10644	RBM13	8	33477704	2	5.967345
rs10644	RBM13	8	33477704	5	1.771223
rs10644	RBM13	8	33477704	6	5.586212
rs3735951	RNF122	8	33535764	7	0.25774
rs3735951	RNF122	8	33535764	2	4.84973
rs3735951	RNF122	8	33535764	5	0.387707
rs3735951	RNF122	8	33535764	6	0.22712
rs3735952	RNF122	8	33544088	6	3.441934
rs11776875	LOC642879	8	37072804	4	0.759445
rs4994	ADRB3	8	37942955	5	1.332855
rs35045158	STAR	8	38120492	1	3.1105
rs2234558	WHSC1L1	8	38295592	6	6.207902
rs3925	FGFR1	8	38400815	8	2.108261

rs3925	FGFR1	8	38400815	3	0.697368
rs3925	FGFR1	8	38400815	7	0.516598
rs2304000	FGFR1	8	38405968	7	0.2102
rs2304000	FGFR1	8	38405968	8	2.128108
rs2304000	FGFR1	8	38405968	3	0.047394
rs4733951	FLJ43582	8	38488988	5	0.190834
SNP8-38796479	TACC1	8	38796479	1	6.182807
rs1802267	TACC1	8	38825526	1	3.780039
rs1135990	TACC1	8	38828710	1	4.866418
rs1135990	TACC1	8	38828710	6	1.08966
rs10105311	TM2D2	8	38973198	6	1.00102
rs10105311	TM2D2	8	38973198	3	1.734968
rs10105311	TM2D2	8	38973198	5	1.075971
rs10199	C8orf4	8	40131220	1	1.620236
rs753133	ZMAT4	8	40507475	5	0.565959
rs17851751	ZMAT4	8	40557914	5	0.457086
rs17851751	ZMAT4	8	40557914	1	1.229408
rs17851751	ZMAT4	8	40557914	7	1.376834
rs11337	GOLGA7	8	41487443	2	5.487799
rs508419	ANK1	8	41642148	2	0.219181
rs750625	ANK1	8	41645071	2	4.690162
rs504574	ANK1	8	41673085	5	0.021275
rs504574	ANK1	8	41673085	1	0.042231
rs2304880	ANK1	8	41678766	2	2.493177
rs2304880	ANK1	8	41678766	3	1.656645
rs2304880	ANK1	8	41678766	1	1.231781
rs2304880	ANK1	8	41678766	8	1.303731
rs2304873	ANK1	8	41702451	2	3.079811
rs2304871	ANK1	8	41704595	5	3.013699
rs2304871	ANK1	8	41704595	3	2.736891
rs3739365	MYST3	8	41910440	3	0.205419
rs7006698	MYST3	8	42025851	2	3.013747
rs7006698	MYST3	8	42025851	1	0.06831
rs7006698	MYST3	8	42025851	4	1.084489
rs1136159	PLAT	8	42155734	4	1.467339
rs1136159	PLAT	8	42155734	1	0.966707
rs6841	SLC20A2	8	42393421	8	0.961734
rs8178216	PRKDC	8	48882397	4	1.542498
rs8178046	PRKDC	8	49004261	8	6.088364
rs7827446	PXDNL	8	52395961	2	4.869016
rs2977020	PXDNL	8	52483796	4	2.290398
rs2278152	ST18	8	53234014	7	0.249847
SNP8-53749259	RB1CC1	8	53749259	4	7.455423
SNP8-53749259	RB1CC1	8	53749259	8	5.13316
SNP8-53749259	RB1CC1	8	53749259	7	0.157944
rs11992022	OPRK1	8	54300844	3	0.294376
rs7832417	OPRK1	8	54301171	3	0.173841

rs7832417	OPRK1	8	54301171	8	1.418505
rs7832417	OPRK1	8	54301171	6	0.503993
rs16918842	OPRK1	8	54301377	6	1.334342
rs3802279	OPRK1	8	54303501	6	0.649345
rs3802279	OPRK1	8	54303501	3	0.740425
rs3802280	OPRK1	8	54303552	6	0.22085
rs3802280	OPRK1	8	54303552	3	0.257545
rs3802281	OPRK1	8	54303716	3	1.060327
rs3802282	OPRK1	8	54303757	6	2.510038
rs3802282	OPRK1	8	54303757	3	1.016765
rs963549	OPRK1	8	54304377	3	1.974292
rs963549	OPRK1	8	54304377	6	0.592434
rs702764	OPRK1	8	54304710	6	0.519995
rs702764	OPRK1	8	54304710	3	0.04356
rs7820949	SEC11B	8	55598186	7	3.456994
rs7820949	SEC11B	8	55598186	1	4.145486
rs7820949	SEC11B	8	55598186	5	3.724943
rs7820949	SEC11B	8	55598186	8	2.424565
rs444772	RP1	8	55701610	2	4.606011
rs444772	RP1	8	55701610	5	0.121435
rs2293869	RP1	8	55701948	3	0.750357
rs2293869	RP1	8	55701948	1	0.061037
rs2293869	RP1	8	55701948	4	0.339986
rs2293869	RP1	8	55701948	5	1.841283
rs2293869	RP1	8	55701948	6	0.838001
rs2293869	RP1	8	55701948	2	0.037363
rs446227	RP1	8	55704003	5	0.44231
rs446227	RP1	8	55704003	2	2.399114
rs446227	RP1	8	55704003	8	2.471356
rs441800	RP1	8	55704170	8	0.417582
rs441800	RP1	8	55704170	5	0.051907
SNP8-55705093	RP1	8	55705093	3	0.771028
SNP8-55705093	RP1	8	55705093	6	0.420072
SNP8-55705093	RP1	8	55705093	4	0.18066
SNP8-55705093	RP1	8	55705093	1	0.561045
SNP8-55705093	RP1	8	55705093	5	4.957068
rs7464499	TMEM68	8	56839089	6	0.048354
rs13276393	TGS1	8	56848487	5	0.034347
rs13276393	TGS1	8	56848487	6	2.684452
rs1818	TGS1	8	56848778	5	5.188852
rs2976047	RPS20	8	57148368	6	3.683661
rs2976047	RPS20	8	57148368	3	0.282345
rs35883156	PLAG1	8	57241487	3	0.927999
SNP8-60014461	TOX	8	60014461	8	1.078331
rs3140	CA8	8	61264264	1	6.175226
rs3140	CA8	8	61264264	4	0.764807
rs3140	CA8	8	61264264	7	5.576098

rs6999971	CHD7	8	61927973	6	0.730736
rs4739003	NKAIN3	8	63822189	1	0.418351
rs4739003	NKAIN3	8	63822189	2	0.321486
rs4739003	NKAIN3	8	63822189	7	0.139609
rs16930023	NULL	8	64055139	5	0.811974
rs16930023	NULL	8	64055139	4	3.425673
rs11545078	GGH	8	64101318	1	2.922189
SNP8-64113866	GGH	8	64113866	3	0.02282
SNP8-64113866	GGH	8	64113866	4	0.6552
SNP8-64113866	GGH	8	64113866	2	5.004317
SNP8-64113866	GGH	8	64113866	6	0.108323
rs9650210	BHLHB5	8	65658613	1	0.583468
rs1053088	ARMC1	8	66678337	4	1.903119
rs1053088	ARMC1	8	66678337	7	2.378813
rs7011464	PDE7A	8	66797841	7	0.668201
rs7011464	PDE7A	8	66797841	4	0.126214
rs3739334	RRS1	8	67503932	7	0.604979
SNP8-67543082	ADHFE1	8	67543082	8	1.263145
SNP8-67543082	ADHFE1	8	67543082	2	3.240024
SNP8-67543082	ADHFE1	8	67543082	7	0.412804
rs12265	ADHFE1	8	67543291	2	6.621294
rs1108	ADHFE1	8	67543417	8	1.169091
rs1108	ADHFE1	8	67543417	7	0.748227
rs17332410	C8orf45	8	67958674	6	0.213605
rs4260880	DEPDC2	8	69183050	1	1.042333
rs4260880	DEPDC2	8	69183050	6	1.857545
rs4260880	DEPDC2	8	69183050	5	1.245698
rs2289831	C8orf34	8	69596749	2	2.018174
rs16935065	C8orf34	8	69862310	7	0.032617
rs13264163	SULF1	8	70576714	7	1.143665
rs3802278	SULF1	8	70733867	6	0.730742
rs3802278	SULF1	8	70733867	1	4.59415
rs6990375	SULF1	8	70734085	6	0.138098
rs6990375	SULF1	8	70734085	1	3.309761
rs10448034	SLCO5A1	8	70751432	3	0.922532
rs10448034	SLCO5A1	8	70751432	7	1.437925
rs3750266	SLCO5A1	8	70907366	3	0.46591
rs10089937	PRDM14	8	71126953	4	0.885679
rs10089937	PRDM14	8	71126953	1	2.439875
rs10089937	PRDM14	8	71126953	2	2.406997
rs10089937	PRDM14	8	71126953	3	2.349999
rs10089937	PRDM14	8	71126953	5	2.272128
rs12681420	XKR9	8	71755752	4	2.323685
rs12681420	XKR9	8	71755752	3	2.743438
rs6991329	XKR9	8	71755979	8	2.539664
rs6991329	XKR9	8	71755979	4	3.604714
rs6991329	XKR9	8	71755979	3	2.051314

rs2290702	XKR9	8	71809534	3	2.246223
rs4738118	EYA1	8	72291563	8	4.307797
rs900126	MSC	8	72916896	1	1.210869
rs900126	MSC	8	72916896	7	0.091933
rs900126	MSC	8	72916896	6	0.574001
rs900126	MSC	8	72916896	3	0.288833
rs3779760	MSC	8	72917193	5	0.150299
rs3779760	MSC	8	72917193	3	0.392566
rs3779760	MSC	8	72917193	8	4.746916
rs3779760	MSC	8	72917193	2	0.375349
rs3779761	MSC	8	72917302	7	0.353621
rs2044270	TRPA1	8	73096328	3	0.030079
rs13280644	TRPA1	8	73111142	6	0.858588
rs13280644	TRPA1	8	73111142	3	1.464497
rs3735943	TRPA1	8	73128556	7	3.320051
rs3735943	TRPA1	8	73128556	1	1.368635
rs16937976	TRPA1	8	73146595	5	0.411508
rs16937976	TRPA1	8	73146595	1	0.232525
rs16937976	TRPA1	8	73146595	6	1.107644
rs13268757	TRPA1	8	73150192	6	1.121466
SNP8-74167685	RPESP	8	74167685	5	6.151323
SNP8-74167685	RPESP	8	74167685	4	0.257269
rs7819319	NULL	8	74527921	7	0.137134
rs7819319	NULL	8	74527921	5	0.319304
rs7819319	NULL	8	74527921	4	0.568005
rs2291217	TMEM70	8	75057302	4	0.013692
rs2291217	TMEM70	8	75057302	2	0.350063
rs2291217	TMEM70	8	75057302	1	0.505682
rs2291217	TMEM70	8	75057302	6	2.191292
rs2291217	TMEM70	8	75057302	8	0.697189
rs2291217	TMEM70	8	75057302	5	4.121328
rs7835278	PI15	8	75928502	5	0.668489
rs2085276	CRISPLD1	8	76108405	3	0.401118
rs2085276	CRISPLD1	8	76108405	1	4.667213
rs2085276	CRISPLD1	8	76108405	5	5.213248
rs13248650	CRISPLD1	8	76109250	4	0.863702
rs13248650	CRISPLD1	8	76109250	7	0.01274
rs13248650	CRISPLD1	8	76109250	8	0.312746
rs2272669	HNF4G	8	76639012	4	0.260037
rs2272669	HNF4G	8	76639012	8	0.913259
rs2272669	HNF4G	8	76639012	6	0.553566
rs2941477	HNF4G	8	76639225	4	0.777573
rs2941477	HNF4G	8	76639225	6	1.353883
rs2941478	HNF4G	8	76639292	2	1.251747
rs2941478	HNF4G	8	76639292	1	0.715608
rs2941478	HNF4G	8	76639292	5	0.306609
rs2941479	HNF4G	8	76639514	7	0.031019

rs2941479	HNF4G	8	76639514	8	0.60387
rs2941480	HNF4G	8	76639677	7	0.615302
rs2941480	HNF4G	8	76639677	8	1.549879
rs2941480	HNF4G	8	76639677	2	4.187472
rs2941481	HNF4G	8	76639923	6	2.874902
rs2941481	HNF4G	8	76639923	8	3.371664
rs2941481	HNF4G	8	76639923	4	0.136316
rs17306807	HNF4G	8	76640084	4	0.274853
rs17306807	HNF4G	8	76640084	6	1.462865
rs2941482	HNF4G	8	76640550	6	1.159574
rs2941483	HNF4G	8	76641171	7	0.269566
rs2941484	HNF4G	8	76641323	4	0.0606
rs2941484	HNF4G	8	76641323	2	1.877497
SNP8-77779074	ZFHX4	8	77779074	6	5.752803
SNP8-77779074	ZFHX4	8	77779074	3	0.511157
rs2728455	ZFHX4	8	77928420	6	0.975251
rs2728455	ZFHX4	8	77928420	2	1.974922
rs2728455	ZFHX4	8	77928420	5	0.014512
rs2728455	ZFHX4	8	77928420	7	1.194703
rs1046472	HEY1	8	80839084	4	1.04793
SNP8-81104896	MRPS28	8	81104896	4	4.526748
rs7464898	LOC646437	8	81652979	7	0.582904
rs7464898	LOC646437	8	81652979	1	1.351205
rs10755966	ZNF704	8	81714207	3	0.27354
rs10755966	ZNF704	8	81714207	8	0.852601
rs10755966	ZNF704	8	81714207	2	1.782414
rs11743	PAG1	8	82042651	5	1.766558
rs3739342	PAG1	8	82045405	8	0.152376
rs1445560	PAG1	8	82047632	4	0.166919
rs7017984	PAG1	8	82049241	2	2.339281
rs2016465	PAG1	8	82067952	6	0.857932
rs2167255	PMP2	8	82515633	6	2.698261
rs2167255	PMP2	8	82515633	3	4.605055
rs10113055	PMP2	8	82516711	1	2.880318
rs6473276	PMP2	8	82518176	3	1.628282
rs2229015	PMP2	8	82519667	3	0.649292
SNP8-82833326	CHMP4C	8	82833326	7	1.74708
rs13267859	RALYL	8	85995723	7	0.062219
rs12604	RALYL	8	85996372	8	3.5567
rs2072696	CA3	8	86538303	4	0.956914
rs2072696	CA3	8	86538303	2	3.397774
rs2072696	CA3	8	86538303	7	0.304179
rs16876805	PSKH2	8	87129959	4	0.475139
rs6998760	PSKH2	8	87145636	6	1.09295
rs6998760	PSKH2	8	87145636	5	0.256772
rs6983781	ATP6V0D2	8	87235512	5	0.883513
rs6983781	ATP6V0D2	8	87235512	1	1.716919

SNP8-87590010	FAM82B	8	87590010	3	0.815908
SNP8-87590010	FAM82B	8	87590010	6	4.404978
rs990192	CNGB3	8	87655509	5	2.743769
rs17683284	CNGB3	8	87655845	3	0.651343
rs6471482	CNGB3	8	87748419	3	0.098895
rs1805794	NBN	8	91059655	1	5.95845
rs1055146	NECAB1	8	92038845	1	3.286178
rs1055146	NECAB1	8	92038845	5	0.914642
rs10109254	SLC26A7	8	92433276	1	3.141233
rs16912814	SLC26A7	8	92477506	7	0.27312
rs4734031	SLC26A7	8	92478223	7	0.351432
rs4734032	SLC26A7	8	92478246	7	0.132266
rs16916191	RBM12B	8	94818741	2	1.783832
rs16916191	RBM12B	8	94818741	7	0.433139
rs2513797	CDH17	8	95212314	1	2.236493
rs2513797	CDH17	8	95212314	7	0.420256
rs2513797	CDH17	8	95212314	5	0.784859
rs1051624	CDH17	8	95212348	1	2.471249
rs2251734	CDH17	8	95227435	1	2.586688
rs2251741	CDH17	8	95227558	1	1.701805
SNP8-95258026	CDH17	8	95258026	2	2.590622
SNP8-95258026	CDH17	8	95258026	7	0.990325
SNP8-95258026	CDH17	8	95258026	3	0.744671
SNP8-95258026	CDH17	8	95258026	6	0.116292
rs2623167	GEM	8	95330858	6	2.24304
rs2623167	GEM	8	95330858	2	2.118246
rs1050627	GEM	8	95330869	5	0.273521
rs1050627	GEM	8	95330869	1	0.207952
rs1050627	GEM	8	95330869	6	0.916322
rs1050627	GEM	8	95330869	3	0.301931
rs1050627	GEM	8	95330869	2	1.177244
rs1050627	GEM	8	95330869	7	0.850411
rs11985	GEM	8	95330892	1	3.97917
rs17036	GEM	8	95331131	1	4.908241
rs2170363	GEM	8	95341781	3	0.634423
rs2170363	GEM	8	95341781	7	0.585184
rs2250208	GEM	8	95343533	1	3.547743
rs2250208	GEM	8	95343533	6	0.622101
rs2250208	GEM	8	95343533	5	0.046177
rs2250208	GEM	8	95343533	3	0.676301
rs957448	KIAA1429	8	95610478	5	0.591526
rs957446	KIAA1429	8	95610547	8	0.121398
rs957446	KIAA1429	8	95610547	7	0.004634
rs957446	KIAA1429	8	95610547	1	0.141789
rs957446	KIAA1429	8	95610547	5	0.172496
rs957446	KIAA1429	8	95610547	6	0.899444
rs957446	KIAA1429	8	95610547	2	0.742026

rs880938	SDC2	8	97575549	8	5.323067
rs3816208	SDC2	8	97683801	3	3.076951
rs2635164	TSPYL5	8	98358890	2	0.54762
rs2635164	TSPYL5	8	98358890	6	0.247757
rs2635164	TSPYL5	8	98358890	1	0.594612
rs2635164	TSPYL5	8	98358890	7	0.039295
rs2290470	MATN2	8	99012622	8	1.571306
rs2290470	MATN2	8	99012622	6	1.520854
rs2290470	MATN2	8	99012622	4	0.901803
rs2290470	MATN2	8	99012622	7	0.067631
rs35407519	MATN2	8	99023284	4	0.522342
rs35407519	MATN2	8	99023284	5	0.737218
rs1869608	MATN2	8	99060399	4	0.844392
rs1869608	MATN2	8	99060399	5	0.24278
rs2255317	MATN2	8	99113704	2	4.015294
rs4735538	NPAL2	8	99273942	7	1.181313
rs3802197	KCNS2	8	99511080	1	3.745246
rs3802197	KCNS2	8	99511080	4	1.000695
SNP8-10051306	VPS13B	8	100513061	3	0.499371
rs6468694	VPS13B	8	100935012	1	5.746871
rs4518636	COX6C	8	100973453	1	3.602454
rs7841915	RGS22	8	101059353	7	1.407398
rs2446917	FBXO43	8	101214885	5	5.531954
rs1460934	FBXO43	8	101222200	6	1.559196
rs1460934	FBXO43	8	101222200	5	0.381846
rs17335870	SPAG1	8	101275567	6	0.852861
rs17335870	SPAG1	8	101275567	8	0.512125
rs2022923	MGC39715	8	101655309	4	0.507126
rs2022923	MGC39715	8	101655309	1	1.730487
rs2022923	MGC39715	8	101655309	3	2.165215
rs3824090	GRHL2	8	102748392	7	0.086825
rs3824090	GRHL2	8	102748392	4	5.874889
rs3735715	GRHL2	8	102749085	3	0.276649
rs3735715	GRHL2	8	102749085	4	2.819827
rs3735715	GRHL2	8	102749085	1	4.932859
rs3735715	GRHL2	8	102749085	7	1.633271
rs6468783	GRHL2	8	102750936	1	3.356691
rs16868201	NCALD	8	102769812	4	0.335487
rs16868201	NCALD	8	102769812	7	0.369122
rs1265116	RRM2B	8	103288049	6	0.697389
rs1265116	RRM2B	8	103288049	8	3.819268
rs3808357	ODF1	8	103633083	6	2.80973
rs3808357	ODF1	8	103633083	1	4.166613
rs3808357	ODF1	8	103633083	5	3.571724
rs2304347	AZIN1	8	103914548	7	0.427409
rs1802539	AZIN1	8	103945359	2	6.150192
rs2454042	LOC643972	8	104102681	2	1.667529

rs4734684	ATP6V1C1	8	104151587	1	4.480156
rs4734684	ATP6V1C1	8	104151587	3	0.314357
rs2453994	ATP6V1C1	8	104152827	1	6.571431
rs2453994	ATP6V1C1	8	104152827	4	0.172132
rs16870033	ATP6V1C1	8	104152962	5	0.078493
rs16870033	ATP6V1C1	8	104152962	4	1.345103
rs16870033	ATP6V1C1	8	104152962	6	0.857252
rs16870033	ATP6V1C1	8	104152962	1	6.055816
rs16870270	BAALC	8	104310942	1	4.017399
rs3098233	CTHRC1	8	104463920	2	2.717926
rs3098233	CTHRC1	8	104463920	3	0.560343
rs17803441	SLC25A32	8	104486221	3	0.381492
rs3134295	WDSOF1	8	104496535	7	0.150002
rs3134295	WDSOF1	8	104496535	2	1.751614
rs3134295	WDSOF1	8	104496535	3	0.09818
rs3134295	WDSOF1	8	104496535	5	0.168148
rs3134295	WDSOF1	8	104496535	6	0.390212
rs3134295	WDSOF1	8	104496535	1	0.363645
rs3134296	WDSOF1	8	104496717	2	2.148039
rs10461	RIMS2	8	105333155	7	0.054077
rs10461	RIMS2	8	105333155	8	3.220354
rs1060441	RIMS2	8	105333792	7	0.879753
rs1060441	RIMS2	8	105333792	5	0.490338
rs1060441	RIMS2	8	105333792	6	1.270938
rs1060441	RIMS2	8	105333792	4	1.064136
rs1060441	RIMS2	8	105333792	2	3.489857
rs13279448	LRP12	8	105570989	2	4.561985
rs3134127	LOC643319	8	107779021	3	3.698668
rs3134127	LOC643319	8	107779021	1	2.639265
rs3134127	LOC643319	8	107779021	2	4.593234
rs11996457	ABRA	8	107851511	3	0.728296
rs11996457	ABRA	8	107851511	6	0.999108
rs11996466	ABRA	8	107851571	3	0.194159
rs11996466	ABRA	8	107851571	6	1.11216
rs2507800	ANGPT1	8	108331845	1	2.973823
rs2507800	ANGPT1	8	108331845	5	0.15514
rs2507800	ANGPT1	8	108331845	3	0.147505
rs1954727	ANGPT1	8	108332310	1	3.670195
rs1954727	ANGPT1	8	108332310	3	0.228358
rs1954727	ANGPT1	8	108332310	8	3.375007
rs1548083	NUDCD1	8	110323055	6	0.117661
rs1548083	NUDCD1	8	110323055	8	5.023253
rs1548083	NUDCD1	8	110323055	3	0.029404
rs1548083	NUDCD1	8	110323055	7	4.617231
rs1548083	NUDCD1	8	110323055	1	5.277639
rs1548083	NUDCD1	8	110323055	5	0.064176
rs1379369	PKHD1L1	8	110481695	8	2.492998

rs1379369	PKHD1L1	8	110481695	3	2.75327
rs1379369	PKHD1L1	8	110481695	6	1.995403
rs1379369	PKHD1L1	8	110481695	1	3.046196
rs13252234	PKHD1L1	8	110489605	3	3.597381
rs10093885	PKHD1L1	8	110517811	6	3.061454
rs1673404	PKHD1L1	8	110534236	6	1.986614
rs1673404	PKHD1L1	8	110534236	3	0.162697
rs1673398	PKHD1L1	8	110546169	3	1.202326
rs1783174	PKHD1L1	8	110608362	5	0.70665
rs1895013	CSMD3	8	113304905	2	3.074133
rs1895013	CSMD3	8	113304905	4	1.243325
rs17629268	CSMD3	8	113304918	4	0.63453
rs4876458	CSMD3	8	113306221	7	3.034608
rs4876458	CSMD3	8	113306221	8	0.08897
rs11778209	CSMD3	8	113433872	1	3.322416
rs11778209	CSMD3	8	113433872	4	2.571334
rs11778209	CSMD3	8	113433872	3	0.330195
rs12155677	CSMD3	8	113554111	7	0.24206
rs17608189	CSMD3	8	114360001	1	5.973108
rs16884568	CSMD3	8	114458524	5	2.22532
rs13266634	SLC30A8	8	118253964	3	0.379887
rs3802177	SLC30A8	8	118254206	3	0.958204
rs3802177	SLC30A8	8	118254206	7	4.269674
rs3802177	SLC30A8	8	118254206	5	2.699185
rs3802177	SLC30A8	8	118254206	6	2.382108
rs2466294	SLC30A8	8	118254244	5	3.426318
rs2466294	SLC30A8	8	118254244	3	0.240762
rs2466294	SLC30A8	8	118254244	7	5.312917
rs2466294	SLC30A8	8	118254244	6	2.531344
rs11558471	SLC30A8	8	118254914	7	4.952926
rs11558471	SLC30A8	8	118254914	6	1.433247
rs11558471	SLC30A8	8	118254914	5	3.235866
rs11558471	SLC30A8	8	118254914	3	0.153211
rs2466293	SLC30A8	8	118255119	3	1.465134
rs2466293	SLC30A8	8	118255119	2	2.125235
rs2466293	SLC30A8	8	118255119	6	0.655417
rs2466293	SLC30A8	8	118255119	7	4.424007
rs10282940	SLC30A8	8	118257007	2	2.787155
rs17439693	EXT1	8	118901201	7	0.515728
rs17439693	EXT1	8	118901201	1	0.565298
rs17439693	EXT1	8	118901201	6	2.079833
rs17439875	EXT1	8	118916963	3	0.791811
rs17439875	EXT1	8	118916963	1	3.16683
rs11992702	SAMD12	8	119460458	7	0.129342
rs11992702	SAMD12	8	119460458	8	0.129779
rs11992702	SAMD12	8	119460458	3	0.747646
rs11992702	SAMD12	8	119460458	2	3.706365

rs11992702	SAMD12	8	119460458	5	0.008894
rs11992702	SAMD12	8	119460458	6	6.460093
rs5020517	SAMD12	8	119460972	5	0.124502
rs5020517	SAMD12	8	119460972	2	5.040233
rs5020517	SAMD12	8	119460972	3	0.638503
rs2073617	TNFRSF11B	8	120033464	4	0.427671
rs2073617	TNFRSF11B	8	120033464	1	4.381981
rs2073617	TNFRSF11B	8	120033464	3	0.380834
rs2073617	TNFRSF11B	8	120033464	8	0.795883
rs17787689	MAL2	8	120326285	4	7.388267
rs2279112	NOV	8	120498205	8	2.722102
rs2279112	NOV	8	120498205	2	0.805024
rs14324	NOV	8	120505667	2	6.107021
rs14324	NOV	8	120505667	3	0.934936
rs14324	NOV	8	120505667	8	0.772007
rs11526	NOV	8	120505722	8	0.86985
rs11526	NOV	8	120505722	2	2.873238
rs2305129	ENPP2	8	120639058	3	1.040653
rs4871364	ENPP2	8	120663983	2	3.789802
rs4871364	ENPP2	8	120663983	5	0.83626
rs10283100	ENPP2	8	120665204	8	1.1642
rs10283100	ENPP2	8	120665204	7	2.562642
rs956749	TAF2	8	120813580	5	6.572453
rs1055133	DCC1	8	120916259	5	0.048113
rs1055133	DCC1	8	120916259	4	0.245564
rs1055133	DCC1	8	120916259	7	1.1528
rs1055133	DCC1	8	120916259	1	6.340328
SNP8-12113106(	DEPDC6	8	121131060	5	0.52643
SNP8-12113106(	DEPDC6	8	121131060	3	0.140294
SNP8-12113106(	DEPDC6	8	121131060	7	0.319144
rs17833992	COL14A1	8	121367337	4	0.316347
rs17833992	COL14A1	8	121367337	2	3.600509
rs17833992	COL14A1	8	121367337	1	0.218857
rs12548759	ZHX2	8	124035295	3	0.33528
rs3802265	ZHX2	8	124035340	3	0.206902
rs3802265	ZHX2	8	124035340	6	0.23319
rs3802265	ZHX2	8	124035340	8	1.24866
rs2272722	DERL1	8	124100722	4	0.005853
rs2272722	DERL1	8	124100722	5	0.259463
rs16898013	WDR67	8	124208072	3	0.053698
rs16898013	WDR67	8	124208072	8	5.558989
rs3739291	FAM83A	8	124264149	2	2.388606
rs3739291	FAM83A	8	124264149	4	0.191561
rs3739291	FAM83A	8	124264149	7	0.829419
SNP8-12429090	FAM83A	8	124290904	8	5.839843
rs6470147	C8orf32	8	124517985	3	0.060933
rs6470147	C8orf32	8	124517985	7	1.239761

rs6470147	C8orf32	8	124517985	1	6.115551
rs6470147	C8orf32	8	124517985	4	0.215512
rs4871385	FBXO32	8	124622403	4	6.121065
rs4871385	FBXO32	8	124622403	2	2.80106
rs16898671	C8ORFK36	8	124727391	1	5.503013
rs16898671	C8ORFK36	8	124727391	7	0.611503
rs16898671	C8ORFK36	8	124727391	4	0.012985
rs16898671	C8ORFK36	8	124727391	6	1.034648
rs11779866	C8ORFK36	8	124727395	3	1.069699
rs16898689	C8ORFK36	8	124733076	8	3.059157
rs16898691	C8ORFK36	8	124733168	8	1.024484
rs4471017	ANXA13	8	124762591	1	3.946914
rs4471017	ANXA13	8	124762591	3	3.952818
rs2294013	ANXA13	8	124779910	5	1.385881
rs2294013	ANXA13	8	124779910	1	0.074803
rs2294013	ANXA13	8	124779910	4	0.426532
rs2294013	ANXA13	8	124779910	2	0.040595
rs6470179	ANXA13	8	124818791	5	0.351786
rs2272684	FAM91A1	8	124887628	3	0.104352
rs2272684	FAM91A1	8	124887628	6	1.048842
rs1897318	FER1L6	8	124933585	3	0.391239
SNP8-125037424	FER1L6	8	125037424	3	1.084456
rs7465584	FER1L6	8	125056659	8	1.690644
rs7465584	FER1L6	8	125056659	1	0.906416
rs7465584	FER1L6	8	125056659	5	1.074476
rs4358790	FER1L6	8	125104969	7	4.002996
rs4358790	FER1L6	8	125104969	5	0.897297
rs4358790	FER1L6	8	125104969	1	3.229558
rs4358790	FER1L6	8	125104969	6	0.5813
rs4870887	FER1L6	8	125131076	8	2.076994
rs4870887	FER1L6	8	125131076	7	1.447384
rs4483140	FER1L6	8	125141607	6	0.825717
rs6470219	FER1L6	8	125176382	6	0.074587
rs3812473	TRMT12	8	125532264	2	3.690074
rs3812473	TRMT12	8	125532264	7	0.96052
rs3829033	MTSS1	8	125633823	2	5.461551
rs3829033	MTSS1	8	125633823	7	6.657586
rs10104558	ZNF572	8	126058641	5	4.116953
rs10105106	ZNF572	8	126058830	5	0.459048
rs16900167	ZNF572	8	126058879	1	5.204756
rs16900167	ZNF572	8	126058879	4	0.607858
rs10107774	ZNF572	8	126059033	5	0.413859
rs7829785	ZNF572	8	126060039	5	0.105349
SNP8-128820383	MYC	8	128820383	4	1.444901
SNP8-128820383	MYC	8	128820383	1	3.533303
rs12677518	PVT1	8	129021490	2	4.404695
rs4736529	EFR3A	8	133092345	2	4.040414

rs4736529	EFR3A	8	133092345	3	0.486669
rs4736529	EFR3A	8	133092345	4	6.667648
rs4736529	EFR3A	8	133092345	5	0.184841
rs8859	EFR3A	8	133094853	2	4.608044
rs11779306	OC90	8	133116253	4	0.188164
rs11779306	OC90	8	133116253	1	0.70086
rs11779306	OC90	8	133116253	7	0.138709
rs1048490	TMEM71	8	133791463	5	0.783767
rs1048490	TMEM71	8	133791463	4	0.200166
rs1048490	TMEM71	8	133791463	7	0.744975
rs180222	TG	8	133968342	4	0.649381
rs180223	TG	8	133969434	3	0.408705
rs2069550	TG	8	133969568	3	0.293917
rs2069550	TG	8	133969568	8	0.639549
rs2069550	TG	8	133969568	7	0.24101
rs2069550	TG	8	133969568	6	0.997455
rs853326	TG	8	133979156	8	0.185757
rs853326	TG	8	133979156	7	1.005206
rs853326	TG	8	133979156	3	0.103954
rs853304	TG	8	134000930	3	0.211071
rs2069561	TG	8	134044465	4	0.55032
rs2069561	TG	8	134044465	7	0.018445
SNP8-13417772	TG	8	134177728	3	0.010846
SNP8-13417772	TG	8	134177728	1	0.591923
SNP8-13417772	TG	8	134177728	2	1.087234
SNP8-13417772	TG	8	134177728	4	0.441799
SNP8-13417772	TG	8	134177728	6	0.200079
rs1133076	TG	8	134194864	3	2.62071
rs1133076	TG	8	134194864	1	2.038875
rs1133076	TG	8	134194864	8	4.958293
rs1133076	TG	8	134194864	4	0.616228
rs1133076	TG	8	134194864	7	2.26588
rs1133076	TG	8	134194864	6	2.239331
rs2294024	TG	8	134213295	2	3.428352
rs2294024	TG	8	134213295	7	0.554961
rs2294024	TG	8	134213295	8	5.051645
rs2294024	TG	8	134213295	3	2.036323
rs2294024	TG	8	134213295	4	1.522494
rs3739261	WISP1	8	134308952	8	2.669684
rs3739261	WISP1	8	134308952	2	1.972635
rs2929969	WISP1	8	134309879	3	0.968139
rs12164193	WISP1	8	134310105	4	0.650882
rs12164193	WISP1	8	134310105	2	1.683659
rs2929970	WISP1	8	134310319	2	1.983832
rs2929970	WISP1	8	134310319	8	1.182759
rs2929970	WISP1	8	134310319	3	0.152498
rs3739429	ZFAT1	8	135593979	5	8.370706

rs16905194	ZFAT1	8	135679389	5	0.055611
rs16905194	ZFAT1	8	135679389	8	0.15266
rs6982146	ZFAT1	8	135680299	5	5.471214
rs6982146	ZFAT1	8	135680299	8	2.670643
rs12549256	ZFAT1	8	135680336	1	0.511572
rs12549256	ZFAT1	8	135680336	2	0.289239
rs894343	ZFAT1	8	135681777	5	4.577228
rs894343	ZFAT1	8	135681777	4	1.299756
rs894343	ZFAT1	8	135681777	6	2.194351
rs4131277	COL22A1	8	139712156	7	0.699473
rs10091563	COL22A1	8	139716444	7	0.78074
rs4909444	COL22A1	8	139770391	3	0.189272
rs4909444	COL22A1	8	139770391	5	1.746814
rs199226	NIBP	8	140812382	6	0.507037
rs199226	NIBP	8	140812382	1	0.273449
rs199226	NIBP	8	140812382	2	0.307905
rs2233230	C8orf17	8	141012685	6	0.557618
rs2233230	C8orf17	8	141012685	2	4.053087
rs11166965	NIBP	8	141332197	4	7.220378
rs11166965	NIBP	8	141332197	7	7.519614
rs3735801	NIBP	8	141530448	5	0.323973
rs2231516	LOC729563	8	141590333	7	0.146845
rs2231516	LOC729563	8	141590333	5	0.146428
rs2231516	LOC729563	8	141590333	3	0.614763
rs10216653	CHRAC1	8	141596167	7	7.287837
rs10216653	CHRAC1	8	141596167	2	5.308562
rs10216653	CHRAC1	8	141596167	5	4.40542
rs10216653	CHRAC1	8	141596167	6	5.62647
rs10216653	CHRAC1	8	141596167	4	2.891079
rs10216653	CHRAC1	8	141596167	3	0.31441
rs2977461	EIF2C2	8	141610470	7	0.274745
rs2977461	EIF2C2	8	141610470	4	5.018185
rs2977461	EIF2C2	8	141610470	5	0.479276
rs7460	PTK2	8	141738042	5	0.155563
rs7460	PTK2	8	141738042	4	0.539871
rs7460	PTK2	8	141738042	3	0.134999
rs7460	PTK2	8	141738042	6	0.616784
rs2304284	DENND3	8	142231055	2	4.706683
rs2304283	DENND3	8	142231070	2	4.984022
rs1045248	DENND3	8	142273508	5	0.103846
rs1045248	DENND3	8	142273508	4	1.846571
rs1045248	DENND3	8	142273508	6	3.588362
rs3739242	SLC45A4	8	142288992	8	0.36287
rs3739242	SLC45A4	8	142288992	4	2.63278
rs3739242	SLC45A4	8	142288992	3	0.266158
rs3739238	SLC45A4	8	142291627	3	1.064383
rs3739238	SLC45A4	8	142291627	4	1.948492

rs3739238	SLC45A4	8	142291627	5	0.844344
rs2748421	FLJ43860	8	142586404	7	2.844028
rs10100935	TSNARE1	8	143423627	8	2.870647
rs10100935	TSNARE1	8	143423627	5	0.737349
rs10100935	TSNARE1	8	143423627	1	0.653286
rs10100935	TSNARE1	8	143423627	7	1.322473
rs10100935	TSNARE1	8	143423627	2	1.32801
rs7814359	TSNARE1	8	143433941	6	1.103199
rs7814359	TSNARE1	8	143433941	3	1.476342
rs10097505	ARC	8	143691186	3	0.24145
rs10097505	ARC	8	143691186	7	0.012627
rs3735995	JRK	8	143741708	3	0.00413
rs750530	JRK	8	143741933	5	0.061568
rs750530	JRK	8	143741933	8	3.616951
rs750530	JRK	8	143741933	4	6.189205
rs2976399	JRK	8	143743052	3	0.0175
rs2976399	JRK	8	143743052	4	1.942222
rs2976399	JRK	8	143743052	5	2.700096
rs3802234	JRK	8	143743367	7	1.341885
rs754957	JRK	8	143743418	7	0.411689
rs754957	JRK	8	143743418	8	0.107895
rs754957	JRK	8	143743418	5	0.957991
rs3802232	JRK	8	143743703	5	0.208328
rs3802232	JRK	8	143743703	7	0.99367
rs3802232	JRK	8	143743703	8	0.392074
rs2978973	JRK	8	143744273	5	4.469451
rs2978973	JRK	8	143744273	3	0.11924
rs2978973	JRK	8	143744273	8	5.636205
rs2978973	JRK	8	143744273	4	2.192468
rs3736001	PSCA	8	143759809	7	1.091651
rs2585138	C8orf55	8	143805953	4	0.376077
rs2585138	C8orf55	8	143805953	6	1.168689
rs2585138	C8orf55	8	143805953	1	0.410598
rs2585138	C8orf55	8	143805953	2	0.955389
rs2585138	C8orf55	8	143805953	8	0.492725
rs2585138	C8orf55	8	143805953	5	0.959341
rs13362	LYNX1	8	143849966	1	5.234575
rs13362	LYNX1	8	143849966	6	2.21627
rs2572925	LY6D	8	143864907	7	3.755792
rs2572925	LY6D	8	143864907	5	2.18801
rs2572925	LY6D	8	143864907	8	2.191851
rs2572925	LY6D	8	143864907	6	1.441361
rs5303	CYP11B1	8	143952097	5	1.190545
rs5303	CYP11B1	8	143952097	6	0.710306
rs5303	CYP11B1	8	143952097	1	2.304521
rs5283	CYP11B1	8	143957599	5	0.136294
rs5283	CYP11B1	8	143957599	1	0.453617

rs5283	CYP11B1	8	143957599	7	0.169603
rs5283	CYP11B1	8	143957599	3	0.220658
rs12543416	C8orf31	8	144192163	1	3.432395
SNP8-144195984	C8orf31	8	144195984	7	1.108187
SNP8-144195984	C8orf31	8	144195984	1	3.941125
SNP8-144195984	C8orf31	8	144195984	6	0.451996
SNP8-144195984	C8orf31	8	144195984	4	0.398557
SNP8-144195984	C8orf31	8	144195984	8	0.575504
rs13248517	C8orf31	8	144206336	4	3.62432
rs13248517	C8orf31	8	144206336	8	5.325205
rs13248517	C8orf31	8	144206336	1	5.300499
rs13248517	C8orf31	8	144206336	5	2.336971
rs13248517	C8orf31	8	144206336	6	0.590351
rs7823214	C8orf31	8	144206344	8	0.74254
rs7823214	C8orf31	8	144206344	1	6.103526
rs7823214	C8orf31	8	144206344	4	6.576227
rs7823214	C8orf31	8	144206344	5	0.920645
rs7823214	C8orf31	8	144206344	6	1.498278
rs4977119	C8orf31	8	144206762	1	6.084457
rs4977119	C8orf31	8	144206762	4	3.110379
rs4977119	C8orf31	8	144206762	6	4.331455
rs4977119	C8orf31	8	144206762	5	2.52834
rs6988480	HHCM	8	144242199	3	0.120932
rs7007949	LY6H	8	144311035	5	1.202825
rs7007949	LY6H	8	144311035	3	0.804801
GA007224	GPIHBP1	8	144367157	1	0.783868
GA007224	GPIHBP1	8	144367157	2	3.3603
GA007224	GPIHBP1	8	144367157	8	0.830999
GA007224	GPIHBP1	8	144367157	5	0.972454
rs12545739	LOC338328	8	144369042	5	3.277635
rs12545739	LOC338328	8	144369042	3	1.735142
rs12545739	LOC338328	8	144369042	2	1.086888
rs12545739	LOC338328	8	144369042	1	3.046652
rs7387879	ZFP41	8	144400672	3	1.61918
rs7387879	ZFP41	8	144400672	7	1.232165
rs7387879	ZFP41	8	144400672	1	1.349842
rs7387879	ZFP41	8	144400672	4	0.0747
rs7387879	ZFP41	8	144400672	2	0.014099
rs7387879	ZFP41	8	144400672	6	0.187924
rs3750213	ZFP41	8	144415762	2	0.780744
rs3750213	ZFP41	8	144415762	5	2.886854
rs3750213	ZFP41	8	144415762	7	0.115327
rs3750217	ZNF696	8	144450627	8	1.038071
rs3750217	ZNF696	8	144450627	5	0.407227
rs3750217	ZNF696	8	144450627	6	2.665295
rs3750217	ZNF696	8	144450627	4	1.399935
rs3750217	ZNF696	8	144450627	7	0.212022

SNP8-144478080	TOP1MT	8	144478080	8	7.914945
SNP8-144478080	TOP1MT	8	144478080	4	0.144226
SNP8-144478080	TOP1MT	8	144478080	3	0.581757
SNP8-144478080	TOP1MT	8	144478080	6	9.531603
rs2450772	TOP1MT	8	144484791	3	0.189014
rs2450772	TOP1MT	8	144484791	1	1.472078
rs3812440	RHPN1	8	144533312	7	1.462064
rs4874147	ZC3H3	8	144691326	8	0.937666
rs3750206	ZC3H3	8	144692234	2	0.534101
rs3750206	ZC3H3	8	144692234	1	0.039768
rs3750206	ZC3H3	8	144692234	5	0.23213
SNP8-144725392	C8orf73	8	144725392	4	0.332335
SNP8-144725392	C8orf73	8	144725392	6	0.301414
SNP8-144725392	C8orf73	8	144725392	5	0.542725
SNP8-144725392	C8orf73	8	144725392	1	1.56688
SNP8-144725392	C8orf73	8	144725392	3	0.576476
SNP8-144725392	C8orf73	8	144725392	8	0.301167
rs4873803	C8orf73	8	144725737	5	0.343017
rs4873803	C8orf73	8	144725737	3	0.366859
rs2290416	NAPRT1	8	144728743	1	2.370367
rs1062391	EEF1D	8	144733496	7	1.425437
rs1062391	EEF1D	8	144733496	1	1.42895
rs1062391	EEF1D	8	144733496	6	1.598433
rs1062391	EEF1D	8	144733496	5	0.436116
rs1062391	EEF1D	8	144733496	3	0.131498
rs3812448	EEF1D	8	144743065	5	0.952952
rs3812448	EEF1D	8	144743065	7	0.599707
rs3812448	EEF1D	8	144743065	1	3.465515
rs3812448	EEF1D	8	144743065	6	7.062067
rs3210186	TIGD5	8	144753454	3	0.27553
rs3210186	TIGD5	8	144753454	6	1.125301
rs3210186	TIGD5	8	144753454	5	0.659459
rs3210186	TIGD5	8	144753454	1	4.829423
rs3210186	TIGD5	8	144753454	7	0.424741
rs2242089	PYCRL	8	144760289	5	0.399953
rs2242089	PYCRL	8	144760289	8	2.513625
rs2242089	PYCRL	8	144760289	4	1.247428
rs2242089	PYCRL	8	144760289	3	0.626504
rs2978	TSTA3	8	144766033	6	0.095481
rs2978	TSTA3	8	144766033	4	0.448367
rs2978	TSTA3	8	144766033	1	7.475385
rs2978	TSTA3	8	144766033	3	0.832311
rs2978	TSTA3	8	144766033	5	0.990309
rs2978	TSTA3	8	144766033	8	7.123637
rs3750204	ZNF623	8	144803593	5	0.358633
rs3750204	ZNF623	8	144803593	1	6.870106
rs3204171	ZNF707	8	144849354	5	0.129242

rs3204171	ZNF707	8	144849354	7	0.867357
rs11136316	ZNF707	8	144849509	7	1.938932
rs11136316	ZNF707	8	144849509	5	1.264456
SNP8-144873581	MAPK15	8	144873581	3	0.878235
rs6558394	SCRIB	8	144965072	3	3.101487
rs7839934	EPPK1	8	145013169	6	5.506662
rs7839934	EPPK1	8	145013169	8	0.166489
rs12542653	EPPK1	8	145016164	4	2.177909
rs12542653	EPPK1	8	145016164	8	0.606476
SNP8-145073019	PLEC1	8	145073019	1	4.236478
SNP8-145073019	PLEC1	8	145073019	3	0.386718
rs11544989	PARP10	8	145129856	1	1.515036
rs11544989	PARP10	8	145129856	4	0.800077
rs11544989	PARP10	8	145129856	5	1.476257
rs11136344	PARP10	8	145131413	5	1.261183
rs11136344	PARP10	8	145131413	7	0.316276
rs11136344	PARP10	8	145131413	1	3.360886
rs11136345	PARP10	8	145132100	8	0.190737
rs11136345	PARP10	8	145132100	7	0.522953
rs11136345	PARP10	8	145132100	5	0.360342
rs11136345	PARP10	8	145132100	4	0.913855
rs11136345	PARP10	8	145132100	1	3.942909
rs11993554	OPLAH	8	145183517	7	0.703527
rs6558292	OPLAH	8	145186631	7	1.072882
rs4977196	KIAA1875	8	145235402	7	0.516827
SNP8-145295276	KIAA1833	8	145295276	3	0.246475
rs2272663	C8ORFK29	8	145549104	7	1.751691
rs2272663	C8ORFK29	8	145549104	1	1.705286
rs2272663	C8ORFK29	8	145549104	6	0.141627
rs6599528	ADCK5	8	145573922	6	1.177602
rs6599528	ADCK5	8	145573922	2	3.162037
rs6599528	ADCK5	8	145573922	7	7.577289
rs6599528	ADCK5	8	145573922	5	0.685505
SNP8-145610489	SLC39A4	8	145610489	3	0.510052
rs2272662	SLC39A4	8	145610534	5	2.68014
rs2272662	SLC39A4	8	145610534	6	3.236341
rs2280839	SLC39A4	8	145612372	5	0.545025
rs2280839	SLC39A4	8	145612372	7	0.447868
rs1046830	CYHR1	8	145660058	4	5.684203
rs750472	FOXH1	8	145672261	4	1.57782
rs750472	FOXH1	8	145672261	5	0.322261
rs750472	FOXH1	8	145672261	3	1.092342
rs750472	FOXH1	8	145672261	2	3.591821
rs1063739	GPT	8	145700535	2	4.77532
rs1063739	GPT	8	145700535	5	0.603499
rs1063739	GPT	8	145700535	3	1.851469
rs1063739	GPT	8	145700535	4	0.906377

SNP8-145708624	RECQL4	8	145708624	3	0.773676
SNP8-145708624	RECQL4	8	145708624	4	0.629121
SNP8-145708624	RECQL4	8	145708624	5	5.925172
SNP8-145712510	RECQL4	8	145712510	4	0.247934
SNP8-145712510	RECQL4	8	145712510	2	5.113111
SNP8-145712510	RECQL4	8	145712510	5	4.897736
rs2306386	RECQL4	8	145713687	4	2.221615
rs2306386	RECQL4	8	145713687	2	2.186096
rs2306386	RECQL4	8	145713687	5	3.949452
rs2306386	RECQL4	8	145713687	1	0.228392
rs2620651	LRRC24	8	145719561	3	0.295437
rs2730059	ZNF251	8	145952565	3	1.054326
rs1060859	RPL8	8	145986553	3	0.631049
rs2954658	RPL8	8	145988586	4	4.114035
rs2954658	RPL8	8	145988586	7	0.521091
rs2954658	RPL8	8	145988586	1	4.968027
rs2954658	RPL8	8	145988586	5	0.999987
rs2242650	COMMD5	8	146037222	1	2.000726
rs2242650	COMMD5	8	146037222	7	1.150324
rs2242650	COMMD5	8	146037222	5	3.428479
rs2242650	COMMD5	8	146037222	4	1.272793
rs2242650	COMMD5	8	146037222	2	0.927677
rs2236547	C9orf66	9	204864	7	0.910556
rs2236547	C9orf66	9	204864	3	1.288805
rs2236547	C9orf66	9	204864	5	0.154355
rs11789099	DOCK8	9	302134	8	0.166338
rs12348944	DOCK8	9	367066	4	3.363222
rs2297079	DOCK8	9	411032	2	3.885871
rs2297079	DOCK8	9	411032	5	8.197984
rs2297079	DOCK8	9	411032	6	2.904754
rs2297079	DOCK8	9	411032	3	0.440708
rs10491684	DOCK8	9	439798	3	0.639689
rs10491684	DOCK8	9	439798	5	0.135823
rs4465020	ANKRD15	9	702060	2	8.056294
rs912175	ANKRD15	9	702137	6	1.165621
SNP9-1045870	DMRT2	9	1045870	3	0.870541
rs17641078	DMRT2	9	1046959	5	0.050353
rs4741030	DMRT2	9	1047351	3	0.880642
rs34881325	VLDLR	9	2612134	2	4.920065
rs34881325	VLDLR	9	2612134	5	5.314839
rs34881325	VLDLR	9	2612134	8	7.391539
rs34881325	VLDLR	9	2612134	4	1.75795
rs34881325	VLDLR	9	2612134	3	0.470912
rs10967705	KCNV2	9	2707922	1	4.463718
rs10967705	KCNV2	9	2707922	7	5.619167
rs17656693	KCNV2	9	2708588	3	2.388311
rs12352254	KCNV2	9	2719686	1	3.457624

rs41306094	KCNV2	9	2719733	1	1.964491
SNP9-2818742	KIAA0020	9	2818742	4	0.415716
SNP9-2818742	KIAA0020	9	2818742	8	0.110478
SNP9-2818742	KIAA0020	9	2818742	3	0.597679
rs17715051	RFX3	9	3237107	4	5.892805
rs17715051	RFX3	9	3237107	1	5.038545
rs2228622	SLC1A1	9	4554432	3	0.90185
rs2228622	SLC1A1	9	4554432	6	1.616648
rs2228622	SLC1A1	9	4554432	5	0.321238
SNP9-4607999	C9orf68	9	4607999	5	1.697516
rs301487	PPAPDC2	9	4652369	2	1.501342
rs301487	PPAPDC2	9	4652369	5	0.934605
rs301487	PPAPDC2	9	4652369	6	1.233376
rs301487	PPAPDC2	9	4652369	1	1.398388
rs1485202	PPAPDC2	9	4654749	6	6.511515
rs1485202	PPAPDC2	9	4654749	1	3.483414
rs1485202	PPAPDC2	9	4654749	3	0.353596
rs1485202	PPAPDC2	9	4654749	5	8.978486
rs1053872	RCL1	9	4850643	8	6.527235
rs1053872	RCL1	9	4850643	5	0.92762
rs1053872	RCL1	9	4850643	1	0.117181
rs1053872	RCL1	9	4850643	3	0.267565
rs1053872	RCL1	9	4850643	2	1.295964
rs2273782	RLN1	9	5325470	4	0.561438
rs2273782	RLN1	9	5325470	3	0.070263
rs4143815	CD274	9	5458257	3	0.274481
rs16923189	PDCD1LG2	9	5500644	8	1.393749
rs3739646	KIAA1432	9	5752600	3	0.019027
rs1056798	MLANA	9	5899259	5	0.695964
rs1056798	MLANA	9	5899259	3	0.519738
rs10975519	IL33	9	6243571	7	1.251795
rs10975519	IL33	9	6243571	4	0.527528
rs10975519	IL33	9	6243571	5	1.834445
rs10975519	IL33	9	6243571	3	1.972909
rs10975519	IL33	9	6243571	1	4.793776
rs1048274	IL33	9	6246292	4	0.965268
rs1048274	IL33	9	6246292	6	6.449054
rs1048274	IL33	9	6246292	5	0.346801
rs1048274	IL33	9	6246292	3	1.5369
rs1048274	IL33	9	6246292	1	3.270742
rs1048274	IL33	9	6246292	7	0.38871
rs8172	IL33	9	6247898	1	5.79807
rs33962342	UHRF2	9	6496038	5	0.747163
rs33962342	UHRF2	9	6496038	7	0.336272
rs7848919	GLDC	9	6522477	3	0.063234
rs7848919	GLDC	9	6522477	4	1.193387
rs7848919	GLDC	9	6522477	5	0.706998

rs1061407	GLDC	9	6522544	1	2.412076
rs1061407	GLDC	9	6522544	5	2.002972
rs1061407	GLDC	9	6522544	3	1.541286
rs1061407	GLDC	9	6522544	7	1.356962
rs1061407	GLDC	9	6522544	8	1.799068
rs1061407	GLDC	9	6522544	4	1.864956
rs1061407	GLDC	9	6522544	6	1.550575
rs35374927	GLDC	9	6600326	5	1.334928
rs10976088	JMJD2C	9	7164813	5	6.280111
rs10976088	JMJD2C	9	7164813	4	0.154697
rs2279776	PTPRD	9	8379364	5	0.703316
rs2279776	PTPRD	9	8379364	4	0.592518
rs2279776	PTPRD	9	8379364	1	0.219028
rs2279776	PTPRD	9	8379364	7	1.758702
rs2279776	PTPRD	9	8379364	2	3.377404
rs3763653	PTPRD	9	8508395	3	0.675267
rs683	TYRP1	9	12699305	3	0.583344
rs683	TYRP1	9	12699305	1	2.493045
rs683	TYRP1	9	12699305	4	0.545163
SNP9-12811695	C9orf150	9	12811695	1	2.86357
rs4741289	MPDZ	9	13180163	4	3.827814
rs7858	NFIB	9	14077769	4	0.30117
rs3209160	ZDHHC21	9	14608316	4	0.874375
rs10961689	FREM1	9	14727506	3	2.239466
rs4741426	FREM1	9	14737412	7	1.726999
rs10738380	FREM1	9	14791687	3	0.768429
SNP9-14791710	FREM1	9	14791710	1	0.415637
SNP9-14791710	FREM1	9	14791710	3	1.278057
rs7023244	FREM1	9	14809370	3	1.780785
rs7023244	FREM1	9	14809370	1	0.456166
rs2665515	PSIP1	9	15454743	3	0.155592
rs4741510	C9orf93	9	15581372	5	1.025329
rs2296863	BNC2	9	16405411	5	2.214863
rs2296863	BNC2	9	16405411	1	2.626356
rs2296863	BNC2	9	16405411	8	0.579954
rs2296864	BNC2	9	16405459	8	0.197613
rs2296864	BNC2	9	16405459	5	1.310452
rs3739715	BNC2	9	16425714	5	0.968266
rs3739715	BNC2	9	16425714	8	0.365124
rs3739715	BNC2	9	16425714	1	1.15328
rs7860932	LOC648570	9	16776898	3	0.686665
rs3739489	C9orf39	9	17384996	4	0.209784
rs3739489	C9orf39	9	17384996	3	0.776922
rs41304755	ADAMTSL1	9	18819952	3	3.786835
rs10117672	NULL	9	18899703	8	0.112839
rs10117672	NULL	9	18899703	3	0.323422
rs10117672	NULL	9	18899703	4	0.858297

rs10117672	NULL	9	18899703	2	4.473411
rs11794832	NULL	9	18900581	7	0.209501
rs7021572	C9orf138	9	18940895	3	0.45833
rs17810415	C9orf138	9	19022907	1	0.040209
rs10963922	C9orf138	9	19023058	3	0.442491
rs10511670	FAM29A	9	19048743	7	0.889678
rs10511670	FAM29A	9	19048743	1	5.38826
rs13286328	FAM29A	9	19066673	3	0.01001
rs13286328	FAM29A	9	19066673	7	0.956218
rs13286328	FAM29A	9	19066673	5	0.501631
rs13286328	FAM29A	9	19066673	8	3.936988
rs2228416	ADFP	9	19116281	7	6.36671
rs3808657	LOC729925	9	19117877	7	0.141542
rs3808657	LOC729925	9	19117877	3	0.166911
rs10964136	ASAH3L	9	19424980	5	6.515391
rs12343276	ASAH3L	9	19441807	7	0.748414
rs12349140	LOC392288	9	19451193	6	0.653789
rs12349140	LOC392288	9	19451193	5	2.411853
rs4977308	SLC24A2	9	19612268	7	0.370969
rs4977881	KIAA1797	9	20978426	5	1.005889
rs4977881	KIAA1797	9	20978426	1	4.141045
rs4977881	KIAA1797	9	20978426	3	1.141172
rs4977881	KIAA1797	9	20978426	4	0.911412
rs2298260	PTPLAD2	9	21019330	3	1.476944
rs2298260	PTPLAD2	9	21019330	4	1.71126
rs2298260	PTPLAD2	9	21019330	1	1.442365
rs10964982	IFNA8	9	21399901	5	0.664423
rs10964982	IFNA8	9	21399901	1	1.734363
rs12380505	LOC402359	9	21685893	2	1.983698
rs12380505	LOC402359	9	21685893	5	2.404684
rs12380505	LOC402359	9	21685893	7	1.826265
rs12380505	LOC402359	9	21685893	1	3.297236
rs12380505	LOC402359	9	21685893	4	1.839579
SNP9-21854378	MTAP	9	21854378	8	8.064959
SNP9-21854378	MTAP	9	21854378	5	5.430204
SNP9-21854378	MTAP	9	21854378	3	0.000117
SNP9-21854378	MTAP	9	21854378	1	0.014395
SNP9-21854378	MTAP	9	21854378	2	5.921109
SNP9-22442002	DMRTA1	9	22442002	1	1.120759
SNP9-22442002	DMRTA1	9	22442002	2	2.48236
rs1128957	TUSC1	9	25667257	3	0.008512
rs1128957	TUSC1	9	25667257	5	0.006324
rs1128957	TUSC1	9	25667257	1	5.967599
rs1128957	TUSC1	9	25667257	6	0.57083
rs10967649	LRRC19	9	26983755	1	3.325426
rs10812509	LRRC19	9	26983889	1	3.567941
rs1334811	TEK	9	27180655	1	1.611038

rs1334811	TEK	9	27180655	7	0.855712
rs639225	TEK	9	27192870	5	0.812554
rs639225	TEK	9	27192870	4	1.410907
rs639225	TEK	9	27192870	1	0.527932
rs639225	TEK	9	27192870	2	1.172495
rs639225	TEK	9	27192870	3	1.588765
rs542913	TEK	9	27195021	1	5.133862
rs542913	TEK	9	27195021	4	0.133703
rs542913	TEK	9	27195021	5	1.420514
rs542913	TEK	9	27195021	3	0.248142
rs3737188	TEK	9	27203580	4	1.477346
rs3737188	TEK	9	27203580	2	4.470013
rs7037246	TEK	9	27219544	8	1.188819
rs7037246	TEK	9	27219544	6	0.070981
rs857	TEK	9	27219786	2	2.732777
rs857	TEK	9	27219786	7	0.760954
rs9987817	TEK	9	27220152	1	4.615392
rs1057524	C9orf11	9	27274708	4	1.270077
rs1057524	C9orf11	9	27274708	2	4.84743
rs1057524	C9orf11	9	27274708	5	0.619635
rs3739530	MOBKL2B	9	27320514	5	0.0912
rs700782	IFNK	9	27516047	6	1.036134
rs700782	IFNK	9	27516047	5	0.417882
rs700782	IFNK	9	27516047	1	6.071346
rs3739527	IFNK	9	27516480	6	0.797757
rs3849944	C9orf72	9	27550594	6	1.251186
rs3849944	C9orf72	9	27550594	3	1.004039
rs3849944	C9orf72	9	27550594	1	0.87097
rs12347222	C9orf72	9	27550965	6	1.478846
rs813504	LOC392298	9	27599207	3	3.300534
rs813504	LOC392298	9	27599207	7	2.151401
rs813504	LOC392298	9	27599207	4	1.968848
rs2889829	LOC392298	9	27600657	4	2.433335
rs16918170	LOC392301	9	32323630	8	2.283289
rs16918170	LOC392301	9	32323630	4	1.756137
rs12555727	DDX58	9	32446656	4	3.167038
rs12555727	DDX58	9	32446656	1	4.064765
rs12555727	DDX58	9	32446656	5	5.108715
rs17217280	DDX58	9	32470251	4	0.218942
rs10813831	DDX58	9	32516146	2	2.444882
rs3739674	DDX58	9	32516233	8	0.986754
rs3739674	DDX58	9	32516233	1	3.748376
rs3739674	DDX58	9	32516233	5	0.681771
rs10971128	LOC401498	9	32775076	7	0.462975
rs10971128	LOC401498	9	32775076	6	1.60564
rs2297218	DNAJA1	9	33015251	3	0.483893
rs2297218	DNAJA1	9	33015251	1	5.96331

rs2228332	AQP3	9	33432952	4	3.827877
rs2228332	AQP3	9	33432952	2	1.24586
rs2228332	AQP3	9	33432952	1	0.077891
rs2228332	AQP3	9	33432952	5	1.245843
rs2228332	AQP3	9	33432952	3	3.134581
rs12555686	AQP3	9	33437547	7	0.688107
rs2777744	NOL6	9	33463893	5	0.09898
rs2777744	NOL6	9	33463893	2	0.284969
rs2777744	NOL6	9	33463893	1	1.176191
rs1378	WDR40A	9	34097504	2	2.152793
SNP9-34247095	KIF24	9	34247095	6	0.635762
SNP9-34247095	KIF24	9	34247095	4	0.234589
SNP9-34247095	KIF24	9	34247095	1	5.966204
SNP9-34612389	ARID3C	9	34612389	8	0.316065
SNP9-34612389	ARID3C	9	34612389	5	0.314169
SNP9-34612389	ARID3C	9	34612389	4	0.017485
SNP9-34612389	ARID3C	9	34612389	1	3.260335
SNP9-34612389	ARID3C	9	34612389	6	0.246825
rs11574915	CCL21	9	34700084	2	0.9308
rs3739873	KIAA1045	9	34968431	7	0.259513
rs3739873	KIAA1045	9	34968431	3	0.171275
rs13301981	KIAA1045	9	34969623	6	0.206114
rs13301981	KIAA1045	9	34969623	3	1.754252
rs13301981	KIAA1045	9	34969623	1	1.95572
rs885451	DNAJB5	9	34987569	6	0.986042
rs885451	DNAJB5	9	34987569	1	5.415193
rs885451	DNAJB5	9	34987569	3	0.533415
rs615474	C9orf131	9	35033291	3	1.028134
rs615474	C9orf131	9	35033291	8	0.964915
rs615474	C9orf131	9	35033291	1	3.992308
SNP9-35034398	C9orf131	9	35034398	1	2.660172
SNP9-35034398	C9orf131	9	35034398	7	0.484132
rs755774	LOC730112	9	35552549	7	1.583141
rs755774	LOC730112	9	35552549	8	0.84504
rs755774	LOC730112	9	35552549	6	0.11748
rs755774	LOC730112	9	35552549	4	0.335809
rs755774	LOC730112	9	35552549	3	0.6328
SNP9-35608053	CD72	9	35608053	7	6.312865
SNP9-35655153	C9orf100	9	35655153	1	4.365793
SNP9-35655153	C9orf100	9	35655153	6	1.932793
SNP9-35816038	C9orf128	9	35816038	6	1.147501
SNP9-35816038	C9orf128	9	35816038	5	0.256774
SNP9-35816038	C9orf128	9	35816038	4	1.982307
SNP9-35816058	C9orf128	9	35816058	4	0.377845
SNP9-35816058	C9orf128	9	35816058	5	0.444152
rs2236293	C9orf127	9	35831783	7	0.798768
rs2236293	C9orf127	9	35831783	8	0.193844

rs10491554	LOC158376	9	35900690	7	0.129179
rs10491554	LOC158376	9	35900690	4	1.906407
rs10491554	LOC158376	9	35900690	1	1.204751
rs10814297	LOC158376	9	35900945	4	1.108923
rs10814297	LOC158376	9	35900945	7	0.002572
rs10814297	LOC158376	9	35900945	2	0.1671
rs10814297	LOC158376	9	35900945	1	0.302788
rs11788747	RECK	9	36095264	7	0.597093
rs11788747	RECK	9	36095264	3	0.934494
rs10972727	RECK	9	36100063	7	5.853789
rs10972727	RECK	9	36100063	8	0.723669
rs10972727	RECK	9	36100063	3	0.163636
rs6610	C9orf19	9	36153563	1	0.758604
rs6610	C9orf19	9	36153563	3	0.707604
rs2840246	ZBTB5	9	37428526	1	5.516098
rs2840246	ZBTB5	9	37428526	6	2.365787
rs16934158	POLR1E	9	37479378	3	0.593321
rs16934158	POLR1E	9	37479378	4	0.805463
rs3739576	POLR1E	9	37482679	2	4.350395
rs7021977	C9orf105	9	37582452	2	4.228872
rs7021977	C9orf105	9	37582452	3	0.250955
rs2281872	FRMPD1	9	37730637	8	0.092628
rs3747541	FRMPD1	9	37735413	3	2.126293
rs3747541	FRMPD1	9	37735413	1	1.825974
rs3747541	FRMPD1	9	37735413	7	0.490756
rs10115457	WDR32	9	37851794	1	4.825812
rs10115457	WDR32	9	37851794	7	0.068172
rs4646769	LOC730157	9	38382938	2	3.077257
rs4646769	LOC730157	9	38382938	3	0.670276
rs3043	ALDH1B1	9	38387355	4	0.233378
rs7857013	IGFBPL1	9	38399037	7	1.72737
rs7857013	IGFBPL1	9	38399037	8	1.78868
rs7857013	IGFBPL1	9	38399037	1	1.974213
rs7857243	IGFBPL1	9	38399081	5	0.043905
rs7046879	C9orf122	9	38612368	5	0.353596
rs7046879	C9orf122	9	38612368	4	0.792339
rs13294677	LOC647051	9	38633129	3	0.085759
rs10869119	LOC347097	9	70414748	1	4.144689
rs10869119	LOC347097	9	70414748	3	3.91448
rs10869119	LOC347097	9	70414748	6	4.895982
rs2003195	LOC347097	9	70414952	1	4.950215
rs3730386	PRKACG	9	70818027	3	0.129407
rs2309428	TJP2	9	71032843	5	0.255094
SNP9-71931132	MAMDC2	9	71931132	4	0.228569
SNP9-72123594	SMC5	9	72123594	8	6.887509
SNP9-72123594	SMC5	9	72123594	4	0.539883
rs1123	GDA	9	74053707	8	1.754011

rs1123	GDA	9	74053707	1	5.378836
rs1123	GDA	9	74053707	4	0.214485
rs2208744	GDA	9	74054897	8	0.056723
rs2208744	GDA	9	74054897	6	1.247681
rs2208744	GDA	9	74054897	2	2.867208
rs2208744	GDA	9	74054897	7	0.103818
rs7022441	TMC1	9	74326609	1	3.576524
rs13959	ALDH1A1	9	74735702	6	1.7255
rs13959	ALDH1A1	9	74735702	2	3.108972
rs13959	ALDH1A1	9	74735702	1	0.391185
rs2273975	RORB	9	76447166	4	0.351156
rs2274924	TRPM6	9	76566467	5	0.082018
rs2274924	TRPM6	9	76566467	4	1.157818
rs4145894	TRPM6	9	76597456	8	3.62256
rs4145894	TRPM6	9	76597456	1	4.062367
rs4145894	TRPM6	9	76597456	7	0.42468
rs7859201	TRPM6	9	76605104	1	0.5437
rs7859201	TRPM6	9	76605104	8	4.651495
rs7859201	TRPM6	9	76605104	6	3.684346
rs11144089	TRPM6	9	76606792	6	1.347188
rs11144089	TRPM6	9	76606792	4	0.49078
rs2273769	OSTF1	9	76942330	2	1.94861
rs34417623	PCSK5	9	77828617	1	5.452226
rs2490582	RFK	9	78198926	4	0.445121
rs683969	GCNT1	9	78309572	7	0.21578
rs589561	GCNT1	9	78310295	2	0.700076
rs13290974	LOC392352	9	78354466	7	0.48185
rs1425286	NULL	9	78418844	1	5.04091
rs1425286	NULL	9	78418844	7	0.461267
rs4745571	NULL	9	78508187	4	0.277154
rs4745571	NULL	9	78508187	5	2.063767
rs560094	NULL	9	78508291	3	1.236468
rs560094	NULL	9	78508291	1	1.000641
rs560094	NULL	9	78508291	7	1.164928
rs560094	NULL	9	78508291	4	0.745229
rs560094	NULL	9	78508291	2	1.861112
rs561970	NULL	9	78508497	4	0.188974
rs561970	NULL	9	78508497	1	0.151565
rs561970	NULL	9	78508497	3	0.745729
rs561970	NULL	9	78508497	2	0.840238
rs620985	NULL	9	78508741	3	0.504451
rs620985	NULL	9	78508741	8	3.020438
rs620985	NULL	9	78508741	1	1.379374
rs620985	NULL	9	78508741	2	1.02826
rs620985	NULL	9	78508741	4	0.929345
rs3739524	NULL	9	78509915	8	0.379503
rs512110	NULL	9	78510460	3	0.709589

rs512110	NULL	9	78510460	1	1.09478
rs517634	NULL	9	78511096	2	0.399606
rs517634	NULL	9	78511096	1	0.121641
rs517634	NULL	9	78511096	4	0.080304
rs517634	NULL	9	78511096	3	0.586042
rs577993	NULL	9	78511691	4	1.206195
rs577993	NULL	9	78511691	7	0.718636
rs577993	NULL	9	78511691	2	2.24605
rs577993	NULL	9	78511691	3	1.021195
rs577993	NULL	9	78511691	8	3.778418
rs577993	NULL	9	78511691	1	1.516688
rs683866	NULL	9	78511888	3	0.715477
rs683866	NULL	9	78511888	1	1.295784
rs683866	NULL	9	78511888	2	1.142554
rs680775	NULL	9	78512494	3	1.007974
rs680775	NULL	9	78512494	1	0.994253
rs680729	NULL	9	78512540	2	1.944561
rs680729	NULL	9	78512540	1	0.561509
rs680729	NULL	9	78512540	3	0.457511
rs530978	NULL	9	78513999	8	4.2758
rs530978	NULL	9	78513999	3	1.060702
rs530978	NULL	9	78513999	2	3.755509
rs530978	NULL	9	78513999	4	5.602209
rs530978	NULL	9	78513999	1	0.879173
rs530978	NULL	9	78513999	7	1.366605
rs12238013	NULL	9	78514259	8	5.341271
rs557352	NULL	9	78514561	8	0.218808
rs557352	NULL	9	78514561	3	0.384577
rs557352	NULL	9	78514561	1	0.426676
rs557352	NULL	9	78514561	6	1.391375
rs557352	NULL	9	78514561	4	0.901552
rs557352	NULL	9	78514561	7	0.245058
rs557352	NULL	9	78514561	2	4.150951
rs11145017	NULL	9	78514775	8	0.135331
rs611461	NULL	9	78515435	4	0.246399
rs611461	NULL	9	78515435	3	0.437885
rs611461	NULL	9	78515435	1	0.005462
rs611461	NULL	9	78515435	2	0.818926
rs11145226	LOC645225	9	78845248	7	0.006144
rs11145227	LOC645225	9	78845315	7	0.32816
SNP9-79080826	VPS13A	9	79080826	1	5.3735
rs1801258	GNA14	9	79239118	5	0.576981
rs1801258	GNA14	9	79239118	4	5.583468
rs1801258	GNA14	9	79239118	3	0.38889
rs10122426	KRT18P24	9	80841766	1	1.967396
rs4750	GKAP1	9	85544331	5	0.159744
rs4750	GKAP1	9	85544331	1	6.567031

rs4750	GKAP1	9	85544331	7	0.333494
SNP9-85663935	KIF27	9	85663935	1	0.188162
SNP9-85663935	KIF27	9	85663935	2	2.379779
SNP9-85663935	KIF27	9	85663935	6	3.176423
SNP9-85663935	KIF27	9	85663935	5	0.49039
rs167203	HNRPK	9	85783134	7	0.597766
rs296887	HNRPK	9	85784890	6	1.200469
rs296887	HNRPK	9	85784890	2	3.766343
rs296887	HNRPK	9	85784890	7	2.298413
rs796003	RMI1	9	85785621	6	0.28865
rs796003	RMI1	9	85785621	2	3.875077
rs796003	RMI1	9	85785621	7	0.743017
rs1982151	RMI1	9	85807085	7	1.158232
rs1982151	RMI1	9	85807085	6	2.537904
rs1982151	RMI1	9	85807085	2	4.216603
rs1187325	NTRK2	9	86475415	4	0.466609
rs1187325	NTRK2	9	86475415	3	0.220521
rs1187325	NTRK2	9	86475415	5	0.31512
rs1187325	NTRK2	9	86475415	7	0.262067
rs7816	NTRK2	9	86620341	7	0.234428
rs7816	NTRK2	9	86620341	5	0.656625
rs10780691	NTRK2	9	86681073	1	2.555476
rs10780691	NTRK2	9	86681073	8	0.49491
rs10780691	NTRK2	9	86681073	7	0.098225
rs7450	MAK10	9	87826707	4	5.199428
rs7450	MAK10	9	87826707	5	8.609934
rs2225237	GOLM1	9	87904824	5	0.268606
rs2225237	GOLM1	9	87904824	4	1.253268
rs4440696	ZCCHC6	9	88093187	5	6.63483
rs4440696	ZCCHC6	9	88093187	6	0.026298
rs4440696	ZCCHC6	9	88093187	3	0.52235
rs4440696	ZCCHC6	9	88093187	2	6.078143
rs4440696	ZCCHC6	9	88093187	8	6.152801
rs791323	ZCCHC6	9	88149758	2	3.14876
rs791323	ZCCHC6	9	88149758	8	0.295622
rs791323	ZCCHC6	9	88149758	5	9.636997
rs461126	FLJ45537	9	88953394	8	0.663035
rs277755	FLJ45537	9	88962233	7	2.33343
rs277755	FLJ45537	9	88962233	1	0.8219
rs277755	FLJ45537	9	88962233	3	0.189267
rs277753	FLJ45537	9	88962836	2	1.513674
rs277753	FLJ45537	9	88962836	1	2.004314
rs3818584	DAPK1	9	89454835	3	0.913095
rs3818584	DAPK1	9	89454835	1	1.759863
SNP9-89511843	DAPK1	9	89511843	2	0.360928
SNP9-89511843	DAPK1	9	89511843	5	6.519736
rs10735592	CTSL3	9	89578359	6	4.248715

rs4076795	C9orf79	9	89691268	4	0.326924
rs4076795	C9orf79	9	89691268	3	0.030428
rs4076794	C9orf79	9	89691334	5	1.056391
rs11789780	C9orf79	9	89692827	5	1.194036
rs11789780	C9orf79	9	89692827	3	0.592682
rs11789780	C9orf79	9	89692827	4	0.786284
rs11789780	C9orf79	9	89692827	8	1.659427
rs11789780	C9orf79	9	89692827	6	1.009356
rs11789780	C9orf79	9	89692827	2	0.356484
rs28364935	CCRK	9	89778790	6	0.072647
rs3818707	LOC389768	9	89985821	5	4.806337
rs3818707	LOC389768	9	89985821	6	4.062048
rs2814847	LOC389768	9	89986087	1	1.1248
rs2814848	LOC389768	9	89986088	7	1.068496
rs4269628	NXNL2	9	90379897	3	0.095934
rs6559331	C9orf47	9	90797689	6	0.326482
rs6559331	C9orf47	9	90797689	5	1.41344
rs3934594	C9orf47	9	90800363	6	1.427921
rs3934594	C9orf47	9	90800363	1	2.364408
rs7045576	C9orf47	9	90800749	1	1.789361
rs1867	EDG3	9	90809604	2	2.702022
rs1867	EDG3	9	90809604	1	2.138068
rs12519	SHC3	9	90817902	3	0.472134
rs12519	SHC3	9	90817902	1	2.871161
rs4876978	C9orf164	9	91166340	2	6.107763
rs4876978	C9orf164	9	91166340	6	2.050291
rs4876978	C9orf164	9	91166340	7	1.411558
rs4876978	C9orf164	9	91166340	3	2.356458
rs4876978	C9orf164	9	91166340	5	1.850656
rs13283164	C9orf164	9	91169258	3	0.248111
rs11526468	SEMA4D	9	91193499	3	0.361329
rs11526468	SEMA4D	9	91193499	5	3.517191
rs1542479	DIRAS2	9	92412869	4	0.843222
rs1542479	DIRAS2	9	92412869	7	0.037461
rs726214	DIRAS2	9	92413821	1	4.387597
rs726214	DIRAS2	9	92413821	5	0.353217
rs1049164	SYK	9	92698027	1	3.021935
rs7874056	AUH	9	93127443	1	3.749268
rs10820900	ROR2	9	93535429	5	5.059938
rs10820900	ROR2	9	93535429	4	0.77278
rs16907720	ROR2	9	93539618	3	0.862837
rs16907720	ROR2	9	93539618	4	0.097695
rs16907720	ROR2	9	93539618	5	2.969201
rs1058751	IARS	9	94012737	7	9.164506
rs710162	NOL8	9	94099611	1	4.341421
rs710162	NOL8	9	94099611	8	1.002625
rs710162	NOL8	9	94099611	6	3.65012

rs710162	NOL8	9	94099611	3	0.014712
rs4298540	NOL8	9	94099654	3	0.062596
rs7027898	NOL8	9	94107867	1	4.350023
rs7027898	NOL8	9	94107867	6	0.250142
rs7027898	NOL8	9	94107867	3	0.213933
rs17678735	NOL8	9	94117648	3	0.157595
rs10123342	CENPP	9	94127645	1	4.444129
rs10123342	CENPP	9	94127645	6	0.453556
rs10123342	CENPP	9	94127645	8	1.827937
rs1053446	OGN	9	94186582	1	6.650029
rs1380	OGN	9	94187255	1	4.884429
rs1053441	OGN	9	94187661	3	0.188724
rs1053441	OGN	9	94187661	6	1.313539
SNP9-94219000	OMD	9	94219000	3	0.796741
rs17519719	ASPN	9	94258420	3	0.223319
rs8067	ASPN	9	94258650	1	4.569908
rs8067	ASPN	9	94258650	3	0.172415
rs3174352	ASPN	9	94259069	3	0.326142
rs3174352	ASPN	9	94259069	1	3.626031
rs17591776	ASPN	9	94259140	3	0.163669
rs2296667	ECM2	9	94314159	6	0.675117
rs2296667	ECM2	9	94314159	3	1.364303
rs2296667	ECM2	9	94314159	1	6.038015
rs968040	ECM2	9	94319806	1	4.037371
SNP9-94415289	CENPP	9	94415289	3	0.369994
rs2277170	IPPK	9	94436533	3	0.042421
rs7045305	EEF1DP2	9	94639646	7	0.600218
rs7045305	EEF1DP2	9	94639646	5	1.383566
rs3802381	FGD3	9	94778602	7	0.276591
rs3802381	FGD3	9	94778602	1	1.35559
rs4073460	FGD3	9	94837820	5	0.020658
rs4073460	FGD3	9	94837820	4	0.832385
rs4073460	FGD3	9	94837820	6	1.491975
rs4073460	FGD3	9	94837820	1	0.737987
rs4744201	WNK2	9	95055077	5	0.914281
rs4744201	WNK2	9	95055077	1	3.894948
rs4744201	WNK2	9	95055077	6	4.665025
rs4744201	WNK2	9	95055077	3	0.448966
SNP9-95061133	WNK2	9	95061133	3	0.279207
SNP9-95061133	WNK2	9	95061133	1	2.817756
SNP9-95061133	WNK2	9	95061133	5	7.823352
SNP9-95061133	WNK2	9	95061133	6	0.157557
rs4744216	WNK2	9	95092116	1	3.522521
rs1055710	FAM120AOS	9	95254749	5	0.790021
rs1055710	FAM120AOS	9	95254749	3	0.972989
rs1055710	FAM120AOS	9	95254749	1	2.661333
rs7038310	PHF2	9	95447741	6	0.468286

rs7038310	PHF2	9	95447741	3	0.45105
rs1536690	ZNF169	9	96095131	4	3.06015
rs1536690	ZNF169	9	96095131	2	3.362957
rs1536690	ZNF169	9	96095131	8	4.280505
rs1048510	FBP2	9	96360948	1	2.373249
rs1048510	FBP2	9	96360948	7	1.177992
rs2296707	FBP1	9	96441607	3	2.860147
rs357564	PTCH1	9	97249415	3	1.009017
rs357564	PTCH1	9	97249415	7	0.693559
rs357564	PTCH1	9	97249415	5	4.761265
rs357564	PTCH1	9	97249415	1	6.248459
rs690528	LOC375748	9	97678109	6	0.34681
rs690528	LOC375748	9	97678109	8	6.009525
rs690528	LOC375748	9	97678109	1	3.759259
rs690528	LOC375748	9	97678109	3	0.096383
rs10512243	C9orf102	9	97815280	1	6.369836
rs2066479	HSD17B3	9	98037631	3	0.522543
rs2066484	HSD17B3	9	98104217	3	1.336082
rs2066474	HSD17B3	9	98104246	5	2.286399
rs7030316	HABP4	9	98290284	1	4.611615
rs7030316	HABP4	9	98290284	4	1.312928
rs2493444	KIAA1529	9	99089791	4	2.066379
rs2487874	KIAA1529	9	99089793	4	0.682955
rs3747504	KIAA1529	9	99114267	3	1.587542
rs3747504	KIAA1529	9	99114267	7	0.380124
rs3747504	KIAA1529	9	99114267	5	2.158977
rs3747504	KIAA1529	9	99114267	2	4.487398
rs3747504	KIAA1529	9	99114267	8	3.538989
SNP9-99127166	KIAA1529	9	99127166	3	0.700685
SNP9-99127166	KIAA1529	9	99127166	7	0.29685
SNP9-99127166	KIAA1529	9	99127166	2	2.080209
SNP9-99127166	KIAA1529	9	99127166	4	1.058357
SNP9-99127166	KIAA1529	9	99127166	8	1.329132
SNP9-99127166	KIAA1529	9	99127166	5	4.637595
rs1947717	KIAA1529	9	99145556	5	1.225646
rs1947717	KIAA1529	9	99145556	8	2.145619
rs1947717	KIAA1529	9	99145556	3	1.462536
rs1947717	KIAA1529	9	99145556	4	5.566036
rs1947717	KIAA1529	9	99145556	2	3.475156
rs1947717	KIAA1529	9	99145556	7	0.290398
rs2061634	KIAA1529	9	99145603	4	1.432282
rs3747495	KIAA1529	9	99162112	7	0.42506
rs3747495	KIAA1529	9	99162112	2	2.479161
rs3747495	KIAA1529	9	99162112	8	1.145505
rs3747495	KIAA1529	9	99162112	3	1.262105
rs3747495	KIAA1529	9	99162112	5	0.998287
rs11581	KIAA1529	9	99178982	2	0.684506

rs11581	KIAA1529	9	99178982	3	0.447632
rs11581	KIAA1529	9	99178982	7	1.695554
rs898870	KIAA1529	9	99179217	3	1.106439
rs898870	KIAA1529	9	99179217	2	2.034759
rs898870	KIAA1529	9	99179217	7	0.414358
rs1800975	XPA	9	99499399	3	1.807622
rs1800975	XPA	9	99499399	4	1.621055
rs1800975	XPA	9	99499399	6	0.592557
rs1800975	XPA	9	99499399	5	1.265142
rs10984009	FOXE1	9	99658481	6	2.809338
rs6586	C9orf156	9	99706752	7	0.071189
rs6586	C9orf156	9	99706752	5	5.580099
rs6586	C9orf156	9	99706752	2	3.585231
rs6586	C9orf156	9	99706752	8	1.136907
rs3802475	C9orf156	9	99724540	7	0.797751
rs3802475	C9orf156	9	99724540	1	0.172436
rs3183927	C9orf156	9	99724578	8	2.740747
rs3183927	C9orf156	9	99724578	7	0.804032
rs3183927	C9orf156	9	99724578	4	0.277279
rs3183927	C9orf156	9	99724578	1	3.366579
rs1573025	TBC1D2	9	100031251	7	0.598589
rs10124070	GABBR2	9	100091700	1	2.499878
rs2808536	GABBR2	9	100380122	5	1.689854
rs10760479	ANKS6	9	100538385	4	4.819118
rs10760479	ANKS6	9	100538385	2	6.046799
rs2273846	GALNT12	9	100651156	3	0.127878
rs2075662	COL15A1	9	100788055	4	2.667198
rs2297603	COL15A1	9	100818086	4	4.073938
rs420549	TGFBR1	9	100954694	5	0.105201
rs1526267	NR4A3	9	101629566	2	2.35512
rs1526267	NR4A3	9	101629566	6	2.192721
rs1526267	NR4A3	9	101629566	5	0.693957
rs10988904	NR4A3	9	101635506	4	0.106149
rs10988904	NR4A3	9	101635506	6	5.768388
rs10988904	NR4A3	9	101635506	5	5.518325
rs2900223	NR4A3	9	101636142	5	0.085703
rs1131339	NR4A3	9	101667123	7	0.821788
rs1131339	NR4A3	9	101667123	6	3.672651
rs1131339	NR4A3	9	101667123	8	1.154909
rs1131339	NR4A3	9	101667123	4	3.319874
rs1131339	NR4A3	9	101667123	1	5.583018
rs7024375	INVS	9	101901434	6	0.364602
rs7024375	INVS	9	101901434	4	0.087189
rs7024375	INVS	9	101901434	1	3.63506
rs1226590	LOC347273	9	102380190	3	1.113424
rs1226590	LOC347273	9	102380190	4	0.076112
rs1226590	LOC347273	9	102380190	8	2.51163

rs1226590	LOC347273	9	102380190	5	1.210512
rs1226590	LOC347273	9	102380190	2	4.397941
rs1226590	LOC347273	9	102380190	1	1.081678
rs2780955	LOC347273	9	102389031	5	0.549367
rs2780955	LOC347273	9	102389031	1	0.470718
rs2780955	LOC347273	9	102389031	8	0.819318
rs2780955	LOC347273	9	102389031	2	0.152761
rs2780955	LOC347273	9	102389031	4	0.228084
rs2780955	LOC347273	9	102389031	3	0.359693
rs13302189	LOC644160	9	102533779	8	0.434184
rs4742792	LOC644160	9	102533937	3	3.656658
rs7043029	BAAT	9	103163583	5	1.191817
rs7043029	BAAT	9	103163583	4	1.265706
rs7043029	BAAT	9	103163583	3	0.693589
rs2229594	BAAT	9	103164485	3	0.6338
rs2229594	BAAT	9	103164485	4	0.941554
rs2229594	BAAT	9	103164485	5	1.680249
rs1572983	BAAT	9	103173449	3	0.074967
rs1572983	BAAT	9	103173449	4	0.144233
rs1572983	BAAT	9	103173449	5	0.593595
rs546577	ZNF189	9	103212757	5	0.998725
rs546577	ZNF189	9	103212757	1	4.592554
rs546577	ZNF189	9	103212757	7	0.168124
rs17772869	ALDOB	9	103223706	4	2.425953
rs17772869	ALDOB	9	103223706	2	3.048503
rs17187712	C9orf125	9	103278551	7	1.218681
rs11546567	C9orf125	9	103279142	7	7.093332
rs11546567	C9orf125	9	103279142	4	0.88693
rs16920477	RNF20	9	103342446	1	3.988952
rs10521057	RNF20	9	103364366	5	1.206734
rs10521057	RNF20	9	103364366	6	0.588276
rs10521057	RNF20	9	103364366	1	5.023428
rs1983812	GRIN3A	9	103372864	1	0.654701
rs3824473	GRIN3A	9	103374598	2	2.156434
rs10989563	GRIN3A	9	103425532	6	4.787436
rs10989563	GRIN3A	9	103425532	5	3.396133
rs10989563	GRIN3A	9	103425532	8	2.626205
rs10989563	GRIN3A	9	103425532	2	2.970078
rs10989563	GRIN3A	9	103425532	1	5.106517
rs10820341	CYLC2	9	104820583	5	2.533133
rs3818626	SMC2	9	105896454	1	2.89126
rs3818626	SMC2	9	105896454	8	4.948279
rs3818626	SMC2	9	105896454	3	0.885153
rs3818626	SMC2	9	105896454	6	0.634472
rs10991205	LOC347281	9	106078011	3	0.114739
rs10991205	LOC347281	9	106078011	1	1.554638
rs1851724	OR13C5	9	106400743	3	0.788312

rs10761054	OR13C9	9	106419716	1	1.299479
rs10761054	OR13C9	9	106419716	2	1.758657
rs12347076	OR13D1	9	106496754	3	0.33692
rs12347076	OR13D1	9	106496754	5	0.466817
rs12338899	OR13D1	9	106497546	3	1.234402
rs12338899	OR13D1	9	106497546	2	2.015297
rs12338899	OR13D1	9	106497546	5	0.391231
rs10761084	NIPSNAP3B	9	106570973	5	3.724983
rs10761084	NIPSNAP3B	9	106570973	3	3.089435
rs3739741	NIPSNAP3B	9	106572996	3	0.992545
rs3739741	NIPSNAP3B	9	106572996	5	0.013597
rs10820730	NIPSNAP3B	9	106575843	3	0.457866
rs10820730	NIPSNAP3B	9	106575843	5	1.598872
rs363717	ABCA1	9	106584521	1	0.085323
rs363717	ABCA1	9	106584521	6	0.024496
rs4149313	ABCA1	9	106626574	4	0.159314
rs7031748	ABCA1	9	106631093	5	0.940015
rs7031748	ABCA1	9	106631093	4	1.351532
rs2274873	ABCA1	9	106642499	7	0.538179
rs2274873	ABCA1	9	106642499	3	0.221585
rs2230806	ABCA1	9	106660688	6	0.190521
rs2230806	ABCA1	9	106660688	5	0.875569
rs2230806	ABCA1	9	106660688	7	0.174325
rs2230805	ABCA1	9	106663850	5	2.013792
rs2230805	ABCA1	9	106663850	7	0.436826
rs2271247	TMEM38B	9	107496709	2	4.403366
rs2271247	TMEM38B	9	107496709	7	0.612979
SNP9-10872957	ZNF462	9	108729573	8	1.878077
SNP9-10872957	ZNF462	9	108729573	3	0.038898
rs10759212	LOC644661	9	108972698	1	2.842333
rs10759212	LOC644661	9	108972698	6	2.344125
rs10759212	LOC644661	9	108972698	5	1.817056
rs10759212	LOC644661	9	108972698	3	3.100299
rs3204145	IKBKAP	9	110691441	2	3.433416
rs10979601	IKBKAP	9	110700672	6	0.542698
rs10979601	IKBKAP	9	110700672	7	0.701365
rs10979604	IKBKAP	9	110703575	2	7.019769
rs10979605	IKBKAP	9	110703614	2	4.492873
rs12340246	IKBKAP	9	110719693	7	0.705532
rs2275639	IKBKAP	9	110735980	7	0.738917
rs2275640	IKBKAP	9	110736035	7	0.417883
rs10979727	EPB41L4B	9	110976609	7	0.275458
rs10979727	EPB41L4B	9	110976609	4	0.31546
rs10979729	EPB41L4B	9	110978747	7	0.686101
rs3750450	EPB41L4B	9	110984870	1	0.581769
rs3750450	EPB41L4B	9	110984870	5	0.539703
rs3750450	EPB41L4B	9	110984870	6	0.035829

rs3750447	EPB41L4B	9	110996420	7	0.766824
rs3750447	EPB41L4B	9	110996420	4	0.618088
rs17803052	PTPN3	9	111212495	2	2.511366
rs17803052	PTPN3	9	111212495	7	0.456006
rs3793524	PTPN3	9	111259295	7	0.360008
rs12376235	LOC402375	9	111335604	2	0.792952
rs12376235	LOC402375	9	111335604	7	1.442955
rs12376235	LOC402375	9	111335604	3	1.572812
rs3741894	PALM2	9	111751340	4	0.693795
SNP9-111939182	PALM2-AKAP2	9	111939182	2	6.193352
SNP9-111939182	PALM2-AKAP2	9	111939182	5	0.622987
SNP9-111939182	PALM2-AKAP2	9	111939182	7	0.21739
rs4978888	C9orf152	9	112003523	6	6.310619
rs4978888	C9orf152	9	112003523	8	0.995291
rs4978888	C9orf152	9	112003523	3	1.182714
rs7041938	TXNDC8	9	112131344	1	2.167402
rs1410049	SVEP1	9	112206518	7	6.737721
rs1410049	SVEP1	9	112206518	4	2.014439
rs1410049	SVEP1	9	112206518	6	6.121155
rs1410049	SVEP1	9	112206518	2	4.503621
rs3739451	SVEP1	9	112206613	1	2.518646
rs2281937	SVEP1	9	112208947	1	3.157221
rs7853155	SVEP1	9	112236495	3	1.540577
rs1889323	SVEP1	9	112249016	6	0.46451
rs1889323	SVEP1	9	112249016	2	2.817624
rs1889323	SVEP1	9	112249016	4	0.093665
rs7038903	SVEP1	9	112261068	3	2.409659
rs872665	SVEP1	9	112301304	4	1.845106
rs872665	SVEP1	9	112301304	3	1.706382
rs872665	SVEP1	9	112301304	8	8.16515
rs872665	SVEP1	9	112301304	2	5.663153
rs872665	SVEP1	9	112301304	7	0.844191
rs3818764	SVEP1	9	112316177	1	6.789428
rs2274419	MUSK	9	112577943	3	2.528347
rs2274419	MUSK	9	112577943	1	1.878042
rs955888	EDG2	9	112677483	7	0.321493
rs955888	EDG2	9	112677483	2	5.375846
rs955888	EDG2	9	112677483	8	7.248286
rs955888	EDG2	9	112677483	1	0.309734
rs6477824	ZNF483	9	113327274	5	0.643465
rs6477824	ZNF483	9	113327274	3	0.389359
rs10759501	ZNF483	9	113345397	2	4.121701
rs10759501	ZNF483	9	113345397	5	0.394303
rs10759501	ZNF483	9	113345397	4	2.160772
rs10759501	ZNF483	9	113345397	3	0.463833
SNP9-113399445	LTB4DH	9	113399445	2	1.878345
SNP9-113399445	LTB4DH	9	113399445	4	0.308285

rs7869279	C9orf84	9	113494365	3	0.887531
rs6477845	C9orf84	9	113502143	3	0.016431
rs11791445	C9orf84	9	113516569	7	0.339727
rs12352352	C9orf84	9	113524604	7	0.142832
rs12352352	C9orf84	9	113524604	8	2.718492
rs12352352	C9orf84	9	113524604	2	2.434775
rs10512411	C9orf84	9	113530050	2	3.932814
rs10512411	C9orf84	9	113530050	7	0.470605
rs7470491	C9orf84	9	113530129	3	0.418776
rs2281788	SUSD1	9	113843107	8	7.310449
rs2281790	SUSD1	9	113843452	4	7.248973
rs6575	ROD1	9	114020788	5	7.290025
rs6575	ROD1	9	114020788	6	5.742983
rs6575	ROD1	9	114020788	4	1.433155
rs14255	ROD1	9	114021199	6	3.569104
rs14255	ROD1	9	114021199	5	7.149445
rs7095	HSDL2	9	114274215	4	0.084518
rs2796036	SNX30	9	114551866	2	4.474129
rs2796036	SNX30	9	114551866	6	1.197068
rs2796036	SNX30	9	114551866	3	0.468645
rs2796036	SNX30	9	114551866	4	0.339634
SNP9-114638431	SNX30	9	114638431	8	1.131833
SNP9-114638431	SNX30	9	114638431	1	3.394988
rs16917417	SNX30	9	114672719	3	0.127442
rs16917440	SLC46A2	9	114681390	1	3.607178
rs3802491	SLC46A2	9	114692702	1	1.255179
rs3802491	SLC46A2	9	114692702	4	0.7393
rs13283782	SLC46A2	9	114692847	3	0.231412
rs10513176	LOC169834	9	114801042	3	0.191238
rs10981592	LOC169834	9	114804168	5	0.113725
rs2282076	ZFP37	9	114858770	8	0.900371
rs2282076	ZFP37	9	114858770	5	0.131273
rs1128786	SLC31A2	9	114966084	7	1.13103
rs1128786	SLC31A2	9	114966084	5	0.003333
rs1128786	SLC31A2	9	114966084	4	0.398497
SNP9-114973801	FKBP15	9	114973801	4	0.476755
SNP9-114973801	FKBP15	9	114973801	1	4.01743
rs10465129	FKBP15	9	114989977	8	5.261974
rs1133618	FKBP15	9	115008618	7	1.22574
rs1133618	FKBP15	9	115008618	2	4.55005
rs1133618	FKBP15	9	115008618	5	0.015802
rs2233914	FKBP15	9	115023361	2	6.040877
rs9299214	CDC26	9	115075517	8	1.383388
rs9299214	CDC26	9	115075517	4	0.663256
rs2418249	PRPF4	9	115093861	8	6.966588
rs3750535	RNF183	9	115099554	8	1.145228
rs3750535	RNF183	9	115099554	4	0.388052

rs3750533	RNF183	9	115100042	4	1.161859
rs16932583	WDR31	9	115118192	6	5.914128
rs41307479	WDR31	9	115122468	6	1.893634
rs10817479	WDR31	9	115125244	5	1.153674
rs818711	BSPRY	9	115171913	8	0.603647
rs17765613	HDHD3	9	115175976	4	6.155419
rs8177822	ALAD	9	115189530	2	2.264693
rs17525042	POLE3	9	115212488	7	1.03495
rs3810925	C9orf43	9	115213027	7	1.609853
SNP9-11522636	C9orf43	9	115226365	3	0.725382
SNP9-11522636	C9orf43	9	115226365	5	1.028824
rs34682731	RGS3	9	115331335	4	1.986237
rs7851718	RGS3	9	115343986	5	7.007119
rs7851718	RGS3	9	115343986	3	0.939267
rs7851937	RGS3	9	115344125	3	1.041684
rs7851937	RGS3	9	115344125	8	0.404557
rs3747813	RGS3	9	115367281	7	5.622447
rs12339493	RGS3	9	115382865	3	0.374491
SNP9-11538593	RGS3	9	115385939	5	5.361562
SNP9-11538593	RGS3	9	115385939	3	0.292932
rs3810927	RGS3	9	115386108	1	5.014909
rs12350531	RGS3	9	115396194	7	0.604328
rs12350531	RGS3	9	115396194	1	0.025776
rs12350531	RGS3	9	115396194	3	0.289717
rs7030167	ZNF618	9	115830982	3	0.278948
rs7030167	ZNF618	9	115830982	6	5.258834
rs12378906	ZNF618	9	115850025	7	0.491313
rs12554621	NULL	9	115856601	2	0.432238
rs12554621	NULL	9	115856601	1	0.418342
rs12554621	NULL	9	115856601	3	0.747297
rs2567707	COL27A1	9	115969921	7	3.349374
rs2567706	COL27A1	9	115970015	7	0.911306
rs2567706	COL27A1	9	115970015	4	1.51613
rs2241671	COL27A1	9	115970920	1	4.900235
rs2241671	COL27A1	9	115970920	4	0.123567
rs2808770	COL27A1	9	115971266	7	5.89232
rs2808770	COL27A1	9	115971266	4	0.020984
rs2808771	COL27A1	9	115971558	7	0.503395
rs2808771	COL27A1	9	115971558	4	0.43625
rs4143245	COL27A1	9	116072843	5	0.409344
rs4143245	COL27A1	9	116072843	4	0.30621
rs4143245	COL27A1	9	116072843	3	0.038025
rs4143245	COL27A1	9	116072843	8	1.548378
rs4143245	COL27A1	9	116072843	6	0.231665
rs4143245	COL27A1	9	116072843	1	0.427356
rs1388095	COL27A1	9	116109816	1	4.193126
rs2250242	AKNA	9	116143794	5	0.090629

rs3748176	AKNA	9	116164552	5	0.403381
rs3748176	AKNA	9	116164552	1	5.249444
rs1058278	C9orf91	9	116446422	1	3.512267
rs3810936	TNFSF15	9	116592706	6	0.36393
rs12347433	TNC	9	116837418	6	1.080803
rs12347433	TNC	9	116837418	4	0.264195
rs12347433	TNC	9	116837418	3	2.3035
rs12347433	TNC	9	116837418	5	0.957848
rs2104772	TNC	9	116848606	2	6.351362
rs2104772	TNC	9	116848606	7	0.36583
rs17819466	TNC	9	116859257	8	0.641105
rs17819466	TNC	9	116859257	4	4.487885
SNP9-11687572(	TNC	9	116875720	7	1.036359
SNP9-11687572(	TNC	9	116875720	2	6.940724
SNP9-11687572(	TNC	9	116875720	3	1.500835
SNP9-11687572(	TNC	9	116875720	1	1.165933
SNP9-116886401	TNC	9	116886401	8	0.546374
SNP9-116886401	TNC	9	116886401	5	0.290465
SNP9-116886401	TNC	9	116886401	1	4.015336
SNP9-116886401	TNC	9	116886401	6	0.099564
rs944510	TNC	9	116892843	8	7.486469
rs944510	TNC	9	116892843	6	4.28214
rs944510	TNC	9	116892843	5	5.859266
rs944510	TNC	9	116892843	1	5.42493
rs10982521	TNC	9	116892924	4	8.252887
rs1989775	C9orf27	9	117690510	1	5.148893
rs1661300	TRIM32	9	118501096	6	0.689759
rs3761845	ASTN2	9	118810301	5	0.412869
rs4986791	TLR4	9	119515423	5	0.073212
rs10984217	LOC442434	9	120564068	3	0.55751
rs10984217	LOC442434	9	120564068	1	1.242635
rs10984217	LOC442434	9	120564068	4	1.605974
rs7029069	LOC442434	9	120564106	1	2.594479
rs7029069	LOC442434	9	120564106	5	1.966607
rs7849696	LOC442434	9	120566091	3	0.408657
rs1472883	LOC442434	9	120566228	7	0.148058
rs1472883	LOC442434	9	120566228	5	1.740384
rs1472883	LOC442434	9	120566228	1	1.429921
rs3739822	CDK5RAP2	9	122202874	2	5.059011
rs3780679	CDK5RAP2	9	122245733	2	5.629279
SNP9-12227946	CDK5RAP2	9	122279464	8	0.329582
rs4836822	CDK5RAP2	9	122330857	3	0.053203
rs3750494	CDK5RAP2	9	122341167	3	0.858132
rs9886724	TRAF1	9	122704840	7	0.091723
rs9886724	TRAF1	9	122704840	2	1.328106
rs9886724	TRAF1	9	122704840	1	0.54206
rs9886724	TRAF1	9	122704840	6	3.304524

rs2239657	TRAF1	9	122711341	6	1.003807
rs2239657	TRAF1	9	122711341	5	1.685506
rs2239657	TRAF1	9	122711341	7	1.030805
rs2239657	TRAF1	9	122711341	1	0.816235
rs2239657	TRAF1	9	122711341	2	1.120331
rs2239657	TRAF1	9	122711341	4	3.952249
rs34119250	TRAF1	9	122728038	5	0.717699
rs17611	C5	9	122809021	7	0.136979
rs17611	C5	9	122809021	4	10.16889
rs17611	C5	9	122809021	1	4.731477
SNP9-12282212(	C5	9	122822120	8	5.152308
rs17216529	C5	9	122840039	4	0.196117
rs17216529	C5	9	122840039	1	0.306164
rs10818503	CEP110	9	122890591	2	5.229843
rs10818503	CEP110	9	122890591	4	0.277049
rs10818503	CEP110	9	122890591	1	0.442147
rs9408926	CEP110	9	122954695	6	4.988095
rs3736855	CEP110	9	122956841	1	2.546701
rs3736855	CEP110	9	122956841	4	0.901789
SNP9-12296959	CEP110	9	122969591	6	0.024184
SNP9-12296959	CEP110	9	122969591	2	0.956149
SNP9-12312872	GSN	9	123128729	3	0.047276
SNP9-12312872	GSN	9	123128729	7	0.176076
SNP9-12312951	GSN	9	123129513	7	0.639858
SNP9-12312951	GSN	9	123129513	3	1.011276
SNP9-12389503	TTLL11	9	123895032	6	5.120058
SNP9-12389503	TTLL11	9	123895032	5	0.07137
SNP9-12389503	TTLL11	9	123895032	3	0.702882
rs10306196	PTGS1	9	124197137	6	3.330253
rs4836891	OR1J2	9	124313395	3	0.001921
rs4836891	OR1J2	9	124313395	6	0.341248
rs1341043	OR1N2	9	124355863	1	1.089965
rs1341043	OR1N2	9	124355863	2	0.501976
rs1341043	OR1N2	9	124355863	7	0.58557
rs1341043	OR1N2	9	124355863	8	0.488112
rs1341044	OR1N2	9	124356000	8	2.076536
rs1341044	OR1N2	9	124356000	1	2.67342
rs1341044	OR1N2	9	124356000	2	3.834769
rs1341044	OR1N2	9	124356000	6	2.358972
rs1411272	OR1N2	9	124356171	1	0.308996
rs1411272	OR1N2	9	124356171	6	0.647229
rs1411272	OR1N2	9	124356171	2	0.473922
rs10739614	OR1L8	9	124369946	6	0.349622
rs10739614	OR1L8	9	124369946	1	0.019531
rs10739614	OR1L8	9	124369946	2	0.330144
rs10739614	OR1L8	9	124369946	7	0.19626
rs10985702	OR1L8	9	124370005	6	1.008175

rs10985702	OR1L8	9	124370005	8	0.188429
rs10985704	OR1L8	9	124370499	2	1.001953
rs10985704	OR1L8	9	124370499	6	0.274085
rs10985704	OR1L8	9	124370499	7	0.100108
rs10985704	OR1L8	9	124370499	1	1.200955
rs10985704	OR1L8	9	124370499	8	0.035066
rs1999182	OR1L8	9	124370560	8	0.754496
rs1999182	OR1L8	9	124370560	6	0.041715
rs1999182	OR1L8	9	124370560	1	0.436392
rs1999182	OR1L8	9	124370560	7	1.408643
rs1999182	OR1L8	9	124370560	2	0.893928
rs972925	OR1Q1	9	124416908	5	0.350709
rs972925	OR1Q1	9	124416908	4	4.050265
rs972925	OR1Q1	9	124416908	1	3.69732
rs1329957	OR1Q1	9	124417326	4	0.373079
rs1329957	OR1Q1	9	124417326	1	2.799151
rs1329957	OR1Q1	9	124417326	5	0.313701
rs1329957	OR1Q1	9	124417326	7	0.07965
rs1476860	OR1B1	9	124431062	8	3.984562
rs1536929	OR1B1	9	124431190	1	4.348625
rs1536929	OR1B1	9	124431190	4	0.825159
rs1536929	OR1B1	9	124431190	5	0.670105
rs1536929	OR1B1	9	124431190	6	0.660964
rs1536928	OR1B1	9	124431230	8	0.297271
rs1536928	OR1B1	9	124431230	3	0.299753
rs1536928	OR1B1	9	124431230	1	2.133013
rs1536928	OR1B1	9	124431230	4	0.951512
rs1536928	OR1B1	9	124431230	5	0.387174
SNP9-12516803	CRB2	9	125168032	5	0.215034
rs1048616	DENND1A	9	125182054	7	0.283949
rs3739837	DENND1A	9	125183483	1	4.322503
rs3739837	DENND1A	9	125183483	4	0.207951
rs2065221	LOC613206	9	126060649	8	0.056391
rs2065221	LOC613206	9	126060649	3	1.417842
rs2065221	LOC613206	9	126060649	2	3.177044
rs2065221	LOC613206	9	126060649	5	0.053216
rs2065221	LOC613206	9	126060649	7	0.389367
rs2065221	LOC613206	9	126060649	4	0.4065
rs2416	NEK6	9	126153481	8	0.394899
rs7861556	GPR144	9	126255165	3	0.799883
rs7861556	GPR144	9	126255165	1	1.195293
rs10760365	GPR144	9	126270712	1	0.059608
rs10760365	GPR144	9	126270712	5	0.155311
rs10760365	GPR144	9	126270712	4	0.087485
rs10760365	GPR144	9	126270712	3	0.518306
rs915034	NR5A1	9	126284776	3	0.001967
rs915034	NR5A1	9	126284776	1	0.16159

rs1051358	ARPC5L	9	126679516	4	0.5877
rs1051358	ARPC5L	9	126679516	5	0.01436
rs17327817	GOLGA1	9	126681957	3	0.836728
rs17327817	GOLGA1	9	126681957	8	7.803924
rs17327817	GOLGA1	9	126681957	5	0.251293
rs764304	C9orf126	9	126804126	3	0.876214
rs764304	C9orf126	9	126804126	5	3.62661
rs459311	LOC645842	9	126992031	1	0.972658
rs459311	LOC645842	9	126992031	3	1.060962
rs459311	LOC645842	9	126992031	6	0.172146
rs16927965	RABEPK	9	127015475	3	0.197708
rs16927965	RABEPK	9	127015475	4	0.937219
rs16927965	RABEPK	9	127015475	5	1.538414
rs722538	FAM125B	9	128197823	3	1.030617
rs722538	FAM125B	9	128197823	6	6.24618
rs722538	FAM125B	9	128197823	5	0.176359
rs11793111	FAM125B	9	128198192	5	0.270768
rs2241011	FAM125B	9	128199379	3	0.017555
rs2241011	FAM125B	9	128199379	5	0.285865
rs2241011	FAM125B	9	128199379	6	0.045333
rs2241011	FAM125B	9	128199379	8	0.426892
rs2241011	FAM125B	9	128199379	4	0.476356
rs2052156	FAM125B	9	128199704	1	1.44227
rs3814127	FAM125B	9	128305563	8	0.527297
rs3814127	FAM125B	9	128305563	7	1.60111
rs3814127	FAM125B	9	128305563	3	0.760441
rs3814126	FAM125B	9	128305618	3	0.076655
rs3814126	FAM125B	9	128305618	1	2.314265
rs3814126	FAM125B	9	128305618	4	0.760194
rs7861319	FAM125B	9	128308838	4	0.230424
rs7861319	FAM125B	9	128308838	8	1.328279
rs7861319	FAM125B	9	128308838	7	0.23476
rs13295990	LMX1B	9	128495408	3	0.166611
rs13295990	LMX1B	9	128495408	7	0.651211
rs13295990	LMX1B	9	128495408	6	2.124265
rs13295990	LMX1B	9	128495408	5	0.09734
rs10819246	ZBTB34	9	128683117	7	1.622202
rs10819246	ZBTB34	9	128683117	5	5.076562
rs3118832	LOC729721	9	128716872	7	0.674323
rs3118832	LOC729721	9	128716872	5	0.310032
rs8838	ANGPTL2	9	128889701	6	2.318534
rs8838	ANGPTL2	9	128889701	1	2.905605
rs8838	ANGPTL2	9	128889701	5	2.147597
rs8838	ANGPTL2	9	128889701	3	0.947785
rs2297866	ANGPTL2	9	128894020	5	2.246324
rs2297866	ANGPTL2	9	128894020	3	0.399431
rs1058266	RALGPS1	9	129024929	6	3.747189

SNP9-129156479	GARNL3	9	129156479	2	3.700167
SNP9-129205794	SLC2A8	9	129205794	7	0.335684
rs3088163	SLC2A8	9	129209821	7	0.700653
rs3088163	SLC2A8	9	129209821	1	5.708051
rs13292096	ZNF79	9	129231007	5	0.316592
rs13292096	ZNF79	9	129231007	1	3.395975
rs4240420	FAM129B	9	129381074	7	0.570066
rs522328	C9orf117	9	129514832	7	0.774932
rs482095	TTC16	9	129533334	7	1.043217
rs505268	TTC16	9	129533545	7	0.899427
rs534652	TOR2A	9	129534488	7	0.798767
rs564754	TOR2A	9	129535454	7	1.228626
rs514024	SH2D3C	9	129543891	5	0.926452
rs514024	SH2D3C	9	129543891	7	1.272508
rs514024	SH2D3C	9	129543891	3	1.060044
rs8083	CDK9	9	129592000	1	0.536939
rs8083	CDK9	9	129592000	3	0.419733
rs8083	CDK9	9	129592000	2	6.464406
rs6781	DPM2	9	129737864	5	0.029639
rs6781	DPM2	9	129737864	8	6.226412
rs3003609	DNM1	9	130024576	4	3.377394
rs3003609	DNM1	9	130024576	3	0.276963
rs3003609	DNM1	9	130024576	1	5.356839
rs7258	CERCAM	9	130236516	5	1.023286
rs7258	CERCAM	9	130236516	3	1.024443
rs7259	CERCAM	9	130236525	3	1.834598
rs7259	CERCAM	9	130236525	5	1.864048
rs3750333	SPTAN1	9	130419819	2	5.312304
rs11539209	ZDHHC12	9	130523372	6	1.10295
rs11539209	ZDHHC12	9	130523372	4	0.611088
SNP9-130605375	TBC1D13	9	130605375	6	0.127027
SNP9-130605375	TBC1D13	9	130605375	4	0.440975
rs7033070	C9orf114	9	130626195	4	1.232606
rs6478854	C9orf114	9	130628709	6	0.10741
rs6478854	C9orf114	9	130628709	4	0.084066
rs10988134	CCBL1	9	130635228	6	1.069198
rs10988134	CCBL1	9	130635228	4	0.417634
rs3750318	LRRC8A	9	130710773	5	0.475436
rs11542344	LRRC8A	9	130719998	1	3.490463
rs751340	PHYHD1	9	130729182	2	3.61361
rs751340	PHYHD1	9	130729182	8	6.179027
rs751340	PHYHD1	9	130729182	4	4.96894
rs751340	PHYHD1	9	130729182	6	7.669844
rs751340	PHYHD1	9	130729182	3	1.015946
rs2273866	PHYHD1	9	130742712	4	0.125799
rs2273866	PHYHD1	9	130742712	6	1.359146
rs2273866	PHYHD1	9	130742712	2	0.125964

rs2273866	PHYHD1	9	130742712	3	0.195855
rs2273866	PHYHD1	9	130742712	8	6.041235
rs11564096	DOLPP1	9	130886778	8	0.296102
rs3118625	PPP2R4	9	130913077	6	0.378806
rs3118625	PPP2R4	9	130913077	7	0.132885
rs3118625	PPP2R4	9	130913077	8	0.721228
rs3118625	PPP2R4	9	130913077	5	1.309036
rs2480452	PPP2R4	9	130949557	5	0.004024
rs2480452	PPP2R4	9	130949557	3	0.257631
SNP9-130949914	PPP2R4	9	130949914	6	0.142545
SNP9-130949914	PPP2R4	9	130949914	5	2.846447
SNP9-130949914	PPP2R4	9	130949914	2	3.102496
SNP9-130949914	PPP2R4	9	130949914	7	0.127754
rs4713	PPP2R4	9	130950626	5	0.288206
rs4713	PPP2R4	9	130950626	2	2.388898
rs4713	PPP2R4	9	130950626	8	6.517242
rs4713	PPP2R4	9	130950626	6	7.470672
rs4713	PPP2R4	9	130950626	7	0.241607
rs913770	C9orf106	9	131124442	3	0.011064
rs6478890	C9orf106	9	131125058	5	0.178078
rs6478890	C9orf106	9	131125058	3	1.129815
rs6478890	C9orf106	9	131125058	4	0.371553
rs7034728	C9orf106	9	131125114	3	1.828338
rs913772	C9orf106	9	131125456	6	0.686046
rs913772	C9orf106	9	131125456	3	0.354697
SNP9-131415253	C9orf50	9	131415253	1	1.320893
rs918165	C9orf50	9	131417721	6	0.706668
rs16936946	TOR1B	9	131609353	2	6.364392
rs3814547	C9orf78	9	131636842	4	1.29391
rs709611	FREQ	9	132038180	1	5.191463
rs11552451	FREQ	9	132038378	1	5.240579
rs10901072	ASS1	9	132336047	5	0.510078
rs10901072	ASS1	9	132336047	3	1.354422
rs4740197	LOC158301	9	132443241	4	0.722824
rs2229070	ABL1	9	132749850	5	0.550127
rs2229070	ABL1	9	132749850	4	3.034534
rs3847193	QRFP	9	132758990	6	2.106704
rs3847193	QRFP	9	132758990	8	2.463515
rs3847193	QRFP	9	132758990	5	0.501266
rs11789517	FIBCD1	9	132767814	5	0.433182
rs12349966	LAMC3	9	132918166	3	0.768206
rs12349966	LAMC3	9	132918166	5	1.329184
rs12349966	LAMC3	9	132918166	1	1.57275
rs3739510	LAMC3	9	132926392	8	0.011114
rs3739510	LAMC3	9	132926392	1	3.569088
rs3739510	LAMC3	9	132926392	3	0.819242
rs10901345	LAMC3	9	132936736	5	1.374712

rs10901345	LAMC3	9	132936736	3	1.926407
rs2275140	LAMC3	9	132937870	1	0.127591
rs818056	LAMC3	9	132957061	7	1.298581
rs818056	LAMC3	9	132957061	5	0.942044
rs818056	LAMC3	9	132957061	3	1.351111
rs710074	LAMC3	9	132957116	3	1.425569
rs710074	LAMC3	9	132957116	7	0.849692
rs353510	C9orf58	9	132986324	4	0.410236
rs353510	C9orf58	9	132986324	5	0.224481
rs1043169	C9orf58	9	132987364	4	0.118893
rs9966	FAM78A	9	133126069	1	0.544327
rs9966	FAM78A	9	133126069	7	0.34384
rs9966	FAM78A	9	133126069	5	0.486563
rs9966	FAM78A	9	133126069	2	1.208268
rs9966	FAM78A	9	133126069	8	0.273962
rs4740427	FAM78A	9	133141684	8	0.463186
rs4740427	FAM78A	9	133141684	3	1.305207
rs4740427	FAM78A	9	133141684	7	0.998272
rs4740427	FAM78A	9	133141684	1	3.464986
rs7041276	PPAPDC3	9	133155069	7	0.795893
rs7041276	PPAPDC3	9	133155069	1	1.485808
rs7041276	PPAPDC3	9	133155069	8	0.341528
rs7029366	PPAPDC3	9	133155189	8	1.649988
rs7029366	PPAPDC3	9	133155189	3	2.385025
rs7029366	PPAPDC3	9	133155189	1	0.455386
rs7029366	PPAPDC3	9	133155189	7	1.783783
rs2966332	PPAPDC3	9	133173200	7	0.106095
rs2986606	PPAPDC3	9	133173324	7	1.220662
rs2483476	PPAPDC3	9	133173734	4	5.389005
rs2483476	PPAPDC3	9	133173734	8	4.377651
rs1541122	PPAPDC3	9	133174294	4	0.742638
rs1541122	PPAPDC3	9	133174294	8	1.658436
rs1541122	PPAPDC3	9	133174294	7	0.09688
SNP9-13331177	KIAA0515	9	133311776	8	0.517708
SNP9-13332440	KIAA0515	9	133324409	5	0.165259
SNP9-13334027	KIAA0515	9	133340279	4	0.3046
SNP9-13334027	KIAA0515	9	133340279	6	7.448488
SNP9-13334027	KIAA0515	9	133340279	8	0.020359
SNP9-13336098	KIAA0515	9	133360986	6	0.059704
rs3887873	POMT1	9	133375256	4	2.845202
rs2296949	POMT1	9	133375257	6	0.646712
rs4740164	POMT1	9	133376602	6	4.462221
rs3739494	POMT1	9	133377309	6	4.418641
rs3739495	POMT1	9	133388355	6	0.059995
rs10793885	POMT1	9	133388592	6	0.777844
rs4740167	LOC642515	9	133423284	5	1.26996
rs11243480	RAPGEF1	9	133575002	6	0.371692

rs11243480	RAPGEF1	9	133575002	5	0.301862
rs11243480	RAPGEF1	9	133575002	7	1.345101
rs11243480	RAPGEF1	9	133575002	1	0.158551
rs2149171	NTNG2	9	134092075	5	0.068606
rs2149171	NTNG2	9	134092075	7	0.462691
rs562350	FLJ46082	9	134364719	5	0.160884
rs562350	FLJ46082	9	134364719	1	0.263853
rs562350	FLJ46082	9	134364719	6	2.515139
rs2231403	C9orf98	9	134743831	1	4.303122
rs2231403	C9orf98	9	134743831	6	1.33734
rs2231403	C9orf98	9	134743831	3	1.215148
rs7862221	TSC1	9	134772042	8	2.19747
rs667805	GFI1B	9	134856514	6	0.914907
rs637435	GTF3C5	9	134922056	3	0.638298
rs7324	GTF3C5	9	134923582	8	7.388444
rs7324	GTF3C5	9	134923582	1	0.135391
rs7324	GTF3C5	9	134923582	7	0.115606
rs1062356	RALGDS	9	134963900	5	0.741743
rs1062356	RALGDS	9	134963900	4	0.37131
rs1062356	RALGDS	9	134963900	6	0.906173
rs2073924	GBGT1	9	135027563	5	0.794967
rs2073923	GBGT1	9	135027666	2	2.130284
rs2073923	GBGT1	9	135027666	7	0.240404
rs2073923	GBGT1	9	135027666	5	0.078445
rs2073923	GBGT1	9	135027666	8	0.418945
rs11244035	OBP2B	9	135071140	3	0.078729
rs4507838	LCN1L1	9	135092020	8	4.905166
rs8176749	ABO	9	135121009	4	3.592316
rs8176745	ABO	9	135121168	5	1.082968
rs8176745	ABO	9	135121168	8	0.511519
rs8176745	ABO	9	135121168	1	3.227532
rs8176743	ABO	9	135121236	4	2.588633
rs8176742	ABO	9	135121258	5	0.517375
rs8176742	ABO	9	135121258	8	0.359555
rs8176742	ABO	9	135121258	1	3.641313
rs8176741	ABO	9	135121282	4	1.764854
rs8176740	ABO	9	135121293	5	0.618167
rs8176740	ABO	9	135121293	8	0.213531
rs8176740	ABO	9	135121293	1	3.922985
rs8176739	ABO	9	135121344	7	0.640704
rs8176739	ABO	9	135121344	5	0.723527
rs8176720	ABO	9	135122694	1	3.460869
rs8176720	ABO	9	135122694	5	0.998763
rs8176720	ABO	9	135122694	8	0.963785
rs8176720	ABO	9	135122694	4	2.203547
SNP9-13512272	ABO	9	135122729	3	0.523914
SNP9-13512272	ABO	9	135122729	6	0.287525

SNP9-13512272[illegible]	ABO	9	135122729	4	0.909323
rs512770	ABO	9	135123327	1	5.443033
rs512770	ABO	9	135123327	7	0.45736
rs549446	ABO	9	135125059	5	0.449056
rs549446	ABO	9	135125059	1	4.481977
rs688976	ABO	9	135126591	8	1.523368
rs688976	ABO	9	135126591	5	0.020306
rs688976	ABO	9	135126591	1	6.074163
rs10793964	SURF6	9	135188410	1	5.127559
rs10793964	SURF6	9	135188410	3	0.423917
rs622415	MED22	9	135198014	2	7.331351
rs616836	MED22	9	135203342	3	0.225419
rs616836	MED22	9	135203342	5	0.39867
rs616836	MED22	9	135203342	1	1.664832
rs616836	MED22	9	135203342	7	0.525714
rs616836	MED22	9	135203342	8	0.018052
rs553877	MED22	9	135204776	3	0.644573
rs553877	MED22	9	135204776	8	0.421517
rs553877	MED22	9	135204776	5	1.700826
rs553877	MED22	9	135204776	7	0.470688
rs553877	MED22	9	135204776	1	5.917439
SNP9-13520926[illegible]	SURF1	9	135209269	4	0.788466
SNP9-13525790[illegible]	C9orf96	9	135257905	3	0.120427
SNP9-13526035[illegible]	C9orf96	9	135260359	6	3.055393
rs3124782	C9orf96	9	135260817	7	0.952565
rs3124782	C9orf96	9	135260817	1	1.345572
rs3124782	C9orf96	9	135260817	3	1.516565
rs3124782	C9orf96	9	135260817	5	0.572523
rs3124782	C9orf96	9	135260817	2	1.961703
rs3118667	ADAMTS13	9	135280884	3	0.671143
rs3118667	ADAMTS13	9	135280884	7	1.317675
rs3118667	ADAMTS13	9	135280884	1	0.61812
rs3118667	ADAMTS13	9	135280884	2	3.545173
rs2301612	ADAMTS13	9	135291803	7	0.37759
rs2301612	ADAMTS13	9	135291803	1	4.180925
rs2301612	ADAMTS13	9	135291803	3	0.141668
rs3124768	ADAMTS13	9	135294318	7	0.685876
rs3124768	ADAMTS13	9	135294318	1	3.629564
rs3124768	ADAMTS13	9	135294318	3	0.390906
rs2301614	C9orf7	9	135314981	7	0.247871
rs2301614	C9orf7	9	135314981	3	0.440836
rs2301614	C9orf7	9	135314981	1	1.322302
GA026127	C9orf7	9	135318478	3	0.554907
GA026127	C9orf7	9	135318478	6	6.117198
GA026127	C9orf7	9	135318478	2	5.009055
GA026127	C9orf7	9	135318478	4	0.602826
rs3094379	C9orf7	9	135324731	3	0.216659

rs45489391	C9orf7	9	135325696	2	2.48132
rs3094378	SLC2A6	9	135326989	2	3.649561
rs17758315	RP13-388O5.1	9	135369711	3	0.521898
rs2073874	ADAMTSL2	9	135401991	7	0.140679
rs2073874	ADAMTSL2	9	135401991	8	0.775412
GA007926	ADAMTSL2	9	135424419	3	0.451354
GA007926	ADAMTSL2	9	135424419	7	0.530311
rs1108580	DBH	9	135494935	5	1.343687
rs5320	DBH	9	135497294	5	0.434193
rs129932	SARDH	9	135526500	3	2.321594
rs129932	SARDH	9	135526500	5	0.420446
rs129932	SARDH	9	135526500	2	1.898202
rs886016	SARDH	9	135545450	5	0.081129
rs2073817	SARDH	9	135549281	7	1.057023
rs2073817	SARDH	9	135549281	6	0.751206
rs2502740	SARDH	9	135551188	7	0.177245
rs573904	SARDH	9	135588967	3	0.908629
rs509590	VAV2	9	135618591	6	0.676933
rs509590	VAV2	9	135618591	8	0.449661
rs509590	VAV2	9	135618591	7	0.596916
rs509590	VAV2	9	135618591	1	4.355279
rs602990	VAV2	9	135633815	5	0.637761
rs7865299	VAV2	9	135652749	5	0.025176
rs408307	FLJ35348	9	135882344	3	0.866174
GA026139	WDR5	9	136013800	1	5.087874
GA026139	WDR5	9	136013800	7	4.908848
GA026139	WDR5	9	136013800	3	0.232114
GA007943	WDR5	9	136013888	4	1.305474
GA007943	WDR5	9	136013888	6	5.821666
rs41310207	COL5A1	9	136826772	6	0.010515
rs41310207	COL5A1	9	136826772	5	0.02517
rs13946	COL5A1	9	136874053	4	0.438521
rs13946	COL5A1	9	136874053	3	0.556702
rs13946	COL5A1	9	136874053	5	0.47162
rs13946	COL5A1	9	136874053	2	2.415482
rs3128575	COL5A1	9	136876471	7	0.776772
rs7851696	FCN2	9	136918912	5	1.535204
rs7851696	FCN2	9	136918912	2	2.63983
rs4521835	FCN2	9	136919127	7	0.728369
rs500461	OLFM1	9	137121948	4	4.802671
rs10858334	OLFM1	9	137129606	4	0.3765
rs10858334	OLFM1	9	137129606	7	0.647384
rs10858334	OLFM1	9	137129606	3	1.23243
rs11103684	OLFM1	9	137151891	6	0.781365
rs2254812	KIAA0649	9	137515693	6	8.683557
rs2254812	KIAA0649	9	137515693	4	6.015474
rs2254812	KIAA0649	9	137515693	8	5.179118

rs2254812	KIAA0649	9	137515693	5	3.341687
SNP9-137579613	OBP2A	9	137579613	1	2.577971
SNP9-137579627	OBP2A	9	137579627	5	4.008623
SNP9-137579627	OBP2A	9	137579627	4	1.583002
SNP9-137579627	OBP2A	9	137579627	7	4.729664
rs3178137	OBP2A	9	137580383	8	1.086697
rs3178137	OBP2A	9	137580383	7	0.072962
rs3178137	OBP2A	9	137580383	5	2.218447
rs3178137	OBP2A	9	137580383	4	1.756827
rs3178137	OBP2A	9	137580383	2	1.400101
rs2853652	OBP2A	9	137580462	7	4.005971
rs2853652	OBP2A	9	137580462	1	3.815152
rs2853652	OBP2A	9	137580462	8	3.807746
rs45618736	OBP2A	9	137580983	3	0.24721
SNP9-137581581	OBP2A	9	137581581	5	3.4948
SNP9-137581581	OBP2A	9	137581581	7	4.419397
rs17040344	GLT6D1	9	137655940	8	1.312617
rs914428	KCNT1	9	137809082	1	3.461273
rs914428	KCNT1	9	137809082	3	1.599844
rs2280486	BTBD14A	9	138044957	5	0.732964
rs2280486	BTBD14A	9	138044957	1	3.94531
rs2280486	BTBD14A	9	138044957	6	0.515081
rs2280486	BTBD14A	9	138044957	8	0.319938
rs2280486	BTBD14A	9	138044957	3	0.056556
rs2275107	BTBD14A	9	138048071	8	5.865259
rs2275107	BTBD14A	9	138048071	7	0.145203
SNP9-138258494	QSOX2	9	138258494	6	0.303874
SNP9-138258494	QSOX2	9	138258494	7	0.417993
SNP9-138258494	QSOX2	9	138258494	3	1.142247
SNP9-138355236	GPSM1	9	138355236	8	0.359815
SNP9-138355236	GPSM1	9	138355236	4	0.664366
SNP9-138355236	GPSM1	9	138355236	5	0.090621
SNP9-138355236	GPSM1	9	138355236	1	0.65497
SNP9-138355236	GPSM1	9	138355236	3	1.242132
SNP9-138355236	GPSM1	9	138355236	2	0.807606
rs3812547	GPSM1	9	138372316	5	2.985379
rs3812547	GPSM1	9	138372316	6	3.579974
rs3812547	GPSM1	9	138372316	3	0.860042
rs3812547	GPSM1	9	138372316	4	2.484677
rs3812547	GPSM1	9	138372316	2	2.042208
rs3812552	DNLZ	9	138376289	7	0.497997
rs3812552	DNLZ	9	138376289	6	0.485627
rs3812552	DNLZ	9	138376289	5	0.176934
rs10781499	CARD9	9	138386226	8	0.037987
rs10781499	CARD9	9	138386226	4	6.072834
rs10781499	CARD9	9	138386226	2	4.119631
rs10781499	CARD9	9	138386226	3	1.087054

rs10781499	CARD9	9	138386226	6	0.72666
rs10781499	CARD9	9	138386226	7	0.690184
rs4077515	CARD9	9	138386317	3	0.551669
rs4077515	CARD9	9	138386317	2	3.809314
rs4077515	CARD9	9	138386317	4	0.226373
rs4077515	CARD9	9	138386317	8	0.103676
rs4077515	CARD9	9	138386317	7	0.605159
rs4077515	CARD9	9	138386317	6	1.235916
rs3812570	SNAPC4	9	138395025	7	0.304238
rs3812570	SNAPC4	9	138395025	3	0.698836
rs10781510	SNAPC4	9	138398994	4	0.364969
rs10781510	SNAPC4	9	138398994	6	3.710604
rs10781510	SNAPC4	9	138398994	8	4.226657
rs10781510	SNAPC4	9	138398994	3	0.536143
rs10781510	SNAPC4	9	138398994	7	0.131635
SNP9-138418401	SDCCAG3	9	138418401	7	0.937054
SNP9-138418401	SDCCAG3	9	138418401	5	7.755934
SNP9-138418401	SDCCAG3	9	138418401	6	1.147779
rs4418446	SDCCAG3	9	138424845	3	0.109334
rs4418446	SDCCAG3	9	138424845	4	0.214138
rs4418446	SDCCAG3	9	138424845	5	1.05127
rs10870144	PMPCA	9	138426288	7	0.686308
rs10870144	PMPCA	9	138426288	5	0.962905
rs10870144	PMPCA	9	138426288	6	0.597455
rs33982662	INPP5E	9	138444558	8	0.563196
rs33982662	INPP5E	9	138444558	3	0.54156
rs33982662	INPP5E	9	138444558	4	0.048064
rs10870194	INPP5E	9	138446855	2	0.566728
rs10870194	INPP5E	9	138446855	3	0.181744
rs10870194	INPP5E	9	138446855	4	0.630667
rs10870194	INPP5E	9	138446855	7	0.603893
SNP9-138491383	SEC16A	9	138491383	7	0.04294
SNP9-138491383	SEC16A	9	138491383	6	7.022561
rs1139057	C9orf163	9	138500141	6	0.508034
rs1139057	C9orf163	9	138500141	8	0.62553
rs1139057	C9orf163	9	138500141	1	4.604046
rs1139057	C9orf163	9	138500141	4	0.541307
rs1139057	C9orf163	9	138500141	7	0.952556
rs45494498	EGFL7	9	138686942	4	0.132412
SNP9-138736563	FAM69B	9	138736563	7	0.222406
rs7038042	FAM69B	9	138737447	8	0.82757
rs7038042	FAM69B	9	138737447	5	3.645705
rs7038042	FAM69B	9	138737447	6	4.337083
rs6874	FAM69B	9	138737657	3	0.399059
rs6873	FAM69B	9	138738104	6	1.452838
rs6873	FAM69B	9	138738104	8	1.869521
rs6873	FAM69B	9	138738104	3	0.2907

rs6873	FAM69B	9	138738104	5	1.787769
rs2282258	LCN8	9	138769433	7	1.327856
rs945386	KIAA1984	9	138813417	2	1.061043
rs945386	KIAA1984	9	138813417	4	0.427707
rs945386	KIAA1984	9	138813417	7	0.375774
rs945386	KIAA1984	9	138813417	6	2.414959
rs7859194	KIAA1984	9	138814390	6	0.524354
rs7859194	KIAA1984	9	138814390	4	0.14012
rs7859194	KIAA1984	9	138814390	7	0.297514
rs7859194	KIAA1984	9	138814390	2	4.191488
rs2811795	KIAA1984	9	138820426	6	0.04046
rs2811795	KIAA1984	9	138820426	7	1.039764
rs2254143	KIAA1984	9	138820929	6	2.025561
rs2254143	KIAA1984	9	138820929	7	0.6402
rs2811748	C9orf86	9	138846060	2	6.361213
rs2811748	C9orf86	9	138846060	5	2.898711
rs2811748	C9orf86	9	138846060	3	2.64417
rs2811748	C9orf86	9	138846060	6	6.082608
rs2811748	C9orf86	9	138846060	1	2.455896
rs45449193	MAMDC4	9	138868304	5	0.750452
rs45449193	MAMDC4	9	138868304	8	0.042456
SNP9-138960381	C8G	9	138960381	7	1.298679
SNP9-138960381	C8G	9	138960381	8	3.682156
SNP9-138960381	C8G	9	138960381	1	0.625239
SNP9-138960381	C8G	9	138960381	5	1.368504
SNP9-138960381	C8G	9	138960381	2	0.73841
rs6926	PTGDS	9	138995962	4	0.801078
rs6926	PTGDS	9	138995962	5	0.021855
rs2049043	FLJ45224	9	138996950	5	3.922467
rs2049043	FLJ45224	9	138996950	6	3.644372
rs2049043	FLJ45224	9	138996950	8	0.072569
rs2049043	FLJ45224	9	138996950	4	2.847213
rs7029565	FLJ45224	9	138998138	5	0.446123
rs2292925	ENTPD2	9	139065184	5	0.657576
rs2292925	ENTPD2	9	139065184	8	0.887676
rs2292925	ENTPD2	9	139065184	6	1.117645
rs3750517	C9orf140	9	139078117	6	0.05362
rs3750517	C9orf140	9	139078117	5	0.921997
rs3750517	C9orf140	9	139078117	8	0.686192
rs867476	C9orf140	9	139078404	6	0.156106
rs867476	C9orf140	9	139078404	1	0.912502
rs867476	C9orf140	9	139078404	2	4.296648
GA008048	UAP1L1	9	139095016	1	5.716063
rs6293	GRIN1	9	139171059	3	0.755291
rs11549105	ANAPC2	9	139197460	3	1.222028
rs11549105	ANAPC2	9	139197460	1	4.699523
rs11556580	SSNA1	9	139204197	1	4.208374

rs11556580	SSNA1	9	139204197	3	0.089033
rs11526461	NDOR1	9	139231092	2	4.340072
rs28407527	SLC34A3	9	139247906	1	0.172466
rs28407527	SLC34A3	9	139247906	8	1.5557
rs28407527	SLC34A3	9	139247906	5	0.561169
rs28407527	SLC34A3	9	139247906	4	0.508954
rs4073074	LOC401565	9	139259005	2	2.242397
rs28740782	RP13-122B23.3	9	139287806	7	7.239234
rs28740782	RP13-122B23.3	9	139287806	4	1.038819
rs28740782	RP13-122B23.3	9	139287806	2	2.878873
rs28740782	RP13-122B23.3	9	139287806	5	0.136854
rs28740782	RP13-122B23.3	9	139287806	3	0.162007
rs13301565	C9orf167	9	139296861	1	4.315201
rs13301565	C9orf167	9	139296861	7	0.158478
SNP9-139363665	FLJ20433	9	139363665	1	0.47519
SNP9-139363665	FLJ20433	9	139363665	3	0.753808
SNP9-139366964	FLJ20433	9	139366964	7	0.897062
SNP9-139366964	FLJ20433	9	139366964	2	1.53683
SNP9-139366964	FLJ20433	9	139366964	1	0.861049
rs7389423	FLJ20433	9	139370639	7	0.266728
rs7389423	FLJ20433	9	139370639	1	0.474401
rs13291830	FLJ20433	9	139397647	5	1.724753
rs34155071	NOXA1	9	139447484	5	1.928356
rs13297080	ENTPD8	9	139450797	4	3.919765
rs6606582	ENTPD8	9	139452299	4	0.599734
rs4962237	PNPLA7	9	139478402	3	0.48808
rs11137410	PNPLA7	9	139534231	3	0.904038
rs1045777	C9orf37	9	139632376	6	5.457963
rs1045777	C9orf37	9	139632376	3	0.280796
rs1045777	C9orf37	9	139632376	1	5.742015
rs1129767	EHMT1	9	139758237	5	5.308984
rs1129767	EHMT1	9	139758237	2	2.699837
rs45450992	EHMT1	9	139768563	4	0.651272
SNP9-140037600	CACNA1B	9	140037600	3	0.501379
SNP9-140037600	CACNA1B	9	140037600	1	0.254224
SNP9-140037600	CACNA1B	9	140037600	5	0.139054
rs2229948	CACNA1B	9	140128697	7	3.077065
rs2229949	CACNA1B	9	140136352	3	0.119157
rs1017361	ZMYND11	10	284953	4	0.661269
rs10904083	DIP2C	10	419977	5	0.044105
rs10904083	DIP2C	10	419977	1	4.405673
rs12772979	GTPBP4	10	1024412	6	1.745727
rs13377037	IDI2	10	1058639	3	1.518255
rs13377037	IDI2	10	1058639	1	0.636288
rs11250238	C10orf110	10	1079616	1	6.177379
rs11250238	C10orf110	10	1079616	7	2.749445
rs11818314	LOC727843	10	1138157	3	0.324457

rs11818314	LOC727843	10	1138157	1	3.010669
rs12570526	C10orf139	10	1195850	1	1.995803
rs12570526	C10orf139	10	1195850	2	1.860682
rs1046914	ADARB2	10	1218206	4	4.025776
rs904957	ADARB2	10	1218296	4	3.364728
rs3750679	ADARB2	10	1218623	4	2.136758
rs11250317	ADARB2	10	1218905	3	1.159034
rs904960	ADARB2	10	1219009	4	0.63402
rs2271275	ADARB2	10	1220968	4	2.666729
rs12411758	LOC728209	10	2254135	7	3.266514
rs11542777	PFKP	10	3133667	3	0.050949
rs11251721	PFKP	10	3140898	5	0.294191
rs11251721	PFKP	10	3140898	8	2.594927
rs1132173	PFKP	10	3140973	6	0.015786
rs1132173	PFKP	10	3140973	7	0.87656
rs1132173	PFKP	10	3140973	5	0.479
rs1132173	PFKP	10	3140973	2	1.974654
rs2306302	PFKP	10	3144461	1	1.905444
rs1052929	PFKP	10	3168814	1	0.905595
rs1052929	PFKP	10	3168814	2	8.686784
rs1052929	PFKP	10	3168814	8	6.14182
rs7673	PFKP	10	3168905	2	6.223527
rs7673	PFKP	10	3168905	4	5.130594
rs9129	PITRM1	10	3169968	2	7.511189
rs9129	PITRM1	10	3169968	1	0.372872
rs9129	PITRM1	10	3169968	8	7.160145
rs6901	PITRM1	10	3170227	2	2.929963
rs6901	PITRM1	10	3170227	7	6.788783
rs35779348	PITRM1	10	3170288	3	0.121844
rs10175	PITRM1	10	3170298	7	2.825575
rs10175	PITRM1	10	3170298	6	4.039044
rs10175	PITRM1	10	3170298	4	3.130985
rs2388556	PITRM1	10	3181823	2	1.461651
rs2388556	PITRM1	10	3181823	1	1.551444
rs4242746	PITRM1	10	3192065	7	0.87325
rs9423502	PITRM1	10	3197705	6	0.591593
rs1127047	PITRM1	10	3198512	6	6.850431
rs12249207	LOC338588	10	4688360	7	0.011165
rs12249207	LOC338588	10	4688360	8	0.583514
rs12249207	LOC338588	10	4688360	5	0.012058
SNP10-4869748	AKR1CL2	10	4869748	5	5.797677
rs12573669	AKR1CL2	10	4874642	6	0.486565
rs17133779	AKR1CL2	10	4879974	6	3.178512
rs7092190	tAKR	10	4908182	1	6.240312
rs7092190	tAKR	10	4908182	5	1.579926
rs10904368	tAKR	10	4922085	5	2.01441
rs10904368	tAKR	10	4922085	1	5.496139

rs12529	AKR1C3	10	5126651	5	9.503338
rs12529	AKR1C3	10	5126651	4	0.368048
rs12529	AKR1C3	10	5126651	6	6.556791
rs7741	AKR1C3	10	5128607	3	0.592864
rs7741	AKR1C3	10	5128607	6	0.805977
rs7741	AKR1C3	10	5128607	7	0.786457
rs7741	AKR1C3	10	5128607	8	0.545393
rs2210680	AKR1CL1	10	5186993	1	1.694822
rs1781931	AKR1CL1	10	5187881	5	2.469525
rs11253021	AKR1CL1	10	5193864	3	0.341196
rs17134592	AKR1C4	10	5250682	3	0.017033
rs17134592	AKR1C4	10	5250682	4	0.369768
rs17307359	AKR1C4	10	5250859	4	0.007412
rs3255	TUBAL3	10	5425181	3	0.187334
rs3255	TUBAL3	10	5425181	6	3.32221
rs3255	TUBAL3	10	5425181	2	4.299958
rs3255	TUBAL3	10	5425181	8	0.185704
rs7097775	TUBAL3	10	5425918	2	5.861044
rs7097775	TUBAL3	10	5425918	4	2.118923
rs7097775	TUBAL3	10	5425918	8	0.886366
rs7097775	TUBAL3	10	5425918	1	1.659163
rs7097775	TUBAL3	10	5425918	3	0.423036
rs7477466	TUBAL3	10	5436794	1	0.587939
rs7477466	TUBAL3	10	5436794	3	0.399215
rs7477466	TUBAL3	10	5436794	6	1.227426
rs7477466	TUBAL3	10	5436794	8	0.512111
rs10904517	CALML5	10	5531183	2	0.888222
rs10904517	CALML5	10	5531183	8	6.488138
rs10904517	CALML5	10	5531183	1	0.467462
rs10904517	CALML5	10	5531183	3	0.133474
rs2797486	C10orf18	10	5821975	3	0.059969
rs2797486	C10orf18	10	5821975	4	5.208173
rs2797486	C10orf18	10	5821975	6	0.817551
rs2797486	C10orf18	10	5821975	7	7.084282
SNP10-5824157	C10orf18	10	5824157	5	0.758762
SNP10-5824157	C10orf18	10	5824157	4	7.014564
SNP10-5824157	C10orf18	10	5824157	8	7.389222
SNP10-5843374	C10orf18	10	5843374	7	0.397582
rs1046393	C10orf18	10	5845850	4	6.7422
rs1046393	C10orf18	10	5845850	5	5.70491
rs11255072	C10orf18	10	5846254	2	4.511948
rs1057943	C10orf18	10	5846260	6	0.366826
rs1057943	C10orf18	10	5846260	3	0.743643
rs2296139	IL15RA	10	6048178	8	3.595693
rs3829148	PFKFB3	10	6315790	2	2.137277
rs3829148	PFKFB3	10	6315790	4	0.934663
rs3829148	PFKFB3	10	6315790	5	0.610463

rs3829148	PFKFB3	10	6315790	6	0.594526
rs3829148	PFKFB3	10	6315790	1	0.651714
rs582052	PRKCQ	10	6509161	7	0.563421
rs582052	PRKCQ	10	6509161	3	0.118347
rs2453	PRKCQ	10	6509679	3	0.035049
rs2453	PRKCQ	10	6509679	8	0.079427
rs2453	PRKCQ	10	6509679	2	5.176111
rs11258747	PRKCQ	10	6512897	7	0.423687
rs11258747	PRKCQ	10	6512897	2	4.006057
rs2248474	SFMBT2	10	7325638	5	0.941447
rs2248474	SFMBT2	10	7325638	7	1.360052
rs2248474	SFMBT2	10	7325638	6	2.18484
rs1244229	TAF3	10	8047566	5	7.008794
rs1244229	TAF3	10	8047566	8	1.754742
rs1244185	FLJ45983	10	8132580	4	2.116922
rs1244185	FLJ45983	10	8132580	5	0.344218
rs1244185	FLJ45983	10	8132580	3	0.568228
rs1244185	FLJ45983	10	8132580	6	4.071643
rs501764	FLJ45983	10	8133040	4	0.03947
rs501764	FLJ45983	10	8133040	3	0.911318
rs501764	FLJ45983	10	8133040	5	1.353442
rs485411	FLJ45983	10	8133191	5	0.05444
rs485411	FLJ45983	10	8133191	6	0.356856
rs485411	FLJ45983	10	8133191	3	0.4467
rs485411	FLJ45983	10	8133191	4	0.606702
rs2275806	FLJ45983	10	8135346	6	0.372463
rs2275806	FLJ45983	10	8135346	8	0.32571
rs2275806	FLJ45983	10	8135346	4	0.330434
rs2275806	FLJ45983	10	8135346	5	0.368735
rs2275806	FLJ45983	10	8135346	3	0.54033
rs2279809	C10orf31	10	11400679	8	0.146501
rs2279809	C10orf31	10	11400679	1	0.487354
rs2279809	C10orf31	10	11400679	2	2.224869
rs2279809	C10orf31	10	11400679	6	1.642789
rs1051765	CUGBP2	10	11417005	6	3.960489
rs1051765	CUGBP2	10	11417005	5	0.314546
rs1051765	CUGBP2	10	11417005	7	5.932539
rs2062988	DHTKD1	10	12183111	3	0.110907
rs2062988	DHTKD1	10	12183111	5	6.917859
rs2062988	DHTKD1	10	12183111	6	0.74916
rs10466280	SEC61A2	10	12231939	4	0.625223
rs10466280	SEC61A2	10	12231939	1	0.685634
rs10466280	SEC61A2	10	12231939	8	4.07831
rs3232	CCDC3	10	12978760	5	0.381335
rs3232	CCDC3	10	12978760	6	0.532452
rs3232	CCDC3	10	12978760	4	0.325824
rs2280076	CCDC3	10	12978967	8	0.387844

rs2280076	CCDC3	10	12978967	4	0.169052
rs10752280	CCDC3	10	12980383	1	1.491019
rs34630110	MCM10	10	13254759	1	0.86876
rs34630110	MCM10	10	13254759	2	2.723591
rs7905784	MCM10	10	13274362	1	0.259558
rs7905784	MCM10	10	13274362	2	1.1738
SNP10-13380242	PHYH	10	13380242	6	6.621987
SNP10-13380242	PHYH	10	13380242	3	0.624989
SNP10-13380242	PHYH	10	13380242	4	0.913502
rs4750356	C10orf30	10	13520795	4	2.036216
rs4750356	C10orf30	10	13520795	1	1.779544
rs4750356	C10orf30	10	13520795	2	0.757794
rs3814659	C10orf30	10	13584870	5	0.527591
rs3814659	C10orf30	10	13584870	1	4.066965
rs3814659	C10orf30	10	13584870	7	0.212566
rs9473	FRMD4A	10	13727177	5	0.908075
rs9473	FRMD4A	10	13727177	7	0.573721
rs9473	FRMD4A	10	13727177	4	0.057758
rs9473	FRMD4A	10	13727177	1	0.5691
rs9473	FRMD4A	10	13727177	2	1.12494
rs3780915	FAM107B	10	14603103	5	1.692044
rs3780915	FAM107B	10	14603103	3	0.103047
rs2278871	ARMETL1	10	14901407	5	0.298254
rs17353856	SUV39H2	10	14981660	3	0.654833
rs4750568	MEIG1	10	15048499	5	2.53749
rs4750568	MEIG1	10	15048499	7	2.633825
rs4750568	MEIG1	10	15048499	4	2.002904
rs4418687	MEIG1	10	15054704	5	0.097227
rs4418687	MEIG1	10	15054704	7	0.528821
rs4418687	MEIG1	10	15054704	4	0.705663
rs5019235	MEIG1	10	15054850	4	0.504249
rs5019235	MEIG1	10	15054850	5	1.00277
rs4750593	ACBD7	10	15157547	5	0.521277
rs4750593	ACBD7	10	15157547	1	5.141597
rs4750593	ACBD7	10	15157547	3	0.062707
rs6602811	C10orf111	10	15177912	6	0.055788
rs7896053	C10orf111	10	15178621	5	1.434447
rs7896053	C10orf111	10	15178621	7	1.89064
rs15772	RPP38	10	15185861	7	6.865941
rs15772	RPP38	10	15185861	6	5.775016
rs15772	RPP38	10	15185861	8	4.775252
rs7922050	PTER	10	16519013	4	0.989059
rs7922050	PTER	10	16519013	5	1.120977
rs7922050	PTER	10	16519013	3	0.851216
rs7922050	PTER	10	16519013	1	3.695155
rs7922050	PTER	10	16519013	7	0.231338
rs3087659	PTER	10	16594729	4	0.667747

rs3087659	PTER	10	16594729	3	1.047959
rs3087659	PTER	10	16594729	8	0.763356
rs3087659	PTER	10	16594729	5	0.557285
rs1055340	PTER	10	16595534	3	1.310793
rs1055340	PTER	10	16595534	5	2.312475
rs1055340	PTER	10	16595534	4	2.926813
rs1055340	PTER	10	16595534	8	2.29222
rs1801232	CUBN	10	16910918	4	0.350749
SNP10-1695900З	CUBN	10	16959003	5	0.314227
SNP10-1695900З	CUBN	10	16959003	8	4.732297
rs1801231	CUBN	10	17064509	7	0.572327
rs1801231	CUBN	10	17064509	4	0.991663
rs1873469	CUBN	10	17127162	5	0.781407
rs1801225	CUBN	10	17153569	5	0.222256
rs1801225	CUBN	10	17153569	4	0.358288
rs1801225	CUBN	10	17153569	8	3.918638
rs1801225	CUBN	10	17153569	1	1.078763
rs7905349	CUBN	10	17166389	5	5.319118
rs1801222	CUBN	10	17196157	1	5.268238
rs1801222	CUBN	10	17196157	5	4.892531
rs1801222	CUBN	10	17196157	7	1.586692
rs11254401	TRDMT1	10	17228824	1	6.228011
rs11254401	TRDMT1	10	17228824	7	0.263036
rs11254408	TRDMT1	10	17239784	1	4.702236
rs11254408	TRDMT1	10	17239784	7	2.471765
rs11254413	TRDMT1	10	17244193	5	0.067683
rs11254413	TRDMT1	10	17244193	2	6.409945
rs7096233	TRDMT1	10	17250887	1	4.103822
rs7096233	TRDMT1	10	17250887	7	0.124501
rs1061162	STAM	10	17797302	1	5.04356
rs2461153	FAM23B	10	18129628	3	0.029823
rs11012350	SLC39A12	10	18371998	5	1.97749
rs2228645	CACNB2	10	18868377	7	0.009603
SNP10-1886864	CACNB2	10	18868641	2	4.66233
rs12359433	ARL5B	10	19004103	6	6.988505
rs11593491	LOC645120	10	19065203	5	0.144027
rs11593491	LOC645120	10	19065203	3	1.61659
rs7096907	LOC645120	10	19066847	6	1.555378
rs7096907	LOC645120	10	19066847	1	1.304937
rs12771333	C10orf112	10	19716567	5	2.278765
rs12771333	C10orf112	10	19716567	1	1.446326
rs2151234	C10orf112	10	19818062	1	0.235789
rs2151234	C10orf112	10	19818062	5	0.192829
rs2151234	C10orf112	10	19818062	2	0.913033
rs2151234	C10orf112	10	19818062	3	0.127847
rs2151234	C10orf112	10	19818062	7	1.0242
rs16918863	C10orf112	10	19820469	1	3.210076

rs10827499	C10orf112	10	19820549	3	0.370532
rs10827499	C10orf112	10	19820549	2	1.655242
rs10827628	C10orf112	10	19924270	1	3.205379
rs10827628	C10orf112	10	19924270	3	0.096137
rs10827628	C10orf112	10	19924270	6	3.28019
rs2778979	PLXDC2	10	20574339	8	9.691453
rs7074847	SPAG6	10	22715863	3	1.022128
rs1539322	SPAG6	10	22745781	7	2.632581
rs4747442	LOC643475	10	22884105	7	0.285762
rs4747442	LOC643475	10	22884105	5	0.438273
rs4747442	LOC643475	10	22884105	4	0.141663
rs4747443	LOC643475	10	22884332	5	0.412827
rs4748813	LOC643475	10	22886741	4	0.204033
rs4748814	LOC643475	10	22887204	4	0.067274
rs4748814	LOC643475	10	22887204	2	4.467864
rs4748814	LOC643475	10	22887204	7	0.858511
rs7094131	LOC643475	10	22887445	7	0.623215
rs7094131	LOC643475	10	22887445	4	0.532682
rs4326695	C10orf67	10	23645936	1	0.419505
rs2297329	KIAA1217	10	24853288	3	0.356737
rs2307047	C10orf63	10	25311276	3	0.899395
rs12257119	MYO3A	10	26326165	2	0.025883
rs12257119	MYO3A	10	26326165	1	1.066508
rs3824699	MYO3A	10	26395998	3	0.817184
rs3758449	MYO3A	10	26486318	8	0.907218
rs3758449	MYO3A	10	26486318	3	0.120443
rs3740232	MYO3A	10	26502796	5	0.897995
rs3740232	MYO3A	10	26502796	3	0.362895
rs1999240	MYO3A	10	26503136	7	0.615825
rs1999240	MYO3A	10	26503136	3	0.558666
rs1999240	MYO3A	10	26503136	1	2.293791
rs10829163	ANKRD26	10	27357846	7	0.733916
rs2297145	ANKRD26	10	27421355	7	1.803533
rs3737056	ANKRD26	10	27429320	7	1.093755
rs10764690	ACBD5	10	27548764	4	0.152115
rs10764690	ACBD5	10	27548764	5	0.252094
rs10764690	ACBD5	10	27548764	8	2.94573
rs2505323	PTCHD3	10	27727231	8	3.967471
rs2505323	PTCHD3	10	27727231	4	2.59624
rs2505323	PTCHD3	10	27727231	5	2.57249
rs1638630	PTCHD3	10	27727781	4	3.478834
rs2505327	PTCHD3	10	27727971	6	3.843355
rs2505327	PTCHD3	10	27727971	3	0.541254
rs2505327	PTCHD3	10	27727971	8	2.431197
rs2505327	PTCHD3	10	27727971	4	3.925542
rs2484173	PTCHD3	10	27728000	4	3.892377
rs2484173	PTCHD3	10	27728000	3	1.959534

rs2484173	PTCHD3	10	27728000	8	5.527506
rs2484173	PTCHD3	10	27728000	6	4.635059
rs2484173	PTCHD3	10	27728000	2	4.623122
rs2429485	PTCHD3	10	27728115	5	1.484926
rs2429485	PTCHD3	10	27728115	4	4.259051
rs2429485	PTCHD3	10	27728115	3	0.470554
rs2429485	PTCHD3	10	27728115	6	4.654571
rs2429485	PTCHD3	10	27728115	2	2.786904
rs6482625	PTCHD3	10	27742175	8	3.701364
rs6482625	PTCHD3	10	27742175	3	5.002977
rs6482625	PTCHD3	10	27742175	6	1.591055
rs6482626	PTCHD3	10	27742731	6	2.353918
rs6482626	PTCHD3	10	27742731	3	0.521566
rs11015754	PTCHD3	10	27742750	8	1.919972
rs2492909	MKX	10	28003252	7	0.244737
rs2492909	MKX	10	28003252	3	0.194794
rs2172085	MKX	10	28003678	3	0.046875
rs2172085	MKX	10	28003678	4	0.89418
rs2172085	MKX	10	28003678	7	0.360815
rs2130809	MKX	10	28003898	3	0.442525
rs2130809	MKX	10	28003898	4	1.338984
rs2253230	MKX	10	28004476	4	1.210611
rs2253230	MKX	10	28004476	3	1.136125
rs2253230	MKX	10	28004476	1	0.23425
rs11006957	MPP7	10	28583741	6	1.004123
rs11006957	MPP7	10	28583741	2	1.172126
rs11006957	MPP7	10	28583741	7	0.091942
rs1063205	KIAA1462	10	30344924	2	1.890667
rs1063205	KIAA1462	10	30344924	7	0.3562
rs3739998	KIAA1462	10	30356078	7	0.519368
rs3739998	KIAA1462	10	30356078	3	0.590586
rs2185724	KIAA1462	10	30356214	7	0.486924
rs3739996	KIAA1462	10	30356878	7	0.069445
rs3739996	KIAA1462	10	30356878	5	0.617414
rs9337951	KIAA1462	10	30357079	5	1.305509
rs9337951	KIAA1462	10	30357079	3	0.870664
rs9337951	KIAA1462	10	30357079	8	3.332934
rs9337951	KIAA1462	10	30357079	7	3.988267
rs9337951	KIAA1462	10	30357079	2	2.367506
rs7920682	KIAA1462	10	30357832	5	0.41018
rs7920682	KIAA1462	10	30357832	7	0.275986
rs2478835	KIAA1462	10	30357955	3	0.559285
rs2478835	KIAA1462	10	30357955	5	0.29764
rs2478835	KIAA1462	10	30357955	2	2.001192
rs2478835	KIAA1462	10	30357955	7	0.991477
rs8177036	MAP3K8	10	30790650	4	3.393874
rs8177036	MAP3K8	10	30790650	5	0.036451

rs2994626	ZNF438	10	31239356	1	5.927479
rs2994626	ZNF438	10	31239356	3	1.160566
SNP10-32620211	EPC1	10	32620211	4	6.83033
rs11009107	C10orf68	10	33163780	5	0.142367
rs11009107	C10orf68	10	33163780	3	1.870918
rs11009107	C10orf68	10	33163780	4	2.94578
rs11009107	C10orf68	10	33163780	7	0.103017
rs2504011	C10orf68	10	33177557	3	0.080552
rs2504011	C10orf68	10	33177557	7	0.475775
rs2504011	C10orf68	10	33177557	5	0.136665
rs1316757	ITGB1	10	33237203	3	0.810735
rs7079624	ITGB1	10	33257116	3	0.00595
rs13324	NRP1	10	33507656	4	5.976103
rs13324	NRP1	10	33507656	3	0.139463
rs2506144	NRP1	10	33508462	4	6.642202
rs2228638	NRP1	10	33515288	6	0.744325
rs2228638	NRP1	10	33515288	3	0.330521
rs2228638	NRP1	10	33515288	4	0.999671
rs3750733	NRP1	10	33663257	4	0.160703
rs17499247	CREM	10	35466761	2	0.218505
rs17499247	CREM	10	35466761	5	0.767421
rs17499247	CREM	10	35466761	8	0.268209
rs17499247	CREM	10	35466761	1	0.353027
rs17499247	CREM	10	35466761	3	0.591755
rs1209750	ANKRD30A	10	37528695	1	3.077947
rs7091441	hCG_25653	10	37675828	1	2.679891
rs2295872	ZNF248	10	38186224	8	3.566149
rs2295872	ZNF248	10	38186224	1	6.05894
rs1148295	ZNF248	10	38186265	8	1.847367
rs1148295	ZNF248	10	38186265	7	4.407538
rs1148295	ZNF248	10	38186265	3	0.334285
rs10508862	ZNF33A	10	38385471	7	3.581092
rs10508862	ZNF33A	10	38385471	3	0.37602
rs10508862	ZNF33A	10	38385471	8	0.412998
rs2142349	ZNF33A	10	38386098	4	5.596322
rs2472161	ZNF33A	10	38386162	8	0.128412
rs2472165	ZNF33A	10	38387812	8	4.530655
rs2472165	ZNF33A	10	38387812	3	0.143172
rs2472165	ZNF33A	10	38387812	7	0.049732
rs2472175	LOC340947	10	38422907	4	3.027816
rs2472175	LOC340947	10	38422907	7	3.607618
rs2472175	LOC340947	10	38422907	1	3.276801
rs2474570	LOC340947	10	38423763	3	0.524011
rs2505860	CCNYL2	10	42227207	1	1.988833
rs2505861	CCNYL2	10	42227266	1	2.578002
rs12261168	ZNF33B	10	42404995	3	0.644703
rs12261168	ZNF33B	10	42404995	7	4.513384

rs12261168	ZNF33B	10	42404995	4	4.293138
rs1799939	RET	10	42930125	7	0.489257
rs1800861	RET	10	42933849	3	0.227412
rs1800861	RET	10	42933849	5	0.115175
rs2075912	RET	10	42942223	3	0.388078
rs2075912	RET	10	42942223	5	0.558582
rs17028	RET	10	42943818	3	0.37321
rs17028	RET	10	42943818	5	0.759544
rs17028	RET	10	42943818	1	2.768722
SNP10-42944111	RET	10	42944111	7	1.088881
SNP10-42944111	RET	10	42944111	1	0.128171
rs2435355	RET	10	42944839	3	1.777448
rs2435355	RET	10	42944839	1	1.894762
rs1254967	RASGEF1A	10	43010397	5	0.35445
rs1254967	RASGEF1A	10	43010397	3	0.039662
rs10793442	ZNF239	10	43371825	7	1.122762
rs10793442	ZNF239	10	43371825	5	0.861794
rs10793442	ZNF239	10	43371825	2	5.321347
rs2999277	ZNF239	10	43371907	4	0.360713
rs2999277	ZNF239	10	43371907	8	7.131142
rs2999277	ZNF239	10	43371907	1	6.454421
rs2999277	ZNF239	10	43371907	3	0.478236
rs2230660	ZNF239	10	43373019	1	5.063129
rs2230660	ZNF239	10	43373019	4	0.422769
rs2393938	ZNF239	10	43383818	5	1.207565
rs2393938	ZNF239	10	43383818	7	1.806146
rs1998748	HNRPA3P1	10	43605165	8	1.511497
rs1998748	HNRPA3P1	10	43605165	3	1.611523
rs266093	CXCL12	10	44186214	3	1.54444
rs17886066	CXCL12	10	44194001	4	0.882751
rs12773699	RASSF4	10	44775233	8	4.05802
rs870957	RASSF4	10	44798098	3	0.204717
rs11239305	RASSF4	10	44809230	3	0.603499
rs11239305	RASSF4	10	44809230	8	5.075356
rs12269028	C10orf25	10	44816126	2	5.724641
rs12269028	C10orf25	10	44816126	6	5.005913
SNP10-44816132	C10orf25	10	44816132	3	0.292799
SNP10-44816132	C10orf25	10	44816132	8	4.037671
rs3740092	ZNF22	10	44819278	3	0.159826
rs1048156	PPYR1	10	46507377	7	0.223384
rs1048156	PPYR1	10	46507377	1	1.872668
rs2999345	SLC9A3P4	10	47486220	3	0.306443
rs3739968	ASAH2C	10	47523998	8	4.101578
rs3739968	ASAH2C	10	47523998	7	4.19184
rs3739968	ASAH2C	10	47523998	6	2.641218
rs3739968	ASAH2C	10	47523998	2	5.021584
rs11204210	ZNF488	10	47990601	6	0.568209

rs11204210	ZNF488	10	47990601	5	0.000295
rs11204210	ZNF488	10	47990601	1	0.376341
rs11204210	ZNF488	10	47990601	4	0.80891
rs11204210	ZNF488	10	47990601	2	1.27012
rs11204210	ZNF488	10	47990601	7	0.688321
SNP10-47991283	ZNF488	10	47991283	4	0.117262
SNP10-47991283	ZNF488	10	47991283	7	0.211993
rs17095800	ZNF488	10	47992263	2	4.314974
rs4080665	ARHGAP22	10	49357806	5	1.161883
rs4080665	ARHGAP22	10	49357806	2	3.231394
rs7072606	C10orf64	10	49603980	5	0.44162
rs4838645	LOC642343	10	49614255	8	4.939486
rs17773851	C10orf73	10	50010310	3	1.460982
SNP10-50205192	C10orf71	10	50205192	3	1.291237
SNP10-50205192	C10orf71	10	50205192	2	1.289349
SNP10-50205192	C10orf71	10	50205192	6	1.415791
SNP10-50417077	ERCC6	10	50417077	5	4.116185
SNP10-50417077	ERCC6	10	50417077	2	5.737489
rs3729496	CHAT	10	50491197	5	0.400966
rs3729496	CHAT	10	50491197	7	0.960067
rs8178992	CHAT	10	50533153	4	1.947925
rs8178992	CHAT	10	50533153	3	2.424871
rs6816	OGDHL	10	50612769	4	0.228213
rs6816	OGDHL	10	50612769	5	0.496231
rs13736	OGDHL	10	50613074	4	0.386202
rs13736	OGDHL	10	50613074	5	1.107613
rs1258256	OGDHL	10	50613192	5	0.514109
rs11101224	OGDHL	10	50620982	7	0.123264
rs11101224	OGDHL	10	50620982	3	0.257979
rs1258184	OGDHL	10	50630215	6	2.800584
rs1258184	OGDHL	10	50630215	4	0.005424
rs1258184	OGDHL	10	50630215	5	1.555467
rs1258184	OGDHL	10	50630215	2	1.066918
rs11006229	SGMS1	10	52020012	3	0.418475
rs11006229	SGMS1	10	52020012	5	2.195436
rs11006229	SGMS1	10	52020012	6	1.111605
rs4497350	LOC729047	10	52169886	8	1.151325
rs1881597	PRKG1	10	53723823	7	1.841671
rs1881597	PRKG1	10	53723823	6	0.042473
rs1896368	LOC440558	10	53738910	5	0.598565
rs1896368	LOC440558	10	53738910	7	0.603336
rs930507	MBL2	10	54198272	3	0.034565
rs11004439	PCDH15	10	56093974	3	1.555155
rs11005328	ZWINT	10	57790994	1	2.842731
rs1049432	TFAM	10	59825126	3	0.461992
rs1049432	TFAM	10	59825126	5	4.992768
rs1049432	TFAM	10	59825126	4	6.610035

rs1049432	TFAM	10	59825126	6	1.359724
rs4948550	BICC1	10	60258559	1	2.283407
rs4948550	BICC1	10	60258559	5	0.189642
rs2452505	PHYHIPL	10	60675250	5	0.377992
rs17776046	SLC16A9	10	61082207	6	1.806577
rs17776046	SLC16A9	10	61082207	5	0.917565
rs3763747	SLC16A9	10	61082341	5	0.728773
rs2242206	SLC16A9	10	61084017	7	0.694586
rs2242206	SLC16A9	10	61084017	5	2.506656
rs2242206	SLC16A9	10	61084017	1	2.999278
rs12356776	ANK3	10	61489562	1	3.4366
SNP10-6150165	ANK3	10	61501657	6	1.206512
rs17208576	ANK3	10	61504579	4	5.648045
rs3750800	ANK3	10	61538722	8	1.497731
rs10994754	TMEM26	10	62836779	3	0.411702
rs2176929	TMEM26	10	62837138	5	2.561956
rs2280449	TMEM26	10	62839957	5	0.917676
rs1992625	C10orf107	10	63190704	3	0.931765
rs1992625	C10orf107	10	63190704	6	0.054615
SNP10-6364692	RTKN2	10	63646920	7	1.40049
SNP10-6364692	RTKN2	10	63646920	4	0.529774
rs11819488	ZNF365	10	63909715	1	5.912771
rs729739	ZNF365	10	64100308	3	0.237521
rs10995311	ADO	10	64234940	4	0.70702
rs10995311	ADO	10	64234940	2	3.673858
rs10995311	ADO	10	64234940	8	8.425517
rs1509964	ADO	10	64237370	5	0.808643
rs1227236	ANXA2P3	10	66256311	5	0.459572
rs2273771	DNAJC12	10	69267792	4	5.921671
rs2273771	DNAJC12	10	69267792	3	0.037635
rs2273771	DNAJC12	10	69267792	7	0.309308
rs2273771	DNAJC12	10	69267792	8	4.417839
rs2234975	SIRT1	10	69348084	3	0.601538
rs2673794	MYPN	10	69596103	1	4.722919
rs2673794	MYPN	10	69596103	7	4.438302
rs2673794	MYPN	10	69596103	6	4.4561
rs731063	PBLD	10	69713196	8	0.15783
rs731063	PBLD	10	69713196	1	0.094174
rs731063	PBLD	10	69713196	2	0.989796
rs4142048	PBLD	10	69714037	8	6.862468
rs4142048	PBLD	10	69714037	1	0.694748
rs4142048	PBLD	10	69714037	2	0.802378
rs1162756	HNRPH3	10	69772942	6	0.075148
rs1801041	DNA2	10	69844713	7	0.275885
rs1801041	DNA2	10	69844713	6	0.660312
rs1801041	DNA2	10	69844713	4	0.343727
rs1535539	DNA2	10	69901707	1	2.677509

rs1535539	DNA2	10	69901707	6	0.701793
rs10998219	SLC25A16	10	69912911	6	0.430305
rs10823229	CXXC6	10	70002586	7	0.659487
rs10823229	CXXC6	10	70002586	2	3.435234
rs10823229	CXXC6	10	70002586	1	0.007858
rs10823229	CXXC6	10	70002586	3	0.647215
rs10823229	CXXC6	10	70002586	4	4.130752
rs10509304	STOX1	10	70315113	8	2.104523
rs10509306	STOX1	10	70315703	1	5.248794
rs5030895	DDX50	10	70366719	3	0.093615
rs5030895	DDX50	10	70366719	2	0.515355
rs5030900	DDX50	10	70370950	3	0.147863
rs5030900	DDX50	10	70370950	1	0.054035
rs10823277	LOC389976	10	70453081	8	2.947487
rs12437	SRGN	10	70534071	3	0.876996
rs12437	SRGN	10	70534071	4	0.500328
rs2292192	SUPV3L1	10	70610197	4	2.015
rs10823320	HKDC1	10	70657066	3	0.16885
rs874556	HKDC1	10	70662670	7	0.159735
rs874556	HKDC1	10	70662670	5	0.045386
rs874556	HKDC1	10	70662670	6	2.068717
rs2611	HKDC1	10	70697237	7	1.870702
rs2611	HKDC1	10	70697237	4	0.241824
rs2611	HKDC1	10	70697237	2	0.209977
rs2611	HKDC1	10	70697237	3	0.557912
rs2611	HKDC1	10	70697237	1	0.062078
rs1133189	HK1	10	70773603	3	0.083009
rs1133189	HK1	10	70773603	7	0.268593
rs1133189	HK1	10	70773603	5	0.073621
rs12020	C10orf35	10	71062563	1	0.334684
rs12020	C10orf35	10	71062563	2	0.832223
rs12020	C10orf35	10	71062563	4	0.105981
rs1052152	C10orf35	10	71062698	4	6.841353
rs1052152	C10orf35	10	71062698	2	0.096475
rs1052152	C10orf35	10	71062698	1	0.428124
rs2395272	COL13A1	10	71310261	3	0.829188
rs2395272	COL13A1	10	71310261	6	0.377125
rs2395272	COL13A1	10	71310261	4	0.092785
rs1061966	COL13A1	10	71388730	1	4.91065
rs2394655	AIFM2	10	71542141	3	0.13481
rs10823484	AIFM2	10	71542578	3	0.876608
rs7893137	AIFM2	10	71542953	3	0.395465
rs7908957	AIFM2	10	71543587	3	0.694397
rs10999144	AIFM2	10	71543925	1	4.351006
SNP10-71550864	AIFM2	10	71550864	7	0.101268
rs3750774	TYSND1	10	71575575	4	0.505177
rs3750774	TYSND1	10	71575575	8	0.451025

rs3750774	TYSND1	10	71575575	5	0.393875
rs3750774	TYSND1	10	71575575	1	0.565998
rs10999250	LRRC20	10	71731005	4	1.211356
rs1904589	NODAL	10	71865445	7	0.10077
rs1904589	NODAL	10	71865445	8	0.270784
rs1904589	NODAL	10	71865445	5	0.818041
rs1904589	NODAL	10	71865445	3	0.097805
rs1904589	NODAL	10	71865445	4	0.13508
rs1904589	NODAL	10	71865445	1	0.654986
rs2275059	KIAA1274	10	71959707	3	0.801958
rs2275060	KIAA1274	10	71959784	8	0.198245
rs3740447	KIAA1274	10	71977107	3	1.024675
SNP10-7199640	KIAA1274	10	71996406	6	0.590981
rs3740444	KIAA1274	10	71996689	1	1.262523
rs3740444	KIAA1274	10	71996689	5	0.208514
rs3740442	KIAA1274	10	71996948	6	1.444687
rs3740442	KIAA1274	10	71996948	7	0.398562
rs1889490	PRF1	10	72027719	3	0.214053
rs1889490	PRF1	10	72027719	5	0.440998
rs1889490	PRF1	10	72027719	4	0.606418
rs10823607	ADAMTS14	10	72170769	2	0.171917
rs2587475	ADAMTS14	10	72183688	7	0.407555
rs2587475	ADAMTS14	10	72183688	5	1.446157
rs2587475	ADAMTS14	10	72183688	1	0.295105
rs10999516	ADAMTS14	10	72187836	5	0.48243
rs827249	SGPL1	10	72299573	7	4.730491
rs1060184	UNC5B	10	72729364	6	0.073513
rs1060184	UNC5B	10	72729364	5	0.544508
rs1060184	UNC5B	10	72729364	3	0.302853
rs1060184	UNC5B	10	72729364	8	8.36786
rs1084004	SLC29A3	10	72791951	8	5.870646
rs2275579	SLC29A3	10	72793082	4	1.618816
rs3802720	CDH23	10	72940912	1	4.044562
rs3802720	CDH23	10	72940912	3	1.24071
rs10999947	CDH23	10	73104912	1	0.578513
rs3752752	CDH23	10	73125207	5	0.82808
rs3752752	CDH23	10	73125207	3	0.0513
rs3752752	CDH23	10	73125207	1	1.743241
rs3752751	CDH23	10	73125279	3	0.506617
SNP10-7316031	CDH23	10	73160316	1	5.27187
SNP10-7316031	CDH23	10	73160316	5	4.0528
rs1227065	CDH23	10	73162085	1	1.292256
rs1227065	CDH23	10	73162085	3	1.230503
rs1227051	CDH23	10	73171562	1	0.01291
rs1227051	CDH23	10	73171562	2	0.823896
rs1227051	CDH23	10	73171562	3	0.922706
rs748113	C10orf54	10	73178797	5	0.048908

rs748113	C10orf54	10	73178797	2	0.761684
rs748113	C10orf54	10	73178797	3	0.631123
rs748113	C10orf54	10	73178797	7	0.408579
rs748113	C10orf54	10	73178797	1	0.923105
rs748505	C10orf54	10	73179772	1	2.866894
rs748505	C10orf54	10	73179772	7	0.195428
SNP10-73203183	C10orf54	10	73203183	3	0.239608
SNP10-73203183	C10orf54	10	73203183	7	0.456568
SNP10-73203183	C10orf54	10	73203183	2	5.249336
SNP10-73203183	C10orf54	10	73203183	1	0.138425
rs17712523	CDH23	10	73207620	3	1.119819
rs17712523	CDH23	10	73207620	7	0.068306
rs3802711	CDH23	10	73214092	8	0.595714
rs11592462	CDH23	10	73220123	1	1.794244
rs11592462	CDH23	10	73220123	2	0.643305
rs11592462	CDH23	10	73220123	4	1.080426
rs10466026	CDH23	10	73220975	5	3.143649
rs10466026	CDH23	10	73220975	4	0.931787
rs10466026	CDH23	10	73220975	8	3.272905
rs2290021	CDH23	10	73244849	4	0.700896
rs2070188	PSAP	10	73280988	5	0.562845
rs3740129	CHST3	10	73437865	5	0.26293
rs3740129	CHST3	10	73437865	6	1.063808
rs3740129	CHST3	10	73437865	7	0.188411
rs3740129	CHST3	10	73437865	1	2.861845
rs1871452	CHST3	10	73442667	1	5.250543
rs1871452	CHST3	10	73442667	8	4.307011
rs1871452	CHST3	10	73442667	7	5.930022
rs1871452	CHST3	10	73442667	6	0.321058
rs1871452	CHST3	10	73442667	5	0.04831
rs7307	SPOCK2	10	73488939	7	3.18348
rs7307	SPOCK2	10	73488939	3	0.291918
rs7307	SPOCK2	10	73488939	6	1.58165
rs7307	SPOCK2	10	73488939	4	2.796139
rs7307	SPOCK2	10	73488939	5	1.333277
rs7307	SPOCK2	10	73488939	1	4.179596
rs1049269	SPOCK2	10	73490007	1	4.218668
rs1049269	SPOCK2	10	73490007	5	0.192863
rs1049269	SPOCK2	10	73490007	8	0.994482
rs1049269	SPOCK2	10	73490007	6	0.070337
rs1049269	SPOCK2	10	73490007	7	0.23319
rs1049269	SPOCK2	10	73490007	4	0.511881
rs896079	SPOCK2	10	73490402	5	1.762897
rs896079	SPOCK2	10	73490402	1	3.469005
rs896079	SPOCK2	10	73490402	7	1.618964
rs896079	SPOCK2	10	73490402	4	3.140226
rs896079	SPOCK2	10	73490402	3	0.074823

rs896079	SPOCK2	10	73490402	6	1.013528
rs15256	SPOCK2	10	73490554	3	1.478395
rs15256	SPOCK2	10	73490554	8	0.29409
rs3180	SPOCK2	10	73490628	7	3.816307
rs3180	SPOCK2	10	73490628	1	5.427369
rs3180	SPOCK2	10	73490628	8	0.103694
rs3180	SPOCK2	10	73490628	5	0.201621
rs3180	SPOCK2	10	73490628	6	0.000298
rs3180	SPOCK2	10	73490628	4	0.933577
rs1049246	SPOCK2	10	73492031	7	0.015274
rs1049246	SPOCK2	10	73492031	1	1.186952
rs1049245	SPOCK2	10	73492249	5	7.735084
rs1049245	SPOCK2	10	73492249	3	0.186414
rs1049245	SPOCK2	10	73492249	7	5.427973
rs1049245	SPOCK2	10	73492249	6	0.844717
rs1049245	SPOCK2	10	73492249	1	4.948673
rs1049245	SPOCK2	10	73492249	4	0.100168
rs11591720	ZMYND17	10	74854450	7	0.735366
rs2227553	C10orf55	10	75339949	1	3.298437
rs2227568	PLAU	10	75343885	4	0.815674
rs2131956	VCL	10	75541741	7	0.548655
rs2131956	VCL	10	75541741	3	0.601499
rs2131956	VCL	10	75541741	2	6.864528
rs703258	VCL	10	75549517	3	0.036858
rs703258	VCL	10	75549517	7	7.090466
rs703258	VCL	10	75549517	2	9.007542
rs3740317	DUSP13	10	76525418	4	0.96705
rs3740317	DUSP13	10	76525418	3	0.741098
rs3740316	DUSP13	10	76525427	1	0.622652
rs3740316	DUSP13	10	76525427	7	1.193072
SNP10-76531686	DUSP13	10	76531686	2	1.366849
SNP10-76531686	DUSP13	10	76531686	7	0.785191
SNP10-76531686	DUSP13	10	76531686	1	0.035062
SNP10-76531686	DUSP13	10	76531686	4	1.856946
SNP10-76531686	DUSP13	10	76531686	3	0.529441
rs7912300	DUSP13	10	76537905	3	0.454143
rs10762666	LOC439985	10	76827495	6	0.065974
rs10762666	LOC439985	10	76827495	4	0.580118
rs11001819	LOC729716	10	77985230	5	0.261931
rs11001819	LOC729716	10	77985230	3	0.741256
rs12761718	LOC399783	10	79162976	1	2.83864
rs12761718	LOC399783	10	79162976	4	0.005835
rs12768275	LOC399783	10	79163930	4	2.304395
rs12768275	LOC399783	10	79163930	1	5.024027
rs2289310	DLG5	10	79240879	6	1.014089
rs1248629	DLG5	10	79254184	1	6.05549
rs1248629	DLG5	10	79254184	3	0.063877

rs2241547	POLR3A	10	79406970	8	0.651786
rs2241547	POLR3A	10	79406970	4	0.47372
rs2241547	POLR3A	10	79406970	5	0.000961
rs1782652	ZMIZ1	10	80744131	3	0.208581
rs1782652	ZMIZ1	10	80744131	2	2.579371
rs1782652	ZMIZ1	10	80744131	8	10.86702
rs1782652	ZMIZ1	10	80744131	7	5.861484
rs3088308	SFTPD	10	81687848	4	7.085073
rs2243639	SFTPD	10	81691702	8	6.331562
rs2243639	SFTPD	10	81691702	7	6.40214
rs2243639	SFTPD	10	81691702	4	1.047174
rs2243639	SFTPD	10	81691702	5	2.070338
rs2243639	SFTPD	10	81691702	3	1.963455
rs2243639	SFTPD	10	81691702	2	2.661547
rs721917	SFTPD	10	81696304	7	0.414784
rs1932574	C10orf57	10	81831672	8	0.241063
rs1932574	C10orf57	10	81831672	4	0.756211
rs2789686	ANXA11	10	81905116	7	0.431879
rs17100686	ANXA11	10	81917039	6	0.453391
rs1832683	MAT1A	10	82022311	3	1.005394
rs1832683	MAT1A	10	82022311	5	0.027863
rs1832683	MAT1A	10	82022311	4	3.546366
rs2993763	MAT1A	10	82023574	3	0.233887
rs2993763	MAT1A	10	82023574	6	0.929663
rs10887711	MAT1A	10	82024822	4	0.016995
rs10887711	MAT1A	10	82024822	3	1.125786
rs10887711	MAT1A	10	82024822	8	1.185562
rs10788546	MAT1A	10	82024834	4	0.812134
rs10788546	MAT1A	10	82024834	3	1.031883
rs1143694	MAT1A	10	82030032	4	0.279548
rs1143694	MAT1A	10	82030032	8	1.275949
rs1143694	MAT1A	10	82030032	3	1.011486
rs1538818	DYDC2	10	82112773	7	0.808207
rs1047952	DYDC2	10	82116788	4	0.515051
rs8735	C10orf58	10	82182693	3	0.422106
rs8735	C10orf58	10	82182693	5	0.468281
rs8735	C10orf58	10	82182693	8	0.972489
rs11185932	LOC727923	10	82285378	7	3.562619
rs11185932	LOC727923	10	82285378	6	0.757314
rs11185932	LOC727923	10	82285378	5	0.463271
rs12257181	SH2D4B	10	82394116	4	0.986182
rs17107451	SH2D4B	10	82394945	3	0.275081
rs11186419	SH2D4B	10	82395063	8	3.265709
rs7087704	SH2D4B	10	82395234	1	0.455229
rs7087704	SH2D4B	10	82395234	5	0.501232
rs7081754	SH2D4B	10	82395520	2	1.372345
rs7081754	SH2D4B	10	82395520	3	0.268133

rs7081754	SH2D4B	10	82395520	5	0.982668
rs7077636	SH2D4B	10	82395567	4	0.840859
rs7096619	LOC728027	10	85052021	1	5.184002
rs7096619	LOC728027	10	85052021	4	3.508707
rs12781048	PCDH21	10	85946248	5	0.514166
rs10749482	PCDH21	10	85962023	5	0.590056
rs10749482	PCDH21	10	85962023	2	2.466268
rs10749482	PCDH21	10	85962023	6	6.9516
rs10749482	PCDH21	10	85962023	3	1.523744
rs3814212	PCDH21	10	85964361	1	1.069922
rs1059341	PCDH21	10	85966945	6	0.058655
rs1059342	PCDH21	10	85967000	5	0.235679
rs1059342	PCDH21	10	85967000	7	4.607345
rs1059342	PCDH21	10	85967000	6	0.133807
rs7074150	LRIT2	10	85970295	3	4.562715
rs4933982	LRIT2	10	85970845	7	1.408124
rs11200925	LRIT2	10	85974303	5	0.428704
rs11200925	LRIT2	10	85974303	7	0.383101
rs10887259	LRIT1	10	85987230	5	0.072186
rs10887259	LRIT1	10	85987230	1	0.661807
rs10887259	LRIT1	10	85987230	3	0.258375
rs10887259	LRIT1	10	85987230	6	0.232671
rs2279227	RGR	10	85994853	7	0.148436
rs2279227	RGR	10	85994853	6	0.7916
rs2279227	RGR	10	85994853	1	2.01672
rs2279227	RGR	10	85994853	3	0.846254
rs1042454	RGR	10	86002693	5	0.675852
rs1042454	RGR	10	86002693	8	0.577593
rs1042454	RGR	10	86002693	1	2.072333
SNP10-8600772	RGR	10	86007720	7	0.086041
rs1050943	KIAA1128	10	86266936	4	0.745849
rs1050943	KIAA1128	10	86266936	2	4.902389
rs2664404	GRID1	10	87350703	5	0.649345
rs7094132	WAPAL	10	88193048	3	0.063322
rs7094132	WAPAL	10	88193048	5	0.556558
rs34191521	OPN4	10	88412008	4	1.010626
rs1079610	OPN4	10	88412096	7	1.305112
rs4934281	MMRN2	10	88692330	1	4.938396
rs10887673	MMRN2	10	88692370	6	1.891003
rs3750823	MMRN2	10	88707134	3	1.659226
rs760113	SNCG	10	88709769	7	0.319545
rs286484	C10orf59	10	90034883	1	4.534078
rs2296545	C10orf59	10	90332817	8	0.208331
rs2296545	C10orf59	10	90332817	3	0.522188
rs2296545	C10orf59	10	90332817	6	0.289699
rs2296545	C10orf59	10	90332817	5	0.327934
rs1214464	LIPK	10	90502286	7	0.488495

rs41284088	LIPN	10	90514315	3	1.44444
rs41284088	LIPN	10	90514315	4	2.087478
rs17382301	CH25H	10	90956373	2	3.562461
rs17382301	CH25H	10	90956373	1	0.959157
rs17382301	CH25H	10	90956373	8	0.100226
rs1131706	LIPA	10	90963656	1	0.856337
rs954439	IFIT2	10	91056440	3	0.012059
rs954439	IFIT2	10	91056440	1	7.174821
rs954439	IFIT2	10	91056440	5	0.610752
rs954439	IFIT2	10	91056440	7	8.619853
rs4934470	IFIT2	10	91057418	8	1.950561
rs4934470	IFIT2	10	91057418	5	0.432132
rs4934470	IFIT2	10	91057418	3	0.155439
rs4934470	IFIT2	10	91057418	4	2.551354
rs4934470	IFIT2	10	91057418	6	0.425219
rs4933504	IFIT2	10	91057575	5	0.486492
rs4933504	IFIT2	10	91057575	3	0.373877
rs4933504	IFIT2	10	91057575	1	1.547329
rs4933504	IFIT2	10	91057575	8	4.781227
rs4933504	IFIT2	10	91057575	7	0.990989
rs3780878	IFIT3	10	91089610	7	0.05251
rs3780878	IFIT3	10	91089610	3	0.251077
rs17119665	IFIT3	10	91089637	8	0.608088
rs10749607	IFIT3	10	91089923	7	2.044706
rs10749607	IFIT3	10	91089923	3	0.214028
rs304504	IFIT1L	10	91127806	3	0.013518
rs303210	IFIT1	10	91152657	8	2.547069
rs2296616	PANK1	10	91342946	6	0.142019
rs2296616	PANK1	10	91342946	3	0.721565
rs2296616	PANK1	10	91342946	5	1.262906
rs2296616	PANK1	10	91342946	7	0.389603
rs2296616	PANK1	10	91342946	8	0.486356
rs2296616	PANK1	10	91342946	2	5.718439
rs2296616	PANK1	10	91342946	4	0.620887
SNP10-91395024	PANK1	10	91395024	5	0.519707
rs3758383	MPHOSPH1	10	91458905	7	4.689477
rs12572012	MPHOSPH1	10	91477629	7	0.465432
rs1062465	MPHOSPH1	10	91487611	3	0.375823
rs1062465	MPHOSPH1	10	91487611	4	0.853605
rs1062465	MPHOSPH1	10	91487611	5	5.751588
rs1062465	MPHOSPH1	10	91487611	1	0.396298
rs1062465	MPHOSPH1	10	91487611	2	3.922943
rs1126480	MPHOSPH1	10	91493640	2	0.217868
rs1126480	MPHOSPH1	10	91493640	3	0.24361
rs1126480	MPHOSPH1	10	91493640	5	0.103108
rs1126480	MPHOSPH1	10	91493640	4	0.033273
rs1126480	MPHOSPH1	10	91493640	1	0.27718

rs3740040	hCG_2024094	10	91587406	1	1.436664
rs3740040	hCG_2024094	10	91587406	5	1.26707
rs10881718	LOC119358	10	91728735	6	1.914124
rs10881718	LOC119358	10	91728735	7	3.676837
rs10881718	LOC119358	10	91728735	3	3.69974
rs1045949	HTR7	10	92490704	3	1.736479
rs1418213	HTR7	10	92491630	6	4.598456
rs3939	ANKRD1	10	92662544	4	1.007637
rs3939	ANKRD1	10	92662544	1	0.002771
rs3939	ANKRD1	10	92662544	5	0.095405
rs10881855	ANKRD1	10	92670909	1	0.884105
rs6583740	LOC643599	10	92710906	4	0.23455
rs6583740	LOC643599	10	92710906	3	0.852908
rs6583740	LOC643599	10	92710906	1	0.984306
rs6583740	LOC643599	10	92710906	7	1.218723
rs1044563	PPP1R3C	10	93380129	3	1.57021
rs1044563	PPP1R3C	10	93380129	1	4.706492
rs3758499	TNKS2	10	93598122	1	2.237951
rs3758499	TNKS2	10	93598122	5	0.681286
rs3758499	TNKS2	10	93598122	7	1.829429
rs3758499	TNKS2	10	93598122	8	1.046567
rs11186737	FGFBP3	10	93656329	5	1.262787
rs11186737	FGFBP3	10	93656329	7	0.352653
rs11186737	FGFBP3	10	93656329	1	1.737182
rs1107947	FGFBP3	10	93658090	1	3.461692
rs1107947	FGFBP3	10	93658090	4	2.176484
rs1107947	FGFBP3	10	93658090	7	7.118555
rs1107947	FGFBP3	10	93658090	5	0.257144
rs746842	5-Mar	10	94040968	3	0.06658
rs746842	5-Mar	10	94040968	5	0.923492
rs1057848	5-Mar	10	94102136	8	0.134169
rs1057848	5-Mar	10	94102136	5	0.29196
rs1057848	5-Mar	10	94102136	3	0.267137
rs2209972	LOC643768	10	94169008	2	0.585022
rs2209972	LOC643768	10	94169008	8	0.731193
rs2209972	LOC643768	10	94169008	1	1.089876
rs2209972	LOC643768	10	94169008	3	1.300823
rs2490741	EXOC6	10	94584545	3	0.070166
rs11187225	EXOC6	10	94704407	6	0.441771
rs11187225	EXOC6	10	94704407	7	5.439477
rs7070947	LOC387703	10	94957066	5	4.667833
rs7070947	LOC387703	10	94957066	4	2.77897
rs7070947	LOC387703	10	94957066	1	4.467407
rs7070947	LOC387703	10	94957066	8	3.300088
rs7070947	LOC387703	10	94957066	2	2.836952
rs11187393	FER1L3	10	95097418	4	1.584569
rs11187393	FER1L3	10	95097418	1	4.938193

SNP10-95151258	FER1L3	10	95151258	8	4.593122
rs12262721	FER1L3	10	95153799	2	9.474972
rs3740370	CEP55	10	95249969	1	6.297589
rs3740370	CEP55	10	95249969	7	0.124319
rs3740370	CEP55	10	95249969	5	0.1168
rs12781149	PDE6C	10	95362754	6	1.485076
rs714550	PDE6C	10	95379031	1	1.298259
rs714550	PDE6C	10	95379031	7	2.06185
rs714550	PDE6C	10	95379031	4	2.063571
rs714550	PDE6C	10	95379031	2	2.915796
rs2275438	C10orf4	10	95444671	7	0.545434
rs2275438	C10orf4	10	95444671	5	0.122414
rs12768993	PIPSL	10	95708027	5	0.132075
rs10882386	PLCE1	10	95780659	5	0.564315
rs17517313	PLCE1	10	96077718	1	2.421548
rs12572897	NOC3L	10	96104825	3	1.510171
SNP10-96285436	TBC1D12	10	96285436	8	5.050084
rs1042192	CYP2C18	10	96485274	3	0.046893
rs4244285	CYP2C19	10	96531606	3	0.159128
VG10S3890	CYP2C9	10	96731043	3	0.123422
rs1057911	CYP2C9	10	96738727	3	1.925063
rs9332242	CYP2C9	10	96738883	7	0.396797
rs9332242	CYP2C9	10	96738883	5	0.397424
rs9332242	CYP2C9	10	96738883	2	0.524289
rs1058932	CYP2C8	10	96786851	4	2.793628
rs1058932	CYP2C8	10	96786851	3	0.537157
rs10509681	CYP2C8	10	96788739	2	4.715539
rs10509681	CYP2C8	10	96788739	7	1.115927
rs10509681	CYP2C8	10	96788739	5	0.134793
rs1058930	CYP2C8	10	96808109	4	5.013978
rs591157	C10orf129	10	96944288	5	1.096052
rs591157	C10orf129	10	96944288	3	1.38595
rs591157	C10orf129	10	96944288	1	1.095751
rs11188225	C10orf129	10	96944350	3	0.267171
rs689207	C10orf129	10	96975533	3	0.535494
rs689207	C10orf129	10	96975533	1	1.56743
rs688706	C10orf129	10	96975691	3	0.084624
rs688706	C10orf129	10	96975691	1	0.041049
rs1132810	SORBS1	10	97061739	2	4.022068
rs9313	SORBS1	10	97061862	2	3.616979
rs2281939	SORBS1	10	97164342	8	0.538236
rs10509698	CC2D2B	10	97782342	3	0.381745
SNP10-97909001	ZNF518A	10	97909001	1	0.088429
SNP10-97909001	ZNF518A	10	97909001	3	0.172941
SNP10-97909001	ZNF518A	10	97909001	2	4.949771
rs4917734	TLL2	10	98115387	2	0.09931
rs4917734	TLL2	10	98115387	4	0.188612

rs4917734	TLL2	10	98115387	6	0.394767
rs3814221	TLL2	10	98117580	7	0.450434
rs7448	PIK3AP1	10	98343203	3	0.452552
rs7448	PIK3AP1	10	98343203	7	0.417353
rs7448	PIK3AP1	10	98343203	2	1.324336
SNP10-98370127	PIK3AP1	10	98370127	1	3.611142
SNP10-98370127	PIK3AP1	10	98370127	7	0.088245
SNP10-98370127	PIK3AP1	10	98370127	6	1.770521
rs3748229	PIK3AP1	10	98459497	4	0.299803
rs3748229	PIK3AP1	10	98459497	7	0.122429
rs3748229	PIK3AP1	10	98459497	6	2.234466
rs3748229	PIK3AP1	10	98459497	3	0.805484
rs17112076	PIK3AP1	10	98459683	5	0.433768
rs7098255	C10orf12	10	98732011	1	3.850732
rs7098255	C10orf12	10	98732011	8	0.41835
rs3814163	C10orf12	10	98732595	1	5.552813
rs12572241	ARHGAP19	10	98975602	7	0.749961
rs13439	ARHGAP19	10	98996073	7	0.400424
rs13439	ARHGAP19	10	98996073	5	0.399876
rs13439	ARHGAP19	10	98996073	3	0.277474
rs928603	LOC644215	10	99173392	1	0.60481
rs928603	LOC644215	10	99173392	2	1.063111
rs928603	LOC644215	10	99173392	3	0.727887
rs928603	LOC644215	10	99173392	6	3.316973
rs928603	LOC644215	10	99173392	4	0.019637
rs4919098	EXOSC1	10	99195721	7	0.637315
rs4919098	EXOSC1	10	99195721	3	1.126788
rs7094973	ANKRD2	10	99327562	6	4.397575
rs7093643	C10orf62	10	99340118	2	2.278769
rs7093643	C10orf62	10	99340118	7	1.753744
rs7093643	C10orf62	10	99340118	1	4.867271
rs7093643	C10orf62	10	99340118	5	0.440108
rs933057	C10orf65	10	99361719	7	2.011873
rs933057	C10orf65	10	99361719	4	1.449894
rs1977640	C10orf65	10	99362043	7	1.198386
rs1977640	C10orf65	10	99362043	4	0.015815
rs3814556	C10orf83	10	99366142	7	0.652773
SNP10-99383224	C10orf83	10	99383224	7	1.360948
rs2039826	SFRP5	10	99516658	3	1.653141
rs2039826	SFRP5	10	99516658	7	1.121528
SNP10-99959465	C10orf28	10	99959465	3	0.125507
SNP10-99959465	C10orf28	10	99959465	5	1.058097
SNP10-99959465	C10orf28	10	99959465	4	0.667709
rs4345897	C10orf33	10	100137050	5	3.843261
rs4345897	C10orf33	10	100137050	3	0.102964
rs4345897	C10orf33	10	100137050	8	5.056016
rs12763326	C10orf33	10	100157386	3	1.269903

rs12763326	C10orf33	10	100157386	1	3.798969
rs1061437	HPS1	10	100166144	8	0.844612
rs1061437	HPS1	10	100166144	3	0.044248
rs1061437	HPS1	10	100166144	1	5.070756
rs3830020	HPS1	10	100166605	3	0.296864
rs3830019	HPS1	10	100166838	1	4.339053
rs10883099	HPSE2	10	100209304	2	1.746312
rs10883099	HPSE2	10	100209304	4	1.358498
rs10883099	HPSE2	10	100209304	8	1.118569
rs10883099	HPSE2	10	100209304	1	1.782955
rs10883099	HPSE2	10	100209304	6	0.641478
rs10883100	HPSE2	10	100209364	6	0.287205
rs10883100	HPSE2	10	100209364	1	1.243293
rs10883100	HPSE2	10	100209364	2	1.262502
rs10883100	HPSE2	10	100209364	4	0.642832
rs2862600	CNNM1	10	101080418	1	0.536978
SNP10-10113768	CNNM1	10	101137682	1	5.123082
SNP10-10113768	CNNM1	10	101137682	7	0.626804
rs2641	GOT1	10	101146749	5	5.039377
rs11867	GOT1	10	101147150	5	0.953171
rs2234971	GOT1	10	101156534	3	0.279014
rs11190245	ENTPD7	10	101441249	7	0.460496
rs11190245	ENTPD7	10	101441249	4	3.713639
rs11190245	ENTPD7	10	101441249	8	1.139277
rs2231687	COX15	10	101463208	1	5.168186
rs717620	ABCC2	10	101532568	1	2.520728
rs2273697	ABCC2	10	101553805	6	2.57831
rs2273697	ABCC2	10	101553805	7	0.885591
rs2273697	ABCC2	10	101553805	4	0.091339
rs2273697	ABCC2	10	101553805	2	3.972447
rs2273697	ABCC2	10	101553805	8	0.071762
rs1137968	ABCC2	10	101596851	2	4.655564
rs8187707	ABCC2	10	101600523	2	6.057557
SNP10-10165780	DNMBP	10	101657804	5	0.765662
SNP10-10170610	DNMBP	10	101706102	6	7.862457
rs2270962	CWF19L1	10	102006034	3	0.478607
rs12784396	CWF19L1	10	102017397	7	1.250459
rs12784396	CWF19L1	10	102017397	3	1.431816
rs11557934	SCD	10	102112702	5	1.602756
rs11557934	SCD	10	102112702	1	2.405097
rs3793772	WNT8B	10	102212815	3	0.757035
rs3793772	WNT8B	10	102212815	6	0.424245
rs3793772	WNT8B	10	102212815	5	2.865299
rs3793771	WNT8B	10	102212947	3	1.100338
rs3793771	WNT8B	10	102212947	5	0.361329
rs11190581	WNT8B	10	102233377	7	0.908357
rs11190581	WNT8B	10	102233377	6	0.283027

rs11190581	WNT8B	10	102233377	5	1.39175
rs11190581	WNT8B	10	102233377	3	0.93357
rs2298075	SEC31B	10	102237398	3	0.904856
rs2298075	SEC31B	10	102237398	5	0.100303
rs2298075	SEC31B	10	102237398	6	2.756122
rs2298075	SEC31B	10	102237398	7	0.287316
rs41290542	SEC31B	10	102248981	7	0.814232
rs3793706	SEC31B	10	102259075	3	1.604394
rs3793706	SEC31B	10	102259075	5	0.880601
rs3763695	SEC31B	10	102259196	6	0.280271
rs3763695	SEC31B	10	102259196	3	0.323136
rs3763695	SEC31B	10	102259196	5	0.141294
rs3763695	SEC31B	10	102259196	7	0.097858
rs1800898	PAX2	10	102558973	7	2.313592
rs1800898	PAX2	10	102558973	2	1.868646
rs1800898	PAX2	10	102558973	6	2.607718
rs1800898	PAX2	10	102558973	4	1.450364
rs12098288	PAX2	10	102577564	6	2.049003
rs927302	C10orf6	10	102712906	2	7.157846
rs927302	C10orf6	10	102712906	1	0.696315
GA009036	C10orf2	10	102743778	3	0.064084
GA009036	C10orf2	10	102743778	8	2.743096
GA009036	C10orf2	10	102743778	6	0.934741
rs3740488	C10orf2	10	102744023	2	3.426668
rs3740488	C10orf2	10	102744023	1	0.291229
rs752974	LZTS2	10	102752246	1	1.96189
rs752974	LZTS2	10	102752246	4	0.109154
rs701836	LZTS2	10	102757107	2	0.799249
rs701836	LZTS2	10	102757107	1	0.04064
rs701836	LZTS2	10	102757107	4	0.319424
rs14177	LZTS2	10	102757145	1	0.243985
rs14177	LZTS2	10	102757145	4	0.620658
rs807023	NULL	10	102760072	2	4.917305
GA027197	KAZALD1	10	102814339	4	1.034296
GA027197	KAZALD1	10	102814339	5	1.506701
rs807037	KAZALD1	10	102814339	4	8.614371
rs807037	KAZALD1	10	102814339	2	5.820859
rs807037	KAZALD1	10	102814339	5	5.361336
rs12359843	KAZALD1	10	102814750	5	4.997954
rs17113735	TLX1	10	102886839	1	0.976869
rs1051723	TLX1	10	102887047	4	1.428338
rs4919551	BTRC	10	103303536	7	0.770061
rs4919552	BTRC	10	103305168	7	0.512883
rs10883689	KCNIP2	10	103576346	2	1.937287
rs10883689	KCNIP2	10	103576346	6	0.093171
rs10883689	KCNIP2	10	103576346	7	0.550435
rs10883689	KCNIP2	10	103576346	4	0.752765

rs11596387	C10orf76	10	103596112	4	0.108702
rs3737243	HPS6	10	103815737	4	0.002869
rs3737243	HPS6	10	103815737	6	0.512744
rs3737243	HPS6	10	103815737	2	2.739481
rs11191274	GBF1	10	104130340	1	4.154084
rs1057050	GBF1	10	104132284	5	7.930473
rs11574851	NFKB2	10	104150949	8	1.404136
rs2282295	PSD	10	104164885	1	2.926664
rs11191295	TMEM180	10	104221044	2	3.635098
rs11191295	TMEM180	10	104221044	1	0.781738
rs1056744	TMEM180	10	104226677	1	0.56027
rs1056744	TMEM180	10	104226677	2	0.851068
rs17114808	SUFU	10	104381275	1	5.807912
rs12573077	ARL3	10	104424620	6	0.140575
rs12573077	ARL3	10	104424620	8	3.927767
rs6163	CYP17A1	10	104586914	1	1.713482
rs743572	CYP17A1	10	104587142	1	0.635169
rs943037	CNNM2	10	104825909	1	3.468187
rs2296568	CNNM2	10	104826930	8	4.095263
rs12573199	NT5C2	10	104838834	2	1.233159
rs1163236	NT5C2	10	104942997	2	3.628722
rs1063461	INA	10	105039095	7	0.627558
rs1063461	INA	10	105039095	6	0.223371
rs2986014	PDCD11	10	105184076	4	0.740373
rs11191692	FAM26C	10	105203755	7	0.624112
rs11191692	FAM26C	10	105203755	4	1.705531
rs11191692	FAM26C	10	105203755	1	1.099883
rs11191692	FAM26C	10	105203755	2	0.137877
rs11191692	FAM26C	10	105203755	6	0.989832
rs2986016	FAM26C	10	105205006	6	1.534605
rs7918186	NEURL	10	105340437	5	3.163869
rs7918186	NEURL	10	105340437	4	0.520802
rs41287516	SH3PXD2A	10	105362913	3	1.083602
rs10786775	OBFC1	10	105647306	2	0.257051
rs10786775	OBFC1	10	105647306	1	0.553486
rs10786775	OBFC1	10	105647306	3	0.690163
rs2274100	COL17A1	10	105788862	8	0.644659
rs2274100	COL17A1	10	105788862	1	3.431923
rs2296219	COL17A1	10	105789206	1	3.156043
rs2296219	COL17A1	10	105789206	8	1.495852
rs2296219	COL17A1	10	105789206	4	1.190581
rs4918079	COL17A1	10	105791243	4	1.531865
rs4918079	COL17A1	10	105791243	8	3.438286
rs4918079	COL17A1	10	105791243	1	1.881884
rs805722	COL17A1	10	105800390	3	0.266719
rs805722	COL17A1	10	105800390	8	0.904988
rs805722	COL17A1	10	105800390	4	1.047247

rs17750886	C10orf79	10	105917450	4	0.132391
rs17750886	C10orf79	10	105917450	3	1.145593
rs17750886	C10orf79	10	105917450	8	2.981967
rs505701	KIAA1754	10	106063556	4	6.300952
rs17754543	KIAA1754	10	106083473	4	0.316886
rs17754543	KIAA1754	10	106083473	8	2.163329
rs3750261	SORCS3	10	107013380	5	0.615138
rs3750261	SORCS3	10	107013380	3	0.670077
rs874241	RBM20	10	112586560	5	3.439571
rs874241	RBM20	10	112586560	1	2.207697
rs7081726	PDCD4	10	112631043	7	6.118694
rs11195419	ADRA2A	10	112829358	7	0.390972
rs13306146	ADRA2A	10	112830161	7	1.667874
rs1129555	GPAM	10	113900711	1	3.671683
rs1129555	GPAM	10	113900711	7	0.59789
rs1129555	GPAM	10	113900711	3	0.11246
rs2254537	GPAM	10	113907075	7	4.186681
rs2254537	GPAM	10	113907075	3	0.223477
rs2254537	GPAM	10	113907075	1	5.080107
rs10787428	GPAM	10	113925369	7	1.107417
rs10787428	GPAM	10	113925369	1	5.528226
rs10787428	GPAM	10	113925369	3	0.635892
rs2792751	GPAM	10	113930319	1	5.654753
rs2290965	ZDHHC6	10	114180342	4	5.056522
rs2290965	ZDHHC6	10	114180342	7	8.21628
rs3740530	HABP2	10	115324114	2	0.082712
rs2245056	HABP2	10	115331743	8	1.855334
rs3832698	NRAP	10	115338717	8	0.508787
rs3127106	NRAP	10	115395605	6	1.353907
rs3127106	NRAP	10	115395605	7	0.525121
rs3127106	NRAP	10	115395605	2	0.704163
rs3121478	NRAP	10	115395654	6	1.252367
rs3121478	NRAP	10	115395654	7	1.867333
rs3121478	NRAP	10	115395654	2	2.20891
rs2154028	NRAP	10	115401604	2	1.741747
rs2227310	CASP7	10	115479142	7	0.802084
rs2227310	CASP7	10	115479142	5	1.35689
rs2227310	CASP7	10	115479142	3	0.193282
rs10567	DCLRE1A	10	115584715	5	2.592142
rs7095762	C10orf118	10	115900918	1	5.525638
rs7095762	C10orf118	10	115900918	8	4.824356
rs1061159	C10orf118	10	115912764	8	8.766489
rs1061159	C10orf118	10	115912764	1	5.441538
rs12266538	TDRD1	10	115981735	6	0.774661
rs9664945	VWA2	10	115998487	1	3.253517
rs7350409	AFAP1L2	10	116044575	1	4.207278
rs2781806	AFAP1L2	10	116050417	2	5.35481

rs621375	AFAP1L2	10	116063791	3	0.72405
rs621375	AFAP1L2	10	116063791	5	0.510443
rs6646	ABLIM1	10	116181164	4	0.218664
rs6646	ABLIM1	10	116181164	6	0.969984
rs3802734	ABLIM1	10	116184200	2	3.49081
rs3802734	ABLIM1	10	116184200	4	0.91516
rs3802734	ABLIM1	10	116184200	8	4.949328
rs1061413	GFRA1	10	117812953	1	1.76865
rs2072277	GFRA1	10	117813194	2	6.401078
rs1078080	GFRA1	10	117813729	1	0.526047
rs2245020	GFRA1	10	117874940	7	1.388664
rs2245020	GFRA1	10	117874940	1	0.848369
rs2245020	GFRA1	10	117874940	6	2.754101
rs2245020	GFRA1	10	117874940	2	7.915876
rs10787669	PNLIPRP3	10	118177389	5	0.524116
rs11544712	PNLIPRP2	10	118377272	3	1.158584
rs2301179	PNLIPRP2	10	118394610	3	0.492757
rs2301179	PNLIPRP2	10	118394610	6	1.264777
rs2301179	PNLIPRP2	10	118394610	2	2.508899
rs2301179	PNLIPRP2	10	118394610	4	1.316778
rs17095095	HSPA12A	10	118421287	6	1.236751
rs17095095	HSPA12A	10	118421287	4	0.753897
rs17095095	HSPA12A	10	118421287	3	0.138758
rs17095095	HSPA12A	10	118421287	1	4.699339
rs11197793	HSPA12A	10	118425979	5	0.247516
rs7083543	DKFZp781N1041	10	118606100	8	0.95339
rs7083543	DKFZp781N1041	10	118606100	2	0.547062
rs11197835	DKFZp781N1041	10	118610449	4	0.157316
rs11197835	DKFZp781N1041	10	118610449	7	0.228794
rs3814230	PDZD8	10	119123958	4	0.212191
rs3814230	PDZD8	10	119123958	6	0.409455
rs3814230	PDZD8	10	119123958	3	0.149816
rs2768327	EMX2OS	10	119237657	1	1.776551
rs242965	EMX2OS	10	119240201	1	1.106307
rs242964	EMX2OS	10	119240373	5	0.066541
rs1369	CASC2	10	119957233	5	0.403957
rs1369	CASC2	10	119957233	1	4.062564
rs703434	C10orf84	10	120059107	5	0.080418
rs7899785	LOC729666	10	120682761	7	3.451338
rs10749291	SFXN4	10	120910578	7	1.803483
rs10749291	SFXN4	10	120910578	1	4.097441
rs10749291	SFXN4	10	120910578	4	2.209666
rs11199005	RGS10	10	121286019	7	1.9908
rs11199005	RGS10	10	121286019	2	0.728764
rs11199005	RGS10	10	121286019	1	1.060068
rs11199005	RGS10	10	121286019	6	1.424004
rs7919868	TIAL1	10	121346372	3	1.66717

rs7919868	TIAL1	10	121346372	1	1.976263
rs7919868	TIAL1	10	121346372	6	0.179503
rs11199065	BAG3	10	121427319	7	1.764863
rs11199065	BAG3	10	121427319	1	3.998759
rs2901257	C10orf85	10	122348453	8	5.066378
rs2901257	C10orf85	10	122348453	3	0.430412
rs35350755	ATE1	10	123539681	6	3.407081
rs35350755	ATE1	10	123539681	8	0.839979
rs35350755	ATE1	10	123539681	1	4.306954
rs35350755	ATE1	10	123539681	3	0.93682
rs11200385	TACC2	10	123832513	3	0.192268
rs11200385	TACC2	10	123832513	6	0.491585
rs4752642	TACC2	10	123836278	8	1.940363
rs4752642	TACC2	10	123836278	3	0.50686
rs4752642	TACC2	10	123836278	2	2.716678
rs4752642	TACC2	10	123836278	6	0.479746
rs4751871	TACC2	10	123836475	3	0.070388
rs4751871	TACC2	10	123836475	6	0.280995
rs7100442	BTBD16	10	124056721	4	1.303117
rs2421013	BTBD16	10	124079026	7	0.488407
rs2421013	BTBD16	10	124079026	4	1.938709
rs2421013	BTBD16	10	124079026	1	3.716661
rs2421013	BTBD16	10	124079026	3	0.36638
rs3817281	BTBD16	10	124086025	1	0.580261
rs3817281	BTBD16	10	124086025	4	0.285009
rs3817281	BTBD16	10	124086025	3	1.378092
rs11523871	DMBT1	10	124319700	8	3.254501
rs11523871	DMBT1	10	124319700	1	1.823238
rs11523871	DMBT1	10	124319700	6	2.107878
rs11523871	DMBT1	10	124319700	2	0.998725
rs11523871	DMBT1	10	124319700	3	1.422671
rs11523871	DMBT1	10	124319700	7	2.597405
rs11248317	LOC729800	10	124575823	2	1.29913
rs11248317	LOC729800	10	124575823	1	1.246802
rs36212407	CUZD1	10	124595656	6	0.994173
rs36212407	CUZD1	10	124595656	8	0.841935
rs36212407	CUZD1	10	124595656	7	0.804992
rs36212407	CUZD1	10	124595656	5	0.709643
rs10902838	LOC399815	10	124638140	1	5.911601
rs10902838	LOC399815	10	124638140	5	0.274438
rs10902838	LOC399815	10	124638140	4	0.640844
rs10902838	LOC399815	10	124638140	8	0.032486
rs10902838	LOC399815	10	124638140	7	6.751032
rs10902838	LOC399815	10	124638140	3	1.214173
rs2293436	FAM24A	10	124660288	4	0.074818
rs3736582	PSTK	10	124732885	1	3.67981
rs3736582	PSTK	10	124732885	4	4.450808

rs3736582	PSTK	10	124732885	5	0.765449
rs12263012	ACADSB	10	124758573	1	2.083692
rs12263012	ACADSB	10	124758573	7	1.131449
SNP10-12479676	ACADSB	10	124796760	4	0.394713
rs11248371	ACADSB	10	124804471	5	0.030432
rs11248371	ACADSB	10	124804471	2	5.98311
rs6838	ACADSB	10	124807738	5	0.077192
rs6838	ACADSB	10	124807738	4	0.457892
rs11248409	HMX3	10	124884159	1	1.663572
rs7897156	BUB3	10	124903986	3	0.634893
rs7095154	BUB3	10	124914392	3	0.238259
rs17608415	GPR26	10	125441603	5	0.996595
rs8277	CPXM2	10	125495391	1	0.648687
rs10794566	CPXM2	10	125511523	8	0.983211
rs10751601	FAM53B	10	126299438	3	0.538687
rs10751601	FAM53B	10	126299438	1	2.762642
rs10751601	FAM53B	10	126299438	7	0.229889
rs3740536	FAM53B	10	126301485	7	0.316488
rs2946994	CTBP2	10	126704704	4	4.832959
rs2946994	CTBP2	10	126704704	1	0.254901
rs2946994	CTBP2	10	126704704	2	6.391717
rs2946994	CTBP2	10	126704704	6	0.133928
rs2946994	CTBP2	10	126704704	8	4.712567
rs2946994	CTBP2	10	126704704	5	2.431553
rs3012075	CTBP2	10	126704956	6	0.010224
rs3012075	CTBP2	10	126704956	2	0.649748
rs3012075	CTBP2	10	126704956	5	0.064129
rs3012075	CTBP2	10	126704956	8	1.447157
rs3012075	CTBP2	10	126704956	1	0.162953
rs3781413	CTBP2	10	126705065	3	0.328478
rs3781413	CTBP2	10	126705065	5	0.273213
rs3781413	CTBP2	10	126705065	1	1.904992
rs3781411	CTBP2	10	126705426	5	1.152123
rs3781411	CTBP2	10	126705426	1	4.701676
rs3781411	CTBP2	10	126705426	3	2.196332
rs3781409	CTBP2	10	126705619	2	0.265113
rs3781409	CTBP2	10	126705619	4	0.181739
rs3781408	CTBP2	10	126705682	5	1.16114
rs2938006	CTBP2	10	126706336	2	0.834554
rs2938006	CTBP2	10	126706336	8	2.938824
rs2938006	CTBP2	10	126706336	6	0.108241
rs2938006	CTBP2	10	126706336	4	0.813182
rs2938006	CTBP2	10	126706336	1	0.460122
rs2938006	CTBP2	10	126706336	5	0.31895
rs17153510	C10orf137	10	127431429	2	2.907063
rs7913591	ADAM12	10	127695394	3	0.894479
rs7913591	ADAM12	10	127695394	8	5.580747

rs2292692	ADAM12	10	127727920	4	0.80661
rs2292692	ADAM12	10	127727920	3	0.115255
rs1278279	ADAM12	10	127743468	8	4.803943
rs1278279	ADAM12	10	127743468	4	1.131559
rs7077404	hCG_2044975	10	128137874	7	1.149405
rs7077404	hCG_2044975	10	128137874	3	1.421522
rs7077404	hCG_2044975	10	128137874	5	1.209292
SNP10-12818335	C10orf90	10	128183358	2	4.458402
rs1436803	C10orf90	10	128192425	8	6.240668
rs1436803	C10orf90	10	128192425	2	4.763984
rs4962342	C10orf90	10	128199949	3	0.115409
rs11595374	C10orf141	10	128825473	6	0.851167
rs11595374	C10orf141	10	128825473	1	5.076645
rs2229599	DOCK1	10	129097419	3	1.207287
rs1051039	DOCK1	10	129140593	6	0.140827
rs1051039	DOCK1	10	129140593	2	1.947024
rs1051039	DOCK1	10	129140593	8	0.083081
rs1051039	DOCK1	10	129140593	1	0.372343
rs1051039	DOCK1	10	129140593	5	0.454038
rs4143258	LOC387720	10	129423831	7	0.434284
SNP10-12974440	PTPRE	10	129744404	1	5.578781
rs7100666	PTPRE	10	129756439	7	0.551799
rs7100666	PTPRE	10	129756439	1	5.322532
rs8436	MKI67	10	129784973	4	0.621512
rs8436	MKI67	10	129784973	7	0.271807
rs8436	MKI67	10	129784973	8	0.551672
rs8436	MKI67	10	129784973	2	6.858843
rs1050793	MKI67	10	129785351	7	0.799297
rs1050793	MKI67	10	129785351	8	11.94529
rs1050793	MKI67	10	129785351	2	7.323301
rs1050793	MKI67	10	129785351	4	0.305421
rs1050793	MKI67	10	129785351	1	0.262457
rs4750935	MKI67	10	129787304	7	0.351481
rs2071496	MKI67	10	129791491	1	2.688748
rs2071496	MKI67	10	129791491	5	2.638221
rs2071496	MKI67	10	129791491	6	8.016119
SNP10-12979173	MKI67	10	129791737	1	1.845386
SNP10-12979173	MKI67	10	129791737	5	0.472774
rs12777740	MKI67	10	129792148	7	0.351389
SNP10-12979446	MKI67	10	129794464	3	0.872726
SNP10-12979446	MKI67	10	129794464	7	0.758237
SNP10-12979446	MKI67	10	129794467	7	0.72524
SNP10-12979446	MKI67	10	129794467	4	3.260813
rs3740423	MKI67	10	129795886	6	0.212156
rs3740423	MKI67	10	129795886	1	3.539714
rs2240	MKI67	10	129797534	7	6.990494
rs2240	MKI67	10	129797534	4	0.825317

SNP10-12979760	MKI67	10	129797600	5	1.576401
rs2071498	MKI67	10	129807550	4	2.117279
rs2071498	MKI67	10	129807550	1	4.104061
rs2071498	MKI67	10	129807550	8	0.832094
rs2071498	MKI67	10	129807550	6	0.802697
rs3740427	hCG_1795091	10	130603278	7	1.234846
rs12917	MGMT	10	131396273	2	6.176935
rs12917	MGMT	10	131396273	1	0.465458
rs12917	MGMT	10	131396273	3	0.21378
rs8217	EBF3	10	131523710	4	0.063507
rs8217	EBF3	10	131523710	2	2.022042
rs8217	EBF3	10	131523710	5	1.332794
rs8217	EBF3	10	131523710	7	0.993928
rs8217	EBF3	10	131523710	8	0.093935
rs13991	GLRX3	10	131824737	6	0.447488
rs13991	GLRX3	10	131824737	2	0.146637
rs7088377	TCERG1L	10	132781016	8	0.583068
rs7088377	TCERG1L	10	132781016	3	1.447327
rs7088377	TCERG1L	10	132781016	1	0.310908
rs7088377	TCERG1L	10	132781016	5	0.12038
rs7088377	TCERG1L	10	132781016	2	0.363058
rs7088377	TCERG1L	10	132781016	6	1.282479
rs7088377	TCERG1L	10	132781016	4	1.479833
rs7071145	TCERG1L	10	132781192	3	0.650197
rs7071145	TCERG1L	10	132781192	2	1.334309
rs7071145	TCERG1L	10	132781192	6	4.014691
rs7071145	TCERG1L	10	132781192	5	1.113459
rs9419600	FLJ46300	10	133457501	2	0.115616
rs9419600	FLJ46300	10	133457501	3	0.070252
rs9419600	FLJ46300	10	133457501	4	0.885133
rs4897739	FLJ46300	10	133457882	3	0.346268
rs4897783	FLJ46300	10	133458227	2	0.540549
rs4897783	FLJ46300	10	133458227	3	0.153116
rs4897742	FLJ46300	10	133458281	4	1.234799
rs2818384	C10orf39	10	133811484	5	0.069751
rs2818384	C10orf39	10	133811484	7	0.475717
rs2818384	C10orf39	10	133811484	1	0.826988
rs2818384	C10orf39	10	133811484	2	0.788271
rs2247705	DPYSL4	10	133863980	4	0.415474
rs2247705	DPYSL4	10	133863980	6	3.212413
rs2247705	DPYSL4	10	133863980	1	4.46288
rs12313	DPYSL4	10	133867285	1	1.420738
rs12313	DPYSL4	10	133867285	4	0.672512
rs12313	DPYSL4	10	133867285	2	3.668167
rs2474329	LRRC27	10	134011507	5	0.664803
rs4443996	LRRC27	10	134040995	5	1.355089
rs4443996	LRRC27	10	134040995	1	1.408032

rs4443996	LRRC27	10	134040995	6	1.520476
rs4443996	LRRC27	10	134040995	7	2.286313
rs4443996	LRRC27	10	134040995	2	1.89891
rs10870302	LRRC27	10	134041728	1	0.225338
rs10870303	LRRC27	10	134041801	5	0.907976
rs10870303	LRRC27	10	134041801	1	1.606969
rs10870303	LRRC27	10	134041801	7	1.282884
rs11146378	C10orf91	10	134111800	1	3.499603
rs945943	INPP5A	10	134390390	5	0.226481
rs7090011	C10orf92	10	134476373	7	0.975525
rs4880433	C10orf92	10	134496978	7	0.095513
rs4880433	C10orf92	10	134496978	4	0.059983
rs4880433	C10orf92	10	134496978	6	0.184975
rs10870341	C10orf92	10	134509616	7	1.150663
rs12781609	C10orf93	10	134598321	1	5.341069
rs12781609	C10orf93	10	134598321	8	3.582228
rs12781609	C10orf93	10	134598321	4	0.767382
rs12781609	C10orf93	10	134598321	6	1.824087
rs2998139	KNDC1	10	134862152	7	1.462647
rs2998139	KNDC1	10	134862152	2	2.739445
rs2998139	KNDC1	10	134862152	4	2.231241
rs1131718	ADAM8	10	134935744	3	0.175934
rs1131718	ADAM8	10	134935744	5	1.089672
rs3008326	ADAM8	10	134937511	6	0.643158
rs2275725	ADAM8	10	134939025	5	0.870641
SNP10-13504513	PAOX	10	135045138	5	0.26753
rs1046175	PAOX	10	135054940	7	0.216522
rs1046175	PAOX	10	135054940	6	1.534847
rs1329151	SPRN	10	135084383	1	2.001637
rs10776681	SPRN	10	135085679	1	4.231163
rs2492654	LOC619207	10	135128183	3	0.512455
rs2492654	LOC619207	10	135128183	1	3.435976
rs10776682	LOC619207	10	135131627	1	6.502957
rs10776682	LOC619207	10	135131627	3	0.936184
rs10857722	LOC619207	10	135131734	3	0.173085
rs10857722	LOC619207	10	135131734	1	6.922055
rs6413419	CYP2E1	10	135195665	5	0.721752
rs6413419	CYP2E1	10	135195665	2	6.747701
rs2515641	CYP2E1	10	135201352	1	0.07777
rs2515641	CYP2E1	10	135201352	2	2.857474
rs2515641	CYP2E1	10	135201352	5	0.53249
rs7081484	CYP2E1	10	135202480	1	2.612894
rs8181357	SYCE1	10	135220629	8	2.609795
rs12226697	SIRT3	11	206056	3	0.89098
rs11555236	SIRT3	11	223212	1	4.646088
rs11555236	SIRT3	11	223212	5	4.694514
rs11555236	SIRT3	11	223212	8	5.385862

rs11555236	SIRT3	11	223212	6	2.268603
rs7116130	PSMD13	11	234129	7	2.047704
rs7116130	PSMD13	11	234129	8	0.662912
rs7116130	PSMD13	11	234129	5	0.968187
rs7128029	PSMD13	11	234141	7	1.713488
rs7128029	PSMD13	11	234141	5	1.73124
rs7128029	PSMD13	11	234141	6	2.707653
rs1128320	PSMD13	11	234167	5	1.629566
rs1128320	PSMD13	11	234167	8	4.81929
rs1128320	PSMD13	11	234167	6	5.621275
rs1128321	PSMD13	11	234171	6	1.811678
rs1128321	PSMD13	11	234171	5	0.540073
rs1128321	PSMD13	11	234171	7	5.994257
rs1128322	PSMD13	11	234197	5	0.907549
rs1128322	PSMD13	11	234197	8	1.586649
rs1128322	PSMD13	11	234197	6	0.770325
rs1128322	PSMD13	11	234197	7	0.706743
rs11246050	NLRP6	11	274257	1	3.771123
rs11246050	NLRP6	11	274257	6	2.430403
rs11246050	NLRP6	11	274257	3	2.543894
rs2242564	ATHL1	11	285006	1	0.434032
rs2242564	ATHL1	11	285006	5	4.224383
rs2242564	ATHL1	11	285006	2	5.362169
SNP11-371754	B4GALNT4	11	371754	7	0.521135
rs3210908	SIGIRR	11	396483	7	0.044277
rs3210908	SIGIRR	11	396483	6	5.224015
rs10794324	TMEM16J	11	418489	6	0.801671
rs10794324	TMEM16J	11	418489	3	1.202963
rs10794324	TMEM16J	11	418489	5	0.978843
GA009268	PTDSS2	11	480035	5	0.255025
GA009268	PTDSS2	11	480035	3	0.032222
rs10540	RNH1	11	484662	5	7.04973
rs10540	RNH1	11	484662	6	1.024376
rs7944548	RNH1	11	492217	1	4.956624
rs7944548	RNH1	11	492217	3	0.304746
rs7944548	RNH1	11	492217	6	4.254479
rs7944548	RNH1	11	492217	5	0.684749
SNP11-541753	LRRC56	11	541753	7	1.219652
SNP11-541753	LRRC56	11	541753	3	0.559066
SNP11-541753	LRRC56	11	541753	5	0.945671
SNP11-541753	LRRC56	11	541753	4	1.099073
rs12277611	LRRC56	11	544798	6	2.15736
rs12277611	LRRC56	11	544798	5	0.163744
rs12277611	LRRC56	11	544798	7	0.758502
rs2061586	C11orf35	11	548884	5	0.110334
rs2061586	C11orf35	11	548884	7	0.151393
rs2061586	C11orf35	11	548884	4	0.778204

rs12285942	LOC143666	11	564813	7	1.61806
rs12285942	LOC143666	11	564813	4	1.06456
rs12285942	LOC143666	11	564813	5	1.478635
SNP11-599577	KIAA1542	11	599577	4	0.577095
SNP11-599577	KIAA1542	11	599577	7	0.167818
SNP11-599577	KIAA1542	11	599577	5	6.510931
rs702965	KIAA1542	11	601868	8	8.280188
rs702966	KIAA1542	11	601919	5	0.224023
rs702966	KIAA1542	11	601919	4	0.106597
SNP11-604318	IRF7	11	604318	7	4.971803
SNP11-604318	IRF7	11	604318	5	0.332163
SNP11-604318	IRF7	11	604318	4	1.0757
rs7946354	CEND1	11	777679	7	1.6136
rs7946354	CEND1	11	777679	3	0.236987
rs6597982	CEND1	11	778007	6	1.25061
rs6597982	CEND1	11	778007	8	0.758664
rs6597982	CEND1	11	778007	7	1.446764
rs10902221	LRDD	11	792379	3	0.70875
rs7126805	EFCAB4A	11	818916	4	0.487124
rs7126805	EFCAB4A	11	818916	6	7.921579
rs7126805	EFCAB4A	11	818916	5	0.124044
rs7126805	EFCAB4A	11	818916	2	1.264613
rs7126805	EFCAB4A	11	818916	1	0.444862
rs28558789	EFCAB4A	11	819539	6	1.692979
rs28558789	EFCAB4A	11	819539	4	0.434694
rs28558789	EFCAB4A	11	819539	1	2.098572
rs28558789	EFCAB4A	11	819539	2	3.026375
rs28558789	EFCAB4A	11	819539	5	1.169762
SNP11-820044	EFCAB4A	11	820044	8	0.181745
rs35042031	CD151	11	828222	3	0.561622
rs8672	CD151	11	828634	6	0.245332
rs8672	CD151	11	828634	5	0.591927
rs8672	CD151	11	828634	1	0.710999
rs8672	CD151	11	828634	2	5.743292
rs8672	CD151	11	828634	4	0.44988
rs8672	CD151	11	828634	8	6.721175
rs1130719	CD151	11	828760	4	6.83917
rs1130719	CD151	11	828760	5	0.349745
rs1130719	CD151	11	828760	8	0.478966
rs1130719	CD151	11	828760	2	1.641614
rs1130719	CD151	11	828760	1	0.484534
rs1130719	CD151	11	828760	6	0.534209
GA009352	AP2A2	11	1000649	6	0.644513
GA009352	AP2A2	11	1000649	3	0.183063
SNP11-1001597	AP2A2	11	1001597	8	4.773722
rs7481521	MUC6	11	1017811	1	0.625096
rs7481521	MUC6	11	1017811	7	1.73368

rs11245929	MUC2	11	1070954	1	0.75977
rs11245929	MUC2	11	1070954	6	0.066677
rs41345745	MUC2	11	1071112	8	0.194563
rs41345745	MUC2	11	1071112	4	0.713543
rs41345745	MUC2	11	1071112	6	0.424561
rs41345745	MUC2	11	1071112	3	0.264821
rs41345745	MUC2	11	1071112	1	0.927915
SNP11-1071757	MUC2	11	1071757	1	1.597587
SNP11-1071757	MUC2	11	1071757	3	0.062707
rs11245936	MUC2	11	1074362	1	4.49409
rs11245936	MUC2	11	1074362	6	1.036472
SNP11-1083769	MUC2	11	1083769	1	5.346468
rs11245950	MUC2	11	1084629	3	0.373254
rs6421972	MUC2	11	1086494	3	0.601261
rs6421972	MUC2	11	1086494	5	0.096468
rs11245954	MUC2	11	1091078	1	4.038243
rs7126405	MUC2	11	1093296	1	3.788242
rs7126405	MUC2	11	1093296	5	0.585061
rs35783651	NULL	11	1146646	7	0.245713
rs4072884	BRSK2	11	1439296	6	1.091163
rs4072884	BRSK2	11	1439296	4	0.334322
rs4072884	BRSK2	11	1439296	3	0.87826
rs10742185	HCCA2	11	1458246	2	4.572846
SNP11-1737443	CTSD	11	1737443	5	1.215149
SNP11-1737443	CTSD	11	1737443	1	1.86089
rs10743009	LOC390029	11	1780829	7	1.524531
rs10743009	LOC390029	11	1780829	8	1.139102
rs10743009	LOC390029	11	1780829	3	2.216578
GA009397	SYT8	11	1814327	5	8.104205
GA009397	SYT8	11	1814327	2	6.847538
GA009397	SYT8	11	1814327	3	0.122138
GA009397	SYT8	11	1814327	1	0.085206
rs484955	SYT8	11	1814838	5	7.597427
SNP11-1934128	MRPL23	11	1934128	6	5.973927
SNP11-1934128	MRPL23	11	1934128	4	8.995149
SNP11-1934128	MRPL23	11	1934128	2	2.582929
rs2839698	H19	11	1975429	6	0.459862
rs2839698	H19	11	1975429	1	0.355703
rs2585	IGF2	11	2107020	4	5.031769
rs7873	IGF2	11	2107273	5	0.923487
rs2230949	IGF2	11	2110764	6	1.554761
rs1003483	IGF2AS	11	2124119	3	0.05587
rs1003483	IGF2AS	11	2124119	5	1.358003
rs1003483	IGF2AS	11	2124119	7	0.580929
rs1003483	IGF2AS	11	2124119	1	0.401412
rs10770125	IGF2AS	11	2125590	7	1.414231
rs3741205	IGF2AS	11	2126460	1	1.917894

rs3741205	IGF2AS	11	2126460	6	1.208524
rs6357	TH	11	2144814	5	1.838534
rs6357	TH	11	2144814	3	0.644848
rs6357	TH	11	2144814	7	0.907
rs2072072	ASCL2	11	2246772	6	1.170962
rs1501466	C11orf21	11	2277460	3	0.607654
rs756924	C11orf21	11	2279605	5	0.155615
rs1057128	KCNQ1	11	2753813	1	5.574856
rs1057128	KCNQ1	11	2753813	4	2.655369
rs10798	KCNQ1	11	2826741	2	1.037153
rs10798	KCNQ1	11	2826741	4	4.626581
rs10798	KCNQ1	11	2826741	1	2.306383
rs10798	KCNQ1	11	2826741	5	3.417628
rs1048046	SLC22A18	11	2881167	3	0.17607
rs1048046	SLC22A18	11	2881167	4	0.462082
rs1048046	SLC22A18	11	2881167	6	0.886734
rs1129782	SLC22A18	11	2900247	4	3.962619
rs1129782	SLC22A18	11	2900247	6	0.123066
rs1056819	PHLDA2	11	2906437	6	7.561292
rs8505	NAP1L4	11	2922869	1	4.610391
rs8505	NAP1L4	11	2922869	3	0.355132
rs8505	NAP1L4	11	2922869	4	0.442628
rs8505	NAP1L4	11	2922869	8	0.55812
rs2411767	NAP1L4	11	2949929	6	0.276046
rs729662	CARS	11	2984716	3	0.657181
rs729662	CARS	11	2984716	1	2.862735
rs729662	CARS	11	2984716	7	0.25096
rs729662	CARS	11	2984716	4	6.776284
rs3741350	OSBPL5	11	3085603	4	0.261002
rs3741350	OSBPL5	11	3085603	3	1.113371
rs11026004	C11orf36	11	3199922	3	0.422271
rs11026004	C11orf36	11	3199922	5	0.456944
rs12275900	C11orf36	11	3200003	3	0.636896
rs12275900	C11orf36	11	3200003	1	0.485399
rs12275900	C11orf36	11	3200003	5	1.01826
rs10766783	C11orf36	11	3200214	3	0.001386
rs7924967	ZNF195	11	3336572	5	1.918265
rs7924967	ZNF195	11	3336572	7	8.757331
rs2271584	TRPC2	11	3613097	5	0.745197
rs2271584	TRPC2	11	3613097	2	0.913709
rs2271584	TRPC2	11	3613097	3	0.699093
rs2271584	TRPC2	11	3613097	7	1.305897
rs1514691	TRPC2	11	3614012	2	3.57363
rs1514691	TRPC2	11	3614012	7	2.333268
rs1514691	TRPC2	11	3614012	5	0.137707
rs6578404	TRPC2	11	3615061	5	0.754699
rs6578404	TRPC2	11	3615061	7	2.754303

rs2271585	ART5	11	3616344	3	1.45099
rs2271585	ART5	11	3616344	5	1.539881
rs2271586	ART5	11	3616569	7	0.091366
rs2271586	ART5	11	3616569	3	0.205274
rs2271586	ART5	11	3616569	5	4.028309
rs7128137	ART5	11	3619777	8	1.003476
rs7128137	ART5	11	3619777	1	1.914636
rs7128137	ART5	11	3619777	4	2.955573
rs7128137	ART5	11	3619777	7	2.710927
rs15820	FRAG1	11	3804006	6	0.724017
rs15820	FRAG1	11	3804006	8	3.443345
rs15820	FRAG1	11	3804006	1	3.31772
rs926101	TRIM21	11	4367817	5	1.623813
rs926101	TRIM21	11	4367817	1	3.883668
rs1426378	TRIM21	11	4371449	1	6.522783
rs1426378	TRIM21	11	4371449	5	0.373299
rs12295898	OR52M1	11	4523761	1	0.184365
rs12795289	C11orf40	11	4551121	3	0.078382
rs4910638	TRIM68	11	4577437	6	0.779988
rs4910638	TRIM68	11	4577437	3	2.433299
rs4910638	TRIM68	11	4577437	5	4.260857
rs2231975	TRIM68	11	4578215	3	0.354972
rs905871	OR51D1	11	4617861	6	3.50057
rs3817098	OR51E1	11	4631151	1	1.266681
rs10836532	LOC643447	11	4701730	8	1.400932
rs10836532	LOC643447	11	4701730	1	0.912994
rs10836532	LOC643447	11	4701730	4	1.032601
rs10836532	LOC643447	11	4701730	3	0.602884
rs11033544	LOC643447	11	4701741	7	0.04453
rs11033544	LOC643447	11	4701741	3	0.722435
rs1030726	OR51F1	11	4746844	3	1.040538
rs11033796	OR51F1	11	4747334	4	3.472557
rs11033796	OR51F1	11	4747334	3	0.77154
rs2053116	OR52R1	11	4781454	3	0.393037
rs2499953	MMP26	11	4967481	1	2.172769
rs10768450	OR51L1	11	4977408	5	1.617923
rs10768450	OR51L1	11	4977408	3	0.166663
rs2500017	OR52J3	11	5024713	3	0.638956
rs2500017	OR52J3	11	5024713	7	0.277073
rs2500017	OR52J3	11	5024713	1	1.006876
rs17350764	OR52J3	11	5025007	8	0.150934
rs10837374	OR52A4	11	5098478	2	2.916264
rs7947334	OR52A4	11	5099125	7	0.380771
rs7947334	OR52A4	11	5099125	1	6.277489
rs2472530	OR52A5	11	5109837	2	2.223158
rs2472530	OR52A5	11	5109837	7	2.567839
rs1894397	HBG2	11	5232563	5	1.810971

rs11036814	OR51B2	11	5301168	3	0.813311
rs4910551	OR51B5	11	5320852	1	1.339052
rs11036913	OR51B5	11	5321318	8	4.485866
rs4910756	OR51B6	11	5329432	1	1.235955
rs5006887	OR51B6	11	5329705	3	1.035235
rs5006884	OR51B6	11	5329827	3	0.294199
rs5006884	OR51B6	11	5329827	1	0.057045
rs5006884	OR51B6	11	5329827	7	0.319523
rs5006884	OR51B6	11	5329827	6	1.457643
rs5024042	OR51B6	11	5330138	3	0.039551
rs2647598	LOC643745	11	5332852	1	1.767002
rs2647598	LOC643745	11	5332852	3	0.524103
rs17270818	LOC643753	11	5340646	6	2.032888
rs17270818	LOC643753	11	5340646	3	2.627629
rs17270818	LOC643753	11	5340646	1	4.188945
rs17270818	LOC643753	11	5340646	7	2.863736
rs2736588	OR51Q1	11	5400276	1	2.119082
rs10838092	OR51Q1	11	5400443	3	1.10892
rs10838092	OR51Q1	11	5400443	1	1.190215
rs10838093	OR51Q1	11	5400463	1	1.291456
rs10838093	OR51Q1	11	5400463	7	2.627786
rs10838093	OR51Q1	11	5400463	3	2.043424
rs10838095	OR51Q1	11	5400539	3	0.333841
rs10838095	OR51Q1	11	5400539	1	2.420731
rs10838095	OR51Q1	11	5400539	8	3.707262
rs2736586	OR51Q1	11	5400637	3	0.140296
rs2736586	OR51Q1	11	5400637	1	2.671853
rs11037445	OR51I1	11	5418831	1	0.57519
rs12577167	OR51I2	11	5431694	4	0.145776
rs7950082	OR52D1	11	5467174	4	1.379943
rs7101919	OR52D1	11	5467264	2	0.311614
rs2227271	UBQLN3	11	5485494	4	0.456473
rs1044392	UBQLNL	11	5492247	2	2.329056
rs1044392	UBQLNL	11	5492247	6	0.628462
rs2017434	UBQLNL	11	5493428	2	4.240033
rs2017434	UBQLNL	11	5493428	6	0.841908
rs872751	UBQLNL	11	5493621	6	0.483443
rs872751	UBQLNL	11	5493621	2	4.205136
rs2047456	UBQLNL	11	5493737	6	1.088683
rs7131665	LOC643810	11	5496246	8	0.070752
rs7945342	LOC643810	11	5500281	6	0.559482
rs7934354	OR52H1	11	5522482	4	0.187114
rs1995158	OR52H1	11	5522606	3	1.503279
rs1995157	OR52H1	11	5522627	4	0.569931
rs1995156	OR52H1	11	5522688	4	0.485558
rs1566275	OR52H1	11	5522941	3	1.667214
rs1566275	OR52H1	11	5522941	8	0.36863

rs10769054	OR52H1	11	5523065	6	0.770295
rs10769054	OR52H1	11	5523065	4	1.49311
rs11038141	LOC728732	11	5548802	4	4.491746
rs1077126	OR52B6	11	5558851	4	0.889999
rs2341434	OR52B6	11	5559191	7	1.184716
rs2163946	OR52B6	11	5559366	7	0.947135
rs2163946	OR52B6	11	5559366	6	0.007563
rs2163946	OR52B6	11	5559366	2	1.124882
rs10769086	OR52B6	11	5559544	1	3.046909
rs12272467	TRIM6-TRIM34	11	5574563	3	0.938926
rs12272467	TRIM6-TRIM34	11	5574563	2	4.403503
rs12272467	TRIM6-TRIM34	11	5574563	6	0.741438
rs12272467	TRIM6-TRIM34	11	5574563	1	0.894217
rs12272467	TRIM6-TRIM34	11	5574563	7	0.036196
rs7104648	TRIM6	11	5590323	7	1.71456
rs11038346	TRIM6	11	5590621	7	1.318634
rs3740998	TRIM34	11	5621407	1	4.899729
rs3740998	TRIM34	11	5621407	6	5.635925
rs3740995	TRIM5	11	5656916	6	4.66283
rs10838525	TRIM5	11	5657577	2	5.136875
rs10838525	TRIM5	11	5657577	3	1.015766
rs10838525	TRIM5	11	5657577	8	1.516392
rs10838525	TRIM5	11	5657577	7	1.501328
rs1063303	TRIM22	11	5676326	4	1.761627
rs1063303	TRIM22	11	5676326	6	0.243353
rs1063303	TRIM22	11	5676326	7	1.452588
rs12364019	TRIM22	11	5686919	4	2.734281
rs12417798	TRIM22	11	5688158	2	4.467559
rs12417798	TRIM22	11	5688158	1	0.529011
rs7397032	OR56B1	11	5714638	6	0.796551
rs7397032	OR56B1	11	5714638	1	1.321857
rs7397032	OR56B1	11	5714638	2	1.129563
rs4910844	OR52N4	11	5733060	3	1.067305
rs12363178	OR52N4	11	5733171	1	1.18095
rs10769224	OR52N1	11	5766249	3	2.908937
rs4758435	OR52N2	11	5798432	7	1.479095
rs8181512	OR52N2	11	5798932	1	0.640284
rs8181512	OR52N2	11	5798932	7	1.182736
rs8181512	OR52N2	11	5798932	3	1.610102
rs10160336	OR52E5	11	5878798	7	0.61634
rs1840178	OR56A3	11	5925304	7	0.717297
rs1840178	OR56A3	11	5925304	3	1.080212
rs1462983	OR56B4	11	6086413	4	1.150409
rs10839531	OR52W1	11	6177745	5	0.561561
rs10839531	OR52W1	11	6177745	2	0.455031
rs11040799	OR52W1	11	6177790	3	0.841461
rs11040799	OR52W1	11	6177790	5	0.208514

rs10769671	C11orf42	11	6188307	1	1.345259
rs10769671	C11orf42	11	6188307	8	0.187233
rs10769671	C11orf42	11	6188307	5	0.075785
rs10769671	C11orf42	11	6188307	3	0.594058
rs10769671	C11orf42	11	6188307	2	0.581289
SNP11-6195239	C11orf56	11	6195239	7	0.094972
rs11040809	C11orf56	11	6195715	3	0.599708
rs11040809	C11orf56	11	6195715	7	0.306708
rs11040809	C11orf56	11	6195715	4	0.591307
rs11040809	C11orf56	11	6195715	8	3.259348
rs3750945	C11orf56	11	6200380	2	4.738434
rs3750945	C11orf56	11	6200380	5	0.101678
rs7129498	CNGA4	11	6219291	5	0.275421
rs7129498	CNGA4	11	6219291	2	2.648361
rs1042047	CCKBR	11	6249758	3	1.397637
rs1042047	CCKBR	11	6249758	2	3.895615
rs1042047	CCKBR	11	6249758	7	0.644145
SNP11-6297941	PRKCDBP	11	6297941	8	1.350355
SNP11-6297941	PRKCDBP	11	6297941	4	1.176491
SNP11-6297941	PRKCDBP	11	6297941	7	0.181774
rs10606	TRIM3	11	6434407	8	0.372242
rs10606	TRIM3	11	6434407	4	0.619457
rs10606	TRIM3	11	6434407	7	0.920861
rs11604726	FXC1	11	6459303	8	1.686937
rs11604726	FXC1	11	6459303	4	1.034039
rs11555935	FXC1	11	6459356	4	0.035254
rs11555935	FXC1	11	6459356	7	0.241129
SNP11-6476218	C11orf47	11	6476218	1	4.607425
SNP11-6476218	C11orf47	11	6476218	3	0.617249
rs2555158	C11orf47	11	6476740	3	0.382693
rs2555158	C11orf47	11	6476740	1	5.099719
rs11603869	C11orf47	11	6497801	8	1.058044
rs11603869	C11orf47	11	6497801	4	0.504554
rs11040923	LOC728714	11	6541583	6	3.918961
rs11040923	LOC728714	11	6541583	1	3.211869
rs11040923	LOC728714	11	6541583	4	0.787852
rs1045450	TPP1	11	6591235	4	0.133343
rs1128396	TPP1	11	6592682	4	0.594705
rs4758443	DCHS1	11	6605000	2	2.817892
rs4758443	DCHS1	11	6605000	7	0.821549
rs4758443	DCHS1	11	6605000	4	1.032468
rs7937911	GVIN1	11	6692445	3	0.517519
rs11040982	GVIN1	11	6694555	2	2.711665
rs11040982	GVIN1	11	6694555	7	1.105497
rs11040983	GVIN1	11	6694867	6	0.501378
rs10769716	GVIN1	11	6695528	3	0.291968
rs10769716	GVIN1	11	6695528	4	1.571699

rs12284429	GVIN1	11	6695983	3	1.16754
rs12284429	GVIN1	11	6695983	7	0.940881
rs12284429	GVIN1	11	6695983	5	0.57847
rs12284429	GVIN1	11	6695983	4	0.461613
rs12284429	GVIN1	11	6695983	6	0.821365
rs12223353	GVIN1	11	6696884	7	0.80357
rs12223353	GVIN1	11	6696884	3	0.272948
rs11040999	GVIN1	11	6724185	5	0.988044
rs11040999	GVIN1	11	6724185	4	1.712662
rs11040999	GVIN1	11	6724185	2	2.876496
rs11040999	GVIN1	11	6724185	3	0.339103
rs10839616	OR2AG2	11	6746604	7	0.008335
rs10839616	OR2AG2	11	6746604	4	1.566481
rs10839616	OR2AG2	11	6746604	5	0.430167
rs866347	OR6A2	11	6773354	3	0.349219
rs866347	OR6A2	11	6773354	6	0.698662
rs7926083	OR10A2	11	6848334	4	1.458412
rs7926083	OR10A2	11	6848334	7	0.284406
rs7926083	OR10A2	11	6848334	3	0.451689
rs10839635	OR10A4	11	6855239	5	3.61059
rs10839635	OR10A4	11	6855239	6	0.908987
rs10839635	OR10A4	11	6855239	2	3.132854
rs7102808	OR10A4	11	6855334	4	0.949499
rs7102808	OR10A4	11	6855334	3	0.930789
rs7102808	OR10A4	11	6855334	6	0.815507
rs2741804	OR2D2	11	6869703	6	2.037151
rs2741804	OR2D2	11	6869703	5	1.135274
rs1965209	OR2D2	11	6869866	4	0.681615
rs1965209	OR2D2	11	6869866	6	4.139277
rs1965209	OR2D2	11	6869866	3	0.842335
rs10839659	OR2D3	11	6899302	1	6.281151
rs10839659	OR2D3	11	6899302	7	0.495402
rs10839659	OR2D3	11	6899302	3	0.22929
rs10839659	OR2D3	11	6899302	8	0.818266
rs1466682	ZNF215	11	6904337	6	0.728761
rs1466682	ZNF215	11	6904337	1	0.389753
rs1466682	ZNF215	11	6904337	2	7.568447
rs1466682	ZNF215	11	6904337	7	0.0416
rs2239731	ZNF215	11	6933564	1	6.378442
rs2239731	ZNF215	11	6933564	5	4.431909
rs2239731	ZNF215	11	6933564	6	0.507128
rs2239730	ZNF215	11	6933751	5	0.363664
rs2239730	ZNF215	11	6933751	8	4.752633
rs2239730	ZNF215	11	6933751	6	6.665671
rs2239730	ZNF215	11	6933751	1	5.750574
rs2638096	ZNF215	11	6934492	5	0.275501
rs2638096	ZNF215	11	6934492	8	4.289837

rs2638096	ZNF215	11	6934492	1	5.26647
rs2638096	ZNF215	11	6934492	6	5.531185
rs1156525	ZNF214	11	6979107	5	0.206196
rs1156525	ZNF214	11	6979107	4	1.521547
rs1156525	ZNF214	11	6979107	8	0.005066
rs1156525	ZNF214	11	6979107	1	0.149722
rs1156525	ZNF214	11	6979107	2	2.443158
rs1156525	ZNF214	11	6979107	3	0.016291
rs1156526	ZNF214	11	6979293	5	0.500755
rs1156526	ZNF214	11	6979293	3	0.804578
rs1156526	ZNF214	11	6979293	8	0.713869
rs1156526	ZNF214	11	6979293	1	0.094785
rs1156526	ZNF214	11	6979293	4	1.059319
SNP11-7016557	NLRP14	11	7016557	4	3.987681
SNP11-7016557	NLRP14	11	7016557	1	0.08272
SNP11-7016557	NLRP14	11	7016557	3	0.219276
rs10839708	NLRP14	11	7035614	5	0.618304
rs10839708	NLRP14	11	7035614	2	1.375424
rs10839708	NLRP14	11	7035614	3	1.084493
rs10839708	NLRP14	11	7035614	1	1.91211
rs17280682	NLRP14	11	7048145	4	0.367391
rs17280682	NLRP14	11	7048145	3	1.293468
rs17280682	NLRP14	11	7048145	2	0.905476
rs17280682	NLRP14	11	7048145	8	0.030632
rs17280682	NLRP14	11	7048145	5	0.001144
rs17280682	NLRP14	11	7048145	1	0.408412
rs4344466	SYT9	11	7444917	5	0.410292
rs4344466	SYT9	11	7444917	4	2.014573
rs10732509	SYT9	11	7445448	1	0.579528
rs10732509	SYT9	11	7445448	4	0.675618
rs10732510	SYT9	11	7445503	1	2.123441
rs10732510	SYT9	11	7445503	4	0.470734
rs10732510	SYT9	11	7445503	2	0.722026
rs10732510	SYT9	11	7445503	8	0.790181
rs4408288	SYT9	11	7446016	4	0.886528
rs4408288	SYT9	11	7446016	2	3.182616
rs4408288	SYT9	11	7446016	5	1.297877
rs4351800	SYT9	11	7446395	1	5.397428
rs4351800	SYT9	11	7446395	4	0.088685
rs12803046	OLFML1	11	7487561	1	3.102233
rs3884596	OLFML1	11	7488751	3	8.325927
rs3884596	OLFML1	11	7488751	8	2.934689
rs3884596	OLFML1	11	7488751	7	0.381676
rs11041490	PPFIBP2	11	7613385	1	0.650744
rs11041490	PPFIBP2	11	7613385	6	0.409424
rs945888	CYB5R2	11	7651338	3	0.659819
rs4509745	OVCH2	11	7669047	3	1.145745

rs4509745	OVCH2	11	7669047	1	3.12875
rs4509745	OVCH2	11	7669047	4	0.500355
rs4633461	OVCH2	11	7669099	3	0.593223
SNP11-7678550	OVCH2	11	7678550	3	0.901111
rs1482791	OR5P3	11	7803784	5	1.381464
rs1482791	OR5P3	11	7803784	7	0.538887
rs1482791	OR5P3	11	7803784	4	0.636886
rs1482791	OR5P3	11	7803784	6	0.573821
rs4758258	OR10A6	11	7905926	7	0.309195
rs12363522	NLRP10	11	7938670	3	0.426028
rs17847539	TUB	11	8017807	2	1.630009
rs17323959	TUB	11	8081804	4	3.160434
rs4758291	TUB	11	8081870	5	0.97797
rs11041742	TUB	11	8083499	3	0.383331
rs11041742	TUB	11	8083499	8	4.278822
rs11041742	TUB	11	8083499	6	1.043592
rs1055233	TUB	11	8084071	6	0.653225
SNP11-8116419	RIC3	11	8116419	5	0.744386
SNP11-8116419	RIC3	11	8116419	3	0.2233
SNP11-8116433	RIC3	11	8116433	5	0.353327
rs1042359	LMO1	11	8202757	4	0.234241
rs3750952	LMO1	11	8208497	8	0.248225
rs3750952	LMO1	11	8208497	2	0.408056
rs3750952	LMO1	11	8208497	4	0.575774
rs7934876	STK33	11	8370177	7	0.858522
rs7934876	STK33	11	8370177	4	0.034831
rs7934876	STK33	11	8370177	3	0.085833
rs7934876	STK33	11	8370177	2	1.736324
rs35296353	STK33	11	8370805	3	0.719458
rs3751096	STK33	11	8391654	8	4.757671
rs3751096	STK33	11	8391654	2	1.237849
rs3751096	STK33	11	8391654	1	0.245421
rs3751096	STK33	11	8391654	7	0.655153
rs3751096	STK33	11	8391654	4	1.751855
rs1446464	STK33	11	8442886	8	5.097145
rs1446464	STK33	11	8442886	7	0.821686
rs1446464	STK33	11	8442886	1	0.596378
rs1446464	STK33	11	8442886	3	0.924542
rs2289923	STK33	11	8453092	7	1.068995
rs2289923	STK33	11	8453092	1	1.81961
rs2289923	STK33	11	8453092	8	1.735323
rs2289923	STK33	11	8453092	3	0.743044
rs2289921	STK33	11	8453187	8	1.92435
rs2289921	STK33	11	8453187	2	1.725431
rs2289921	STK33	11	8453187	7	0.025528
rs2289921	STK33	11	8453187	1	0.519359
rs2073687	RPL27A	11	8663809	1	4.040846

rs10840112	RPL27A	11	8667087	1	0.599966
rs3812762	ST5	11	8708216	4	0.014835
rs3812762	ST5	11	8708216	6	0.572672
rs3812762	ST5	11	8708216	7	0.395267
rs3812762	ST5	11	8708216	8	7.738828
rs3794153	ST5	11	8708465	6	3.776795
rs3794153	ST5	11	8708465	3	0.027023
rs3794153	ST5	11	8708465	4	0.133543
rs2653601	C11orf16	11	8899518	2	0.072859
rs2653601	C11orf16	11	8899518	4	0.5889
rs2653601	C11orf16	11	8899518	1	0.621014
rs2653601	C11orf16	11	8899518	7	0.032619
rs11042127	C11orf16	11	8904162	7	0.921435
rs11042127	C11orf16	11	8904162	3	0.184472
rs1136966	SCUBE2	11	8998907	7	1.421577
rs1136966	SCUBE2	11	8998907	2	1.908383
rs1136966	SCUBE2	11	8998907	1	1.807559
rs1367	SCUBE2	11	8999145	1	2.865658
rs1367	SCUBE2	11	8999145	7	0.086626
rs3751057	SCUBE2	11	9008051	1	2.465363
rs3751057	SCUBE2	11	9008051	7	0.055575
rs3751055	SCUBE2	11	9025622	3	0.654267
rs3751055	SCUBE2	11	9025622	8	0.62981
GA009535	TMEM41B	11	9292647	1	4.869978
GA009535	TMEM41B	11	9292647	6	3.812778
rs4910052	IPO7	11	9411950	3	0.140625
rs7102464	SBF2	11	9836414	5	0.27148
rs899011	AMPD3	11	10433474	6	1.10347
rs899011	AMPD3	11	10433474	2	1.014579
rs899011	AMPD3	11	10433474	1	0.225493
rs899011	AMPD3	11	10433474	8	3.577665
rs899011	AMPD3	11	10433474	5	1.569621
rs11042836	AMPD3	11	10460312	5	0.284748
rs11042883	MRVI1	11	10554247	4	0.139495
rs11042883	MRVI1	11	10554247	3	2.606328
rs11042883	MRVI1	11	10554247	6	1.091928
rs2241489	MRVI1	11	10604278	8	0.26683
rs2241489	MRVI1	11	10604278	3	0.821643
rs2241489	MRVI1	11	10604278	6	0.74107
rs2278125	MRVI1	11	10606905	7	0.679162
rs2278125	MRVI1	11	10606905	3	1.547627
rs11345	LOC729013	11	10856885	8	8.77733
rs11345	LOC729013	11	10856885	4	0.216755
rs11345	LOC729013	11	10856885	1	3.40103
rs11345	LOC729013	11	10856885	3	0.903917
rs2270511	MICAL2	11	12222118	3	0.976745
rs2270511	MICAL2	11	12222118	6	0.784308

SNP11-12272218	MICALCL	11	12272218	8	3.854443
rs3812754	MICALCL	11	12272965	4	0.025171
rs10831923	TEAD1	11	12917502	1	3.807469
rs10831923	TEAD1	11	12917502	8	0.672256
rs10831923	TEAD1	11	12917502	5	0.098432
rs10831923	TEAD1	11	12917502	4	0.210044
rs16911836	TEAD1	11	12918836	5	8.458844
rs16911836	TEAD1	11	12918836	4	0.076026
rs16911836	TEAD1	11	12918836	2	6.88853
rs1507535	LOC729147	11	13816574	3	0.008164
rs12794714	CYP2R1	11	14870151	7	0.584166
rs12794714	CYP2R1	11	14870151	2	5.801785
rs12794714	CYP2R1	11	14870151	8	0.663292
rs12794714	CYP2R1	11	14870151	5	0.066018
rs12274674	CALCB	11	15056271	8	1.263
rs12274674	CALCB	11	15056271	3	0.155905
rs12274674	CALCB	11	15056271	4	0.249672
rs3928	CALCB	11	15056472	4	2.710559
rs3928	CALCB	11	15056472	3	0.116239
rs3928	CALCB	11	15056472	8	1.626436
rs1054952	INSC	11	15224173	1	0.090512
rs1054952	INSC	11	15224173	7	3.415253
rs1054952	INSC	11	15224173	4	0.156428
rs1054952	INSC	11	15224173	8	0.60952
rs1054952	INSC	11	15224173	2	1.102103
rs1054952	INSC	11	15224173	5	2.641915
rs10832616	AKR1B1P3	11	16462666	1	0.123462
rs1846936	C11orf58	11	16716820	1	0.51648
rs1846936	C11orf58	11	16716820	8	1.547741
rs1846936	C11orf58	11	16716820	2	0.278916
rs217750	PLEKHA7	11	16794364	4	0.097498
rs217750	PLEKHA7	11	16794364	3	0.717589
rs217750	PLEKHA7	11	16794364	8	5.285807
rs214087	NUCB2	11	17254941	7	4.408206
rs214087	NUCB2	11	17254941	6	0.479255
rs10832778	DKFZp686O24166	11	17350649	3	0.042308
rs10832778	DKFZp686O24166	11	17350649	2	4.017969
rs10832778	DKFZp686O24166	11	17350649	8	0.244177
rs10832778	DKFZp686O24166	11	17350649	6	0.025871
rs10832781	DKFZp686O24166	11	17353093	3	0.053375
rs10832781	DKFZp686O24166	11	17353093	6	2.299007
rs10832782	DKFZp686O24166	11	17353849	2	1.891096
rs10832782	DKFZp686O24166	11	17353849	3	0.659811
rs10832782	DKFZp686O24166	11	17353849	7	0.068008
rs2285676	KCNJ11	11	17364601	3	0.402495
rs2285676	KCNJ11	11	17364601	6	1.748121
rs5213	KCNJ11	11	17364980	8	1.134841

rs5213	KCNJ11	11	17364980	2	1.163464
rs5213	KCNJ11	11	17364980	3	1.232631
rs5213	KCNJ11	11	17364980	6	1.345017
rs5215	KCNJ11	11	17365206	2	0.374497
rs5215	KCNJ11	11	17365206	3	1.417716
rs5215	KCNJ11	11	17365206	6	0.659751
rs1800467	KCNJ11	11	17365407	1	4.295188
rs5219	KCNJ11	11	17366148	3	0.557438
rs5219	KCNJ11	11	17366148	6	1.051372
rs757110	ABCC8	11	17375053	2	4.731425
rs757110	ABCC8	11	17375053	6	0.900145
rs757110	ABCC8	11	17375053	3	2.314725
rs1799857	ABCC8	11	17409068	1	0.527045
rs1799857	ABCC8	11	17409068	3	0.633267
rs1799857	ABCC8	11	17409068	5	0.026847
rs1055581	USH1C	11	17472244	7	1.576072
rs1055581	USH1C	11	17472244	5	0.308158
rs1055577	USH1C	11	17472269	3	0.547971
rs1055577	USH1C	11	17472269	5	1.56869
rs1055577	USH1C	11	17472269	7	1.267874
rs1055577	USH1C	11	17472269	1	0.902046
rs1055574	USH1C	11	17472409	3	1.036811
rs1055574	USH1C	11	17472409	2	1.453384
rs1055574	USH1C	11	17472409	1	1.220471
rs1055574	USH1C	11	17472409	7	1.339119
rs1055574	USH1C	11	17472409	5	0.236008
rs1064074	USH1C	11	17476318	4	2.233808
rs1064074	USH1C	11	17476318	2	3.219271
rs1064074	USH1C	11	17476318	8	1.877851
rs11024333	OTOG	11	17571779	7	0.950985
rs875696	OTOG	11	17571825	8	4.380358
rs10832824	OTOG	11	17620020	2	2.463209
rs10832824	OTOG	11	17620020	5	1.773493
SNP11-18065088	SAAL1	11	18065088	7	0.150657
rs11024532	MRGPRX4	11	18151924	8	3.342091
rs11024532	MRGPRX4	11	18151924	6	2.986614
rs7936382	LOC645312	11	18167390	8	5.204717
rs7936382	LOC645312	11	18167390	6	5.1409
rs16935005	LOC645319	11	18175254	5	0.19855
rs4757627	LOC494141	11	18187865	1	5.404602
rs4757627	LOC494141	11	18187865	8	2.363653
rs10741739	HPS5	11	18284260	5	0.37918
rs10741739	HPS5	11	18284260	6	0.801592
rs10741739	HPS5	11	18284260	3	0.922609
rs10741739	HPS5	11	18284260	4	2.761734
rs4150530	HPS5	11	18300270	6	0.293725
SNP11-18590473	SPTY2D1	11	18590473	2	1.89772

rs16935599	SPTY2D1	11	18593057	7	3.480217
SNP11-18694905	IGSF22	11	18694905	8	0.182446
SNP11-18694905	IGSF22	11	18694905	6	3.161385
rs1550870	PTPN5	11	18707617	5	0.172596
rs1550870	PTPN5	11	18707617	8	0.995089
rs7946550	LOC390101	11	18950041	8	0.022603
rs7946550	LOC390101	11	18950041	5	0.28264
rs7126647	MRGPRX2	11	19033193	4	1.360798
rs7126647	MRGPRX2	11	19033193	7	1.962096
rs7126647	MRGPRX2	11	19033193	3	1.082152
rs7126647	MRGPRX2	11	19033193	5	1.777463
rs7126647	MRGPRX2	11	19033193	8	1.549051
rs7126647	MRGPRX2	11	19033193	6	0.59488
rs7112398	MRGPRX2	11	19033234	4	2.041522
rs7112398	MRGPRX2	11	19033234	7	3.287447
rs7112398	MRGPRX2	11	19033234	3	2.27968
rs4757028	NAV2	11	20055688	1	6.010008
rs4757028	NAV2	11	20055688	7	0.345687
rs6483642	NAV2	11	20056117	7	0.049725
rs1443547	SLC6A5	11	20579551	5	3.390584
rs8176789	NELL1	11	21538381	4	0.894587
rs8176789	NELL1	11	21538381	3	0.814264
rs4312063	TMEM16E	11	22199305	8	0.861589
rs4312063	TMEM16E	11	22199305	1	5.194271
rs4312063	TMEM16E	11	22199305	7	0.380956
rs4312063	TMEM16E	11	22199305	5	0.799916
rs7481951	TMEM16E	11	22228446	7	1.265362
rs7481951	TMEM16E	11	22228446	1	5.883161
rs7481951	TMEM16E	11	22228446	5	1.532943
rs7481951	TMEM16E	11	22228446	3	0.538408
rs922571	GAS2	11	22653027	8	2.194843
rs922571	GAS2	11	22653027	2	1.761626
rs922571	GAS2	11	22653027	3	1.353656
rs2302423	LOC645581	11	22825513	2	1.229378
rs3213706	LOC645581	11	22837578	2	0.786855
rs3213706	LOC645581	11	22837578	4	0.678642
rs3213706	LOC645581	11	22837578	5	0.246993
rs10500994	LUZP2	11	25056805	5	1.022968
rs10500994	LUZP2	11	25056805	3	1.196365
rs1531394	TMEM16C	11	26310219	3	0.005094
rs1531394	TMEM16C	11	26310219	4	0.154594
rs7946841	TMEM16C	11	26613139	8	6.124429
rs7946841	TMEM16C	11	26613139	5	0.089149
rs7946841	TMEM16C	11	26613139	2	2.652635
rs3802753	TMEM16C	11	26638963	6	1.64696
rs3802754	TMEM16C	11	26639010	4	0.461726
rs3802754	TMEM16C	11	26639010	6	1.386574

rs3802754	TMEM16C	11	26639010	3	0.652408
rs3802754	TMEM16C	11	26639010	5	0.976748
rs3802755	TMEM16C	11	26639410	4	0.198256
rs3802755	TMEM16C	11	26639410	3	0.515558
rs2305095	BBOX1	11	27033553	8	9.481888
rs10734392	CCDC34	11	27328116	3	0.001245
rs10734392	CCDC34	11	27328116	8	1.254298
rs10734392	CCDC34	11	27328116	1	5.787271
rs10734392	CCDC34	11	27328116	5	0.738859
rs7124442	BDNF	11	27633617	2	0.197017
rs7124442	BDNF	11	27633617	1	0.767806
rs7124442	BDNF	11	27633617	4	0.685389
rs7124442	BDNF	11	27633617	8	0.145176
rs6265	BDNFOS	11	27636492	3	0.660499
rs2726836	HSP90AA2	11	27868222	5	1.828406
rs11821836	LOC645846	11	29704373	3	3.252292
rs506197	FSHB	11	30212399	4	1.857264
rs506197	FSHB	11	30212399	3	1.726159
rs506306	FSHB	11	30212443	3	2.620348
rs676349	FSHB	11	30212558	3	0.796025
rs13642	MPPED2	11	30388796	3	1.248094
rs2616815	DCDC1	11	31283892	4	0.35929
rs2616815	DCDC1	11	31283892	1	3.429065
rs5030317	WT1	11	32366913	3	1.750401
rs5030317	WT1	11	32366913	1	1.777966
rs5030317	WT1	11	32366913	2	0.323069
rs16754	WT1	11	32374521	1	0.487137
rs7946086	WIT1	11	32417666	8	5.448214
rs966191	DEPDC7	11	33009683	4	6.298993
rs966191	DEPDC7	11	33009683	1	6.409074
rs966191	DEPDC7	11	33009683	3	0.834717
rs966191	DEPDC7	11	33009683	8	2.272487
rs12420931	CD59	11	33682725	1	5.100543
rs11032349	CD59	11	33686248	3	0.173802
rs3740616	LMO2	11	33837472	7	0.909628
rs3740616	LMO2	11	33837472	5	0.224968
rs3740616	LMO2	11	33837472	3	7.690727
rs3740617	LMO2	11	33837592	5	0.687243
rs3740617	LMO2	11	33837592	6	0.085028
rs3740617	LMO2	11	33837592	8	3.076751
rs2038602	LMO2	11	33842870	7	0.526594
rs2038602	LMO2	11	33842870	5	0.279726
rs2038602	LMO2	11	33842870	2	1.882614
rs2038602	LMO2	11	33842870	6	0.327932
rs1535719	ELF5	11	34457142	1	5.680827
rs1535719	ELF5	11	34457142	6	5.648575
rs1535719	ELF5	11	34457142	4	0.147317

rs10836251	LOC729710	11	34491707	7	1.231914
rs10836251	LOC729710	11	34491707	1	5.284451
rs10836251	LOC729710	11	34491707	8	0.95105
rs10836251	LOC729710	11	34491707	3	0.156637
rs286925	EHF	11	34599244	7	1.533257
rs286925	EHF	11	34599244	4	0.261556
rs2281910	EHF	11	34624719	3	0.207741
rs2281910	EHF	11	34624719	1	0.676328
rs2281910	EHF	11	34624719	2	2.731853
rs286891	EHF	11	34636976	4	0.954142
rs286891	EHF	11	34636976	7	3.809829
rs286887	EHF	11	34639343	4	1.151766
rs1977420	APIP	11	34866973	3	1.242985
SNP11-3487323	APIP	11	34873233	2	3.453139
SNP11-3487323	APIP	11	34873233	6	1.50735
rs497582	PDHX	11	34948303	1	4.046098
rs1071695	CD44	11	35158418	3	1.020056
rs1467558	CD44	11	35186249	7	0.266037
rs3088168	SLC1A2	11	35229844	5	0.431624
rs3088168	SLC1A2	11	35229844	3	1.784399
rs3088168	SLC1A2	11	35229844	4	0.642733
rs3088168	SLC1A2	11	35229844	2	3.893641
rs1043101	SLC1A2	11	35231405	4	0.03772
rs1043101	SLC1A2	11	35231405	3	0.881209
rs12360706	SLC1A2	11	35232635	3	0.206268
rs3829280	SLC1A2	11	35233299	3	0.199692
rs7936950	SLC1A2	11	35235535	1	2.93175
rs12294045	SLC1A2	11	35235877	1	0.468822
rs10768121	SLC1A2	11	35236232	3	1.030794
rs1042113	SLC1A2	11	35264945	3	0.235579
rs7767	DKFZP586H2123	11	35410295	6	3.723764
rs7767	DKFZP586H2123	11	35410295	3	0.945905
rs7767	DKFZP586H2123	11	35410295	5	1.740384
rs16927500	DKFZP586H2123	11	35448927	2	2.447345
rs597254	TRIM44	11	35785579	1	4.628731
rs597254	TRIM44	11	35785579	6	7.288165
rs597254	TRIM44	11	35785579	5	0.013823
rs597254	TRIM44	11	35785579	4	1.124137
rs2422249	C11orf55	11	36247836	6	1.302586
rs2422249	C11orf55	11	36247836	2	4.232011
rs4756305	C11orf55	11	36248641	1	0.312705
rs4756305	C11orf55	11	36248641	8	0.502584
rs4756305	C11orf55	11	36248641	2	0.07171
rs4756305	C11orf55	11	36248641	6	0.349135
rs7107433	C11orf55	11	36249624	2	3.224941
rs7107433	C11orf55	11	36249624	6	0.681331
rs7107433	C11orf55	11	36249624	3	1.077096

rs3740958	FLJ14213	11	36429376	8	2.812225
rs331439	FLJ14213	11	36441921	6	0.300538
rs331439	FLJ14213	11	36441921	7	2.764344
rs331436	FLJ14213	11	36442640	6	1.276525
rs331436	FLJ14213	11	36442640	3	0.257734
rs331436	FLJ14213	11	36442640	7	3.992675
rs331436	FLJ14213	11	36442640	1	5.355266
rs3740955	RAG1	11	36552176	7	0.755234
rs3740955	RAG1	11	36552176	3	1.201144
rs3740955	RAG1	11	36552176	2	2.097845
rs3740955	RAG1	11	36552176	5	0.709376
rs3740955	RAG1	11	36552176	6	0.759583
rs2227973	RAG1	11	36553889	2	2.66608
rs4151044	RAG1	11	36556540	5	0.196595
rs4151044	RAG1	11	36556540	3	0.019917
rs1056403	RAG1	11	36557746	5	0.087551
rs1056403	RAG1	11	36557746	6	2.910781
rs1056403	RAG1	11	36557746	3	0.167384
rs1056403	RAG1	11	36557746	7	1.063068
rs1056403	RAG1	11	36557746	2	0.918839
rs3740957	C11orf74	11	36572688	5	0.340492
rs3740957	C11orf74	11	36572688	3	0.064925
rs6485450	LOC729798	11	43696998	7	1.687687
rs11555762	HSD17B12	11	43833274	6	2.991419
rs12799548	ALKBH3	11	43859196	1	5.610402
rs2460130	LOC729799	11	43876270	3	1.260579
rs2460130	LOC729799	11	43876270	2	5.348457
rs2460130	LOC729799	11	43876270	7	2.160549
rs2460130	LOC729799	11	43876270	6	4.191957
rs2460130	LOC729799	11	43876270	5	4.156013
rs2460131	LOC729799	11	43876362	3	0.656224
rs2460131	LOC729799	11	43876362	7	3.103609
rs2460131	LOC729799	11	43876362	2	5.410533
rs2460131	LOC729799	11	43876362	6	4.332522
rs2460131	LOC729799	11	43876362	8	10.72282
rs1973717	LOC387763	11	43903543	3	0.742319
SNP11-4404592	ACCS	11	44045928	4	1.176439
rs7950395	PHACS	11	44057694	2	3.591768
rs10458911	EXT2	11	44073678	4	0.483144
rs10458911	EXT2	11	44073678	5	0.080062
rs10458911	EXT2	11	44073678	1	0.405085
rs4755798	ALX4	11	44242752	7	3.583194
rs3802805	ALX4	11	44243142	3	0.653803
rs12419361	ALX4	11	44245647	7	0.855496
rs12802362	TSPAN18	11	44838573	1	5.058187
rs860694	TSPAN18	11	44908193	1	0.086396
rs860694	TSPAN18	11	44908193	3	0.274371

rs860694	TSPAN18	11	44908193	4	0.705394
rs2911	TSPAN18	11	44908407	8	1.366774
rs2911	TSPAN18	11	44908407	4	0.307054
rs836001	TSPAN18	11	44909245	2	3.871986
rs1078931	PRDM11	11	45160424	7	0.425633
rs1078931	PRDM11	11	45160424	6	1.095488
rs1078931	PRDM11	11	45160424	1	3.726369
rs3740784	PRDM11	11	45203146	2	1.653695
rs3740784	PRDM11	11	45203146	3	0.202954
rs3740784	PRDM11	11	45203146	4	2.697547
rs714215	PRDM11	11	45203445	7	0.052886
rs714215	PRDM11	11	45203445	1	1.072847
rs714215	PRDM11	11	45203445	3	0.810294
rs8929	SYT13	11	45218702	3	0.019943
rs2863173	SYT13	11	45218818	3	0.141831
rs2902427	SYT13	11	45218832	3	0.599478
rs2902427	SYT13	11	45218832	5	0.27525
rs2902427	SYT13	11	45218832	2	1.290157
rs6416129	SYT13	11	45220270	5	0.719633
rs4756026	SLC35C1	11	45790536	2	3.188068
rs4756026	SLC35C1	11	45790536	4	6.321761
rs4756026	SLC35C1	11	45790536	6	0.063677
rs7476	CREB3L1	11	46299410	3	0.007807
SNP11-46344444	DGKZ	11	46344444	3	1.431118
SNP11-46344444	DGKZ	11	46344444	5	1.480078
rs8914	ARHGAP1	11	46655700	5	0.76027
rs5898	F2	11	46706224	1	0.558056
rs5898	F2	11	46706224	3	0.335196
rs5898	F2	11	46706224	4	0.549943
rs3816614	LRP4	11	46846741	3	0.140828
rs3816614	LRP4	11	46846741	1	0.253762
rs6485702	LRP4	11	46855347	3	0.393517
rs6485702	LRP4	11	46855347	5	0.832488
rs6485702	LRP4	11	46855347	8	0.71582
rs6485702	LRP4	11	46855347	1	1.033107
SNP11-46872755	LRP4	11	46872755	7	0.540765
rs17787930	C11orf49	11	46914875	4	3.41739
rs17787930	C11orf49	11	46914875	3	0.000519
rs17787930	C11orf49	11	46914875	1	0.742274
rs17787930	C11orf49	11	46914875	2	0.184209
rs3740694	C11orf49	11	47139502	1	0.690663
rs3740694	C11orf49	11	47139502	2	1.080492
rs11039114	C11orf49	11	47142256	6	3.265806
rs11039114	C11orf49	11	47142256	1	0.390112
rs11039114	C11orf49	11	47142256	2	0.369667
rs2167079	ACP2	11	47226831	4	0.837139
rs2167079	ACP2	11	47226831	5	0.054187

rs1449627	MADD	11	47247560	5	0.261329
rs1449627	MADD	11	47247560	4	0.194015
rs3736101	MADD	11	47263204	4	4.93122
SNP11-47263206	MADD	11	47263206	5	2.443293
rs11570058	MYBPC3	11	47326019	5	0.38275
SNP11-47388279	SLC39A13	11	47388279	5	0.162123
SNP11-47388279	SLC39A13	11	47388279	4	0.327128
rs7126210	RAPSN	11	47416882	4	1.00419
rs7111873	RAPSN	11	47426015	4	1.224183
rs7111873	RAPSN	11	47426015	5	0.790667
rs2280231	KBTBD4	11	47557014	1	6.155967
rs2280231	KBTBD4	11	47557014	5	2.960969
rs1064608	MTCH2	11	47597005	8	7.976683
rs1064608	MTCH2	11	47597005	3	0.259035
rs1064608	MTCH2	11	47597005	2	8.830401
rs1064608	MTCH2	11	47597005	4	0.273365
rs1064608	MTCH2	11	47597005	1	0.645246
rs1056387	AGBL2	11	47637946	1	0.590492
rs1056387	AGBL2	11	47637946	2	0.279985
rs1056387	AGBL2	11	47637946	4	8.193455
rs12286721	AGBL2	11	47658104	4	0.193238
rs12286721	AGBL2	11	47658104	1	0.713682
SNP11-47668789	AGBL2	11	47668789	5	4.256341
rs2270993	PTPRJ	11	48101742	8	1.018025
rs4752904	PTPRJ	11	48122843	1	0.635753
rs4752904	PTPRJ	11	48122843	8	4.295466
rs4752904	PTPRJ	11	48122843	3	0.019961
rs4752904	PTPRJ	11	48122843	2	7.667586
rs12790125	OR4C15	11	55079115	1	0.195754
rs557590	OR4C16	11	55096405	3	0.129388
rs557590	OR4C16	11	55096405	2	1.230793
rs11230600	OR4C6	11	55189616	7	0.717087
rs11230600	OR4C6	11	55189616	1	1.798377
rs297055	OR5D14	11	55320476	2	0.570115
rs297055	OR5D14	11	55320476	5	0.679422
rs297055	OR5D14	11	55320476	7	0.742715
rs297055	OR5D14	11	55320476	3	0.447389
rs12790505	OR5L1	11	55336377	5	1.082312
rs10791893	OR5T2	11	55756979	5	0.401193
rs10896290	OR8J1	11	55884657	2	2.700131
rs10896290	OR8J1	11	55884657	7	1.58531
rs10896290	OR8J1	11	55884657	1	3.371258
rs10896290	OR8J1	11	55884657	5	3.965289
rs10896290	OR8J1	11	55884657	3	1.883709
rs12788990	OR8U8	11	55900000	5	0.334773
rs10791961	OR8U8	11	55900010	4	4.685716
rs605734	OR5M3	11	55994298	3	0.358167

rs513873	OR9G4	11	56267199	7	0.367001
rs11820215	LRRC55	11	56712253	3	1.892536
rs1124847	LRRC55	11	56712658	3	0.600908
SNP11-56833396	TNKS1BP1	11	56833396	6	6.139753
SNP11-56837773	TNKS1BP1	11	56837773	4	0.29338
SNP11-56837773	TNKS1BP1	11	56837773	2	4.965244
SNP11-56837773	TNKS1BP1	11	56837773	1	0.090335
SNP11-56837773	TNKS1BP1	11	56837773	3	0.031657
rs2276038	P2RX3	11	56894000	3	0.605519
rs2276038	P2RX3	11	56894000	5	0.06847
SNP11-56902801	PRG3	11	56902801	6	6.652679
SNP11-56902801	PRG3	11	56902801	1	0.917461
rs28362944	SERPING1	11	57122299	6	0.337176
rs4926	SERPING1	11	57138565	4	1.237485
rs1783978	ZDHHC5	11	57220076	6	1.24178
rs1783978	ZDHHC5	11	57220076	1	2.769892
rs10896644	CTNND1	11	57320567	1	2.644661
rs708228	CTNND1	11	57342238	8	5.765987
rs708228	CTNND1	11	57342238	4	0.368419
rs708228	CTNND1	11	57342238	6	6.393664
rs921135	OR6Q1	11	57555299	3	0.613759
rs1966836	OR1S1	11	57738805	7	0.672455
rs11229410	OR5B3	11	57926867	2	2.334175
rs11229410	OR5B3	11	57926867	4	2.204637
rs11229410	OR5B3	11	57926867	1	2.878853
rs11229411	OR5B3	11	57926918	1	3.649054
rs11229411	OR5B3	11	57926918	4	3.304897
rs11229411	OR5B3	11	57926918	2	4.194493
rs4298923	OR5B2	11	57946712	2	4.062878
rs2515362	CNTF	11	58149527	1	0.712448
rs2515362	CNTF	11	58149527	3	0.180171
rs2515362	CNTF	11	58149527	2	0.59181
rs2515362	CNTF	11	58149527	8	7.593804
rs1938717	LOC643589	11	58282640	3	0.069082
rs1938717	LOC643589	11	58282640	5	0.009964
rs11229651	GLYATL2	11	58358885	5	1.633057
rs11229651	GLYATL2	11	58358885	8	3.235779
rs11229651	GLYATL2	11	58358885	3	2.114623
rs1943281	GLYATL2	11	58368525	3	2.053897
rs1943281	GLYATL2	11	58368525	5	0.457165
rs1943281	GLYATL2	11	58368525	2	0.146464
rs1943281	GLYATL2	11	58368525	8	2.193012
rs1943282	GLYATL2	11	58368570	8	1.989147
rs1943282	GLYATL2	11	58368570	5	2.283511
rs1943282	GLYATL2	11	58368570	3	2.665607
rs517039	GLYATL1	11	58480725	4	0.535543
rs517039	GLYATL1	11	58480725	8	1.204924

rs1048696	DTX4	11	58733210	5	4.365342
rs3847	DTX4	11	58733287	2	4.864047
rs3847	DTX4	11	58733287	3	0.542129
rs5029315	DTX4	11	58734344	3	1.62165
rs5029315	DTX4	11	58734344	5	0.442107
rs7926933	MPEG1	11	58735260	5	2.53267
rs7926933	MPEG1	11	58735260	8	1.236906
rs544864	MPEG1	11	58735516	2	0.810421
rs544864	MPEG1	11	58735516	3	0.734505
rs544864	MPEG1	11	58735516	1	0.190733
rs11229895	LOC341112	11	58783477	3	0.057804
rs11229895	LOC341112	11	58783477	1	1.008659
rs7941190	OR5AN1	11	58889374	3	0.411746
rs7941190	OR5AN1	11	58889374	2	0.356636
rs1453541	OR4D6	11	58981797	6	2.345115
rs1453541	OR4D6	11	58981797	3	2.490002
rs1453541	OR4D6	11	58981797	1	2.397426
rs34528912	TCN1	11	59388111	2	3.592474
rs569108	MS4A2	11	59619680	2	2.417438
rs1019670	MS4A6A	11	59697175	3	0.265043
rs12453	MS4A6A	11	59702321	3	1.607426
rs12453	MS4A6A	11	59702321	4	1.395797
rs2304934	MS4A6E	11	59858972	3	0.251083
rs2304933	MS4A6E	11	59859083	3	0.310694
rs950802	MS4A7	11	59909160	1	1.620811
SNP11-6002157	MS4A12	11	60021578	1	1.786686
SNP11-6002157	MS4A12	11	60021578	6	0.536363
SNP11-6002157	MS4A12	11	60021578	5	0.852052
rs6591595	MS4A13	11	60047989	7	1.290618
rs12419635	MS4A10	11	60322497	6	4.126635
rs592637	MS4A10	11	60324731	8	1.751016
rs489172	ZP1	11	60393740	8	0.138179
rs489172	ZP1	11	60393740	5	0.354895
rs489172	ZP1	11	60393740	3	0.84089
rs10897122	ZP1	11	60395037	1	4.136195
rs10897122	ZP1	11	60395037	5	2.166846
rs10897122	ZP1	11	60395037	3	0.055239
rs10897122	ZP1	11	60395037	8	0.587611
rs3763840	PRPF19	11	60422917	3	0.399525
rs3763840	PRPF19	11	60422917	2	1.538564
SNP11-6046035	TMEM132A	11	60460353	3	0.165355
SNP11-6046035	TMEM132A	11	60460353	1	4.369452
SNP11-6046035	TMEM132A	11	60460353	5	0.119231
rs1050922	CD6	11	60541928	5	1.223439
rs1050922	CD6	11	60541928	1	0.844603
rs1050922	CD6	11	60541928	6	4.938635
rs1050922	CD6	11	60541928	2	2.254042

rs2186409	CD5	11	60651068	7	0.836333
rs11230648	VWCE	11	60782706	8	0.173816
rs11230648	VWCE	11	60782706	3	1.11731
rs198430	DAGLA	11	61244266	1	5.850488
rs198430	DAGLA	11	61244266	7	0.765165
rs149803	C11orf9	11	61295596	7	0.057397
rs149803	C11orf9	11	61295596	3	0.699146
rs149803	C11orf9	11	61295596	6	0.203575
rs149803	C11orf9	11	61295596	2	3.219575
rs174538	C11orf10	11	61316657	6	1.217966
rs174538	C11orf10	11	61316657	2	5.556189
rs412334	FEN1	11	61316837	6	1.954026
rs412334	FEN1	11	61316837	3	0.99309
rs174545	FADS1	11	61325882	6	0.935381
rs174545	FADS1	11	61325882	5	0.336546
rs174545	FADS1	11	61325882	2	6.595754
GA009790	FADS2	11	61390523	6	3.345833
rs13966	RAB3IL1	11	61421568	8	4.347717
rs13966	RAB3IL1	11	61421568	3	0.656599
rs174474	RAB3IL1	11	61428841	5	0.804712
rs739789	RAB3IL1	11	61441545	6	0.748893
rs1109748	BEST1	11	61479221	5	0.410645
rs1047738	INCENP	11	61676091	8	5.208643
rs1047738	INCENP	11	61676091	2	4.522167
rs2232942	SCGB1D1	11	61717555	3	0.311229
rs3741245	ASRGL1	11	61862017	4	4.328814
rs4281490	ASRGL1	11	61916502	4	0.496596
rs3741240	SCGB1A1	11	61943118	4	0.695656
rs3888173	TUT1	11	62102707	5	0.127604
rs11553576	EML3	11	62135377	5	0.906236
rs11553576	EML3	11	62135377	8	0.409401
rs2512561	LOC221091	11	62210528	3	0.985163
rs2512561	LOC221091	11	62210528	4	1.959894
SNP11-62214851	BSCL2	11	62214851	6	6.399303
SNP11-62214851	BSCL2	11	62214851	5	0.093004
SNP11-62214851	BSCL2	11	62214851	4	0.655012
SNP11-62214851	BSCL2	11	62214851	1	2.592262
SNP11-62214851	BSCL2	11	62214851	3	0.300916
rs2067477	CHRM1	11	62434882	4	1.141795
rs11568628	SLC22A6	11	62508487	6	4.332405
rs11568628	SLC22A6	11	62508487	4	2.31299
rs11568628	SLC22A6	11	62508487	2	3.540407
rs4963245	MGC34821	11	62643376	3	0.181409
rs1939748	MGC34821	11	62667655	1	0.015245
rs1939748	MGC34821	11	62667655	3	1.220723
rs1790218	SLC22A10	11	62814501	3	0.160946
rs940611	HRASLS5	11	63013017	8	0.858156

rs940611	HRASLS5	11	63013017	6	0.197236
rs3781604	LGALS12	11	63040611	6	2.088556
rs11551944	RTN3	11	63205701	8	1.578383
rs11551944	RTN3	11	63205701	6	5.663688
rs11551942	RTN3	11	63283277	8	7.783292
rs11551942	RTN3	11	63283277	6	9.174203
rs3740637	NAT11	11	63469893	7	0.769798
rs3740637	NAT11	11	63469893	6	2.396064
rs3740637	NAT11	11	63469893	4	2.162206
rs3740637	NAT11	11	63469893	5	2.372192
rs10897465	NAT11	11	63479024	5	0.252638
rs10897465	NAT11	11	63479024	2	5.535333
rs638227	FLRT1	11	63628125	1	0.059404
rs638227	FLRT1	11	63628125	3	0.800766
rs638227	FLRT1	11	63628125	5	0.388253
rs638227	FLRT1	11	63628125	6	0.423909
rs614397	FLRT1	11	63640561	6	4.638441
rs614397	FLRT1	11	63640561	4	0.910335
rs3751120	FLRT1	11	63641029	6	5.80327
rs3751120	FLRT1	11	63641029	4	0.539656
rs3751121	FLRT1	11	63641050	6	0.928874
rs947939	FLRT1	11	63641863	3	0.459715
rs947939	FLRT1	11	63641863	1	1.207436
rs947939	FLRT1	11	63641863	5	0.333086
rs677447	FLRT1	11	63642280	1	1.31614
rs677447	FLRT1	11	63642280	6	0.240063
rs677447	FLRT1	11	63642280	4	3.616736
rs677447	FLRT1	11	63642280	2	1.553134
rs677447	FLRT1	11	63642280	3	0.887152
rs677447	FLRT1	11	63642280	5	0.710639
rs663149	FLRT1	11	63643115	2	8.710609
rs663149	FLRT1	11	63643115	3	0.53399
rs663149	FLRT1	11	63643115	4	0.23923
rs663149	FLRT1	11	63643115	5	3.928681
rs663149	FLRT1	11	63643115	1	0.912319
rs2286612	NUDT22	11	63750806	7	0.177094
rs3741403	DNAJC4	11	63756105	5	0.124767
rs3741403	DNAJC4	11	63756105	1	0.844671
rs3741403	DNAJC4	11	63756105	2	2.438062
rs953778	KCNK4	11	63823575	6	1.338749
SNP11-63865694	CCDC88B	11	63865694	7	0.866372
rs685870	CCDC88B	11	63868504	7	0.978853
rs612448	CCDC88B	11	63881091	7	0.818233
rs600377	CCDC88B	11	63881516	7	0.106243
rs9734313	SLC22A12	11	64114887	5	0.765355
rs9734313	SLC22A12	11	64114887	1	2.337042
rs11231825	SLC22A12	11	64116850	5	4.097795

rs607969	MEN1	11	64332081	4	6.154584
rs10792447	CDC42BPG	11	64348548	8	0.124772
rs10792447	CDC42BPG	11	64348548	5	0.654307
rs10792447	CDC42BPG	11	64348548	2	2.45229
rs10792447	CDC42BPG	11	64348548	4	0.43354
rs10792447	CDC42BPG	11	64348548	7	1.03606
rs3741395	CDC42BPG	11	64354082	6	0.501261
rs3741395	CDC42BPG	11	64354082	4	0.717917
rs3741395	CDC42BPG	11	64354082	7	0.223209
rs3741395	CDC42BPG	11	64354082	5	0.370118
rs3741395	CDC42BPG	11	64354082	8	0.07931
rs7933683	CDC42BPG	11	64358509	7	0.040641
rs7933683	CDC42BPG	11	64358509	4	0.468354
rs7933683	CDC42BPG	11	64358509	2	2.403386
rs7933683	CDC42BPG	11	64358509	6	0.602306
rs7933683	CDC42BPG	11	64358509	5	0.584328
rs1211284	EHD1	11	64402447	7	0.25958
rs1211284	EHD1	11	64402447	6	6.576282
rs673995	GPHA2	11	64458776	3	0.233502
rs673995	GPHA2	11	64458776	1	1.305558
rs542175	SNX15	11	64562846	2	5.553077
rs542175	SNX15	11	64562846	8	0.608954
SNP11-64563811	SNX15	11	64563811	8	0.722179
rs11605377	CDCA5	11	64602146	3	0.119635
rs13807	FAU	11	64645610	6	6.478919
rs3953797	LOC728975	11	64704853	7	0.335762
rs3953797	LOC728975	11	64704853	1	3.874437
SNP11-64880984	TIGD3	11	64880984	4	0.393785
SNP11-64880984	TIGD3	11	64880984	2	4.007596
rs2073800	FRMD8	11	64917705	3	0.774737
rs1144924	TncRNA	11	64960137	5	0.835115
rs12790427	PCNXL3	11	65141303	4	0.049844
rs1193851	PCNXL3	11	65142782	2	2.748621
rs1193851	PCNXL3	11	65142782	7	7.067056
rs1193851	PCNXL3	11	65142782	3	0.731187
rs746429	SIPA1	11	65174010	1	1.365186
rs746429	SIPA1	11	65174010	7	0.647941
rs746429	SIPA1	11	65174010	3	0.751283
rs746429	SIPA1	11	65174010	4	0.26644
rs610037	DKFZp761E198	11	65303433	5	0.652115
rs610037	DKFZp761E198	11	65303433	7	0.893185
rs610037	DKFZp761E198	11	65303433	2	2.966818
rs610037	DKFZp761E198	11	65303433	4	0.827779
rs610037	DKFZp761E198	11	65303433	3	1.069291
rs656040	FLJ30934	11	65377633	8	4.894553
rs656040	FLJ30934	11	65377633	7	0.663628
rs656040	FLJ30934	11	65377633	1	4.244901

rs656040	FLJ30934	11	65377633	3	0.933664
rs13817	MUS81	11	65384910	1	1.777395
rs13817	MUS81	11	65384910	3	0.450843
rs659857	MUS81	11	65386508	7	0.981978
rs659857	MUS81	11	65386508	3	0.990959
rs34891773	MUS81	11	65387937	6	1.766089
rs630303	MUS81	11	65388549	7	1.689727
rs630303	MUS81	11	65388549	3	1.615222
rs630303	MUS81	11	65388549	1	3.069286
rs687672	CTSW	11	65406560	8	2.239454
rs491973	TSGA10IP	11	65483877	6	0.615549
rs491973	TSGA10IP	11	65483877	2	1.494959
rs491973	TSGA10IP	11	65483877	8	0.707365
rs1047464	EIF1AD	11	65526032	2	0.636902
rs1047464	EIF1AD	11	65526032	1	0.578208
rs1203998	CATSPER1	11	65550030	2	4.90674
rs1203998	CATSPER1	11	65550030	3	0.151884
rs1893316	CATSPER1	11	65550373	3	1.060927
GA028131	SF3B2	11	65583280	3	0.546703
GA028131	SF3B2	11	65583280	6	1.473255
GA028131	SF3B2	11	65583280	2	4.44526
rs3741367	CD248	11	65839705	1	0.90772
rs3741367	CD248	11	65839705	4	0.866732
rs3741367	CD248	11	65839705	3	0.06912
rs3741367	CD248	11	65839705	2	1.260097
rs3741368	CD248	11	65840358	3	0.206934
rs11550299	DPP3	11	66010661	4	0.349731
rs11550299	DPP3	11	66010661	8	1.734212
rs2305535	DPP3	11	66028813	8	1.946303
rs2305535	DPP3	11	66028813	4	1.138138
rs17065	DPP3	11	66033611	8	6.629381
rs17065	DPP3	11	66033611	4	6.990897
rs3816492	BBS1	11	66053939	4	0.430657
rs3816492	BBS1	11	66053939	8	5.973736
rs1815739	ACTN3	11	66084671	6	1.73903
rs540874	ACTN3	11	66086308	3	1.319526
rs540874	ACTN3	11	66086308	4	1.110229
rs572846	CTSF	11	66088034	5	1.026689
rs572846	CTSF	11	66088034	3	0.262107
rs1127894	CTSF	11	66092124	6	2.82237
rs1127894	CTSF	11	66092124	4	2.997234
rs1127894	CTSF	11	66092124	5	1.129045
rs1127894	CTSF	11	66092124	3	1.233154
rs486584	CCDC87	11	66114702	8	5.137405
rs486584	CCDC87	11	66114702	4	5.897844
rs486584	CCDC87	11	66114702	3	1.181287
rs486584	CCDC87	11	66114702	6	0.412831

rs486584	CCDC87	11	66114702	5	0.155758
GA028156	CCS	11	66129881	4	0.511946
rs35532855	SPTBN2	11	66214257	3	0.259977
rs623022	SPTBN2	11	66215556	8	6.434323
rs3741194	LRFN4	11	66382810	6	0.87185
rs3741194	LRFN4	11	66382810	5	3.012166
rs3741194	LRFN4	11	66382810	3	0.465226
rs1626067	KIAA1394	11	66949131	5	0.361202
rs1626067	KIAA1394	11	66949131	2	4.438927
rs1626067	KIAA1394	11	66949131	4	1.047214
rs1626067	KIAA1394	11	66949131	3	1.799818
rs8191439	GSTP1	11	67107873	4	0.438554
rs1695	GSTP1	11	67109265	7	0.445498
rs1695	GSTP1	11	67109265	2	1.950525
rs1695	GSTP1	11	67109265	5	0.309005
rs4024254	C11orf72	11	67127342	5	0.958985
rs4024254	C11orf72	11	67127342	6	0.919517
rs12421329	C11orf72	11	67129053	6	0.86233
rs3127	ALDH3B2	11	67187165	3	0.109945
rs3127	ALDH3B2	11	67187165	2	6.790769
rs2286168	ALDH3B1	11	67545714	8	0.524551
rs2286168	ALDH3B1	11	67545714	1	0.541042
rs3751082	ALDH3B1	11	67551929	1	0.529499
rs3751082	ALDH3B1	11	67551929	8	3.056572
rs3751082	ALDH3B1	11	67551929	4	4.402077
rs3751082	ALDH3B1	11	67551929	7	0.063509
GA010046	NDUFS8	11	67560650	2	11.81793
rs36027301	TCIRG1	11	67565844	3	0.265946
rs11481	CHKA	11	67576911	2	5.519087
rs11481	CHKA	11	67576911	6	0.543124
rs11481	CHKA	11	67576911	1	0.081089
rs11481	CHKA	11	67576911	4	0.232851
rs6591334	SUV420H1	11	67681135	3	0.052763
rs2277268	LRP5	11	67930698	4	0.25443
rs17149104	LRP5	11	67949206	5	0.743955
rs3736228	LRP5	11	67957871	3	0.489372
rs3736228	LRP5	11	67957871	6	0.242205
rs11574422	LRP5	11	67970565	5	0.848121
rs1996	SAPS3	11	68139167	7	1.491131
rs1996	SAPS3	11	68139167	4	0.701425
rs1042577	GAL	11	68215046	3	0.294815
rs1042577	GAL	11	68215046	7	0.25491
rs1042577	GAL	11	68215046	6	8.534685
rs2228502	CPT1A	11	68305916	7	0.431816
rs11228356	CPT1A	11	68317363	4	0.8587
rs17610395	CPT1A	11	68318904	4	5.771464
rs17610395	CPT1A	11	68318904	1	4.705216

rs2236654	IGHMBP2	11	68460604	5	7.04324
rs2236654	IGHMBP2	11	68460604	7	0.141461
rs2236654	IGHMBP2	11	68460604	8	9.822748
rs2236654	IGHMBP2	11	68460604	6	4.963927
rs546382	IGHMBP2	11	68460840	5	5.493331
rs546382	IGHMBP2	11	68460840	8	3.16727
rs546382	IGHMBP2	11	68460840	3	0.152317
rs17612126	IGHMBP2	11	68462250	7	1.26494
rs17612126	IGHMBP2	11	68462250	3	0.997776
rs17612126	IGHMBP2	11	68462250	6	0.882081
rs17612126	IGHMBP2	11	68462250	4	1.874526
rs896973	TPCN2	11	68608042	8	0.399685
rs896973	TPCN2	11	68608042	7	0.023131
rs3829241	TPCN2	11	68611939	7	3.85672
rs3829241	TPCN2	11	68611939	8	0.043699
rs3862792	CCND1	11	69172037	6	0.386898
rs3862792	CCND1	11	69172037	1	4.31298
rs948992	FGF19	11	69222519	2	1.57658
rs948992	FGF19	11	69222519	5	0.078441
rs948992	FGF19	11	69222519	4	0.953376
rs948992	FGF19	11	69222519	7	0.683283
rs643301	CTTN	11	69957414	4	0.363283
rs643301	CTTN	11	69957414	7	0.520589
rs643301	CTTN	11	69957414	6	0.339327
rs2664	KRTAP5-9	11	70937891	5	1.126413
rs2663	KRTAP5-9	11	70937944	1	2.492143
rs392565	PDE2A	11	71968277	1	0.806178
rs2306615	CENTD2	11	72092977	3	0.004694
rs2306615	CENTD2	11	72092977	5	0.203379
rs3741152	P2RY6	11	72686562	1	3.795018
rs7115806	P2RY6	11	72687080	1	5.489985
rs12272360	ARHGEF17	11	72697706	8	2.94095
rs10898932	RAB6A	11	73066237	4	9.4089
rs1792174	MRPL48	11	73176581	7	1.520164
GA010135	CHCHD8	11	73265481	4	1.41845
rs2067912	PAAF1	11	73288997	1	5.299846
rs3741138	PAAF1	11	73298185	6	0.585652
rs653263	DNAJB13	11	73348293	1	0.813924
rs2306819	DNAJB13	11	73358738	5	1.032929
rs2306819	DNAJB13	11	73358738	7	0.498735
rs2306819	DNAJB13	11	73358738	1	1.975345
rs647126	UCP3	11	73389668	1	0.303696
rs647126	UCP3	11	73389668	2	1.29928
rs1800006	UCP3	11	73394902	2	1.884997
rs1800006	UCP3	11	73394902	5	1.063716
rs1800006	UCP3	11	73394902	6	1.452201
SNP11-73446201	C2CD3	11	73446201	7	0.080105

rs4453265	C2CD3	11	73462974	1	0.273047
rs4453265	C2CD3	11	73462974	5	0.138173
rs4453265	C2CD3	11	73462974	4	1.985507
rs4453265	C2CD3	11	73462974	7	0.555662
rs826058	C2CD3	11	73474565	1	4.69522
rs826058	C2CD3	11	73474565	4	1.669926
rs826058	C2CD3	11	73474565	7	0.840824
rs11235995	C2CD3	11	73484091	4	3.887882
rs3741133	PPME1	11	73642337	4	0.628694
rs3741133	PPME1	11	73642337	2	4.589447
rs17132882	PPME1	11	73642810	4	1.255633
rs17132882	PPME1	11	73642810	5	7.267884
rs3741132	P4HA3	11	73655891	4	0.046509
rs3741132	P4HA3	11	73655891	2	3.875196
rs8471	PGM2L1	11	73719240	5	0.8019
rs1053021	PGM2L1	11	73722205	7	0.783598
rs1053021	PGM2L1	11	73722205	6	0.807277
rs1053021	PGM2L1	11	73722205	1	4.867454
SNP11-7378681	PGM2L1	11	73786814	4	0.118695
SNP11-7378681	PGM2L1	11	73786814	2	2.779314
rs9516	KCNE3	11	73843926	4	1.29738
rs9516	KCNE3	11	73843926	1	0.318176
rs9516	KCNE3	11	73843926	6	2.397416
rs7789	KCNE3	11	73843968	1	2.870716
rs12271177	KCNE3	11	73845278	4	1.034545
rs12271177	KCNE3	11	73845278	6	1.496485
rs12271177	KCNE3	11	73845278	5	0.380371
rs12271177	KCNE3	11	73845278	1	1.022137
rs1280091	NULL	11	73886874	1	4.924663
rs1280091	NULL	11	73886874	7	1.604467
rs7948433	CHRDL2	11	74097026	6	0.219707
rs2298746	XRRA1	11	74231482	5	1.383941
rs2298746	XRRA1	11	74231482	6	5.388471
rs4944960	XRRA1	11	74237094	5	0.968774
rs4944960	XRRA1	11	74237094	6	5.681388
rs4944961	XRRA1	11	74237129	6	5.734375
rs4944961	XRRA1	11	74237129	5	2.511163
rs2165163	XRRA1	11	74337791	6	0.259695
rs2165163	XRRA1	11	74337791	5	1.681281
rs7115499	NEU3	11	74383250	1	5.156972
rs2851069	SLCO2B1	11	74540004	3	1.593733
rs2851069	SLCO2B1	11	74540004	5	1.411413
rs2851069	SLCO2B1	11	74540004	6	2.050754
rs877711	ARRB1	11	74672000	1	0.242538
rs877711	ARRB1	11	74672000	4	1.100024
rs651581	SERPINH1	11	74955640	1	2.874995
rs651581	SERPINH1	11	74955640	7	1.565077

rs651581	SERPINH1	11	74955640	6	1.032837
rs651581	SERPINH1	11	74955640	8	1.221902
rs3197153	LRRC32	11	76046720	2	1.050943
rs3197153	LRRC32	11	76046720	1	0.792773
rs3197153	LRRC32	11	76046720	3	0.046334
rs3197153	LRRC32	11	76046720	6	0.386205
rs3197153	LRRC32	11	76046720	8	0.398226
rs3197153	LRRC32	11	76046720	5	0.100778
rs3197153	LRRC32	11	76046720	7	0.5701
rs3781701	LRRC32	11	76047067	5	0.513902
rs3781701	LRRC32	11	76047067	6	0.298938
rs3781701	LRRC32	11	76047067	3	0.537568
rs3781701	LRRC32	11	76047067	1	0.936656
rs3781700	LRRC32	11	76047357	6	0.034012
rs3781700	LRRC32	11	76047357	3	0.425571
rs3781700	LRRC32	11	76047357	1	0.722285
rs11236839	LRRC32	11	76047561	3	0.642828
rs3824890	LRRC32	11	76047652	7	0.183189
rs1320646	LRRC32	11	76047695	1	1.177082
rs1320645	LRRC32	11	76047736	7	0.661346
rs1320645	LRRC32	11	76047736	1	0.648406
rs10899257	GUCY2E	11	76092857	5	0.405947
rs11546727	TSKU	11	76184536	2	5.458511
SNP11-7631529	PHCA	11	76315299	1	0.131237
SNP11-7631529	PHCA	11	76315299	5	0.80836
SNP11-7631529	PHCA	11	76315299	2	1.258204
rs3740767	PHCA	11	76379254	1	0.390946
rs3740767	PHCA	11	76379254	5	0.967373
rs3740767	PHCA	11	76379254	2	0.247179
rs2233549	OMP	11	76491758	4	1.960532
rs2277276	CAPN5	11	76513488	1	4.530737
rs2276288	MYO7A	11	76590284	8	0.759982
rs2276288	MYO7A	11	76590284	1	0.463483
rs2276288	MYO7A	11	76590284	2	3.509236
rs2276288	MYO7A	11	76590284	7	6.271622
rs41298757	MYO7A	11	76600516	2	2.322488
rs12363944	GDPD4	11	76605673	3	0.412881
rs2276415	AQP11	11	76978989	4	5.571201
rs2276415	AQP11	11	76978989	5	0.487725
rs588217	C11orf67	11	77261024	1	0.359048
rs588217	C11orf67	11	77261024	6	0.827637
rs588217	C11orf67	11	77261024	3	0.060166
rs11237362	KCTD14	11	77411857	1	3.404686
rs655445	THRSP	11	77456459	5	0.659967
rs655445	THRSP	11	77456459	1	7.351521
rs1263505	ALG8	11	77489638	5	6.464677
rs1263505	ALG8	11	77489638	1	3.142054

rs2510044	USP35	11	77586662	4	0.530282
rs2510044	USP35	11	77586662	1	5.020264
rs2510045	USP35	11	77598211	1	3.583192
rs2510045	USP35	11	77598211	4	1.020062
rs2512527	USP35	11	77598511	1	3.555327
rs2512527	USP35	11	77598511	4	0.473988
SNP11-77598578	USP35	11	77598578	1	4.505023
SNP11-77598578	USP35	11	77598578	4	0.771831
rs2450138	USP35	11	77602518	4	1.827957
rs2450138	USP35	11	77602518	1	4.622886
rs1055250	USP35	11	77603356	1	1.232003
rs1055250	USP35	11	77603356	4	0.353984
rs2248407	GAB2	11	77615448	1	5.390025
rs2248407	GAB2	11	77615448	4	4.815573
rs2292572	GAB2	11	77730512	1	4.531417
rs2292572	GAB2	11	77730512	4	0.123479
rs10501429	NARS2	11	77957438	1	0.291485
rs10501429	NARS2	11	77957438	4	1.589635
rs10501429	NARS2	11	77957438	5	8.458336
rs585747	LOC646112	11	79377793	1	2.085808
rs585747	LOC646112	11	79377793	3	2.407179
rs585747	LOC646112	11	79377793	5	2.246855
rs585747	LOC646112	11	79377793	8	1.930518
rs2291793	MGC33846	11	82121465	3	3.811129
rs2291793	MGC33846	11	82121465	8	0.696259
rs2291793	MGC33846	11	82121465	6	2.968682
rs2291793	MGC33846	11	82121465	7	2.707129
SNP11-82303462	C11orf82	11	82303462	5	0.051296
rs11826199	C11orf82	11	82322412	7	4.41721
rs11826199	C11orf82	11	82322412	6	4.208806
rs11601610	RAB30	11	82370170	4	0.211441
rs12284052	PCF11	11	82557989	6	5.038976
rs557913	LOC729964	11	82598706	4	3.683575
rs557913	LOC729964	11	82598706	8	2.793393
rs557913	LOC729964	11	82598706	6	7.408422
rs646348	CCDC90B	11	82650652	2	1.468188
rs646348	CCDC90B	11	82650652	4	0.844399
rs646348	CCDC90B	11	82650652	5	0.277376
rs646348	CCDC90B	11	82650652	6	0.250237
rs646348	CCDC90B	11	82650652	1	0.180218
rs2512676	DLG2	11	82844368	1	0.692939
rs1991923	CREBZF	11	85046846	4	4.576534
rs1237203	CCDC89	11	85072828	1	3.012892
rs290185	CCDC89	11	85073366	5	0.313307
rs290185	CCDC89	11	85073366	8	5.458645
rs290185	CCDC89	11	85073366	4	2.412308
rs290185	CCDC89	11	85073366	1	5.06026

SNP11-85114000	SYTL2	11	85114000	8	0.018842
SNP11-85114000	SYTL2	11	85114000	4	0.146152
SNP11-85114000	SYTL2	11	85114000	1	5.153602
SNP11-85114000	SYTL2	11	85114000	5	6.498245
SNP11-85114000	SYTL2	11	85114000	6	5.163502
rs17744245	SYTL2	11	85126327	4	0.263647
rs7927222	CCDC83	11	85304756	3	0.513892
rs12805520	CCDC83	11	85308059	1	0.29576
rs7118279	CCDC83	11	85308485	1	0.240122
rs7118279	CCDC83	11	85308485	2	0.559699
rs694353	PICALM	11	85369829	5	0.331319
rs694353	PICALM	11	85369829	3	0.463602
rs974144	EED	11	85646271	3	0.59212
rs974144	EED	11	85646271	6	0.36423
rs11234604	C11orf73	11	85691028	3	0.161243
rs3741005	CCDC81	11	85801204	1	4.153796
rs1056194	CCDC81	11	85811432	3	0.982551
rs1056194	CCDC81	11	85811432	7	5.255199
rs1056194	CCDC81	11	85811432	4	0.727397
rs11234648	CCDC81	11	85811585	3	1.002428
rs12287767	ME3	11	85945362	7	0.737158
rs12287767	ME3	11	85945362	1	4.212014
rs12287767	ME3	11	85945362	3	0.484397
rs10898563	FZD4	11	86336861	8	1.977572
rs10898563	FZD4	11	86336861	5	0.889587
rs3802892	FZD4	11	86338534	3	0.711963
rs3802892	FZD4	11	86338534	2	3.557976
rs3812730	RAB38	11	87548211	3	1.294435
rs3812730	RAB38	11	87548211	6	0.634417
rs11600158	CTSC	11	87710562	5	0.701994
rs16924538	GAPDHL15	11	87782001	3	0.53815
rs16924538	GAPDHL15	11	87782001	4	0.805799
rs16924538	GAPDHL15	11	87782001	7	0.910096
rs16924538	GAPDHL15	11	87782001	5	0.394995
rs1138531	NAALAD2	11	89565060	1	4.494759
rs1138531	NAALAD2	11	89565060	4	0.976916
rs1138531	NAALAD2	11	89565060	7	0.47933
rs1138531	NAALAD2	11	89565060	5	0.269864
rs1061470	NAALAD2	11	89565410	1	0.520968
rs1061470	NAALAD2	11	89565410	2	2.688348
rs1061470	NAALAD2	11	89565410	8	2.099478
rs1061470	NAALAD2	11	89565410	6	0.541762
SNP11-89595854	CHORDC1	11	89595854	3	0.641666
rs1458209	LOC727998	11	91598124	3	0.264048
rs1458209	LOC727998	11	91598124	5	1.308051
rs1458209	LOC727998	11	91598124	8	1.578118
rs1458209	LOC727998	11	91598124	7	0.072591

rs11823754	FAT3	11	92265592	5	1.225205
rs11823754	FAT3	11	92265592	4	1.237345
rs2197678	FAT3	11	92266076	5	0.787832
rs2197678	FAT3	11	92266076	2	0.267307
SNP11-92535568	SLC36A4	11	92535568	5	0.111143
rs3020061	CCDC67	11	92702848	1	0.968345
rs12282288	CCDC67	11	92769132	5	0.874631
rs7108501	HEPHL1	11	93485000	3	1.56103
rs7108501	HEPHL1	11	93485000	1	4.171407
rs7108501	HEPHL1	11	93485000	6	0.114565
rs7108501	HEPHL1	11	93485000	4	2.940235
rs7108501	HEPHL1	11	93485000	8	1.964382
rs7933565	GPR83	11	93751456	1	2.08082
rs7933565	GPR83	11	93751456	3	0.74445
rs7933565	GPR83	11	93751456	4	0.047496
rs2155209	MRE11A	11	93790438	2	1.115959
rs2155209	MRE11A	11	93790438	8	5.761142
rs2155209	MRE11A	11	93790438	1	0.207778
rs2155209	MRE11A	11	93790438	4	0.426906
SNP11-93866600	MRE11A	11	93866600	5	3.658851
SNP11-93866600	MRE11A	11	93866600	6	1.84765
rs647484	LOC643037	11	93900903	8	5.790566
rs10741490	FUT4	11	93921662	5	0.245525
rs7110167	PIWIL4	11	93959885	7	0.359631
rs7110167	PIWIL4	11	93959885	2	0.088963
rs7110167	PIWIL4	11	93959885	1	0.508161
rs7110167	PIWIL4	11	93959885	5	0.292659
rs7119735	PIWIL4	11	93966358	3	0.818455
rs7119735	PIWIL4	11	93966358	2	3.955772
SNP11-93966413	PIWIL4	11	93966413	3	0.124576
SNP11-93966413	PIWIL4	11	93966413	6	1.423484
SNP11-93966413	PIWIL4	11	93966413	1	3.973573
SNP11-93966413	PIWIL4	11	93966413	4	4.427902
rs593690	PIWIL4	11	93976868	6	0.622986
rs593690	PIWIL4	11	93976868	1	0.326575
rs593690	PIWIL4	11	93976868	3	0.027767
rs593690	PIWIL4	11	93976868	4	0.246858
rs593690	PIWIL4	11	93976868	5	0.120449
rs593690	PIWIL4	11	93976868	2	0.503865
rs593690	PIWIL4	11	93976868	7	0.167323
rs3740853	JMJD2D	11	94371407	3	0.619183
rs3740853	JMJD2D	11	94371407	4	1.203863
rs16921265	LOC390245	11	94398486	8	1.958007
rs12275165	CEP57	11	95205488	2	4.673575
rs16922613	MTMR2	11	95205696	5	0.367226
rs7123272	MAML2	11	95352672	5	0.678001
rs11021542	MAML2	11	95715937	5	5.469197

rs543146	FLJ32810	11	100319780	1	0.650903
rs543146	FLJ32810	11	100319780	2	1.132655
rs569336	FLJ32810	11	100352279	4	0.855807
rs569336	FLJ32810	11	100352279	2	3.755015
rs569336	FLJ32810	11	100352279	1	0.011981
rs1047624	TMEM133	11	100368943	5	4.765356
rs17654386	TMEM133	11	100369024	5	0.302715
rs17654410	TMEM133	11	100369356	5	0.773553
rs500760	PGR	11	100415201	3	0.801701
rs1042838	PGR	11	100438622	2	0.004062
rs1042838	PGR	11	100438622	5	0.008911
rs7105709	ANGPTL5	11	101292322	5	0.956992
rs7105709	ANGPTL5	11	101292322	2	4.58918
rs7105709	ANGPTL5	11	101292322	3	1.012625
rs7105709	ANGPTL5	11	101292322	4	0.424621
rs7105709	ANGPTL5	11	101292322	7	0.205334
rs7105709	ANGPTL5	11	101292322	6	0.266203
SNP11-10133421	KIAA1377	11	101334216	8	0.529349
rs11225089	KIAA1377	11	101337800	5	9.68056
rs11225091	KIAA1377	11	101337880	5	0.692274
rs3740930	KIAA1377	11	101337918	3	0.192489
rs11225174	YAP1	11	101607156	5	0.99144
rs11225174	YAP1	11	101607156	6	5.820054
rs484068	TMEM123	11	101773737	1	4.566735
rs484068	TMEM123	11	101773737	5	0.203043
rs484068	TMEM123	11	101773737	3	0.546736
rs485020	TMEM123	11	101773838	1	4.354529
rs485020	TMEM123	11	101773838	3	0.52872
rs1784424	MMP20	11	101982587	4	0.199233
rs1784424	MMP20	11	101982587	1	0.468958
rs1784424	MMP20	11	101982587	5	4.448846
rs1784424	MMP20	11	101982587	7	1.844884
rs1784423	MMP20	11	101982605	6	2.710102
rs1784423	MMP20	11	101982605	4	2.865874
rs1784423	MMP20	11	101982605	2	3.47948
rs1784423	MMP20	11	101982605	7	1.299618
rs1784423	MMP20	11	101982605	1	2.061799
rs1784423	MMP20	11	101982605	5	1.480465
rs2509010	MMP27	11	102067910	7	0.858974
rs2509010	MMP27	11	102067910	1	3.591887
rs2509010	MMP27	11	102067910	8	0.35455
rs470168	MMP10	11	102146592	5	7.90153
rs470168	MMP10	11	102146592	2	1.705229
rs470168	MMP10	11	102146592	6	0.803948
rs470168	MMP10	11	102146592	3	0.812477
rs17359286	MMP10	11	102148928	5	2.653793
rs17293607	MMP10	11	102155599	7	0.640709

rs17435959	MMP10	11	102156523	6	1.754824
rs5854	MMP1	11	102166084	2	1.307956
rs5854	MMP1	11	102166084	6	1.063095
rs5854	MMP1	11	102166084	3	0.754866
rs2239008	MMP1	11	102166290	8	4.580434
rs2239008	MMP1	11	102166290	3	1.476276
rs470558	MMP1	11	102171526	4	0.299753
rs10488	MMP1	11	102173232	4	0.616695
rs10488	MMP1	11	102173232	6	0.80473
rs10488	MMP1	11	102173232	7	2.239534
rs2510091	DCUN1D5	11	102468042	7	0.624193
rs17301028	DYNC2H1	11	102485534	2	8.224899
rs12146610	DYNC2H1	11	102493714	8	0.750421
rs1053861	PDGFD	11	103283364	4	0.748543
rs1053861	PDGFD	11	103283364	8	4.34672
rs1053861	PDGFD	11	103283364	7	0.526176
rs11226057	PDGFD	11	103284235	7	1.351129
rs11226057	PDGFD	11	103284235	8	0.253456
rs10895534	PDGFD	11	103285003	4	3.928059
rs10895534	PDGFD	11	103285003	8	0.475306
rs1056205	PDGFD	11	103285305	8	4.716636
rs35045740	PDGFD	11	103323605	4	5.738715
rs2279789	DDI1	11	103414178	3	2.450433
rs2279789	DDI1	11	103414178	1	1.778611
rs2279789	DDI1	11	103414178	5	1.670865
rs523104	CASP5	11	104374918	1	0.127372
rs523104	CASP5	11	104374918	2	0.872595
rs523104	CASP5	11	104374918	5	0.749214
rs523104	CASP5	11	104374918	3	0.248858
rs507879	CASP5	11	104383137	3	1.12145
rs10895893	KIAA1826	11	105397209	7	1.077171
rs10895893	KIAA1826	11	105397209	5	0.643873
rs10895893	KIAA1826	11	105397209	6	3.806563
rs2236663	KIAA1826	11	105397361	5	0.755333
rs2236663	KIAA1826	11	105397361	6	1.113803
rs2236663	KIAA1826	11	105397361	7	2.252743
rs17093811	KBTBD3	11	105427145	6	0.34741
rs4754188	LOC341230	11	106553206	7	0.876283
rs4754188	LOC341230	11	106553206	5	0.717861
rs1046095	CWF19L2	11	106702445	1	4.302009
rs1046095	CWF19L2	11	106702445	5	0.553049
rs1046095	CWF19L2	11	106702445	7	1.504731
rs1046095	CWF19L2	11	106702445	8	5.133224
rs1046095	CWF19L2	11	106702445	4	0.608668
rs512574	CWF19L2	11	106702529	1	4.45628
rs630693	CWF19L2	11	106702816	4	0.898076
rs630693	CWF19L2	11	106702816	5	0.199275

rs630693	CWF19L2	11	106702816	2	5.634277
rs630693	CWF19L2	11	106702816	8	6.393379
SNP11-10680483	CWF19L2	11	106804833	6	0.032916
rs583362	SLN	11	107083931	8	0.591159
rs583362	SLN	11	107083931	2	0.054904
rs583362	SLN	11	107083931	5	0.189296
rs518073	SLC35F2	11	107168051	2	2.469736
rs518073	SLC35F2	11	107168051	7	0.142032
rs518073	SLC35F2	11	107168051	8	8.188636
rs518073	SLC35F2	11	107168051	5	0.330691
rs516091	SLC35F2	11	107168313	6	1.417726
rs516091	SLC35F2	11	107168313	8	0.930999
rs516091	SLC35F2	11	107168313	3	0.305935
rs516091	SLC35F2	11	107168313	5	0.387936
rs516091	SLC35F2	11	107168313	2	10.27461
rs516091	SLC35F2	11	107168313	7	0.090645
rs3741065	SLC35F2	11	107234771	5	0.854508
rs3741065	SLC35F2	11	107234771	8	1.716172
rs3741065	SLC35F2	11	107234771	1	1.378671
rs3741065	SLC35F2	11	107234771	3	1.346898
rs2442683	SLC35F2	11	107234825	5	2.438372
rs2442683	SLC35F2	11	107234825	3	1.186562
rs2442683	SLC35F2	11	107234825	1	0.913171
rs2442683	SLC35F2	11	107234825	7	0.004854
rs2442683	SLC35F2	11	107234825	8	0.856735
rs4754282	RAB39	11	107338755	3	1.968358
rs7117111	CUL5	11	107422297	5	0.189844
rs4144901	NPAT	11	107549301	6	0.227885
rs4144901	NPAT	11	107549301	7	0.180973
rs4144901	NPAT	11	107549301	8	0.135139
rs609261	ATM	11	107663344	2	2.590494
rs609261	ATM	11	107663344	7	0.759204
rs609261	ATM	11	107663344	5	0.348667
rs609261	ATM	11	107663344	6	0.709891
rs664143	C11orf65	11	107730871	7	1.146977
rs664143	C11orf65	11	107730871	8	4.739764
rs664143	C11orf65	11	107730871	6	1.791475
rs664143	C11orf65	11	107730871	5	0.764801
rs664143	C11orf65	11	107730871	2	4.947237
rs2640785	EXPH5	11	107914994	6	3.479448
rs2640785	EXPH5	11	107914994	2	3.348097
rs2640785	EXPH5	11	107914994	5	0.423516
rs7936226	ARHGAP20	11	109959548	7	0.991403
rs11213821	FLJ45803	11	110669861	1	0.321635
rs11213821	FLJ45803	11	110669861	6	0.228432
rs11213821	FLJ45803	11	110669861	2	1.490986
rs11213821	FLJ45803	11	110669861	3	0.589085

rs6589218	FLJ45803	11	110672767	4	0.266782
rs6589218	FLJ45803	11	110672767	1	0.852497
rs10244	FLJ45803	11	110674320	5	0.172832
rs1046872	LOC120376	11	110684600	1	5.330492
rs1046872	LOC120376	11	110684600	3	0.171748
rs1046872	LOC120376	11	110684600	7	0.975507
rs1046872	LOC120376	11	110684600	6	0.699501
rs1046872	LOC120376	11	110684600	4	5.224811
rs1042752	POU2AF1	11	110728321	7	0.264978
rs1042752	POU2AF1	11	110728321	1	0.70544
rs1042752	POU2AF1	11	110728321	2	0.796441
rs542275	LAYN	11	110936824	8	0.925164
rs542275	LAYN	11	110936824	7	0.83422
rs542275	LAYN	11	110936824	1	1.0785
rs647080	PPP2R1B	11	111114384	1	5.889512
rs647080	PPP2R1B	11	111114384	4	3.496936
rs10502151	ALG9	11	111229343	5	0.095017
rs10502151	ALG9	11	111229343	8	2.983204
rs10502151	ALG9	11	111229343	4	0.597826
rs4252588	HSPB2	11	111289439	4	4.472651
rs4252590	HSPB2	11	111290026	1	4.140631
rs11214077	SDHD	11	111463887	1	1.976582
rs360712	TEX12	11	111545237	1	0.071547
rs360712	TEX12	11	111545237	2	0.727935
rs360712	TEX12	11	111545237	5	0.004026
rs2564872	C11orf34	11	111628305	5	4.065214
rs2564872	C11orf34	11	111628305	1	5.100149
rs2564872	C11orf34	11	111628305	4	1.137281
rs2518369	LOC399950	11	111644045	5	1.585574
rs2518369	LOC399950	11	111644045	1	2.41385
rs2518369	LOC399950	11	111644045	6	0.794146
rs584427	NCAM1	11	112609206	7	0.180748
rs584427	NCAM1	11	112609206	8	2.161981
rs584427	NCAM1	11	112609206	6	0.341967
rs584427	NCAM1	11	112609206	2	2.208604
rs584427	NCAM1	11	112609206	3	0.743642
rs635903	NCAM1	11	112651995	3	0.934132
rs635903	NCAM1	11	112651995	8	0.46927
rs17115439	ANKK1	11	112769482	7	0.403164
rs17115439	ANKK1	11	112769482	3	0.857964
rs17115439	ANKK1	11	112769482	5	0.954953
rs17115439	ANKK1	11	112769482	2	3.517488
rs4938013	ANKK1	11	112769680	3	1.150701
rs4938013	ANKK1	11	112769680	7	0.362817
rs4938013	ANKK1	11	112769680	2	3.132026
rs7118900	ANKK1	11	112772031	7	0.012533
rs7118900	ANKK1	11	112772031	3	0.238539

rs4938016	ANKK1	11	112775225	5	0.066928
rs2734849	ANKK1	11	112775370	7	0.328685
rs2734849	ANKK1	11	112775370	5	0.322701
rs2734849	ANKK1	11	112775370	3	0.587081
rs2734849	ANKK1	11	112775370	4	0.566653
rs6278	DRD2	11	112785934	2	4.386745
rs6278	DRD2	11	112785934	3	0.754757
rs6278	DRD2	11	112785934	7	0.587044
rs6279	DRD2	11	112786283	4	0.486801
rs6279	DRD2	11	112786283	5	0.46296
rs6277	DRD2	11	112788669	2	2.263823
rs6277	DRD2	11	112788669	3	0.705914
rs6277	DRD2	11	112788669	4	0.006368
rs6277	DRD2	11	112788669	5	1.00699
rs6277	DRD2	11	112788669	7	0.746082
rs1800499	DRD2	11	112792904	8	3.159615
rs17115826	TMPRSS5	11	113064055	5	6.601131
rs17115841	TMPRSS5	11	113070532	5	1.236249
rs11601425	TMPRSS5	11	113075595	4	0.317065
rs11601425	TMPRSS5	11	113075595	3	2.422745
rs3802854	TMPRSS5	11	113076062	2	0.30754
rs3802854	TMPRSS5	11	113076062	1	0.274953
rs3802854	TMPRSS5	11	113076062	5	1.613693
rs3802854	TMPRSS5	11	113076062	3	0.742734
rs1713676	LOC390251	11	113165786	2	1.744417
rs1713676	LOC390251	11	113165786	7	2.365254
rs1713676	LOC390251	11	113165786	1	2.681814
rs1633550	USP28	11	113174415	4	0.41443
rs1633550	USP28	11	113174415	3	0.006817
rs1062613	HTR3A	11	113351216	1	2.129049
rs1062613	HTR3A	11	113351216	4	0.134032
rs17629751	C11orf71	11	113776432	5	1.170337
rs17629751	C11orf71	11	113776432	3	1.327701
rs7944960	FAM55A	11	113906821	1	1.025386
rs7944960	FAM55A	11	113906821	8	1.499997
rs10891705	FAM55D	11	113947153	1	3.024857
rs11215158	FAM55B	11	114074152	6	2.234575
rs1712838	FAM55B	11	114074264	6	1.346838
rs1712838	FAM55B	11	114074264	2	2.432236
rs11820589	BUD13	11	116139072	4	0.50531
rs11820589	BUD13	11	116139072	2	4.882337
SNP11-11616081	ZNF259	11	116160810	4	0.433331
rs619054	APOA5	11	116166023	1	0.242092
rs619054	APOA5	11	116166023	3	0.080717
rs12225230	KIAA0999	11	116233840	6	7.745255
rs12225230	KIAA0999	11	116233840	1	6.33339
rs2305830	CEP164	11	116771522	2	4.48342

rs2305830	CEP164	11	116771522	4	0.696033
rs2305830	CEP164	11	116771522	7	0.34243
rs521099	CEP164	11	116788189	3	0.208527
rs3741283	DSCAML1	11	116804249	5	1.908078
rs35503235	DSCAML1	11	116897320	5	0.933407
rs11999	FXYD2	11	117196128	3	0.873266
rs11999	FXYD2	11	117196128	4	0.417433
rs11999	FXYD2	11	117196128	1	2.214332
rs869789	FXYD2	11	117196596	1	1.785699
rs869789	FXYD2	11	117196596	5	0.76032
rs7117314	FXYD2	11	117203972	6	0.702589
rs7117314	FXYD2	11	117203972	7	0.422375
rs7117314	FXYD2	11	117203972	8	1.548554
rs520333	FXYD6	11	117213835	7	1.395826
rs520333	FXYD6	11	117213835	8	4.584275
rs520333	FXYD6	11	117213835	2	4.646197
rs520333	FXYD6	11	117213835	6	0.271752
rs520333	FXYD6	11	117213835	4	5.46994
rs520333	FXYD6	11	117213835	5	1.198481
rs520333	FXYD6	11	117213835	3	0.316428
rs575535	TMPRSS13	11	117277439	5	0.869458
rs575535	TMPRSS13	11	117277439	3	0.857826
rs575535	TMPRSS13	11	117277439	6	6.943379
rs575535	TMPRSS13	11	117277439	7	3.370086
rs491822	TMPRSS13	11	117278058	6	3.697568
rs491822	TMPRSS13	11	117278058	7	0.002664
rs491822	TMPRSS13	11	117278058	3	0.891308
rs10892196	TMPRSS13	11	117305293	5	4.597755
rs10892196	TMPRSS13	11	117305293	4	0.388721
rs10892196	TMPRSS13	11	117305293	3	1.54384
rs2256111	IL10RA	11	117369257	5	0.590878
rs2256111	IL10RA	11	117369257	6	1.446544
rs3135932	IL10RA	11	117369273	8	1.385113
rs3135932	IL10RA	11	117369273	5	1.296379
rs2228054	IL10RA	11	117369323	2	2.72766
rs2228054	IL10RA	11	117369323	3	1.674513
rs17121493	IL10RA	11	117370056	3	0.302092
rs2229114	IL10RA	11	117375088	5	0.625835
rs9610	IL10RA	11	117377296	8	0.329806
rs9610	IL10RA	11	117377296	5	1.416606
rs7123209	TMPRSS4	11	117493864	4	1.243704
rs7123209	TMPRSS4	11	117493864	7	0.546108
rs1814964	SCN4B	11	117512167	3	1.68301
rs1814964	SCN4B	11	117512167	7	0.324723
rs1814964	SCN4B	11	117512167	2	2.128958
rs625464	SCN2B	11	117538755	7	1.189981
rs625464	SCN2B	11	117538755	1	0.441994

rs625464	SCN2B	11	117538755	4	3.361331
rs625464	SCN2B	11	117538755	8	1.691382
rs11216788	SCN2B	11	117539411	7	1.511763
rs11216788	SCN2B	11	117539411	4	0.508666
rs11216788	SCN2B	11	117539411	1	0.170732
rs11216788	SCN2B	11	117539411	8	0.371043
rs11216788	SCN2B	11	117539411	5	1.078933
rs688911	SCN2B	11	117541742	7	0.729777
rs688911	SCN2B	11	117541742	8	0.118775
rs676685	SCN2B	11	117542152	7	0.738321
rs676685	SCN2B	11	117542152	5	3.106035
rs676685	SCN2B	11	117542152	4	1.715441
rs676685	SCN2B	11	117542152	1	2.38218
rs676685	SCN2B	11	117542152	8	1.994284
rs2298831	AMICA1	11	117573962	6	1.269362
rs17121881	AMICA1	11	117586555	2	1.170259
rs17121881	AMICA1	11	117586555	6	0.487492
rs17121881	AMICA1	11	117586555	4	0.538654
rs17121881	AMICA1	11	117586555	8	0.012011
rs2853006	MPZL3	11	117616187	3	1.000028
rs2853006	MPZL3	11	117616187	8	3.243188
rs2853006	MPZL3	11	117616187	4	0.682964
rs9332871	MLL	11	117899066	1	5.150269
rs12289253	TMEM25	11	117910278	6	2.36955
rs12289253	TMEM25	11	117910278	4	0.171345
rs3741324	TMEM25	11	117911045	6	0.85508
rs3741324	TMEM25	11	117911045	2	3.656727
rs2277291	C11orf60	11	117940966	4	0.22931
rs2277291	C11orf60	11	117940966	6	1.159089
rs10892245	ARCN1	11	117977564	4	0.877467
rs10892245	ARCN1	11	117977564	2	3.335381
GA028457	ARCN1	11	117978212	5	0.387328
rs498872	PHLDB1	11	117982577	6	0.739042
rs498872	PHLDB1	11	117982577	3	0.865995
rs10892246	LOC729024	11	117986641	3	0.44812
rs35435609	PHLDB1	11	118007382	2	0.732322
rs483598	PHLDB1	11	118014878	5	2.404009
rs483598	PHLDB1	11	118014878	4	1.513565
rs483598	PHLDB1	11	118014878	6	0.348697
rs483598	PHLDB1	11	118014878	8	0.336529
rs483598	PHLDB1	11	118014878	7	0.68438
rs483598	PHLDB1	11	118014878	1	4.46919
rs7928371	TREH	11	118034337	3	2.240631
rs7928371	TREH	11	118034337	4	0.730308
SNP11-11803582	TREH	11	118035821	4	3.023703
SNP11-11803582	TREH	11	118035821	7	2.261808
SNP11-11803582	TREH	11	118035821	3	2.571458

rs598207	BLR1	11	118270477	5	0.83117
rs598207	BLR1	11	118270477	4	0.003977
rs598207	BLR1	11	118270477	2	0.936157
rs598207	BLR1	11	118270477	3	0.741757
rs598207	BLR1	11	118270477	7	0.58119
rs3922	BLR1	11	118270810	1	0.160343
rs3922	BLR1	11	118270810	5	1.917351
rs3922	BLR1	11	118270810	3	0.496396
rs3922	BLR1	11	118270810	7	0.168458
rs676925	BLR1	11	118270898	8	0.568664
rs676925	BLR1	11	118270898	3	0.151314
rs676925	BLR1	11	118270898	7	1.067
rs676925	BLR1	11	118270898	5	0.131725
rs676925	BLR1	11	118270898	1	0.989535
rs676925	BLR1	11	118270898	4	0.142945
rs2846281	SLC37A4	11	118406376	1	0.089199
rs2846281	SLC37A4	11	118406376	5	1.923104
rs13929	HYOU1	11	118420965	7	6.461143
rs13929	HYOU1	11	118420965	1	5.526747
rs13929	HYOU1	11	118420965	4	0.612308
rs1784302	VPS11	11	118446167	7	0.156848
rs1784302	VPS11	11	118446167	1	0.655087
rs1784302	VPS11	11	118446167	4	0.678751
rs1784302	VPS11	11	118446167	5	0.138548
rs540261	VPS11	11	118452844	5	5.53523
rs540261	VPS11	11	118452844	7	1.160846
rs540261	VPS11	11	118452844	1	5.151719
rs540261	VPS11	11	118452844	4	0.368531
rs15818	VPS11	11	118457383	1	0.526777
rs15818	VPS11	11	118457383	7	1.054223
rs15818	VPS11	11	118457383	4	1.049786
rs15818	VPS11	11	118457383	2	1.903214
rs643423	NLRX1	11	118548866	4	0.858215
rs643423	NLRX1	11	118548866	2	2.238454
rs1815811	PDZD3	11	118564614	3	0.364084
rs11604328	CBL	11	118679786	1	4.638683
rs11604328	CBL	11	118679786	3	0.398928
rs11604328	CBL	11	118679786	7	0.030945
rs1052121	CBL	11	118683260	5	4.599022
rs1052121	CBL	11	118683260	3	0.027874
rs1052121	CBL	11	118683260	7	0.20722
rs1052121	CBL	11	118683260	1	3.010401
SNP11-11869088	MCAM	11	118690887	5	0.373134
SNP11-11869088	MCAM	11	118690887	4	4.610629
rs6649	RNF26	11	118713138	2	4.267071
rs587985	USP2	11	118749305	1	4.006624
rs587985	USP2	11	118749305	5	0.506481

rs12280992	LOC390255	11	119197667	3	0.491875
rs542903	LOC390255	11	119197873	4	3.830405
rs542903	LOC390255	11	119197873	6	3.601638
rs11604169	TRIM29	11	119491329	4	0.911201
rs4936509	LOC729173	11	119546654	5	1.199798
rs4936509	LOC729173	11	119546654	6	0.164724
rs4936509	LOC729173	11	119546654	3	1.015913
SNP11-11960486	OAF	11	119604868	3	5.151904
rs2305013	ARHGEF12	11	119845270	3	0.091276
rs2230297	GRIK4	11	120251084	8	0.356995
rs35380371	TBCEL	11	120434361	4	4.933968
rs35507522	TECTA	11	120478640	4	3.621932
rs612969	TECTA	11	120494545	8	0.41273
rs612969	TECTA	11	120494545	6	0.387618
rs612969	TECTA	11	120494545	7	0.93892
rs536069	TECTA	11	120501502	7	0.226185
rs10502247	TECTA	11	120504152	4	0.18138
rs520805	TECTA	11	120505984	8	1.012197
rs586473	TECTA	11	120505994	4	0.177316
rs586473	TECTA	11	120505994	7	1.13173
rs2155369	TECTA	11	120544020	3	1.340311
rs4936621	LOC283155	11	120738021	3	2.777407
rs4936621	LOC283155	11	120738021	4	2.775009
rs2298813	SORL1	11	120898894	1	6.837737
rs2298813	SORL1	11	120898894	4	5.196343
rs2070045	SORL1	11	120953300	3	0.632413
rs7127978	UBASH3B	11	122031711	2	1.445332
rs7127978	UBASH3B	11	122031711	3	0.184065
rs7127978	UBASH3B	11	122031711	5	0.960889
rs7127978	UBASH3B	11	122031711	6	3.567114
rs872414	UBASH3B	11	122170647	3	0.135938
rs872414	UBASH3B	11	122170647	7	0.038331
rs1996841	UBASH3B	11	122187321	3	0.117722
rs11218828	UBASH3B	11	122189565	3	0.353604
rs1057704	C11orf63	11	122335484	7	1.351025
rs1057704	C11orf63	11	122335484	3	0.370472
rs1057704	C11orf63	11	122335484	6	0.303844
rs1057704	C11orf63	11	122335484	1	0.795734
rs1057704	C11orf63	11	122335484	2	0.110949
rs1057704	C11orf63	11	122335484	5	2.552134
rs7933723	LOC341056	11	122393780	1	4.574534
rs7933723	LOC341056	11	122393780	4	1.621951
rs7933723	LOC341056	11	122393780	6	3.664544
rs7933723	LOC341056	11	122393780	7	4.191927
rs7933723	LOC341056	11	122393780	2	4.458663
rs2276075	HSPA8	11	122438012	8	4.991063
rs2276075	HSPA8	11	122438012	1	6.257876

rs2276077	HSPA8	11	122438027	8	8.830704
rs2276077	HSPA8	11	122438027	1	4.205683
rs2027767	SCN3B	11	123005969	3	0.192725
rs2027767	SCN3B	11	123005969	1	2.674699
rs2027767	SCN3B	11	123005969	4	0.400178
rs10400374	SCN3B	11	123007971	8	0.734732
rs1275085	SCN3B	11	123018371	5	1.851372
rs11825443	ZNF202	11	123105107	7	0.600734
rs7952150	OR6M1	11	123181623	7	0.574538
rs7952150	OR6M1	11	123181623	3	0.474112
rs10790610	OR8D4	11	123282946	4	2.102339
rs17759447	OR10S1	11	123353274	5	0.375672
rs17686210	OR10S1	11	123353331	8	1.877913
rs9326269	LOH11CR2A	11	123521950	7	5.694364
rs9326269	LOH11CR2A	11	123521950	2	2.774271
rs9326269	LOH11CR2A	11	123521950	3	0.240782
rs3741107	LOH11CR2A	11	123522127	1	3.85883
rs3741107	LOH11CR2A	11	123522127	3	0.158033
rs2512167	OR8G5	11	123640648	3	1.071347
rs2512167	OR8G5	11	123640648	5	2.122215
rs4936919	OR8D1	11	123685292	3	0.567899
rs4057750	OR8B4	11	123799587	4	3.08218
SNP11-12403587	SIAE	11	124035874	2	4.727011
SNP11-12403587	SIAE	11	124035874	4	5.762231
SNP11-12403587	SIAE	11	124035874	3	0.286475
SNP11-12403587	SIAE	11	124035874	8	4.59568
rs12541	ESAM	11	124128702	4	5.225204
rs12792040	ESAM	11	124129360	1	4.543924
rs12792040	ESAM	11	124129360	7	0.069957
rs7933204	ROBO3	11	124253047	4	0.416306
rs12823	ROBO4	11	124259821	1	4.225185
SNP11-12427227	ROBO4	11	124272277	8	0.810812
rs3802904	HEPN1	11	124294965	4	2.155521
rs3802904	HEPN1	11	124294965	2	3.583153
rs3802904	HEPN1	11	124294965	6	0.090976
rs3802904	HEPN1	11	124294965	5	0.479334
rs10790716	HEPACAM	11	124298926	3	1.057339
rs3809054	CCDC15	11	124329276	7	0.389257
rs3809054	CCDC15	11	124329276	5	0.001345
rs3809054	CCDC15	11	124329276	6	0.140243
rs3809054	CCDC15	11	124329276	1	3.587739
rs3809054	CCDC15	11	124329276	3	1.030095
SNP11-12445260	SLC37A2	11	124452606	8	9.608348
SNP11-12445260	SLC37A2	11	124452606	1	3.58627
SNP11-12445260	SLC37A2	11	124452606	3	0.49393
rs7110840	SLC37A2	11	124463536	3	0.399864
rs7110840	SLC37A2	11	124463536	5	1.203482

rs7110840	SLC37A2	11	124463536	7	0.145196
rs3802809	SLC37A2	11	124463607	3	0.683543
rs3802809	SLC37A2	11	124463607	5	1.203842
rs3802809	SLC37A2	11	124463607	7	0.768086
rs4936978	SLC37A2	11	124464124	7	0.8911
rs4936978	SLC37A2	11	124464124	3	0.77706
rs4936978	SLC37A2	11	124464124	5	0.492915
rs7108003	LOC219854	11	124471683	5	1.122818
rs7108003	LOC219854	11	124471683	7	0.41466
rs7108003	LOC219854	11	124471683	3	0.354522
rs2155666	LOC219854	11	124477901	3	0.75255
rs2155666	LOC219854	11	124477901	7	1.155119
rs2155666	LOC219854	11	124477901	6	0.595709
rs2155666	LOC219854	11	124477901	5	0.557397
rs7118508	LOC219854	11	124478042	5	2.141069
rs7118508	LOC219854	11	124478042	8	0.364904
rs7118508	LOC219854	11	124478042	7	1.320127
rs645172	PKNOX2	11	124807932	7	0.752264
rs645172	PKNOX2	11	124807932	3	0.271707
rs531312	FEZ1	11	124863800	6	0.70833
rs2241502	STT3A	11	124984573	4	0.448418
rs506504	CHEK1	11	125030405	6	0.218066
rs2114084	PATE	11	125122820	4	0.266827
rs2114084	PATE	11	125122820	5	0.122451
rs2114084	PATE	11	125122820	3	1.052101
rs2114084	PATE	11	125122820	1	0.383072
rs1054997	PATE	11	125124023	6	1.632307
rs1056417	PATE	11	125124788	5	0.630758
rs1056417	PATE	11	125124788	6	2.691816
rs1056417	PATE	11	125124788	1	0.757693
rs4937035	C11orf38	11	125151433	1	0.810807
rs484670	C11orf38	11	125151496	5	0.15578
rs484670	C11orf38	11	125151496	4	1.341001
rs484670	C11orf38	11	125151496	1	0.773642
rs12224646	FLJ41047	11	125213503	2	0.966876
rs12224646	FLJ41047	11	125213503	1	0.665322
rs10893435	FLJ41047	11	125213995	4	0.331385
rs11220237	FLJ41047	11	125214047	4	1.382327
rs7110718	FLJ41047	11	125214220	3	0.031945
rs3862621	FLJ41047	11	125214437	3	0.835736
rs3851106	FLJ41047	11	125214634	7	1.141885
rs3851106	FLJ41047	11	125214634	3	1.501171
SNP11-12527073	PUS3	11	125270738	2	8.103459
rs600761	PUS3	11	125271414	7	4.980257
rs483216	LOC338667	11	125323447	7	1.282024
rs635346	LOC338667	11	125323615	1	0.837903
rs635346	LOC338667	11	125323615	3	0.68476

rs635346	LOC338667	11	125323615	7	0.023124
rs635346	LOC338667	11	125323615	4	0.646
rs4937076	LOC338667	11	125331912	7	1.015142
rs4937076	LOC338667	11	125331912	4	1.385578
rs4937076	LOC338667	11	125331912	3	1.900996
rs3039	CDON	11	125331995	6	1.59783
rs684535	CDON	11	125336180	1	6.888701
rs684535	CDON	11	125336180	3	4.09839
rs684535	CDON	11	125336180	4	5.927728
rs684535	CDON	11	125336180	7	8.997784
rs2276061	CDON	11	125336911	6	2.212802
rs12274923	CDON	11	125376925	1	5.354731
rs12274923	CDON	11	125376925	7	0.14769
rs12274923	CDON	11	125376925	5	3.994003
rs12274923	CDON	11	125376925	3	1.091234
rs3740909	CDON	11	125394736	2	3.809934
rs3740909	CDON	11	125394736	3	1.043764
rs3740909	CDON	11	125394736	7	2.109854
rs1786702	FOXRED1	11	125644310	5	5.55351
rs3802814	TIRAP	11	125667817	7	0.300532
rs611953	TIRAP	11	125668901	2	4.937728
rs611953	TIRAP	11	125668901	5	0.490908
rs611953	TIRAP	11	125668901	8	5.894424
rs12269776	KIRREL3	11	125896507	5	0.596537
rs675759	KCNJ1	11	128213185	3	1.230348
rs675759	KCNJ1	11	128213185	2	1.416695
rs675388	KCNJ1	11	128213219	3	0.51897
rs673992	KCNJ1	11	128213554	3	0.237977
rs673992	KCNJ1	11	128213554	2	0.792257
rs1231254	KCNJ1	11	128214157	3	0.770798
rs12362800	C11orf45	11	128277347	4	0.190973
rs12362800	C11orf45	11	128277347	2	3.330721
rs6590357	KCNJ5	11	128286549	2	0.450575
rs6590357	KCNJ5	11	128286549	3	0.429116
rs6590357	KCNJ5	11	128286549	1	0.884292
rs6590357	KCNJ5	11	128286549	6	0.606751
rs2846675	KCNJ5	11	128292246	3	0.507937
rs2846675	KCNJ5	11	128292246	1	1.989927
rs2846675	KCNJ5	11	128292246	6	1.035092
rs7925056	KCNJ5	11	128292764	1	0.021759
rs7925056	KCNJ5	11	128292764	6	0.24991
rs10791011	BARX2	11	128826276	1	4.388622
rs11221912	PRDM10	11	129336006	7	5.901921
rs11221912	PRDM10	11	129336006	4	5.103775
rs704625	ST14	11	129585247	2	7.718427
rs704625	ST14	11	129585247	1	0.429764
rs704625	ST14	11	129585247	4	0.090112

rs704625	ST14	11	129585247	6	0.282427
rs5852	ST14	11	129585275	4	1.119467
rs5852	ST14	11	129585275	6	6.388192
rs5852	ST14	11	129585275	1	0.128382
rs5852	ST14	11	129585275	2	0.312621
rs11222085	ADAMTS8	11	129780959	6	0.864059
rs11222085	ADAMTS8	11	129780959	8	0.232599
rs2131535	ADAMTS8	11	129786698	1	0.51787
rs2291348	ADAMTS8	11	129794327	5	0.184794
rs731446	ADAMTS15	11	129844522	5	0.284665
rs7933829	C11orf44	11	130048092	5	0.049078
rs1050071	SNX19	11	130252441	2	0.509726
rs1050071	SNX19	11	130252441	4	0.82116
rs1050071	SNX19	11	130252441	5	0.772556
rs1050071	SNX19	11	130252441	3	8.772753
rs4373936	SNX19	11	130281823	5	0.005693
rs4373936	SNX19	11	130281823	1	5.197229
rs4373936	SNX19	11	130281823	4	1.073553
SNP11-13028960	SNX19	11	130289606	2	3.845348
SNP11-13028960	SNX19	11	130289606	1	0.349763
SNP11-13028960	SNX19	11	130289606	3	0.281713
rs2658859	C11orf39	11	131036433	1	2.649708
rs10894557	OPCML	11	131794070	4	1.131075
rs3360	OPCML	11	131794971	3	0.545625
rs3360	OPCML	11	131794971	5	0.75563
rs3360	OPCML	11	131794971	8	0.322547
rs3360	OPCML	11	131794971	7	0.4227
rs2250123	SPATA19	11	133216006	3	0.794441
rs329652	LOC283174	11	133274909	4	0.232097
rs329652	LOC283174	11	133274909	6	0.307467
rs329652	LOC283174	11	133274909	1	4.285701
rs329652	LOC283174	11	133274909	7	1.204554
rs3824992	IGSF9B	11	133291800	3	0.8009
rs3802923	IGSF9B	11	133292034	3	0.717556
rs3802923	IGSF9B	11	133292034	1	1.541748
rs3802920	IGSF9B	11	133292211	4	1.66728
rs3802920	IGSF9B	11	133292211	1	3.675181
rs3802920	IGSF9B	11	133292211	7	1.040499
rs12800905	IGSF9B	11	133294202	3	1.297864
rs12800905	IGSF9B	11	133294202	2	1.477721
rs12800905	IGSF9B	11	133294202	1	1.904923
rs12800905	IGSF9B	11	133294202	5	0.482745
rs12800905	IGSF9B	11	133294202	4	1.039002
rs12800905	IGSF9B	11	133294202	6	4.724415
rs12800905	IGSF9B	11	133294202	7	2.562649
rs329637	IGSF9B	11	133310795	4	2.129979
rs329637	IGSF9B	11	133310795	1	4.23571

rs329637	IGSF9B	11	133310795	3	0.354404
rs329637	IGSF9B	11	133310795	5	5.773912
rs329637	IGSF9B	11	133310795	7	0.89921
rs329637	IGSF9B	11	133310795	6	6.150681
rs595986	IGSF9B	11	133319401	5	0.156848
rs595986	IGSF9B	11	133319401	6	0.772881
rs595986	IGSF9B	11	133319401	4	0.694471
rs641024	JAM3	11	133526058	2	4.421217
rs11607699	NCAPD3	11	133599098	8	5.976175
rs11607699	NCAPD3	11	133599098	6	0.525522
rs11607699	NCAPD3	11	133599098	3	0.193496
rs12180	VPS26B	11	133621335	6	0.025935
rs12180	VPS26B	11	133621335	2	4.784023
rs12180	VPS26B	11	133621335	3	0.328975
rs12180	VPS26B	11	133621335	1	1.018421
rs2443558	LOC729860	11	133651235	1	2.208161
rs2443558	LOC729860	11	133651235	3	0.723426
rs2443558	LOC729860	11	133651235	5	1.305206
rs2443558	LOC729860	11	133651235	7	0.973637
rs3824995	GLB1L3	11	133663955	4	1.606387
rs3824995	GLB1L3	11	133663955	5	0.865608
rs3824995	GLB1L3	11	133663955	3	2.456111
rs481252	GLB1L3	11	133693752	8	1.005345
rs481252	GLB1L3	11	133693752	1	1.80128
rs7124693	LOC89944	11	133707246	3	0.617617
rs893952	B3GAT1	11	133754559	2	2.524971
rs893952	B3GAT1	11	133754559	8	2.271825
rs893952	B3GAT1	11	133754559	1	0.645057
rs893952	B3GAT1	11	133754559	4	1.592697
SNP12-118463	IQSEC3	12	118463	5	1.445104
SNP12-118463	IQSEC3	12	118463	3	0.768852
SNP12-118463	IQSEC3	12	118463	1	1.123369
rs542736	SLC6A12	12	169976	1	0.609967
rs542736	SLC6A12	12	169976	5	2.106472
rs542736	SLC6A12	12	169976	3	0.058589
rs2075228	SLC6A12	12	170148	3	0.416368
rs2075228	SLC6A12	12	170148	1	2.040711
rs2075228	SLC6A12	12	170148	5	0.284221
rs577294	SLC6A13	12	203454	7	1.155908
rs2302258	CCDC77	12	421721	6	3.939224
rs2075033	B4GALNT3	12	514598	6	0.240838
rs2075033	B4GALNT3	12	514598	7	0.717043
rs11063529	B4GALNT3	12	527665	7	0.428725
rs11063529	B4GALNT3	12	527665	2	0.003248
rs11063529	B4GALNT3	12	527665	1	0.182549
rs7298766	B4GALNT3	12	531917	6	0.080462
rs7298766	B4GALNT3	12	531917	1	0.788392

rs7298766	B4GALNT3	12	531917	7	0.043088
rs2245918	NINJ2	12	543776	2	1.435301
rs2245918	NINJ2	12	543776	3	1.639291
rs2245910	NINJ2	12	543965	3	0.103356
rs2245910	NINJ2	12	543965	2	0.234921
rs2245906	NINJ2	12	544049	3	1.313997
rs34578646	NINJ2	12	545600	3	0.475171
rs1064125	ERC1	12	1471239	6	2.244378
rs1064125	ERC1	12	1471239	1	5.834318
rs35466364	FBXL14	12	1573173	1	5.230947
rs3803163	WNT5B	12	1626221	3	0.220892
rs3803163	WNT5B	12	1626221	1	4.447506
rs2286380	ADIPOR2	12	1767545	7	5.708216
rs2286380	ADIPOR2	12	1767545	2	5.965259
rs1044825	CACNA2D4	12	1771746	1	0.728391
rs1044825	CACNA2D4	12	1771746	6	1.591489
rs1044825	CACNA2D4	12	1771746	4	5.153153
rs1044825	CACNA2D4	12	1771746	5	0.372214
rs2286379	CACNA2D4	12	1772425	2	1.326169
rs2286379	CACNA2D4	12	1772425	1	1.212531
rs2286379	CACNA2D4	12	1772425	5	0.38629
rs2286379	CACNA2D4	12	1772425	6	3.799402
rs2286379	CACNA2D4	12	1772425	4	0.65851
rs2058111	CACNA2D4	12	1773027	6	0.914325
rs2058111	CACNA2D4	12	1773027	5	1.161912
rs2058111	CACNA2D4	12	1773027	1	0.070178
rs34730825	DCP1B	12	1934863	1	0.294664
rs1544514	CACNA1C	12	2428447	8	0.778603
rs11062319	CACNA1C	12	2670910	3	0.639819
rs2907608	C12orf32	12	2867658	7	3.697833
rs2907608	C12orf32	12	2867658	6	0.437609
rs634076	TSPAN9	12	3262819	1	0.697486
rs634076	TSPAN9	12	3262819	2	0.154741
rs634076	TSPAN9	12	3262819	3	0.252037
rs634076	TSPAN9	12	3262819	4	1.156942
rs887305	EFCAB4B	12	3627514	5	3.360775
rs887305	EFCAB4B	12	3627514	7	0.264753
rs887305	EFCAB4B	12	3627514	2	2.548722
rs887305	EFCAB4B	12	3627514	6	2.25663
rs877554	EFCAB4B	12	3627685	6	0.115642
rs877554	EFCAB4B	12	3627685	2	3.418389
rs877554	EFCAB4B	12	3627685	7	3.252891
rs877554	EFCAB4B	12	3627685	5	0.372553
rs877554	EFCAB4B	12	3627685	3	0.090932
rs887304	EFCAB4B	12	3627809	1	6.04109
rs887304	EFCAB4B	12	3627809	3	1.874088
SNP12-3652908	EFCAB4B	12	3652908	7	0.254433

rs3803135	EFCAB4B	12	3658449	7	0.065762
rs242017	EFCAB4B	12	3658483	1	4.763704
rs10491974	EFCAB4B	12	3712450	6	1.458271
rs10491974	EFCAB4B	12	3712450	4	1.059806
rs10491974	EFCAB4B	12	3712450	5	6.965749
rs700481	PARP11	12	3788736	7	0.019044
rs700481	PARP11	12	3788736	6	1.175558
rs12823996	PARP11	12	3852736	7	1.159381
rs7315461	LOC399988	12	4077029	4	0.304484
rs7309402	C12orf5	12	4329293	1	5.298956
rs11063112	FGF23	12	4348341	3	0.681752
rs11063112	FGF23	12	4348341	7	0.362323
rs2241279	FGF6	12	4423644	4	0.878738
rs2241279	FGF6	12	4423644	5	0.922324
SNP12-4572472	DYRK4	12	4572472	7	0.562561
rs3741925	DYRK4	12	4575578	3	0.063676
rs3741925	DYRK4	12	4575578	1	0.154561
rs3741925	DYRK4	12	4575578	4	0.235283
rs3741925	DYRK4	12	4575578	2	0.953853
SNP12-4606830	AKAP3	12	4606830	4	1.712098
rs34076756	NDUFA9	12	4661712	5	0.051652
rs2286578	GALNT8	12	4706143	4	0.505816
rs2286578	GALNT8	12	4706143	7	5.061582
rs2286581	GALNT8	12	4752027	1	2.194874
rs2286581	GALNT8	12	4752027	7	0.107098
rs2286581	GALNT8	12	4752027	4	0.823402
rs2286582	GALNT8	12	4752036	1	2.673664
rs2286582	GALNT8	12	4752036	5	0.25568
rs758534	KCNA6	12	4789528	5	2.566066
rs2239503	KCNA6	12	4791489	7	1.75501
rs3741930	KCNA5	12	5023408	5	2.558087
rs6332	NTF3	12	5473893	1	0.251083
rs6332	NTF3	12	5473893	6	0.948492
rs6332	NTF3	12	5473893	2	1.20443
rs6332	NTF3	12	5473893	7	1.536839
rs6332	NTF3	12	5473893	3	0.505777
rs10849277	NTF3	12	5474196	6	1.038322
rs10849277	NTF3	12	5474196	8	0.84693
rs10849277	NTF3	12	5474196	7	1.026475
rs10849277	NTF3	12	5474196	3	1.020698
SNP12-5948685	VWF	12	5948685	7	1.021839
rs216902	VWF	12	5975648	3	0.221872
rs7312411	VWF	12	6037457	3	2.286406
rs7312411	VWF	12	6037457	1	3.41441
SNP12-6053089	VWF	12	6053089	1	0.757756
rs740842	PLEKHG6	12	6291756	1	3.231939
rs740842	PLEKHG6	12	6291756	7	4.727143

rs740842	PLEKHG6	12	6291756	8	4.814869
rs740842	PLEKHG6	12	6291756	4	10.07127
rs740842	PLEKHG6	12	6291756	3	0.118077
rs4149584	TNFRSF1A	12	6312904	8	5.716362
rs25680	CD27	12	6424889	6	0.067572
rs25680	CD27	12	6424889	5	1.60009
rs1059501	CD27	12	6430834	6	1.426018
rs1059501	CD27	12	6430834	2	2.1601
rs1059501	CD27	12	6430834	4	0.442171
rs1045546	TAPBPL	12	6438168	2	3.840463
rs1045546	TAPBPL	12	6438168	6	0.044582
rs1045546	TAPBPL	12	6438168	3	1.066845
rs2534712	VAMP1	12	6442810	6	1.835856
rs2534712	VAMP1	12	6442810	2	4.864945
rs2534713	VAMP1	12	6442848	8	5.113341
rs2534713	VAMP1	12	6442848	4	5.253699
rs2534713	VAMP1	12	6442848	3	0.06631
rs2534715	VAMP1	12	6443407	3	0.086419
rs2534715	VAMP1	12	6443407	4	0.601046
rs2534717	VAMP1	12	6444010	4	0.255721
rs2534717	VAMP1	12	6444010	8	0.499098
rs2534717	VAMP1	12	6444010	3	0.710191
rs1034969	VAMP1	12	6444117	8	0.317093
rs1034969	VAMP1	12	6444117	4	0.662073
rs1034969	VAMP1	12	6444117	3	0.892645
rs2072375	VAMP1	12	6445305	4	0.707082
rs2072375	VAMP1	12	6445305	3	0.88051
rs2072376	VAMP1	12	6450073	4	0.649198
rs2072376	VAMP1	12	6450073	3	1.216301
rs2072376	VAMP1	12	6450073	6	0.34289
rs3180589	MRPL51	12	6472683	2	4.716038
rs3180589	MRPL51	12	6472683	6	0.261209
rs7976498	MRPL51	12	6472694	2	3.826087
rs7976498	MRPL51	12	6472694	6	1.323366
rs714774	NCAPD2	12	6489545	4	0.16266
rs714774	NCAPD2	12	6489545	8	0.069202
rs714774	NCAPD2	12	6489545	3	0.194393
rs740850	NCAPD2	12	6508377	6	0.920213
rs1136666	GAPDH	12	6514252	6	5.078034
rs1136666	GAPDH	12	6514252	8	6.799929
rs1047792	HOM-TES-103	12	6519360	8	0.351347
GA028649	CHD4	12	6577787	2	6.035218
rs11539542	CHD4	12	6579320	6	4.3576
rs11539542	CHD4	12	6579320	2	4.170676
rs1639122	CHD4	12	6581408	4	1.175765
rs1639122	CHD4	12	6581408	3	1.239565
rs1639122	CHD4	12	6581408	6	0.250191

rs7964510	ZNF384	12	6657883	7	0.580504
rs870849	LAG3	12	6757281	8	0.421353
rs870849	LAG3	12	6757281	1	0.76886
rs870849	LAG3	12	6757281	4	0.478265
rs870849	LAG3	12	6757281	3	0.412858
rs870849	LAG3	12	6757281	2	0.567392
rs11612427	GPR162	12	6806238	4	0.376139
rs11612427	GPR162	12	6806238	3	0.776226
rs11612427	GPR162	12	6806238	5	0.500206
rs11612427	GPR162	12	6806238	7	0.86669
rs10744716	LEPREL2	12	6810111	2	1.679438
rs10744716	LEPREL2	12	6810111	3	0.530777
rs10744716	LEPREL2	12	6810111	6	0.6916
SNP12-6817457	LEPREL2	12	6817457	8	5.950159
rs1129649	LEPREL2	12	6818729	3	0.292648
rs1129649	LEPREL2	12	6818729	1	0.509279
rs1984564	MBOAT5	12	6960454	6	1.042572
rs1984564	MBOAT5	12	6960454	7	4.739348
rs12146727	C1S	12	7040597	8	3.182184
rs12146727	C1S	12	7040597	6	0.189641
rs7965055	C1S	12	7041881	2	3.051282
SNP12-7172584	RBP5	12	7172584	4	0.335849
rs7306824	CD163L1	12	7419738	7	0.580015
rs10845159	CD163L1	12	7477238	1	0.114159
rs10772331	LOC727815	12	7483406	1	4.267474
rs10772331	LOC727815	12	7483406	5	4.86262
rs4883263	CD163	12	7540751	5	1.538217
rs4294629	NANOG	12	7836826	5	0.581629
rs35297839	FOXJ2	12	8077120	4	0.585274
rs7842	C3AR1	12	8102454	7	0.500603
rs7842	C3AR1	12	8102454	2	3.585209
rs7842	C3AR1	12	8102454	8	2.851069
rs7842	C3AR1	12	8102454	5	0.280036
rs7842	C3AR1	12	8102454	6	1.883533
rs1062836	NECAP1	12	8140317	5	0.051263
rs1062836	NECAP1	12	8140317	6	3.485997
rs2024301	CLEC4A	12	8169448	6	2.958223
rs2024301	CLEC4A	12	8169448	7	0.788999
rs2024301	CLEC4A	12	8169448	5	0.064239
rs10841845	CLEC4E	12	8577700	7	0.354849
rs10841845	CLEC4E	12	8577700	3	1.132438
rs14541	MFAP5	12	8691833	8	1.727919
rs1860967	A2ML1	12	8905022	1	3.652746
rs1860967	A2ML1	12	8905022	8	4.275124
rs1860967	A2ML1	12	8905022	3	0.145288
SNP12-8907723	A2ML1	12	8907723	2	4.778116
rs1476910	A2ML1	12	8912179	6	5.696188

rs1476910	A2ML1	12	8912179	7	0.895389
rs1805723	KLRG1	12	9033564	5	0.064291
rs1805716	KLRG1	12	9054163	7	1.028332
rs1805716	KLRG1	12	9054163	8	1.609755
rs1805716	KLRG1	12	9054163	5	0.547459
rs12826054	KLRG1	12	9054570	7	0.427074
rs12826054	KLRG1	12	9054570	5	0.777234
rs226380	A2M	12	9159740	5	0.898197
rs226380	A2M	12	9159740	7	0.308284
rs226380	A2M	12	9159740	1	2.480256
rs226380	A2M	12	9159740	8	2.850664
rs10842971	PZP	12	9194563	1	0.549551
rs10771357	PZP	12	9196740	8	0.779075
rs10771357	PZP	12	9196740	7	2.354546
rs10771357	PZP	12	9196740	1	0.848407
rs10771357	PZP	12	9196740	5	0.721788
SNP12-9204218	PZP	12	9204218	1	1.958019
rs10771407	PZP	12	9236540	1	2.254682
SNP12-9238059	PZP	12	9238059	3	0.534141
SNP12-9238059	PZP	12	9238059	1	2.485678
SNP12-9238059	PZP	12	9238059	5	0.658088
rs1135816	KLRB1	12	9641936	5	1.2634
rs1135816	KLRB1	12	9641936	1	2.285788
rs1135816	KLRB1	12	9641936	4	1.312315
SNP12-9724791	CLEC2D	12	9724791	6	1.063024
SNP12-9724791	CLEC2D	12	9724791	7	0.514424
SNP12-9724791	CLEC2D	12	9724791	4	0.549483
SNP12-9724791	CLEC2D	12	9724791	5	0.014129
rs11052877	CD69	12	9796957	1	1.565542
rs11052877	CD69	12	9796957	5	0.804809
rs917911	CD69	12	9797118	1	0.061155
rs917911	CD69	12	9797118	5	0.555194
rs2232548	KLRF1	12	9877182	5	2.646249
rs7980232	KLRF1	12	9888542	5	6.244244
rs479499	CLEC12A	12	10028824	2	3.733127
rs479499	CLEC12A	12	10028824	4	4.705637
rs494312	CLEC12B	12	10059978	8	3.631846
rs494312	CLEC12B	12	10059978	4	1.761287
rs7315231	CLEC9A	12	10098192	5	1.057396
rs7315231	CLEC9A	12	10098192	1	1.145275
rs7959451	CLEC7A	12	10162322	4	1.147128
rs7959451	CLEC7A	12	10162322	5	0.928931
rs1050289	OLR1	12	10202433	1	4.184905
rs1050286	OLR1	12	10202830	1	3.261717
rs17808009	OLR1	12	10203196	3	0.581254
rs10505755	OLR1	12	10203229	5	0.12842
rs10505755	OLR1	12	10203229	7	1.267848

rs10505755	OLR1	12	10203229	1	1.134424
rs10505755	OLR1	12	10203229	3	1.991835
rs4523782	C12orf59	12	10234360	5	1.025557
rs1049172	KLRK1	12	10417007	1	5.949605
rs1049172	KLRK1	12	10417007	5	0.089589
rs2255336	KLRK1	12	10423593	4	4.917839
rs2255336	KLRK1	12	10423593	5	0.876607
rs1841957	KLRC4	12	10453292	1	3.988187
rs2244387	KLRA1	12	10637652	1	4.023879
rs2168749	KLRA1	12	10639581	1	0.862019
rs2168749	KLRA1	12	10639581	3	1.162329
rs2682484	KLRA1	12	10639670	1	5.255132
rs2682484	KLRA1	12	10639670	5	0.981892
rs1054396	FLJ10292	12	10648122	3	0.35552
rs1054396	FLJ10292	12	10648122	1	0.069516
rs1054396	FLJ10292	12	10648122	5	0.098508
rs1548803	TAS2R8	12	10850298	1	0.556135
rs3741845	TAS2R9	12	10853382	5	0.98207
rs3741845	TAS2R9	12	10853382	2	1.174726
rs3741845	TAS2R9	12	10853382	1	0.350767
rs1047699	PRR4	12	10890975	1	0.169857
rs1047699	PRR4	12	10890975	2	0.441417
rs1015442	TAS2R13	12	10951962	5	0.858227
rs1015442	TAS2R13	12	10951962	7	0.804346
rs1015442	TAS2R13	12	10951962	1	0.430766
rs1015443	TAS2R13	12	10952389	1	0.358381
rs1015443	TAS2R13	12	10952389	2	2.917399
rs1015443	TAS2R13	12	10952389	6	0.188231
rs1015443	TAS2R13	12	10952389	5	0.189559
rs1015443	TAS2R13	12	10952389	7	1.03339
rs10492101	PRH2	12	10977431	5	0.442466
rs10492102	PRH2	12	10977650	5	4.586023
rs10492102	PRH2	12	10977650	7	3.548877
rs10772397	TAS2R50	12	11029950	5	1.383329
rs10772397	TAS2R50	12	11029950	1	1.111936
rs10772397	TAS2R50	12	11029950	7	1.282216
rs10772397	TAS2R50	12	11029950	2	0.362161
rs10845279	TAS2R49	12	11040978	5	0.906716
rs10845279	TAS2R49	12	11040978	1	3.907149
rs10845279	TAS2R49	12	11040978	4	1.354362
rs10845279	TAS2R49	12	11040978	3	0.887756
rs10845280	TAS2R49	12	11040987	1	3.944212
rs10845280	TAS2R49	12	11040987	3	0.268545
rs10845280	TAS2R49	12	11040987	5	0.840393
rs10845280	TAS2R49	12	11040987	4	0.413762
rs12226919	TAS2R49	12	11041300	4	0.478275
rs12226919	TAS2R49	12	11041300	5	0.14976

rs12226919	TAS2R49	12	11041300	3	0.426542
rs12226919	TAS2R49	12	11041300	1	3.610975
rs12226920	TAS2R49	12	11041313	3	0.156548
rs12226920	TAS2R49	12	11041313	5	0.119015
rs12226920	TAS2R49	12	11041313	1	4.344348
rs12318612	TAS2R44	12	11074375	3	1.549245
rs12318612	TAS2R44	12	11074375	2	6.375581
rs12318612	TAS2R44	12	11074375	4	0.602593
rs12318612	TAS2R44	12	11074375	7	1.067498
rs1451772	TAS2R42	12	11230017	2	2.347885
rs1451772	TAS2R42	12	11230017	5	1.739586
rs1669413	TAS2R42	12	11230048	1	3.934806
rs1650019	TAS2R42	12	11230250	1	2.510404
rs1863842	PRB4	12	11351330	1	3.035991
rs1863842	PRB4	12	11351330	5	1.117019
rs11611479	ETV6	12	11883435	4	0.180972
rs2287154	BCL2L14	12	12115810	1	4.379607
rs11054682	BCL2L14	12	12138861	1	4.418007
rs11054683	BCL2L14	12	12138883	1	2.864888
rs1797647	BCL2L14	12	12143345	2	1.762181
rs17375215	MANSC1	12	12375031	5	5.110914
rs3751262	LOH12CR1	12	12509957	2	4.126451
rs3751262	LOH12CR1	12	12509957	5	1.182273
rs3803098	LOH12CR1	12	12510393	2	4.303171
rs3803098	LOH12CR1	12	12510393	5	0.263115
rs4237941	LOC644467	12	12612565	3	4.159403
rs4237941	LOC644467	12	12612565	5	4.126291
rs4237941	LOC644467	12	12612565	6	2.721052
rs4237941	LOC644467	12	12612565	1	0.891824
rs1802173	CREBL2	12	12689037	7	0.044922
rs4763861	GPR19	12	12706005	8	3.828522
rs34330	CDKN1B	12	12761962	7	4.723854
rs34330	CDKN1B	12	12761962	3	0.465409
rs34330	CDKN1B	12	12761962	4	3.517488
rs34330	CDKN1B	12	12761962	1	0.48147
rs2110596	APOLD1	12	12832097	5	0.38724
rs2110596	APOLD1	12	12832097	4	0.234523
rs2110596	APOLD1	12	12832097	1	0.721821
rs1420022	APOLD1	12	12832963	4	2.853935
rs1420022	APOLD1	12	12832963	3	0.488903
rs1420022	APOLD1	12	12832963	8	2.855671
rs1420022	APOLD1	12	12832963	1	5.25437
rs1420022	APOLD1	12	12832963	7	0.923736
rs1420022	APOLD1	12	12832963	6	0.713953
rs7301360	APOLD1	12	12833800	4	0.315404
rs7301360	APOLD1	12	12833800	8	0.84567
rs7301360	APOLD1	12	12833800	6	3.004058

rs7301360	APOLD1	12	12833800	3	0.843534
rs7301360	APOLD1	12	12833800	1	5.287297
rs1061036	GPRC5A	12	12956971	4	0.39216
rs1061036	GPRC5A	12	12956971	3	0.638194
rs1061036	GPRC5A	12	12956971	8	1.062714
rs3825272	HTR7P	12	13046606	7	2.18224
rs3825272	HTR7P	12	13046606	3	1.018199
rs3825272	HTR7P	12	13046606	1	5.524107
rs3825272	HTR7P	12	13046606	4	5.456228
rs4763924	KIAA1467	12	13099819	2	2.792149
rs3741818	KIAA1467	12	13105804	2	6.042129
rs17821405	C12orf36	12	13417595	7	2.048014
rs17821405	C12orf36	12	13417595	6	2.936726
rs17821405	C12orf36	12	13417595	8	3.267469
rs11055390	C12orf36	12	13420903	1	2.973365
rs1805502	GRIN2B	12	13605448	6	0.83148
rs1805502	GRIN2B	12	13605448	5	0.172638
rs1805502	GRIN2B	12	13605448	4	0.744605
rs1805476	GRIN2B	12	13605630	2	0.653012
rs1805476	GRIN2B	12	13605630	7	4.611781
rs1805476	GRIN2B	12	13605630	4	0.141758
rs1805476	GRIN2B	12	13605630	1	0.394896
rs1805503	GRIN2B	12	13606417	1	3.922485
rs890	GRIN2B	12	13606575	8	1.311597
rs890	GRIN2B	12	13606575	5	0.392349
rs890	GRIN2B	12	13606575	1	1.001419
rs890	GRIN2B	12	13606575	7	0.923802
rs890	GRIN2B	12	13606575	2	1.277094
rs1806191	GRIN2B	12	13607905	7	1.305974
rs1806191	GRIN2B	12	13607905	1	1.549769
rs3026160	GRIN2B	12	13611310	6	1.571236
rs1805482	GRIN2B	12	13656041	3	0.709764
rs1805482	GRIN2B	12	13656041	1	0.8976
rs2231908	ATF7IP	12	14468948	4	0.076399
rs3748289	H2AFJ	12	14820934	7	0.143231
rs3748289	H2AFJ	12	14820934	4	1.000128
rs3748289	H2AFJ	12	14820934	5	0.630505
rs2241221	LOC440087	12	14850658	7	0.061539
rs7304054	C12orf60	12	14867330	7	0.458612
rs7304054	C12orf60	12	14867330	2	3.858356
rs7304054	C12orf60	12	14867330	8	0.458859
rs3088189	ART4	12	14884875	6	0.013133
rs3088189	ART4	12	14884875	3	0.459389
rs3088189	ART4	12	14884875	1	3.756081
rs1861698	ART4	12	14885121	7	0.480152
rs1861698	ART4	12	14885121	3	0.240593
rs1861698	ART4	12	14885121	1	5.60833

rs1861698	ART4	12	14885121	6	0.170427
rs3748299	PTPRO	12	15624936	1	0.436561
rs11056575	PTPRO	12	15639682	6	0.566772
rs11875	MGST1	12	16408261	2	5.103265
rs941047	RERGL	12	18125364	1	2.672494
rs12312266	PIK3C2G	12	18540324	3	0.851455
rs12312266	PIK3C2G	12	18540324	8	7.264722
rs11044142	PIK3C2G	12	18547492	1	4.948025
rs11044142	PIK3C2G	12	18547492	5	7.037895
rs10505830	PLCZ1	12	18732382	5	0.533317
rs1075421	CAPZA3	12	18782346	1	0.368547
rs1075421	CAPZA3	12	18782346	7	1.24965
rs1075421	CAPZA3	12	18782346	4	0.468381
rs1844950	LOC400013	12	19985791	6	0.199111
rs4149117	SLCO1B3	12	20902747	3	0.767396
SNP12-20919475	SLCO1B3	12	20919475	7	2.001443
SNP12-20919475	SLCO1B3	12	20919475	2	4.90341
rs11045676	LST-3TM12	12	21087691	4	0.519637
rs4149056	SLCO1B1	12	21222816	3	2.700069
rs4149056	SLCO1B1	12	21222816	1	1.927104
rs4149057	SLCO1B1	12	21222866	1	0.63803
rs4149087	SLCO1B1	12	21283829	1	1.726015
rs4149087	SLCO1B1	12	21283829	3	1.559442
rs4149088	SLCO1B1	12	21283853	3	2.663321
rs4149088	SLCO1B1	12	21283853	2	1.840059
rs4149088	SLCO1B1	12	21283853	6	2.91977
rs2058464	PYROXD1	12	21481888	4	4.091116
rs2058464	PYROXD1	12	21481888	3	0.657805
rs2058464	PYROXD1	12	21481888	6	0.283782
rs2058464	PYROXD1	12	21481888	8	0.188808
rs2110161	GOLT1B	12	21561122	1	3.820281
rs11046097	GOLT1B	12	21561151	5	10.28052
rs936	GYS2	12	21581003	4	1.08006
rs936	GYS2	12	21581003	1	0.874368
rs936	GYS2	12	21581003	7	0.105845
rs936	GYS2	12	21581003	2	0.102668
rs704219	ST8SIA1	12	22246188	3	0.81277
rs3803101	ST8SIA1	12	22378575	7	2.445204
rs3803101	ST8SIA1	12	22378575	3	0.470769
rs2292488	KIAA0528	12	22588690	8	0.893907
rs2129613	ETNK1	12	22731003	3	0.792224
rs2129613	ETNK1	12	22731003	1	3.316269
rs1043881	BCAT1	12	24855650	8	0.085379
rs1043881	BCAT1	12	24855650	4	0.362836
rs1872644	BCAT1	12	24858424	6	3.676037
rs1872644	BCAT1	12	24858424	3	1.543612
rs1872644	BCAT1	12	24858424	4	0.852965

rs1872644	BCAT1	12	24858424	1	2.596019
rs1872645	BCAT1	12	24858479	6	0.574332
rs1872645	BCAT1	12	24858479	3	0.782984
rs1872645	BCAT1	12	24858479	1	2.924054
rs1872645	BCAT1	12	24858479	4	0.607572
rs6487419	BCAT1	12	24858737	6	0.265938
rs6487419	BCAT1	12	24858737	4	0.198395
rs1054281	BCAT1	12	24859042	3	0.065731
rs1054281	BCAT1	12	24859042	1	4.426697
rs1054281	BCAT1	12	24859042	6	0.315544
rs1054281	BCAT1	12	24859042	4	0.750835
rs1054279	BCAT1	12	24859336	1	4.94418
rs1054279	BCAT1	12	24859336	4	0.307196
rs1054279	BCAT1	12	24859336	3	0.258467
rs1054277	BCAT1	12	24859398	6	0.027575
rs1054277	BCAT1	12	24859398	4	3.577537
rs1054277	BCAT1	12	24859398	1	2.742525
rs1054277	BCAT1	12	24859398	3	0.163625
rs6487420	BCAT1	12	24859417	6	0.071169
rs6487420	BCAT1	12	24859417	4	2.644639
rs6487420	BCAT1	12	24859417	1	3.675415
rs6487420	BCAT1	12	24859417	3	0.598946
rs1471842	BCAT1	12	24860095	6	3.625798
rs1471842	BCAT1	12	24860095	4	2.920663
rs1471842	BCAT1	12	24860095	1	4.237644
rs1471842	BCAT1	12	24860095	3	0.809431
rs2046524	BCAT1	12	24860321	1	3.905419
rs2046524	BCAT1	12	24860321	6	0.713964
rs2046524	BCAT1	12	24860321	4	0.663162
rs2046524	BCAT1	12	24860321	3	0.829166
rs7305794	BCAT1	12	24860894	6	1.171912
rs7305794	BCAT1	12	24860894	4	0.515191
rs7305794	BCAT1	12	24860894	3	0.128866
rs7305794	BCAT1	12	24860894	1	1.662527
rs6487421	BCAT1	12	24861502	6	1.039458
rs6487421	BCAT1	12	24861502	4	0.517951
rs6487421	BCAT1	12	24861502	3	1.185508
rs6487421	BCAT1	12	24861502	1	3.581275
rs2200509	BCAT1	12	24861855	3	0.40825
rs2200509	BCAT1	12	24861855	4	0.039259
rs2200509	BCAT1	12	24861855	1	2.437757
rs2200509	BCAT1	12	24861855	6	0.223076
rs2220269	BCAT1	12	24861975	3	0.241651
rs2220269	BCAT1	12	24861975	1	3.557285
rs2220269	BCAT1	12	24861975	4	0.505788
rs2220269	BCAT1	12	24861975	6	0.268679
rs16928315	LOC196415	12	25038459	5	4.115056

rs1908946	LRMP	12	25134382	6	0.60083
rs1908946	LRMP	12	25134382	8	0.450449
rs1908946	LRMP	12	25134382	7	1.526155
rs1908946	LRMP	12	25134382	5	1.174548
rs10842496	CASC1	12	25202756	4	0.773814
rs10842496	CASC1	12	25202756	5	0.612903
rs12313736	SSPN	12	26275226	2	2.924317
rs17381729	SSPN	12	26276878	7	0.970152
rs17381729	SSPN	12	26276878	2	1.992149
rs9214	SSPN	12	26277053	5	0.081043
rs9214	SSPN	12	26277053	3	0.110593
rs2230376	ITPR2	12	26624254	6	0.008453
rs2230376	ITPR2	12	26624254	7	0.526121
rs2230376	ITPR2	12	26624254	5	0.170352
rs2230376	ITPR2	12	26624254	1	5.203889
rs2230375	ITPR2	12	26624329	5	1.411892
rs2230375	ITPR2	12	26624329	1	4.951081
rs2230375	ITPR2	12	26624329	6	4.47471
rs2230375	ITPR2	12	26624329	7	0.472328
rs2230377	ITPR2	12	26641098	7	0.450213
rs2230377	ITPR2	12	26641098	5	0.365943
rs2230377	ITPR2	12	26641098	1	3.819921
rs3963548	MED21	12	27066761	3	2.104338
rs708165	hCG_1794298	12	27125467	2	0.632903
rs12228215	hCG_1794298	12	27126546	1	4.441467
rs12228215	hCG_1794298	12	27126546	6	2.275855
rs411816	LOC341346	12	27519072	6	2.973014
rs411816	LOC341346	12	27519072	2	3.231154
rs411816	LOC341346	12	27519072	5	0.231164
SNP12-27692013	PPFIBP1	12	27692013	2	3.150522
SNP12-27692013	PPFIBP1	12	27692013	3	3.179822
SNP12-27692013	PPFIBP1	12	27692013	7	3.117917
SNP12-27692013	PPFIBP1	12	27692013	8	11.72038
SNP12-27692013	PPFIBP1	12	27692013	4	7.833741
SNP12-27692013	PPFIBP1	12	27692013	1	2.852279
SNP12-27692013	PPFIBP1	12	27692013	6	5.120539
SNP12-27692013	PPFIBP1	12	27692013	5	3.579013
rs12819160	REP15	12	27741380	4	0.061842
rs12819160	REP15	12	27741380	3	0.440619
SNP12-29487632	OVCH1	12	29487632	6	1.258384
rs7967676	OVCH1	12	29489559	5	0.965981
rs7967676	OVCH1	12	29489559	2	0.750518
rs1347570	OVCH1	12	29495659	7	1.082821
rs1347570	OVCH1	12	29495659	1	0.079979
rs1347570	OVCH1	12	29495659	2	3.167267
rs11050243	OVCH1	12	29508817	6	1.063326
rs11050243	OVCH1	12	29508817	5	1.612502

rs2043623	OVCH1	12	29519283	5	0.931107
rs2043623	OVCH1	12	29519283	1	1.012865
rs3847680	OVCH1	12	29530453	7	2.578869
rs3847680	OVCH1	12	29530453	4	0.15537
rs967181	OVCH1	12	29533840	7	0.836545
rs10843438	OVCH1	12	29539542	6	0.212442
rs10843438	OVCH1	12	29539542	7	2.041344
rs10843438	OVCH1	12	29539542	4	0.470037
rs6487834	TMTC1	12	29548400	1	0.045952
rs6487834	TMTC1	12	29548400	2	2.259661
rs11834712	TMTC1	12	29549461	5	0.351226
rs11834712	TMTC1	12	29549461	1	0.145827
rs11834712	TMTC1	12	29549461	2	1.176299
rs11834712	TMTC1	12	29549461	8	6.049682
rs4931199	TMTC1	12	29550042	7	0.356186
rs4931199	TMTC1	12	29550042	3	0.566949
rs12809335	IPO8	12	30739942	3	0.554772
rs12809335	IPO8	12	30739942	4	5.981275
SNP12-3077024З	CAPRIN2	12	30770243	2	4.409315
SNP12-3077024З	CAPRIN2	12	30770243	5	3.915348
SNP12-3077024З	CAPRIN2	12	30770243	4	0.247507
rs17688567	CAPRIN2	12	30797625	5	1.462102
rs17688567	CAPRIN2	12	30797625	6	1.480881
rs17688567	CAPRIN2	12	30797625	4	3.538576
rs2075333	TSPAN11	12	31026846	6	1.1841
rs2075333	TSPAN11	12	31026846	1	0.112999
rs35076	TSPAN11	12	31036151	3	0.102203
rs35076	TSPAN11	12	31036151	1	0.613859
rs2075322	DDX11	12	31141128	1	6.873134
rs2075322	DDX11	12	31141128	4	0.617118
rs2075322	DDX11	12	31141128	7	0.694376
rs11051266	OVOS2	12	31180222	4	4.743429
rs11051266	OVOS2	12	31180222	8	3.745695
rs11051266	OVOS2	12	31180222	2	1.825621
rs11051266	OVOS2	12	31180222	1	5.342634
rs11051266	OVOS2	12	31180222	5	3.486775
rs11051274	OVOS2	12	31193155	1	4.697006
rs11051274	OVOS2	12	31193155	5	0.443996
rs7966525	MGC50559	12	31713091	7	1.124059
rs3759295	LOC440093	12	31836252	4	1.836223
rs16919122	C12orf35	12	32026082	5	0.269763
rs16919122	C12orf35	12	32026082	6	5.394612
rs3207618	C12orf35	12	32026193	1	5.631402
rs3759302	C12orf35	12	32026453	5	0.00807
rs3759301	C12orf35	12	32026708	3	0.153247
rs3759299	C12orf35	12	32028779	3	0.02456
rs3759297	C12orf35	12	32029119	5	4.989743

rs1151016	LOC729457	12	32150634	4	0.915164
rs11051966	BICD1	12	32421847	1	1.052031
rs11051966	BICD1	12	32421847	6	1.66398
rs11051966	BICD1	12	32421847	2	4.555833
rs11052110	FGD4	12	32668629	7	0.481607
rs11052110	FGD4	12	32668629	5	0.706178
rs1059422	DNM1L	12	32787632	5	0.008102
rs1059422	DNM1L	12	32787632	2	3.533295
rs1059422	DNM1L	12	32787632	1	1.14802
rs1059422	DNM1L	12	32787632	4	0.387863
rs1059422	DNM1L	12	32787632	7	0.176566
rs1020670	DNM1L	12	32787882	7	1.672401
rs1020670	DNM1L	12	32787882	2	5.358761
rs1020670	DNM1L	12	32787882	4	0.62914
rs1020670	DNM1L	12	32787882	1	0.121229
rs1020670	DNM1L	12	32787882	5	0.287369
rs3759139	CPNE8	12	37373876	4	3.157355
rs3759139	CPNE8	12	37373876	7	0.048903
rs3759139	CPNE8	12	37373876	1	4.841079
rs35598213	CPNE8	12	37410361	2	1.19222
SNP12-3802161	KIF21A	12	38021615	5	0.049451
rs11173440	SLC2A13	12	38435649	4	0.207414
rs4322461	SLC2A13	12	38437190	4	0.19366
rs10735885	SLC2A13	12	38437813	4	4.998029
SNP12-3870854	SLC2A13	12	38708546	8	0.1617
SNP12-3870854	SLC2A13	12	38708546	7	1.424652
SNP12-3870854	SLC2A13	12	38708546	3	1.61984
SNP12-3870854	SLC2A13	12	38708546	4	0.563811
SNP12-3870854	SLC2A13	12	38708546	2	3.630745
SNP12-3870854	SLC2A13	12	38708546	6	0.220721
SNP12-3870854	SLC2A13	12	38708546	5	0.788442
SNP12-3870854	SLC2A13	12	38708546	1	0.653526
rs10878245	LRRK2	12	38918058	2	4.869528
rs7966550	LRRK2	12	38974962	2	2.67355
rs7966550	LRRK2	12	38974962	8	0.299066
rs11176013	LRRK2	12	39000140	3	0.411291
rs11564148	LRRK2	12	39000168	6	5.799387
rs11564148	LRRK2	12	39000168	3	0.437933
rs11564148	LRRK2	12	39000168	2	1.050991
rs10878371	LRRK2	12	39002527	3	0.450482
rs3761863	LRRK2	12	39044919	2	2.114093
rs3761863	LRRK2	12	39044919	8	6.295028
rs3761863	LRRK2	12	39044919	3	1.192834
rs10878441	LRRK2	12	39048198	8	5.757386
rs10878441	LRRK2	12	39048198	2	4.94744
rs10878441	LRRK2	12	39048198	3	0.547635
rs3886747	LRRK2	12	39048218	8	4.770317

rs10784548	LRRK2	12	39048813	2	2.754817
rs10784548	LRRK2	12	39048813	8	0.415537
rs10784548	LRRK2	12	39048813	3	0.71575
rs11564245	MUC19	12	39109621	5	0.102389
rs11564245	MUC19	12	39109621	6	0.036606
rs2114567	MUC19	12	39111754	1	1.213644
rs7966110	MUC19	12	39122001	3	1.586309
rs11176655	MUC19	12	39122016	7	4.786028
rs1492313	MUC19	12	39159293	3	0.128856
rs1492313	MUC19	12	39159293	4	0.198861
rs2588400	MUC19	12	39159518	2	2.529746
rs2588400	MUC19	12	39159518	4	3.972191
rs2588411	MUC19	12	39164719	3	0.065091
rs2638868	MUC19	12	39166327	3	0.389685
rs2638868	MUC19	12	39166327	7	0.22152
rs2638868	MUC19	12	39166327	1	2.243546
rs2638868	MUC19	12	39166327	4	0.470511
rs2197356	MUC19	12	39180255	7	0.194167
rs2197356	MUC19	12	39180255	1	3.38022
rs4768288	MUC19	12	39208358	5	2.384769
rs4768288	MUC19	12	39208358	1	0.724723
rs1444220	MUC19	12	39208371	4	3.057191
rs1444220	MUC19	12	39208371	5	3.052437
rs935105	CNTN1	12	39616878	3	0.458695
rs935105	CNTN1	12	39616878	4	0.432796
rs935105	CNTN1	12	39616878	8	7.637064
rs2406567	YAF2	12	40837746	1	5.665498
rs2406567	YAF2	12	40837746	5	0.386686
rs2406567	YAF2	12	40837746	6	5.095947
rs2406567	YAF2	12	40837746	8	0.523279
rs2406567	YAF2	12	40837746	7	0.166777
rs2406567	YAF2	12	40837746	3	0.214117
rs11181355	YAF2	12	40838113	6	5.200976
rs7968314	LOC727884	12	40967935	3	4.10565
rs7968314	LOC727884	12	40967935	7	5.402549
rs7968314	LOC727884	12	40967935	5	0.14657
rs7968314	LOC727884	12	40967935	8	5.03416
rs7295326	LOC727884	12	40968332	3	5.843545
rs7295326	LOC727884	12	40968332	2	5.248809
rs7295326	LOC727884	12	40968332	7	2.144918
rs7295326	LOC727884	12	40968332	8	3.319148
rs7295326	LOC727884	12	40968332	5	4.231208
rs7295326	LOC727884	12	40968332	1	5.632441
rs7295326	LOC727884	12	40968332	6	5.022581
rs7295326	LOC727884	12	40968332	4	6.787029
rs12371762	LOC727884	12	40968618	5	4.647023
rs12371762	LOC727884	12	40968618	6	3.435349

rs12371762	LOC727884	12	40968618	3	5.096076
rs12371762	LOC727884	12	40968618	8	4.845126
rs3747560	ZCRB1	12	41006157	2	0.810365
rs3747560	ZCRB1	12	41006157	7	1.821953
rs3747560	ZCRB1	12	41006157	6	2.007374
rs3747560	ZCRB1	12	41006157	3	0.973927
rs3747560	ZCRB1	12	41006157	5	0.957946
rs10506226	ADAMTS20	12	42055495	1	3.435895
rs11182088	ADAMTS20	12	42146793	5	2.7598
rs11182235	PUS7L	12	42410062	7	0.244897
rs4251545	IRAK4	12	42466562	7	0.937084
rs7974460	TMEM117	12	43069390	2	3.876509
rs3803178	TMEM16F	12	44110848	4	5.758322
rs724909	SFRS2IP	12	44609978	1	3.538594
rs724909	SFRS2IP	12	44609978	7	0.6078
rs724909	SFRS2IP	12	44609978	4	0.934035
rs724909	SFRS2IP	12	44609978	3	0.538861
rs2242355	SLC38A1	12	44948752	3	0.875139
rs2242355	SLC38A1	12	44948752	4	1.39402
rs2242355	SLC38A1	12	44948752	5	0.166383
rs11183605	SLC38A4	12	45445235	4	0.470077
rs12582305	SLC38A4	12	45445262	8	0.10339
rs2430921	SLC38A4	12	45445463	4	1.023991
rs2429467	SLC38A4	12	45473037	3	0.21617
rs2429467	SLC38A4	12	45473037	2	5.196938
rs2244818	FAM113B	12	45915635	8	4.147722
rs215351	LOC643063	12	46021667	5	1.279234
rs215351	LOC643063	12	46021667	3	1.133123
rs215351	LOC643063	12	46021667	8	1.179246
rs9859	HDAC7A	12	46463167	7	0.256446
rs9859	HDAC7A	12	46463167	4	10.18748
rs3847987	VDR	12	46524335	5	0.187179
rs3847987	VDR	12	46524335	7	0.248446
rs3847987	VDR	12	46524335	2	4.490133
rs10735810	VDR	12	46559162	3	1.108249
rs10735810	VDR	12	46559162	6	0.603801
rs10735810	VDR	12	46559162	1	0.76925
rs10735810	VDR	12	46559162	2	0.413888
rs11168293	VDR	12	46579983	8	6.435713
rs11168293	VDR	12	46579983	1	6.3246
rs11168293	VDR	12	46579983	6	1.178325
rs11168293	VDR	12	46579983	4	1.009304
rs2070739	COL2A1	12	46654243	7	0.41652
rs12721379	COL2A1	12	46654352	7	1.527465
SNP12-4665480[illegible]	COL2A1	12	46654808	1	6.030547
rs7487682	LOC387856	12	46865932	7	0.46613
rs11168453	DKFZP779L1853	12	46878606	7	0.477714

rs11168453	DKFZP779L1853	12	46878606	5	3.614543
rs10506276	DKFZP779L1853	12	46880781	4	0.076664
rs10506277	DKFZP779L1853	12	46880886	4	0.649798
rs11168459	OR10AD1	12	46882508	6	1.015652
rs11168459	OR10AD1	12	46882508	8	0.889759
rs17224674	OR10AD1	12	46882846	4	0.961334
rs17823193	OR10AD1	12	46883157	4	3.843255
rs17122812	OR10AD1	12	46883320	8	2.591222
rs17122812	OR10AD1	12	46883320	6	1.526494
rs11458	C12orf54	12	47174861	8	0.190904
rs11458	C12orf54	12	47174861	2	3.101323
rs11458	C12orf54	12	47174861	4	0.133226
rs2232565	LALBA	12	47249288	1	0.400261
rs3741628	C12orf41	12	47335005	3	0.161945
rs3741628	C12orf41	12	47335005	5	0.401049
rs3741628	C12orf41	12	47335005	6	3.282103
rs3741628	C12orf41	12	47335005	7	1.572662
rs3013	CCNT1	12	47373923	3	0.621179
rs2291728	CCNT1	12	47396849	5	0.364855
rs1057908	DDX23	12	47516302	3	0.693199
rs1057908	DDX23	12	47516302	1	5.249254
rs1057908	DDX23	12	47516302	8	4.762302
rs1565933	RND1	12	47542065	3	0.106406
rs3809147	CCDC65	12	47584326	6	0.62306
rs3809147	CCDC65	12	47584326	3	0.707631
rs3809147	CCDC65	12	47584326	5	0.236832
SNP12-47594551	CCDC65	12	47594551	4	0.949397
SNP12-47594551	CCDC65	12	47594551	6	0.057179
SNP12-47594551	CCDC65	12	47594551	2	0.964626
SNP12-47594551	CCDC65	12	47594551	3	0.817845
SNP12-47594551	CCDC65	12	47594551	5	0.957523
SNP12-47597054	CCDC65	12	47597054	4	0.368457
SNP12-47597054	CCDC65	12	47597054	3	0.766782
SNP12-47598807	CCDC65	12	47598807	4	0.765646
SNP12-47598807	CCDC65	12	47598807	3	0.112611
rs10875894	CCDC65	12	47601129	3	1.051981
rs10875894	CCDC65	12	47601129	5	0.549718
rs10875894	CCDC65	12	47601129	4	0.505945
rs4760600	CCDC65	12	47601261	3	0.324259
rs4760600	CCDC65	12	47601261	4	5.916472
rs4760600	CCDC65	12	47601261	2	5.256187
rs4760600	CCDC65	12	47601261	5	6.052627
rs4760600	CCDC65	12	47601261	6	2.685364
rs3741627	WNT10B	12	47645854	3	0.242839
rs3741627	WNT10B	12	47645854	6	6.492293
rs3741627	WNT10B	12	47645854	4	0.265303
rs3741627	WNT10B	12	47645854	5	0.031788

rs10783299	DDN	12	47676944	2	2.16568
rs10783299	DDN	12	47676944	1	0.078277
rs3782357	MLL2	12	47713919	1	0.460588
rs3782357	MLL2	12	47713919	6	5.139888
rs10747561	LMBR1L	12	47786776	6	5.620928
rs10747561	LMBR1L	12	47786776	2	4.652408
rs1150057	KCNH3	12	48224080	3	0.092023
rs7953534	LOC283331	12	48484147	6	0.024071
rs7953534	LOC283331	12	48484147	3	1.517439
rs7953534	LOC283331	12	48484147	2	4.356709
rs4898510	BCDIN3D	12	48518958	5	0.478903
rs1044677	FAIM2	12	48547092	5	2.10165
rs1044677	FAIM2	12	48547092	4	0.709651
rs1044677	FAIM2	12	48547092	6	3.706727
rs1064722	FAIM2	12	48547596	1	0.31002
rs1064722	FAIM2	12	48547596	7	1.414389
rs1064722	FAIM2	12	48547596	5	0.022637
rs1454193	FAIM2	12	48548036	7	1.035077
rs1454193	FAIM2	12	48548036	5	0.241494
rs426496	AQP2	12	48634345	5	0.265265
rs467323	AQP2	12	48636032	3	0.540383
rs2878771	AQP2	12	48638660	5	0.446405
rs2878771	AQP2	12	48638660	6	1.855781
rs2878771	AQP2	12	48638660	2	1.27454
rs2878771	AQP2	12	48638660	3	0.616055
rs2878771	AQP2	12	48638660	1	0.453283
rs653576	ACCN2	12	48738975	8	5.951782
rs836180	GPD1	12	48789536	1	0.082437
rs836180	GPD1	12	48789536	8	0.506809
rs7532	LASS5	12	48810231	8	7.547651
rs7532	LASS5	12	48810231	2	1.727182
rs7532	LASS5	12	48810231	1	0.279471
rs3741562	LASS5	12	48816003	1	5.858983
rs3741562	LASS5	12	48816003	8	0.702835
rs3741562	LASS5	12	48816003	4	0.381368
rs7302981	LASS5	12	48824082	1	0.485187
rs7302981	LASS5	12	48824082	2	1.169454
rs7302981	LASS5	12	48824082	8	6.042248
rs6580741	LOC643275	12	49013973	1	3.679177
rs6580742	LOC643275	12	49014078	1	0.565897
rs7296291	LOC643275	12	49030386	1	1.052943
rs7312252	LOC643275	12	49030438	1	1.423011
rs4421818	LOC121006	12	49035561	4	0.507505
rs4421818	LOC121006	12	49035561	1	1.663017
rs12303082	LOC121006	12	49040830	1	2.636797
SNP12-49366631	DIP2B	12	49366631	4	6.751543
SNP12-49366631	DIP2B	12	49366631	7	0.384967

rs11169524	DIP2B	12	49376001	4	4.389194
rs11169524	DIP2B	12	49376001	5	0.091552
rs11169524	DIP2B	12	49376001	1	3.539818
rs4768915	DIP2B	12	49428150	8	9.143355
rs17291650	ATF1	12	49499700	1	5.219681
rs861204	TMPRSS12	12	49524083	1	3.993327
rs11169736	TFCP2	12	49852924	2	2.478844
rs11169736	TFCP2	12	49852924	8	4.393534
rs11169736	TFCP2	12	49852924	6	0.682892
rs11169736	TFCP2	12	49852924	5	1.105229
SNP12-4995041	LOC57228	12	49950414	1	6.328279
rs1044981	BIN2	12	49961133	6	0.930928
rs1044981	BIN2	12	49961133	5	0.282592
rs7312857	BIN2	12	49993893	4	3.706471
SNP12-5000976	ELA1	12	50009766	1	1.491902
rs17860299	ELA1	12	50023874	4	1.94394
rs17860299	ELA1	12	50023874	1	2.747481
rs4761936	LOC728476	12	50063395	5	0.940688
rs4761936	LOC728476	12	50063395	6	3.014251
rs7305599	SLC4A8	12	50187657	8	0.395199
rs2292221	SLC4A8	12	50188262	8	0.024883
rs2292220	SLC4A8	12	50188516	6	0.955133
rs4761829	SCN8A	12	50367232	1	0.804487
rs60637	SCN8A	12	50487009	1	0.867531
rs3741705	SCN8A	12	50488291	2	2.773767
rs303782	LOC728503	12	50527973	1	2.343791
rs303782	LOC728503	12	50527973	5	1.641294
rs697633	ANKRD33	12	50568187	5	1.396214
rs697633	ANKRD33	12	50568187	3	2.728242
rs7303030	ANKRD33	12	50570750	8	0.395174
rs7303030	ANKRD33	12	50570750	5	0.774431
rs7303030	ANKRD33	12	50570750	2	0.21717
rs7303030	ANKRD33	12	50570750	3	0.493783
rs12368048	ANKRD33	12	50570935	3	0.978164
rs12368048	ANKRD33	12	50570935	5	1.624776
rs12368048	ANKRD33	12	50570935	2	2.743409
rs2252518	ACVR1B	12	50667293	8	5.308931
rs2603751	NR4A1	12	50739549	1	0.743854
rs3660	KRT81	12	50966204	6	0.059803
rs6580873	KRT81	12	50968192	4	0.030691
rs2852456	KRT83	12	50994508	5	0.521531
rs2852456	KRT83	12	50994508	8	5.582062
rs2852456	KRT83	12	50994508	3	0.99393
rs1732293	KRT84	12	51060572	1	0.915596
rs1732304	KRT84	12	51065511	3	0.365357
rs1732262	KRT82	12	51074279	3	0.633294
rs2852473	KRT82	12	51074570	1	0.872645

rs2852473	KRT82	12	51074570	4	0.181771
rs2852473	KRT82	12	51074570	5	0.013142
rs2852473	KRT82	12	51074570	8	0.183524
rs2852473	KRT82	12	51074570	2	0.378552
rs2658658	KRT82	12	51075195	2	2.73097
rs2293639	KRT75	12	51104124	4	1.261627
rs2293639	KRT75	12	51104124	3	0.744535
rs2293639	KRT75	12	51104124	1	2.268425
rs188462	KRT75	12	51104337	7	0.977435
rs188462	KRT75	12	51104337	5	0.70449
rs1798640	KRT75	12	51111630	1	2.004204
rs1798640	KRT75	12	51111630	2	1.630144
rs1798640	KRT75	12	51111630	4	0.098864
rs1798640	KRT75	12	51111630	8	1.857673
rs298109	KRT75	12	51114085	8	2.402372
rs298109	KRT75	12	51114085	1	0.537002
rs583678	KRT6C	12	51148881	1	1.130961
rs583678	KRT6C	12	51148881	2	0.791637
rs583678	KRT6C	12	51148881	4	0.337099
rs11608915	KRT6C	12	51152327	8	3.61522
rs11608915	KRT6C	12	51152327	4	3.021112
rs11608915	KRT6C	12	51152327	2	4.206581
rs11608915	KRT6C	12	51152327	1	3.394094
rs11170164	KRT5	12	51199935	2	1.92458
rs12821071	KRT5	12	51200478	4	0.702459
rs12821071	KRT5	12	51200478	7	7.517198
rs665470	KRT71	12	51232765	3	0.368402
rs665522	KRT71	12	51232810	6	0.181147
rs628819	KRT71	12	51233157	3	0.965337
rs628819	KRT71	12	51233157	6	1.779533
rs13121	KRT74	12	51245972	8	0.887696
rs13121	KRT74	12	51245972	1	0.513214
rs584436	KRT74	12	51246335	3	0.104513
rs584436	KRT74	12	51246335	2	0.628073
rs584436	KRT74	12	51246335	6	1.201708
rs584436	KRT74	12	51246335	1	1.886283
rs11170183	KRT72	12	51267709	4	5.569502
rs11170183	KRT72	12	51267709	3	2.373321
rs11170183	KRT72	12	51267709	5	3.180212
rs12818575	KRT72	12	51270887	3	0.218943
rs12833456	KRT72	12	51272454	3	0.165798
rs2634041	KRT2	12	51331893	5	1.158835
rs2634041	KRT2	12	51331893	4	1.751577
rs2634041	KRT2	12	51331893	2	1.633736
rs2634041	KRT2	12	51331893	6	3.536285
rs2634041	KRT2	12	51331893	1	1.103794
rs2634041	KRT2	12	51331893	3	1.638806

rs10783528	KRT77	12	51374751	6	2.863535
rs10783528	KRT77	12	51374751	1	2.312205
rs1567759	KRT77	12	51377833	5	1.752562
rs1567759	KRT77	12	51377833	7	3.702576
rs1567759	KRT77	12	51377833	1	4.166288
rs668313	KRT77	12	51377943	7	1.52202
rs10876328	LOC400036	12	51412714	8	2.118121
rs10876328	LOC400036	12	51412714	1	3.03151
rs10876328	LOC400036	12	51412714	5	2.219734
rs2280481	KRT76	12	51448473	5	2.277922
SNP12-51471759	KRT3	12	51471759	4	1.169033
rs4432093	KRT3	12	51472389	4	2.263016
rs7956809	KRT4	12	51488450	3	0.577898
rs7959052	KRT4	12	51492024	1	1.511598
SNP12-51493895	KRT4	12	51493895	3	1.281346
SNP12-51493895	KRT4	12	51493895	1	6.314938
rs2070910	KRT8	12	51585102	4	1.277264
rs2070910	KRT8	12	51585102	3	0.529131
rs12816417	TENC1	12	51743885	5	0.970224
rs12816417	TENC1	12	51743885	3	0.380288
rs12816417	TENC1	12	51743885	2	5.082938
rs7398691	MFSD5	12	51932152	7	0.176455
rs6580942	ESPL1	12	51948891	5	0.393281
rs6580942	ESPL1	12	51948891	8	5.274025
rs6580942	ESPL1	12	51948891	3	0.861473
rs6580942	ESPL1	12	51948891	4	0.228376
rs6580942	ESPL1	12	51948891	6	0.214367
rs1318648	ESPL1	12	51956812	6	0.314101
rs1318648	ESPL1	12	51956812	4	0.083262
rs1318648	ESPL1	12	51956812	5	0.204239
rs1318648	ESPL1	12	51956812	8	0.230862
rs1318648	ESPL1	12	51956812	2	5.079337
SNP12-51987127	C12orf10	12	51987127	7	3.90776
SNP12-51987127	C12orf10	12	51987127	3	3.489562
SNP12-51987127	C12orf10	12	51987127	8	2.191677
SNP12-51987127	C12orf10	12	51987127	2	5.935968
SNP12-51987127	C12orf10	12	51987127	5	2.867713
SNP12-51987127	C12orf10	12	51987127	4	2.710121
SNP12-51987127	C12orf10	12	51987127	1	3.868971
rs12427129	HOXC11	12	52653957	1	3.221024
rs12427129	HOXC11	12	52653957	6	0.82062
rs2241820	HOXC9	12	52680764	3	0.6688
rs2241820	HOXC9	12	52680764	4	1.221783
rs2241820	HOXC9	12	52680764	1	1.048648
rs2241820	HOXC9	12	52680764	7	0.541319
rs4016778	HOXC5	12	52714638	8	2.563285
rs4016778	HOXC5	12	52714638	3	0.348481

rs2071450	HOXC5	12	52714799	7	0.411844
rs2071450	HOXC5	12	52714799	2	1.397559
rs2071450	HOXC5	12	52714799	1	0.779615
GA010838	HOXC4	12	52735926	8	5.170058
GA010838	HOXC4	12	52735926	3	0.401789
rs1140681	CBX5	12	52937738	1	4.015302
rs1140681	CBX5	12	52937738	7	0.84702
rs1140681	CBX5	12	52937738	4	1.393999
rs1140681	CBX5	12	52937738	8	1.364421
rs884510	PDE1B	12	53258566	2	2.177276
rs884510	PDE1B	12	53258566	8	3.727477
rs884510	PDE1B	12	53258566	7	2.421057
rs884510	PDE1B	12	53258566	1	3.716003
rs1048371	MUCL1	12	53534624	7	0.61337
rs1048371	MUCL1	12	53534624	2	2.221673
rs1048371	MUCL1	12	53534624	4	1.908392
rs1048371	MUCL1	12	53534624	1	1.945757
rs1801876	KIAA0748	12	53630291	2	1.054577
rs1801876	KIAA0748	12	53630291	3	1.241972
rs1801876	KIAA0748	12	53630291	1	1.983521
rs7301705	OR6C74	12	53927342	1	1.403827
rs7132076	LOC653441	12	54094736	4	6.249505
rs7132076	LOC653441	12	54094736	2	5.065926
rs7304753	OR6C68	12	54172756	6	0.145374
rs10876838	OR10P1	12	54317205	3	0.682153
rs10876838	OR10P1	12	54317205	7	1.726553
rs10876838	OR10P1	12	54317205	6	0.567885
rs10876838	OR10P1	12	54317205	1	0.645365
rs7970885	OR10P1	12	54317540	3	0.792214
rs11171631	OR10P1	12	54317878	5	3.932609
rs17117883	ITGA7	12	54368292	1	5.038906
rs17117883	ITGA7	12	54368292	3	0.682206
rs17117883	ITGA7	12	54368292	6	1.099517
rs17117883	ITGA7	12	54368292	7	3.938039
rs1800974	ITGA7	12	54375624	5	5.228092
rs1800974	ITGA7	12	54375624	3	0.982419
rs1800974	ITGA7	12	54375624	7	1.249434
rs750667	LOC440104	12	54514771	6	0.074077
rs750667	LOC440104	12	54514771	4	0.402225
rs750667	LOC440104	12	54514771	7	0.578631
rs750667	LOC440104	12	54514771	1	0.014543
rs750667	LOC440104	12	54514771	2	0.097836
rs750667	LOC440104	12	54514771	5	0.387902
rs1136082	DGKA	12	54621374	2	1.376897
rs1136082	DGKA	12	54621374	8	1.345397
rs1052165	SILV	12	54637613	2	3.853297
rs1052165	SILV	12	54637613	3	0.572078

rs1052165	SILV	12	54637613	8	1.330695
rs1131017	RPS26	12	54722196	1	4.086179
rs1131017	RPS26	12	54722196	5	0.240827
rs1131017	RPS26	12	54722196	6	0.407324
SNP12-54773468	ERBB3	12	54773468	1	3.805029
rs2271192	FAM62A	12	54817421	4	0.992499
rs2271192	FAM62A	12	54817421	1	2.855327
rs17118409	SLC39A5	12	54917031	3	0.115316
rs11171806	IL23A	12	55019798	3	0.514673
rs2066807	STAT2	12	55026949	3	1.245702
rs2291739	TIMELESS	12	55100920	7	3.481946
rs2291739	TIMELESS	12	55100920	6	0.656272
rs2291739	TIMELESS	12	55100920	5	0.337168
rs2291739	TIMELESS	12	55100920	4	2.722976
rs774027	TIMELESS	12	55108645	4	3.856829
rs774027	TIMELESS	12	55108645	7	1.426926
rs774027	TIMELESS	12	55108645	5	0.704792
rs774027	TIMELESS	12	55108645	2	5.396734
rs774027	TIMELESS	12	55108645	6	7.806148
rs2371455	MIP	12	55130160	3	0.739263
rs2371455	MIP	12	55130160	4	2.872543
rs3809125	MIP	12	55130616	4	1.047242
rs3809125	MIP	12	55130616	3	1.276376
rs3809125	MIP	12	55130616	2	2.605582
rs2657880	SPRYD4	12	55150037	3	0.074779
rs2638315	GLS2	12	55151323	3	0.719342
rs2638315	GLS2	12	55151323	6	0.280742
rs2638315	GLS2	12	55151323	7	1.038606
GA010905	NACA	12	55394470	3	0.659421
GA010905	NACA	12	55394470	5	1.13743
GA010905	NACA	12	55394470	6	6.539768
GA010905	NACA	12	55394470	7	0.240576
rs2279743	LOC390332	12	55663776	1	1.625867
rs2279743	LOC390332	12	55663776	8	0.935662
rs3741578	ZBTB39	12	55680902	6	0.809699
rs3741578	ZBTB39	12	55680902	1	6.752812
rs748894	ZBTB39	12	55684561	8	0.625378
rs12814239	LRP1	12	55855745	8	5.391882
SNP12-55910968	SHMT2	12	55910968	6	1.001808
rs3204635	STAC3	12	55923860	1	0.467547
rs3204635	STAC3	12	55923860	5	0.371212
rs4075325	R3HDM2	12	55936107	1	0.331562
rs4075325	R3HDM2	12	55936107	5	0.078304
rs4075325	R3HDM2	12	55936107	2	3.297802
rs4075325	R3HDM2	12	55936107	8	4.601325
rs4075325	R3HDM2	12	55936107	4	2.51267
rs2228225	GLI1	12	56145698	5	0.065921

rs2228225	GLI1	12	56145698	1	0.690054
rs2228225	GLI1	12	56145698	2	1.143109
rs2228225	GLI1	12	56145698	3	0.612701
rs2228225	GLI1	12	56145698	8	0.746206
rs2228225	GLI1	12	56145698	4	0.031144
rs2228226	GLI1	12	56152088	3	1.161924
rs2228226	GLI1	12	56152088	1	1.079742
rs2228226	GLI1	12	56152088	5	0.400665
rs2228226	GLI1	12	56152088	8	0.358375
rs11544238	ARHGAP9	12	56156422	3	0.038312
rs11544238	ARHGAP9	12	56156422	8	0.690948
rs11544238	ARHGAP9	12	56156422	1	0.522391
rs11544238	ARHGAP9	12	56156422	7	1.100917
rs34160414	ARHGAP9	12	56157704	7	1.62262
rs2277319	PIP4K2C	12	56280946	1	5.192119
rs923828	SLC26A10	12	56301761	6	2.226824
rs923828	SLC26A10	12	56301761	1	2.118718
rs774898	B4GALNT1	12	56313053	5	2.874983
rs799265	OS9	12	56398456	1	3.153944
rs799265	OS9	12	56398456	4	0.293715
rs799265	OS9	12	56398456	3	0.678308
GA029106	CENTG1	12	56412501	3	0.298461
GA029106	CENTG1	12	56412501	6	5.081379
GA029106	CENTG1	12	56412501	4	1.17153
rs2291617	FAM119B	12	56452670	5	1.212705
rs923829	FAM119B	12	56460573	4	1.061796
rs923829	FAM119B	12	56460573	3	0.238869
rs923829	FAM119B	12	56460573	5	1.020108
rs3751325	XRCC6BP1	12	56621893	5	0.77486
rs3751325	XRCC6BP1	12	56621893	1	6.180772
rs3751325	XRCC6BP1	12	56621893	4	2.783959
rs3751325	XRCC6BP1	12	56621893	3	0.364287
rs11172796	LRIG3	12	57565927	5	5.868339
rs11172796	LRIG3	12	57565927	1	6.903915
rs11172796	LRIG3	12	57565927	3	0.694044
rs4432061	SLC16A7	12	58460155	4	1.807845
rs4432061	SLC16A7	12	58460155	1	6.353131
rs10877783	LOC645000	12	60706430	1	1.377977
rs2044846	USP15	12	61064332	4	7.111801
rs2044846	USP15	12	61064332	3	0.598596
rs2044846	USP15	12	61064332	5	1.227071
rs2044846	USP15	12	61064332	8	7.375789
rs2044846	USP15	12	61064332	6	4.49891
rs11174557	MON2	12	61265483	6	0.794178
rs11174557	MON2	12	61265483	1	3.765646
rs10877885	C12orf61	12	61282251	4	5.280571
rs10877901	PPM1H	12	61324905	7	6.794378

rs1532394	PPM1H	12	61325946	5	0.236639
rs1532394	PPM1H	12	61325946	8	5.107744
rs17098126	PPM1H	12	61326530	2	6.085851
rs772580	PPM1H	12	61614824	7	0.748389
rs772580	PPM1H	12	61614824	3	0.111539
rs12426017	hCG_1820764	12	61682539	4	3.359567
rs12426017	hCG_1820764	12	61682539	1	3.56669
rs1042615	AVPR1A	12	61830476	4	0.579758
rs1042615	AVPR1A	12	61830476	3	1.331288
rs1042615	AVPR1A	12	61830476	7	0.166926
rs1042615	AVPR1A	12	61830476	5	0.311487
rs10878073	DPY19L2	12	62348290	6	1.561344
rs10878073	DPY19L2	12	62348290	7	1.051954
SNP12-62771420	SRGAP1	12	62771420	1	4.313215
rs1002392	C12orf56	12	62955357	1	0.148375
rs11609829	LOC729435	12	63018561	3	1.07761
rs11609829	LOC729435	12	63018561	2	0.912352
rs11609829	LOC729435	12	63018561	8	1.123053
rs11609829	LOC729435	12	63018561	4	0.557415
SNP12-63033100	C12orf56	12	63033100	6	1.291018
rs7486100	TBK1	12	63162054	4	0.167996
rs7486100	TBK1	12	63162054	6	0.851332
rs7486100	TBK1	12	63162054	5	0.384545
rs1061743	GNS	12	63394427	8	0.340645
rs17764513	KIAA0984	12	63556483	5	0.157931
rs7311657	KIAA0984	12	63556703	5	0.586074
rs7311657	KIAA0984	12	63556703	7	0.617821
rs7311657	KIAA0984	12	63556703	8	0.171953
rs3741742	KIAA0984	12	63559303	5	0.022501
rs3741742	KIAA0984	12	63559303	7	0.292226
rs3741742	KIAA0984	12	63559303	8	0.092636
rs7316024	MSRB3	12	64143822	2	0.915541
rs7316024	MSRB3	12	64143822	7	0.543992
rs7316024	MSRB3	12	64143822	1	0.171256
rs7316024	MSRB3	12	64143822	4	0.073772
SNP12-64644388	HMGA2	12	64644388	3	0.802877
rs1152888	IRAK3	12	64891495	1	3.449309
rs3741604	HELB	12	64982677	4	0.164208
rs3741604	HELB	12	64982677	5	0.328282
rs17224845	MDM1	12	67006602	4	4.695107
rs2287477	LOC729376	12	67112000	5	0.318637
rs2259588	NUP107	12	67369072	6	0.289421
rs7971479	CPM	12	67533750	7	0.026387
rs7971479	CPM	12	67533750	3	0.067046
rs7971479	CPM	12	67533750	1	3.400357
rs9817	CPM	12	67534215	3	0.276254
rs9817	CPM	12	67534215	1	3.107582

rs9817	CPM	12	67534215	7	0.71328
rs10082893	CPM	12	67551845	7	0.287312
rs10082893	CPM	12	67551845	1	3.233964
rs12184493	CPSF6	12	67950119	1	4.985015
rs3177122	CCT2	12	68281614	3	0.31128
rs28203	LRRC10	12	68289706	1	0.884105
rs17225440	BEST3	12	68334286	6	0.552244
rs17225440	BEST3	12	68334286	1	1.753412
rs17225440	BEST3	12	68334286	8	0.485653
rs17225440	BEST3	12	68334286	3	1.163479
rs17225440	BEST3	12	68334286	5	0.464964
rs775419	BEST3	12	68369170	5	0.656511
rs775419	BEST3	12	68369170	1	1.357021
rs775419	BEST3	12	68369170	2	0.616005
rs11177879	RAB3IP	12	68497094	1	0.109915
rs11177879	RAB3IP	12	68497094	8	0.94212
rs11177879	RAB3IP	12	68497094	2	3.008127
rs11177879	RAB3IP	12	68497094	4	0.048169
rs11177879	RAB3IP	12	68497094	5	0.876407
rs1882194	RAB3IP	12	68501353	4	0.431013
rs1466711	RAB3IP	12	68502227	7	0.422406
rs1466711	RAB3IP	12	68502227	3	0.408813
rs1466711	RAB3IP	12	68502227	5	4.770273
rs1466711	RAB3IP	12	68502227	4	0.73358
rs1466711	RAB3IP	12	68502227	6	5.347136
rs3752703	PTPRB	12	69214883	7	0.496364
rs3752703	PTPRB	12	69214883	4	0.200884
rs3752703	PTPRB	12	69214883	3	0.891101
rs3752703	PTPRB	12	69214883	5	1.165583
rs1051344	TSPAN8	12	69805363	3	1.235428
rs1051344	TSPAN8	12	69805363	5	0.813615
rs1051344	TSPAN8	12	69805363	4	1.200942
rs1051344	TSPAN8	12	69805363	1	1.864723
rs3763978	TSPAN8	12	69819801	1	2.199362
rs3763978	TSPAN8	12	69819801	5	0.031233
rs3763978	TSPAN8	12	69819801	3	0.987005
SNP12-6982421	TSPAN8	12	69824218	2	3.333999
rs3741688	RAB21	12	70450662	1	5.547964
rs11609372	TBC1D15	12	70604075	1	5.371749
rs17110747	TPH2	12	70712221	5	1.474019
rs11180483	GLIPR1L2	12	74071367	3	1.868843
rs11180483	GLIPR1L2	12	74071367	7	2.353752
rs11180483	GLIPR1L2	12	74071367	5	2.132334
rs11180483	GLIPR1L2	12	74071367	4	2.461626
rs17042412	LOC441642	12	75518380	5	0.053729
rs1587110	LOC727777	12	75797341	1	0.959082
rs1587110	LOC727777	12	75797341	7	0.846595

rs1587110	LOC727777	12	75797341	6	0.747352
rs7958377	E2F7	12	75940098	2	3.445538
rs7958377	E2F7	12	75940098	1	0.060213
rs310831	E2F7	12	75943472	1	0.312641
rs310831	E2F7	12	75943472	2	6.83695
rs366527	NAV3	12	77066830	6	0.310546
rs9971904	NAV3	12	77123022	7	0.595203
rs3803039	NAV3	12	77129759	4	3.30489
rs10778677	LOC338756	12	78464026	3	5.22211
rs10778679	LOC338756	12	78476708	3	0.342245
rs10862089	NULL	12	79223606	5	0.156764
rs10862089	NULL	12	79223606	7	0.001432
rs1551118	C12orf64	12	79285561	6	5.722969
rs1551118	C12orf64	12	79285561	8	0.738733
rs1551122	C12orf64	12	79289931	6	1.438544
rs1551122	C12orf64	12	79289931	5	0.108734
rs1551122	C12orf64	12	79289931	4	0.418195
rs2717477	C12orf64	12	79295944	6	1.091733
rs1153046	PTPRQ	12	79598034	5	2.147363
rs1153046	PTPRQ	12	79598034	1	2.713224
rs1153046	PTPRQ	12	79598034	3	1.136069
rs3121	MYF6	12	79627052	1	0.459845
rs1047185	MYF6	12	79627275	2	6.579528
rs11114581	MYF5	12	79637013	3	5.014896
rs17552524	TMTC2	12	82050990	5	0.04208
rs17183577	SLC6A15	12	83801692	8	0.26549
rs3765044	LRRIQ1	12	83962630	2	3.296232
rs3765044	LRRIQ1	12	83962630	5	0.500847
rs3765044	LRRIQ1	12	83962630	4	2.914958
rs3765044	LRRIQ1	12	83962630	3	0.178139
rs7974418	LRRIQ1	12	83973596	5	0.274988
rs7974418	LRRIQ1	12	83973596	6	0.181187
rs7974418	LRRIQ1	12	83973596	4	1.344749
rs7974418	LRRIQ1	12	83973596	8	0.035934
rs7974418	LRRIQ1	12	83973596	1	3.453042
SNP12-83990854	LRRIQ1	12	83990854	2	2.048128
SNP12-83990854	LRRIQ1	12	83990854	3	0.802956
SNP12-84039650	LRRIQ1	12	84039650	5	0.159964
rs7397266	PAMCI	12	84723066	1	0.129926
rs7397266	PAMCI	12	84723066	2	1.032165
rs1907699	KITLG	12	87414108	4	0.762345
rs1907699	KITLG	12	87414108	8	5.999954
rs7973791	KERA	12	89968856	4	1.876391
rs12694	BTG1	12	91063721	3	0.641824
rs12694	BTG1	12	91063721	6	1.100048
rs12694	BTG1	12	91063721	2	3.24804
rs12580153	CLLU1	12	91342613	1	1.220835

rs7303412	CLLU1	12	91347241	5	0.381222
rs7303412	CLLU1	12	91347241	6	1.259483
rs7303412	CLLU1	12	91347241	4	1.133668
rs6538329	CLLU1	12	91348724	4	2.998798
rs6538329	CLLU1	12	91348724	5	0.306142
rs6538329	CLLU1	12	91348724	6	0.30601
rs7308198	CLLU1	12	91348829	3	0.865472
rs7308198	CLLU1	12	91348829	4	1.314567
rs7308198	CLLU1	12	91348829	6	0.300343
rs7308198	CLLU1	12	91348829	5	0.370478
rs7308198	CLLU1	12	91348829	2	2.738691
rs2036553	PLEKHG7	12	91655334	3	0.139555
rs2036553	PLEKHG7	12	91655334	1	1.773193
SNP12-91674233	PLEKHG7	12	91674233	1	0.199334
SNP12-91674233	PLEKHG7	12	91674233	3	0.125517
rs17790340	PLEKHG7	12	91689023	5	7.357733
rs10507017	PLEKHG7	12	91689067	5	0.283816
rs17194402	PLEKHG7	12	91689750	5	1.259017
rs17194451	EEA1	12	91782796	6	6.422246
rs17194451	EEA1	12	91782796	3	0.643731
rs17194451	EEA1	12	91782796	2	3.259201
rs1498708	SOCS2	12	92488095	6	0.529506
rs1498708	SOCS2	12	92488095	1	3.363524
rs1498708	SOCS2	12	92488095	7	0.965371
SNP12-93353232	CCDC41	12	93353232	1	5.865001
rs7139313	LOC283398	12	93466354	1	3.777523
rs7139313	LOC283398	12	93466354	5	4.175623
rs7139313	LOC283398	12	93466354	3	3.301719
rs7139313	LOC283398	12	93466354	6	4.039619
rs7139313	LOC283398	12	93466354	2	3.696616
rs7139313	LOC283398	12	93466354	8	3.464715
rs10859724	LOC283398	12	93467678	8	5.994382
rs10859724	LOC283398	12	93467678	4	0.168022
rs10859724	LOC283398	12	93467678	5	3.253375
rs10859724	LOC283398	12	93467678	1	5.588622
rs10859725	LOC283398	12	93468003	4	0.053423
rs10859725	LOC283398	12	93468003	1	0.183795
rs10859725	LOC283398	12	93468003	5	0.585868
rs17022649	LOC283398	12	93468108	6	0.823155
rs17022649	LOC283398	12	93468108	3	0.263482
rs4761630	LOC283398	12	93468312	5	1.293184
rs4761630	LOC283398	12	93468312	1	2.337434
rs1290004	TMCC3	12	93486922	1	5.108474
rs1290004	TMCC3	12	93486922	3	1.068449
rs1290005	TMCC3	12	93488288	7	2.915353
rs1290005	TMCC3	12	93488288	6	2.564744
rs1290005	TMCC3	12	93488288	5	1.362342

rs1290005	TMCC3	12	93488288	4	3.466549
rs1290005	TMCC3	12	93488288	1	5.506721
rs1290005	TMCC3	12	93488288	8	4.870172
rs11107606	TMCC3	12	93488496	3	2.34371
rs11107606	TMCC3	12	93488496	8	1.072799
rs11107606	TMCC3	12	93488496	1	2.848378
rs11107606	TMCC3	12	93488496	6	1.453956
rs11107606	TMCC3	12	93488496	7	0.519823
rs1290910	TMCC3	12	93489189	6	1.190121
rs1290910	TMCC3	12	93489189	5	0.691901
rs1290910	TMCC3	12	93489189	1	5.627155
rs1290910	TMCC3	12	93489189	8	0.547336
rs1290910	TMCC3	12	93489189	4	2.947986
rs2270893	TMCC3	12	93496421	4	1.114258
rs2270893	TMCC3	12	93496421	7	0.175975
rs2270893	TMCC3	12	93496421	3	0.4955
rs2306884	FGD6	12	94126803	3	2.210901
rs2306884	FGD6	12	94126803	2	4.3997
rs17023760	FGD6	12	94127229	7	0.119399
rs6538617	FGD6	12	94135272	8	2.677023
rs6538617	FGD6	12	94135272	1	2.888999
rs6538617	FGD6	12	94135272	7	0.27016
rs6538617	FGD6	12	94135272	5	0.172587
rs3812814	USP44	12	94451168	6	1.226621
rs3812813	USP44	12	94451893	5	3.913902
rs3812813	USP44	12	94451893	3	0.607454
rs17024783	SNRPF	12	94776964	1	0.05844
rs7960706	CCDC38	12	94785078	6	0.45589
rs7960706	CCDC38	12	94785078	4	3.936638
rs2117914	CCDC38	12	94790166	4	1.100287
rs2117914	CCDC38	12	94790166	5	0.1391
rs2230885	HAL	12	94892031	3	0.805405
rs2230885	HAL	12	94892031	1	4.218284
rs2230885	HAL	12	94892031	4	0.427977
rs2160501	C12orf55	12	95452103	1	2.418177
rs2160501	C12orf55	12	95452103	2	1.973425
rs2160502	C12orf55	12	95452117	2	0.368204
rs2160502	C12orf55	12	95452117	4	0.507086
rs2160502	C12orf55	12	95452117	1	0.22407
SNP12-95569612	C12orf63	12	95569612	7	1.721384
rs7978894	C12orf63	12	95660349	8	1.970084
rs7978894	C12orf63	12	95660349	1	2.364734
rs829863	LOC121456	12	97372827	1	0.843861
rs829863	LOC121456	12	97372827	3	0.826971
rs17028572	IKIP	12	97543761	4	3.833976
SNP12-98164290	ANKS1B	12	98164290	6	0.223601
SNP12-98164290	ANKS1B	12	98164290	4	0.103851

SNP12-98164290	ANKS1B	12	98164290	1	0.043714
rs3751323	ANKS1B	12	98164559	1	4.354478
rs3751323	ANKS1B	12	98164559	4	2.988887
rs3751323	ANKS1B	12	98164559	6	0.363148
rs1552759	ANKS1B	12	98164688	3	0.012769
rs1552759	ANKS1B	12	98164688	4	0.809799
rs1552759	ANKS1B	12	98164688	1	3.490188
rs1552759	ANKS1B	12	98164688	6	0.007935
rs11109969	FAM71C	12	98566294	4	1.312905
rs11613157	FAM71C	12	98567776	4	0.89413
SNP12-99006997	UHRF1BP1L	12	99006997	6	0.527422
rs7307415	DEPDC4	12	99181595	8	9.132805
rs7307415	DEPDC4	12	99181595	5	0.269799
rs33968174	SCYL2	12	99232498	4	0.290868
SNP12-99542192	GAS2L3	12	99542192	7	0.759619
rs1045603	GAS2L3	12	99542292	7	0.858185
rs1842887	LOC728467	12	99986949	2	3.186545
rs1842887	LOC728467	12	99986949	6	0.964683
rs1842887	LOC728467	12	99986949	8	3.003609
rs1842887	LOC728467	12	99986949	1	3.894566
rs1849710	TMEM16D	12	100046209	8	4.512396
rs1849710	TMEM16D	12	100046209	6	0.23761
rs2671444	SLC5A8	12	100076206	7	0.48087
rs2671444	SLC5A8	12	100076206	1	1.927431
rs9634221	SLC5A8	12	100086071	5	1.051628
rs4764643	UTP20	12	100217665	7	0.963433
rs4764643	UTP20	12	100217665	8	0.671153
rs4764643	UTP20	12	100217665	3	0.129199
rs3817552	MYBPC1	12	100569294	8	7.971531
rs3817552	MYBPC1	12	100569294	6	1.893638
rs3817552	MYBPC1	12	100569294	1	5.330707
rs731682	ASCL1	12	101876779	5	0.678812
rs10778257	C12orf42	12	102396302	7	0.286709
rs697212	STAB2	12	102624747	7	2.037893
rs2292688	STAB2	12	102660446	7	1.394535
rs2292688	STAB2	12	102660446	1	5.370501
rs11616136	NT5DC3	12	102690225	1	5.280768
rs11616136	NT5DC3	12	102690225	4	3.516036
rs4964830	NT5DC3	12	102693038	1	3.899156
rs6539101	NT5DC3	12	102693112	3	0.217927
rs6539101	NT5DC3	12	102693112	4	0.271845
rs11111761	NT5DC3	12	102693958	7	0.956574
rs11111761	NT5DC3	12	102693958	4	2.185749
rs11111764	NT5DC3	12	102694481	7	0.702137
rs10861097	NT5DC3	12	102694604	7	0.694495
rs10861097	NT5DC3	12	102694604	1	4.650759
rs3751205	NT5DC3	12	102695401	7	6.550654

rs3751205	NT5DC3	12	102695401	4	6.335227
rs1370780	LOC253724	12	102761959	3	1.283426
rs10778292	LOC253724	12	102784417	7	0.330473
rs10778292	LOC253724	12	102784417	3	1.420001
rs10745988	LOC253724	12	102807336	1	0.110275
rs10745988	LOC253724	12	102807336	3	0.129543
rs7298655	LOC253724	12	102811086	3	1.315151
rs7298655	LOC253724	12	102811086	6	0.0549
rs7298655	LOC253724	12	102811086	1	0.702147
rs2583235	LOC253724	12	102811245	7	0.570627
rs9788136	LOC253724	12	102829287	1	3.891221
rs7645	HSP90B1	12	102865233	3	0.821628
rs7645	HSP90B1	12	102865233	8	0.964227
rs7645	HSP90B1	12	102865233	5	0.446384
rs7645	HSP90B1	12	102865233	7	0.308556
rs17035132	GLT8D2	12	102937566	1	2.179745
rs11111979	TXNRD1	12	103204912	1	4.720717
rs11111979	TXNRD1	12	103204912	7	0.457776
rs11111979	TXNRD1	12	103204912	6	0.09461
rs11111979	TXNRD1	12	103204912	5	0.615694
rs11111979	TXNRD1	12	103204912	4	0.882392
rs4964287	TXNRD1	12	103233689	4	7.578656
rs4964287	TXNRD1	12	103233689	5	0.272023
rs4964287	TXNRD1	12	103233689	6	0.363883
rs4964287	TXNRD1	12	103233689	7	0.276289
rs4964287	TXNRD1	12	103233689	8	0.650775
rs11611385	TXNRD1	12	103266440	7	0.303479
rs11611385	TXNRD1	12	103266440	4	0.238592
rs34775780	APPL2	12	104113252	8	0.48861
rs6539247	NUAK1	12	104984032	5	5.531903
rs6539247	NUAK1	12	104984032	8	5.815425
rs6539247	NUAK1	12	104984032	3	0.640961
rs3741883	NUAK1	12	104985068	6	5.146896
rs3741883	NUAK1	12	104985068	2	4.259902
rs3853777	LOC341378	12	105090304	1	0.097705
rs11831752	TCP11L2	12	105220716	4	1.115939
rs11831752	TCP11L2	12	105220716	6	0.391972
rs2252929	TCP11L2	12	105264670	4	0.661783
rs2252929	TCP11L2	12	105264670	2	0.340847
rs2252929	TCP11L2	12	105264670	1	0.049254
rs2252929	TCP11L2	12	105264670	3	0.098659
SNP12-10567924	RFX4	12	105679247	5	0.39882
rs1055332	LOC728705	12	105872755	7	0.238703
rs1043156	MTERFD3	12	105896407	1	4.586616
rs1043156	MTERFD3	12	105896407	3	0.380396
rs2287162	MTERFD3	12	105905035	1	4.299421
rs2287162	MTERFD3	12	105905035	6	4.034196

rs2287162	MTERFD3	12	105905035	4	0.042347
rs2287162	MTERFD3	12	105905035	3	0.324181
rs3809235	CRY1	12	106011199	8	2.652236
rs3809235	CRY1	12	106011199	3	0.332924
rs12303478	BTBD11	12	106575536	1	2.534088
rs12303478	BTBD11	12	106575536	3	0.017142
rs11893	BTBD11	12	106577349	3	0.163162
rs11893	BTBD11	12	106577349	1	2.036196
rs11113472	ASCL4	12	106692715	3	1.256531
rs10861834	LOC728739	12	106821041	5	0.630256
rs10861834	LOC728739	12	106821041	1	0.247741
rs10861834	LOC728739	12	106821041	3	0.158933
rs12579216	SART3	12	107465809	2	2.738259
rs7980427	DAO	12	107805439	2	0.378065
rs10778660	SVOP	12	107829650	5	1.481959
rs11065772	ACACB	12	108102248	3	0.293215
rs2300455	ACACB	12	108107899	7	1.686092
rs2300455	ACACB	12	108107899	8	1.56843
rs2300455	ACACB	12	108107899	4	0.910224
SNP12-10817643	ACACB	12	108176436	4	0.355918
SNP12-10817643	ACACB	12	108176436	1	5.090928
SNP12-10817643	ACACB	12	108176436	5	0.158358
rs2075263	ACACB	12	108188708	4	0.812141
rs2075438	FOXN4	12	108201593	3	0.148121
rs10774641	FOXN4	12	108202080	3	1.041035
rs7295954	KCTD10	12	108372975	7	4.611768
rs877710	MMAB	12	108478359	2	3.741123
rs877710	MMAB	12	108478359	4	0.886509
rs877710	MMAB	12	108478359	7	0.649511
rs877710	MMAB	12	108478359	8	8.559825
rs11067233	MMAB	12	108478591	2	5.365279
rs9593	MMAB	12	108479253	8	4.963033
rs9593	MMAB	12	108479253	2	1.684024
rs9593	MMAB	12	108479253	7	8.155943
rs9593	MMAB	12	108479253	4	6.906627
rs10774775	MMAB	12	108495613	7	0.945817
rs10774775	MMAB	12	108495613	2	2.290234
rs3742037	TRPV4	12	108710762	3	0.322588
rs3742030	TRPV4	12	108736930	1	3.585735
rs17680262	TCHP	12	108838919	6	6.54992
rs7314498	GIT2	12	108854524	7	2.742796
rs7314498	GIT2	12	108854524	1	4.016156
rs7314498	GIT2	12	108854524	3	0.155209
rs11068997	GIT2	12	108867524	1	5.931691
rs2287174	ANKRD13A	12	108958453	1	2.499179
rs34513555	RAD9B	12	109444579	7	1.083454
rs1973505	PPP1CC	12	109644386	3	0.744658

rs1973505	PPP1CC	12	109644386	1	4.958706
rs1973505	PPP1CC	12	109644386	7	0.044109
rs12303008	CCDC63	12	109769214	7	1.066912
rs2301610	MYL2	12	109837939	3	1.521467
rs2301610	MYL2	12	109837939	7	1.432074
rs3803167	CUX2	12	110269969	1	0.610857
rs3803167	CUX2	12	110269969	3	0.613269
rs7398833	CUX2	12	110271275	3	0.276579
rs7398833	CUX2	12	110271275	6	0.95841
rs7398833	CUX2	12	110271275	1	3.393652
rs7398833	CUX2	12	110271275	4	0.534213
rs10849938	FAM109A	12	110284442	3	0.521435
rs10849938	FAM109A	12	110284442	1	3.006722
rs10849938	FAM109A	12	110284442	6	0.978717
rs3184504	SH2B3	12	110368991	8	4.213616
rs3184504	SH2B3	12	110368991	5	0.295541
rs3184504	SH2B3	12	110368991	2	1.841621
rs3184504	SH2B3	12	110368991	3	0.884697
rs3184504	SH2B3	12	110368991	6	0.14749
rs3184504	SH2B3	12	110368991	4	0.1658
rs3184504	SH2B3	12	110368991	1	0.132883
rs695871	ATXN2	12	110521383	1	1.258181
rs695871	ATXN2	12	110521383	4	1.507627
rs34245489	ACAD10	12	110670657	8	0.314999
rs3177647	C12orf47	12	110761959	1	3.772458
rs3177647	C12orf47	12	110761959	4	0.096927
rs7135514	LOC728543	12	110932868	8	4.213221
rs7135514	LOC728543	12	110932868	5	0.323799
rs7295294	C12orf30	12	110949276	1	3.977106
rs3519	NULL	12	111082704	1	4.054058
rs1005902	NULL	12	111152058	5	0.548823
rs1005902	NULL	12	111152058	4	6.649547
rs1005902	NULL	12	111152058	1	2.722488
rs1005902	NULL	12	111152058	6	1.036764
rs1005902	NULL	12	111152058	8	6.995534
rs2240194	RPH3A	12	111803983	4	1.830455
rs4141253	RPH3A	12	111810012	3	0.190522
rs2285933	OAS3	12	111871162	5	0.219677
rs2285933	OAS3	12	111871162	2	4.700678
rs2240188	OAS3	12	111888067	2	3.344286
rs2010549	OAS3	12	111893380	1	6.947328
rs2010549	OAS3	12	111893380	3	0.114754
rs2010549	OAS3	12	111893380	5	0.314876
rs2010549	OAS3	12	111893380	7	0.316018
rs7023	OAS3	12	111895358	2	4.450263
rs7023	OAS3	12	111895358	5	0.587011
rs2072138	OAS2	12	111909276	2	4.38726

rs2072138	OAS2	12	111909276	5	0.167627
rs1293767	OAS2	12	111909537	7	0.621523
rs1293767	OAS2	12	111909537	8	1.193766
rs1293767	OAS2	12	111909537	3	0.153117
rs1293766	OAS2	12	111909665	7	0.592821
rs1293766	OAS2	12	111909665	3	1.098898
rs1293766	OAS2	12	111909665	1	3.144348
rs1293766	OAS2	12	111909665	5	0.653416
rs1293765	OAS2	12	111909876	3	0.815907
rs1293765	OAS2	12	111909876	7	0.545424
rs1293765	OAS2	12	111909876	8	0.316556
rs1293765	OAS2	12	111909876	4	0.375227
rs12425514	OAS2	12	111909920	5	1.603071
rs12425514	OAS2	12	111909920	2	6.362084
rs1293764	OAS2	12	111910062	8	0.217618
rs1293764	OAS2	12	111910062	4	0.438548
rs1293764	OAS2	12	111910062	7	0.316898
rs1293764	OAS2	12	111910062	3	0.762355
rs1293755	OAS2	12	111919833	1	4.471197
rs1293755	OAS2	12	111919833	4	2.282768
rs1293755	OAS2	12	111919833	3	0.507156
rs1293755	OAS2	12	111919833	7	0.447728
rs1293755	OAS2	12	111919833	8	0.693554
rs1293743	OAS2	12	111931955	2	0.961174
rs1293743	OAS2	12	111931955	6	4.888826
rs1293743	OAS2	12	111931955	7	3.918853
rs1293743	OAS2	12	111931955	1	0.764724
rs1293743	OAS2	12	111931955	4	4.746427
rs1293743	OAS2	12	111931955	3	0.890337
rs13311	OAS2	12	111933035	5	0.103399
rs12427377	DTX1	12	112017000	2	1.368976
rs12427377	DTX1	12	112017000	4	0.765261
rs12426056	DTX1	12	112017268	8	1.013482
rs12423570	RASAL1	12	112026481	2	2.09628
rs12423570	RASAL1	12	112026481	8	0.140265
rs12423570	RASAL1	12	112026481	4	0.898873
rs34456143	RASAL1	12	112039329	8	0.540309
rs34456143	RASAL1	12	112039329	4	5.399217
rs34456143	RASAL1	12	112039329	2	5.363894
rs3812826	SDS	12	112315040	2	2.623291
rs2075387	RBM19	12	112766879	7	0.217118
SNP12-11285927	RBM19	12	112859270	1	2.689878
SNP12-11285927	RBM19	12	112859270	6	4.895917
SNP12-11285927	RBM19	12	112859270	8	3.569518
rs12321179	RBM19	12	112868034	6	2.06547
rs12321179	RBM19	12	112868034	1	6.113993
rs10850665	KRTHB5	12	115481578	4	0.702975

SNP12-11565999	C12orf49	12	115659991	3	0.612341
SNP12-11565999	C12orf49	12	115659991	8	6.111263
rs4767452	TMEM118	12	115701524	7	0.49769
rs903772	TMEM118	12	115774984	2	3.417296
rs903772	TMEM118	12	115774984	7	0.452868
rs4076700	FBXW8	12	115867703	8	1.698012
rs4076700	FBXW8	12	115867703	2	4.471141
rs4076700	FBXW8	12	115867703	6	3.468673
rs11068296	FBXW8	12	115951755	6	0.998198
rs11068296	FBXW8	12	115951755	8	3.789843
rs3741467	FBXO21	12	116068449	3	0.187661
rs7955803	KSR2	12	116477447	1	4.753264
rs7955803	KSR2	12	116477447	5	0.233802
rs7955803	KSR2	12	116477447	8	0.243657
rs7955803	KSR2	12	116477447	6	1.860577
rs617641	KSR2	12	116683669	8	0.276334
SNP12-11699357	FLJ20674	12	116993574	7	1.000093
SNP12-11699357	FLJ20674	12	116993574	8	1.166099
SNP12-11699357	FLJ20674	12	116993574	3	0.794437
SNP12-11699357	FLJ20674	12	116993574	4	1.091871
SNP12-11699357	FLJ20674	12	116993574	5	0.54639
rs428073	TAOK3	12	117167134	6	1.206955
rs10850968	SUDS3	12	117339455	7	9.97401
rs7305647	SUDS3	12	117339949	5	0.717306
rs7305647	SUDS3	12	117339949	4	8.035619
rs7305647	SUDS3	12	117339949	3	1.198112
rs1568923	KIAA1853	12	117903986	5	1.151631
rs1568924	KIAA1853	12	117904015	5	1.60592
rs1568924	KIAA1853	12	117904015	3	1.102716
rs1568924	KIAA1853	12	117904015	4	0.932527
SNP12-11805297	KIAA1853	12	118052979	7	0.413588
rs2555272	KIAA1853	12	118079212	3	0.256648
rs3809304	KIAA1853	12	118080126	1	1.070724
rs3809304	KIAA1853	12	118080126	3	0.600326
rs3809304	KIAA1853	12	118080126	7	0.102482
rs1064319	CCDC60	12	118350916	1	2.702408
rs1064319	CCDC60	12	118350916	4	0.820928
rs1064319	CCDC60	12	118350916	3	0.013604
rs784847	CCDC60	12	118463203	5	1.073472
rs278109	CIT	12	118656378	1	4.633065
rs278109	CIT	12	118656378	5	0.024277
rs17442937	CIT	12	118755021	3	0.065877
SNP12-11879833	CIT	12	118798336	5	6.242741
rs2286046	GCN1L1	12	119073543	7	0.17878
rs2286046	GCN1L1	12	119073543	2	4.641958
rs2286046	GCN1L1	12	119073543	1	0.017344
rs4767884	PXN	12	119146360	1	0.205404

rs4767884	PXN	12	119146360	2	1.88456
rs5637	PLA2G1B	12	119247148	7	1.249835
rs5637	PLA2G1B	12	119247148	1	2.818838
rs2522137	MSI1	12	119264314	5	0.422043
rs2522137	MSI1	12	119264314	3	1.087327
rs8903	COX6A1	12	119362913	3	0.911731
rs8903	COX6A1	12	119362913	7	0.540667
rs17431446	GATC	12	119368674	1	4.514714
rs11548264	SFRS9	12	119391715	1	3.832216
rs3088345	RNF10	12	119499224	5	0.133581
rs3088345	RNF10	12	119499224	7	0.708801
rs949443	KIAA0152	12	119609374	1	0.338614
rs949443	KIAA0152	12	119609374	2	5.852875
rs3914	ACADS	12	119659282	7	0.357323
rs3914	ACADS	12	119659282	3	0.557584
rs3914	ACADS	12	119659282	5	0.131422
rs1799958	ACADS	12	119660466	3	0.12884
rs1799958	ACADS	12	119660466	7	0.204615
rs3915	ACADS	12	119661062	3	0.664697
rs3915	ACADS	12	119661062	5	2.925326
rs3915	ACADS	12	119661062	7	0.080177
rs3916	ACADS	12	119661655	1	0.015965
rs3916	ACADS	12	119661655	2	1.421261
rs1169309	HNF1A	12	119923575	5	0.320727
rs1169309	HNF1A	12	119923575	1	4.642653
rs1169309	HNF1A	12	119923575	8	1.499689
rs1169309	HNF1A	12	119923575	7	0.279117
rs1169310	HNF1A	12	119923816	1	4.969739
rs1169310	HNF1A	12	119923816	8	0.536798
rs1169310	HNF1A	12	119923816	7	0.413373
rs1169310	HNF1A	12	119923816	5	5.518805
SNP12-11992464	HNF1A	12	119924646	4	0.507024
rs2258227	C12orf43	12	119938640	3	0.670185
rs2258227	C12orf43	12	119938640	1	4.988211
rs2258227	C12orf43	12	119938640	4	0.016423
rs2258227	C12orf43	12	119938640	5	0.39404
rs17525809	P2RX7	12	120077072	3	2.7987
rs17525809	P2RX7	12	120077072	7	2.266616
rs7958311	P2RX7	12	120089738	5	1.585387
rs1718119	P2RX7	12	120099486	5	0.218837
rs1718119	P2RX7	12	120099486	1	0.031973
rs1063843	CAMKK2	12	120166070	3	0.428768
rs3817190	CAMKK2	12	120196460	7	6.531827
SNP12-12024046	ANAPC5	12	120240467	2	2.186881
rs3751131	FBXL10	12	120364903	5	0.163297
rs3751131	FBXL10	12	120364903	3	0.480135
rs3751131	FBXL10	12	120364903	4	0.621145

rs3741595	ORAI1	12	120563572	3	0.184495
rs3741595	ORAI1	12	120563572	1	2.615955
rs1154510	HPD	12	120779718	4	1.322446
rs2230681	PSMD9	12	120811195	8	8.093091
SNP12-12083923	PSMD9	12	120839231	8	4.880174
SNP12-12083923	PSMD9	12	120839231	3	0.70568
SNP12-12083923	PSMD9	12	120839231	7	0.677491
SNP12-12083923	PSMD9	12	120839231	5	0.667522
rs11043265	WDR66	12	120876421	3	0.781991
SNP12-12088077	WDR66	12	120880778	7	3.184591
SNP12-12088077	WDR66	12	120880778	8	7.050903
SNP12-12088077	WDR66	12	120880778	5	2.644845
SNP12-12088077	WDR66	12	120880778	3	1.050864
SNP12-12088077	WDR66	12	120880778	2	5.283085
SNP12-12089757	WDR66	12	120897579	3	0.450589
rs11060094	LRRC43	12	121240733	2	1.147271
rs11060094	LRRC43	12	121240733	1	0.696197
rs1129167	CLIP1	12	121378456	2	1.408061
rs1129167	CLIP1	12	121378456	7	0.582952
SNP12-12138353	CLIP1	12	121383533	6	0.858479
SNP12-12138353	CLIP1	12	121383533	5	0.893486
SNP12-12162374	KNTC1	12	121623745	3	0.184479
rs2270775	KNTC1	12	121671096	6	0.095029
rs2270775	KNTC1	12	121671096	7	1.486883
rs676404	GPR109A	12	121753258	8	7.667925
rs676404	GPR109A	12	121753258	7	0.018
rs676404	GPR109A	12	121753258	5	1.379067
rs676404	GPR109A	12	121753258	4	0.466853
rs676404	GPR109A	12	121753258	6	0.645075
rs676404	GPR109A	12	121753258	2	1.319429
rs676404	GPR109A	12	121753258	1	0.690829
rs676404	GPR109A	12	121753258	3	2.04348
rs7131791	GPR81	12	121778693	4	1.688654
rs7131791	GPR81	12	121778693	3	1.883154
rs7131791	GPR81	12	121778693	1	2.698823
rs2280424	C12orf65	12	122304447	5	0.089151
rs6633	CDK2AP1	12	122311762	3	0.665387
rs6633	CDK2AP1	12	122311762	8	4.754585
rs6633	CDK2AP1	12	122311762	1	1.665059
rs3825141	SBNO1	12	122360057	5	6.764383
rs6488868	SBNO1	12	122365927	5	4.051943
rs6488868	SBNO1	12	122365927	3	1.249183
rs12322888	SBNO1	12	122391512	3	0.121216
rs28434767	RILPL2	12	122487217	6	0.4736
rs28434767	RILPL2	12	122487217	1	5.339504
rs28434767	RILPL2	12	122487217	5	0.291649
rs28434767	RILPL2	12	122487217	8	0.131525

rs10846532	EIF2B1	12	122671525	8	0.511457
rs7966867	TCTN2	12	122745370	7	0.053312
rs7966867	TCTN2	12	122745370	6	0.960175
rs7966867	TCTN2	12	122745370	1	5.139486
rs11830658	ATP6V0A2	12	122775285	1	1.348553
SNP12-12280851	ATP6V0A2	12	122808510	6	0.190698
SNP12-12283164	DNAH10	12	122831640	7	0.542482
SNP12-12283164	DNAH10	12	122831640	2	2.148592
rs11057355	DNAH10	12	122833656	6	0.851002
rs11057355	DNAH10	12	122833656	2	0.054368
rs11057355	DNAH10	12	122833656	4	0.565128
rs11057355	DNAH10	12	122833656	8	1.000131
rs11057355	DNAH10	12	122833656	7	0.128775
rs11057401	CCDC92	12	122993259	2	1.476016
rs11057401	CCDC92	12	122993259	5	0.532573
rs12318072	FAM101A	12	123364754	8	2.854468
rs904647	FAM101A	12	123365340	8	3.43635
rs904647	FAM101A	12	123365340	3	1.913058
rs904647	FAM101A	12	123365340	5	3.15655
rs904647	FAM101A	12	123365340	4	2.33914
rs10846654	FAM101A	12	123365411	6	2.106074
rs10846654	FAM101A	12	123365411	5	2.464911
rs10846654	FAM101A	12	123365411	8	5.299276
rs10846654	FAM101A	12	123365411	3	2.325651
rs4765540	FAM101A	12	123365595	3	0.030183
rs4765540	FAM101A	12	123365595	6	1.381212
rs4765540	FAM101A	12	123365595	8	3.636371
rs12551	FAM101A	12	123366271	8	4.879814
SNP12-12338736	NCOR2	12	123387369	5	5.511125
rs2229840	NCOR2	12	123392415	4	0.198605
rs2229840	NCOR2	12	123392415	8	1.005112
rs41276688	UBC	12	123965086	7	0.034493
rs13624	UBC	12	123965120	7	0.308094
rs12427062	DHX37	12	124033111	1	1.919353
rs12427062	DHX37	12	124033111	5	0.133645
rs1491893	TMEM132B	12	124400048	7	0.180888
rs1491893	TMEM132B	12	124400048	8	0.615442
rs1491893	TMEM132B	12	124400048	6	0.4699
SNP12-12457008	TMEM132B	12	124570083	7	4.741684
SNP12-12457008	TMEM132B	12	124570083	3	4.225696
SNP12-12457008	TMEM132B	12	124570083	2	3.622654
SNP12-12457008	TMEM132B	12	124570083	5	3.072467
SNP12-12457008	TMEM132B	12	124570083	8	3.749694
SNP12-12457008	TMEM132B	12	124570083	1	4.388525
SNP12-12457008	TMEM132B	12	124570083	6	3.892424
SNP12-12457008	TMEM132B	12	124570083	4	3.253617
rs2240496	TMEM132B	12	124705478	2	2.085311

rs740328	TMEM132B	12	124708795	5	3.350084
rs740330	TMEM132B	12	124708897	5	0.981582
rs1043607	TMEM132B	12	124709299	8	1.028949
rs2302271	LOC728173	12	125484541	4	2.680704
rs4760085	FZD10	12	129215496	4	1.830107
rs1046893	FZD10	12	129215969	6	1.214045
rs1046895	FZD10	12	129215994	6	2.934234
rs1046895	FZD10	12	129215994	7	1.866394
rs28416520	LOC728983	12	129389610	4	0.504231
rs28416520	LOC728983	12	129389610	5	0.665472
rs28416520	LOC728983	12	129389610	2	2.289907
rs10848087	PIWIL1	12	129405118	5	0.804375
rs2277361	RIMBP2	12	129507103	5	1.176169
rs2277361	RIMBP2	12	129507103	4	0.014232
rs1236	STX2	12	129840347	3	0.557924
rs1236	STX2	12	129840347	2	2.36133
rs1236	STX2	12	129840347	8	0.286418
rs1236	STX2	12	129840347	6	5.318069
rs34515698	MMP17	12	130891329	3	0.99925
rs34515698	MMP17	12	130891329	4	0.094372
rs11835665	MMP17	12	130901545	6	7.422127
SNP12-13096751	ULK1	12	130967519	6	2.315645
SNP12-13096751	ULK1	12	130967519	3	0.904806
SNP12-13096751	ULK1	12	130967519	5	0.078084
rs12313437	EP400	12	131128364	2	5.646249
rs12309945	EP400	12	131129207	2	4.755502
rs7484529	EP400	12	131129324	2	3.932737
rs1133690	DDX51	12	131192621	3	0.987517
rs12308155	GALNT9	12	131248434	4	1.943315
rs12308155	GALNT9	12	131248434	3	1.261712
rs12308155	GALNT9	12	131248434	1	0.030557
rs11246991	GALNT9	12	131254068	2	6.046157
rs7486927	GALNT9	12	131254090	1	1.019501
rs7964634	P2RX2	12	131706669	7	0.431621
rs7973007	P2RX2	12	131708982	3	0.515527
rs5744944	POLE	12	131729904	7	0.323643
SNP12-13173059	POLE	12	131730599	5	0.2041
SNP12-13178254	PXMP2	12	131782543	3	0.511309
SNP12-13178254	PXMP2	12	131782543	2	5.14026
SNP12-13178254	PXMP2	12	131782543	8	5.252012
rs7979132	PXMP2	12	131791549	3	0.24277
rs7979132	PXMP2	12	131791549	2	4.697402
rs7979132	PXMP2	12	131791549	8	0.299519
SNP12-13181678	KIAA0692	12	131816783	2	3.264306
rs7134809	GOLGA3	12	131900071	3	0.853071
rs7134809	GOLGA3	12	131900071	1	2.083352
rs7134809	GOLGA3	12	131900071	4	0.486401

rs7134809	GOLGA3	12	131900071	2	0.657768
rs2291256	GOLGA3	12	131903396	1	4.734256
rs2291256	GOLGA3	12	131903396	7	3.357774
rs3741490	CHFR	12	131927981	7	0.07917
rs3741490	CHFR	12	131927981	2	4.965315
rs3741490	CHFR	12	131927981	5	0.576268
rs2306537	CHFR	12	131933768	2	4.604096
rs2306537	CHFR	12	131933768	4	5.75673
rs2306537	CHFR	12	131933768	8	1.019417
rs2306537	CHFR	12	131933768	3	0.96965
rs2306537	CHFR	12	131933768	7	0.998981
rs9888336	ZNF26	12	132073327	3	0.983232
rs9888336	ZNF26	12	132073327	6	0.223018
SNP12-13212806	ZNF84	12	132128064	7	5.843286
rs7961682	ZNF84	12	132128095	7	0.402723
rs601679	ZNF84	12	132145686	3	6.741029
SNP12-13219309	ZNF140	12	132193093	6	0.411368
rs905225	ZNF140	12	132193353	5	0.309212
rs905225	ZNF140	12	132193353	8	7.092015
rs905225	ZNF140	12	132193353	6	0.932129
rs905225	ZNF140	12	132193353	3	0.179743
rs905225	ZNF140	12	132193353	4	4.526696
rs2292029	ZNF10	12	132244186	7	0.637617
rs2292029	ZNF10	12	132244186	8	5.84896
rs2292029	ZNF10	12	132244186	5	0.026068
rs2292029	ZNF10	12	132244186	1	4.40364
SNP12-13227862	ZNF268	12	132278626	7	5.072872
SNP12-13227862	ZNF268	12	132278626	1	3.922742
SNP12-13227862	ZNF268	12	132278626	5	0.44851
SNP12-13227862	ZNF268	12	132278626	8	5.543869
rs9553323	DKFZp686A1627	13	18523340	6	5.536339
rs9553323	DKFZp686A1627	13	18523340	5	5.323467
rs9553323	DKFZp686A1627	13	18523340	4	3.506903
rs9553323	DKFZp686A1627	13	18523340	3	5.068422
rs9553323	DKFZp686A1627	13	18523340	2	4.634592
rs9553323	DKFZp686A1627	13	18523340	1	6.43239
rs4608194	DKFZp686A1627	13	18524818	5	4.990272
rs4608194	DKFZp686A1627	13	18524818	2	3.691737
rs4608194	DKFZp686A1627	13	18524818	4	1.933938
rs4608194	DKFZp686A1627	13	18524818	8	2.194771
rs9512066	LOC642395	13	18680646	1	4.826775
rs9512066	LOC642395	13	18680646	5	4.961741
rs9512066	LOC642395	13	18680646	3	3.884557
rs4112696	LOC645771	13	18841770	3	0.554692
rs4112696	LOC645771	13	18841770	7	0.68567
rs4112696	LOC645771	13	18841770	1	0.762008
rs1057093	MPHOSPH8	13	19118659	5	2.993817

rs1057093	MPHOSPH8	13	19118659	2	5.241718
rs1057093	MPHOSPH8	13	19118659	1	4.054203
rs9578176	MPHOSPH8	13	19122387	1	0.645186
rs9578176	MPHOSPH8	13	19122387	2	0.759861
rs9578176	MPHOSPH8	13	19122387	4	1.062932
rs9508887	PSPC1	13	19254941	7	1.227115
rs9508887	PSPC1	13	19254941	1	0.360693
rs9508887	PSPC1	13	19254941	2	0.19613
rs9508887	PSPC1	13	19254941	4	6.652818
rs7988277	ZMYM5	13	19309701	2	0.807068
rs7988277	ZMYM5	13	19309701	1	0.5893
rs7988277	ZMYM5	13	19309701	7	0.467254
rs7988277	ZMYM5	13	19309701	4	1.113031
SNP13-19323948	ZMYM5	13	19323948	4	6.760339
SNP13-19323948	ZMYM5	13	19323948	1	0.084237
SNP13-19323948	ZMYM5	13	19323948	7	1.84488
SNP13-19323948	ZMYM5	13	19323948	2	6.032827
rs10161995	LOC400099	13	19336935	7	2.637592
rs10161995	LOC400099	13	19336935	1	0.036671
rs10161995	LOC400099	13	19336935	4	6.948872
rs9509372	XPO4	13	20253927	2	5.022347
SNP13-20460832	LATS2	13	20460832	8	6.203898
SNP13-20460948	LATS2	13	20460948	3	0.21053
rs3742221	SAP18	13	20618956	1	4.19675
rs1046530	SAP18	13	20620190	1	3.506828
rs9316143	C13orf3	13	20626012	4	0.037515
rs9316143	C13orf3	13	20626012	5	6.534063
rs3087726	C13orf3	13	20626088	4	0.823393
rs3087726	C13orf3	13	20626088	5	0.104152
rs11147977	C13orf3	13	20627267	4	3.856906
rs11147977	C13orf3	13	20627267	6	2.292968
rs17345690	C13orf3	13	20633998	4	0.9978
rs17345690	C13orf3	13	20633998	5	0.746587
SNP13-20640311	C13orf3	13	20640311	8	5.281552
SNP13-20640311	C13orf3	13	20640311	6	2.449756
SNP13-20640311	C13orf3	13	20640311	4	5.237402
rs4638420	LOC650794	13	20770348	7	0.473164
rs4638420	LOC650794	13	20770348	6	0.315113
rs4638420	LOC650794	13	20770348	4	2.516545
rs9509664	LOC728099	13	20830459	4	0.188261
rs9509664	LOC728099	13	20830459	3	3.640577
rs9509664	LOC728099	13	20830459	6	0.518371
rs4770145	LOC728099	13	20833210	4	0.273836
rs1044069	EFHA1	13	20964934	1	5.433878
rs1800353	SGCG	13	22796509	7	0.401274
rs3751372	SGCG	13	22796816	5	1.117382
rs3751371	SGCG	13	22796934	4	1.77567

rs3751371	SGCG	13	22796934	5	1.908664
rs3751371	SGCG	13	22796934	3	1.032987
rs3751371	SGCG	13	22796934	2	5.666636
rs3829352	SGCG	13	22796975	4	1.251497
rs3829352	SGCG	13	22796975	3	1.464207
rs3829352	SGCG	13	22796975	5	1.150009
rs2737700	SACS	13	22806034	4	5.198041
rs2737700	SACS	13	22806034	5	5.170554
rs2737700	SACS	13	22806034	3	0.241003
rs9550987	TNFRSF19	13	23065505	1	4.162743
rs3751363	TNFRSF19	13	23141200	6	2.1406
rs3751363	TNFRSF19	13	23141200	8	5.06824
rs3751361	TNFRSF19	13	23141360	2	3.605813
rs6490814	TNFRSF19	13	23147957	6	0.087326
rs9580931	SPATA13	13	23775550	4	0.747047
rs9580931	SPATA13	13	23775550	3	0.322512
rs9580931	SPATA13	13	23775550	6	2.145265
SNP13-23793684	C1QTNF9	13	23793684	7	5.722221
rs1822135	PARP4	13	23906630	6	0.740109
rs9511259	PARP4	13	23907099	6	5.042128
rs9511259	PARP4	13	23907099	7	0.387562
rs2722	ATP12A	13	24183768	1	4.856248
rs2722	ATP12A	13	24183768	4	1.109221
rs17081086	ATP12A	13	24183811	1	3.640609
rs17081086	ATP12A	13	24183811	4	1.446088
rs10507338	ATP12A	13	24183914	1	4.81836
rs10507338	ATP12A	13	24183914	4	1.604846
rs1451568	RNF17	13	24265282	1	1.25703
rs1451568	RNF17	13	24265282	7	1.527232
rs3783082	RNF17	13	24326002	2	0.947185
rs3783082	RNF17	13	24326002	7	0.416255
SNP13-24385103	CENPJ	13	24385103	7	1.386699
SNP13-24385103	CENPJ	13	24385103	4	0.725047
SNP13-24385103	CENPJ	13	24385103	2	4.117828
rs7995033	MTMR6	13	24729888	4	0.121308
rs7995033	MTMR6	13	24729888	1	4.758967
rs7335858	ATP8A2	13	25171403	6	2.284965
rs7335858	ATP8A2	13	25171403	5	1.687057
rs1055252	TMEM46	13	25516922	3	0.003235
rs12100154	TMEM46	13	25517547	8	2.141851
rs12100154	TMEM46	13	25517547	2	6.193283
rs12100154	TMEM46	13	25517547	3	0.018045
rs11558132	RNF6	13	25693463	2	1.06107
rs11558132	RNF6	13	25693463	6	0.908182
rs11558132	RNF6	13	25693463	5	0.216235
rs7669	MTIF3	13	26907851	5	1.40997
rs7669	MTIF3	13	26907851	1	5.076824

rs7669	MTIF3	13	26907851	3	0.372403
rs7669	MTIF3	13	26907851	6	8.140965
rs11617897	LNX2	13	27053920	3	0.677743
SNP13-27460605	PRHOXNB	13	27460605	7	0.073031
rs1933437	FLT3	13	27522294	3	0.019799
rs11619230	PAN3	13	27765245	2	2.994739
rs4769636	SLC46A3	13	28172633	3	0.142129
rs4769636	SLC46A3	13	28172633	7	6.496422
rs4769636	SLC46A3	13	28172633	5	8.018923
rs4769636	SLC46A3	13	28172633	2	3.736382
rs4769636	SLC46A3	13	28172633	4	0.797829
rs2987346	KIAA0774	13	28498415	3	0.697124
rs3751336	KIAA0774	13	28753847	8	0.648688
SNP13-28796783	KIAA0774	13	28796783	1	2.202121
rs3011623	SLC7A1	13	28984225	8	0.740162
rs3011624	SLC7A1	13	28985146	8	1.781263
rs17689385	KATNAL1	13	29676781	4	0.620883
rs10161651	KATNAL1	13	29678458	5	0.86867
rs10161651	KATNAL1	13	29678458	4	0.323713
rs17632858	KATNAL1	13	29678588	4	0.29659
rs17632858	KATNAL1	13	29678588	2	1.693896
rs9506275	KATNAL1	13	29679111	5	0.072978
rs9506275	KATNAL1	13	29679111	4	0.611985
rs7328946	KATNAL1	13	29680099	2	4.624394
rs7328946	KATNAL1	13	29680099	4	0.829752
rs6490453	KATNAL1	13	29779573	4	1.25357
rs6490453	KATNAL1	13	29779573	8	1.55015
rs6490453	KATNAL1	13	29779573	3	1.394475
rs6490453	KATNAL1	13	29779573	1	1.068183
rs6490453	KATNAL1	13	29779573	6	0.630705
rs6490453	KATNAL1	13	29779573	2	2.212094
rs1047086	HSPH1	13	30627729	1	1.735716
rs1047086	HSPH1	13	30627729	3	0.511828
rs1047086	HSPH1	13	30627729	7	6.730349
rs17703938	B3GALTL	13	30803498	3	0.409739
rs756910	EEF1DP3	13	31422656	4	0.335518
rs756910	EEF1DP3	13	31422656	5	0.139373
rs17077179	FRY	13	31645654	3	0.114044
rs17077179	FRY	13	31645654	5	1.315596
rs1799943	BRCA2	13	31788572	5	0.335248
rs1799943	BRCA2	13	31788572	4	2.969679
rs1799943	BRCA2	13	31788572	3	0.239659
rs15869	BRCA2	13	31871012	8	0.762488
rs15869	BRCA2	13	31871012	1	4.434159
rs15869	BRCA2	13	31871012	6	0.367288
rs11571836	BRCA2	13	31871439	4	1.643498
rs11571836	BRCA2	13	31871439	7	0.202422

rs7994229	PDS5B	13	32058705	5	6.85705
rs7994229	PDS5B	13	32058705	1	3.942491
rs7994229	PDS5B	13	32058705	8	5.454602
rs648202	KL	13	32533463	1	0.581237
rs770266	STARD13	13	32576065	4	0.441077
SNP13-32602065	STARD13	13	32602065	6	0.260765
rs2274550	NBEA	13	35127873	4	0.410165
rs1045696	DCLK1	13	35243816	4	0.679925
rs2322807	DCLK1	13	35283031	6	7.43507
rs2322807	DCLK1	13	35283031	4	3.114372
rs2296968	SOHLH2	13	35642910	5	2.013781
rs9546784	LOC728591	13	35699256	4	9.789636
rs9546784	LOC728591	13	35699256	1	5.529642
rs9546784	LOC728591	13	35699256	6	1.484146
rs9546784	LOC728591	13	35699256	5	1.70262
rs9546785	LOC728591	13	35699415	6	3.201366
rs9546785	LOC728591	13	35699415	5	1.992393
rs9546785	LOC728591	13	35699415	8	2.537705
rs9546785	LOC728591	13	35699415	1	4.430015
rs9546785	LOC728591	13	35699415	4	1.693265
rs9546897	LOC728591	13	35726237	1	4.825002
rs9546897	LOC728591	13	35726237	5	0.585014
rs9546897	LOC728591	13	35726237	6	0.67922
rs9546897	LOC728591	13	35726237	4	0.147671
rs1998800	LOC728591	13	35755639	8	1.40665
rs1998800	LOC728591	13	35755639	5	0.075541
rs1886856	LOC646871	13	35841777	6	0.300401
rs1886856	LOC646871	13	35841777	5	1.451557
rs1886856	LOC646871	13	35841777	2	3.832701
rs1886856	LOC646871	13	35841777	3	0.773989
rs1886856	LOC646871	13	35841777	4	4.985795
rs10976	RFXAP	13	36301143	1	4.504364
rs10976	RFXAP	13	36301143	7	4.679219
rs1127446	EXOSC8	13	36478139	8	0.167946
rs1127446	EXOSC8	13	36478139	2	0.427454
rs1127446	EXOSC8	13	36478139	4	0.887251
rs1127446	EXOSC8	13	36478139	7	0.32693
rs1127446	EXOSC8	13	36478139	5	4.200342
rs1127446	EXOSC8	13	36478139	1	0.726726
rs1856028	CSNK1A1L	13	36576149	1	3.906044
rs1856028	CSNK1A1L	13	36576149	4	3.546399
rs17773251	CSNK1A1L	13	36576884	3	6.01061
rs17773251	CSNK1A1L	13	36576884	6	3.683056
rs9576175	CSNK1A1L	13	36577268	3	1.001134
rs9576175	CSNK1A1L	13	36577268	4	0.386449
rs9576175	CSNK1A1L	13	36577268	1	0.938811
rs9576176	CSNK1A1L	13	36577535	1	3.042425

rs9576177	CSNK1A1L	13	36577729	1	1.197459
rs9576177	CSNK1A1L	13	36577729	3	1.315913
rs9576178	CSNK1A1L	13	36577776	1	0.278869
rs12583681	TRPC4	13	37255494	4	4.358498
rs7999240	LOC646910	13	37466017	4	0.621569
rs7999240	LOC646910	13	37466017	2	0.91597
rs7999240	LOC646910	13	37466017	8	1.101776
rs7999240	LOC646910	13	37466017	1	2.121048
rs2485783	UFM1	13	37834310	2	4.179258
rs2485783	UFM1	13	37834310	8	5.084849
rs2485783	UFM1	13	37834310	3	4.869584
rs2485783	UFM1	13	37834310	7	0.382807
rs2485783	UFM1	13	37834310	4	4.191046
rs2485783	UFM1	13	37834310	1	4.857592
rs2485783	UFM1	13	37834310	5	4.146133
rs9576597	FREM2	13	38160435	3	0.496603
rs9548505	FREM2	13	38320624	3	0.529965
rs7990956	STOML3	13	38438353	8	0.025537
rs9576697	C13orf23	13	38484343	8	0.267183
rs9576697	C13orf23	13	38484343	6	0.09417
rs9576711	NHLRC3	13	38521621	6	8.533583
rs9576711	NHLRC3	13	38521621	8	5.412198
rs3812883	COG6	13	39127957	3	0.513899
rs3812883	COG6	13	39127957	2	5.338726
rs3783167	MRPS31	13	40221397	7	1.37192
rs3783167	MRPS31	13	40221397	8	1.044221
rs3783167	MRPS31	13	40221397	3	0.862769
rs3783167	MRPS31	13	40221397	4	0.024686
rs9577152	SLC25A15	13	40277272	6	1.783477
rs1056824	ELF1	13	40404884	2	2.43521
rs1056824	ELF1	13	40404884	3	0.67415
rs1056824	ELF1	13	40404884	1	0.846292
rs7324235	DGKH	13	41662564	6	3.562043
rs7324235	DGKH	13	41662564	3	0.487513
rs7324235	DGKH	13	41662564	8	0.101674
rs545602	DGKH	13	41701341	2	4.840586
rs545602	DGKH	13	41701341	3	0.18273
rs545602	DGKH	13	41701341	6	2.043557
rs545602	DGKH	13	41701341	8	3.241995
rs9525605	AKAP11	13	41771782	4	1.246673
rs9315906	AKAP11	13	41775265	4	0.064315
rs9533064	AKAP11	13	41792287	4	0.431903
rs7335649	C13orf30	13	42262859	7	1.054989
rs7335649	C13orf30	13	42262859	1	0.773905
rs7335649	C13orf30	13	42262859	6	2.410728
rs1044856	EPSTI1	13	42360422	8	6.221404
rs1044856	EPSTI1	13	42360422	4	1.519566

rs1044856	EPSTI1	13	42360422	6	0.635028
rs1359184	EPSTI1	13	42367211	2	2.446707
rs1359184	EPSTI1	13	42367211	7	0.489933
rs1359184	EPSTI1	13	42367211	1	0.733159
rs1359184	EPSTI1	13	42367211	4	0.792977
rs3825511	EPSTI1	13	42464373	1	0.808489
rs3825511	EPSTI1	13	42464373	3	0.130879
rs3825511	EPSTI1	13	42464373	6	0.106748
rs3825511	EPSTI1	13	42464373	8	0.459064
rs3825511	EPSTI1	13	42464373	4	1.819052
rs17553284	DNAJC15	13	42495706	8	1.337632
rs17553284	DNAJC15	13	42495706	3	2.59
rs17553284	DNAJC15	13	42495706	6	2.305016
rs17553284	DNAJC15	13	42495706	5	0.909664
rs17553284	DNAJC15	13	42495706	1	2.295093
rs3816311	CCDC122	13	43351783	1	1.233214
rs3816311	CCDC122	13	43351783	3	0.517098
rs3816311	CCDC122	13	43351783	7	1.176097
rs3816311	CCDC122	13	43351783	2	0.245271
rs3816311	CCDC122	13	43351783	8	5.888304
rs11692	TSC22D1	13	43905697	6	0.145999
rs1053948	TSC22D1	13	43906333	3	0.13484
SNP13-44046257	TSC22D1	13	44046257	5	4.329238
rs9534059	KCTD4	13	44666759	3	0.146094
rs9534059	KCTD4	13	44666759	4	4.448516
rs7331090	GTF2F2	13	44739502	3	0.180778
rs7331090	GTF2F2	13	44739502	4	0.263151
rs7997155	SLC25A30	13	44883608	5	0.323182
SNP13-45185374	SPERT	13	45185374	1	3.32654
rs4942433	LOC283514	13	45251691	8	6.119086
rs4942433	LOC283514	13	45251691	3	0.864802
rs4942434	LOC283514	13	45251787	3	0.451688
rs17350833	ZC3H13	13	45435758	3	1.243705
rs17350833	ZC3H13	13	45435758	5	0.651967
rs1087	CPB2	13	45525440	6	2.211912
rs1087	CPB2	13	45525440	4	1.836436
rs1087	CPB2	13	45525440	8	1.208954
rs1087	CPB2	13	45525440	1	0.495851
rs1087	CPB2	13	45525440	2	5.236637
rs940	CPB2	13	45525481	6	1.499099
rs940	CPB2	13	45525481	5	1.533306
rs940	CPB2	13	45525481	3	1.220757
rs1926447	CPB2	13	45527945	6	0.937712
rs1926447	CPB2	13	45527945	1	0.796433
rs1926447	CPB2	13	45527945	5	0.326149
rs1926447	CPB2	13	45527945	2	1.175915
rs1926447	CPB2	13	45527945	4	0.865208

rs1926447	CPB2	13	45527945	3	0.520881
rs2277440	CPB2	13	45536827	8	0.465771
rs9316179	CPB2	13	45539467	4	4.560537
rs9316179	CPB2	13	45539467	6	1.820812
rs9316179	CPB2	13	45539467	1	3.759314
rs9316179	CPB2	13	45539467	8	0.14964
rs7337140	CPB2	13	45539482	5	0.756303
rs7337140	CPB2	13	45539482	3	1.827657
rs7337140	CPB2	13	45539482	2	3.490048
rs3742264	CPB2	13	45546095	4	0.331098
rs3742264	CPB2	13	45546095	1	4.186135
rs3742264	CPB2	13	45546095	6	0.887184
rs2296642	CPB2	13	45554670	5	0.948646
rs1409431	LCP1	13	45599207	7	4.037829
rs17601331	LCP1	13	45603069	5	4.244719
rs2296118	LCP1	13	45654333	1	3.503648
rs1408184	C13orf18	13	45844158	5	0.500976
rs1408184	C13orf18	13	45844158	1	4.658958
rs1408184	C13orf18	13	45844158	8	0.566363
rs6313	HTR2A	13	46367941	5	0.045798
rs6313	HTR2A	13	46367941	3	1.163339
rs8248	LOC647131	13	48447572	2	5.191309
rs8248	LOC647131	13	48447572	7	1.262873
rs8248	LOC647131	13	48447572	3	6.106871
rs8248	LOC647131	13	48447572	8	7.6235
rs9535193	CDADC1	13	48764323	8	0.072896
rs9535193	CDADC1	13	48764323	5	4.080569
rs3751418	CDADC1	13	48764870	5	1.173273
rs3751418	CDADC1	13	48764870	8	0.563036
rs3751418	CDADC1	13	48764870	2	1.587427
rs3087770	CDADC1	13	48765291	8	4.778588
rs7998427	SETDB2	13	48948621	1	0.211417
rs7998427	SETDB2	13	48948621	2	4.206449
rs2031532	PHF11	13	48978848	1	0.173694
rs2031532	PHF11	13	48978848	2	0.297402
rs3033	PHF11	13	49000993	1	0.153788
rs3033	PHF11	13	49000993	2	1.932637
rs9562900	RCBTB1	13	49039363	7	0.889757
rs9526582	ARL11	13	49105221	2	0.789851
rs9526582	ARL11	13	49105221	1	0.525144
rs9526582	ARL11	13	49105221	3	1.446295
rs9526582	ARL11	13	49105221	4	0.29379
rs9526582	ARL11	13	49105221	6	0.01201
rs1127021	KPNA3	13	49194116	4	0.846108
rs9568354	C13orf1	13	49385994	2	3.862104
rs11839292	C13orf1	13	49386112	7	0.456521
rs11839292	C13orf1	13	49386112	3	0.028454

rs11839292	C13orf1	13	49386112	8	2.889083
rs1609571	C13orf1	13	49386856	2	5.477463
rs1063181	C13orf1	13	49408402	4	3.739681
rs2066549	TRIM13	13	49488424	4	0.734063
rs2066549	TRIM13	13	49488424	2	4.264405
rs2066549	TRIM13	13	49488424	3	0.318449
rs2066549	TRIM13	13	49488424	7	0.02466
rs3803200	LOC647166	13	50556357	3	0.606707
rs959952	LOC647166	13	50584110	3	0.375903
rs7318961	LOC341674	13	50634084	3	1.913948
rs17075482	FAM124A	13	50724045	1	5.42915
rs9535646	LOC647174	13	50811709	2	2.257521
rs9535646	LOC647174	13	50811709	5	1.039071
rs9535646	LOC647174	13	50811709	4	0.763728
rs17837214	INTS6	13	50834201	7	0.236242
rs17837216	INTS6	13	50834636	7	0.516001
rs2277445	INTS6	13	50922937	8	0.867004
rs2277445	INTS6	13	50922937	2	1.424904
rs2277445	INTS6	13	50922937	7	0.616028
rs2277445	INTS6	13	50922937	5	0.275123
rs2277445	INTS6	13	50922937	3	0.081023
rs2277445	INTS6	13	50922937	4	0.918087
rs1885854	WDFY2	13	51211196	7	0.626103
rs1885854	WDFY2	13	51211196	4	0.336895
rs7334118	ATP7B	13	51411267	1	3.365443
rs1061472	ATP7B	13	51422489	8	1.177522
rs1061472	ATP7B	13	51422489	1	4.710793
rs17402034	UTP14C	13	51501897	5	10.95165
SNP13-51574276	NEK5	13	51574276	3	1.601736
SNP13-51574276	NEK5	13	51574276	6	0.563081
rs1886540	NEK3	13	51604877	1	4.364955
rs9536067	VPS36	13	51886351	5	3.242629
rs617660	RP11-78J21.1	13	52101390	5	0.848383
rs9536243	LECT1	13	52175640	5	0.86254
rs9536244	LECT1	13	52175647	5	2.638293
rs7330220	LECT1	13	52184951	8	0.167179
rs17053844	OLFM4	13	52523422	4	0.119321
SNP13-59464645	DIAPH3	13	59464645	1	6.042481
rs339531	DIAPH3	13	59485257	4	0.980385
rs9563808	LOC440142	13	59746943	1	0.257446
rs9563808	LOC440142	13	59746943	7	0.25393
rs9563808	LOC440142	13	59746943	5	0.609753
rs9563808	LOC440142	13	59746943	3	0.22376
rs9563808	LOC440142	13	59746943	2	1.93889
rs9528094	LOC440142	13	59747049	8	4.866386
rs9528094	LOC440142	13	59747049	5	4.534335
rs9528094	LOC440142	13	59747049	7	4.521824

rs9528094	LOC440142	13	59747049	3	5.301778
rs3829388	PCDH20	13	60882782	5	0.470512
rs3812872	PCDH20	13	60884919	5	1.416393
rs3812872	PCDH20	13	60884919	6	2.591432
rs3812860	PCDH9	13	66702233	1	5.467533
rs7319217	LOC647277	13	70396781	5	3.911792
rs7319217	LOC647277	13	70396781	3	3.256719
rs7319217	LOC647277	13	70396781	8	3.143134
rs1465895	RP11-11C5.2	13	72199805	7	0.452192
rs1465895	RP11-11C5.2	13	72199805	5	1.153497
rs2165390	DIS3	13	72229945	3	0.616834
rs2165390	DIS3	13	72229945	6	3.778865
rs2165390	DIS3	13	72229945	5	0.923513
rs7332388	DIS3	13	72247360	3	0.383373
rs7332388	DIS3	13	72247360	6	0.366912
rs7332388	DIS3	13	72247360	4	2.384081
rs9565025	LOC730242	13	73059553	3	0.082874
rs9600137	LOC730242	13	73060077	4	1.059291
rs9543429	KLF12	13	73164045	3	0.14196
rs9318219	KLF12	13	73284794	1	3.229507
rs9318219	KLF12	13	73284794	4	0.001592
rs1998571	KLF12	13	73284810	6	0.320774
rs1998571	KLF12	13	73284810	1	3.453905
rs1998574	KLF12	13	73284990	6	0.131596
rs1998574	KLF12	13	73284990	1	3.012578
rs1998574	KLF12	13	73284990	3	0.363706
rs1998574	KLF12	13	73284990	5	0.8469
rs9543534	LOC730245	13	73606697	1	0.29132
rs477274	LOC647288	13	74711555	8	1.872075
rs477274	LOC647288	13	74711555	4	1.246024
rs477274	LOC647288	13	74711555	6	1.583502
rs477274	LOC647288	13	74711555	2	3.761239
rs477274	LOC647288	13	74711555	5	3.860337
rs477274	LOC647288	13	74711555	3	2.180241
rs7327548	TBC1D4	13	74953821	6	5.957108
rs7327548	TBC1D4	13	74953821	7	6.823753
rs7327548	TBC1D4	13	74953821	4	1.094057
rs9565177	LMO7	13	75093404	2	5.068205
rs9565177	LMO7	13	75093404	4	0.425795
SNP13-7529346[illegible]	LMO7	13	75293465	7	0.175681
SNP13-7532540[illegible]	LMO7	13	75325404	4	0.035218
SNP13-7532540[illegible]	LMO7	13	75325404	1	3.644006
SNP13-7532540[illegible]	LMO7	13	75325404	8	6.406438
rs9600566	FLJ35379	13	75343190	3	1.83088
rs9600566	FLJ35379	13	75343190	7	1.137375
rs9600567	FLJ35379	13	75343317	1	0.121394
rs9600567	FLJ35379	13	75343317	3	0.211496

rs10507837	FLJ35379	13	75355396	1	1.376235
rs700371	KCTD12	13	76358368	5	1.480015
rs654196	CLN5	13	76463973	5	0.404994
rs654196	CLN5	13	76463973	8	0.127745
rs654196	CLN5	13	76463973	7	3.586158
rs654196	CLN5	13	76463973	3	0.735772
rs654196	CLN5	13	76463973	6	0.356599
rs2813739	SCEL	13	77114916	6	0.194804
rs2813739	SCEL	13	77114916	7	0.784542
rs2813739	SCEL	13	77114916	3	0.66697
rs2813739	SCEL	13	77114916	4	0.267091
rs9601092	POU4F1	13	78072092	3	0.137422
rs9601092	POU4F1	13	78072092	7	1.420387
rs1044385	C13orf7	13	78086535	6	4.613369
rs1044385	C13orf7	13	78086535	7	5.274555
rs1044385	C13orf7	13	78086535	3	0.586895
rs1044385	C13orf7	13	78086535	8	5.798254
rs9565815	LOC647317	13	82295537	1	0.008455
rs553717	GPC5	13	91143580	3	1.829799
rs553717	GPC5	13	91143580	2	3.865137
rs553717	GPC5	13	91143580	7	0.350301
rs17645969	GPC6	13	92677391	5	0.214015
rs2274020	GPC6	13	93832764	6	5.916105
rs2274020	GPC6	13	93832764	5	0.702162
rs9524570	GPR180	13	94079619	6	5.200311
rs9524570	GPR180	13	94079619	4	1.347776
rs9524570	GPR180	13	94079619	1	4.705975
rs9524570	GPR180	13	94079619	5	0.056368
rs9524570	GPR180	13	94079619	7	0.713757
rs3765534	ABCC4	13	94613416	1	5.89511
rs2274405	ABCC4	13	94656979	2	0.711973
rs2274405	ABCC4	13	94656979	5	0.601342
rs2274405	ABCC4	13	94656979	1	0.787654
rs899494	ABCC4	13	94659805	2	3.675817
rs7333332	DZIP1	13	95030684	7	0.096879
rs7333332	DZIP1	13	95030684	1	5.390683
rs7333332	DZIP1	13	95030684	6	1.359081
rs11070136	DZIP1	13	95037806	1	5.440623
rs11070136	DZIP1	13	95037806	7	0.872422
SNP13-95091632	DZIP1	13	95091632	8	6.905829
SNP13-95091632	DZIP1	13	95091632	3	0.246633
SNP13-95091632	DZIP1	13	95091632	1	2.907219
rs816142	UGCGL2	13	95436637	5	0.507475
rs12863903	UGCGL2	13	95436652	8	6.506473
rs12863903	UGCGL2	13	95436652	2	3.123766
rs12863903	UGCGL2	13	95436652	7	0.397111
rs4771259	HS6ST3	13	96284378	1	6.120171

rs4771259	HS6ST3	13	96284378	4	0.193001
rs4771259	HS6ST3	13	96284378	8	1.006051
rs7318501	HS6ST3	13	96284946	8	1.19816
rs3742123	HS6ST3	13	96286117	3	0.765465
rs3864	HS6ST3	13	96286350	7	0.550802
rs3864	HS6ST3	13	96286350	5	0.662458
rs3864	HS6ST3	13	96286350	4	1.980487
rs3864	HS6ST3	13	96286350	6	0.549314
rs2282135	HS6ST3	13	96287906	1	4.706278
rs2282135	HS6ST3	13	96287906	6	0.378342
rs2282135	HS6ST3	13	96287906	7	0.000624
rs2282135	HS6ST3	13	96287906	5	1.572946
rs9556649	OXGR1	13	96444584	3	0.31446
rs9556649	OXGR1	13	96444584	2	4.066356
rs9584575	LOC727934	13	96882676	6	6.075967
rs1159277	LOC727934	13	96883325	5	0.450739
rs1159277	LOC727934	13	96883325	7	0.402805
rs1159277	LOC727934	13	96883325	4	0.652911
rs1159277	LOC727934	13	96883325	1	5.373127
rs1159277	LOC727934	13	96883325	6	1.389245
rs12868356	LOC728155	13	97593090	2	2.947481
rs774690	RNF113B	13	97626261	4	1.263446
rs774690	RNF113B	13	97626261	8	6.21502
rs9584850	FARP1	13	97899427	7	6.676537
rs9584850	FARP1	13	97899427	5	0.001882
rs9584850	FARP1	13	97899427	8	7.868573
rs9584850	FARP1	13	97899427	3	0.09317
rs4584	FARP1	13	97899870	7	0.024957
rs4584	FARP1	13	97899870	3	0.124647
rs4584	FARP1	13	97899870	8	0.131129
rs4584	FARP1	13	97899870	5	0.200896
rs7331216	SLC15A1	13	98134920	7	1.72392
rs8187832	SLC15A1	13	98138772	7	2.704051
rs12859979	DOCK9	13	98260478	1	3.569344
rs3742130	GPR18	13	98705342	6	6.711658
rs3742130	GPR18	13	98705342	5	0.459642
rs3742130	GPR18	13	98705342	3	0.387922
rs3742130	GPR18	13	98705342	7	2.439493
rs13542	ZIC2	13	99436393	7	0.47989
rs3742244	TMTC4	13	100054564	4	0.460706
rs3742244	TMTC4	13	100054564	8	7.116412
rs8922	NALCN	13	100504194	5	1.703288
rs8922	NALCN	13	100504194	4	0.304565
rs8922	NALCN	13	100504194	3	0.291244
rs686141	NALCN	13	100534076	8	0.076687
rs686141	NALCN	13	100534076	4	0.301821
rs1140605	ITGBL1	13	101018194	3	0.15417

rs7331453	LOC728123	13	101252468	1	1.216321
rs7331453	LOC728123	13	101252468	8	5.378572
rs2282282	LOC283481	13	101845260	8	4.321504
rs2282282	LOC283481	13	101845260	2	9.330354
rs2282282	LOC283481	13	101845260	1	0.856842
rs1587	LOC196541	13	102136231	5	0.582233
rs660207	LOC196541	13	102137366	5	0.011541
SNP13-10214480	LOC196541	13	102144807	1	0.275965
rs9554897	FLJ40176	13	102181418	3	0.965575
rs17592564	LOC643677	13	102198034	3	0.995147
rs17592564	LOC643677	13	102198034	5	0.14686
rs1026158	LOC643677	13	102198084	6	0.078516
rs1026158	LOC643677	13	102198084	3	1.01924
rs12583104	LOC643677	13	102198730	1	2.88111
rs4743	BIVM	13	102291826	2	2.36894
rs4743	BIVM	13	102291826	1	0.061804
rs4743	BIVM	13	102291826	4	0.15266
rs4743	BIVM	13	102291826	5	0.008436
rs751402	ERCC5	13	102296199	8	1.762352
rs751402	ERCC5	13	102296199	5	0.081341
rs17655	ERCC5	13	102326003	4	0.271121
rs17655	ERCC5	13	102326003	8	0.666543
rs17655	ERCC5	13	102326003	1	6.254016
rs873601	ERCC5	13	102326338	1	4.036133
rs873601	ERCC5	13	102326338	8	0.602836
rs873601	ERCC5	13	102326338	6	1.195394
rs9518965	LOC728183	13	102727440	7	2.134623
rs2769914	LOC728215	13	106620247	8	1.324436
rs2769914	LOC728215	13	106620247	7	0.627302
rs2769914	LOC728215	13	106620247	6	0.089239
rs2769914	LOC728215	13	106620247	1	2.246353
rs2769914	LOC728215	13	106620247	2	1.561404
rs2769914	LOC728215	13	106620247	3	1.121057
rs9521141	MYO16	13	108505828	1	2.621828
rs157024	MYO16	13	108575504	1	0.04976
rs9559516	MYO16	13	108657946	7	0.90098
rs7993754	MYO16	13	108658175	3	1.08453
rs4773092	IRS2	13	109233954	2	1.459491
rs4773092	IRS2	13	109233954	7	0.106455
rs4773092	IRS2	13	109233954	3	0.654838
rs16975492	COL4A1	13	109631703	5	0.058754
rs16975492	COL4A1	13	109631703	3	0.016002
rs16975492	COL4A1	13	109631703	8	1.226066
rs7990009	COL4A2	13	109757689	8	0.998305
rs7990009	COL4A2	13	109757689	4	0.886557
rs7990009	COL4A2	13	109757689	5	0.42614
rs7990009	COL4A2	13	109757689	1	0.112796

rs7990009	COL4A2	13	109757689	2	1.075715
rs7327528	LOC728814	13	109758045	3	0.677952
rs4103	COL4A2	13	109896227	8	0.104742
rs4103	COL4A2	13	109896227	3	0.222363
rs4103	COL4A2	13	109896227	5	2.531658
rs7990214	COL4A2	13	109909174	8	4.634658
rs7990214	COL4A2	13	109909174	5	0.600156
rs7990214	COL4A2	13	109909174	1	4.592398
rs7990214	COL4A2	13	109909174	3	0.875808
rs7990383	COL4A2	13	109909236	5	0.287925
rs7990383	COL4A2	13	109909236	1	3.5791
rs7990383	COL4A2	13	109909236	8	4.119253
rs7990383	COL4A2	13	109909236	3	0.187313
rs9583500	COL4A2	13	109919621	1	2.287269
rs9583500	COL4A2	13	109919621	3	0.972265
rs9521879	FLJ10769	13	110084940	4	0.690325
rs12875067	ING1	13	110163105	3	0.548369
rs12875067	ING1	13	110163105	7	1.514859
rs12875067	ING1	13	110163105	2	0.47468
rs1441043	ING1	13	110165651	7	1.123701
rs1441043	ING1	13	110165651	3	1.082602
rs4771713	C13orf29	13	110317400	8	4.822746
rs2277424	C13orf29	13	110320421	4	0.911362
rs2296354	ARHGEF7	13	110668038	7	1.231273
rs2296354	ARHGEF7	13	110668038	6	0.497501
rs1555751	ARHGEF7	13	110753466	1	5.614749
rs6492352	ARHGEF7	13	110753908	4	0.924057
rs6492352	ARHGEF7	13	110753908	1	3.648374
rs12855715	C13orf16	13	110771099	7	4.864538
rs12855715	C13orf16	13	110771099	6	1.031501
rs1359428	C13orf16	13	110778538	7	0.487369
rs1359428	C13orf16	13	110778538	3	0.49064
rs4907715	LOC729192	13	112155252	3	0.943899
rs4907715	LOC729192	13	112155252	2	0.295601
rs4907733	FLJ26443	13	112349790	2	2.962944
rs9603932	FLJ26443	13	112386009	5	1.403571
rs1290177	ATP11A	13	112584133	1	1.109558
rs1290177	ATP11A	13	112584133	2	0.602713
rs3814264	MCF2L	13	112776782	1	1.6464
rs2281723	MCF2L	13	112787227	7	0.813806
rs2281723	MCF2L	13	112787227	6	0.553831
rs9604022	MCF2L	13	112789591	3	0.096733
rs9604022	MCF2L	13	112789591	1	3.880515
rs6046	F7	13	112821160	5	0.214728
rs3024772	PROZ	13	112874101	7	5.379518
rs3024772	PROZ	13	112874101	1	4.149325
rs7331116	DKFZp451A211	13	113103906	8	0.140464

rs7331116	DKFZp451A211	13	113103906	5	0.479158
rs7331116	DKFZp451A211	13	113103906	7	0.358213
rs1018649	DKFZp451A211	13	113105029	7	3.524585
rs1018648	DKFZp451A211	13	113105198	5	0.063188
rs9577553	DCUN1D2	13	113158146	2	2.706701
rs9577553	DCUN1D2	13	113158146	8	2.864203
rs1386	DCUN1D2	13	113158236	7	1.254716
rs13705	DCUN1D2	13	113158408	1	3.343014
rs2260335	TMCO3	13	113223033	1	0.111823
rs2260335	TMCO3	13	113223033	8	1.379409
rs7319493	TMCO3	13	113223039	4	0.323804
rs7399982	FLJ44054	13	113490063	3	0.566978
rs9604529	FLJ44054	13	113491346	7	0.358324
rs7400002	GAS6	13	113588999	7	0.4271
rs7400002	GAS6	13	113588999	2	0.881896
rs7400002	GAS6	13	113588999	1	2.058998
rs3752105	UPF3A	13	114065407	3	0.396068
rs3752105	UPF3A	13	114065407	7	1.150969
rs1958716	OR4L1	14	19598161	5	3.333266
rs1958716	OR4L1	14	19598161	7	2.185894
rs1958717	OR4L1	14	19598202	5	0.853554
rs1958717	OR4L1	14	19598202	7	3.052655
rs2775253	OR4L1	14	19598345	5	1.591523
rs4981088	OR11G2	14	19735680	7	1.835776
rs4981088	OR11G2	14	19735680	5	3.434697
rs4981088	OR11G2	14	19735680	6	2.472178
rs17277228	OR11H6	14	19762483	6	2.289737
rs17277228	OR11H6	14	19762483	8	1.740199
rs17277228	OR11H6	14	19762483	7	2.458763
rs1132644	CCNB1IP1	14	19854558	4	0.952751
rs3093872	RPPH1	14	19881172	8	0.487477
rs17211355	TEP1	14	19914223	6	0.205234
SNP14-1992159(	TEP1	14	19921596	8	3.203073
rs1760904	TEP1	14	19921869	4	0.455324
rs1760904	TEP1	14	19921869	7	1.044307
rs1760904	TEP1	14	19921869	8	0.207104
rs2228041	TEP1	14	19922107	7	0.364303
rs1760903	TEP1	14	19922657	8	0.147741
rs1760903	TEP1	14	19922657	4	3.039474
rs1760903	TEP1	14	19922657	7	0.133465
rs7145318	KLHL33	14	19966904	7	0.959486
rs7145318	KLHL33	14	19966904	1	3.508088
rs7145318	KLHL33	14	19966904	3	1.263421
rs1953225	KLHL33	14	19967516	1	1.557028
rs8017682	LOC728219	14	19973618	7	0.775205
rs8017603	LOC728219	14	19973746	7	1.446436
rs8017603	LOC728219	14	19973746	3	0.472305

rs938883	OSGEP	14	19986798	2	2.428915
rs938883	OSGEP	14	19986798	4	0.887411
rs938883	OSGEP	14	19986798	7	0.838329
rs1760944	OSGEP	14	19992989	3	2.79496
rs1760944	OSGEP	14	19992989	4	2.516783
rs1760944	OSGEP	14	19992989	2	4.542563
rs3136814	APEX1	14	19993137	3	0.97137
rs1049564	NP	14	20010446	6	0.508929
rs1049564	NP	14	20010446	2	6.472862
rs45440293	RNASE11	14	20128162	7	3.34221
rs45440293	RNASE11	14	20128162	4	3.169806
rs11622794	OR6S1	14	20179566	1	0.184872
rs3748338	RNASE4	14	20237416	3	0.472878
rs3748338	RNASE4	14	20237416	1	4.429773
rs1815437	FAM12A	14	20283985	4	0.182491
rs1815437	FAM12A	14	20283985	1	2.844267
rs1815437	FAM12A	14	20283985	3	0.276387
rs10135976	FAM12A	14	20285568	3	2.45995
rs10135976	FAM12A	14	20285568	1	2.20938
rs10135976	FAM12A	14	20285568	4	5.268983
rs11847654	FAM12A	14	20285837	5	0.029524
rs11847654	FAM12A	14	20285837	7	0.351268
rs11847654	FAM12A	14	20285837	1	4.145565
rs3827907	FAM12B	14	20308638	5	0.761384
rs17096021	FAM12B	14	20308702	3	0.636041
rs17096253	FAM12B	14	20308944	4	1.364837
rs17096253	FAM12B	14	20308944	3	0.205062
rs2234632	SLC39A2	14	20537753	3	0.225651
rs2234632	SLC39A2	14	20537753	4	0.907323
rs3748348	RNASE13	14	20571035	7	4.212187
rs17099500	RNASE13	14	20572665	3	1.431519
rs17099500	RNASE13	14	20572665	5	0.42838
rs2765901	RNASE7	14	20580320	4	0.587286
rs2765901	RNASE7	14	20580320	3	1.069405
SNP14-2061619	FLJ10357	14	20616194	4	1.019726
rs7143633	FLJ10357	14	20619733	4	0.521785
rs7143633	FLJ10357	14	20619733	3	0.012565
rs1958395	FLJ10357	14	20620898	4	1.216698
rs1958395	FLJ10357	14	20620898	3	0.052337
rs1958396	FLJ10357	14	20620909	3	0.065236
rs749731	FLJ10357	14	20622844	5	5.69743
rs749731	FLJ10357	14	20622844	1	5.028364
rs943992	FLJ10357	14	20622897	5	0.885771
rs943992	FLJ10357	14	20622897	1	4.076629
rs4982408	C14orf176	14	20640454	5	5.369728
rs4982408	C14orf176	14	20640454	1	5.944941
rs4982408	C14orf176	14	20640454	3	3.487414

rs1057718	C14orf176	14	20641463	1	8.63602
rs1057718	C14orf176	14	20641463	3	0.210697
rs1057718	C14orf176	14	20641463	6	6.781207
rs1057718	C14orf176	14	20641463	4	3.553404
rs45486304	ZNF219	14	20642680	6	1.791278
rs7161544	OR5AU1	14	20693488	5	0.139918
rs7161544	OR5AU1	14	20693488	6	0.199734
rs4982419	OR5AU1	14	20693676	5	2.958193
rs4982419	OR5AU1	14	20693676	8	2.105527
rs10149869	LOC401744	14	20701266	5	0.003707
rs10138887	LOC401744	14	20701491	7	1.005117
rs11852150	LOC401744	14	20701763	3	3.452677
rs11852150	LOC401744	14	20701763	7	4.113117
rs11852150	LOC401744	14	20701763	5	2.684607
rs11852150	LOC401744	14	20701763	2	3.82504
rs11852150	LOC401744	14	20701763	1	3.707154
rs11852150	LOC401744	14	20701763	4	2.953828
rs6571751	RPGRIP1	14	20840570	4	2.875135
rs6571751	RPGRIP1	14	20840570	8	4.001225
rs6571751	RPGRIP1	14	20840570	3	0.757878
SNP14-2085988(	RPGRIP1	14	20859880	5	0.170278
SNP14-2085988(	RPGRIP1	14	20859880	1	3.538648
SNP14-2085988(	RPGRIP1	14	20859880	8	1.665938
rs9322965	RPGRIP1	14	20862651	5	0.536393
rs9322965	RPGRIP1	14	20862651	1	4.216892
rs9322965	RPGRIP1	14	20862651	3	0.425175
rs9322965	RPGRIP1	14	20862651	8	0.496967
rs3748361	RPGRIP1	14	20866624	5	0.049417
rs1061027	TOX4	14	21036973	5	7.681009
rs1061027	TOX4	14	21036973	2	3.38762
rs1061027	TOX4	14	21036973	7	0.048397
rs1061027	TOX4	14	21036973	3	0.029039
rs1061027	TOX4	14	21036973	1	0.049376
rs10467787	TRAV32	14	21723351	8	1.651666
rs227003	TRAJ13	14	22069902	1	0.42
rs227003	TRAJ13	14	22069902	3	0.067556
rs15795	TRAC	14	22090478	3	0.387218
rs3700	TRAC	14	22090759	5	0.336168
rs3700	TRAC	14	22090759	4	0.061539
rs3700	TRAC	14	22090759	1	2.015739
rs3700	TRAC	14	22090759	6	0.443561
rs3700	TRAC	14	22090759	8	1.502259
rs9125	TRAC	14	22090879	1	0.419997
rs1051101	DAD1	14	22127924	5	7.41858
rs1051101	DAD1	14	22127924	6	6.736469
rs1957374	OXA1L	14	22305648	1	0.984586
rs1957374	OXA1L	14	22305648	2	4.796699

rs1957374	OXA1L	14	22305648	3	1.401924
rs1957374	OXA1L	14	22305648	8	5.633421
rs3811188	MRPL52	14	22368975	3	0.220608
rs3811188	MRPL52	14	22368975	8	2.640931
rs3811188	MRPL52	14	22368975	1	2.472224
rs4982685	MRPL52	14	22369126	3	0.468502
rs4982685	MRPL52	14	22369126	1	4.00174
rs8014119	REM2	14	22423906	3	0.896778
rs2295680	RBM23	14	22444275	5	0.011708
rs2295680	RBM23	14	22444275	3	0.844304
rs2295680	RBM23	14	22444275	8	0.706848
rs2295680	RBM23	14	22444275	7	0.281163
SNP14-22445404	RBM23	14	22445404	6	1.20052
SNP14-22445404	RBM23	14	22445404	1	4.179563
SNP14-22618627	ACIN1	14	22618627	7	4.066987
SNP14-22618633	ACIN1	14	22618633	7	0.699676
rs3751501	ACIN1	14	22619125	1	5.521504
rs3751501	ACIN1	14	22619125	4	3.230176
rs6736	C14orf119	14	22637614	1	6.01562
rs6736	C14orf119	14	22637614	5	0.179181
rs6736	C14orf119	14	22637614	4	4.652188
rs6736	C14orf119	14	22637614	7	0.624611
rs3742485	C14orf119	14	22638126	7	0.775849
rs3742485	C14orf119	14	22638126	1	4.36058
rs3742486	C14orf119	14	22638216	1	3.228927
rs3742486	C14orf119	14	22638216	4	0.806178
rs9972278	C14orf119	14	22638334	3	0.652225
rs8007215	C14orf119	14	22638946	3	0.21469
rs2236135	SLC7A8	14	22665561	4	3.714194
rs2236135	SLC7A8	14	22665561	5	6.481053
rs17183863	SLC7A8	14	22668816	8	7.415189
rs12879346	SLC7A8	14	22722548	1	0.439628
rs12879346	SLC7A8	14	22722548	6	0.977278
rs2231301	BCL2L2	14	22846939	1	5.3634
rs2239579	PABPN1	14	22859899	1	3.787398
rs2239579	PABPN1	14	22859899	6	0.188452
rs4671	EFS	14	22895553	5	7.227642
rs4671	EFS	14	22895553	1	0.399769
rs4671	EFS	14	22895553	6	0.034915
rs4671	EFS	14	22895553	2	6.979807
SNP14-22899004	EFS	14	22899004	5	0.157508
rs4553531	IL25	14	22914819	2	0.595446
rs4553531	IL25	14	22914819	3	0.771768
rs723840	CMTM5	14	22918151	1	1.096478
rs723840	CMTM5	14	22918151	7	0.253766
rs8004990	MYH6	14	22923995	7	4.610616
rs2277474	MYH6	14	22944363	6	0.549814

rs2277474	MYH6	14	22944363	7	1.321665
rs2069540	MYH7	14	22972593	3	0.820283
rs45566639	MYH7	14	22973296	3	0.223612
rs4982766	ZFHX2	14	23064357	8	4.160561
rs4982766	ZFHX2	14	23064357	2	2.481086
rs4982766	ZFHX2	14	23064357	3	0.675955
rs45484702	LRRC16B	14	23596046	5	0.05375
rs45484702	LRRC16B	14	23596046	8	0.713157
rs4982853	C14orf121	14	23597060	1	0.88856
rs1951634	PCK2	14	23639574	8	3.511334
rs1951634	PCK2	14	23639574	6	3.677072
rs927493	RNF31	14	23686623	8	0.236362
rs4761	REC8	14	23711366	3	0.506998
rs4761	REC8	14	23711366	2	3.589772
rs4761	REC8	14	23711366	4	0.048913
rs11547058	REC8	14	23712188	8	1.240398
rs11547058	REC8	14	23712188	5	0.056345
rs1885711	REC8	14	23717654	4	0.826541
rs1885711	REC8	14	23717654	5	0.79673
rs1885711	REC8	14	23717654	2	2.47538
rs1885711	REC8	14	23717654	3	0.798574
rs7146310	IPO4	14	23723794	3	0.038624
rs7146310	IPO4	14	23723794	8	0.799698
rs7146310	IPO4	14	23723794	7	3.111247
SNP14-23732017	TM9SF1	14	23732017	8	11.45759
SNP14-23732017	TM9SF1	14	23732017	7	0.227631
rs2295322	CHMP4A	14	23749717	2	0.016521
rs2295322	CHMP4A	14	23749717	4	0.527022
rs2295322	CHMP4A	14	23749717	1	0.411278
rs2295322	CHMP4A	14	23749717	3	0.503367
rs17794752	GMPR2	14	23771545	8	1.619165
rs17794752	GMPR2	14	23771545	7	3.434433
rs10134537	DHRS1	14	23830604	1	4.920663
rs10134537	DHRS1	14	23830604	8	8.317803
rs12892419	DHRS1	14	23838374	7	1.058167
rs12892419	DHRS1	14	23838374	3	3.617568
rs12892419	DHRS1	14	23838374	5	0.334178
rs12892419	DHRS1	14	23838374	1	3.718895
rs12892419	DHRS1	14	23838374	4	0.974294
rs3181385	ADCY4	14	23857428	1	1.32648
rs3181385	ADCY4	14	23857428	8	2.033548
rs2228233	NFATC4	14	23909005	7	1.70715
rs2228233	NFATC4	14	23909005	1	2.933811
rs2228233	NFATC4	14	23909005	3	1.574549
rs10143703	CBLN3	14	23968195	5	0.989036
rs10143703	CBLN3	14	23968195	1	0.410642
rs3742520	KIAA0323	14	23971089	6	0.256079

rs17795094	KIAA0323	14	23971273	2	0.386785
rs17795094	KIAA0323	14	23971273	5	0.44844
rs17795094	KIAA0323	14	23971273	1	0.201827
rs17103743	KIAA0323	14	23977392	8	6.68389
rs10083379	KIAA0323	14	23977902	8	0.632742
rs10083368	KIAA0323	14	23978024	8	0.464313
SNP14-23979202	KIAA0323	14	23979202	3	0.127838
SNP14-23979202	KIAA0323	14	23979202	8	0.39033
rs5247	CMA1	14	24046414	5	0.444578
rs8192917	GZMB	14	24172000	7	0.73535
rs8192917	GZMB	14	24172000	4	1.204911
rs2273843	GZMB	14	24173249	7	1.907842
rs2273844	GZMB	14	24173254	1	3.652525
rs2273844	GZMB	14	24173254	4	0.098422
rs2273844	GZMB	14	24173254	7	0.055212
rs3088325	STXBP6	14	24351522	5	0.265035
rs1045644	COCH	14	30424847	7	1.119433
rs1045644	COCH	14	30424847	1	0.387293
rs1045644	COCH	14	30424847	4	0.064382
rs1045644	COCH	14	30424847	3	0.801186
rs13122	COCH	14	30428775	3	0.519513
rs13122	COCH	14	30428775	6	0.984682
rs13122	COCH	14	30428775	4	1.723034
rs13122	COCH	14	30428775	5	0.303193
rs13122	COCH	14	30428775	1	5.22498
SNP14-32085148	AKAP6	14	32085148	7	1.070158
SNP14-32085148	AKAP6	14	32085148	3	0.475072
SNP14-32085148	AKAP6	14	32085148	4	4.17875
SNP14-32361334	AKAP6	14	32361334	5	0.232071
rs1051695	AKAP6	14	32362873	8	3.916476
rs1051695	AKAP6	14	32362873	7	0.874023
rs1051695	AKAP6	14	32362873	2	0.437375
rs1051695	AKAP6	14	32362873	1	0.69968
rs1051695	AKAP6	14	32362873	6	4.898959
rs1051695	AKAP6	14	32362873	4	1.202435
SNP14-34300731	BAZ1A	14	34300731	4	0.230413
GA029775	SRP54	14	34568194	3	1.653727
GA029775	SRP54	14	34568194	6	2.101037
GA029775	SRP54	14	34568194	4	6.684024
GA029775	SRP54	14	34568194	5	1.550568
rs1957106	NFKBIA	14	34943521	4	6.791818
rs1957106	NFKBIA	14	34943521	2	8.402355
rs1957106	NFKBIA	14	34943521	6	2.242593
rs1957106	NFKBIA	14	34943521	3	0.52667
rs1957106	NFKBIA	14	34943521	5	1.468514
rs1952391	INSM2	14	35073060	2	2.719641
rs1952391	INSM2	14	35073060	7	1.450697

rs2296919	INSM2	14	35075410	2	3.787097
rs2274068	GARNL1	14	35222928	7	1.436239
rs2274068	GARNL1	14	35222928	2	2.739684
rs2296168	GARNL1	14	35260781	7	0.038271
rs2296168	GARNL1	14	35260781	6	0.074878
rs2296168	GARNL1	14	35260781	1	3.713897
rs107196	LOC644616	14	35593849	5	0.925208
rs4900833	MBIP	14	35838027	5	7.884751
SNP14-3585948(	MBIP	14	35859480	5	1.071122
SNP14-3585952(	MBIP	14	35859526	5	6.632892
rs17105087	SLC25A21	14	36250436	3	0.137732
rs8019549	MIPOL1	14	36766730	2	5.659211
rs8019549	MIPOL1	14	36766730	5	4.955666
rs7144658	FOXA1	14	37131493	3	1.422702
rs7144658	FOXA1	14	37131493	6	0.703446
rs7144658	FOXA1	14	37131493	2	1.030323
rs7144658	FOXA1	14	37131493	5	0.608479
rs7144658	FOXA1	14	37131493	1	1.714796
rs7144658	FOXA1	14	37131493	7	1.676908
rs9805910	TTC6	14	37335825	3	1.881157
rs9805910	TTC6	14	37335825	4	0.51411
rs9805910	TTC6	14	37335825	7	0.251407
rs9805910	TTC6	14	37335825	6	0.736166
rs9805910	TTC6	14	37335825	2	3.065567
rs2228497	SSTR1	14	37749224	1	0.159516
rs2228497	SSTR1	14	37749224	5	1.180231
rs2228497	SSTR1	14	37749224	7	1.122524
rs12889916	SSTR1	14	37749831	1	1.197166
rs12889916	SSTR1	14	37749831	7	0.018756
rs4143959	SSTR1	14	37751020	7	0.099361
rs11558739	PNN	14	38718380	5	0.513838
rs2180792	PNN	14	38719985	2	0.608356
rs2180792	PNN	14	38719985	3	0.025839
rs1050136	PNN	14	38720575	2	3.845852
rs3825549	MIA2	14	38786559	3	0.026809
rs8015094	LOC729153	14	38913781	5	0.117392
rs8015094	LOC729153	14	38913781	1	0.479057
rs8015094	LOC729153	14	38913781	3	1.392083
rs8005625	LOC390472	14	43080539	2	0.005218
rs2282279	LOC729165	14	44017747	2	2.991495
rs2282279	LOC729165	14	44017747	6	1.388631
rs2282279	LOC729165	14	44017747	3	2.792137
rs392559	LOC729165	14	44017794	3	2.272062
rs392559	LOC729165	14	44017794	5	2.156857
rs392559	LOC729165	14	44017794	2	2.609132
rs9635162	LOC729165	14	44019556	2	0.061596
rs9635162	LOC729165	14	44019556	4	0.476303

rs3825632	FSCB	14	44044672	1	1.197935
rs3825632	FSCB	14	44044672	5	0.21196
rs3825632	FSCB	14	44044672	3	0.209185
rs11629125	FSCB	14	44045991	4	0.264561
rs11629125	FSCB	14	44045991	6	0.584524
rs1367580	FANCM	14	44714339	8	4.882224
SNP14-4477015	C14orf106	14	44770159	5	0.920946
SNP14-4477015	C14orf106	14	44770159	7	0.283629
SNP14-4478106	C14orf106	14	44781066	5	0.158559
rs17121605	PPIL5	14	49143872	5	0.07509
rs7519	MGAT2	14	49157503	6	0.584765
rs3218791	POLE2	14	49201596	5	0.904189
rs17252094	LOC283551	14	49518469	4	1.42694
rs17252094	LOC283551	14	49518469	2	4.399399
rs17252094	LOC283551	14	49518469	1	2.038717
rs17252094	LOC283551	14	49518469	8	1.884982
rs6572635	LOC283551	14	49542155	5	0.313278
rs6572635	LOC283551	14	49542155	3	1.078965
rs6572635	LOC283551	14	49542155	6	1.151218
rs6572635	LOC283551	14	49542155	4	0.664634
rs6572635	LOC283551	14	49542155	7	4.179314
rs10150798	C14orf138	14	49645589	1	4.351309
SNP14-4972505	SOS2	14	49725057	5	0.6615
rs2275591	L2HGDH	14	49848566	1	3.771721
rs2275591	L2HGDH	14	49848566	7	0.163952
rs2275591	L2HGDH	14	49848566	6	1.031541
rs2275591	L2HGDH	14	49848566	5	0.830286
rs8017316	ATP5S	14	49859328	7	0.086102
rs8017316	ATP5S	14	49859328	5	0.758927
rs8017316	ATP5S	14	49859328	1	6.842886
rs8017316	ATP5S	14	49859328	6	1.739741
rs11376	NIN	14	50274746	4	0.752919
rs11376	NIN	14	50274746	8	0.491162
rs11376	NIN	14	50274746	7	0.458655
rs17793018	NIN	14	50307451	4	1.134155
rs17793018	NIN	14	50307451	2	0.500965
rs17793018	NIN	14	50307451	1	0.636346
rs2297893	ABHD12B	14	50422358	4	0.677308
rs4901065	ABHD12B	14	50440871	3	1.148262
rs4901065	ABHD12B	14	50440871	7	2.06417
rs11157780	ABHD12B	14	50440880	7	3.241086
rs11157780	ABHD12B	14	50440880	6	0.246171
rs11157780	ABHD12B	14	50440880	3	1.148304
rs11157781	ABHD12B	14	50440900	3	0.033096
rs11157781	ABHD12B	14	50440900	6	0.159992
rs11157781	ABHD12B	14	50440900	7	2.575977
rs7159068	ABHD12B	14	50441151	7	1.343335

rs11157782	ABHD12B	14	50441274	7	2.398268
rs11157782	ABHD12B	14	50441274	6	6.578443
rs11157782	ABHD12B	14	50441274	3	0.976132
rs2075643	PYGL	14	50453182	6	6.622677
rs2075643	PYGL	14	50453182	3	0.113017
rs1049371	TRIM9	14	50512420	4	4.984565
rs883690	TRIM9	14	50513110	5	0.637392
rs883690	TRIM9	14	50513110	4	1.064594
rs2275462	TRIM9	14	50515966	3	1.011092
rs2275462	TRIM9	14	50515966	5	0.386861
rs2275462	TRIM9	14	50515966	8	3.532393
rs2275462	TRIM9	14	50515966	4	0.830301
rs3813398	TRIM9	14	50533573	8	0.96964
rs12883270	TRIM9	14	50533833	4	0.911249
rs1057804	GNG2	14	51503805	3	0.408742
rs1057804	GNG2	14	51503805	5	0.487343
rs3825596	GNG2	14	51504633	2	4.518676
rs3825596	GNG2	14	51504633	4	0.651811
rs3825596	GNG2	14	51504633	7	1.310556
rs3204006	GNG2	14	51504796	3	0.514318
rs1540703	GNG2	14	51505245	3	0.063765
rs1051069	NID2	14	51548065	3	0.709891
rs1051069	NID2	14	51548065	6	7.978908
rs1051069	NID2	14	51548065	4	2.074053
rs1051069	NID2	14	51548065	5	0.016789
rs946615	NID2	14	51551667	4	0.876776
rs946615	NID2	14	51551667	3	0.527648
rs946615	NID2	14	51551667	2	3.205388
rs946615	NID2	14	51551667	6	0.062336
rs12887495	NID2	14	51566025	7	0.268817
rs3742536	NID2	14	51577179	7	3.628362
rs3742536	NID2	14	51577179	2	1.194973
rs3920038	NID2	14	51605398	7	0.247449
rs17125273	PTGDR	14	51811396	3	1.469974
rs802986	PTGDR	14	51812464	8	0.498094
rs802986	PTGDR	14	51812464	7	1.267317
rs1042618	PTGER2	14	51864850	3	0.132502
rs2767540	TXNDC16	14	51968417	5	6.09373
rs2767540	TXNDC16	14	51968417	7	0.804654
rs762063	GPR137C	14	52168652	2	5.027092
rs762063	GPR137C	14	52168652	4	0.678432
rs762063	GPR137C	14	52168652	5	4.54451
rs3742532	GPR137C	14	52172434	4	0.126572
rs12885451	GPR137C	14	52173306	4	0.564408
rs1957844	LOC645560	14	53527828	5	0.529669
rs7146775	WDHD1	14	54477507	2	5.700834
rs7147147	WDHD1	14	54477694	2	1.114722

rs15870	DLG7	14	54689064	5	0.085368
rs1045002	FBXO34	14	54888270	6	0.089366
rs1045002	FBXO34	14	54888270	5	0.026051
rs1045002	FBXO34	14	54888270	8	5.45039
rs1045002	FBXO34	14	54888270	2	9.194036
rs1572611	KIAA0831	14	54903627	6	6.804814
rs1572611	KIAA0831	14	54903627	5	0.549171
rs1572611	KIAA0831	14	54903627	8	4.834428
rs8019270	TBPL2	14	54976925	6	0.329005
rs12886220	KTN1	14	55148492	5	0.34075
rs12886220	KTN1	14	55148492	7	0.482878
rs12886220	KTN1	14	55148492	8	0.681557
rs2184557	LOC645683	14	55302723	6	4.4547
rs2184557	LOC645683	14	55302723	2	3.843239
rs2184557	LOC645683	14	55302723	7	4.186937
rs10144431	LOC645683	14	55302822	7	3.982447
rs10144431	LOC645683	14	55302822	6	3.517999
rs10144431	LOC645683	14	55302822	2	0.835213
rs1152466	PELI2	14	55833639	4	0.799563
rs3742578	EXOC5	14	56742468	8	7.440295
rs1152531	C14orf105	14	57007877	6	0.086049
rs2747106	C14orf37	14	57540752	4	1.049845
rs2747106	C14orf37	14	57540752	1	2.480953
rs2256270	C14orf37	14	57541042	5	0.772384
rs2256270	C14orf37	14	57541042	3	0.728757
rs2256270	C14orf37	14	57541042	7	0.918305
rs2256270	C14orf37	14	57541042	6	0.244609
rs2256270	C14orf37	14	57541042	1	2.619008
rs2256270	C14orf37	14	57541042	4	0.56073
rs2295139	C14orf37	14	57541108	7	0.470147
rs2295139	C14orf37	14	57541108	8	0.449012
rs2295139	C14orf37	14	57541108	3	0.642215
SNP14-5767479	C14orf37	14	57674796	6	3.156384
rs3829765	C14orf37	14	57675543	4	0.696128
rs17832998	DACT1	14	58182485	5	1.380757
SNP14-5818318	DACT1	14	58183183	1	1.130531
rs17833004	DACT1	14	58184200	2	1.670135
rs11541	DACT1	14	58184523	2	1.662322
rs11541	DACT1	14	58184523	8	1.052125
rs11541	DACT1	14	58184523	1	0.535613
rs167437	C14orf135	14	59661640	7	0.253596
rs409866	DHRS7	14	59681281	7	0.756212
rs409866	DHRS7	14	59681281	5	1.725141
rs381839	DHRS7	14	59681428	7	6.694275
rs381839	DHRS7	14	59681428	5	0.285238
rs4899016	SIX4	14	60248662	1	4.900934
rs4899016	SIX4	14	60248662	5	0.570288

rs4899016	SIX4	14	60248662	4	0.038583
rs698028	FLJ22447	14	61190328	5	1.291478
rs698028	FLJ22447	14	61190328	7	0.827199
rs698027	FLJ22447	14	61190520	7	1.716163
rs698027	FLJ22447	14	61190520	5	0.27185
rs698027	FLJ22447	14	61190520	3	1.353872
rs1255720	PPP2R5E	14	63079274	3	1.119191
rs1255720	PPP2R5E	14	63079274	7	0.761485
rs1255720	PPP2R5E	14	63079274	6	0.736041
rs1255720	PPP2R5E	14	63079274	4	4.386169
rs10498511	WDR89	14	63178291	8	2.013678
SNP14-63607251	SYNE2	14	63607251	4	0.575895
SNP14-63629844	SYNE2	14	63629844	5	0.326584
SNP14-63629844	SYNE2	14	63629844	1	1.848725
SNP14-63682611	SYNE2	14	63682611	4	0.993805
SNP14-63682611	SYNE2	14	63682611	6	0.595176
SNP14-63682611	SYNE2	14	63682611	7	0.798405
rs1152582	SYNE2	14	63762383	7	0.791034
rs1152582	SYNE2	14	63762383	1	4.15738
rs1152582	SYNE2	14	63762383	4	5.192139
rs1255998	ESR2	14	63763624	4	0.191866
rs8018687	ESR2	14	63763835	7	0.461925
rs8018687	ESR2	14	63763835	4	2.958327
rs8018687	ESR2	14	63763835	8	1.338924
rs4986938	ESR2	14	63769569	1	5.229449
rs4986938	ESR2	14	63769569	3	0.053303
rs1256049	ESR2	14	63793804	8	1.072619
rs2230491	AKAP5	14	64005164	6	5.639208
SNP14-64058583	ZBTB1	14	64058583	2	5.525948
rs12385918	ZBTB1	14	64069698	6	8.598892
rs12385918	ZBTB1	14	64069698	3	1.39753
rs12385910	ZBTB1	14	64069723	6	1.35404
rs12385910	ZBTB1	14	64069723	3	0.094158
rs12385910	ZBTB1	14	64069723	8	0.496267
rs229624	PLEKHG3	14	64267520	7	0.740473
rs229624	PLEKHG3	14	64267520	1	0.084316
rs229624	PLEKHG3	14	64267520	8	1.5558
rs229624	PLEKHG3	14	64267520	6	0.532387
SNP14-64279620	PLEKHG3	14	64279620	1	4.940516
SNP14-64279620	PLEKHG3	14	64279620	7	0.192648
rs17180132	PLEKHG3	14	64279662	5	3.153471
rs1695769	SPTB	14	64284918	6	0.177952
rs1695769	SPTB	14	64284918	7	0.41543
rs1695769	SPTB	14	64284918	1	4.70406
rs229592	SPTB	14	64310981	6	0.686061
rs229592	SPTB	14	64310981	8	0.703677
rs229592	SPTB	14	64310981	7	1.101584

rs229592	SPTB	14	64310981	1	2.867394
rs229592	SPTB	14	64310981	5	2.611756
rs184528	SPTB	14	64311797	7	0.404819
rs184528	SPTB	14	64311797	1	0.029431
rs184528	SPTB	14	64311797	5	0.291233
rs1741488	SPTB	14	64315709	8	5.011017
rs1741488	SPTB	14	64315709	7	1.730265
rs1626923	SPTB	14	64316376	5	0.070895
SNP14-64322985	SPTB	14	64322985	5	0.805339
SNP14-64322985	SPTB	14	64322985	7	2.68872
rs11623956	SPTB	14	64340144	6	2.934212
rs6745	CHURC1	14	64471448	8	3.527235
rs6745	CHURC1	14	64471448	1	3.915419
rs6745	CHURC1	14	64471448	7	2.768294
rs6745	CHURC1	14	64471448	5	2.342251
rs2296326	RAB15	14	64484125	2	5.04202
rs2296326	RAB15	14	64484125	7	5.119547
rs2296326	RAB15	14	64484125	8	1.276437
rs7159443	MAX	14	64620569	2	6.788877
rs7159443	MAX	14	64620569	1	0.046328
rs2411822	FUT8	14	64948148	2	5.364166
rs1054218	FUT8	14	65278943	2	7.023215
rs1054218	FUT8	14	65278943	5	0.161849
rs1054218	FUT8	14	65278943	4	1.151267
rs1054218	FUT8	14	65278943	8	7.044234
rs1054218	FUT8	14	65278943	3	0.224609
rs1055136	MGC88374	14	66034953	2	0.566721
rs1055136	MGC88374	14	66034953	1	0.443365
rs928553	GPHN	14	66044227	4	0.568134
rs928553	GPHN	14	66044227	7	0.310304
rs11158655	FAM71D	14	66741454	4	0.240821
rs35236021	FAM71D	14	66744674	2	1.552649
rs10141879	FAM71D	14	66764943	4	0.511109
rs10141879	FAM71D	14	66764943	7	0.649235
rs13379210	C14orf83	14	66990924	3	1.733184
rs3825723	PLEKHH1	14	67093887	3	1.284015
rs3825723	PLEKHH1	14	67093887	7	0.610602
rs3825723	PLEKHH1	14	67093887	6	0.558187
rs3825723	PLEKHH1	14	67093887	5	1.118039
rs2236235	PLEKHH1	14	67099066	7	1.651672
rs2236235	PLEKHH1	14	67099066	5	0.022951
rs2236235	PLEKHH1	14	67099066	3	0.321731
rs2236235	PLEKHH1	14	67099066	6	1.453149
rs1885043	PLEKHH1	14	67099139	2	3.791292
rs734028	PLEKHH1	14	67123555	1	5.694604
rs734028	PLEKHH1	14	67123555	6	0.740711
rs734028	PLEKHH1	14	67123555	8	0.328248

rs4902496	PLEKHH1	14	67125366	2	3.586927
rs4902496	PLEKHH1	14	67125366	4	0.531613
rs8005363	PIGH	14	67126143	1	5.47629
rs8005363	PIGH	14	67126143	8	0.516901
rs7160492	LOC729868	14	67150294	7	0.732931
rs2235967	ZFYVE26	14	67319252	8	4.758334
rs2235967	ZFYVE26	14	67319252	6	2.989634
rs2235967	ZFYVE26	14	67319252	1	1.033406
rs2235967	ZFYVE26	14	67319252	2	1.404613
rs2235967	ZFYVE26	14	67319252	4	1.309326
rs2235967	ZFYVE26	14	67319252	3	0.643496
rs8016546	GALNTL1	14	68890167	1	5.387475
rs8016546	GALNTL1	14	68890167	4	0.138722
rs2296724	GALNTL1	14	68890699	6	0.347022
rs2296724	GALNTL1	14	68890699	4	4.012472
rs2296724	GALNTL1	14	68890699	1	3.551497
SNP14-6898976	SLC39A9	14	68989764	2	5.224622
rs7494244	C14orf162	14	69107364	3	0.146103
rs7494244	C14orf162	14	69107364	1	2.904285
rs7494244	C14orf162	14	69107364	6	1.22715
rs945310	KIAA0247	14	69248090	3	0.404958
rs945310	KIAA0247	14	69248090	2	0.581299
rs945310	KIAA0247	14	69248090	1	0.33686
rs3809396	KIAA0247	14	69248188	3	0.085687
rs4646285	SLC10A1	14	69333401	6	5.360028
rs4646285	SLC10A1	14	69333401	4	0.722424
rs3742909	SMOC1	14	69488634	6	2.14086
rs184477	SLC8A3	14	69580879	5	0.533257
rs184477	SLC8A3	14	69580879	1	4.43417
rs3809401	SLC8A3	14	69581159	5	1.026144
rs3809401	SLC8A3	14	69581159	4	2.325215
SNP14-7020759	TTC9	14	70207597	8	0.883901
rs2074647	RGS6	14	72098931	7	0.008185
rs11846274	DPF3	14	72146069	7	1.137828
rs3742836	DPF3	14	72289773	7	0.112402
rs3742836	DPF3	14	72289773	4	0.830012
rs3742836	DPF3	14	72289773	8	0.454527
rs3742836	DPF3	14	72289773	3	0.558463
rs3742836	DPF3	14	72289773	5	0.25017
rs3742836	DPF3	14	72289773	1	1.901806
rs2098322	LOC729953	14	72463037	6	2.8284
rs2098322	LOC729953	14	72463037	3	2.05594
rs2302588	WDR21A	14	72474505	7	1.644877
rs2302588	WDR21A	14	72474505	4	0.399891
rs3815460	WDR21A	14	72492012	4	0.104298
rs3815460	WDR21A	14	72492012	7	12.91307
rs17569391	WDR21A	14	72495820	1	6.766825

rs17569391	WDR21A	14	72495820	6	4.67945
rs2286838	ZFYVE1	14	72518336	4	0.50484
rs2286838	ZFYVE1	14	72518336	7	0.609815
rs2280792	PAPLN	14	72781147	1	0.109245
rs2280792	PAPLN	14	72781147	3	2.375273
rs932256	PAPLN	14	72810402	6	5.108368
rs17781919	NUMB	14	72813211	4	0.5628
rs1074501	HEATR4	14	73059612	7	3.147924
rs1074501	HEATR4	14	73059612	5	2.725566
rs1074501	HEATR4	14	73059612	3	2.071595
rs12891009	ACOT6	14	73153599	8	0.784182
rs8003105	DNAL1	14	73232598	7	0.209319
rs8003105	DNAL1	14	73232598	4	0.025474
rs17782124	C14orf43	14	73273542	3	0.791787
rs17782128	C14orf43	14	73275631	3	0.252915
rs758774	C14orf43	14	73276234	6	5.998736
rs758774	C14orf43	14	73276234	3	0.183155
rs758773	C14orf43	14	73276411	3	0.129716
rs8020630	ZADH1	14	73388426	4	1.735647
rs8020630	ZADH1	14	73388426	3	1.58257
rs8020630	ZADH1	14	73388426	5	2.608824
rs8020630	ZADH1	14	73388426	2	3.229301
rs8020267	ZADH1	14	73388467	1	3.937674
rs8020267	ZADH1	14	73388467	6	0.6833
rs2270073	ZADH1	14	73388507	4	1.327783
rs2270073	ZADH1	14	73388507	5	1.296812
rs2270073	ZADH1	14	73388507	3	1.359738
rs8020523	ZADH1	14	73388539	6	9.387188
rs3177095	ZNF410	14	73458661	4	0.333616
rs3177095	ZNF410	14	73458661	3	0.63348
rs8500	COQ6	14	73498198	4	6.632945
rs13743	ENTPD5	14	73503305	3	0.151173
rs13743	ENTPD5	14	73503305	2	6.357908
rs2074932	ENTPD5	14	73511437	4	0.104853
rs2074932	ENTPD5	14	73511437	5	0.66334
rs2074932	ENTPD5	14	73511437	6	0.239732
rs2302117	ENTPD5	14	73524566	2	5.169408
rs2302117	ENTPD5	14	73524566	4	1.779194
rs2006731	LIN52	14	73621271	4	0.76975
rs2006731	LIN52	14	73621271	5	0.393064
rs2006732	LIN52	14	73621279	5	1.637997
rs2006732	LIN52	14	73621279	4	1.041656
rs2301345	ABCD4	14	73836105	6	0.502321
rs2301345	ABCD4	14	73836105	7	0.491975
rs2301345	ABCD4	14	73836105	5	0.363393
rs2301345	ABCD4	14	73836105	3	0.542508
rs2286425	C14orf115	14	73884992	5	2.967544

rs2240624	TMEM90A	14	73942679	3	0.791105
rs2240624	TMEM90A	14	73942679	4	0.831358
rs2240624	TMEM90A	14	73942679	6	1.742761
rs2240623	TMEM90A	14	73942935	7	1.455496
rs2240623	TMEM90A	14	73942935	4	0.607574
rs2240623	TMEM90A	14	73942935	3	1.678633
rs2240622	TMEM90A	14	73942959	4	3.01638
rs2240622	TMEM90A	14	73942959	7	3.378399
rs2240622	TMEM90A	14	73942959	8	1.81658
rs2240622	TMEM90A	14	73942959	3	3.06669
rs2240622	TMEM90A	14	73942959	6	2.355514
rs4903221	TMEM90A	14	73943327	4	0.587787
rs4026651	LOC729988	14	73946254	8	1.104416
rs4026651	LOC729988	14	73946254	6	0.055671
rs4026651	LOC729988	14	73946254	3	0.753328
rs7145480	LTBP2	14	74058440	3	0.5692
rs699374	LTBP2	14	74062553	6	0.240817
rs699374	LTBP2	14	74062553	3	0.449361
rs699374	LTBP2	14	74062553	2	1.462361
rs2304707	LTBP2	14	74092024	5	0.572563
rs9441	KIAA0317	14	74198178	2	2.992995
rs9441	KIAA0317	14	74198178	3	0.819019
rs9441	KIAA0317	14	74198178	4	8.446306
rs2270425	KIAA0317	14	74249487	2	3.903587
rs2270425	KIAA0317	14	74249487	1	0.243294
rs2270425	KIAA0317	14	74249487	8	0.627209
rs2270425	KIAA0317	14	74249487	4	0.501237
rs2302834	YLPM1	14	74349148	7	0.897787
rs2302834	YLPM1	14	74349148	3	0.041127
rs2302834	YLPM1	14	74349148	1	2.628467
rs4140930	DLST	14	74429423	4	3.656234
rs4140930	DLST	14	74429423	2	1.874452
rs4140930	DLST	14	74429423	7	0.225547
rs4140930	DLST	14	74429423	3	0.314995
rs2286913	RPS6KL1	14	74456329	2	2.852025
rs2286913	RPS6KL1	14	74456329	7	0.249681
rs2286913	RPS6KL1	14	74456329	4	0.046717
rs2286913	RPS6KL1	14	74456329	3	0.059344
rs7156590	RPS6KL1	14	74457936	2	2.45673
rs7156590	RPS6KL1	14	74457936	7	0.15991
rs7156590	RPS6KL1	14	74457936	3	0.858872
rs7156590	RPS6KL1	14	74457936	4	0.216554
rs11159109	RPS6KL1	14	74458827	2	4.740665
rs11159109	RPS6KL1	14	74458827	4	0.135693
rs11159109	RPS6KL1	14	74458827	3	0.310939
rs12411	PGF	14	74478796	1	6.182485
rs12411	PGF	14	74478796	4	1.91052

rs4556	EIF2B2	14	74545824	7	7.924857
rs4556	EIF2B2	14	74545824	1	0.042577
rs4556	EIF2B2	14	74545824	2	7.630974
rs4556	EIF2B2	14	74545824	6	7.386271
rs4556	EIF2B2	14	74545824	3	0.856751
rs175049	MLH3	14	74551121	2	0.759986
rs175049	MLH3	14	74551121	3	0.548695
rs175049	MLH3	14	74551121	4	0.071657
rs175049	MLH3	14	74551121	1	0.260437
rs175049	MLH3	14	74551121	6	3.553412
rs175049	MLH3	14	74551121	7	1.326014
rs108622	MLH3	14	74551661	6	1.770848
rs108622	MLH3	14	74551661	3	1.660097
rs108622	MLH3	14	74551661	7	1.37287
rs108622	MLH3	14	74551661	1	1.282735
rs108622	MLH3	14	74551661	2	3.241708
rs108622	MLH3	14	74551661	4	2.340954
SNP14-74583581	MLH3	14	74583581	4	0.444328
SNP14-74583581	MLH3	14	74583581	7	1.462808
SNP14-74583581	MLH3	14	74583581	3	0.429368
SNP14-74583581	MLH3	14	74583581	6	0.876013
SNP14-74583581	MLH3	14	74583581	1	0.86656
GA011719	C14orf140	14	74607134	1	0.467118
GA011719	C14orf140	14	74607134	7	0.529025
GA011719	C14orf140	14	74607134	4	0.648481
GA011719	C14orf140	14	74607134	6	1.753372
rs8011292	FLVCR2	14	75115444	8	4.846465
rs8011292	FLVCR2	14	75115444	5	0.362183
rs8011292	FLVCR2	14	75115444	3	0.151668
rs2287017	FLVCR2	14	75115716	1	0.788452
rs2287017	FLVCR2	14	75115716	7	1.856179
rs2287017	FLVCR2	14	75115716	2	0.841313
rs3815550	VASH1	14	76306133	6	0.093731
rs3815550	VASH1	14	76306133	1	0.583522
rs3815550	VASH1	14	76306133	4	0.134139
rs3815550	VASH1	14	76306133	7	0.729333
rs3815550	VASH1	14	76306133	2	2.036958
rs3815550	VASH1	14	76306133	3	1.536977
rs2280796	VASH1	14	76316511	6	1.554829
rs2280796	VASH1	14	76316511	1	0.920332
rs2280796	VASH1	14	76316511	3	0.792624
rs2280796	VASH1	14	76316511	5	1.104273
rs926120	C14orf166B	14	76362745	6	0.045881
rs7160583	C14orf166B	14	76402161	3	1.618611
rs7160583	C14orf166B	14	76402161	1	3.498107
rs7160583	C14orf166B	14	76402161	8	2.169672
rs7160583	C14orf166B	14	76402161	6	1.112679

rs7160583	C14orf166B	14	76402161	7	2.120535
rs4903543	KIAA1737	14	76651014	4	0.122858
rs4903543	KIAA1737	14	76651014	6	0.494402
rs4903543	KIAA1737	14	76651014	8	0.287587
rs4903543	KIAA1737	14	76651014	7	4.497572
rs403291	TMEM63C	14	76772810	4	0.200193
rs403291	TMEM63C	14	76772810	6	2.983212
rs403291	TMEM63C	14	76772810	1	4.800579
rs429443	TMEM63C	14	76793012	1	1.540852
rs429443	TMEM63C	14	76793012	4	0.309729
rs429443	TMEM63C	14	76793012	2	2.216375
rs429443	TMEM63C	14	76793012	3	1.333342
rs429443	TMEM63C	14	76793012	7	1.437966
rs384492	TMEM63C	14	76793223	3	2.488468
rs384492	TMEM63C	14	76793223	1	0.763548
rs3813539	TMEM63C	14	76793979	1	3.313688
rs2270421	POMT2	14	76856841	1	4.807322
rs2270421	POMT2	14	76856841	4	0.704665
rs2270421	POMT2	14	76856841	3	4.470926
rs8177538	GSTZ1	14	76857459	3	0.395258
rs3177427	GSTZ1	14	76862960	3	0.314577
rs3177427	GSTZ1	14	76862960	1	4.044258
rs7972	GSTZ1	14	76862990	3	0.329296
rs3742737	TMED8	14	76913137	2	0.412408
rs3742737	TMED8	14	76913137	1	0.458097
rs3742737	TMED8	14	76913137	4	2.972102
rs3742737	TMED8	14	76913137	6	0.412623
rs11844594	C14orf174	14	76913567	2	0.021296
rs11844594	C14orf174	14	76913567	1	0.11624
rs11844594	C14orf174	14	76913567	3	0.037597
rs11844594	C14orf174	14	76913567	4	0.230795
rs4903576	C14orf174	14	76914622	1	1.24025
rs4903576	C14orf174	14	76914622	3	1.367127
rs4903576	C14orf174	14	76914622	4	0.668236
rs2193595	C14orf174	14	76914874	4	4.411928
rs2193595	C14orf174	14	76914874	1	0.541787
rs2193595	C14orf174	14	76914874	3	1.443455
rs10130481	C14orf148	14	76950058	6	0.891272
rs10130481	C14orf148	14	76950058	3	1.300212
rs4223	C14orf133	14	76963029	4	6.467381
rs4223	C14orf133	14	76963029	1	0.041265
rs4223	C14orf133	14	76963029	3	0.406316
rs11539461	AHSA1	14	77005412	2	9.175023
rs11539461	AHSA1	14	77005412	1	11.00847
rs12434449	THSD3	14	77034414	3	0.63627
rs6494	ALKBH1	14	77210108	3	0.095789
rs6494	ALKBH1	14	77210108	2	0.659102

rs6494	ALKBH1	14	77210108	5	0.02192
rs6494	ALKBH1	14	77210108	6	0.079091
rs11159286	C14orf156	14	77244226	1	3.680067
rs12879016	NRXN3	14	79399885	2	2.610244
rs12879016	NRXN3	14	79399885	7	2.894602
rs12879016	NRXN3	14	79399885	5	0.826785
rs225015	DIO2	14	79737332	1	2.347987
rs225015	DIO2	14	79737332	8	0.057588
rs225015	DIO2	14	79737332	5	1.112286
rs7140952	DIO2	14	79738025	6	0.674325
rs7140952	DIO2	14	79738025	2	6.05896
rs225014	DIO2	14	79739333	8	0.019605
rs225014	DIO2	14	79739333	1	4.78891
rs225014	DIO2	14	79739333	7	0.828207
rs225014	DIO2	14	79739333	5	0.405819
rs3783942	TSHR	14	80644712	5	0.143452
rs3783942	TSHR	14	80644712	1	5.850596
rs3783942	TSHR	14	80644712	6	0.582121
rs3783941	TSHR	14	80644758	5	0.112515
rs3783941	TSHR	14	80644758	1	4.782716
rs3783941	TSHR	14	80644758	6	0.3945
rs1991517	TSHR	14	80680336	6	3.009447
rs7144481	TSHR	14	80680695	3	1.595233
rs7144481	TSHR	14	80680695	6	0.385478
rs7144481	TSHR	14	80680695	2	2.535677
rs17630128	TSHR	14	80680881	7	0.430241
rs2288493	TSHR	14	80681359	7	1.012291
rs2288496	TSHR	14	80681867	2	4.757827
rs2288496	TSHR	14	80681867	8	0.656469
rs2288496	TSHR	14	80681867	3	1.178693
rs2288496	TSHR	14	80681867	6	0.006632
rs2288496	TSHR	14	80681867	4	0.910067
rs11159706	LOC730034	14	85065440	2	4.534251
rs11159706	LOC730034	14	85065440	5	0.573835
rs11159707	LOC730034	14	85065552	5	0.256955
rs11159707	LOC730034	14	85065552	2	0.008041
rs11159707	LOC730034	14	85065552	7	0.722058
rs17714100	FLRT2	14	85159006	8	7.127356
rs17121383	FLRT2	14	85161663	8	0.505655
rs2038494	FLRT2	14	85161999	2	4.696458
rs1042029	GALC	14	87470239	7	0.209373
rs1042029	GALC	14	87470239	1	0.508265
rs1042029	GALC	14	87470239	5	6.116139
rs398076	GALC	14	87483964	5	0.669502
rs398076	GALC	14	87483964	1	0.119177
rs17687109	GALC	14	87499443	4	1.336131
rs10139328	GPR65	14	87541315	7	2.006789

rs10139328	GPR65	14	87541315	1	4.141187
rs10139328	GPR65	14	87541315	5	1.729903
rs6574978	GPR65	14	87547166	5	2.158692
rs6574978	GPR65	14	87547166	1	3.22317
rs17762463	KCNK10	14	87721715	8	2.445846
rs2277524	KCNK10	14	87763478	5	1.351555
rs2277524	KCNK10	14	87763478	4	1.526537
rs9026	C14orf143	14	89333253	3	1.110729
rs8778	C14orf143	14	89468660	6	1.339463
rs12147332	KCNK13	14	89721110	7	1.741177
rs12147332	KCNK13	14	89721110	3	1.088091
rs3742671	C14orf102	14	89826513	2	7.10777
rs3179089	CALM1	14	89943165	7	0.568235
rs3179089	CALM1	14	89943165	3	0.129319
rs3179089	CALM1	14	89943165	8	6.115819
rs3179089	CALM1	14	89943165	6	0.985879
rs3179089	CALM1	14	89943165	5	0.030977
rs3742660	TTC7B	14	90193328	5	0.392418
rs3742660	TTC7B	14	90193328	3	0.077785
rs1285859	C14orf159	14	90761107	7	0.013107
rs6562	C14orf159	14	90761224	7	0.264491
rs12891713	CCDC88C	14	90830275	1	3.703616
rs12891713	CCDC88C	14	90830275	5	4.60389
rs1970912	CCDC88C	14	90843321	5	0.361509
rs1970912	CCDC88C	14	90843321	1	0.533252
rs1970912	CCDC88C	14	90843321	6	0.046189
rs1970912	CCDC88C	14	90843321	3	1.089368
rs1970912	CCDC88C	14	90843321	7	1.075701
rs2430347	FBLN5	14	91417433	1	4.187016
rs2430347	FBLN5	14	91417433	4	0.359339
rs17127898	TRIP11	14	91575668	8	0.119329
rs13350	NDUFB1	14	91652225	1	1.919125
rs13350	NDUFB1	14	91652225	5	0.518733
rs4900131	SLC24A4	14	92029901	4	0.068072
rs12888354	SLC24A4	14	92029925	5	0.074835
rs12888354	SLC24A4	14	92029925	3	0.031534
rs4900132	SLC24A4	14	92029991	5	0.996391
rs4900132	SLC24A4	14	92029991	1	0.86269
rs3814830	RIN3	14	92187951	3	0.635054
rs9791	LGMN	14	92240746	8	1.39443
rs9791	LGMN	14	92240746	1	0.352369
rs729940	CHGA	14	92468854	7	0.179453
rs941581	CHGA	14	92470931	1	0.296359
rs941581	CHGA	14	92470931	6	3.228117
rs941581	CHGA	14	92470931	4	0.15206
rs941581	CHGA	14	92470931	7	0.269726
rs941581	CHGA	14	92470931	8	4.324055

rs8017818	MOAP1	14	92720807	6	1.252483
rs8017818	MOAP1	14	92720807	4	0.515658
rs8008104	PRIMA1	14	93256125	1	1.798373
rs8008104	PRIMA1	14	93256125	4	1.686438
rs1887197	PRIMA1	14	93257585	2	3.240656
rs1887197	PRIMA1	14	93257585	3	0.261915
rs7147919	ASB2	14	93487174	4	2.024799
SNP14-93487284	ASB2	14	93487284	1	1.513468
rs4277287	ASB2	14	93487294	5	0.395246
rs4277287	ASB2	14	93487294	4	0.867974
rs4483793	ASB2	14	93487339	4	1.99102
rs10142728	C14orf48	14	93534242	3	0.194935
rs2273226	OTUB2	14	93580717	1	0.173417
rs2273226	OTUB2	14	93580717	7	0.313188
rs2273226	OTUB2	14	93580717	8	0.596271
rs1950643	KIAA1622	14	93815323	6	1.723708
rs1950643	KIAA1622	14	93815323	8	0.955418
rs941591	SERPINA10	14	93826503	6	0.856151
rs941590	SERPINA10	14	93826547	3	1.78319
rs941590	SERPINA10	14	93826547	5	2.015664
rs941590	SERPINA10	14	93826547	7	1.107108
SNP14-93845974	SERPINA6	14	93845974	6	1.34889
SNP14-93845974	SERPINA6	14	93845974	7	0.488916
SNP14-93845974	SERPINA6	14	93845974	5	2.612686
SNP14-93845974	SERPINA6	14	93845974	3	2.168865
SNP14-93845974	SERPINA6	14	93845974	2	1.919059
rs4592582	SERPINA2	14	93902549	2	0.105528
rs1243166	SERPINA2	14	93913571	3	1.041115
rs1243166	SERPINA2	14	93913571	1	6.049724
rs1243163	SERPINA1	14	93914459	8	2.601376
rs1243163	SERPINA1	14	93914459	5	2.09938
rs12888805	SERPINA11	14	93982552	1	0.425718
rs12888805	SERPINA11	14	93982552	2	1.745474
rs12888805	SERPINA11	14	93982552	3	0.244438
SNP14-93982649	SERPINA11	14	93982649	7	0.721428
SNP14-93982649	SERPINA11	14	93982649	5	1.090514
SNP14-94003393	SERPINA9	14	94003393	7	0.255613
SNP14-94003393	SERPINA9	14	94003393	1	1.751449
rs12879019	SERPINA9	14	94005654	6	3.56623
rs5508	SERPINA4	14	94099605	4	0.370918
rs5508	SERPINA4	14	94099605	5	0.562397
rs5510	SERPINA4	14	94103109	6	0.763161
rs6118	SERPINA5	14	94123616	6	2.88603
SNP14-94123643	SERPINA5	14	94123643	1	4.380231
SNP14-94123643	SERPINA5	14	94123643	8	2.705608
SNP14-94123643	SERPINA5	14	94123643	5	0.038341
rs6108	SERPINA5	14	94128384	8	3.65E-06

rs6108	SERPINA5	14	94128384	7	0.359862
rs6108	SERPINA5	14	94128384	5	0.270505
rs6108	SERPINA5	14	94128384	6	8.407777
rs6108	SERPINA5	14	94128384	1	4.365543
rs12885659	LOC390503	14	94170528	1	0.115746
rs17826595	SERPINA13	14	94179586	7	0.030736
rs17826595	SERPINA13	14	94179586	4	0.105926
rs13078	DICER1	14	94626500	5	8.168851
rs13078	DICER1	14	94626500	2	5.81606
rs13078	DICER1	14	94626500	3	0.364007
rs3742345	C14orf139	14	94945095	6	0.571699
rs3742343	C14orf139	14	94946140	5	0.876883
rs12434757	C14orf49	14	94954076	6	0.856808
rs12434757	C14orf49	14	94954076	1	0.445601
rs12434757	C14orf49	14	94954076	2	0.781953
rs12434757	C14orf49	14	94954076	3	0.564659
rs9671369	C14orf49	14	94976074	8	1.137364
rs9671369	C14orf49	14	94976074	6	0.227328
rs9671369	C14orf49	14	94976074	3	1.198625
rs9919918	C14orf49	14	94980686	4	1.41499
rs9919918	C14orf49	14	94980686	6	0.831405
rs9919918	C14orf49	14	94980686	1	3.322095
rs9919918	C14orf49	14	94980686	3	0.588234
rs4905318	C14orf49	14	94988378	7	0.882601
rs4905318	C14orf49	14	94988378	6	2.922405
rs4905318	C14orf49	14	94988378	3	0.370728
rs4905318	C14orf49	14	94988378	5	0.737476
rs4905318	C14orf49	14	94988378	8	2.354778
rs2024863	C14orf49	14	94993423	7	0.829215
rs2024863	C14orf49	14	94993423	3	1.201484
rs2024863	C14orf49	14	94993423	4	1.308188
rs2024863	C14orf49	14	94993423	6	3.548605
rs2024863	C14orf49	14	94993423	8	6.18978
rs2024863	C14orf49	14	94993423	5	2.101225
rs6575536	TCL6	14	95187492	1	1.372815
rs8018690	TCL6	14	95187537	6	0.719945
rs8018690	TCL6	14	95187537	1	0.309568
rs9591	TCL6	14	95205284	5	1.689089
SNP14-95227432	TCL1B	14	95227432	8	2.917584
SNP14-95227432	TCL1B	14	95227432	5	2.399825
SNP14-95227432	TCL1B	14	95227432	1	3.989578
SNP14-95227432	TCL1B	14	95227432	4	3.012628
rs3208738	C14orf132	14	95625170	6	0.112188
rs3208738	C14orf132	14	95625170	7	5.422697
rs3742480	C14orf132	14	95626021	7	0.962611
rs3742480	C14orf132	14	95626021	6	0.495817
rs3809416	C14orf132	14	95627626	3	0.732119

rs3809416	C14orf132	14	95627626	5	0.105845
rs1062709	C14orf132	14	95628302	7	0.180875
rs1062709	C14orf132	14	95628302	5	1.855657
rs1062709	C14orf132	14	95628302	3	0.53114
rs2146934	C14orf132	14	95628634	6	0.89575
rs2146934	C14orf132	14	95628634	7	1.128322
rs1062710	C14orf132	14	95629212	7	1.177571
rs1062710	C14orf132	14	95629212	6	0.467
rs10082	C14orf132	14	95629544	6	2.372526
rs10082	C14orf132	14	95629544	5	1.611182
rs10082	C14orf132	14	95629544	7	1.915791
rs10752	C14orf132	14	95629836	7	0.873962
rs10752	C14orf132	14	95629836	5	1.043959
rs5224	BDKRB2	14	95777210	2	1.443989
rs5224	BDKRB2	14	95777210	6	0.689848
rs5225	BDKRB2	14	95777976	7	0.769837
rs2069589	BDKRB2	14	95778469	7	5.843275
rs2069589	BDKRB2	14	95778469	3	1.281393
rs2069590	BDKRB2	14	95778535	3	0.524886
rs2069590	BDKRB2	14	95778535	5	0.042977
rs2069590	BDKRB2	14	95778535	4	0.896269
rs2069591	BDKRB2	14	95778585	3	1.05925
rs2069591	BDKRB2	14	95778585	7	1.103169
rs2069592	BDKRB2	14	95778601	3	1.912564
rs2069592	BDKRB2	14	95778601	7	0.978593
rs2071084	BDKRB1	14	95799895	2	2.994261
rs2369679	AK7	14	95992505	7	0.447172
rs2369679	AK7	14	95992505	1	6.049146
rs7160148	LOC730202	14	96038357	7	1.362828
rs11160553	EML1	14	99401629	4	0.16525
rs11160553	EML1	14	99401629	7	0.525264
rs2250718	EML1	14	99450701	3	0.161613
rs2250718	EML1	14	99450701	8	0.257923
rs2250718	EML1	14	99450701	1	4.369869
rs1044631	EML1	14	99477134	3	0.115519
rs1044631	EML1	14	99477134	8	4.667554
rs2236317	DEGS2	14	99682846	8	5.929688
rs2236317	DEGS2	14	99682846	6	7.649096
SNP14-99864892	C14orf68	14	99864892	5	1.180423
rs2234519	WARS	14	99911648	7	0.767797
rs12893951	BEGAIN	14	100074968	3	0.757457
rs12893951	BEGAIN	14	100074968	1	0.762039
rs12893951	BEGAIN	14	100074968	6	1.698763
rs12892787	C14orf70	14	100208760	4	0.793938
rs1802710	DLK1	14	100270398	6	1.613068
rs1802710	DLK1	14	100270398	8	4.468732
rs1802710	DLK1	14	100270398	7	1.538976

rs941575	MEG3	14	100365583	8	3.8554
rs3825569	RTL1	14	100420051	5	0.629424
rs3809403	FLJ41170	14	100542369	6	0.927399
rs8011440	LOC730241	14	101094911	7	1.731757
rs945006	DIO3	14	101099030	3	1.160898
rs945006	DIO3	14	101099030	5	0.020555
rs8005905	HSP90AA1	14	101638120	7	1.313536
SNP14-10186238	C14orf131	14	101862384	7	0.487002
SNP14-10186238	C14orf131	14	101862384	1	5.513414
rs17100874	KIAA0329	14	101912810	5	7.213415
SNP14-10197095	KIAA0329	14	101970954	3	0.121788
rs17100923	KIAA0329	14	101979744	7	0.599093
GA011875	KIAA0329	14	102034407	3	0.35471
GA011875	KIAA0329	14	102034407	8	6.737414
GA011875	KIAA0329	14	102034407	2	1.966705
GA011875	KIAA0329	14	102034407	4	0.238267
rs2273905	ANKRD9	14	102044752	3	0.017141
rs2273905	ANKRD9	14	102044752	4	0.303794
rs2273905	ANKRD9	14	102044752	8	2.347075
rs2273905	ANKRD9	14	102044752	2	4.192043
rs1131877	TRAF3	14	102411802	4	0.259367
rs1131877	TRAF3	14	102411802	3	0.424468
rs1131877	TRAF3	14	102411802	8	0.317828
rs1190233	AMN	14	102466808	8	4.534729
rs1190233	AMN	14	102466808	3	0.589258
rs1190233	AMN	14	102466808	6	1.085085
rs8009219	CDC42BPB	14	102510226	3	0.647695
rs8009219	CDC42BPB	14	102510226	7	0.762581
rs35954043	CDC42BPB	14	102535801	8	3.593104
rs729185	C14orf73	14	102646148	4	0.026964
rs3870	C14orf73	14	102646528	4	0.242814
rs3178152	TNFAIP2	14	102663703	8	0.345353
rs3178152	TNFAIP2	14	102663703	2	0.584785
rs3178152	TNFAIP2	14	102663703	3	0.316692
rs3178152	TNFAIP2	14	102663703	6	0.939152
rs3178152	TNFAIP2	14	102663703	7	5.797685
rs1052912	TNFAIP2	14	102673406	3	0.055795
rs1052912	TNFAIP2	14	102673406	4	0.049835
rs1052912	TNFAIP2	14	102673406	5	5.614944
rs7161305	C14orf172	14	103071987	3	0.354952
rs7161305	C14orf172	14	103071987	4	6.988496
rs7161305	C14orf172	14	103071987	6	0.782234
rs7148456	BAG5	14	103098023	4	0.661963
rs7148456	BAG5	14	103098023	6	0.682731
rs7148456	BAG5	14	103098023	2	2.094054
rs3212068	LOC730176	14	103241511	8	7.576896
rs3212068	LOC730176	14	103241511	3	2.094777

rs17292804	PPP1R13B	14	103274876	3	0.281099
rs17292804	PPP1R13B	14	103274876	2	3.973749
rs17292804	PPP1R13B	14	103274876	4	0.117558
SNP14-10328912	PPP1R13B	14	103289121	3	1.020131
SNP14-10328912	PPP1R13B	14	103289121	8	0.565121
SNP14-10328912	PPP1R13B	14	103289121	7	1.369514
SNP14-10328912	PPP1R13B	14	103289121	1	0.104493
SNP14-10328912	PPP1R13B	14	103289121	4	6.12831
SNP14-10328912	PPP1R13B	14	103289121	6	1.2502
SNP14-10328912	PPP1R13B	14	103289121	5	3.880781
SNP14-10328912	PPP1R13B	14	103289121	2	0.968206
rs7150461	TDRD9	14	103588206	2	1.733106
rs7150461	TDRD9	14	103588206	6	0.961086
rs7150461	TDRD9	14	103588206	1	0.31085
rs1744284	LOC374569	14	103629675	5	1.098274
rs1465163	LOC374569	14	103633685	3	0.168475
rs2487305	KIF26A	14	103711545	2	1.715684
rs2487305	KIF26A	14	103711545	6	0.6385
rs1547350	KIF26A	14	103716079	3	0.264245
rs1547350	KIF26A	14	103716079	7	0.723088
rs1547350	KIF26A	14	103716079	5	0.047762
rs1547350	KIF26A	14	103716079	4	0.143412
rs947000	KIF26A	14	103716470	5	0.115273
rs947000	KIF26A	14	103716470	2	2.702455
rs947000	KIF26A	14	103716470	3	1.258568
rs947000	KIF26A	14	103716470	6	0.601041
rs947000	KIF26A	14	103716470	7	0.404605
rs10083486	C14orf180	14	104126989	5	0.215207
rs10083489	C14orf180	14	104127004	5	0.925449
rs3809461	LOC730185	14	104129543	8	2.593107
rs3809461	LOC730185	14	104129543	3	0.274993
rs3809457	LOC730185	14	104129855	3	1.242394
rs3809457	LOC730185	14	104129855	5	0.411558
SNP14-10425183	INF2	14	104251830	4	0.739137
rs10138227	AKT1	14	104330751	8	0.982816
rs41314519	KIAA0284	14	104415868	6	7.684837
rs2028414	KIAA0284	14	104420433	4	0.219169
rs2028414	KIAA0284	14	104420433	7	0.792766
rs2028414	KIAA0284	14	104420433	5	0.331194
rs45449696	KIAA0284	14	104421223	3	0.554907
rs45449696	KIAA0284	14	104421223	1	0.854283
GA011942	KIAA0284	14	104421913	7	0.764879
GA011942	KIAA0284	14	104421913	1	1.172872
rs2582548	KIAA0284	14	104425338	1	2.552081
rs2582548	KIAA0284	14	104425338	5	0.317548
rs2582548	KIAA0284	14	104425338	7	0.868944
rs752265	KIAA0284	14	104432979	4	0.690874

rs752265	KIAA0284	14	104432979	5	0.158877
rs752265	KIAA0284	14	104432979	1	3.82127
rs3742935	AHNAK2	14	104476644	2	7.381842
rs3742935	AHNAK2	14	104476644	8	6.006611
rs3742935	AHNAK2	14	104476644	3	0.35234
rs3742935	AHNAK2	14	104476644	7	1.996502
rs2272591	JAG2	14	104682327	4	4.737453
rs2272591	JAG2	14	104682327	8	8.353449
rs2272591	JAG2	14	104682327	6	5.31078
rs2272591	JAG2	14	104682327	1	5.199
rs2272591	JAG2	14	104682327	3	0.606092
rs3087836	BRF1	14	104747093	1	0.301626
rs3087836	BRF1	14	104747093	7	1.217254
rs3087836	BRF1	14	104747093	3	0.487427
rs3087836	BRF1	14	104747093	2	5.032006
rs3087836	BRF1	14	104747093	6	7.60662
rs2816605	BTBD6	14	104786560	3	0.658578
rs2816605	BTBD6	14	104786560	8	1.775791
rs14592	BTBD6	14	104788310	2	0.285986
rs14592	BTBD6	14	104788310	1	1.162716
rs4983413	MTA1	14	105001451	5	0.142957
rs885883	IGHVII-78-1	14	106345097	7	0.360432
rs885883	IGHVII-78-1	14	106345097	1	0.520944
rs548	TUBGCP5	15	20425225	8	2.863434
rs7170838	NIPA2	15	20572679	3	1.12096
rs7170838	NIPA2	15	20572679	7	3.494834
rs6606820	NIPA1	15	20597755	6	5.11087
rs6606820	NIPA1	15	20597755	8	6.905277
rs6606820	NIPA1	15	20597755	4	0.364115
rs6606820	NIPA1	15	20597755	2	6.183186
rs6606820	NIPA1	15	20597755	3	0.218624
rs6606820	NIPA1	15	20597755	1	0.556488
rs1059774	NIPA1	15	20599143	3	0.080796
rs1059774	NIPA1	15	20599143	4	0.296611
rs1059774	NIPA1	15	20599143	6	0.105762
rs1059774	NIPA1	15	20599143	1	0.063693
rs1059774	NIPA1	15	20599143	2	0.262585
rs1059774	NIPA1	15	20599143	8	0.97928
rs2239669	MKRN3	15	21362685	8	4.813231
rs2239669	MKRN3	15	21362685	4	3.243786
rs9785	MAGEL2	15	21440126	1	0.469362
rs9785	MAGEL2	15	21440126	6	0.544688
rs9785	MAGEL2	15	21440126	3	0.16304
rs9785	MAGEL2	15	21440126	4	0.18882
rs3742950	C15orf2	15	22473323	5	0.521009
rs3742950	C15orf2	15	22473323	1	0.334022
rs4906743	ATP10A	15	23473746	5	0.504863

rs4906743	ATP10A	15	23473746	2	3.111062
rs4906743	ATP10A	15	23473746	8	0.714049
rs4906744	ATP10A	15	23473810	5	0.276412
rs2066710	ATP10A	15	23474580	6	2.708249
rs2066710	ATP10A	15	23474580	3	1.61071
rs2066710	ATP10A	15	23474580	5	2.17195
rs2066710	ATP10A	15	23474580	1	4.657134
rs2066708	ATP10A	15	23474805	7	0.219163
rs2066708	ATP10A	15	23474805	5	0.593143
rs2066708	ATP10A	15	23474805	3	0.012273
rs3816800	ATP10A	15	23476187	3	0.738494
rs3816800	ATP10A	15	23476187	2	0.62154
rs3816800	ATP10A	15	23476187	7	7.551534
rs3816800	ATP10A	15	23476187	5	0.046307
rs3816800	ATP10A	15	23476187	1	1.334269
SNP15-23477291	ATP10A	15	23477291	5	1.458254
SNP15-23477291	ATP10A	15	23477291	8	1.96956
rs2076741	ATP10A	15	23477297	5	1.422621
rs2076741	ATP10A	15	23477297	8	0.01076
rs17555920	ATP10A	15	23504291	6	5.496567
rs2066704	ATP10A	15	23504535	5	0.334966
rs17116056	ATP10A	15	23520183	6	4.852524
rs2017247	GABRB3	15	24340255	3	1.622053
rs2017247	GABRB3	15	24340255	5	0.110544
rs12595837	LOC730017	15	24421157	5	0.272825
rs12595837	LOC730017	15	24421157	3	1.934229
rs12595837	LOC730017	15	24421157	6	0.630876
rs140685	GABRA5	15	24771205	8	4.672818
rs1800407	OCA2	15	25903913	5	1.210973
rs3751555	APBA2	15	27197365	4	3.53374
SNP15-27205812	KIAA0574	15	27205812	4	0.819845
SNP15-27205812	KIAA0574	15	27205812	2	0.402375
rs798088	LOC390561	15	28876818	7	1.992871
rs798088	LOC390561	15	28876818	8	1.060633
rs798088	LOC390561	15	28876818	3	0.203288
rs798088	LOC390561	15	28876818	2	1.519073
rs798088	LOC390561	15	28876818	4	0.621823
rs1852033	LOC390561	15	28877893	1	1.225028
rs1852033	LOC390561	15	28877893	7	0.738469
rs1852033	LOC390561	15	28877893	5	0.197651
rs1852033	LOC390561	15	28877893	4	1.251106
rs1852033	LOC390561	15	28877893	3	1.085482
rs1852033	LOC390561	15	28877893	8	0.577205
rs1852033	LOC390561	15	28877893	6	0.761015
rs3512	MTMR15	15	29022297	7	8.40815
rs3512	MTMR15	15	29022297	8	5.180155
rs11629793	MTMR10	15	29025715	7	1.013237

rs11629793	MTMR10	15	29025715	8	0.893976
rs2949569	MTMR10	15	29025864	3	0.984674
rs2949569	MTMR10	15	29025864	4	0.926382
rs2949569	MTMR10	15	29025864	1	4.304022
rs2949569	MTMR10	15	29025864	7	0.953006
rs12898290	TRPM1	15	29081635	4	0.614348
rs1035705	TRPM1	15	29130036	1	0.703789
rs7164569	OTUD7A	15	29581222	1	0.020059
rs7164569	OTUD7A	15	29581222	4	2.709873
rs7164569	OTUD7A	15	29581222	8	0.799154
rs7164569	OTUD7A	15	29581222	3	0.675721
rs7164569	OTUD7A	15	29581222	6	0.40584
SNP15-3071691(	ARHGAP11A	15	30716916	4	0.015412
rs12915554	GREM1	15	30810778	1	2.584344
rs12915554	GREM1	15	30810778	6	1.651808
rs12915554	GREM1	15	30810778	7	1.363007
rs12915554	GREM1	15	30810778	4	0.789287
rs3743105	GREM1	15	30811243	7	1.110253
rs3743103	GREM1	15	30812919	7	0.872542
rs10318	GREM1	15	30813271	1	0.276126
rs10318	GREM1	15	30813271	5	1.168943
rs10318	GREM1	15	30813271	2	1.185704
rs1129456	GREM1	15	30813959	5	0.513452
rs2306277	FMN1	15	31144554	4	0.834524
rs2306277	FMN1	15	31144554	3	0.166862
rs2306277	FMN1	15	31144554	6	0.81567
rs2229116	RYR3	15	31692702	3	1.205628
rs2229116	RYR3	15	31692702	6	1.402211
rs6495228	RYR3	15	31803566	4	1.130865
rs2288613	RYR3	15	31900302	6	0.695648
rs1044129	RYR3	15	31945558	6	2.568608
rs1044129	RYR3	15	31945558	8	0.743253
rs1044129	RYR3	15	31945558	7	0.698523
rs17236798	SLC12A6	15	32330164	7	0.259818
rs17236798	SLC12A6	15	32330164	4	0.373164
rs7173	NOLA3	15	32421312	4	0.543997
rs7173	NOLA3	15	32421312	1	2.21571
rs4984215	LOC441720	15	32453352	3	0.41014
rs4984215	LOC441720	15	32453352	8	0.321526
rs7164070	AQR	15	33049244	6	0.773398
rs7164070	AQR	15	33049244	5	3.948439
rs1015943	C15orf41	15	34889080	5	5.287947
rs1015943	C15orf41	15	34889080	1	2.396071
rs12595568	C15orf53	15	36778095	3	0.781163
rs1495185	C15orf53	15	36778728	2	1.56311
rs1495185	C15orf53	15	36778728	4	0.186326
rs12916379	C15orf53	15	36778812	7	1.662046

rs1495187	C15orf53	15	36778999	8	0.316303
rs156783	C15orf54	15	37332324	6	5.265903
rs156783	C15orf54	15	37332324	2	3.19308
rs156783	C15orf54	15	37332324	1	0.942254
rs13329154	C15orf54	15	37332440	3	0.967777
rs17691608	C15orf54	15	37332773	3	0.425384
rs1478605	THBS1	15	37660576	3	0.201147
rs1478605	THBS1	15	37660576	6	0.717504
rs2229364	THBS1	15	37667009	4	0.400466
rs2229364	THBS1	15	37667009	5	1.013961
rs2229364	THBS1	15	37667009	3	0.01145
rs6492905	FSIP1	15	37679694	8	0.539605
rs6492905	FSIP1	15	37679694	6	2.010491
rs6492905	FSIP1	15	37679694	7	1.073323
rs12908846	FSIP1	15	37697696	5	0.320325
rs12908846	FSIP1	15	37697696	4	0.153949
rs937961	FSIP1	15	37793064	2	6.049704
rs937961	FSIP1	15	37793064	6	3.762827
rs35602605	EIF2AK4	15	38096151	5	0.197541
rs2277559	BUB1B	15	38240599	2	1.272252
rs2277559	BUB1B	15	38240599	7	1.321953
rs2277559	BUB1B	15	38240599	1	1.020833
rs2277559	BUB1B	15	38240599	6	4.915869
rs2277559	BUB1B	15	38240599	4	2.994536
rs2277559	BUB1B	15	38240599	5	0.241586
rs1801376	BUB1B	15	38265123	1	0.831997
rs34790429	BUB1B	15	38297954	6	0.015716
rs2229690	PLCB2	15	38377426	5	0.492927
rs2229690	PLCB2	15	38377426	3	2.096575
rs2229690	PLCB2	15	38377426	7	1.392499
rs28602975	LOC390578	15	38392154	4	1.167725
rs28602975	LOC390578	15	38392154	1	1.563773
rs4924454	C15orf52	15	38412066	3	0.237993
rs4924454	C15orf52	15	38412066	6	0.070013
rs4924454	C15orf52	15	38412066	8	0.365305
rs12443160	DISP2	15	38443433	5	0.239509
rs7169262	C15orf23	15	38462785	6	5.243865
SNP15-38470994	C15orf23	15	38470994	6	0.339433
rs8042729	CCDC32	15	38632594	3	0.655922
rs10152546	CCDC32	15	38642502	8	0.100926
rs10152546	CCDC32	15	38642502	7	0.332975
rs10152546	CCDC32	15	38642502	3	0.460969
rs10152546	CCDC32	15	38642502	2	5.053995
rs3803353	CCDC32	15	38644532	7	0.650007
rs7177192	CASC5	15	38685935	3	0.024841
rs33931006	CASC5	15	38702123	1	5.430217
rs2619679	LOC729029	15	38773529	7	7.159061

rs2619679	LOC729029	15	38773529	8	1.198806
rs2619679	LOC729029	15	38773529	3	0.212164
rs2619679	LOC729029	15	38773529	2	2.269227
rs11855560	RAD51	15	38811595	3	1.844168
rs11855560	RAD51	15	38811595	7	1.803778
rs11855560	RAD51	15	38811595	2	1.907468
rs1866170	LOC643338	15	38850536	2	4.214548
rs1866170	LOC643338	15	38850536	7	0.03895
rs1866170	LOC643338	15	38850536	6	3.914697
SNP15-38856063	DNAJC17	15	38856063	2	3.322814
SNP15-38856063	DNAJC17	15	38856063	5	1.258693
rs690347	ZFYVE19	15	38893218	1	1.04703
rs690347	ZFYVE19	15	38893218	6	0.647403
rs690347	ZFYVE19	15	38893218	3	0.228032
rs690347	ZFYVE19	15	38893218	5	1.434461
rs690136	SPINT1	15	38935455	3	0.480729
rs676180	SPINT1	15	38937009	3	0.878746
rs690733	EXDL1	15	39263757	3	0.77692
rs28493702	CHP	15	39359994	8	3.166014
GA012333	CHP	15	39361343	7	0.314283
rs12900702	NDUFAF1	15	39466977	7	0.191085
rs12900702	NDUFAF1	15	39466977	5	4.408551
rs12900702	NDUFAF1	15	39466977	6	0.455869
rs11630901	RPAP1	15	39606659	7	1.082581
rs11630901	RPAP1	15	39606659	1	1.935141
rs11630901	RPAP1	15	39606659	5	0.234172
rs2289741	RPAP1	15	39607492	1	4.552457
SNP15-39609455	RPAP1	15	39609455	3	0.208802
SNP15-39609455	RPAP1	15	39609455	4	1.088373
rs2297382	RPAP1	15	39615049	4	0.508417
rs2297382	RPAP1	15	39615049	6	0.565374
rs2297382	RPAP1	15	39615049	3	0.044173
rs2605570	MGA	15	39846813	8	0.38372
rs2605570	MGA	15	39846813	7	7.622262
rs2605570	MGA	15	39846813	3	0.283805
rs1201689	MAPKBP1	15	39893210	3	0.003432
rs1201689	MAPKBP1	15	39893210	5	0.679591
rs1201689	MAPKBP1	15	39893210	2	5.834549
rs1201689	MAPKBP1	15	39893210	7	0.435456
rs2277535	MAPKBP1	15	39907003	6	1.404915
rs2277535	MAPKBP1	15	39907003	2	4.121285
rs1672466	PLA2G4B	15	39923280	3	0.145895
rs1197669	PLA2G4B	15	39925748	5	0.071493
rs1197669	PLA2G4B	15	39925748	7	1.302702
rs1197669	PLA2G4B	15	39925748	2	0.340464
rs1197669	PLA2G4B	15	39925748	3	0.310737
SNP15-39930410	SPTBN5	15	39930410	6	0.640962

rs1197660	SPTBN5	15	39933228	7	0.743122
rs1197660	SPTBN5	15	39933228	3	0.487016
SNP15-39936798	SPTBN5	15	39936798	7	1.039481
rs1197675	SPTBN5	15	39936888	5	1.109972
rs1197675	SPTBN5	15	39936888	2	0.239894
rs1197675	SPTBN5	15	39936888	7	0.077755
rs1197675	SPTBN5	15	39936888	3	0.557248
rs16972193	SPTBN5	15	39942030	6	0.836583
SNP15-39946582	SPTBN5	15	39946582	2	0.252162
SNP15-39946582	SPTBN5	15	39946582	5	0.959943
SNP15-39946582	SPTBN5	15	39946582	3	0.064457
rs12593397	SPTBN5	15	39953792	7	0.532562
SNP15-39955692	SPTBN5	15	39955692	3	0.093082
SNP15-39955692	SPTBN5	15	39955692	5	2.848889
SNP15-39955692	SPTBN5	15	39955692	2	2.257282
SNP15-39955692	SPTBN5	15	39955692	7	0.343809
rs12708402	SPTBN5	15	39958754	1	1.682423
rs12708402	SPTBN5	15	39958754	3	1.0389
rs12708402	SPTBN5	15	39958754	2	1.159562
rs12708402	SPTBN5	15	39958754	5	1.078548
rs1197682	SPTBN5	15	39958775	4	1.142432
SNP15-39958829	SPTBN5	15	39958829	5	0.182134
SNP15-39958829	SPTBN5	15	39958829	3	1.008696
SNP15-39958829	SPTBN5	15	39958829	4	1.665011
SNP15-39958829	SPTBN5	15	39958829	8	0.435563
rs2280016	SPTBN5	15	39965414	4	0.206867
rs1210795	SPTBN5	15	39965447	4	0.741896
rs1210795	SPTBN5	15	39965447	5	0.474721
rs1210795	SPTBN5	15	39965447	3	0.343912
rs1210795	SPTBN5	15	39965447	8	0.644935
rs1048175	EHD4	15	39979201	3	0.217648
rs1048175	EHD4	15	39979201	2	0.390272
rs1048175	EHD4	15	39979201	7	0.441633
rs1048175	EHD4	15	39979201	1	0.232749
rs11549015	EHD4	15	40022608	2	3.144238
rs11549015	EHD4	15	40022608	8	1.105737
rs1668579	PLA2G4E	15	40069011	8	1.079252
rs750052	PLA2G4D	15	40148314	6	3.134202
rs750052	PLA2G4D	15	40148314	5	2.557042
SNP15-40150335	PLA2G4D	15	40150335	5	0.112108
SNP15-40150335	PLA2G4D	15	40150335	6	0.620251
rs655427	PLA2G4F	15	40221546	1	5.083475
rs655427	PLA2G4F	15	40221546	5	0.197428
rs655427	PLA2G4F	15	40221546	7	3.453931
rs7086	VPS39	15	40238400	8	0.609291
rs8024732	GANC	15	40358010	8	5.653877
rs2578652	GANC	15	40405823	2	2.116684

rs3743006	GANC	15	40433060	8	6.798361
rs2241827	CAPN3	15	40482236	5	0.252751
rs2241827	CAPN3	15	40482236	7	0.747674
rs1062038	ZFP106	15	40495135	8	0.191528
rs10851411	CEP27	15	40646527	8	1.561811
rs10851411	CEP27	15	40646527	5	3.403069
rs6493059	STARD9	15	40764818	5	0.576678
rs6493059	STARD9	15	40764818	8	4.265425
rs8030587	STARD9	15	40769098	8	3.385292
rs8030587	STARD9	15	40769098	5	0.800128
rs1058846	STARD9	15	40799316	8	6.072998
rs1058846	STARD9	15	40799316	3	1.042295
rs1058846	STARD9	15	40799316	5	3.53586
rs9269	STARD9	15	40799566	5	2.092478
rs9269	STARD9	15	40799566	8	6.703066
rs1995939	STARD9	15	40799809	8	6.50994
rs1995939	STARD9	15	40799809	5	2.812696
rs8023524	CDAN1	15	40808275	5	0.081778
rs4265781	CDAN1	15	40816041	1	1.077823
rs4265781	CDAN1	15	40816041	7	5.914438
rs4265781	CDAN1	15	40816041	5	0.531844
rs4265781	CDAN1	15	40816041	6	0.322805
rs3742970	LCMT2	15	41409557	3	0.51321
rs560191	TP53BP1	15	41555066	3	0.729296
rs560191	TP53BP1	15	41555066	2	1.468019
rs560191	TP53BP1	15	41555066	6	0.406997
rs1060939	MAP1A	15	41604209	6	0.910895
rs12912505	MAP1A	15	41604696	2	2.863811
rs12912505	MAP1A	15	41604696	6	0.427897
rs480108	MAP1A	15	41604698	2	2.164702
rs480108	MAP1A	15	41604698	6	0.348368
rs689931	MAP1A	15	41610120	5	0.022866
rs689931	MAP1A	15	41610120	8	0.32385
rs572837	MAP1A	15	41611105	6	1.370837
rs2411284	PDIA3	15	41826191	6	1.013347
rs2411284	PDIA3	15	41826191	2	1.580002
rs678084	WDR76	15	41907851	6	2.457306
rs678084	WDR76	15	41907851	2	2.889022
rs2470911	TRIM69	15	42834426	5	0.787712
rs2470911	TRIM69	15	42834426	1	2.427048
rs2470911	TRIM69	15	42834426	4	0.553747
rs2470911	TRIM69	15	42834426	3	0.908725
rs17588988	TRIM69	15	42834694	1	4.207644
rs17588988	TRIM69	15	42834694	3	0.808645
rs17588988	TRIM69	15	42834694	8	1.08261
rs3100139	TRIM69	15	42834865	4	0.053741
rs3100139	TRIM69	15	42834865	3	1.12307

rs3100139	TRIM69	15	42834865	1	2.843126
rs3759880	TRIM69	15	42835943	4	0.781196
rs3759880	TRIM69	15	42835943	1	4.841008
rs3759880	TRIM69	15	42835943	3	2.738087
rs1648281	DUOXA1	15	43209050	6	0.327844
rs1648281	DUOXA1	15	43209050	7	0.804733
rs1648281	DUOXA1	15	43209050	2	1.162214
SNP15-43278428	SHF	15	43278428	7	0.173685
SNP15-43332770	SLC28A2	15	43332770	1	0.211536
SNP15-43332770	SLC28A2	15	43332770	6	0.396412
rs1049518	GATM	15	43440659	8	1.016924
rs1049518	GATM	15	43440659	6	0.397854
rs1049518	GATM	15	43440659	1	2.976418
rs17618637	GATM	15	43440686	2	2.540613
rs1288775	GATM	15	43448970	7	1.382196
rs1288775	GATM	15	43448970	8	4.577262
rs1288775	GATM	15	43448970	2	2.661148
rs1365610	SPATA5L1	15	43481902	2	3.973813
rs1365610	SPATA5L1	15	43481902	7	3.49151
rs1365610	SPATA5L1	15	43481902	8	4.190327
rs2433600	SLC30A4	15	43563716	2	1.014843
rs2433600	SLC30A4	15	43563716	1	1.013777
rs2433600	SLC30A4	15	43563716	6	1.243026
rs8042811	C15orf21	15	43635213	6	0.167416
rs8042811	C15orf21	15	43635213	1	0.522193
rs8042811	C15orf21	15	43635213	2	0.231399
rs532598	SEMA6D	15	45845363	8	0.531114
rs568215	SEMA6D	15	45850368	8	1.578304
rs11070641	FBN1	15	46488321	1	4.318698
rs11070641	FBN1	15	46488321	5	0.924709
rs8033037	FBN1	15	46584599	1	3.677722
rs8033037	FBN1	15	46584599	5	3.577819
rs8033037	FBN1	15	46584599	4	0.327582
GA030476	DTWD1	15	47704633	6	3.084144
GA030476	DTWD1	15	47704633	5	0.436874
GA030476	DTWD1	15	47704633	7	0.202513
rs10519239	LOC645453	15	47873772	4	5.342205
rs7497350	ATP8B4	15	47938195	1	1.495095
rs7497350	ATP8B4	15	47938195	3	0.072869
rs1059851	ATP8B4	15	47938725	3	0.911099
rs4494482	ATP8B4	15	47939458	3	1.108073
rs4494483	ATP8B4	15	47939566	6	1.056252
rs4494483	ATP8B4	15	47939566	3	0.454308
rs4494483	ATP8B4	15	47939566	5	0.206707
rs4494483	ATP8B4	15	47939566	7	0.252436
rs1648348	SLC27A2	15	48262058	4	3.361045
rs1648348	SLC27A2	15	48262058	7	0.395727

rs1648348	SLC27A2	15	48262058	1	4.046592
rs1648348	SLC27A2	15	48262058	3	0.051307
rs8034633	SLC27A2	15	48315841	1	3.157589
rs8034633	SLC27A2	15	48315841	3	1.375132
rs1549521	HDC	15	48322124	1	2.150304
rs8042919	TRPM7	15	48665922	1	4.777518
rs10046	CYP19A1	15	49290278	8	0.250656
rs898135	GLDN	15	49485817	8	2.641265
rs3751585	DMXL2	15	49528348	2	9.246578
rs3751585	DMXL2	15	49528348	6	6.779194
rs3751585	DMXL2	15	49528348	1	0.093315
rs6493514	DMXL2	15	49702070	3	6.25574
rs35664837	SCG3	15	49771654	7	0.721447
rs16964532	TMOD2	15	49888196	3	0.704032
rs2623258	TMOD2	15	49888886	4	0.399704
rs2623258	TMOD2	15	49888886	2	5.070399
rs2623258	TMOD2	15	49888886	8	4.500912
rs2623258	TMOD2	15	49888886	1	0.018448
rs16953082	TMOD2	15	49889548	3	0.439758
rs2554327	LOC645615	15	49940470	4	4.183121
rs2554327	LOC645615	15	49940470	1	4.612637
rs2554327	LOC645615	15	49940470	3	4.302564
rs2227308	BCL2L10	15	50189243	1	5.00598
rs2227308	BCL2L10	15	50189243	3	0.404005
SNP15-5019215	BCL2L10	15	50192154	5	0.138176
SNP15-5019215	BCL2L10	15	50192154	7	0.294027
SNP15-5019215	BCL2L10	15	50192154	3	1.077285
SNP15-5019215	BCL2L10	15	50192154	1	0.651057
rs12396	GNB5	15	50200608	1	0.066868
rs12396	GNB5	15	50200608	2	0.083264
rs12396	GNB5	15	50200608	7	5.731162
rs11070883	GNB5	15	50270857	7	0.498655
rs11070883	GNB5	15	50270857	3	0.50498
rs11070883	GNB5	15	50270857	4	0.87037
SNP15-5030867	MYO5C	15	50308675	7	0.42227
SNP15-5031524	MYO5C	15	50315243	3	0.082598
rs8041208	WDR72	15	51595449	1	2.336336
rs3206108	WDR72	15	51595909	1	1.869337
SNP15-5179038	WDR72	15	51790383	7	0.488803
SNP15-5179038	WDR72	15	51790383	1	1.914343
SNP15-5179038	WDR72	15	51790383	3	0.744726
rs1061823	RAB27A	15	53283335	4	0.731802
rs1061823	RAB27A	15	53283335	3	0.109198
rs1061823	RAB27A	15	53283335	6	5.823609
rs1061823	RAB27A	15	53283335	1	5.351239
rs3203152	CCPG1	15	53451512	5	0.064885
rs17819126	DYX1C1	15	53577202	4	0.157885

rs3743205	DYX1C1	15	53577822	5	2.863773
rs11550869	NEDD4	15	53907424	4	0.543551
rs11550869	NEDD4	15	53907424	7	6.771495
rs2899593	NEDD4	15	53908013	8	5.246649
SNP15-53995103	NEDD4	15	53995103	6	1.160585
rs8032922	LOC729530	15	54082828	1	2.717786
rs8032922	LOC729530	15	54082828	4	1.313708
rs8032922	LOC729530	15	54082828	7	2.643753
rs17819312	LOC729530	15	54084719	1	6.407156
rs2165461	LOC145783	15	54997919	2	4.382111
rs2165461	LOC145783	15	54997919	7	0.415485
rs2165461	LOC145783	15	54997919	4	0.788749
rs2165461	LOC145783	15	54997919	5	1.143269
rs12442879	TCF12	15	55312274	7	0.221628
rs734101	LOC283663	15	55385763	4	1.196179
SNP15-55518865	CGNL1	15	55518865	8	0.308993
SNP15-55518865	CGNL1	15	55518865	2	4.302724
rs2131730	CGNL1	15	55627080	1	5.143535
rs2131731	CGNL1	15	55627123	1	3.75235
rs3742966	CGNL1	15	55628028	1	0.984962
rs3742966	CGNL1	15	55628028	8	0.792154
rs3742966	CGNL1	15	55628028	4	1.04048
rs937248	CGNL1	15	55628061	1	5.459523
rs2069133	Gcom1	15	55705361	3	0.633334
rs2069133	Gcom1	15	55705361	5	0.825779
rs2069133	Gcom1	15	55705361	1	2.722882
rs11858659	GRINL1A	15	55786596	3	0.342945
rs11858659	GRINL1A	15	55786596	1	3.766988
rs9325	ALDH1A2	15	56033278	8	3.407534
rs9325	ALDH1A2	15	56033278	6	0.900011
rs1061278	ALDH1A2	15	56033321	3	2.328717
rs1061278	ALDH1A2	15	56033321	6	1.434626
rs1061278	ALDH1A2	15	56033321	8	1.349463
rs1063666	ALDH1A2	15	56033894	8	0.541122
rs1063666	ALDH1A2	15	56033894	6	0.454828
rs4646643	ALDH1A2	15	56033988	3	0.40614
rs3204690	ALDH1A2	15	56034060	6	1.069275
rs3204690	ALDH1A2	15	56034060	3	0.190739
rs3204690	ALDH1A2	15	56034060	2	0.727108
rs3204689	ALDH1A2	15	56034094	6	0.729639
rs16940856	FAM63B	15	56934840	2	6.40548
rs7164756	GTF2A2	15	57736917	1	2.129944
rs7164756	GTF2A2	15	57736917	4	0.592084
SNP15-58440497	ANXA2	15	58440497	3	1.619441
SNP15-58440497	ANXA2	15	58440497	4	9.240591
rs17303894	VPS13C	15	59952869	6	2.709215
rs4775538	TLN2	15	60835085	3	0.59935

rs4775538	TLN2	15	60835085	4	0.499439
rs3816988	TLN2	15	60898792	4	1.374184
rs3816988	TLN2	15	60898792	2	0.474249
rs8038919	TLN2	15	60915036	2	3.413013
rs8038919	TLN2	15	60915036	4	2.183334
rs937418	TLN2	15	60918170	2	4.5988
rs937418	TLN2	15	60918170	4	3.247136
rs1043256	CA12	15	61403532	6	6.692531
rs1043256	CA12	15	61403532	1	4.433259
rs1043256	CA12	15	61403532	7	1.100112
rs1043256	CA12	15	61403532	3	1.324699
rs10851731	HERC1	15	61709805	6	3.654179
rs10851731	HERC1	15	61709805	2	6.353594
rs2228517	HERC1	15	61719566	3	0.287639
rs2228517	HERC1	15	61719566	2	1.803379
rs2228517	HERC1	15	61719566	6	3.447918
rs2228517	HERC1	15	61719566	7	0.360441
rs2228517	HERC1	15	61719566	4	0.712763
rs7162473	HERC1	15	61724262	2	3.443615
rs7162473	HERC1	15	61724262	6	1.123223
SNP15-6177541(	HERC1	15	61775410	6	0.328853
SNP15-61808529	HERC1	15	61808529	5	0.110555
rs749504	SNX1	15	62217660	5	0.08532
rs749504	SNX1	15	62217660	1	4.98213
rs6494466	CSNK1G1	15	62295816	1	4.895463
rs6494466	CSNK1G1	15	62295816	5	0.096775
rs2010875	PLEKHO2	15	62944535	1	1.030112
rs2010875	PLEKHO2	15	62944535	4	0.906729
rs11853777	PUNC	15	63408191	1	1.10889
rs2280345	NOPE	15	63461230	8	1.28395
rs2280345	NOPE	15	63461230	4	0.604411
SNP15-63490678	NOPE	15	63490678	1	0.475407
SNP15-63490678	NOPE	15	63490678	5	0.082477
SNP15-63490678	NOPE	15	63490678	2	4.429177
rs422390	PTPLAD1	15	63609876	8	6.294011
rs3743169	C15orf44	15	63658492	8	4.931328
rs11484	C15orf44	15	63658751	8	7.472484
GA012458	C15orf44	15	63664227	4	1.498426
rs4366668	C15orf44	15	63690451	7	5.584761
rs4366668	C15orf44	15	63690451	4	0.145946
rs4366668	C15orf44	15	63690451	5	0.676211
rs4366668	C15orf44	15	63690451	3	1.188146
rs1369312	DENND4A	15	63871461	3	0.763415
rs1369312	DENND4A	15	63871461	2	1.050994
rs1369312	DENND4A	15	63871461	4	0.418645
rs1369312	DENND4A	15	63871461	1	0.485222
rs1369312	DENND4A	15	63871461	7	0.965256

rs11634482	MEGF11	15	63977021	6	0.347909
rs11634482	MEGF11	15	63977021	7	0.171088
rs6494602	LOC390597	15	64888338	5	0.771818
rs41305272	MAP2K5	15	65886497	7	0.781874
rs41305272	MAP2K5	15	65886497	2	6.899925
rs11071991	CALML4	15	66285106	7	0.667434
rs11071991	CALML4	15	66285106	5	4.190878
rs11071991	CALML4	15	66285106	4	0.271325
rs11071991	CALML4	15	66285106	1	0.556413
rs4777035	ITGA11	15	66392223	1	2.830831
rs4777035	ITGA11	15	66392223	6	0.966947
rs4777035	ITGA11	15	66392223	3	0.457052
rs2271725	ITGA11	15	66396701	1	4.484761
rs3736493	ITGA11	15	66400841	6	1.209859
rs3736493	ITGA11	15	66400841	4	0.93183
rs3736493	ITGA11	15	66400841	3	0.390976
rs7168069	ITGA11	15	66411450	7	0.826958
rs7168069	ITGA11	15	66411450	6	1.211282
rs7168069	ITGA11	15	66411450	3	1.160013
rs7168069	ITGA11	15	66411450	4	0.420813
rs2306023	ITGA11	15	66415197	4	0.679069
rs2306023	ITGA11	15	66415197	3	0.154984
rs2306023	ITGA11	15	66415197	6	0.677205
rs12907890	ITGA11	15	66436641	7	3.211197
rs12907890	ITGA11	15	66436641	8	0.142501
rs17279860	CORO2B	15	66806013	4	2.774868
rs17279860	CORO2B	15	66806013	6	3.194854
SNP15-6711528(	NOX5	15	67115280	1	0.18366
SNP15-6711528(	NOX5	15	67115280	5	0.625821
SNP15-6711528(	NOX5	15	67115280	2	2.963035
SNP15-6711528(	NOX5	15	67115280	3	0.126421
rs16953674	GLCE	15	67350228	5	0.759763
rs16953466	LOC145837	15	67644716	8	0.023719
rs1057865	TLE3	15	68132680	1	4.796903
rs1057865	TLE3	15	68132680	3	0.696422
rs1057865	TLE3	15	68132680	8	7.476464
rs1057865	TLE3	15	68132680	5	0.794599
rs17759219	TLE3	15	68140022	5	0.184894
rs17759219	TLE3	15	68140022	1	4.589426
rs17759219	TLE3	15	68140022	4	0.045557
rs17759219	TLE3	15	68140022	7	0.107202
rs4777310	LOC729611	15	68876192	7	1.195969
rs2959174	THAP10	15	68971332	7	0.665236
rs2959174	THAP10	15	68971332	5	0.20569
rs2959174	THAP10	15	68971332	8	3.157577
rs2959174	THAP10	15	68971332	4	6.240277
rs2955035	THAP10	15	68971549	7	2.708

rs2955035	THAP10	15	68971549	5	0.235046
rs2955035	THAP10	15	68971549	4	3.84594
rs2955035	THAP10	15	68971549	8	0.322526
SNP15-69063542	LRRC49	15	69063542	6	7.707871
SNP15-69063542	LRRC49	15	69063542	5	1.057104
SNP15-69063542	LRRC49	15	69063542	8	0.915971
SNP15-69063542	LRRC49	15	69063542	4	0.312625
SNP15-69063542	LRRC49	15	69063542	7	0.624451
rs17811309	THSD4	15	69860243	6	3.478158
rs10468050	THSD4	15	69860993	5	0.113451
rs2625529	MYO9A	15	69905831	6	1.23061
rs2625529	MYO9A	15	69905831	7	5.526649
rs13858	SENP8	15	70220198	7	0.815761
rs13858	SENP8	15	70220198	8	0.526741
rs13858	SENP8	15	70220198	6	1.120779
rs1800428	HEXA	15	70455359	8	5.110207
rs1131854	NEO1	15	71351934	2	4.880328
rs1131854	NEO1	15	71351934	4	3.795438
rs11574479	CD276	15	71782226	8	5.691521
rs11574479	CD276	15	71782226	4	1.548292
rs11574479	CD276	15	71782226	1	4.469094
rs2291012	CD276	15	71787782	8	0.712968
rs2291012	CD276	15	71787782	4	0.519598
rs2291012	CD276	15	71787782	1	4.94011
rs11574491	CD276	15	71789046	1	1.559385
rs11574491	CD276	15	71789046	4	0.336572
rs1048661	LOXL1	15	72006599	4	1.766069
rs1048661	LOXL1	15	72006599	7	0.782696
rs1048661	LOXL1	15	72006599	3	1.416441
rs3825942	LOXL1	15	72006635	5	0.378958
rs3825942	LOXL1	15	72006635	8	1.361911
rs3825942	LOXL1	15	72006635	1	2.21173
rs11854957	ISLR	15	72254849	7	1.380905
rs11854957	ISLR	15	72254849	6	0.476775
rs11854957	ISLR	15	72254849	2	0.841367
rs11854957	ISLR	15	72254849	1	0.457678
rs11854957	ISLR	15	72254849	3	0.554122
rs1052622	ISLR	15	72254909	1	2.527713
rs1052622	ISLR	15	72254909	7	1.68935
rs1052622	ISLR	15	72254909	3	0.003747
rs3848160	ISLR	15	72255547	2	1.965162
rs3848160	ISLR	15	72255547	4	0.202809
rs3848160	ISLR	15	72255547	1	1.076271
rs3848160	ISLR	15	72255547	6	1.414953
rs3888004	ISLR	15	72255819	6	1.943885
rs3888004	ISLR	15	72255819	3	1.728531
rs3888004	ISLR	15	72255819	2	2.345604

rs3888004	ISLR	15	72255819	4	0.499739
rs3888004	ISLR	15	72255819	1	1.891467
rs13259	ISLR	15	72256039	6	0.307003
rs13259	ISLR	15	72256039	2	1.940584
rs13259	ISLR	15	72256039	3	0.651277
rs13259	ISLR	15	72256039	1	0.000764
rs13259	ISLR	15	72256039	7	0.247532
rs13259	ISLR	15	72256039	4	0.306322
rs3848161	ISLR	15	72256150	3	0.240881
rs3848161	ISLR	15	72256150	2	3.201707
rs3848161	ISLR	15	72256150	1	1.078162
rs3848161	ISLR	15	72256150	4	0.105043
rs3848161	ISLR	15	72256150	7	1.99175
rs736118	STRA6	15	72260792	2	2.828983
rs736118	STRA6	15	72260792	4	0.496783
rs11857410	STRA6	15	72275477	6	1.623749
rs11857410	STRA6	15	72275477	4	0.028987
rs11857410	STRA6	15	72275477	1	1.689031
rs11857410	STRA6	15	72275477	3	2.560759
rs11857410	STRA6	15	72275477	7	0.943698
rs1564782	CCDC33	15	72409731	1	0.727564
rs900802	CCDC33	15	72415295	7	0.120983
rs2075594	SEMA7A	15	72489714	5	0.385024
rs2075593	SEMA7A	15	72489831	5	1.430044
rs2075592	SEMA7A	15	72490075	5	0.901795
rs1046146	SEMA7A	15	72493996	5	5.40786
rs1128628	EDC3	15	72710388	7	0.451987
rs1128628	EDC3	15	72710388	3	0.158842
rs3803568	LMAN1L	15	72895689	3	1.771536
rs3743487	CPLX3	15	72909909	3	0.323615
rs9210	ULK3	15	72915554	5	7.851292
rs9210	ULK3	15	72915554	2	2.080749
rs4886615	ULK3	15	72918714	2	3.863259
rs4886615	ULK3	15	72918714	5	0.812604
rs7174129	SCAMP5	15	73092093	3	1.909147
rs13056	SCAMP5	15	73099240	3	2.968979
rs3812943	SCAMP5	15	73099768	3	1.898282
rs1873379	C15orf39	15	73285797	5	0.461118
rs1873379	C15orf39	15	73285797	3	0.57908
rs1873379	C15orf39	15	73285797	6	7.649022
rs1873379	C15orf39	15	73285797	2	3.544368
rs11637068	LOC647520	15	73530055	5	1.708481
rs11637068	LOC647520	15	73530055	1	0.735615
SNP15-73700315	SNUPN	15	73700315	6	4.55944
rs1127649	CSPG4	15	73754109	4	0.51097
rs1127649	CSPG4	15	73754109	2	4.488656
rs1127649	CSPG4	15	73754109	7	0.491395

rs1127649	CSPG4	15	73754109	5	0.089801
rs1127649	CSPG4	15	73754109	8	0.21582
rs1127646	CSPG4	15	73754869	5	0.75552
rs1127646	CSPG4	15	73754869	2	1.663802
SNP15-73757125	CSPG4	15	73757125	5	0.004975
SNP15-73757125	CSPG4	15	73757125	8	1.110234
SNP15-73757125	CSPG4	15	73757125	2	0.820955
SNP15-73757125	CSPG4	15	73757125	4	0.972826
SNP15-73757125	CSPG4	15	73757125	7	0.369663
rs4414463	CSPG4	15	73767167	2	2.02005
rs4414463	CSPG4	15	73767167	4	1.602015
rs4414463	CSPG4	15	73767167	8	0.081284
rs4414463	CSPG4	15	73767167	7	0.986954
SNP15-73804507	ODF3L1	15	73804507	1	1.662007
SNP15-73804507	ODF3L1	15	73804507	8	10.00387
SNP15-73804507	ODF3L1	15	73804507	3	1.655065
SNP15-73804507	ODF3L1	15	73804507	6	1.929588
rs8026234	LOC730054	15	73840625	7	0.344583
SNP15-74283287	C15orf27	15	74283287	1	3.432168
SNP15-74283287	C15orf27	15	74283287	3	0.981711
rs937733	C15orf27	15	74283395	3	0.820147
rs937733	C15orf27	15	74283395	1	1.356387
rs937733	C15orf27	15	74283395	8	1.904615
rs2959850	C15orf27	15	74284272	1	0.398771
rs2959850	C15orf27	15	74284272	7	0.742311
rs2959850	C15orf27	15	74284272	3	0.90256
rs4636879	ISL2	15	74417358	2	0.404051
rs2469225	SCAPER	15	74427713	2	5.380689
SNP15-74513520	SCAPER	15	74513520	8	6.613893
SNP15-74513520	SCAPER	15	74513520	3	0.235336
SNP15-74513520	SCAPER	15	74513520	1	5.054474
rs3812908	SCAPER	15	74963213	1	2.630357
rs3812908	SCAPER	15	74963213	3	0.073671
rs1706848	RCN2	15	75028806	2	3.821775
rs35538044	PSTPIP1	15	75115310	5	2.100283
rs11737	TSPAN3	15	75131848	3	0.419841
rs11737	TSPAN3	15	75131848	1	0.550948
rs11639197	SGK269	15	75190283	7	0.206325
rs11639197	SGK269	15	75190283	6	2.188838
rs17381821	SGK269	15	75190532	1	0.05402
rs17381821	SGK269	15	75190532	3	1.474973
rs17381821	SGK269	15	75190532	4	1.100317
rs17381821	SGK269	15	75190532	2	5.262575
rs17381821	SGK269	15	75190532	6	0.308841
rs12901370	SGK269	15	75190805	3	0.401077
rs12901370	SGK269	15	75190805	2	0.895385
rs12901370	SGK269	15	75190805	1	0.11482

rs4886850	SGK269	15	75191215	7	0.202615
rs4886850	SGK269	15	75191215	4	0.088685
rs1867780	SGK269	15	75194169	3	0.176401
rs1867780	SGK269	15	75194169	2	2.445448
rs1867780	SGK269	15	75194169	1	0.272033
rs1867780	SGK269	15	75194169	6	2.392864
rs1867780	SGK269	15	75194169	4	0.52956
SNP15-75238019	SGK269	15	75238019	7	0.003179
rs1058129	LINGO1	15	75692716	3	1.448363
rs1058129	LINGO1	15	75692716	1	0.967104
rs3144	LINGO1	15	75693259	6	0.88953
rs3144	LINGO1	15	75693259	3	0.585697
rs3144	LINGO1	15	75693259	2	1.987122
rs2271396	LINGO1	15	75694590	3	0.162521
rs2271396	LINGO1	15	75694590	1	2.288563
rs2271397	LINGO1	15	75694830	3	0.202744
rs10519181	TBC1D2B	15	76076030	6	1.24793
rs10519181	TBC1D2B	15	76076030	3	1.052311
rs10519181	TBC1D2B	15	76076030	1	4.677289
rs10519181	TBC1D2B	15	76076030	8	0.779767
rs12593575	hCG_38941	15	76177964	2	1.15506
rs3825847	ACSBG1	15	76250541	5	0.200196
rs2304824	ACSBG1	15	76253182	5	0.272032
rs2304824	ACSBG1	15	76253182	1	0.157729
rs2304824	ACSBG1	15	76253182	7	1.211518
rs17479031	ACSBG1	15	76259009	5	1.538653
rs2280364	WDR61	15	76372161	3	0.401131
rs1127472	CRABP1	15	76419885	3	0.645717
rs13180	IREB2	15	76576543	2	0.758058
rs13180	IREB2	15	76576543	1	0.264763
rs13180	IREB2	15	76576543	6	1.658923
rs13180	IREB2	15	76576543	3	0.732214
rs13180	IREB2	15	76576543	5	0.028311
rs3813571	PSMA4	15	76619847	7	0.977566
rs3813571	PSMA4	15	76619847	4	0.104853
rs3813571	PSMA4	15	76619847	8	3.653184
rs3813571	PSMA4	15	76619847	3	0.801824
rs615470	CHRNA5	15	76673043	1	6.718272
rs615470	CHRNA5	15	76673043	7	0.397569
rs615470	CHRNA5	15	76673043	4	6.033565
rs615470	CHRNA5	15	76673043	8	5.303035
rs660652	CHRNA3	15	76674887	4	1.679704
rs660652	CHRNA3	15	76674887	8	1.248683
rs660652	CHRNA3	15	76674887	7	0.852896
rs660652	CHRNA3	15	76674887	1	4.366459
rs578776	CHRNA3	15	76675455	1	6.053731
rs578776	CHRNA3	15	76675455	4	1.791066

rs578776	CHRNA3	15	76675455	5	1.53879
rs578776	CHRNA3	15	76675455	6	0.818496
rs8040868	CHRNA3	15	76698236	5	1.201499
rs8040868	CHRNA3	15	76698236	7	0.001714
rs8040868	CHRNA3	15	76698236	1	0.816616
rs8192475	CHRNA3	15	76698285	1	1.623692
rs1948	CHRNB4	15	76704454	7	0.067093
rs1948	CHRNB4	15	76704454	1	2.844226
rs12914008	CHRNB4	15	76710560	1	2.274929
rs1964562	LOC646938	15	76833546	7	5.382834
rs1964562	LOC646938	15	76833546	3	4.522306
rs1964562	LOC646938	15	76833546	2	5.022619
rs1964562	LOC646938	15	76833546	4	5.043021
rs3825807	ADAMTS7	15	76876166	2	3.662022
rs3129	CTSH	15	77001270	3	1.101172
SNP15-77011782	CTSH	15	77011782	3	0.536653
SNP15-77011802	CTSH	15	77011802	3	0.721811
rs1036938	CTSH	15	77024302	8	0.386848
rs1036938	CTSH	15	77024302	3	0.749193
rs894780	RASGRF1	15	77085578	1	1.244296
rs1004724	KIAA1024	15	77537362	4	0.165916
rs2297773	KIAA1024	15	77542659	6	0.008216
rs2297773	KIAA1024	15	77542659	4	2.265858
rs17266017	KIAA1024	15	77549580	7	0.999404
rs17266017	KIAA1024	15	77549580	8	1.036276
rs1765255	LOC390617	15	77639533	8	1.611389
rs1765255	LOC390617	15	77639533	6	2.582395
rs1765255	LOC390617	15	77639533	7	2.828959
rs12442408	C15orf37	15	78003380	4	3.468396
rs12442408	C15orf37	15	78003380	1	1.153368
rs12442408	C15orf37	15	78003380	2	3.102492
rs2006863	LOC401822	15	78024287	3	0.474935
rs3826007	BCL2A1	15	78050272	7	0.381928
rs1138357	BCL2A1	15	78050461	7	0.548978
rs1801374	FAH	15	78259616	5	1.994638
rs8033706	ARNT2	15	78673246	3	0.197598
rs7172733	ARNT2	15	78673539	4	0.888012
rs4301984	ARNT2	15	78674486	6	0.61657
rs4301984	ARNT2	15	78674486	3	1.560582
rs4459508	ARNT2	15	78674561	3	0.26698
SNP15-78676603	ARNT2	15	78676603	4	0.53859
rs1139651	ARNT2	15	78677061	4	0.353877
rs1139651	ARNT2	15	78677061	6	3.93572
rs9801	ARNT2	15	78677244	6	0.668996
rs3743040	MESDC2	15	79057196	1	4.952276
rs3743040	MESDC2	15	79057196	7	0.493931
rs2250029	LOC647020	15	79197543	5	0.460421

rs2250029	LOC647020	15	79197543	1	0.437886
rs2250029	LOC647020	15	79197543	3	0.49511
rs4072111	IL16	15	79365194	8	0.1076
rs8031107	IL16	15	79369923	4	0.762769
rs17875557	IL16	15	79389202	2	5.99643
rs8041557	IL16	15	79391159	4	0.077958
rs8041557	IL16	15	79391159	6	0.022323
rs8041557	IL16	15	79391159	7	0.545152
rs17875563	IL16	15	79391348	6	0.020525
rs17875563	IL16	15	79391348	8	0.084881
rs17875563	IL16	15	79391348	4	0.002299
rs17875563	IL16	15	79391348	7	0.128897
rs17875563	IL16	15	79391348	3	0.737494
rs4778641	IL16	15	79391443	6	0.773478
rs4778641	IL16	15	79391443	8	6.510476
rs4778641	IL16	15	79391443	3	1.774387
rs4778641	IL16	15	79391443	7	0.240595
rs4778641	IL16	15	79391443	4	1.396981
SNP15-79452076	TMC3	15	79452076	8	1.886747
SNP15-79452076	TMC3	15	79452076	3	2.095712
SNP15-80230879	EFTUD1	15	80230879	8	1.873503
SNP15-80351046	FAM154B	15	80351046	7	2.238457
SNP15-80351046	FAM154B	15	80351046	2	1.911718
SNP15-80351046	FAM154B	15	80351046	8	3.032353
SNP15-80351046	FAM154B	15	80351046	6	1.370523
SNP15-80351046	FAM154B	15	80351046	4	1.578501
rs1267657	CPEB1	15	81012306	3	0.048817
rs1267657	CPEB1	15	81012306	1	0.243792
rs9806343	AP3B2	15	81131300	8	1.397459
rs1108134	FSD2	15	81225246	6	0.495797
rs1108135	FSD2	15	81225247	5	0.967263
rs1108135	FSD2	15	81225247	3	0.960593
rs1108135	FSD2	15	81225247	4	0.509411
rs8042254	WHDC1	15	81275223	2	1.327853
rs8042254	WHDC1	15	81275223	4	1.123975
rs8042254	WHDC1	15	81275223	5	5.251671
rs3814282	WHDC1	15	81296719	8	4.907795
rs4842860	C15orf40	15	81471291	8	0.592661
rs4842860	C15orf40	15	81471291	3	0.391692
rs4842860	C15orf40	15	81471291	7	0.007102
rs4842860	C15orf40	15	81471291	4	2.550558
rs4842860	C15orf40	15	81471291	1	3.766472
rs17361375	C15orf40	15	81471333	2	0.667468
rs17361375	C15orf40	15	81471333	8	3.276881
rs17361375	C15orf40	15	81471333	5	7.232267
rs17361375	C15orf40	15	81471333	7	1.36574
rs17361375	C15orf40	15	81471333	1	0.092405

rs1989	TM6SF1	15	81572635	6	6.822029
rs1050789	SH3GL3	15	82078223	7	1.495649
rs4842838	ADAMTSL3	15	82373128	4	0.027153
rs4842838	ADAMTSL3	15	82373128	7	1.333575
rs4842838	ADAMTSL3	15	82373128	3	0.056926
SNP15-82402371	ADAMTSL3	15	82402371	1	2.321745
SNP15-82402371	ADAMTSL3	15	82402371	7	1.441795
SNP15-82402371	ADAMTSL3	15	82402371	4	0.29053
rs2277849	ADAMTSL3	15	82430354	6	1.083745
rs2277849	ADAMTSL3	15	82430354	4	3.319384
rs2277849	ADAMTSL3	15	82430354	8	3.803442
rs2277849	ADAMTSL3	15	82430354	1	5.049806
rs2277849	ADAMTSL3	15	82430354	7	0.91473
rs2277849	ADAMTSL3	15	82430354	5	4.627001
rs7176737	ADAMTSL3	15	82442294	3	0.088689
rs2044502	ZSCAN2	15	82965002	3	0.034646
rs12442117	SCAND2	15	82978902	1	2.953651
rs3803403	ALPK3	15	83184149	7	1.794931
rs3803403	ALPK3	15	83184149	4	2.493929
rs3803403	ALPK3	15	83184149	5	1.982631
SNP15-83184713	ALPK3	15	83184713	1	0.387036
rs34122391	ALPK3	15	83203614	2	2.315263
rs3748380	ALPK3	15	83212696	2	1.971178
rs3748380	ALPK3	15	83212696	6	0.474857
rs3748379	ALPK3	15	83213324	6	0.14325
rs893162	ALPK3	15	83215223	2	2.21019
rs12900463	ALPK3	15	83216390	5	3.820265
rs12900463	ALPK3	15	83216390	4	0.59585
rs3803390	SLC28A1	15	83268279	6	5.291672
rs2242046	SLC28A1	15	83279733	1	3.040717
rs2242046	SLC28A1	15	83279733	3	0.861084
rs2242046	SLC28A1	15	83279733	4	1.392708
rs2242046	SLC28A1	15	83279733	7	1.221691
rs2242046	SLC28A1	15	83279733	6	0.330798
rs8025045	SLC28A1	15	83289576	5	0.102736
rs8025045	SLC28A1	15	83289576	1	6.078627
SNP15-83888099	AKAP13	15	83888099	1	4.433695
rs745191	AKAP13	15	83924174	3	1.0852
rs745191	AKAP13	15	83924174	8	4.864185
SNP15-83924368	AKAP13	15	83924368	3	0.235449
SNP15-83924368	AKAP13	15	83924368	1	4.870223
SNP15-83924368	AKAP13	15	83924368	6	0.368506
SNP15-83924368	AKAP13	15	83924368	7	0.377735
SNP15-83924368	AKAP13	15	83924368	4	5.628249
rs2241268	AKAP13	15	84079313	8	0.723928
rs2241268	AKAP13	15	84079313	3	0.328528
SNP15-84087791	AKAP13	15	84087791	7	0.004424

rs11630274	AKAP13	15	84089391	8	5.88699
rs1053992	AKAP13	15	84089522	8	7.571066
rs1053992	AKAP13	15	84089522	4	0.228445
rs1053992	AKAP13	15	84089522	1	4.830869
rs3743326	AKAP13	15	84090883	6	5.511746
rs3743326	AKAP13	15	84090883	7	0.658582
rs3743328	AKAP13	15	84091420	1	5.394112
rs3743328	AKAP13	15	84091420	5	0.259469
rs8110	AKAP13	15	84093328	7	8.609222
rs8110	AKAP13	15	84093328	1	4.119245
rs8110	AKAP13	15	84093328	6	1.470952
rs3743332	KLHL25	15	84104755	8	0.562332
rs3743332	KLHL25	15	84104755	3	1.054426
rs2614657	KLHL25	15	84114052	4	0.557289
rs2614657	KLHL25	15	84114052	5	1.294779
rs2614657	KLHL25	15	84114052	6	0.4929
rs2614657	KLHL25	15	84114052	7	1.631746
rs1566088	AGBL1	15	84607033	6	1.654501
rs10520617	AGBL1	15	84608546	3	1.169602
rs10520618	AGBL1	15	84608765	2	3.23981
rs10520618	AGBL1	15	84608765	4	2.791135
rs10520618	AGBL1	15	84608765	3	3.363131
rs10520618	AGBL1	15	84608765	1	3.102953
rs11858435	AGBL1	15	84615870	1	2.43186
rs11858435	AGBL1	15	84615870	2	3.124928
rs11858435	AGBL1	15	84615870	5	1.573998
rs8027765	ISG20L1	15	86970862	1	5.806359
rs17777460	ISG20L1	15	86974560	6	0.313741
rs3826038	ISG20L1	15	86974661	1	3.58697
rs3743473	ISG20L1	15	86975052	1	4.567061
rs3200942	ISG20	15	86983600	4	0.564145
rs3200942	ISG20	15	86983600	5	0.38752
SNP15-8718765(	ACAN	15	87187656	7	1.114819
rs2351491	ACAN	15	87199109	3	0.366216
rs3743399	ACAN	15	87199334	3	0.07639
rs938608	ACAN	15	87199609	2	0.071268
rs938608	ACAN	15	87199609	3	1.56703
rs4932439	ACAN	15	87202113	3	0.563656
rs3825994	ACAN	15	87202619	3	1.088932
rs3825994	ACAN	15	87202619	2	3.393404
rs1042630	ACAN	15	87203055	3	0.312166
rs1042630	ACAN	15	87203055	6	0.500637
rs1042631	ACAN	15	87203243	3	7.404661
rs17201864	HAPLN3	15	87221978	4	0.79523
SNP15-8725754ε	MFGE8	15	87257548	8	1.658557
SNP15-8725754ε	MFGE8	15	87257548	6	0.181631
SNP15-8725754ε	MFGE8	15	87257548	4	0.615669

SNP15-87257548	MFGE8	15	87257548	1	4.583294
SNP15-87257548	MFGE8	15	87257548	5	4.493024
rs8036512	LOC728003	15	87541377	2	5.551065
rs8036512	LOC728003	15	87541377	5	5.899682
rs2710	RLBP1	15	87554224	6	0.741056
rs2710	RLBP1	15	87554224	5	0.058685
rs3087374	POLG	15	87660998	3	0.184302
SNP15-87824562	RHCG	15	87824562	7	0.545631
SNP15-87824562	RHCG	15	87824562	2	5.771788
rs12900805	KIF7	15	87975828	2	5.297507
rs3803530	KIF7	15	87977077	2	3.485669
rs4316730	WDR93	15	88046257	4	1.088546
rs16974175	WDR93	15	88047340	7	0.463955
rs16974175	WDR93	15	88047340	3	0.4772
rs17238056	WDR93	15	88049823	3	0.131036
rs7163367	WDR93	15	88061149	4	4.356942
rs17240268	ANPEP	15	88148818	4	0.723727
rs1044246	SEMA4B	15	88573204	5	0.527819
rs1044246	SEMA4B	15	88573204	1	5.423566
rs10153004	LOC390637	15	88586064	3	0.088159
rs10153004	LOC390637	15	88586064	8	6.038224
rs4932171	TTLL13	15	88595106	7	3.017625
rs4932171	TTLL13	15	88595106	5	2.304981
rs2063742	TTLL13	15	88601412	7	7.790635
rs2063742	TTLL13	15	88601412	5	0.630906
rs11910	LOC400446	15	88621725	5	0.688379
rs11910	LOC400446	15	88621725	7	0.602054
rs2271552	LOC728477	15	88639397	4	0.213635
rs2271552	LOC728477	15	88639397	1	3.944099
rs8042825	ZNF774	15	88698889	5	5.895599
rs8042825	ZNF774	15	88698889	4	0.4726
rs8042825	ZNF774	15	88698889	7	1.215701
rs11854320	ZNF774	15	88704506	8	5.497652
rs6226	FURIN	15	89225578	5	6.741079
rs6226	FURIN	15	89225578	1	4.09718
rs6226	FURIN	15	89225578	7	10.62772
rs6226	FURIN	15	89225578	6	3.984322
rs6226	FURIN	15	89225578	3	0.367224
SNP15-89260479	MAN2A2	15	89260479	8	0.22913
SNP15-89260479	MAN2A2	15	89260479	2	4.7533
SNP15-89260479	MAN2A2	15	89260479	4	0.483272
SNP15-89262549	MAN2A2	15	89262549	4	2.210986
rs1266482	MAN2A2	15	89264356	4	8.181539
rs1266479	MAN2A2	15	89265836	8	0.92224
rs1266479	MAN2A2	15	89265836	2	4.005661
rs1266479	MAN2A2	15	89265836	4	0.217372
rs8034551	UNC45A	15	89287309	8	0.522329

rs8034551	UNC45A	15	89287309	2	4.70838
rs16945091	RCCD1	15	89300085	6	1.077754
rs16945091	RCCD1	15	89300085	1	0.762155
rs16945091	RCCD1	15	89300085	8	0.356526
rs16945091	RCCD1	15	89300085	2	4.363621
rs17600420	ST8SIA2	15	90812139	1	2.702978
rs1455773	C15orf32	15	90816431	1	0.183155
rs7174854	C15orf32	15	90844980	1	0.421276
rs496942	LOC400451	15	90962842	8	1.496697
rs496942	LOC400451	15	90962842	6	0.659607
rs4777755	CHD2	15	91311607	4	6.970104
rs11074121	CHD2	15	91322608	4	0.343053
rs8025851	MCTP2	15	92685112	1	6.181946
rs8025851	MCTP2	15	92685112	6	0.211075
rs8032343	LOC728800	15	94747879	3	0.386202
rs7180888	LOC388181	15	95102199	3	1.627696
rs7180888	LOC388181	15	95102199	5	0.719242
SNP15-96313435	ARRDC4	15	96313435	8	8.544279
rs1521484	LOC145814	15	97329316	3	0.636353
rs1521484	LOC145814	15	97329316	5	0.35011
rs2715423	LOC145814	15	97329396	3	0.44944
SNP15-97487788	DMN	15	97487788	5	6.884954
rs1670227	DMN	15	97488041	5	1.559893
rs1670227	DMN	15	97488041	3	0.193825
SNP15-97489288	DMN	15	97489288	2	6.138457
rs2602016	TTC23	15	97532875	3	1.195951
rs2602016	TTC23	15	97532875	2	4.255262
rs2602016	TTC23	15	97532875	5	1.184504
rs2573652	ADAMTS17	15	98332137	4	0.381716
rs2573652	ADAMTS17	15	98332137	2	1.268313
rs2573652	ADAMTS17	15	98332137	6	2.254083
rs2573652	ADAMTS17	15	98332137	8	1.731749
rs2573652	ADAMTS17	15	98332137	1	2.571922
SNP15-98510368	ADAMTS17	15	98510368	4	0.331411
SNP15-98510368	ADAMTS17	15	98510368	2	2.134216
rs4369638	ADAMTS17	15	98611886	1	2.513154
rs4369638	ADAMTS17	15	98611886	5	0.192728
rs4369638	ADAMTS17	15	98611886	3	0.529214
rs4965613	ADAMTS17	15	98619221	7	0.877541
rs4965613	ADAMTS17	15	98619221	3	0.749567
rs7496640	ADAMTS17	15	98638990	1	3.530854
rs7496640	ADAMTS17	15	98638990	7	0.03258
rs7496640	ADAMTS17	15	98638990	8	0.074816
rs7496668	ADAMTS17	15	98639099	1	2.310697
rs7496668	ADAMTS17	15	98639099	8	0.265165
rs7496668	ADAMTS17	15	98639099	7	0.12913
rs4965643	FLJ42289	15	98707907	3	1.016541

rs12157	LINS1	15	98927317	3	0.037739
rs12157	LINS1	15	98927317	2	4.479515
rs2411837	LINS1	15	98927825	2	4.434504
rs2411837	LINS1	15	98927825	3	1.350943
rs748386	LOC440313	15	99143607	4	0.228549
rs748386	LOC440313	15	99143607	2	2.927892
rs748386	LOC440313	15	99143607	7	0.202566
rs7045	ALDH1A3	15	99274306	8	1.593461
rs7045	ALDH1A3	15	99274306	1	3.699585
rs2412000	LRRK1	15	99347117	5	0.140661
rs4965778	LRRK1	15	99409446	1	0.803041
rs4965778	LRRK1	15	99409446	5	0.547329
rs4965778	LRRK1	15	99409446	3	0.067033
rs11857803	LRRK1	15	99415723	7	0.156968
rs11857803	LRRK1	15	99415723	1	2.544786
rs11857803	LRRK1	15	99415723	6	0.475588
rs11857803	LRRK1	15	99415723	3	1.083258
rs11857803	LRRK1	15	99415723	5	0.459943
rs1048327	LRRK1	15	99427260	8	7.945312
rs3784526	CHSY1	15	99534320	2	4.445931
rs3784526	CHSY1	15	99534320	3	0.085497
rs2005180	CHSY1	15	99536223	5	5.424181
rs7175303	CHSY1	15	99609128	4	0.017337
rs7175303	CHSY1	15	99609128	6	0.855958
rs12438654	SELS	15	99628816	5	4.867952
rs2949	PCSK6	15	99705290	7	0.159954
rs2949	PCSK6	15	99705290	5	0.308873
rs2949	PCSK6	15	99705290	4	1.97689
rs2949	PCSK6	15	99705290	3	0.014969
rs2949	PCSK6	15	99705290	1	1.249957
rs20543	PCSK6	15	99728073	3	2.156394
rs20543	PCSK6	15	99728073	5	1.580465
rs1135911	PCSK6	15	99742069	3	2.004787
rs1135911	PCSK6	15	99742069	7	0.490546
rs2939587	TM2D3	15	100010071	3	0.940767
rs216590	hCG_1644884	16	41263	5	0.418499
rs216590	hCG_1644884	16	41263	1	0.769045
rs216590	hCG_1644884	16	41263	7	0.418518
rs2541593	hCG_1644884	16	43423	7	0.592556
rs2541593	hCG_1644884	16	43423	5	0.658803
rs2541593	hCG_1644884	16	43423	2	0.778071
rs2541593	hCG_1644884	16	43423	1	0.846738
rs1045001	C16orf33	16	47275	7	3.397736
rs1045001	C16orf33	16	47275	1	0.069299
rs216598	RHBDF1	16	48585	2	0.575716
rs216598	RHBDF1	16	48585	1	0.033381
rs216598	RHBDF1	16	48585	7	0.239635

rs3176380	MPG	16	69266	7	1.520492
rs11248850	LOC728037	16	103598	7	0.0203
rs11248850	LOC728037	16	103598	4	0.286297
rs11248850	LOC728037	16	103598	2	1.24674
rs11865131	LOC728037	16	103667	7	0.642855
rs11865131	LOC728037	16	103667	2	0.831773
rs11865131	LOC728037	16	103667	4	0.085582
rs11865131	LOC728037	16	103667	8	0.186236
rs2541608	C16orf35	16	128606	5	0.73045
rs2541608	C16orf35	16	128606	4	0.984916
rs2541608	C16orf35	16	128606	1	4.215411
rs2685126	RGS11	16	264053	1	3.096408
rs2685126	RGS11	16	264053	5	0.087385
rs443338	ARHGDIG	16	272993	7	1.806003
rs443338	ARHGDIG	16	272993	6	1.358581
rs419949	PDIA2	16	274891	1	1.354321
rs419949	PDIA2	16	274891	5	1.304484
rs419949	PDIA2	16	274891	3	0.063504
rs2685127	PDIA2	16	275374	5	0.815138
rs400037	PDIA2	16	276397	5	0.770619
rs400037	PDIA2	16	276397	1	4.024113
rs400037	PDIA2	16	276397	3	0.567326
rs11647490	PDIA2	16	276661	2	1.97661
rs11647490	PDIA2	16	276661	7	0.709283
rs11647490	PDIA2	16	276661	5	2.104624
rs11647490	PDIA2	16	276661	3	1.399439
rs11647490	PDIA2	16	276661	1	0.633978
rs34665498	AXIN1	16	277692	2	4.045132
rs214250	AXIN1	16	288223	5	0.70801
rs214250	AXIN1	16	288223	1	5.963004
rs214250	AXIN1	16	288223	3	0.510935
rs758033	AXIN1	16	337045	1	5.527774
rs3743887	TMEM8	16	364278	2	2.928174
rs3743887	TMEM8	16	364278	4	0.501218
rs3213574	SOLH	16	541633	1	3.148338
rs3213574	SOLH	16	541633	3	0.101993
rs3213574	SOLH	16	541633	6	1.277739
rs2071979	PIGQ	16	564115	3	0.132127
rs2071979	PIGQ	16	564115	6	0.054271
rs2071979	PIGQ	16	564115	4	1.323467
rs35227916	PIGQ	16	565960	2	5.488682
rs1045274	PIGQ	16	570903	1	5.066921
rs1045274	PIGQ	16	570903	3	0.069551
rs2269557	RAB40C	16	617630	6	5.32469
rs2269557	RAB40C	16	617630	5	0.381745
rs2269557	RAB40C	16	617630	4	0.810697
rs2269557	RAB40C	16	617630	1	4.446559

SNP16-638252	C16orf14	16	638252	1	2.802906
rs13337278	WDR90	16	640645	6	0.500832
rs11642546	WDR90	16	641657	1	0.639945
SNP16-645361	WDR90	16	645361	4	0.756426
SNP16-645361	WDR90	16	645361	6	0.125169
SNP16-645361	WDR90	16	645361	1	4.244731
SNP16-645361	WDR90	16	645361	5	0.155873
rs28485323	WDR90	16	646535	1	3.070174
rs28485323	WDR90	16	646535	5	0.70425
rs28485323	WDR90	16	646535	4	0.921995
SNP16-648276	WDR90	16	648276	4	0.146865
SNP16-648276	WDR90	16	648276	1	4.901245
SNP16-648276	WDR90	16	648276	5	0.281337
rs4984906	WDR90	16	649002	1	0.503639
rs4984906	WDR90	16	649002	5	0.265137
rs2301426	WDR90	16	651906	5	0.488358
SNP16-655991	WDR90	16	655991	4	2.401318
SNP16-655991	WDR90	16	655991	5	0.040294
SNP16-655991	WDR90	16	655991	1	2.723488
SNP16-655991	WDR90	16	655991	6	0.130417
rs9934288	WDR90	16	656288	6	5.071492
rs11557858	RHOT2	16	659593	6	1.57853
rs3743912	RHOT2	16	660961	6	4.816232
rs1139897	RHOT2	16	660987	1	3.119322
rs1139897	RHOT2	16	660987	4	1.119286
rs1139897	RHOT2	16	660987	5	2.401431
SNP16-662332	RHOT2	16	662332	4	4.80376
SNP16-662332	RHOT2	16	662332	5	2.133713
SNP16-662332	RHOT2	16	662332	1	3.565364
SNP16-662332	RHOT2	16	662332	6	0.468901
rs4984683	FBXL16	16	686885	5	0.809339
rs4984683	FBXL16	16	686885	4	0.19027
rs4984683	FBXL16	16	686885	1	3.594942
rs12599130	CCDC78	16	712843	6	0.330857
rs12599130	CCDC78	16	712843	4	0.708802
rs12599130	CCDC78	16	712843	5	0.044629
rs12599130	CCDC78	16	712843	1	4.224697
rs2071950	CCDC78	16	714693	5	0.181523
SNP16-758560	MSLN	16	758560	8	0.096566
SNP16-758560	MSLN	16	758560	6	2.061692
rs3765263	CHTF18	16	780379	1	1.54
rs3765265	CHTF18	16	780598	1	3.921904
rs1065368	SOX8	16	976861	3	0.286958
rs1065368	SOX8	16	976861	8	6.546796
rs1065368	SOX8	16	976861	4	5.18155
rs1065368	SOX8	16	976861	1	4.994851
rs2076422	C1QTNF8	16	1086229	5	2.74813

rs8044363	CACNA1H	16	1190560	5	0.985924
rs8044363	CACNA1H	16	1190560	8	0.977733
rs8044363	CACNA1H	16	1190560	4	0.925068
rs8044363	CACNA1H	16	1190560	1	1.105327
rs4984636	CACNA1H	16	1192442	1	1.958485
rs1054645	CACNA1H	16	1210163	3	0.467346
rs4247094	CACNA1H	16	1210350	5	1.645156
GA030918	CACNA1H	16	1211190	3	0.14147
GA030918	CACNA1H	16	1211190	6	2.122504
GA030918	CACNA1H	16	1211190	5	1.078817
rs1004041	TPSG1	16	1211891	1	0.593712
rs1004041	TPSG1	16	1211891	3	0.378773
rs1004041	TPSG1	16	1211891	5	0.61408
rs11248860	TPSG1	16	1212039	6	1.133617
rs11248860	TPSG1	16	1212039	5	1.012506
rs1139155	TPSB2	16	1218635	3	0.390136
rs11866635	BAIAP3	16	1331133	1	2.099226
rs1132356	BAIAP3	16	1334508	3	0.488556
rs1132358	BAIAP3	16	1337816	1	0.618835
rs1132358	BAIAP3	16	1337816	7	0.808584
rs1132358	BAIAP3	16	1337816	5	0.384745
rs4984817	BAIAP3	16	1338819	3	1.163976
rs4984817	BAIAP3	16	1338819	7	0.478758
rs11865640	NULL	16	1355300	1	7.23905
rs12935737	CLCN7	16	1444438	3	0.42495
rs2745097	LOC390667	16	1476500	7	0.5154
rs2235624	TELO2	16	1485449	5	0.117179
rs2235624	TELO2	16	1485449	8	0.380018
rs2235624	TELO2	16	1485449	3	0.673164
rs3180228	TELO2	16	1499839	1	2.300833
rs3180228	TELO2	16	1499839	5	1.146832
rs3180228	TELO2	16	1499839	3	0.551534
rs9922661	TMEM204	16	1545164	1	4.167515
rs9922661	TMEM204	16	1545164	3	0.771837
rs9922661	TMEM204	16	1545164	5	0.947785
rs9922661	TMEM204	16	1545164	7	2.300277
rs1057610	TMEM204	16	1545538	3	0.375779
rs1057610	TMEM204	16	1545538	5	0.776547
rs1057610	TMEM204	16	1545538	7	1.389908
rs1057610	TMEM204	16	1545538	1	5.125999
rs1894649	IFT140	16	1597285	3	1.247546
rs1894649	IFT140	16	1597285	7	0.813318
rs1894649	IFT140	16	1597285	5	0.629413
rs1894649	IFT140	16	1597285	1	3.053956
rs2294616	MAPK8IP3	16	1752973	3	0.447882
rs2294619	MAPK8IP3	16	1754441	5	1.144525
rs344359	NUBP2	16	1778641	2	0.207662

rs344359	NUBP2	16	1778641	1	0.329235
rs344359	NUBP2	16	1778641	5	7.532146
rs3751893	IGFALS	16	1782210	1	1.913056
rs3751893	IGFALS	16	1782210	2	1.830684
rs3751893	IGFALS	16	1782210	5	1.524612
rs3817902	IGFALS	16	1783673	5	0.608995
rs3817902	IGFALS	16	1783673	3	0.486363
rs3817902	IGFALS	16	1783673	1	0.503787
rs1057070	HAGH	16	1799154	1	2.106979
rs28364709	HAGH	16	1817176	1	7.153746
rs3743852	FAHD1	16	1817699	5	0.304132
rs3743852	FAHD1	16	1817699	3	0.584167
rs3813760	FAHD1	16	1829646	1	4.665915
rs3848348	FAHD1	16	1830057	1	3.313163
rs9806826	C16orf73	16	1834913	3	0.560039
rs9806826	C16orf73	16	1834913	5	0.39141
rs9806826	C16orf73	16	1834913	1	1.75315
rs9806945	C16orf73	16	1834962	1	2.02681
rs8055325	HS3ST6	16	1902133	1	3.798695
rs8055325	HS3ST6	16	1902133	3	2.297766
SNP16-1970128	NOXO1	16	1970128	8	0.263734
SNP16-1975869	GFER	16	1975869	8	4.71252
SNP16-1975869	GFER	16	1975869	5	0.485299
rs11860328	GFER	16	1977596	2	1.063178
SNP16-1989004	ZNF598	16	1989004	8	0.957862
SNP16-1989004	ZNF598	16	1989004	2	3.382631
SNP16-1989004	ZNF598	16	1989004	5	0.725005
rs11248905	ZNF598	16	1989729	8	5.038267
rs11556528	ZNF598	16	1990193	2	1.364465
rs11556528	ZNF598	16	1990193	8	0.270868
rs41292293	SLC9A3R2	16	2026967	1	5.140484
GA012845	SLC9A3R2	16	2028411	2	3.35068
rs34012042	TSC2	16	2054408	7	4.465786
rs45498900	TSC2	16	2078683	5	0.863524
rs3087631	PKD1	16	2079128	4	0.699237
rs26862	GBL	16	2197106	3	0.230853
rs26862	GBL	16	2197106	4	0.686907
SNP16-2200568	C16orf79	16	2200568	3	0.540947
SNP16-2200568	C16orf79	16	2200568	7	0.279244
SNP16-2200613	C16orf79	16	2200613	7	0.255237
SNP16-2200613	C16orf79	16	2200613	3	1.150489
rs1126	LOC283871	16	2202114	7	0.519446
rs1126	LOC283871	16	2202114	4	0.469757
rs1126	LOC283871	16	2202114	3	0.046122
rs149532	ABCA3	16	2271431	1	3.349579
rs323043	ABCA3	16	2288529	6	3.253562
rs323043	ABCA3	16	2288529	5	0.237899

rs17758402	LOC441744	16	2322355	3	0.402434
rs4332747	ABCA17	16	2400424	4	3.637478
rs10866	PDPK1	16	2587877	7	0.845797
GA012858	PDPK1	16	2591815	1	4.386871
SNP16-2820746	LOC124220	16	2820746	6	0.508604
SNP16-2820746	LOC124220	16	2820746	1	4.544541
rs886953	LOC124220	16	2822268	2	1.825318
rs886953	LOC124220	16	2822268	5	1.963545
rs886953	LOC124220	16	2822268	1	1.587729
rs886953	LOC124220	16	2822268	4	0.638954
rs886953	LOC124220	16	2822268	3	2.43495
rs886953	LOC124220	16	2822268	6	2.136633
rs12786	PRSS22	16	2842833	8	4.32111
rs12786	PRSS22	16	2842833	4	0.153996
rs12786	PRSS22	16	2842833	6	1.177764
rs12786	PRSS22	16	2842833	2	4.986504
rs12786	PRSS22	16	2842833	7	5.169744
rs1063677	FLYWCH2	16	2885249	7	0.548353
rs1063677	FLYWCH2	16	2885249	4	7.491523
rs9673618	LOC729718	16	2892424	7	0.224838
rs9673618	LOC729718	16	2892424	4	0.398875
rs2074365	FLYWCH1	16	2904284	6	0.748629
rs2074365	FLYWCH1	16	2904284	8	3.788945
rs2074365	FLYWCH1	16	2904284	4	1.25664
rs8064223	CLDN6	16	3004864	4	0.372411
rs8064223	CLDN6	16	3004864	8	4.446899
rs8064223	CLDN6	16	3004864	7	3.879023
rs2269911	CLDN6	16	3005925	7	5.679938
rs2269911	CLDN6	16	3005925	2	0.57214
rs2269911	CLDN6	16	3005925	8	0.031387
rs10508	HCFC1R1	16	3013241	8	5.88358
rs10508	HCFC1R1	16	3013241	5	0.288293
rs10508	HCFC1R1	16	3013241	7	0.613928
SNP16-3019686	CCDC64B	16	3019686	5	0.647028
SNP16-3019686	CCDC64B	16	3019686	8	7.446038
SNP16-3019686	CCDC64B	16	3019686	1	4.479336
SNP16-3019686	CCDC64B	16	3019686	6	5.861197
rs11862977	CCDC64B	16	3021319	2	2.244532
rs11862977	CCDC64B	16	3021319	8	1.488579
rs11862977	CCDC64B	16	3021319	7	0.518044
rs11862977	CCDC64B	16	3021319	5	1.413332
SNP16-3025336	CCDC64B	16	3025336	1	3.037859
SNP16-3025336	CCDC64B	16	3025336	8	5.379934
SNP16-3025336	CCDC64B	16	3025336	5	4.43848
rs10431961	NULL	16	3040096	5	0.84577
rs10431961	NULL	16	3040096	1	0.989304
rs10431961	NULL	16	3040096	6	5.441366

rs10431961	NULL	16	3040096	2	1.110052
rs1064948	NULL	16	3046995	5	5.443422
rs1064948	NULL	16	3046995	4	1.159275
rs1064948	NULL	16	3046995	7	5.836298
rs11228	ZNF200	16	3212392	5	0.126985
rs11228	ZNF200	16	3212392	6	0.358382
rs224206	MEFV	16	3237074	1	1.328368
rs224206	MEFV	16	3237074	5	0.359527
rs224207	MEFV	16	3237176	5	0.52572
rs224208	MEFV	16	3237182	5	1.031435
rs224213	MEFV	16	3239750	1	1.491962
rs224213	MEFV	16	3239750	5	0.192223
rs220379	ZNF263	16	3279436	7	0.422122
rs220380	ZNF263	16	3280846	7	0.748842
rs1228348	OR2C1	16	3345995	3	0.980515
rs27241	ZNF434	16	3387283	7	1.357475
rs27241	ZNF434	16	3387283	1	6.216853
rs37810	ZNF174	16	3391882	7	0.150543
rs37810	ZNF174	16	3391882	1	2.777591
rs37824	ZNF597	16	3426237	7	2.018432
rs37824	ZNF597	16	3426237	1	6.899368
rs1137454	FLJ14154	16	3473578	7	0.302232
SNP16-3534317	NLRC3	16	3534317	3	1.92965
rs3810813	BTBD12	16	3579828	3	0.552587
rs3810813	BTBD12	16	3579828	2	4.842103
rs8061528	BTBD12	16	3596483	2	2.767886
rs8061528	BTBD12	16	3596483	3	0.21843
rs8061528	BTBD12	16	3596483	4	0.192823
rs1136948	TRAP1	16	3661774	4	1.6444
rs1136948	TRAP1	16	3661774	7	3.371143
rs1136948	TRAP1	16	3661774	3	0.065166
rs2240668	SRL	16	4180908	2	0.963274
rs2240668	SRL	16	4180908	7	2.313064
rs10852642	SRL	16	4182029	5	0.372268
rs10852642	SRL	16	4182029	8	0.769118
rs10852642	SRL	16	4182029	1	3.167233
rs10852642	SRL	16	4182029	7	0.709065
rs8051399	SRL	16	4182094	7	0.590242
rs8051399	SRL	16	4182094	1	2.754346
rs8051399	SRL	16	4182094	4	0.464692
rs10852643	SRL	16	4185694	5	0.43239
rs10852643	SRL	16	4185694	1	1.271325
rs10852643	SRL	16	4185694	7	0.35061
rs868302	SRL	16	4197687	3	0.148587
rs2304633	DNAJA3	16	4436974	8	0.060924
rs2304633	DNAJA3	16	4436974	6	0.892107
rs2304633	DNAJA3	16	4436974	5	0.09036

rs2304633	DNAJA3	16	4436974	7	0.058452
rs17137053	NMRAL1	16	4464156	5	0.437868
rs17137056	NMRAL1	16	4464686	5	5.70069
rs8129	C16orf5	16	4502352	8	7.500759
rs8129	C16orf5	16	4502352	7	0.197442
rs8129	C16orf5	16	4502352	1	4.992277
rs8129	C16orf5	16	4502352	6	7.428606
rs7665	C16orf5	16	4502513	7	0.257183
rs7665	C16orf5	16	4502513	1	5.686936
rs7665	C16orf5	16	4502513	6	8.780749
rs7195763	LOC342346	16	4589301	5	0.872209
rs3747587	MGRN1	16	4614955	6	0.3003
rs3747587	MGRN1	16	4614955	8	5.711292
rs863980	ANKS3	16	4691046	4	3.236825
rs863980	ANKS3	16	4691046	6	1.060014
rs863980	ANKS3	16	4691046	8	5.387583
rs863980	ANKS3	16	4691046	3	0.713351
rs863980	ANKS3	16	4691046	5	0.586333
rs859305	C16orf71	16	4730274	4	1.634698
rs859305	C16orf71	16	4730274	7	0.874973
rs859305	C16orf71	16	4730274	6	0.32073
rs859305	C16orf71	16	4730274	3	1.436498
rs859305	C16orf71	16	4730274	5	1.345899
rs859305	C16orf71	16	4730274	8	3.046746
rs2075469	C16orf71	16	4734874	4	0.16599
rs2075469	C16orf71	16	4734874	3	1.328665
rs2075469	C16orf71	16	4734874	8	0.911769
rs2075469	C16orf71	16	4734874	5	0.158637
rs2075469	C16orf71	16	4734874	7	1.327714
rs737700	C16orf71	16	4736394	7	2.772839
rs737700	C16orf71	16	4736394	3	0.695722
rs737700	C16orf71	16	4736394	8	1.043199
rs737700	C16orf71	16	4736394	6	2.158009
rs737700	C16orf71	16	4736394	5	2.285438
rs737700	C16orf71	16	4736394	4	1.307751
GA012923	C16orf71	16	4737458	5	1.516262
GA012923	C16orf71	16	4737458	3	0.029554
GA012923	C16orf71	16	4737458	4	6.439401
GA012923	C16orf71	16	4737458	1	2.323224
rs2290560	C16orf71	16	4739042	3	0.817728
rs2290560	C16orf71	16	4739042	1	6.354192
rs2290560	C16orf71	16	4739042	6	0.174173
rs2290560	C16orf71	16	4739042	7	1.070975
rs2290560	C16orf71	16	4739042	5	0.279911
rs2290560	C16orf71	16	4739042	4	0.496067
SNP16-4752706	ZNF500	16	4752706	1	6.164448
rs759991	12-Sep	16	4773971	3	0.382307

rs759991	12-Sep	16	4773971	4	3.104598
rs759991	12-Sep	16	4773971	7	0.927299
rs1045643	UBN1	16	4871514	8	5.380241
rs1049207	PPL	16	4874565	1	5.525055
rs2075639	PPL	16	4875637	8	4.407935
rs2286966	SEC14L5	16	4990898	8	0.124249
rs2286966	SEC14L5	16	4990898	4	1.381478
rs2286966	SEC14L5	16	4990898	3	0.07967
rs2286966	SEC14L5	16	4990898	6	0.627422
rs2286966	SEC14L5	16	4990898	7	0.704339
rs2286966	SEC14L5	16	4990898	1	0.765735
rs2972284	SEC14L5	16	5008153	6	0.557313
rs2072455	SEC14L5	16	5008778	5	5.200843
rs2072455	SEC14L5	16	5008778	7	1.040886
rs8055684	MGC45438	16	5034571	3	0.165455
rs9674145	MGC45438	16	5037948	3	0.621222
rs9674145	MGC45438	16	5037948	4	0.29018
rs9674145	MGC45438	16	5037948	7	0.412163
rs9674145	MGC45438	16	5037948	6	0.871367
rs9674145	MGC45438	16	5037948	5	0.088617
rs741164	MGC45438	16	5046185	6	0.451278
rs741164	MGC45438	16	5046185	7	1.17466
rs7198383	LOC283953	16	8559751	4	1.47703
rs7198383	LOC283953	16	8559751	7	0.707081
rs2270286	C16orf68	16	8630130	4	0.302879
rs2270286	C16orf68	16	8630130	5	0.0887
rs2270286	C16orf68	16	8630130	3	0.118856
rs2270286	C16orf68	16	8630130	1	6.56909
rs1731017	ABAT	16	8747455	6	2.103433
rs1731017	ABAT	16	8747455	2	4.028867
rs1731017	ABAT	16	8747455	7	0.631037
rs1731017	ABAT	16	8747455	3	0.674839
rs2270288	ABAT	16	8783030	4	0.720856
rs2270288	ABAT	16	8783030	3	0.840604
rs2270288	ABAT	16	8783030	7	3.495833
rs2270288	ABAT	16	8783030	8	5.188134
rs3743801	ABAT	16	8783703	4	0.583056
rs3743801	ABAT	16	8783703	3	0.841567
rs3743801	ABAT	16	8783703	7	0.645999
rs4985000	ABAT	16	8783778	1	6.213716
rs4985000	ABAT	16	8783778	6	2.592554
rs4985000	ABAT	16	8783778	7	0.006754
rs1061842	ABAT	16	8784381	8	7.140268
rs1061842	ABAT	16	8784381	7	4.515437
rs1061842	ABAT	16	8784381	3	0.218838
rs1061842	ABAT	16	8784381	4	0.00781
rs1061842	ABAT	16	8784381	2	3.09945

rs9456	ABAT	16	8785092	8	4.855873
rs9456	ABAT	16	8785092	4	3.966244
rs9456	ABAT	16	8785092	1	0.057565
rs9456	ABAT	16	8785092	2	0.563822
rs6898	CARHSP1	16	8856475	1	4.120143
rs1014531	GRIN2A	16	9763295	1	1.187268
rs12444387	LOC727844	16	10038372	4	0.765716
rs12444387	LOC727844	16	10038372	7	0.482972
rs12444387	LOC727844	16	10038372	5	0.594459
rs12444387	LOC727844	16	10038372	6	1.472218
rs4782258	LOC727833	16	10112746	7	0.007355
rs7188171	LOC727833	16	10112913	1	4.973116
rs7188171	LOC727833	16	10112913	6	0.872688
rs7188171	LOC727833	16	10112913	5	3.241865
rs9932051	ATF7IP2	16	10482297	7	0.705231
rs1970817	ATF7IP2	16	10483325	5	0.544613
rs1970817	ATF7IP2	16	10483325	7	0.022452
rs1970817	ATF7IP2	16	10483325	3	1.454902
rs2719710	TEKT5	16	10677459	5	2.176795
rs2719710	TEKT5	16	10677459	7	1.362478
rs2719710	TEKT5	16	10677459	1	0.812774
rs2719710	TEKT5	16	10677459	2	0.465426
rs12918646	TEKT5	16	10683356	1	4.291086
rs12918646	TEKT5	16	10683356	5	1.665003
SNP16-10770465	NUBP1	16	10770465	1	3.7953
SNP16-10770465	NUBP1	16	10770465	5	0.007792
SNP16-10770465	NUBP1	16	10770465	6	0.165712
rs4774	CIITA	16	10908349	1	0.166243
rs4774	CIITA	16	10908349	2	0.374521
rs4774	CIITA	16	10908349	3	0.648645
rs2229320	CIITA	16	10909244	3	0.102175
rs2229320	CIITA	16	10909244	5	0.62915
rs2286973	CLEC16A	16	11062271	3	7.440396
rs2286973	CLEC16A	16	11062271	6	0.406026
rs2286973	CLEC16A	16	11062271	1	4.939555
rs2286973	CLEC16A	16	11062271	7	2.15961
rs11647285	CLEC16A	16	11180073	7	4.925854
rs737008	PRM1	16	11282367	3	0.179754
rs4381620	LOC388210	16	11385258	5	0.213249
rs4381620	LOC388210	16	11385258	6	0.227098
rs1139052	LOC388210	16	11385649	3	0.991098
rs8058507	LOC388210	16	11386346	4	0.404808
rs8058507	LOC388210	16	11386346	1	1.337714
rs4502229	LOC400499	16	11448261	7	0.178194
rs4502229	LOC400499	16	11448261	1	1.504818
rs4502229	LOC400499	16	11448261	5	0.589573
rs8050530	LOC400499	16	11462368	7	1.123522

rs8050530	LOC400499	16	11462368	5	1.925729
rs4781107	LOC400499	16	11479920	7	0.444285
rs4781110	LOC400499	16	11490071	4	0.33959
rs4781110	LOC400499	16	11490071	7	0.066339
rs4781110	LOC400499	16	11490071	1	2.064889
rs4781110	LOC400499	16	11490071	3	0.381876
rs4781110	LOC400499	16	11490071	5	0.740915
rs8052900	RSL1D1	16	11847891	7	0.662341
rs8052900	RSL1D1	16	11847891	3	0.104316
rs8052900	RSL1D1	16	11847891	4	2.96352
rs434762	GSPT1	16	11917282	5	0.637815
rs1641843	LOC92017	16	12070470	7	0.52662
rs1650996	FLJ11151	16	12665747	8	0.130476
rs3748983	FLJ11151	16	12665760	3	0.178972
rs9938751	LOC440338	16	12909860	1	5.073069
rs1800067	ERCC4	16	13936534	7	2.325282
rs1799801	ERCC4	16	13949459	2	2.864585
rs1799801	ERCC4	16	13949459	4	6.483226
rs30128	MKL2	16	14264151	3	0.801257
rs30128	MKL2	16	14264151	6	4.003863
rs30122	MKL2	16	14266734	6	6.237354
rs30122	MKL2	16	14266734	3	0.172619
rs35722504	PARN	16	14448420	6	0.094558
rs4985154	PDXDC1	16	15039143	1	6.972501
rs2740	PDXDC1	16	15039609	1	5.033776
rs2071332	C16orf45	16	15588143	5	0.050844
rs1050163	MYH11	16	15718524	3	1.504681
rs1050163	MYH11	16	15718524	4	0.514327
rs1050162	MYH11	16	15718563	3	0.928121
rs1050162	MYH11	16	15718563	4	0.520173
rs2075511	NDE1	16	15725642	1	1.697992
rs2075511	NDE1	16	15725642	4	0.408472
rs2075511	NDE1	16	15725642	3	0.635818
rs16967494	MYH11	16	15728364	4	0.929644
rs16967494	MYH11	16	15728364	3	0.607625
rs12931799	MYH11	16	15749274	2	4.518111
rs880071	MYH11	16	15749524	1	0.18866
rs2272554	MYH11	16	15757705	4	1.036503
rs2272554	MYH11	16	15757705	3	1.530257
rs2272554	MYH11	16	15757705	1	1.308734
rs35587	ABCC1	16	16047215	8	2.077572
rs35605	ABCC1	16	16069520	8	2.161936
rs35605	ABCC1	16	16069520	4	0.632469
rs8187858	ABCC1	16	16069540	2	3.826755
GA013073	ABCC1	16	16080733	3	0.581018
rs129081	ABCC1	16	16143440	5	0.725041
rs129081	ABCC1	16	16143440	4	0.517435

rs2238472	ABCC6	16	16159100	6	0.055313
rs2238472	ABCC6	16	16159100	1	3.544744
rs2238472	ABCC6	16	16159100	5	0.408237
rs9930886	ABCC6	16	16199484	3	0.719171
rs28709752	XYLT1	16	17199575	4	3.149134
rs17663059	RPS15A	16	18709084	6	1.504014
rs17663059	RPS15A	16	18709084	1	4.921379
SNP16-18949096	TMC7	16	18949096	5	6.68253
SNP16-18949096	TMC7	16	18949096	6	3.953487
rs35480813	COQ7	16	18986538	6	0.25463
rs35480813	COQ7	16	18986538	4	0.493142
rs35480813	COQ7	16	18986538	2	1.816534
rs1548450	LOC728276	16	19217593	3	1.858509
rs1548450	LOC728276	16	19217593	4	1.337749
SNP16-19379072	TMC5	16	19379072	3	5.973903
SNP16-19382600	TMC5	16	19382600	1	4.785526
rs2147865	GPR139	16	19950831	3	0.033496
rs28544423	UMOD	16	20267134	6	1.503626
rs13335818	UMOD	16	20267332	7	0.701328
rs13335818	UMOD	16	20267332	1	0.503344
rs3743690	ACSM1	16	20542919	6	0.461366
rs3743690	ACSM1	16	20542919	2	4.975679
rs1136268	THUMPD1	16	20654605	5	6.004176
rs11074471	THUMPD1	16	20655832	5	0.665165
rs5710	LOC730079	16	20682804	5	0.220114
rs5716	ACSM3	16	20703888	5	0.222987
rs11648835	NULL	16	20716784	5	1.012732
rs7187522	DCUN1D3	16	20781268	2	0.037802
rs7187522	DCUN1D3	16	20781268	8	0.367153
rs7187522	DCUN1D3	16	20781268	7	3.540801
SNP16-20867419	DNAH3	16	20867419	5	0.723553
rs3743697	DNAH3	16	20882804	5	0.340883
SNP16-20883861	DNAH3	16	20883861	5	1.049078
rs12929546	DNAH3	16	20888663	7	3.286825
rs12929546	DNAH3	16	20888663	5	0.762519
rs330150	DNAH3	16	20958710	4	0.114144
SNP16-21046572	DNAH3	16	21046572	1	3.803695
SNP16-21059414	DNAH3	16	21059414	8	0.005582
rs226058	LOC646823	16	21220184	5	1.835808
rs226058	LOC646823	16	21220184	3	1.098455
rs1055740	IGSF6	16	21560221	5	0.426863
SNP16-21562393	IGSF6	16	21562393	6	0.126146
rs1105929	VWA3A	16	22051819	7	0.87578
rs9937453	VWA3A	16	22063130	7	1.033531
rs9937453	VWA3A	16	22063130	1	3.090833
rs17841292	EEF2K	16	22144774	8	5.337849
rs2290829	EEF2K	16	22203987	7	0.054483

rs2290829	EEF2K	16	22203987	8	4.962906
rs208951	HS3ST2	16	22733840	3	1.947254
rs208951	HS3ST2	16	22733840	2	3.504629
rs5737	SCNN1G	16	23108424	2	3.245561
rs5723	SCNN1G	16	23134288	1	3.745595
rs5726	SCNN1G	16	23134527	4	4.580538
rs5729	SCNN1G	16	23134897	3	0.718498
rs5729	SCNN1G	16	23134897	4	2.182971
rs5729	SCNN1G	16	23134897	1	3.992372
rs250587	COG7	16	23307695	5	5.203601
rs2285521	GGA2	16	23429281	5	0.297771
rs6497671	EARS2	16	23444185	5	0.595992
rs7187920	EARS2	16	23471002	3	0.553834
rs7187920	EARS2	16	23471002	5	0.270315
rs26764	ERN2	16	23619426	1	0.809419
rs3809682	ERN2	16	23632254	5	1.059339
rs3809682	ERN2	16	23632254	6	0.792584
rs11646957	CACNG3	16	24174810	4	0.030388
rs11646957	CACNG3	16	24174810	1	1.763952
rs11646957	CACNG3	16	24174810	5	3.422715
rs9922288	RBBP6	16	24458431	1	0.636918
rs9922288	RBBP6	16	24458431	7	0.193886
rs11639856	TNRC6A	16	24696146	4	0.404368
SNP16-24708398	TNRC6A	16	24708398	3	0.316774
SNP16-24708398	TNRC6A	16	24708398	7	0.530026
rs6497759	TNRC6A	16	24709238	4	1.072247
rs13336754	TNRC6A	16	24709480	2	1.775146
rs13336754	TNRC6A	16	24709480	6	0.237167
rs3803716	TNRC6A	16	24709826	4	0.069844
rs17772929	SLC5A11	16	24825549	2	2.439055
rs17772929	SLC5A11	16	24825549	6	0.581288
rs17772929	SLC5A11	16	24825549	4	0.864516
rs2277961	ZKSCAN2	16	25155265	7	6.358545
rs2277961	ZKSCAN2	16	25155265	6	5.981357
rs7184511	ZKSCAN2	16	25156872	2	4.722036
rs7184511	ZKSCAN2	16	25156872	6	7.650516
rs8059494	ZKSCAN2	16	25162743	7	0.564307
rs8059494	ZKSCAN2	16	25162743	6	6.881676
rs11642611	ZKSCAN2	16	25163007	7	0.118888
rs11642611	ZKSCAN2	16	25163007	2	8.236369
rs11649280	ZKSCAN2	16	25165536	7	0.300967
rs3803711	HS3ST4	16	26055412	1	2.688807
rs3803711	HS3ST4	16	26055412	3	0.251385
rs2280183	C16orf82	16	26987458	5	0.604417
rs11823	C16orf82	16	26987807	5	1.180462
rs1134367	JMJD5	16	27140384	6	6.032052
rs1134367	JMJD5	16	27140384	3	0.878225

rs1134367	JMJD5	16	27140384	7	0.802094
rs1134367	JMJD5	16	27140384	8	0.343099
rs1134367	JMJD5	16	27140384	4	5.014609
SNP16-27154118	NSMCE1	16	27154118	3	0.018442
SNP16-27154118	NSMCE1	16	27154118	1	2.014675
rs3024633	IL4R	16	27274000	6	0.54893
rs3024633	IL4R	16	27274000	8	2.615835
rs2234924	IL4R	16	27282740	6	0.71872
rs2285452	IL21R	16	27368398	8	0.338309
rs2285452	IL21R	16	27368398	3	1.503199
rs2285452	IL21R	16	27368398	4	0.706894
rs2285452	IL21R	16	27368398	2	1.596387
rs2285452	IL21R	16	27368398	1	0.4629
rs2285452	IL21R	16	27368398	7	0.215514
rs3093387	IL21R	16	27368569	2	1.030582
rs3093387	IL21R	16	27368569	1	0.184435
rs3093387	IL21R	16	27368569	7	0.722905
rs3093387	IL21R	16	27368569	8	2.235077
rs3093387	IL21R	16	27368569	3	0.880312
rs3093410	IL21R	16	27368903	5	1.167385
rs3093412	IL21R	16	27369022	2	1.59662
rs12919017	GTF3C1	16	27382411	1	3.051449
rs12919017	GTF3C1	16	27382411	7	1.839356
rs12919017	GTF3C1	16	27382411	8	0.219521
rs11644502	KIAA0556	16	27688810	7	0.094929
rs2292646	GSG1L	16	27709838	3	0.874251
rs9939733	LOC647001	16	28159357	5	0.005446
rs9939733	LOC647001	16	28159357	3	0.175089
rs9939733	LOC647001	16	28159357	1	0.500567
rs11540497	SBK1	16	28242495	1	3.841272
rs151233	APOB48R	16	28413929	5	0.561821
rs151233	APOB48R	16	28413929	7	0.735722
rs149271	APOB48R	16	28414373	1	1.369279
rs180743	APOB48R	16	28415145	1	0.198316
rs40831	APOB48R	16	28415948	1	2.267012
rs40837	IL27	16	28418346	1	1.134214
rs181206	IL27	16	28420904	1	1.264758
rs11074908	SULT1A2	16	28515857	7	2.50387
rs2077412	SULT1A1	16	28528812	1	5.884319
rs2077412	SULT1A1	16	28528812	4	0.232335
rs2077412	SULT1A1	16	28528812	5	1.631421
rs2077412	SULT1A1	16	28528812	3	1.718964
rs2077412	SULT1A1	16	28528812	8	0.950727
rs2077412	SULT1A1	16	28528812	6	4.171271
rs7193733	SH2B1	16	28782983	1	0.033812
rs7193733	SH2B1	16	28782983	8	0.334812
rs7193733	SH2B1	16	28782983	2	2.893339

rs1131543	LAT	16	28905498	5	0.01906
rs1131543	LAT	16	28905498	1	5.079443
rs1646087	LOC728804	16	29047272	3	0.837804
rs889682	LOC728809	16	29053044	7	2.223617
rs889682	LOC728809	16	29053044	2	1.23649
rs889682	LOC728809	16	29053044	3	1.199879
rs2303255	QPRT	16	29613945	6	4.398495
rs2303255	QPRT	16	29613945	2	4.240165
rs2303255	QPRT	16	29613945	7	0.766949
rs2303255	QPRT	16	29613945	3	0.590402
rs1045968	PRRT2	16	29733866	2	3.387184
rs1045968	PRRT2	16	29733866	6	0.733484
SNP16-29891874	LOC124446	16	29891874	1	0.636829
SNP16-29891874	LOC124446	16	29891874	7	0.226388
rs3814883	TAOK2	16	29902423	8	2.05786
rs3814883	TAOK2	16	29902423	2	4.325074
rs3814883	TAOK2	16	29902423	3	0.689105
rs3814883	TAOK2	16	29902423	6	0.065959
rs1129003	DOC2A	16	29924784	1	5.976987
rs3935873	DOC2A	16	29926001	6	1.797957
rs12444973	YPEL3	16	30014786	1	0.581318
rs12444973	YPEL3	16	30014786	7	0.344077
rs12444973	YPEL3	16	30014786	2	1.089103
rs12444973	YPEL3	16	30014786	6	2.664824
rs7698	MAPK3	16	30033301	1	3.672895
rs7698	MAPK3	16	30033301	8	7.408096
rs7542	MAPK3	16	30033341	2	6.257843
rs7542	MAPK3	16	30033341	8	6.5417
rs7542	MAPK3	16	30033341	6	4.797835
rs7542	MAPK3	16	30033341	3	0.013579
rs1132812	CORO1A	16	30105652	5	1.589548
rs1132812	CORO1A	16	30105652	3	0.089485
rs8051096	LOC647068	16	30255232	8	1.113243
rs8051096	LOC647068	16	30255232	7	0.905626
rs8051096	LOC647068	16	30255232	6	1.008508
rs8051096	LOC647068	16	30255232	3	3.220717
rs8051096	LOC647068	16	30255232	2	0.307462
rs8051096	LOC647068	16	30255232	5	1.219293
rs4787643	1-Sep	16	30301161	3	0.789928
rs4787643	1-Sep	16	30301161	5	0.320165
rs12921440	ZNF553	16	30316266	1	0.556805
rs12921440	ZNF553	16	30316266	2	1.110462
rs12921440	ZNF553	16	30316266	5	0.328198
rs12921440	ZNF553	16	30316266	3	0.492655
rs12921440	ZNF553	16	30316266	8	5.644839
rs2230434	ITGAL	16	30425597	2	4.486777
rs10871453	ZNF768	16	30444419	1	0.660122

rs10871453	ZNF768	16	30444419	7	5.777703
rs10871453	ZNF768	16	30444419	3	0.857063
rs9934806	ZNF785	16	30500560	6	0.620562
rs8046001	LOC647086	16	30740822	1	0.728215
rs8046001	LOC647086	16	30740822	3	1.610055
rs8046001	LOC647086	16	30740822	7	1.343495
rs11642733	SETD1A	16	30876562	8	3.485462
rs11642733	SETD1A	16	30876562	6	0.916224
rs11642733	SETD1A	16	30876562	1	5.531435
rs11642733	SETD1A	16	30876562	5	0.323184
SNP16-30906699	HSD3B7	16	30906699	1	5.746521
SNP16-30906699	HSD3B7	16	30906699	5	3.989056
SNP16-30906699	HSD3B7	16	30906699	6	7.262484
SNP16-30906699	HSD3B7	16	30906699	8	0.229939
rs2305880	HSD3B7	16	30906963	3	0.2415
rs2305880	HSD3B7	16	30906963	5	0.140493
rs2305880	HSD3B7	16	30906963	7	0.298423
rs2305880	HSD3B7	16	30906963	1	4.421078
rs2305880	HSD3B7	16	30906963	6	0.034238
rs2303223	ZNF668	16	30982676	2	1.580901
rs2303223	ZNF668	16	30982676	1	0.7602
rs2303223	ZNF668	16	30982676	3	0.527542
rs2303223	ZNF668	16	30982676	7	5.524139
rs749671	ZNF646	16	30995848	7	0.516413
rs749671	ZNF646	16	30995848	1	0.012156
rs3751855	ZNF646	16	30998710	6	0.018884
rs3751855	ZNF646	16	30998710	3	0.378722
rs3751855	ZNF646	16	30998710	1	3.981559
rs3751855	ZNF646	16	30998710	7	0.393175
rs3751855	ZNF646	16	30998710	5	4.717323
rs7199949	POL3S	16	31003665	3	0.030806
rs7199949	POL3S	16	31003665	7	0.560529
rs7199949	POL3S	16	31003665	1	3.157783
rs7199949	POL3S	16	31003665	5	0.392496
rs7199949	POL3S	16	31003665	6	0.068526
SNP16-31049772	MYST1	16	31049772	1	0.522715
SNP16-31049772	MYST1	16	31049772	2	5.759734
rs1549299	PRSS36	16	31061647	3	0.581689
SNP16-31101443	FUS	16	31101443	8	4.868756
rs11861251	ITGAM	16	31196897	7	0.271611
SNP16-31244220	ITGAM	16	31244220	5	0.253238
rs1143683	ITGAM	16	31244389	7	1.332435
rs1143678	ITGAM	16	31250506	7	0.470416
rs1143678	ITGAM	16	31250506	5	0.412691
SNP16-31250736	ITGAM	16	31250736	8	0.984753
SNP16-31250736	ITGAM	16	31250736	7	1.046386
SNP16-31250736	ITGAM	16	31250736	6	0.93132

SNP16-31250736	ITGAM	16	31250736	4	0.483602
SNP16-31250744	ITGAM	16	31250744	8	1.046875
SNP16-31250887	ITGAM	16	31250887	2	3.997274
SNP16-31250887	ITGAM	16	31250887	7	3.519027
SNP16-31250887	ITGAM	16	31250887	5	2.046334
rs4597342	ITGAM	16	31251270	6	0.384837
rs11574633	ITGAX	16	31274819	7	1.81172
rs11574633	ITGAX	16	31274819	4	1.376574
rs11574633	ITGAX	16	31274819	6	1.823848
rs2929	ITGAX	16	31300809	1	0.344004
rs1140195	ITGAX	16	31301680	6	0.940718
rs12444660	ITGAD	16	31329930	2	0.03727
rs3813002	ARMC5	16	31378041	4	0.231174
rs3813002	ARMC5	16	31378041	2	1.373085
rs3813002	ARMC5	16	31378041	6	3.233868
rs3813002	ARMC5	16	31378041	8	3.105216
rs3813002	ARMC5	16	31378041	1	0.126251
rs11150624	ARMC5	16	31383959	5	0.443426
rs11150624	ARMC5	16	31383959	3	0.831514
rs10596	TGFB1I1	16	31396417	3	0.474267
rs10596	TGFB1I1	16	31396417	5	0.951507
rs10596	TGFB1I1	16	31396417	4	0.684669
rs11862739	ZNF720	16	31678525	8	4.616069
rs11862739	ZNF720	16	31678525	6	0.408733
rs33994299	ORC6L	16	45281099	3	0.02036
SNP16-46680083	ABCC12	16	46680083	5	1.196284
SNP16-46761579	ABCC11	16	46761579	8	0.487583
rs12443685	ABCC11	16	46783980	4	0.607309
rs17822471	ABCC11	16	46799880	5	0.086497
rs8047091	ABCC11	16	46806419	2	3.451585
rs8047091	ABCC11	16	46806419	8	1.045105
rs8047091	ABCC11	16	46806419	5	0.65636
rs17822931	ABCC11	16	46815699	1	4.097671
rs17822931	ABCC11	16	46815699	7	1.587307
rs17822931	ABCC11	16	46815699	3	1.204814
rs16945988	ABCC11	16	46823278	2	1.319287
rs2062553	LOC643114	16	47088672	6	8.047429
rs2062553	LOC643114	16	47088672	8	5.218537
rs2062553	LOC643114	16	47088672	2	5.464539
rs2062553	LOC643114	16	47088672	1	4.811702
rs1039341	N4BP1	16	47132370	8	6.558361
rs7197732	N4BP1	16	47133379	2	2.7725
rs7197732	N4BP1	16	47133379	6	4.202934
rs7197732	N4BP1	16	47133379	8	5.975886
rs3743776	CBLN1	16	47870626	1	2.9311
SNP16-48228678	ZNF423	16	48228678	1	0.263092
SNP16-48228678	ZNF423	16	48228678	7	0.092074

rs12913	NKD1	16	49225927	5	0.565154
rs12913	NKD1	16	49225927	1	1.934571
rs12913	NKD1	16	49225927	2	1.67756
rs5743266	NOD2	16	49288597	7	0.03526
rs2066843	NOD2	16	49302700	6	0.208691
rs2066843	NOD2	16	49302700	5	0.516436
rs2066843	NOD2	16	49302700	8	0.42768
rs2066843	NOD2	16	49302700	7	0.398529
rs2066845	NOD2	16	49314041	6	2.634817
rs11645288	SALL1	16	49730178	7	0.845801
rs11645288	SALL1	16	49730178	3	0.580987
rs10221156	LOC390730	16	51526927	7	0.035937
SNP16-51899304	CHD9	16	51899304	1	2.706874
SNP16-51899304	CHD9	16	51899304	4	0.863384
rs4784320	RPGRIP1L	16	52193501	7	0.678652
rs2111119	RPGRIP1L	16	52229255	8	1.252278
rs2111119	RPGRIP1L	16	52229255	7	1.136249
SNP16-52277937	RPGRIP1L	16	52277937	7	0.109788
SNP16-52875673	IRX3	16	52875673	7	0.034311
SNP16-52875673	IRX3	16	52875673	4	7.080605
rs2588994	LOC654106	16	53915182	7	0.561159
rs2008953	IRX6	16	53921960	8	0.105853
rs2008953	IRX6	16	53921960	7	1.688702
rs2008953	IRX6	16	53921960	3	0.534483
rs1132896	MMP2	16	54077036	8	0.291333
rs1053605	MMP2	16	54077108	8	6.195953
rs1053605	MMP2	16	54077108	7	0.16519
rs10775332	MMP2	16	54094228	8	0.729561
rs10775332	MMP2	16	54094228	2	4.906795
rs5569	SLC6A2	16	54289336	1	0.23009
rs5569	SLC6A2	16	54289336	3	0.419442
rs7194256	SLC6A2	16	54295192	6	0.649099
rs11864688	CES7	16	54437824	1	0.945906
rs2397965	CES7	16	54465312	2	6.121874
rs7194945	CES7	16	54466664	1	1.955218
rs1065375	GNAO1	16	54926190	1	0.032366
rs1065375	GNAO1	16	54926190	2	0.470356
rs1065375	GNAO1	16	54926190	4	0.985891
rs1065375	GNAO1	16	54926190	5	0.489464
rs1065375	GNAO1	16	54926190	3	0.415089
rs1065375	GNAO1	16	54926190	8	0.399219
rs1065375	GNAO1	16	54926190	7	6.976961
rs1799917	GNAO1	16	54935249	5	0.205049
rs1799917	GNAO1	16	54935249	4	0.399578
rs11544885	OGFOD1	16	55057677	7	0.257249
rs17841923	BBS2	16	55111619	7	0.997455
rs11643815	MT4	16	55160299	3	3.606388

rs11643815	MT4	16	55160299	1	4.096873
GA031500	MT1E	16	55218452	1	4.708094
rs37024	HERPUD1	16	55523572	3	1.472771
rs37024	HERPUD1	16	55523572	5	2.081919
rs37024	HERPUD1	16	55523572	7	1.798334
rs9497	HERPUD1	16	55535075	2	0.502702
rs5882	CETP	16	55573593	5	1.228953
SNP16-55616928	NLRC5	16	55616928	3	0.479325
SNP16-55617854	NLRC5	16	55617854	3	0.715404
SNP16-55617854	NLRC5	16	55617854	5	0.239117
SNP16-55617854	NLRC5	16	55617854	8	8.735778
rs13339199	NLRC5	16	55618225	1	5.563685
rs13339199	NLRC5	16	55618225	5	4.641929
rs289723	NLRC5	16	55638029	7	0.830324
rs289723	NLRC5	16	55638029	2	0.988674
rs289723	NLRC5	16	55638029	3	1.053314
rs7190199	NLRC5	16	55658841	8	0.659417
rs4784800	CCL22	16	55957003	2	4.218938
rs178602	CIAPIN1	16	56038806	5	0.333414
rs178602	CIAPIN1	16	56038806	4	0.617469
rs223869	COQ9	16	56052493	5	0.189174
GA031518	DOK4	16	56071040	6	2.007081
rs9922763	CCDC102A	16	56103647	5	0.255569
rs28446687	CCDC102A	16	56112577	5	0.193786
rs28446687	CCDC102A	16	56112577	6	0.697038
rs1801257	GPR56	16	56246886	3	0.703409
rs1801257	GPR56	16	56246886	8	4.20757
rs1801257	GPR56	16	56246886	5	7.626573
rs12444859	GPR97	16	56279829	5	0.083644
rs12444859	GPR97	16	56279829	4	0.082124
rs12444859	GPR97	16	56279829	8	0.435555
rs11648336	GPR97	16	56280077	1	0.014873
rs11648336	GPR97	16	56280077	7	1.106151
rs11648336	GPR97	16	56280077	5	0.132218
rs11648684	CCDC135	16	56290389	1	3.057817
rs11648684	CCDC135	16	56290389	3	0.217265
rs7196016	CCDC135	16	56293401	3	0.043013
rs7196016	CCDC135	16	56293401	5	0.625065
rs2923147	CCDC135	16	56319902	1	2.404282
rs1061546	KATNB1	16	56328627	1	4.060856
rs1061546	KATNB1	16	56328627	7	5.425585
rs1061546	KATNB1	16	56328627	6	0.038771
rs7200390	LOC388282	16	56408106	3	1.473205
rs10459809	CNGB1	16	56506725	6	0.197454
rs10459809	CNGB1	16	56506725	3	0.723395
rs376270	CNGB1	16	56507558	5	1.174966
rs2161703	CNGB1	16	56511949	3	0.911688

rs12927214	CNGB1	16	56514717	3	1.445826
rs9934227	TEPP	16	56576897	7	0.883594
rs9934227	TEPP	16	56576897	5	3.369513
rs9934227	TEPP	16	56576897	1	0.558754
rs3743554	ZNF319	16	56587693	1	3.396079
rs3743554	ZNF319	16	56587693	3	0.651384
rs3743554	ZNF319	16	56587693	8	5.562853
SNP16-56601412	C16orf57	16	56601412	3	0.765974
rs3743563	MMP15	16	56636666	5	0.103104
rs3743563	MMP15	16	56636666	8	0.236497
rs3743563	MMP15	16	56636666	6	1.490326
rs3743574	C16orf80	16	56720597	4	0.229329
rs17241091	Klkbl4	16	56871449	1	5.710029
rs1052276	Klkbl4	16	56872099	6	0.457631
rs1052276	Klkbl4	16	56872099	4	3.245443
rs1052276	Klkbl4	16	56872099	5	0.439562
rs16960029	CCDC113	16	56872626	6	1.058867
rs16960029	CCDC113	16	56872626	4	0.333319
rs16960029	CCDC113	16	56872626	5	0.525855
rs16960032	CCDC113	16	56873082	5	0.555052
rs16960032	CCDC113	16	56873082	4	0.035837
rs16960035	CCDC113	16	56873132	5	0.790844
rs16960035	CCDC113	16	56873132	6	0.777992
rs16960035	CCDC113	16	56873132	4	0.018077
rs3743580	CCDC113	16	56874016	5	0.619439
rs3743580	CCDC113	16	56874016	4	4.360149
rs42945	NDRG4	16	57102927	3	0.975783
rs42945	NDRG4	16	57102927	1	0.126951
rs42945	NDRG4	16	57102927	7	1.90675
rs1058132	NDRG4	16	57103798	4	0.275431
rs1058132	NDRG4	16	57103798	3	0.712609
rs1058132	NDRG4	16	57103798	1	5.91298
rs1058132	NDRG4	16	57103798	7	5.136789
rs4784964	FLJ10815	16	57258304	6	0.820368
rs7191331	FLJ10815	16	57271299	1	6.114413
rs7191331	FLJ10815	16	57271299	4	0.296488
rs6993	GOT2	16	57298868	6	0.315676
rs6993	GOT2	16	57298868	3	0.248983
rs6993	GOT2	16	57298868	2	9.750479
rs30842	GOT2	16	57300955	6	0.205986
rs30842	GOT2	16	57300955	3	0.742116
SNP16-57325630	GOT2	16	57325630	4	1.828637
rs35213	CDH11	16	63573588	8	1.105957
rs35213	CDH11	16	63573588	6	0.178911
rs35213	CDH11	16	63573588	2	0.845387
rs35213	CDH11	16	63573588	3	1.855588
rs35195	CDH11	16	63583219	7	0.392582

rs12444408	LOC283867	16	64167662	5	0.845837
rs12444408	LOC283867	16	64167662	3	1.22483
rs355944	CMTM4	16	65209746	6	4.811281
rs363162	CCDC79	16	65370298	1	3.410344
rs152164	NAE1	16	65419467	1	1.309278
rs152164	NAE1	16	65419467	2	1.95146
SNP16-65594555	FLJ37464	16	65594555	7	0.192024
rs2233455	NOL3	16	65765434	7	0.727666
rs12923138	ELMO3	16	65790767	2	0.965549
rs12923138	ELMO3	16	65790767	8	0.280488
rs12923138	ELMO3	16	65790767	1	0.422552
rs12051247	LRRC29	16	65798783	7	1.064648
SNP16-65799431	LRRC29	16	65799431	7	1.098532
rs7186310	SLC9A5	16	65863014	7	1.32634
rs8050745	PLEKHG4	16	65869268	2	1.593434
rs11860295	PLEKHG4	16	65873735	2	4.665608
rs9922085	LRRC36	16	65955081	2	1.358568
rs16957415	LRRC36	16	65976458	2	2.14812
rs1053612	TPPP3	16	65981440	2	3.520708
rs11552319	TPPP3	16	65981623	2	3.526176
rs5479	HSD11B2	16	66027234	2	6.128364
GA013416	HSD11B2	16	66028604	7	6.583283
rs4360931	ATP6V0D1	16	66029427	7	0.225414
rs4474673	RANBP10	16	66316279	8	8.090074
SNP16-66477550	NRN1L	16	66477550	8	1.491396
SNP16-66477550	NRN1L	16	66477550	7	1.825997
SNP16-66477550	NRN1L	16	66477550	6	4.05019
rs1134760	CTRL	16	66521704	1	0.231705
rs1134760	CTRL	16	66521704	7	0.346223
rs1134760	CTRL	16	66521704	8	3.71067
rs1134760	CTRL	16	66521704	6	4.242541
rs1134760	CTRL	16	66521704	2	0.263048
rs4986970	LCAT	16	66533821	8	7.023939
rs11860125	SLC12A4	16	66538470	1	0.029143
rs11860125	SLC12A4	16	66538470	2	3.295974
rs11542821	SLC12A4	16	66542090	1	0.550044
rs11542821	SLC12A4	16	66542090	2	0.710807
rs1133090	DPEP2	16	66578969	1	0.463325
rs13816	DDX28	16	66612996	6	0.15718
rs13816	DDX28	16	66612996	7	4.654556
rs13816	DDX28	16	66612996	4	2.252098
rs237831	DDX28	16	66614597	3	0.105794
rs12598	NFATC3	16	66783016	2	2.620507
rs3743739	LYPLA3	16	66850821	1	0.895461
rs3743739	LYPLA3	16	66850821	2	6.121294
rs3743739	LYPLA3	16	66850821	8	6.012573
rs3743739	LYPLA3	16	66850821	4	4.669218

rs12599876	SMPD3	16	66950906	6	2.124724
rs12599876	SMPD3	16	66950906	7	0.626343
rs1532211	SMPD3	16	66951808	1	4.09891
rs1532211	SMPD3	16	66951808	5	0.855815
rs1532211	SMPD3	16	66951808	7	0.748905
rs1532211	SMPD3	16	66951808	4	1.340731
rs2279539	SMPD3	16	66952595	7	0.122953
rs2279539	SMPD3	16	66952595	1	1.299526
rs1170444	ZFP90	16	67130788	1	3.929147
rs1170444	ZFP90	16	67130788	5	0.966172
rs1170443	ZFP90	16	67130796	5	4.037733
rs1170443	ZFP90	16	67130796	1	4.001575
rs1126933	CDH3	16	67279034	4	5.716966
rs1126933	CDH3	16	67279034	3	0.485111
rs2232227	HAS3	16	67700982	7	0.717293
rs2232227	HAS3	16	67700982	1	3.275169
rs2232228	HAS3	16	67701078	2	1.157223
rs2232228	HAS3	16	67701078	3	0.836195
rs2232228	HAS3	16	67701078	6	3.272864
rs2232228	HAS3	16	67701078	5	1.338175
rs2232228	HAS3	16	67701078	4	0.275537
rs2232228	HAS3	16	67701078	1	1.261685
SNP16-67758551	CIRH1A	16	67758551	1	3.378669
SNP16-67861549	SNTB2	16	67861549	3	1.126688
rs1127231	VPS4A	16	67912464	7	0.832314
rs1127231	VPS4A	16	67912464	2	3.340837
rs1127231	VPS4A	16	67912464	5	1.196591
rs1127231	VPS4A	16	67912464	3	1.13198
rs1127231	VPS4A	16	67912464	8	0.953888
rs1127231	VPS4A	16	67912464	1	1.353123
GA031593	VPS4A	16	67912656	3	0.431272
rs1055401	PDF	16	67920278	7	6.693046
rs1055401	PDF	16	67920278	1	5.092177
rs11542583	COG8	16	67930915	8	3.812378
rs11542583	COG8	16	67930915	2	2.046446
rs11542583	COG8	16	67930915	1	0.785358
rs11542583	COG8	16	67930915	3	6.428479
rs11542583	COG8	16	67930915	5	1.128996
rs11641233	NFAT5	16	68292064	1	0.997365
rs11641233	NFAT5	16	68292064	7	0.362702
rs11641233	NFAT5	16	68292064	2	0.622799
rs11641233	NFAT5	16	68292064	4	0.223421
rs11641233	NFAT5	16	68292064	8	0.676692
rs2965757	NFAT5	16	68295347	8	1.871289
rs2965757	NFAT5	16	68295347	6	5.441938
rs689453	NQO1	16	68309874	3	0.148045
rs1075935	NOB1	16	68333878	4	0.862679

rs1075935	NOB1	16	68333878	1	0.808407
rs1075935	NOB1	16	68333878	2	5.330387
rs1075935	NOB1	16	68333878	7	0.94875
rs1875245	WWP2	16	68520904	6	5.910637
rs1875245	WWP2	16	68520904	4	0.028433
rs2270841	WWP2	16	68525398	1	6.059133
rs2270841	WWP2	16	68525398	5	0.440244
rs2270841	WWP2	16	68525398	8	0.436793
rs2270841	WWP2	16	68525398	6	0.16218
rs1983016	WWP2	16	68527830	6	3.784473
rs1983016	WWP2	16	68527830	5	0.618293
rs1983016	WWP2	16	68527830	8	5.686357
rs1983016	WWP2	16	68527830	1	5.136553
rs2270844	WWP2	16	68530798	3	0.010867
rs3192882	WWP2	16	68533132	5	0.081296
rs3192882	WWP2	16	68533132	2	4.755489
rs3192882	WWP2	16	68533132	3	0.519104
rs9926963	PDPR	16	68752144	6	0.510351
rs9926963	PDPR	16	68752144	4	0.362618
rs11537663	AARS	16	68844051	1	0.942122
rs11537663	AARS	16	68844051	2	0.699513
rs11537663	AARS	16	68844051	7	0.537998
rs11537663	AARS	16	68844051	4	5.628827
rs1134074	DDX19A	16	68952888	3	0.241874
rs1134074	DDX19A	16	68952888	7	3.976625
rs1134074	DDX19A	16	68952888	5	0.212026
rs7192865	FUK	16	69064408	4	0.647791
rs7192865	FUK	16	69064408	7	0.556191
rs7192865	FUK	16	69064408	3	0.413472
rs7192865	FUK	16	69064408	6	0.36511
rs7192865	FUK	16	69064408	2	1.324516
rs17884050	FUK	16	69067102	6	0.742277
rs33910368	SF3B3	16	69126716	6	5.097774
rs33910368	SF3B3	16	69126716	4	6.255003
rs33910368	SF3B3	16	69126716	3	0.095481
rs33910368	SF3B3	16	69126716	7	0.493954
rs33910368	SF3B3	16	69126716	1	0.157573
rs33910368	SF3B3	16	69126716	2	1.090189
rs3813904	C16orf77	16	69238245	4	1.123154
rs8046424	C16orf77	16	69248490	4	1.531166
rs4985556	C16orf77	16	69251501	4	3.521911
rs4985556	C16orf77	16	69251501	6	1.140325
rs1022217	HYDIN	16	69618793	8	2.52129
rs1022217	HYDIN	16	69618793	7	0.566509
SNP16-6977825	HYDIN	16	69778256	8	1.456547
rs17851296	CALB2	16	69969137	5	0.106606
rs17851296	CALB2	16	69969137	4	0.023799

rs17851296	CALB2	16	69969137	6	0.04184
rs17851296	CALB2	16	69969137	3	0.104382
rs17851296	CALB2	16	69969137	7	0.690089
rs17851296	CALB2	16	69969137	1	1.403784
rs4620964	LOC390738	16	70023589	1	1.309336
rs4620964	LOC390738	16	70023589	5	0.300068
rs4620964	LOC390738	16	70023589	6	0.44012
rs4620964	LOC390738	16	70023589	3	0.082268
rs1609868	LOC729556	16	70047794	4	0.700671
rs1609868	LOC729556	16	70047794	5	0.068799
rs1609868	LOC729556	16	70047794	3	0.424566
rs2303788	LOC729556	16	70055419	4	0.308231
rs2303788	LOC729556	16	70055419	5	0.28534
rs2303788	LOC729556	16	70055419	3	1.79162
rs2303788	LOC729556	16	70055419	2	3.862309
rs2288489	ZNF19	16	70067186	5	0.00116
rs2288489	ZNF19	16	70067186	8	0.129373
rs10500557	ZNF19	16	70067280	8	8.715527
rs8050871	ZNF19	16	70067297	5	0.196365
rs8050871	ZNF19	16	70067297	2	1.443716
rs7184486	NULL	16	70444031	1	4.923528
rs7184486	NULL	16	70444031	4	0.995212
rs2291947	NULL	16	70447990	7	2.170921
rs2291947	NULL	16	70447990	8	0.501419
rs2291947	NULL	16	70447990	5	0.068651
rs2291947	NULL	16	70447990	3	0.167217
rs3088285	NULL	16	70448051	8	6.04109
rs3088285	NULL	16	70448051	1	4.097905
rs6558	KIAA0174	16	70520092	4	1.472433
rs7195988	PKD1L3	16	70520687	4	5.80513
rs7195988	PKD1L3	16	70520687	8	4.285952
rs7195988	PKD1L3	16	70520687	1	4.544195
rs17358402	PKD1L3	16	70525428	4	0.406689
rs17358402	PKD1L3	16	70525428	1	4.117237
rs17358402	PKD1L3	16	70525428	8	2.987173
rs1035543	PKD1L3	16	70541273	3	1.648769
rs9921412	PKD1L3	16	70545607	1	2.543134
rs9921412	PKD1L3	16	70545607	4	0.878779
rs1559400	PKD1L3	16	70568694	3	0.279368
rs4788591	PKD1L3	16	70577824	3	0.643399
rs3213422	DHODH	16	70600183	5	0.217138
rs3213422	DHODH	16	70600183	7	0.214473
rs3213422	DHODH	16	70600183	1	3.70367
SNP16-70614922	DHODH	16	70614922	5	0.529895
rs5475	HP	16	70651849	6	1.19363
rs5475	HP	16	70651849	7	2.117028
rs5475	HP	16	70651849	4	2.31844

rs11642506	HPR	16	70665366	4	0.46173
rs1050362	DHX38	16	70688316	5	6.908885
rs4788459	PMFBP1	16	70710626	1	4.877574
rs16973716	PMFBP1	16	70714343	4	0.452361
rs16973716	PMFBP1	16	70714343	1	4.176984
rs16973716	PMFBP1	16	70714343	7	0.902913
rs34832584	PMFBP1	16	70720467	1	4.382964
rs34832584	PMFBP1	16	70720467	7	0.855998
rs34832584	PMFBP1	16	70720467	4	1.193359
SNP16-70742067	PMFBP1	16	70742067	1	3.082284
SNP16-70742067	PMFBP1	16	70742067	4	0.587592
rs217180	PMFBP1	16	70742130	4	1.207022
rs217180	PMFBP1	16	70742130	6	0.675506
rs699444	ZFHX3	16	71385259	1	3.120054
rs699444	ZFHX3	16	71385259	8	7.05317
rs699444	ZFHX3	16	71385259	3	1.746215
SNP16-71551361	ZFHX3	16	71551361	4	0.001025
SNP16-71551361	ZFHX3	16	71551361	5	4.543794
rs8058133	RFWD3	16	73214986	2	0.196952
rs8058133	RFWD3	16	73214986	6	3.870047
rs8058133	RFWD3	16	73214986	4	3.078756
rs8058133	RFWD3	16	73214986	7	0.121684
rs8058133	RFWD3	16	73214986	1	0.043719
SNP16-73229367	RFWD3	16	73229367	1	0.53967
SNP16-73229367	RFWD3	16	73229367	2	0.220705
rs4887783	RFWD3	16	73258244	4	1.466491
rs4887783	RFWD3	16	73258244	2	0.809854
rs4887783	RFWD3	16	73258244	6	0.179668
rs4887783	RFWD3	16	73258244	7	0.012935
rs4887783	RFWD3	16	73258244	1	0.5301
rs4395097	LOC645726	16	73259931	3	0.667453
rs4395097	LOC645726	16	73259931	4	1.037527
rs4395097	LOC645726	16	73259931	7	0.31975
rs929881	FA2H	16	73365926	1	1.78894
rs929881	FA2H	16	73365926	5	5.774853
rs929881	FA2H	16	73365926	6	5.115095
rs929881	FA2H	16	73365926	2	2.214765
rs14308	WDR59	16	73477692	5	0.694698
rs14308	WDR59	16	73477692	3	1.744278
rs12448858	WDR59	16	73576375	4	4.325581
rs12448858	WDR59	16	73576375	5	6.127176
rs11647917	ZNRF1	16	73700743	3	1.554661
rs1043503	ZNRF1	16	73701798	2	1.283103
rs1043503	ZNRF1	16	73701798	4	0.297447
rs1043509	ZNRF1	16	73701909	3	0.383584
rs4737	CTRB2	16	73795604	1	1.015496
rs11640206	BCAR1	16	73820430	5	0.380374

rs3743613	BCAR1	16	73827035	5	5.077504
rs3743613	BCAR1	16	73827035	4	2.304072
rs4888432	CHST6	16	74068474	7	1.187899
rs4888432	CHST6	16	74068474	6	0.453797
rs4888432	CHST6	16	74068474	3	0.627178
rs8048818	CHST5	16	74121247	2	3.099811
rs8048818	CHST5	16	74121247	4	0.244179
rs3784932	CHST5	16	74121788	2	3.231773
rs3784932	CHST5	16	74121788	4	0.739372
rs2641806	CHST5	16	74122921	5	0.001767
SNP16-7420030	ADAT1	16	74200302	2	5.175818
SNP16-7420418	ADAT1	16	74204186	1	3.753204
SNP16-7420418	ADAT1	16	74204186	6	0.34958
SNP16-7420418	ADAT1	16	74204186	5	0.870141
rs12599021	CNTNAP4	16	75150134	5	2.765014
rs2278045	MON1B	16	75782364	7	0.410598
rs2278045	MON1B	16	75782364	5	0.931747
rs1137647	ADAMTS18	16	75874335	5	0.15343
rs889705	ADAMTS18	16	75874898	3	0.372446
rs889705	ADAMTS18	16	75874898	2	0.765656
rs889707	ADAMTS18	16	75875257	2	1.245325
rs3743749	ADAMTS18	16	75880736	2	0.961016
rs3743749	ADAMTS18	16	75880736	3	1.037682
rs12935229	ADAMTS18	16	75886396	5	1.42713
rs9930984	ADAMTS18	16	75911474	1	1.840081
rs8059275	ADAMTS18	16	75947457	3	0.629482
rs11643211	ADAMTS18	16	75959046	1	0.420607
rs308925	NULL	16	76327335	8	4.553426
rs308925	NULL	16	76327335	5	1.295649
rs1133110	NULL	16	76333560	6	3.82515
rs1133110	NULL	16	76333560	5	0.36787
rs1133110	NULL	16	76333560	4	2.227653
rs1133110	NULL	16	76333560	3	0.336203
rs16946927	KIAA1576	16	76570887	3	0.271913
rs2072665	CLEC3A	16	76622989	6	3.372367
rs11545028	WWOX	16	76691172	6	0.949268
SNP16-7687002	WWOX	16	76870028	4	2.452095
rs7192635	LOC645957	16	77415860	7	1.602556
rs12325279	LOC645957	16	77416906	4	0.065472
rs12325279	LOC645957	16	77416906	6	0.086952
rs12930924	LOC645957	16	77417091	3	0.327821
rs12930924	LOC645957	16	77417091	8	0.614389
rs12930924	LOC645957	16	77417091	4	0.217503
SNP16-7780328	WWOX	16	77803287	4	7.715004
rs4575545	LOC440389	16	78312947	8	4.607923
rs11866734	DYNLRB2	16	79140998	1	2.536595
rs8049284	CDYL2	16	79276380	8	0.28933

rs8049284	CDYL2	16	79276380	3	1.032852
rs8049284	CDYL2	16	79276380	5	1.053807
rs1469122	C16orf61	16	79567574	1	0.057937
rs1469122	C16orf61	16	79567574	2	7.104007
rs2287991	CENPN	16	79598056	1	3.660485
rs16954513	ATMIN	16	79634705	1	4.834084
rs1869348	PKD1L2	16	79699758	4	3.469292
rs16954698	PKD1L2	16	79714854	2	5.78033
rs16954698	PKD1L2	16	79714854	4	0.479593
SNP16-7971907	PKD1L2	16	79719079	1	4.481004
SNP16-7971907	PKD1L2	16	79719079	8	5.239039
rs4889244	PKD1L2	16	79730694	3	1.258457
rs4889244	PKD1L2	16	79730694	7	0.570519
rs4889244	PKD1L2	16	79730694	4	1.062811
rs4889244	PKD1L2	16	79730694	5	0.724361
rs4889244	PKD1L2	16	79730694	6	1.407974
rs35941327	PKD1L2	16	79732604	3	0.518956
SNP16-7973856	PKD1L2	16	79738567	1	2.251145
SNP16-7973856	PKD1L2	16	79738567	3	1.742033
SNP16-7973856	PKD1L2	16	79738567	4	0.260049
SNP16-7973856	PKD1L2	16	79738567	5	1.733826
rs9921748	PKD1L2	16	79739284	5	2.355006
rs9921748	PKD1L2	16	79739284	1	1.466036
rs9921748	PKD1L2	16	79739284	4	0.220708
rs9921748	PKD1L2	16	79739284	3	1.394803
SNP16-7974506	PKD1L2	16	79745069	7	1.753792
SNP16-7974513	PKD1L2	16	79745136	7	1.531406
SNP16-7974521	PKD1L2	16	79745210	7	0.10101
rs1453324	PKD1L2	16	79748114	5	0.447668
rs1453324	PKD1L2	16	79748114	1	3.281961
rs1453324	PKD1L2	16	79748114	3	1.036007
rs1453324	PKD1L2	16	79748114	4	3.43956
SNP16-7975082	PKD1L2	16	79750822	4	2.172369
SNP16-7975082	PKD1L2	16	79750822	8	0.255473
SNP16-7975082	PKD1L2	16	79750822	1	3.944635
SNP16-7975082	PKD1L2	16	79750822	5	2.009832
rs2317117	PKD1L2	16	79751883	1	3.979951
rs10400943	PKD1L2	16	79755807	1	4.392961
rs12931217	PKD1L2	16	79757021	4	1.365441
rs12931217	PKD1L2	16	79757021	5	1.628017
rs12925771	PKD1L2	16	79757045	3	0.063023
rs12925771	PKD1L2	16	79757045	7	0.275843
rs12925771	PKD1L2	16	79757045	1	1.518264
rs12925771	PKD1L2	16	79757045	4	2.399263
rs12925771	PKD1L2	16	79757045	6	1.242494
rs12925771	PKD1L2	16	79757045	2	2.244486
rs12926898	PKD1L2	16	79757055	1	1.799392

rs12926898	PKD1L2	16	79757055	7	0.057539
rs12926898	PKD1L2	16	79757055	2	2.73784
rs12926898	PKD1L2	16	79757055	3	0.8918
rs12926898	PKD1L2	16	79757055	4	1.852611
rs12596941	PKD1L2	16	79766016	3	0.498417
rs1869349	PKD1L2	16	79766748	4	2.055337
rs9935113	PKD1L2	16	79768997	3	0.404629
rs9935113	PKD1L2	16	79768997	7	2.42756
rs9935113	PKD1L2	16	79768997	4	0.887339
rs6564838	PKD1L2	16	79799695	3	3.161331
rs6564838	PKD1L2	16	79799695	7	2.63878
SNP16-7981126(	PKD1L2	16	79811260	1	0.553897
rs7501331	BCMO1	16	79871997	3	0.353983
rs7501331	BCMO1	16	79871997	6	1.955347
rs7501331	BCMO1	16	79871997	5	1.45263
rs7501331	BCMO1	16	79871997	2	0.23308
rs7501331	BCMO1	16	79871997	1	1.005406
rs1816122	GAN	16	79969174	2	4.971545
rs1816122	GAN	16	79969174	7	0.200284
rs1816122	GAN	16	79969174	4	0.641083
rs4586425	PLCG2	16	80377269	6	1.086021
rs4586425	PLCG2	16	80377269	7	1.275729
rs13333716	PLCG2	16	80487028	2	6.782697
rs4445895	HSD17B2	16	80626398	1	2.838981
rs4445895	HSD17B2	16	80626398	4	2.214256
rs4445895	HSD17B2	16	80626398	3	1.764665
rs1056690	MPHOSPH6	16	80739299	1	0.170337
rs1056690	MPHOSPH6	16	80739299	2	1.749263
rs1056690	MPHOSPH6	16	80739299	5	0.130285
SNP16-8076125‹	MPHOSPH6	16	80761259	7	0.848008
rs1048612	CDH13	16	82374520	3	0.506121
rs1048612	CDH13	16	82374520	1	3.705837
rs3743621	CDH13	16	82386630	2	5.151246
rs3743621	CDH13	16	82386630	3	0.57286
rs925331	NECAB2	16	82569605	5	0.244229
rs8044745	NECAB2	16	82593795	8	4.416577
rs8044745	NECAB2	16	82593795	7	0.473727
rs3881	NECAB2	16	82593826	4	0.740955
rs8045237	NECAB2	16	82593858	7	1.01269
rs1058444	HSDL1	16	82713781	5	0.021078
rs1058444	HSDL1	16	82713781	3	0.135907
rs1058444	HSDL1	16	82713781	2	5.603638
rs3199061	HSDL1	16	82714502	5	0.615322
rs3199061	HSDL1	16	82714502	2	5.758617
rs3199061	HSDL1	16	82714502	4	1.123969
rs3199061	HSDL1	16	82714502	3	0.068786
rs11644164	LRRC50	16	82761440	7	1.354909

rs11644164	LRRC50	16	82761440	1	0.948206
rs11644164	LRRC50	16	82761440	5	0.589937
rs2288019	LRRC50	16	82767135	5	0.509604
rs2288019	LRRC50	16	82767135	4	0.095578
rs2288019	LRRC50	16	82767135	7	0.317098
rs2288019	LRRC50	16	82767135	1	0.733424
rs2288020	LRRC50	16	82767239	4	2.743892
rs2288020	LRRC50	16	82767239	5	1.63472
rs2288020	LRRC50	16	82767239	7	2.883068
SNP16-82767317	LRRC50	16	82767317	6	1.438617
SNP16-82767317	LRRC50	16	82767317	2	0.534791
SNP16-82767317	LRRC50	16	82767317	7	1.046861
SNP16-82767317	LRRC50	16	82767317	5	0.683187
SNP16-82767317	LRRC50	16	82767317	4	0.854276
rs2288023	LRRC50	16	82767365	5	0.197713
rs2288023	LRRC50	16	82767365	4	2.574371
rs2288023	LRRC50	16	82767365	7	1.753734
rs2288023	LRRC50	16	82767365	6	1.039371
rs2288024	TAF1C	16	82768966	3	0.92041
rs8692	TAF1C	16	82768984	7	1.006367
rs8692	TAF1C	16	82768984	5	0.973578
rs1056616	TAF1C	16	82769219	1	4.283073
rs4150176	TAF1C	16	82770211	1	5.296231
rs3743642	TAF1C	16	82770429	1	4.298835
rs2230129	TAF1C	16	82770935	2	1.118785
rs2230129	TAF1C	16	82770935	1	0.156834
rs2230129	TAF1C	16	82770935	5	0.275331
rs2230129	TAF1C	16	82770935	7	0.143994
rs2230129	TAF1C	16	82770935	4	0.105894
rs8044351	ADAD2	16	82782306	4	0.332077
rs8044351	ADAD2	16	82782306	7	0.964281
rs11646787	LOC654780	16	82785738	1	0.058052
rs11646787	LOC654780	16	82785738	3	0.152752
rs11646787	LOC654780	16	82785738	8	3.770601
rs11149631	ADAD2	16	82786271	8	1.496228
rs11865115	ADAD2	16	82786374	1	0.47596
rs11865115	ADAD2	16	82786374	8	0.27918
rs11865115	ADAD2	16	82786374	3	2.532094
rs2303238	ADAD2	16	82787060	3	0.308198
rs2303239	ADAD2	16	82787081	5	0.000783
rs2303243	LOC654780	16	82788001	1	1.408127
rs2303243	LOC654780	16	82788001	5	0.955108
rs2303243	LOC654780	16	82788001	3	1.545785
rs244811	KCNG4	16	82827882	7	0.160966
rs244811	KCNG4	16	82827882	3	0.249568
rs12933084	WFDC1	16	82918034	5	1.599913
rs12933084	WFDC1	16	82918034	4	2.350497

rs12933084	WFDC1	16	82918034	7	1.136945
rs2303853	ATP2C2	16	83031985	2	4.15237
rs2241640	ATP2C2	16	83051776	2	2.528776
rs2241640	ATP2C2	16	83051776	7	0.185407
rs2241640	ATP2C2	16	83051776	6	0.679624
rs2241640	ATP2C2	16	83051776	1	0.139659
rs2241633	ATP2C2	16	83055081	7	7.03028
rs2241633	ATP2C2	16	83055081	3	0.083174
rs3826150	KIAA1609	16	83068966	7	0.658182
rs3826150	KIAA1609	16	83068966	5	4.77048
rs3826150	KIAA1609	16	83068966	1	3.657559
rs3826150	KIAA1609	16	83068966	3	0.009867
rs433293	KIAA1609	16	83073810	8	0.284089
rs433293	KIAA1609	16	83073810	1	2.730268
rs433293	KIAA1609	16	83073810	4	0.560457
rs433293	KIAA1609	16	83073810	7	1.057639
rs3743667	KIAA1609	16	83077793	1	5.385835
rs436278	KIAA1609	16	83080398	4	0.946762
rs436278	KIAA1609	16	83080398	7	0.565926
rs1047121	COTL1	16	83156905	4	0.555891
rs1047121	COTL1	16	83156905	3	0.728645
rs1047121	COTL1	16	83156905	8	0.803363
rs1047121	COTL1	16	83156905	5	0.369906
rs1047121	COTL1	16	83156905	2	7.989465
rs7458	COTL1	16	83157031	1	3.507356
rs13521	COTL1	16	83157471	8	8.944488
rs13521	COTL1	16	83157471	5	6.367229
rs13521	COTL1	16	83157471	7	0.168382
rs12932018	USP10	16	83354104	7	0.76422
rs12932018	USP10	16	83354104	1	4.562956
rs2172623	CRISPLD2	16	83411123	8	0.260435
rs2172623	CRISPLD2	16	83411123	1	1.971838
SNP16-83436965	CRISPLD2	16	83436965	7	0.596573
SNP16-83436965	CRISPLD2	16	83436965	4	1.063745
SNP16-83436965	CRISPLD2	16	83436965	6	0.126006
SNP16-83436965	CRISPLD2	16	83436965	2	3.789832
rs8061351	CRISPLD2	16	83440603	5	0.701699
SNP16-83459984	CRISPLD2	16	83459984	7	0.153555
SNP16-83459984	CRISPLD2	16	83459984	8	0.473981
rs721005	CRISPLD2	16	83463599	1	0.586113
rs767050	CRISPLD2	16	83480360	3	0.988821
rs1874008	CRISPLD2	16	83497980	3	0.524883
rs1874008	CRISPLD2	16	83497980	5	3.78281
rs3803632	CRISPLD2	16	83498026	4	0.357547
rs3803632	CRISPLD2	16	83498026	6	0.509439
rs4783099	CRISPLD2	16	83498830	3	1.354294
rs7195377	ZDHHC7	16	83567471	8	6.01939

rs7195377	ZDHHC7	16	83567471	4	0.12693
rs7195377	ZDHHC7	16	83567471	6	1.926032
rs7195377	ZDHHC7	16	83567471	3	0.064412
rs7195377	ZDHHC7	16	83567471	1	3.385982
rs16975086	ZDHHC7	16	83573042	3	0.131599
rs16975086	ZDHHC7	16	83573042	8	7.376009
rs16975086	ZDHHC7	16	83573042	1	4.41005
rs7186182	KIAA0513	16	83681783	2	3.402351
rs16975222	KIAA0513	16	83683961	4	0.281822
rs16975222	KIAA0513	16	83683961	5	0.565423
rs16975222	KIAA0513	16	83683961	1	0.403843
rs16975222	KIAA0513	16	83683961	2	0.058147
rs715707	KIAA0513	16	83684973	2	7.135839
rs4565	GINS2	16	84268912	4	0.527113
rs4565	GINS2	16	84268912	5	0.121447
rs4565	GINS2	16	84268912	3	0.001197
rs8587	COX4NB	16	84370732	1	4.100687
rs8587	COX4NB	16	84370732	8	0.687862
GA031728	COX4I1	16	84392319	8	5.078588
rs17444416	IRF8	16	84505599	1	1.781357
rs17444416	IRF8	16	84505599	6	1.325122
rs7204236	LOC283904	16	85079989	1	0.698263
rs43140	LOC283904	16	85081584	1	1.165123
rs1364225	LOC283904	16	85100527	3	0.292668
rs11860845	FOXF1	16	85104997	5	1.053539
rs11860845	FOXF1	16	85104997	3	0.632552
rs16964183	LOC401865	16	85117143	2	0.078195
rs16964183	LOC401865	16	85117143	1	0.595306
rs16944719	MTHFSD	16	85122688	1	4.091643
rs16944719	MTHFSD	16	85122688	3	1.286409
rs34005514	MTHFSD	16	85143244	1	6.21905
rs34005514	MTHFSD	16	85143244	3	0.167891
rs387148	LOC729979	16	85325973	7	1.073417
rs387148	LOC729979	16	85325973	2	6.213938
rs2288482	FBXO31	16	85922164	5	0.861005
rs2288482	FBXO31	16	85922164	4	0.305507
rs9903	MAP1LC3B	16	85994608	3	0.458084
rs918368	JPH3	16	86235645	3	0.36682
rs17699859	KLHDC4	16	86346337	4	0.380112
rs2303771	KLHDC4	16	86346365	8	5.035156
rs2303771	KLHDC4	16	86346365	7	1.07494
rs2303771	KLHDC4	16	86346365	4	1.748595
rs2303772	KLHDC4	16	86353081	4	0.112477
SNP16-87126524	ZFPM1	16	87126524	4	1.713149
SNP16-87126524	ZFPM1	16	87126524	7	0.553279
rs2879897	C16orf85	16	87147696	3	1.277762
rs8057773	C16orf85	16	87160848	1	3.186863

rs8057773	C16orf85	16	87160848	4	1.03571
rs6500476	C16orf85	16	87160855	7	0.090976
rs6500476	C16orf85	16	87160855	3	0.449037
rs4782451	C16orf85	16	87161479	4	1.088167
rs4782451	C16orf85	16	87161479	3	0.505571
rs4782451	C16orf85	16	87161479	7	0.683024
rs4782451	C16orf85	16	87161479	6	1.516218
rs11646848	C16orf85	16	87162811	4	0.008841
rs11646848	C16orf85	16	87162811	3	0.585315
rs12599209	C16orf85	16	87163569	4	1.188906
rs12599209	C16orf85	16	87163569	3	1.260055
rs899728	ZC3H18	16	87171506	3	0.268243
rs899728	ZC3H18	16	87171506	1	4.046452
rs899728	ZC3H18	16	87171506	7	0.173782
rs2306049	RNF166	16	87295270	5	0.015717
rs2306049	RNF166	16	87295270	2	3.771344
rs2306049	RNF166	16	87295270	8	0.445467
rs2306049	RNF166	16	87295270	6	0.679674
rs2306049	RNF166	16	87295270	4	0.148799
rs2290896	C16orf84	16	87306533	5	0.052404
rs2290896	C16orf84	16	87306533	2	5.717807
rs6500493	FLJ45121	16	87331483	1	6.217164
rs10775348	FLJ45121	16	87333849	5	0.747444
rs10775348	FLJ45121	16	87333849	1	0.719609
rs2016979	FLJ45121	16	87334593	6	0.027588
rs2016979	FLJ45121	16	87334593	2	0.302498
rs2016979	FLJ45121	16	87334593	4	0.280194
rs2016979	FLJ45121	16	87334593	5	0.120269
rs750739	FLJ45121	16	87335109	4	0.426442
rs750739	FLJ45121	16	87335109	5	1.372262
rs9925084	FLJ40448	16	87336935	2	0.354433
rs475667	CDT1	16	87398468	7	0.939846
rs510862	CDT1	16	87400012	7	0.821985
rs510862	CDT1	16	87400012	3	0.354791
rs572275	CDT1	16	87402133	2	0.951046
rs572275	CDT1	16	87402133	3	0.89172
rs572275	CDT1	16	87402133	1	0.489878
rs572275	CDT1	16	87402133	4	0.515093
SNP16-87418741	GALNS	16	87418741	1	5.345336
SNP16-87418741	GALNS	16	87418741	7	6.031664
SNP16-87418741	GALNS	16	87418741	6	5.226037
rs3743979	ACSF3	16	87708384	7	0.281636
rs3743979	ACSF3	16	87708384	2	4.084808
GA013636	CDH15	16	87787494	1	1.032585
GA013636	CDH15	16	87787494	3	0.965745
rs9937322	C16orf55	16	88263424	2	2.270641
rs9937322	C16orf55	16	88263424	7	6.767893

rs9937322	C16orf55	16	88263424	4	0.887441
rs9937322	C16orf55	16	88263424	1	0.493697
rs17177787	C16orf55	16	88264414	5	1.263071
rs3751680	C16orf55	16	88264830	5	0.927806
rs3803690	SPATA2L	16	88290860	4	1.268739
rs3803690	SPATA2L	16	88290860	3	0.22719
rs17177891	ZNF276	16	88327451	2	3.467033
rs17232910	FANCA	16	88367267	2	3.161019
rs1800331	FANCA	16	88385918	2	0.331898
SNP16-8850510	TCF25	16	88505105	6	6.043647
SNP16-8850510	TCF25	16	88505105	7	0.182745
SNP16-8850510	TCF25	16	88505105	4	4.563032
rs3212363	MC1R	16	88512942	8	4.256418
rs3212363	MC1R	16	88512942	1	1.927919
rs3212363	MC1R	16	88512942	3	0.229637
rs1805005	MC1R	16	88513345	1	0.241987
rs2228479	MC1R	16	88513441	2	2.122691
rs1805008	MC1R	16	88513645	1	4.180152
rs885479	MC1R	16	88513655	8	0.953328
rs4408545	AFG3L1	16	88571529	1	0.663089
rs4408545	AFG3L1	16	88571529	2	0.963336
rs4408545	AFG3L1	16	88571529	7	0.913238
rs4408545	AFG3L1	16	88571529	3	0.959294
rs2241085	DBNDD1	16	88599766	6	0.407883
rs4362387	DBNDD1	16	88602727	2	5.345217
rs3785183	C16orf3	16	88623062	3	0.924398
rs3785183	C16orf3	16	88623062	7	0.568972
rs12925087	C16orf3	16	88623568	3	1.224099
rs17178299	GAS8	16	88631160	2	4.7236
SNP16-88638451	GAS8	16	88638451	8	0.421189
SNP16-88638451	GAS8	16	88638451	1	4.736389
rs8046635	PRDM7	16	88650994	1	0.264037
rs3809643	PRDM7	16	88651774	1	0.834565
rs2396789	FLJ43681	17	8547	1	0.010949
rs9907477	NULL	17	62646	1	4.97298
rs9907477	NULL	17	62646	7	0.583825
rs9896436	KRTAP2-2	17	73422	7	1.590909
rs8078929	LOC729000	17	78232	1	3.467543
rs9675043	LOC729000	17	78382	1	1.836348
rs9675043	LOC729000	17	78382	7	0.218289
rs9675043	LOC729000	17	78382	4	0.090139
rs9675043	LOC729000	17	78382	8	0.430281
rs12603796	LOC728901	17	87761	5	0.57893
rs7223759	LOC728956	17	102209	7	0.127473
rs9900685	LOC728860	17	103469	3	0.307672
rs7208188	LOC728860	17	113034	1	1.666371
rs7208188	LOC728860	17	113034	7	0.659646

rs12947571	LOC728860	17	113794	8	0.979192
rs12947571	LOC728860	17	113794	1	4.968399
rs10871511	LOC728824	17	126701	3	0.775789
rs7223270	LOC728824	17	128389	1	3.860306
rs9789059	LOC646301	17	129225	1	1.900587
rs9788983	LOC646301	17	129457	3	0.444907
rs9788983	LOC646301	17	129457	7	0.676921
rs9788983	LOC646301	17	129457	1	0.62448
rs11867532	LOC728760	17	146428	7	0.139128
rs4556857	LOC644132	17	173906	3	0.927321
rs4556857	LOC644132	17	173906	7	0.691057
rs7224337	LOC644132	17	174121	3	0.968298
rs8069166	FAM101B	17	293366	8	1.23571
rs7813	GEMIN4	17	594936	2	4.16369
rs7813	GEMIN4	17	594936	6	6.555669
rs7813	GEMIN4	17	594936	4	1.143422
rs3744741	GEMIN4	17	595982	7	0.19962
rs2740348	GEMIN4	17	596685	6	0.011302
rs2740348	GEMIN4	17	596685	1	4.901728
rs2740348	GEMIN4	17	596685	4	1.002076
rs16955542	RNMTL1	17	632356	6	0.085976
rs2273454	RNMTL1	17	632390	7	0.041763
rs540226	NXN	17	650152	2	0.82432
rs540226	NXN	17	650152	7	2.751872
rs540226	NXN	17	650152	3	0.90627
rs540226	NXN	17	650152	5	4.442876
rs540226	NXN	17	650152	1	1.969439
rs3744755	ABR	17	854979	7	4.540375
rs3744755	ABR	17	854979	1	4.430745
rs3263	ABR	17	855545	1	3.730688
SNP17-906036	ABR	17	906036	7	0.076984
rs6502774	TUSC5	17	1130104	5	1.938039
rs6502774	TUSC5	17	1130104	4	0.652977
rs6502774	TUSC5	17	1130104	8	3.56527
rs6502774	TUSC5	17	1130104	7	1.645769
rs4790289	TUSC5	17	1150431	4	0.595831
rs4790289	TUSC5	17	1150431	1	0.751345
rs4790289	TUSC5	17	1150431	3	0.414471
rs2302458	MYO1C	17	1317292	8	0.360472
SNP17-1320268	MYO1C	17	1320268	5	4.851425
SNP17-1320268	MYO1C	17	1320268	2	4.880104
SNP17-1320268	MYO1C	17	1320268	7	0.050233
SNP17-1583684	WDR81	17	1583684	6	1.875731
rs8065251	WDR81	17	1584208	5	0.352608
rs8065251	WDR81	17	1584208	4	0.687479
rs2070862	SERPINF2	17	1595044	3	0.044315
rs2070862	SERPINF2	17	1595044	5	0.746602

rs6828	SERPINF1	17	1626752	8	6.36505
rs9902398	SMYD4	17	1633160	8	5.378359
SNP17-1729702	RPA1	17	1729702	3	0.806626
rs2230930	RPA1	17	1729707	4	7.39075
rs2230930	RPA1	17	1729707	6	2.964532
rs2230930	RPA1	17	1729707	1	2.968831
rs2230930	RPA1	17	1729707	3	0.289095
rs9906592	RTN4RL1	17	1786022	8	2.845343
rs9906592	RTN4RL1	17	1786022	5	0.882163
rs2236375	DPH1-OVCA2	17	1890638	6	0.800356
rs2236375	DPH1-OVCA2	17	1890638	3	1.412637
rs1051322	DPH1-OVCA2	17	1893253	2	8.70165
rs1051322	DPH1-OVCA2	17	1893253	4	0.440543
rs1051322	DPH1-OVCA2	17	1893253	3	0.199286
rs1051322	DPH1-OVCA2	17	1893253	1	0.014375
rs1051322	DPH1-OVCA2	17	1893253	8	0.195367
rs1051322	DPH1-OVCA2	17	1893253	5	0.484711
rs903157	SMG6	17	1911147	3	0.611681
rs903157	SMG6	17	1911147	6	9.222008
rs1885986	SMG6	17	2149925	2	0.642375
rs1885986	SMG6	17	2149925	3	1.047612
rs1885986	SMG6	17	2149925	8	0.364661
rs1885986	SMG6	17	2149925	1	0.002039
SNP17-2174405	SRR	17	2174405	3	0.477141
rs4613098	LOC284009	17	2265300	5	0.044623
rs4613098	LOC284009	17	2265300	3	0.387983
rs4613098	LOC284009	17	2265300	6	2.340182
rs4613098	LOC284009	17	2265300	4	0.332143
rs4613098	LOC284009	17	2265300	7	0.629803
rs4790335	LOC284009	17	2265445	5	0.247893
rs4790335	LOC284009	17	2265445	4	3.025226
rs4790335	LOC284009	17	2265445	7	0.603747
rs4790335	LOC284009	17	2265445	1	3.210924
rs4790115	GARNL4	17	2877089	5	5.312607
rs4790115	GARNL4	17	2877089	8	4.190708
rs4790115	GARNL4	17	2877089	7	0.520039
rs4790115	GARNL4	17	2877089	1	4.550014
rs4790115	GARNL4	17	2877089	3	0.719396
rs4264423	GARNL4	17	2886332	1	4.8036
rs4264423	GARNL4	17	2886332	6	0.189747
rs4264423	GARNL4	17	2886332	3	0.708151
rs769426	OR1A1	17	3066423	5	0.909471
rs769427	OR1A1	17	3066517	1	1.088397
rs2013809	OR3A2	17	3128305	5	1.960358
rs9903671	OR3A4	17	3160661	7	0.10502
rs231678	OR3A4	17	3160931	8	1.920532
rs11556563	SPATA22	17	3299059	8	3.55756

rs11556563	SPATA22	17	3299059	7	4.209235
rs11556563	SPATA22	17	3299059	5	5.114294
rs12948217	ASPA	17	3344452	8	1.181793
rs12948217	ASPA	17	3344452	2	0.866337
rs12948217	ASPA	17	3344452	1	0.272907
rs7208811	TRPV3	17	3363305	7	0.338305
rs7208811	TRPV3	17	3363305	5	3.473275
rs7208811	TRPV3	17	3363305	8	0.303122
rs395357	TRPV3	17	3382830	8	0.827388
rs395357	TRPV3	17	3382830	6	1.383153
rs395357	TRPV3	17	3382830	5	0.686713
rs395357	TRPV3	17	3382830	3	3.351912
rs11078458	TRPV3	17	3392651	1	3.41602
rs11078458	TRPV3	17	3392651	3	4.405721
rs11078458	TRPV3	17	3392651	6	4.498836
rs11078458	TRPV3	17	3392651	5	0.415318
SNP17-3393635	TRPV3	17	3393635	1	2.692931
SNP17-3393635	TRPV3	17	3393635	2	1.045931
SNP17-3393635	TRPV3	17	3393635	5	0.07745
SNP17-3393635	TRPV3	17	3393635	6	1.211413
rs1039519	TRPV3	17	3394664	5	0.688507
rs1039519	TRPV3	17	3394664	3	2.743533
rs1039519	TRPV3	17	3394664	1	1.615789
rs1039519	TRPV3	17	3394664	2	1.926236
rs1039519	TRPV3	17	3394664	6	0.64369
rs4790522	TRPV1	17	3416603	3	0.161856
rs4790522	TRPV1	17	3416603	1	0.73453
rs222747	TRPV1	17	3439949	4	1.503413
rs222747	TRPV1	17	3439949	6	4.755636
rs222747	TRPV1	17	3439949	5	0.605054
rs222749	TRPV1	17	3442123	1	4.16479
rs222749	TRPV1	17	3442123	7	5.494768
rs460716	TRPV1	17	3458557	5	4.309601
rs460716	TRPV1	17	3458557	7	0.407017
rs150857	CARKL	17	3473386	7	0.785401
rs17707869	CTNS	17	3486791	7	5.015667
rs1048682	CTNS	17	3511528	1	5.322126
rs160589	TAX1BP3	17	3518612	3	0.373758
rs3744679	ITGAE	17	3582489	3	0.406872
rs9135	CAMKK1	17	3710682	7	4.612816
rs2532947	CAMKK1	17	3715428	3	1.290769
rs7214864	CAMKK1	17	3722665	4	1.201355
rs917605	ATP2A3	17	3774251	7	0.537506
rs887387	ATP2A3	17	3775451	2	2.691376
rs3179783	ATP2A3	17	3787677	3	0.516647
rs3179783	ATP2A3	17	3787677	8	1.603097
rs3179783	ATP2A3	17	3787677	5	0.330616

rs1052617	ZZEF1	17	3856153	6	5.656858
rs1052617	ZZEF1	17	3856153	2	4.151622
rs1052617	ZZEF1	17	3856153	5	0.401896
rs1052617	ZZEF1	17	3856153	3	0.416321
rs1052617	ZZEF1	17	3856153	7	0.151622
rs8075562	ZZEF1	17	3863572	2	3.202737
rs8075562	ZZEF1	17	3863572	3	0.380369
rs1006954	ZZEF1	17	3894282	1	2.917288
rs1043592	ANKFY1	17	4014064	2	6.159711
rs1043592	ANKFY1	17	4014064	3	0.099862
rs1043592	ANKFY1	17	4014064	5	0.490489
rs1043587	ANKFY1	17	4014301	8	10.20184
rs11655342	SPNS3	17	4303124	8	0.016256
rs11655342	SPNS3	17	4303124	6	0.992379
rs11655342	SPNS3	17	4303124	5	1.756478
rs11655342	SPNS3	17	4303124	1	1.736763
rs333121	SPNS3	17	4338179	7	3.061323
rs333121	SPNS3	17	4338179	8	2.094297
rs333121	SPNS3	17	4338179	1	2.584515
rs3809849	MYBBP1A	17	4405347	1	0.753027
rs3809849	MYBBP1A	17	4405347	6	3.115903
rs3809849	MYBBP1A	17	4405347	2	0.489853
rs7216284	GGT6	17	4410448	6	1.646307
rs7216284	GGT6	17	4410448	1	4.12606
rs7216474	GGT6	17	4410545	2	1.859186
rs7216474	GGT6	17	4410545	6	2.80634
rs7216474	GGT6	17	4410545	1	1.7018
rs4790654	SMTNL2	17	4442431	5	0.269639
rs12103697	SMTNL2	17	4445360	1	1.43419
rs12103697	SMTNL2	17	4445360	5	0.41583
rs916055	ALOX15	17	4481583	3	0.544819
rs743646	ALOX15	17	4482990	5	0.657614
rs743646	ALOX15	17	4482990	3	0.385191
rs8081507	PELP1	17	4524699	1	3.293513
rs9895782	LOC728244	17	4555207	1	3.766729
rs3826517	ZMYND15	17	4592073	3	1.932047
rs3826517	ZMYND15	17	4592073	2	4.7991
rs3826517	ZMYND15	17	4592073	8	1.224432
rs3826517	ZMYND15	17	4592073	5	5.761061
rs3826517	ZMYND15	17	4592073	6	2.413108
rs2279961	VMO1	17	4636053	1	4.671741
rs4790706	VMO1	17	4636312	2	4.265812
rs4790706	VMO1	17	4636312	3	1.424759
rs4790706	VMO1	17	4636312	5	0.701112
rs4790706	VMO1	17	4636312	8	0.3825
rs12951761	GLTPD2	17	4639794	3	1.276023
rs12951761	GLTPD2	17	4639794	2	3.992582

rs12951761	GLTPD2	17	4639794	5	0.267974
GA031922	PSMB6	17	4647046	6	0.278036
rs7211943	PLD2	17	4659359	7	6.928855
rs11556635	MINK1	17	4737062	6	1.673606
rs11556635	MINK1	17	4737062	2	0.039284
rs11556635	MINK1	17	4737062	5	0.66217
rs33970119	CHRNE	17	4745681	8	0.669315
rs33970119	CHRNE	17	4745681	4	1.049233
rs33970119	CHRNE	17	4745681	5	0.564067
rs6065	GP1BA	17	4777161	3	0.156474
rs238234	CAMTA2	17	4824542	4	3.870743
rs238234	CAMTA2	17	4824542	5	0.790059
SNP17-4832900	INCA1	17	4832900	1	0.131263
SNP17-4832900	INCA1	17	4832900	3	0.082258
SNP17-4832900	INCA1	17	4832900	2	0.27194
rs2304445	GPR172B	17	4877622	4	1.831406
rs2304445	GPR172B	17	4877622	5	1.969235
rs3744704	GPR172B	17	4878925	3	0.486146
rs3744705	ZFP3	17	4937694	1	3.554057
rs3744705	ZFP3	17	4937694	3	0.537653
rs3744706	ZFP3	17	4937763	4	0.910016
rs3744706	ZFP3	17	4937763	5	0.002319
rs12600437	ZFP3	17	4938157	5	0.955068
rs12600437	ZFP3	17	4938157	4	0.519865
rs3744708	ZFP3	17	4939576	1	4.851391
rs3744708	ZFP3	17	4939576	3	7.573606
rs3890287	USP6	17	4972892	3	1.183154
SNP17-4983439	USP6	17	4983439	1	4.681804
SNP17-4983439	USP6	17	4983439	3	3.212642
rs11658877	USP6	17	5013040	6	5.003775
rs11658877	USP6	17	5013040	5	6.587946
rs11658877	USP6	17	5013040	3	4.691195
rs11658877	USP6	17	5013040	8	1.334553
rs11658877	USP6	17	5013040	4	5.39679
rs11658877	USP6	17	5013040	2	5.760955
rs9908414	ZNF594	17	5027764	5	5.04009
rs9908414	ZNF594	17	5027764	3	0.645069
rs9908414	ZNF594	17	5027764	8	0.954078
rs2641256	C17orf87	17	5067398	1	5.294673
rs2641256	C17orf87	17	5067398	3	0.182148
rs2585271	C17orf87	17	5067413	8	4.819813
rs2585271	C17orf87	17	5067413	3	1.188079
SNP17-5212487	RABEP1	17	5212487	1	3.832526
rs10792	RABEP1	17	5228903	3	0.650894
rs10792	RABEP1	17	5228903	1	2.040195
rs10792	RABEP1	17	5228903	8	0.737368
rs1058400	RABEP1	17	5229518	5	0.005945

rs1058400	RABEP1	17	5229518	8	7.005245
rs1058400	RABEP1	17	5229518	3	0.021313
rs1058400	RABEP1	17	5229518	2	4.882268
rs2301739	NUP88	17	5249101	3	0.741742
rs2301739	NUP88	17	5249101	1	5.266774
rs1058117	RPAIN	17	5263930	2	0.495731
rs1058117	RPAIN	17	5263930	3	3.173266
rs1058117	RPAIN	17	5263930	6	2.542087
rs1058117	RPAIN	17	5263930	5	7.781685
rs1806269	RPAIN	17	5263952	5	2.579197
rs2189335	RPAIN	17	5266869	2	0.87805
rs2189335	RPAIN	17	5266869	1	0.720209
rs2189335	RPAIN	17	5266869	8	7.076888
rs2189335	RPAIN	17	5266869	3	0.874986
rs11653658	DHX33	17	5294928	6	4.735175
rs16954697	DHX33	17	5297637	6	3.75249
rs16954697	DHX33	17	5297637	1	6.22114
rs8069315	DHX33	17	5307671	5	4.670467
rs2309399	MIS12	17	5332414	8	0.292703
rs2309399	MIS12	17	5332414	5	0.090967
rs2309399	MIS12	17	5332414	3	0.798672
rs872569	LOC728392	17	5344986	5	0.592926
rs11653832	NLRP1	17	5365630	4	4.846325
rs11653580	NLRP1	17	5365715	4	5.16901
rs11653580	NLRP1	17	5365715	6	0.366211
rs11653580	NLRP1	17	5365715	2	5.991268
rs11651270	NLRP1	17	5365801	2	2.864865
rs11651270	NLRP1	17	5365801	4	0.078433
rs11651270	NLRP1	17	5365801	7	1.200503
rs11651270	NLRP1	17	5365801	3	4.317286
rs11651270	NLRP1	17	5365801	5	2.314794
rs11651270	NLRP1	17	5365801	8	3.550599
rs34733791	NLRP1	17	5378009	6	1.059344
rs11654427	NLRP1	17	5378020	3	3.253006
rs11657747	NLRP1	17	5385967	4	1.894819
rs11657747	NLRP1	17	5385967	6	6.366241
rs11657747	NLRP1	17	5385967	2	0.667788
rs12942931	NLRP1	17	5386038	4	1.711742
SNP17-5402395	NLRP1	17	5402395	6	5.642582
rs2001363	NLRP1	17	5403378	4	2.012209
rs11651595	NLRP1	17	5404003	4	1.785828
rs11651595	NLRP1	17	5404003	2	3.822973
rs11651595	NLRP1	17	5404003	6	0.319341
rs884367	NLRP1	17	5427888	8	6.813885
rs884367	NLRP1	17	5427888	4	0.549879
rs884367	NLRP1	17	5427888	3	1.50914
rs2302836	WSCD1	17	5932068	6	0.556366

rs11868551	WSCD1	17	5966196	1	2.681601
rs3826521	WSCD1	17	5966243	2	0.73741
rs3826521	WSCD1	17	5966243	1	0.101189
rs3744725	WSCD1	17	5966604	7	0.791883
rs3744725	WSCD1	17	5966604	5	0.795661
rs3826520	WSCD1	17	5967291	3	0.389138
rs28493751	PITPNM3	17	6382100	1	5.432992
rs4796519	KIAA0753	17	6452505	6	0.174573
rs4796519	KIAA0753	17	6452505	5	0.992554
rs2304977	KIAA0753	17	6454053	7	1.40652
rs2304977	KIAA0753	17	6454053	5	0.018152
SNP17-6456174	KIAA0753	17	6456174	1	4.406753
rs9889363	KIAA0753	17	6465022	6	4.668777
rs9889363	KIAA0753	17	6465022	5	0.362333
rs9889363	KIAA0753	17	6465022	7	0.546247
rs2072149	KIAA0753	17	6472372	5	0.434498
rs2301873	KIAA0753	17	6479079	5	0.111126
rs2301873	KIAA0753	17	6479079	6	0.692804
rs4796532	hCG_1985469	17	6497050	1	3.80512
rs218689	SLC13A5	17	6530074	2	3.817538
rs218689	SLC13A5	17	6530074	3	0.012151
rs218689	SLC13A5	17	6530074	4	0.920956
SNP17-6538204	SLC13A5	17	6538204	3	0.318895
SNP17-6538204	SLC13A5	17	6538204	4	2.155122
SNP17-6539819	SLC13A5	17	6539819	4	0.067815
SNP17-6539819	SLC13A5	17	6539819	3	0.527187
rs4796555	FBXO39	17	6623939	2	0.362149
rs4796555	FBXO39	17	6623939	3	0.527012
rs4796555	FBXO39	17	6623939	1	0.580726
SNP17-6630888	FBXO39	17	6630888	5	0.121975
SNP17-6630888	FBXO39	17	6630888	2	1.650504
SNP17-6630888	FBXO39	17	6630888	6	0.982635
rs17804647	TEKT1	17	6644795	5	1.236089
rs3744395	TEKT1	17	6656966	3	0.811295
rs11571326	LOC728478	17	6839621	2	2.695402
rs312466	LOC728478	17	6839945	5	1.097535
rs312466	LOC728478	17	6839945	1	2.373548
rs312466	LOC728478	17	6839945	6	1.264554
rs312466	LOC728478	17	6839945	4	0.057348
rs1042356	ALOX12	17	6843467	6	3.214092
rs1042356	ALOX12	17	6843467	8	0.776125
rs1042356	ALOX12	17	6843467	5	0.069041
rs1042356	ALOX12	17	6843467	4	4.825364
rs1042356	ALOX12	17	6843467	1	4.619121
rs1126667	ALOX12	17	6843484	8	1.495944
rs1126667	ALOX12	17	6843484	5	2.083181
rs1126667	ALOX12	17	6843484	1	6.116346

rs1126667	ALOX12	17	6843484	4	1.986702
rs1126667	ALOX12	17	6843484	6	1.544036
rs434473	ALOX12	17	6845658	1	3.817493
rs434473	ALOX12	17	6845658	5	0.727339
rs434473	ALOX12	17	6845658	4	7.840412
rs434473	ALOX12	17	6845658	6	0.066515
rs2307214	ALOX12	17	6850562	2	3.490302
SNP17-6858574	RNASEK	17	6858574	2	1.479992
rs14309	C17orf49	17	6859817	2	3.36847
rs11653054	ASGR2	17	6945651	1	2.829439
rs12453853	LOC728486	17	6985470	6	1.434248
rs12453853	LOC728486	17	6985470	2	3.808771
rs12453853	LOC728486	17	6985470	3	0.432821
rs17203281	DLG4	17	7040535	4	1.474199
rs2017365	DLG4	17	7063348	7	0.545909
rs222836	DVL2	17	7073886	4	0.751735
rs222836	DVL2	17	7073886	7	5.597602
rs222836	DVL2	17	7073886	8	0.117916
rs8082645	SLC2A4	17	7131159	1	0.950391
rs11078672	EIF5A	17	7155866	6	6.422132
rs11078672	EIF5A	17	7155866	5	2.951968
rs11078672	EIF5A	17	7155866	3	1.260196
rs2270981	GPS2	17	7157264	1	3.526497
SNP17-7165645	KIAA1787	17	7165645	3	0.487913
SNP17-7171569	KIAA1787	17	7171569	7	0.148549
rs4796407	CENTB1	17	7186095	1	0.010307
rs4796407	CENTB1	17	7186095	3	1.734584
rs3809828	CENTB1	17	7187575	7	1.177764
rs35985803	CENTB1	17	7195039	7	0.188595
rs1554947	TNK1	17	7226990	8	0.298135
rs1554947	TNK1	17	7226990	4	3.416161
rs2075759	TNK1	17	7233409	6	5.603767
rs4613118	PLSCR3	17	7238176	6	1.719412
rs4613118	PLSCR3	17	7238176	3	0.448385
GA013897	NLGN2	17	7260049	2	8.583724
GA013901	NLGN2	17	7263185	5	5.528653
GA013901	NLGN2	17	7263185	2	7.755016
GA013901	NLGN2	17	7263185	1	0.776709
rs13290	C17orf74	17	7270356	1	0.334864
rs17856697	CHRNB1	17	7289349	7	0.022716
rs2228129	POLR2A	17	7343324	1	3.928391
rs2228129	POLR2A	17	7343324	5	0.594329
rs2228132	POLR2A	17	7352494	1	3.548093
rs2228132	POLR2A	17	7352494	5	7.01151
rs1128963	TNFSF12	17	7401409	2	0.207392
rs1128963	TNFSF12	17	7401409	4	0.242254
rs1128963	TNFSF12	17	7401409	6	0.200881

rs1128963	TNFSF12	17	7401409	1	0.46282
rs1128963	TNFSF12	17	7401409	3	0.283647
rs9901673	CD68	17	7424825	5	0.914181
rs17607	CD68	17	7425506	2	4.740665
rs17607	CD68	17	7425506	3	0.396991
rs858520	SAT2	17	7470996	6	0.097885
rs858520	SAT2	17	7470996	3	0.478996
rs858520	SAT2	17	7470996	1	0.473356
rs858520	SAT2	17	7470996	4	4.557399
rs858520	SAT2	17	7470996	2	5.772322
rs1642762	ATP1B2	17	7495497	3	0.176999
rs1642763	ATP1B2	17	7498144	1	4.878292
rs1642763	ATP1B2	17	7498144	5	0.661724
rs1642763	ATP1B2	17	7498144	6	0.475681
rs1641511	ATP1B2	17	7500402	5	0.329697
rs1641511	ATP1B2	17	7500402	1	2.916023
rs1641511	ATP1B2	17	7500402	6	1.905931
rs1050533	ATP1B2	17	7501019	1	0.813891
rs1050541	ATP1B2	17	7501560	4	3.437815
rs1050541	ATP1B2	17	7501560	1	4.967848
rs2287499	WDR79	17	7532893	3	2.930518
rs2287498	WDR79	17	7533285	3	0.747329
SNP17-7547447	WDR79	17	7547447	6	6.658387
SNP17-7547447	WDR79	17	7547447	2	1.501041
SNP17-7547447	WDR79	17	7547447	1	0.741349
SNP17-7547447	WDR79	17	7547447	3	2.450323
rs7141	EFNB3	17	7555326	7	0.420014
rs7141	EFNB3	17	7555326	6	0.098243
SNP17-7587121	DNAH2	17	7587121	4	0.669727
SNP17-7587121	DNAH2	17	7587121	7	0.425301
SNP17-7587121	DNAH2	17	7587121	3	0.655292
SNP17-7587121	DNAH2	17	7587121	6	0.305218
SNP17-7587121	DNAH2	17	7587121	1	0.281065
SNP17-7587121	DNAH2	17	7587121	5	0.582453
rs9909288	DNAH2	17	7614653	7	2.850659
rs9909288	DNAH2	17	7614653	4	1.987927
rs9909288	DNAH2	17	7614653	3	3.315129
rs9909288	DNAH2	17	7614653	6	0.808661
rs17806045	DNAH2	17	7623330	7	1.103122
rs17806045	DNAH2	17	7623330	3	2.969042
rs17806045	DNAH2	17	7623330	6	0.932189
rs11656500	DNAH2	17	7638370	3	0.629994
SNP17-7691863	JMJD3	17	7691863	7	1.12846
rs3744251	LSMD1	17	7701708	4	0.466059
rs12453250	CYB5D1	17	7702237	2	5.244699
rs12453250	CYB5D1	17	7702237	7	0.393014
rs3744252	CYB5D1	17	7702651	4	3.963572

rs4435314	CNTROB	17	7776384	5	0.656676
rs4435314	CNTROB	17	7776384	2	0.699836
rs4435314	CNTROB	17	7776384	8	0.953957
rs11078719	CNTROB	17	7783778	2	3.949267
rs11078719	CNTROB	17	7783778	8	0.279774
rs11078719	CNTROB	17	7783778	5	0.579864
SNP17-7788562	CNTROB	17	7788562	8	0.238946
rs11650083	CNTROB	17	7788680	2	1.716575
rs11650083	CNTROB	17	7788680	5	1.292324
rs4462665	CNTROB	17	7789812	5	0.036043
rs4462665	CNTROB	17	7789812	2	2.023919
rs4462665	CNTROB	17	7789812	8	3.821115
rs7217194	CNTROB	17	7793612	5	1.010733
rs7217194	CNTROB	17	7793612	8	2.519225
rs34598902	GUCY2D	17	7856637	7	0.65836
rs2816	GUCY2D	17	7864289	5	1.682125
SNP17-7987497	PER1	17	7987497	1	4.284901
rs2253820	PER1	17	7988894	1	6.275416
rs2253820	PER1	17	7988894	3	0.445818
rs2241909	AURKB	17	8049064	5	5.859133
rs2241909	AURKB	17	8049064	3	0.066157
rs2241909	AURKB	17	8049064	4	4.222552
rs3826543	C17orf68	17	8073488	5	0.299478
rs3826543	C17orf68	17	8073488	1	4.375406
rs3027238	C17orf68	17	8075786	1	1.287104
rs3027238	C17orf68	17	8075786	3	1.641197
rs3027235	C17orf68	17	8076171	3	0.356658
rs4791641	PFAS	17	8101874	6	0.792848
rs4791641	PFAS	17	8101874	4	1.804806
rs4791641	PFAS	17	8101874	7	0.83861
SNP17-8107183	PFAS	17	8107183	1	5.24023
rs11078738	PFAS	17	8108325	5	0.450364
rs11078738	PFAS	17	8108325	7	0.242557
rs11078738	PFAS	17	8108325	1	4.48727
rs3744651	ARHGEF15	17	8156259	5	0.450165
rs3744651	ARHGEF15	17	8156259	1	0.347796
rs3744651	ARHGEF15	17	8156259	3	0.574717
rs3744647	ARHGEF15	17	8165001	5	2.04937
rs3744647	ARHGEF15	17	8165001	1	3.159223
rs1045172	ARHGEF15	17	8166493	3	0.976147
rs12601097	ODF4	17	8184386	5	0.404967
rs2430949	KRBA2	17	8213492	1	3.556054
rs10520	MYH10	17	8318991	5	1.274332
rs9915880	PIK3R5	17	8732728	1	0.333699
rs9915880	PIK3R5	17	8732728	5	1.140417
rs9915880	PIK3R5	17	8732728	6	0.055492
rs9915880	PIK3R5	17	8732728	3	1.087705

rs8974	STX8	17	9094586	1	3.578093
rs8974	STX8	17	9094586	4	2.843226
rs8974	STX8	17	9094586	5	0.346443
rs1133295	STX8	17	9412436	6	0.510716
rs1133295	STX8	17	9412436	4	0.138977
rs6503235	WDR16	17	9456502	3	1.153223
rs274899	WDR16	17	9487276	4	0.198027
rs274899	WDR16	17	9487276	3	0.545768
rs274899	WDR16	17	9487276	1	5.463105
rs16958933	RCVRN	17	9742095	2	1.761636
rs4668	GAS7	17	9755148	1	1.177875
rs4668	GAS7	17	9755148	4	5.858817
rs4668	GAS7	17	9755148	6	2.856062
rs4668	GAS7	17	9755148	2	6.199298
rs4668	GAS7	17	9755148	8	0.583151
rs1047334	GAS7	17	9755912	4	0.911468
rs1047334	GAS7	17	9755912	6	1.361629
rs1047334	GAS7	17	9755912	5	0.865675
rs1047339	GAS7	17	9756136	6	0.385051
rs1047339	GAS7	17	9756136	5	3.492847
rs1047339	GAS7	17	9756136	4	0.835384
rs1047339	GAS7	17	9756136	1	1.538815
rs1129463	GAS7	17	9756951	4	0.498485
rs1129463	GAS7	17	9756951	2	1.023656
rs1129463	GAS7	17	9756951	1	2.548273
rs1129463	GAS7	17	9756951	5	0.315861
rs15814	GAS7	17	9757115	1	0.337083
rs15814	GAS7	17	9757115	2	0.689148
rs15814	GAS7	17	9757115	6	1.306241
rs15814	GAS7	17	9757115	4	0.069226
rs1129464	GAS7	17	9757177	2	0.703563
rs1129464	GAS7	17	9757177	4	0.622336
rs1129464	GAS7	17	9757177	5	1.40959
rs1129464	GAS7	17	9757177	1	0.138877
rs3177567	GAS7	17	9757356	1	0.772458
rs3177567	GAS7	17	9757356	4	1.305396
rs3177567	GAS7	17	9757356	6	4.012859
rs3177567	GAS7	17	9757356	5	2.075477
rs3177567	GAS7	17	9757356	2	4.285282
rs2270121	GAS7	17	9757396	5	0.008061
rs2270121	GAS7	17	9757396	6	0.491468
rs2270121	GAS7	17	9757396	2	4.62992
rs2270121	GAS7	17	9757396	1	1.365301
rs2270121	GAS7	17	9757396	4	1.425397
rs1047365	GAS7	17	9759695	6	5.267177
rs1047365	GAS7	17	9759695	4	6.626852
rs1047365	GAS7	17	9759695	1	1.164017

rs1047365	GAS7	17	9759695	2	3.813767
rs1047365	GAS7	17	9759695	8	0.23211
rs1047367	GAS7	17	9759851	5	6.743519
rs1047367	GAS7	17	9759851	1	0.839515
rs1047367	GAS7	17	9759851	2	2.51788
rs1047367	GAS7	17	9759851	4	1.134109
rs1047367	GAS7	17	9759851	8	0.219787
rs1047367	GAS7	17	9759851	6	6.321355
rs2240739	GAS7	17	9760866	8	1.378142
rs2240739	GAS7	17	9760866	1	0.186557
rs2240739	GAS7	17	9760866	6	0.944641
rs2240739	GAS7	17	9760866	4	1.420248
rs2286532	GAS7	17	9761003	1	1.301936
rs2286532	GAS7	17	9761003	6	0.997103
rs2286532	GAS7	17	9761003	8	3.884464
rs2286532	GAS7	17	9761003	2	6.382789
rs2286532	GAS7	17	9761003	4	2.228132
rs3744550	MYH13	17	10147320	5	0.521483
rs2074877	MYH13	17	10164439	5	0.037725
rs3760423	MYH13	17	10184442	5	1.288333
rs3744551	MYH8	17	10234420	4	1.889915
rs2285479	MYH3	17	10476743	4	0.233912
rs2285479	MYH3	17	10476743	3	0.730419
rs2285479	MYH3	17	10476743	1	0.523238
rs2285477	MYH3	17	10482240	4	0.816387
rs2285477	MYH3	17	10482240	2	0.857778
rs2285477	MYH3	17	10482240	1	0.706804
rs2285477	MYH3	17	10482240	3	1.454675
rs201626	MYH3	17	10482466	3	0.908193
rs201626	MYH3	17	10482466	1	2.951925
rs2285475	MYH3	17	10483196	1	0.613214
rs2285475	MYH3	17	10483196	4	0.278595
rs2285475	MYH3	17	10483196	3	0.843724
rs2285474	MYH3	17	10483490	3	0.363988
rs2285474	MYH3	17	10483490	4	1.818937
rs2285474	MYH3	17	10483490	1	1.233249
rs2285472	MYH3	17	10483611	4	0.296849
rs2285472	MYH3	17	10483611	2	0.161394
rs2285472	MYH3	17	10483611	3	0.239064
rs2285472	MYH3	17	10483611	1	0.406215
rs876657	MYH3	17	10485141	4	1.387284
rs876657	MYH3	17	10485141	1	0.802891
rs876657	MYH3	17	10485141	3	1.284751
rs876657	MYH3	17	10485141	2	1.357393
SNP17-1054924	C17orf48	17	10549243	6	4.52497
rs445928	C17orf48	17	10555398	5	0.024968
rs445928	C17orf48	17	10555398	4	1.122489

rs445928	C17orf48	17	10555398	7	0.589266
rs445928	C17orf48	17	10555398	6	0.726481
rs379403	C17orf48	17	10555592	7	1.004381
rs379403	C17orf48	17	10555592	6	4.194903
rs379403	C17orf48	17	10555592	5	0.243131
rs379403	C17orf48	17	10555592	4	0.744112
rs387022	C17orf48	17	10555594	7	1.100736
rs387022	C17orf48	17	10555594	4	0.434649
rs387022	C17orf48	17	10555594	5	0.635909
rs617655	LOC388335	17	10557491	5	0.76274
rs617655	LOC388335	17	10557491	7	2.53147
rs617655	LOC388335	17	10557491	6	3.327547
rs617655	LOC388335	17	10557491	4	1.278342
rs411511	LOC388335	17	10557959	7	0.460158
rs411511	LOC388335	17	10557959	4	4.724356
rs411511	LOC388335	17	10557959	6	1.941681
rs411511	LOC388335	17	10557959	5	1.173631
rs375437	LOC388335	17	10558006	7	1.841767
rs375437	LOC388335	17	10558006	6	0.725545
rs375437	LOC388335	17	10558006	5	0.635706
rs375437	LOC388335	17	10558006	4	1.711897
rs387142	LOC388335	17	10558039	6	0.476282
rs387142	LOC388335	17	10558039	5	2.021597
rs387142	LOC388335	17	10558039	7	0.287575
rs387142	LOC388335	17	10558039	4	0.410601
rs435382	LOC388335	17	10558389	4	1.316816
rs435382	LOC388335	17	10558389	5	0.730553
rs435382	LOC388335	17	10558389	7	1.36497
rs374034	LOC388335	17	10558455	7	2.796561
rs374034	LOC388335	17	10558455	4	1.408532
rs440655	LOC388335	17	10558528	4	2.354906
rs440655	LOC388335	17	10558528	6	2.355877
rs440655	LOC388335	17	10558528	5	1.510189
rs202311	LOC388335	17	10574083	4	1.876169
rs202311	LOC388335	17	10574083	5	2.59527
rs202311	LOC388335	17	10574083	6	2.558365
rs202311	LOC388335	17	10574083	7	1.948766
rs11078022	DNAH9	17	11452182	3	0.472884
rs12449553	DNAH9	17	11698281	1	1.045156
rs3760435	DNAH9	17	11727016	5	1.399773
rs8070256	DNAH9	17	11727676	4	0.369507
rs8070256	DNAH9	17	11727676	2	0.656778
rs8070256	DNAH9	17	11727676	5	0.553491
rs2286303	DNAH9	17	11774012	5	0.761874
rs2072254	RICH2	17	12793184	7	2.599478
rs3213687	RICH2	17	12802895	6	5.215968
rs3213687	RICH2	17	12802895	7	0.25731

rs6949	ELAC2	17	12836580	3	0.04153
rs17552022	ELAC2	17	12839020	6	0.828093
rs34342426	COX10	17	13920783	3	0.56973
rs3785655	HS3ST3B1	17	14189892	1	1.315689
rs3785655	HS3ST3B1	17	14189892	3	0.705633
rs3785655	HS3ST3B1	17	14189892	5	1.274345
rs3785655	HS3ST3B1	17	14189892	2	2.309484
rs11653482	FLJ45831	17	14611752	5	0.080924
rs6502391	FLJ45831	17	14613416	4	0.535953
rs6502391	FLJ45831	17	14613416	7	0.571399
rs6502391	FLJ45831	17	14613416	1	0.229064
rs1860379	FLJ45831	17	14613785	1	0.161217
rs1860379	FLJ45831	17	14613785	6	0.61834
rs1860379	FLJ45831	17	14613785	2	1.100237
rs11654383	PMP22	17	15073839	1	4.960111
rs230898	TEKT3	17	15158162	2	5.316911
rs230898	TEKT3	17	15158162	3	0.349839
SNP17-15437452	CDRT1	17	15437452	6	1.195611
rs9905520	ZNF286A	17	15561942	3	1.829656
rs9905520	ZNF286A	17	15561942	8	1.917656
rs9905520	ZNF286A	17	15561942	7	0.734073
rs9905520	ZNF286A	17	15561942	5	0.559843
rs11650318	MGC51025	17	15585231	7	0.544758
rs2240593	MGC51025	17	15588448	7	1.40348
rs2240593	MGC51025	17	15588448	1	2.95145
rs2240593	MGC51025	17	15588448	5	0.213306
rs3813769	TRPV2	17	16259657	7	0.686109
rs8121	TRPV2	17	16266693	7	1.31902
rs1129235	TRPV2	17	16266730	1	2.359635
rs1129235	TRPV2	17	16266730	7	0.585945
rs4570894	C17orf76	17	16286906	3	0.70261
rs1141283	C17orf76	17	16287482	3	0.966373
rs1141283	C17orf76	17	16287482	5	0.57192
rs1141283	C17orf76	17	16287482	8	0.543493
rs4792745	C17orf76	17	16287657	1	4.496996
rs4792745	C17orf76	17	16287657	8	0.268456
rs4792745	C17orf76	17	16287657	4	1.233073
rs4792745	C17orf76	17	16287657	7	2.717383
rs4792745	C17orf76	17	16287657	6	4.370714
rs10491106	ZNF287	17	16395636	3	1.045249
rs3813762	LOC644422	17	16461809	6	3.490971
rs9906261	ZNF624	17	16465654	6	4.784702
rs8065506	ZNF624	17	16468520	6	0.041096
rs8065506	ZNF624	17	16468520	5	5.559606
rs11078355	TNFRSF13B	17	16783637	4	0.025091
rs14003	LOC201164	17	17045439	3	0.928996
rs14003	LOC201164	17	17045439	7	2.824822

rs14003	LOC201164	17	17045439	1	2.141698
rs9908205	LOC201164	17	17046340	3	0.462877
rs9908205	LOC201164	17	17046340	2	0.066702
rs9908205	LOC201164	17	17046340	1	0.216452
rs9908205	LOC201164	17	17046340	7	3.530674
rs4985749	LOC201164	17	17046407	7	1.248636
rs4985749	LOC201164	17	17046407	1	5.295955
rs4985749	LOC201164	17	17046407	3	0.037199
rs3803761	FLCN	17	17057137	7	2.760759
rs3803761	FLCN	17	17057137	1	4.892584
rs1736209	FLCN	17	17081210	1	0.318681
rs1736209	FLCN	17	17081210	5	0.85098
rs2240876	NT5M	17	17190884	3	1.638443
rs35590625	LOC644909	17	17267088	3	0.179836
rs35590625	LOC644909	17	17267088	5	0.441444
rs35590625	LOC644909	17	17267088	6	0.763501
rs1242489	MED9	17	17335336	5	1.588335
rs1242489	MED9	17	17335336	1	0.812951
rs1242489	MED9	17	17335336	8	1.960976
rs1242489	MED9	17	17335336	3	1.252666
rs711352	RASD1	17	17338543	2	1.730709
rs711352	RASD1	17	17338543	4	0.054151
SNP17-17637256	RAI1	17	17637256	7	8.627
SNP17-17637256	RAI1	17	17637256	4	5.408154
SNP17-17637256	RAI1	17	17637256	6	6.72255
rs4584886	LRRC48	17	17836930	3	0.741307
rs4584886	LRRC48	17	17836930	4	0.555
rs4584886	LRRC48	17	17836930	6	1.034499
SNP17-17848493	LRRC48	17	17848493	1	5.372146
rs33997182	ATPAF2	17	17862720	1	4.223751
rs2280777	MYO15A	17	17982232	2	0.642794
rs2280777	MYO15A	17	17982232	7	0.119184
rs2280777	MYO15A	17	17982232	1	0.188734
rs2280777	MYO15A	17	17982232	3	0.287272
rs854777	MYO15A	17	17987623	1	2.299532
rs854777	MYO15A	17	17987623	2	2.095009
rs854777	MYO15A	17	17987623	8	2.191937
rs2272571	MYO15A	17	17987914	5	1.705614
rs854772	MYO15A	17	17995954	1	1.368104
rs854772	MYO15A	17	17995954	2	2.227953
rs854772	MYO15A	17	17995954	5	1.397557
rs854772	MYO15A	17	17995954	6	1.294887
rs854772	MYO15A	17	17995954	8	1.854064
rs854772	MYO15A	17	17995954	7	1.924305
rs712270	MYO15A	17	17997892	3	1.810789
rs712272	MYO15A	17	17999246	1	0.286778
rs8077577	MYO15A	17	18005455	3	0.472265

rs8077577	MYO15A	17	18005455	8	0.240363
rs854800	MYO15A	17	18017900	1	1.850013
rs854800	MYO15A	17	18017900	4	0.813754
rs854800	MYO15A	17	18017900	8	2.626163
rs13284	ALKBH5	17	18052855	3	1.293737
rs13284	ALKBH5	17	18052855	8	0.022908
rs3088233	ALKBH5	17	18053400	3	0.799927
rs3088233	ALKBH5	17	18053400	8	4.617925
rs2605143	SMCR7	17	18108230	8	0.471365
SNP17-18108409	SMCR7	17	18108409	5	0.687146
rs3783	SHMT1	17	18172742	2	5.051488
rs3783	SHMT1	17	18172742	3	0.121022
rs3783	SHMT1	17	18172742	1	0.329302
rs2461838	LOC645023	17	18205989	3	0.787157
rs2461838	LOC645023	17	18205989	1	0.060163
rs2461838	LOC645023	17	18205989	2	0.704511
rs4924765	LOC729288	17	18525961	2	0.404681
rs4924765	LOC729288	17	18525961	1	3.580106
rs4924765	LOC729288	17	18525961	8	1.19452
rs4924765	LOC729288	17	18525961	3	2.721359
SNP17-18593795	FBXW10	17	18593795	5	4.534826
SNP17-18593795	FBXW10	17	18593795	3	4.528796
SNP17-18593795	FBXW10	17	18593795	8	3.769953
SNP17-18593795	FBXW10	17	18593795	2	4.584997
rs4393623	PRPSAP2	17	18716625	3	0.703528
rs4393623	PRPSAP2	17	18716625	7	0.11198
rs4393623	PRPSAP2	17	18716625	5	0.415369
rs1044787	PRPSAP2	17	18775277	4	0.73125
rs1044787	PRPSAP2	17	18775277	7	0.896442
rs2472717	FAM83G	17	18820374	1	1.767777
rs2472717	FAM83G	17	18820374	5	0.673652
rs2472717	FAM83G	17	18820374	6	0.835266
rs2472717	FAM83G	17	18820374	4	1.481974
rs2472717	FAM83G	17	18820374	3	0.979671
rs2472717	FAM83G	17	18820374	7	1.571285
rs2472715	SLC5A10	17	18820993	6	1.323808
rs2472715	SLC5A10	17	18820993	5	0.818632
rs2472715	SLC5A10	17	18820993	3	1.741217
rs2472715	SLC5A10	17	18820993	1	2.231201
rs2469860	FAM83G	17	18821386	3	0.383315
rs2469860	FAM83G	17	18821386	8	0.411919
rs2469860	FAM83G	17	18821386	5	0.442991
rs2469860	FAM83G	17	18821386	7	0.289399
rs2469860	FAM83G	17	18821386	1	0.576016
rs2074271	FAM83G	17	18822939	4	1.505361
rs1624825	SLC5A10	17	18864543	3	0.058281
rs2233072	MAPK7	17	19222421	6	1.677012

rs2233072	MAPK7	17	19222421	2	4.72699
rs1042183	ALDH3A1	17	19581913	8	0.319961
rs1042183	ALDH3A1	17	19581913	4	0.474427
rs1042183	ALDH3A1	17	19581913	6	1.279428
rs1042183	ALDH3A1	17	19581913	2	1.88078
rs2228100	ALDH3A1	17	19583544	3	0.543194
rs2228100	ALDH3A1	17	19583544	1	5.565546
rs2072327	LOC441784	17	19592074	1	0.313941
rs9898251	LOC441784	17	19599891	7	4.752755
rs9898251	LOC441784	17	19599891	6	4.202442
rs9898251	LOC441784	17	19599891	5	4.509213
rs115326	ULK2	17	19618341	1	6.91526
rs115326	ULK2	17	19618341	6	7.209334
rs150122	ULK2	17	19654332	1	1.815274
rs157397	ULK2	17	19670087	1	1.035641
rs2108978	AKAP10	17	19802050	6	2.7486
rs2108978	AKAP10	17	19802050	7	2.236842
rs3764436	SPECC1	17	19954394	5	0.040403
rs3764436	SPECC1	17	19954394	1	3.882634
rs9902683	LOC729404	17	19956020	1	4.85105
rs9902683	LOC729404	17	19956020	5	1.074538
rs2703806	SPECC1	17	20048831	7	0.091968
rs2703806	SPECC1	17	20048831	4	7.122042
rs2703806	SPECC1	17	20048831	6	0.340473
rs2703806	SPECC1	17	20048831	2	7.319666
rs2703806	SPECC1	17	20048831	1	0.268153
rs1815569	LOC729334	17	20581369	6	2.701084
rs8068101	LOC645294	17	20598390	1	7.105595
rs8068101	LOC645294	17	20598390	8	3.778179
rs8068101	LOC645294	17	20598390	3	6.448637
rs8068101	LOC645294	17	20598390	7	3.933955
rs8068101	LOC645294	17	20598390	6	3.907262
rs8073465	MGC87631	17	20739584	5	1.566487
rs11204329	USP22	17	20843295	6	0.805647
rs11204329	USP22	17	20843295	2	4.005354
rs11204329	USP22	17	20843295	7	0.336517
rs11541314	USP22	17	20846737	6	0.074747
rs11541314	USP22	17	20846737	1	4.750713
rs11541311	USP22	17	20851796	6	3.955504
rs11541311	USP22	17	20851796	8	5.874126
rs11541311	USP22	17	20851796	7	2.430861
rs12449311	MGC33894	17	21088113	1	0.02607
rs6587170	LOC645479	17	21437192	5	0.785171
rs35094692	LOC729550	17	22777629	7	0.199331
rs35094692	LOC729550	17	22777629	2	1.483224
rs35094692	LOC729550	17	22777629	1	1.701236
rs2945379	LOC729538	17	22877777	2	1.474269

rs2945379	LOC729538	17	22877777	1	0.400076
rs2945379	LOC729538	17	22877777	4	5.065828
rs2945379	LOC729538	17	22877777	7	0.214793
rs2945379	LOC729538	17	22877777	6	0.472241
rs7222355	LOC729538	17	22878315	1	0.509851
rs7222355	LOC729538	17	22878315	7	0.962903
rs7222355	LOC729538	17	22878315	6	0.408751
rs7222355	LOC729538	17	22878315	4	3.192188
rs7222355	LOC729538	17	22878315	2	1.186336
rs3751093	LGALS9	17	22982431	3	0.649663
rs3751093	LGALS9	17	22982431	5	0.82744
rs3751093	LGALS9	17	22982431	6	0.56789
rs2297518	NOS2A	17	23120724	3	1.658799
rs2297518	NOS2A	17	23120724	4	1.906813
rs2297518	NOS2A	17	23120724	5	2.640682
rs35668234	NLK	17	23393462	3	0.380039
rs9897022	PYY2	17	23578902	5	0.131958
rs9897022	PYY2	17	23578902	6	0.156049
rs9897022	PYY2	17	23578902	4	1.306741
rs3093704	TNFAIP1	17	23697443	5	0.037231
rs3093704	TNFAIP1	17	23697443	7	0.774445
rs2277667	SEBOX	17	23716043	7	0.405781
rs2277667	SEBOX	17	23716043	5	0.575074
rs2227728	VTN	17	23718610	5	0.983473
SNP17-23718988	VTN	17	23718988	3	1.065907
SNP17-23718988	VTN	17	23718988	1	0.196209
rs739439	SARM1	17	23747949	8	0.21047
rs739439	SARM1	17	23747949	4	3.138601
rs739439	SARM1	17	23747949	3	0.729623
rs739439	SARM1	17	23747949	1	4.646869
rs739439	SARM1	17	23747949	5	5.996466
rs739439	SARM1	17	23747949	7	1.763466
rs11568466	SLC13A2	17	23841664	1	0.806918
rs12449554	FOXN1	17	23881915	1	2.353045
rs12051648	SUPT6H	17	24033876	8	3.473009
rs12051648	SUPT6H	17	24033876	2	2.485201
rs12051648	SUPT6H	17	24033876	4	1.027961
rs12051648	SUPT6H	17	24033876	5	3.698298
rs12051648	SUPT6H	17	24033876	7	1.352075
GA014112	SUPT6H	17	24038523	5	0.70631
rs3809797	NEK8	17	24089338	5	1.453323
rs2242345	ERAL1	17	24209953	5	0.313036
rs2242345	ERAL1	17	24209953	2	2.495092
rs2242345	ERAL1	17	24209953	7	0.454147
rs2242345	ERAL1	17	24209953	8	0.830714
rs4795475	PHF12	17	24263502	7	0.383317
rs12941884	SEZ6	17	24308569	7	0.613418

rs1976165	SEZ6	17	24310977	7	1.849013
rs1976165	SEZ6	17	24310977	3	0.596044
rs2320786	MYO18A	17	24446179	5	0.106473
rs2320786	MYO18A	17	24446179	3	0.00489
rs12601535	MYO18A	17	24465203	5	0.298527
rs12601535	MYO18A	17	24465203	3	1.043519
rs12601535	MYO18A	17	24465203	7	1.196434
rs12601535	MYO18A	17	24465203	2	5.292878
rs1047790	CRYBA1	17	24604882	5	0.718052
rs1047790	CRYBA1	17	24604882	7	0.968577
rs3785961	NUFIP2	17	24615451	7	0.129948
rs11657079	CORO6	17	24968215	7	0.953307
rs9897794	EFCAB5	17	25320453	7	0.982357
rs9897794	EFCAB5	17	25320453	5	0.652073
rs9897794	EFCAB5	17	25320453	2	2.611304
rs9897794	EFCAB5	17	25320453	3	1.408213
rs1042173	SLC6A4	17	25549137	3	0.721985
rs1042173	SLC6A4	17	25549137	5	0.697067
rs7342921	BLMH	17	25636574	8	0.377042
rs7342921	BLMH	17	25636574	2	4.480173
rs7342921	BLMH	17	25636574	3	2.341466
rs7342921	BLMH	17	25636574	5	6.939708
rs999797	ATAD5	17	26183455	5	0.259038
rs9910051	ATAD5	17	26185328	5	0.01071
rs3816780	ATAD5	17	26185484	5	0.092033
rs17826219	ATAD5	17	26185971	5	0.044202
rs3087591	EVI2B	17	26655096	5	4.823534
rs3087591	EVI2B	17	26655096	4	1.631993
rs3087591	EVI2B	17	26655096	1	4.264715
rs1992	RAB11FIP4	17	26883136	4	4.4558
rs2290436	RAB11FIP4	17	26883626	4	1.079066
rs4795615	RAB11FIP4	17	26884772	3	0.00973
rs4795615	RAB11FIP4	17	26884772	4	1.682629
rs4795615	RAB11FIP4	17	26884772	7	0.959766
rs1548470	RAB11FIP4	17	26886982	3	0.233769
rs1548471	RAB11FIP4	17	26887119	1	2.89473
rs1548471	RAB11FIP4	17	26887119	3	0.898892
rs1548471	RAB11FIP4	17	26887119	7	1.063551
rs1551356	RAB11FIP4	17	26888126	7	0.306147
rs1551356	RAB11FIP4	17	26888126	3	0.534514
GA032273	UTP6	17	27252769	1	6.510684
rs739455	MYO1D	17	27844963	1	4.619067
rs739455	MYO1D	17	27844963	6	3.877723
rs34727343	SPACA3	17	28346540	1	2.569226
rs28933	ACCN1	17	28364795	7	1.742528
rs28933	ACCN1	17	28364795	3	1.837716
rs28933	ACCN1	17	28364795	2	5.019427

rs28933	ACCN1	17	28364795	8	4.25703
rs28933	ACCN1	17	28364795	4	0.452484
rs7214382	ACCN1	17	28643858	7	0.546508
rs4795846	TLK2P1	17	29388835	8	6.000017
rs4795846	TLK2P1	17	29388835	1	6.234254
rs4795846	TLK2P1	17	29388835	4	5.196982
rs4795846	TLK2P1	17	29388835	3	6.185878
rs4795846	TLK2P1	17	29388835	5	5.604916
rs3744516	TLK2P1	17	29390113	2	5.202023
rs3744516	TLK2P1	17	29390113	5	3.845401
rs3744516	TLK2P1	17	29390113	1	4.876304
rs3744516	TLK2P1	17	29390113	3	4.785503
rs3744516	TLK2P1	17	29390113	7	1.309623
rs41274294	CCL11	17	29639284	3	2.359412
rs4795007	FLJ44815	17	29927412	3	3.082838
rs2230553	CCT6B	17	30293761	3	0.481293
rs2230553	CCT6B	17	30293761	1	0.364022
rs11658803	CCT6B	17	30310777	4	2.680227
SNP17-3031247	CCT6B	17	30312476	4	0.746157
SNP17-3031247	CCT6B	17	30312476	3	1.380381
SNP17-3031247	CCT6B	17	30312476	1	3.549257
SNP17-3031247	CCT6B	17	30312476	7	0.74429
rs3744355	CCDC16	17	30313159	4	0.491738
rs8249	CCDC16	17	30314050	3	0.108258
rs8249	CCDC16	17	30314050	4	0.539237
rs8249	CCDC16	17	30314050	7	0.251544
rs8249	CCDC16	17	30314050	1	3.442048
rs1871892	FNDC8	17	30472931	5	0.527597
rs1871892	FNDC8	17	30472931	8	0.43947
SNP17-3050135	UNC45B	17	30501355	6	1.841859
SNP17-3050135	UNC45B	17	30501355	1	3.561637
rs3744365	UNC45B	17	30537704	8	1.075524
rs17547201	AMAC1	17	30544230	8	1.87441
rs17547201	AMAC1	17	30544230	1	1.701636
rs1047268	SLFN11	17	30701608	1	0.724988
SNP17-3071457	SLFN11	17	30714579	3	1.448815
SNP17-3071457	SLFN11	17	30714579	1	2.78965
rs7210421	SLFN13	17	30788845	5	0.090712
SNP17-3079610	SLFN13	17	30796109	6	0.075868
rs8065635	SLFN12L	17	30888820	3	0.01057
rs1001441	SLFN14	17	30900196	1	2.609894
rs321613	SLFN14	17	30905831	3	0.83865
rs10068	PEX12	17	30926078	1	0.190847
rs10068	PEX12	17	30926078	7	0.571268
rs10068	PEX12	17	30926078	8	1.109575
rs10068	PEX12	17	30926078	2	0.394823
rs2278950	PEX12	17	30929687	6	2.082492

rs17670584	AP2B1	17	31023017	1	0.495289
rs2240903	AP2B1	17	31076270	3	0.011689
rs2240902	RASL10B	17	31093691	3	1.323175
rs3744374	GAS2L2	17	31096668	7	0.035291
rs11654604	GAS2L2	17	31101345	8	0.568138
rs11654604	GAS2L2	17	31101345	7	0.257238
rs2306630	C17orf66	17	31206454	3	1.107952
rs1065341	CCL5	17	31222706	5	0.812567
rs2072667	CCL23	17	31369075	3	0.458837
rs1063340	CCL3	17	31439876	3	0.362024
rs1063340	CCL3	17	31439876	7	1.351027
rs2306590	MYO19	17	31928393	2	3.966389
rs2306590	MYO19	17	31928393	7	0.025963
rs1106908	GGNBP2	17	32016708	1	0.68116
rs2306658	AATF	17	32380425	2	4.654458
rs2306658	AATF	17	32380425	4	1.618615
rs2306658	AATF	17	32380425	7	0.469679
rs2306658	AATF	17	32380425	8	2.560995
rs2306658	AATF	17	32380425	1	0.221434
rs2229416	ACACA	17	32683979	3	0.113646
rs2229416	ACACA	17	32683979	7	4.615476
rs1714987	C17orf78	17	32817123	3	0.405323
rs1714987	C17orf78	17	32817123	1	2.180092
rs7211875	TADA2L	17	32845581	7	1.266594
rs1051838	DUSP14	17	32944946	3	0.080201
rs1051838	DUSP14	17	32944946	7	0.122798
rs1051838	DUSP14	17	32944946	1	3.297161
rs3110623	AP1GBP1	17	32976338	5	0.994546
rs3110623	AP1GBP1	17	32976338	3	1.21613
rs3110623	AP1GBP1	17	32976338	8	4.287084
rs3110623	AP1GBP1	17	32976338	1	5.872524
rs2688	HNF1B	17	33121044	5	0.023496
rs2688	HNF1B	17	33121044	3	0.600015
rs1058166	HNF1B	17	33121104	3	0.308655
rs2689	HNF1B	17	33121214	1	3.015559
rs9896972	LOC284100	17	33308019	7	3.787636
rs8075324	ARHGAP23	17	33876008	3	0.455708
rs8075324	ARHGAP23	17	33876008	1	0.039284
rs8075324	ARHGAP23	17	33876008	2	1.496379
rs8075324	ARHGAP23	17	33876008	7	1.280679
rs228290	PIP4K2B	17	34180293	4	3.522056
rs228290	PIP4K2B	17	34180293	6	0.400892
rs228290	PIP4K2B	17	34180293	8	0.298209
rs228290	PIP4K2B	17	34180293	5	1.240647
rs228290	PIP4K2B	17	34180293	3	0.210037
rs1130638	LASP1	17	34308298	4	1.142649
rs1130638	LASP1	17	34308298	3	0.866705

rs1130638	LASP1	17	34308298	1	4.48748
rs12603439	PLXDC1	17	34473898	7	1.204165
rs12603439	PLXDC1	17	34473898	3	0.486747
rs12603439	PLXDC1	17	34473898	4	1.695734
rs12603439	PLXDC1	17	34473898	8	0.619454
rs12603439	PLXDC1	17	34473898	6	0.274813
rs12453592	PLXDC1	17	34475999	4	0.472458
rs12453592	PLXDC1	17	34475999	3	0.520393
rs12453595	PLXDC1	17	34476009	3	1.377946
rs12453595	PLXDC1	17	34476009	4	0.228192
rs12453595	PLXDC1	17	34476009	7	0.438597
rs7221482	PLXDC1	17	34476652	1	0.031863
rs13755	PLXDC1	17	34476984	2	0.622181
rs13755	PLXDC1	17	34476984	1	1.040568
rs13755	PLXDC1	17	34476984	7	0.139251
rs13755	PLXDC1	17	34476984	3	0.403743
rs13755	PLXDC1	17	34476984	8	6.633419
rs3254	PLXDC1	17	34477162	3	0.307288
rs3254	PLXDC1	17	34477162	7	1.56988
rs3254	PLXDC1	17	34477162	4	2.101838
rs14325	PLXDC1	17	34477254	8	0.468828
rs14325	PLXDC1	17	34477254	6	7.856469
rs14325	PLXDC1	17	34477254	7	0.698355
rs14325	PLXDC1	17	34477254	4	1.728003
rs14325	PLXDC1	17	34477254	3	0.451735
rs12602945	PLXDC1	17	34497453	7	4.170878
rs12602945	PLXDC1	17	34497453	3	0.517262
rs12602945	PLXDC1	17	34497453	4	3.413317
rs12602945	PLXDC1	17	34497453	8	0.911692
rs12602945	PLXDC1	17	34497453	2	4.873986
rs12602945	PLXDC1	17	34497453	6	1.921006
rs519512	STAC2	17	34620514	6	0.056788
rs521397	STAC2	17	34620732	8	7.854725
rs521397	STAC2	17	34620732	6	2.511629
rs1054488	CRKRS	17	34941494	8	5.661398
rs11078918	NEUROD2	17	35014711	5	1.158306
GA032374	STARD3	17	35069275	4	0.047722
GA032377	STARD3	17	35072983	4	4.512317
rs1053651	TCAP	17	35075837	3	0.533329
rs1053651	TCAP	17	35075837	7	0.292055
rs1053651	TCAP	17	35075837	5	1.285688
rs2941503	PERLD1	17	35082271	3	0.496244
rs2941503	PERLD1	17	35082271	7	0.247291
rs2941503	PERLD1	17	35082271	2	3.866197
rs2941503	PERLD1	17	35082271	8	0.301602
rs2941503	PERLD1	17	35082271	5	0.106384
rs907087	PERLD1	17	35082313	7	2.264707

rs907087	PERLD1	17	35082313	3	1.550895
rs907087	PERLD1	17	35082313	2	0.609276
rs907087	PERLD1	17	35082313	8	3.523352
rs907087	PERLD1	17	35082313	4	2.048966
rs907087	PERLD1	17	35082313	5	4.081007
SNP17-35137702	ERBB2	17	35137702	4	5.389282
rs2305479	GSDML	17	35315743	4	0.559215
rs2305479	GSDML	17	35315743	3	0.785928
rs2305479	GSDML	17	35315743	5	0.36445
rs2305479	GSDML	17	35315743	8	0.94009
rs3902025	GSDM1	17	35372780	5	0.945584
rs3902025	GSDM1	17	35372780	3	0.285667
rs3902025	GSDM1	17	35372780	7	0.477103
rs3902025	GSDM1	17	35372780	8	5.989913
rs7212944	GSDM1	17	35376212	6	0.54682
rs7212944	GSDM1	17	35376212	7	3.010623
rs7212944	GSDM1	17	35376212	1	3.932185
SNP17-35384713	GSDM1	17	35384713	5	0.838639
SNP17-35384713	GSDM1	17	35384713	8	3.930477
SNP17-35384713	GSDM1	17	35384713	7	0.847567
SNP17-35384713	GSDM1	17	35384713	1	4.490907
rs25645	CSF3	17	35426669	7	0.8526
rs25645	CSF3	17	35426669	8	1.135504
rs2827	CSF3	17	35427263	3	0.544595
rs1042657	CSF3	17	35427318	6	6.97298
rs1042658	CSF3	17	35427428	5	0.553244
rs1042658	CSF3	17	35427428	1	1.839775
rs1042658	CSF3	17	35427428	3	0.595726
rs17850739	MED24	17	35439632	3	0.739884
GA032434	RAPGEFL1	17	35604109	5	0.24703
GA032434	RAPGEFL1	17	35604109	6	0.99238
rs13706	CDC6	17	35710677	6	5.143622
rs3169906	TNS4	17	35886287	3	0.520391
rs3169906	TNS4	17	35886287	2	4.725943
rs874889	KRT24	17	36110972	2	0.444076
rs874889	KRT24	17	36110972	1	0.914807
rs981684	KRT27	17	36189338	2	1.707261
rs981684	KRT27	17	36189338	1	3.148692
rs17558532	KRT27	17	36189402	2	1.921277
SNP17-36209517	KRT28	17	36209517	6	1.660972
GA014320	KRT10	17	36231988	5	0.889663
GA014320	KRT10	17	36231988	1	0.918338
rs2240078	TMEM99	17	36244360	3	0.446658
rs11650915	KRT12	17	36276922	1	3.930829
rs11650915	KRT12	17	36276922	3	0.001091
rs11650915	KRT12	17	36276922	4	0.367781
rs3169914	KRT20	17	36285921	7	0.177694

rs3169914	KRT20	17	36285921	2	3.473845
rs3169914	KRT20	17	36285921	3	0.515015
rs3169914	KRT20	17	36285921	5	0.358242
rs7212483	KRT20	17	36294578	2	3.788225
rs7212483	KRT20	17	36294578	7	0.525563
rs7212483	KRT20	17	36294578	3	0.952752
rs9257	KRT23	17	36338030	1	1.056114
rs9257	KRT23	17	36338030	2	4.879586
rs9257	KRT23	17	36338030	7	0.829066
rs9257	KRT23	17	36338030	4	1.329079
SNP17-3638861(	KRT40	17	36388610	2	1.842973
SNP17-3638861(	KRT40	17	36388610	7	1.779642
SNP17-3639082	KRT40	17	36390823	7	0.647031
SNP17-3639082	KRT40	17	36390823	1	0.735421
SNP17-3639082	KRT40	17	36390823	3	0.703138
SNP17-3639082	KRT40	17	36390823	4	0.261677
SNP17-3639082	KRT40	17	36390823	6	1.214681
SNP17-3639082	KRT40	17	36390823	2	1.607319
SNP17-3639289(	KRT40	17	36392896	2	2.572095
SNP17-3639289(	KRT40	17	36392896	7	1.605129
SNP17-3639374	KRT40	17	36393747	2	6.49166
SNP17-3639374	KRT40	17	36393747	7	0.702785
rs937403	KRTAP4-6	17	36549542	2	2.529314
rs937403	KRTAP4-6	17	36549542	8	2.206692
rs937403	KRTAP4-6	17	36549542	7	0.641297
rs16966703	KRT33A	17	36755924	3	0.350211
rs16966703	KRT33A	17	36755924	1	2.17657
rs1047449	KRT31	17	36803677	1	2.51691
rs1047449	KRT31	17	36803677	3	0.104749
rs1047449	KRT31	17	36803677	7	0.088881
rs1047448	KRT31	17	36803778	1	4.182413
rs1047448	KRT31	17	36803778	3	1.568847
rs1047448	KRT31	17	36803778	7	4.865269
rs17737019	KRT37	17	36830706	4	0.584739
rs8071814	KRT37	17	36830741	3	0.545388
SNP17-3683426	KRT37	17	36834265	8	0.155188
SNP17-3683426	KRT37	17	36834265	5	0.202581
SNP17-3683426	KRT37	17	36834265	6	0.722987
rs877459	KRT38	17	36846946	2	3.037435
rs897416	KRT38	17	36847294	3	2.885098
rs12948056	KRT32	17	36873925	4	0.016642
rs12948056	KRT32	17	36873925	2	2.03165
rs12948056	KRT32	17	36873925	8	0.843001
rs12948056	KRT32	17	36873925	3	0.391625
SNP17-3687409	KRT32	17	36874091	6	1.492106
SNP17-3687409	KRT32	17	36874091	8	5.097775
SNP17-3687409	KRT32	17	36874091	7	0.909757

SNP17-36874091	KRT32	17	36874091	5	1.012722
SNP17-36874091	KRT32	17	36874091	4	0.046628
SNP17-36875594	KRT32	17	36875594	1	0.999047
SNP17-36875594	KRT32	17	36875594	4	0.628944
SNP17-36875594	KRT32	17	36875594	5	1.503486
SNP17-36875594	KRT32	17	36875594	6	1.947137
rs1807434	KRT32	17	36877148	1	0.802357
rs1807434	KRT32	17	36877148	5	1.403954
rs743686	KRT35	17	36890770	1	0.52148
rs743686	KRT35	17	36890770	5	0.127999
rs743686	KRT35	17	36890770	2	0.217994
rs743686	KRT35	17	36890770	3	0.386712
rs743686	KRT35	17	36890770	4	0.000707
rs743686	KRT35	17	36890770	7	0.985597
rs11657323	KRT36	17	36896866	3	0.934871
rs11657323	KRT36	17	36896866	2	0.877679
rs11657323	KRT36	17	36896866	8	1.116298
rs2301354	KRT36	17	36897172	5	0.579141
rs2301354	KRT36	17	36897172	4	0.480996
rs9891361	KRT13	17	36913439	5	1.722445
rs9891361	KRT13	17	36913439	6	0.905034
rs4796681	KRT16	17	37021270	3	0.471747
rs4796681	KRT16	17	37021270	8	4.823433
rs7213337	HAP1	17	37132954	2	2.52025
rs33914436	HAP1	17	37137085	7	0.082613
rs35612698	HAP1	17	37137591	4	0.62188
rs35612698	HAP1	17	37137591	5	0.435996
rs4796693	HAP1	17	37138109	6	0.045664
rs4796693	HAP1	17	37138109	8	1.410501
rs4796693	HAP1	17	37138109	2	2.739842
rs4796693	HAP1	17	37138109	7	0.538303
rs1128966	NT5C3L	17	37237375	6	6.580381
rs1128966	NT5C3L	17	37237375	5	0.099088
rs1128966	NT5C3L	17	37237375	8	1.150504
rs1128966	NT5C3L	17	37237375	3	0.936401
rs4796712	NT5C3L	17	37240656	6	0.808928
rs4796712	NT5C3L	17	37240656	8	0.842462
rs17498875	KLHL11	17	37263481	7	1.177519
rs17498875	KLHL11	17	37263481	4	0.28473
rs3809878	TTC25	17	37348361	1	3.655796
rs3809878	TTC25	17	37348361	3	1.226908
rs3809878	TTC25	17	37348361	5	0.226524
rs11079028	CNP	17	37379659	4	1.31891
rs4796751	CNP	17	37381063	8	0.317736
rs4796751	CNP	17	37381063	1	4.078381
rs4796751	CNP	17	37381063	3	0.156843
rs4796751	CNP	17	37381063	6	0.53605

rs4796760	ZNF385C	17	37431741	6	2.044323
rs4796760	ZNF385C	17	37431741	1	4.66093
SNP17-37510581	DHX58	17	37510581	5	6.225215
SNP17-37510581	DHX58	17	37510581	1	4.702464
rs2074158	DHX58	17	37510689	5	0.399081
rs2074158	DHX58	17	37510689	1	2.570976
rs17659391	GCN5L2	17	37520465	2	3.720459
rs1122326	HSPB9	17	37528399	1	2.959342
rs1122326	HSPB9	17	37528399	5	0.863735
rs939881	KCNH4	17	37575778	3	0.552786
rs4796777	HCRT	17	37590929	5	0.589053
rs1135669	STAT5A	17	37713263	3	0.872767
rs1135669	STAT5A	17	37713263	1	4.723357
rs1135669	STAT5A	17	37713263	5	3.946311
rs2067694	STAT5A	17	37716057	1	5.216234
rs709631	PTRF	17	37809465	6	0.132498
rs709631	PTRF	17	37809465	1	3.626879
rs709631	PTRF	17	37809465	5	0.23575
rs709631	PTRF	17	37809465	3	1.111947
rs607795	HSD17BP1	17	37952858	4	6.257066
rs607795	HSD17BP1	17	37952858	3	2.902295
rs607795	HSD17BP1	17	37952858	6	3.106926
rs2277618	PLEKHH3	17	38076204	8	1.78133
rs2277618	PLEKHH3	17	38076204	6	1.324631
rs2277618	PLEKHH3	17	38076204	5	0.352826
rs2277618	PLEKHH3	17	38076204	2	5.336431
rs2277618	PLEKHH3	17	38076204	4	1.497462
rs2271029	CNTNAP1	17	38089448	5	0.338066
rs12449771	CNTNAP1	17	38105311	8	1.676724
rs1043284	RUNDC1	17	38399053	1	4.482001
rs10840	IFI35	17	38419943	1	4.962604
rs4986852	BRCA1	17	38497955	7	4.042541
rs4986850	BRCA1	17	38498997	1	5.54742
rs15359	DUSP3	17	39199505	1	4.058142
rs15359	DUSP3	17	39199505	7	0.062783
rs11713	DUSP3	17	39201350	7	0.18175
rs231522	MPP2	17	39310128	8	0.975869
rs231521	MPP2	17	39310684	6	0.210473
rs231521	MPP2	17	39310684	8	2.879728
rs124721	MPP2	17	39315334	8	0.258959
rs1058046	PYY	17	39386057	8	0.768648
rs2070592	PYY	17	39386857	1	0.472896
rs2070592	PYY	17	39386857	2	0.078945
rs375171	HDAC5	17	39509996	7	0.688277
rs375171	HDAC5	17	39509996	8	4.940766
rs375171	HDAC5	17	39509996	5	0.741098
rs375171	HDAC5	17	39509996	4	0.995898

rs228757	HDAC5	17	39520411	4	2.915094
rs228757	HDAC5	17	39520411	8	0.453354
rs13726	C17orf53	17	39595087	4	5.883203
rs13726	C17orf53	17	39595087	5	1.074662
rs13726	C17orf53	17	39595087	1	4.831663
SNP17-39609807	ASB16	17	39609807	5	0.242647
SNP17-39609807	ASB16	17	39609807	4	0.237114
SNP17-39609807	ASB16	17	39609807	1	4.311696
SNP17-39609807	ASB16	17	39609807	6	0.078715
rs7217858	ASB16	17	39610053	5	0.348614
rs7217858	ASB16	17	39610053	1	1.020401
rs7217858	ASB16	17	39610053	8	0.94688
rs7217858	ASB16	17	39610053	6	0.916086
rs12937692	ASB16	17	39611956	5	1.540777
rs12937692	ASB16	17	39611956	4	2.271052
rs12937692	ASB16	17	39611956	8	1.465634
rs12937692	ASB16	17	39611956	1	0.935982
SNP17-39623475	TMUB2	17	39623475	5	0.644045
rs5910	ITGA2B	17	39805315	6	0.724982
rs5910	ITGA2B	17	39805315	1	0.718183
rs5910	ITGA2B	17	39805315	4	0.692159
rs5911	ITGA2B	17	39808591	1	0.020577
rs2070605	ADAM11	17	40211080	8	0.593448
rs2070605	ADAM11	17	40211080	3	0.797967
rs8067254	GFAP	17	40339903	1	1.318253
rs2289681	GFAP	17	40344614	3	0.026764
SNP17-40359962	KIF18B	17	40359962	3	0.537614
rs17546822	LOC146909	17	40364496	3	0.349652
rs1055646	C1QL1	17	40392717	7	0.905838
rs1055646	C1QL1	17	40392717	5	0.886738
rs1055646	C1QL1	17	40392717	4	0.571066
rs2239922	NMT1	17	40531432	4	0.724765
rs1989803	NMT1	17	40541018	7	7.405315
rs1989803	NMT1	17	40541018	3	0.578181
rs11651968	C17orf46	17	40688908	5	1.817478
rs11651968	C17orf46	17	40688908	1	4.075995
rs11651968	C17orf46	17	40688908	3	1.805491
rs11651968	C17orf46	17	40688908	4	2.414817
rs11651968	C17orf46	17	40688908	7	1.949315
rs11651968	C17orf46	17	40688908	6	3.540798
rs1047841	MAP3K14	17	40696414	6	0.51884
rs1047833	MAP3K14	17	40697924	5	0.037572
rs1047833	MAP3K14	17	40697924	7	4.674048
rs1047833	MAP3K14	17	40697924	8	3.224248
rs2942168	MGC57346	17	41070633	3	0.571678
rs2942168	MGC57346	17	41070633	4	1.078094
rs2942168	MGC57346	17	41070633	6	4.332281

rs16940665	CRHR1	17	41263677	3	3.892015
rs16940665	CRHR1	17	41263677	6	4.257601
rs878886	CRHR1	17	41268271	6	2.664446
rs878888	CRHR1	17	41268416	6	6.238033
rs878888	CRHR1	17	41268416	3	3.801708
rs878888	CRHR1	17	41268416	4	2.401171
rs4525537	CRHR1	17	41268504	4	4.298423
rs4525537	CRHR1	17	41268504	3	5.139514
rs4525537	CRHR1	17	41268504	6	5.742503
rs4640231	CRHR1	17	41268567	3	3.071154
rs4640231	CRHR1	17	41268567	6	3.22567
rs4640231	CRHR1	17	41268567	4	1.509761
rs12185233	IMP5	17	41279434	3	1.584527
rs12185268	IMP5	17	41279463	3	0.516839
rs11079725	IMP5	17	41279714	3	1.147299
SNP17-41416612	MAPT	17	41416612	3	0.373808
rs10445337	MAPT	17	41423237	6	0.817724
rs10445337	MAPT	17	41423237	3	1.66196
rs1052551	MAPT	17	41424761	6	0.487521
rs1052551	MAPT	17	41424761	3	0.10598
rs1052551	MAPT	17	41424761	4	0.994317
rs1052553	MAPT	17	41429726	6	2.692923
rs1052553	MAPT	17	41429726	4	0.018236
rs1052553	MAPT	17	41429726	3	0.364449
rs17652121	MAPT	17	41429810	4	0.020294
rs17652121	MAPT	17	41429810	3	0.991639
rs11568305	MAPT	17	41429855	1	5.68667
rs1052587	MAPT	17	41458449	4	0.280291
rs1052587	MAPT	17	41458449	3	0.271228
rs1052587	MAPT	17	41458449	6	1.781358
rs17574040	MAPT	17	41458711	4	0.357157
rs17574040	MAPT	17	41458711	3	1.702504
rs17574040	MAPT	17	41458711	6	0.163292
rs16940799	MAPT	17	41458779	6	0.081297
rs16940799	MAPT	17	41458779	4	1.570031
rs16940799	MAPT	17	41458779	3	0.907269
rs2158257	MAPT	17	41460189	3	1.651313
rs2158257	MAPT	17	41460189	6	0.30643
rs17574228	MAPT	17	41460355	6	0.54691
rs17574228	MAPT	17	41460355	4	0.17463
rs17574228	MAPT	17	41460355	3	1.139239
rs7521	MAPT	17	41461242	1	0.97248
rs7521	MAPT	17	41461242	8	0.639255
rs7521	MAPT	17	41461242	2	0.524307
rs7521	MAPT	17	41461242	7	0.470349
rs7521	MAPT	17	41461242	3	0.63316
rs36076725	KIAA1267	17	41466379	4	1.828805

rs36076725	KIAA1267	17	41466379	6	3.413402
rs36076725	KIAA1267	17	41466379	3	2.057864
rs35833914	KIAA1267	17	41466388	3	0.220519
rs35833914	KIAA1267	17	41466388	6	2.227563
rs35833914	KIAA1267	17	41466388	4	0.427929
rs2240758	KIAA1267	17	41604276	8	9.898429
rs12944167	GOSR2	17	42355564	3	1.037172
SNP17-42569622	CDC27	17	42569622	4	4.741595
SNP17-42569622	CDC27	17	42569622	1	5.672499
SNP17-42569622	CDC27	17	42569622	3	5.022777
SNP17-42569622	CDC27	17	42569622	2	4.565713
rs5918	ITGB3	17	42715729	7	3.452383
rs5918	ITGB3	17	42715729	6	1.749655
rs16941864	ITGB3	17	42743240	7	1.931583
rs2317676	ITGB3	17	42743282	7	0.55131
rs2317676	ITGB3	17	42743282	2	0.810787
rs3809865	ITGB3	17	42743585	1	5.272098
rs3809865	ITGB3	17	42743585	7	1.782874
rs3809865	ITGB3	17	42743585	4	0.639451
rs3809865	ITGB3	17	42743585	6	0.4529
rs4968318	C17orf57	17	42806893	4	0.509582
rs4968318	C17orf57	17	42806893	8	1.98744
rs4968318	C17orf57	17	42806893	7	0.305262
rs4968318	C17orf57	17	42806893	1	5.103702
rs4968318	C17orf57	17	42806893	6	1.492664
rs3883318	C17orf57	17	42845250	3	0.76499
rs6503807	TBKBP1	17	43127931	3	1.775359
SNP17-43141620	TBKBP1	17	43141620	2	1.505492
SNP17-43141620	TBKBP1	17	43141620	1	0.229205
SNP17-43141620	TBKBP1	17	43141620	3	1.009893
rs9907142	OSBPL7	17	43240686	2	5.669433
rs2301690	OSBPL7	17	43245706	6	0.328245
rs2301690	OSBPL7	17	43245706	2	0.881133
rs2301690	OSBPL7	17	43245706	3	0.135997
rs2301690	OSBPL7	17	43245706	1	0.374758
rs17700839	OSBPL7	17	43254056	1	0.705216
rs17700839	OSBPL7	17	43254056	6	0.287027
rs17700839	OSBPL7	17	43254056	2	0.501894
rs17700839	OSBPL7	17	43254056	3	0.006224
rs16948568	MRPL10	17	43259419	1	0.51927
rs16948568	MRPL10	17	43259419	3	0.759362
rs16948568	MRPL10	17	43259419	6	0.219507
rs2277693	LRRC46	17	43264182	1	0.664745
rs2277693	LRRC46	17	43264182	6	3.439945
rs2277693	LRRC46	17	43264182	3	0.000617
rs2277693	LRRC46	17	43264182	2	3.02048
rs10428	SCRN2	17	43270085	7	0.181483

rs16948835	SP6	17	43288164	4	1.319637
rs16948835	SP6	17	43288164	1	1.220783
SNP17-43355405	SP2	17	43355405	7	0.0207
SNP17-43355405	SP2	17	43355405	8	6.811776
rs11079804	PNPO	17	43375697	2	5.541673
rs12936731	NFE2L1	17	43483358	8	7.733629
rs12936731	NFE2L1	17	43483358	6	0.14606
GA014554	NFE2L1	17	43492904	6	11.63232
rs4794453	SNX11	17	43539925	6	1.122144
rs4794453	SNX11	17	43539925	8	1.01588
rs7222136	SNX11	17	43554591	8	6.617226
rs7222136	SNX11	17	43554591	6	8.475531
rs2278868	SKAP1	17	43617170	8	0.407138
rs2278868	SKAP1	17	43617170	7	5.290208
rs12939811	HOXB1	17	43962957	7	0.416339
rs7406910	HOXB7	17	44043255	4	1.589092
rs1054072	PRAC	17	44154811	1	1.895998
rs1054072	PRAC	17	44154811	3	0.383674
rs1054072	PRAC	17	44154811	6	2.248726
rs8556	HOXB13	17	44160589	7	0.052863
rs2271890	TTLL6	17	44194911	3	0.211942
rs1422645	CALCOCO2	17	44294657	1	4.736338
rs1422645	CALCOCO2	17	44294657	3	0.315209
rs11434	CALCOCO2	17	44296244	3	0.009293
rs2270576	SNF8	17	44362962	1	5.525173
rs2270576	SNF8	17	44362962	3	0.641018
rs3744085	IGF2BP1	17	44486900	4	4.709804
rs3744085	IGF2BP1	17	44486900	7	0.245414
rs6504593	IGF2BP1	17	44487818	7	0.06351
rs596783	FLJ40194	17	44684704	7	2.42691
rs596783	FLJ40194	17	44684704	5	1.40127
rs596783	FLJ40194	17	44684704	4	2.091037
rs2906100	ZNF652	17	44727763	7	0.665611
rs11079869	ZNF652	17	44728416	5	0.674386
rs11079869	ZNF652	17	44728416	4	0.048665
rs11079869	ZNF652	17	44728416	7	0.443078
rs11466155	NGFR	17	44942999	6	0.465918
rs741072	NGFR	17	44946702	2	3.302836
rs741072	NGFR	17	44946702	1	1.207908
rs741072	NGFR	17	44946702	8	5.844885
rs741072	NGFR	17	44946702	4	0.036138
rs11466173	NGFR	17	44947200	6	0.833556
rs2676793	NXPH3	17	45012187	6	2.826261
rs2676793	NXPH3	17	45012187	4	2.311853
rs2676793	NXPH3	17	45012187	2	1.619241
rs2676793	NXPH3	17	45012187	5	2.5088
rs8186	SLC35B1	17	45133792	6	3.320365

rs1135034	SLC35B1	17	45138662	3	0.131493
rs1135034	SLC35B1	17	45138662	8	6.827728
rs1058564	DLX4	17	45406771	4	7.722125
rs1058564	DLX4	17	45406771	3	0.858721
rs1058564	DLX4	17	45406771	7	2.985608
rs1058564	DLX4	17	45406771	1	4.93739
rs2278163	DLX3	17	45427425	1	1.43593
rs2278163	DLX3	17	45427425	4	2.309384
rs2278163	DLX3	17	45427425	2	1.396114
SNP17-45510424	ITGA3	17	45510424	7	1.566735
rs3744538	ITGA3	17	45521979	7	0.224975
rs3178055	PDK2	17	45529907	7	0.046631
rs3178055	PDK2	17	45529907	2	4.638909
rs3178055	PDK2	17	45529907	5	0.258685
rs1800215	COL1A1	17	45620494	8	2.513106
rs1969513	TMEM92	17	45712299	4	1.189058
rs1969513	TMEM92	17	45712299	2	3.210782
rs2272380	TMEM92	17	45713446	8	4.79407
rs2272380	TMEM92	17	45713446	1	5.442328
SNP17-45788305	XYLT2	17	45788305	1	5.687908
rs6504649	XYLT2	17	45792455	3	0.221341
rs6504649	XYLT2	17	45792455	5	0.63137
rs6504649	XYLT2	17	45792455	7	1.159729
rs6504649	XYLT2	17	45792455	4	1.615645
rs6504649	XYLT2	17	45792455	2	4.84678
rs6504649	XYLT2	17	45792455	8	6.677778
rs7212696	MRPL27	17	45801906	1	5.178028
rs4793651	MRPL27	17	45802124	4	0.353579
rs4793651	MRPL27	17	45802124	1	4.23726
rs4793651	MRPL27	17	45802124	8	4.585001
rs16948970	MRPL27	17	45805535	1	3.286819
SNP17-45807775	EME1	17	45807775	6	2.092723
SNP17-45807775	EME1	17	45807775	5	0.5963
rs2189712	EME1	17	45810984	4	0.677229
rs2189712	EME1	17	45810984	7	0.522925
rs2189712	EME1	17	45810984	1	6.068887
rs2189712	EME1	17	45810984	8	0.130037
rs17714940	EME1	17	45811250	1	5.472209
rs2305998	ACSF2	17	45904790	4	0.852014
rs2305998	ACSF2	17	45904790	7	0.138705
rs2305998	ACSF2	17	45904790	6	0.473974
rs7627	ACSF2	17	45907153	6	2.518203
rs2290862	RSAD1	17	45912325	1	0.501
rs2290862	RSAD1	17	45912325	7	1.335009
rs2290862	RSAD1	17	45912325	4	0.374057
rs3744531	RSAD1	17	45918171	1	4.05035
rs557	RSAD1	17	45918233	6	0.943166

rs557	RSAD1	17	45918233	1	4.941356
rs9890721	MYCBPAP	17	45958502	3	1.09982
rs9890721	MYCBPAP	17	45958502	8	2.90603
rs9890721	MYCBPAP	17	45958502	6	2.553574
rs9890721	MYCBPAP	17	45958502	2	2.930337
rs9890721	MYCBPAP	17	45958502	4	1.630976
rs1064055	MYCBPAP	17	45963716	5	0.165303
rs4148416	ABCC3	17	46108422	1	3.429284
rs11568591	ABCC3	17	46116052	7	0.263219
rs2302285	SPAG9	17	46432038	5	0.068462
rs7222463	MBTD1	17	46624365	3	0.201046
rs7222463	MBTD1	17	46624365	6	1.781169
rs7222463	MBTD1	17	46624365	5	0.698057
rs2318789	UTP18	17	46705801	1	1.065937
rs2318789	UTP18	17	46705801	5	1.061804
rs2318789	UTP18	17	46705801	6	2.158707
rs2318789	UTP18	17	46705801	3	0.636485
rs244300	STXBP4	17	50594239	1	5.63907
rs244300	STXBP4	17	50594239	4	0.731597
rs244300	STXBP4	17	50594239	6	3.297695
rs244298	STXBP4	17	50596386	6	3.585649
rs244298	STXBP4	17	50596386	1	7.586666
rs244298	STXBP4	17	50596386	5	0.7742
rs244298	STXBP4	17	50596386	4	0.202735
rs957724	ANKFN1	17	51805037	3	0.203644
rs957724	ANKFN1	17	51805037	7	0.219412
rs957724	ANKFN1	17	51805037	1	3.118708
rs8069322	ANKFN1	17	51805133	1	3.195054
rs8069322	ANKFN1	17	51805133	3	0.802276
SNP17-5222743	C17orf67	17	52227438	3	0.255252
rs3760158	DGKE	17	52276493	2	5.144587
rs3760158	DGKE	17	52276493	8	1.479443
rs9897759	DGKE	17	52295915	8	0.476725
rs9897759	DGKE	17	52295915	2	5.839307
rs9897759	DGKE	17	52295915	7	0.892287
rs3213728	RNF126P1	17	52478747	5	0.019724
rs3213728	RNF126P1	17	52478747	4	0.283701
rs3213728	RNF126P1	17	52478747	7	0.444637
rs1468753	LOC729417	17	52517324	6	7.250982
rs17761023	AKAP1	17	52537877	5	0.075092
rs17762338	CUEDC1	17	53306036	3	0.071059
rs17762338	CUEDC1	17	53306036	1	6.815725
rs16942650	LOC729460	17	53517798	6	0.982428
rs16942650	LOC729460	17	53517798	7	1.132178
rs16942650	LOC729460	17	53517798	8	1.607573
rs9912728	OR4D2	17	53602453	1	0.998018
rs9894859	OR4D2	17	53603471	5	1.938232

rs12602891	EPX	17	53625120	5	0.202915
rs2240815	EPX	17	53627389	3	2.714604
rs2301870	LPO	17	53687326	3	0.761577
rs2759	MPO	17	53703105	1	1.7733
rs2680689	BZRAP1	17	53741117	3	2.070964
rs9905604	BZRAP1	17	53744630	6	6.361344
rs2070107	SUPT4H1	17	53778200	7	0.808444
rs2680701	RNF43	17	53793300	3	2.597507
rs3803752	HSF5	17	53895266	5	0.053581
rs3744108	MTMR4	17	53940871	7	9.357398
rs17822735	C17orf47	17	53975166	7	1.009966
rs6503867	C17orf47	17	53976358	3	0.753211
rs6503867	C17orf47	17	53976358	5	0.598097
rs7225128	TEX14	17	53991853	5	0.688966
rs7225128	TEX14	17	53991853	8	0.415132
rs7225128	TEX14	17	53991853	3	1.421737
rs9903050	TEX14	17	53991907	3	0.980126
rs34818467	TEX14	17	53998108	5	1.001641
rs3809724	PPM1E	17	54416020	5	0.513426
rs3809723	PPM1E	17	54416760	5	0.164836
rs3809723	PPM1E	17	54416760	8	3.058869
rs6503905	C17orf71	17	54642236	7	0.553947
rs6503905	C17orf71	17	54642236	6	1.005885
rs10853012	YPEL2	17	54833058	4	0.235745
rs10853012	YPEL2	17	54833058	1	0.707785
rs10853012	YPEL2	17	54833058	2	7.825541
rs1057987	TBX2	17	56840337	7	0.475204
rs1057987	TBX2	17	56840337	5	1.411483
rs1057987	TBX2	17	56840337	1	0.869181
rs11871637	C17orf82	17	56845017	8	0.147297
rs11871637	C17orf82	17	56845017	5	0.074536
rs11871637	C17orf82	17	56845017	7	0.745895
rs3744447	TBX4	17	56889769	7	0.133471
rs3744438	TBX4	17	56912382	3	0.561568
rs7218485	TBX4	17	56915245	3	0.144719
rs7213430	BRIP1	17	57114956	1	5.436913
rs7213430	BRIP1	17	57114956	6	0.075936
rs7213430	BRIP1	17	57114956	7	0.476487
rs7213430	BRIP1	17	57114956	4	1.998259
rs4986763	BRIP1	17	57115778	7	0.061877
rs4986763	BRIP1	17	57115778	4	0.401388
rs4986763	BRIP1	17	57115778	1	4.619082
rs12325728	LOC645804	17	57679230	1	3.073544
rs12325728	LOC645804	17	57679230	6	2.087849
rs12325728	LOC645804	17	57679230	4	1.996274
rs8078132	TLK2	17	58037235	3	0.888982
rs8078132	TLK2	17	58037235	4	0.361056

rs8078132	TLK2	17	58037235	5	0.056431
rs2014055	MRC2	17	58095649	3	0.731636
rs2041283	10-Mar	17	58167359	2	1.703437
rs2041283	10-Mar	17	58167359	7	2.021693
rs2041283	10-Mar	17	58167359	3	0.829722
rs2041283	10-Mar	17	58167359	5	1.910884
SNP17-58168005	10-Mar	17	58168005	2	3.427769
SNP17-58168005	10-Mar	17	58168005	5	2.378258
SNP17-58168005	10-Mar	17	58168005	3	0.44434
SNP17-58168005	10-Mar	17	58168005	7	0.915414
rs4298	ACE	17	58910932	7	12.20508
rs4298	ACE	17	58910932	6	6.381345
rs4316	ACE	17	58916041	1	0.484325
rs4316	ACE	17	58916041	5	0.285188
rs4343	ACE	17	58919763	1	3.756113
rs4343	ACE	17	58919763	5	5.09827
rs4343	ACE	17	58919763	6	1.08508
rs4343	ACE	17	58919763	4	0.857161
rs7225568	KCNH6	17	58965155	6	0.610156
rs7225568	KCNH6	17	58965155	1	0.914009
rs7225568	KCNH6	17	58965155	3	0.587748
SNP17-58976784	KCNH6	17	58976784	1	1.060052
rs15637	MAP3K3	17	59125782	7	0.10239
rs15637	MAP3K3	17	59125782	1	0.36235
rs15637	MAP3K3	17	59125782	4	5.769224
rs15637	MAP3K3	17	59125782	2	0.354107
rs15637	MAP3K3	17	59125782	8	0.930729
rs968719	PSMC5	17	59262003	8	4.938644
rs968719	PSMC5	17	59262003	6	5.032647
rs6919	SMARCD2	17	59263217	2	6.107662
rs6919	SMARCD2	17	59263217	6	0.970918
rs7921	CD79B	17	59359991	4	0.01
rs7921	CD79B	17	59359991	2	4.362677
rs7921	CD79B	17	59359991	1	0.273257
rs12603821	CD79B	17	59360164	2	2.558591
rs12603821	CD79B	17	59360164	6	0.028573
rs1051688	CD79B	17	59360165	6	5.308241
rs1051688	CD79B	17	59360165	7	2.679382
rs1051684	CD79B	17	59360229	6	2.78072
rs1051684	CD79B	17	59360229	7	0.227938
rs1051684	CD79B	17	59360229	1	4.879024
rs2058194	SCN4A	17	59374080	7	1.399753
rs2058194	SCN4A	17	59374080	1	1.029986
rs6504191	SCN4A	17	59394800	5	1.985502
rs6504191	SCN4A	17	59394800	2	1.315751
rs196912	ERN1	17	59475212	5	5.91878
rs196912	ERN1	17	59475212	7	0.444072

rs196912	ERN1	17	59475212	4	0.570743
rs6808	PECAM1	17	59754307	6	0.870316
rs6808	PECAM1	17	59754307	8	0.74661
rs6808	PECAM1	17	59754307	1	3.255683
rs6808	PECAM1	17	59754307	7	10.14398
rs6808	PECAM1	17	59754307	5	0.3657
rs2812	PECAM1	17	59754850	1	5.032723
rs2812	PECAM1	17	59754850	6	1.815238
rs2812	PECAM1	17	59754850	7	3.82452
rs2812	PECAM1	17	59754850	5	3.865531
rs2812	PECAM1	17	59754850	8	0.551871
rs2812	PECAM1	17	59754850	4	0.018546
rs1131012	PECAM1	17	59781521	5	0.055505
SNP17-59787297	PECAM1	17	59787297	8	1.112969
SNP17-59787297	PECAM1	17	59787297	7	1.079133
SNP17-59787297	PECAM1	17	59787297	5	1.222771
rs9303474	SMURF2	17	59971771	2	4.126727
rs9303474	SMURF2	17	59971771	6	0.592271
rs7591	AXIN2	17	60955544	5	0.624908
rs7591	AXIN2	17	60955544	7	0.388135
rs7591	AXIN2	17	60955544	3	0.040715
rs7591	AXIN2	17	60955544	4	2.251054
rs2240308	AXIN2	17	60985053	3	0.602818
rs8178822	APOH	17	61655991	3	1.636571
rs7220996	PRKCA	17	62235162	4	2.831062
rs11652480	CACNG5	17	62304014	7	1.083325
rs2286678	CACNG5	17	62311537	3	0.472843
rs11649752	CACNG4	17	62457348	1	5.019163
rs11649752	CACNG4	17	62457348	7	0.679781
rs11649752	CACNG4	17	62457348	5	0.539951
rs11649752	CACNG4	17	62457348	3	1.619246
rs1799938	CACNG1	17	62482766	3	0.065905
rs2684	CACNG1	17	62483067	3	0.062649
rs8080100	HELZ	17	62642504	4	0.398188
rs8080100	HELZ	17	62642504	3	0.083785
rs8080100	HELZ	17	62642504	7	0.47012
rs11079691	PSMD12	17	62793014	6	3.13679
rs11079691	PSMD12	17	62793014	1	4.168725
rs11079691	PSMD12	17	62793014	7	1.612618
rs8866	LOC729822	17	62804441	1	5.149526
rs8866	LOC729822	17	62804441	3	0.678348
rs2291284	NOL11	17	63147987	6	0.452859
rs12945591	LOC727804	17	63714376	7	2.572572
rs12945591	LOC727804	17	63714376	2	3.037448
rs12945591	LOC727804	17	63714376	1	3.593695
rs3760274	LOC727804	17	63714674	5	1.380333
rs3760274	LOC727804	17	63714674	3	0.121909

rs3760274	LOC727804	17	63714674	1	0.962008
rs3760274	LOC727804	17	63714674	2	1.149019
rs3764855	AMZ2	17	63756173	8	6.435171
rs3764855	AMZ2	17	63756173	2	5.892256
rs3764855	AMZ2	17	63756173	1	0.435586
rs3213690	AMZ2	17	63758011	1	0.576756
rs3213690	AMZ2	17	63758011	2	0.620979
rs3744307	SLC16A6	17	63779245	8	0.936794
rs3744307	SLC16A6	17	63779245	3	1.160571
rs35397826	SLC16A6	17	63781677	8	0.067844
rs35397826	SLC16A6	17	63781677	3	1.627982
rs1558876	ARSG	17	63876286	4	2.982106
rs1558877	ARSG	17	63876344	6	0.27483
SNP17-63927952	ARSG	17	63927952	1	4.570214
SNP17-63927952	ARSG	17	63927952	7	0.254906
SNP17-63927952	ARSG	17	63927952	3	0.381749
rs883541	WIPI1	17	63960717	6	0.140689
rs8080306	PRKAR1A	17	64019838	4	3.647845
rs8080306	PRKAR1A	17	64019838	3	0.321297
rs2302234	FAM20A	17	64049834	4	0.389055
rs2302234	FAM20A	17	64049834	6	0.563366
rs2286562	FAM20A	17	64051442	1	3.945822
rs2286562	FAM20A	17	64051442	6	1.307363
rs3826430	ABCA8	17	64440029	3	0.628309
rs11077858	ABCA9	17	64483170	3	0.682869
rs11077858	ABCA9	17	64483170	1	0.324533
rs11077859	ABCA9	17	64483474	3	1.339765
rs11077859	ABCA9	17	64483474	1	1.015795
rs11077860	ABCA9	17	64483493	3	1.01942
rs11077860	ABCA9	17	64483493	1	0.636101
rs2302294	ABCA9	17	64497587	1	0.350802
rs2302291	ABCA9	17	64539855	3	0.677079
SNP17-64621428	ABCA6	17	64621428	5	0.306566
rs4968843	ABCA10	17	64655792	3	0.308209
rs4968843	ABCA10	17	64655792	8	0.50949
rs4968843	ABCA10	17	64655792	1	4.147153
rs2233767	ABCA10	17	64655990	5	2.861985
rs2233767	ABCA10	17	64655990	7	0.652124
rs10491178	ABCA10	17	64661568	5	0.026653
rs11657804	ABCA10	17	64722587	1	0.078179
rs9909216	ABCA10	17	64724018	3	0.596847
rs9909216	ABCA10	17	64724018	1	2.688592
rs9909216	ABCA10	17	64724018	5	0.565504
rs12941264	ABCA10	17	64727307	1	1.502672
rs9302891	ABCA10	17	64736442	4	0.022948
rs15886	ABCA5	17	64754884	5	0.210942
rs15886	ABCA5	17	64754884	8	0.302786

rs6788	KCNJ16	17	65643121	1	0.662315
rs6788	KCNJ16	17	65643121	3	0.0282
rs173135	KCNJ2	17	65683921	3	1.12615
rs173135	KCNJ2	17	65683921	2	4.925683
rs236514	KCNJ2	17	65685326	3	0.229951
rs236514	KCNJ2	17	65685326	7	0.577167
rs236514	KCNJ2	17	65685326	4	3.795695
rs1042678	SOX9	17	67633703	2	0.08463
rs1042678	SOX9	17	67633703	6	8.114104
rs1042678	SOX9	17	67633703	5	1.578749
rs9898179	LOC727973	17	67729080	1	3.55135
rs9898179	LOC727973	17	67729080	3	0.220098
rs9898179	LOC727973	17	67729080	6	3.180427
rs9898179	LOC727973	17	67729080	4	0.370927
rs1126966	SLC39A11	17	68154371	2	1.053154
rs7210080	SSTR2	17	68678697	4	0.597282
rs7210080	SSTR2	17	68678697	2	0.568905
rs7210080	SSTR2	17	68678697	1	1.115606
rs745142	C17orf80	17	68744476	4	0.418698
rs745142	C17orf80	17	68744476	1	6.679134
rs745142	C17orf80	17	68744476	7	1.58361
rs745142	C17orf80	17	68744476	8	0.875594
rs11869253	C17orf80	17	68744725	4	1.234179
rs11869253	C17orf80	17	68744725	1	2.769293
rs11869253	C17orf80	17	68744725	8	0.603921
rs11869253	C17orf80	17	68744725	7	1.055371
rs3195419	SDK2	17	68845849	6	1.754583
rs3195419	SDK2	17	68845849	3	0.771734
rs3195419	SDK2	17	68845849	4	1.752138
rs2270716	SDK2	17	68891682	1	3.25981
rs9302962	SDK2	17	68909765	8	7.26154
rs9302962	SDK2	17	68909765	7	6.890315
rs9302962	SDK2	17	68909765	6	0.531136
rs12386051	SDK2	17	68922486	5	0.904854
rs1105354	SDK2	17	68938265	3	0.199545
rs1105354	SDK2	17	68938265	7	0.63401
rs1105354	SDK2	17	68938265	4	0.135858
rs16977768	C17orf54	17	69257971	8	1.834175
rs2683163	C17orf54	17	69258074	5	0.365055
rs2683163	C17orf54	17	69258074	1	0.258514
rs2683163	C17orf54	17	69258074	3	0.18229
rs2683163	C17orf54	17	69258074	6	1.897715
rs2683163	C17orf54	17	69258074	4	0.551692
rs35682745	TTYH2	17	69751763	5	0.529563
rs3897753	TTYH2	17	69757696	3	0.703142
rs3897753	TTYH2	17	69757696	1	3.050471
rs12600564	TTYH2	17	69760107	5	0.489249

rs9899862	TTYH2	17	69760824	3	0.457784
rs34159194	DNAI2	17	69807561	1	1.231185
rs34159194	DNAI2	17	69807561	8	2.729641
rs1699597	LOC441800	17	70077946	8	1.829095
rs7212069	C17orf77	17	70092709	7	1.216471
rs7212069	C17orf77	17	70092709	3	0.315586
rs493430	C17orf77	17	70099921	8	0.559085
rs545652	C17orf77	17	70100401	7	0.321696
rs12949223	C17orf77	17	70100859	1	0.208151
rs553781	C17orf77	17	70101862	1	4.487847
rs3178300	RAB37	17	70254715	8	0.236408
rs3178300	RAB37	17	70254715	4	0.557409
rs3178300	RAB37	17	70254715	3	0.831187
rs3178300	RAB37	17	70254715	5	0.769466
rs3178300	RAB37	17	70254715	6	3.170358
rs1054131	RAB37	17	70254797	6	0.956602
rs745666	SLC9A3R1	17	70256393	8	0.214641
rs745666	SLC9A3R1	17	70256393	2	0.327054
rs745666	SLC9A3R1	17	70256393	4	2.144486
rs3744215	GRIN2C	17	70350244	3	0.43328
rs3744215	GRIN2C	17	70350244	1	0.482659
rs690514	FDXR	17	70374188	3	0.065076
rs690514	FDXR	17	70374188	6	0.323104
rs1568448	USH1G	17	70423860	5	1.702935
rs1568448	USH1G	17	70423860	6	0.71553
rs1013013	USH1G	17	70424457	8	0.267355
rs1013013	USH1G	17	70424457	4	0.901023
rs690566	USH1G	17	70424667	5	1.287838
rs690566	USH1G	17	70424667	6	0.409067
rs690566	USH1G	17	70424667	3	2.062873
SNP17-70438718	OTOP2	17	70438718	3	0.338765
SNP17-70438718	OTOP2	17	70438718	6	0.574313
rs9890664	OTOP3	17	70449446	4	0.687478
rs9890664	OTOP3	17	70449446	7	0.00654
rs9890664	OTOP3	17	70449446	3	0.530201
rs9890664	OTOP3	17	70449446	5	0.508299
rs8066909	OTOP3	17	70454721	7	0.995161
rs8066909	OTOP3	17	70454721	5	0.909066
rs2307010	C17orf28	17	70460741	3	0.07148
rs3744198	C17orf28	17	70467719	7	0.098882
SNP17-70511266	CDR2L	17	70511266	6	0.187918
rs34496172	ICT1	17	70528084	6	3.772635
rs4789128	LOC643177	17	70582383	8	0.128218
rs4789128	LOC643177	17	70582383	7	0.245194
rs4412997	ARMC7	17	70637369	5	0.397623
rs2291029	NUP85	17	70733034	7	1.162851
rs3178020	GGA3	17	70746423	7	0.710649

rs16967764	MIF4GD	17	70774210	7	1.865863
rs7198	SLC25A19	17	70780853	1	3.54739
rs7198	SLC25A19	17	70780853	3	0.2575
rs7198	SLC25A19	17	70780853	6	0.538943
rs12949173	KIAA0195	17	71005469	3	0.21074
rs12949173	KIAA0195	17	71005469	8	0.265642
rs12949173	KIAA0195	17	71005469	7	0.187951
rs13357	KIAA0195	17	71007287	3	0.516363
rs13357	KIAA0195	17	71007287	1	4.161189
rs13357	KIAA0195	17	71007287	6	0.135699
rs13357	KIAA0195	17	71007287	4	6.434276
rs13357	KIAA0195	17	71007287	5	10.07093
rs13357	KIAA0195	17	71007287	8	9.19813
rs13357	KIAA0195	17	71007287	7	2.39975
rs4789206	CASKIN2	17	71021607	1	1.293402
rs6501818	TSEN54	17	71024884	5	0.218756
rs6501818	TSEN54	17	71024884	2	4.775302
rs9911502	TSEN54	17	71029798	2	0.595706
rs9911502	TSEN54	17	71029798	5	8.275351
rs9911502	TSEN54	17	71029798	1	0.122176
rs6501820	TSEN54	17	71029879	6	1.744084
rs6501820	TSEN54	17	71029879	1	7.925185
SNP17-7106378(	LLGL2	17	71063780	4	0.714289
SNP17-7106378(	LLGL2	17	71063780	8	5.249441
SNP17-7106378(	LLGL2	17	71063780	1	0.480253
SNP17-7106378(	LLGL2	17	71063780	2	0.079386
rs1126939	LLGL2	17	71082313	2	3.052912
rs1126939	LLGL2	17	71082313	1	0.31813
rs1126939	LLGL2	17	71082313	3	0.259481
rs1126939	LLGL2	17	71082313	8	4.396669
rs820242	NULL	17	71097953	1	0.619391
rs820242	NULL	17	71097953	3	0.504067
rs820242	NULL	17	71097953	4	1.161382
rs820242	NULL	17	71097953	6	1.463191
rs820242	NULL	17	71097953	2	2.529341
rs2290455	NULL	17	71098863	5	0.164723
rs936056	NULL	17	71099379	4	0.806882
rs936056	NULL	17	71099379	8	2.724886
rs936056	NULL	17	71099379	5	0.878434
rs3803728	NULL	17	71120734	3	0.623704
rs3803728	NULL	17	71120734	2	1.808352
rs820144	NULL	17	71123418	1	0.739366
rs820144	NULL	17	71123418	2	2.098364
rs820144	NULL	17	71123418	3	1.735776
rs820145	NULL	17	71125570	3	0.470849
rs4788902	RECQL5	17	71137025	1	1.149925
rs4788902	RECQL5	17	71137025	2	0.029825

rs4788902	RECQL5	17	71137025	3	0.347912
rs820196	RECQL5	17	71139134	3	0.287721
rs1661744	RECQL5	17	71158733	2	4.852022
rs1661744	RECQL5	17	71158733	3	0.046254
rs1661745	RECQL5	17	71158734	3	0.750577
rs7342	H3F3B	17	71284451	3	0.428593
rs9916685	UNC13D	17	71335315	6	0.693379
SNP17-71338800	UNC13D	17	71338800	2	2.242012
SNP17-71338800	UNC13D	17	71338800	3	0.008967
SNP17-71338800	UNC13D	17	71338800	4	0.059709
rs3760128	TRIM65	17	71398483	3	0.300284
rs3760128	TRIM65	17	71398483	4	0.738483
rs34974290	TRIM65	17	71399949	6	6.353087
rs34974290	TRIM65	17	71399949	4	4.335911
rs7219918	FBF1	17	71431116	6	0.97171
rs7218738	FBF1	17	71431133	6	1.18868
rs2305913	FBF1	17	71434536	8	0.659775
rs2305913	FBF1	17	71434536	4	0.547762
rs2305913	FBF1	17	71434536	3	1.085396
SNP17-71445830	FBF1	17	71445830	8	4.554217
rs1137582	ACOX1	17	71461135	4	0.323972
rs1137582	ACOX1	17	71461135	3	0.504551
rs1137582	ACOX1	17	71461135	8	0.716231
rs3744032	ACOX1	17	71481430	8	1.486021
rs2069528	CDK3	17	71509069	5	0.043272
rs2069528	CDK3	17	71509069	4	0.695181
rs2257020	LGICZ1	17	71588010	8	1.898078
rs8836	EXOC7	17	71589092	7	11.48109
rs1043149	LGICZ1	17	71589392	8	2.689503
rs881502	RNF157	17	71663961	1	1.760026
rs881502	RNF157	17	71663961	5	1.667253
rs881502	RNF157	17	71663961	8	2.496993
rs881502	RNF157	17	71663961	6	3.231119
SNP17-71675354	RNF157	17	71675354	2	2.985978
rs2289603	RNF157	17	71675416	2	5.275664
rs6501867	LOC643159	17	71733942	6	3.280994
SNP17-71784894	QRICH2	17	71784894	4	0.810403
rs347675	QRICH2	17	71786755	7	0.48423
rs4789273	QRICH2	17	71797677	7	1.341096
rs4789273	QRICH2	17	71797677	6	1.354995
rs4789273	QRICH2	17	71797677	4	5.023185
rs4789273	QRICH2	17	71797677	8	5.698134
rs4789274	QRICH2	17	71797701	4	1.042499
rs4789274	QRICH2	17	71797701	7	2.485577
rs4789274	QRICH2	17	71797701	6	2.545634
rs2279052	QRICH2	17	71798799	2	4.70307
rs2279052	QRICH2	17	71798799	7	0.401727

rs2279052	QRICH2	17	71798799	8	2.443141
rs2279052	QRICH2	17	71798799	4	0.617261
rs2279052	QRICH2	17	71798799	6	4.315507
rs2279054	QRICH2	17	71799189	4	0.277291
rs2279054	QRICH2	17	71799189	6	0.10229
rs2279054	QRICH2	17	71799189	7	1.11636
rs2279054	QRICH2	17	71799189	2	4.967805
rs2279054	QRICH2	17	71799189	8	2.641381
rs3803737	QRICH2	17	71801095	4	0.954219
rs3803737	QRICH2	17	71801095	6	0.006479
rs3803737	QRICH2	17	71801095	7	0.434339
rs3803737	QRICH2	17	71801095	2	3.020784
rs6501880	QRICH2	17	71801300	6	4.535283
rs6501880	QRICH2	17	71801300	8	3.506782
rs6501880	QRICH2	17	71801300	7	0.37234
rs6501880	QRICH2	17	71801300	4	3.702499
rs6501880	QRICH2	17	71801300	2	3.834601
rs6501881	QRICH2	17	71801434	6	1.384941
rs6501881	QRICH2	17	71801434	4	0.76391
rs6501882	QRICH2	17	71801470	8	0.21896
rs6501882	QRICH2	17	71801470	7	0.767129
rs6501882	QRICH2	17	71801470	6	0.193609
rs6501882	QRICH2	17	71801470	4	1.179975
rs6501882	QRICH2	17	71801470	2	3.385725
rs6501883	QRICH2	17	71801503	7	0.41165
rs6501883	QRICH2	17	71801503	6	0.492832
rs6501883	QRICH2	17	71801503	4	0.898585
rs7225131	QRICH2	17	71801692	8	1.837812
rs7225131	QRICH2	17	71801692	6	0.782748
rs7225131	QRICH2	17	71801692	2	3.689428
rs7225131	QRICH2	17	71801692	7	0.213572
rs7225131	QRICH2	17	71801692	4	3.342571
SNP17-7181209	QRICH2	17	71812092	4	0.417861
SNP17-7189316	SPHK1	17	71893162	5	0.793008
rs346801	SPHK1	17	71895273	4	7.657664
rs8150	RHBDF2	17	71978612	8	5.514733
rs8150	RHBDF2	17	71978612	3	0.261831
rs8150	RHBDF2	17	71978612	5	0.002874
SNP17-7198661	RHBDF2	17	71986619	4	2.119173
SNP17-7198661	RHBDF2	17	71986619	1	3.659013
rs8189	CYGB	17	72035386	4	1.593811
rs8189	CYGB	17	72035386	5	2.193909
rs8189	CYGB	17	72035386	6	3.021794
rs34216716	CYGB	17	72038805	7	0.367978
rs8073763	ST6GALNAC1	17	72132893	3	1.25199
rs8073763	ST6GALNAC1	17	72132893	7	3.06481
rs8073763	ST6GALNAC1	17	72132893	6	4.177603

rs8073763	ST6GALNAC1	17	72132893	1	5.087655
rs8073763	ST6GALNAC1	17	72132893	5	0.027653
rs8073763	ST6GALNAC1	17	72132893	8	0.334669
rs237059	SFRS2	17	72242438	7	0.67488
rs3744061	SFRS2	17	72244998	2	1.587023
rs3744061	SFRS2	17	72244998	1	1.16917
rs3744061	SFRS2	17	72244998	6	0.023511
rs3744061	SFRS2	17	72244998	7	1.214595
rs3744061	SFRS2	17	72244998	8	1.051857
rs11077880	MGAT5B	17	72457542	5	0.480509
rs11077880	MGAT5B	17	72457542	8	3.955455
rs8074685	MGAT5B	17	72457881	8	0.658299
rs8074685	MGAT5B	17	72457881	6	0.208991
rs8074685	MGAT5B	17	72457881	2	1.776058
SNP17-7300037	9-Sep	17	73000379	7	1.954478
SNP17-7300630	9-Sep	17	73006300	3	0.013187
rs12449858	TMC6	17	73632913	1	1.487273
rs12449858	TMC6	17	73632913	8	2.797808
rs7208422	TMC8	17	73642170	7	0.032938
rs7208422	TMC8	17	73642170	4	0.224978
rs7208422	TMC8	17	73642170	8	0.493014
rs12452890	TMC8	17	73642665	7	0.830799
rs1079170	SYNGR2	17	73678534	3	1.325365
rs12449983	SYNGR2	17	73680452	1	5.863006
rs1143697	TK1	17	73690343	1	1.153589
rs35330043	TK1	17	73690346	3	0.965227
SNP17-7371256	AFMID	17	73712560	1	4.700935
rs2292642	PGS1	17	73907025	5	0.655654
rs2292642	PGS1	17	73907025	3	1.06713
rs2271613	DNAH17	17	73934692	1	2.686588
SNP17-7394549	DNAH17	17	73945494	6	1.86233
SNP17-7394549	DNAH17	17	73945494	5	0.958722
rs2289751	DNAH17	17	73961100	2	0.704202
rs2289751	DNAH17	17	73961100	1	0.115647
rs2289751	DNAH17	17	73961100	7	0.224919
rs2289754	DNAH17	17	73966813	8	0.450211
rs2289754	DNAH17	17	73966813	7	0.535048
rs691225	DNAH17	17	73974365	2	0.555974
rs691225	DNAH17	17	73974365	1	0.618626
rs691225	DNAH17	17	73974365	8	1.383394
rs691225	DNAH17	17	73974365	3	0.41648
SNP17-7401515	DNAH17	17	74015155	4	1.807254
rs3744793	USP36	17	74328685	5	0.251211
rs3744793	USP36	17	74328685	3	0.25596
rs3744793	USP36	17	74328685	1	0.770508
rs3744793	USP36	17	74328685	2	0.332703
rs3744793	USP36	17	74328685	4	0.135993

rs3744166	LGALS3BP	17	74482474	7	1.109501
rs7213410	C1QTNF1	17	74542172	7	0.523403
rs11077409	C1QTNF1	17	74554313	4	0.533072
rs11077409	C1QTNF1	17	74554313	3	0.473423
rs11769	C1QTNF1	17	74556996	6	7.452996
rs11769	C1QTNF1	17	74556996	8	0.533463
rs11769	C1QTNF1	17	74556996	3	0.130036
rs11769	C1QTNF1	17	74556996	1	0.786834
rs11769	C1QTNF1	17	74556996	4	4.142279
rs11769	C1QTNF1	17	74556996	2	0.238349
rs11769	C1QTNF1	17	74556996	7	5.787015
rs3744181	FLJ21865	17	74585161	3	0.524138
rs3744176	FLJ21865	17	74595214	5	0.995871
rs3744176	FLJ21865	17	74595214	3	6.217795
rs3744176	FLJ21865	17	74595214	8	0.709971
rs3744176	FLJ21865	17	74595214	1	1.73458
rs3744176	FLJ21865	17	74595214	6	1.50228
rs3744176	FLJ21865	17	74595214	4	0.555674
rs3744176	FLJ21865	17	74595214	7	0.072652
rs3744176	FLJ21865	17	74595214	2	1.860267
rs11557	FLJ21865	17	74596249	3	0.883525
rs11557	FLJ21865	17	74596249	1	0.576148
rs7217829	FLJ21865	17	74596270	3	1.75201
rs7217829	FLJ21865	17	74596270	5	0.072815
rs7217829	FLJ21865	17	74596270	6	5.121646
rs7217829	FLJ21865	17	74596270	8	7.609342
rs7217829	FLJ21865	17	74596270	4	0.64933
rs7217829	FLJ21865	17	74596270	2	0.656759
rs7217829	FLJ21865	17	74596270	1	0.426944
rs3751956	CBX2	17	75373079	2	1.262163
rs3751956	CBX2	17	75373079	7	0.690032
rs3751956	CBX2	17	75373079	3	0.77199
rs3751956	CBX2	17	75373079	1	0.600536
rs3751956	CBX2	17	75373079	8	2.661534
rs3751957	CBX2	17	75373286	3	1.114922
rs2289530	CCDC40	17	75636973	3	0.097166
rs2289530	CCDC40	17	75636973	7	0.907219
SNP17-75674484	CCDC40	17	75674484	1	3.049921
SNP17-75674484	CCDC40	17	75674484	5	0.402805
rs12952612	CCDC40	17	75685647	3	0.468483
rs12952612	CCDC40	17	75685647	7	0.83938
rs2304854	CCDC40	17	75688157	2	0.797567
rs2304854	CCDC40	17	75688157	5	1.268781
rs2304854	CCDC40	17	75688157	1	0.394586
rs2304854	CCDC40	17	75688157	3	0.086974
rs2304854	CCDC40	17	75688157	4	0.959233
rs2304853	CCDC40	17	75688184	1	1.56319

rs2304853	CCDC40	17	75688184	5	1.18036
rs2304852	CCDC40	17	75688318	5	0.630932
rs2304852	CCDC40	17	75688318	6	1.423374
rs2304851	CCDC40	17	75688355	6	0.555893
rs2304851	CCDC40	17	75688355	3	0.097639
rs2304851	CCDC40	17	75688355	5	0.307591
rs2304851	CCDC40	17	75688355	1	1.707058
rs1126690	GAA	17	75706000	3	0.080712
rs2229221	GAA	17	75707816	2	5.162844
rs2229221	GAA	17	75707816	1	6.633126
rs4889990	CARD14	17	75772590	2	0.16859
rs4889990	CARD14	17	75772590	1	1.726558
SNP17-75786887	CARD14	17	75786887	5	0.545072
rs11652075	CARD14	17	75793488	1	2.721349
rs11652075	CARD14	17	75793488	7	0.377877
SNP17-75933731	RNF213	17	75933731	8	8.306476
SNP17-75933731	RNF213	17	75933731	2	5.584365
SNP17-75933731	RNF213	17	75933731	3	0.242297
rs4890012	RNF213	17	75933975	2	2.633287
rs4890012	RNF213	17	75933975	8	6.505386
SNP17-75952179	RNF213	17	75952179	3	0.200349
SNP17-75952179	RNF213	17	75952179	7	0.300529
rs34933300	FLJ35220	17	76010329	8	2.267865
SNP17-76025428	FLJ35220	17	76025428	2	0.649068
SNP17-76025428	FLJ35220	17	76025428	1	0.320101
rs4889852	NPTX1	17	76058102	5	0.29704
rs4889852	NPTX1	17	76058102	4	0.532121
rs4077719	NPTX1	17	76058893	5	1.774917
rs4077719	NPTX1	17	76058893	8	2.17859
rs4077719	NPTX1	17	76058893	2	0.131151
rs4077719	NPTX1	17	76058893	1	0.460528
rs4077719	NPTX1	17	76058893	3	1.202813
rs12943620	NPTX1	17	76059253	5	1.335046
rs12943620	NPTX1	17	76059253	6	0.685746
rs12943620	NPTX1	17	76059253	4	0.519622
rs4890026	NPTX1	17	76060297	5	0.30963
rs2589156	KIAA1303	17	76434969	3	0.533172
rs2289759	KIAA1303	17	76468818	6	0.901293
rs2289759	KIAA1303	17	76468818	7	0.270644
rs2289759	KIAA1303	17	76468818	4	4.796139
rs2289759	KIAA1303	17	76468818	3	0.137144
rs1567962	KIAA1303	17	76534153	3	0.043384
rs1567962	KIAA1303	17	76534153	6	0.40984
rs4969391	BAIAP2	17	76704185	4	0.189575
rs4969391	BAIAP2	17	76704185	5	0.004813
rs4969391	BAIAP2	17	76704185	2	0.310177
rs12949063	BAIAP2	17	76704933	1	1.273733

rs12949063	BAIAP2	17	76704933	6	0.205932
rs1542961	AZI1	17	76785171	2	2.084595
rs1542961	AZI1	17	76785171	4	1.324592
SNP17-76840859	SLC38A10	17	76840859	4	0.288164
SNP17-76840859	SLC38A10	17	76840859	1	4.247716
rs3803767	MGC15523	17	76840957	7	0.258995
rs2292184	MGC15523	17	76859397	7	0.404982
rs2292184	MGC15523	17	76859397	6	5.990509
rs2292184	MGC15523	17	76859397	2	1.423282
rs2292184	MGC15523	17	76859397	1	1.287526
rs2292184	MGC15523	17	76859397	4	0.445363
rs9911063	TMEM105	17	76900043	3	1.008098
rs9911063	TMEM105	17	76900043	7	1.189079
rs9911063	TMEM105	17	76900043	4	0.332348
rs9911063	TMEM105	17	76900043	6	0.794279
rs8076437	TMEM105	17	76901658	6	0.011172
rs8076437	TMEM105	17	76901658	3	0.782542
rs8076437	TMEM105	17	76901658	7	1.363668
rs7213444	BAHCC1	17	77028902	3	0.284273
rs7213444	BAHCC1	17	77028902	8	1.386665
SNP17-77029229	BAHCC1	17	77029229	7	0.258059
SNP17-77029229	BAHCC1	17	77029229	6	3.994994
SNP17-77033620	BAHCC1	17	77033620	5	0.113153
SNP17-77039111	BAHCC1	17	77039111	5	1.376449
SNP17-77039111	BAHCC1	17	77039111	3	0.731015
SNP17-77039111	BAHCC1	17	77039111	8	0.061839
rs8746	BAHCC1	17	77044447	6	0.182272
rs8746	BAHCC1	17	77044447	3	0.282628
rs8746	BAHCC1	17	77044447	8	5.223887
rs8077430	C17orf70	17	77121588	6	1.100838
rs8068511	NPLOC4	17	77135234	6	0.29275
rs8068511	NPLOC4	17	77135234	7	0.711947
rs8068511	NPLOC4	17	77135234	3	0.010727
rs8068511	NPLOC4	17	77135234	1	3.723985
rs9894429	NPLOC4	17	77207216	2	2.767806
rs9894429	NPLOC4	17	77207216	1	0.783388
rs9894429	NPLOC4	17	77207216	7	0.557661
rs6565616	TSPAN10	17	77222566	7	1.253062
SNP17-77222802	TSPAN10	17	77222802	2	0.555347
SNP17-77222802	TSPAN10	17	77222802	7	1.269043
SNP17-77222802	TSPAN10	17	77222802	1	0.771536
SNP17-77222946	TSPAN10	17	77222946	8	0.375076
rs8908	PDE6G	17	77228276	1	1.708122
rs8908	PDE6G	17	77228276	6	0.714792
rs8908	PDE6G	17	77228276	7	0.665329
rs8477	PDE6G	17	77228464	1	0.708591
rs8477	PDE6G	17	77228464	6	2.989414

rs8477	PDE6G	17	77228464	7	1.348806
GA033144	CCDC137	17	77247754	1	4.596887
GA033144	CCDC137	17	77247754	8	4.952039
SNP17-77547833	ASPSCR1	17	77547833	2	2.968731
SNP17-77547833	ASPSCR1	17	77547833	8	8.495136
SNP17-77547833	ASPSCR1	17	77547833	6	1.661854
SNP17-77547833	ASPSCR1	17	77547833	5	0.219594
rs4072801	LRRC45	17	77574605	5	0.125947
SNP17-77611191	DUS1L	17	77611191	5	0.217253
rs7406162	CCDC57	17	77679675	7	0.692914
rs7406162	CCDC57	17	77679675	1	3.165313
SNP17-77679678	CCDC57	17	77679678	1	5.174515
SNP17-77679678	CCDC57	17	77679678	7	0.278352
SNP17-77945589	FLJ22222	17	77945589	5	6.001795
SNP17-77945592	FLJ22222	17	77945592	2	0.22333
SNP17-77945592	FLJ22222	17	77945592	8	6.728028
SNP17-78138365	FOXK2	17	78138365	1	4.273769
rs2248257	LOC728694	17	78227280	5	0.224925
rs2248257	LOC728694	17	78227280	8	0.49813
rs2248257	LOC728694	17	78227280	1	4.562581
rs2248257	LOC728694	17	78227280	4	0.343591
rs1056534	FN3K	17	78301890	1	5.878048
rs1056534	FN3K	17	78301890	5	0.323
SNP17-78382757	ZNF750	17	78382757	6	1.050118
SNP17-78382757	ZNF750	17	78382757	3	0.07568
SNP17-78382757	ZNF750	17	78382757	4	0.471259
rs12450046	ZNF750	17	78383677	3	0.282494
rs12450046	ZNF750	17	78383677	6	0.187362
rs12450046	ZNF750	17	78383677	4	2.750701
rs1127986	TBCD	17	78480533	8	1.17234
rs1127986	TBCD	17	78480533	5	1.703815
rs1127986	TBCD	17	78480533	1	2.702886
rs3785522	TBCD	17	78489222	3	0.700349
rs3785522	TBCD	17	78489222	4	0.43509
rs3603	TBCD	17	78494291	1	5.013155
rs3603	TBCD	17	78494291	5	0.666168
rs3603	TBCD	17	78494291	8	4.148763
rs1044661	TBCD	17	78494309	1	0.394528
rs1044661	TBCD	17	78494309	3	0.532133
rs1044661	TBCD	17	78494309	2	1.945964
rs1044661	TBCD	17	78494309	7	0.989451
rs8180	TBCD	17	78494325	4	7.255083
rs8180	TBCD	17	78494325	3	0.843237
rs8180	TBCD	17	78494325	6	5.70252
rs11077950	B3GNTL1	17	78495039	5	0.865406
rs11077950	B3GNTL1	17	78495039	1	4.888919
rs7406119	B3GNTL1	17	78599918	5	1.495186

SNP17-78636464	METRNL	17	78636464	5	1.131216
rs10660	COLEC12	18	309446	5	1.086333
rs12190	COLEC12	18	309935	7	0.82465
rs2305027	COLEC12	18	324742	3	0.136886
rs8098850	COLEC12	18	325099	4	0.501259
rs3826628	COLEC12	18	336821	3	0.379084
SNP18-648001	C18orf56	18	648001	7	0.983708
rs1047363	METTL4	18	2527589	3	0.614972
rs1047363	METTL4	18	2527589	1	5.113475
rs17534687	METTL4	18	2527639	5	5.318946
rs17534687	METTL4	18	2527639	6	2.932258
rs2644189	METTL4	18	2561411	5	4.798528
rs2644189	METTL4	18	2561411	7	0.531533
rs2644189	METTL4	18	2561411	6	1.147911
rs12456560	NDC80	18	2585443	8	7.438756
rs12456560	NDC80	18	2585443	7	0.49231
rs492170	LOC645158	18	2642364	1	1.317485
rs492170	LOC645158	18	2642364	6	1.05401
rs492170	LOC645158	18	2642364	5	0.167959
rs1059282	EMILIN2	18	2903918	2	1.66395
rs1059282	EMILIN2	18	2903918	5	0.099555
rs1985	LPIN2	18	2907223	2	1.793658
rs1985	LPIN2	18	2907223	8	0.140442
rs1985	LPIN2	18	2907223	6	3.835164
rs607549	LPIN2	18	2909606	6	0.679117
rs607549	LPIN2	18	2909606	5	0.310826
rs643507	LOC727896	18	2936291	5	1.461231
rs1042731	MYOM1	18	3056944	5	0.461171
rs1042731	MYOM1	18	3056944	8	0.658979
rs1042731	MYOM1	18	3056944	4	0.057814
rs1042731	MYOM1	18	3056944	6	0.393531
rs2230164	MYOM1	18	3057278	5	0.198603
rs2230164	MYOM1	18	3057278	2	1.663045
rs2230164	MYOM1	18	3057278	4	2.663195
rs2230164	MYOM1	18	3057278	6	0.430566
rs2230164	MYOM1	18	3057278	8	0.275339
rs16944353	MYOM1	18	3065746	6	0.541495
rs16944353	MYOM1	18	3065746	4	1.697993
rs8086066	MYOM1	18	3158816	8	0.403755
rs8086066	MYOM1	18	3158816	4	2.007708
rs8099021	MYOM1	18	3166040	1	1.459013
rs8099021	MYOM1	18	3166040	5	1.222379
rs9964300	MYOM1	18	3205030	4	0.127532
rs1662315	MYOM1	18	3205230	4	0.918015
rs238533	TGIF1	18	3441762	3	0.090524
rs3888654	FLJ35776	18	3587864	5	0.690592
rs3888654	FLJ35776	18	3587864	8	0.056816

rs3786431	DLGAP1	18	3804148	7	0.491723
rs1395063	LOC642597	18	5135609	6	0.995579
rs2789	ZFP161	18	5279887	5	0.229562
rs2789	ZFP161	18	5279887	4	0.024127
rs2789	ZFP161	18	5279887	7	0.681855
rs2789	ZFP161	18	5279887	2	2.295837
rs2789	ZFP161	18	5279887	6	4.77411
rs2789	ZFP161	18	5279887	1	0.023791
rs2789	ZFP161	18	5279887	3	0.139829
SNP18-6291903	L3MBTL4	18	6291903	5	0.122572
rs1116757	ARHGAP28	18	6880434	4	0.398643
rs2163467	ARHGAP28	18	6902317	8	0.581495
rs2163467	ARHGAP28	18	6902317	6	2.183776
rs2163467	ARHGAP28	18	6902317	4	0.359943
rs1431390	ARHGAP28	18	6902332	8	0.70115
rs1431390	ARHGAP28	18	6902332	6	1.160503
rs4798512	ARHGAP28	18	6902944	6	3.987884
rs4798512	ARHGAP28	18	6902944	4	1.985041
rs5029322	ARHGAP28	18	6903016	8	3.200275
rs5029322	ARHGAP28	18	6903016	6	2.391932
rs5029322	ARHGAP28	18	6903016	4	0.168496
rs4398174	ARHGAP28	18	6903378	4	1.358534
rs4398174	ARHGAP28	18	6903378	8	4.626441
rs4486988	ARHGAP28	18	6903420	8	0.243108
rs4486988	ARHGAP28	18	6903420	4	4.955008
rs4486988	ARHGAP28	18	6903420	6	0.639657
rs7236448	ARHGAP28	18	6903534	8	0.651602
rs1052892	ARHGAP28	18	6903716	8	1.672388
rs1052892	ARHGAP28	18	6903716	6	5.15463
rs1052892	ARHGAP28	18	6903716	4	0.083546
rs4393650	ARHGAP28	18	6903841	6	2.21512
rs4393650	ARHGAP28	18	6903841	8	5.107831
rs4393650	ARHGAP28	18	6903841	4	1.021919
rs1431389	ARHGAP28	18	6903975	6	1.594866
rs1431389	ARHGAP28	18	6903975	8	0.088882
rs1431389	ARHGAP28	18	6903975	4	3.698629
rs1431388	ARHGAP28	18	6904033	8	1.486351
rs1431388	ARHGAP28	18	6904033	6	0.23233
rs1431388	ARHGAP28	18	6904033	4	0.004007
rs1431387	ARHGAP28	18	6904054	6	3.473861
rs1431387	ARHGAP28	18	6904054	8	1.387551
rs1431387	ARHGAP28	18	6904054	4	0.244172
rs1060012	ARHGAP28	18	6904458	4	0.321009
rs1060012	ARHGAP28	18	6904458	6	5.80661
rs1060027	ARHGAP28	18	6905639	8	1.178887
rs1060027	ARHGAP28	18	6905639	4	0.141332
rs1060027	ARHGAP28	18	6905639	6	2.283516

rs4797265	FLJ38028	18	6918180	1	0.883682
rs607230	LAMA1	18	6970523	8	0.152943
rs607230	LAMA1	18	6970523	6	4.4404
rs607230	LAMA1	18	6970523	4	3.579344
rs607230	LAMA1	18	6970523	1	0.865347
rs11661974	LAMA1	18	6973194	3	0.545213
SNP18-6976227	LAMA1	18	6976227	1	3.636219
SNP18-6976227	LAMA1	18	6976227	6	0.526321
SNP18-6976227	LAMA1	18	6976227	4	4.752266
rs12961939	LAMA1	18	6987818	8	0.119623
rs12970643	LAMA1	18	6989963	3	0.839968
rs619106	LAMA1	18	7001413	4	0.364372
rs619106	LAMA1	18	7001413	1	2.066227
rs619106	LAMA1	18	7001413	3	1.177176
rs684634	LAMA1	18	7013371	3	1.628289
rs684634	LAMA1	18	7013371	1	3.467047
rs684634	LAMA1	18	7013371	7	1.754372
rs334407	LAMA1	18	7107782	1	1.210444
rs334407	LAMA1	18	7107782	2	2.183626
rs334407	LAMA1	18	7107782	5	1.542941
rs593950	PTPRM	18	8377219	8	3.164151
rs593950	PTPRM	18	8377219	7	1.138108
rs593950	PTPRM	18	8377219	3	0.554519
rs647241	LOC729391	18	8403996	5	1.229102
rs12960076	RAB12	18	8600640	5	0.983141
SNP18-8773835	KIAA0802	18	8773835	1	2.77143
SNP18-8773835	KIAA0802	18	8773835	6	0.130145
rs918272	KIAA0802	18	8774370	5	0.122489
SNP18-8774612	KIAA0802	18	8774612	5	0.42415
SNP18-8774612	KIAA0802	18	8774612	1	1.644766
SNP18-8774612	KIAA0802	18	8774612	3	0.802958
SNP18-8774723	KIAA0802	18	8774723	3	1.755922
SNP18-8774723	KIAA0802	18	8774723	1	0.311701
SNP18-8774723	KIAA0802	18	8774723	7	0.806122
SNP18-8796925	KIAA0802	18	8796925	8	0.613023
rs581894	KIAA0802	18	8815192	8	3.72114
rs581894	KIAA0802	18	8815192	1	3.698655
rs581894	KIAA0802	18	8815192	7	0.094195
rs2242273	KIAA0802	18	8818952	6	0.024986
rs17496551	LOC645573	18	9010334	6	5.523759
rs16954690	LOC645573	18	9010701	6	0.530036
rs16954690	LOC645573	18	9010701	2	2.425291
SNP18-9112611	NDUFV2	18	9112611	3	1.541639
SNP18-9112611	NDUFV2	18	9112611	7	5.502766
SNP18-9112611	NDUFV2	18	9112611	6	2.200387
SNP18-9245784	ANKRD12	18	9245784	3	0.355136
SNP18-9246258	ANKRD12	18	9246258	4	5.002873

SNP18-9248537	ANKRD12	18	9248537	3	0.208087
rs34595349	TWSG1	18	9386524	1	2.828572
rs527575	RAB31	18	9852138	2	1.208637
rs527575	RAB31	18	9852138	6	6.133086
rs527575	RAB31	18	9852138	7	8.344031
rs11664080	TXNDC2	18	9878027	4	0.332203
rs2240905	TXNDC2	18	9878069	3	0.531575
rs29104	VAPA	18	9945753	1	2.271677
rs29104	VAPA	18	9945753	3	0.059584
rs28212	VAPA	18	9949882	3	0.398093
rs28212	VAPA	18	9949882	7	0.674842
rs28212	VAPA	18	9949882	5	0.265043
rs3748415	APCDD1	18	10461732	1	5.17807
rs3748421	FAM38B	18	10681357	8	1.131057
rs662515	MPPE1	18	11876563	7	0.003084
rs662515	MPPE1	18	11876563	2	0.040426
rs662515	MPPE1	18	11876563	6	0.278827
rs662515	MPPE1	18	11876563	3	0.178946
rs4643402	LOC284226	18	12046850	8	2.041277
rs4643402	LOC284226	18	12046850	3	2.363319
rs4643402	LOC284226	18	12046850	4	1.650756
rs12458258	LOC728211	18	12066365	6	4.326846
rs12458258	LOC728211	18	12066365	3	4.027132
rs1986751	DKFZp779B1634	18	12086249	3	5.509267
rs1986751	DKFZp779B1634	18	12086249	4	2.622212
rs1986751	DKFZp779B1634	18	12086249	6	2.208094
rs6505715	DKFZp779B1634	18	12089654	3	0.554795
rs4519391	DKFZp779B1634	18	12105509	4	2.742965
rs4519391	DKFZp779B1634	18	12105509	3	0.293573
rs7244929	CIDEA	18	12267358	3	0.001458
rs7244929	CIDEA	18	12267358	2	5.400581
rs7244929	CIDEA	18	12267358	6	6.430032
rs4797701	CEP76	18	12692610	7	0.653274
rs4797701	CEP76	18	12692610	6	0.20645
rs4797701	CEP76	18	12692610	5	0.185055
rs4797701	CEP76	18	12692610	8	0.988158
rs2282542	CEP192	18	13046682	7	0.009906
rs7228940	CEP192	18	13058109	7	0.322836
SNP18-13085591	CEP192	18	13085591	7	0.162901
rs9965848	LOC729713	18	13251896	5	1.261359
rs9965848	LOC729713	18	13251896	3	1.181388
rs9965848	LOC729713	18	13251896	7	1.417114
rs9965848	LOC729713	18	13251896	1	2.462695
rs1284441	C18orf1	18	13602072	1	3.479814
rs1284441	C18orf1	18	13602072	5	0.735163
rs11659762	RNMT	18	13750954	8	3.647136
rs11659762	RNMT	18	13750954	7	5.402451

rs9303806	LOC390834	18	14382456	6	2.454393
rs11872553	LOC729888	18	17574821	7	0.10374
rs11872553	LOC729888	18	17574821	1	0.005207
rs7449	MIB1	18	17700991	3	0.155762
rs7449	MIB1	18	17700991	6	8.535721
rs1805081	NPC1	18	19394430	5	0.068313
rs1805081	NPC1	18	19394430	4	0.308823
rs1805081	NPC1	18	19394430	3	0.47912
rs1652364	ANKRD29	18	19434361	2	3.114889
rs1652364	ANKRD29	18	19434361	7	0.669015
rs35756007	LAMA3	18	19671024	4	0.199903
rs1154226	LAMA3	18	19735231	5	0.590306
rs1154226	LAMA3	18	19735231	4	0.016445
rs1154226	LAMA3	18	19735231	8	7.278048
rs1154226	LAMA3	18	19735231	3	0.132969
rs1154232	LAMA3	18	19765032	3	0.695743
rs1154232	LAMA3	18	19765032	1	1.270413
rs1154232	LAMA3	18	19765032	4	0.388667
rs1131521	LAMA3	18	19767766	5	4.177268
rs1131521	LAMA3	18	19767766	1	3.976524
rs1131521	LAMA3	18	19767766	3	0.331856
rs12327412	C18orf17	18	19826835	5	1.388308
rs12327412	C18orf17	18	19826835	4	0.693507
rs12327412	C18orf17	18	19826835	3	0.208163
rs1049683	CABYR	18	19990931	1	5.750111
rs582234	IMPACT	18	20274465	3	0.010748
SNP18-20310764	HRH4	18	20310764	3	0.205436
SNP18-20310764	HRH4	18	20310764	7	0.560104
SNP18-20310764	HRH4	18	20310764	1	1.904595
rs11662595	HRH4	18	20310968	3	0.101788
rs1421126	HRH4	18	20311736	5	1.118417
rs12457139	LOC390843	18	20356499	7	2.064759
rs11083084	LOC729950	18	20494393	7	1.400799
rs11083084	LOC729950	18	20494393	2	3.447459
rs11083084	LOC729950	18	20494393	3	1.160584
rs1620992	LOC729950	18	20494862	6	0.472427
rs1620992	LOC729950	18	20494862	2	4.755619
rs1620992	LOC729950	18	20494862	5	3.409425
rs1620992	LOC729950	18	20494862	4	1.572901
rs1620992	LOC729950	18	20494862	8	0.017466
rs2069310	PSMA8	18	22026571	3	0.105612
SNP18-22126233	TAF4B	18	22126233	2	2.896929
SNP18-22126233	TAF4B	18	22126233	1	0.450503
rs1677016	TAF4B	18	22127461	8	0.395298
rs1677016	TAF4B	18	22127461	1	0.971416
rs1677016	TAF4B	18	22127461	2	3.823749
rs546234	LOC728599	18	22433854	3	0.618026

rs546234	LOC728599	18	22433854	1	1.142934
rs546234	LOC728599	18	22433854	5	0.280221
rs546234	LOC728599	18	22433854	7	1.467601
rs546234	LOC728599	18	22433854	4	0.408592
rs2438413	LOC728599	18	22434687	6	4.542129
rs2438413	LOC728599	18	22434687	7	4.378679
rs567555	LOC728599	18	22434888	7	4.540007
rs567555	LOC728599	18	22434888	3	4.52214
rs567555	LOC728599	18	22434888	6	3.284772
rs8096168	hCG_38480	18	22523384	5	0.434976
rs8096168	hCG_38480	18	22523384	4	1.604768
rs8096168	hCG_38480	18	22523384	6	1.725583
rs8096168	hCG_38480	18	22523384	2	2.62313
rs8096168	hCG_38480	18	22523384	7	1.142009
rs1058427	AQP4	18	22689093	4	0.328371
rs1058427	AQP4	18	22689093	3	1.63104
rs1058424	AQP4	18	22689543	4	3.890258
rs335929	AQP4	18	22689585	1	0.255271
rs162008	AQP4	18	22699690	1	0.994958
rs163057	C18orf16	18	22768586	5	0.290088
rs163057	C18orf16	18	22768586	1	0.804739
rs163056	C18orf16	18	22768712	5	0.32068
rs163056	C18orf16	18	22768712	1	3.023594
rs16942974	C18orf16	18	22768781	1	2.352819
rs11564422	CDH2	18	23786078	3	0.96119
rs17656284	DSC3	18	26827165	5	1.192642
rs7244822	DSC1	18	26964020	3	0.888249
rs985862	DSC1	18	26964430	3	0.075061
SNP18-26967992	DSC1	18	26967992	4	0.897285
rs36081634	DSC1	18	26976131	1	1.193379
rs2249810	DSC1	18	26988747	8	0.129331
rs2249810	DSC1	18	26988747	1	3.92641
rs1426310	DSG1	18	27152292	5	0.551676
rs12967407	DSG1	18	27167597	8	0.929918
rs12967407	DSG1	18	27167597	6	0.077817
rs12967407	DSG1	18	27167597	5	0.311599
rs12967407	DSG1	18	27167597	1	1.902485
rs12967407	DSG1	18	27167597	3	0.579118
rs8091117	DSG1	18	27173792	8	3.555961
rs8091117	DSG1	18	27173792	6	0.192208
SNP18-27173909	DSG1	18	27173909	8	5.824623
SNP18-27173909	DSG1	18	27173909	6	0.832359
rs16959856	DSG4	18	27222369	2	2.603053
rs16959856	DSG4	18	27222369	8	4.430922
rs16959856	DSG4	18	27222369	3	0.745415
rs9956865	DSG4	18	27222957	8	1.904417
rs9956865	DSG4	18	27222957	2	4.111501

rs9956865	DSG4	18	27222957	3	0.475088
rs4799570	DSG4	18	27240331	7	0.164774
rs4799570	DSG4	18	27240331	3	1.135146
rs2278792	DSG2	18	27376797	5	0.521449
rs2278792	DSG2	18	27376797	6	1.256255
rs17662563	RNF125	18	27852807	7	0.614538
rs17662563	RNF125	18	27852807	1	3.214934
SNP18-27879683	RNF125	18	27879683	3	0.291625
rs4799640	MEP1B	18	28042105	8	0.239123
rs4799640	MEP1B	18	28042105	3	1.229558
rs4799640	MEP1B	18	28042105	1	0.910475
rs173032	MEP1B	18	28049074	3	0.473252
rs173032	MEP1B	18	28049074	8	0.340173
rs173032	MEP1B	18	28049074	1	0.230604
rs233223	MEP1B	18	28049099	8	0.136768
rs233223	MEP1B	18	28049099	2	1.633839
rs233223	MEP1B	18	28049099	1	0.706938
rs233223	MEP1B	18	28049099	4	1.893693
rs616114	MEP1B	18	28051919	4	0.004025
rs616114	MEP1B	18	28051919	3	0.941659
rs616114	MEP1B	18	28051919	1	0.935739
rs616114	MEP1B	18	28051919	8	0.225937
rs2282632	ASXL3	18	29574227	5	0.307463
rs2282632	ASXL3	18	29574227	1	0.33842
rs2282632	ASXL3	18	29574227	2	2.524018
rs2282632	ASXL3	18	29574227	7	0.632759
rs2282632	ASXL3	18	29574227	4	3.19408
rs7243541	ASXL3	18	29581294	6	0.247283
rs3810021	MAPRE2	18	30975076	7	1.170949
rs1050377	MAPRE2	18	30975400	7	0.216121
SNP18-31141538	ZNF271	18	31141538	1	2.978716
rs9963614	ZNF271	18	31141931	7	4.0674
rs10502647	ZNF396	18	31201178	6	0.759949
rs2298716	C18orf21	18	31806596	3	0.396001
rs2298716	C18orf21	18	31806596	8	3.514089
rs2298716	C18orf21	18	31806596	5	0.502869
SNP18-31811464	C18orf21	18	31811464	3	0.27433
SNP18-31811464	C18orf21	18	31811464	5	1.166249
rs8299	ELP2	18	32004044	3	0.22414
rs3744900	MOCOS	18	32033703	7	0.258079
rs2017027	KIAA1328	18	33059192	7	1.582886
rs2017027	KIAA1328	18	33059192	5	0.151447
SNP18-33104844	BRUNOL4	18	33104844	5	0.854318
rs1804730	RIT2	18	38577495	3	1.200162
rs11082414	SETBP1	18	40783994	7	0.422009
rs1064204	SETBP1	18	40786691	1	4.952072
rs2156609	SLC14A2	18	41500999	4	0.427335

rs2156609	SLC14A2	18	41500999	3	0.017319
rs2156609	SLC14A2	18	41500999	6	0.379121
rs2156609	SLC14A2	18	41500999	7	0.269841
rs3745009	SLC14A2	18	41516357	7	1.879758
rs3745009	SLC14A2	18	41516357	5	0.610503
rs3745009	SLC14A2	18	41516357	4	0.700696
rs2298718	SLC14A1	18	41570536	1	1.318574
rs1058396	SLC14A1	18	41573517	1	3.328363
rs1058396	SLC14A1	18	41573517	8	3.410656
rs1058396	SLC14A1	18	41573517	7	1.384183
rs9954521	SLC14A1	18	41584894	3	0.802091
SNP18-41674175	SIGLEC15	18	41674175	4	0.344092
SNP18-41674175	SIGLEC15	18	41674175	3	0.933527
rs500899	SIGLEC15	18	41676278	4	0.03736
rs500899	SIGLEC15	18	41676278	3	1.609804
rs1055066	KIAA1632	18	41681621	3	0.890532
rs1055066	KIAA1632	18	41681621	8	0.260252
rs1055066	KIAA1632	18	41681621	4	0.488297
rs507653	KIAA1632	18	41686290	3	0.062752
rs507653	KIAA1632	18	41686290	4	3.782138
rs507653	KIAA1632	18	41686290	8	5.401717
SNP18-41788822	KIAA1632	18	41788822	3	0.539829
rs7229227	PSTPIP2	18	41818294	1	5.647152
rs1438388	PSTPIP2	18	41818925	1	7.396822
SNP18-42087699	C18orf25	18	42087699	7	0.610575
SNP18-42087699	C18orf25	18	42087699	4	0.489935
SNP18-42087699	C18orf25	18	42087699	8	1.441717
rs9965681	RNF165	18	42292441	1	0.868748
rs9965681	RNF165	18	42292441	7	0.266883
rs9965681	RNF165	18	42292441	4	1.977967
rs12968582	RNF165	18	42292555	1	2.174995
rs12968582	RNF165	18	42292555	7	0.399324
rs12968582	RNF165	18	42292555	4	0.229436
rs17766830	RNF165	18	42294658	7	5.823904
rs17766830	RNF165	18	42294658	2	2.913888
rs17766830	RNF165	18	42294658	3	1.152075
rs12606417	LOXHD1	18	42358435	5	1.632163
rs674948	LOXHD1	18	42435594	2	1.427044
rs7233515	KATNAL2	18	42839953	5	0.999603
rs7233515	KATNAL2	18	42839953	2	4.235729
rs2289130	KATNAL2	18	42880628	4	0.841361
rs2289130	KATNAL2	18	42880628	5	3.350734
rs2277712	KIAA0427	18	44417047	4	0.013562
rs2276170	MYO5B	18	45623756	3	0.928003
rs1942418	MYO5B	18	45637241	2	5.65479
rs1942418	MYO5B	18	45637241	3	0.138899
SNP18-45820645	MYO5B	18	45820645	6	1.165625

SNP18-45820645	MYO5B	18	45820645	4	0.819223
SNP18-45820645	MYO5B	18	45820645	5	0.357828
SNP18-45820645	MYO5B	18	45820645	2	2.980174
SNP18-46031242	CCDC11	18	46031242	4	0.791742
SNP18-46031242	CCDC11	18	46031242	6	0.516881
SNP18-46031242	CCDC11	18	46031242	7	0.395841
SNP18-46031242	CCDC11	18	46031242	3	0.233248
rs140686	MBD1	18	46057352	4	0.718299
rs7228084	CXXC1	18	46064349	7	6.643759
rs11663953	C18orf24	18	46155469	8	1.906583
rs6507992	C18orf24	18	46162554	8	5.189403
rs6507992	C18orf24	18	46162554	1	0.853637
rs6507992	C18orf24	18	46162554	2	0.52373
rs6507992	C18orf24	18	46162554	7	0.944106
rs6507992	C18orf24	18	46162554	5	0.159971
rs6507992	C18orf24	18	46162554	3	1.062387
rs10460085	C18orf24	18	46172637	6	3.369437
rs10460085	C18orf24	18	46172637	5	3.718624
rs10460085	C18orf24	18	46172637	7	0.782177
rs10460085	C18orf24	18	46172637	3	1.515775
rs10460085	C18orf24	18	46172637	1	1.077456
SNP18-46444438	MAPK4	18	46444438	8	7.188244
SNP18-46444438	MAPK4	18	46444438	2	2.887026
rs11873058	MRO	18	46578885	1	1.641525
rs2276186	MRO	18	46581813	8	0.886535
rs2276186	MRO	18	46581813	7	1.107149
rs2276186	MRO	18	46581813	5	0.341213
rs2586776	MRO	18	46585522	5	0.765549
rs2849233	MRO	18	46585551	4	0.763575
rs2849233	MRO	18	46585551	8	0.958846
rs2849233	MRO	18	46585551	3	0.307323
rs2849233	MRO	18	46585551	5	0.373782
SNP18-46589771	MRO	18	46589771	5	0.266633
SNP18-46589771	MRO	18	46589771	2	0.3243
rs1259938	MBD2	18	49934755	2	4.763519
rs1259938	MBD2	18	49934755	4	0.295808
rs1259938	MBD2	18	49934755	7	0.760237
rs1259938	MBD2	18	49934755	5	2.974173
rs17470798	STARD6	18	50112195	1	2.50034
rs3753054	C18orf54	18	50158839	2	4.882534
rs1061830	C18orf54	18	50162065	2	3.37545
rs9947055	C18orf26	18	50416306	5	0.990187
rs9947055	C18orf26	18	50416306	1	1.476826
rs9947055	C18orf26	18	50416306	4	0.250942
rs1944362	CCDC68	18	50721343	1	1.071842
rs1944362	CCDC68	18	50721343	5	1.186707
rs4801109	CCDC68	18	50722432	6	0.594024

SNP18-50755081	CCDC68	18	50755081	1	1.34099
rs1017253	FLJ45743	18	51843614	1	3.692426
rs12606608	FLJ45743	18	52006549	3	2.942596
rs12606608	FLJ45743	18	52006549	2	2.883066
rs681176	TXNL1	18	52456755	4	0.295664
rs681176	TXNL1	18	52456755	1	0.252352
rs681176	TXNL1	18	52456755	2	0.189204
rs2302845	WDR7	18	52599776	4	0.607664
rs2302845	WDR7	18	52599776	1	0.50522
rs3745072	ONECUT2	18	53295033	3	0.286674
rs3745071	ONECUT2	18	53295043	3	0.903129
rs13016	ALPK2	18	54299761	3	1.536842
rs3809983	ALPK2	18	54353748	8	0.624539
rs3809982	ALPK2	18	54354054	8	0.017922
rs3809977	ALPK2	18	54354878	7	0.701448
SNP18-5439858(	ALPK2	18	54398580	1	2.023812
SNP18-5451880:	MALT1	18	54518803	4	0.728489
SNP18-55091287	RAX	18	55091287	1	2.285005
SNP18-55091287	RAX	18	55091287	8	2.09846
SNP18-55091287	RAX	18	55091287	3	2.22922
rs10871768	CPLX4	18	55114099	1	0.928439
rs7243416	CPLX4	18	55114252	2	3.074495
rs7243416	CPLX4	18	55114252	1	0.541057
rs7243416	CPLX4	18	55114252	8	0.203869
rs7243416	CPLX4	18	55114252	4	1.061255
rs4940456	CPLX4	18	55114722	2	0.527403
rs4940456	CPLX4	18	55114722	1	1.151171
rs9954602	CCBE1	18	55253306	6	1.86007
rs1893515	LOC728115	18	55835408	3	3.103731
rs12955810	hCG_1659830	18	56613006	3	0.329221
rs12964819	CDH20	18	57317521	4	0.680332
rs12964819	CDH20	18	57317521	5	2.185072
rs12964819	CDH20	18	57317521	2	1.515647
rs4362478	RNF152	18	57634290	3	0.509064
SNP18-5793137:	PIGN	18	57931373	8	0.354722
SNP18-5793137:	PIGN	18	57931373	1	4.777276
rs13381627	PIGN	18	57932780	1	1.806295
rs13381627	PIGN	18	57932780	3	0.679551
rs34227891	PIGN	18	57961543	3	0.583465
rs34227891	PIGN	18	57961543	6	0.360192
rs34227891	PIGN	18	57961543	4	1.022357
rs34227891	PIGN	18	57961543	1	4.686385
rs35211496	TNFRSF11A	18	58172741	4	0.690416
rs35211496	TNFRSF11A	18	58172741	3	0.466346
rs35211496	TNFRSF11A	18	58172741	2	0.417803
rs35211496	TNFRSF11A	18	58172741	7	0.274328
rs35211496	TNFRSF11A	18	58172741	1	0.648418

rs1805034	TNFRSF11A	18	58178221	8	4.916459
rs1805034	TNFRSF11A	18	58178221	1	4.043417
rs1805034	TNFRSF11A	18	58178221	7	0.407673
rs1805034	TNFRSF11A	18	58178221	5	0.352689
rs1805034	TNFRSF11A	18	58178221	4	0.219611
rs1805034	TNFRSF11A	18	58178221	6	2.273957
rs8092336	TNFRSF11A	18	58187063	8	1.998439
rs12956093	ZCCHC2	18	58382831	3	0.66134
rs624821	PHLPP	18	58713278	3	0.649619
rs624821	PHLPP	18	58713278	5	4.979534
rs4987855	BCL2	18	58944529	8	1.658637
rs4987853	BCL2	18	58944635	5	0.222321
rs4987853	BCL2	18	58944635	4	0.702885
rs4987852	BCL2	18	58944901	4	1.091603
rs4987851	BCL2	18	58945239	7	1.214039
rs1564483	BCL2	18	58945634	3	0.302489
rs1564483	BCL2	18	58945634	5	1.131265
rs1016860	BCL2	18	58946054	5	0.136484
rs1016860	BCL2	18	58946054	4	0.304482
rs4987845	BCL2	18	58946168	8	4.725687
rs4987843	BCL2	18	58946521	5	3.710776
rs4987843	BCL2	18	58946521	4	0.532728
rs1801018	BCL2	18	59136859	5	0.637035
rs1801018	BCL2	18	59136859	1	4.631974
rs1801018	BCL2	18	59136859	3	0.839529
rs1801018	BCL2	18	59136859	6	4.009504
rs1395266	SERPINB11	18	59541312	7	1.04861
rs953695	SERPINB11	18	59541550	7	0.561848
rs17781440	SERPINB7	18	59593890	5	0.034313
rs17781440	SERPINB7	18	59593890	7	0.863552
rs6098	SERPINB2	18	59715374	7	0.642627
rs6098	SERPINB2	18	59715374	5	1.423592
rs6102	SERPINB2	18	59721450	7	0.194159
rs6103	SERPINB2	18	59721483	7	0.675271
rs6103	SERPINB2	18	59721483	5	0.957242
rs6104	SERPINB2	18	59721509	5	0.992158
rs6104	SERPINB2	18	59721509	7	0.166226
rs8097425	SERPINB10	18	59733847	5	1.586586
rs8097425	SERPINB10	18	59733847	7	1.103889
rs922210	C18orf20	18	59967166	5	0.902196
rs922210	C18orf20	18	59967166	3	0.381531
rs922210	C18orf20	18	59967166	7	0.353428
SNP18-62362231	CDH19	18	62362231	1	4.108923
SNP18-62362231	CDH19	18	62362231	8	0.334305
SNP18-62362231	CDH19	18	62362231	5	0.777313
rs7227616	DSEL	18	63325804	1	0.345954
rs11151371	DSEL	18	63326628	8	6.521955

rs17077389	DSEL	18	63326692	6	0.158833
rs2187094	CCDC102B	18	64664595	1	2.394446
rs2187094	CCDC102B	18	64664595	6	0.615249
SNP18-64829172	CCDC102B	18	64829172	7	0.717336
rs727088	CD226	18	65681419	1	0.335111
rs763361	CD226	18	65682622	1	0.150095
rs763361	CD226	18	65682622	3	1.060124
rs3911730	RTTN	18	66022323	7	0.207961
rs2231562	SOCS6	18	66143259	3	7.97497
rs7238987	CYB5A	18	70079130	8	0.574175
rs2278156	C18orf51	18	70254113	5	0.095557
rs2278156	C18orf51	18	70254113	2	3.589379
rs2278156	C18orf51	18	70254113	6	2.436137
rs1046406	C18orf51	18	70254177	6	2.164807
rs1046406	C18orf51	18	70254177	7	1.340383
rs1046406	C18orf51	18	70254177	2	4.254221
rs2278155	C18orf51	18	70254626	8	3.992198
rs2278155	C18orf51	18	70254626	6	2.369541
rs3764509	CNDP2	18	70318104	2	4.854691
rs3764509	CNDP2	18	70318104	7	5.109633
rs3764509	CNDP2	18	70318104	6	4.024351
rs3764509	CNDP2	18	70318104	4	0.407157
rs3764509	CNDP2	18	70318104	1	0.20401
rs3764509	CNDP2	18	70318104	3	0.467545
rs3764509	CNDP2	18	70318104	8	0.61055
rs2278161	CNDP2	18	70327063	8	1.451874
rs2278161	CNDP2	18	70327063	7	0.379919
rs2278161	CNDP2	18	70327063	4	0.012117
rs2278161	CNDP2	18	70327063	6	2.893558
rs2278161	CNDP2	18	70327063	1	0.873266
rs11151964	CNDP1	18	70352898	3	0.520195
rs12960862	CNDP1	18	70385615	6	0.767559
rs12960862	CNDP1	18	70385615	4	1.256645
rs12960862	CNDP1	18	70385615	2	3.553653
rs12960862	CNDP1	18	70385615	5	1.916121
SNP18-70389452	CNDP1	18	70389452	2	4.601454
rs2887	CNDP1	18	70403117	4	1.18855
rs2887	CNDP1	18	70403117	3	0.245778
rs632157	ZADH2	18	71040536	3	0.037821
rs632157	ZADH2	18	71040536	7	0.005878
rs3744908	TSHZ1	18	71126992	6	7.283036
rs3744908	TSHZ1	18	71126992	5	0.654132
SNP18-71128347	TSHZ1	18	71128347	8	5.94596
SNP18-71128347	TSHZ1	18	71128347	5	0.454205
SNP18-71128347	TSHZ1	18	71128347	2	6.788959
SNP18-71128347	TSHZ1	18	71128347	6	0.632569
rs11660753	C18orf62	18	71251586	1	0.706297

rs17059302	ZNF516	18	72220706	8	0.398116
rs3813102	FLJ44313	18	72336563	8	2.038264
rs3813102	FLJ44313	18	72336563	6	1.993019
rs3813102	FLJ44313	18	72336563	5	1.022945
rs470403	ZNF236	18	72800756	1	0.422653
rs470403	ZNF236	18	72800756	3	0.603264
rs470403	ZNF236	18	72800756	6	2.764913
rs470403	ZNF236	18	72800756	2	1.367714
rs17026	MBP	18	72819967	2	4.491949
rs17026	MBP	18	72819967	3	0.472517
rs17026	MBP	18	72819967	6	4.673988
rs7229288	MBP	18	72820440	6	6.074424
rs7229288	MBP	18	72820440	3	0.535146
rs7229288	MBP	18	72820440	1	6.091284
rs7229288	MBP	18	72820440	8	6.832454
rs9199	MBP	18	72821324	1	0.741537
rs9199	MBP	18	72821324	6	0.217139
rs9199	MBP	18	72821324	3	1.133566
rs9199	MBP	18	72821324	2	2.6917
rs2282557	MBP	18	72854154	6	5.2851
rs470871	MBP	18	72854336	5	0.446184
rs7236968	MBP	18	72856491	6	4.628196
rs5374	GALR1	18	73091633	3	0.353035
rs11664381	ATP9B	18	75168171	8	0.624672
rs35099556	NFATC1	18	75328542	6	3.362105
rs599554	CTDP1	18	75574074	1	3.27865
rs2279103	CTDP1	18	75574115	1	4.666087
rs1134231	LOC440498	18	75831795	3	0.961441
rs1134231	LOC440498	18	75831795	8	1.439454
rs1134231	LOC440498	18	75831795	5	2.912496
rs1134231	LOC440498	18	75831795	4	1.403013
SNP18-75995461	ADNP2	18	75995461	5	0.158035
rs3744878	ADNP2	18	75996183	4	0.972277
rs10407115	PPAP2C	19	233753	5	1.225708
rs3764656	THEG	19	313283	3	0.267084
rs10422863	THEG	19	318089	1	2.752152
rs2241407	FAM148C	19	356929	8	0.882055
rs2241407	FAM148C	19	356929	1	0.603157
rs1052882	C19orf19	19	414528	2	0.280551
rs1052882	C19orf19	19	414528	3	0.070321
rs1052882	C19orf19	19	414528	7	0.834916
rs1052882	C19orf19	19	414528	1	1.260759
rs28989772	BSG	19	522397	1	5.46075
rs28989772	BSG	19	522397	8	6.547143
rs6758	BSG	19	534460	2	4.596658
rs1054786	HCN2	19	564898	6	0.395622
rs1054786	HCN2	19	564898	8	4.287092

rs7249518	FLJ45684	19	597900	5	0.687015
rs2074460	FLJ45684	19	598039	7	0.807831
rs2074460	FLJ45684	19	598039	4	0.055898
rs757331	FLJ45684	19	598251	5	2.030537
rs2285751	RNF126	19	602852	8	5.461548
rs13730	FSTL3	19	633010	1	5.249817
rs13730	FSTL3	19	633010	7	0.173376
rs4375794	PRSSL1	19	642909	1	0.44327
rs1050457	PALM	19	682144	3	0.055535
rs1050457	PALM	19	682144	7	0.361205
rs1050457	PALM	19	682144	1	2.51924
SNP19-708752	C19orf21	19	708752	2	4.345933
rs2285696	C19orf21	19	714952	8	7.075985
rs595844	AZU1	19	781854	7	0.343267
rs595844	AZU1	19	781854	5	0.107337
rs351111	PRTN3	19	795020	3	1.308582
rs351111	PRTN3	19	795020	5	0.630536
rs2240154	GRIN3B	19	954172	2	1.917151
SNP19-955740	GRIN3B	19	955740	1	1.818523
rs4806908	GRIN3B	19	955823	5	1.710197
rs4806908	GRIN3B	19	955823	6	0.603305
rs12973948	GRIN3B	19	956531	6	0.282128
rs12973948	GRIN3B	19	956531	5	0.448736
SNP19-960585	GRIN3B	19	960585	1	3.650256
SNP19-960585	GRIN3B	19	960585	3	0.476148
rs4807440	CNN2	19	977477	1	2.630785
rs3752232	ABCA7	19	994748	4	3.180159
rs3752232	ABCA7	19	994748	3	1.192901
SNP19-996173	ABCA7	19	996173	4	0.146737
SNP19-996173	ABCA7	19	996173	3	0.76519
rs3752237	ABCA7	19	998161	1	1.624332
rs3752237	ABCA7	19	998161	7	0.202838
rs3752237	ABCA7	19	998161	4	1.22289
SNP19-998537	ABCA7	19	998537	3	0.097811
SNP19-998537	ABCA7	19	998537	4	3.945299
rs9282560	ABCA7	19	999982	1	4.878987
rs3764652	ABCA7	19	1003005	2	0.153656
rs3764652	ABCA7	19	1003005	3	0.441092
rs3764652	ABCA7	19	1003005	1	1.447787
rs3745842	ABCA7	19	1006191	2	0.039307
rs3745842	ABCA7	19	1006191	3	0.244668
rs3745842	ABCA7	19	1006191	1	1.022229
rs3752246	ABCA7	19	1007492	5	0.647318
rs3752246	ABCA7	19	1007492	2	5.51163
rs1801284	HMHA1	19	1019738	1	1.231721
rs1801284	HMHA1	19	1019738	2	0.423712
rs2074442	HMHA1	19	1025000	5	0.025389

SNP19-1030959	HMHA1	19	1030959	6	0.099874
SNP19-1030959	HMHA1	19	1030959	2	3.200631
rs10404947	HMHA1	19	1032617	5	0.14421
rs10404947	HMHA1	19	1032617	1	1.365421
rs713041	GPX4	19	1057615	2	3.358946
rs713041	GPX4	19	1057615	7	0.012287
rs713041	GPX4	19	1057615	4	1.366284
rs713041	GPX4	19	1057615	3	0.640557
rs2072280	SBNO2	19	1073147	7	0.043238
rs2072280	SBNO2	19	1073147	3	0.472582
rs2074915	SBNO2	19	1078615	8	0.798638
rs2074915	SBNO2	19	1078615	3	0.123471
rs2074915	SBNO2	19	1078615	1	5.717091
rs12975875	C19orf23	19	1219178	6	4.617178
rs12975875	C19orf23	19	1219178	7	0.689821
rs12975875	C19orf23	19	1219178	3	1.04538
rs757292	C19orf24	19	1228275	3	0.172901
rs757292	C19orf24	19	1228275	2	4.032225
rs757292	C19orf24	19	1228275	7	0.78209
rs757292	C19orf24	19	1228275	6	1.565104
rs713042	MUM1	19	1308082	3	0.168058
rs12608765	MUM1	19	1321675	4	1.585683
rs12608765	MUM1	19	1321675	7	0.48177
rs12608765	MUM1	19	1321675	6	0.396475
rs12608765	MUM1	19	1321675	8	1.397372
rs12608765	MUM1	19	1321675	2	3.899923
rs12608765	MUM1	19	1321675	1	0.407122
rs7251165	RPS15	19	1389865	6	1.26927
rs7251165	RPS15	19	1389865	1	1.11913
rs265278	APC2	19	1420030	1	3.272485
rs265278	APC2	19	1420030	7	2.721041
SNP19-1448547	REEP6	19	1448547	1	4.597543
rs2292449	LOC126520	19	1476307	3	0.606247
rs2256653	LOC126520	19	1477539	3	1.68457
rs12462146	LOC126520	19	1486291	3	2.362917
SNP19-1570350	TCF3	19	1570350	3	0.380894
SNP19-1570350	TCF3	19	1570350	1	5.490839
rs1054597	ATP8B3	19	1733189	5	0.300986
rs1054597	ATP8B3	19	1733189	2	6.146169
rs3764605	ATP8B3	19	1735890	5	0.4347
rs3764605	ATP8B3	19	1735890	2	3.375071
rs3764606	ATP8B3	19	1735944	5	4.443202
rs3764606	ATP8B3	19	1735944	2	4.439507
rs8100856	ATP8B3	19	1747166	5	0.474694
rs45574836	ATP8B3	19	1757667	1	0.015432
rs12609187	ATP8B3	19	1762547	7	0.86031
rs12609187	ATP8B3	19	1762547	6	1.02346

rs2396359	REXO1	19	1770125	4	2.473448
SNP19-1772637	REXO1	19	1772637	8	4.228371
SNP19-1772637	REXO1	19	1772637	2	4.145614
SNP19-1772637	REXO1	19	1772637	6	0.698347
SNP19-1772637	REXO1	19	1772637	7	6.257186
rs3746043	KLF16	19	1804667	8	0.499009
rs3746043	KLF16	19	1804667	6	3.582533
rs3746043	KLF16	19	1804667	7	6.33191
rs12974606	SCAMP4	19	1856405	1	1.257526
rs12974606	SCAMP4	19	1856405	6	0.474126
rs12974606	SCAMP4	19	1856405	7	0.552863
rs11669357	C19orf34	19	1903586	7	0.347094
rs2396366	C19orf34	19	1903841	1	2.599926
rs2396366	C19orf34	19	1903841	4	0.660861
rs2396366	C19orf34	19	1903841	7	0.197313
SNP19-1904495	C19orf34	19	1904495	3	0.730477
SNP19-1904495	C19orf34	19	1904495	4	0.585736
rs1610045	BTBD2	19	1948363	7	0.822404
rs1610045	BTBD2	19	1948363	8	0.037646
rs4476281	MOBKL2A	19	2023599	5	0.409757
rs4476281	MOBKL2A	19	2023599	6	4.819535
rs4476281	MOBKL2A	19	2023599	8	0.862166
rs4476281	MOBKL2A	19	2023599	2	3.522665
rs20567	AP3D1	19	2061746	5	8.38491
rs2523178	C19orf35	19	2226079	5	0.119959
rs3848635	TMPRSS9	19	2361332	1	2.718458
rs8112518	GNG7	19	2463151	5	7.062185
rs8112518	GNG7	19	2463151	1	3.379798
rs8112518	GNG7	19	2463151	3	0.167987
rs7258864	GNG7	19	2463606	3	0.237746
rs7258864	GNG7	19	2463606	6	0.943919
rs917414	GNG7	19	2465360	6	0.079011
rs917414	GNG7	19	2465360	1	1.208384
rs917414	GNG7	19	2465360	3	1.562287
rs917414	GNG7	19	2465360	5	0.302625
rs917415	GNG7	19	2465373	5	0.131805
rs917415	GNG7	19	2465373	1	0.129868
rs917415	GNG7	19	2465373	3	0.400247
rs3752174	GNG7	19	2465565	3	1.106053
rs3752174	GNG7	19	2465565	1	5.776379
rs17755118	GNG7	19	2465732	6	0.056101
rs3764584	GNG7	19	2471716	3	0.701385
rs3764584	GNG7	19	2471716	5	0.545164
rs3764584	GNG7	19	2471716	8	0.241314
rs3764584	GNG7	19	2471716	1	5.496488
rs8737	DIRAS1	19	2665720	1	2.841337
rs8737	DIRAS1	19	2665720	5	0.26933

rs8737	DIRAS1	19	2665720	8	1.519039
rs3745812	DIRAS1	19	2668256	8	0.859295
rs3745812	DIRAS1	19	2668256	1	4.530144
rs9160	SLC39A3	19	2683741	6	8.03552
rs9160	SLC39A3	19	2683741	8	10.61184
rs35301697	THOP1	19	2741618	5	5.441979
rs867169	ZNF554	19	2784864	7	0.313869
SNP19-2887535	ZNF77	19	2887535	5	1.69032
rs12609268	ZNF77	19	2890396	2	2.250197
rs17681742	TLE6	19	2928599	5	1.959823
rs17681742	TLE6	19	2928599	8	1.433002
rs17681742	TLE6	19	2928599	7	1.147739
rs3746072	EDG6	19	3130884	6	0.608552
rs312071	NCLN	19	3159418	8	0.301116
rs312071	NCLN	19	3159418	4	0.266055
rs312071	NCLN	19	3159418	6	0.112172
rs312071	NCLN	19	3159418	1	0.449586
rs312071	NCLN	19	3159418	7	3.118643
rs312071	NCLN	19	3159418	3	0.020017
rs312072	NCLN	19	3159430	8	5.198896
rs312072	NCLN	19	3159430	7	0.169353
rs312072	NCLN	19	3159430	1	3.007696
rs312072	NCLN	19	3159430	3	0.751412
rs312072	NCLN	19	3159430	4	2.010251
rs10155	FZR1	19	3485842	4	0.968292
rs10155	FZR1	19	3485842	2	4.341974
rs7248746	FZR1	19	3489090	2	4.157412
rs7248746	FZR1	19	3489090	4	0.819862
rs17348907	GIPC3	19	3541334	6	0.144337
rs17348907	GIPC3	19	3541334	5	0.838838
rs17348907	GIPC3	19	3541334	3	0.241758
SNP19-3562927	C19orf29	19	3562927	6	0.599566
rs2074789	C19orf29	19	3564346	7	0.208837
rs2074789	C19orf29	19	3564346	3	0.05873
rs7255692	C19orf29	19	3565403	2	3.665728
rs2067019	TJP3	19	3679609	5	1.415891
rs2301843	KIAA1086	19	3771191	4	0.435577
rs2301843	KIAA1086	19	3771191	3	0.121604
rs2301843	KIAA1086	19	3771191	1	0.193114
rs2240233	KIAA1086	19	3782525	5	2.657986
rs2240233	KIAA1086	19	3782525	3	1.800669
rs2240233	KIAA1086	19	3782525	1	2.561032
rs2240233	KIAA1086	19	3782525	7	1.2567
rs2240233	KIAA1086	19	3782525	4	1.793662
rs10424392	ATCAY	19	3876413	3	0.451439
rs16992128	ITGB1BP3	19	3884124	4	4.408758
rs10419272	DAPK3	19	3909708	3	0.776877

rs10419272	DAPK3	19	3909708	4	0.140244
rs3745982	DAPK3	19	3909895	3	0.185394
rs3745982	DAPK3	19	3909895	4	0.38441
rs36527	EEF2	19	3928486	5	1.690909
rs17851657	MAP2K2	19	4053449	3	0.738078
rs17851657	MAP2K2	19	4053449	7	0.526686
rs2240669	ANKRD24	19	4158940	6	0.404228
rs2240669	ANKRD24	19	4158940	7	0.406172
rs12978469	ANKRD24	19	4161356	3	0.355485
rs12978469	ANKRD24	19	4161356	5	1.277127
SNP19-4167910	ANKRD24	19	4167910	6	0.454814
SNP19-4167910	ANKRD24	19	4167910	4	0.290733
SNP19-4167910	ANKRD24	19	4167910	3	0.132138
rs4905	EBI3	19	4188067	2	0.567852
rs4905	EBI3	19	4188067	1	1.013413
rs4905	EBI3	19	4188067	4	0.049447
rs888932	TMIGD2	19	4243597	5	0.121278
SNP19-4245623	TMIGD2	19	4245623	5	0.465229
SNP19-4245623	TMIGD2	19	4245623	8	0.5115
SNP19-4245807	TMIGD2	19	4245807	5	2.262713
SNP19-4245807	TMIGD2	19	4245807	8	2.841837
rs4807578	FSD1	19	4257311	7	0.379605
rs7247504	STAP2	19	4284711	7	0.057986
rs7247504	STAP2	19	4284711	3	0.044244
rs7247504	STAP2	19	4284711	4	2.029702
rs7247504	STAP2	19	4284711	5	4.240654
SNP19-4310191	MPND	19	4310191	7	0.295683
SNP19-4310191	MPND	19	4310191	3	0.031898
rs34148381	CHAF1A	19	4360756	8	0.481048
rs34148381	CHAF1A	19	4360756	6	0.197967
rs9352	CHAF1A	19	4393336	6	0.870308
rs9352	CHAF1A	19	4393336	1	0.482332
rs1044510	UBXD1	19	4408650	8	0.160513
rs1044510	UBXD1	19	4408650	2	1.519491
rs1044510	UBXD1	19	4408650	1	0.601615
rs892161	HDGF2	19	4444708	1	0.690591
rs11878277	HDGF2	19	4449000	3	0.075412
rs11878277	HDGF2	19	4449000	7	0.831617
rs13041	KIAA1881	19	4453282	5	0.172602
rs13041	KIAA1881	19	4453282	8	0.671998
rs13041	KIAA1881	19	4453282	6	6.895957
rs13041	KIAA1881	19	4453282	7	1.242646
rs35929524	KIAA1881	19	4454169	7	1.017403
rs7250947	KIAA1881	19	4461530	1	0.711151
SNP19-4462525	KIAA1881	19	4462525	6	5.229845
SNP19-4462525	KIAA1881	19	4462525	1	3.542686
rs7260518	KIAA1881	19	4462955	5	0.07969

rs966384	LRG1	19	4489599	8	1.94591
rs966384	LRG1	19	4489599	4	1.030139
rs966384	LRG1	19	4489599	2	2.469568
rs3760951	C19orf30	19	4723463	1	1.160972
rs3760951	C19orf30	19	4723463	7	0.317372
rs9973235	M6PRBP1	19	4798713	1	0.196003
rs9304915	M6PRBP1	19	4798874	1	0.07212
rs9304915	M6PRBP1	19	4798874	2	6.647682
rs9304915	M6PRBP1	19	4798874	8	0.281371
rs2271058	M6PRBP1	19	4803106	2	8.356177
rs2271058	M6PRBP1	19	4803106	8	6.864767
rs2271058	M6PRBP1	19	4803106	1	0.175576
rs262560	M6PRBP1	19	4818650	7	5.307875
rs262560	M6PRBP1	19	4818650	6	4.948808
rs262560	M6PRBP1	19	4818650	3	0.73296
rs2656938	ARRDC5	19	4841914	4	2.092251
rs2656938	ARRDC5	19	4841914	5	0.66304
rs2656938	ARRDC5	19	4841914	1	4.089605
rs2656927	C19orf31	19	4859275	1	5.494555
rs2656927	C19orf31	19	4859275	5	3.292523
rs2656927	C19orf31	19	4859275	8	0.303579
rs2261988	UHRF1	19	4861889	4	0.383531
rs2261988	UHRF1	19	4861889	1	0.450732
GA015633	UHRF1	19	4861924	1	1.046869
GA015633	UHRF1	19	4861924	4	1.625373
GA015633	UHRF1	19	4861924	2	4.97747
rs2307205	UHRF1	19	4881838	8	4.877327
rs2307213	UHRF1	19	4901770	3	0.799269
rs2307213	UHRF1	19	4901770	4	8.680738
rs2307213	UHRF1	19	4901770	7	1.093641
rs2307213	UHRF1	19	4901770	5	2.110253
rs2307213	UHRF1	19	4901770	6	2.677959
rs2307213	UHRF1	19	4901770	1	0.631876
rs2240678	JMJD2B	19	4992178	5	6.629533
rs17179181	JMJD2B	19	5088298	1	0.457342
rs17179181	JMJD2B	19	5088298	2	0.735659
rs4456628	PTPRS	19	5157622	6	0.546006
rs4456628	PTPRS	19	5157622	8	5.577145
rs1143700	PTPRS	19	5157775	6	0.491718
rs1143700	PTPRS	19	5157775	1	3.885801
rs2230611	PTPRS	19	5163482	5	0.003653
rs2240744	ZNRF4	19	5406735	3	0.831898
rs420325	ZNRF4	19	5406796	2	4.029711
rs420325	ZNRF4	19	5406796	5	3.041585
rs420325	ZNRF4	19	5406796	3	2.539495
rs420325	ZNRF4	19	5406796	4	2.066665
rs8111108	ZNRF4	19	5407450	5	0.812265

rs8111108	ZNRF4	19	5407450	2	0.408623
rs8111108	ZNRF4	19	5407450	4	0.161722
rs8111108	ZNRF4	19	5407450	3	0.712832
rs10451477	PLAC2	19	5511812	7	0.083318
rs10451477	PLAC2	19	5511812	5	0.022333
rs2288947	PLAC2	19	5513115	3	0.709623
rs2288947	PLAC2	19	5513115	7	0.263929
SNP19-5555875	SAFB2	19	5555875	5	0.668074
rs806706	SAFB2	19	5561045	3	0.069646
rs2290776	DUS3L	19	5742167	8	2.249138
rs2290776	DUS3L	19	5742167	3	1.699564
rs12986368	FUT6	19	5781922	3	0.488465
rs778798	FUT6	19	5790613	7	4.202779
rs778981	FUT5	19	5817036	5	0.387398
rs1056987	NDUFA11	19	5854936	5	3.576886
rs1056987	NDUFA11	19	5854936	6	2.875631
SNP19-5859969	VMAC	19	5859969	2	4.952182
rs1046391	RFX2	19	5944896	2	4.055181
rs2288847	RFX2	19	5958724	8	0.221207
rs2288846	RFX2	19	5993059	7	0.277893
rs2288846	RFX2	19	5993059	6	0.185753
rs2288846	RFX2	19	5993059	1	5.409034
rs16993453	ACSBG2	19	6138657	8	1.432478
rs16993453	ACSBG2	19	6138657	3	0.617351
rs16993553	ASAH3	19	6284476	8	1.433236
rs16993553	ASAH3	19	6284476	3	2.022981
rs1049343	CLPP	19	6319874	4	0.10489
rs2304198	PSPN	19	6326831	4	0.992833
rs12984546	SLC25A41	19	6377277	3	0.362441
rs12984546	SLC25A41	19	6377277	4	0.784579
rs3760750	SLC25A41	19	6378408	6	2.179175
rs34488963	SLC25A41	19	6381106	5	1.357269
rs348362	CRB3	19	6417482	3	0.899133
SNP19-6419331	DENND1C	19	6419331	5	0.286854
SNP19-6419331	DENND1C	19	6419331	8	0.118581
SNP19-6419331	DENND1C	19	6419331	2	7.660585
rs1053395	TUBB4	19	6445371	3	0.932612
rs1053395	TUBB4	19	6445371	8	0.926927
rs1053377	TUBB4	19	6445502	5	0.443046
rs3099129	TUBB4	19	6445915	2	3.488366
rs3099129	TUBB4	19	6445915	1	0.102495
rs3099129	TUBB4	19	6445915	6	0.792647
rs3099129	TUBB4	19	6445915	5	0.316299
rs3099129	TUBB4	19	6445915	4	0.415893
rs3099129	TUBB4	19	6445915	3	0.182392
rs3760746	TNFSF14	19	6621529	1	3.210857
rs17030	C3	19	6628989	1	1.787764

rs428453	C3	19	6653157	1	0.45363
rs428453	C3	19	6653157	2	0.866649
rs428453	C3	19	6653157	5	0.064142
rs428453	C3	19	6653157	7	0.075447
rs428453	C3	19	6653157	6	0.292972
rs2230203	C3	19	6661782	6	0.583111
rs2230203	C3	19	6661782	3	0.666497
rs2230203	C3	19	6661782	7	0.342872
rs4807897	GPR108	19	6687607	3	1.086621
rs4807897	GPR108	19	6687607	8	3.712592
rs340138	GPR108	19	6688481	7	0.069368
rs36097961	VAV1	19	6801767	2	4.694083
rs36097961	VAV1	19	6801767	4	6.230262
rs443658	EMR1	19	6854920	2	2.085181
rs457857	EMR1	19	6864811	7	1.413314
rs457857	EMR1	19	6864811	1	1.900823
rs4807916	EMR1	19	6877378	1	2.806138
rs1051690	INSR	19	7067963	8	8.396664
rs2963	INSR	19	7114154	1	3.670057
rs9282756	INSR	19	7117388	1	4.625956
rs9282756	INSR	19	7117388	8	9.653456
rs891087	INSR	19	7135518	1	4.550414
rs891087	INSR	19	7135518	8	4.788666
rs2287915	ARHGEF18	19	7430855	8	0.03979
rs2287915	ARHGEF18	19	7430855	5	5.794118
rs2287915	ARHGEF18	19	7430855	2	4.002888
SNP19-7439850	ARHGEF18	19	7439850	4	0.216819
rs685034	C19orf45	19	7476978	3	0.104131
rs685034	C19orf45	19	7476978	8	1.682786
rs685034	C19orf45	19	7476978	6	0.052825
rs685034	C19orf45	19	7476978	4	0.275148
rs3826736	C19orf45	19	7477030	7	0.628834
rs3826736	C19orf45	19	7477030	2	0.779354
rs3826736	C19orf45	19	7477030	1	0.358471
rs3826736	C19orf45	19	7477030	8	0.648605
rs1133378	C19orf45	19	7479287	7	0.235734
rs1133378	C19orf45	19	7479287	8	0.314783
rs1133378	C19orf45	19	7479287	1	1.136759
rs1133378	C19orf45	19	7479287	2	2.253488
rs608144	C19orf45	19	7479297	7	0.930845
rs608144	C19orf45	19	7479297	1	0.461437
rs608144	C19orf45	19	7479297	8	0.591317
SNP19-7490483	ZNF358	19	7490483	1	2.937629
SNP19-7512908	PNPLA6	19	7512908	4	3.851665
SNP19-7512908	PNPLA6	19	7512908	3	1.11967
SNP19-7512908	PNPLA6	19	7512908	6	6.498743
SNP19-7512908	PNPLA6	19	7512908	7	0.907804

SNP19-7512908	PNPLA6	19	7512908	1	2.517743
rs4134809	XAB2	19	7600393	5	0.20822
rs4134809	XAB2	19	7600393	3	1.036732
rs4134809	XAB2	19	7600393	6	6.786782
rs4134809	XAB2	19	7600393	1	5.237111
SNP19-7649821	C19orf59	19	7649821	4	1.027492
SNP19-7649821	C19orf59	19	7649821	8	0.899724
SNP19-7668440	FCER2	19	7668440	5	0.523036
SNP19-7668440	FCER2	19	7668440	3	2.223737
GA015756	EVI5L	19	7822620	6	5.148854
rs2288430	EVI5L	19	7834727	6	0.123471
rs2288430	EVI5L	19	7834727	1	3.160301
rs2288430	EVI5L	19	7834727	8	6.713848
rs2288430	EVI5L	19	7834727	7	0.576621
rs11260038	hCG_2003956	19	7850849	1	1.850497
rs11260038	hCG_2003956	19	7850849	4	1.396199
SNP19-7869949	LRRC8E	19	7869949	5	6.257359
SNP19-7869949	LRRC8E	19	7869949	7	0.548629
SNP19-7869949	LRRC8E	19	7869949	8	0.52376
SNP19-7869949	LRRC8E	19	7869949	6	1.435761
rs3745383	LRRC8E	19	7871339	6	0.153561
rs2303701	MAP2K7	19	7881586	6	4.811632
rs3745386	MAP2K7	19	7883870	8	5.25858
rs3745386	MAP2K7	19	7883870	2	0.317727
rs3745386	MAP2K7	19	7883870	1	0.62789
rs7788	SNAPC2	19	7894117	2	1.19628
rs7788	SNAPC2	19	7894117	8	1.561296
rs7899	TIMM44	19	7897679	1	5.015882
rs7899	TIMM44	19	7897679	5	0.509956
rs7899	TIMM44	19	7897679	4	0.380651
rs4804244	ELAVL1	19	7933055	3	0.08772
rs4804244	ELAVL1	19	7933055	4	6.179094
rs4804244	ELAVL1	19	7933055	2	3.529903
rs4804244	ELAVL1	19	7933055	1	0.864949
rs4804244	ELAVL1	19	7933055	7	0.651969
SNP19-8027096	CCL25	19	8027096	2	1.843566
rs1129763	CCL25	19	8027369	6	0.042391
rs2303164	CCL25	19	8028737	5	0.876483
rs2303164	CCL25	19	8028737	7	0.3395
rs2303164	CCL25	19	8028737	3	2.209568
rs41320348	CCL25	19	8033523	2	0.821671
rs3848570	FBN3	19	8051928	7	2.918941
rs7245429	FBN3	19	8065362	6	0.888353
rs7245429	FBN3	19	8065362	2	2.723689
rs7245429	FBN3	19	8065362	3	0.384762
rs7245429	FBN3	19	8065362	5	0.742893
rs12608849	FBN3	19	8066334	6	1.949696

rs12608849	FBN3	19	8066334	5	1.235122
rs12608849	FBN3	19	8066334	2	6.33435
SNP19-8066897	FBN3	19	8066897	8	1.079605
SNP19-8066897	FBN3	19	8066897	3	0.208512
rs33967815	FBN3	19	8074545	3	0.895417
rs33967815	FBN3	19	8074545	7	0.994047
rs33967815	FBN3	19	8074545	6	1.544955
rs33967815	FBN3	19	8074545	5	0.8906
rs17160196	FBN3	19	8082009	7	0.70146
rs3813778	FBN3	19	8097401	3	0.523223
rs3813778	FBN3	19	8097401	5	1.334469
rs3813778	FBN3	19	8097401	6	0.825311
rs3813778	FBN3	19	8097401	7	1.297101
rs12974280	FBN3	19	8102508	3	0.246741
rs12974280	FBN3	19	8102508	5	1.137921
rs12974280	FBN3	19	8102508	7	0.630411
rs7246376	FBN3	19	8109328	4	0.195467
rs12891	LASS4	19	8233196	2	0.675451
rs12891	LASS4	19	8233196	1	0.304535
rs12891	LASS4	19	8233196	3	1.179802
rs12891	LASS4	19	8233196	8	5.087739
rs12891	LASS4	19	8233196	6	0.090858
rs12981073	ANKRD47	19	8295405	8	1.416369
rs12981073	ANKRD47	19	8295405	4	1.35096
rs12981073	ANKRD47	19	8295405	5	0.628357
rs1019757	ANKRD47	19	8295520	8	5.82164
SNP19-8304006	KANK3	19	8304006	8	2.404635
SNP19-8304006	KANK3	19	8304006	4	1.153166
rs8106699	ANKRD47	19	8305272	4	0.710577
rs8106699	ANKRD47	19	8305272	5	0.0027
rs8106699	ANKRD47	19	8305272	8	0.117503
rs710949	ANKRD47	19	8305628	5	1.620787
rs710949	ANKRD47	19	8305628	1	2.736063
rs890853	ANKRD47	19	8305635	4	0.95655
rs11882865	2-Mar	19	8392884	1	0.93239
rs11882865	2-Mar	19	8392884	3	0.979148
rs2910443	ZNF414	19	8482307	1	4.637251
rs2910443	ZNF414	19	8482307	8	0.806796
rs2910443	ZNF414	19	8482307	5	3.90706
rs2910443	ZNF414	19	8482307	3	1.304144
rs2910443	ZNF414	19	8482307	6	3.679183
rs2287863	ZNF414	19	8485011	2	0.55998
rs1054623	MYO1F	19	8492086	2	5.352942
rs4476282	ADAMTS10	19	8557562	6	0.576476
rs2340550	MGC33407	19	8669942	4	1.889868
rs2340550	MGC33407	19	8669942	7	3.103667
rs2340550	MGC33407	19	8669942	3	0.110866

rs2340550	MGC33407	19	8669942	2	1.723136
rs2340550	MGC33407	19	8669942	1	2.129345
rs1035442	MUC16	19	8822981	3	0.886214
SNP19-8854018	MUC16	19	8854018	2	1.169002
SNP19-8854018	MUC16	19	8854018	1	0.790633
SNP19-8854018	MUC16	19	8854018	6	0.68846
SNP19-8864645	MUC16	19	8864645	4	1.04858
SNP19-8864645	MUC16	19	8864645	2	3.352606
SNP19-8867749	MUC16	19	8867749	7	1.095439
SNP19-8867749	MUC16	19	8867749	4	0.30628
SNP19-8867749	MUC16	19	8867749	5	0.786275
SNP19-8867749	MUC16	19	8867749	8	0.9777
SNP19-8889373	MUC16	19	8889373	3	0.707788
SNP19-8889373	MUC16	19	8889373	5	0.678322
SNP19-8889373	MUC16	19	8889373	7	0.240564
SNP19-8889373	MUC16	19	8889373	4	0.206857
SNP19-8906821	MUC16	19	8906821	6	0.699861
SNP19-8906821	MUC16	19	8906821	2	5.432427
rs10415818	MUC16	19	8908305	2	7.175627
rs10415818	MUC16	19	8908305	5	0.753784
rs10415818	MUC16	19	8908305	6	0.35115
rs10415818	MUC16	19	8908305	1	0.676895
SNP19-8909170	MUC16	19	8909170	2	1.751661
SNP19-8909170	MUC16	19	8909170	1	1.005115
SNP19-8909170	MUC16	19	8909170	5	1.205563
SNP19-8909320	MUC16	19	8909320	3	0.481364
SNP19-8909320	MUC16	19	8909320	7	0.408182
SNP19-8909320	MUC16	19	8909320	1	3.987851
SNP19-8909342	MUC16	19	8909342	6	6.234374
SNP19-8909342	MUC16	19	8909342	4	0.638005
SNP19-8909342	MUC16	19	8909342	5	0.434287
SNP19-8909342	MUC16	19	8909342	2	2.702445
SNP19-8909342	MUC16	19	8909342	7	0.354382
SNP19-8909342	MUC16	19	8909342	3	0.136604
rs11883220	MUC16	19	8910339	2	3.651761
rs11883220	MUC16	19	8910339	3	0.041246
rs11883220	MUC16	19	8910339	7	8.105216
SNP19-8910910	MUC16	19	8910910	3	0.217935
SNP19-8910910	MUC16	19	8910910	1	3.12584
SNP19-8910910	MUC16	19	8910910	7	0.370322
SNP19-8917941	MUC16	19	8917941	5	0.913872
SNP19-8917941	MUC16	19	8917941	2	1.608024
SNP19-8917941	MUC16	19	8917941	6	0.134255
SNP19-8917941	MUC16	19	8917941	1	1.026057
SNP19-8918408	MUC16	19	8918408	4	0.633507
rs12459532	MUC16	19	8920181	4	1.083034
SNP19-8920307	MUC16	19	8920307	4	0.50937

SNP19-8920307	MUC16	19	8920307	8	6.942275
rs7508222	MUC16	19	8920808	4	6.57288
SNP19-8921085	MUC16	19	8921085	4	0.664194
SNP19-8921085	MUC16	19	8921085	5	5.131482
SNP19-8921085	MUC16	19	8921085	3	0.486741
SNP19-8921085	MUC16	19	8921085	2	0.278853
SNP19-8921085	MUC16	19	8921085	1	1.272764
SNP19-8921085	MUC16	19	8921085	8	10.61604
SNP19-8921085	MUC16	19	8921085	7	0.675236
SNP19-8921085	MUC16	19	8921085	6	0.176368
rs1820099	MUC16	19	8921812	4	0.697717
rs1862458	MUC16	19	8930792	3	0.325876
rs1862458	MUC16	19	8930792	1	5.091044
rs1344572	MUC16	19	8931194	6	0.68802
SNP19-8931402	MUC16	19	8931402	3	0.190274
SNP19-8931402	MUC16	19	8931402	2	1.272514
SNP19-8931402	MUC16	19	8931402	4	6.050568
SNP19-8931402	MUC16	19	8931402	5	0.891491
SNP19-8931402	MUC16	19	8931402	8	4.597375
SNP19-8931402	MUC16	19	8931402	7	0.211238
SNP19-8931402	MUC16	19	8931402	6	5.622536
rs10422567	MUC16	19	8932763	6	0.634918
SNP19-8933975	MUC16	19	8933975	3	0.519866
SNP19-8933975	MUC16	19	8933975	1	1.366214
SNP19-8933975	MUC16	19	8933975	7	9.461304
SNP19-8935073	MUC16	19	8935073	7	4.121697
SNP19-8935073	MUC16	19	8935073	1	4.757811
SNP19-8935073	MUC16	19	8935073	3	0.000216
rs2547068	MUC16	19	8936217	7	0.939673
rs2547068	MUC16	19	8936217	3	0.499274
rs2547068	MUC16	19	8936217	8	5.576574
rs2547068	MUC16	19	8936217	5	5.120081
rs2547068	MUC16	19	8936217	6	6.012682
rs2547068	MUC16	19	8936217	2	5.140573
rs2591592	MUC16	19	8936346	8	6.458695
rs2591592	MUC16	19	8936346	7	0.404335
rs2591592	MUC16	19	8936346	6	8.681656
rs2591592	MUC16	19	8936346	1	3.693002
rs2591592	MUC16	19	8936346	3	0.439792
SNP19-8936565	MUC16	19	8936565	4	0.239088
rs2547069	MUC16	19	8936584	3	0.076095
rs2547069	MUC16	19	8936584	7	0.026163
rs2547069	MUC16	19	8936584	5	0.111382
rs2547069	MUC16	19	8936584	6	10.96966
rs2547074	MUC16	19	8937858	6	6.60616
rs2547074	MUC16	19	8937858	2	4.156752
rs2547074	MUC16	19	8937858	3	0.977401

rs2547074	MUC16	19	8937858	5	0.279255
SNP19-8938581	MUC16	19	8938581	1	4.832344
SNP19-8938581	MUC16	19	8938581	7	0.183696
SNP19-8938581	MUC16	19	8938581	3	1.177528
rs2547065	MUC16	19	8941462	5	0.268958
rs2547065	MUC16	19	8941462	4	0.023958
SNP19-8944791	MUC16	19	8944791	4	2.638797
rs1823056	MUC16	19	8947819	1	4.860074
rs1823056	MUC16	19	8947819	7	6.319131
rs1823056	MUC16	19	8947819	3	0.217528
SNP19-8951784	MUC16	19	8951784	4	0.016711
rs4804401	OR7G2	19	9074079	4	1.139859
rs4804401	OR7G2	19	9074079	1	2.304676
rs7246969	OR7G1	19	9086940	8	2.920667
rs7246969	OR7G1	19	9086940	3	1.000473
rs6511918	OR7D2	19	9159364	3	1.338159
rs2240927	OR7E24	19	9223343	7	1.503636
SNP19-9313698	ZNF559	19	9313698	3	0.291234
rs10416984	ZNF177	19	9343310	1	4.554879
rs10416984	ZNF177	19	9343310	3	0.433917
rs10416984	ZNF177	19	9343310	5	0.409796
rs2230752	ZNF177	19	9353370	5	0.118817
rs2230752	ZNF177	19	9353370	1	3.886337
rs2230752	ZNF177	19	9353370	3	0.170065
rs2230752	ZNF177	19	9353370	6	0.272508
rs1978713	ZNF266	19	9390783	4	0.258814
rs1978713	ZNF266	19	9390783	5	1.912803
rs1978713	ZNF266	19	9390783	1	3.916008
rs1978713	ZNF266	19	9390783	6	1.530719
rs2042200	ZNF426	19	9507898	2	2.534158
rs2042200	ZNF426	19	9507898	3	1.211817
rs2303100	OLFM2	19	9829434	2	4.856576
rs2303100	OLFM2	19	9829434	3	0.979613
rs10406152	COL5A3	19	9932063	7	0.941129
rs2287813	COL5A3	19	9945292	7	1.074891
rs2287813	COL5A3	19	9945292	5	0.630288
rs3815745	COL5A3	19	9946054	4	1.063186
rs2161468	COL5A3	19	9949271	4	3.063822
rs7249755	LOC388503	19	10015022	7	0.091309
rs8113341	LOC388503	19	10026932	8	3.923597
rs10417403	LOC388503	19	10033516	4	1.702124
rs10417403	LOC388503	19	10033516	3	2.262207
rs3745601	P2RY11	19	10085548	1	1.887953
rs3745601	P2RY11	19	10085548	6	0.38278
rs3745601	P2RY11	19	10085548	3	0.301989
rs3816556	P2RY11	19	10086682	1	3.504765
rs3816556	P2RY11	19	10086682	5	1.390047

rs3816556	P2RY11	19	10086682	8	0.418003
rs2305795	P2RY11	19	10087052	7	1.326507
rs2305795	P2RY11	19	10087052	4	0.265179
rs2305795	P2RY11	19	10087052	1	3.854086
rs2305795	P2RY11	19	10087052	5	0.299096
rs721186	DNMT1	19	10126312	6	1.219206
rs721186	DNMT1	19	10126312	7	2.841282
rs2228611	DNMT1	19	10128077	7	0.589013
rs10404517	LOC646106	19	10188893	6	0.350417
rs10404517	LOC646106	19	10188893	4	1.254857
rs10404517	LOC646106	19	10188893	3	0.980746
rs1799969	ICAM1	19	10255792	8	2.596222
rs1799969	ICAM1	19	10255792	4	0.603844
rs5498	ICAM1	19	10256683	6	0.957449
rs5498	ICAM1	19	10256683	8	0.746237
rs3093032	ICAM1	19	10257336	8	5.547666
rs923366	ICAM1	19	10258223	8	1.623421
rs281437	ICAM1	19	10258238	1	1.544224
rs281437	ICAM1	19	10258238	6	0.480127
rs281437	ICAM1	19	10258238	2	0.367376
rs2230399	ICAM3	19	10305603	7	0.159577
rs7258015	ICAM3	19	10310358	7	0.52491
rs7258015	ICAM3	19	10310358	4	0.476547
rs2304240	ICAM3	19	10310392	6	0.709553
rs2304240	ICAM3	19	10310392	7	1.518669
rs281414	ICAM3	19	10311285	4	0.807587
rs12720356	TYK2	19	10330975	3	1.236252
rs280523	TYK2	19	10338206	1	4.787954
rs1051738	PDE4A	19	10438843	3	0.120569
rs1051738	PDE4A	19	10438843	1	5.577988
rs3826709	KRI1	19	10531184	7	0.55976
rs3826709	KRI1	19	10531184	8	0.095624
rs3826709	KRI1	19	10531184	4	0.992722
rs3826709	KRI1	19	10531184	2	5.617808
rs3826709	KRI1	19	10531184	1	0.878603
rs3745249	KRI1	19	10531992	3	0.663464
rs3745249	KRI1	19	10531992	7	0.884458
rs3745249	KRI1	19	10531992	8	0.053989
rs3745249	KRI1	19	10531992	1	0.9288
rs12984043	KRI1	19	10533493	1	0.821694
rs12984043	KRI1	19	10533493	4	0.800725
rs12984043	KRI1	19	10533493	7	0.518713
rs3218222	KRI1	19	10537681	3	0.168972
rs2304163	AP1M2	19	10544602	5	4.47181
rs3745674	DNM2	19	10754735	5	0.955231
SNP19-10895582	YIPF2	19	10895582	5	0.207736
rs17001073	SMARCA4	19	10962993	3	0.29344

rs28997582	SMARCA4	19	11006691	6	1.685225
rs14158	LDLR	19	11103044	3	0.161489
rs14158	LDLR	19	11103044	2	4.084925
rs14158	LDLR	19	11103044	8	0.549055
rs17243011	LDLR	19	11103215	8	7.532562
rs2738464	LDLR	19	11103307	5	0.465864
rs2738464	LDLR	19	11103307	3	0.246302
rs3745681	ANKRD25	19	11164943	1	0.835279
rs3745681	ANKRD25	19	11164943	8	0.609275
rs3745681	ANKRD25	19	11164943	6	0.442248
rs3745681	ANKRD25	19	11164943	7	0.87668
rs3745681	ANKRD25	19	11164943	2	0.13566
rs3745681	ANKRD25	19	11164943	4	0.867958
rs755237	ANKRD25	19	11165404	6	0.139929
rs755237	ANKRD25	19	11165404	4	0.48809
rs755237	ANKRD25	19	11165404	8	0.391188
rs755238	ANKRD25	19	11165498	4	0.027935
rs755238	ANKRD25	19	11165498	8	4.261747
rs755238	ANKRD25	19	11165498	6	0.49274
rs754528	ANKRD25	19	11166245	8	1.1621
rs754528	ANKRD25	19	11166245	4	0.646768
rs754528	ANKRD25	19	11166245	6	1.14858
rs7250778	ANKRD25	19	11167265	4	2.794718
rs7250778	ANKRD25	19	11167265	6	0.051735
rs7250778	ANKRD25	19	11167265	7	0.086137
rs7250778	ANKRD25	19	11167265	2	3.904099
SNP19-11185965	DOCK6	19	11185965	6	0.598262
rs3810307	DOCK6	19	11193570	8	0.469434
rs892066	LOC55908	19	11211340	1	0.602399
rs11667275	LPPR2	19	11334358	3	0.234432
rs2291516	RGL3	19	11369177	5	0.061621
rs2291516	RGL3	19	11369177	3	0.135981
rs10416704	RGL3	19	11377007	7	0.241449
rs167479	RGL3	19	11387765	1	3.554764
rs167479	RGL3	19	11387765	6	1.000475
SNP19-11419275	PRKCSH	19	11419275	2	6.812708
SNP19-11419275	PRKCSH	19	11419275	7	4.926801
SNP19-11419275	PRKCSH	19	11419275	4	1.408835
SNP19-11459224	ZNF653	19	11459224	3	0.6907
rs311805	ZNF653	19	11459483	3	0.354986
rs2305799	ACP5	19	11548351	2	4.129445
rs799193	ZNF441	19	11752003	8	0.34123
rs799193	ZNF441	19	11752003	1	6.053361
rs11670714	ZNF491	19	11779878	7	0.389205
rs11670714	ZNF491	19	11779878	4	0.841245
rs436411	ZNF440	19	11803771	4	0.786861
rs436411	ZNF440	19	11803771	7	0.109564

SNP19-11920645	ZNF700	19	11920645	5	0.941794
rs1975349	ZNF788	19	12083861	4	3.894031
rs1975349	ZNF788	19	12083861	6	0.671697
rs1975349	ZNF788	19	12083861	7	0.260556
rs1975349	ZNF788	19	12083861	1	3.607272
rs1975349	ZNF788	19	12083861	8	3.958713
rs17706075	ZNF799	19	12352779	3	0.495285
rs17706075	ZNF799	19	12352779	8	5.565864
rs17706075	ZNF799	19	12352779	5	0.048211
rs17706075	ZNF799	19	12352779	6	0.173277
rs17706075	ZNF799	19	12352779	4	0.218708
rs11668074	ZNF799	19	12352955	4	1.398551
rs11668074	ZNF799	19	12352955	5	0.447679
rs11668074	ZNF799	19	12352955	6	0.687901
rs11668074	ZNF799	19	12352955	3	0.098279
rs10414984	ZNF799	19	12353039	3	0.509428
rs10414984	ZNF799	19	12353039	4	0.07844
rs3745658	ZNF443	19	12412789	6	0.468293
rs3745658	ZNF443	19	12412789	3	0.867058
rs35836657	MAN2B1	19	12629948	5	0.761743
rs17706412	BEST2	19	12726821	7	1.046777
rs897804	HOOK2	19	12737964	5	0.42244
rs897804	HOOK2	19	12737964	2	5.395012
rs897804	HOOK2	19	12737964	7	0.149199
rs2290688	MAST1	19	12819697	1	3.051613
rs11085822	MAST1	19	12846576	7	0.729677
rs11085822	MAST1	19	12846576	1	2.296352
GA034056	NFIX	19	13068284	4	0.40733
rs8112067	BTBD14B	19	13110244	1	0.035909
rs8112067	BTBD14B	19	13110244	4	6.297724
rs8112067	BTBD14B	19	13110244	6	6.596356
rs8112067	BTBD14B	19	13110244	3	0.231596
rs897790	NANOS3	19	13849040	6	1.063989
rs897790	NANOS3	19	13849040	1	2.732204
rs897790	NANOS3	19	13849040	5	0.532943
rs2016163	NANOS3	19	13849416	1	2.00745
rs2016163	NANOS3	19	13849416	5	0.403841
rs2242130	C19orf57	19	13854457	6	5.169179
rs2242130	C19orf57	19	13854457	4	1.748648
rs2242130	C19orf57	19	13854457	1	6.132773
rs2242130	C19orf57	19	13854457	5	1.004707
rs2305775	C19orf57	19	13861870	5	0.748188
rs2305775	C19orf57	19	13861870	1	3.53898
rs6511898	C19orf57	19	13876711	5	0.023944
rs6511898	C19orf57	19	13876711	1	4.641987
rs6511898	C19orf57	19	13876711	6	3.005732
rs11669628	CC2D1A	19	13901896	1	1.709778

SNP19-14014293	IL27RA	19	14014293	2	2.957391
SNP19-14014293	IL27RA	19	14014293	7	3.003606
SNP19-14014293	IL27RA	19	14014293	5	6.040995
rs3810256	LPHN1	19	14120846	1	5.413409
rs3810256	LPHN1	19	14120846	4	0.795858
rs3810256	LPHN1	19	14120846	6	6.279457
rs11668633	PTGER1	19	14444582	3	1.149841
rs11668633	PTGER1	19	14444582	1	4.293968
rs1803768	DNAJB1	19	14487197	2	4.426433
rs4606855	EMR3	19	14630339	1	0.059447
SNP19-14690749	ZNF333	19	14690749	2	0.690332
SNP19-14690749	ZNF333	19	14690749	1	0.37933
rs757298	EMR2	19	14704292	3	0.005334
rs757299	EMR2	19	14704490	3	0.41494
rs2524383	EMR2	19	14723430	2	6.651659
rs2524383	EMR2	19	14723430	1	0.85621
rs2524383	EMR2	19	14723430	4	0.816553
rs2524383	EMR2	19	14723430	7	3.260198
rs2524383	EMR2	19	14723430	6	0.028914
SNP19-14736388	EMR2	19	14736388	4	5.247858
SNP19-14736388	EMR2	19	14736388	8	6.172343
SNP19-14736388	EMR2	19	14736388	2	0.243932
SNP19-14736388	EMR2	19	14736388	5	1.145744
SNP19-14736388	EMR2	19	14736388	6	0.621454
rs16979912	OR7C1	19	14771321	4	0.763912
rs16979912	OR7C1	19	14771321	5	1.05648
rs17230134	OR7C1	19	14771654	2	3.139628
rs17230134	OR7C1	19	14771654	1	1.055186
rs17230134	OR7C1	19	14771654	4	0.256662
rs17230134	OR7C1	19	14771654	5	4.784847
rs2190686	OR7A5	19	14799616	5	0.61962
rs2190686	OR7A5	19	14799616	6	1.96374
rs11883178	OR7C2	19	14913665	1	4.857505
rs16980148	SLC1A6	19	14924757	1	1.85593
rs16980148	SLC1A6	19	14924757	4	0.798171
rs16980148	SLC1A6	19	14924757	3	0.667947
rs16980148	SLC1A6	19	14924757	2	5.356968
rs16980148	SLC1A6	19	14924757	7	4.577965
rs10424547	CCDC105	19	14994787	1	0.858847
rs8112667	CCDC105	19	14994926	3	0.974165
rs8105737	OR1I1	19	15059631	7	1.218875
rs16980312	OR1I1	19	15059751	3	0.188876
rs16980313	OR1I1	19	15059851	3	2.486408
rs2074262	ILVBL	19	15087970	1	0.608718
rs2074262	ILVBL	19	15087970	5	1.054388
rs12082	NOTCH3	19	15131636	2	0.951165
rs12082	NOTCH3	19	15131636	1	0.467129

rs757291	ABHD9	19	15198785	6	1.575845
rs2304200	PGLYRP2	19	15441658	3	0.616905
SNP19-15443863	PGLYRP2	19	15443863	2	2.761326
SNP19-15443863	PGLYRP2	19	15443863	1	3.092514
rs2280434	CYP4F22	19	15523732	8	1.978279
rs2280434	CYP4F22	19	15523732	1	1.604398
rs4239614	CYP4F8	19	15601220	4	3.119403
rs4239614	CYP4F8	19	15601220	8	3.568482
rs4239614	CYP4F8	19	15601220	3	1.31881
rs4239614	CYP4F8	19	15601220	6	2.742351
rs2239367	CYP4F8	19	15601268	8	2.545582
rs2239367	CYP4F8	19	15601268	4	1.595529
rs2239367	CYP4F8	19	15601268	3	0.934859
rs4019753	CYP4F8	19	15601445	6	1.436745
rs4019753	CYP4F8	19	15601445	4	0.53027
rs4019753	CYP4F8	19	15601445	1	0.192857
rs2733760	CYP4F3	19	15631752	3	0.389908
rs28371511	CYP4F3	19	15632028	7	0.265997
rs28371511	CYP4F3	19	15632028	6	1.901319
rs28371514	CYP4F3	19	15632250	5	1.015234
rs28371514	CYP4F3	19	15632250	1	1.525123
rs7258240	CYP4F3	19	15633414	4	2.451298
rs7258240	CYP4F3	19	15633414	6	1.830125
rs7258240	CYP4F3	19	15633414	5	1.249721
rs7258240	CYP4F3	19	15633414	1	3.356959
rs2947240	CYP4F3	19	15633521	3	0.194546
rs4343408	CYP4F3	19	15633530	1	4.385343
rs4343408	CYP4F3	19	15633530	5	2.628593
rs4343408	CYP4F3	19	15633530	6	1.477376
rs4343408	CYP4F3	19	15633530	4	0.165434
rs10425683	CYP4F3	19	15633548	1	3.265486
rs10425683	CYP4F3	19	15633548	5	0.974921
rs10425683	CYP4F3	19	15633548	4	0.411732
rs4358060	CYP4F3	19	15633564	4	0.2968
rs4358060	CYP4F3	19	15633564	6	0.819144
rs4358060	CYP4F3	19	15633564	5	0.832976
rs4358060	CYP4F3	19	15633564	1	2.864376
rs3204846	CYP4F3	19	15633717	4	0.519199
rs3204846	CYP4F3	19	15633717	6	0.398536
rs3204846	CYP4F3	19	15633717	1	3.043841
rs3204846	CYP4F3	19	15633717	5	1.05915
rs1140863	CYP4F3	19	15633738	4	0.689452
rs1140863	CYP4F3	19	15633738	6	0.492807
rs1140863	CYP4F3	19	15633738	5	1.241855
rs1140863	CYP4F3	19	15633738	1	3.794981
rs28738979	CYP4F3	19	15634369	4	0.058316
rs28738979	CYP4F3	19	15634369	6	2.780185

rs609636	CYP4F12	19	15650098	8	2.389903
rs609636	CYP4F12	19	15650098	2	2.158475
SNP19-15654235	CYP4F12	19	15654235	8	5.775719
SNP19-15654235	CYP4F12	19	15654235	2	0.744289
SNP19-15654235	CYP4F12	19	15654235	1	6.511417
SNP19-15654235	CYP4F12	19	15654235	3	0.49434
SNP19-15654235	CYP4F12	19	15654235	4	6.994592
rs11670326	OR10H3	19	15713544	7	1.751783
rs11670326	OR10H3	19	15713544	5	2.611523
rs2240229	OR10H3	19	15714080	1	0.488532
rs3746151	OR10H5	19	15766639	3	3.057672
rs3746151	OR10H5	19	15766639	1	3.756166
rs4808383	OR10H1	19	15779802	3	0.351738
rs2108622	CYP4F2	19	15851431	2	0.650634
rs2108622	CYP4F2	19	15851431	5	3.320182
rs2108622	CYP4F2	19	15851431	7	2.950678
rs12459140	LOC729654	19	15882888	8	0.142455
rs12459140	LOC729654	19	15882888	2	0.105981
rs12459140	LOC729654	19	15882888	1	1.381772
rs12610962	CYP4F11	19	15884377	1	0.257739
rs12610962	CYP4F11	19	15884377	6	2.295541
rs12610962	CYP4F11	19	15884377	2	4.990931
rs12610962	CYP4F11	19	15884377	3	2.613692
SNP19-15895714	CYP4F11	19	15895714	3	2.013309
SNP19-15895714	CYP4F11	19	15895714	8	6.086197
SNP19-15895714	CYP4F11	19	15895714	6	3.423732
rs2305800	CYP4F11	19	15906294	1	1.039998
rs2305800	CYP4F11	19	15906294	5	2.346326
rs16980822	OR10H4	19	15921248	1	2.164276
rs16980822	OR10H4	19	15921248	2	0.85945
rs16980822	OR10H4	19	15921248	5	1.866603
rs4808423	LOC126536	19	15997405	4	0.564955
rs4808423	LOC126536	19	15997405	2	0.087895
rs4808423	LOC126536	19	15997405	5	0.004606
rs1043452	RAB8A	19	16105326	7	8.297383
rs1043452	RAB8A	19	16105326	2	0.917929
rs1043452	RAB8A	19	16105326	6	7.73146
rs1043452	RAB8A	19	16105326	1	0.316287
rs6512087	CIB3	19	16136655	4	4.282007
rs6512087	CIB3	19	16136655	3	0.497172
rs6512087	CIB3	19	16136655	7	0.078509
rs9305079	CALR3	19	16452464	7	1.084176
rs9305079	CALR3	19	16452464	5	0.816578
rs8111801	C19orf44	19	16492776	6	5.246058
rs2287869	SLC35E1	19	16527101	4	0.72931
rs2287869	SLC35E1	19	16527101	1	4.849312
rs2287869	SLC35E1	19	16527101	3	0.745443

rs12983375	NWD1	19	16692060	1	4.678402
rs12983375	NWD1	19	16692060	6	4.847049
SNP19-16703052	NWD1	19	16703052	8	0.011787
SNP19-16703052	NWD1	19	16703052	1	6.025539
SNP19-16703052	NWD1	19	16703052	4	0.237186
rs773905	F2RL3	19	16860897	7	0.059584
rs773902	F2RL3	19	16861632	1	1.536269
rs773902	F2RL3	19	16861632	7	1.018222
rs2227349	F2RL3	19	16862551	3	0.628134
rs2227349	F2RL3	19	16862551	5	1.213299
rs773901	CPAMD8	19	16864789	7	0.494604
rs773901	CPAMD8	19	16864789	3	2.338229
rs773901	CPAMD8	19	16864789	2	0.933897
rs773901	CPAMD8	19	16864789	5	1.365233
rs706761	CPAMD8	19	16886292	3	1.916734
rs706761	CPAMD8	19	16886292	4	0.97521
rs706761	CPAMD8	19	16886292	1	3.528567
rs706761	CPAMD8	19	16886292	8	0.10584
rs2305758	NY-SAR-48	19	17024661	1	0.026373
rs2305758	NY-SAR-48	19	17024661	7	0.472171
rs2305758	NY-SAR-48	19	17024661	2	1.45109
rs12459084	NY-SAR-48	19	17031885	7	0.171764
rs12459084	NY-SAR-48	19	17031885	1	0.588755
rs12459084	NY-SAR-48	19	17031885	6	0.098896
rs12459084	NY-SAR-48	19	17031885	2	0.445995
rs3745349	MYO9B	19	17073992	5	0.969869
rs3745349	MYO9B	19	17073992	7	1.972809
SNP19-17177782	MYO9B	19	17177782	4	0.960027
SNP19-17177782	MYO9B	19	17177782	2	5.663577
rs17535005	MYO9B	19	17179018	2	3.943591
rs1045201	OCEL1	19	17198928	8	6.579294
rs744067	OCEL1	19	17200947	8	7.028817
rs3745168	USHBP1	19	17221878	2	3.457354
rs3745168	USHBP1	19	17221878	3	1.902971
rs3745168	USHBP1	19	17221878	6	3.059317
rs1864116	ANKRD41	19	17254015	5	0.850505
rs2363956	ANKRD41	19	17255124	3	0.579661
rs2363956	ANKRD41	19	17255124	4	0.683243
rs2363956	ANKRD41	19	17255124	5	0.332728
rs891017	ANKRD41	19	17255504	4	1.067767
rs891017	ANKRD41	19	17255504	3	0.292078
rs891017	ANKRD41	19	17255504	5	0.313089
rs891017	ANKRD41	19	17255504	8	0.381191
rs11086065	ANKRD41	19	17256003	6	4.133692
rs11086065	ANKRD41	19	17256003	4	1.722453
rs11086065	ANKRD41	19	17256003	3	0.027228
rs751599	ANKRD41	19	17257549	3	0.303871

rs751599	ANKRD41	19	17257549	5	0.74718
rs751599	ANKRD41	19	17257549	6	0.856809
rs751599	ANKRD41	19	17257549	4	2.538342
rs751599	ANKRD41	19	17257549	8	0.582362
rs10401700	ANKRD41	19	17258700	4	0.8752
rs10401700	ANKRD41	19	17258700	5	0.165723
rs10401700	ANKRD41	19	17258700	6	4.458718
rs10401700	ANKRD41	19	17258700	3	0.240851
SNP19-17273218	ABHD8	19	17273218	5	0.379239
rs10259	DDA1	19	17291915	2	5.117493
rs10259	DDA1	19	17291915	6	4.474352
rs10259	DDA1	19	17291915	5	0.876756
rs1864112	GTPBP3	19	17309961	5	0.809168
rs1864112	GTPBP3	19	17309961	8	2.408564
rs1864112	GTPBP3	19	17309961	6	0.435796
rs1864112	GTPBP3	19	17309961	7	0.219756
rs1864112	GTPBP3	19	17309961	4	0.725793
rs1864112	GTPBP3	19	17309961	3	0.311832
rs3810206	GTPBP3	19	17311016	7	0.408473
rs3810206	GTPBP3	19	17311016	6	0.455214
rs3810206	GTPBP3	19	17311016	3	0.2453
rs3810206	GTPBP3	19	17311016	4	2.417609
rs3810206	GTPBP3	19	17311016	5	0.52229
rs15401	SLC27A1	19	17477747	1	4.179309
rs11878663	FAM129C	19	17512370	3	0.23696
SNP19-17521300	FAM129C	19	17521300	6	2.44781
SNP19-17521300	FAM129C	19	17521300	5	1.212766
SNP19-17521300	FAM129C	19	17521300	8	2.343417
SNP19-17521300	FAM129C	19	17521300	7	0.661419
SNP19-17523616	FAM129C	19	17523616	5	0.232534
rs34631213	FAM129C	19	17525418	3	0.257814
rs34631213	FAM129C	19	17525418	5	0.285998
rs34631213	FAM129C	19	17525418	6	0.613279
rs34631213	FAM129C	19	17525418	7	0.182192
rs7250113	UNC13A	19	17576183	1	3.409462
rs7250113	UNC13A	19	17576183	4	1.316185
rs7250113	UNC13A	19	17576183	7	1.678285
rs7250113	UNC13A	19	17576183	5	0.631364
rs7254755	UNC13A	19	17577582	1	0.273103
rs7254755	UNC13A	19	17577582	4	0.546274
rs7254755	UNC13A	19	17577582	3	0.277756
rs7254755	UNC13A	19	17577582	5	0.218866
rs7254755	UNC13A	19	17577582	7	0.571293
SNP19-17627900	UNC13A	19	17627900	1	1.702328
SNP19-17783666	B3GNT3	19	17783666	8	5.430233
rs265539	B3GNT3	19	17784160	6	0.259406
rs265539	B3GNT3	19	17784160	3	1.275467

rs1003887	INSL3	19	17788400	8	0.159274
rs1003887	INSL3	19	17788400	5	1.158878
rs1003887	INSL3	19	17788400	4	2.667495
rs1003887	INSL3	19	17788400	2	2.965639
rs1003887	INSL3	19	17788400	6	4.608176
rs17750642	INSL3	19	17788562	3	0.488405
rs17750642	INSL3	19	17788562	1	6.080026
rs3008	JAK3	19	17798429	8	0.435567
rs3008	JAK3	19	17798429	5	2.35874
rs7259041	ARRDC2	19	17984738	6	0.971827
rs7259041	ARRDC2	19	17984738	5	0.346342
rs401502	IL12RB1	19	18041413	1	1.370908
SNP19-18041451	IL12RB1	19	18041451	1	1.394601
rs17852635	IL12RB1	19	18047575	1	0.143593
rs740691	MAST3	19	18095441	6	4.725369
rs740691	MAST3	19	18095441	1	0.472669
rs740691	MAST3	19	18095441	3	3.386631
rs740691	MAST3	19	18095441	8	1.194415
rs17683509	FKSG24	19	18167990	5	0.358241
rs1044821	FKSG24	19	18168108	6	7.989897
rs7259012	PDE4C	19	18182273	5	3.809206
rs2229228	PDE4C	19	18190784	5	1.320607
rs999813	KIAA1683	19	18229810	5	0.555182
rs999813	KIAA1683	19	18229810	3	0.528342
rs8103177	KIAA1683	19	18236815	5	0.756171
rs8103177	KIAA1683	19	18236815	3	0.406449
rs2277921	KIAA1683	19	18236846	3	0.381457
rs2277921	KIAA1683	19	18236846	5	0.19913
rs8110972	KIAA1683	19	18237407	4	0.666441
rs8110972	KIAA1683	19	18237407	3	0.846915
rs8110972	KIAA1683	19	18237407	5	0.233657
rs2277922	KIAA1683	19	18237522	5	1.41232
rs2277922	KIAA1683	19	18237522	4	0.13773
rs2277922	KIAA1683	19	18237522	3	0.661766
rs3746186	KIAA1683	19	18238274	5	1.80616
rs3746186	KIAA1683	19	18238274	4	3.948705
rs3746186	KIAA1683	19	18238274	3	2.052685
rs8104533	KIAA1683	19	18238496	3	1.210651
rs8104533	KIAA1683	19	18238496	5	1.849783
rs8104533	KIAA1683	19	18238496	4	0.277411
rs1469023	KIAA1683	19	18239219	5	4.122853
rs1469023	KIAA1683	19	18239219	3	0.806901
rs1469023	KIAA1683	19	18239219	4	1.094711
rs1079166	KIAA1683	19	18246214	4	0.788423
rs1079166	KIAA1683	19	18246214	1	0.025306
rs3746184	PGPEP1	19	18335587	7	0.504384
rs3746184	PGPEP1	19	18335587	5	0.003564

rs1043063	PGPEP1	19	18341171	5	0.64549
rs1043063	PGPEP1	19	18341171	3	0.174306
rs1043063	PGPEP1	19	18341171	2	3.137797
rs1043063	PGPEP1	19	18341171	7	4.386063
rs7226	PGPEP1	19	18341609	8	0.213405
rs7226	PGPEP1	19	18341609	4	2.384524
rs7226	PGPEP1	19	18341609	3	0.063032
rs1059519	GDF15	19	18358024	1	0.393219
rs1059519	GDF15	19	18358024	4	0.099701
rs1059519	GDF15	19	18358024	5	9.602384
rs1059519	GDF15	19	18358024	2	5.558943
rs1059519	GDF15	19	18358024	7	0.588352
rs1059369	GDF15	19	18358141	4	3.126217
rs1059369	GDF15	19	18358141	3	0.285305
rs1059369	GDF15	19	18358141	8	0.62076
rs6512262	LRRC25	19	18363565	5	0.489404
rs1859948	C19orf50	19	18540866	8	9.748118
rs2238647	CRLF1	19	18571535	8	0.258696
rs709679	TMEM59L	19	18587856	5	0.577889
rs1043192	TMEM59L	19	18592658	8	0.359819
rs1076235	KLHL26	19	18636077	2	0.046986
rs1076235	KLHL26	19	18636077	8	0.275328
rs1076235	KLHL26	19	18636077	1	0.61616
rs10410987	UPF1	19	18838937	7	0.089685
rs10410987	UPF1	19	18838937	1	6.504153
rs10416469	UPF1	19	18839250	5	8.264357
rs2074797	COPE	19	18872244	6	0.442402
rs2074797	COPE	19	18872244	4	0.961518
rs2074797	COPE	19	18872244	1	1.453721
rs2231992	DDX49	19	18891571	5	0.207875
rs4808897	SFRS14	19	18966259	5	0.852046
rs4808897	SFRS14	19	18966259	4	7.371745
rs4808921	SLC25A42	19	19082846	5	0.085251
rs4808921	SLC25A42	19	19082846	4	0.215788
rs11729	TMEM161A	19	19091482	1	4.576252
rs11729	TMEM161A	19	19091482	3	0.685701
rs1064395	NCAN	19	19222735	3	0.499156
SNP19-19240549	TM6SF2	19	19240549	6	2.039983
SNP19-19240549	TM6SF2	19	19240549	1	1.255429
rs11555053	SF4	19	19251185	3	0.736681
rs2033481	GATAD2A	19	19478134	4	0.475489
rs2033481	GATAD2A	19	19478134	1	3.2809
rs7250893	TSSK6	19	19486547	6	4.479512
rs7250893	TSSK6	19	19486547	4	5.239385
rs7250893	TSSK6	19	19486547	3	0.5477
rs17684164	NDUFA13	19	19487769	3	2.540434
rs880090	GMIP	19	19601729	7	0.545824

rs880090	GMIP	19	19601729	1	6.266871
rs880090	GMIP	19	19601729	4	0.648949
rs880090	GMIP	19	19601729	3	0.106078
rs35075083	GMIP	19	19608520	7	0.991672
rs12976582	ZNF101	19	19640706	4	1.151161
rs12976582	ZNF101	19	19640706	1	5.053067
rs7246866	ZNF14	19	19683049	2	1.973148
rs16996360	ZNF506	19	19763916	6	0.824066
rs16996360	ZNF506	19	19763916	1	0.412942
rs16996360	ZNF506	19	19763916	2	0.868109
SNP19-1999484€	ZNF682	19	19994846	2	5.387204
SNP19-1999484€	ZNF682	19	19994846	7	1.324869
rs7247683	FLJ44894	19	20367988	2	4.117495
rs7247683	FLJ44894	19	20367988	4	0.942683
rs2701302	FLJ44894	19	20368239	1	0.410305
rs2701302	FLJ44894	19	20368239	5	1.411668
SNP19-2092341	ZNF85	19	20923411	3	0.20001
SNP19-2092341	ZNF85	19	20923411	1	1.501141
SNP19-2092350	ZNF85	19	20923504	3	0.52029
rs488125	ZNF708	19	21266013	7	0.41215
rs488125	ZNF708	19	21266013	8	0.245091
rs489845	ZNF708	19	21266154	3	0.757152
rs8112775	ZNF708	19	21266229	3	0.142792
rs504280	ZNF708	19	21269219	8	3.900647
rs504280	ZNF708	19	21269219	7	0.945017
rs7252798	ZNF100	19	21699893	8	1.74321
rs7252798	ZNF100	19	21699893	3	0.176401
rs4932954	ZNF257	19	22064294	6	1.925873
rs4932954	ZNF257	19	22064294	5	0.859049
rs4932954	ZNF257	19	22064294	1	3.381687
rs10417916	ZNF257	19	22064455	5	0.857843
rs10424582	C19orf12	19	34885561	6	1.951516
rs10424582	C19orf12	19	34885561	4	0.360284
rs10424582	C19orf12	19	34885561	8	4.570165
rs10424582	C19orf12	19	34885561	5	0.009834
rs741445	ZNF507	19	37528432	7	0.457326
rs741445	ZNF507	19	37528432	5	1.358764
rs741445	ZNF507	19	37528432	4	0.055986
rs10500258	ZNF507	19	37567247	5	6.006674
rs7253039	DPY19L3	19	37664917	4	7.255308
rs7247420	ANKRD27	19	37788626	2	4.036281
rs7248273	ANKRD27	19	37788656	2	5.370264
rs2302970	ANKRD27	19	37790472	2	1.563641
rs2302970	ANKRD27	19	37790472	6	0.638756
rs2302970	ANKRD27	19	37790472	5	5.093007
rs2302970	ANKRD27	19	37790472	1	0.114173
rs11881633	TDRD12	19	37973764	3	0.943036

rs11881633	TDRD12	19	37973764	6	0.149264
rs12151363	TDRD12	19	38000870	2	4.820699
rs12151363	TDRD12	19	38000870	6	0.962512
rs12151363	TDRD12	19	38000870	1	0.936974
rs2287884	SLC7A9	19	38013287	4	0.124184
rs2287884	SLC7A9	19	38013287	1	4.669243
rs1007161	SLC7A9	19	38044881	4	0.393205
rs1007161	SLC7A9	19	38044881	2	0.089741
rs1007160	SLC7A9	19	38044901	2	1.371738
rs12150889	SLC7A9	19	38046895	2	4.564111
rs12150889	SLC7A9	19	38046895	4	4.741427
rs12150890	SLC7A9	19	38046909	6	1.665591
rs12150890	SLC7A9	19	38046909	1	1.765908
rs745960	CCDC123	19	38061924	6	0.19927
rs745960	CCDC123	19	38061924	5	0.308284
rs745960	CCDC123	19	38061924	1	0.288064
rs11556219	C19orf40	19	38155068	6	0.651366
rs11556219	C19orf40	19	38155068	4	0.006177
rs11556219	C19orf40	19	38155068	1	4.640946
rs34723366	C19orf40	19	38155087	4	0.324945
rs34723366	C19orf40	19	38155087	1	2.542781
rs2304102	C19orf40	19	38159197	4	0.843837
rs2304102	C19orf40	19	38159197	5	0.661067
rs10410414	RHPN2	19	38190911	7	0.029981
rs10410414	RHPN2	19	38190911	1	5.105452
rs10410414	RHPN2	19	38190911	4	1.500227
rs2287681	GPATCH1	19	38300573	3	0.09088
rs6510356	GPATCH1	19	38309423	3	1.213392
rs6510356	GPATCH1	19	38309423	2	5.484316
rs6510356	GPATCH1	19	38309423	1	0.0118
rs3848596	WDR88	19	38327601	3	0.408475
SNP19-3857067	PEPD	19	38570677	7	0.028869
SNP19-3857067	PEPD	19	38570677	6	5.753987
rs2056180	KCTD15	19	38994889	5	0.298622
rs10418860	LOC643613	19	39205753	3	0.35457
rs10418860	LOC643613	19	39205753	1	0.981597
rs16968855	LOC126235	19	39275498	1	0.691771
rs574004	LOC126235	19	39275977	5	2.595895
rs574004	LOC126235	19	39275977	8	3.837053
rs574004	LOC126235	19	39275977	3	3.228853
SNP19-3939837	LSM14A	19	39398371	4	4.183521
rs392340	KIAA0355	19	39534328	3	0.11038
rs392340	KIAA0355	19	39534328	6	0.721995
rs392340	KIAA0355	19	39534328	5	0.290651
rs1801015	GPI	19	39560482	8	2.523178
rs1801015	GPI	19	39560482	4	0.040606
rs4806060	LOC401913	19	40049178	3	0.45121

SNP19-40126288	ZNF30	19	40126288	6	3.465548
rs2651109	ZNF30	19	40126352	1	0.463049
rs2651109	ZNF30	19	40126352	8	0.135727
rs2651109	ZNF30	19	40126352	4	6.72816
rs2651109	ZNF30	19	40126352	7	0.791159
rs2651109	ZNF30	19	40126352	2	3.597856
rs3761066	ZNF30	19	40127429	5	0.202567
rs3761066	ZNF30	19	40127429	6	0.464554
rs3761066	ZNF30	19	40127429	7	4.316212
rs3761066	ZNF30	19	40127429	1	3.227885
rs1053216	ZNF30	19	40127807	1	5.397637
rs1053216	ZNF30	19	40127807	7	1.153907
rs1053216	ZNF30	19	40127807	5	0.187506
rs1053216	ZNF30	19	40127807	6	0.0376
rs2290647	GRAMD1A	19	40198569	3	1.73693
rs2290647	GRAMD1A	19	40198569	2	3.996946
SNP19-40202144	GRAMD1A	19	40202144	5	0.730251
rs45512696	HPN	19	40242718	5	0.329749
rs1042328	HPN	19	40249100	1	4.532828
rs1042328	HPN	19	40249100	7	1.382894
rs1042328	HPN	19	40249100	5	0.027074
rs1688029	HPN	19	40249280	1	3.471117
rs1688029	HPN	19	40249280	5	0.54511
rs1688029	HPN	19	40249280	7	0.663718
rs7253620	LOC728630	19	40257392	5	1.407634
rs4835	FXYD3	19	40306374	3	0.282766
rs4835	FXYD3	19	40306374	8	7.895872
rs4835	FXYD3	19	40306374	5	0.20354
rs4835	FXYD3	19	40306374	6	1.622982
rs4835	FXYD3	19	40306374	7	0.498126
rs4835	FXYD3	19	40306374	1	0.430946
rs4835	FXYD3	19	40306374	4	1.19095
rs12852	FXYD3	19	40307019	4	1.713997
rs12852	FXYD3	19	40307019	6	0.376877
rs14358	LGI4	19	40307835	7	0.087113
rs14358	LGI4	19	40307835	4	1.345003
rs14358	LGI4	19	40307835	6	3.633037
rs14358	LGI4	19	40307835	1	5.313674
rs14358	LGI4	19	40307835	5	3.737024
rs14358	LGI4	19	40307835	8	0.040066
rs14358	LGI4	19	40307835	3	0.035588
rs1319969	LGI4	19	40309110	7	0.834834
rs3458	FXYD5	19	40352510	6	1.534895
rs3458	FXYD5	19	40352510	4	7.527251
rs564117	TMEM162	19	40410778	2	1.556083
SNP19-40410860	TMEM162	19	40410860	4	7.035326
SNP19-40410860	TMEM162	19	40410860	1	1.512898

SNP19-40410860	TMEM162	19	40410860	5	1.679107
SNP19-40410860	TMEM162	19	40410860	3	1.130976
rs565791	TMEM162	19	40410946	6	0.42146
SNP19-40411417	TMEM162	19	40411417	1	0.430114
rs10416254	LOC643951	19	40415474	5	0.984542
rs10416254	LOC643951	19	40415474	1	0.871516
rs10416254	LOC643951	19	40415474	2	1.198162
rs10416254	LOC643951	19	40415474	3	1.363978
rs10416254	LOC643951	19	40415474	4	5.013596
rs10418562	LOC643951	19	40415512	8	0.533547
rs10418562	LOC643951	19	40415512	1	0.247908
rs10418562	LOC643951	19	40415512	3	0.156077
rs10418562	LOC643951	19	40415512	5	0.493591
rs10418562	LOC643951	19	40415512	4	2.516823
rs10418562	LOC643951	19	40415512	6	1.719273
rs2073900	LSR	19	40433296	3	0.845303
rs2073900	LSR	19	40433296	6	0.801737
GA016224	USF2	19	40462132	2	1.690435
rs10406069	CD22	19	40528370	8	4.558386
rs3088063	CD22	19	40529916	6	0.213065
rs2301151	FFAR1	19	40534926	3	0.547564
rs7976	KRTDAP	19	40670139	7	0.36547
rs7976	KRTDAP	19	40670139	5	0.248414
rs7976	KRTDAP	19	40670139	8	0.0045
rs4806162	DMKN	19	40694550	6	2.728412
rs4806163	DMKN	19	40695946	1	4.901214
rs4806163	DMKN	19	40695946	4	1.505216
rs12460932	DMKN	19	40696011	6	1.538956
rs17705633	SBSN	19	40707492	3	0.024734
SNP19-40709438	SBSN	19	40709438	1	4.963317
SNP19-40721080	GAPDHS	19	40721080	3	1.356598
rs2239945	GAPDHS	19	40725300	2	6.6011
SNP19-40805430	KIAA0841	19	40805430	4	0.340914
rs17705854	KIAA0841	19	40805918	1	4.756512
rs2285421	UPK1A	19	40860754	3	0.331265
rs2285421	UPK1A	19	40860754	4	0.305895
rs2285421	UPK1A	19	40860754	8	1.510843
rs2285421	UPK1A	19	40860754	7	10.11273
rs2285421	UPK1A	19	40860754	6	5.580158
rs2285421	UPK1A	19	40860754	5	6.045457
rs11882996	UPK1A	19	40861125	7	0.462203
rs2227278	ZBTB32	19	40897890	7	0.608457
SNP19-40903582	MLL4	19	40903582	5	0.120257
rs16970649	MLL4	19	40914697	7	0.016536
rs12459634	TMEM149	19	40922014	3	2.030409
rs12459634	TMEM149	19	40922014	6	1.035207
rs12459634	TMEM149	19	40922014	2	4.874865

rs3746278	U2AF1L4	19	40925310	4	1.114498
SNP19-40950561	C19orf55	19	40950561	7	7.212655
SNP19-40951255	C19orf55	19	40951255	8	5.624267
rs231228	SNX26	19	40960611	7	0.634366
rs2291067	SNX26	19	40965374	6	0.558565
rs2291067	SNX26	19	40965374	2	2.55075
rs2291067	SNX26	19	40965374	5	0.389449
rs2291067	SNX26	19	40965374	3	0.654848
SNP19-40966914	SNX26	19	40966914	3	0.476285
SNP19-40966914	SNX26	19	40966914	6	1.06777
rs12462360	PRODH2	19	40994761	1	1.745748
rs12462360	PRODH2	19	40994761	2	2.798838
SNP19-41014441	NPHS1	19	41014441	1	0.978795
rs10420353	THAP8	19	41222172	4	0.402244
rs10421966	THAP8	19	41222183	7	7.895492
rs10421966	THAP8	19	41222183	4	1.407217
rs10421966	THAP8	19	41222183	8	4.657428
SNP19-41222268	THAP8	19	41222268	2	4.619858
SNP19-41241524	WDR62	19	41241524	2	2.29465
rs2301734	WDR62	19	41267485	5	0.655218
rs2301734	WDR62	19	41267485	4	6.536127
rs2285745	WDR62	19	41282169	5	0.289044
rs2285745	WDR62	19	41282169	4	0.926624
SNP19-41286378	WDR62	19	41286378	2	0.746919
SNP19-41286378	WDR62	19	41286378	6	0.464109
SNP19-41286378	WDR62	19	41286378	8	2.207919
SNP19-41286378	WDR62	19	41286378	3	0.210957
SNP19-41286378	WDR62	19	41286378	5	0.44299
SNP19-41286378	WDR62	19	41286378	4	0.238422
SNP19-41286378	WDR62	19	41286378	1	0.116156
rs2072605	LOC728361	19	41295543	2	2.216469
rs2072605	LOC728361	19	41295543	4	0.408402
rs2231569	POLR2I	19	41297715	2	3.856494
rs753420	COX7A1	19	41335535	4	0.986845
rs753420	COX7A1	19	41335535	6	0.460894
rs753420	COX7A1	19	41335535	3	0.369786
rs17206393	ZNF566	19	41630434	2	2.055729
SNP19-41810279	ZNF382	19	41810279	5	4.101533
SNP19-41810279	ZNF382	19	41810279	4	0.066459
SNP19-41810279	ZNF382	19	41810279	3	0.130861
rs1234514	ZNF382	19	41810529	4	0.166868
rs1234514	ZNF382	19	41810529	3	0.05552
rs1234514	ZNF382	19	41810529	5	0.139517
rs7248477	ZNF461	19	41849441	3	0.369801
rs7248477	ZNF461	19	41849441	8	1.064339
rs1673087	LOC728485	19	41956030	2	2.438055
rs1673087	LOC728485	19	41956030	5	0.283132

rs4806370	ZNF345	19	42060727	6	0.060824
rs555023	ZNF345	19	42061294	1	1.602851
rs547483	ZNF568	19	42133205	2	6.45056
rs547483	ZNF568	19	42133205	3	0.669126
rs547483	ZNF568	19	42133205	4	0.452471
rs547483	ZNF568	19	42133205	7	0.863021
rs546589	ZNF568	19	42133302	5	0.18199
rs546589	ZNF568	19	42133302	3	0.362341
rs546589	ZNF568	19	42133302	7	0.54869
rs1644634	ZNF568	19	42133820	5	1.239441
rs1644634	ZNF568	19	42133820	3	0.55047
rs1402468	ZNF420	19	42312088	5	0.280356
rs1402468	ZNF420	19	42312088	7	0.760918
rs1402468	ZNF420	19	42312088	8	0.187704
rs1402468	ZNF420	19	42312088	2	3.065661
rs1402468	ZNF420	19	42312088	3	0.160944
rs2305115	HKR1	19	42527498	6	5.247578
rs2921563	HKR1	19	42545880	6	4.649374
rs12977460	ZNF793	19	42716111	1	1.524346
rs12977460	ZNF793	19	42716111	2	1.88781
rs4803216	ZNF793	19	42725514	2	1.018685
rs4803216	ZNF793	19	42725514	5	2.121677
rs4803216	ZNF793	19	42725514	6	0.418144
rs4803216	ZNF793	19	42725514	1	0.279557
rs2291002	ZNF793	19	42725766	4	1.240995
rs2291002	ZNF793	19	42725766	5	0.498382
rs2291002	ZNF793	19	42725766	8	0.291521
rs7254170	ZFP30	19	42815633	1	0.251192
rs7254170	ZFP30	19	42815633	5	0.861718
rs7259272	ZNF781	19	42874955	7	0.455813
rs7259272	ZNF781	19	42874955	2	2.462664
rs7259272	ZNF781	19	42874955	4	0.917524
rs958305	ZNF607	19	42881280	5	5.651968
rs958305	ZNF607	19	42881280	4	6.027908
rs958305	ZNF607	19	42881280	8	3.486473
rs12461753	ZNF607	19	42894355	7	0.468253
rs17306508	ZNF607	19	42902291	5	0.477145
rs17306508	ZNF607	19	42902291	1	1.181084
rs2075283	LOC728533	19	42975630	6	2.491158
rs2075283	LOC728533	19	42975630	7	4.841095
rs2075283	LOC728533	19	42975630	2	1.893938
rs2075283	LOC728533	19	42975630	3	4.679263
rs241942	LOC644554	19	42999744	2	1.674057
rs16975930	LOC728853	19	43012654	6	0.89894
rs10402530	LOC728853	19	43013478	8	0.340683
rs10402530	LOC728853	19	43013478	5	0.765828
rs10422056	WDR87	19	43070379	3	0.183289

rs10422056	WDR87	19	43070379	1	0.310551
rs10422056	WDR87	19	43070379	5	0.05375
rs6508750	WDR87	19	43071286	1	2.200406
rs6508750	WDR87	19	43071286	7	0.150499
SNP19-43264734	SIPA1L3	19	43264734	2	4.113608
rs3195803	SIPA1L3	19	43390837	7	0.00213
rs3195803	SIPA1L3	19	43390837	1	0.602758
rs34837947	DPF1	19	43398726	4	0.740996
rs11556992	YIF1B	19	43492016	4	7.216425
rs2286475	C19orf15	19	43539271	7	0.259453
rs2286475	C19orf15	19	43539271	6	2.167691
rs2286475	C19orf15	19	43539271	4	1.172937
rs2286475	C19orf15	19	43539271	5	0.194245
rs2286475	C19orf15	19	43539271	3	0.533738
rs1052375	C19orf15	19	43553173	4	0.546666
rs1052375	C19orf15	19	43553173	7	1.735425
rs1052375	C19orf15	19	43553173	2	2.616674
rs1052375	C19orf15	19	43553173	8	1.982985
rs3745961	FAM98C	19	43585942	6	8.034997
rs3745964	FAM98C	19	43591388	6	0.838737
rs4802399	RASGRP4	19	43591839	3	0.235292
SNP19-43602627	RASGRP4	19	43602627	3	0.800466
rs2288888	RYR1	19	43638022	3	1.158505
rs2228069	RYR1	19	43648643	3	2.899573
rs2228069	RYR1	19	43648643	5	2.865325
rs2228069	RYR1	19	43648643	7	3.360518
rs2228070	RYR1	19	43648679	5	2.142935
rs2228070	RYR1	19	43648679	7	0.729145
rs2228070	RYR1	19	43648679	8	1.53256
rs1469698	RYR1	19	43685396	1	3.580428
rs11553600	ACTN4	19	43888576	4	5.639642
rs11553600	ACTN4	19	43888576	1	5.544479
SNP19-43920084	CAPN12	19	43920084	1	2.808236
SNP19-43920084	CAPN12	19	43920084	4	0.365119
rs2229259	ECH1	19	43998943	4	6.684592
rs1001413	FLJ45909	19	44058951	3	0.194757
rs1001413	FLJ45909	19	44058951	4	0.77127
rs730078	SARS2	19	44113228	8	0.586494
rs730078	SARS2	19	44113228	1	1.321051
rs730078	SARS2	19	44113228	5	0.024672
rs730078	SARS2	19	44113228	3	0.39863
rs2304118	FBXO17	19	44132806	5	0.366674
rs2304118	FBXO17	19	44132806	3	0.937528
rs533573	FBXO27	19	44207779	5	3.913998
rs774727	FLJ16165	19	44266877	6	0.140999
rs8111996	FLJ16165	19	44267590	3	0.506069
rs8111996	FLJ16165	19	44267590	5	0.440394

rs45598837	PAK4	19	44351312	8	1.191846
rs45598837	PAK4	19	44351312	3	1.166729
rs31728	PLEKHG2	19	44607598	4	7.007113
rs31728	PLEKHG2	19	44607598	2	4.209217
rs31728	PLEKHG2	19	44607598	7	0.482413
rs31728	PLEKHG2	19	44607598	5	7.391426
rs17628	RPS16	19	44618349	4	0.098357
rs17628	RPS16	19	44618349	2	2.419917
rs17628	RPS16	19	44618349	5	0.614113
rs17795475	EID2B	19	44713599	6	3.056077
rs17795475	EID2B	19	44713599	4	5.156435
rs7258833	LOC400696	19	44866076	3	1.017276
rs7258833	LOC400696	19	44866076	5	0.141288
rs4803286	LOC400696	19	44868676	1	0.595982
rs10755	LGALS14	19	44891754	1	0.360333
rs10755	LGALS14	19	44891754	3	0.911305
rs10755	LGALS14	19	44891754	5	1.607296
rs17608	CLC	19	44917486	7	0.214467
rs3746013	FCGBP	19	45055766	5	0.894646
rs3746013	FCGBP	19	45055766	7	0.292287
rs885723	FCGBP	19	45091270	5	0.948729
rs2111543	ZNF546	19	45211745	1	5.452083
rs2111543	ZNF546	19	45211745	4	0.010221
rs2111544	ZNF546	19	45211775	4	4.701668
rs2111544	ZNF546	19	45211775	1	5.158801
rs2304184	CNTD2	19	45420257	8	4.891454
rs2304184	CNTD2	19	45420257	1	0.123134
rs268674	PRX	19	45592705	4	0.868683
rs3745202	PRX	19	45592851	7	7.22174
rs268673	PRX	19	45593336	7	0.597799
rs268673	PRX	19	45593336	5	0.022519
rs268672	PRX	19	45593444	5	0.495641
rs268672	PRX	19	45593444	7	0.062204
rs268671	PRX	19	45593454	5	0.864875
rs7258094	SPTBN4	19	45704030	1	1.684858
rs7258094	SPTBN4	19	45704030	5	0.436706
rs814501	SPTBN4	19	45730414	6	1.631281
rs814501	SPTBN4	19	45730414	3	0.289723
rs1131620	LTBP4	19	45809709	8	0.200583
rs1131620	LTBP4	19	45809709	2	3.218883
rs1131620	LTBP4	19	45809709	5	0.196388
rs11668767	LTBP4	19	45820149	8	0.76163
rs11668767	LTBP4	19	45820149	5	1.342061
rs7367	LTBP4	19	45821682	2	4.102803
rs7367	LTBP4	19	45821682	8	0.479432
rs7367	LTBP4	19	45821682	5	0.065891
rs12975396	NUMBL	19	45865212	1	5.786368

rs11673492	ADCK4	19	45912369	5	0.994564
rs11673492	ADCK4	19	45912369	1	4.228613
rs10420685	ITPKC	19	45926960	5	0.835508
rs1869710	C19orf54	19	45948215	5	6.070811
rs2604875	SNRPA	19	45955243	5	0.438468
rs2233156	MIA	19	45973540	5	3.414179
rs2545763	EGLN2	19	45999010	5	7.045738
rs3733829	EGLN2	19	46002411	8	0.849237
rs3733829	EGLN2	19	46002411	1	3.258465
rs3733829	EGLN2	19	46002411	7	0.232345
rs3733828	EGLN2	19	46002449	5	0.133207
rs2241720	CCDC97	19	46521149	1	5.319835
rs2241718	CCDC97	19	46521446	1	4.568562
rs1800472	TGFB1	19	46539700	1	3.810993
rs1800471	TGFB1	19	46550716	4	1.211774
rs1800471	TGFB1	19	46550716	6	5.893151
SNP19-4661187(	BCKDHA	19	46611870	3	0.196554
rs284661	B3GNT8	19	46623960	4	0.875191
rs284661	B3GNT8	19	46623960	5	1.18657
rs284661	B3GNT8	19	46623960	3	1.233538
rs284662	B3GNT8	19	46624115	3	0.876464
rs284662	B3GNT8	19	46624115	5	1.172453
rs284662	B3GNT8	19	46624115	4	0.136334
rs284662	B3GNT8	19	46624115	7	1.271802
rs284663	B3GNT8	19	46624452	4	0.206455
rs284663	B3GNT8	19	46624452	7	0.82148
rs284663	B3GNT8	19	46624452	3	0.590075
rs284663	B3GNT8	19	46624452	5	0.720653
rs1043413	ATP5SL	19	46631137	4	2.615656
rs1043413	ATP5SL	19	46631137	7	0.160953
rs1043413	ATP5SL	19	46631137	3	0.246866
rs1043413	ATP5SL	19	46631137	5	0.715951
rs714106	CEACAM21	19	46775689	3	0.852237
rs714106	CEACAM21	19	46775689	5	1.957594
rs714106	CEACAM21	19	46775689	1	1.767435
rs1041994	CEACAM4	19	46817500	1	1.501074
rs1041994	CEACAM4	19	46817500	5	0.299303
rs1041994	CEACAM4	19	46817500	4	2.492001
rs8102488	CEACAM7	19	46882699	3	1.124918
rs899662	CEACAM3	19	47007121	7	0.537492
rs2217342	ATP1A3	19	47181356	1	2.030871
rs11557114	ERF	19	47445489	1	5.721392
rs1206038	MEGF8	19	47554875	1	4.91415
GA034671	MEGF8	19	47573935	1	4.959151
rs11673645	CXCL17	19	47638953	1	3.367037
SNP19-4772335	CEACAM1	19	47723354	7	6.762188
SNP19-4772335	CEACAM1	19	47723354	5	0.706284

SNP19-47723354	CEACAM1	19	47723354	1	1.72095
SNP19-47723354	CEACAM1	19	47723354	6	1.105065
SNP19-47723354	CEACAM1	19	47723354	2	3.83994
SNP19-47723354	CEACAM1	19	47723354	3	1.565545
SNP19-47723354	CEACAM1	19	47723354	8	5.520143
SNP19-47723354	CEACAM1	19	47723354	4	0.98719
rs12459171	PSG1	19	48075641	3	1.350813
rs12459171	PSG1	19	48075641	4	0.73285
rs12459171	PSG1	19	48075641	1	3.734182
rs3746096	PSG2	19	48267714	5	1.732116
GA034678	CD177	19	48549906	1	2.337513
GA034678	CD177	19	48549906	3	0.528625
GA034681	CD177	19	48558289	3	0.209239
GA034681	CD177	19	48558289	1	3.210707
rs7257560	CD177	19	48558663	3	0.131766
rs7257560	CD177	19	48558663	1	0.236674
rs7257560	CD177	19	48558663	2	0.7854
rs7257560	CD177	19	48558663	5	0.473074
rs11673673	TEX101	19	48602489	1	0.025406
SNP19-48612452	TEX101	19	48612452	3	0.053161
rs3816438	TEX101	19	48613901	1	1.492189
rs3816438	TEX101	19	48613901	4	0.123772
rs1133929	PHLDB3	19	48675580	7	0.39959
rs2682547	NULL	19	48692897	5	0.699324
rs2256507	LOC729244	19	48730146	7	0.501358
rs915927	XRCC1	19	48749067	6	0.306323
rs915927	XRCC1	19	48749067	2	2.164216
rs1799782	XRCC1	19	48749414	6	0.850499
rs1799782	XRCC1	19	48749414	8	0.627737
rs3746002	ZNF428	19	48815734	8	0.005118
rs3746002	ZNF428	19	48815734	2	4.241057
rs3746002	ZNF428	19	48815734	5	0.151037
rs3746002	ZNF428	19	48815734	7	0.765465
rs349046	LYPD5	19	48992466	1	3.805511
SNP19-48994464	LYPD5	19	48994464	5	0.614686
rs11547806	LYPD5	19	48994906	6	0.873753
rs11547806	LYPD5	19	48994906	1	4.627814
rs11547806	LYPD5	19	48994906	5	1.091801
rs11547806	LYPD5	19	48994906	7	6.462532
SNP19-49044506	ZNF283	19	49044506	6	6.207747
SNP19-49044506	ZNF283	19	49044506	4	7.545035
SNP19-49044506	ZNF283	19	49044506	1	0.058379
SNP19-49044506	ZNF283	19	49044506	3	0.17601
SNP19-49044506	ZNF283	19	49044506	2	2.786513
rs365745	ZNF221	19	49163049	1	4.449379
SNP19-49163092	ZNF221	19	49163092	1	2.838737
rs11883068	ZNF230	19	49208804	1	6.163752

rs11883068	ZNF230	19	49208804	7	1.128861
SNP19-4922783	ZNF222	19	49227839	6	6.298726
SNP19-4922783	ZNF222	19	49227839	7	0.107294
SNP19-4922783	ZNF222	19	49227839	5	2.614403
SNP19-4922783	ZNF222	19	49227839	8	0.173784
SNP19-4922783	ZNF222	19	49227839	1	5.810243
SNP19-4930268	ZNF224	19	49302683	6	0.385311
SNP19-4930268	ZNF224	19	49302683	1	3.689111
rs4508518	ZNF224	19	49303260	8	6.930885
rs1105561	ZNF226	19	49361800	3	0.433946
rs1105561	ZNF226	19	49361800	7	5.202989
rs1105561	ZNF226	19	49361800	8	0.226941
rs2884016	ZNF233	19	49470639	1	5.18235
rs4280359	ZNF228	19	49525691	3	0.045108
rs4280359	ZNF228	19	49525691	7	1.202767
rs4280359	ZNF228	19	49525691	1	7.099659
rs4280359	ZNF228	19	49525691	6	1.68354
rs4280359	ZNF228	19	49525691	8	0.698818
rs2734455	ZNF229	19	49622375	1	6.458318
rs2734455	ZNF229	19	49622375	3	0.617841
rs1062093	ZNF229	19	49623835	6	4.709345
rs1062093	ZNF229	19	49623835	7	0.676139
rs1062093	ZNF229	19	49623835	2	7.9389
rs1062093	ZNF229	19	49623835	4	0.537894
rs1897824	ZNF285B	19	49668911	3	3.722328
rs1897824	ZNF285B	19	49668911	7	3.62928
rs2020083	ZNF180	19	49673365	5	1.24749
rs2020083	ZNF180	19	49673365	3	0.941002
rs2253563	ZNF180	19	49675407	7	1.671115
rs2253563	ZNF180	19	49675407	4	0.706938
rs2253563	ZNF180	19	49675407	5	0.071206
rs17714646	FLJ41856	19	49734335	7	1.035916
rs17714646	FLJ41856	19	49734335	1	0.389623
rs7250946	LOC147710	19	49831860	4	0.375549
rs2289494	CEACAM19	19	49878805	3	0.311717
rs2289494	CEACAM19	19	49878805	2	4.696503
rs3729640	PVRL2	19	50073757	5	0.3468
rs3729640	PVRL2	19	50073757	4	1.024771
rs3729640	PVRL2	19	50073757	2	3.319033
rs3729640	PVRL2	19	50073757	3	1.146336
rs157581	TOMM40	19	50087554	1	0.898704
rs157581	TOMM40	19	50087554	7	1.37242
rs157581	TOMM40	19	50087554	2	0.700631
rs157581	TOMM40	19	50087554	6	0.081811
rs157581	TOMM40	19	50087554	3	0.260244
SNP19-5009851	TOMM40	19	50098513	3	0.676361
SNP19-5009851	TOMM40	19	50098513	1	0.970376

SNP19-50098513	TOMM40	19	50098513	7	1.122318
SNP19-50098513	TOMM40	19	50098513	5	0.766839
SNP19-50098513	TOMM40	19	50098513	2	8.536758
rs1064725	APOC1	19	50114401	7	1.969573
rs1064725	APOC1	19	50114401	6	0.394794
rs1132899	APOC4	19	50139876	6	2.062309
rs1132899	APOC4	19	50139876	1	0.721541
rs1132899	APOC4	19	50139876	3	0.735522
rs5167	APOC4	19	50140305	7	0.529979
rs5167	APOC4	19	50140305	2	1.885411
rs5167	APOC4	19	50140305	1	0.338626
rs5167	APOC4	19	50140305	3	0.386716
rs3178166	GEMIN7	19	50286010	1	1.916491
rs3178166	GEMIN7	19	50286010	5	1.122464
rs3178166	GEMIN7	19	50286010	7	1.507671
rs3178166	GEMIN7	19	50286010	2	1.767186
rs17642665	GEMIN7	19	50286344	6	1.530645
rs17642665	GEMIN7	19	50286344	1	5.188041
rs17642665	GEMIN7	19	50286344	5	0.352236
rs3745157	GEMIN7	19	50286435	6	1.581306
rs3745157	GEMIN7	19	50286435	7	1.243658
rs3745157	GEMIN7	19	50286435	8	2.333362
rs11667430	EXOC3L2	19	50408037	3	0.301502
rs11667430	EXOC3L2	19	50408037	2	0.038787
rs11667430	EXOC3L2	19	50408037	1	0.391053
rs11667430	EXOC3L2	19	50408037	5	0.422318
rs11667430	EXOC3L2	19	50408037	6	0.888136
rs2395	MARK4	19	50498449	7	0.176253
rs2395	MARK4	19	50498449	5	4.735054
rs2395	MARK4	19	50498449	3	0.035102
rs10415720	MARK4	19	50499771	1	0.542483
rs10415720	MARK4	19	50499771	7	0.135539
rs10415720	MARK4	19	50499771	5	0.431966
rs12971645	MARK4	19	50499785	1	0.770679
rs4884	CKM	19	50501875	5	4.54897
rs4884	CKM	19	50501875	3	0.433112
rs4884	CKM	19	50501875	7	1.336089
rs17875616	CKM	19	50506873	4	4.44981
rs17875616	CKM	19	50506873	3	4.443688
rs1133190	CKM	19	50510675	3	0.795932
rs967591	CD3EAP	19	50601774	3	0.482308
rs1007616	CD3EAP	19	50604933	1	0.3802
rs1007616	CD3EAP	19	50604933	8	0.015575
rs28381239	FOSB	19	50663206	7	1.114393
rs28381239	FOSB	19	50663206	5	0.91322
rs28381239	FOSB	19	50663206	1	0.878216
rs2282695	FOSB	19	50663801	5	7.122887

rs708905	FOSB	19	50669312	4	1.583591
rs708905	FOSB	19	50669312	2	5.908739
rs3745821	FLJ40125	19	50694296	3	0.376095
rs3745821	FLJ40125	19	50694296	1	0.731551
rs3745821	FLJ40125	19	50694296	7	1.634685
rs2301283	VASP	19	50716424	3	0.865379
rs1045217	GPR4	19	50785051	5	0.954689
rs1045217	GPR4	19	50785051	3	1.238132
rs3745818	GPR4	19	50787491	3	0.698165
rs3745818	GPR4	19	50787491	5	0.625089
rs1800437	GIPR	19	50873232	6	0.91848
rs1800437	GIPR	19	50873232	4	0.731312
rs1800437	GIPR	19	50873232	2	4.088482
rs1800437	GIPR	19	50873232	7	0.203662
rs1800437	GIPR	19	50873232	3	0.317017
rs1800437	GIPR	19	50873232	5	0.597829
rs12709891	GIPR	19	50877057	8	2.388007
rs2014576	SIX5	19	50960916	1	0.372711
rs2014576	SIX5	19	50960916	4	0.453441
rs2014576	SIX5	19	50960916	3	0.578621
GA034759	DMWD	19	50981349	6	2.01381
rs2303014	SYMPK	19	51043962	7	0.705415
rs2303014	SYMPK	19	51043962	8	0.187071
rs2303014	SYMPK	19	51043962	5	0.520515
rs2303014	SYMPK	19	51043962	6	2.491293
rs16980091	FOXA3	19	51067619	5	1.716925
rs16980091	FOXA3	19	51067619	1	0.734871
rs16980091	FOXA3	19	51067619	6	0.965609
rs16980091	FOXA3	19	51067619	3	1.087185
rs10423899	LOC339344	19	51096419	1	4.538735
rs10423899	LOC339344	19	51096419	6	0.272337
rs8111011	NANOS2	19	51108487	3	0.448953
rs8111011	NANOS2	19	51108487	5	0.476852
rs1422627	NANOS2	19	51108578	3	0.467976
rs1047061	NOVA2	19	51135726	7	2.198433
rs1047061	NOVA2	19	51135726	3	1.660838
rs1047061	NOVA2	19	51135726	1	3.069475
SNP19-51210522	CCDC61	19	51210522	5	0.391049
SNP19-51210522	CCDC61	19	51210522	1	3.309433
rs17271272	IGFL4	19	51235406	1	3.155183
rs10408162	IGFL2	19	51356240	5	0.974886
SNP19-51425249	IGFL1	19	51425249	1	0.665979
SNP19-51425249	IGFL1	19	51425249	2	0.020244
SNP19-51425249	IGFL1	19	51425249	8	5.150072
SNP19-51504291	HIF3A	19	51504291	7	0.506464
rs3764609	HIF3A	19	51515542	6	1.264281
rs917948	PPP5C	19	51548855	7	0.297406

GA034771	PNMAL1	19	51665821	4	1.694643
GA034771	PNMAL1	19	51665821	7	2.435816
GA034771	PNMAL1	19	51665821	6	1.376743
rs12978825	PNMAL1	19	51666146	4	2.17971
rs12978825	PNMAL1	19	51666146	7	2.191911
rs12978825	PNMAL1	19	51666146	6	2.364684
rs4803967	LOC400707	19	51690076	6	0.570424
rs12974613	GNG8	19	51829299	7	0.437272
rs12974613	GNG8	19	51829299	3	2.672764
rs12974613	GNG8	19	51829299	1	3.20234
rs313841	STRN4	19	51915789	1	6.534516
rs2070246	SLC1A5	19	51974002	3	0.604642
rs2070246	SLC1A5	19	51974002	4	0.098638
rs2070246	SLC1A5	19	51974002	6	3.455257
rs3027952	SLC1A5	19	51983410	3	0.754502
rs10401590	LOC390948	19	52005171	5	1.03367
rs3745615	NPAS1	19	52235614	7	0.572808
rs3745615	NPAS1	19	52235614	1	1.102975
rs3745615	NPAS1	19	52235614	6	0.066232
rs889169	NPAS1	19	52240518	4	0.491948
rs889169	NPAS1	19	52240518	6	0.134133
rs889169	NPAS1	19	52240518	1	2.920085
rs12985905	CCDC9	19	52453486	8	0.56309
rs12985905	CCDC9	19	52453486	7	1.218885
rs12985905	CCDC9	19	52453486	3	0.274908
rs1064202	DHX34	19	52547980	1	6.283566
rs12984558	DHX34	19	52548176	6	0.128695
rs12984558	DHX34	19	52548176	5	0.4893
rs2245046	DHX34	19	52550264	7	0.824937
rs2694558	DHX34	19	52571077	7	0.178166
rs2547378	DHX34	19	52577594	7	0.175858
rs3745173	SLC8A2	19	52624200	6	0.454958
rs3745173	SLC8A2	19	52624200	3	0.879717
rs3745173	SLC8A2	19	52624200	4	2.235168
rs2272295	KPTN	19	52670483	6	1.130919
rs12972658	ZNF541	19	52734788	6	0.112954
rs2914439	GLTSCR1	19	52874320	3	1.181852
rs2914439	GLTSCR1	19	52874320	1	0.582928
rs2914439	GLTSCR1	19	52874320	4	0.298102
rs2914439	GLTSCR1	19	52874320	5	0.005841
rs2914439	GLTSCR1	19	52874320	2	0.535404
rs2911005	GLTSCR1	19	52874380	4	0.456008
rs2911005	GLTSCR1	19	52874380	7	1.161611
rs2911005	GLTSCR1	19	52874380	5	0.628803
rs2911005	GLTSCR1	19	52874380	2	2.009608
rs2911005	GLTSCR1	19	52874380	8	0.422999
SNP19-5295159	GLTSCR2	19	52951599	6	0.705192

SNP19-52951599	GLTSCR2	19	52951599	4	1.044
rs8101799	LOC729626	19	52963864	4	0.023708
rs8101799	LOC729626	19	52963864	7	0.80868
rs8106690	LOC390949	19	52989690	2	1.244877
rs8106690	LOC390949	19	52989690	7	2.046172
rs35445888	TPRX1	19	52996817	3	0.856448
rs7248427	CRX	19	53037973	3	0.748141
rs7248427	CRX	19	53037973	5	0.78912
rs11670620	CRX	19	53038337	5	1.2907
rs11670620	CRX	19	53038337	4	2.373819
rs10409288	ELSPBP1	19	53189753	8	2.758346
rs35535557	CABP5	19	53236649	3	1.351657
rs35535557	CABP5	19	53236649	5	2.195536
rs1045376	PLA2G4C	19	53243221	1	1.708058
rs9226	PLA2G4C	19	53243358	8	3.709672
rs9226	PLA2G4C	19	53243358	7	0.022086
rs9226	PLA2G4C	19	53243358	3	1.673264
rs251684	PLA2G4C	19	53293266	2	3.789469
rs251684	PLA2G4C	19	53293266	5	2.309731
rs2307279	PLA2G4C	19	53300410	5	3.097803
rs2307279	PLA2G4C	19	53300410	4	3.342312
rs11564500	PLA2G4C	19	53305809	3	0.516466
rs20581	LIG1	19	53314239	3	0.060885
rs20581	LIG1	19	53314239	6	3.126888
rs20579	LIG1	19	53360642	1	3.072007
rs20579	LIG1	19	53360642	7	0.138578
rs1968441	CARD8	19	53403601	8	6.593567
rs1968440	CARD8	19	53403714	8	1.672612
rs2043211	CARD8	19	53429518	3	0.27353
rs2043211	CARD8	19	53429518	4	0.232646
rs2043211	CARD8	19	53429518	6	7.546446
rs2043211	CARD8	19	53429518	5	6.176303
rs2242463	CCDC114	19	53498789	2	4.664289
rs2242463	CCDC114	19	53498789	4	0.255952
rs8355	EMP3	19	53525612	2	2.647385
rs8355	EMP3	19	53525612	4	0.493695
rs8355	EMP3	19	53525612	1	0.412134
rs8355	EMP3	19	53525612	7	0.611346
rs3826828	TMEM143	19	53528485	6	0.312158
rs3826828	TMEM143	19	53528485	7	0.828376
rs919804	SYNGR4	19	53560990	5	0.872941
rs1050671	KDELR1	19	53577730	7	2.897702
rs1050671	KDELR1	19	53577730	2	7.046717
rs8111684	GRIN2D	19	53639138	3	0.77644
rs8111684	GRIN2D	19	53639138	2	0.484477
rs2544794	SULT2B1	19	53771058	3	0.151483
rs2544794	SULT2B1	19	53771058	1	5.195132

rs368654	FAM83E	19	53808367	7	0.415163
rs368654	FAM83E	19	53808367	1	2.821309
rs443527	FAM83E	19	53808572	6	3.200786
rs443527	FAM83E	19	53808572	2	3.211754
rs443527	FAM83E	19	53808572	4	4.063772
rs281391	LOC126147	19	53856552	2	2.636653
rs281391	LOC126147	19	53856552	5	0.101063
rs3826838	LOC653677	19	53875096	2	2.491825
rs1800027	FUT2	19	53898538	1	4.874287
rs569970	FUT2	19	53899530	4	0.790837
rs569970	FUT2	19	53899530	1	3.349073
rs569970	FUT2	19	53899530	8	0.606275
rs569970	FUT2	19	53899530	6	0.746748
rs281385	FLJ36070	19	53909073	1	3.102211
rs281385	FLJ36070	19	53909073	5	0.499899
SNP19-5390992	FLJ36070	19	53909923	6	0.382993
SNP19-5390992	FLJ36070	19	53909923	1	2.42262
SNP19-5390992	FLJ36070	19	53909923	4	0.196868
SNP19-5390992	FLJ36070	19	53909923	5	0.723631
rs2307019	IZUMO1	19	53936032	2	1.629362
rs2307019	IZUMO1	19	53936032	1	2.156817
rs2307019	IZUMO1	19	53936032	5	2.088333
rs2307019	IZUMO1	19	53936032	6	1.360512
rs2307019	IZUMO1	19	53936032	8	2.358126
rs2307019	IZUMO1	19	53936032	4	2.044268
rs8108468	IZUMO1	19	53939598	4	2.420338
rs8108468	IZUMO1	19	53939598	2	0.697885
rs8108468	IZUMO1	19	53939598	8	2.257119
rs8108468	IZUMO1	19	53939598	1	1.779931
rs8108468	IZUMO1	19	53939598	6	1.230531
rs8108468	IZUMO1	19	53939598	5	2.371274
rs12975781	IZUMO1	19	53941510	1	0.135756
rs12975781	IZUMO1	19	53941510	8	0.528277
rs12975781	IZUMO1	19	53941510	5	0.267618
rs8102032	IZUMO1	19	53941607	1	0.485441
rs8102032	IZUMO1	19	53941607	5	0.623961
rs8102032	IZUMO1	19	53941607	6	1.588129
rs8104897	IZUMO1	19	53941626	1	0.396136
rs8105137	IZUMO1	19	53941700	1	0.807774
rs8105137	IZUMO1	19	53941700	5	1.046952
rs8105137	IZUMO1	19	53941700	6	0.011114
rs12611028	FUT1	19	53944801	2	0.78037
rs12611028	FUT1	19	53944801	6	0.421645
rs12611028	FUT1	19	53944801	1	0.517198
rs4015	FUT1	19	53945201	1	0.266018
rs4015	FUT1	19	53945201	2	0.3943
rs4015	FUT1	19	53945201	6	2.305524

SNP19-53953180	FGF21	19	53953180	1	0.922465
rs8110220	HSD17B14	19	54030910	1	2.847564
rs506425	PLEKHA4	19	54060655	8	0.893699
rs506425	PLEKHA4	19	54060655	6	0.217898
SNP19-54068396	PPP1R15A	19	54068396	4	0.134882
SNP19-54068396	PPP1R15A	19	54068396	3	0.420629
rs557806	PPP1R15A	19	54069054	6	6.388899
rs556052	PPP1R15A	19	54069248	6	0.354165
rs8112811	TULP2	19	54083216	5	1.43562
rs1058483	NUCB1	19	54099472	8	3.371448
rs2270941	DHDH	19	54130175	5	0.025087
rs2270939	DHDH	19	54134745	4	4.09275
rs2270939	DHDH	19	54134745	5	5.197867
rs10423255	DHDH	19	54137586	4	1.085012
rs11666105	DHDH	19	54137629	5	1.434963
rs11666105	DHDH	19	54137629	4	0.382647
rs2287754	GYS1	19	54188306	4	0.477863
rs2287754	GYS1	19	54188306	7	0.625263
rs12461085	CGB7	19	54250541	8	4.894445
rs12461085	CGB7	19	54250541	6	2.9833
rs12461085	CGB7	19	54250541	4	1.39452
rs12461085	CGB7	19	54250541	2	2.120545
rs12461085	CGB7	19	54250541	7	0.15194
rs12461088	CGB7	19	54250591	7	3.775237
rs17206784	KCNA7	19	54262925	3	0.008448
rs11673003	KCNA7	19	54263769	3	0.257603
rs11083959	KCNA7	19	54265018	8	0.40649
rs11083959	KCNA7	19	54265018	4	0.430956
rs11083959	KCNA7	19	54265018	1	0.773435
rs1017219	KCNA7	19	54265250	1	0.836855
rs3810188	KCNA7	19	54265330	3	0.545665
rs1611775	KCNA7	19	54265937	4	4.070889
rs1611775	KCNA7	19	54265937	2	0.638696
rs1611775	KCNA7	19	54265937	6	0.521118
rs1611775	KCNA7	19	54265937	8	5.957569
rs1611775	KCNA7	19	54265937	1	0.045584
rs4802551	SNRP70	19	54280367	3	0.878241
rs2232003	FLJ10490	19	54313776	7	0.614864
rs2232003	FLJ10490	19	54313776	5	3.165629
rs2232003	FLJ10490	19	54313776	3	0.72224
rs2232003	FLJ10490	19	54313776	8	0.07032
rs2303053	PPFIA3	19	54331814	5	0.114996
rs2303053	PPFIA3	19	54331814	7	0.402871
SNP19-54350021	HRC	19	54350021	1	0.725828
SNP19-54350021	HRC	19	54350021	5	0.561836
SNP19-54350021	HRC	19	54350021	7	0.439132
rs3745298	HRC	19	54350179	5	1.14125

rs3745299	HRC	19	54350202	7	2.292147
rs3745299	HRC	19	54350202	5	1.673253
rs1054770	DKKL1	19	54569927	7	0.504371
rs1054770	DKKL1	19	54569927	5	0.425524
rs2288481	DKKL1	19	54570008	5	0.953113
rs2288481	DKKL1	19	54570008	6	0.383714
rs2288481	DKKL1	19	54570008	7	0.789003
rs10421748	FLJ32658	19	54593130	6	0.574836
rs10421748	FLJ32658	19	54593130	5	1.362086
rs1043558	SLC17A7	19	54624574	5	0.060779
rs13394	PIH1D1	19	54642110	4	0.174281
rs13394	PIH1D1	19	54642110	5	4.51212
rs13394	PIH1D1	19	54642110	3	1.081227
rs1320303	ALDH16A1	19	54656789	3	0.854501
rs1320303	ALDH16A1	19	54656789	5	6.068919
rs2293009	ALDH16A1	19	54659492	1	0.809956
rs2293009	ALDH16A1	19	54659492	7	0.850011
rs2293009	ALDH16A1	19	54659492	3	0.583203
GA034912	ALDH16A1	19	54659793	1	2.343837
GA034912	ALDH16A1	19	54659793	5	0.407034
GA034912	ALDH16A1	19	54659793	4	0.18962
rs11669553	RPL13A	19	54687348	5	0.09311
rs2878342	FCGRT	19	54709536	7	0.153004
rs2878342	FCGRT	19	54709536	1	3.488325
rs3745474	PRRG2	19	54778618	7	1.553996
rs11083979	PRR12	19	54787956	7	0.702124
rs11083979	PRR12	19	54787956	3	0.538742
rs3745475	PRR12	19	54792107	3	0.346068
rs3745475	PRR12	19	54792107	7	0.941189
rs2304206	BCL2L12	19	54860683	1	5.321674
rs2304206	BCL2L12	19	54860683	8	0.877578
rs2304206	BCL2L12	19	54860683	7	0.812403
rs2304204	IRF3	19	54860832	1	3.926681
rs2304204	IRF3	19	54860832	8	4.410667
rs2304204	IRF3	19	54860832	7	0.738954
rs2305921	FUZ	19	55004465	6	0.200097
SNP19-55052801	PTOV1	19	55052801	3	0.108009
SNP19-55052801	PTOV1	19	55052801	1	1.951742
rs731826	TBC1D17	19	55072670	3	1.13234
rs731826	TBC1D17	19	55072670	5	0.302954
rs731826	TBC1D17	19	55072670	2	3.891917
rs731826	TBC1D17	19	55072670	4	1.169178
rs731826	TBC1D17	19	55072670	7	0.735227
rs3745486	TBC1D17	19	55075448	5	1.439509
rs3745486	TBC1D17	19	55075448	3	1.672656
rs3745486	TBC1D17	19	55075448	4	1.3644
rs1290754	IL4I1	19	55096751	7	0.326875

rs1290754	IL4I1	19	55096751	3	0.434416
rs1290754	IL4I1	19	55096751	1	0.501451
rs9304690	SIGLEC11	19	55145129	7	2.349317
rs2278410	VRK3	19	55220530	7	0.317894
rs2278410	VRK3	19	55220530	6	0.743491
rs2278410	VRK3	19	55220530	5	0.242212
rs3745504	MYH14	19	55463421	6	0.110409
rs3745504	MYH14	19	55463421	1	0.695405
rs3745504	MYH14	19	55463421	2	0.761938
rs3745504	MYH14	19	55463421	3	1.965728
rs11669191	MYH14	19	55471894	3	0.843248
rs11669191	MYH14	19	55471894	4	0.94579
SNP19-5555734	NAPSA	19	55557347	5	1.369177
rs1137895	SPIB	19	55623776	7	0.750603
rs25668	MYBPC2	19	55657084	7	1.387661
rs25668	MYBPC2	19	55657084	1	1.977096
rs25668	MYBPC2	19	55657084	3	0.505304
rs889131	LOC554235	19	55709647	3	1.5379
rs889131	LOC554235	19	55709647	7	1.307102
rs889131	LOC554235	19	55709647	1	0.524196
rs2287820	SYT3	19	55832501	3	0.377735
rs2287819	SYT3	19	55832564	3	0.545972
rs41275782	SHANK1	19	55864338	8	0.934546
SNP19-5598963	ACPT	19	55989638	1	0.266219
SNP19-5598963	ACPT	19	55989638	2	0.40575
SNP19-5598963	ACPT	19	55989638	5	0.09429
SNP19-5598963	ACPT	19	55989638	8	4.46927
rs3745526	C19orf48	19	55994014	5	0.60181
rs3745526	C19orf48	19	55994014	8	3.806372
rs3745526	C19orf48	19	55994014	1	0.242642
SNP19-5601504	KLK1	19	56015044	7	0.111253
SNP19-5601504	KLK1	19	56015044	5	0.823566
SNP19-5601504	KLK1	19	56015044	2	2.437977
rs5516	KLK1	19	56015285	6	4.852678
rs5516	KLK1	19	56015285	1	0.559085
rs5516	KLK1	19	56015285	2	0.180378
rs5516	KLK1	19	56015285	3	0.145776
rs5515	KLK1	19	56015488	8	4.930217
rs3212810	KLK15	19	56020548	5	0.432252
rs2659122	KLK3	19	56054838	1	0.302506
rs1058205	KLK3	19	56055210	1	1.478683
rs1058205	KLK3	19	56055210	7	2.132951
rs1058274	KLK3	19	56055260	7	1.129458
rs1058274	KLK3	19	56055260	5	1.810118
rs1058274	KLK3	19	56055260	1	2.106722
rs198972	KLK2	19	56071705	1	1.055684
rs198972	KLK2	19	56071705	5	0.95215

rs198972	KLK2	19	56071705	8	1.332008
SNP19-56073589	KLK2	19	56073589	4	1.367166
rs1654552	KLK4	19	56104478	7	0.274901
rs1701950	KLK6	19	56156908	1	0.016746
rs1701950	KLK6	19	56156908	2	0.417004
rs1654528	KLK7	19	56172652	2	2.634828
rs10404395	KLK9	19	56197908	8	0.182258
rs10404395	KLK9	19	56197908	3	0.046605
rs2659096	KLK9	19	56197960	1	0.374085
rs2659096	KLK9	19	56197960	5	6.312746
rs2659096	KLK9	19	56197960	8	6.900748
rs2659096	KLK9	19	56197960	4	0.844996
rs2569454	KLK10	19	56215015	2	0.366319
rs1048328	KLK11	19	56219176	4	2.402747
rs8104554	KLK12	19	56229928	1	1.573019
rs8104554	KLK12	19	56229928	4	2.621028
rs8104554	KLK12	19	56229928	6	4.426856
rs2569491	KLK14	19	56276728	1	2.501996
rs2569491	KLK14	19	56276728	7	0.538258
rs2569491	KLK14	19	56276728	3	1.251055
rs35287116	KLK14	19	56276763	7	0.081353
rs17801681	SIGLECP3	19	56368461	4	0.634135
rs35112940	CD33	19	56430729	5	0.005321
rs3887787	LOC402665	19	56525816	7	2.932508
rs3887787	LOC402665	19	56525816	2	2.351183
rs3009	NKG7	19	56566762	5	0.856526
rs3009	NKG7	19	56566762	7	0.677227
rs3810092	LOC147646	19	56583369	3	0.009899
rs3826657	LOC147646	19	56583660	7	0.515819
rs3826657	LOC147646	19	56583660	5	4.425705
rs3826657	LOC147646	19	56583660	1	5.281016
rs3826659	LOC147646	19	56583721	1	1.836421
rs3826659	LOC147646	19	56583721	5	0.199187
rs3826660	LOC147646	19	56583794	1	2.721494
rs3826660	LOC147646	19	56583794	7	1.967002
rs3826660	LOC147646	19	56583794	5	1.773043
rs3826663	LOC147646	19	56583847	5	0.036934
rs3826663	LOC147646	19	56583847	7	0.227517
rs3826663	LOC147646	19	56583847	1	3.365049
rs3826664	LOC147646	19	56583921	7	1.055786
rs3826665	LOC147646	19	56584030	5	0.140023
rs3826665	LOC147646	19	56584030	1	2.424362
rs1974821	SIGLEC10	19	56609547	5	0.018871
rs1974821	SIGLEC10	19	56609547	3	0.260023
rs36491	SIGLEC8	19	56647438	2	1.050309
rs36491	SIGLEC8	19	56647438	5	3.474353
SNP19-56724970	SIGLEC6	19	56724970	6	0.53441

SNP19-56724970	SIGLEC6	19	56724970	1	1.380369
SNP19-56724970	SIGLEC6	19	56724970	7	0.822173
rs1551559	SIGLEC6	19	56726752	1	0.46442
rs1551559	SIGLEC6	19	56726752	4	1.153976
SNP19-56781826	ZNF175	19	56781826	2	1.190524
SNP19-56781826	ZNF175	19	56781826	3	0.612862
rs12975196	FLJ30403	19	56786249	2	1.030344
rs12975196	FLJ30403	19	56786249	6	0.653065
rs12975196	FLJ30403	19	56786249	1	1.556163
rs6509556	FLJ30403	19	56786328	1	2.352014
rs7259649	FLJ30403	19	56787253	6	0.96404
rs7259649	FLJ30403	19	56787253	1	0.371807
rs7259649	FLJ30403	19	56787253	8	0.860877
rs7259649	FLJ30403	19	56787253	2	0.529433
rs3794983	FLJ30403	19	56787865	7	0.663232
rs3794982	FLJ30403	19	56788082	7	0.178432
rs2278831	SIGLEC5	19	56822931	1	1.899347
rs2167418	LOC729781	19	56896754	5	0.693717
rs2167418	LOC729781	19	56896754	3	0.540794
rs2167418	LOC729781	19	56896754	8	3.520042
rs2167418	LOC729781	19	56896754	6	1.32168
rs3829649	HAS1	19	56908276	4	0.50162
rs3829649	HAS1	19	56908276	8	1.857311
rs3829647	HAS1	19	56908375	2	2.092177
rs3829647	HAS1	19	56908375	7	0.487931
rs3829647	HAS1	19	56908375	3	0.533241
rs3829647	HAS1	19	56908375	6	0.120786
rs867341	FPR1	19	56940932	3	0.234155
rs2070745	FPR1	19	56941759	4	3.201618
rs17695052	FPRL1	19	56965038	1	0.861448
rs3752125	FPRL2	19	57019596	3	0.256407
rs7253924	FPRL2	19	57020025	3	0.879665
rs9807901	ZNF577	19	57066514	7	0.190383
rs8113388	ZNF577	19	57067051	8	0.713471
rs8113388	ZNF577	19	57067051	2	4.263715
rs8113388	ZNF577	19	57067051	7	0.9093
rs8113388	ZNF577	19	57067051	4	4.03918
rs8113388	ZNF577	19	57067051	3	0.904956
rs1055408	ZNF577	19	57067236	3	0.564404
rs1055408	ZNF577	19	57067236	7	0.44416
rs1055408	ZNF577	19	57067236	4	0.669429
rs1055408	ZNF577	19	57067236	8	0.014447
rs1055408	ZNF577	19	57067236	2	5.881413
rs6509586	ZNF577	19	57067326	7	1.048744
rs10407911	ZNF577	19	57067931	7	0.609621
rs10407547	ZNF577	19	57067938	7	0.664509
rs9807842	ZNF577	19	57068019	7	0.326686

rs6509592	ZNF649	19	57084962	8	6.554547
rs6509592	ZNF649	19	57084962	3	1.23957
rs6509592	ZNF649	19	57084962	2	4.180615
rs6509592	ZNF649	19	57084962	7	5.448419
rs12461478	ZNF613	19	57122551	7	2.049619
rs12461478	ZNF613	19	57122551	1	1.795547
rs4986770	ZNF350	19	57159846	2	4.037765
rs12462876	ZNF614	19	57209050	3	0.034556
rs11670943	ZNF432	19	57243771	2	1.528055
rs3752120	ZNF432	19	57243833	3	0.187959
rs10413435	PPP2R1A	19	57420842	5	0.044533
rs10413435	PPP2R1A	19	57420842	7	4.580639
SNP19-57516844	ZNF480	19	57516844	1	4.878821
SNP19-57516844	ZNF480	19	57516844	6	0.523952
rs1961205	ZNF610	19	57548767	5	1.508133
SNP19-57749291	ZNF808	19	57749291	6	0.029667
SNP19-57749291	ZNF808	19	57749291	1	4.998158
SNP19-57749291	ZNF808	19	57749291	4	3.853579
rs12986137	ZNF808	19	57751684	4	0.654406
rs2279152	ZNF137	19	57791787	6	1.29126
rs2279152	ZNF137	19	57791787	7	0.07988
rs7250630	ZNF137	19	57792135	6	0.710011
rs7250630	ZNF137	19	57792135	1	6.518385
rs7250630	ZNF137	19	57792135	3	0.169857
rs1802617	ZNF137	19	57792397	1	4.285436
rs1802617	ZNF137	19	57792397	7	2.331392
rs1802617	ZNF137	19	57792397	6	0.80487
rs11668347	ZNF137	19	57794680	8	5.115785
rs4664	ZNF83	19	57807597	1	4.261233
rs10775556	ZNF83	19	57830234	7	0.207704
rs10775556	ZNF83	19	57830234	1	1.074611
rs8104631	ZNF600	19	57959291	5	0.315328
rs11667063	ZNF600	19	57959318	6	0.062441
rs11667063	ZNF600	19	57959318	5	1.322057
rs11667063	ZNF600	19	57959318	7	0.523587
rs11667063	ZNF600	19	57959318	8	0.149125
rs3194868	ZNF28	19	57992529	1	0.21395
rs3194868	ZNF28	19	57992529	2	2.820515
rs3194868	ZNF28	19	57992529	4	0.400768
rs10407516	ZNF28	19	57994265	2	0.88416
rs10407516	ZNF28	19	57994265	4	0.556835
rs10407516	ZNF28	19	57994265	5	0.015525
rs10407516	ZNF28	19	57994265	1	0.447346
rs10410012	ZNF28	19	57994549	5	0.047058
rs10410012	ZNF28	19	57994549	1	1.279754
rs10410012	ZNF28	19	57994549	4	2.225582
rs10417163	ZNF28	19	57995517	4	0.290437

rs10417163	ZNF28	19	57995517	7	0.029122
rs10417163	ZNF28	19	57995517	1	0.171771
rs10417163	ZNF28	19	57995517	6	0.77301
rs10417163	ZNF28	19	57995517	2	1.26237
rs10417163	ZNF28	19	57995517	3	0.445935
rs1966574	LOC729851	19	58059204	3	1.212264
rs8109900	ZNF321	19	58123806	3	0.285643
rs8109900	ZNF321	19	58123806	5	0.958588
rs8109900	ZNF321	19	58123806	1	3.374983
rs12459008	ZNF816A	19	58146601	5	2.200852
rs3814	ZNF415	19	58302999	4	0.447789
rs3814	ZNF415	19	58302999	6	10.81769
rs3814	ZNF415	19	58302999	1	0.01182
rs1650972	ZNF347	19	58335309	1	3.458845
rs1133146	ZNF665	19	58358472	4	0.976129
rs1133146	ZNF665	19	58358472	1	3.718549
rs1133146	ZNF665	19	58358472	6	0.964201
rs1133146	ZNF665	19	58358472	7	0.460039
rs1133146	ZNF665	19	58358472	5	1.122095
rs1133145	ZNF665	19	58358557	4	0.368939
rs1133145	ZNF665	19	58358557	7	0.280622
rs1133145	ZNF665	19	58358557	5	0.281638
rs1133145	ZNF665	19	58358557	1	3.428602
rs1133145	ZNF665	19	58358557	6	2.785313
rs12971799	ZNF665	19	58358938	5	1.278131
rs12971799	ZNF665	19	58358938	7	0.321733
rs12971799	ZNF665	19	58358938	1	3.279639
rs12971799	ZNF665	19	58358938	4	3.205613
rs6509729	ZNF665	19	58358996	2	2.948955
rs12972121	ZNF665	19	58359107	5	1.511019
rs12985467	ZNF665	19	58359169	1	0.752447
rs12985467	ZNF665	19	58359169	5	1.218304
rs12985467	ZNF665	19	58359169	4	0.444038
rs12985467	ZNF665	19	58359169	6	0.88722
rs12459469	ZNF665	19	58359725	7	0.109842
rs12459469	ZNF665	19	58359725	5	0.548402
rs12459469	ZNF665	19	58359725	4	3.029794
rs12459469	ZNF665	19	58359725	1	3.73002
rs10409924	ZNF665	19	58360361	6	0.163352
rs10409924	ZNF665	19	58360361	4	0.289201
rs10409924	ZNF665	19	58360361	5	4.06172
rs10409924	ZNF665	19	58360361	7	0.406743
rs4801959	ZNF665	19	58361085	2	1.728333
rs10853858	ZNF818	19	58408090	7	0.001903
rs10853858	ZNF818	19	58408090	3	7.608842
rs6509740	ZNF677	19	58430797	5	0.007819
rs6509740	ZNF677	19	58430797	3	1.338334

rs6509740	ZNF677	19	58430797	4	0.239213
rs17272966	ZNF677	19	58431770	4	0.486985
rs17272966	ZNF677	19	58431770	5	0.403887
rs8113436	ZNF677	19	58432634	5	0.414099
rs8113436	ZNF677	19	58432634	4	1.369771
rs12984188	ZNF677	19	58432880	5	0.451462
rs12984188	ZNF677	19	58432880	6	0.243854
rs12984473	ZNF677	19	58433000	5	0.843481
rs12984473	ZNF677	19	58433000	4	0.53326
rs12609049	VN1R2	19	58454349	5	1.077226
rs8109165	BIRC8	19	58484854	3	2.799807
rs8109165	BIRC8	19	58484854	5	3.254512
rs6509753	ZNF765	19	58605367	7	1.196333
SNP19-58649938	ZNF761	19	58649938	1	0.751618
SNP19-58649938	ZNF761	19	58649938	7	0.188965
SNP19-58649938	ZNF761	19	58649938	2	2.458566
SNP19-58685873	ZNF813	19	58685873	3	0.911071
SNP19-58685873	ZNF813	19	58685873	5	5.461034
SNP19-58685873	ZNF813	19	58685873	4	1.061138
rs2708844	ZNF813	19	58687503	2	1.758519
rs2708844	ZNF813	19	58687503	1	0.14091
rs2708844	ZNF813	19	58687503	6	1.205738
rs2708840	ZNF813	19	58688926	8	0.852081
rs2708781	NULL	19	58716026	5	1.628984
rs8100247	ZNF331	19	58717013	3	0.735317
rs16984961	NULL	19	58717090	3	0.530442
rs16985052	ZNF331	19	58772592	8	5.235749
rs16985052	ZNF331	19	58772592	2	3.066722
rs8110538	ZNF331	19	58773710	7	2.721639
rs8110538	ZNF331	19	58773710	5	3.008963
rs8110538	ZNF331	19	58773710	6	2.454326
rs8110350	ZNF331	19	58773727	5	2.531808
rs8110350	ZNF331	19	58773727	4	2.067732
rs8110350	ZNF331	19	58773727	8	3.439408
rs8110350	ZNF331	19	58773727	7	1.767841
rs8110350	ZNF331	19	58773727	6	1.75852
rs8103787	LOC646565	19	58853397	3	2.243139
rs4806773	NLRP12	19	59000366	6	1.405722
rs4806773	NLRP12	19	59000366	1	1.849509
rs1657535	OSCAR	19	59292044	1	3.242829
rs11556769	PRPF31	19	59319727	1	5.988585
rs43211	CNOT3	19	59344015	8	5.468177
rs43211	CNOT3	19	59344015	4	0.466018
rs36657	TMC4	19	59356021	6	0.219352
rs36657	TMC4	19	59356021	5	0.007368
rs36657	TMC4	19	59356021	1	5.747118
rs36657	TMC4	19	59356021	3	0.26625

rs36657	TMC4	19	59356021	8	1.170316
SNP19-59494505	LILRA3	19	59494505	5	0.853449
rs1035451	LENG8	19	59654279	4	0.511863
rs1035451	LENG8	19	59654279	5	0.763202
rs3813148	LENG8	19	59657672	4	0.221417
rs3813148	LENG8	19	59657672	6	3.630923
rs889147	LENG8	19	59658391	5	0.056028
rs889147	LENG8	19	59658391	6	0.248637
rs889147	LENG8	19	59658391	4	4.656394
rs10423424	LENG9	19	59665092	6	0.863976
rs10423424	LENG9	19	59665092	4	0.902138
rs10423424	LENG9	19	59665092	5	6.164101
rs10406453	LENG9	19	59666130	6	3.028924
rs10406453	LENG9	19	59666130	5	1.107366
rs10406453	LENG9	19	59666130	4	1.375532
rs1981829	LENG9	19	59666586	4	1.011582
rs1981829	LENG9	19	59666586	5	0.880366
rs1981829	LENG9	19	59666586	6	4.158435
SNP19-59938543	KIR3DL3	19	59938543	1	0.075041
rs269950	NLRP7	19	60133714	3	0.225133
rs2217659	NLRP2	19	60177711	3	1.007319
rs17699678	NLRP2	19	60185540	8	6.456903
rs11672113	NLRP2	19	60186693	1	0.329922
rs11672113	NLRP2	19	60186693	5	2.99412
rs11672113	NLRP2	19	60186693	3	0.727127
rs11672113	NLRP2	19	60186693	2	1.205557
rs12768	NLRP2	19	60203949	4	2.191528
rs12768	NLRP2	19	60203949	5	0.269802
rs12768	NLRP2	19	60203949	2	3.410015
rs12768	NLRP2	19	60203949	3	1.528151
rs12768	NLRP2	19	60203949	6	0.514919
rs1043673	NLRP2	19	60204044	3	0.037221
rs1043673	NLRP2	19	60204044	1	1.011604
rs1043680	NLRP2	19	60204159	2	0.780941
rs1043680	NLRP2	19	60204159	1	0.614606
rs1043680	NLRP2	19	60204159	3	0.170175
rs10417981	GP6	19	60217200	6	0.302599
rs1671150	GP6	19	60217309	6	0.461477
rs1671152	GP6	19	60218157	6	1.624999
rs1613662	GP6	19	60228407	4	0.229304
rs1613662	GP6	19	60228407	7	1.053451
rs1613662	GP6	19	60228407	6	0.611944
rs892090	GP6	19	60230884	4	3.700679
rs892090	GP6	19	60230884	7	0.789444
rs892090	GP6	19	60230884	6	0.187915
rs1189	RDH13	19	60247966	3	0.020189
rs1189	RDH13	19	60247966	4	9.667239

SNP19-60259896	RDH13	19	60259896	5	0.285146
rs2532060	PPP1R12C	19	60306735	7	0.208034
rs2532060	PPP1R12C	19	60306735	3	0.438658
rs2532060	PPP1R12C	19	60306735	1	1.918342
rs34521018	PPP1R12C	19	60315925	5	0.392693
rs34521018	PPP1R12C	19	60315925	3	0.039568
rs2365725	C19orf51	19	60364282	8	1.775288
rs17458	PTPRH	19	60384827	6	3.481179
rs2288420	PTPRH	19	60384935	6	2.565983
rs2288419	PTPRH	19	60385056	6	1.295836
rs2288419	PTPRH	19	60385056	8	0.144276
SNP19-60389091	PTPRH	19	60389091	1	2.972436
rs36092369	PTPRH	19	60390767	6	2.031744
rs2288523	PTPRH	19	60394722	2	3.90228
rs2288523	PTPRH	19	60394722	6	0.966619
rs2288521	PTPRH	19	60400369	3	1.283417
rs2288521	PTPRH	19	60400369	4	2.475676
rs2288521	PTPRH	19	60400369	1	1.328473
rs2288521	PTPRH	19	60400369	2	2.424643
rs2288521	PTPRH	19	60400369	8	4.505081
rs2288520	PTPRH	19	60400385	1	5.596685
SNP19-60407154	PTPRH	19	60407154	6	1.968819
rs3826884	TMEM86B	19	60429993	1	5.647676
rs4252574	FAM71E2	19	60565454	3	1.254236
rs4252574	FAM71E2	19	60565454	5	0.664298
rs1126760	IL11	19	60567886	5	0.326652
rs1126760	IL11	19	60567886	3	1.202824
rs2298885	IL11	19	60568052	1	0.093135
rs2298885	IL11	19	60568052	7	0.669746
rs2298885	IL11	19	60568052	2	1.796706
rs1042506	IL11	19	60568923	5	0.549878
rs1042506	IL11	19	60568923	3	0.474832
rs17700376	RPL28	19	60589139	3	1.948237
rs17700376	RPL28	19	60589139	7	6.200469
rs17700376	RPL28	19	60589139	1	1.980397
rs12981911	RPL28	19	60592681	1	0.268865
rs12981911	RPL28	19	60592681	8	0.987571
rs12981911	RPL28	19	60592681	2	3.644321
rs7255648	RPL28	19	60593994	2	5.961499
rs4075908	LOC284296	19	60633639	3	0.350756
rs4075908	LOC284296	19	60633639	1	0.007838
rs2303089	ISOC2	19	60656532	2	3.623787
SNP19-60690074	NAT14	19	60690074	3	0.353313
SNP19-60690074	NAT14	19	60690074	6	0.944842
rs7478	NAT14	19	60690323	3	0.364049
rs7478	NAT14	19	60690323	2	4.727479
rs7478	NAT14	19	60690323	5	0.181423

rs310475	ZNF580	19	60845680	4	1.13527
rs310475	ZNF580	19	60845680	8	0.960595
rs310475	ZNF580	19	60845680	3	1.013356
rs310475	ZNF580	19	60845680	7	0.502054
rs504850	U2AF2	19	60857287	6	0.190782
rs504850	U2AF2	19	60857287	4	0.228736
rs504850	U2AF2	19	60857287	8	0.310764
rs2616940	NLRP11	19	60999395	4	1.15676
rs2616940	NLRP11	19	60999395	6	2.005423
rs302456	NLRP4	19	61084687	5	2.336237
rs302456	NLRP4	19	61084687	1	1.236257
rs302456	NLRP4	19	61084687	2	3.247917
rs12975929	NLRP4	19	61084732	6	2.726141
rs12975929	NLRP4	19	61084732	3	3.241313
rs12975929	NLRP4	19	61084732	5	2.47623
rs306475	NLRP8	19	61182590	7	0.320764
rs306475	NLRP8	19	61182590	5	1.651043
rs306475	NLRP8	19	61182590	1	1.202935
rs397977	NLRP5	19	61231052	4	0.427014
rs397977	NLRP5	19	61231052	5	0.908344
rs397977	NLRP5	19	61231052	3	0.624307
rs16986899	NLRP5	19	61241322	1	0.021871
SNP19-6124426	NLRP5	19	61244268	5	1.376915
rs10409555	NLRP5	19	61264644	1	1.389646
rs575756	GALP	19	61386317	6	2.021461
SNP19-6139506	LOC342933	19	61395060	1	4.648973
SNP19-6139510	LOC342933	19	61395101	7	4.937546
SNP19-6139510	LOC342933	19	61395101	6	4.171706
SNP19-6139510	LOC342933	19	61395101	4	4.646721
SNP19-6139510	LOC342933	19	61395101	1	5.413689
SNP19-6139510	LOC342933	19	61395101	3	4.863115
SNP19-6139510	LOC342933	19	61395101	8	4.772706
SNP19-6139510	LOC342933	19	61395101	5	4.054827
rs12979551	LOC649137	19	61411217	7	1.220872
rs12979551	LOC649137	19	61411217	1	0.467142
rs12979551	LOC649137	19	61411217	6	1.567186
rs12979551	LOC649137	19	61411217	2	2.640612
rs12979551	LOC649137	19	61411217	8	1.237833
rs7245993	LOC729999	19	61523320	6	1.191879
rs7245993	LOC729999	19	61523320	1	1.177936
rs7245993	LOC729999	19	61523320	3	1.277471
rs11667052	ZNF471	19	61727824	1	5.243985
rs11667052	ZNF471	19	61727824	4	0.945839
rs3752176	ZNF471	19	61728465	4	0.388928
rs960997	ZNF471	19	61731283	4	9.253086
rs960997	ZNF471	19	61731283	1	3.996247
rs958338	ZNF471	19	61731511	4	4.363411

rs7258886	ZFP28	19	61759851	4	0.772107
rs7258886	ZFP28	19	61759851	6	1.239246
rs7258886	ZFP28	19	61759851	1	4.868027
rs10421285	ZNF470	19	61777581	6	2.392237
rs10421285	ZNF470	19	61777581	1	5.785161
rs10421285	ZNF470	19	61777581	4	1.103138
rs1047063	ZNF71	19	61826386	1	4.002004
rs1047063	ZNF71	19	61826386	4	3.212105
rs2286751	ZIM2	19	61985206	5	0.995007
rs2286751	ZIM2	19	61985206	1	1.259681
rs2286751	ZIM2	19	61985206	3	2.132367
rs1055359	PEG3	19	62016152	1	4.131757
rs1055359	PEG3	19	62016152	5	5.087465
rs1055359	PEG3	19	62016152	3	5.083118
rs1860565	PEG3	19	62026834	4	1.666382
rs1860565	PEG3	19	62026834	3	3.887909
rs1860565	PEG3	19	62026834	1	1.257605
rs1027392	USP29	19	62332958	5	1.146664
rs3795003	USP29	19	62333611	7	1.440893
rs8103779	USP29	19	62334863	5	4.571258
rs8103793	USP29	19	62334911	4	1.764347
SNP19-62340089	ZIM3	19	62340089	5	1.28642
SNP19-62340089	ZIM3	19	62340089	4	0.889033
rs17305556	ZIM3	19	62341713	7	1.518516
rs11882189	DUXA	19	62357085	1	1.992373
rs11882189	DUXA	19	62357085	4	0.603884
rs11882189	DUXA	19	62357085	5	0.837127
rs8108591	DUXA	19	62357562	5	7.230625
SNP19-62414819	ZNF264	19	62414819	7	0.683638
SNP19-62414819	ZNF264	19	62414819	1	6.788472
SNP19-62414819	ZNF264	19	62414819	4	0.475584
SNP19-62414819	ZNF264	19	62414819	6	0.057754
rs10414299	ZNF264	19	62416927	8	0.738257
rs10414299	ZNF264	19	62416927	1	5.023615
rs2361129	ZNF264	19	62421509	5	0.933762
rs3746228	ZNF460	19	62496174	4	5.272229
rs6510057	ZNF543	19	62529830	4	0.449771
rs6510057	ZNF543	19	62529830	7	0.529986
rs6510057	ZNF543	19	62529830	1	0.278342
rs6510057	ZNF543	19	62529830	5	0.088614
rs6510057	ZNF543	19	62529830	2	7.63814
rs6510057	ZNF543	19	62529830	8	7.9142
rs6510057	ZNF543	19	62529830	6	5.688195
rs12460323	ZNF548	19	62593073	8	0.529362
rs12460323	ZNF548	19	62593073	5	0.690408
rs12460323	ZNF548	19	62593073	3	0.939657
rs12460323	ZNF548	19	62593073	6	2.496862

SNP19-62601639	ZNF548	19	62601639	6	11.59512
SNP19-62601639	ZNF548	19	62601639	5	4.143778
rs12974211	ZNF17	19	62614525	5	3.266108
rs12974211	ZNF17	19	62614525	6	4.629242
rs2240037	ZNF749	19	62647531	8	5.412473
rs4801488	ZNF772	19	62672813	1	0.612434
rs4801488	ZNF772	19	62672813	6	0.072994
rs2360765	ZNF772	19	62674663	1	0.167877
rs2360765	ZNF772	19	62674663	6	0.088831
rs2360764	ZNF772	19	62674890	2	3.396312
rs2360764	ZNF772	19	62674890	1	0.888846
rs2360764	ZNF772	19	62674890	6	0.572301
rs10426249	ZNF772	19	62675606	6	3.656686
rs10426249	ZNF772	19	62675606	2	2.686067
rs10426249	ZNF772	19	62675606	1	0.185967
rs10426255	ZNF772	19	62675617	1	0.393617
rs10426255	ZNF772	19	62675617	2	0.197626
rs10407734	ZNF772	19	62675857	2	2.013382
rs10407734	ZNF772	19	62675857	1	0.076978
rs2074059	ZNF772	19	62677272	2	1.394778
rs2074059	ZNF772	19	62677272	1	0.407312
rs2074059	ZNF772	19	62677272	6	6.459862
rs2074060	ZNF772	19	62677378	2	4.076304
rs2074060	ZNF772	19	62677378	6	2.087268
rs2074060	ZNF772	19	62677378	1	0.239428
rs12463137	ZNF773	19	62703233	6	0.444449
rs12463137	ZNF773	19	62703233	4	0.816591
rs2885007	ZNF134	19	62826084	1	4.254962
rs11879465	ZNF211	19	62844607	1	1.868337
rs3746215	ZNF211	19	62845408	1	5.400128
rs12618	ZNF211	19	62845568	1	5.339385
rs9676604	ZSCAN4	19	62881099	1	1.722192
rs2188736	ZNF154	19	62905585	6	1.67155
rs2188736	ZNF154	19	62905585	1	4.471473
rs13037	ZNF776	19	62960985	4	0.227183
rs13037	ZNF776	19	62960985	5	0.044854
rs13037	ZNF776	19	62960985	2	4.221986
rs13037	ZNF776	19	62960985	7	1.597051
rs13037	ZNF776	19	62960985	1	0.119894
rs12609890	ZNF587	19	63064422	8	0.924902
rs12609890	ZNF587	19	63064422	3	0.578674
rs7253514	ZNF418	19	63131118	3	1.127438
rs7253514	ZNF418	19	63131118	8	0.459146
rs7247254	LOC646820	19	63156396	3	1.411578
rs7247254	LOC646820	19	63156396	5	2.208987
rs7247254	LOC646820	19	63156396	4	2.805755
rs7247254	LOC646820	19	63156396	8	0.110595

rs257662	LOC646820	19	63156441	8	2.63343
rs257662	LOC646820	19	63156441	3	0.281865
rs257662	LOC646820	19	63156441	7	0.164679
rs257663	LOC646820	19	63156742	5	0.08703
rs257663	LOC646820	19	63156742	7	0.026681
rs7249794	C19orf18	19	63177640	3	1.296321
rs9304809	ZNF606	19	63206287	3	0.449654
rs9304809	ZNF606	19	63206287	8	0.989849
rs8104122	ZSCAN1	19	63257243	1	1.206207
rs2288274	ZNF135	19	63264771	7	2.528771
rs10421350	ZSCAN18	19	63301255	5	1.143128
rs10420801	ZSCAN18	19	63301269	5	0.217583
rs2547360	ZNF329	19	63330211	3	0.571448
rs11666156	ZNF8	19	63482171	3	0.597677
rs11666156	ZNF8	19	63482171	7	0.80762
rs11666156	ZNF8	19	63482171	5	0.097572
rs1265956	A1BG	19	63548943	3	1.12326
rs893184	A1BG	19	63556291	3	1.580968
rs2241787	RPS5	19	63597975	3	0.568294
rs2241787	RPS5	19	63597975	4	0.028512
rs2241787	RPS5	19	63597975	6	3.504141
rs2241787	RPS5	19	63597975	7	0.496968
SNP19-63620121	ZNF584	19	63620121	4	0.061003
SNP19-63620121	ZNF584	19	63620121	7	4.867968
rs3764532	ZNF584	19	63621034	4	0.256331
rs3764531	ZNF584	19	63621117	1	5.708968
rs3764531	ZNF584	19	63621117	3	0.144911
SNP19-63660991	ZNF324B	19	63660991	3	1.422211
SNP19-63660991	ZNF324B	19	63660991	5	2.438839
SNP19-63660991	ZNF324B	19	63660991	6	0.578836
SNP19-63660991	ZNF324B	19	63660991	8	1.335531
SNP19-63660991	ZNF324B	19	63660991	4	0.217747
SNP19-63660991	ZNF324B	19	63660991	1	5.817794
rs4801595	ZNF324	19	63670329	5	0.750928
rs4801595	ZNF324	19	63670329	6	0.378754
rs4801595	ZNF324	19	63670329	1	2.84279
rs4801595	ZNF324	19	63670329	3	0.767902
rs4801595	ZNF324	19	63670329	8	0.309147
rs35350976	SLC27A5	19	63714986	1	0.468775
rs35350976	SLC27A5	19	63714986	4	0.666532
rs4756	MZF1	19	63766465	4	0.426275
rs4756	MZF1	19	63766465	1	4.078117
rs3752109	MZF1	19	63774417	1	1.905951
rs3752109	MZF1	19	63774417	8	0.817872
rs3752109	MZF1	19	63774417	5	0.073291
rs3752109	MZF1	19	63774417	3	0.622181
rs3827147	C20orf96	20	204727	5	0.124821

rs3827147	C20orf96	20	204727	3	0.187009
rs3827147	C20orf96	20	204727	2	4.96227
rs7271033	C20orf96	20	207969	3	0.212187
rs7271033	C20orf96	20	207969	4	5.592106
rs7271033	C20orf96	20	207969	5	6.869672
rs7271033	C20orf96	20	207969	2	0.094736
rs7271033	C20orf96	20	207969	1	0.276487
rs1057189	ZCCHC3	20	226806	3	0.899548
rs6050213	SOX12	20	256878	3	0.498338
rs6050344	SOX12	20	258493	3	0.504239
rs6050344	SOX12	20	258493	6	4.824365
rs1001549	NRSN2	20	283225	3	0.726776
rs1001549	NRSN2	20	283225	7	5.662362
rs6695	TBC1D20	20	364149	1	2.968059
rs6116997	SCRT2	20	591677	3	0.827528
SNP20-692382	C20orf54	20	692382	8	0.757633
SNP20-692382	C20orf54	20	692382	3	0.708861
rs2282050	FAM110A	20	774492	8	4.936883
rs2282050	FAM110A	20	774492	7	0.774983
rs2282050	FAM110A	20	774492	1	6.597691
rs2282050	FAM110A	20	774492	6	0.002967
rs11907018	ANGPT4	20	801502	1	0.469406
rs944110	ANGPT4	20	802940	5	1.424048
rs944110	ANGPT4	20	802940	3	0.884962
rs734940	RSPO4	20	887596	6	0.824542
rs734940	RSPO4	20	887596	4	0.258671
rs734940	RSPO4	20	887596	5	0.376119
rs1803415	PSMF1	20	1047523	2	4.554862
rs3795139	SNPH	20	1233933	3	1.140147
rs3795139	SNPH	20	1233933	4	0.464467
rs3795139	SNPH	20	1233933	7	1.185253
rs3795139	SNPH	20	1233933	1	4.332849
rs3764714	SNPH	20	1235105	3	0.800225
rs6134520	SNPH	20	1236223	5	4.729269
rs6134520	SNPH	20	1236223	4	1.727458
rs6134520	SNPH	20	1236223	8	3.595765
rs6134520	SNPH	20	1236223	6	0.407885
rs3210915	NSFL1C	20	1371533	7	6.128446
rs3210915	NSFL1C	20	1371533	3	0.035405
rs3210915	NSFL1C	20	1371533	5	0.130068
rs3210915	NSFL1C	20	1371533	6	0.709229
rs3210915	NSFL1C	20	1371533	4	0.208034
rs6135033	NSFL1C	20	1396366	7	0.681533
rs6135033	NSFL1C	20	1396366	2	4.076162
rs6135033	NSFL1C	20	1396366	5	0.712429
rs6042507	SIRPB2	20	1407060	5	1.277361
rs2253693	SIRPB1	20	1493468	2	3.909791

rs2253693	SIRPB1	20	1493468	4	0.82005
rs2253693	SIRPB1	20	1493468	5	1.340649
rs2253693	SIRPB1	20	1493468	7	5.90221
rs1048055	SIRPG	20	1558062	7	8.155765
rs1048055	SIRPG	20	1558062	4	2.336915
rs2281807	SIRPG	20	1558201	7	1.134813
rs2281807	SIRPG	20	1558201	1	6.031602
rs3746721	SIRPG	20	1558894	4	0.374063
rs3746721	SIRPG	20	1558894	5	0.747843
rs3746721	SIRPG	20	1558894	8	0.032669
rs3746722	SIRPG	20	1558939	7	2.514822
SNP20-1564137	SIRPG	20	1564137	7	0.265972
SNP20-1564137	SIRPG	20	1564137	1	2.787827
SNP20-1564137	SIRPG	20	1564137	4	1.215878
rs2273329	LOC441938	20	1626188	4	0.439182
rs2273329	LOC441938	20	1626188	8	0.469854
rs2273329	LOC441938	20	1626188	7	0.054642
rs2273329	LOC441938	20	1626188	5	1.235828
rs513201	LOC441938	20	1636844	3	1.028713
rs2185675	STK35	20	2074180	1	5.751109
rs2185675	STK35	20	2074180	8	0.058009
rs2185675	STK35	20	2074180	5	0.153912
rs214803	TGM3	20	2238333	4	3.683647
rs214803	TGM3	20	2238333	8	3.676283
rs214803	TGM3	20	2238333	7	2.648061
rs214831	TGM3	20	2269363	3	2.450643
rs214831	TGM3	20	2269363	5	2.585414
rs214832	TGM3	20	2269665	3	0.839747
rs214832	TGM3	20	2269665	5	0.028837
rs214832	TGM3	20	2269665	4	0.66268
rs1543385	ZNF343	20	2410905	6	7.701491
rs1543385	ZNF343	20	2410905	1	3.180659
rs6114607	ZNF343	20	2429311	1	4.583082
rs6138281	ZNF343	20	2437531	3	0.32052
rs6138281	ZNF343	20	2437531	1	4.958459
rs2422812	TMC2	20	2570390	4	0.050536
rs2273137	NOL5A	20	2583212	4	0.229872
rs6051545	GNRH2	20	2973107	3	0.78573
rs1078152	LOC642991	20	3022040	1	0.442505
rs913555	UBOX5	20	3038124	3	0.526845
rs913555	UBOX5	20	3038124	7	0.026573
rs913555	UBOX5	20	3038124	5	0.396477
rs913555	UBOX5	20	3038124	8	0.794405
rs34205880	UBOX5	20	3038942	7	2.283288
rs34205880	UBOX5	20	3038942	6	0.57674
rs34205880	UBOX5	20	3038942	4	0.886542
rs8362	ITPA	20	3141978	5	6.413624

rs8362	ITPA	20	3141978	6	6.982891
rs8362	ITPA	20	3141978	3	0.869701
rs8362	ITPA	20	3141978	1	5.302103
rs17784288	ATRN	20	3577843	2	4.589432
rs641362	ATRN	20	3578563	3	0.158993
rs641362	ATRN	20	3578563	8	2.531967
SNP20-3598234	ADAM33	20	3598234	7	1.20122
SNP20-3598234	ADAM33	20	3598234	1	1.58531
SNP20-3598234	ADAM33	20	3598234	4	1.775227
rs673114	SIGLEC1	20	3625736	3	1.190097
rs611847	SIGLEC1	20	3632022	1	0.940451
rs625372	SIGLEC1	20	3632729	3	0.917519
SNP20-3634436	SIGLEC1	20	3634436	3	1.583576
SNP20-3634436	SIGLEC1	20	3634436	1	1.623031
rs3088007	HSPA12B	20	3681487	3	0.44106
rs3088007	HSPA12B	20	3681487	4	0.464445
rs2281479	SPEF1	20	3710095	7	0.073176
rs2281479	SPEF1	20	3710095	5	0.124663
rs8156	CDC25B	20	3734637	1	3.433869
rs8156	CDC25B	20	3734637	8	1.461338
rs8156	CDC25B	20	3734637	5	0.015956
rs17857295	VISA	20	3786441	3	0.080246
SNP20-3792929	VISA	20	3792929	5	1.036414
SNP20-3792929	VISA	20	3792929	1	5.068538
SNP20-3792929	VISA	20	3792929	4	6.392965
SNP20-3794397	VISA	20	3794397	8	7.576424
SNP20-3794397	VISA	20	3794397	7	0.232069
rs1741327	SMOX	20	4112283	1	0.752183
rs1741327	SMOX	20	4112283	6	0.491632
rs1741327	SMOX	20	4112283	2	0.803904
rs1741327	SMOX	20	4112283	4	0.825591
rs1741327	SMOX	20	4112283	7	0.111126
rs709024	ADRA1D	20	4149984	7	0.821113
rs709024	ADRA1D	20	4149984	1	0.455985
rs709024	ADRA1D	20	4149984	8	0.593953
rs709024	ADRA1D	20	4149984	5	0.275827
rs2236554	ADRA1D	20	4150041	7	0.302625
SNP20-4629310	PRNP	20	4629310	5	0.76042
rs2245220	PRND	20	4653718	1	0.451108
rs2245220	PRND	20	4653718	3	1.181163
rs2735675	PRND	20	4655388	5	0.634121
rs2735675	PRND	20	4655388	1	0.02357
rs3746673	RASSF2	20	4710577	5	1.361302
rs2422973	RASSF2	20	4710701	8	0.728167
rs2422974	RASSF2	20	4710736	7	0.057754
rs2422974	RASSF2	20	4710736	8	0.69112
rs2422974	RASSF2	20	4710736	4	0.925658

rs2422977	RASSF2	20	4711093	1	1.735282
rs6116508	RASSF2	20	4711299	1	0.261554
rs6139551	RASSF2	20	4711438	1	1.951492
rs3803969	RASSF2	20	4711904	1	2.251595
rs3746674	RASSF2	20	4712629	5	0.440091
rs3746674	RASSF2	20	4712629	2	1.603897
rs2422979	RASSF2	20	4712682	1	0.03088
rs2422979	RASSF2	20	4712682	5	1.043183
rs3746684	PROKR2	20	5231376	8	2.234967
rs4544519	FLJ33544	20	5245172	7	0.114251
rs4315595	FLJ33544	20	5245193	7	0.391832
rs4316997	FLJ33544	20	5245859	5	0.087954
rs4316997	FLJ33544	20	5245859	7	0.886125
rs4281978	FLJ33544	20	5245984	7	1.074434
rs6085189	LOC728329	20	5436644	3	0.82206
SNP20-5851067	CHGB	20	5851067	2	3.872717
SNP20-5851067	CHGB	20	5851067	1	3.930453
SNP20-5851067	CHGB	20	5851067	8	4.025432
rs236150	CHGB	20	5851141	8	1.555103
rs910122	CHGB	20	5851323	7	0.386783
rs910122	CHGB	20	5851323	1	1.225202
rs910122	CHGB	20	5851323	8	0.902648
SNP20-5851388	CHGB	20	5851388	4	0.479586
SNP20-5851388	CHGB	20	5851388	7	2.23485
rs236151	CHGB	20	5851517	4	0.434117
rs236151	CHGB	20	5851517	5	0.377972
rs236151	CHGB	20	5851517	1	4.147918
rs236152	CHGB	20	5851848	1	0.66501
rs236152	CHGB	20	5851848	4	1.178965
rs236152	CHGB	20	5851848	7	0.596095
rs236153	CHGB	20	5851894	1	0.244379
rs236153	CHGB	20	5851894	2	0.621314
rs236153	CHGB	20	5851894	7	0.954723
rs236153	CHGB	20	5851894	4	0.598799
rs742710	CHGB	20	5852028	4	0.778389
rs742710	CHGB	20	5852028	7	0.127532
rs742711	CHGB	20	5852040	1	1.863045
rs742711	CHGB	20	5852040	2	0.990135
rs742711	CHGB	20	5852040	8	0.096184
rs2821	CHGB	20	5853779	7	1.421103
rs2821	CHGB	20	5853779	5	1.121217
rs236156	CHGB	20	5853972	8	0.815765
rs1059391	FERMT1	20	6004356	2	5.736988
rs1059391	FERMT1	20	6004356	6	0.939674
rs753927	FERMT1	20	6012710	3	1.170185
rs35413391	FERMT1	20	6017723	3	1.45677
rs35413391	FERMT1	20	6017723	5	0.545355

rs35413391	FERMT1	20	6017723	6	1.406669
SNP20-6036265	FERMT1	20	6036265	5	0.002427
rs1056141	FERMT1	20	6044632	5	0.453122
rs2021349	LOC643503	20	6129948	5	0.426376
rs2076016	TXNDC13	20	7910707	7	4.673167
rs1012891	TXNDC13	20	7928390	6	4.730473
rs1012891	TXNDC13	20	7928390	4	0.305258
rs1012891	TXNDC13	20	7928390	1	2.715898
rs2205783	TXNDC13	20	7948386	1	0.107862
rs2205783	TXNDC13	20	7948386	5	1.001352
rs2205783	TXNDC13	20	7948386	6	3.259126
rs2205783	TXNDC13	20	7948386	7	1.570344
rs2205783	TXNDC13	20	7948386	4	0.258706
rs6077510	PLCB4	20	9236522	3	1.261262
rs2232257	C20orf103	20	9443297	5	0.759697
rs12151948	C20orf103	20	9443379	3	0.409268
rs10485732	PAK7	20	9467529	1	1.939452
rs2297345	PAK7	20	9491622	2	3.245039
rs2297345	PAK7	20	9491622	8	5.389122
rs6133723	PAK7	20	9494554	8	0.807316
rs2423432	PAK7	20	9640033	7	1.071302
rs652633	ANKRD5	20	9978188	3	0.175122
SNP20-9978452	ANKRD5	20	9978452	3	0.012244
rs6087119	ANKRD5	20	9984202	1	3.954748
rs3746544	SNAP25	20	10235084	6	0.601445
rs3746544	SNAP25	20	10235084	1	0.620158
rs3746544	SNAP25	20	10235084	4	0.23778
rs1545	MKKS	20	10334013	6	1.787671
rs1547	MKKS	20	10334059	6	3.159005
rs3178014	JAG1	20	10581237	6	0.377001
rs3178014	JAG1	20	10581237	7	1.298832
rs3178014	JAG1	20	10581237	4	0.962737
rs3178014	JAG1	20	10581237	2	2.752281
rs3178014	JAG1	20	10581237	8	4.504547
rs6134404	BTBD3	20	11854553	1	4.949273
SNP20-13199388	C20orf82	20	13199388	2	3.807628
rs3180370	ESF1	20	13695441	3	0.28364
rs6042360	ESF1	20	13713507	7	3.380258
rs199306	MACROD2	20	15125520	1	0.480572
rs199307	MACROD2	20	15125580	1	1.485706
rs199308	MACROD2	20	15125625	1	0.246508
rs932501	LOC613266	20	15822325	3	0.481722
SNP20-15915390	MACROD2	20	15915390	8	5.330279
SNP20-15915390	MACROD2	20	15915390	3	0.442629
SNP20-15915390	MACROD2	20	15915390	1	5.896808
rs6135656	MACROD2	20	15979257	3	0.853008
rs1225891	MACROD2	20	15981185	3	0.903764

rs1225891	MACROD2	20	15981185	1	0.095844
rs1225891	MACROD2	20	15981185	2	5.793706
rs4814510	C20orf23	20	16502067	3	1.817581
rs6136118	BFSP1	20	17423217	7	0.434862
rs6136118	BFSP1	20	17423217	3	0.37334
rs2273448	SNX5	20	17880210	6	0.72558
rs2273448	SNX5	20	17880210	3	0.364376
rs2273448	SNX5	20	17880210	8	0.487394
rs2273448	SNX5	20	17880210	4	0.788855
rs910902	SNX5	20	17897452	8	0.253322
rs910902	SNX5	20	17897452	3	0.320826
rs910902	SNX5	20	17897452	4	0.206951
rs6111803	OVOL2	20	17970362	8	5.081814
rs34326039	CSRP2BP	20	18110481	6	0.156293
SNP20-18243730	ZNF133	20	18243730	5	0.29169
rs2228274	ZNF133	20	18243861	4	0.130009
rs1050475	ZNF133	20	18244473	4	1.332852
rs6075337	C20orf12	20	18341312	6	4.227264
rs6075337	C20orf12	20	18341312	7	0.167971
rs6075337	C20orf12	20	18341312	1	5.544208
rs6075337	C20orf12	20	18341312	5	0.374824
rs6035037	C20orf12	20	18343988	3	0.288349
rs6035037	C20orf12	20	18343988	5	0.020382
rs6035042	C20orf12	20	18362356	5	0.593972
rs6035042	C20orf12	20	18362356	6	5.541393
rs6035042	C20orf12	20	18362356	7	3.984443
rs4813333	SEC23B	20	18436399	5	5.320318
rs1053839	C20orf79	20	18742754	4	3.665793
rs1569767	SLC24A3	20	19209623	7	0.048919
rs1569767	SLC24A3	20	19209623	5	0.098879
rs17293432	SLC24A3	20	19513671	2	4.524777
rs2277866	SLC24A3	20	19649943	8	3.11116
rs6046271	SLC24A3	20	19650171	4	1.120736
rs6046271	SLC24A3	20	19650171	1	4.876435
rs6046271	SLC24A3	20	19650171	3	1.029881
rs16981035	SLC24A3	20	19650513	1	4.894589
rs16981035	SLC24A3	20	19650513	3	1.230409
rs16981035	SLC24A3	20	19650513	4	6.670208
rs3748484	SLC24A3	20	19650694	8	0.899046
rs2255255	CRNKL1	20	19980998	1	4.881626
rs2255255	CRNKL1	20	19980998	8	0.700404
rs2255255	CRNKL1	20	19980998	4	7.735797
rs6075614	C20orf26	20	20027360	3	1.108037
rs6081901	C20orf26	20	20092772	7	1.339424
rs6081901	C20orf26	20	20092772	4	1.979208
rs6075630	C20orf26	20	20098027	6	0.622305
rs6075630	C20orf26	20	20098027	3	0.524957

rs2424317	C20orf26	20	20205958	3	0.831071
rs4815025	C20orf19	20	21090523	6	2.642761
rs4815025	C20orf19	20	21090523	7	1.207515
rs4815025	C20orf19	20	21090523	1	0.579279
rs4815025	C20orf19	20	21090523	3	0.297068
rs2567609	SSTR4	20	22965017	1	0.952635
rs2567609	SSTR4	20	22965017	8	0.935267
rs2567609	SSTR4	20	22965017	2	0.44189
rs3176126	THBD	20	22974271	6	0.312437
rs3176126	THBD	20	22974271	4	2.508808
rs1962	THBD	20	22974496	5	0.27802
rs1042579	THBD	20	22976724	2	5.688032
rs1042579	THBD	20	22976724	8	1.048081
rs7988	GZF1	20	23300685	1	6.06859
rs7988	GZF1	20	23300685	4	10.2448
rs7988	GZF1	20	23300685	6	0.062633
rs6137913	NAPB	20	23305725	7	0.046518
rs17750862	NAPB	20	23325815	4	0.963402
rs17750862	NAPB	20	23325815	5	3.38849
SNP20-23372613	CSTL1	20	23372613	1	0.907104
SNP20-23566571	CST3	20	23566571	4	0.307168
SNP20-23566571	CST3	20	23566571	5	5.619359
SNP20-23566571	CST3	20	23566571	1	1.817989
rs1799841	CST5	20	23808178	7	1.50314
rs11087490	CST7	20	24877913	7	1.701385
rs1056033	CST7	20	24887590	3	0.65502
rs1056033	CST7	20	24887590	1	0.932744
rs11698945	C20orf3	20	24921247	5	0.323632
rs11698945	C20orf3	20	24921247	7	1.971624
rs6115001	ACSS1	20	24942275	5	0.936904
rs6115001	ACSS1	20	24942275	7	0.562502
rs6115003	ACSS1	20	24948734	7	1.277215
rs6115003	ACSS1	20	24948734	5	0.611301
rs6050259	ACSS1	20	24959423	7	0.054802
rs6050259	ACSS1	20	24959423	6	0.170339
rs6050259	ACSS1	20	24959423	8	3.85861
rs6050259	ACSS1	20	24959423	5	0.508997
rs2076559	ENTPD6	20	25135213	4	0.805473
rs2076559	ENTPD6	20	25135213	2	3.292065
rs6050446	ENTPD6	20	25143509	1	6.213062
rs2227891	PYGB	20	25210769	2	4.076531
rs2387887	GINS1	20	25375081	6	1.868299
rs437635	RP4-691N24.1	20	25404888	6	0.646357
rs437635	RP4-691N24.1	20	25404888	2	3.085547
rs11700338	NANP	20	25543868	2	2.054909
rs11700338	NANP	20	25543868	8	0.050903
rs45520534	DEFB123	20	29501699	4	0.479936

rs45520534	DEFB123	20	29501699	7	1.247456
rs4911536	DUSP15	20	29916443	7	0.140875
rs6061043	TTLL9	20	29990726	8	0.491698
rs714604	XKR7	20	30049423	4	1.768266
rs714604	XKR7	20	30049423	7	1.100835
rs714604	XKR7	20	30049423	5	2.973873
rs3813922	TSPYL3	20	30240997	3	0.672234
rs11907716	TSPYL3	20	30241461	5	0.718164
rs11907716	TSPYL3	20	30241461	4	0.026363
rs11907716	TSPYL3	20	30241461	7	3.026612
rs11907235	TSPYL3	20	30241521	5	0.384982
rs11907235	TSPYL3	20	30241521	7	0.402094
rs11907235	TSPYL3	20	30241521	4	0.698847
rs17268666	POFUT1	20	30282511	6	0.434476
rs13037236	KIF3B	20	30379122	3	0.270451
rs2070090	MAPRE1	20	30891296	4	6.619069
rs13037496	LOC388795	20	30930195	7	0.17295
rs13043425	LOC388795	20	30968850	7	0.065618
rs13043425	LOC388795	20	30968850	8	0.249021
rs13037781	LOC388795	20	30968953	1	0.060691
rs6058961	LOC388795	20	30981959	7	0.586021
rs6058961	LOC388795	20	30981959	1	0.516625
rs6058961	LOC388795	20	30981959	5	0.981945
rs13045180	LOC729476	20	30995205	5	0.462464
rs1133358	SPAG4L	20	31054347	4	4.697915
rs6057717	C20orf185	20	31120293	4	0.205177
rs2424943	C20orf186	20	31135280	1	1.118828
rs735625	RP11-49G10.8	20	31256665	1	4.092551
rs1999663	C20orf114	20	31361215	6	6.4323
rs291700	CDK5RAP1	20	31445510	1	0.645643
rs291700	CDK5RAP1	20	31445510	8	0.018926
rs291700	CDK5RAP1	20	31445510	7	0.455068
rs291700	CDK5RAP1	20	31445510	6	7.511622
rs3746459	C20orf134	20	31718894	5	0.205771
rs3746459	C20orf134	20	31718894	7	1.852243
rs959144	PXMP4	20	31758245	1	3.951852
rs959144	PXMP4	20	31758245	5	0.060633
rs2747566	ZNF341	20	31843292	7	0.12736
rs2747566	ZNF341	20	31843292	1	4.753394
rs6058017	ASIP	20	32320659	5	1.025075
rs3746455	ITCH	20	32420877	3	0.787417
rs3746455	ITCH	20	32420877	4	1.017396
rs3746455	ITCH	20	32420877	8	0.371196
rs3746455	ITCH	20	32420877	6	1.171736
rs3787220	NCOA6	20	32801412	1	0.185057
rs3787220	NCOA6	20	32801412	2	0.905959
SNP20-3280177	NCOA6	20	32801775	4	6.541963

rs6060137	MYH7B	20	33029141	1	2.223612
rs3746446	MYH7B	20	33038426	3	0.599322
rs2425009	MYH7B	20	33042560	1	4.289055
rs2425012	MYH7B	20	33045616	1	3.506211
rs2425012	MYH7B	20	33045616	3	0.099958
SNP20-33047950	MYH7B	20	33047950	8	0.738689
SNP20-33047950	MYH7B	20	33047950	1	1.067318
SNP20-33047950	MYH7B	20	33047950	3	0.776978
rs41310801	MYH7B	20	33047971	3	2.364649
rs3746435	MYH7B	20	33050859	8	0.983222
rs3746435	MYH7B	20	33050859	3	0.307772
SNP20-33052768	MYH7B	20	33052768	3	0.715834
rs4911167	MYH7B	20	33053757	1	4.924008
rs3746429	EDEM2	20	33167268	3	0.233052
rs3746429	EDEM2	20	33167268	8	6.318413
SNP20-33298124	MMP24	20	33298124	8	5.629441
rs2425042	LOC729580	20	33329309	1	4.360508
rs2425042	LOC729580	20	33329309	3	1.513076
rs2425049	FAM83C	20	33338134	3	1.667375
rs143384	GDF5	20	33489170	8	2.289353
rs143384	GDF5	20	33489170	1	3.379488
SNP20-33489397	GDF5	20	33489397	1	2.703438
SNP20-33489397	GDF5	20	33489397	8	2.188442
rs2277862	FER1L4	20	33616196	8	0.406614
rs1046383	EPB41L1	20	34284061	8	10.02914
rs1146250	C20orf117	20	34901127	5	0.539318
rs1146250	C20orf117	20	34901127	4	0.191426
rs1146250	C20orf117	20	34901127	3	0.458939
rs1146250	C20orf117	20	34901127	2	0.467363
rs1146250	C20orf117	20	34901127	8	7.931765
rs1146250	C20orf117	20	34901127	6	0.321387
rs1146250	C20orf117	20	34901127	1	0.807812
rs1744760	C20orf132	20	35174208	5	0.55192
rs1744760	C20orf132	20	35174208	3	1.280267
rs1744760	C20orf132	20	35174208	1	5.745885
rs1744769	C20orf132	20	35203006	4	1.359123
rs1744769	C20orf132	20	35203006	8	3.42095
rs1615246	C20orf132	20	35216908	1	4.965162
rs1615246	C20orf132	20	35216908	8	1.220255
rs1615246	C20orf132	20	35216908	3	0.615234
rs1615246	C20orf132	20	35216908	5	0.365413
SNP20-35229955	C20orf132	20	35229955	1	3.462651
rs1043415	MANBAL	20	35378652	6	0.825252
rs1043415	MANBAL	20	35378652	4	0.549697
rs1043415	MANBAL	20	35378652	1	5.235427
rs6095711	NULL	20	35740301	3	5.329173
rs12662	VSTM2L	20	36007043	3	2.623201

rs1053932	KIAA1755	20	36274665	7	0.583185
rs1053932	KIAA1755	20	36274665	3	0.380476
rs1053931	KIAA1755	20	36274707	3	1.116732
rs1053931	KIAA1755	20	36274707	7	0.981215
rs1534967	KIAA1755	20	36275170	3	0.247556
rs1534967	KIAA1755	20	36275170	7	1.237893
rs3746471	KIAA1755	20	36275328	7	0.930918
rs760998	KIAA1755	20	36279152	7	1.39277
rs1205418	KIAA1755	20	36293125	2	1.658857
rs1205418	KIAA1755	20	36293125	4	1.820759
rs1205418	KIAA1755	20	36293125	7	1.061754
rs1205418	KIAA1755	20	36293125	8	1.351851
rs1205418	KIAA1755	20	36293125	3	2.152181
rs1341023	BPI	20	36366074	4	0.569301
rs1341023	BPI	20	36366074	6	0.602002
rs1341023	BPI	20	36366074	5	0.454102
rs1341023	BPI	20	36366074	2	2.91311
rs1341023	BPI	20	36366074	3	0.694385
rs1341024	BPI	20	36366090	6	2.378974
rs1341024	BPI	20	36366090	5	1.199022
rs1341024	BPI	20	36366090	2	4.093168
rs1341024	BPI	20	36366090	3	1.684358
rs1341024	BPI	20	36366090	4	2.614759
rs5743509	BPI	20	36373728	3	3.12377
rs4358188	BPI	20	36380262	7	0.332604
rs4358188	BPI	20	36380262	5	0.680049
rs4358188	BPI	20	36380262	4	1.161925
rs2232582	LBP	20	36412679	1	2.287093
rs2232582	LBP	20	36412679	3	2.518092
rs2232582	LBP	20	36412679	2	2.61461
rs2232596	LBP	20	36422795	7	0.263553
rs2232596	LBP	20	36422795	3	0.213551
rs745144	LBP	20	36438950	5	0.722697
rs745144	LBP	20	36438950	7	1.292806
rs745144	LBP	20	36438950	6	0.865168
rs745144	LBP	20	36438950	2	3.439499
rs745144	LBP	20	36438950	4	0.75587
rs6070809	C20orf95	20	36691004	7	1.315587
rs2245231	ACTR5	20	36829534	5	0.343173
rs2245231	ACTR5	20	36829534	3	0.060856
rs4810245	PPP1R16B	20	36982723	3	0.504156
rs10392	PPP1R16B	20	36984349	5	0.548494
rs10392	PPP1R16B	20	36984349	3	1.418687
rs36053162	DHX35	20	37054466	7	0.77346
rs6072300	ZHX3	20	39246101	4	4.149898
rs6072300	ZHX3	20	39246101	5	0.482207
rs2664537	ZHX3	20	39247143	5	0.77137

rs2664537	ZHX3	20	39247143	4	8.367497
rs6029617	ZHX3	20	39362060	2	3.111102
rs41277016	LPIN3	20	39408038	5	0.30597
rs6072351	LPIN3	20	39421553	5	0.27407
rs6072351	LPIN3	20	39421553	2	5.297515
rs6072352	EMILIN3	20	39423140	5	0.024223
rs1156	CHD6	20	39465443	4	8.802556
rs3746539	PTPRT	20	40135485	4	1.141773
rs3746539	PTPRT	20	40135485	3	0.603954
rs2664587	PTPRT	20	40136226	3	0.983908
rs2664587	PTPRT	20	40136226	6	1.229823
rs2664587	PTPRT	20	40136226	4	0.950393
rs2144011	PTPRT	20	40136541	3	1.847588
rs2144011	PTPRT	20	40136541	6	0.327321
rs1065155	PTPRT	20	40139770	3	1.948526
rs2866943	PTPRT	20	40141293	3	0.303876
rs6065627	SGK2	20	41628216	5	1.65939
rs285162	MYBL2	20	41762053	4	2.665528
rs285162	MYBL2	20	41762053	7	6.274189
rs1055716	JPH2	20	42174113	5	0.823684
rs1055716	JPH2	20	42174113	3	0.60462
rs2064381	JPH2	20	42239843	3	0.472992
rs2064381	JPH2	20	42239843	7	0.716908
rs2064381	JPH2	20	42239843	5	1.497726
rs2064381	JPH2	20	42239843	6	0.65429
rs1883790	JPH2	20	42248604	5	0.470247
rs11274	C20orf111	20	42259793	7	0.621092
rs731498	C20orf111	20	42272818	2	7.926658
rs731498	C20orf111	20	42272818	7	0.590104
rs731498	C20orf111	20	42272818	5	5.155751
rs731498	C20orf111	20	42272818	4	1.675555
rs3810511	GDAP1L1	20	42342379	2	3.099612
rs11699901	R3HDML	20	42399255	7	0.684624
rs1800961	HNF4A	20	42475778	8	0.598877
rs6130615	HNF4A	20	42492851	7	1.593763
rs2296530	WISP2	20	42782149	4	0.602839
rs2296530	WISP2	20	42782149	8	1.115801
rs2296530	WISP2	20	42782149	3	0.073971
rs2296530	WISP2	20	42782149	1	2.490228
rs6017414	RIMS4	20	42814589	1	0.769759
rs6017414	RIMS4	20	42814589	5	0.636902
rs1980577	RIMS4	20	42815526	2	1.277773
rs1980577	RIMS4	20	42815526	6	1.107508
rs1980577	RIMS4	20	42815526	1	1.35602
rs6073629	STK4	20	43139973	7	0.787326
rs6073629	STK4	20	43139973	2	7.974929
rs6073629	STK4	20	43139973	3	0.699499

rs6130753	WFDC5	20	43171582	3	0.417991
rs6130753	WFDC5	20	43171582	2	2.033344
rs6130753	WFDC5	20	43171582	5	0.766832
rs6130753	WFDC5	20	43171582	8	3.30828
rs6130753	WFDC5	20	43171582	4	1.149968
rs17422688	WFDC5	20	43172533	3	0.962092
rs17422688	WFDC5	20	43172533	6	1.583249
rs6104017	WFDC12	20	43185582	6	0.176811
rs6104017	WFDC12	20	43185582	5	1.376715
rs6104018	WFDC12	20	43185681	4	0.120714
rs6104018	WFDC12	20	43185681	3	0.619838
rs6104018	WFDC12	20	43185681	5	0.785812
rs2664581	PI3	20	43237936	3	0.109973
rs2664581	PI3	20	43237936	6	0.505559
rs2233882	SEMG1	20	43269097	3	0.117602
rs2233901	SEMG2	20	43284508	6	0.590725
rs2233901	SEMG2	20	43284508	3	0.785621
rs2227275	MATN4	20	43359987	5	0.004002
rs2741500	RBPJL	20	43378372	6	4.529409
SNP20-4343757	C20orf10	20	43437573	3	0.094844
SNP20-4343757	C20orf10	20	43437573	4	1.270293
SNP20-4343757	C20orf10	20	43437573	8	0.058924
rs3746593	WFDC6	20	43596263	7	0.305278
rs765156	SPINLW1	20	43607837	5	0.704835
rs765156	SPINLW1	20	43607837	3	1.381103
rs765156	SPINLW1	20	43607837	1	3.596193
rs2250860	WFDC8	20	43617789	1	5.138929
rs2250860	WFDC8	20	43617789	4	1.957444
rs1487322	WFDC10A	20	43693087	1	4.066603
rs1487322	WFDC10A	20	43693087	8	2.282104
rs1487322	WFDC10A	20	43693087	3	3.134807
rs1487322	WFDC10A	20	43693087	6	1.642445
rs6032449	WFDC13	20	43770130	1	0.312143
rs6032449	WFDC13	20	43770130	3	0.191437
rs6032449	WFDC13	20	43770130	7	0.968984
rs6032449	WFDC13	20	43770130	8	1.316438
rs6032449	WFDC13	20	43770130	2	2.53328
rs6032449	WFDC13	20	43770130	5	1.049611
rs6032449	WFDC13	20	43770130	6	0.498138
rs6017667	SPINT4	20	43786034	2	1.475893
rs6017667	SPINT4	20	43786034	3	0.207896
rs6017667	SPINT4	20	43786034	6	0.055487
rs6017667	SPINT4	20	43786034	8	1.917539
rs6017667	SPINT4	20	43786034	1	0.143662
rs6017667	SPINT4	20	43786034	5	0.907037
rs6032474	SPINT4	20	43787786	5	1.138715
rs6032474	SPINT4	20	43787786	6	1.113747

rs6032474	SPINT4	20	43787786	1	0.525998
rs6032474	SPINT4	20	43787786	3	0.361463
rs6032474	SPINT4	20	43787786	2	1.227976
rs232259	SPINT4	20	43787801	5	1.185921
rs232259	SPINT4	20	43787801	1	2.052275
rs232259	SPINT4	20	43787801	7	1.272991
rs232259	SPINT4	20	43787801	3	1.776792
rs2664529	WFDC3	20	43836276	1	1.378591
rs2664529	WFDC3	20	43836276	8	0.644643
rs2664529	WFDC3	20	43836276	2	1.692748
rs2664529	WFDC3	20	43836276	3	1.30206
rs2664529	WFDC3	20	43836276	6	0.216966
rs6032538	WFDC3	20	43851082	1	0.212635
rs6032538	WFDC3	20	43851082	5	0.026716
rs6032538	WFDC3	20	43851082	6	0.816314
rs6032538	WFDC3	20	43851082	3	0.658635
rs6032538	WFDC3	20	43851082	2	0.638049
rs8860	TNNC2	20	43885308	8	1.288829
rs8860	TNNC2	20	43885308	7	0.077129
rs8860	TNNC2	20	43885308	3	0.063662
rs4629	TNNC2	20	43886104	8	3.927599
rs4629	TNNC2	20	43886104	1	0.851156
rs4629	TNNC2	20	43886104	2	3.93566
rs4629	TNNC2	20	43886104	3	1.022353
rs4629	TNNC2	20	43886104	6	0.371068
rs4638862	SNX21	20	43902697	3	1.491096
rs4638862	SNX21	20	43902697	1	0.971119
rs4638862	SNX21	20	43902697	7	0.671737
rs4638862	SNX21	20	43902697	2	0.783401
rs4638862	SNX21	20	43902697	8	3.744231
rs3848713	SNX21	20	43904083	8	3.277192
rs3848713	SNX21	20	43904083	3	0.342763
SNP20-44015842	ZNF335	20	44015842	8	0.071878
rs6032606	ZNF335	20	44029614	2	4.700167
rs6032606	ZNF335	20	44029614	6	0.0627
rs2250889	MMP9	20	44075813	6	3.956323
rs2274756	MMP9	20	44076518	3	1.762768
rs2274756	MMP9	20	44076518	7	1.086343
rs2274756	MMP9	20	44076518	6	0.975101
rs20544	MMP9	20	44078417	1	5.134213
rs20544	MMP9	20	44078417	3	0.195698
rs20544	MMP9	20	44078417	8	3.615285
rs20544	MMP9	20	44078417	5	1.408057
rs20544	MMP9	20	44078417	4	1.477456
rs1128536	SLC12A5	20	44121406	4	0.168119
rs1128536	SLC12A5	20	44121406	7	1.1467
rs9074	SLC12A5	20	44122072	1	4.296234

rs9074	SLC12A5	20	44122072	3	0.602888
rs9074	SLC12A5	20	44122072	8	0.959732
rs9074	SLC12A5	20	44122072	5	0.446756
rs3746523	NCOA5	20	44123036	8	0.454064
rs3746523	NCOA5	20	44123036	5	0.376472
rs3746523	NCOA5	20	44123036	1	2.195675
rs2281224	LOC728443	20	44313694	5	1.491752
rs2281224	LOC728443	20	44313694	3	1.249664
rs1044369	SLC35C2	20	44420725	3	0.209595
rs1044369	SLC35C2	20	44420725	5	0.946305
rs1044369	SLC35C2	20	44420725	1	5.54143
rs1044369	SLC35C2	20	44420725	7	0.157921
rs1044369	SLC35C2	20	44420725	4	0.807585
rs847052	ZNF334	20	44575240	6	2.980678
rs847052	ZNF334	20	44575240	7	2.958793
rs847052	ZNF334	20	44575240	5	1.080638
rs847052	ZNF334	20	44575240	4	5.34807
rs12617	SLC13A3	20	44620155	7	0.620633
rs12617	SLC13A3	20	44620155	4	0.066466
rs8149	SLC13A3	20	44620648	4	0.101374
rs8149	SLC13A3	20	44620648	7	1.442666
rs202391	SLC13A3	20	44628311	4	5.98287
rs1136544	SLC2A10	20	44798245	8	7.161608
rs1136544	SLC2A10	20	44798245	4	0.56631
rs1136544	SLC2A10	20	44798245	5	0.077158
rs1136544	SLC2A10	20	44798245	3	0.538685
rs1136544	SLC2A10	20	44798245	7	4.720622
rs1136544	SLC2A10	20	44798245	6	2.424795
rs6094752	NCOA3	20	45689831	7	0.053782
rs2230782	NCOA3	20	45698295	7	0.28708
SNP20-4579904	SULF2	20	45799043	7	0.327018
SNP20-4579904	SULF2	20	45799043	4	0.25987
SNP20-4579904	SULF2	20	45799043	6	0.399582
SNP20-4579904	SULF2	20	45799043	8	7.982149
rs2664570	PREX1	20	46674765	3	0.659356
rs2598	PREX1	20	46675025	3	0.69082
rs2598	PREX1	20	46675025	8	1.695422
rs16993997	PREX1	20	46692170	3	0.598023
rs16993997	PREX1	20	46692170	5	0.457449
rs6066802	PREX1	20	46694424	3	0.440704
rs6012504	PREX1	20	46695956	1	2.146622
rs41283558	PREX1	20	46702640	5	0.349622
rs41283558	PREX1	20	46702640	3	0.221041
rs3746820	PREX1	20	46741025	6	0.125163
rs3746820	PREX1	20	46741025	5	0.598845
rs3746820	PREX1	20	46741025	4	1.792446
rs7689	ZNFX1	20	47295857	1	0.513054

rs8123912	ZNFX1	20	47296689	3	0.10378
rs8123912	ZNFX1	20	47296689	4	0.74377
rs8123912	ZNFX1	20	47296689	8	0.83239
rs6512577	ZNFX1	20	47299191	4	0.006238
rs6512577	ZNFX1	20	47299191	3	0.502362
rs6512577	ZNFX1	20	47299191	8	0.134483
GA017266	ZNFX1	20	47305784	1	0.465646
GA017266	ZNFX1	20	47305784	2	7.77665
GA017266	ZNFX1	20	47305784	6	9.431555
rs1051295	KCNB1	20	47422312	3	1.296807
rs5628	PTGIS	20	47574089	2	0.091522
rs2235855	B4GALT5	20	47692441	8	3.002167
rs2235855	B4GALT5	20	47692441	4	0.045057
rs1062840	SLC9A8	20	47938873	3	0.277555
rs495337	SPATA2	20	47955737	3	0.024427
rs495337	SPATA2	20	47955737	1	0.26422
rs6067283	KRT18P4	20	48007458	3	0.60141
rs6067283	KRT18P4	20	48007458	6	1.621145
SNP20-4803403	SNAI1	20	48034038	3	0.886908
rs1543442	SNAI1	20	48038206	4	4.484191
rs1543442	SNAI1	20	48038206	6	0.310738
rs8184448	LOC284751	20	48348196	5	0.169109
rs8184448	LOC284751	20	48348196	3	2.251651
rs8184448	LOC284751	20	48348196	1	5.699613
rs8184448	LOC284751	20	48348196	4	0.840421
rs4811111	KCNG1	20	49058855	7	1.263583
rs4809820	KCNG1	20	49059030	7	0.322063
rs17802735	SALL4	20	49838909	1	2.816881
SNP20-4984090	SALL4	20	49840909	4	7.670384
SNP20-4984090	SALL4	20	49840909	1	0.243648
SNP20-4984090	SALL4	20	49840909	2	0.120857
rs3746415	ZFP64	20	50202590	3	0.645025
rs3746415	ZFP64	20	50202590	4	0.548535
rs739869	TSHZ2	20	51303743	6	3.619137
rs1952	BCAS1	20	51994050	2	3.194376
rs1055251	BCAS1	20	51994191	6	1.369103
rs1055251	BCAS1	20	51994191	1	5.971269
rs1055249	BCAS1	20	51994199	4	3.534964
rs1055249	BCAS1	20	51994199	5	3.110959
rs1055249	BCAS1	20	51994199	1	5.686346
rs1055249	BCAS1	20	51994199	6	0.680302
rs35575210	BCAS1	20	52007378	2	1.948293
rs6022903	BCAS1	20	52045956	3	0.800352
rs394732	BCAS1	20	52108595	6	1.300773
rs394732	BCAS1	20	52108595	3	0.191529
rs394732	BCAS1	20	52108595	8	2.006324
rs394732	BCAS1	20	52108595	1	4.383573

rs2020975	BCAS1	20	52108666	4	0.199622
rs2020975	BCAS1	20	52108666	8	3.178272
rs16998896	BCAS1	20	52120434	7	2.24509
rs16998896	BCAS1	20	52120434	3	3.146753
rs4809957	CYP24A1	20	52204578	3	0.488029
rs4809957	CYP24A1	20	52204578	7	0.085194
rs4809957	CYP24A1	20	52204578	2	2.716496
rs2762934	CYP24A1	20	52204668	1	1.509773
rs2762934	CYP24A1	20	52204668	8	1.182286
rs6068816	CYP24A1	20	52214498	6	5.114048
rs2296241	CYP24A1	20	52219626	4	3.669177
rs2296241	CYP24A1	20	52219626	6	0.083761
rs2296241	CYP24A1	20	52219626	8	2.284395
rs2296241	CYP24A1	20	52219626	1	4.40742
rs2296241	CYP24A1	20	52219626	7	1.216285
rs2296241	CYP24A1	20	52219626	3	1.358599
rs2842	DOK5	20	52700989	7	0.485531
rs15899	DOK5	20	52701034	1	0.394485
rs15899	DOK5	20	52701034	4	1.405197
rs15899	DOK5	20	52701034	3	0.687144
rs15899	DOK5	20	52701034	5	0.235493
rs2843	DOK5	20	52701042	2	0.401677
rs2843	DOK5	20	52701042	3	0.056622
rs2843	DOK5	20	52701042	1	0.753071
rs3746619	MC3R	20	54257212	3	3.923252
rs3827103	MC3R	20	54257436	3	1.366089
rs3746624	C20orf32	20	54467042	1	0.156272
rs3746624	C20orf32	20	54467042	3	0.38276
rs3746626	C20orf32	20	54467120	1	0.979603
rs3746626	C20orf32	20	54467120	3	0.251593
rs2296130	C20orf107	20	54544778	1	3.405687
rs2296130	C20orf107	20	54544778	5	3.397266
rs11697430	LOC728902	20	55170571	2	2.191766
rs11697476	LOC728902	20	55170641	8	0.055243
rs11697476	LOC728902	20	55170641	2	0.187524
rs10375	BMP7	20	55179088	6	2.55044
rs10375	BMP7	20	55179088	5	0.167184
rs10375	BMP7	20	55179088	7	3.73022
rs10375	BMP7	20	55179088	1	3.031939
rs6064531	SPO11	20	55352346	3	0.183396
rs6070067	SPO11	20	55352394	1	1.08819
rs6070067	SPO11	20	55352394	5	1.486386
rs6070067	SPO11	20	55352394	7	2.15145
rs6070122	CTCFL	20	55517169	3	0.862729
rs6070122	CTCFL	20	55517169	4	0.081173
rs1042521	PCK1	20	55569942	1	0.114033
rs1042521	PCK1	20	55569942	4	0.559387

rs1042521	PCK1	20	55569942	7	0.164776
rs1042521	PCK1	20	55569942	6	1.217316
rs1062601	PCK1	20	55571240	1	0.792585
rs1062601	PCK1	20	55571240	4	0.421968
rs1062601	PCK1	20	55571240	7	0.915867
SNP20-55572054	PCK1	20	55572054	4	1.709058
rs1042531	PCK1	20	55574386	7	0.052599
rs1042531	PCK1	20	55574386	3	0.296774
rs4811885	ZBP1	20	55612334	5	0.758263
rs4811885	ZBP1	20	55612334	2	1.122423
rs4811885	ZBP1	20	55612334	1	0.881639
rs6064572	ZBP1	20	55612828	5	0.522352
rs41275646	ZBP1	20	55618615	4	0.087711
rs2073145	ZBP1	20	55624040	5	1.203346
rs2073145	ZBP1	20	55624040	3	0.931099
rs6025692	TMEPAI	20	55658139	1	4.621764
rs584855	C20orf86	20	56227112	5	0.261967
rs6026220	RAB22A	20	56373559	5	0.522025
rs6026220	RAB22A	20	56373559	6	0.800017
rs6026220	RAB22A	20	56373559	3	1.13152
rs1045408	RAB22A	20	56375488	8	7.767637
rs1045408	RAB22A	20	56375488	4	0.167032
rs1045408	RAB22A	20	56375488	2	2.649128
rs6070466	VAPB	20	56453450	7	0.628391
rs1059794	VAPB	20	56453634	1	4.525779
rs2180481	APCDD1L	20	56468469	5	1.145156
rs2295440	APCDD1L	20	56468482	5	0.355857
rs2295440	APCDD1L	20	56468482	1	5.020526
rs2295440	APCDD1L	20	56468482	6	0.530986
rs2295440	APCDD1L	20	56468482	4	1.263607
SNP20-56476071	APCDD1L	20	56476071	5	1.406478
SNP20-56479171	APCDD1L	20	56479171	5	1.035241
rs218457	MGC4294	20	56643660	8	0.405297
rs218457	MGC4294	20	56643660	6	1.634544
rs218457	MGC4294	20	56643660	1	0.346765
rs218457	MGC4294	20	56643660	7	2.718901
rs3761133	MGC4294	20	56643886	6	0.983302
rs3761133	MGC4294	20	56643886	1	0.050915
rs3761133	MGC4294	20	56643886	8	0.854501
rs2296524	STX16	20	56677899	5	0.783205
rs2296524	STX16	20	56677899	7	0.316768
rs2296524	STX16	20	56677899	2	0.389366
rs2296524	STX16	20	56677899	4	0.045706
rs2296524	STX16	20	56677899	6	0.263649
rs2296524	STX16	20	56677899	1	0.011618
rs1800900	GNAS	20	56848505	3	0.805893
rs1800900	GNAS	20	56848505	4	2.303338

rs3730171	GNAS	20	56917636	4	0.558128
rs34563478	TH1L	20	56998090	2	5.679988
rs10485828	TUBB1	20	57034050	5	0.029381
rs10485828	TUBB1	20	57034050	3	0.311916
rs10485828	TUBB1	20	57034050	6	1.962089
rs10485828	TUBB1	20	57034050	8	2.857815
rs259956	C20orf174	20	57262696	1	3.042696
rs13039338	SYCP2	20	57910236	6	0.787896
rs7267644	CDH26	20	58015195	1	4.09367
rs11698042	LOC284757	20	58328202	7	3.636338
rs3761222	CDH4	20	59945423	5	0.257404
rs3761222	CDH4	20	59945423	2	6.896447
rs1135961	PSMA7	20	60146727	6	0.218162
rs1135961	PSMA7	20	60146727	4	0.264059
rs2236527	GTPBP5	20	60201892	5	0.189335
rs2184161	GTPBP5	20	60209670	1	6.138971
rs2184161	GTPBP5	20	60209670	7	0.447329
rs6142991	GTPBP5	20	60209868	5	5.370279
rs2184157	GTPBP5	20	60210550	1	4.201163
rs2184157	GTPBP5	20	60210550	8	6.139837
rs2184157	GTPBP5	20	60210550	7	0.166667
rs6121980	OSBPL2	20	60303075	5	0.230249
rs6121980	OSBPL2	20	60303075	1	5.24812
rs41307205	LAMA5	20	60321086	3	0.358532
rs2297587	LAMA5	20	60329092	1	5.453149
rs2297587	LAMA5	20	60329092	8	0.062708
SNP20-60331167	LAMA5	20	60331167	1	2.530138
SNP20-60331167	LAMA5	20	60331167	8	0.018202
SNP20-60331167	LAMA5	20	60331167	5	0.511524
rs2427286	LAMA5	20	60338248	3	0.604978
rs2151513	LAMA5	20	60340841	3	0.040848
rs709206	CABLES2	20	60397170	3	0.283502
rs6122080	SLCO4A1	20	60758172	7	0.192181
rs6122080	SLCO4A1	20	60758172	3	0.202631
rs34985812	SLCO4A1	20	60769970	7	0.400532
rs2427444	NTSR1	20	60864318	2	4.371992
rs2249903	COL9A3	20	60930569	1	1.94528
rs2249903	COL9A3	20	60930569	4	0.758378
rs751557	COL9A3	20	60933967	8	1.316755
rs751557	COL9A3	20	60933967	1	0.228112
rs751557	COL9A3	20	60933967	7	1.620911
rs751557	COL9A3	20	60933967	2	0.288802
rs751557	COL9A3	20	60933967	4	1.299683
rs2294995	COL9A3	20	60939016	1	4.856257
rs2294995	COL9A3	20	60939016	3	0.55562
rs1048100	COL9A3	20	60942518	7	0.955958
rs1048100	COL9A3	20	60942518	6	3.116586

SNP20-60961939	TCFL5	20	60961939	7	0.833964
SNP20-60961939	TCFL5	20	60961939	6	5.966719
rs6011447	DIDO1	20	60989677	1	0.557339
rs6011447	DIDO1	20	60989677	8	0.699456
rs6011447	DIDO1	20	60989677	3	0.330451
rs6011447	DIDO1	20	60989677	6	0.461658
rs6011447	DIDO1	20	60989677	4	0.145799
rs6011447	DIDO1	20	60989677	2	1.142811
rs3746767	DIDO1	20	60990311	6	0.856206
rs3746767	DIDO1	20	60990311	8	5.575291
rs3746767	DIDO1	20	60990311	3	0.946739
rs3746767	DIDO1	20	60990311	1	3.634219
rs3746766	DIDO1	20	60990433	4	0.959945
rs3746766	DIDO1	20	60990433	8	0.573319
rs3746766	DIDO1	20	60990433	1	0.114715
rs3746766	DIDO1	20	60990433	3	0.589728
rs3746766	DIDO1	20	60990433	2	2.644889
rs3746766	DIDO1	20	60990433	6	0.113706
rs3746765	DIDO1	20	60991239	1	0.139019
rs3746765	DIDO1	20	60991239	8	0.187065
rs3746765	DIDO1	20	60991239	3	0.418754
rs6090153	DIDO1	20	60991628	6	0.146074
rs6090153	DIDO1	20	60991628	1	3.790266
rs6090153	DIDO1	20	60991628	3	0.492939
rs6090153	DIDO1	20	60991628	8	0.117006
rs2273083	DIDO1	20	60991960	3	0.965843
rs2273083	DIDO1	20	60991960	4	0.817065
rs2273083	DIDO1	20	60991960	6	2.189065
rs2273083	DIDO1	20	60991960	8	0.302272
rs2273083	DIDO1	20	60991960	2	3.277773
rs2273083	DIDO1	20	60991960	1	1.197253
rs1883847	DIDO1	20	60998716	1	5.139866
rs1883847	DIDO1	20	60998716	7	0.340546
rs2295000	DIDO1	20	61007383	8	0.418378
rs2295000	DIDO1	20	61007383	3	0.462637
rs2295000	DIDO1	20	61007383	6	3.039217
rs2245341	C20orf59	20	61058604	3	0.45753
rs2245341	C20orf59	20	61058604	6	0.673884
rs2245341	C20orf59	20	61058604	7	1.851294
rs2248900	C20orf59	20	61066081	8	0.848096
rs2248900	C20orf59	20	61066081	6	0.762011
rs2248900	C20orf59	20	61066081	7	0.301476
rs2248900	C20orf59	20	61066081	3	0.337451
rs752430	C20orf59	20	61069833	3	0.125238
rs752430	C20orf59	20	61069833	8	0.838105
rs752430	C20orf59	20	61069833	2	3.923183
rs752430	C20orf59	20	61069833	6	3.271366

rs6090206	C20orf51	20	61138808	3	1.027804
rs1077019	BIRC7	20	61341172	7	0.639462
rs872808	NKAIN4	20	61349395	4	0.569644
rs2236194	NKAIN4	20	61349454	7	1.708544
rs1129659	NKAIN4	20	61350614	7	0.33818
rs1129659	NKAIN4	20	61350614	8	0.985726
rs927081	NKAIN4	20	61351741	8	2.225675
rs11542296	ARFGAP1	20	61378427	8	2.079254
rs11542296	ARFGAP1	20	61378427	4	0.086471
rs1044397	CHRNA4	20	61451548	4	1.665554
rs1044396	CHRNA4	20	61451578	2	1.193985
rs2297385	KCNQ2	20	61541410	5	0.120271
rs2297385	KCNQ2	20	61541410	7	1.906565
rs2297385	KCNQ2	20	61541410	6	0.638656
rs2274860	EEF1A2	20	61596743	4	4.341225
rs11551685	PRIC285	20	61662381	8	8.150501
rs3810481	PRIC285	20	61663889	6	1.667515
rs3810481	PRIC285	20	61663889	2	1.630216
rs3810481	PRIC285	20	61663889	7	0.030143
SNP20-6166515	PRIC285	20	61665157	6	0.496804
rs310630	PRIC285	20	61665531	7	1.144657
rs310630	PRIC285	20	61665531	2	1.316308
rs310630	PRIC285	20	61665531	3	0.540449
SNP20-6166647	PRIC285	20	61666477	7	0.634111
SNP20-6166647	PRIC285	20	61666477	8	1.322038
SNP20-6166647	PRIC285	20	61666477	6	0.232293
rs310631	PRIC285	20	61666697	3	0.255879
rs310631	PRIC285	20	61666697	7	0.048342
rs1757752	PRIC285	20	61671304	3	0.355628
rs311496	GMEB2	20	61692054	5	6.599609
rs311496	GMEB2	20	61692054	3	0.852045
rs41302954	RTEL1	20	61779958	8	0.967483
rs6062302	RTEL1	20	61791412	8	3.090108
rs6062302	RTEL1	20	61791412	3	0.989271
rs6062302	RTEL1	20	61791412	7	0.144779
rs3208007	RTEL1	20	61792732	7	0.772273
rs3208007	RTEL1	20	61792732	3	0.836253
SNP20-6179881	TNFRSF6B	20	61798819	3	0.224774
SNP20-6179881	TNFRSF6B	20	61798819	5	0.27453
rs1291212	ZGPAT	20	61810559	5	0.023409
rs1151622	ZGPAT	20	61836000	7	3.935286
rs1151622	ZGPAT	20	61836000	8	3.40528
rs1151622	ZGPAT	20	61836000	3	0.647024
rs2427533	ZGPAT	20	61836310	7	1.072375
rs2427533	ZGPAT	20	61836310	3	0.687583
rs2427533	ZGPAT	20	61836310	8	2.227095
rs2427535	ZGPAT	20	61837821	5	0.314581

rs1151625	LIME1	20	61840441	5	7.253375
rs2427536	SLC2A4RG	20	61843257	5	0.717547
rs2427537	ZBTB46	20	61846671	4	0.927382
rs2427537	ZBTB46	20	61846671	5	0.02034
rs2427537	ZBTB46	20	61846671	3	0.469057
rs2427537	ZBTB46	20	61846671	7	3.587873
rs2427537	ZBTB46	20	61846671	8	5.159803
rs755017	ZBTB46	20	61892066	5	0.805942
rs2281929	ZBTB46	20	61892524	5	0.737581
rs817325	ZNF512B	20	62065613	5	0.921944
rs6062599	ZNF512B	20	62065991	1	1.946553
rs6062599	ZNF512B	20	62065991	4	0.431858
SNP20-6206643	ZNF512B	20	62066434	1	4.442536
SNP20-6206643	ZNF512B	20	62066434	4	0.709927
SNP20-6206811	ZNF512B	20	62068110	4	0.343341
SNP20-6206811	ZNF512B	20	62068110	1	5.4456
rs817343	SAMD10	20	62077604	1	4.029876
rs2229205	OPRL1	20	62199875	4	1.337775
rs13036542	NPBWR2	20	62207762	6	2.889843
rs4809401	NPBWR2	20	62208012	2	2.304049
rs41279348	MYT1	20	62320802	7	0.60741
rs2983433	MYT1	20	62342396	3	0.058742
rs11702489	LOC441956	21	13974683	3	0.885032
rs11702489	LOC441956	21	13974683	7	2.247581
rs11702489	LOC441956	21	13974683	1	2.567774
rs2822446	RBM11	21	14521719	3	0.951091
rs2822448	RBM11	21	14521925	5	0.291258
rs2822448	RBM11	21	14521925	2	5.030268
rs2822448	RBM11	21	14521925	7	0.533073
rs2822554	ABCC13	21	14585162	3	1.069435
rs2822558	ABCC13	21	14593715	3	0.591087
rs1153312	ABCC13	21	14594247	7	0.840105
rs17002670	STCH	21	14667761	2	3.652702
rs7281104	SAMSN1	21	14840448	3	1.398119
rs1056947	NRIP1	21	15256529	6	4.198194
rs2229742	NRIP1	21	15261043	4	0.224147
rs1047978	C21orf91	21	18091026	1	5.556823
rs1047978	C21orf91	21	18091026	3	1.076684
rs1047978	C21orf91	21	18091026	4	0.705066
rs154742	LOC643081	21	18356061	3	0.893792
rs154742	LOC643081	21	18356061	1	4.337043
rs154742	LOC643081	21	18356061	5	0.164974
rs2051358	CHODL	21	18561045	7	0.13031
rs2051358	CHODL	21	18561045	3	0.503947
rs2051358	CHODL	21	18561045	4	0.968847
rs2051358	CHODL	21	18561045	2	1.350834
rs11909235	CHODL	21	18561066	4	3.787217

rs11909235	CHODL	21	18561066	2	7.707818
rs11909235	CHODL	21	18561066	7	0.271755
rs11909235	CHODL	21	18561066	3	0.141811
rs1051530	CHODL	21	18561448	7	1.895048
rs1051530	CHODL	21	18561448	3	1.603856
rs1051530	CHODL	21	18561448	2	3.854392
rs1051530	CHODL	21	18561448	4	1.239503
rs7276699	PRSS7	21	18564156	4	2.226742
rs7276699	PRSS7	21	18564156	7	1.912432
rs7276699	PRSS7	21	18564156	3	1.670723
rs232518	NCAM2	21	21668058	2	4.285886
rs915617	LOC391273	21	23684542	1	0.176926
rs2187053	LOC284821	21	25657058	4	0.358749
rs2187053	LOC284821	21	25657058	1	0.4338
rs1893650	MIRN155	21	25862686	7	0.456921
rs219643	CYYR1	21	26762438	3	0.799231
rs17002187	CYYR1	21	26762664	6	0.327196
rs409630	ADAMTS1	21	27138545	5	0.254509
rs409630	ADAMTS1	21	27138545	2	1.526113
rs229071	ADAMTS5	21	27212867	7	1.019996
rs1444269	ADAMTS5	21	27213326	2	4.293824
rs1444269	ADAMTS5	21	27213326	6	2.436648
rs2830581	ADAMTS5	21	27215671	6	1.693164
rs9978597	ADAMTS5	21	27216014	5	0.363502
rs229077	ADAMTS5	21	27217210	6	0.176173
rs226794	ADAMTS5	21	27224226	5	4.875884
SNP21-2726016	ADAMTS5	21	27260169	3	3.726226
SNP21-2926099	ZNF294	21	29260991	6	3.323028
rs2245431	ZNF294	21	29279034	6	0.418358
rs2070610	CCT8	21	29367819	6	0.150158
rs2832237	C21orf7	21	29469554	6	1.230305
rs2832237	C21orf7	21	29469554	8	4.871585
rs2832237	C21orf7	21	29469554	5	0.506425
rs2832237	C21orf7	21	29469554	4	0.373985
rs2251071	KRTAP19-3	21	30785718	3	0.529741
rs9977815	TIAM1	21	31758190	4	2.771232
rs9977815	TIAM1	21	31758190	1	0.17032
rs2070371	HUNK	21	32290059	1	1.751924
rs2070371	HUNK	21	32290059	2	1.144593
rs2070371	HUNK	21	32290059	4	0.408238
rs9305483	HUNK	21	32292771	1	0.887711
rs9305483	HUNK	21	32292771	5	3.502038
rs9305483	HUNK	21	32292771	4	0.521803
rs9305483	HUNK	21	32292771	8	0.807763
rs2070372	HUNK	21	32293411	1	0.327256
rs2070372	HUNK	21	32293411	4	0.03677
rs2070373	HUNK	21	32293526	2	0.981186

rs2070373	HUNK	21	32293526	1	1.019202
rs2070373	HUNK	21	32293526	5	0.999678
rs2070373	HUNK	21	32293526	4	0.997518
rs3746854	HUNK	21	32294222	4	1.638461
rs3746854	HUNK	21	32294222	7	0.675437
rs3746854	HUNK	21	32294222	8	0.074552
rs3746854	HUNK	21	32294222	5	0.909221
rs3746854	HUNK	21	32294222	1	3.904216
rs3746856	HUNK	21	32294620	1	0.446467
rs3746856	HUNK	21	32294620	8	2.543504
rs3746856	HUNK	21	32294620	5	0.006191
rs3746856	HUNK	21	32294620	4	5.005193
rs3746856	HUNK	21	32294620	2	2.609701
rs3787694	HUNK	21	32295043	5	0.501781
rs3787694	HUNK	21	32295043	1	0.45519
rs3787694	HUNK	21	32295043	8	0.000424
rs3787694	HUNK	21	32295043	4	0.090322
rs3787694	HUNK	21	32295043	2	7.789116
rs3787697	HUNK	21	32295801	4	5.089874
rs3787697	HUNK	21	32295801	5	3.522662
rs3787697	HUNK	21	32295801	1	0.51113
rs3787697	HUNK	21	32295801	8	0.497168
rs3578	HUNK	21	32295973	4	0.511148
rs3578	HUNK	21	32295973	2	3.090748
rs3578	HUNK	21	32295973	1	0.898947
rs3578	HUNK	21	32295973	5	0.507365
rs3578	HUNK	21	32295973	8	2.260739
rs2833601	HUNK	21	32297384	1	0.58256
rs2833601	HUNK	21	32297384	5	0.183165
rs2833601	HUNK	21	32297384	4	0.160704
rs2833602	HUNK	21	32297433	1	0.748318
rs2833602	HUNK	21	32297433	4	0.952917
rs11702306	C21orf119	21	32687431	4	0.03634
rs2833907	C21orf77	21	32869651	3	0.983877
rs2833907	C21orf77	21	32869651	1	2.755264
rs2833907	C21orf77	21	32869651	5	0.481635
rs7520	TCP10L	21	32870930	5	0.545072
rs1783066	C21orf59	21	32895979	2	6.014234
rs11910271	C21orf49	21	33066416	7	0.113949
rs11910271	C21orf49	21	33066416	6	0.211397
rs11910271	C21orf49	21	33066416	1	8.078261
rs11910271	C21orf49	21	33066416	5	0.044118
rs11910504	C21orf49	21	33066456	5	1.094825
rs11910504	C21orf49	21	33066456	1	3.151175
rs2833955	C21orf62	21	33085302	7	0.159735
rs2833955	C21orf62	21	33085302	5	0.328436
rs2833955	C21orf62	21	33085302	1	3.263638

rs2833955	C21orf62	21	33085302	8	0.81271
rs2833956	C21orf62	21	33085613	1	3.973076
rs2833956	C21orf62	21	33085613	5	0.012958
rs4817501	C21orf62	21	33087650	1	5.07757
rs4817501	C21orf62	21	33087650	5	1.012119
rs4817501	C21orf62	21	33087650	8	0.427908
rs2231390	C21orf62	21	33088213	7	0.788202
rs2231390	C21orf62	21	33088213	1	3.186263
rs2231390	C21orf62	21	33088213	8	0.243114
rs2231390	C21orf62	21	33088213	5	0.500955
rs762178	OLIG2	21	33321271	5	0.665658
rs762178	OLIG2	21	33321271	7	0.936138
rs1059004	OLIG2	21	33322333	7	3.290279
rs9975738	IFNAR2	21	33524116	4	1.071853
rs17860115	IFNAR2	21	33524175	6	3.303555
rs17860115	IFNAR2	21	33524175	5	0.865972
rs17860115	IFNAR2	21	33524175	3	0.863547
rs17860115	IFNAR2	21	33524175	2	3.579545
rs7279064	IFNAR2	21	33536125	2	0.440039
rs7279064	IFNAR2	21	33536125	6	1.471024
rs7279064	IFNAR2	21	33536125	3	0.686565
rs7279064	IFNAR2	21	33536125	5	1.145494
rs8178561	IL10RB	21	33590667	1	4.187281
rs2850015	IFNAR1	21	33619134	7	0.307396
rs2850015	IFNAR1	21	33619134	4	0.828776
rs2850015	IFNAR1	21	33619134	3	0.860454
rs2850015	IFNAR1	21	33619134	5	1.240814
rs17875889	IFNAR1	21	33652172	6	6.936053
rs2834217	TMEM50B	21	33744020	5	0.221011
rs2834217	TMEM50B	21	33744020	1	0.101226
rs2834217	TMEM50B	21	33744020	8	5.617457
rs2834217	TMEM50B	21	33744020	2	6.577825
rs2834217	TMEM50B	21	33744020	7	0.330405
rs2834217	TMEM50B	21	33744020	4	0.894629
SNP21-33847913	SON	21	33847913	7	0.00664
SNP21-33847913	SON	21	33847913	5	0.753105
SNP21-34161432	ITSN1	21	34161432	3	0.689293
SNP21-34161432	ITSN1	21	34161432	2	3.934729
rs2073370	ITSN1	21	34182351	1	0.051083
rs2073370	ITSN1	21	34182351	3	0.449469
rs3453	KCNE1	21	34740933	1	1.349769
rs2070356	KCNE1	21	34742957	7	1.064027
rs2070356	KCNE1	21	34742957	1	0.290621
rs2070356	KCNE1	21	34742957	4	1.556545
rs1805127	KCNE1	21	34743691	3	0.245296
rs1805127	KCNE1	21	34743691	6	0.427489
rs1805127	KCNE1	21	34743691	1	2.171826

rs1805127	KCNE1	21	34743691	4	0.16648
rs2834502	KCNE1	21	34805359	1	1.675181
rs762364	SETD4	21	36328719	1	0.804377
rs762364	SETD4	21	36328719	3	0.251333
rs16993814	SETD4	21	36329941	7	0.167669
rs8738	SETD4	21	36342520	8	6.668913
rs8738	SETD4	21	36342520	4	2.724987
rs2230192	CBR1	21	36366909	7	3.522071
rs9024	CBR1	21	36367183	4	1.446835
SNP21-36681827	CHAF1B	21	36681827	3	0.044446
SNP21-36681827	CHAF1B	21	36681827	6	0.374031
SNP21-36681827	CHAF1B	21	36681827	8	0.66369
rs2073601	SIM2	21	37039178	3	0.625415
rs2073601	SIM2	21	37039178	4	2.916608
rs2073601	SIM2	21	37039178	7	2.701052
rs2073602	SIM2	21	37039375	1	0.593173
rs2073602	SIM2	21	37039375	8	0.222495
rs2073602	SIM2	21	37039375	2	4.151083
rs2073417	SIM2	21	37042588	1	2.116055
rs2073417	SIM2	21	37042588	8	1.299446
rs2051397	SIM2	21	37043407	2	3.70736
rs2051397	SIM2	21	37043407	4	0.179945
rs2051397	SIM2	21	37043407	1	0.945234
rs2051397	SIM2	21	37043407	7	0.086056
rs2051397	SIM2	21	37043407	3	0.902059
SNP21-37366751	PIGP	21	37366751	5	0.020404
SNP21-37366751	PIGP	21	37366751	3	0.213902
rs2298683	PIGP	21	37366939	7	0.273228
rs2298684	PIGP	21	37366965	8	0.909893
rs2298684	PIGP	21	37366965	5	1.038227
rs1053966	TTC3	21	37489879	5	6.196176
rs6579	TTC3	21	37490178	8	0.411047
rs13864	DSCR9	21	37515490	4	1.378085
rs13864	DSCR9	21	37515490	8	0.230185
rs2835727	DYRK1A	21	37713543	7	5.024554
rs2835728	DYRK1A	21	37713633	5	0.43884
rs13049514	DYRK1A	21	37714001	3	0.896848
rs13049514	DYRK1A	21	37714001	5	0.840868
rs13049514	DYRK1A	21	37714001	8	0.092314
rs13050229	LOC441964	21	38566256	7	2.438066
rs9963	KCNJ15	21	38594494	2	4.979592
rs1064273	KCNJ15	21	38595169	5	0.686496
rs1064273	KCNJ15	21	38595169	7	1.577731
rs1064273	KCNJ15	21	38595169	4	1.47152
rs1064273	KCNJ15	21	38595169	2	2.393137
rs2836660	C21orf24	21	39045842	4	5.253378
rs457705	ETS2	21	39113301	8	0.671956

rs457705	ETS2	21	39113301	4	1.07945
rs9653664	FLJ45139	21	39171837	5	1.056205
rs9653664	FLJ45139	21	39171837	1	0.439429
rs9653664	FLJ45139	21	39171837	8	0.078036
rs2836722	FLJ45139	21	39171878	5	1.879368
rs2836722	FLJ45139	21	39171878	8	0.730397
rs2836722	FLJ45139	21	39171878	7	0.477876
rs2836722	FLJ45139	21	39171878	1	1.066693
rs2836725	FLJ45139	21	39173584	8	2.299431
rs2836725	FLJ45139	21	39173584	2	3.08864
rs2836725	FLJ45139	21	39173584	7	4.322109
rs2836725	FLJ45139	21	39173584	1	2.413028
rs992242	FLJ45139	21	39173903	1	0.971063
rs992243	FLJ45139	21	39173997	1	0.619307
rs1882777	LOC391282	21	39421368	3	1.01534
rs9305670	PSMG1	21	39469257	3	0.394312
rs2297255	BRWD1	21	39479848	6	0.36883
rs2836933	BRWD1	21	39486072	3	1.016723
rs2836933	BRWD1	21	39486072	2	11.98566
rs2836933	BRWD1	21	39486072	8	0.367605
rs2836933	BRWD1	21	39486072	1	0.291481
rs2836933	BRWD1	21	39486072	4	1.066493
rs1041439	BRWD1	21	39493116	6	0.166831
rs34613278	HMGN1	21	39642364	2	5.543127
rs34613278	HMGN1	21	39642364	3	2.135181
rs11558767	LCA5L	21	39700052	5	7.707694
rs11558767	LCA5L	21	39700052	7	0.272994
rs11558767	LCA5L	21	39700052	1	2.494849
rs2837029	LCA5L	21	39722241	3	2.069963
rs2837029	LCA5L	21	39722241	8	0.703217
rs2837029	LCA5L	21	39722241	4	0.755933
rs2837029	LCA5L	21	39722241	2	5.249802
rs2837034	LCA5L	21	39729230	3	0.63635
rs2837034	LCA5L	21	39729230	4	0.927752
rs2837034	LCA5L	21	39729230	8	1.02945
rs11575939	SH3BGR	21	39745772	8	2.600432
rs11575939	SH3BGR	21	39745772	3	0.302941
rs11575939	SH3BGR	21	39745772	4	3.108069
rs661650	C21orf88	21	39891491	5	1.356498
rs661650	C21orf88	21	39891491	1	2.182635
rs3746887	B3GALT5	21	39954610	1	3.858142
rs3746887	B3GALT5	21	39954610	6	1.516773
rs3746887	B3GALT5	21	39954610	7	3.581759
rs3746887	B3GALT5	21	39954610	4	1.258794
rs734413	B3GALT5	21	39954674	1	3.843896
rs734413	B3GALT5	21	39954674	6	0.781248
rs734413	B3GALT5	21	39954674	7	1.703024

rs734413	B3GALT5	21	39954674	4	0.420529
rs734412	B3GALT5	21	39954932	4	3.856513
rs734412	B3GALT5	21	39954932	5	2.230592
rs909182	B3GALT5	21	39955686	1	3.004651
rs909182	B3GALT5	21	39955686	5	1.94111
rs909182	B3GALT5	21	39955686	6	0.082255
rs909182	B3GALT5	21	39955686	2	1.960117
rs2837109	B3GALT5	21	39956085	7	3.024134
rs2837109	B3GALT5	21	39956085	2	1.132764
rs2837109	B3GALT5	21	39956085	5	3.457889
rs2837109	B3GALT5	21	39956085	4	0.573508
rs2205204	IGSF5	21	40059377	2	1.685037
rs2205204	IGSF5	21	40059377	8	0.86064
rs2837225	IGSF5	21	40087332	7	2.291572
rs16999204	DSCAM	21	40337936	2	5.893862
rs16999204	DSCAM	21	40337936	5	1.035632
rs2297270	DSCAM	21	40647500	8	0.972174
rs757368	FAM3B	21	41651461	7	0.626718
rs757368	FAM3B	21	41651461	6	0.168836
rs464138	MX1	21	41720043	1	0.564913
rs464138	MX1	21	41720043	7	0.913577
rs3787950	TMPRSS2	21	41788166	6	0.081303
rs3787950	TMPRSS2	21	41788166	7	0.783122
rs6586229	C21orf129	21	42006214	5	2.668291
rs6586229	C21orf129	21	42006214	4	0.619785
rs6586229	C21orf129	21	42006214	3	1.41911
rs6586229	C21orf129	21	42006214	7	1.812789
rs13050509	RIPK4	21	42033326	1	4.86036
rs13050509	RIPK4	21	42033326	4	0.165104
rs1054756	LOC728766	21	42091933	1	5.625651
rs9981428	LOC728766	21	42093947	3	0.486823
rs9981428	LOC728766	21	42093947	8	5.901718
rs2236693	LOC728766	21	42094060	8	7.222055
rs9983923	LOC728766	21	42094180	3	0.426361
rs2236694	PRDM15	21	42094624	1	6.562256
rs4075967	PRDM15	21	42109245	1	0.546829
rs4075967	PRDM15	21	42109245	3	0.304714
rs2839402	C2CD2	21	42180969	1	4.421668
rs2839402	C2CD2	21	42180969	4	1.215989
rs17177111	C2CD2	21	42181439	3	0.848879
rs17177111	C2CD2	21	42181439	4	0.688985
rs220111	C21orf128	21	42397010	1	3.288335
rs220111	C21orf128	21	42397010	6	3.961671
rs11701371	C21orf128	21	42397041	7	2.662716
SNP21-4240407	UMODL1	21	42404077	6	1.876355
rs220146	UMODL1	21	42412362	3	0.559841
rs220146	UMODL1	21	42412362	1	0.687874

rs220159	UMODL1	21	42420942	2	0.575365
rs220159	UMODL1	21	42420942	1	1.219035
rs220159	UMODL1	21	42420942	8	1.123552
rs220159	UMODL1	21	42420942	4	0.329071
rs3819141	UMODL1	21	42430628	8	0.195358
rs915842	ABCG1	21	42552582	3	0.508932
rs915843	ABCG1	21	42552623	2	2.124485
rs915843	ABCG1	21	42552623	3	0.24545
rs2156310	TFF1	21	42659675	1	4.729375
rs2839501	TMPRSS3	21	42678706	4	1.440536
rs2839501	TMPRSS3	21	42678706	3	1.395608
rs2277798	UBASH3A	21	42697175	3	1.197805
rs13052676	UBASH3A	21	42725301	8	0.166732
rs2839536	RSPH1	21	42778956	3	1.070458
rs2839536	RSPH1	21	42778956	8	4.827988
rs2839536	RSPH1	21	42778956	1	5.412524
rs2839536	RSPH1	21	42778956	5	0.516546
rs1573413	RSPH1	21	42789408	8	2.081141
rs1573413	RSPH1	21	42789408	1	4.62875
rs1573413	RSPH1	21	42789408	3	1.927033
rs1573413	RSPH1	21	42789408	5	1.92449
rs228104	SLC37A1	21	42859024	4	0.038293
rs2156316	WDR4	21	43142894	5	4.164802
rs2156316	WDR4	21	43142894	7	6.465694
rs15736	WDR4	21	43146927	5	0.016132
rs15736	WDR4	21	43146927	7	0.358535
rs2248490	WDR4	21	43166813	4	0.868323
rs2248490	WDR4	21	43166813	5	0.585826
rs2248490	WDR4	21	43166813	7	0.586558
rs10595	NDUFV3	21	43197434	4	4.556444
rs10595	NDUFV3	21	43197434	5	2.704644
rs10595	NDUFV3	21	43197434	7	0.246309
rs10595	NDUFV3	21	43197434	6	1.098871
rs10595	NDUFV3	21	43197434	8	1.763103
rs17179001	PKNOX1	21	43324126	6	0.106629
rs17179001	PKNOX1	21	43324126	3	0.855308
rs234736	PKNOX1	21	43324981	8	3.524007
rs234736	PKNOX1	21	43324981	5	0.761018
rs234736	PKNOX1	21	43324981	2	4.036571
rs2839628	PKNOX1	21	43325937	8	0.030293
rs2839628	PKNOX1	21	43325937	6	6.98023
rs2839628	PKNOX1	21	43325937	3	0.521814
rs234740	PKNOX1	21	43326017	5	0.196406
rs234740	PKNOX1	21	43326017	2	4.072018
rs1801181	CBS	21	43353685	6	0.215847
rs1801181	CBS	21	43353685	3	0.092468
rs1801181	CBS	21	43353685	1	2.183872

rs564779	FLJ41733	21	43609850	2	0.192807
rs564779	FLJ41733	21	43609850	5	0.411437
rs28510483	SNF1LK	21	43663336	2	3.249763
rs11088981	C21orf125	21	43694578	2	0.724234
rs2838304	C21orf125	21	43697445	3	1.031845
rs2838304	C21orf125	21	43697445	7	0.431758
rs2838304	C21orf125	21	43697445	5	0.670729
rs2838343	HSF2BP	21	43902463	2	1.247691
rs2838343	HSF2BP	21	43902463	5	1.056612
rs2838343	HSF2BP	21	43902463	1	1.675488
rs2838343	HSF2BP	21	43902463	6	1.374373
rs3204323	PDXK	21	44005136	4	3.465141
rs3194230	PDXK	21	44005617	4	0.729936
rs4566449	RRP1	21	44043952	8	4.332622
rs4566449	RRP1	21	44043952	5	0.56321
rs4566449	RRP1	21	44043952	1	1.067612
rs4566449	RRP1	21	44043952	4	0.190629
rs4566449	RRP1	21	44043952	2	0.667285
rs13847	AGPAT3	21	44227248	1	5.438264
rs2838476	TMEM1	21	44304140	4	0.247055
rs2187313	PWP2	21	44369022	4	5.735649
rs2838497	C21orf33	21	44389194	4	0.476312
rs4819388	ICOSLG	21	44471849	2	1.443518
rs4819388	ICOSLG	21	44471849	6	3.813384
rs4819388	ICOSLG	21	44471849	1	0.189151
rs15927	ICOSLG	21	44472074	7	0.460608
rs15927	ICOSLG	21	44472074	1	4.671486
SNP21-4448120	ICOSLG	21	44481202	1	4.217488
SNP21-4448120	ICOSLG	21	44481202	6	4.264737
SNP21-4448120	ICOSLG	21	44481202	4	0.639944
rs1055311	AIRE	21	44533996	3	0.705167
rs1055311	AIRE	21	44533996	2	0.131515
rs1800522	AIRE	21	44541978	1	1.25634
rs1800522	AIRE	21	44541978	3	1.098836
rs1800522	AIRE	21	44541978	5	0.55664
rs1057037	PFKL	21	44558295	4	3.996035
rs1057037	PFKL	21	44558295	1	4.540667
rs1556314	TRPM2	21	44635771	7	0.987044
rs1556314	TRPM2	21	44635771	5	0.319185
SNP21-4464462	TRPM2	21	44644624	3	0.114784
rs3804034	LRRC3	21	44703065	6	2.953092
rs3804034	LRRC3	21	44703065	1	0.471262
rs3804034	LRRC3	21	44703065	3	0.560562
rs3804034	LRRC3	21	44703065	5	0.436065
rs12483730	KRTAP12-2	21	44910885	3	1.780036
rs12483730	KRTAP12-2	21	44910885	1	2.447822
rs235314	PTTG1IP	21	45095880	2	6.524337

rs235373	PTTG1IP	21	45118120	7	0.577884
rs5030671	ITGB2	21	45137870	1	4.365287
rs11088969	ITGB2	21	45155102	8	3.224064
rs11088969	ITGB2	21	45155102	7	5.317015
rs2070947	ITGB2	21	45165271	5	0.297784
rs2070947	ITGB2	21	45165271	3	2.798037
rs2070947	ITGB2	21	45165271	8	0.542921
rs13048147	LOC728004	21	45241889	5	0.676723
rs13048147	LOC728004	21	45241889	7	1.613577
rs2255774	POFUT2	21	45508279	5	0.659352
rs2255774	POFUT2	21	45508279	3	0.881908
rs2255774	POFUT2	21	45508279	1	4.233137
rs2256000	POFUT2	21	45510337	5	0.183503
rs2256000	POFUT2	21	45510337	1	2.946824
rs6518220	POFUT2	21	45510922	6	0.33749
rs6518220	POFUT2	21	45510922	1	4.197015
rs6518220	POFUT2	21	45510922	5	0.355218
rs6518220	POFUT2	21	45510922	7	0.773498
rs6518220	POFUT2	21	45510922	4	0.256982
rs2838861	POFUT2	21	45511047	5	0.536416
rs2838861	POFUT2	21	45511047	1	5.876377
rs2838862	POFUT2	21	45511154	5	4.158053
rs2838862	POFUT2	21	45511154	1	4.119353
rs2297285	POFUT2	21	45530049	1	0.386999
rs2297285	POFUT2	21	45530049	5	5.548378
rs2297285	POFUT2	21	45530049	3	0.575296
rs6518223	C21orf86	21	45538977	1	2.616377
rs7282557	C21orf86	21	45539014	7	1.854304
rs7282557	C21orf86	21	45539014	3	0.710054
rs11701479	C21orf123	21	45665756	5	0.815921
rs11701479	C21orf123	21	45665756	8	0.267613
rs2236451	COL18A1	21	45700511	3	0.357329
rs2236451	COL18A1	21	45700511	5	0.15311
rs2230688	COL18A1	21	45720740	3	0.380753
rs11702425	COL18A1	21	45732783	5	0.952612
rs11702425	COL18A1	21	45732783	2	1.47516
rs11702425	COL18A1	21	45732783	8	1.383011
rs11702425	COL18A1	21	45732783	6	1.398264
SNP21-4573463	COL18A1	21	45734638	6	1.871785
rs12483761	COL18A1	21	45751917	5	0.487495
rs12483761	COL18A1	21	45751917	8	0.068643
rs12483761	COL18A1	21	45751917	6	0.025635
rs2838952	COL18A1	21	45754591	7	0.180822
rs12659	SLC19A1	21	45775984	2	3.476315
rs12659	SLC19A1	21	45775984	4	3.014532
rs1051266	SLC19A1	21	45782222	4	4.683443
rs11089032	PCBP3	21	46186498	3	0.306321

rs11089032	PCBP3	21	46186498	4	1.399494
rs11089032	PCBP3	21	46186498	5	2.015105
rs11089032	PCBP3	21	46186498	2	5.128708
rs438651	PCBP3	21	46186652	7	0.57961
rs438651	PCBP3	21	46186652	4	1.372394
rs438651	PCBP3	21	46186652	2	3.409871
rs1980982	COL6A1	21	46235359	7	0.686637
rs1980982	COL6A1	21	46235359	8	0.436643
rs1980982	COL6A1	21	46235359	1	1.68571
rs1980982	COL6A1	21	46235359	6	0.137187
SNP21-46356884	COL6A2	21	46356884	4	0.251964
rs2839110	COL6A2	21	46363388	3	0.139858
rs2839110	COL6A2	21	46363388	1	0.087152
rs1044598	COL6A2	21	46374057	5	7.174942
rs1044598	COL6A2	21	46374057	4	5.16249
rs1044598	COL6A2	21	46374057	1	0.093093
rs1044598	COL6A2	21	46374057	6	0.7931
rs1044598	COL6A2	21	46374057	3	0.177005
rs1044598	COL6A2	21	46374057	2	4.101614
rs6652	COL6A2	21	46376813	5	0.329746
rs2839173	MCM3AP	21	46501139	7	1.180873
rs2839173	MCM3AP	21	46501139	3	1.555114
rs2839173	MCM3AP	21	46501139	1	1.093571
rs2839173	MCM3AP	21	46501139	2	0.971664
rs2839173	MCM3AP	21	46501139	5	1.012678
rs2839173	MCM3AP	21	46501139	8	0.212774
rs2839173	MCM3AP	21	46501139	6	1.206703
rs2839173	MCM3AP	21	46501139	4	1.231151
rs11702450	MCM3AP	21	46528077	3	0.842224
rs11702450	MCM3AP	21	46528077	6	2.933093
rs11702450	MCM3AP	21	46528077	8	0.273931
rs11702450	MCM3AP	21	46528077	5	1.552064
rs8651	C21orf57	21	46542044	4	1.878477
rs8651	C21orf57	21	46542044	1	3.135845
rs3747007	C21orf58	21	46546167	4	0.336418
rs3747007	C21orf58	21	46546167	6	0.003492
rs3747007	C21orf58	21	46546167	1	3.289102
SNP21-46559087	C21orf58	21	46559087	6	0.741576
SNP21-46559087	C21orf58	21	46559087	1	0.127133
SNP21-46559087	C21orf58	21	46559087	5	7.19026
SNP21-46559087	C21orf58	21	46559087	3	0.457644
SNP21-46559087	C21orf58	21	46559087	7	0.4145
SNP21-46559087	C21orf58	21	46559087	4	6.727478
SNP21-46559087	C21orf58	21	46559087	2	0.32146
rs2249060	PCNT	21	46597605	1	6.246057
SNP21-46601491	PCNT	21	46601491	1	4.492834
rs2839227	PCNT	21	46610952	1	2.543272

rs2839228	PCNT	21	46611245	1	6.792461
SNP21-4661143(	PCNT	21	46611430	1	6.404511
SNP21-4663310	PCNT	21	46633107	1	4.517654
rs3737438	PCNT	21	46635700	7	1.043613
rs3737438	PCNT	21	46635700	5	0.244535
rs3737438	PCNT	21	46635700	3	2.297959
SNP21-4665593	PCNT	21	46655937	2	3.82375
SNP21-4666063	PCNT	21	46660634	4	0.76465
SNP21-4666063	PCNT	21	46660634	3	0.018976
SNP21-4666063	PCNT	21	46660634	2	0.229044
SNP21-4666063	PCNT	21	46660634	5	6.351503
SNP21-4666063	PCNT	21	46660634	7	4.981382
SNP21-4666063	PCNT	21	46660634	1	1.266436
SNP21-4666082	PCNT	21	46660823	2	4.468485
SNP21-4666097	PCNT	21	46660975	2	0.185134
SNP21-4666108	PCNT	21	46661081	6	0.153233
SNP21-4666108	PCNT	21	46661081	5	0.762455
SNP21-4666108	PCNT	21	46661081	7	0.666536
SNP21-4666108	PCNT	21	46661081	3	0.510273
rs2839256	PCNT	21	46672887	2	3.428488
rs2070426	PCNT	21	46674912	3	0.703104
rs7279002	DIP2A	21	46734951	7	0.487342
rs7279002	DIP2A	21	46734951	3	0.609998
rs7279002	DIP2A	21	46734951	5	0.334207
rs7283507	DIP2A	21	46743090	6	0.852623
rs4818845	DIP2A	21	46790444	6	0.042272
rs2248636	DIP2A	21	46810083	3	0.456485
rs9722	S100B	21	46843667	3	0.521014
rs9722	S100B	21	46843667	5	0.527051
rs2277829	PRMT2	21	46880030	3	0.873226
rs9605145	XKR3	22	15644904	6	0.254721
rs9605145	XKR3	22	15644904	7	1.544321
rs9605146	XKR3	22	15645194	6	0.768377
rs7287116	GAB4	22	15823131	5	2.047658
rs1024732	GAB4	22	15823381	3	0.299434
rs917864	IL17RA	22	15945932	4	2.964171
rs917864	IL17RA	22	15945932	6	6.560655
rs917864	IL17RA	22	15945932	5	0.013859
rs879577	IL17RA	22	15969209	4	0.246643
rs879577	IL17RA	22	15969209	1	0.618226
rs879575	IL17RA	22	15969567	1	3.439289
rs9605220	CECR6	22	15980148	5	0.938407
rs9605220	CECR6	22	15980148	1	2.2561
rs35665085	CECR5	22	16005915	2	4.308209
rs7285896	CECR1	22	16041791	2	2.486618
rs2231495	CECR1	22	16049306	3	0.249202
rs2231495	CECR1	22	16049306	1	4.930678

rs9180	SLC25A18	22	16453568	2	1.55108
rs9180	SLC25A18	22	16453568	1	1.987753
rs1044497	SLC25A18	22	16453592	7	0.630464
rs1044497	SLC25A18	22	16453592	5	8.542692
rs7414	ATP6V1E1	22	16455053	7	0.225129
rs5992807	BCL2L13	22	16590822	7	6.627595
rs1045588	MICAL3	22	16651078	8	6.65441
rs9617630	LOC729269	22	16734766	8	0.8058
rs12484657	PEX26	22	16946288	5	0.851526
SNP22-16951008	PEX26	22	16951008	6	6.858168
SNP22-16951008	PEX26	22	16951008	5	0.444264
SNP22-16951008	PEX26	22	16951008	4	0.315232
SNP22-16951008	PEX26	22	16951008	1	0.121046
SNP22-16951008	PEX26	22	16951008	2	2.178226
rs362203	TUBA8	22	16989642	4	4.561559
rs4819756	PRODH	22	17292678	3	2.66157
rs4819756	PRODH	22	17292678	4	3.639536
rs4819756	PRODH	22	17292678	5	2.886141
rs2008720	PRODH	22	17303745	3	0.581517
rs1052756	NULL	22	17499686	5	4.054991
rs2240111	DGCR14	22	17502665	5	5.139634
rs8141941	SLC25A1	22	17546263	2	4.059361
rs8141941	SLC25A1	22	17546263	4	2.22045
rs8141941	SLC25A1	22	17546263	5	0.895868
rs1061325	CLTCL1	22	17564095	6	6.104856
rs712952	CLTCL1	22	17577949	5	1.750681
rs712952	CLTCL1	22	17577949	4	6.244509
rs712952	CLTCL1	22	17577949	6	0.334814
rs1210690	HIRA	22	17698622	7	0.190331
rs2073730	UFD1L	22	17817946	6	5.505392
rs2073730	UFD1L	22	17817946	1	5.918333
rs2073730	UFD1L	22	17817946	7	0.452052
rs2073730	UFD1L	22	17817946	3	0.345503
rs2073730	UFD1L	22	17817946	5	0.119056
rs4141527	CDC45L	22	17847717	5	0.10192
rs885985	CLDN5	22	17891925	3	0.149196
rs41298814	TBX1	22	18130773	6	1.106582
rs41298814	TBX1	22	18130773	1	1.151196
rs41298814	TBX1	22	18130773	7	0.077416
rs2301558	TBX1	22	18131829	1	0.205086
rs2301558	TBX1	22	18131829	7	0.982076
rs2301558	TBX1	22	18131829	4	2.736495
rs2301558	TBX1	22	18131829	3	0.002413
rs4819522	TBX1	22	18146782	3	1.090834
rs5746826	TBX1	22	18147051	3	0.536165
rs2073770	GNB1L	22	18169541	6	0.195046
rs2073770	GNB1L	22	18169541	1	3.35254

rs5748449	GNB1L	22	18188769	1	4.184691
rs5748449	GNB1L	22	18188769	8	0.220362
rs5748449	GNB1L	22	18188769	3	0.126641
rs13164	C22orf29	22	18213926	5	0.160177
rs13164	C22orf29	22	18213926	3	0.032677
rs13164	C22orf29	22	18213926	1	5.413935
rs13164	C22orf29	22	18213926	6	0.510714
rs13057910	C22orf29	22	18222285	2	4.156151
rs1139795	TXNRD2	22	18247771	2	3.567594
rs11541479	TXNRD2	22	18286511	3	0.882997
rs5748470	TXNRD2	22	18287118	5	0.967155
rs5748470	TXNRD2	22	18287118	3	1.391452
rs4680	COMT	22	18331271	6	0.181062
rs4680	COMT	22	18331271	5	0.419797
rs4680	COMT	22	18331271	7	1.301701
rs165815	ARVCF	22	18339473	5	0.326621
rs2073747	ARVCF	22	18349075	6	0.092871
rs2073747	ARVCF	22	18349075	1	1.484456
rs2073747	ARVCF	22	18349075	3	1.959325
rs2240717	ARVCF	22	18349106	7	1.437533
rs415520	C22orf25	22	18432532	7	2.24281
rs415520	C22orf25	22	18432532	6	5.964427
rs415520	C22orf25	22	18432532	8	7.636832
rs415520	C22orf25	22	18432532	3	0.071547
rs415520	C22orf25	22	18432532	1	5.334173
rs2286928	DGCR8	22	18478521	3	0.070287
rs8137258	LOC388849	22	18515961	3	0.567799
rs8137258	LOC388849	22	18515961	1	0.016021
rs8137258	LOC388849	22	18515961	7	0.816926
rs8137258	LOC388849	22	18515961	6	1.557831
rs1319199	SCARF2	22	19109563	5	0.055357
rs520698	LOC729495	22	19354880	7	1.12103
rs520698	LOC729495	22	19354880	3	1.500644
rs4675	SERPIND1	22	19471300	4	0.730158
rs4675	SERPIND1	22	19471300	3	0.554021
rs4675	SERPIND1	22	19471300	6	0.213701
rs737894	CRKL	22	19634408	4	0.284225
rs1548410	CRKL	22	19634463	4	6.247652
rs81027	LOC728351	22	19641531	4	0.104303
rs7292968	AIFM3	22	19665559	4	4.529129
rs7292968	AIFM3	22	19665559	8	5.229692
rs2075269	THAP7	22	19686029	4	2.124308
rs2075269	THAP7	22	19686029	2	2.441153
rs398577	MGC16703	22	19692849	3	0.691946
rs1548411	P2RXL1	22	19707301	5	0.034947
rs1548411	P2RXL1	22	19707301	7	1.092317
rs1548411	P2RXL1	22	19707301	3	0.958874

rs1548411	P2RXL1	22	19707301	1	1.040742
rs2541953	P2RXL1	22	19711646	1	0.722478
rs2541953	P2RXL1	22	19711646	7	1.46272
rs2541953	P2RXL1	22	19711646	3	0.53276
rs2541953	P2RXL1	22	19711646	2	0.478366
rs2541954	P2RXL1	22	19711682	3	0.234118
rs2541954	P2RXL1	22	19711682	7	1.286414
rs2541954	P2RXL1	22	19711682	1	0.65073
rs3179909	P2RXL1	22	19712167	3	0.371725
rs2072549	SLC7A4	22	19715843	4	0.367167
rs2072549	SLC7A4	22	19715843	1	3.227066
rs2072549	SLC7A4	22	19715843	5	1.174947
rs402931	SLC7A4	22	19715985	1	1.406689
rs402931	SLC7A4	22	19715985	7	0.771298
rs402931	SLC7A4	22	19715985	3	0.489093
rs2072551	SLC7A4	22	19716112	1	1.721478
rs2072551	SLC7A4	22	19716112	5	0.816704
rs464694	HIC2	22	20131489	5	0.686665
rs464694	HIC2	22	20131489	7	1.163216
rs464694	HIC2	22	20131489	3	1.080428
GA017812	UBE2L3	22	20307047	3	0.440356
GA017812	UBE2L3	22	20307047	4	0.857227
rs861854	CCDC116	22	20318524	6	2.077011
rs861854	CCDC116	22	20318524	7	0.604424
rs861854	CCDC116	22	20318524	1	1.475162
rs861854	CCDC116	22	20318524	5	1.140607
SNP22-2032107	CCDC116	22	20321072	6	1.207441
SNP22-2032107	CCDC116	22	20321072	1	2.350312
SNP22-2032107	CCDC116	22	20321072	7	0.777074
SNP22-2032107	CCDC116	22	20321072	5	1.049557
SNP22-2037978	PPIL2	22	20379783	4	1.685982
rs1045681	YPEL1	22	20382814	3	0.088193
rs1045681	YPEL1	22	20382814	1	2.058637
rs1045681	YPEL1	22	20382814	5	0.302435
rs1045681	YPEL1	22	20382814	6	0.623494
rs3747094	YPEL1	22	20383880	5	0.256345
rs3747094	YPEL1	22	20383880	6	4.282007
rs3747094	YPEL1	22	20383880	4	4.931351
rs3747094	YPEL1	22	20383880	3	0.429802
rs3747094	YPEL1	22	20383880	1	3.903618
rs1860	YPEL1	22	20385394	5	4.641898
rs1860	YPEL1	22	20385394	3	1.57639
rs1860	YPEL1	22	20385394	1	4.875971
rs1860	YPEL1	22	20385394	6	0.94086
rs1860	YPEL1	22	20385394	4	4.986163
rs6928	MAPK1	22	20445004	3	0.04113
rs6928	MAPK1	22	20445004	7	1.020009

rs2070506	PPM1F	22	20607307	1	4.22197
rs9610728	TOP3B	22	20648538	7	1.247528
rs239927	TOP3B	22	20660082	3	0.362478
rs1805058	GNAZ	22	21768191	1	2.727147
rs1051875	GNAZ	22	21796031	5	2.210798
rs1051875	GNAZ	22	21796031	3	1.179802
rs4822360	RTDR1	22	21812460	5	0.959237
rs4822360	RTDR1	22	21812460	3	1.949912
SNP22-2183317(	RAB36	22	21833170	1	4.22116
SNP22-2183317(	RAB36	22	21833170	6	0.753044
rs737820	RAB36	22	21833870	1	5.574878
rs2008176	RAB36	22	21835398	1	1.825625
rs2008176	RAB36	22	21835398	6	0.38118
rs2003752	FLJ31568	22	22072105	8	0.893753
rs2003752	FLJ31568	22	22072105	1	0.069359
rs2003752	FLJ31568	22	22072105	2	3.375995
rs5751713	Rgr	22	22363466	8	6.563554
rs738786	Rgr	22	22364288	8	3.48186
rs738786	Rgr	22	22364288	2	3.238479
rs738786	Rgr	22	22364288	6	0.380763
rs738786	Rgr	22	22364288	5	3.17959
rs2070446	Rgr	22	22365970	8	1.648138
rs2070446	Rgr	22	22365970	2	4.153563
rs2070446	Rgr	22	22365970	5	0.056848
rs1007298	Rgr	22	22368847	8	2.074371
rs1007298	Rgr	22	22368847	2	3.262731
rs1007298	Rgr	22	22368847	5	0.222148
rs17630135	ZNF70	22	22415252	6	0.563342
rs2298375	C22orf15	22	22436448	8	0.098941
rs140181	C22orf15	22	22436674	1	1.27453
rs140181	C22orf15	22	22436674	5	0.021351
rs179468	C22orf16	22	22439774	2	0.244869
rs179468	C22orf16	22	22439774	3	0.318501
rs179468	C22orf16	22	22439774	1	0.191895
rs179468	C22orf16	22	22439774	4	0.799914
rs5760061	DERL3	22	22508279	7	0.938193
rs5760061	DERL3	22	22508279	3	0.995011
rs5760062	DERL3	22	22508590	7	0.46184
rs5760062	DERL3	22	22508590	3	0.729519
rs1128127	DERL3	22	22509132	8	0.10303
rs16986337	SLC2A11	22	22529704	7	2.076299
rs16986337	SLC2A11	22	22529704	5	0.473161
SNP22-2255689(	SLC2A11	22	22556890	7	0.566031
rs1622002	GSTT2B	22	22630580	2	1.763664
rs1622002	GSTT2B	22	22630580	4	0.647342
rs7288201	GGTLA1	22	22951293	4	0.482008
rs2275984	GGTLA1	22	22952648	1	1.007722

rs2275984	GGTLA1	22	22952648	8	0.702051
rs2275984	GGTLA1	22	22952648	2	1.369244
SNP22-22970666	GGT5	22	22970666	5	0.999931
SNP22-22970666	GGT5	22	22970666	3	0.085386
SNP22-22970666	GGT5	22	22970666	1	0.764475
SNP22-22970666	GGT5	22	22970666	8	1.928098
rs3747113	SPECC1L	22	23047518	1	5.747331
rs3747114	SPECC1L	22	23141667	5	6.786511
rs3747114	SPECC1L	22	23141667	3	2.042505
rs3747114	SPECC1L	22	23141667	7	5.036058
rs3747114	SPECC1L	22	23141667	6	0.113834
rs7678	SPECC1L	22	23143165	7	0.08193
rs3176991	SNRPD3	22	23294128	2	3.774524
rs3176991	SNRPD3	22	23294128	7	0.907179
rs11703684	PIWIL3	22	23474912	7	2.400704
rs11703684	PIWIL3	22	23474912	5	2.468797
rs9624586	TOP1P2	22	23490785	1	3.125689
rs9624586	TOP1P2	22	23490785	7	1.476117
rs5760637	PIWIL3	22	23500297	3	0.28954
rs9624633	SGSM1	22	23600455	4	0.752592
rs9624633	SGSM1	22	23600455	5	0.360047
rs5760738	SGSM1	22	23650371	4	0.523002
rs5760738	SGSM1	22	23650371	2	1.519472
rs5760738	SGSM1	22	23650371	1	1.223836
rs5760738	SGSM1	22	23650371	7	0.200699
rs5996792	SGSM1	22	23650738	7	0.16705
rs5996792	SGSM1	22	23650738	1	1.32413
rs5996792	SGSM1	22	23650738	2	2.010172
rs5996792	SGSM1	22	23650738	4	2.998389
rs5996793	SGSM1	22	23650814	4	5.573857
rs5996793	SGSM1	22	23650814	1	1.080309
rs5996793	SGSM1	22	23650814	7	0.241699
rs5760739	SGSM1	22	23651787	1	0.892087
rs5760739	SGSM1	22	23651787	4	0.394441
rs5760739	SGSM1	22	23651787	2	1.341635
rs5760739	SGSM1	22	23651787	7	0.910338
rs13054858	NULL	22	23754579	3	0.552387
rs12168578	NULL	22	23903431	5	1.683644
rs133201	LRP5L	22	24087268	1	3.298269
SNP22-24487068	MYO18B	22	24487068	3	1.466982
rs13058434	MYO18B	22	24494413	2	2.170836
rs738643	MYO18B	22	24524045	6	0.760246
rs17704912	MYO18B	22	24561312	3	0.415261
rs17704912	MYO18B	22	24561312	7	0.508512
rs5761268	MYO18B	22	24569850	7	0.419651
rs2236005	MYO18B	22	24752980	8	0.832305
rs2014410	HPS4	22	25190269	5	0.10924

rs2014410	HPS4	22	25190269	7	0.582441
rs2014410	HPS4	22	25190269	6	0.428372
rs9613187	HPS4	22	25205370	3	1.224769
rs9613187	HPS4	22	25205370	5	1.925183
rs9613187	HPS4	22	25205370	6	0.829276
rs1045523	SRRD	22	25217829	4	2.924552
rs3213558	TFIP11	22	25238433	8	1.069436
rs3213558	TFIP11	22	25238433	5	0.273025
rs3213558	TFIP11	22	25238433	6	0.120484
rs3213558	TFIP11	22	25238433	1	5.175751
rs17476776	MN1	22	26475605	5	0.43082
rs1062731	PITPNB	22	26578840	6	6.057669
rs470098	TTC28	22	26707083	3	0.655864
rs470101	TTC28	22	26719453	3	0.45018
rs9625408	TTC28	22	26834183	3	1.341038
SNP22-27512169	CCDC117	22	27512169	3	0.547285
rs12321	ZNRF3	22	27783193	2	0.039709
rs12321	ZNRF3	22	27783193	8	0.019664
rs132280	KREMEN1	22	27870920	3	0.965475
rs132280	KREMEN1	22	27870920	8	0.82266
rs465736	RFPL1	22	28164766	2	1.133009
rs465736	RFPL1	22	28164766	1	1.228814
rs465736	RFPL1	22	28164766	3	0.46232
rs16987627	RFPL1	22	28165060	4	0.440103
rs16987627	RFPL1	22	28165060	8	0.274587
rs5763240	RFPL1S	22	28166926	1	1.550083
rs5763240	RFPL1S	22	28166926	4	0.826787
rs5763240	RFPL1S	22	28166926	3	0.397672
rs3804076	RFPL1	22	28167537	6	8.632973
rs3804076	RFPL1	22	28167537	7	0.316715
rs3804076	RFPL1	22	28167537	3	0.631663
rs3804076	RFPL1	22	28167537	1	4.26742
rs3804076	RFPL1	22	28167537	5	0.405072
rs3804076	RFPL1	22	28167537	8	1.381248
rs1049534	THOC5	22	28238072	7	0.171628
rs737976	THOC5	22	28243272	4	0.040586
rs737976	THOC5	22	28243272	5	0.470138
rs737976	THOC5	22	28243272	7	0.369701
rs469189	THOC5	22	28279509	1	0.769102
rs469189	THOC5	22	28279509	2	0.568916
rs469189	THOC5	22	28279509	4	4.10886
rs8140096	NF2	22	28421765	2	3.035624
rs2530680	NF2	22	28421999	3	0.122954
rs5997507	CABP7	22	28455596	5	1.692595
rs28265	ASCC2	22	28530761	8	4.91979
rs28265	ASCC2	22	28530761	3	0.06011
rs11549795	ASCC2	22	28551120	3	0.572889

rs41173	NULL	22	28754863	1	1.056392
rs41173	NULL	22	28754863	2	1.010924
rs737812	LIF	22	28969110	6	0.443961
rs737812	LIF	22	28969110	5	0.362811
rs4823086	TBC1D10A	22	29018659	3	0.744876
rs10376	SF3A1	22	29058356	2	5.914762
rs740222	LOC550631	22	29091940	1	1.200393
rs740222	LOC550631	22	29091940	5	0.545219
rs740222	LOC550631	22	29091940	3	0.261011
rs740223	LOC550631	22	29092140	1	5.648201
rs740223	LOC550631	22	29092140	3	0.498009
rs740223	LOC550631	22	29092140	5	0.207976
SNP22-29101554	LOC550631	22	29101554	2	4.909579
rs2286442	LOC550631	22	29102686	1	4.405575
rs2286442	LOC550631	22	29102686	4	0.572841
rs2286442	LOC550631	22	29102686	7	0.726585
rs2286442	LOC550631	22	29102686	5	0.843287
rs5997629	RNF215	22	29112089	1	2.336077
rs5997629	RNF215	22	29112089	7	0.302419
rs757660	SEC14L2	22	29123137	8	0.610008
rs757660	SEC14L2	22	29123137	1	4.039234
rs757660	SEC14L2	22	29123137	5	0.068921
rs757660	SEC14L2	22	29123137	7	0.266928
rs9606726	MTP18	22	29153250	3	0.479618
rs4820853	SEC14L3	22	29194610	7	2.028617
rs4820853	SEC14L3	22	29194610	4	1.367941
rs4820853	SEC14L3	22	29194610	3	1.350164
rs4820853	SEC14L3	22	29194610	5	1.860511
rs4820853	SEC14L3	22	29194610	1	2.171484
rs4820853	SEC14L3	22	29194610	2	2.328941
rs4820853	SEC14L3	22	29194610	8	3.009247
rs13750	SEC14L4	22	29215100	6	0.285402
rs17670808	SEC14L4	22	29215980	6	1.607775
SNP22-29218494	SEC14L4	22	29218494	6	0.501782
SNP22-29218494	SEC14L4	22	29218494	5	0.264532
rs9608956	SEC14L4	22	29231592	5	0.802618
rs5753190	LOC730005	22	29264816	7	0.884425
rs2267161	GAL3ST1	22	29283295	3	1.425606
rs2267161	GAL3ST1	22	29283295	5	3.405359
rs737953	PES1	22	29317861	6	1.576175
rs737953	PES1	22	29317861	2	1.894368
rs737953	PES1	22	29317861	3	0.596455
rs737953	PES1	22	29317861	7	0.662792
rs737953	PES1	22	29317861	8	0.254545
rs737953	PES1	22	29317861	1	0.630161
rs9606756	TCN2	22	29336860	4	0.961182
rs9606756	TCN2	22	29336860	1	5.515628

rs9606756	TCN2	22	29336860	3	1.041896
rs1131603	TCN2	22	29348975	1	4.117716
rs4820889	TCN2	22	29349044	4	0.272263
rs9609038	DUSP18	22	29388394	1	5.590133
rs1005887	DUSP18	22	29390029	1	3.865789
rs1005887	DUSP18	22	29390029	6	0.689506
rs1005887	DUSP18	22	29390029	4	6.574466
rs1005887	DUSP18	22	29390029	7	0.417188
rs1005887	DUSP18	22	29390029	3	0.176851
rs11912792	DUSP18	22	29393832	6	1.748481
rs11912792	DUSP18	22	29393832	1	5.249454
rs2072132	MORC2	22	29672376	2	2.397964
rs2072132	MORC2	22	29672376	4	0.193275
rs2072132	MORC2	22	29672376	6	4.750069
rs2072132	MORC2	22	29672376	7	0.409374
rs737924	MORC2	22	29684653	2	1.947495
rs737924	MORC2	22	29684653	4	2.290255
rs2240431	PIB5PA	22	29851850	4	0.655483
rs2240431	PIB5PA	22	29851850	5	0.013519
rs2240431	PIB5PA	22	29851850	1	3.529498
rs757873	PLA2G3	22	29860957	1	3.49578
rs757873	PLA2G3	22	29860957	4	1.214841
rs757873	PLA2G3	22	29860957	5	0.207475
rs757873	PLA2G3	22	29860957	7	0.725434
rs740232	PLA2G3	22	29861133	5	0.501848
rs740232	PLA2G3	22	29861133	3	0.216716
rs740232	PLA2G3	22	29861133	6	5.512427
rs740232	PLA2G3	22	29861133	2	3.342998
rs740231	PLA2G3	22	29861150	2	4.700364
rs740231	PLA2G3	22	29861150	7	0.434286
rs740231	PLA2G3	22	29861150	6	2.302272
rs2232183	PLA2G3	22	29862960	3	0.936143
rs2232183	PLA2G3	22	29862960	1	0.531874
rs2232183	PLA2G3	22	29862960	4	0.514958
rs2074735	PLA2G3	22	29865872	2	2.419485
rs2232176	PLA2G3	22	29866133	4	0.386003
rs2232176	PLA2G3	22	29866133	3	0.167088
rs5997988	EIF4ENIF1	22	30168085	6	3.127332
rs5997988	EIF4ENIF1	22	30168085	2	0.927053
rs5997988	EIF4ENIF1	22	30168085	3	0.386727
rs5997988	EIF4ENIF1	22	30168085	1	0.398486
rs5997988	EIF4ENIF1	22	30168085	4	3.640283
rs12171042	SFI1	22	30341225	1	3.721607
rs5749334	DEPDC5	22	30541412	3	0.484843
rs5749334	DEPDC5	22	30541412	4	0.494605
rs5749334	DEPDC5	22	30541412	5	0.557844
rs5749334	DEPDC5	22	30541412	1	2.814968

rs17683011	SLC5A1	22	30775946	1	0.66529
rs17683430	SLC5A1	22	30817700	1	4.161695
rs17683448	SLC5A1	22	30817744	1	5.202789
rs136466	RFPL2	22	30916632	1	0.397395
rs136466	RFPL2	22	30916632	5	1.015402
rs136478	RFPL2	22	30919023	5	0.183503
rs136478	RFPL2	22	30919023	2	1.590304
rs136478	RFPL2	22	30919023	1	0.599426
SNP22-31086703	RFPL3	22	31086703	5	2.841296
SNP22-31086703	RFPL3	22	31086703	1	2.570999
rs5749409	RFPL3	22	31086899	1	2.274584
rs8136485	FBXO7	22	31201227	1	0.715577
rs8136485	FBXO7	22	31201227	5	0.811596
rs9621461	FBXO7	22	31201365	7	0.815837
rs9621461	FBXO7	22	31201365	6	0.771558
rs9621461	FBXO7	22	31201365	2	0.939383
rs8137714	FBXO7	22	31201383	5	0.323504
rs8137714	FBXO7	22	31201383	1	2.761585
rs8137714	FBXO7	22	31201383	4	0.581291
rs11912802	SYN3	22	31239251	8	8.392834
rs16992034	LARGE	22	31999492	5	0.133987
rs17722172	LARGE	22	32000584	5	0.706406
rs16992986	LARGE	22	32487526	3	1.446968
rs361668	ISX	22	33793179	1	2.858563
rs361668	ISX	22	33793179	5	2.198577
rs361668	ISX	22	33793179	8	3.815051
rs361668	ISX	22	33793179	3	2.136755
rs3810619	ISX	22	33811776	1	1.355033
rs2071747	HMOX1	22	34107185	4	0.142945
rs133427	MCM5	22	34145880	7	7.896644
rs5999860	LOC730090	22	34252550	7	0.963501
rs5999860	LOC730090	22	34252550	1	2.176529
rs5999860	LOC730090	22	34252550	5	0.654974
rs3747159	RASD2	22	34278501	7	0.893822
rs3747159	RASD2	22	34278501	2	0.293273
rs3747159	RASD2	22	34278501	1	0.297794
rs1476000	LOC284912	22	34353386	2	1.658749
rs1476000	LOC284912	22	34353386	1	1.38948
rs2076672	APOL5	22	34453029	3	0.11605
rs2076672	APOL5	22	34453029	5	0.33802
rs2076672	APOL5	22	34453029	7	0.29477
rs2076673	APOL5	22	34454806	5	0.853578
rs2076673	APOL5	22	34454806	4	0.442106
rs2076673	APOL5	22	34454806	6	0.26572
rs916334	APOL3	22	34886910	1	0.903619
rs916334	APOL3	22	34886910	2	0.314611
rs916334	APOL3	22	34886910	3	0.045028

rs132655	APOL3	22	34887496	3	1.126864
rs6000172	APOL4	22	34917148	3	0.703545
rs6000173	APOL4	22	34917169	3	0.455651
rs6000173	APOL4	22	34917169	2	1.075416
rs6000174	APOL4	22	34917225	3	1.849203
rs6000174	APOL4	22	34917225	2	3.713396
rs6000174	APOL4	22	34917225	7	2.285072
rs2227167	APOL4	22	34917432	3	2.270847
rs2227168	APOL4	22	34917457	3	0.811819
rs132700	APOL4	22	34917650	3	0.35464
rs132700	APOL4	22	34917650	1	0.222027
rs132700	APOL4	22	34917650	2	0.254486
rs132700	APOL4	22	34917650	7	1.279036
rs2227169	APOL4	22	34917898	3	0.249569
rs2007468	APOL4	22	34921326	4	2.584863
rs2007468	APOL4	22	34921326	3	2.93756
rs2007468	APOL4	22	34921326	1	3.808363
rs2007468	APOL4	22	34921326	2	3.373975
rs2007468	APOL4	22	34921326	7	3.430518
rs132735	APOL4	22	34927827	4	0.07349
rs132735	APOL4	22	34927827	2	0.14342
rs9619597	APOL2	22	34952768	1	4.760845
rs7285167	APOL2	22	34953866	1	5.54157
rs2010499	APOL2	22	34959412	7	1.397509
rs2010499	APOL2	22	34959412	2	3.730565
rs2010499	APOL2	22	34959412	3	0.371236
rs2010499	APOL2	22	34959412	8	7.998944
rs6000220	APOL1	22	34979912	7	1.02709
SNP22-34991276	APOL1	22	34991276	4	5.366132
SNP22-34991276	APOL1	22	34991276	3	8.758528
SNP22-34991276	APOL1	22	34991276	8	0.309333
rs139999	TXN2	22	35193321	3	0.295687
rs3747163	FOXRED2	22	35214972	3	0.251848
rs3747163	FOXRED2	22	35214972	8	7.063271
rs9622410	EIF3D	22	35251998	8	0.836807
rs131837	FLJ90680	22	35572291	4	2.461735
rs131837	FLJ90680	22	35572291	2	1.585948
rs131837	FLJ90680	22	35572291	6	1.547326
rs5756370	FLJ90680	22	35572422	7	0.226097
rs5756370	FLJ90680	22	35572422	1	0.317533
rs5756370	FLJ90680	22	35572422	5	1.222853
rs131840	CSF2RB	22	35663740	7	0.15626
SNP22-35728147	C22orf33	22	35728147	5	0.355907
rs2235321	TMPRSS6	22	35792872	7	1.861189
rs2235321	TMPRSS6	22	35792872	2	5.234126
rs2235321	TMPRSS6	22	35792872	4	1.552634
rs2235321	TMPRSS6	22	35792872	8	1.773462

rs855791	TMPRSS6	22	35792882	7	0.858131
rs855791	TMPRSS6	22	35792882	4	0.725046
rs2235324	TMPRSS6	22	35815670	5	0.310523
rs2235324	TMPRSS6	22	35815670	8	3.184546
rs228942	IL2RB	22	35854565	7	1.108039
rs229517	C1QTNF6	22	35907683	7	0.135285
rs229526	C1QTNF6	22	35911368	6	1.845059
rs229526	C1QTNF6	22	35911368	1	5.354068
rs229526	C1QTNF6	22	35911368	8	6.503291
rs229526	C1QTNF6	22	35911368	7	4.823211
rs229527	C1QTNF6	22	35911431	6	0.201716
rs229527	C1QTNF6	22	35911431	1	3.917688
rs2071710	SSTR3	22	35932967	3	0.137433
rs86582	SSTR3	22	35933336	3	0.010729
rs86582	SSTR3	22	35933336	7	0.290272
rs86583	SSTR3	22	35933690	3	1.728727
rs9610669	SSTR3	22	35937927	2	2.914609
rs6572	RAC2	22	35951391	8	7.452381
rs6572	RAC2	22	35951391	3	0.103735
rs6572	RAC2	22	35951391	6	8.234414
rs6572	RAC2	22	35951391	4	0.039822
rs6572	RAC2	22	35951391	1	6.370974
rs9610680	RAC2	22	35951897	3	0.10883
rs9610680	RAC2	22	35951897	2	4.489462
rs2239774	RAC2	22	35967599	2	5.412861
rs2239774	RAC2	22	35967599	3	0.239815
rs2239774	RAC2	22	35967599	4	0.992476
rs3213557	PSCD4	22	36008511	5	1.587063
rs3213557	PSCD4	22	36008511	1	1.118057
rs3203726	PSCD4	22	36039959	7	1.181974
rs3203726	PSCD4	22	36039959	3	0.70007
rs3203726	PSCD4	22	36039959	6	0.795349
rs3203727	PSCD4	22	36040077	2	4.981956
rs3203728	PSCD4	22	36040123	6	0.558315
rs3203728	PSCD4	22	36040123	1	0.468504
rs3203728	PSCD4	22	36040123	7	1.109564
rs3203728	PSCD4	22	36040123	3	0.985661
rs5750409	PSCD4	22	36040241	4	1.446309
rs5750409	PSCD4	22	36040241	2	0.94707
rs5750409	PSCD4	22	36040241	6	5.326831
rs5750409	PSCD4	22	36040241	7	0.819383
rs5750409	PSCD4	22	36040241	1	1.593437
rs5750409	PSCD4	22	36040241	8	0.306033
rs5750409	PSCD4	22	36040241	3	1.255583
rs2284051	PSCD4	22	36040515	4	3.476804
rs2284051	PSCD4	22	36040515	7	1.167506
rs2284051	PSCD4	22	36040515	3	1.375382

rs2284051	PSCD4	22	36040515	6	6.32315
rs2284051	PSCD4	22	36040515	1	0.836661
rs2284051	PSCD4	22	36040515	8	0.111607
rs2284051	PSCD4	22	36040515	2	1.803276
rs15996	PSCD4	22	36041024	3	0.242426
rs15996	PSCD4	22	36041024	6	1.329826
rs15996	PSCD4	22	36041024	2	0.580229
rs15996	PSCD4	22	36041024	8	1.079283
rs15996	PSCD4	22	36041024	1	0.145204
rs15996	PSCD4	22	36041024	7	0.053163
rs15996	PSCD4	22	36041024	4	0.856311
rs1076934	ELFN2	22	36096142	7	0.626066
rs8142615	ELFN2	22	36100012	7	3.43466
rs8142615	ELFN2	22	36100012	8	3.560552
rs8142615	ELFN2	22	36100012	6	0.274942
rs2071856	ELFN2	22	36100576	3	2.354819
rs2071856	ELFN2	22	36100576	7	1.256957
rs2071856	ELFN2	22	36100576	5	1.108882
rs2071857	ELFN2	22	36101104	7	1.470528
rs2071857	ELFN2	22	36101104	3	0.438945
rs2071857	ELFN2	22	36101104	5	0.261266
rs4821653	NULL	22	36101931	7	1.622675
rs4821653	NULL	22	36101931	3	1.528207
rs3817805	CARD10	22	36230708	8	0.094173
rs3817805	CARD10	22	36230708	1	3.481556
SNP22-36376641	SH3BP1	22	36376641	8	1.188877
SNP22-36376641	SH3BP1	22	36376641	7	0.243308
SNP22-36381945	SH3BP1	22	36381945	4	9.482065
SNP22-36381945	SH3BP1	22	36381945	7	0.055902
rs9610841	TRIOBP	22	36451098	4	1.130537
rs9610841	TRIOBP	22	36451098	1	5.422587
rs7284476	TRIOBP	22	36459278	1	0.582689
rs8140207	TRIOBP	22	36460405	8	1.323381
rs8140207	TRIOBP	22	36460405	4	0.59959
rs8140207	TRIOBP	22	36460405	1	1.494126
rs3091367	ANKRD54	22	36557767	1	4.895157
rs3091367	ANKRD54	22	36557767	7	0.285663
rs3091367	ANKRD54	22	36557767	4	0.841962
rs2076371	SLC16A8	22	36804642	8	0.488346
rs2076371	SLC16A8	22	36804642	3	0.898405
rs2076371	SLC16A8	22	36804642	1	0.504754
rs2235573	SLC16A8	22	36807876	3	1.375684
rs2235573	SLC16A8	22	36807876	7	0.436661
rs2235573	SLC16A8	22	36807876	8	1.454865
rs2235573	SLC16A8	22	36807876	1	3.879891
rs11570768	PLA2G6	22	36837927	6	5.023196
rs11570597	PLA2G6	22	36907701	7	1.20681

rs2269608	KCNJ4	22	37152467	6	1.147159
rs2269608	KCNJ4	22	37152467	3	0.932594
rs2269608	KCNJ4	22	37152467	1	5.476683
rs196059	KCNJ4	22	37153082	3	3.123494
rs196059	KCNJ4	22	37153082	5	1.10687
rs2071887	KDELR3	22	37208956	7	0.558286
rs2071887	KDELR3	22	37208956	2	4.173231
rs2071887	KDELR3	22	37208956	3	0.123927
rs5757162	LOC646851	22	37323316	4	1.423037
rs5757162	LOC646851	22	37323316	2	4.218731
rs5757162	LOC646851	22	37323316	3	0.952319
rs5757162	LOC646851	22	37323316	7	1.529961
rs6001185	LOC646851	22	37382101	2	3.32735
rs6001185	LOC646851	22	37382101	3	0.557693
rs6001185	LOC646851	22	37382101	7	1.107373
rs5750669	TOMM22	22	37410584	3	0.526419
rs5750669	TOMM22	22	37410584	7	0.693129
rs5750669	TOMM22	22	37410584	2	5.391488
rs5750669	TOMM22	22	37410584	4	3.257219
SNP22-37477181	UNC84B	22	37477181	4	2.676047
rs7288826	NPTXR	22	37544720	2	0.260524
rs7288826	NPTXR	22	37544720	7	2.617211
rs7288826	NPTXR	22	37544720	1	0.036806
rs7288826	NPTXR	22	37544720	5	0.86195
rs2235135	NPTXR	22	37546613	1	0.49053
rs2235135	NPTXR	22	37546613	4	0.196402
rs8140217	NPTXR	22	37547947	1	0.883033
rs8140217	NPTXR	22	37547947	5	1.529969
rs8140217	NPTXR	22	37547947	7	2.137494
rs8140217	NPTXR	22	37547947	2	1.08158
rs5757299	NPTXR	22	37552598	5	1.030271
rs5757299	NPTXR	22	37552598	4	0.698471
rs5757299	NPTXR	22	37552598	6	5.417735
rs7584	APOBEC3C	22	37744645	3	0.137644
rs7584	APOBEC3C	22	37744645	6	0.433421
rs7584	APOBEC3C	22	37744645	7	0.890817
rs7584	APOBEC3C	22	37744645	5	6.908636
rs7584	APOBEC3C	22	37744645	1	4.861861
rs5750728	APOBEC3F	22	37770095	7	1.878556
rs5750728	APOBEC3F	22	37770095	8	4.277114
rs5750728	APOBEC3F	22	37770095	4	2.32985
rs139272	LOC651158	22	37817611	6	1.02475
rs139272	LOC651158	22	37817611	7	1.230045
rs139273	LOC651158	22	37817665	7	2.097912
rs139274	LOC651158	22	37817668	3	1.477823
rs139274	LOC651158	22	37817668	7	0.357167
rs139274	LOC651158	22	37817668	4	1.898367

rs139274	LOC651158	22	37817668	1	5.822972
rs139294	APOBEC3H	22	37826358	4	2.022005
rs139294	APOBEC3H	22	37826358	1	2.957488
rs139297	APOBEC3H	22	37827350	4	0.36092
rs139297	APOBEC3H	22	37827350	1	4.680776
rs139297	APOBEC3H	22	37827350	3	0.319049
rs139297	APOBEC3H	22	37827350	7	0.383953
rs139302	APOBEC3H	22	37827984	1	6.297764
rs139302	APOBEC3H	22	37827984	4	0.469255
rs139302	APOBEC3H	22	37827984	7	3.029705
rs139302	APOBEC3H	22	37827984	3	0.377073
rs139317	APOBEC3H	22	37829848	7	0.09797
rs139317	APOBEC3H	22	37829848	3	0.757981
rs139317	APOBEC3H	22	37829848	1	5.71652
rs139317	APOBEC3H	22	37829848	4	0.756139
rs710191	CBX7	22	37857760	3	0.705948
rs710191	CBX7	22	37857760	5	0.496272
rs710191	CBX7	22	37857760	4	0.124683
rs710191	CBX7	22	37857760	7	0.558915
rs139383	CBX7	22	37857855	4	0.179907
rs139383	CBX7	22	37857855	3	0.181278
rs139383	CBX7	22	37857855	5	0.346725
rs139384	CBX7	22	37857881	4	0.59759
rs139384	CBX7	22	37857881	6	0.30495
rs139384	CBX7	22	37857881	7	5.486482
rs139384	CBX7	22	37857881	5	0.664346
rs713841	CBX7	22	37858975	6	2.794394
rs713841	CBX7	22	37858975	4	1.083172
rs713841	CBX7	22	37858975	5	0.560615
rs11089928	CBX7	22	37859375	4	6.496216
rs1010170	SYNGR1	22	38108273	4	1.040675
rs1010170	SYNGR1	22	38108273	2	4.71012
rs1010170	SYNGR1	22	38108273	3	1.213124
rs5757650	SYNGR1	22	38108365	4	3.452245
rs5757650	SYNGR1	22	38108365	3	1.057778
rs1043395	SYNGR1	22	38110907	4	6.15613
rs2958651	FAM83F	22	38735709	3	0.509329
rs2958651	FAM83F	22	38735709	8	0.059533
rs2958651	FAM83F	22	38735709	4	0.299245
rs2958651	FAM83F	22	38735709	2	3.767345
SNP22-38882065	TNRC6B	22	38882065	1	6.131509
SNP22-38882065	TNRC6B	22	38882065	8	2.063563
SNP22-38882065	TNRC6B	22	38882065	7	3.939839
rs11703324	TNRC6B	22	39061080	7	0.823449
rs133072	MCHR1	22	39405489	3	0.853383
rs133072	MCHR1	22	39405489	1	1.225244
rs133073	MCHR1	22	39405641	8	1.127093

rs133073	MCHR1	22	39405641	5	1.435795
rs133073	MCHR1	22	39405641	3	1.757114
rs133073	MCHR1	22	39405641	4	1.378576
rs133073	MCHR1	22	39405641	1	2.086751
rs4822002	LOC730143	22	39817290	6	1.195295
rs4822002	LOC730143	22	39817290	5	1.982365
rs4822002	LOC730143	22	39817290	3	1.127752
rs9611519	L3MBTL2	22	39943134	3	0.414223
rs71948	RANGAP1	22	39975703	6	0.696384
rs71948	RANGAP1	22	39975703	5	0.062266
rs71948	RANGAP1	22	39975703	8	0.387414
rs139509	RANGAP1	22	39982679	1	3.613522
rs139509	RANGAP1	22	39982679	6	0.523212
rs139509	RANGAP1	22	39982679	3	0.468639
rs139509	RANGAP1	22	39982679	5	0.383309
rs8139784	RANGAP1	22	40011960	2	0.914614
rs8139784	RANGAP1	22	40011960	1	1.468887
rs17365991	TEF	22	40120125	3	0.480062
rs1799932	ACO2	22	40241471	1	0.224905
rs139561	MEI1	22	40525356	8	0.347772
rs4822063	SREBF2	22	40606688	2	4.37975
rs11547820	SREBF2	22	40623086	2	5.197741
rs17848337	SREBF2	22	40630894	2	0.804958
rs710194	3-Sep	22	40722506	3	0.702131
rs710194	3-Sep	22	40722506	4	1.674772
rs710194	3-Sep	22	40722506	2	3.899446
rs133335	WBP2NL	22	40746002	3	1.632522
rs133335	WBP2NL	22	40746002	4	0.77084
rs133335	WBP2NL	22	40746002	2	2.164977
rs2301521	WBP2NL	22	40753056	3	0.040477
rs2301521	WBP2NL	22	40753056	4	7.001214
rs11703233	NAGA	22	40784533	2	1.64746
rs13057094	FAM109B	22	40800263	3	2.108105
rs133383	FAM109B	22	40803549	4	4.543477
rs133383	FAM109B	22	40803549	3	2.610231
rs2269524	C22orf32	22	40805649	4	0.516084
rs2269524	C22orf32	22	40805649	3	1.09317
rs1801311	NDUFA6	22	40816669	2	6.477137
rs1801311	NDUFA6	22	40816669	4	5.362729
rs1801311	NDUFA6	22	40816669	3	0.699111
rs2743456	CYP2D7P1	22	40866257	8	1.474566
rs2743456	CYP2D7P1	22	40866257	2	2.519329
rs2743456	CYP2D7P1	22	40866257	3	1.42794
rs2743456	CYP2D7P1	22	40866257	5	1.342818
rs2743456	CYP2D7P1	22	40866257	4	2.004915
rs731026	NFAM1	22	41109823	7	0.231048
rs34963472	NFAM1	22	41113014	7	0.45285

rs5996153	NFAM1	22	41123817	7	1.382951
rs137057	dJ222E13.2	22	41300459	1	3.294046
rs137088	POLDIP3	22	41311834	3	0.217911
SNP22-41340761	POLDIP3	22	41340761	3	0.619046
rs9623659	A4GALT	22	41418915	5	1.96563
rs9623659	A4GALT	22	41418915	1	0.682943
rs9623659	A4GALT	22	41418915	4	1.994302
rs9623659	A4GALT	22	41418915	6	1.156028
rs9623659	A4GALT	22	41418915	2	0.910835
rs9607957	ARFGAP3	22	41548341	2	2.298643
rs5759013	PACSIN2	22	41619417	5	1.31676
rs5759013	PACSIN2	22	41619417	3	0.311992
rs5759013	PACSIN2	22	41619417	2	2.294565
rs5759013	PACSIN2	22	41619417	7	0.688621
rs5759125	TTLL1	22	41785376	4	0.715033
rs2076156	TTLL1	22	41796161	4	2.476979
rs2076161	TTLL1	22	41796191	7	0.420677
rs6003048	TTLL1	22	41815329	8	0.620813
rs6003048	TTLL1	22	41815329	3	0.539381
rs6003048	TTLL1	22	41815329	1	1.965911
rs13815	MCAT	22	41859258	7	0.396883
rs13815	MCAT	22	41859258	4	8.1721
rs8141806	SCUBE1	22	41929944	5	0.243722
rs34893532	SCUBE1	22	41940050	1	2.280168
rs9614382	EFCAB6	22	42307729	7	0.98723
rs9614382	EFCAB6	22	42307729	8	1.167906
rs137731	EFCAB6	22	42362375	6	0.965839
rs8141450	EFCAB6	22	42410978	7	0.666518
rs137794	EFCAB6	22	42411013	5	0.440836
rs137794	EFCAB6	22	42411013	6	0.023744
rs137794	EFCAB6	22	42411013	2	4.103874
rs5764214	EFCAB6	22	42414775	4	0.271789
rs5764214	EFCAB6	22	42414775	3	1.617423
rs5764214	EFCAB6	22	42414775	7	0.341655
SNP22-42509436	EFCAB6	22	42509436	8	1.35986
rs138057	SULT4A1	22	42552580	5	0.431626
rs138057	SULT4A1	22	42552580	1	0.731523
rs138129	PNPLA5	22	42607438	7	0.956243
rs138129	PNPLA5	22	42607438	8	4.126164
rs138129	PNPLA5	22	42607438	3	0.668357
rs138129	PNPLA5	22	42607438	1	1.217501
rs138129	PNPLA5	22	42607438	4	1.792388
rs138129	PNPLA5	22	42607438	2	1.603799
rs470093	PNPLA5	22	42607504	4	1.137066
rs470093	PNPLA5	22	42607504	6	0.763935
rs470093	PNPLA5	22	42607504	3	0.403221
rs2071884	PNPLA5	22	42618395	5	0.831355

rs2071884	PNPLA5	22	42618395	7	0.848959
rs2071884	PNPLA5	22	42618395	1	1.262566
SNP22-4265430[illegible]	PNPLA3	22	42654303	1	5.374588
SNP22-4265430[illegible]	PNPLA3	22	42654303	7	0.413571
rs2294920	SAMM50	22	42682684	6	0.903606
rs2294920	SAMM50	22	42682684	1	3.987757
rs3177036	SAMM50	22	42699537	4	1.749985
rs3177036	SAMM50	22	42699537	2	0.66758
rs6006604	SAMM50	22	42717614	3	0.646139
rs6006604	SAMM50	22	42717614	2	1.559236
rs6006604	SAMM50	22	42717614	1	0.17365
rs6006604	SAMM50	22	42717614	4	0.410577
rs6006604	SAMM50	22	42717614	5	0.843484
rs1007863	PARVB	22	42726784	1	4.705874
rs1007863	PARVB	22	42726784	5	0.228602
rs1007863	PARVB	22	42726784	7	0.11724
rs1007863	PARVB	22	42726784	6	0.620622
rs11090626	LL22NC03-75B3.6	22	42971233	4	0.73205
rs16991711	LL22NC03-75B3.6	22	42974662	3	0.339247
rs135388	LL22NC03-75B3.6	22	43012945	1	1.225916
rs135388	LL22NC03-75B3.6	22	43012945	5	3.945334
rs131154	LDOC1L	22	43269509	3	0.502316
rs131154	LDOC1L	22	43269509	8	0.000847
rs131154	LDOC1L	22	43269509	5	0.421293
rs131163	LDOC1L	22	43270799	4	9.451654
rs131163	LDOC1L	22	43270799	5	0.967338
rs131163	LDOC1L	22	43270799	8	8.232332
rs131163	LDOC1L	22	43270799	3	1.490704
rs1124898	KRT18P23	22	43312547	5	2.107591
rs138590	KRT18P23	22	43341688	3	3.75587
rs138590	KRT18P23	22	43341688	7	3.47978
rs138590	KRT18P23	22	43341688	1	4.227715
rs138593	RP3-474I12.5	22	43343984	7	0.945947
rs138593	RP3-474I12.5	22	43343984	3	0.247254
rs3213551	PRR5	22	43512199	4	0.811857
rs6007344	ARHGAP8	22	43634301	3	0.037878
rs6007344	ARHGAP8	22	43634301	6	0.690265
rs6007344	ARHGAP8	22	43634301	4	0.037033
rs6007344	ARHGAP8	22	43634301	2	6.626181
rs6007344	ARHGAP8	22	43634301	8	2.85025
rs2071763	ARHGAP8	22	43637203	1	4.880259
rs2071763	ARHGAP8	22	43637203	3	0.192025
rs2071764	ARHGAP8	22	43637278	7	0.393314
rs2071764	ARHGAP8	22	43637278	3	1.125895
rs2071764	ARHGAP8	22	43637278	1	6.101618
rs6519899	ARHGAP8	22	43637314	5	0.327112
rs6519899	ARHGAP8	22	43637314	8	5.144756

rs5766149	PHF21B	22	43656366	5	0.041601
rs5766149	PHF21B	22	43656366	3	0.227242
rs762980	PHF21B	22	43656773	4	1.972827
rs762980	PHF21B	22	43656773	3	3.774713
rs762980	PHF21B	22	43656773	5	1.625072
rs3810628	PHF21B	22	43657163	3	0.030425
rs132875	NUP50	22	43945576	6	0.611038
rs132875	NUP50	22	43945576	7	0.850697
rs226524	NUP50	22	43955895	7	0.786045
rs226524	NUP50	22	43955895	6	1.239098
rs1043731	C22orf9	22	43967041	2	2.978018
rs1043731	C22orf9	22	43967041	3	0.535685
rs1043731	C22orf9	22	43967041	5	0.163636
rs1135360	UPK3A	22	44063666	3	0.336773
rs1135360	UPK3A	22	44063666	5	0.566113
rs1057356	UPK3A	22	44070258	7	0.433752
rs1057356	UPK3A	22	44070258	3	1.394559
rs1056322	FAM118A	22	44115098	3	0.291943
rs1056322	FAM118A	22	44115098	6	7.236178
rs1056322	FAM118A	22	44115098	8	0.118479
rs2064068	FAM118A	22	44115149	3	0.643677
rs2064068	FAM118A	22	44115149	7	0.533291
rs2064068	FAM118A	22	44115149	6	6.099246
rs3747238	SMC1B	22	44118760	7	1.611008
rs3747238	SMC1B	22	44118760	3	0.742187
rs3747239	SMC1B	22	44118790	6	0.283214
rs3747239	SMC1B	22	44118790	3	0.619014
rs3747239	SMC1B	22	44118790	8	1.16223
rs3747240	SMC1B	22	44118888	8	0.885295
rs2142661	RIBC2	22	44200573	3	1.722556
rs2142662	RIBC2	22	44200599	8	2.07993
rs2142662	RIBC2	22	44200599	3	1.58176
rs1022478	RIBC2	22	44200620	7	5.434564
rs1022478	RIBC2	22	44200620	8	4.190371
rs1022478	RIBC2	22	44200620	3	1.884407
rs1119	FBLN1	22	44339948	5	0.358372
rs1119	FBLN1	22	44339948	6	3.738605
rs1119	FBLN1	22	44339948	4	1.250535
rs1119	FBLN1	22	44339948	1	6.012462
rs9627391	C22orf26	22	44825761	7	0.62988
rs9627391	C22orf26	22	44825761	1	3.480461
rs7511013	C22orf26	22	44825796	7	0.181862
rs7511013	C22orf26	22	44825796	8	3.553521
rs7511013	C22orf26	22	44825796	1	5.39115
SNP22-44828555	C22orf26	22	44828555	5	0.631368
SNP22-44828555	C22orf26	22	44828555	3	0.074777
SNP22-44828555	C22orf26	22	44828555	7	0.315281

SNP22-44828555	C22orf26	22	44828555	1	0.797318
SNP22-44828555	C22orf26	22	44828555	8	0.625694
rs8135478	FLJ27365	22	44860649	2	2.909561
rs8135478	FLJ27365	22	44860649	6	0.548986
rs9615264	PPARA	22	45011253	4	0.143809
rs9615264	PPARA	22	45011253	8	1.279125
rs1055659	PPARA	22	45016962	1	0.664967
rs1055659	PPARA	22	45016962	2	4.491777
rs9627281	LOC150383	22	45022832	1	0.402724
rs9627281	LOC150383	22	45022832	7	0.705929
rs8135363	PKDREJ	22	45030377	1	0.428498
rs4508712	PKDREJ	22	45031623	2	1.353178
rs4508712	PKDREJ	22	45031623	8	0.247116
rs4508712	PKDREJ	22	45031623	1	1.112178
rs6007740	PKDREJ	22	45032987	7	2.773847
rs6007740	PKDREJ	22	45032987	2	3.06833
rs6007740	PKDREJ	22	45032987	1	2.834852
rs6008384	PKDREJ	22	45034612	1	1.381097
rs6008384	PKDREJ	22	45034612	2	1.718997
rs6008384	PKDREJ	22	45034612	7	2.902398
rs7291444	PKDREJ	22	45034910	7	1.570808
rs7291444	PKDREJ	22	45034910	1	1.896419
rs7291444	PKDREJ	22	45034910	2	0.714832
rs6519993	PKDREJ	22	45035143	1	1.601282
rs6519993	PKDREJ	22	45035143	7	1.70974
rs6519994	PKDREJ	22	45035469	1	1.521714
rs6519994	PKDREJ	22	45035469	2	2.611752
rs35883013	FLJ20699	22	45059805	5	0.804831
rs6008598	GTSE1	22	45082907	2	0.169778
rs6008598	GTSE1	22	45082907	1	0.230277
rs6008598	GTSE1	22	45082907	7	1.186186
rs6008684	GTSE1	22	45097764	7	0.799582
rs6008684	GTSE1	22	45097764	1	0.279091
rs6008684	GTSE1	22	45097764	2	1.545219
rs6008684	GTSE1	22	45097764	5	0.08857
rs9615949	GTSE1	22	45101195	7	0.835572
rs9615949	GTSE1	22	45101195	1	1.058035
rs6008729	GTSE1	22	45106092	1	0.260069
rs6008729	GTSE1	22	45106092	2	0.204246
rs6008729	GTSE1	22	45106092	7	6.639506
rs9615348	GTSE1	22	45108002	1	0.413007
rs9615348	GTSE1	22	45108002	2	4.577433
rs11090865	TRMU	22	45110353	2	1.416243
rs11090865	TRMU	22	45110353	1	0.712194
rs11090865	TRMU	22	45110353	7	1.304255
rs6007891	CELSR1	22	45135497	3	0.41939
rs6007891	CELSR1	22	45135497	1	4.17879

rs6007891	CELSR1	22	45135497	7	4.002978
SNP22-45138750	CELSR1	22	45138750	4	0.572254
SNP22-45138750	CELSR1	22	45138750	1	5.278499
SNP22-45138750	CELSR1	22	45138750	3	0.701874
SNP22-45138750	CELSR1	22	45138750	7	0.163439
SNP22-45139145	CELSR1	22	45139145	3	0.140683
SNP22-45139145	CELSR1	22	45139145	4	0.8608
SNP22-45139145	CELSR1	22	45139145	7	9.470546
SNP22-45139145	CELSR1	22	45139145	5	0.28268
SNP22-45139145	CELSR1	22	45139145	1	10.18617
rs4044210	CELSR1	22	45164979	7	1.04686
rs4044210	CELSR1	22	45164979	3	0.183916
rs4044210	CELSR1	22	45164979	1	5.885251
SNP22-45208041	CELSR1	22	45208041	5	7.8547
rs17761627	DIP	22	45452525	2	1.635866
rs17761627	DIP	22	45452525	7	0.491342
rs17761627	DIP	22	45452525	5	0.969181
rs2748343	DIP	22	45452539	5	0.680357
rs2748343	DIP	22	45452539	1	0.155767
rs2748343	DIP	22	45452539	7	0.411384
rs2748343	DIP	22	45452539	8	0.719262
rs1047123	CERK	22	45459465	8	7.818617
rs1047123	CERK	22	45459465	7	0.21746
rs1047123	CERK	22	45459465	1	6.464611
rs801719	CERK	22	45461009	7	4.353339
rs801719	CERK	22	45461009	4	0.061242
rs801719	CERK	22	45461009	1	4.524921
rs801719	CERK	22	45461009	5	0.99781
rs13057352	CERK	22	45473899	8	0.380528
rs13057352	CERK	22	45473899	4	0.730205
rs12166204	CERK	22	45495569	3	0.233965
rs15411	TBC1D22A	22	45948269	8	3.670032
rs15411	TBC1D22A	22	45948269	4	0.099244
rs15411	TBC1D22A	22	45948269	6	0.267803
rs877528	RP11-191L9.1	22	46399650	2	1.852622
rs761880	RP11-191L9.1	22	46405418	1	0.709697
SNP22-47531934	FAM19A5	22	47531934	8	7.66422
SNP22-47531934	FAM19A5	22	47531934	7	0.177113
rs2688173	LOC643653	22	47996161	3	0.34212
rs2688173	LOC643653	22	47996161	5	1.051371
rs2688173	LOC643653	22	47996161	6	0.881838
SNP22-48573857	BRD1	22	48573857	6	0.451264
SNP22-48573857	BRD1	22	48573857	1	5.789783
rs910798	ZBED4	22	48664442	7	0.020047
rs910798	ZBED4	22	48664442	1	5.830425
rs910798	ZBED4	22	48664442	3	0.401575
rs5770755	ZBED4	22	48666140	7	0.440661

rs5770755	ZBED4	22	48666140	3	0.606691
rs1608	CRELD2	22	48706947	1	3.096614
SNP22-48742697	PIM3	22	48742697	7	8.863169
SNP22-48742697	PIM3	22	48742697	1	4.05509
SNP22-48742697	PIM3	22	48742697	3	0.466602
SNP22-48777607	IL17REL	22	48777607	1	0.573916
rs738333	RP3-355C18.2	22	48811034	2	2.558283
rs738333	RP3-355C18.2	22	48811034	6	1.999373
rs738333	RP3-355C18.2	22	48811034	3	1.147014
rs137919	MLC1	22	48842091	5	0.096487
rs137919	MLC1	22	48842091	3	1.060139
rs6010165	MLC1	22	48857400	5	3.307328
rs9617066	MOV10L1	22	48872628	7	1.70219
rs9617066	MOV10L1	22	48872628	5	2.166377
rs9617066	MOV10L1	22	48872628	3	0.584412
rs9617066	MOV10L1	22	48872628	2	0.697115
rs3810971	MOV10L1	22	48888793	3	1.850813
rs2340601	MOV10L1	22	48914600	2	2.724411
rs2340601	MOV10L1	22	48914600	5	3.431693
rs2340601	MOV10L1	22	48914600	3	0.897583
rs2340601	MOV10L1	22	48914600	7	1.816172
rs5771206	PANX2	22	48958933	7	0.444935
rs5771206	PANX2	22	48958933	6	0.426724
rs5771206	PANX2	22	48958933	1	0.706523
rs8238	TRABD	22	48979724	3	0.795362
rs8238	TRABD	22	48979724	7	0.354771
rs8238	TRABD	22	48979724	4	0.086235
rs8238	TRABD	22	48979724	1	5.920382
rs6712	TRABD	22	48980049	2	4.584935
rs6712	TRABD	22	48980049	6	6.27156
rs6712	TRABD	22	48980049	4	7.034979
rs4838865	TUBGCP6	22	49006739	8	6.919021
rs4838865	TUBGCP6	22	49006739	7	3.777059
rs1129880	MAPK12	22	49036424	4	0.470083
rs1129880	MAPK12	22	49036424	2	0.272982
rs1129880	MAPK12	22	49036424	7	4.472261
rs1129880	MAPK12	22	49036424	1	0.422431
rs1129880	MAPK12	22	49036424	5	0.638267
rs2076139	MAPK11	22	49046788	3	0.323926
SNP22-49064261	PLXNB2	22	49064261	6	0.166534
SNP22-49064261	PLXNB2	22	49064261	4	5.601664
SNP22-49064261	PLXNB2	22	49064261	7	6.54146
SNP22-49064261	PLXNB2	22	49064261	3	0.137496
SNP22-49094722	FAM116B	22	49094722	2	4.713101
SNP22-49224010	SAPS2	22	49224010	1	5.178111
rs45484793	SAPS2	22	49225315	1	5.399742
rs743615	LMF2	22	49290994	6	0.789117

rs743615	LMF2	22	49290994	1	0.032627
rs743615	LMF2	22	49290994	3	0.434448
rs743615	LMF2	22	49290994	8	6.703037
rs131824	LMF2	22	49292122	3	0.151842
rs131824	LMF2	22	49292122	8	7.3035
rs131824	LMF2	22	49292122	5	1.215413
rs131824	LMF2	22	49292122	6	6.748809
rs131824	LMF2	22	49292122	7	3.620434
rs131824	LMF2	22	49292122	1	0.165268
rs140523	SCO2	22	49309648	5	0.039632
rs140523	SCO2	22	49309648	7	0.580752
rs140523	SCO2	22	49309648	1	5.050699
rs140523	SCO2	22	49309648	4	6.041282
rs140523	SCO2	22	49309648	6	0.214067
rs1140555	MAPK8IP2	22	49392056	8	0.017296
rs1140555	MAPK8IP2	22	49392056	7	0.9356
rs1140555	MAPK8IP2	22	49392056	1	1.08874
rs6151429	ARSA	22	49410343	8	0.955208
rs6151429	ARSA	22	49410343	5	4.783385
rs6151428	ARSA	22	49410476	2	2.973289
rs743616	ARSA	22	49410905	5	2.401798
rs743616	ARSA	22	49410905	1	2.311667
rs743616	ARSA	22	49410905	8	1.790447
rs743616	ARSA	22	49410905	4	1.129568
rs743616	ARSA	22	49410905	7	0.244269
rs743616	ARSA	22	49410905	3	0.077932
rs743616	ARSA	22	49410905	2	0.582551
rs9616915	SHANK3	22	49464446	7	1.453943
rs9616915	SHANK3	22	49464446	3	1.448756
rs2301584	SHANK3	22	49518363	8	8.991745
rs5939319	XG	X	2710157	5	3.120836
rs2306734	GYG2	X	2787985	7	1.749755
rs2306734	GYG2	X	2787985	3	0.024892
rs211659	GYG2	X	2809479	8	2.374155
rs211659	GYG2	X	2809479	6	0.024567
rs211660	GYG2	X	2810295	7	2.174141
rs211660	GYG2	X	2810295	8	0.003686
rs1269	GYG2	X	2810624	2	1.924928
rs1269	GYG2	X	2810624	5	0.315938
rs1269	GYG2	X	2810624	7	1.186827
rs1269	GYG2	X	2810624	3	1.519249
rs1269	GYG2	X	2810624	1	0.730317
rs1268	GYG2	X	2810677	5	1.527842
rs1268	GYG2	X	2810677	1	1.282567
rs1268	GYG2	X	2810677	7	4.008661
rs1268	GYG2	X	2810677	8	4.199967
rs1268	GYG2	X	2810677	3	0.257463

rs17325750	ARSE	X	2877413	5	2.439202
rs17325750	ARSE	X	2877413	6	0.246818
rs5939407	ARSH	X	2952109	7	1.683248
rs5983003	ARSF	X	3012687	7	1.192013
rs17051478	ARSF	X	3038260	1	0.183144
rs17051478	ARSF	X	3038260	2	2.89537
rs10522044	ARSF	X	3040426	1	0.123499
rs1635242	MXRA5	X	3245724	7	0.139564
rs1635242	MXRA5	X	3245724	1	0.455805
rs1974522	MXRA5	X	3248733	7	0.734225
rs1974522	MXRA5	X	3248733	4	3.406662
rs1974522	MXRA5	X	3248733	1	4.463918
rs1726199	MXRA5	X	3249545	1	2.91273
rs1726199	MXRA5	X	3249545	7	0.548324
rs1635246	MXRA5	X	3250343	4	3.956029
rs1635246	MXRA5	X	3250343	3	0.608123
rs1635246	MXRA5	X	3250343	1	4.151975
rs1635246	MXRA5	X	3250343	5	0.317438
rs1635247	MXRA5	X	3251050	7	0.450333
rs1635247	MXRA5	X	3251050	3	0.690367
rs1635247	MXRA5	X	3251050	1	5.119123
rs1635247	MXRA5	X	3251050	4	1.116888
rs5983119	MXRA5	X	3251256	7	2.262541
rs5983119	MXRA5	X	3251256	1	4.612554
rs5983119	MXRA5	X	3251256	3	0.893599
rs5983119	MXRA5	X	3251256	6	0.616861
rs1635249	MXRA5	X	3251317	6	0.172408
rs1635249	MXRA5	X	3251317	3	0.931134
rs1635249	MXRA5	X	3251317	1	5.933102
rs5916269	NLGN4X	X	5820299	8	7.881012
rs2290488	NLGN4X	X	6155855	4	0.207141
rs2290488	NLGN4X	X	6155855	3	0.524243
rs5916355	LOC729193	X	6158318	2	0.704193
rs1061191	CLCN4	X	10162748	7	1.174158
rs5934817	CLCN4	X	10163188	1	4.727814
rs5934817	CLCN4	X	10163188	7	1.230786
rs3827468	FRMPD4	X	12649215	1	0.073539
rs3827468	FRMPD4	X	12649215	7	0.350742
rs3827468	FRMPD4	X	12649215	3	1.127788
rs3827468	FRMPD4	X	12649215	5	1.010957
rs3827468	FRMPD4	X	12649215	4	0.655694
rs5979716	FRMPD4	X	12649280	3	0.212022
rs17321823	FRMPD4	X	12650039	5	1.742159
rs17321823	FRMPD4	X	12650039	4	2.429685
rs17321823	FRMPD4	X	12650039	1	0.534368
rs179008	TLR7	X	12813580	3	0.312023
rs3747414	TLR8	X	12849333	3	0.924646

rs11095602	NULL	X	12972643	8	1.676295
rs11095602	NULL	X	12972643	6	2.460757
rs11095602	NULL	X	12972643	2	8.163481
rs12391470	LOC645769	X	13579329	3	0.246187
rs12391470	LOC645769	X	13579329	1	0.589807
rs2285635	OFD1	X	13662816	2	2.317328
rs2285635	OFD1	X	13662816	8	4.533257
rs2285635	OFD1	X	13662816	5	1.841141
rs2285635	OFD1	X	13662816	4	2.215153
rs7057480	GEMIN8	X	13954163	4	1.726716
rs7057480	GEMIN8	X	13954163	7	1.625187
rs7057480	GEMIN8	X	13954163	6	2.245188
rs7057480	GEMIN8	X	13954163	2	1.337818
rs7057480	GEMIN8	X	13954163	5	1.147334
rs7057480	GEMIN8	X	13954163	1	1.118225
rs9560	ASB9	X	15172293	8	0.531404
rs9560	ASB9	X	15172293	1	5.461388
rs9560	ASB9	X	15172293	3	0.415493
rs9560	ASB9	X	15172293	4	0.4955
rs9560	ASB9	X	15172293	7	8.149341
rs899646	ASB9	X	15198114	3	0.061328
rs899646	ASB9	X	15198114	5	1.975921
rs899646	ASB9	X	15198114	4	1.694452
rs12011065	FIGF	X	15291342	5	0.66466
rs6632528	FIGF	X	15312319	3	0.708863
rs4986945	GRPR	X	16078388	1	4.091976
rs4986945	GRPR	X	16078388	3	0.719418
rs4986945	GRPR	X	16078388	5	2.212576
rs3747411	GRPR	X	16080998	5	1.537698
rs3747411	GRPR	X	16080998	7	0.255599
rs3747411	GRPR	X	16080998	3	0.289072
rs3747411	GRPR	X	16080998	2	1.612829
rs10521655	GRPR	X	16081261	3	0.672158
rs10521655	GRPR	X	16081261	5	1.312024
rs10521655	GRPR	X	16081261	2	3.358579
rs3213544	SCML2	X	18251955	6	3.165441
rs3213544	SCML2	X	18251955	7	1.807341
rs35478150	CDKL5	X	18548003	1	3.298424
rs12009120	RPS6KA3	X	20114382	3	0.810598
rs12009120	RPS6KA3	X	20114382	5	1.221072
rs178710	PHEX	X	21960955	6	0.201614
rs178710	PHEX	X	21960955	1	3.202358
rs8680	APOO	X	23768373	5	2.662803
rs8680	APOO	X	23768373	2	4.2893
rs8680	APOO	X	23768373	3	2.073978
rs5970618	APOO	X	23835755	2	2.41888
rs5970618	APOO	X	23835755	3	0.947015

rs3761611	PCYT1B	X	24488227	4	0.117261
rs3761611	PCYT1B	X	24488227	8	1.636299
rs3761610	PCYT1B	X	24488472	4	3.358787
rs3761610	PCYT1B	X	24488472	1	0.060388
rs3761610	PCYT1B	X	24488472	8	6.078153
rs10156959	PCYT1B	X	24488724	1	0.453618
rs1207651	PCYT1B	X	24575194	4	0.383285
rs11795624	MAGEB6	X	26120507	7	2.422074
rs6526806	IL1RAPL1	X	28717363	4	5.333054
rs5972091	MAGEB2	X	30147947	3	0.401725
rs5927577	LOC392437	X	30558644	1	3.022529
rs228406	DMD	X	32413115	5	0.317254
rs228406	DMD	X	32413115	1	0.090288
rs3813166	PRRG1	X	37198802	1	5.352497
rs3813166	PRRG1	X	37198802	3	0.626488
rs3126414	NULL	X	37364104	3	1.660849
rs3126414	NULL	X	37364104	1	2.823909
rs3126415	NULL	X	37371012	1	3.751878
rs3126415	NULL	X	37371012	3	0.845879
rs5964151	CYBB	X	37555673	3	0.583629
rs4827331	SYTL5	X	37833776	4	0.032242
rs9934	SRPX	X	37893671	2	1.006561
rs9934	SRPX	X	37893671	6	3.49914
rs9934	SRPX	X	37893671	5	1.640917
rs35318931	SRPX	X	37894065	6	0.273579
rs5917557	RPGR	X	38030855	8	0.078836
rs5917557	RPGR	X	38030855	1	1.3479
rs5917557	RPGR	X	38030855	6	0.221372
rs5917557	RPGR	X	38030855	7	0.405011
rs5917557	RPGR	X	38030855	5	0.058217
rs1801688	RPGR	X	38032114	4	0.986641
rs1801687	RPGR	X	38041621	4	5.855637
rs3269	TSPAN7	X	38433027	4	0.51375
rs3269	TSPAN7	X	38433027	7	2.467841
rs6520683	MED14	X	40458101	5	0.772319
rs4827256	USP9X	X	40978357	6	6.463232
rs4827256	USP9X	X	40978357	7	0.792973
rs4827256	USP9X	X	40978357	2	1.758198
rs4827256	USP9X	X	40978357	1	0.197902
rs3013122	NYX	X	41191808	5	0.676536
rs913602	GPR34	X	41439758	7	3.544208
rs1023065	GPR82	X	41472162	7	2.511885
rs1137070	MAOA	X	43488335	1	4.939524
rs1137070	MAOA	X	43488335	6	0.297655
rs1137070	MAOA	X	43488335	3	0.702198
rs4824526	UTX	X	44823507	2	3.62124
rs4824526	UTX	X	44823507	5	0.291314

rs4824526	UTX	X	44823507	8	6.971386
rs735716	CHST7	X	46342730	1	0.087412
rs735716	CHST7	X	46342730	3	0.375794
rs735716	CHST7	X	46342730	5	2.909397
rs735716	CHST7	X	46342730	2	3.785346
rs6611277	SLC9A7	X	46357770	3	0.865697
rs6611277	SLC9A7	X	46357770	2	3.542849
rs6611277	SLC9A7	X	46357770	1	0.124893
rs6611277	SLC9A7	X	46357770	5	0.191692
rs6611277	SLC9A7	X	46357770	7	1.825891
rs1805147	RP2	X	46604442	3	0.303658
rs11558783	UBA1	X	46947478	2	4.575912
rs11558783	UBA1	X	46947478	1	2.821267
rs2294219	SYN1	X	47351305	6	0.106455
rs2294219	SYN1	X	47351305	1	2.265688
rs1048118	CFP	X	47368744	6	1.983301
rs909713	WASF4	X	47547916	1	3.856986
rs909713	WASF4	X	47547916	3	3.982943
rs909713	WASF4	X	47547916	6	3.962655
rs909713	WASF4	X	47547916	7	5.012244
rs909713	WASF4	X	47547916	4	3.677713
rs1207506	WASF4	X	47548932	1	3.550068
rs1207491	LOC441493	X	47580491	3	1.975377
rs1207491	LOC441493	X	47580491	4	0.445928
rs1207491	LOC441493	X	47580491	1	4.131951
rs1207491	LOC441493	X	47580491	7	0.591428
rs235824	WDR13	X	48341167	7	1.550544
rs2075866	CACNA1F	X	48958908	7	0.321567
rs2075866	CACNA1F	X	48958908	3	2.079887
rs2235127	CACNA1F	X	48968235	3	0.903324
rs2235127	CACNA1F	X	48968235	7	0.846069
rs34162630	CACNA1F	X	48969443	3	0.187739
rs4143304	DGKK	X	50163310	3	0.484148
rs1151715	NUDT10	X	51091891	1	0.465911
rs11091720	GPR173	X	53123590	6	0.790098
rs11091720	GPR173	X	53123590	3	1.738096
rs11091720	GPR173	X	53123590	7	0.435616
rs1264013	RIBC1	X	53474347	3	1.511915
rs1264013	RIBC1	X	53474347	4	0.065718
rs6638360	HUWE1	X	53658372	4	0.612015
rs6638360	HUWE1	X	53658372	3	0.881494
rs17316491	ITIH5L	X	54798255	3	1.495038
rs2301416	APEX2	X	55045588	2	7.994389
rs3126255	USP51	X	55531543	7	0.2172
rs3126255	USP51	X	55531543	2	1.112674
rs5960093	RRAGB	X	55761010	2	2.098377
rs5960093	RRAGB	X	55761010	7	2.156099

rs5960093	RRAGB	X	55761010	6	1.167966
rs2516023	FAAH2	X	57330082	6	3.201441
rs2516023	FAAH2	X	57330082	7	1.21716
rs1367830	FAAH2	X	57491857	8	0.271675
rs1367830	FAAH2	X	57491857	6	0.5135
rs1044165	VSIG4	X	65158451	5	0.213594
rs5919015	HEPH	X	65299410	3	0.147424
rs1028348	HEPH	X	65300888	1	0.950723
rs1028348	HEPH	X	65300888	2	0.66783
rs1028348	HEPH	X	65300888	5	2.292542
rs2254886	OPHN1	X	67182142	7	3.735887
rs2254886	OPHN1	X	67182142	6	0.439993
rs2254886	OPHN1	X	67182142	3	0.270876
rs2254886	OPHN1	X	67182142	4	0.855353
rs2230423	EFNB1	X	67977228	4	0.224384
rs3747463	TMEM28	X	68668446	3	2.338753
rs3747463	TMEM28	X	68668446	6	0.349953
rs2281932	DGAT2L4	X	69177661	1	1.577644
rs2281932	DGAT2L4	X	69177661	4	4.426721
rs2281932	DGAT2L4	X	69177661	6	0.607029
rs2281932	DGAT2L4	X	69177661	2	0.908631
rs3829709	P2RY4	X	69395500	4	0.018053
rs1152187	P2RY4	X	69395667	4	0.199308
rs12857550	ARR3	X	69415207	7	0.799239
rs1130009	LOC158830	X	70240882	1	2.214814
rs1130009	LOC158830	X	70240882	8	2.447598
rs9887370	RGAG4	X	71265868	8	1.986651
rs12393722	RGAG4	X	71266499	4	0.443518
rs12393722	RGAG4	X	71266499	8	3.1861
rs7884806	FLJ44635	X	71296427	4	1.094118
rs7884806	FLJ44635	X	71296427	8	3.911727
rs7884806	FLJ44635	X	71296427	7	1.131847
rs660392	HDAC8	X	71466237	4	0.610423
rs660392	HDAC8	X	71466237	8	2.243211
rs660392	HDAC8	X	71466237	7	1.89792
rs660392	HDAC8	X	71466237	5	1.722284
rs11093407	PABPC1L2A	X	72214266	4	1.623508
rs13789	ITM2A	X	78502870	6	0.600386
rs1113265	FAM46D	X	79585249	3	0.438774
rs1113265	FAM46D	X	79585249	2	2.545795
rs16980188	POF1B	X	84419457	6	3.752724
rs16980188	POF1B	X	84419457	4	1.939018
rs12557804	POF1B	X	84420064	6	1.016476
rs12557804	POF1B	X	84420064	7	2.751752
rs12557804	POF1B	X	84420064	4	0.086725
rs363774	POF1B	X	84449791	7	4.4347
rs363774	POF1B	X	84449791	5	0.341161

rs363774	POF1B	X	84449791	8	1.194619
rs363774	POF1B	X	84449791	3	0.241704
rs363774	POF1B	X	84449791	1	4.819035
rs222084	KLHL4	X	86773900	3	0.496941
rs222084	KLHL4	X	86773900	6	0.649824
rs222084	KLHL4	X	86773900	5	0.306835
rs222113	KLHL4	X	86808742	6	0.394835
rs1045686	NAP1L3	X	92814290	7	1.488733
rs1045686	NAP1L3	X	92814290	8	3.009026
rs2642219	RPA4	X	96026062	4	0.297735
rs2642219	RPA4	X	96026062	2	5.174047
rs1335268	PCDH19	X	99437539	8	2.664425
rs1953337	PCDH19	X	99548625	4	0.747521
rs1953337	PCDH19	X	99548625	1	5.538073
rs1953337	PCDH19	X	99548625	3	1.62501
rs1953337	PCDH19	X	99548625	8	2.622112
rs1802288	TSPAN6	X	99776860	7	2.110357
rs1802288	TSPAN6	X	99776860	1	3.729851
rs1802288	TSPAN6	X	99776860	3	0.224067
rs1343213	SRPX2	X	99785900	6	2.619207
rs1343213	SRPX2	X	99785900	3	0.882294
rs8780	SYTL4	X	99816188	6	0.838586
rs8780	SYTL4	X	99816188	3	0.762102
rs8780	SYTL4	X	99816188	1	3.363814
rs8780	SYTL4	X	99816188	7	5.307539
rs8780	SYTL4	X	99816188	4	0.369398
rs41307262	ARL13A	X	100129192	8	0.675901
rs5921705	CXorf34	X	100152208	2	3.808848
rs4828078	DRP2	X	100402448	6	0.240018
rs4828078	DRP2	X	100402448	7	2.520762
rs4828078	DRP2	X	100402448	8	0.55151
rs6523506	ARMCX4	X	100640066	4	0.105761
rs6523506	ARMCX4	X	100640066	1	4.439317
rs6523506	ARMCX4	X	100640066	7	0.623965
rs7060868	ARMCX4	X	100640519	7	1.165217
rs7060868	ARMCX4	X	100640519	3	0.931148
rs7060868	ARMCX4	X	100640519	1	3.499978
rs7060868	ARMCX4	X	100640519	4	3.923877
rs7060491	ARMCX4	X	100640805	4	1.857247
rs7060491	ARMCX4	X	100640805	1	6.627084
rs7055900	ARMCX1	X	100692287	3	0.233033
rs5944778	ZMAT1	X	101028449	1	5.633508
rs5944778	ZMAT1	X	101028449	3	0.64313
rs5944778	ZMAT1	X	101028449	7	1.522984
rs5944778	ZMAT1	X	101028449	5	1.525506
rs5986812	ZMAT1	X	101030810	3	0.988915
rs5986812	ZMAT1	X	101030810	5	1.251883

rs5986812	ZMAT1	X	101030810	1	4.383883
rs5944883	ZMAT1	X	101031610	7	0.69219
rs1045761	TCEAL7	X	102472558	7	1.224607
rs1045761	TCEAL7	X	102472558	1	0.44862
rs1045761	TCEAL7	X	102472558	2	0.166386
rs1180895	RAB40A	X	102642207	5	1.654544
rs1180895	RAB40A	X	102642207	6	0.76927
rs1126707	PLP1	X	102929538	1	0.525522
rs1126707	PLP1	X	102929538	2	3.800319
rs6621755	LOC644261	X	103069416	3	2.218766
rs6621755	LOC644261	X	103069416	5	1.07713
rs6621755	LOC644261	X	103069416	7	2.115617
rs4907851	LOC644261	X	103070129	1	1.429507
rs4907851	LOC644261	X	103070129	6	0.899098
rs5916825	MCART6	X	103234886	4	2.564691
rs5916825	MCART6	X	103234886	7	2.078362
rs5916825	MCART6	X	103234886	5	2.173869
rs3764765	IL1RAPL2	X	104879619	3	1.465966
rs3764765	IL1RAPL2	X	104879619	7	2.757247
rs3764765	IL1RAPL2	X	104879619	1	1.352584
rs209373	NRK	X	105038938	2	2.725855
rs209373	NRK	X	105038938	5	1.992436
rs209109	NRK	X	105088291	5	1.838054
rs209109	NRK	X	105088291	2	3.854401
rs209111	NRK	X	105089256	5	0.046614
rs17254305	VSIG1	X	107202589	6	0.090439
rs5973851	COL4A6	X	107304386	6	3.85648
rs5973851	COL4A6	X	107304386	2	0.327821
rs5973851	COL4A6	X	107304386	7	2.289137
rs5973851	COL4A6	X	107304386	1	1.563258
rs1042065	COL4A6	X	107320344	1	0.374009
rs1801164	IRS4	X	107863596	7	0.129771
rs1801164	IRS4	X	107863596	2	1.164549
rs1801164	IRS4	X	107863596	6	0.056314
rs12008095	GUCY2F	X	108595208	2	1.865511
rs12008095	GUCY2F	X	108595208	3	1.764079
rs494589	GUCY2F	X	108611792	5	1.116762
rs494589	GUCY2F	X	108611792	3	0.421868
rs5942633	RGAG1	X	109584422	1	3.082878
rs2404177	CHRDL1	X	109805537	7	1.123419
rs2404177	CHRDL1	X	109805537	1	1.651292
rs5929415	LHFPL1	X	111760922	1	4.577973
rs5929415	LHFPL1	X	111760922	3	0.340747
rs5929415	LHFPL1	X	111760922	4	0.462815
rs7064462	LHFPL1	X	111800900	3	0.144505
rs583430	AMOT	X	111907348	6	0.979412
rs583430	AMOT	X	111907348	4	0.574687

rs583430	AMOT	X	111907348	3	0.635842
rs5194	AGTR2	X	115218858	1	2.51269
rs6603401	WDR44	X	117412170	7	2.728753
rs2286977	DOCK11	X	117584169	3	0.752997
rs2286977	DOCK11	X	117584169	6	1.140302
rs2286977	DOCK11	X	117584169	1	3.644321
rs2286977	DOCK11	X	117584169	7	1.165173
rs2379118	DOCK11	X	117703801	2	0.944315
rs2379118	DOCK11	X	117703801	1	0.870202
rs2186106	DOCK11	X	117703930	1	0.266986
rs2186106	DOCK11	X	117703930	8	1.523467
rs261663	LOC159007	X	117896179	8	4.093395
rs3813933	LONRF3	X	117992813	5	1.945299
rs3813933	LONRF3	X	117992813	3	0.632179
rs217976	SLC25A43	X	118417394	1	0.066502
rs217976	SLC25A43	X	118417394	5	2.291079
rs17261110	SLC25A43	X	118471475	5	2.326039
rs17261110	SLC25A43	X	118471475	1	5.082413
rs17261110	SLC25A43	X	118471475	8	2.23038
rs12390	SLC25A5	X	118488464	2	1.32529
rs2782224	ANKRD58	X	118777165	1	0.223274
rs2782224	ANKRD58	X	118777165	4	1.519198
rs2782224	ANKRD58	X	118777165	2	0.332047
rs2782224	ANKRD58	X	118777165	5	0.892042
rs708463	RNF113A	X	118889733	8	3.841073
rs708463	RNF113A	X	118889733	6	0.249444
rs708463	RNF113A	X	118889733	5	2.05641
rs708463	RNF113A	X	118889733	2	3.476463
rs42889	LAMP2	X	119455569	8	3.39735
rs42889	LAMP2	X	119455569	4	0.524456
rs42889	LAMP2	X	119455569	3	0.442571
rs42889	LAMP2	X	119455569	7	1.667478
rs42889	LAMP2	X	119455569	1	5.741722
rs502434	GRIA3	X	122364958	3	0.923484
rs502434	GRIA3	X	122364958	7	1.460566
rs502434	GRIA3	X	122364958	5	2.516363
rs502434	GRIA3	X	122364958	1	4.936334
rs502434	GRIA3	X	122364958	4	3.175378
rs4825872	LOC402422	X	122524244	1	0.672779
rs4825872	LOC402422	X	122524244	5	0.920333
rs4825872	LOC402422	X	122524244	6	1.919073
rs4825872	LOC402422	X	122524244	4	1.58691
rs17334746	BIRC4	X	122870388	3	1.027217
rs17334746	BIRC4	X	122870388	6	1.546361
rs17334746	BIRC4	X	122870388	5	1.904042
rs17334746	BIRC4	X	122870388	8	1.105364
rs5958343	BIRC4	X	122873953	7	2.256564

rs5958343	BIRC4	X	122873953	6	1.643037
rs5958343	BIRC4	X	122873953	8	2.92487
rs5958343	BIRC4	X	122873953	3	0.787843
rs5958343	BIRC4	X	122873953	5	2.53427
rs5958343	BIRC4	X	122873953	1	4.625594
rs3747343	XPNPEP2	X	128708295	6	3.937833
rs3747343	XPNPEP2	X	128708295	8	2.369955
rs3747343	XPNPEP2	X	128708295	2	2.507631
rs3747343	XPNPEP2	X	128708295	1	2.461047
rs3747343	XPNPEP2	X	128708295	3	1.853408
rs3747343	XPNPEP2	X	128708295	5	2.156586
rs1053486	ZDHHC9	X	128766639	3	0.843024
rs3788847	ELF4	X	129027497	5	1.152511
rs3788847	ELF4	X	129027497	2	9.691149
rs1139851	AIFM1	X	129111201	6	1.165619
rs1139851	AIFM1	X	129111201	4	3.072134
rs1139851	AIFM1	X	129111201	2	3.468857
rs608117	ZNF280C	X	129166447	6	3.473417
rs209238	ZNF280C	X	129176976	2	2.897915
rs209238	ZNF280C	X	129176976	4	1.614621
rs1128617	IGSF1	X	130243499	8	1.098027
rs17316625	OR13H1	X	130506277	6	3.047969
rs17316625	OR13H1	X	130506277	3	2.473665
rs17316625	OR13H1	X	130506277	1	4.491384
rs17316625	OR13H1	X	130506277	5	3.980474
rs17316625	OR13H1	X	130506277	4	1.603575
rs3764771	FRMD7	X	131038916	1	3.316298
rs5977623	FRMD7	X	131040193	3	0.268225
rs5977623	FRMD7	X	131040193	1	3.787343
rs5977623	FRMD7	X	131040193	5	0.412815
rs7051368	FRMD7	X	131040625	4	2.002085
rs7051368	FRMD7	X	131040625	8	0.985838
rs2498769	LOC644538	X	133954060	3	0.3868
rs2498769	LOC644538	X	133954060	8	1.03456
rs2498769	LOC644538	X	133954060	1	2.591888
rs2498769	LOC644538	X	133954060	6	0.175599
rs2498769	LOC644538	X	133954060	7	1.482352
rs7042	ZNF75	X	134247673	6	0.344817
rs1419828	ZNF75	X	134305279	6	0.292013
rs1419828	ZNF75	X	134305279	3	1.52113
rs1419828	ZNF75	X	134305279	5	2.164535
rs1419828	ZNF75	X	134305279	1	4.7698
rs4829799	SAGE1	X	134821671	1	3.271692
rs4829799	SAGE1	X	134821671	4	2.240966
rs2273221	MAP7D3	X	135138451	6	0.249328
rs2273221	MAP7D3	X	135138451	2	2.313089
rs2273221	MAP7D3	X	135138451	3	1.464105

rs2273221	MAP7D3	X	135138451	4	1.325892
rs4829830	GPR112	X	135257169	5	0.422815
rs4829830	GPR112	X	135257169	3	0.086171
rs912002	GPR112	X	135258149	3	0.322406
rs5930933	GPR112	X	135259024	2	0.612298
rs5930933	GPR112	X	135259024	5	0.950442
rs5930942	GPR112	X	135324064	3	0.242285
rs440051	F9	X	138472583	2	1.25589
rs1051619	MCF2	X	138507378	2	3.050274
rs1051619	MCF2	X	138507378	1	0.052001
rs1051619	MCF2	X	138507378	8	3.345889
rs1051619	MCF2	X	138507378	3	1.283147
rs1051619	MCF2	X	138507378	7	2.888499
rs2239837	LOC645188	X	140542435	3	0.023313
rs3765272	MAGEC2	X	141118531	1	3.295912
rs3765272	MAGEC2	X	141118531	3	2.590793
rs3765272	MAGEC2	X	141118531	2	3.575717
rs3765272	MAGEC2	X	141118531	7	3.14167
rs3765272	MAGEC2	X	141118531	5	3.880769
rs237520	UBE2NL	X	142795134	3	2.413794
rs237520	UBE2NL	X	142795134	4	3.879593
rs3810704	SLITRK2	X	144711944	8	1.841287
rs3810704	SLITRK2	X	144711944	5	0.381243
rs3810704	SLITRK2	X	144711944	2	3.969761
rs12011040	AFF2	X	147842900	2	3.372488
rs6641482	AFF2	X	147887801	7	0.030808
rs10126316	MAGEA11	X	148575735	1	0.778607
rs5925425	CD99L2	X	149687406	1	4.230178
rs5925425	CD99L2	X	149687406	8	3.964463
rs561077	GPR50	X	150100307	5	1.152249
rs561077	GPR50	X	150100307	1	0.04152
rs561077	GPR50	X	150100307	7	0.202543
rs7067140	LOC203547	X	150326043	5	2.39373
rs7067140	LOC203547	X	150326043	4	0.588855
rs7067140	LOC203547	X	150326043	3	0.380303
rs7067140	LOC203547	X	150326043	7	2.893652
rs7067140	LOC203547	X	150326043	1	0.187256
rs7067140	LOC203547	X	150326043	2	2.181237
rs879371	CNGA2	X	150664131	3	0.490399
rs1061418	GABRE	X	150873636	5	2.254177
rs2020030	MAGEA5	X	151034283	6	1.707517
rs2020030	MAGEA5	X	151034283	8	1.206506
rs6627242	KRT8P8	X	151399255	4	1.576174
rs6627242	KRT8P8	X	151399255	1	1.800708
rs6627242	KRT8P8	X	151399255	5	1.595734
rs6627242	KRT8P8	X	151399255	3	0.875769
rs6526155	PNMA3	X	151977198	7	0.057879

rs1045069	PNMA3	X	151979017	7	2.272222
rs1045069	PNMA3	X	151979017	5	0.360269
rs1045069	PNMA3	X	151979017	6	0.267515
rs2301186	PNMA6A	X	151993619	5	1.81714
rs2301186	PNMA6A	X	151993619	6	0.34295
rs2301187	PNMA6A	X	151993851	4	1.698504
rs1467881	ZNF275	X	152263302	1	2.553397
rs1467881	ZNF275	X	152263302	7	1.234809
rs4828611	ZNF275	X	152267020	4	0.452753
rs4828611	ZNF275	X	152267020	7	0.302448
rs4828611	ZNF275	X	152267020	1	6.140096
rs3257	ZNF275	X	152270177	1	5.599891
rs3257	ZNF275	X	152270177	7	2.111662
rs1042103	BGN	X	152427225	3	1.697802
rs743642	BGN	X	152427415	4	2.565157
rs2269415	ATP2B3	X	152476922	1	3.543569
rs2269415	ATP2B3	X	152476922	4	2.678226
rs2266879	PLXNB3	X	152688992	8	2.006698
rs2266879	PLXNB3	X	152688992	5	1.44015
rs2266879	PLXNB3	X	152688992	6	0.89604
rs2266879	PLXNB3	X	152688992	7	2.675201
rs2266879	PLXNB3	X	152688992	4	1.73811
rs2266879	PLXNB3	X	152688992	1	2.456441
rs1802092	SRPK3	X	152704178	5	1.195531
rs1802092	SRPK3	X	152704178	4	0.258075
rs3747312	PDZD4	X	152724193	1	3.360173
rs5201	AVPR2	X	152825187	5	0.565698
rs5201	AVPR2	X	152825187	1	0.731864
rs2070097	ARHGAP4	X	152829448	2	3.638602
rs2070097	ARHGAP4	X	152829448	4	1.321523
rs2070097	ARHGAP4	X	152829448	6	0.495825
rs2070097	ARHGAP4	X	152829448	7	0.675826
rs2070097	ARHGAP4	X	152829448	1	1.247382
rs2070097	ARHGAP4	X	152829448	3	0.194438
rs730106	HCFC1	X	152874851	1	2.441719
rs730106	HCFC1	X	152874851	4	0.704002
rs730106	HCFC1	X	152874851	7	0.997952
rs730106	HCFC1	X	152874851	8	0.556098
rs35567597	PLXNA3	X	153347381	1	4.794392
rs35567597	PLXNA3	X	153347381	5	1.032487
rs1050047	UBL4A	X	153366645	8	6.472296
rs7057286	UBL4A	X	153366981	7	3.220462
GA026663	G6PD	X	153413848	7	3.963838
rs3813455	GAB3	X	153557329	6	0.469916
rs5945108	GAB3	X	153557591	1	3.156637
rs4431759	GAB3	X	153557667	1	3.73837
rs4431759	GAB3	X	153557667	7	1.500249

rs1127051	DKC1	X	153657778	6	0.485948
GA026671	DKC1	X	153658342	7	0.963954
rs559165	CLIC2	X	154160367	7	0.855297
rs35507574	SHOX	X	526325	2	1.587088
rs35507574	SHOX	X	526325	3	0.733652
rs35507574	SHOX	X	526325	5	0.935015
rs35507574	SHOX	X	526325	8	0.64631
rs17879004	IL3RA	X	1427404	1	1.80674
rs11480	IL3RA	X	1461471	4	4.295349
rs11480	IL3RA	X	1461471	7	0.137427
rs11480	IL3RA	X	1461471	1	2.898067
rs11480	IL3RA	X	1461471	8	2.54195
rs4503285	ASMTL	X	1497881	5	0.00182
rs4503285	ASMTL	X	1497881	7	1.308074
rs4503285	ASMTL	X	1497881	8	0.174579
rs4503285	ASMTL	X	1497881	3	1.005935
rs4933063	ASMT	X	1715404	5	0.299394
rs17849631	CD99	X	2642482	5	0.151213
rs700449	SPRY3	X	154663050	3	2.395819
rs700449	SPRY3	X	154663050	4	0.164964
rs700455	SPRY3	X	154664526	1	1.222149
rs700455	SPRY3	X	154664526	2	0.530189
